# Whole-genome selection signatures identified candidate genes associated with cashmere traits in Inner Mongolia cashmere goats

**DOI:** 10.5713/ab.25.0252

**Published:** 2025-07-11

**Authors:** Youjun Rong, Xiaofang Ao, Mingxuan Han, Qincheng Xia, Fangzheng Shang, Qi Lv, Zhiying Wang, Rui Su, Yanhong Zhao, Yanjun Zhang, Ruijun Wang

**Affiliations:** 1 College of Animal Science, Inner Mongolia Agricultural University, Hohhot, China; 2 Key Laboratory of Mutton Sheep Genetics and Breeding, Ministry of Agriculture, Hohhot, China; 3 Key Laboratory of Goat and Sheep Genetics, Breeding and Reproduction in Inner Mongolia Autonomous Region, Hohhot, China

**Keywords:** Candidate Genes, Cashmere Traits, Inner Mongolia Cashmere Goats, Selection Signatures, Whole-genome Resequencing

## Abstract

**Objective:**

Inner Mongolia cashmere goats are superior indigenous breeds developed through long-term natural selection and systematic artificial selection, which have experienced a certain intensity of selection pressure during the breeding process, leading to bipolar differentiation trends in cashmere traits. Therefore, identifying genomic selection signatures associated with cashmere traits in Inner Mongolia cashmere goats is crucial for breeding high-quality cashmere-producing goats.

**Methods:**

To unravel the genetic basis of cashmere traits, this study stratified 375 Inner Mongolia cashmere goats into eight subgroups based on breeding values for cashmere traits: high-yield vs low-yield cashmere types (HYCG vs LYCG), fine vs coarse cashmere types (FCG vs CCG), long vs short cashmere types (LCG vs SCG), and long vs short fleece types (LFCG vs SFCG). Whole-genome resequencing was performed for genotyping, followed by detection of selection signatures.

**Results:**

Results revealed 144, 158, 147, and 147 high-frequency run of homozygosity (ROH) regions in HYCG, FCG, LCG, and LFCG subgroups, respectively, annotating to 515, 565, 510, and 521 genes. Additionally, genomic regions under positive selection were identified using *F_ST_*, θπ ratios, and XP-EHH methods, with overlapping regions detected by ≥2 methods defined as candidate regions. Gene annotation identified 777, 660, 712, and 726 candidate genes in HYCG vs LYCG, FCG vs CCG, LCG vs SCG, and LFCG vs SFCG comparisons, respectively. These genes were enriched in 3,051 GO terms and 318 KEGG pathways, including Hippo, MAPK, Wnt, PI3K-Akt, and mTOR signaling pathways associated with cashmere growth and development, involving genes such as *LGR6*, *RUNX2*, *IGF1R*, *FGF9*, and *TCF7L1*.

**Conclusion:**

In this study, we employed four complementary approaches, including ROHs, *F_ST_*, θπ ratios, and XP-EHH, to identify genomic signatures of selection for cashmere traits in Inner Mongolia cashmere goats. These findings provide valuable insights for improving cashmere production performance and developing novel strains with high-quality cashmere in Inner Mongolia cashmere goats.

## INTRODUCTION

As a major cashmere-producing region, the Inner Mongolia Autonomous Region is renowned for its Inner Mongolia cashmere goats, which are characterized by high cashmere yield, fine fiber diameter, and soft texture. Cashmere traits directly influence production efficiency and economic benefits in the cashmere goat industry. These traits represent complex polygenic characteristics controlled by multiple minor-effect loci, influenced by both genetic and environmental factors. Through prolonged natural and artificial selection, significant phenotypic divergence has emerged in cashmere traits among Inner Mongolia cashmere goat populations. During this selection process, nucleotide diversity and allele frequencies decreased in the genome of Inner Mongolia cashmere goats, while population differentiation and linkage disequilibrium increased and became stabilized within populations. These unique genetic footprints inscribed in the genome are referred to as selection signatures. Therefore, genome-wide scanning for these selection signatures and mapping candidate genes associated with cashmere traits will facilitate the development of genetic markers for genome-wide selective breeding programs in Inner Mongolia cashmere goats.

With the widespread application of whole-genome resequencing technology in livestock breeding, we now have sufficient information to identify selection signatures across the genome. Based on selection effects, methods for detecting selection signatures can be broadly categorized into four types: (1) Run of homozygosity (ROH) based on genomic heterozygosity; (2) Genetic differentiation index (FST) based on increased population divergence; (3) θπ ratios based on reduced nucleotide polymorphism; (4) Cross-Population Extended Haplotype Homozygosity (XP-EHH) based on increased linkage disequilibrium. Integrating all genomic feature information enables more accurate characterization of selection signatures across the genome associated with cashmere trait evolution. ROH refers to contiguous homozygous segments in the genome, whose formation is primarily associated with selection pressure and genetic drift [1]. Recently, ROH has been widely used as a powerful tool for identifying candidate genes and selection signatures related to economic traits in livestock [2–5]. ROH analysis can reveal genetic drift characteristics driven by selection pressure in Inner Mongolia cashmere goat populations and screen candidate genes associated with important economic traits. FST is a metric for measuring population differentiation caused by genetic structure [6], typically estimated from genetic polymorphism data such as single nucleotide polymorphisms (SNPs) or microsatellites. FST represents one of the most commonly used statistics in population genetics. θπ ratios evaluate genetic polymorphism indices by quantifying the proportion of nucleotide polymorphic sites. The XP-EHH method detects ongoing or nearly fixed selection signatures by comparing haplotypes between two populations [7].

To date, various genomic regions and candidate genes associated with cashmere traits have been identified using different approaches. Comparative genomics analysis revealed the critical roles of type IV collagen family genes (*COL4A2*, *COL4A4*, *COL4A5*, *COL6A5*, *COL6A6*) and integrin family genes (*ITGA2*, *ITGA4*, *ITGA9*, *ITGB8*) in cashmere trait formation [8]. F_ST_ and XP-EHH analyses identified key genes such as *WNT10A*, *CSN3*, *TRPS1*, and *IGF1R* in Inner Mongolia vs Liaoning cashmere goats, which may participate in hair follicle morphogenesis and stem cell pluripotency regulation [9]. Integrated multi-omics analysis of metabolome, transcriptome, and proteome demonstrated that *DNMT3B*, *HMCN1*, *CPB2*, *GNG12*, and other genes enriched in mTOR and amino acid metabolism pathways synergistically regulate cashmere fineness phenotypes [10]. RT-qPCR and immunofluorescence detection in skin tissues of Inner Mongolia cashmere goats (Arbas type) showed that *FGF2*, *FGF21*, and *BMP7* exhibited peak expression at 3 months of age, potentially playing important regulatory roles in secondary hair follicle development [11]. Collectively, these studies indicate that cashmere traits are influenced by genetic factors. Despite these valuable findings, the genetic mechanisms underlying cashmere growth and development remain poorly understood. Therefore, identifying genes potentially involved in cashmere growth regulation continues to hold significant importance.

Since breeding values accurately reflect an individual’s genetic superiority, individuals with high breeding values are selected for reproduction during artificial selection, thereby improving the production performance of breeding populations. Therefore, this study stratified Inner Mongolia cashmere goats into contrasting groups based on breeding values: high-yield vs low-yield cashmere types, fine vs coarse cashmere types, long vs short cashmere types, and long vs short fleece types, to investigate genomic differences among these groups. Using SNP genotyping data, we identified high-frequency ROH regions by detecting runs of homozygosity in four subgroups: high-yield cashmere, fine cashmere, long cashmere, and long fleece types. Additionally, we measured F_ST_, θπ ratios, and XP-EHH across eight subgroups to identify selection signatures in the genome of Inner Mongolia cashmere goats, thereby uncovering candidate genes associated with cashmere traits.

## MATERIALS AND METHODS

### Animal materials

Based on the calculated breeding values for four cashmere traits (cashmere yield, cashmere diameter, cashmere thickness, and fleece length) in Inner Mongolia cashmere goats [12], we defined eight subgroups: high-yield cashmere goats (HYCG), low-yield cashmere goats (LYCG), fine cashmere goats (FCG), coarse cashmere goats (CCG), long cashmere goats (LCG), short cashmere goats (SCG), long fleece cashmere goats (LFCG), and short fleece cashmere goats (SFCG). There were 100 individuals in each subgroup. The descriptive statistics of the phenotypic values of each subgroup are shown in Table 1. There are a total of 375 non-duplicate individuals in these 8 subgroups. Detailed sample information is provided in Supplement 1. Raw whole-genome resequencing data for the 375 Inner Mongolia cashmere goats have been deposited in the NCBI SRA database (PRJNA1096828).

### Single nucleotide polymorphism calling

For data preprocessing, raw reads were first cleaned using fastp software (V0.20.0) [13]. Clean reads were then aligned to the goat reference genome (ARS1, GCF_001704415.1) using Burrows-Wheeler Aligner (BWA) software (V0.7.17) [14]. SAMtools software (V1.8–20) [15] was used to convert SAM files to BAM format and sort the BAM files. Genome Analysis Toolkit (GATK) (V3.8) [16] was employed for BAM file processing and SNP calling. After obtaining VCF files, VariantFiltration module was applied for filtering.

### Genotype quality control and principal component analysis

To achieve high-quality SNPs, further quality control was performed using Plink (V1.90) [17] with the following criteria: (1) SNPs with call rate <98% were removed; (2) SNPs with minor allele frequency (MAF) <5% were excluded; (3) SNPs deviating from Hardy-Weinberg equilibrium (HWE) (p<10^−6^) were filtered out; (4) individuals with call rate <98% were excluded. The quality-controlled genotype data were visualized for SNP density distribution across chromosomes using the plot_snp_density() function in the R package HandyCNV (V1.1.6) [18]. The “--pca 3” parameter of Plink (V1.90) software was used to calculate the first three principal components of these eight subgroups, and the principal component analysis (PCA) plot was drawn using R (V3.6.0).

### Runs of homozygosity detection

To identify genomic regions associated with ROHs in the four subgroups (high-yield cashmere, fine cashmere, long cashmere, and long fleece types), ROHs were detected using Plink software (V1.90). The following parameters were applied: sliding window size of 100 SNPs (--homozyg-window-snp 100); maximum 1 heterozygous SNP per window (--homozyg-window-het 1); maximum 1 missing SNP per window (--homozyg-window-missing 1); minimum SNP density of 1 per 50 kb (--homozyg-density 50); maximum gap between consecutive SNPs of 500 kb (--homozyg-gap 500); and minimum ROH length of 100 kb (--homozyg-kb 100).

### Detection of high-frequency run of homozygosity regions

ROH results were summarized using the ‘roh_window ()’ function in the HandyCNV package [18]. A genomic sliding window size of 50 kb (window_size = 0.05) was applied for ROH visualization. ROH lengths were classified into five categories: 0.1–0.2 Mb, 0.2–0.4 Mb, 0.4–0.8 Mb, 0.8–1.6 Mb, and ≥1.6 Mb. Additionally, windows with sample frequency >25% in the four subgroups (high-yield cashmere, fine cashmere, long cashmere, and long fleece types) were defined as high-frequency ROH regions (threshold = 0.25). Adjacent windows exceeding the threshold were merged to generate a list of high-frequency ROH regions. The total autosomal length of the goat reference genome GCF_001704415.1 (ARS1) is approximately 2,466.191 Mb, which was used to calculate the proportion of ROH lengths across the genome.

### Selection signature detection

To characterize the selected genomic regions in Inner Mongolia cashmere goats, we employed FST, θπ Ratio, and XP-EHH methods to detect genomic selection signatures across eight subgroups: HYCG vs LYCG, FCG vs CCG, LCG vs SCG, and LFCG vs SFCG. First, F_ST_ values between subgroup pairs (HYCG/LYCG, FCG/CCG, LCG/SCG, or LFCG/SFCG) were calculated using VCFtools (V0.1.16) [19] with a 50 kb sliding window and 10 kb step size. For θπ ratio calculations, VCFtools (V0.1.16) was used to compute θπ ratios between subgroups (LYCG/HYCG, CCG/FCG, SCG/LCG, or SFCG/LFCG) using the same 50 kb sliding window and 10 kb step size. For XP-EHH analysis, phasing of VCF files for 29 autosomes was performed using Beagle (V5.5), followed by XP-EHH value calculation with Selscan (V1.3.0) [20]. The top 1% of extreme values from each method were empirically selected as potential candidate regions under positive selection. Significant selection regions or SNPs were visualized with threshold lines in Manhattan plots using the CMplot package in R (V4.2.2) [21] (https://github.com/YinLiLin/R-CMplot, 20 January 2019).

### Annotation and enrichment analysis of the candidate genes

Candidate regions identified by four complementary methods (ROHs, *F*_ST_, *θ*π ratios, and XP-EHH) were annotated using Bedtools software (V2.30.0) [22]. For SNPs detected by XP-EHH, 50 kb upstream and downstream regions were annotated. The number of candidate genes from all methods was visualized using the Venny (V2.1) online tool (https://bioinfogp.cnb.csic.es/tools/venny/index.html). Additionally, Gene Ontology (GO) functional annotation and Kyoto Encyclopedia of Genes and Genomes (KEGG) enrichment analysis were performed on candidate genes using the clusterProfiler package in R (V4.2.2) [23]. Manual curation of GO terms and KEGG signaling pathways was conducted to infer potential gene functions.

## RESULTS

### Single nucleotide polymorphism density statistical results

**Figure 1:**
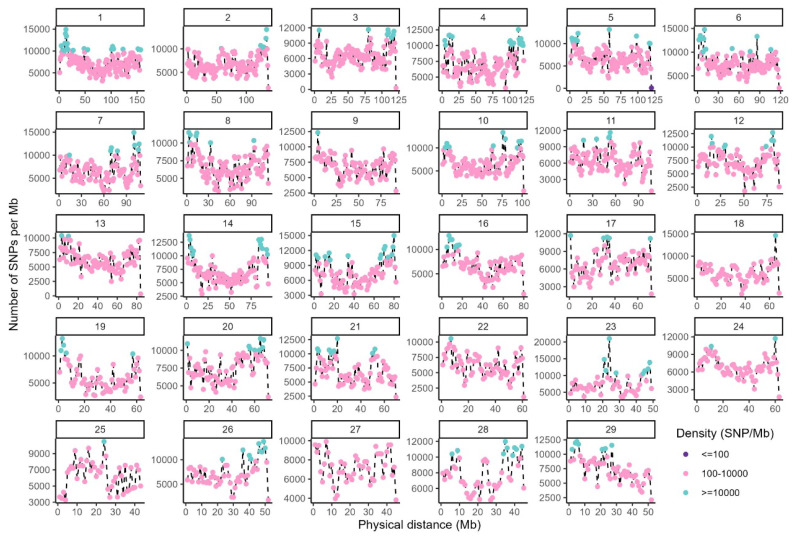
Whole-genome SNP density distribution in Inner Mongolia cashmere goats. Regions with SNP density ≤100 are represented by purple dots, regions with density between 100–10,000 are shown as pink dots, and regions with density ≥10,000 are indicated by green dots. SNP, single nucleotide polymorphism.

Following data quality control, each of the eight subgroups contained 100 individuals and 17,135,082 SNPs distributed across 29 autosomes, with an average density of 6,904 SNPs per megabase (Mb). Among these, 213 regions (1 Mb in length) exhibited SNP density >10,000 SNPs/Mb, 1 region had density <100 SNPs/Mb, and 2,268 regions showed density between 100–10,000 SNPs/Mb (Figure 1), indicating the high quality of our dataset.

### Principal component analysis

**Figure 2:**
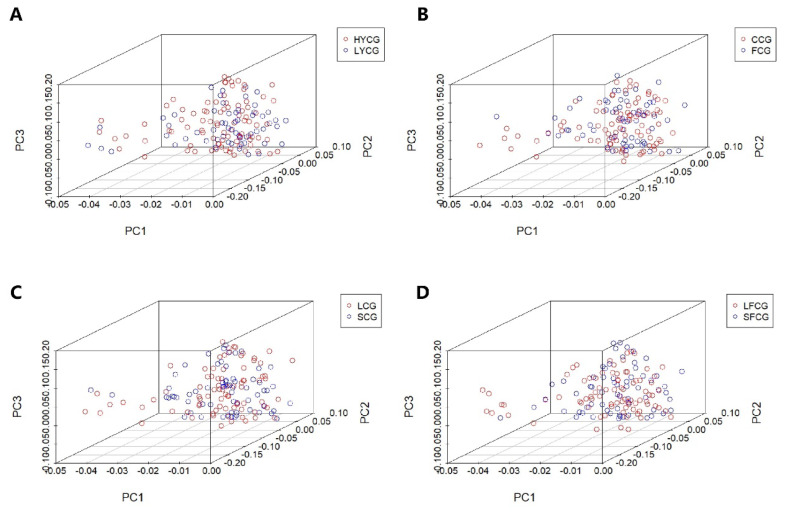
Visualization of PCA. The first three explain the variance percentage (PC1, PC2, and PC3) as the X, Y, and Z axes. (A) represents the PCA visualization of HYCG and LYCG. (B) represents the PCA visualization of CCG and FCG. (C) represents the PCA visualization of LCG and SCG. (D) represents the PCA visualization of LFCG and SFCG. HYCG, high-yield cashmere goats; LYCG, low-yield cashmere goats; CCG, coarse cashmere goats; FCG, fine cashmere goats; LCG, long cashmere goats; SCG, short cashmere goats; LFCG, long fleece cashmere goats; SFCG, short fleece cashmere goats; PCA, principal component analysis.

We calculated the first three principal components by using Plink software, and the results showed that there is no group stratification among the eight subgroups (Figure 2). Therefore, the results of this study are not affected by the population genetic structure.

### Run of homozygosity analysis

**Figure 3:**
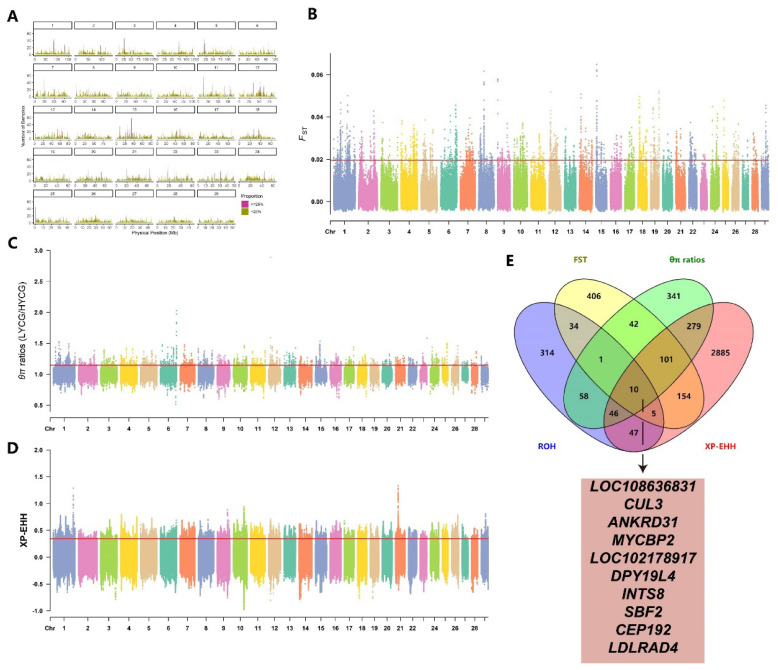
Genome-wide selection scan in high-yield cashmere goats (HYCG) and low-yield cashmere goats (LYCG). Using sliding window analysis (50 kb window size, 10 kb step size). (A) High-frequency ROH Regions. (B) Selection signatures for *F_ST_*. (C) Selection signatures for θπ ratios. (D) Selection signatures for XP-EHH. (E) The number of genes overlapping with each other among the results of four detection methods, and the genes detected simultaneously by all four methods. Threshold (top 1%) of *F_ST_*, θπ ratios, XP-EHH is marked with a horizontal red line. ROH, run of homozygosity.

In the high-yield cashmere subgroup, a total of 59,385 ROHs were detected, with an average of 593.8 ROHs per sample and a maximum length of approximately 2.82 Mb. As the length of ROHs increased, the number of ROHs showed a downward trend. Among them, the number of ROHs in the 0.1–0.2 Mb length interval was the highest, accounting for about 72.8% of the total. On average, each sample had about 432.3 ROHs in this interval. The number of ROHs with a length of over 1.6 Mb was the lowest (Table 2). Additionally, 144 high - frequency ROHs (sample frequency ≥25%) were found, distributed across chromosomes 1–26 (Figure 3A). The total length of these high - frequency ROHs was about 25.9 Mb, accounting for approximately 1.05% of the total length of the autosomal genome. Among them, chromosomes 5 and 11 had the largest number of high - frequency ROH regions, with 14 high - frequency ROH regions detected on each. Moreover, there was a large region on chromosomes 2 and 18 where the frequency of ROH information was low, indicating a high level of genetic variability (Figure 3A). Among the 144 high - frequency ROHs, a total of 120 ROHs were annotated to 515 genes, while the remaining 24 ROHs had no annotations in the reference genome (Supplement 2).

**Figure 4:**
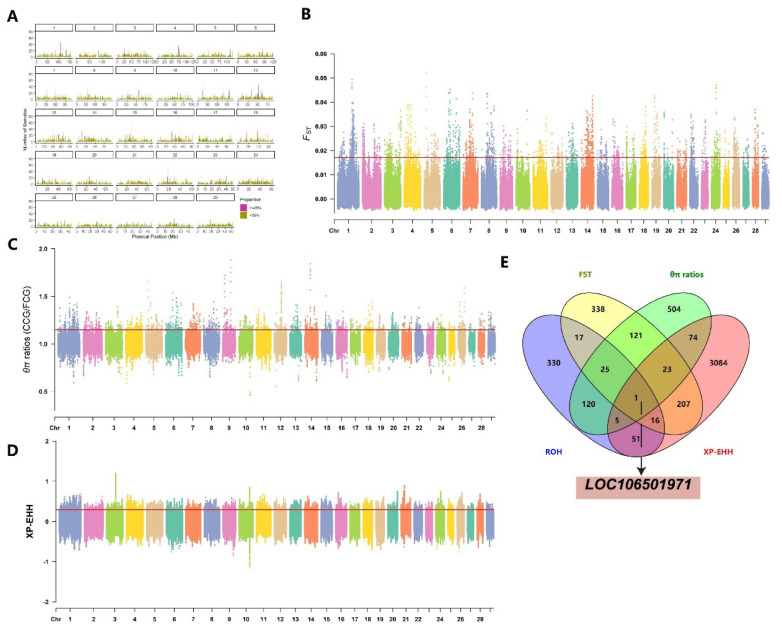
Genome-wide selection scan in fine cashmere goats (FCG) and coarse cashmere goats (CCG). Using sliding window analysis (50 kb window size, 10 kb step size). (A) High-frequency ROH Regions. (B) Selection signatures for *F_ST_*. (C) Selection signatures for θπ ratios. (D) Selection signatures for XP-EHH. (E) The number of genes overlapping with each other among the results of four detection methods, and the genes detected simultaneously by all four methods. Threshold (top 1%) of *F_ST_*, θπ ratios, XP-EHH is marked with a horizontal red line. ROH, run of homozygosity.

In the fine cashmere subgroup, a total of 62,681 ROHs were detected, with the longest ROH measuring approximately 3.26 Mb. The number of ROHs decreased with increasing length, with the highest proportion (70.4% of total) observed in the 0.1–0.2 Mb interval (average 441.3 ROHs per sample), and the lowest proportion in ROHs ≥1.6 Mb (Table 3). Additionally, 158 high-frequency ROHs (sample frequency ≥25%) were identified (Figure 4A), spanning a total length of 28 Mb (1.13% of the autosomal genome). Chromosome 5 harbored the highest number of high-frequency ROH regions (16 regions). Notably, chromosomes 2 and 18 displayed large genomic regions with low ROH frequency, indicating a higher level of variation in this region (Figure 4A). Among the 158 high-frequency ROHs, 141 were annotated to 565 genes, while 17 ROHs had no annotations in the reference genome (Supplement 3).

**Figure 5:**
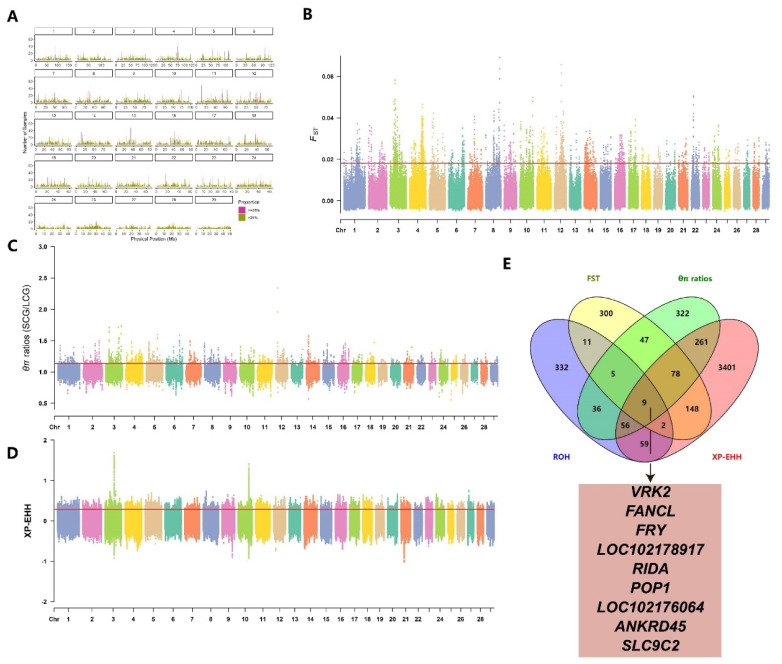
Genome-wide selection scan in long cashmere goats (LCG) and short cashmere goats (SCG). Using sliding window analysis (50 kb window size, 10 kb step size). (A) High-frequency ROH Regions. (B) Selection signatures for *F_ST_*. (C) Selection signatures for θπ ratios. (D) Selection signatures for XP-EHH. (E) The number of genes overlapping with each other among the results of four detection methods, and the genes detected simultaneously by all four methods. Threshold (top 1%) of *F_ST_*, θπ ratios, XP-EHH is marked with a horizontal red line. ROH, run of homozygosity.

In the long cashmere subgroup, a total of 59,071 ROHs were detected, with the maximum length of approximately 3.26 Mb. As the length of ROHs increased, the number of ROHs showed a downward trend. Among them, the number of ROHs in the 0.1–0.2 Mb length interval was the highest, accounting for about 73.3% of the total. On average, each sample had about 432.9 ROHs in this interval. The number of ROHs with a length of over 1.6 Mb was the lowest (Table 4). Additionally, 147 high - frequency ROHs (sample frequency ≥25%) were found, distributed across chromosomes 1–26 (Figure 5A). The total length of these high - frequency ROHs was about 23.8 Mb, accounting for approximately 0.97% of the total length of the autosomal genome. Among them, chromosomes 5 and 11 had the largest number of high - frequency ROH regions, with 14 high - frequency ROH regions detected on each. Moreover, there was a large region on chromosomes 2 and 18 where the frequency of ROH information was low, indicating a high level of genetic variability (Figure 5A). Among the 147 high - frequency ROHs, a total of 123 ROHs were annotated to 510 genes, while the remaining 24 ROHs had no annotations in the reference genome (Supplement 4).

**Figure 6:**
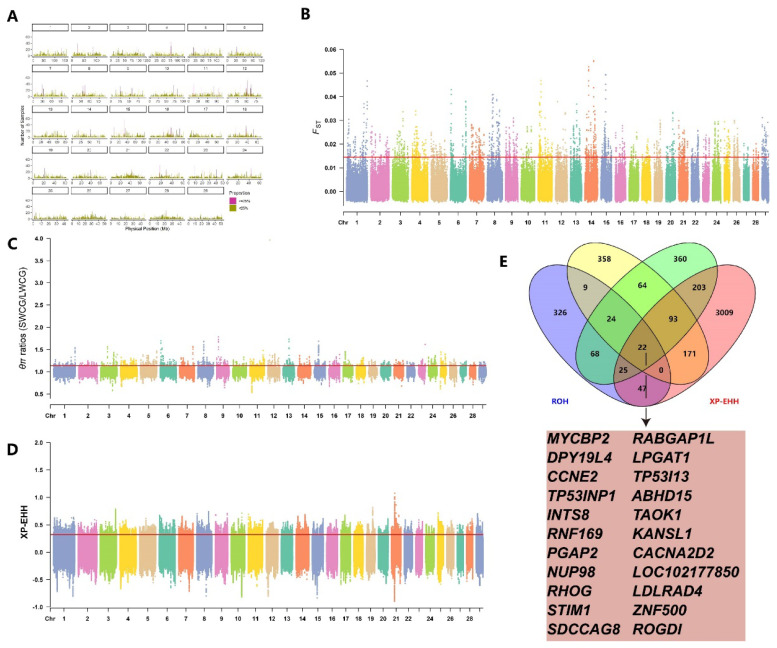
Genome-wide selection scan in long fleece cashmere goats (LFCG) and short fleece cashmere goats (SFCG). Using sliding window analysis (50 kb window size, 10 kb step size). (A) High-frequency ROH Regions. (B) Selection signatures for *F_ST_*. (C) Selection signatures for θπ ratios. (D) Selection signatures for XP-EHH. (E) The number of genes overlapping with each other among the results of four detection methods, and the genes detected simultaneously by all four methods. Threshold (top 1%) of *F_ST_*, θπ ratios, XP-EHH is marked with a horizontal red line. ROH, run of homozygosity.

In the long fleece subgroup, a total of 56,856 ROHs were detected, with the maximum length of approximately 2.57 Mb. As the length of ROHs increased, the number of ROHs showed a downward trend. Among them, the number of ROHs in the 0.1–0.2 Mb length interval was the highest, accounting for about 74.3% of the total. On average, each sample had about 422.6 ROHs in this interval. The number of ROHs with a length of over 1.6 Mb was the lowest (Table 5). Additionally, 149 high - frequency ROHs (sample frequency ≥25%) were found, distributed across chromosomes 1–26 (Figure 6A). The total length of these high - frequency ROHs was about 25.1 Mb, accounting for approximately 1.02% of the total length of the autosomal genome. Among them, chromosomes 5 and 11 had the largest number of high - frequency ROH regions, with 14 high - frequency ROH regions detected on each. Moreover, there were two large genomic regions on chromosome 2 where ROH frequency was low, indicating high genetic variability (Figure 6A). Among the 147 high - frequency ROHs, a total of 127 ROHs were annotated to 521 genes, while the remaining 20 ROHs had no annotations in the reference genome (Supplement 5).

### Genome-wide scan of selection signature detection

#### HYCG VS LYCG

We calculated FST, θπ ratios, and XP-EHH values between HYCG and LYCG subgroups, using the top 1% thresholds (FST≥0.02, θπ ratios≥1.14, XP-EHH≥0.34) to identify significant selection regions. The FST method detected 2,466 selection regions in HYCG, annotated to 753 genes (Figure 3B, Supplement 6). The θπ ratios method also identified 2,466 selection regions, annotated to 878 genes (Figure 3C, Supplement 7). The XP-EHH method detected 171,305 candidate SNPs, annotated to 3,527 genes (Figure 3D, Supplement 8). Additionally, 144 high-frequency ROHs were identified in HYCG, annotating to 515 candidate genes. A total of 777 shared genes were detected by two or more methods, with 10 genes including MYCBP2, DPY19L4, and INTS8 identified by all four methods (Figure 3E).

#### FCG VS CCG

By calculating FST, θπ ratios, and XP-EHH values between FCG and CCG subgroups, we identified genomic selection signatures in the FCG subgroup during artificial selection. The top 1% thresholds (FST≥0.02, θπ ratios≥1.15, XP-EHH≥0.29) were used to identify selection regions. The FST method detected 2,466 selection regions in FCG, annotated to 748 genes (Figure 4B, Supplement 9). The θπ ratios method also identified 2,466 selection regions, annotated to 873 genes (Figure 4C, Supplement 10). The XP-EHH method detected 171,308 candidate SNPs, annotated to 3,461 genes (Figure 4D, Supplement 11). Additionally, 158 high-frequency ROHs were identified in FCG, annotating to 565 candidate genes. A total of 660 shared genes were detected by two or more methods, with LOC106501971 identified by all four methods (Figure 4E).

#### LCG VS SCG

By calculating FST, θπ ratios, and XP-EHH values between LCG and SCG subgroups, we identified genomic selection signatures in the LCG subgroup during artificial selection. The top 1% thresholds (FST≥0.02, θπ ratios≥1.14, XP-EHH ≥0.29) were used to identify selection regions. The FST method detected 2,466 selection regions in LCG, annotated to 600 genes (Figure 5B, Supplement 12). The θπ ratios method also identified 2,466 selection regions, annotated to 814 genes (Figure 5C, Supplement 13). The XP-EHH method detected 171,308 candidate SNPs, annotated to 4,014 genes (Figure 5D, Supplement 14). Additionally, 147 high-frequency ROHs were identified in LCG, annotating to 510 candidate genes. A total of 712 shared genes were detected by two or more methods, with nine genes including VRK2, FANCL, and FRY identified by all four methods (Figure 5E).

#### LFCG VS SFCG

By calculating FST, θπ ratios, and XP-EHH values between LFCG and SFCG subgroups, we identified genomic selection signatures in the LFCG subgroup during artificial selection. The top 1% thresholds (FST≥0.01, θπ ratios≥1.13, XP-EHH≥0.32) were used to identify selection regions. The FST method detected 2,466 selection regions in LFCG, annotated to 741 genes (Figure 6B, Supplement 15). The θπ ratios method also identified 2,466 selection regions, annotated to 859 genes (Figure 6C, Supplement 16). The XP-EHH method detected 171,308 candidate SNPs, annotated to 3,570 genes (Figure 6D, Supplement 17). Additionally, 147 high-frequency ROHs were identified in LFCG, annotating to 521 candidate genes. A total of 726 shared genes were detected by two or more methods, with 22 genes including MYCBP2, DPY19L4, and INTS8 identified by all four methods (Figure 6E).

### Enrichment analysis of candidate gene

**Figure 7:**
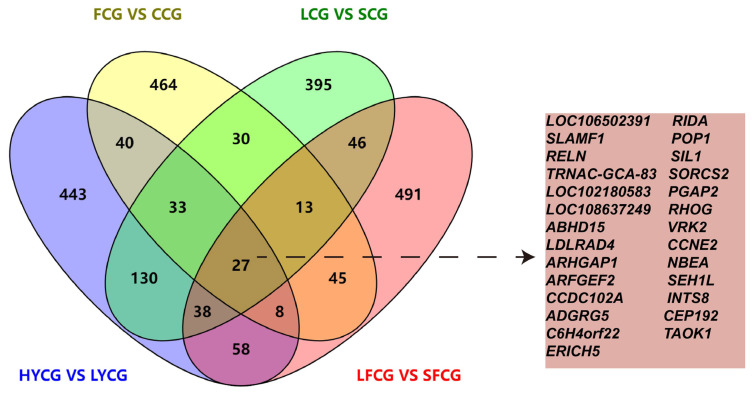
Overlapping selection candidate genes by ROHs, *F_ST_*, θπ ratios, and XP-EHH in HYCG/LYCG, FCG/CCG, LCG/SCG and LFCG/SFCG. FCG, fine cashmere goats; CCG, coarse cashmere goats; LCG, long cashmere goats; SCG, short cashmere goats; HYCG, high-yield cashmere goats; LYCG, low-yield cashmere goats; ROH, run of homozygosity; LFCG, long fleece cashmere goats; SFCG, short fleece cashmere goats.

**Figure 8:**
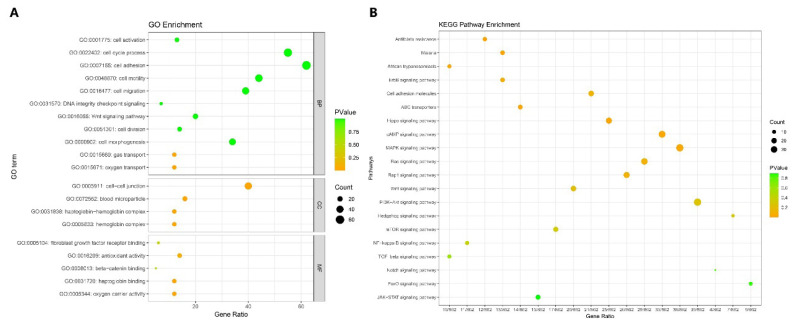
Bubble diagram of GO and KEGG enrichment analysis of the candidate genes. (A) represents the GO enrichment results, in the legend, BP represents biological process, CC represents cellular component, MF represents molecular function. (B) represents the KEGG enrichment results.

A total of 2,261 candidate genes were detected across eight Inner Mongolia cashmere goat subgroups using two or more methods (Figure 7). Based on these genes, we further screened out the top candidate genes with definite functions in hair follicle biology, as shown in Table 6. Functional annotation and enrichment analysis of these genes were performed using the clusterProfiler package, revealing enrichment in 3,051 GO terms and 318 KEGG pathways (Supplements 18, 19). Enriched GO terms included cell-cell junction (GO:0005911), antioxidant activity (GO:0016209), cell adhesion (GO:0007155), fibroblast growth factor receptor binding (GO:0005104), and cell cycle process (GO:0022402) (Figure 8A), which are associated with cashmere growth and development [24–27]. KEGG pathways such as ABC transporters (chx02010), Hippo (chx04390), cAMP (chx04024), MAPK (chx04010), Ras (chx04014), and Wnt (chx04310) were also enriched (Figure 8B) [28]. Notably, 27 shared genes were identified across all eight subgroups. Among them, RELN was enriched in focal adhesion (chx04510), ECM-receptor interaction (chx04512), and PI3K-Akt signaling pathway (chx04151); TAOK1 was enriched in MAPK (chx04010); CCNE2 was involved in cell cycle process (GO:0022402) and PI3K-Akt (chx04151); and SEH1L and INTS8 were enriched in mTOR (chx04150) signaling pathway. These pathways are all associated with cashmere growth and development.

## DISCUSSION

In this study, we first detected ROHs in four target groups: high-yield cashmere, fine cashmere, long cashmere, and long fleece types. The length of ROHs reflects a population’s inbreeding history, as the probability of ROH disruption by recombination increases with generations. Shorter ROHs typically result from ancient inbreeding, while longer ROHs indicate recent inbreeding events [29]. We found that ROH lengths in the four target subgroups (high-yield cashmere, fine cashmere, long cashmere, and long fleece types) were concentrated in the 0.1–0.2 Mb interval. These shorter ROHs suggest no severe inbreeding occurred in recent generations of Inner Mongolia cashmere goats. During selection, the rapid increase in frequency of haplotypes carrying beneficial mutations can also increase ROH number and length, leading to reduced genetic variation in flanking regions of target loci. Thus, ROHs are regarded as genomic footprints of recent positive selection [30]. Therefore, ROHs can be used to identify selection signals and map candidate genes. In this study, gene annotation of high-frequency ROH regions identified 515, 565, 510, and 521 candidate genes in the four target groups.

To identify candidate genes associated with cashmere traits more accurately, we employed F_ST_, θπ ratios, and XP-EHH methods for genomic selection signature analysis in Inner Mongolia cashmere goats. A total of 777, 660, 712, and 726 candidate genes (detected by two or more methods) were identified in HYCG vs LYCG, FCG vs CCG, LCG vs SCG, and LFCG vs SFCG comparisons, respectively. Among them, 27 candidate genes were commonly detected across all populations, suggesting their potential regulatory roles in cashmere trait selection. Studies have shown that *LGR6*, as a biomarker of isthmic stem cells in hair follicles, is enriched in the Wnt signaling pathway and can induce stem cells to differentiate into hair follicles, sebaceous glands and epidermal cells, participating in multilineage differentiation and tissue repair of hair follicles [31,32]. *RUNX2* is enriched in the Wnt and Hedgehog signaling pathways, expressed in the dermal papillae and epithelial cells of developing hair follicles, and affects hair follicle morphogenesis and cycle regulation [33]. *FGF9* is enriched in the FGF signaling pathway, expressed in dermal papillary cells, and promotes the activation of hair follicle stem cells and hair follicle regeneration [34]. *IGF1R* is enriched in the IGF signaling pathway, expressed in dermal papillary cells, participates in the signal transduction of cell proliferation and survival, and plays an important role in maintaining the normal function of dermal papillary cells and hair follicle development [35,36]. *TCF7L1* is enriched in the Wnt signaling pathway and, as a key transcription factor of the Wnt signaling pathway, participates in regulating processes such as cell proliferation and differentiation [37]. After activation of the PI3K-Akt and mTOR signaling pathways, it can promote the proliferation, migration and cell cycle progression of human hair follicle dermal papilla cells, and simultaneously activate the Wnt/β-catenin pathway, jointly promoting hair follicle growth [38]. Another contrary study showed that corticotropin-releasing hormone inhibited autophagy of hair follicle dermal papilla cells and promoted apoptosis by suppressing PTEN in the PI3K/AKT/mTOR signaling pathway, resulting in blocked hair follicle regeneration and triggering stress-induced alopecia [39]. Researchers can simultaneously activate the Wnt/β-catenin and MAPK signaling pathways through alternating current stimulation. The two jointly promote the proliferation of human dermal papillary cells and the expression of hair follicle-related genes (such as *KGF* and *VEGF*). The regulation of this synergistic effect on hair follicle growth and hair growth cycle was further verified in the rabbit model [40]. Collectively, these candidate genes play crucial roles in regulating cashmere growth and development, offering valuable insights for improving cashmere production performance in Inner Mongolia cashmere goats.

## CONCLUSION

In this study, we employed four complementary approaches, including ROHs, F_ST_, θπ ratios, and XP-EHH, to identify genomic signatures of selection for cashmere traits in Inner Mongolia cashmere goats. Numerous candidate regions and genes were identified, such as *LGR6, RUNX2, IGF1R, FGF9, and TCF7L1*, with most genes associated with hair follicle morphogenesis and cashmere growth and development. Our findings contribute to a better understanding of the breeding potential and genetic uniqueness of Inner Mongolia cashmere goats, and are of significant importance for elucidating the genetic mechanisms underlying cashmere trait formation.

## Figures and Tables

**Table 1:** Descriptive statistics of phenotypic values of 8 subgroups

Subgroups	Number of records	Max	Mean	Min	SD	CV (%)
HYCG	100	544.9	190.1799	86.64	91.89	48.32
LYCG	100	−103.75	−179.837	−727.42	96.70	−53.77
CCG	100	1.99	0.8763	0.45	0.38	43.64
FCG	100	−0.31	−0.6599	−2.56	0.38	−58.29
LCG	100	2.23	0.8342	0.42	0.35	41.99
SCG	100	−0.37	−0.7179	−1.9	0.32	−44.69
LFCG	100	5.99	2.5948	1.34	1.05	40.63
SFCG	100	−1.07	−2.5264	−6.21	1.20	−47.41

HYCG, high-yield cashmere goats; LYCG, low-yield cashmere goats; CCG, coarse cashmere goats; FCG, fine cashmere goats; LCG, long cashmere goats; SCG, short cashmere goats; LFCG, long fleece cashmere goats; SFCG, short fleece cashmere goats.

**Table 2:** ROH statistics in different length regions in HYCG

Length_group (Mb)	Number_ROH	Mean	SD	Min	Max	Proportion	N_per_animal
0.1–0.2	43,233	134,476	26,779	100,000	199,998	0.728	432.3
0.2–0.4	12,273	268,820	53,806	200,002	399,975	0.207	122.7
0.4–0.8	3,297	526,140	102,420	400,014	799,560	0.056	33
0.8–1.6	553	1,004,979	175,249	800,033	1,594,910	0.009	5.5
≥1.6	29	2,012,450	326,898	1,610,159	2,823,986	0	0.3
Total	59,385						593.8

ROH, run of homozygosity; HYCG, high-yield cashmere goats; SD, standard deviation.

**Table 3:** ROH statistics in different length regions in FCG

Length_group (Mb)	Number_ROH	Mean	SD	Min	Max	Proportion	N_per_animal
0.1–0.2	44,130	135,062	26,988	100,001	199,998	0.704	441.3
0.2–0.4	13,672	271,104	54,849	200,004	399,999	0.218	136.7
0.4–0.8	4,170	531,446	104,552	400,010	799,793	0.067	41.7
0.8–1.6	672	1,001,052	167,797	800,025	1,572,122	0.011	6.7
≥1.6	37	1,912,380	361,752	1,600,830	3,258,204	0.001	0.4
Total	62,681						626.8

ROH, run of homozygosity; FCG, fine cashmere goats; SD, standard deviation.

**Table 4:** ROH statistics in different length regions in LCG

Length_group (Mb)	Number_ROH	Mean	SD	Min	Max	Proportion	N_per_animal
0.1–0.2	43,288	134,438	26,751	100,000	199,953	0.733	432.9
0.2–0.4	12,128	268,578	53,936	200,009	399,656	0.205	121.3
0.4–0.8	3,160	525,122	101,031	400,010	799,973	0.053	31.6
0.8–1.6	472	1,001,740	172,745	800,760	1,597,842	0.008	4.7
≥1.6	23	1,887,542	350,081	1,602,934	3,184,378	0	0.2
Total	59,071						590.7

ROH, run of homozygosity; LCG, long cashmere goats; SD, standard deviation.

**Table 5:** ROH statistics in different length regions in LFCG

Length_group (Mb)	Number_ROH	Mean	SD	Min	Max	Proportion	N_per_animal
0.1–0.2	42,256	134,338	26,816	100,000	199,993	0.743	422.6
0.2–0.4	11,261	267,044	53,410	200,009	399,987	0.198	112.6
0.4–0.8	2,867	525,337	100,026	400,010	797,377	0.05	28.7
0.8–1.6	448	999,405	164,558	800,892	1,597,842	0.008	4.5
≥1.6	24	1,943,122	282,111	1,612,312	2,571,696	0	0.2
Total	56,856						568.6

ROH, run of homozygosity; LFCG, long fleece cashmere goats; SD, standard deviation.

**Table 6:** The functions of candidate genes in hair follicle biology

Chromosomal region (Mb)	Genes	Methods	Functional association	References
16:78.85–78.90	*LGR6*	F_ST_, XP-EHH	It is expressed in the stem cells located in the central isthmus of hair follicles, and participates in the cell differentiation and regeneration of hair follicles and epidermis.	[31,32]
23:30.04–30.10	*RUNX2*	ROH, F_ST_, XP-EHH	This gene exhibits dynamic stage-specific expression in hair follicles, and its deletion delays hair follicle maturation, leading to significant reduction in skin and epidermal thickness and weakened epidermal cell proliferation.	[33]
12:50.25–51.40	*FGF9*	ROH, θπ ratios	FGF9 can promote the transition of hair follicles from telogen to anagen, accelerate the proliferation of dermal papilla cells, and thus induce hair regeneration by regulating the Wnt/β-catenin signaling pathway.	[34]
21:7.08–7.18	*IGF1R*	F_ST_, XP-EHH	As a target gene of miR-1 and miR-let7a, its expression level is higher in the anagen phase than in the catagen phase of hair follicles in Liaoning cashmere goats, and it participates in the regulation of proliferation and differentiation of hair follicle stem cells through the IGF signaling pathway.	[35,36]
11:49.38–49.48	*TCF7L1*	F_ST_, XP-EHH	As a key transcription factor of the Wnt signaling pathway, the down-regulation of this gene’s expression in canine skin leads to the obstruction of hair follicle stem cell activation, and then participates in regulating the transition process of hair follicles from the resting phase to the anagen phase.	[37]

ROH, run of homozygosity.

## SUPPLEMENTARY MATERIAL

**Supplement 1.** Sample information for 8 subpopulations in IMCGs.

**Supplement 2.** High-frequency ROH regions and annotated genes in HYCG.

**Supplement 3.** High-frequency ROH regions and annotated genes in FCG.

**Supplement 4.** High-frequency ROH regions and annotated genes in LCG.

**Supplement 5.** High-frequency ROH regions and annotated genes in LFCG.

**Supplement 6.** Detected and gene annotation of candidate regions by F_ST_ (HYCG VS LYCG).

**Supplement 7.** Detected and gene annotation of candidate regions by θπ ratios (LYCG VS HYCG).

**Supplement 8.** Detected and gene annotation of candidate regions by XP-EHH (HYCG VS LYCG). **Supplement 9.** Detected and gene annotation of candidate regions by F_ST_ (FCG VS CCG). **Supplement 10.** Detected and gene annotation of candidate regions by θπ ratios (CCG VS FCG). **Supplement 11.** Detected and gene annotation of candidate regions by XP-EHH (FCG VS CCG). **Supplement 12.** Detected and gene annotation of candidate regions by FST (LCG VS SCG). **Supplement 13.** Detected and gene annotation of candidate regions by θπ ratios (SCG VS LCG). **Supplement 14.** Detected and gene annotation of candidate regions by XP-EHH (LCG VS SCG). **Supplement 15.** Detected and gene annotation of candidate regions by FST (LFCG VS SFCG). **Supplement 16.** Detected and gene annotation of candidate regions by θπ ratios (SFCG VS LFCG). **Supplement 17.** Detected and gene annotation of candidate regions by XP-EHH (LFCG VS SFCG). **Supplement 18.** GO enrichment analysis results of candidate genes in 8 Inner Mongolia cashmere goats subgroup. **Supplement 19.** KEGG enrichment analysis results of candidate genes in 8 Inner Mongolia cashmere goats subgroup.
