# Supplementary material for: Whole-genome selection signatures identified candidate genes associated with cashmere traits in Inner Mongolia cashmere goats
Source: Anim Biosci. 2025 Jul 11;38(12):2597–611. doi: 10.5713/ab.25.0252 (PMC12580777; doi:10.5713/ab.25.0252)
Supplement: Supplementary file 2 [file ab-25-0252-Supplementary-2.pdf]

Supplement 2. High-frequency ROH regions and annotated genes in HYCG

Interval_ID	Chr	Start	End	Length	Gene name
ROH_1	1	51200000	51350000	150001	LOC108636831
ROH_2	1	66100000	66200000	100001	LOC102184002
ROH_2	1	66100000	66200000	100001	ILDR1
ROH_3	1	82500000	82750000	250001	THPO
ROH_3	1	82500000	82750000	250001	CLCN2
ROH_3	1	82500000	82750000	250001	LOC106502122
ROH_3	1	82500000	82750000	250001	PSMD2
ROH_3	1	82500000	82750000	250001	CAMK2N2
ROH_3	1	82500000	82750000	250001	MIR1224
ROH_3	1	82500000	82750000	250001	ABCF3
ROH_3	1	82500000	82750000	250001	LOC102185396
ROH_3	1	82500000	82750000	250001	LOC102185870
ROH_3	1	82500000	82750000	250001	POLR2H
ROH_3	1	82500000	82750000	250001	FAM131A
ROH_3	1	82500000	82750000	250001	EIF4G1
ROH_3	1	82500000	82750000	250001	ALG3
ROH_3	1	82500000	82750000	250001	AP2M1
ROH_3	1	82500000	82750000	250001	DVL3
ROH_3	1	82500000	82750000	250001	EIF2B5
ROH_3	1	82500000	82750000	250001	ECE2
ROH_4	1	84950000	85100000	150001	SOX2
ROH_4	1	84950000	85100000	150001	LOC106502138
ROH_5	1	108600000	109000000	400001	SHOX2
ROH_5	1	108600000	109000000	400001	RSRC1
ROH_6	1	110200000	110400000	200001	LEKR1
ROH_7	1	120850000	121050000	200001	LOC106502391
ROH_8	1	132200000	132400000	200001	STAG1
ROH_10	2	23050000	23200000	150001	CUL3
ROH_11	2	29450000	29650000	200001	GPBAR1
ROH_11	2	29450000	29650000	200001	CXCR1
ROH_11	2	29450000	29650000	200001	AAMP
ROH_11	2	29450000	29650000	200001	ARPC2
ROH_11	2	29450000	29650000	200001	RUFY4
ROH_11	2	29450000	29650000	200001	PNKD
ROH_11	2	29450000	29650000	200001	TMBIM1
ROH_11	2	29450000	29650000	200001	CXCR2
ROH_12	2	64300000	64500000	200001	TRNAC-ACA-7
ROH_12	2	64300000	64500000	200001	EPB41L5
ROH_12	2	64300000	64500000	200001	PTPN4
ROH_14	2	115400000	115450000	50001	LNPK
ROH_15	3	7550000	7700000	150001	USP40
ROH_15	3	7550000	7700000	150001	DGKD
ROH_16	3	23500000	23800000	300001	LOC106501971
ROH_17	3	24750000	24950000	200001	ELAVL4
ROH_18	3	25300000	25400000	100001	FAF1
ROH_19	3	80600000	80750000	150001	RNPC3
ROH_19	3	80600000	80750000	150001	LOC102169641
ROH_20	3	87050000	87250000	200001	CSF1
ROH_20	3	87050000	87250000	200001	STRIP1
ROH_20	3	87050000	87250000	200001	ALX3
ROH_20	3	87050000	87250000	200001	LOC102187445
ROH_20	3	87050000	87250000	200001	AHCYL1
ROH_13	3	95400000	95450000	50001	SPAG17
ROH_22	3	110800000	110950000	150001	CD84
ROH_22	3	110800000	110950000	150001	SLAMF1
ROH_23	4	72000000	72050000	50001	HBP1

ROH_23	4	72000000	72050000	50001	PRKAR2B
ROH_24	4	72800000	73050000	250001	NAMPT
ROH_25	4	75100000	75800000	700001	SLC26A5
ROH_25	4	75100000	75800000	700001	PSMC2
ROH_25	4	75100000	75800000	700001	PMPCB
ROH_25	4	75100000	75800000	700001	DNAJC2
ROH_25	4	75100000	75800000	700001	RELN
ROH_26	5	18650000	18800000	150001	TRNAC-GCA-83
ROH_27	5	20800000	20950000	150001	TRNAC-ACA-25
ROH_27	5	20800000	20950000	150001	LOC108636127
ROH_27	5	20800000	20950000	150001	DCN
ROH_28	5	46950000	47050000	100001	LLPH
ROH_28	5	46950000	47050000	100001	TMBIM4
ROH_28	5	46950000	47050000	100001	IRAK3
ROH_29	5	47250000	47350000	100001	HMGA2
ROH_30	5	55650000	55750000	100001	LOC102180828
ROH_30	5	55650000	55750000	100001	STAT6
ROH_30	5	55650000	55750000	100001	NAB2
ROH_30	5	55650000	55750000	100001	NEMP1
ROH_30	5	55650000	55750000	100001	MYO1A
ROH_30	5	55650000	55750000	100001	TAC3
ROH_31	5	56050000	56100000	50001	LOC102185066
ROH_31	5	56050000	56100000	50001	PRIM1
ROH_31	5	56050000	56100000	50001	NACA
ROH_32	5	56650000	56850000	200001	SUOX
ROH_32	5	56650000	56850000	200001	CDK2
ROH_32	5	56650000	56850000	200001	MMP19
ROH_32	5	56650000	56850000	200001	DNAJC14
ROH_32	5	56650000	56850000	200001	ORMDL2
ROH_32	5	56650000	56850000	200001	RPS26
ROH_32	5	56650000	56850000	200001	IKZF4
ROH_32	5	56650000	56850000	200001	RAB5B
ROH_32	5	56650000	56850000	200001	PMEL
ROH_32	5	56650000	56850000	200001	DGKA
ROH_32	5	56650000	56850000	200001	LOC102177414
ROH_32	5	56650000	56850000	200001	SARNP
ROH_32	5	56650000	56850000	200001	PYM1
ROH_33	5	59450000	59550000	100001	TRNAG-ACC
ROH_33	5	59450000	59550000	100001	CFAP54
ROH_34	5	72600000	72700000	100001	RBFOX2
ROH_35	5	91850000	91900000	50001	LMO3
ROH_36	5	109200000	109350000	150001	TOMM22
ROH_36	5	109200000	109350000	150001	CBY1
ROH_36	5	109200000	109350000	150001	LOC102181663
ROH_36	5	109200000	109350000	150001	JOSD1
ROH_36	5	109200000	109350000	150001	GTPBP1
ROH_36	5	109200000	109350000	150001	SUN2
ROH_36	5	109200000	109350000	150001	DNAL4
ROH_36	5	109200000	109350000	150001	NPTXR
ROH_36	5	109200000	109350000	150001	FAM227A
ROH_37	5	109700000	109850000	150001	TAB1
ROH_37	5	109700000	109850000	150001	MGAT3
ROH_37	5	109700000	109850000	150001	SYNGR1
ROH_38	5	110450000	110550000	100001	TNRC6B
ROH_39	5	111650000	111800000	150001	XRCC6
ROH_39	5	111650000	111800000	150001	MEI1
ROH_39	5	111650000	111800000	150001	CCDC134
ROH_39	5	111650000	111800000	150001	SNU13

ROH_39	5	111650000	111800000	150001	SREBF2
ROH_41	6	19250000	19300000	50001	TBCK
ROH_42	6	32500000	32800000	300001	GRID2
ROH_44	6	70600000	70850000	250001	KIT
ROH_13	6	95400000	95700000	300001	TRNAG-CCC-33
ROH_13	6	95400000	95700000	300001	C6H4orf22
ROH_13	6	95400000	95700000	300001	FGF5
ROH_47	6	111400000	111550000	150001	FGFBP1
ROH_47	6	111400000	111550000	150001	PROM1
ROH_49	7	27500000	27800000	300001	XRCC4
ROH_49	7	27500000	27800000	300001	TMEM167A
ROH_52	7	58900000	59000000	100001	TRNAC-GCA-118
ROH_52	7	58900000	59000000	100001	SLC4A9
ROH_52	7	58900000	59000000	100001	HBEGF
ROH_52	7	58900000	59000000	100001	PFDN1
ROH_53	7	59550000	59750000	200001	LOC108636456
ROH_53	7	59550000	59750000	200001	UBE2D2
ROH_53	7	59550000	59750000	200001	CXXC5
ROH_54	7	59900000	59950000	50001	MATR3
ROH_55	7	62950000	63000000	50001	SMAD5
ROH_56	8	200000	300000	100001	MFSD14B
ROH_58	8	37900000	38050000	150001	LOC102175291
ROH_58	8	37900000	38050000	150001	LOC102176120
ROH_58	8	37900000	38050000	150001	KDM4C
ROH_59	8	38450000	38550000	100001	RANBP6
ROH_59	8	38450000	38550000	100001	KIAA2026
ROH_61	8	43900000	43950000	50001	DOCK8
ROH_62	8	44000000	44350000	350001	LOC102186014
ROH_62	8	44000000	44350000	350001	LOC102185181
ROH_62	8	44000000	44350000	350001	PGM5
ROH_62	8	44000000	44350000	350001	DOCK8
ROH_63	9	56700000	56900000	200001	ARG1
ROH_63	9	56700000	56900000	200001	MED23
ROH_63	9	56700000	56900000	200001	ENPP3
ROH_65	10	94550000	94800000	250001	LOC108636845
ROH_65	10	94550000	94800000	250001	GCNT4
ROH_65	10	94550000	94800000	250001	ANKRD31
ROH_66	11	14350000	15300000	950001	LOC108637104
ROH_66	11	14350000	15300000	950001	DPY30
ROH_66	11	14350000	15300000	950001	SPAST
ROH_66	11	14350000	15300000	950001	SLC30A6
ROH_66	11	14350000	15300000	950001	YIPF4
ROH_66	11	14350000	15300000	950001	MEMO1
ROH_66	11	14350000	15300000	950001	BIRC6
ROH_66	11	14350000	15300000	950001	TTC27
ROH_66	11	14350000	15300000	950001	NLRC4
ROH_67	11	26550000	26600000	50001	CAMKMT
ROH_68	11	40300000	40550000	250001	VRK2
ROH_68	11	40300000	40550000	250001	FANCL
ROH_69	11	43250000	43350000	100001	PUS10
ROH_69	11	43250000	43350000	100001	REL
ROH_70	11	60350000	60450000	100001	B3GNT2
ROH_72	11	71200000	71350000	150001	BRE
ROH_23	11	72000000	72050000	50001	SNX17
ROH_23	11	72000000	72050000	50001	EIF2B4
ROH_23	11	72000000	72050000	50001	MPV17
ROH_23	11	72000000	72050000	50001	PPM1G
ROH_23	11	72000000	72050000	50001	ZNF513

ROH_23	11	72000000	72050000	50001	GTF3C2
ROH_74	11	78150000	78350000	200001	LOC108637157
ROH_74	11	78150000	78350000	200001	SDC1
ROH_74	11	78150000	78350000	200001	PUM2
ROH_75	11	78450000	78700000	250001	TTC32
ROH_75	11	78450000	78700000	250001	LAPTM4A
ROH_75	11	78450000	78700000	250001	MATN3
ROH_75	11	78450000	78700000	250001	WDR35
ROH_76	11	94050000	94150000	100001	DENND1A
ROH_77	11	94250000	94650000	400001	LHX2
ROH_77	11	94250000	94650000	400001	DENND1A
ROH_78	11	96150000	96300000	150001	PBX3
ROH_79	11	96950000	97000000	50001	LMX1B
ROH_80	12	10100000	10150000	50001	LOC102177727
ROH_80	12	10100000	10150000	50001	UBAC2
ROH_81	12	33550000	33900000	350001	FBXL3
ROH_81	12	33550000	33900000	350001	MYCBP2
ROH_83	12	50250000	51400000	1150001	LOC108637296
ROH_83	12	50250000	51400000	1150001	LOC108637298
ROH_83	12	50250000	51400000	1150001	GJB6
ROH_83	12	50250000	51400000	1150001	SAP18
ROH_83	12	50250000	51400000	1150001	TRNAE-UUC-50
ROH_83	12	50250000	51400000	1150001	MRPL57
ROH_83	12	50250000	51400000	1150001	CENPJ
ROH_83	12	50250000	51400000	1150001	MPHOSPH8
ROH_83	12	50250000	51400000	1150001	ZMYM5
ROH_83	12	50250000	51400000	1150001	GJA3
ROH_83	12	50250000	51400000	1150001	GJB2
ROH_83	12	50250000	51400000	1150001	CRYL1
ROH_83	12	50250000	51400000	1150001	IL17D
ROH_83	12	50250000	51400000	1150001	EEF1AKMT1
ROH_83	12	50250000	51400000	1150001	LATS2
ROH_83	12	50250000	51400000	1150001	SKA3
ROH_83	12	50250000	51400000	1150001	ZDHHC20
ROH_83	12	50250000	51400000	1150001	LOC106502707
ROH_83	12	50250000	51400000	1150001	FGF9
ROH_83	12	50250000	51400000	1150001	PSPC1
ROH_83	12	50250000	51400000	1150001	ZMYM2
ROH_83	12	50250000	51400000	1150001	IFT88
ROH_83	12	50250000	51400000	1150001	XPO4
ROH_83	12	50250000	51400000	1150001	MICU2
ROH_83	12	50250000	51400000	1150001	PARP4
ROH_84	12	54600000	54750000	150001	PDX1
ROH_84	12	54600000	54750000	150001	CDX2
ROH_84	12	54600000	54750000	150001	URAD
ROH_84	12	54600000	54750000	150001	FLT3
ROH_85	12	57350000	57500000	150001	RXFP2
ROH_86	12	57550000	57700000	150001	LOC102187143
ROH_86	12	57550000	57700000	150001	FRY
ROH_87	12	60100000	60250000	150001	TRNAC-GCA-168
ROH_87	12	60100000	60250000	150001	LOC102178917
ROH_70	12	60350000	60850000	500001	MAB21L1
ROH_70	12	60350000	60850000	500001	TRNAE-UUC-51
ROH_70	12	60350000	60850000	500001	NBEA
ROH_89	13	53050000	53300000	250001	NPBWR2
ROH_89	13	53050000	53300000	250001	LKAAEAR1
ROH_89	13	53050000	53300000	250001	RGS19
ROH_89	13	53050000	53300000	250001	TCEA2

ROH_89	13	53050000	53300000	250001	SOX18
ROH_89	13	53050000	53300000	250001	SAMD10
ROH_89	13	53050000	53300000	250001	ZNF512B
ROH_89	13	53050000	53300000	250001	MIR1388
ROH_89	13	53050000	53300000	250001	MYT1
ROH_89	13	53050000	53300000	250001	OPRL1
ROH_89	13	53050000	53300000	250001	PRPF6
ROH_89	13	53050000	53300000	250001	DNAJC5
ROH_89	13	53050000	53300000	250001	TPD52L2
ROH_89	13	53050000	53300000	250001	ABHD16B
ROH_89	13	53050000	53300000	250001	LOC108637400
ROH_89	13	53050000	53300000	250001	UCKL1
ROH_90	13	53450000	53550000	100001	PTK6
ROH_90	13	53450000	53550000	100001	SRMS
ROH_90	13	53450000	53550000	100001	PPDPF
ROH_90	13	53450000	53550000	100001	KCNQ2
ROH_90	13	53450000	53550000	100001	EEF1A2
ROH_91	13	53750000	53800000	50001	BHLHE23
ROH_91	13	53750000	53800000	50001	SLC17A9
ROH_91	13	53750000	53800000	50001	LOC108637401
ROH_91	13	53750000	53800000	50001	GID8
ROH_91	13	53750000	53800000	50001	DIDO1
ROH_55	13	62950000	63150000	200001	LOC108637417
ROH_55	13	62950000	63150000	200001	EIF2S2
ROH_55	13	62950000	63150000	200001	RALY
ROH_93	13	63450000	63500000	50001	MAP1LC3A
ROH_93	13	63450000	63500000	50001	DYNLRB1
ROH_93	13	63450000	63500000	50001	ITCH
ROH_94	13	76700000	76750000	50001	CSE1L
ROH_94	13	76700000	76750000	50001	ARFGEF2
ROH_95	13	76800000	76850000	50001	STAU1
ROH_96	14	12650000	12800000	150001	DPY19L4
ROH_96	14	12650000	12800000	150001	CCNE2
ROH_96	14	12650000	12800000	150001	TP53INP1
ROH_96	14	12650000	12800000	150001	INTS8
ROH_97	14	40100000	40200000	100001	IL7
ROH_97	14	40100000	40200000	100001	ZC2HC1A
ROH_98	14	50700000	50800000	100001	ARFGEF1
ROH_98	14	50700000	50800000	100001	CSPP1
ROH_99	15	6300000	6500000	200001	ZNF408
ROH_99	15	6300000	6500000	200001	HARBI1
ROH_99	15	6300000	6500000	200001	TRNAW-CCA-81
ROH_99	15	6300000	6500000	200001	F2
ROH_99	15	6300000	6500000	200001	ARHGAP1
ROH_99	15	6300000	6500000	200001	ATG13
ROH_99	15	6300000	6500000	200001	AMBRA1
ROH_99	15	6300000	6500000	200001	CKAP5
ROH_100	15	23850000	24150000	300001	KIF18A
ROH_100	15	23850000	24150000	300001	METTL15
ROH_101	15	24200000	24250000	50001	KIF18A
ROH_102	15	28800000	28900000	100001	SPCS2
ROH_102	15	28800000	28900000	100001	XRRA1
ROH_102	15	28800000	28900000	100001	RNF169
ROH_103	15	30450000	30550000	100001	FCHSD2
ROH_104	15	31750000	32300000	550001	IL18BP
ROH_104	15	31750000	32300000	550001	LOC102178093
ROH_104	15	31750000	32300000	550001	LOC102177821
ROH_104	15	31750000	32300000	550001	LOC108637670

ROH_104	15	31750000	32300000	550001	PGAP2
ROH_104	15	31750000	32300000	550001	LOC102169978
ROH_104	15	31750000	32300000	550001	ART5
ROH_104	15	31750000	32300000	550001	LOC102169399
ROH_104	15	31750000	32300000	550001	LOC102169116
ROH_104	15	31750000	32300000	550001	ART1
ROH_104	15	31750000	32300000	550001	NUP98
ROH_104	15	31750000	32300000	550001	RHOG
ROH_104	15	31750000	32300000	550001	NUMA1
ROH_104	15	31750000	32300000	550001	RNF121
ROH_104	15	31750000	32300000	550001	STIM1
ROH_105	15	33900000	34200000	300001	LOC102185107
ROH_105	15	33900000	34200000	300001	LOC102184830
ROH_105	15	33900000	34200000	300001	LOC102184268
ROH_105	15	33900000	34200000	300001	LOC102183437
ROH_105	15	33900000	34200000	300001	LOC102176982
ROH_105	15	33900000	34200000	300001	LOC102183163
ROH_105	15	33900000	34200000	300001	LOC102176710
ROH_105	15	33900000	34200000	300001	LOC102174765
ROH_105	15	33900000	34200000	300001	LOC102175876
ROH_105	15	33900000	34200000	300001	LOC102175600
ROH_105	15	33900000	34200000	300001	LOC102182894
ROH_105	15	33900000	34200000	300001	LOC102175317
ROH_105	15	33900000	34200000	300001	HBBC
ROH_105	15	33900000	34200000	300001	LOC102182615
ROH_105	15	33900000	34200000	300001	LOC102176442
ROH_105	15	33900000	34200000	300001	LOC102174495
ROH_105	15	33900000	34200000	300001	LOC102182330
ROH_105	15	33900000	34200000	300001	LOC102182057
ROH_105	15	33900000	34200000	300001	LOC102183709
ROH_106	15	34850000	34950000	100001	LOC102181861
ROH_106	15	34850000	34950000	100001	LOC102180484
ROH_106	15	34850000	34950000	100001	LOC102180752
ROH_106	15	34850000	34950000	100001	LOC102176880
ROH_106	15	34850000	34950000	100001	LOC102182689
ROH_106	15	34850000	34950000	100001	LOC102182401
ROH_107	15	39900000	39950000	50001	SBF2
ROH_108	16	40850000	41100000	250001	ANGPTL7
ROH_108	16	40850000	41100000	250001	SRM
ROH_108	16	40850000	41100000	250001	TARDBP
ROH_108	16	40850000	41100000	250001	UBIAD1
ROH_108	16	40850000	41100000	250001	MASP2
ROH_108	16	40850000	41100000	250001	EXOSC10
ROH_108	16	40850000	41100000	250001	MTOR
ROH_109	16	43200000	43300000	100001	RERE
ROH_110	16	49500000	50000000	500001	FNDC10
ROH_110	16	49500000	50000000	500001	TMEM240
ROH_110	16	49500000	50000000	500001	TMEM88B
ROH_110	16	49500000	50000000	500001	MRPL20
ROH_110	16	49500000	50000000	500001	AURKAIP1
ROH_110	16	49500000	50000000	500001	TAS1R3
ROH_110	16	49500000	50000000	500001	CPTP
ROH_110	16	49500000	50000000	500001	PUSL1
ROH_110	16	49500000	50000000	500001	SCNN1D
ROH_110	16	49500000	50000000	500001	B3GALT6
ROH_110	16	49500000	50000000	500001	TNFRSF4
ROH_110	16	49500000	50000000	500001	TNFRSF18
ROH_110	16	49500000	50000000	500001	MIR429

ROH_110	16	49500000	50000000	500001	MIR200A
ROH_110	16	49500000	50000000	500001	MIR200B
ROH_110	16	49500000	50000000	500001	LOC102169118
ROH_110	16	49500000	50000000	500001	LOC106502959
ROH_110	16	49500000	50000000	500001	ISG15
ROH_110	16	49500000	50000000	500001	HES4
ROH_110	16	49500000	50000000	500001	PERM1
ROH_110	16	49500000	50000000	500001	MIB2
ROH_110	16	49500000	50000000	500001	SSU72
ROH_110	16	49500000	50000000	500001	LOC102189890
ROH_110	16	49500000	50000000	500001	VWA1
ROH_110	16	49500000	50000000	500001	ANKRD65
ROH_110	16	49500000	50000000	500001	CCNL2
ROH_110	16	49500000	50000000	500001	DVL1
ROH_110	16	49500000	50000000	500001	CPSF3L
ROH_110	16	49500000	50000000	500001	ACAP3
ROH_110	16	49500000	50000000	500001	UBE2J2
ROH_110	16	49500000	50000000	500001	FAM132A
ROH_110	16	49500000	50000000	500001	SDF4
ROH_110	16	49500000	50000000	500001	C16H1orf159
ROH_110	16	49500000	50000000	500001	RNF223
ROH_110	16	49500000	50000000	500001	PLEKHN1
ROH_110	16	49500000	50000000	500001	MXRA8
ROH_110	16	49500000	50000000	500001	TTLL10
ROH_110	16	49500000	50000000	500001	AGRN
ROH_111	16	64650000	64700000	50001	RNF2
ROH_112	17	3050000	3100000	50001	CCDC117
ROH_112	17	3050000	3100000	50001	HSCB
ROH_112	17	3050000	3100000	50001	CHEK2
ROH_113	17	15850000	16100000	250001	LOC102182159
ROH_113	17	15850000	16100000	250001	BRAP
ROH_113	17	15850000	16100000	250001	SH2B3
ROH_113	17	15850000	16100000	250001	ATXN2
ROH_115	18	15950000	16100000	250001	CHMP1A
ROH_115	18	15950000	16100000	250001	SPATA2L
ROH_115	18	15950000	16100000	250001	CDK10
ROH_115	18	15950000	16100000	250001	FANCA
ROH_115	18	15950000	16100000	250001	SPIRE2
ROH_115	18	15950000	16100000	250001	TCF25
ROH_115	18	15950000	16100000	250001	ZNF276
ROH_115	18	15950000	16100000	250001	VPS9D1
ROH_116	18	26850000	26900000	50001	CCDC102A
ROH_116	18	26850000	26900000	50001	ADGRG5
ROH_117	18	36050000	36250000	200001	TRADD
ROH_117	18	36050000	36250000	200001	FBXL8
ROH_117	18	36050000	36250000	200001	NOL3
ROH_117	18	36050000	36250000	200001	KIAA0895L
ROH_117	18	36050000	36250000	200001	E2F4
ROH_117	18	36050000	36250000	200001	MIR328
ROH_117	18	36050000	36250000	200001	LRRC29
ROH_117	18	36050000	36250000	200001	B3GNT9
ROH_117	18	36050000	36250000	200001	HSF4
ROH_117	18	36050000	36250000	200001	EXOC3L1
ROH_117	18	36050000	36250000	200001	ELMO3
ROH_117	18	36050000	36250000	200001	TMEM208
ROH_117	18	36050000	36250000	200001	FHOD1
ROH_117	18	36050000	36250000	200001	SLC9A5
ROH_117	18	36050000	36250000	200001	KCTD19

ROH_117	18	36050000	36250000	200001	LRRC36
ROH_117	18	36050000	36250000	200001	C18H16orf70
ROH_117	18	36050000	36250000	200001	PLEKHG4
ROH_118	18	36650000	37150000	500001	TRNAS-AGA-6
ROH_118	18	36650000	37150000	500001	LCAT
ROH_118	18	36650000	37150000	500001	DPEP3
ROH_118	18	36650000	37150000	500001	DPEP2
ROH_118	18	36650000	37150000	500001	LOC106503098
ROH_118	18	36650000	37150000	500001	DDX28
ROH_118	18	36650000	37150000	500001	SLC7A6OS
ROH_118	18	36650000	37150000	500001	LOC102169124
ROH_118	18	36650000	37150000	500001	LOC108637978
ROH_118	18	36650000	37150000	500001	PSKH1
ROH_118	18	36650000	37150000	500001	PSMB10
ROH_118	18	36650000	37150000	500001	DUS2
ROH_118	18	36650000	37150000	500001	ESRP2
ROH_118	18	36650000	37150000	500001	PLA2G15
ROH_118	18	36650000	37150000	500001	SLC7A6
ROH_118	18	36650000	37150000	500001	SMPD3
ROH_118	18	36650000	37150000	500001	LOC108637977
ROH_118	18	36650000	37150000	500001	NFATC3
ROH_118	18	36650000	37150000	500001	PRMT7
ROH_118	18	36650000	37150000	500001	SLC12A4
ROH_119	18	37300000	37350000	50001	LOC102170170
ROH_119	18	37300000	37350000	50001	CDH1
ROH_121	18	57000000	57100000	100001	PRRG2
ROH_121	18	57000000	57100000	100001	RRAS
ROH_121	18	57000000	57100000	100001	IRF3
ROH_121	18	57000000	57100000	100001	PRMT1
ROH_121	18	57000000	57100000	100001	NOSIP
ROH_121	18	57000000	57100000	100001	SCAF1
ROH_121	18	57000000	57100000	100001	BCL2L12
ROH_121	18	57000000	57100000	100001	ADM5
ROH_121	18	57000000	57100000	100001	CPT1C
ROH_121	18	57000000	57100000	100001	PRR12
ROH_122	19	20500000	20650000	150001	TP53I13
ROH_122	19	20500000	20650000	150001	ABHD15
ROH_122	19	20500000	20650000	150001	TAOK1
ROH_123	19	22850000	23000000	150001	OVCA2
ROH_123	19	22850000	23000000	150001	DPH1
ROH_123	19	22850000	23000000	150001	HIC1
ROH_123	19	22850000	23000000	150001	SMG6
ROH_124	20	25400000	25550000	150001	NDUFS4
ROH_125	20	38850000	39000000	150001	PRLR
ROH_127	21	19600000	19800000	200001	ACAN
ROH_127	21	19600000	19800000	200001	HAPLN3
ROH_127	21	19600000	19800000	200001	MFGE8
ROH_128	21	64200000	64400000	200001	MIR342
ROH_128	21	64200000	64400000	200001	DEGS2
ROH_128	21	64200000	64400000	200001	EVL
ROH_129	22	16200000	16350000	150001	ZNF852
ROH_129	22	16200000	16350000	150001	ZNF502
ROH_129	22	16200000	16350000	150001	ZNF501
ROH_129	22	16200000	16350000	150001	KIAA1143
ROH_129	22	16200000	16350000	150001	LOC102177570
ROH_129	22	16200000	16350000	150001	KIF15
ROH_130	22	17250000	17300000	50001	SRGAP3
ROH_131	22	17350000	17550000	200001	SRGAP3

ROH_131	22	17350000	17550000	200001	RAD18
ROH_102	22	28800000	29150000	350001	RYBP
ROH_102	22	28800000	29150000	350001	SHQ1
ROH_133	22	49950000	50000000	500001	CACNA2D2
ROH_134	23	8600000	8900000	300001	JARID2
ROH_134	23	8600000	8900000	300001	DTNBP1
ROH_135	23	22350000	22450000	100001	LOC102180547
ROH_135	23	22350000	22450000	100001	LY6G6D
ROH_135	23	22350000	22450000	100001	LY6G6C
ROH_135	23	22350000	22450000	100001	C23H6orf25
ROH_135	23	22350000	22450000	100001	CLIC1
ROH_135	23	22350000	22450000	100001	SAPCD1
ROH_135	23	22350000	22450000	100001	VARS
ROH_135	23	22350000	22450000	100001	LSM2
ROH_135	23	22350000	22450000	100001	HSP70.1
ROH_135	23	22350000	22450000	100001	ABHD16A
ROH_135	23	22350000	22450000	100001	LY6G6F
ROH_135	23	22350000	22450000	100001	DDAH2
ROH_135	23	22350000	22450000	100001	MSH5
ROH_135	23	22350000	22450000	100001	LOC102177850
ROH_135	23	22350000	22450000	100001	VWA7
ROH_136	23	30200000	30400000	200001	RUNX2
ROH_136	23	30200000	30400000	200001	SUPT3H
ROH_103	23	30450000	30650000	200001	SUPT3H
ROH_138	23	39950000	40100000	150001	C23H6orf106
ROH_138	23	39950000	40100000	150001	SPDEF
ROH_140	24	34750000	34800000	50001	LOC106503532
ROH_140	24	34750000	34800000	50001	MIB1
ROH_141	24	43500000	43900000	400001	MC5R
ROH_141	24	43500000	43900000	400001	PTPN2
ROH_141	24	43500000	43900000	400001	SEH1L
ROH_141	24	43500000	43900000	400001	CEP192
ROH_141	24	43500000	43900000	400001	FAM210A
ROH_141	24	43500000	43900000	400001	RNMT
ROH_141	24	43500000	43900000	400001	LDLRAD4
ROH_141	24	43500000	43900000	400001	MC2R
ROH_142	25	100000	300000	100001	TRNAY-GUA-31
ROH_142	25	100000	300000	100001	LOC108633873
ROH_142	25	100000	300000	100001	LOC108633874
ROH_142	25	100000	300000	100001	HBM
ROH_142	25	100000	300000	100001	LOC102168680
ROH_142	25	100000	300000	100001	LOC102168959
ROH_142	25	100000	300000	100001	LOC102186172
ROH_142	25	100000	300000	100001	RGS11
ROH_142	25	100000	300000	100001	ARHGDIG
ROH_142	25	100000	300000	100001	MRPL28
ROH_142	25	100000	300000	100001	LUC7L
ROH_142	25	100000	300000	100001	FAM234A
ROH_142	25	100000	300000	100001	PDIA2
ROH_142	25	100000	300000	100001	TMEM8A
ROH_142	25	100000	300000	100001	NPRL3
ROH_142	25	100000	300000	100001	AXIN1
ROH_103	26	30450000	30600000	150001	PKD2L1
ROH_103	26	30450000	30600000	150001	BLOC1S2
ROH_103	26	30450000	30600000	150001	CWF19L1
ROH_103	26	30450000	30600000	150001	ERLIN1
ROH_103	26	30450000	30600000	150001	CHUK
ROH_144	26	33350000	33450000	100001	LCOR
