# Supplementary material for: Whole-genome selection signatures identified candidate genes associated with cashmere traits in Inner Mongolia cashmere goats
Source: Anim Biosci. 2025 Jul 11;38(12):2597–611. doi: 10.5713/ab.25.0252 (PMC12580777; doi:10.5713/ab.25.0252)
Supplement: Supplementary file 3 [file ab-25-0252-Supplementary-3.pdf]

Supplement 3. High-frequency ROH regions and annotated genes in FCG

Interval_ID	Chr	Start	End	Length	Gene name
ROH_1	1	42950000	43000000	50001	TRNAE-UUC
ROH_1	1	42950000	43000000	50001	DCBLD2
ROH_2	1	51200000	51250000	50001	LOC108636831
ROH_3	1	82500000	82750000	250001	THPO
ROH_3	1	82500000	82750000	250001	CLCN2
ROH_3	1	82500000	82750000	250001	LOC106502122
ROH_3	1	82500000	82750000	250001	PSMD2
ROH_3	1	82500000	82750000	250001	CAMK2N2
ROH_3	1	82500000	82750000	250001	MIR1224
ROH_3	1	82500000	82750000	250001	ABCF3
ROH_3	1	82500000	82750000	250001	LOC102185396
ROH_3	1	82500000	82750000	250001	LOC102185870
ROH_3	1	82500000	82750000	250001	POLR2H
ROH_3	1	82500000	82750000	250001	FAM131A
ROH_3	1	82500000	82750000	250001	EIF4G1
ROH_3	1	82500000	82750000	250001	ALG3
ROH_3	1	82500000	82750000	250001	AP2M1
ROH_3	1	82500000	82750000	250001	DVL3
ROH_3	1	82500000	82750000	250001	EIF2B5
ROH_3	1	82500000	82750000	250001	ECE2
ROH_4	1	84950000	85100000	150001	SOX2
ROH_4	1	84950000	85100000	150001	LOC106502138
ROH_5	1	108500000	109000000	500001	SHOX2
ROH_5	1	108500000	109000000	500001	RSRC1
ROH_6	1	120800000	121050000	250001	LOC106502391
ROH_7	1	132200000	132400000	200001	STAG1
ROH_8	1	152550000	152650000	100001	LOC108636923
ROH_8	1	152550000	152650000	100001	ANKRD28
ROH_9	1	154150000	154200000	50001	TBC1D5
ROH_11	2	29500000	29600000	100001	GPBAR1
ROH_11	2	29500000	29600000	100001	CXCR1
ROH_11	2	29500000	29600000	100001	AAMP
ROH_11	2	29500000	29600000	100001	ARPC2
ROH_11	2	29500000	29600000	100001	PNKD
ROH_11	2	29500000	29600000	100001	TMBIM1
ROH_12	2	64300000	64550000	250001	TRNAC-ACA-7
ROH_12	2	64300000	64550000	250001	EPB41L5
ROH_12	2	64300000	64550000	250001	PTPN4
ROH_14	3	23600000	23650000	50001	LOC106501971
ROH_15	3	24750000	24950000	200001	ELAVL4
ROH_16	3	25350000	25500000	150001	CDKN2C
ROH_16	3	25350000	25500000	150001	FAF1
ROH_17	3	80600000	80750000	150001	RNPC3
ROH_17	3	80600000	80750000	150001	LOC102169641
ROH_18	3	87100000	87200000	100001	STRIP1
ROH_18	3	87100000	87200000	100001	AHCYL1
ROH_19	3	95350000	95450000	100001	SPAG17
ROH_20	3	106950000	107000000	50001	LOC102183650
ROH_21	3	110800000	110950000	150001	CD84
ROH_21	3	110800000	110950000	150001	SLAMF1
ROH_22	4	68350000	68400000	50001	CAV2
ROH_24	4	72800000	73050000	250001	NAMPT
ROH_26	4	75100000	75450000	350001	RELN
ROH_27	4	75500000	75750000	250001	SLC26A5
ROH_27	4	75500000	75750000	250001	PSMC2
ROH_27	4	75500000	75750000	250001	DNAJC2

ROH_27	4	75500000	75750000	250001	RELN
ROH_28	5	15600000	15800000	200001	MGAT4C
ROH_29	5	18600000	18850000	250001	TRNAC-GCA-83
ROH_30	5	20800000	20950000	150001	TRNAC-ACA-25
ROH_30	5	20800000	20950000	150001	LOC108636127
ROH_30	5	20800000	20950000	150001	DCN
ROH_31	5	47000000	47050000	50001	LLPH
ROH_32	5	47250000	47350000	100001	HMGA2
ROH_33	5	55600000	55700000	100001	TRNAG-CCC-29
ROH_33	5	55600000	55700000	100001	LOC102180828
ROH_33	5	55600000	55700000	100001	STAT6
ROH_33	5	55600000	55700000	100001	NAB2
ROH_33	5	55600000	55700000	100001	NEMP1
ROH_33	5	55600000	55700000	100001	LRP1
ROH_34	5	56050000	56200000	150001	ATP5B
ROH_34	5	56050000	56200000	150001	LOC102185066
ROH_34	5	56050000	56200000	150001	PRIM1
ROH_34	5	56050000	56200000	150001	PTGES3
ROH_34	5	56050000	56200000	150001	BAZ2A
ROH_34	5	56050000	56200000	150001	NACA
ROH_35	5	56750000	56800000	50001	MMP19
ROH_35	5	56750000	56800000	50001	DGKA
ROH_35	5	56750000	56800000	50001	LOC102177414
ROH_35	5	56750000	56800000	50001	PYM1
ROH_36	5	59650000	59800000	150001	CFAP54
ROH_37	5	91850000	91900000	50001	LMO3
ROH_38	5	96100000	96300000	200001	MANSC1
ROH_38	5	96100000	96300000	200001	LRP6
ROH_39	5	109200000	109400000	200001	TOMM22
ROH_39	5	109200000	109400000	200001	CBY1
ROH_39	5	109200000	109400000	200001	LOC102181663
ROH_39	5	109200000	109400000	200001	JOSD1
ROH_39	5	109200000	109400000	200001	GTPBP1
ROH_39	5	109200000	109400000	200001	SUN2
ROH_39	5	109200000	109400000	200001	DNAL4
ROH_39	5	109200000	109400000	200001	NPTXR
ROH_39	5	109200000	109400000	200001	CBX6
ROH_39	5	109200000	109400000	200001	FAM227A
ROH_40	5	109650000	109850000	200001	RPL3
ROH_40	5	109650000	109850000	200001	TAB1
ROH_40	5	109650000	109850000	200001	MGAT3
ROH_40	5	109650000	109850000	200001	SYNGR1
ROH_41	5	110300000	110350000	50001	FAM83F
ROH_41	5	110300000	110350000	50001	TNRC6B
ROH_42	5	110400000	110550000	150001	TNRC6B
ROH_43	5	111650000	111800000	150001	XRCC6
ROH_43	5	111650000	111800000	150001	MEI1
ROH_43	5	111650000	111800000	150001	CCDC134
ROH_43	5	111650000	111800000	150001	SNU13
ROH_43	5	111650000	111800000	150001	SREBF2
ROH_45	6	19100000	19300000	200001	GIMD1
ROH_45	6	19100000	19300000	200001	AIMP1
ROH_45	6	19100000	19300000	200001	TBCK
ROH_46	6	30800000	30950000	150001	HPGDS
ROH_46	6	30800000	30950000	150001	SMARCAD1
ROH_47	6	32500000	32550000	50001	GRID2
ROH_48	6	32650000	32850000	200001	GRID2
ROH_49	6	69450000	69600000	150001	FIP1L1

ROH_49	6	69450000	69600000	150001	LNX1
ROH_49	6	69450000	69600000	150001	SCFD2
ROH_50	6	70650000	70850000	200001	KIT
ROH_52	6	95350000	95650000	300001	TRNAG-CCC-33
ROH_52	6	95350000	95650000	300001	PRDM8
ROH_52	6	95350000	95650000	300001	C6H4orf22
ROH_52	6	95350000	95650000	300001	FGF5
ROH_53	6	111400000	111550000	150001	FGFBP1
ROH_53	6	111400000	111550000	150001	PROM1
ROH_55	7	27400000	27450000	50001	XRCC4
ROH_56	7	27500000	27750000	250001	XRCC4
ROH_56	7	27500000	27750000	250001	TMEM167A
ROH_57	7	31300000	31350000	50001	LOC102187402
ROH_60	7	58800000	59000000	200001	TRNAC-GCA-118
ROH_60	7	58800000	59000000	200001	SLC4A9
ROH_60	7	58800000	59000000	200001	HBEGF
ROH_60	7	58800000	59000000	200001	PFDN1
ROH_60	7	58800000	59000000	200001	LOC102175120
ROH_61	7	59750000	59950000	200001	TMEM173
ROH_61	7	59750000	59950000	200001	ECSCR
ROH_61	7	59750000	59950000	200001	SPATA24
ROH_61	7	59750000	59950000	200001	PROB1
ROH_61	7	59750000	59950000	200001	DNAJC18
ROH_61	7	59750000	59950000	200001	MZB1
ROH_61	7	59750000	59950000	200001	SLC23A1
ROH_61	7	59750000	59950000	200001	PAIP2
ROH_61	7	59750000	59950000	200001	MATR3
ROH_62	7	62850000	63000000	150001	TRPC7
ROH_62	7	62850000	63000000	150001	SMAD5
ROH_63	7	72150000	72300000	150001	HK3
ROH_63	7	72150000	72300000	150001	UNC5A
ROH_63	7	72150000	72300000	150001	UIMC1
ROH_64	7	91800000	91950000	150001	TRNAK-UUU-10
ROH_64	7	91800000	91950000	150001	C7H19orf70
ROH_64	7	91800000	91950000	150001	HSD11B1L
ROH_64	7	91800000	91950000	150001	LOC106502310
ROH_64	7	91800000	91950000	150001	SAFB2
ROH_64	7	91800000	91950000	150001	RPL36
ROH_64	7	91800000	91950000	150001	LONP1
ROH_64	7	91800000	91950000	150001	CATSPERD
ROH_64	7	91800000	91950000	150001	SAFB
ROH_65	8	150000	350000	200001	LOC102190689
ROH_65	8	150000	350000	200001	MFSD14B
ROH_67	8	38400000	38750000	350001	RANBP6
ROH_67	8	38400000	38750000	350001	MLANA
ROH_67	8	38400000	38750000	350001	ERMP1
ROH_67	8	38400000	38750000	350001	IL33
ROH_67	8	38400000	38750000	350001	KIAA2026
ROH_69	8	44000000	44350000	350001	LOC102186014
ROH_69	8	44000000	44350000	350001	LOC102185181
ROH_69	8	44000000	44350000	350001	PGM5
ROH_69	8	44000000	44350000	350001	DOCK8
ROH_70	9	300000	450000	150001	SLC17A5
ROH_70	9	300000	450000	150001	CD109
ROH_71	9	56700000	57100000	400001	ARG1
ROH_71	9	56700000	57100000	400001	MED23
ROH_71	9	56700000	57100000	400001	ENPP3
ROH_71	9	56700000	57100000	400001	ENPP1

ROH_72	9	74800000	74850000	50001	IYD
ROH_73	9	76150000	76250000	100001	ESR1
ROH_75	10	82350000	82500000	150001	MYO9A
ROH_76	10	94600000	94800000	200001	LOC108636845
ROH_76	10	94600000	94800000	200001	GCNT4
ROH_76	10	94600000	94800000	200001	ANKRD31
ROH_77	11	14350000	15300000	950001	LOC108637104
ROH_77	11	14350000	15300000	950001	DPY30
ROH_77	11	14350000	15300000	950001	SPAST
ROH_77	11	14350000	15300000	950001	SLC30A6
ROH_77	11	14350000	15300000	950001	YIPF4
ROH_77	11	14350000	15300000	950001	MEMO1
ROH_77	11	14350000	15300000	950001	BIRC6
ROH_77	11	14350000	15300000	950001	TTC27
ROH_77	11	14350000	15300000	950001	NLRC4
ROH_78	11	40300000	40500000	200001	VRK2
ROH_78	11	40300000	40500000	200001	FANCL
ROH_79	11	48900000	49000000	100001	TMEM150A
ROH_79	11	48900000	49000000	100001	VAMP8
ROH_79	11	48900000	49000000	100001	C11H2orf68
ROH_79	11	48900000	49000000	100001	RNF181
ROH_79	11	48900000	49000000	100001	VAMP5
ROH_79	11	48900000	49000000	100001	GGCX
ROH_79	11	48900000	49000000	100001	MAT2A
ROH_79	11	48900000	49000000	100001	USP39
ROH_80	11	60350000	60500000	150001	B3GNT2
ROH_81	11	71200000	71250000	50001	BRE
ROH_82	11	72000000	72050000	50001	SNX17
ROH_82	11	72000000	72050000	50001	EIF2B4
ROH_82	11	72000000	72050000	50001	MPV17
ROH_82	11	72000000	72050000	50001	PPM1G
ROH_82	11	72000000	72050000	50001	ZNF513
ROH_82	11	72000000	72050000	50001	GTF3C2
ROH_83	11	78100000	78400000	300001	RHOB
ROH_83	11	78100000	78400000	300001	LOC108637157
ROH_83	11	78100000	78400000	300001	SDC1
ROH_83	11	78100000	78400000	300001	LOC106502652
ROH_83	11	78100000	78400000	300001	PUM2
ROH_84	11	78450000	78700000	250001	TTC32
ROH_84	11	78450000	78700000	250001	LAPTM4A
ROH_84	11	78450000	78700000	250001	MATN3
ROH_84	11	78450000	78700000	250001	WDR35
ROH_85	11	94300000	94650000	350001	LHX2
ROH_85	11	94300000	94650000	350001	DENND1A
ROH_86	11	96150000	96300000	150001	PBX3
ROH_87	12	10100000	10150000	50001	LOC102177727
ROH_87	12	10100000	10150000	50001	UBAC2
ROH_88	12	33550000	33900000	350001	FBXL3
ROH_88	12	33550000	33900000	350001	MYCBP2
ROH_92	12	50050000	51400000	1350001	LOC108637296
ROH_92	12	50050000	51400000	1350001	LOC108637298
ROH_92	12	50050000	51400000	1350001	GJB6
ROH_92	12	50050000	51400000	1350001	SAP18
ROH_92	12	50050000	51400000	1350001	TRNAE-UUC-50
ROH_92	12	50050000	51400000	1350001	MRPL57
ROH_92	12	50050000	51400000	1350001	ATP12A
ROH_92	12	50050000	51400000	1350001	CENPJ
ROH_92	12	50050000	51400000	1350001	MPHOSPH8

ROH_92	12	50050000	51400000	1350001	ZMYM5
ROH_92	12	50050000	51400000	1350001	GJA3
ROH_92	12	50050000	51400000	1350001	GJB2
ROH_92	12	50050000	51400000	1350001	CRYL1
ROH_92	12	50050000	51400000	1350001	IL17D
ROH_92	12	50050000	51400000	1350001	EEF1AKMT1
ROH_92	12	50050000	51400000	1350001	LATS2
ROH_92	12	50050000	51400000	1350001	SKA3
ROH_92	12	50050000	51400000	1350001	ZDHHC20
ROH_92	12	50050000	51400000	1350001	LOC106502707
ROH_92	12	50050000	51400000	1350001	FGF9
ROH_92	12	50050000	51400000	1350001	RNF17
ROH_92	12	50050000	51400000	1350001	PSPC1
ROH_92	12	50050000	51400000	1350001	ZMYM2
ROH_92	12	50050000	51400000	1350001	IFT88
ROH_92	12	50050000	51400000	1350001	XPO4
ROH_92	12	50050000	51400000	1350001	MICU2
ROH_92	12	50050000	51400000	1350001	PARP4
ROH_93	12	54600000	54650000	50001	PDX1
ROH_93	12	54600000	54650000	50001	CDX2
ROH_93	12	54600000	54650000	50001	URAD
ROH_94	12	54700000	54900000	200001	FLT3
ROH_94	12	54700000	54900000	200001	PAN3
ROH_94	12	54700000	54900000	200001	FLT1
ROH_95	12	57350000	57700000	350001	LOC102187143
ROH_95	12	57350000	57700000	350001	RXFP2
ROH_95	12	57350000	57700000	350001	FRY
ROH_96	12	60350000	60900000	550001	MAB21L1
ROH_96	12	60350000	60900000	550001	TRNAE-UUC-51
ROH_96	12	60350000	60900000	550001	NBEA
ROH_97	13	39800000	39950000	150001	KIZ
ROH_98	13	51200000	51350000	150001	TRNAS-GGA-72
ROH_98	13	51200000	51350000	150001	ITPA
ROH_98	13	51200000	51350000	150001	DDRGK1
ROH_98	13	51200000	51350000	150001	SLC4A11
ROH_98	13	51200000	51350000	150001	C13H20orf194
ROH_99	13	53100000	53300000	200001	LKAAEAR1
ROH_99	13	53100000	53300000	200001	RGS19
ROH_99	13	53100000	53300000	200001	TCEA2
ROH_99	13	53100000	53300000	200001	SOX18
ROH_99	13	53100000	53300000	200001	SAMD10
ROH_99	13	53100000	53300000	200001	ZNF512B
ROH_99	13	53100000	53300000	200001	MIR1388
ROH_99	13	53100000	53300000	200001	OPRL1
ROH_99	13	53100000	53300000	200001	PRPF6
ROH_99	13	53100000	53300000	200001	DNAJC5
ROH_99	13	53100000	53300000	200001	TPD52L2
ROH_99	13	53100000	53300000	200001	ABHD16B
ROH_99	13	53100000	53300000	200001	LOC108637400
ROH_99	13	53100000	53300000	200001	UCKL1
ROH_100	13	53750000	53900000	150001	BHLHE23
ROH_100	13	53750000	53900000	150001	OGFR
ROH_100	13	53750000	53900000	150001	SLC17A9
ROH_100	13	53750000	53900000	150001	LOC108637401
ROH_100	13	53750000	53900000	150001	GID8
ROH_100	13	53750000	53900000	150001	DIDO1
ROH_100	13	53750000	53900000	150001	TCFL5
ROH_100	13	53750000	53900000	150001	MRGBP

ROH_100	13	53750000	53900000	150001	COL9A3
ROH_101	13	62500000	62550000	50001	SNTA1
ROH_101	13	62500000	62550000	50001	CBFA2T2
ROH_102	13	62950000	63100000	150001	LOC108637417
ROH_102	13	62950000	63100000	150001	EIF2S2
ROH_102	13	62950000	63100000	150001	RALY
ROH_103	13	63450000	63600000	150001	MAP1LC3A
ROH_103	13	63450000	63600000	150001	DYNLRB1
ROH_103	13	63450000	63600000	150001	ITCH
ROH_103	13	63450000	63600000	150001	PIGU
ROH_104	13	76700000	76850000	150001	CSE1L
ROH_104	13	76700000	76850000	150001	ARFGEF2
ROH_104	13	76700000	76850000	150001	STAU1
ROH_105	14	12650000	12850000	200001	DPY19L4
ROH_105	14	12650000	12850000	200001	CCNE2
ROH_105	14	12650000	12850000	200001	TP53INP1
ROH_105	14	12650000	12850000	200001	INTS8
ROH_105	14	12650000	12850000	200001	LOC102184166
ROH_106	14	17300000	17350000	50001	VPS13B
ROH_107	14	17400000	17450000	50001	VPS13B
ROH_108	14	40100000	40300000	200001	IL7
ROH_108	14	40100000	40300000	200001	ZC2HC1A
ROH_108	14	40100000	40300000	200001	PKIA
ROH_109	14	45000000	45050000	50001	STAU2
ROH_110	14	45100000	45200000	100001	STAU2
ROH_111	14	50650000	50800000	150001	ARFGEF1
ROH_111	14	50650000	50800000	150001	CSPP1
ROH_112	15	6350000	6400000	50001	ARHGAP1
ROH_112	15	6350000	6400000	50001	ATG13
ROH_113	15	6450000	6500000	50001	AMBRA1
ROH_114	15	24100000	24150000	50001	KIF18A
ROH_114	15	24100000	24150000	50001	METTL15
ROH_115	15	30350000	30550000	200001	FCHSD2
ROH_116	15	31900000	32300000	400001	LOC102178093
ROH_116	15	31900000	32300000	400001	LOC102177821
ROH_116	15	31900000	32300000	400001	LOC108637670
ROH_116	15	31900000	32300000	400001	PGAP2
ROH_116	15	31900000	32300000	400001	LOC102169978
ROH_116	15	31900000	32300000	400001	ART5
ROH_116	15	31900000	32300000	400001	LOC102169399
ROH_116	15	31900000	32300000	400001	LOC102169116
ROH_116	15	31900000	32300000	400001	ART1
ROH_116	15	31900000	32300000	400001	NUP98
ROH_116	15	31900000	32300000	400001	RHOG
ROH_116	15	31900000	32300000	400001	STIM1
ROH_117	15	33900000	34200000	300001	LOC102185107
ROH_117	15	33900000	34200000	300001	LOC102184830
ROH_117	15	33900000	34200000	300001	LOC102184268
ROH_117	15	33900000	34200000	300001	LOC102183437
ROH_117	15	33900000	34200000	300001	LOC102176982
ROH_117	15	33900000	34200000	300001	LOC102183163
ROH_117	15	33900000	34200000	300001	LOC102176710
ROH_117	15	33900000	34200000	300001	LOC102174765
ROH_117	15	33900000	34200000	300001	LOC102175876
ROH_117	15	33900000	34200000	300001	LOC102175600
ROH_117	15	33900000	34200000	300001	LOC102182894
ROH_117	15	33900000	34200000	300001	LOC102175317
ROH_117	15	33900000	34200000	300001	HBBC

ROH_117	15	33900000	34200000	300001	LOC102182615
ROH_117	15	33900000	34200000	300001	LOC102176442
ROH_117	15	33900000	34200000	300001	LOC102174495
ROH_117	15	33900000	34200000	300001	LOC102182330
ROH_117	15	33900000	34200000	300001	LOC102182057
ROH_117	15	33900000	34200000	300001	LOC102183709
ROH_118	15	34850000	34950000	100001	LOC102181861
ROH_118	15	34850000	34950000	100001	LOC102180484
ROH_118	15	34850000	34950000	100001	LOC102180752
ROH_118	15	34850000	34950000	100001	LOC102176880
ROH_118	15	34850000	34950000	100001	LOC102182689
ROH_118	15	34850000	34950000	100001	LOC102182401
ROH_119	16	32100000	32300000	200001	AKT3
ROH_119	16	32100000	32300000	200001	SDCCAG8
ROH_120	16	33600000	33800000	200001	OPN3
ROH_120	16	33600000	33800000	200001	KMO
ROH_120	16	33600000	33800000	200001	CHML
ROH_120	16	33600000	33800000	200001	FH
ROH_120	16	33600000	33800000	200001	WDR64
ROH_121	16	35900000	36000000	100001	METTL18
ROH_121	16	35900000	36000000	100001	SELE
ROH_121	16	35900000	36000000	100001	C16H1orf112
ROH_122	16	40900000	40950000	50001	ANGPTL7
ROH_122	16	40900000	40950000	50001	MTOR
ROH_123	16	41700000	41750000	50001	KIF1B
ROH_124	16	49500000	50000000	500001	FNDC10
ROH_124	16	49500000	50000000	500001	TMEM240
ROH_124	16	49500000	50000000	500001	TMEM88B
ROH_124	16	49500000	50000000	500001	MRPL20
ROH_124	16	49500000	50000000	500001	AURKAIP1
ROH_124	16	49500000	50000000	500001	TAS1R3
ROH_124	16	49500000	50000000	500001	CPTP
ROH_124	16	49500000	50000000	500001	PUSL1
ROH_124	16	49500000	50000000	500001	SCNN1D
ROH_124	16	49500000	50000000	500001	B3GALT6
ROH_124	16	49500000	50000000	500001	TNFRSF4
ROH_124	16	49500000	50000000	500001	TNFRSF18
ROH_124	16	49500000	50000000	500001	MIR429
ROH_124	16	49500000	50000000	500001	MIR200A
ROH_124	16	49500000	50000000	500001	MIR200B
ROH_124	16	49500000	50000000	500001	LOC102169118
ROH_124	16	49500000	50000000	500001	LOC106502959
ROH_124	16	49500000	50000000	500001	ISG15
ROH_124	16	49500000	50000000	500001	HES4
ROH_124	16	49500000	50000000	500001	PERM1
ROH_124	16	49500000	50000000	500001	MIB2
ROH_124	16	49500000	50000000	500001	SSU72
ROH_124	16	49500000	50000000	500001	LOC102189890
ROH_124	16	49500000	50000000	500001	VWA1
ROH_124	16	49500000	50000000	500001	ANKRD65
ROH_124	16	49500000	50000000	500001	CCNL2
ROH_124	16	49500000	50000000	500001	DVL1
ROH_124	16	49500000	50000000	500001	CPSF3L
ROH_124	16	49500000	50000000	500001	ACAP3
ROH_124	16	49500000	50000000	500001	UBE2J2
ROH_124	16	49500000	50000000	500001	FAM132A
ROH_124	16	49500000	50000000	500001	SDF4
ROH_124	16	49500000	50000000	500001	C16H1orf159

ROH_124	16	49500000	50000000	500001	RNF223
ROH_124	16	49500000	50000000	500001	PLEKHN1
ROH_124	16	49500000	50000000	500001	MXRA8
ROH_124	16	49500000	50000000	500001	TTLL10
ROH_124	16	49500000	50000000	500001	AGRN
ROH_125	16	64650000	64750000	100001	RNF2
ROH_125	16	64650000	64750000	100001	TRMT1L
ROH_126	16	79200000	79300000	100001	LOC108637802
ROH_126	16	79200000	79300000	100001	CSRP1
ROH_126	16	79200000	79300000	100001	PHLDA3
ROH_127	17	2750000	2850000	100001	LOC102189423
ROH_127	17	2750000	2850000	100001	EMID1
ROH_127	17	2750000	2850000	100001	KREMEN1
ROH_127	17	2750000	2850000	100001	C17H22orf31
ROH_129	18	15950000	16100000	150001	CHMP1A
ROH_129	18	15950000	16100000	150001	SPATA2L
ROH_129	18	15950000	16100000	150001	CDK10
ROH_129	18	15950000	16100000	150001	FANCA
ROH_129	18	15950000	16100000	150001	SPIRE2
ROH_129	18	15950000	16100000	150001	TCF25
ROH_129	18	15950000	16100000	150001	ZNF276
ROH_129	18	15950000	16100000	150001	VPS9D1
ROH_130	18	26850000	26900000	50001	CCDC102A
ROH_130	18	26850000	26900000	50001	ADGRG5
ROH_131	18	36050000	36150000	100001	TRADD
ROH_131	18	36050000	36150000	100001	FBXL8
ROH_131	18	36050000	36150000	100001	NOL3
ROH_131	18	36050000	36150000	100001	KIAA0895L
ROH_131	18	36050000	36150000	100001	E2F4
ROH_131	18	36050000	36150000	100001	MIR328
ROH_131	18	36050000	36150000	100001	LRRC29
ROH_131	18	36050000	36150000	100001	B3GNT9
ROH_131	18	36050000	36150000	100001	HSF4
ROH_131	18	36050000	36150000	100001	EXOC3L1
ROH_131	18	36050000	36150000	100001	ELMO3
ROH_131	18	36050000	36150000	100001	TMEM208
ROH_131	18	36050000	36150000	100001	FHOD1
ROH_131	18	36050000	36150000	100001	SLC9A5
ROH_131	18	36050000	36150000	100001	C18H16orf70
ROH_132	18	36650000	37150000	500001	TRNAS-AGA-6
ROH_132	18	36650000	37150000	500001	LCAT
ROH_132	18	36650000	37150000	500001	DPEP3
ROH_132	18	36650000	37150000	500001	DPEP2
ROH_132	18	36650000	37150000	500001	LOC106503098
ROH_132	18	36650000	37150000	500001	DDX28
ROH_132	18	36650000	37150000	500001	SLC7A6OS
ROH_132	18	36650000	37150000	500001	LOC102169124
ROH_132	18	36650000	37150000	500001	LOC108637978
ROH_132	18	36650000	37150000	500001	PSKH1
ROH_132	18	36650000	37150000	500001	PSMB10
ROH_132	18	36650000	37150000	500001	DUS2
ROH_132	18	36650000	37150000	500001	ESRP2
ROH_132	18	36650000	37150000	500001	PLA2G15
ROH_132	18	36650000	37150000	500001	SLC7A6
ROH_132	18	36650000	37150000	500001	SMPD3
ROH_132	18	36650000	37150000	500001	LOC108637977
ROH_132	18	36650000	37150000	500001	NFATC3
ROH_132	18	36650000	37150000	500001	PRMT7

ROH_132	18	36650000	37150000	500001	SLC12A4
ROH_133	18	37300000	37350000	50001	LOC102170170
ROH_133	18	37300000	37350000	50001	CDH1
ROH_135	18	51850000	51950000	100001	PRR19
ROH_135	18	51850000	51950000	100001	ERF
ROH_135	18	51850000	51950000	100001	GSK3A
ROH_135	18	51850000	51950000	100001	MEGF8
ROH_135	18	51850000	51950000	100001	TMEM145
ROH_135	18	51850000	51950000	100001	PAFAH1B3
ROH_135	18	51850000	51950000	100001	CIC
ROH_135	18	51850000	51950000	100001	ZNF526
ROH_135	18	51850000	51950000	100001	DEDD2
ROH_136	18	57050000	57100000	50001	RRAS
ROH_136	18	57050000	57100000	50001	IRF3
ROH_136	18	57050000	57100000	50001	PRMT1
ROH_136	18	57050000	57100000	50001	SCAF1
ROH_136	18	57050000	57100000	50001	BCL2L12
ROH_136	18	57050000	57100000	50001	ADM5
ROH_136	18	57050000	57100000	50001	CPT1C
ROH_137	19	20500000	20550000	50001	TAOK1
ROH_138	19	22800000	23000000	200001	OVCA2
ROH_138	19	22800000	23000000	200001	DPH1
ROH_138	19	22800000	23000000	200001	HIC1
ROH_138	19	22800000	23000000	200001	RTN4RL1
ROH_138	19	22800000	23000000	200001	SMG6
ROH_139	19	27100000	27200000	100001	ATP1B2
ROH_139	19	27100000	27200000	100001	EFNB3
ROH_139	19	27100000	27200000	100001	TP53
ROH_139	19	27100000	27200000	100001	DNAH2
ROH_139	19	27100000	27200000	100001	WRAP53
ROH_140	20	25400000	25550000	150001	NDUFS4
ROH_141	20	38850000	39000000	150001	PRLR
ROH_142	21	44850000	45000000	150001	PSMA6
ROH_142	21	44850000	45000000	150001	LOC108638466
ROH_142	21	44850000	45000000	150001	KIAA0391
ROH_142	21	44850000	45000000	150001	LOC108638517
ROH_143	21	64250000	64400000	150001	MIR342
ROH_143	21	64250000	64400000	150001	DEGS2
ROH_143	21	64250000	64400000	150001	EVL
ROH_144	22	16200000	16350000	150001	ZNF852
ROH_144	22	16200000	16350000	150001	ZNF502
ROH_144	22	16200000	16350000	150001	ZNF501
ROH_144	22	16200000	16350000	150001	KIAA1143
ROH_144	22	16200000	16350000	150001	LOC102177570
ROH_144	22	16200000	16350000	150001	KIF15
ROH_145	22	16700000	16850000	150001	LOC108633373
ROH_145	22	16700000	16850000	150001	TADA3
ROH_145	22	16700000	16850000	150001	OGG1
ROH_145	22	16700000	16850000	150001	RPUSD3
ROH_145	22	16700000	16850000	150001	LOC102184009
ROH_145	22	16700000	16850000	150001	ARPC4
ROH_145	22	16700000	16850000	150001	BRPF1
ROH_145	22	16700000	16850000	150001	CPNE9
ROH_145	22	16700000	16850000	150001	MTMR14
ROH_145	22	16700000	16850000	150001	CAMK1
ROH_146	22	17150000	17600000	450001	OXTR
ROH_146	22	17150000	17600000	450001	SRGAP3
ROH_146	22	17150000	17600000	450001	RAD18

ROH_147	22	29100000	29150000	50001	RYBP
ROH_148	22	49900000	49950000	50001	CACNA2D2
ROH_149	23	8600000	8850000	250001	JARID2
ROH_150	23	19350000	19500000	150001	ZSCAN16
ROH_150	23	19350000	19500000	150001	LOC102177207
ROH_150	23	19350000	19500000	150001	TRNAS-GCU-17
ROH_150	23	19350000	19500000	150001	ZSCAN9
ROH_150	23	19350000	19500000	150001	ZKSCAN4
ROH_150	23	19350000	19500000	150001	ZNF165
ROH_150	23	19350000	19500000	150001	LOC108633410
ROH_150	23	19350000	19500000	150001	LOC108633417
ROH_150	23	19350000	19500000	150001	LOC108633266
ROH_150	23	19350000	19500000	150001	ZKSCAN8
ROH_151	23	30450000	30650000	200001	SUPT3H
ROH_152	23	39950000	40100000	150001	C23H6orf106
ROH_152	23	39950000	40100000	150001	SPDEF
ROH_153	24	34650000	34800000	150001	LOC106503532
ROH_153	24	34650000	34800000	150001	MIB1
ROH_154	24	43500000	43900000	400001	MC5R
ROH_154	24	43500000	43900000	400001	PTPN2
ROH_154	24	43500000	43900000	400001	SEH1L
ROH_154	24	43500000	43900000	400001	CEP192
ROH_154	24	43500000	43900000	400001	FAM210A
ROH_154	24	43500000	43900000	400001	RNMT
ROH_154	24	43500000	43900000	400001	LDLRAD4
ROH_154	24	43500000	43900000	400001	MC2R
ROH_155	25	100000	350000	250001	TRNAY-GUA-31
ROH_155	25	100000	350000	250001	LOC108633873
ROH_155	25	100000	350000	250001	LOC108633874
ROH_155	25	100000	350000	250001	HBM
ROH_155	25	100000	350000	250001	LOC102168680
ROH_155	25	100000	350000	250001	LOC102168959
ROH_155	25	100000	350000	250001	LOC102186172
ROH_155	25	100000	350000	250001	RGS11
ROH_155	25	100000	350000	250001	ARHGDIG
ROH_155	25	100000	350000	250001	MRPL28
ROH_155	25	100000	350000	250001	NME4
ROH_155	25	100000	350000	250001	LUC7L
ROH_155	25	100000	350000	250001	FAM234A
ROH_155	25	100000	350000	250001	PDIA2
ROH_155	25	100000	350000	250001	TMEM8A
ROH_155	25	100000	350000	250001	DECR2
ROH_155	25	100000	350000	250001	NPRL3
ROH_155	25	100000	350000	250001	AXIN1
ROH_155	25	100000	350000	250001	RAB11FIP3
ROH_156	25	400000	450000	50001	PRR35
ROH_156	25	400000	450000	50001	NHLRC4
ROH_156	25	400000	450000	50001	CAPN15
ROH_156	25	400000	450000	50001	PIGQ
ROH_156	25	400000	450000	50001	RAB11FIP3
ROH_157	26	33350000	33450000	100001	LCOR
ROH_158	28	21050000	21200000	150001	HERC4
