# Supplementary material for: Whole-genome selection signatures identified candidate genes associated with cashmere traits in Inner Mongolia cashmere goats
Source: Anim Biosci. 2025 Jul 11;38(12):2597–611. doi: 10.5713/ab.25.0252 (PMC12580777; doi:10.5713/ab.25.0252)
Supplement: Supplementary file 4 [file ab-25-0252-Supplementary-4.pdf]

Supplement 4. High-frequency ROH regions and annotated genes in LCG

Interval_ID	Chr	Start	End	Length	Gene name
ROH_1	1	70000000	70050000	50001	LRCH3
ROH_2	1	70100000	70150000	50001	LRCH3
ROH_2	1	70100000	70150000	50001	FYTTD1
ROH_3	1	82500000	82750000	250001	THPO
ROH_3	1	82500000	82750000	250001	CLCN2
ROH_3	1	82500000	82750000	250001	LOC106502122
ROH_3	1	82500000	82750000	250001	PSMD2
ROH_3	1	82500000	82750000	250001	CAMK2N2
ROH_3	1	82500000	82750000	250001	MIR1224
ROH_3	1	82500000	82750000	250001	ABCF3
ROH_3	1	82500000	82750000	250001	LOC102185396
ROH_3	1	82500000	82750000	250001	LOC102185870
ROH_3	1	82500000	82750000	250001	POLR2H
ROH_3	1	82500000	82750000	250001	FAM131A
ROH_3	1	82500000	82750000	250001	EIF4G1
ROH_3	1	82500000	82750000	250001	ALG3
ROH_3	1	82500000	82750000	250001	AP2M1
ROH_3	1	82500000	82750000	250001	DVL3
ROH_3	1	82500000	82750000	250001	EIF2B5
ROH_3	1	82500000	82750000	250001	ECE2
ROH_4	1	84950000	85100000	150001	SOX2
ROH_4	1	84950000	85100000	150001	LOC106502138
ROH_5	1	108650000	109000000	350001	SHOX2
ROH_5	1	108650000	109000000	350001	RSRC1
ROH_6	1	118600000	118750000	150001	CP
ROH_6	1	118600000	118750000	150001	HLTF
ROH_6	1	118600000	118750000	150001	HPS3
ROH_6	1	118600000	118750000	150001	GYG1
ROH_7	1	120850000	121000000	150001	LOC106502391
ROH_9	1	132150000	132500000	350001	PCCB
ROH_9	1	132150000	132500000	350001	STAG1
ROH_10	1	152700000	152750000	50001	ANKRD28
ROH_12	2	23050000	23150000	100001	CUL3
ROH_13	2	29450000	29600000	150001	GPBAR1
ROH_13	2	29450000	29600000	150001	CXCR1
ROH_13	2	29450000	29600000	150001	AAMP
ROH_13	2	29450000	29600000	150001	ARPC2
ROH_13	2	29450000	29600000	150001	PNKD
ROH_13	2	29450000	29600000	150001	TMBIM1
ROH_14	2	74800000	74850000	50001	DARS
ROH_18	3	16750000	16850000	100001	GUCA2B
ROH_18	3	16750000	16850000	100001	GUCA2A
ROH_18	3	16750000	16850000	100001	FOXJ3
ROH_19	3	23600000	23650000	50001	LOC106501971
ROH_20	3	24750000	24950000	200001	ELAVL4
ROH_21	3	25300000	25400000	100001	FAF1
ROH_22	3	80600000	80750000	150001	RNPC3
ROH_22	3	80600000	80750000	150001	LOC102169641
ROH_23	3	87100000	87200000	100001	STRIP1
ROH_23	3	87100000	87200000	100001	AHCYL1
ROH_24	3	95400000	95450000	50001	SPAG17
ROH_25	4	71850000	71900000	50001	COG5
ROH_26	4	72000000	72050000	50001	HBP1
ROH_26	4	72000000	72050000	50001	PRKAR2B
ROH_27	4	72800000	73050000	250001	NAMPT
ROH_28	4	75050000	75800000	750001	SLC26A5

ROH_28	4	75050000	75800000	750001	PSMC2
ROH_28	4	75050000	75800000	750001	PMPCB
ROH_28	4	75050000	75800000	750001	DNAJC2
ROH_28	4	75050000	75800000	750001	RELN
ROH_29	5	18600000	18850000	250001	TRNAC-GCA-83
ROH_30	5	20850000	20900000	50001	TRNAC-ACA-25
ROH_30	5	20850000	20900000	50001	LOC108636127
ROH_30	5	20850000	20900000	50001	DCN
ROH_31	5	46950000	47050000	100001	LLPH
ROH_31	5	46950000	47050000	100001	TMBIM4
ROH_31	5	46950000	47050000	100001	IRAK3
ROH_32	5	47250000	47350000	100001	HMGA2
ROH_33	5	55650000	55750000	100001	LOC102180828
ROH_33	5	55650000	55750000	100001	STAT6
ROH_33	5	55650000	55750000	100001	NAB2
ROH_33	5	55650000	55750000	100001	NEMP1
ROH_33	5	55650000	55750000	100001	MYO1A
ROH_33	5	55650000	55750000	100001	TAC3
ROH_34	5	56050000	56100000	50001	LOC102185066
ROH_34	5	56050000	56100000	50001	PRIM1
ROH_34	5	56050000	56100000	50001	NACA
ROH_35	5	56650000	56900000	250001	SUOX
ROH_35	5	56650000	56900000	250001	CDK2
ROH_35	5	56650000	56900000	250001	MMP19
ROH_35	5	56650000	56900000	250001	DNAJC14
ROH_35	5	56650000	56900000	250001	ORMDL2
ROH_35	5	56650000	56900000	250001	CD63
ROH_35	5	56650000	56900000	250001	RPS26
ROH_35	5	56650000	56900000	250001	IKZF4
ROH_35	5	56650000	56900000	250001	RAB5B
ROH_35	5	56650000	56900000	250001	PMEL
ROH_35	5	56650000	56900000	250001	DGKA
ROH_35	5	56650000	56900000	250001	LOC102177414
ROH_35	5	56650000	56900000	250001	SARNP
ROH_35	5	56650000	56900000	250001	GDF11
ROH_35	5	56650000	56900000	250001	RDH5
ROH_35	5	56650000	56900000	250001	PYM1
ROH_36	5	72550000	72850000	300001	LOC102170588
ROH_36	5	72550000	72850000	300001	RBFOX2
ROH_37	5	91850000	91900000	50001	LMO3
ROH_38	5	96250000	96300000	50001	LRP6
ROH_39	5	109200000	109350000	150001	TOMM22
ROH_39	5	109200000	109350000	150001	CBY1
ROH_39	5	109200000	109350000	150001	LOC102181663
ROH_39	5	109200000	109350000	150001	JOSD1
ROH_39	5	109200000	109350000	150001	GTPBP1
ROH_39	5	109200000	109350000	150001	SUN2
ROH_39	5	109200000	109350000	150001	DNAL4
ROH_39	5	109200000	109350000	150001	NPTXR
ROH_39	5	109200000	109350000	150001	FAM227A
ROH_40	5	109650000	109850000	200001	RPL3
ROH_40	5	109650000	109850000	200001	TAB1
ROH_40	5	109650000	109850000	200001	MGAT3
ROH_40	5	109650000	109850000	200001	SYNGR1
ROH_41	5	111650000	111700000	50001	XRCC6
ROH_41	5	111650000	111700000	50001	MEI1
ROH_41	5	111650000	111700000	50001	SNU13
ROH_42	5	111750000	111800000	50001	CCDC134

ROH_42	5	111750000	111800000	50001	SREBF2
ROH_43	5	118750000	118800000	50001	LMF2
ROH_43	5	118750000	118800000	50001	LOC102189751
ROH_43	5	118750000	118800000	50001	ODF3B
ROH_43	5	118750000	118800000	50001	NCAPH2
ROH_46	6	70650000	70850000	200001	KIT
ROH_48	6	95400000	95700000	300001	TRNAG-CCC-33
ROH_48	6	95400000	95700000	300001	C6H4orf22
ROH_48	6	95400000	95700000	300001	FGF5
ROH_49	6	111400000	111550000	150001	FGFBP1
ROH_49	6	111400000	111550000	150001	PROM1
ROH_51	7	27500000	27750000	250001	XRCC4
ROH_51	7	27500000	27750000	250001	TMEM167A
ROH_55	7	57400000	57550000	150001	GNPDA1
ROH_55	7	57400000	57550000	150001	RNF14
ROH_55	7	57400000	57550000	150001	PCDH12
ROH_55	7	57400000	57550000	150001	KIAA0141
ROH_56	7	58950000	59000000	50001	HBEGF
ROH_56	7	58950000	59000000	50001	PFDN1
ROH_57	7	72150000	72250000	100001	HK3
ROH_57	7	72150000	72250000	100001	UIMC1
ROH_58	8	150000	300000	150001	LOC102190689
ROH_58	8	150000	300000	150001	MFSD14B
ROH_62	8	38450000	38750000	300001	RANBP6
ROH_62	8	38450000	38750000	300001	MLANA
ROH_62	8	38450000	38750000	300001	ERMP1
ROH_62	8	38450000	38750000	300001	KIAA2026
ROH_65	8	44050000	44350000	300001	LOC102186014
ROH_65	8	44050000	44350000	300001	LOC102185181
ROH_65	8	44050000	44350000	300001	PGM5
ROH_65	8	44050000	44350000	300001	DOCK8
ROH_67	9	56700000	56950000	250001	ARG1
ROH_67	9	56700000	56950000	250001	MED23
ROH_67	9	56700000	56950000	250001	ENPP3
ROH_69	11	14350000	15300000	950001	LOC108637104
ROH_69	11	14350000	15300000	950001	DPY30
ROH_69	11	14350000	15300000	950001	SPAST
ROH_69	11	14350000	15300000	950001	SLC30A6
ROH_69	11	14350000	15300000	950001	YIPF4
ROH_69	11	14350000	15300000	950001	MEMO1
ROH_69	11	14350000	15300000	950001	BIRC6
ROH_69	11	14350000	15300000	950001	TTC27
ROH_69	11	14350000	15300000	950001	NLRC4
ROH_70	11	40300000	40550000	250001	VRK2
ROH_70	11	40300000	40550000	250001	FANCL
ROH_71	11	71950000	72050000	100001	KRTCAP3
ROH_71	11	71950000	72050000	100001	SNX17
ROH_71	11	71950000	72050000	100001	EIF2B4
ROH_71	11	71950000	72050000	100001	MPV17
ROH_71	11	71950000	72050000	100001	IFT172
ROH_71	11	71950000	72050000	100001	PPM1G
ROH_71	11	71950000	72050000	100001	ZNF513
ROH_71	11	71950000	72050000	100001	GTF3C2
ROH_71	11	71950000	72050000	100001	NRBP1
ROH_72	11	78100000	78350000	250001	RHOB
ROH_72	11	78100000	78350000	250001	LOC108637157
ROH_72	11	78100000	78350000	250001	SDC1
ROH_72	11	78100000	78350000	250001	LOC106502652

ROH_72	11	78100000	78350000	250001	PUM2
ROH_73	11	78450000	78650000	200001	TTC32
ROH_73	11	78450000	78650000	200001	LAPTM4A
ROH_73	11	78450000	78650000	200001	MATN3
ROH_73	11	78450000	78650000	200001	WDR35
ROH_74	11	94050000	94150000	100001	DENND1A
ROH_75	11	94250000	94600000	350001	DENND1A
ROH_76	11	96150000	96200000	50001	PBX3
ROH_77	12	10100000	10150000	50001	LOC102177727
ROH_77	12	10100000	10150000	50001	UBAC2
ROH_78	12	19400000	19450000	50001	GPC5
ROH_79	12	19500000	19550000	50001	GPC5
ROH_80	12	33550000	33900000	350001	FBXL3
ROH_80	12	33550000	33900000	350001	MYCBP2
ROH_82	12	50100000	50150000	50001	ATP12A
ROH_82	12	50100000	50150000	50001	RNF17
ROH_83	12	50350000	51400000	1050001	LOC108637296
ROH_83	12	50350000	51400000	1050001	LOC108637298
ROH_83	12	50350000	51400000	1050001	GJB6
ROH_83	12	50350000	51400000	1050001	SAP18
ROH_83	12	50350000	51400000	1050001	TRNAE-UUC-50
ROH_83	12	50350000	51400000	1050001	MRPL57
ROH_83	12	50350000	51400000	1050001	MPHOSPH8
ROH_83	12	50350000	51400000	1050001	ZMYM5
ROH_83	12	50350000	51400000	1050001	GJA3
ROH_83	12	50350000	51400000	1050001	GJB2
ROH_83	12	50350000	51400000	1050001	CRYL1
ROH_83	12	50350000	51400000	1050001	IL17D
ROH_83	12	50350000	51400000	1050001	EEF1AKMT1
ROH_83	12	50350000	51400000	1050001	LATS2
ROH_83	12	50350000	51400000	1050001	SKA3
ROH_83	12	50350000	51400000	1050001	ZDHHC20
ROH_83	12	50350000	51400000	1050001	LOC106502707
ROH_83	12	50350000	51400000	1050001	FGF9
ROH_83	12	50350000	51400000	1050001	PSPC1
ROH_83	12	50350000	51400000	1050001	ZMYM2
ROH_83	12	50350000	51400000	1050001	IFT88
ROH_83	12	50350000	51400000	1050001	XPO4
ROH_83	12	50350000	51400000	1050001	MICU2
ROH_83	12	50350000	51400000	1050001	PARP4
ROH_84	12	57550000	57650000	100001	LOC102187143
ROH_84	12	57550000	57650000	100001	FRY
ROH_85	12	59050000	59150000	100001	STARD13
ROH_86	12	60150000	60200000	50001	LOC102178917
ROH_87	12	60400000	60550000	150001	NBEA
ROH_88	12	60600000	60800000	200001	MAB21L1
ROH_88	12	60600000	60800000	200001	TRNAE-UUC-51
ROH_88	12	60600000	60800000	200001	NBEA
ROH_89	13	33250000	33400000	150001	ZEB1
ROH_90	13	37850000	37900000	50001	LOC108637388
ROH_90	13	37850000	37900000	50001	LOC102189769
ROH_90	13	37850000	37900000	50001	ZNF133
ROH_90	13	37850000	37900000	50001	DZANK1
ROH_91	13	39800000	39950000	150001	KIZ
ROH_92	13	46200000	46350000	150001	ZMYND11
ROH_92	13	46200000	46350000	150001	DIP2C
ROH_93	13	53150000	53300000	150001	SAMD10
ROH_93	13	53150000	53300000	150001	ZNF512B

ROH_93	13	53150000	53300000	150001	MIR1388
ROH_93	13	53150000	53300000	150001	PRPF6
ROH_93	13	53150000	53300000	150001	DNAJC5
ROH_93	13	53150000	53300000	150001	TPD52L2
ROH_93	13	53150000	53300000	150001	ABHD16B
ROH_93	13	53150000	53300000	150001	LOC108637400
ROH_93	13	53150000	53300000	150001	UCKL1
ROH_94	13	63000000	63100000	100001	EIF2S2
ROH_94	13	63000000	63100000	100001	RALY
ROH_95	13	76700000	76850000	150001	CSE1L
ROH_95	13	76700000	76850000	150001	ARFGEF2
ROH_95	13	76700000	76850000	150001	STAU1
ROH_96	14	12650000	12850000	200001	DPY19L4
ROH_96	14	12650000	12850000	200001	CCNE2
ROH_96	14	12650000	12850000	200001	TP53INP1
ROH_96	14	12650000	12850000	200001	INTS8
ROH_96	14	12650000	12850000	200001	LOC102184166
ROH_97	14	16100000	16150000	50001	RIDA
ROH_97	14	16100000	16150000	50001	POP1
ROH_98	14	40100000	40250000	150001	IL7
ROH_98	14	40100000	40250000	150001	ZC2HC1A
ROH_98	14	40100000	40250000	150001	PKIA
ROH_99	14	50700000	50800000	100001	ARFGEF1
ROH_99	14	50700000	50800000	100001	CSPP1
ROH_100	15	6200000	6250000	50001	LRP4
ROH_100	15	6200000	6250000	50001	CKAP5
ROH_101	15	6350000	6500000	150001	HARBI1
ROH_101	15	6350000	6500000	150001	TRNAW-CCA-81
ROH_101	15	6350000	6500000	150001	ARHGAP1
ROH_101	15	6350000	6500000	150001	ATG13
ROH_101	15	6350000	6500000	150001	AMBRA1
ROH_102	15	23950000	24150000	200001	KIF18A
ROH_102	15	23950000	24150000	200001	METTL15
ROH_103	15	24200000	24250000	50001	KIF18A
ROH_104	15	30350000	30550000	200001	FCHSD2
ROH_105	15	31900000	32250000	350001	LOC102178093
ROH_105	15	31900000	32250000	350001	LOC102177821
ROH_105	15	31900000	32250000	350001	LOC108637670
ROH_105	15	31900000	32250000	350001	PGAP2
ROH_105	15	31900000	32250000	350001	LOC102169978
ROH_105	15	31900000	32250000	350001	ART5
ROH_105	15	31900000	32250000	350001	LOC102169399
ROH_105	15	31900000	32250000	350001	LOC102169116
ROH_105	15	31900000	32250000	350001	ART1
ROH_105	15	31900000	32250000	350001	NUP98
ROH_105	15	31900000	32250000	350001	RHOG
ROH_105	15	31900000	32250000	350001	STIM1
ROH_106	15	33900000	34200000	300001	LOC102185107
ROH_106	15	33900000	34200000	300001	LOC102184830
ROH_106	15	33900000	34200000	300001	LOC102184268
ROH_106	15	33900000	34200000	300001	LOC102183437
ROH_106	15	33900000	34200000	300001	LOC102176982
ROH_106	15	33900000	34200000	300001	LOC102183163
ROH_106	15	33900000	34200000	300001	LOC102176710
ROH_106	15	33900000	34200000	300001	LOC102174765
ROH_106	15	33900000	34200000	300001	LOC102175876
ROH_106	15	33900000	34200000	300001	LOC102175600
ROH_106	15	33900000	34200000	300001	LOC102182894

ROH_106	15	33900000	34200000	300001	LOC102175317
ROH_106	15	33900000	34200000	300001	HBBC
ROH_106	15	33900000	34200000	300001	LOC102182615
ROH_106	15	33900000	34200000	300001	LOC102176442
ROH_106	15	33900000	34200000	300001	LOC102174495
ROH_106	15	33900000	34200000	300001	LOC102182330
ROH_106	15	33900000	34200000	300001	LOC102182057
ROH_106	15	33900000	34200000	300001	LOC102183709
ROH_107	16	35900000	36050000	150001	METTL18
ROH_107	16	35900000	36050000	150001	LOC106502954
ROH_107	16	35900000	36050000	150001	SELE
ROH_107	16	35900000	36050000	150001	C16H1orf112
ROH_107	16	35900000	36050000	150001	SCYL3
ROH_108	16	40850000	41100000	250001	ANGPTL7
ROH_108	16	40850000	41100000	250001	SRM
ROH_108	16	40850000	41100000	250001	TARDBP
ROH_108	16	40850000	41100000	250001	UBIAD1
ROH_108	16	40850000	41100000	250001	MASP2
ROH_108	16	40850000	41100000	250001	EXOSC10
ROH_108	16	40850000	41100000	250001	MTOR
ROH_109	16	49500000	49700000	200001	FNDC10
ROH_109	16	49500000	49700000	200001	TMEM240
ROH_109	16	49500000	49700000	200001	TMEM88B
ROH_109	16	49500000	49700000	200001	MRPL20
ROH_109	16	49500000	49700000	200001	AURKAIP1
ROH_109	16	49500000	49700000	200001	TAS1R3
ROH_109	16	49500000	49700000	200001	CPTP
ROH_109	16	49500000	49700000	200001	MIB2
ROH_109	16	49500000	49700000	200001	SSU72
ROH_109	16	49500000	49700000	200001	LOC102189890
ROH_109	16	49500000	49700000	200001	VWA1
ROH_109	16	49500000	49700000	200001	ANKRD65
ROH_109	16	49500000	49700000	200001	CCNL2
ROH_109	16	49500000	49700000	200001	DVL1
ROH_109	16	49500000	49700000	200001	MXRA8
ROH_110	16	49750000	50000000	250001	B3GALT6
ROH_110	16	49750000	50000000	250001	TNFRSF4
ROH_110	16	49750000	50000000	250001	TNFRSF18
ROH_110	16	49750000	50000000	250001	MIR429
ROH_110	16	49750000	50000000	250001	MIR200A
ROH_110	16	49750000	50000000	250001	MIR200B
ROH_110	16	49750000	50000000	250001	LOC102169118
ROH_110	16	49750000	50000000	250001	LOC106502959
ROH_110	16	49750000	50000000	250001	ISG15
ROH_110	16	49750000	50000000	250001	HES4
ROH_110	16	49750000	50000000	250001	PERM1
ROH_110	16	49750000	50000000	250001	UBE2J2
ROH_110	16	49750000	50000000	250001	FAM132A
ROH_110	16	49750000	50000000	250001	SDF4
ROH_110	16	49750000	50000000	250001	C16H1orf159
ROH_110	16	49750000	50000000	250001	RNF223
ROH_110	16	49750000	50000000	250001	PLEKHN1
ROH_110	16	49750000	50000000	250001	TTLL10
ROH_110	16	49750000	50000000	250001	AGRN
ROH_111	16	53450000	53600000	150001	LOC102176064
ROH_111	16	53450000	53600000	150001	PRDX6
ROH_111	16	53450000	53600000	150001	ANKRD45
ROH_111	16	53450000	53600000	150001	SLC9C2

ROH_112	16	79050000	79150000	100001	IPO9
ROH_112	16	79050000	79150000	100001	LMOD1
ROH_112	16	79050000	79150000	100001	TIMM17A
ROH_112	16	79050000	79150000	100001	RNPEP
ROH_113	17	9050000	9100000	50001	LOC108637851
ROH_113	17	9050000	9100000	50001	TRAFD1
ROH_113	17	9050000	9100000	50001	NAA25
ROH_113	17	9050000	9100000	50001	HECTD4
ROH_114	17	15850000	16100000	250001	LOC102182159
ROH_114	17	15850000	16100000	250001	BRAP
ROH_114	17	15850000	16100000	250001	SH2B3
ROH_114	17	15850000	16100000	250001	ATXN2
ROH_117	18	15950000	16100000	150001	CHMP1A
ROH_117	18	15950000	16100000	150001	SPATA2L
ROH_117	18	15950000	16100000	150001	CDK10
ROH_117	18	15950000	16100000	150001	FANCA
ROH_117	18	15950000	16100000	150001	SPIRE2
ROH_117	18	15950000	16100000	150001	TCF25
ROH_117	18	15950000	16100000	150001	ZNF276
ROH_117	18	15950000	16100000	150001	VPS9D1
ROH_118	18	26850000	26900000	50001	CCDC102A
ROH_118	18	26850000	26900000	50001	ADGRG5
ROH_119	18	36000000	36200000	200001	TRADD
ROH_119	18	36000000	36200000	200001	FBXL8
ROH_119	18	36000000	36200000	200001	NOL3
ROH_119	18	36000000	36200000	200001	KIAA0895L
ROH_119	18	36000000	36200000	200001	E2F4
ROH_119	18	36000000	36200000	200001	MIR328
ROH_119	18	36000000	36200000	200001	LRRC29
ROH_119	18	36000000	36200000	200001	CBFB
ROH_119	18	36000000	36200000	200001	B3GNT9
ROH_119	18	36000000	36200000	200001	HSF4
ROH_119	18	36000000	36200000	200001	EXOC3L1
ROH_119	18	36000000	36200000	200001	ELMO3
ROH_119	18	36000000	36200000	200001	TMEM208
ROH_119	18	36000000	36200000	200001	FHOD1
ROH_119	18	36000000	36200000	200001	SLC9A5
ROH_119	18	36000000	36200000	200001	KCTD19
ROH_119	18	36000000	36200000	200001	C18H16orf70
ROH_119	18	36000000	36200000	200001	PLEKHG4
ROH_120	18	36650000	37350000	700001	TRNAS-AGA-6
ROH_120	18	36650000	37350000	700001	LCAT
ROH_120	18	36650000	37350000	700001	DPEP3
ROH_120	18	36650000	37350000	700001	DPEP2
ROH_120	18	36650000	37350000	700001	LOC106503098
ROH_120	18	36650000	37350000	700001	DDX28
ROH_120	18	36650000	37350000	700001	SLC7A6OS
ROH_120	18	36650000	37350000	700001	LOC102169124
ROH_120	18	36650000	37350000	700001	LOC108637978
ROH_120	18	36650000	37350000	700001	LOC102170170
ROH_120	18	36650000	37350000	700001	PSKH1
ROH_120	18	36650000	37350000	700001	PSMB10
ROH_120	18	36650000	37350000	700001	DUS2
ROH_120	18	36650000	37350000	700001	ESRP2
ROH_120	18	36650000	37350000	700001	PLA2G15
ROH_120	18	36650000	37350000	700001	SLC7A6
ROH_120	18	36650000	37350000	700001	SMPD3
ROH_120	18	36650000	37350000	700001	LOC108637977

ROH_120	18	36650000	37350000	700001	ZFP90
ROH_120	18	36650000	37350000	700001	CDH3
ROH_120	18	36650000	37350000	700001	NFATC3
ROH_120	18	36650000	37350000	700001	PRMT7
ROH_120	18	36650000	37350000	700001	CDH1
ROH_120	18	36650000	37350000	700001	SLC12A4
ROH_123	18	56950000	57100000	150001	RPS11
ROH_123	18	56950000	57100000	150001	MIR150
ROH_123	18	56950000	57100000	150001	FCGRT
ROH_123	18	56950000	57100000	150001	PRRG2
ROH_123	18	56950000	57100000	150001	RRAS
ROH_123	18	56950000	57100000	150001	IRF3
ROH_123	18	56950000	57100000	150001	PRMT1
ROH_123	18	56950000	57100000	150001	RPL13A
ROH_123	18	56950000	57100000	150001	RCN3
ROH_123	18	56950000	57100000	150001	NOSIP
ROH_123	18	56950000	57100000	150001	SCAF1
ROH_123	18	56950000	57100000	150001	BCL2L12
ROH_123	18	56950000	57100000	150001	ADM5
ROH_123	18	56950000	57100000	150001	CPT1C
ROH_123	18	56950000	57100000	150001	PRR12
ROH_124	19	20500000	20650000	150001	TP53I13
ROH_124	19	20500000	20650000	150001	ABHD15
ROH_124	19	20500000	20650000	150001	TAOK1
ROH_125	19	22850000	23000000	150001	OVCA2
ROH_125	19	22850000	23000000	150001	DPH1
ROH_125	19	22850000	23000000	150001	HIC1
ROH_125	19	22850000	23000000	150001	SMG6
ROH_126	19	51100000	51150000	50001	RPTOR
ROH_127	20	25400000	25550000	150001	NDUFS4
ROH_128	20	38850000	38950000	100001	PRLR
ROH_129	20	41250000	41300000	50001	MTMR12
ROH_130	21	19600000	19700000	100001	ACAN
ROH_131	21	44850000	44900000	50001	KIAA0391
ROH_132	21	64250000	64400000	150001	MIR342
ROH_132	21	64250000	64400000	150001	DEGS2
ROH_132	21	64250000	64400000	150001	EVL
ROH_133	22	16200000	16350000	150001	ZNF852
ROH_133	22	16200000	16350000	150001	ZNF502
ROH_133	22	16200000	16350000	150001	ZNF501
ROH_133	22	16200000	16350000	150001	KIAA1143
ROH_133	22	16200000	16350000	150001	LOC102177570
ROH_133	22	16200000	16350000	150001	KIF15
ROH_134	22	17350000	17550000	200001	SRGAP3
ROH_134	22	17350000	17550000	200001	RAD18
ROH_135	22	31600000	31650000	50001	MITF
ROH_136	22	49950000	50000000	50001	CACNA2D2
ROH_137	23	8600000	8900000	300001	JARID2
ROH_137	23	8600000	8900000	300001	DTNBP1
ROH_138	23	19350000	19500000	150001	ZSCAN16
ROH_138	23	19350000	19500000	150001	LOC102177207
ROH_138	23	19350000	19500000	150001	TRNAS-GCU-17
ROH_138	23	19350000	19500000	150001	ZSCAN9
ROH_138	23	19350000	19500000	150001	ZKSCAN4
ROH_138	23	19350000	19500000	150001	ZNF165
ROH_138	23	19350000	19500000	150001	LOC108633410
ROH_138	23	19350000	19500000	150001	LOC108633417
ROH_138	23	19350000	19500000	150001	LOC108633266

ROH_138	23	19350000	19500000	150001	ZKSCAN8
ROH_139	23	22400000	22500000	100001	SAPCD1
ROH_139	23	22400000	22500000	100001	VARS
ROH_139	23	22400000	22500000	100001	LSM2
ROH_139	23	22400000	22500000	100001	HSP70.1
ROH_139	23	22400000	22500000	100001	LOC102178315
ROH_139	23	22400000	22500000	100001	LOC102177673
ROH_139	23	22400000	22500000	100001	SLC44A4
ROH_139	23	22400000	22500000	100001	MSH5
ROH_139	23	22400000	22500000	100001	LOC102177850
ROH_139	23	22400000	22500000	100001	NEU1
ROH_139	23	22400000	22500000	100001	EHMT2
ROH_139	23	22400000	22500000	100001	VWA7
ROH_140	23	30250000	30350000	100001	RUNX2
ROH_140	23	30250000	30350000	100001	SUPT3H
ROH_141	23	30600000	30650000	50001	SUPT3H
ROH_142	23	39950000	40100000	150001	C23H6orf106
ROH_142	23	39950000	40100000	150001	SPDEF
ROH_143	24	43500000	43900000	400001	MC5R
ROH_143	24	43500000	43900000	400001	PTPN2
ROH_143	24	43500000	43900000	400001	SEH1L
ROH_143	24	43500000	43900000	400001	CEP192
ROH_143	24	43500000	43900000	400001	FAM210A
ROH_143	24	43500000	43900000	400001	RNMT
ROH_143	24	43500000	43900000	400001	LDLRAD4
ROH_143	24	43500000	43900000	400001	MC2R
ROH_144	25	100000	300000	200001	TRNAY-GUA-31
ROH_144	25	100000	300000	200001	LOC108633873
ROH_144	25	100000	300000	200001	LOC108633874
ROH_144	25	100000	300000	200001	HBM
ROH_144	25	100000	300000	200001	LOC102168680
ROH_144	25	100000	300000	200001	LOC102168959
ROH_144	25	100000	300000	200001	LOC102186172
ROH_144	25	100000	300000	200001	RGS11
ROH_144	25	100000	300000	200001	ARHGDIG
ROH_144	25	100000	300000	200001	MRPL28
ROH_144	25	100000	300000	200001	LUC7L
ROH_144	25	100000	300000	200001	FAM234A
ROH_144	25	100000	300000	200001	PDIA2
ROH_144	25	100000	300000	200001	TMEM8A
ROH_144	25	100000	300000	200001	NPRL3
ROH_144	25	100000	300000	200001	AXIN1
ROH_145	26	28900000	29200000	300001	HPS6
ROH_145	26	28900000	29200000	300001	FGF8
ROH_145	26	28900000	29200000	300001	MGEA5
ROH_145	26	28900000	29200000	300001	NPM3
ROH_145	26	28900000	29200000	300001	C26H10orf76
ROH_145	26	28900000	29200000	300001	KCNIP2
ROH_146	26	30450000	30600000	150001	PKD2L1
ROH_146	26	30450000	30600000	150001	BLOC1S2
ROH_146	26	30450000	30600000	150001	CWF19L1
ROH_146	26	30450000	30600000	150001	ERLIN1
ROH_146	26	30450000	30600000	150001	CHUK
ROH_147	26	33350000	33450000	100001	LCOR
