# Supplementary material for: Whole-genome selection signatures identified candidate genes associated with cashmere traits in Inner Mongolia cashmere goats
Source: Anim Biosci. 2025 Jul 11;38(12):2597–611. doi: 10.5713/ab.25.0252 (PMC12580777; doi:10.5713/ab.25.0252)
Supplement: Supplementary file 5 [file ab-25-0252-Supplementary-5.pdf]

Supplement 5. High-frequency ROH regions and annotated genes in LFCG

**Interval_ID**	**Chr**	**Start**	**End**	**Length**	**Gene name**
ROH_1	1	82550000	82750000	200001	PSMD2
ROH_1	1	82550000	82750000	200001	CAMK2N2
ROH_1	1	82550000	82750000	200001	MIR1224
ROH_1	1	82550000	82750000	200001	ABCF3
ROH_1	1	82550000	82750000	200001	LOC102185396
ROH_1	1	82550000	82750000	200001	LOC102185870
ROH_1	1	82550000	82750000	200001	EIF4G1
ROH_1	1	82550000	82750000	200001	ALG3
ROH_1	1	82550000	82750000	200001	AP2M1
ROH_1	1	82550000	82750000	200001	DVL3
ROH_1	1	82550000	82750000	200001	EIF2B5
ROH_1	1	82550000	82750000	200001	ECE2
ROH_2	1	84950000	85100000	150001	SOX2
ROH_2	1	84950000	85100000	150001	LOC106502138
ROH_4	1	108550000	109000000	450001	SHOX2
ROH_4	1	108550000	109000000	450001	RSRC1
ROH_5	1	110250000	110450000	200001	LEKR1
ROH_6	1	120850000	121050000	200001	LOC106502391
ROH_7	1	132200000	132400000	200001	STAG1
ROH_8	1	152550000	152650000	100001	LOC108636923
ROH_8	1	152550000	152650000	100001	ANKRD28
ROH_10	2	29450000	29500000	50001	PNKD
ROH_10	2	29450000	29500000	50001	TMBIM1
ROH_11	2	29550000	29650000	100001	CXCR1
ROH_11	2	29550000	29650000	100001	RUFY4
ROH_11	2	29550000	29650000	100001	CXCR2
ROH_15	3	7650000	7700000	50001	DGKD
ROH_17	3	25350000	25400000	50001	FAF1
ROH_18	3	80550000	80750000	200001	RNPC3
ROH_18	3	80550000	80750000	200001	LOC102169641
ROH_19	3	87100000	87250000	150001	STRIP1
ROH_19	3	87100000	87250000	150001	ALX3
ROH_19	3	87100000	87250000	150001	LOC102187445
ROH_19	3	87100000	87250000	150001	AHCYL1
ROH_20	3	90900000	91000000	100001	LOC108635585
ROH_20	3	90900000	91000000	100001	LOC108635586
ROH_20	3	90900000	91000000	100001	PHTF1
ROH_20	3	90900000	91000000	100001	LOC106501937
ROH_20	3	90900000	91000000	100001	RSBN1
ROH_21	3	95350000	95450000	100001	SPAG17
ROH_22	4	72800000	73050000	250001	NAMPT
ROH_23	4	75100000	75750000	650001	SLC26A5
ROH_23	4	75100000	75750000	650001	PSMC2
ROH_23	4	75100000	75750000	650001	DNAJC2
ROH_23	4	75100000	75750000	650001	RELN
ROH_25	5	18650000	18800000	150001	TRNAC-GCA-83
ROH_26	5	20850000	20950000	100001	TRNAC-ACA-25
ROH_26	5	20850000	20950000	100001	LOC108636127
ROH_26	5	20850000	20950000	100001	DCN
ROH_27	5	47000000	47050000	50001	LLPH
ROH_28	5	55650000	55700000	50001	LOC102180828
ROH_28	5	55650000	55700000	50001	STAT6
ROH_28	5	55650000	55700000	50001	NAB2
ROH_28	5	55650000	55700000	50001	NEMP1
ROH_29	5	56050000	56150000	100001	LOC102185066
ROH_29	5	56050000	56150000	100001	PRIM1

ROH_29	5	56050000	56150000	100001	PTGES3
ROH_29	5	56050000	56150000	100001	NACA
ROH_30	5	56650000	56950000	300001	SUOX
ROH_30	5	56650000	56950000	300001	CDK2
ROH_30	5	56650000	56950000	300001	MMP19
ROH_30	5	56650000	56950000	300001	DNAJC14
ROH_30	5	56650000	56950000	300001	ORMDL2
ROH_30	5	56650000	56950000	300001	CD63
ROH_30	5	56650000	56950000	300001	BLOC1S1
ROH_30	5	56650000	56950000	300001	METTL7B
ROH_30	5	56650000	56950000	300001	RPS26
ROH_30	5	56650000	56950000	300001	IKZF4
ROH_30	5	56650000	56950000	300001	RAB5B
ROH_30	5	56650000	56950000	300001	PMEL
ROH_30	5	56650000	56950000	300001	DGKA
ROH_30	5	56650000	56950000	300001	LOC102177414
ROH_30	5	56650000	56950000	300001	SARNP
ROH_30	5	56650000	56950000	300001	GDF11
ROH_30	5	56650000	56950000	300001	RDH5
ROH_30	5	56650000	56950000	300001	ITGA7
ROH_30	5	56650000	56950000	300001	PYM1
ROH_31	5	72550000	72700000	150001	LOC102170588
ROH_31	5	72550000	72700000	150001	RBFOX2
ROH_32	5	91850000	91900000	50001	LMO3
ROH_33	5	109200000	109350000	150001	TOMM22
ROH_33	5	109200000	109350000	150001	CBY1
ROH_33	5	109200000	109350000	150001	LOC102181663
ROH_33	5	109200000	109350000	150001	JOSD1
ROH_33	5	109200000	109350000	150001	GTPBP1
ROH_33	5	109200000	109350000	150001	SUN2
ROH_33	5	109200000	109350000	150001	DNAL4
ROH_33	5	109200000	109350000	150001	NPTXR
ROH_33	5	109200000	109350000	150001	FAM227A
ROH_34	5	109700000	109850000	150001	TAB1
ROH_34	5	109700000	109850000	150001	MGAT3
ROH_34	5	109700000	109850000	150001	SYNGR1
ROH_35	5	111650000	111800000	150001	XRCC6
ROH_35	5	111650000	111800000	150001	MEI1
ROH_35	5	111650000	111800000	150001	CCDC134
ROH_35	5	111650000	111800000	150001	SNU13
ROH_35	5	111650000	111800000	150001	SREBF2
ROH_37	6	19100000	19300000	200001	GIMD1
ROH_37	6	19100000	19300000	200001	AIMP1
ROH_37	6	19100000	19300000	200001	TBCK
ROH_38	6	30800000	30950000	150001	HPGDS
ROH_38	6	30800000	30950000	150001	SMARCAD1
ROH_39	6	54450000	54550000	100001	TRNAW-CCA-33
ROH_40	6	69450000	69600000	150001	FIP1L1
ROH_40	6	69450000	69600000	150001	LNX1
ROH_40	6	69450000	69600000	150001	SCFD2
ROH_42	6	70650000	70850000	200001	KIT
ROH_44	6	95400000	95700000	300001	TRNAG-CCC-33
ROH_44	6	95400000	95700000	300001	C6H4orf22
ROH_44	6	95400000	95700000	300001	FGF5
ROH_45	6	95750000	95800000	50001	C6H4orf22
ROH_46	6	111400000	111550000	150001	FGFBP1
ROH_46	6	111400000	111550000	150001	PROM1
ROH_48	7	27400000	27450000	50001	XRCC4

ROH_49	7	27500000	27750000	250001	XRCC4
ROH_49	7	27500000	27750000	250001	TMEM167A
ROH_51	7	58850000	59100000	250001	TRNAC-GCA-118
ROH_51	7	58850000	59100000	250001	LOC106502354
ROH_51	7	58850000	59100000	250001	SLC4A9
ROH_51	7	58850000	59100000	250001	HBEGF
ROH_51	7	58850000	59100000	250001	PFDN1
ROH_51	7	58850000	59100000	250001	LOC102175120
ROH_51	7	58850000	59100000	250001	CYSTM1
ROH_52	7	59800000	59900000	100001	SPATA24
ROH_52	7	59800000	59900000	100001	PROB1
ROH_52	7	59800000	59900000	100001	DNAJC18
ROH_52	7	59800000	59900000	100001	MZB1
ROH_52	7	59800000	59900000	100001	SLC23A1
ROH_52	7	59800000	59900000	100001	PAIP2
ROH_52	7	59800000	59900000	100001	MATR3
ROH_53	7	59950000	60000000	50001	SIL1
ROH_54	7	62850000	63000000	150001	TRPC7
ROH_54	7	62850000	63000000	150001	SMAD5
ROH_55	8	150000	300000	150001	LOC102190689
ROH_55	8	150000	300000	150001	MFSD14B
ROH_57	8	38400000	38650000	250001	RANBP6
ROH_57	8	38400000	38650000	250001	MLANA
ROH_57	8	38400000	38650000	250001	IL33
ROH_57	8	38400000	38650000	250001	KIAA2026
ROH_61	8	44050000	44300000	250001	LOC102186014
ROH_61	8	44050000	44300000	250001	LOC102185181
ROH_61	8	44050000	44300000	250001	PGM5
ROH_61	8	44050000	44300000	250001	DOCK8
ROH_63	9	56700000	56900000	200001	ARG1
ROH_63	9	56700000	56900000	200001	MED23
ROH_63	9	56700000	56900000	200001	ENPP3
ROH_64	9	76150000	76200000	50001	ESR1
ROH_66	10	80450000	80600000	150001	REC8
ROH_66	10	80450000	80600000	150001	TM9SF1
ROH_66	10	80450000	80600000	150001	CHMP4A
ROH_66	10	80450000	80600000	150001	LOC102188408
ROH_66	10	80450000	80600000	150001	GMPR2
ROH_66	10	80450000	80600000	150001	TINF2
ROH_66	10	80450000	80600000	150001	RABGGTA
ROH_66	10	80450000	80600000	150001	NOP9
ROH_66	10	80450000	80600000	150001	CIDEB
ROH_66	10	80450000	80600000	150001	LTB4R2
ROH_66	10	80450000	80600000	150001	RIPK3
ROH_66	10	80450000	80600000	150001	IPO4
ROH_66	10	80450000	80600000	150001	NEDD8
ROH_66	10	80450000	80600000	150001	TGM1
ROH_66	10	80450000	80600000	150001	DHRS1
ROH_66	10	80450000	80600000	150001	LTB4R
ROH_66	10	80450000	80600000	150001	ADCY4
ROH_66	10	80450000	80600000	150001	TSSK4
ROH_67	10	82350000	82500000	150001	MYO9A
ROH_68	10	94200000	94350000	150001	ANKDD1B
ROH_68	10	94200000	94350000	150001	POLK
ROH_68	10	94200000	94350000	150001	COL4A3BP
ROH_69	10	94600000	94700000	100001	ANKRD31
ROH_70	11	14350000	14550000	200001	DPY30
ROH_70	11	14350000	14550000	200001	MEMO1

ROH_71	11	14600000	15300000	700001	LOC108637104
ROH_71	11	14600000	15300000	700001	SPAST
ROH_71	11	14600000	15300000	700001	SLC30A6
ROH_71	11	14600000	15300000	700001	YIPF4
ROH_71	11	14600000	15300000	700001	BIRC6
ROH_71	11	14600000	15300000	700001	TTC27
ROH_71	11	14600000	15300000	700001	NLRC4
ROH_72	11	40300000	40550000	250001	VRK2
ROH_72	11	40300000	40550000	250001	FANCL
ROH_73	11	43250000	43350000	100001	PUS10
ROH_73	11	43250000	43350000	100001	REL
ROH_74	11	60350000	60500000	150001	B3GNT2
ROH_75	11	62000000	62100000	100001	UGP2
ROH_75	11	62000000	62100000	100001	VPS54
ROH_76	11	78150000	78400000	250001	LOC108637157
ROH_76	11	78150000	78400000	250001	SDC1
ROH_76	11	78150000	78400000	250001	PUM2
ROH_77	11	78450000	78650000	200001	TTC32
ROH_77	11	78450000	78650000	200001	LAPTM4A
ROH_77	11	78450000	78650000	200001	MATN3
ROH_77	11	78450000	78650000	200001	WDR35
ROH_78	11	94050000	94150000	100001	DENND1A
ROH_79	11	94250000	94650000	400001	LHX2
ROH_79	11	94250000	94650000	400001	DENND1A
ROH_80	11	96150000	96250000	100001	PBX3
ROH_81	12	10100000	10150000	50001	LOC102177727
ROH_81	12	10100000	10150000	50001	UBAC2
ROH_82	12	33550000	33900000	350001	FBXL3
ROH_82	12	33550000	33900000	350001	MYCBP2
ROH_85	12	50050000	51450000	1400001	LOC108637296
ROH_85	12	50050000	51450000	1400001	LOC108637298
ROH_85	12	50050000	51450000	1400001	GJB6
ROH_85	12	50050000	51450000	1400001	SAP18
ROH_85	12	50050000	51450000	1400001	TRNAE-UUC-50
ROH_85	12	50050000	51450000	1400001	MRPL57
ROH_85	12	50050000	51450000	1400001	ATP12A
ROH_85	12	50050000	51450000	1400001	CENPJ
ROH_85	12	50050000	51450000	1400001	MPHOSPH8
ROH_85	12	50050000	51450000	1400001	ZMYM5
ROH_85	12	50050000	51450000	1400001	GJA3
ROH_85	12	50050000	51450000	1400001	GJB2
ROH_85	12	50050000	51450000	1400001	CRYL1
ROH_85	12	50050000	51450000	1400001	IL17D
ROH_85	12	50050000	51450000	1400001	EEF1AKMT1
ROH_85	12	50050000	51450000	1400001	LATS2
ROH_85	12	50050000	51450000	1400001	SKA3
ROH_85	12	50050000	51450000	1400001	ZDHHC20
ROH_85	12	50050000	51450000	1400001	LOC106502707
ROH_85	12	50050000	51450000	1400001	FGF9
ROH_85	12	50050000	51450000	1400001	RNF17
ROH_85	12	50050000	51450000	1400001	PSPC1
ROH_85	12	50050000	51450000	1400001	ZMYM2
ROH_85	12	50050000	51450000	1400001	IFT88
ROH_85	12	50050000	51450000	1400001	XPO4
ROH_85	12	50050000	51450000	1400001	MICU2
ROH_85	12	50050000	51450000	1400001	PARP4
ROH_86	12	54700000	54750000	50001	FLT3
ROH_87	12	57350000	57500000	150001	RXFP2

ROH_88	12	57550000	57700000	150001	LOC102187143
ROH_88	12	57550000	57700000	150001	FRY
ROH_89	12	59050000	59150000	100001	STARD13
ROH_90	12	60150000	60200000	50001	LOC102178917
ROH_91	12	60400000	60800000	400001	MAB21L1
ROH_91	12	60400000	60800000	400001	TRNAE-UUC-51
ROH_91	12	60400000	60800000	400001	NBEA
ROH_92	13	46200000	46250000	50001	ZMYND11
ROH_92	13	46200000	46250000	50001	DIP2C
ROH_93	13	53100000	53250000	150001	LKAAEAR1
ROH_93	13	53100000	53250000	150001	RGS19
ROH_93	13	53100000	53250000	150001	TCEA2
ROH_93	13	53100000	53250000	150001	SOX18
ROH_93	13	53100000	53250000	150001	SAMD10
ROH_93	13	53100000	53250000	150001	ZNF512B
ROH_93	13	53100000	53250000	150001	MIR1388
ROH_93	13	53100000	53250000	150001	OPRL1
ROH_93	13	53100000	53250000	150001	PRPF6
ROH_93	13	53100000	53250000	150001	DNAJC5
ROH_93	13	53100000	53250000	150001	LOC108637400
ROH_93	13	53100000	53250000	150001	UCKL1
ROH_94	13	62950000	63100000	150001	LOC108637417
ROH_94	13	62950000	63100000	150001	EIF2S2
ROH_94	13	62950000	63100000	150001	RALY
ROH_95	13	76700000	76850000	150001	CSE1L
ROH_95	13	76700000	76850000	150001	ARFGEF2
ROH_95	13	76700000	76850000	150001	STAU1
ROH_96	14	12650000	12800000	150001	DPY19L4
ROH_96	14	12650000	12800000	150001	CCNE2
ROH_96	14	12650000	12800000	150001	TP53INP1
ROH_96	14	12650000	12800000	150001	INTS8
ROH_97	14	17500000	17600000	100001	VPS13B
ROH_98	14	50750000	50800000	50001	ARFGEF1
ROH_98	14	50750000	50800000	50001	CSPP1
ROH_99	15	6350000	6500000	150001	HARBI1
ROH_99	15	6350000	6500000	150001	TRNAW-CCA-81
ROH_99	15	6350000	6500000	150001	ARHGAP1
ROH_99	15	6350000	6500000	150001	ATG13
ROH_99	15	6350000	6500000	150001	AMBRA1
ROH_100	15	23950000	24150000	200001	KIF18A
ROH_100	15	23950000	24150000	200001	METTL15
ROH_101	15	24200000	24250000	50001	KIF18A
ROH_102	15	28850000	28900000	50001	XRRA1
ROH_102	15	28850000	28900000	50001	RNF169
ROH_103	15	30400000	30500000	100001	FCHSD2
ROH_104	15	31900000	32300000	400001	LOC102178093
ROH_104	15	31900000	32300000	400001	LOC102177821
ROH_104	15	31900000	32300000	400001	LOC108637670
ROH_104	15	31900000	32300000	400001	PGAP2
ROH_104	15	31900000	32300000	400001	LOC102169978
ROH_104	15	31900000	32300000	400001	ART5
ROH_104	15	31900000	32300000	400001	LOC102169399
ROH_104	15	31900000	32300000	400001	LOC102169116
ROH_104	15	31900000	32300000	400001	ART1
ROH_104	15	31900000	32300000	400001	NUP98
ROH_104	15	31900000	32300000	400001	RHOG
ROH_104	15	31900000	32300000	400001	STIM1
ROH_105	15	33900000	34200000	300001	LOC102185107

ROH_105	15	33900000	34200000	300001	LOC102184830
ROH_105	15	33900000	34200000	300001	LOC102184268
ROH_105	15	33900000	34200000	300001	LOC102183437
ROH_105	15	33900000	34200000	300001	LOC102176982
ROH_105	15	33900000	34200000	300001	LOC102183163
ROH_105	15	33900000	34200000	300001	LOC102176710
ROH_105	15	33900000	34200000	300001	LOC102174765
ROH_105	15	33900000	34200000	300001	LOC102175876
ROH_105	15	33900000	34200000	300001	LOC102175600
ROH_105	15	33900000	34200000	300001	LOC102182894
ROH_105	15	33900000	34200000	300001	LOC102175317
ROH_105	15	33900000	34200000	300001	HBBC
ROH_105	15	33900000	34200000	300001	LOC102182615
ROH_105	15	33900000	34200000	300001	LOC102176442
ROH_105	15	33900000	34200000	300001	LOC102174495
ROH_105	15	33900000	34200000	300001	LOC102182330
ROH_105	15	33900000	34200000	300001	LOC102182057
ROH_105	15	33900000	34200000	300001	LOC102183709
ROH_106	15	34800000	34950000	150001	LOC102181861
ROH_106	15	34800000	34950000	150001	LOC102180484
ROH_106	15	34800000	34950000	150001	LOC102180752
ROH_106	15	34800000	34950000	150001	LOC102176880
ROH_106	15	34800000	34950000	150001	LOC102182689
ROH_106	15	34800000	34950000	150001	LOC102179935
ROH_106	15	34800000	34950000	150001	LOC102182401
ROH_107	15	39800000	39950000	150001	SBF2
ROH_108	15	45950000	46100000	150001	SOX6
ROH_109	16	32200000	32250000	50001	SDCCAG8
ROH_110	16	32350000	32400000	50001	SDCCAG8
ROH_111	16	35900000	36050000	150001	METTL18
ROH_111	16	35900000	36050000	150001	LOC106502954
ROH_111	16	35900000	36050000	150001	SELE
ROH_111	16	35900000	36050000	150001	C16H1orf112
ROH_111	16	35900000	36050000	150001	SCYL3
ROH_112	16	40850000	41100000	250001	ANGPTL7
ROH_112	16	40850000	41100000	250001	SRM
ROH_112	16	40850000	41100000	250001	TARDBP
ROH_112	16	40850000	41100000	250001	UBIAD1
ROH_112	16	40850000	41100000	250001	MASP2
ROH_112	16	40850000	41100000	250001	EXOSC10
ROH_112	16	40850000	41100000	250001	MTOR
ROH_113	16	43200000	43250000	50001	RERE
ROH_114	16	49500000	50000000	500001	FNDC10
ROH_114	16	49500000	50000000	500001	TMEM240
ROH_114	16	49500000	50000000	500001	TMEM88B
ROH_114	16	49500000	50000000	500001	MRPL20
ROH_114	16	49500000	50000000	500001	AURKAIP1
ROH_114	16	49500000	50000000	500001	TAS1R3
ROH_114	16	49500000	50000000	500001	CPTP
ROH_114	16	49500000	50000000	500001	PUSL1
ROH_114	16	49500000	50000000	500001	SCNN1D
ROH_114	16	49500000	50000000	500001	B3GALT6
ROH_114	16	49500000	50000000	500001	TNFRSF4
ROH_114	16	49500000	50000000	500001	TNFRSF18
ROH_114	16	49500000	50000000	500001	MIR429
ROH_114	16	49500000	50000000	500001	MIR200A
ROH_114	16	49500000	50000000	500001	MIR200B
ROH_114	16	49500000	50000000	500001	LOC102169118

ROH_114	16	49500000	50000000	500001	LOC106502959
ROH_114	16	49500000	50000000	500001	ISG15
ROH_114	16	49500000	50000000	500001	HES4
ROH_114	16	49500000	50000000	500001	PERM1
ROH_114	16	49500000	50000000	500001	MIB2
ROH_114	16	49500000	50000000	500001	SSU72
ROH_114	16	49500000	50000000	500001	LOC102189890
ROH_114	16	49500000	50000000	500001	VWA1
ROH_114	16	49500000	50000000	500001	ANKRD65
ROH_114	16	49500000	50000000	500001	CCNL2
ROH_114	16	49500000	50000000	500001	DVL1
ROH_114	16	49500000	50000000	500001	CPSF3L
ROH_114	16	49500000	50000000	500001	ACAP3
ROH_114	16	49500000	50000000	500001	UBE2J2
ROH_114	16	49500000	50000000	500001	FAM132A
ROH_114	16	49500000	50000000	500001	SDF4
ROH_114	16	49500000	50000000	500001	C16H1orf159
ROH_114	16	49500000	50000000	500001	RNF223
ROH_114	16	49500000	50000000	500001	PLEKHN1
ROH_114	16	49500000	50000000	500001	MXRA8
ROH_114	16	49500000	50000000	500001	TTLL10
ROH_114	16	49500000	50000000	500001	AGRN
ROH_115	16	54350000	54500000	150001	RABGAP1L
ROH_116	16	70250000	70400000	150001	LPGAT1
ROH_118	18	15950000	16100000	150001	CHMP1A
ROH_118	18	15950000	16100000	150001	SPATA2L
ROH_118	18	15950000	16100000	150001	CDK10
ROH_118	18	15950000	16100000	150001	FANCA
ROH_118	18	15950000	16100000	150001	SPIRE2
ROH_118	18	15950000	16100000	150001	TCF25
ROH_118	18	15950000	16100000	150001	ZNF276
ROH_118	18	15950000	16100000	150001	VPS9D1
ROH_119	18	26850000	26900000	50001	CCDC102A
ROH_119	18	26850000	26900000	50001	ADGRG5
ROH_120	18	36050000	36200000	150001	TRADD
ROH_120	18	36050000	36200000	150001	FBXL8
ROH_120	18	36050000	36200000	150001	NOL3
ROH_120	18	36050000	36200000	150001	KIAA0895L
ROH_120	18	36050000	36200000	150001	E2F4
ROH_120	18	36050000	36200000	150001	MIR328
ROH_120	18	36050000	36200000	150001	LRRC29
ROH_120	18	36050000	36200000	150001	B3GNT9
ROH_120	18	36050000	36200000	150001	HSF4
ROH_120	18	36050000	36200000	150001	EXOC3L1
ROH_120	18	36050000	36200000	150001	ELMO3
ROH_120	18	36050000	36200000	150001	TMEM208
ROH_120	18	36050000	36200000	150001	FHOD1
ROH_120	18	36050000	36200000	150001	SLC9A5
ROH_120	18	36050000	36200000	150001	KCTD19
ROH_120	18	36050000	36200000	150001	C18H16orf70
ROH_120	18	36050000	36200000	150001	PLEKHG4
ROH_121	18	36750000	37150000	400001	SLC7A6OS
ROH_121	18	36750000	37150000	400001	LOC102169124
ROH_121	18	36750000	37150000	400001	LOC108637978
ROH_121	18	36750000	37150000	400001	DUS2
ROH_121	18	36750000	37150000	400001	ESRP2
ROH_121	18	36750000	37150000	400001	PLA2G15
ROH_121	18	36750000	37150000	400001	SLC7A6

ROH_121	18	36750000	37150000	400001	SMPD3
ROH_121	18	36750000	37150000	400001	LOC108637977
ROH_121	18	36750000	37150000	400001	NFATC3
ROH_121	18	36750000	37150000	400001	PRMT7
ROH_122	18	51850000	51950000	100001	PRR19
ROH_122	18	51850000	51950000	100001	ERF
ROH_122	18	51850000	51950000	100001	GSK3A
ROH_122	18	51850000	51950000	100001	MEGF8
ROH_122	18	51850000	51950000	100001	TMEM145
ROH_122	18	51850000	51950000	100001	PAFAH1B3
ROH_122	18	51850000	51950000	100001	CIC
ROH_122	18	51850000	51950000	100001	ZNF526
ROH_122	18	51850000	51950000	100001	DEDD2
ROH_123	18	56950000	57100000	150001	RPS11
ROH_123	18	56950000	57100000	150001	MIR150
ROH_123	18	56950000	57100000	150001	FCGRT
ROH_123	18	56950000	57100000	150001	PRRG2
ROH_123	18	56950000	57100000	150001	RRAS
ROH_123	18	56950000	57100000	150001	IRF3
ROH_123	18	56950000	57100000	150001	PRMT1
ROH_123	18	56950000	57100000	150001	RPL13A
ROH_123	18	56950000	57100000	150001	RCN3
ROH_123	18	56950000	57100000	150001	NOSIP
ROH_123	18	56950000	57100000	150001	SCAF1
ROH_123	18	56950000	57100000	150001	BCL2L12
ROH_123	18	56950000	57100000	150001	ADM5
ROH_123	18	56950000	57100000	150001	CPT1C
ROH_123	18	56950000	57100000	150001	PRR12
ROH_124	19	20500000	20650000	150001	TP53I13
ROH_124	19	20500000	20650000	150001	ABHD15
ROH_124	19	20500000	20650000	150001	TAOK1
ROH_125	19	22850000	23000000	150001	OVCA2
ROH_125	19	22850000	23000000	150001	DPH1
ROH_125	19	22850000	23000000	150001	HIC1
ROH_125	19	22850000	23000000	150001	SMG6
ROH_126	19	27050000	27200000	150001	CD68
ROH_126	19	27050000	27200000	150001	MPDU1
ROH_126	19	27050000	27200000	150001	SOX15
ROH_126	19	27050000	27200000	150001	SHBG
ROH_126	19	27050000	27200000	150001	ATP1B2
ROH_126	19	27050000	27200000	150001	EFNB3
ROH_126	19	27050000	27200000	150001	EIF4A1
ROH_126	19	27050000	27200000	150001	FXR2
ROH_126	19	27050000	27200000	150001	SAT2
ROH_126	19	27050000	27200000	150001	TP53
ROH_126	19	27050000	27200000	150001	DNAH2
ROH_126	19	27050000	27200000	150001	WRAP53
ROH_127	19	45750000	45800000	50001	KANSL1
ROH_128	20	25450000	25500000	50001	NDUFS4
ROH_129	20	38850000	39000000	150001	PRLR
ROH_131	21	41800000	42050000	250001	ARHGAP5
ROH_132	21	64250000	64450000	200001	MIR342
ROH_132	21	64250000	64450000	200001	LOC108638482
ROH_132	21	64250000	64450000	200001	DEGS2
ROH_132	21	64250000	64450000	200001	YY1
ROH_132	21	64250000	64450000	200001	EVL
ROH_133	22	16200000	16350000	150001	ZNF852
ROH_133	22	16200000	16350000	150001	ZNF502

ROH_133	22	16200000	16350000	150001	ZNF501
ROH_133	22	16200000	16350000	150001	KIAA1143
ROH_133	22	16200000	16350000	150001	LOC102177570
ROH_133	22	16200000	16350000	150001	KIF15
ROH_134	22	17250000	17550000	300001	SRGAP3
ROH_134	22	17250000	17550000	300001	RAD18
ROH_135	22	28850000	28900000	50001	SHQ1
ROH_136	22	29000000	29150000	150001	RYBP
ROH_137	22	49850000	50000000	150001	HEMK1
ROH_137	22	49850000	50000000	150001	LOC108633349
ROH_137	22	49850000	50000000	150001	C22H3orf18
ROH_137	22	49850000	50000000	150001	CACNA2D2
ROH_138	23	8600000	8900000	300001	JARID2
ROH_138	23	8600000	8900000	300001	DTNBP1
ROH_139	23	19400000	19500000	100001	LOC102177207
ROH_139	23	19400000	19500000	100001	TRNAS-GCU-17
ROH_139	23	19400000	19500000	100001	ZSCAN9
ROH_139	23	19400000	19500000	100001	ZKSCAN4
ROH_139	23	19400000	19500000	100001	LOC108633266
ROH_139	23	19400000	19500000	100001	ZKSCAN8
ROH_140	23	22350000	22450000	100001	LOC102180547
ROH_140	23	22350000	22450000	100001	LY6G6D
ROH_140	23	22350000	22450000	100001	LY6G6C
ROH_140	23	22350000	22450000	100001	C23H6orf25
ROH_140	23	22350000	22450000	100001	CLIC1
ROH_140	23	22350000	22450000	100001	SAPCD1
ROH_140	23	22350000	22450000	100001	VARS
ROH_140	23	22350000	22450000	100001	LSM2
ROH_140	23	22350000	22450000	100001	HSP70.1
ROH_140	23	22350000	22450000	100001	ABHD16A
ROH_140	23	22350000	22450000	100001	LY6G6F
ROH_140	23	22350000	22450000	100001	DDAH2
ROH_140	23	22350000	22450000	100001	MSH5
ROH_140	23	22350000	22450000	100001	LOC102177850
ROH_140	23	22350000	22450000	100001	VWA7
ROH_141	23	30250000	30350000	100001	RUNX2
ROH_141	23	30250000	30350000	100001	SUPT3H
ROH_142	23	30500000	30650000	150001	SUPT3H
ROH_143	23	39950000	40100000	150001	C23H6orf106
ROH_143	23	39950000	40100000	150001	SPDEF
ROH_144	24	22000000	22050000	50001	ZNF24
ROH_145	24	34750000	34800000	50001	LOC106503532
ROH_145	24	34750000	34800000	50001	MIB1
ROH_146	24	43550000	43850000	300001	SEH1L
ROH_146	24	43550000	43850000	300001	CEP192
ROH_146	24	43550000	43850000	300001	FAM210A
ROH_146	24	43550000	43850000	300001	RNMT
ROH_146	24	43550000	43850000	300001	LDLRAD4
ROH_147	25	150000	300000	150001	LOC108633874
ROH_147	25	150000	300000	150001	HBM
ROH_147	25	150000	300000	150001	LOC102168680
ROH_147	25	150000	300000	150001	LOC102168959
ROH_147	25	150000	300000	150001	LOC102186172
ROH_147	25	150000	300000	150001	RGS11
ROH_147	25	150000	300000	150001	ARHGDIG
ROH_147	25	150000	300000	150001	MRPL28
ROH_147	25	150000	300000	150001	LUC7L
ROH_147	25	150000	300000	150001	FAM234A

ROH_147	25	150000	300000	150001	PDIA2
ROH_147	25	150000	300000	150001	TMEM8A
ROH_147	25	150000	300000	150001	AXIN1
ROH_148	25	3750000	3800000	50001	ZNF500
ROH_148	25	3750000	3800000	50001	SEPT12
ROH_148	25	3750000	3800000	50001	ROGDI
ROH_149	28	21050000	21200000	150001	HERC4
