# Supplementary material for: Whole-genome selection signatures identified candidate genes associated with cashmere traits in Inner Mongolia cashmere goats
Source: Anim Biosci. 2025 Jul 11;38(12):2597–611. doi: 10.5713/ab.25.0252 (PMC12580777; doi:10.5713/ab.25.0252)
Supplement: Supplementary file 6 [file ab-25-0252-Supplementary-6.pdf]

Supplement 6. Detected and gene annotation of candidate regions by F_{ST} (HYCG VS LYCG

Chr	Start	End	WEIGHTED_FST	Gene name
8	38570001	38620000	0.0615978	KIAA2026
9	1870001	1920000	0.057957	COL12A1
9	1880001	1930000	0.0576434	COL12A1
9	1860001	1910000	0.0573936	COL12A1
9	1890001	1940000	0.0567913	COL12A1
8	38580001	38630000	0.0565469	KIAA2026
8	38560001	38610000	0.0558377	KIAA2026
8	38550001	38600000	0.0552228	KIAA2026
19	62220001	62270000	0.0521751	CACNG4
12	13140001	13190000	0.0518397	DNAJC3
12	13140001	13190000	0.0518397	UGGT2
19	62210001	62260000	0.0512351	CACNG4
14	12990001	13040000	0.0507714	PLEKHF2
1	101680001	101730000	0.0500603	SLITRK3
18	12370001	12420000	0.0497444	CRISPLD2
14	13000001	13050000	0.0489846	LOC106502853
14	13000001	13050000	0.0489846	PLEKHF2
19	62280001	62330000	0.0488546	CACNG1
19	62280001	62330000	0.0488546	HELZ
19	62230001	62280000	0.04806	CACNG1
19	62230001	62280000	0.04806	CACNG4
19	62240001	62290000	0.0479285	CACNG1
19	62240001	62290000	0.0479285	CACNG4
9	1900001	1950000	0.047907	COL12A1
8	38540001	38590000	0.0479031	KIAA2026
25	9870001	9920000	0.0477239	RMI2
25	9870001	9920000	0.0477239	LOC102173909
15	8590001	8640000	0.047497	ALX4
19	62270001	62320000	0.0465598	CACNG1
19	62270001	62320000	0.0465598	HELZ
18	12380001	12430000	0.0464767	CRISPLD2
18	12360001	12410000	0.0464477	CRISPLD2
18	12350001	12400000	0.0462364	CRISPLD2
1	101670001	101720000	0.0455603	SLITRK3
29	31080001	31130000	0.0454819	LOC106503726
1	101690001	101740000	0.0454718	SLITRK3
6	108910001	108960000	0.0454541	RAB28
15	8600001	8650000	0.0451199	ALX4
19	62260001	62310000	0.0448009	CACNG1
19	62260001	62310000	0.0448009	CACNG4
19	62260001	62310000	0.0448009	HELZ
25	9880001	9930000	0.0446143	LOC102173909
18	12390001	12440000	0.0446048	CRISPLD2
19	18950001	19000000	0.0444365	LOC102189615
14	12980001	13030000	0.0443321	PLEKHF2
29	31090001	31140000	0.0440702	LOC106503726
29	31090001	31140000	0.0440702	ETS1
15	8610001	8660000	0.0438705	ALX4
15	8610001	8660000	0.0438705	EXT2
19	62250001	62300000	0.0438602	CACNG1
19	62250001	62300000	0.0438602	CACNG4
19	62250001	62300000	0.0438602	HELZ
19	18940001	18990000	0.0438246	LOC102189615
6	108920001	108970000	0.0436931	RAB28
8	38750001	38800000	0.0436285	ERMP1
8	38750001	38800000	0.0436285	RIC1

6	108900001	108950000	0.0435791	RAB28
19	18960001	19010000	0.0432677	LOC106503912
19	18960001	19010000	0.0432677	LOC102189615
6	108890001	108940000	0.0432136	RAB28
8	39250001	39300000	0.04316	JAK2
12	13130001	13180000	0.0430748	DNAJC3
12	13130001	13180000	0.0430748	UGGT2
2	109130001	109180000	0.0427932	ABCB11
29	31070001	31120000	0.0420891	LOC106503726
15	8620001	8670000	0.0419835	ALX4
15	8620001	8670000	0.0419835	EXT2
19	18970001	19020000	0.0417683	LOC106503912
19	18970001	19020000	0.0417683	LOC102189615
6	108880001	108930000	0.0417083	RAB28
6	108870001	108920000	0.0417016	RAB28
1	51690001	51740000	0.0416395	LOC102171918
6	108930001	108980000	0.0416014	RAB28
9	1850001	1900000	0.0415789	COL12A1
24	49650001	49700000	0.0415121	LOC106503542
24	49650001	49700000	0.0415121	ACAA2
24	49650001	49700000	0.0415121	MYO5B
12	13150001	13200000	0.0414049	DNAJC3
12	13150001	13200000	0.0414049	UGGT2
19	62200001	62250000	0.041059	CACNG4
8	38740001	38790000	0.0406571	ERMP1
8	38740001	38790000	0.0406571	RIC1
6	70050001	70100000	0.0405494	CHIC2
15	8580001	8630000	0.0405075	ALX4
8	38530001	38580000	0.040465	KIAA2026
2	109140001	109190000	0.0403906	ABCB11
18	12400001	12450000	0.0402982	CRISPLD2
8	38590001	38640000	0.039991	MLANA
8	38590001	38640000	0.039991	KIAA2026
19	62290001	62340000	0.0397519	HELZ
18	12340001	12390000	0.0396741	CRISPLD2
24	49640001	49690000	0.0395643	LOC106503542
24	49640001	49690000	0.0395643	ACAA2
24	49640001	49690000	0.0395643	MYO5B
12	13840001	13890000	0.0394724	LOC108637251
12	13850001	13900000	0.0394724	LOC108637251
12	13860001	13910000	0.0394724	LOC108637251
12	13870001	13920000	0.0394724	LOC108637251
12	13880001	13930000	0.0394724	LOC108637252
12	13880001	13930000	0.0394724	LOC108637251
7	68880001	68930000	0.0394631	LOC102172144
7	68880001	68930000	0.0394631	LOC102171865
7	68890001	68940000	0.0394631	LOC102172144
7	68890001	68940000	0.0394631	LOC102171865
25	9890001	9940000	0.039374	LOC102173909
8	38490001	38540000	0.0392862	RANBP6
8	38490001	38540000	0.0392862	KIAA2026
19	18930001	18980000	0.0392167	LOC102187672
14	13010001	13060000	0.038911	LOC106502853
14	13010001	13060000	0.038911	PLEKHF2
15	8660001	8710000	0.0388949	EXT2
25	9860001	9910000	0.0388938	RMI2
8	39260001	39310000	0.038881	JAK2
5	31330001	31380000	0.0385625	LOC108636097

15	8630001	8680000	0.0385251	ALX4
15	8630001	8680000	0.0385251	EXT2
14	12970001	13020000	0.0383089	PLEKHF2
2	109120001	109170000	0.0382235	ABCB11
24	49660001	49710000	0.0382203	LOC106503542
24	49660001	49710000	0.0382203	ACAA2
24	49660001	49710000	0.0382203	MYO5B
4	7720001	7770000	0.0382086	LOC102168236
4	7730001	7780000	0.0378522	LOC102168236
6	70060001	70110000	0.0378111	CHIC2
12	10360001	10410000	0.0376474	DOCK9
29	31100001	31150000	0.0376055	ETS1
18	12410001	12460000	0.0375882	CRISPLD2
22	12320001	12370000	0.0374604	LOC108638642
22	12320001	12370000	0.0374604	XIRP1
22	12320001	12370000	0.0374604	CSRNP1
22	12320001	12370000	0.0374604	TTC21A
15	8650001	8700000	0.0374011	EXT2
12	13160001	13210000	0.037321	DNAJC3
12	13160001	13210000	0.037321	UGGT2
3	13650001	13700000	0.0372852	LOC106501986
3	13650001	13700000	0.0372852	GJA9
3	13650001	13700000	0.0372852	RRAGC
3	13650001	13700000	0.0372852	MYCBP
3	13650001	13700000	0.0372852	RHBDL2
2	112240001	112290000	0.0372623	PDK1
14	12920001	12970000	0.0371945	NDUFAF6
2	109110001	109160000	0.0371836	ABCB11
8	38480001	38530000	0.0370536	RANBP6
8	38480001	38530000	0.0370536	KIAA2026
10	66200001	66250000	0.0369351	LOC102182981
10	66200001	66250000	0.0369351	NDUFAF1
10	66200001	66250000	0.0369351	NUSAP1
10	66200001	66250000	0.0369351	RTF1
15	8680001	8730000	0.0368905	EXT2
4	7710001	7760000	0.0367891	LOC106501766
4	7710001	7760000	0.0367891	LOC102168236
4	7740001	7790000	0.0366835	GIMAP8
12	10370001	10420000	0.0366476	DOCK9
1	96730001	96780000	0.0363191	PHC3
7	68900001	68950000	0.0363016	LOC102172144
7	68900001	68950000	0.0363016	LOC102171865
7	68900001	68950000	0.0363016	LOC102171598
10	66190001	66240000	0.0362188	TRNAW-CCA-59
10	66190001	66240000	0.0362188	LOC102182981
10	66190001	66240000	0.0362188	NDUFAF1
10	66190001	66240000	0.0362188	RTF1
8	38760001	38810000	0.03584	ERMP1
8	38760001	38810000	0.03584	RIC1
29	31820001	31870000	0.0357613	ARHGAP32
8	38520001	38570000	0.0357016	KIAA2026
24	42770001	42820000	0.0356823	PIEZO2
19	27810001	27860000	0.0356145	MYH10
15	8670001	8720000	0.0355883	EXT2
12	13120001	13170000	0.0355339	DNAJC3
12	13120001	13170000	0.0355339	UGGT2
1	65120001	65170000	0.0354876	GTF2E1
1	146000001	146050000	0.0353877	PCNT

1	96750001	96800000	0.035369	LOC102179814
1	96750001	96800000	0.035369	PHC3
10	66180001	66230000	0.0352776	TRNAW-CCA-59
10	66180001	66230000	0.0352776	LOC102182981
10	66180001	66230000	0.0352776	NDUFAF1
10	66180001	66230000	0.0352776	RTF1
8	39240001	39290000	0.0352697	JAK2
19	18980001	19030000	0.0350904	LOC106503912
19	18980001	19030000	0.0350904	LOC108638256
19	18980001	19030000	0.0350904	LOC102189615
8	38730001	38780000	0.0350284	ERMP1
8	38730001	38780000	0.0350284	RIC1
8	38470001	38520000	0.0349652	RANBP6
8	38470001	38520000	0.0349652	KIAA2026
1	96760001	96810000	0.0349541	LOC102179814
1	96760001	96810000	0.0349541	PHC3
25	9900001	9950000	0.0348384	LOC102173909
18	12330001	12380000	0.0347933	CRISPLD2
15	8640001	8690000	0.0347564	ALX4
15	8640001	8690000	0.0347564	EXT2
14	46180001	46230000	0.0346993	LOC108637480
14	46180001	46230000	0.0346993	KCNB2
24	49670001	49720000	0.0346761	MYO5B
10	66170001	66220000	0.0345492	TRNAW-CCA-59
10	66170001	66220000	0.0345492	RTF1
8	38500001	38550000	0.0344928	RANBP6
8	38500001	38550000	0.0344928	KIAA2026
8	38710001	38760000	0.0344778	ERMP1
8	38720001	38770000	0.0344347	ERMP1
4	96650001	96700000	0.0343243	AGMO
1	96740001	96790000	0.0342842	LOC102179814
1	96740001	96790000	0.0342842	PHC3
2	112230001	112280000	0.0342839	PDK1
13	8950001	9000000	0.0342362	TRNAW-CCA-67
13	8950001	9000000	0.0342362	MACROD2
14	12930001	12980000	0.0342138	NDUFAF6
6	110420001	110470000	0.0341857	CPEB2
19	62300001	62350000	0.0341653	HELZ
7	58300001	58350000	0.0341403	LOC102188217
5	86910001	86960000	0.0339747	ABCC9
18	64890001	64940000	0.0338893	LOC102171541
18	64890001	64940000	0.0338893	LOC102168291
18	64890001	64940000	0.0338893	LOC102171271
18	64890001	64940000	0.0338893	LOC102191644
18	64890001	64940000	0.0338893	LOC102171004
8	38510001	38560000	0.0338887	RANBP6
8	38510001	38560000	0.0338887	KIAA2026
5	86920001	86970000	0.0338416	ABCC9
6	70070001	70120000	0.033823	CHIC2
12	10350001	10400000	0.0338185	DOCK9
6	114810001	114860000	0.033558	ACOX3
25	9850001	9900000	0.0335127	RMI2
1	51680001	51730000	0.0334574	LOC102171918
15	8690001	8740000	0.0334432	EXT2
29	31810001	31860000	0.0334343	ARHGAP32
1	118540001	118590000	0.0334234	CP
1	118560001	118610000	0.0334038	CP
1	118560001	118610000	0.0334038	HPS3

12	13170001	13220000	0.0333499	DNAJC3
18	64880001	64930000	0.0333011	LOC102171541
18	64880001	64930000	0.0333011	LOC102168291
18	64880001	64930000	0.0333011	LOC102171271
18	64880001	64930000	0.0333011	LOC102191644
18	64880001	64930000	0.0333011	TARM1
19	27820001	27870000	0.0332864	MYH10
14	46190001	46240000	0.0332576	LOC108637480
14	46190001	46240000	0.0332576	KCNB2
1	118550001	118600000	0.03318	CP
1	118550001	118600000	0.03318	HPS3
18	64860001	64910000	0.033152	NDUFA3
18	64860001	64910000	0.033152	LOC102171541
18	64860001	64910000	0.033152	LOC102168291
18	64860001	64910000	0.033152	OSCAR
18	64860001	64910000	0.033152	TARM1
18	64870001	64920000	0.0331265	LOC102171541
18	64870001	64920000	0.0331265	LOC102168291
18	64870001	64920000	0.0331265	LOC102171271
18	64870001	64920000	0.0331265	OSCAR
18	64870001	64920000	0.0331265	TARM1
29	31930001	31980000	0.0330686	BARX2
26	28140001	28190000	0.0330351	CNNM2
8	38770001	38820000	0.0329389	ERMP1
8	38770001	38820000	0.0329389	RIC1
1	65130001	65180000	0.032903	GTF2E1
14	12910001	12960000	0.0328596	NDUFAF6
5	86900001	86950000	0.0328348	ABCC9
22	10210001	10260000	0.0327587	LRRFIP2
8	39270001	39320000	0.0327254	JAK2
12	15260001	15310000	0.0326946	LOC102180583
6	70040001	70090000	0.0326406	CHIC2
6	117570001	117620000	0.0326331	PIGG
7	58290001	58340000	0.0326126	LOC102188497
7	58290001	58340000	0.0326126	LOC102188217
1	65110001	65160000	0.0325769	RABL3
1	65110001	65160000	0.0325769	GTF2E1
18	12420001	12470000	0.0325191	CRISPLD2
2	111680001	111730000	0.0325141	HAT1
6	117580001	117630000	0.0324875	PIGG
6	110430001	110480000	0.0324812	CPEB2
19	27800001	27850000	0.032459	MYH10
29	31900001	31950000	0.0324038	BARX2
5	86890001	86940000	0.0323932	ABCC9
10	66160001	66210000	0.0323696	TRNAW-CCA-59
10	66160001	66210000	0.0323696	RTF1
8	38700001	38750000	0.0322794	ERMP1
8	38460001	38510000	0.0322325	RANBP6
4	96660001	96710000	0.0322319	AGMO
7	83730001	83780000	0.0321485	C7H5orf63
1	96770001	96820000	0.0321484	LOC102179814
1	96770001	96820000	0.0321484	PHC3
1	96770001	96820000	0.0321484	GPR160
4	7700001	7750000	0.0320421	LOC106501766
4	7700001	7750000	0.0320421	LOC102168236
22	11440001	11490000	0.0320315	ACAA1
22	11440001	11490000	0.0320315	MYD88
22	11440001	11490000	0.0320315	DLEC1

2	109150001	109200000	0.0319879	ABCB11
2	109150001	109200000	0.0319879	DHRS9
2	112220001	112270000	0.0319852	PDK1
12	10290001	10340000	0.031968	DOCK9
12	10300001	10350000	0.0319663	TRNAS-GGA-68
12	10300001	10350000	0.0319663	DOCK9
28	10220001	10270000	0.0319172	SFTPD
11	61180001	61230000	0.0318827	OTX1
11	61180001	61230000	0.0318827	EHBP1
26	15080001	15130000	0.0318574	PNLIPRP3
19	62310001	62360000	0.0318443	HELZ
5	26750001	26800000	0.0318325	LOC108636128
5	26750001	26800000	0.0318325	KRT8
20	37490001	37540000	0.0318296	SLC1A3
6	114800001	114850000	0.031793	ACOX3
1	52090001	52140000	0.0317287	BBX
28	10210001	10260000	0.0316967	SFTPD
24	42760001	42810000	0.0316694	PIEZO2
11	61160001	61210000	0.0316388	EHBP1
18	12210001	12260000	0.0315596	KLHL36
18	12210001	12260000	0.0315596	COTL1
25	9910001	9960000	0.0314482	LOC102173909
7	58310001	58360000	0.0313865	PCDHB1
7	58310001	58360000	0.0313865	LOC102188217
16	45100001	45150000	0.031365	CAMTA1
8	39100001	39150000	0.0313644	PLGRKT
10	75930001	75980000	0.0312299	LOC108636865
10	75930001	75980000	0.0312299	LOC102177429
1	52080001	52130000	0.0311741	BBX
6	116710001	116760000	0.0311557	MFSD10
6	116710001	116760000	0.0311557	LOC108636244
6	116710001	116760000	0.0311557	NOP14
6	116710001	116760000	0.0311557	ADD1
12	50060001	50110000	0.0311496	ATP12A
12	50050001	50100000	0.0311481	ATP12A
22	10220001	10270000	0.0311445	LRRFIP2
1	118570001	118620000	0.0311234	CP
1	118570001	118620000	0.0311234	HPS3
1	51300001	51350000	0.0310836	LOC108636831
29	31940001	31990000	0.0310231	BARX2
16	45110001	45160000	0.0310097	CAMTA1
7	58280001	58330000	0.0310074	LOC102188767
7	58280001	58330000	0.0310074	LOC102188497
7	58280001	58330000	0.0310074	LOC102188217
8	39110001	39160000	0.0309918	PLGRKT
16	78850001	78900000	0.0309703	LGR6
16	78850001	78900000	0.0309703	LOC102168445
10	49910001	49960000	0.0309698	TCF12
29	31880001	31930000	0.030911	BARX2
18	56320001	56370000	0.0308867	NTN5
18	56320001	56370000	0.0308867	LOC102178850
18	56320001	56370000	0.0308867	LOC102185028
12	10380001	10430000	0.0308255	DOCK9
8	39280001	39330000	0.0308002	JAK2
4	96680001	96730000	0.0307636	AGMO
4	70510001	70560000	0.0307478	EPDR1
11	61190001	61240000	0.0307446	OTX1
11	61190001	61240000	0.0307446	EHBP1

2	83920001	83970000	0.0307222	GTDC1
6	116720001	116770000	0.0306992	MFSD10
6	116720001	116770000	0.0306992	LOC108636244
6	116720001	116770000	0.0306992	NOP14
6	116720001	116770000	0.0306992	ADD1
6	116720001	116770000	0.0306992	GRK4
2	112250001	112300000	0.0306941	PDK1
13	8940001	8990000	0.030682	TRNAW-CCA-67
13	8940001	8990000	0.030682	MACROD2
10	75920001	75970000	0.0306789	LOC102176964
10	75920001	75970000	0.0306789	LOC108636865
4	70520001	70570000	0.0306473	EPDR1
4	70520001	70570000	0.0306473	SFRP4
26	15070001	15120000	0.0306262	PNLIPRP3
1	52140001	52190000	0.0306258	BBX
2	111690001	111740000	0.0306108	HAT1
19	62320001	62370000	0.030569	HELZ
2	112210001	112260000	0.0305503	PDK1
8	39230001	39280000	0.0305362	JAK2
6	117600001	117650000	0.0305109	PIGG
4	86960001	87010000	0.0305017	KIAA1324L
15	8700001	8750000	0.030493	EXT2
1	51310001	51360000	0.0304919	LOC108636831
10	75940001	75990000	0.0304661	LOC108636865
10	75940001	75990000	0.0304661	LOC102177429
24	49630001	49680000	0.0304588	LOC106503542
24	49630001	49680000	0.0304588	ACAA2
24	49630001	49680000	0.0304588	MYO5B
28	17400001	17450000	0.0304548	ASCC1
16	78840001	78890000	0.0304333	LOC102178170
16	78840001	78890000	0.0304333	LGR6
16	78840001	78890000	0.0304333	LOC102168445
13	8960001	9010000	0.0303484	MACROD2
3	119690001	119740000	0.0303305	TIPRL
10	66210001	66260000	0.0302831	LOC102182981
10	66210001	66260000	0.0302831	NDUFAF1
10	66210001	66260000	0.0302831	NUSAP1
18	56310001	56360000	0.0302696	TRNAE-UUC-76
18	56310001	56360000	0.0302696	NTN5
18	56310001	56360000	0.0302696	LOC102178850
18	56310001	56360000	0.0302696	LOC102185028
18	56310001	56360000	0.0302696	CA11
6	108860001	108910000	0.0302657	RAB28
19	27790001	27840000	0.0302605	NDEL1
19	27790001	27840000	0.0302605	MYH10
12	63210001	63260000	0.0302382	FREM2
1	146010001	146060000	0.0302023	PCNT
10	49880001	49930000	0.0301766	TCF12
12	63200001	63250000	0.0301742	FREM2
2	111700001	111750000	0.0301614	HAT1
2	111700001	111750000	0.0301614	METAP1D
4	86950001	87000000	0.0301571	KIAA1324L
1	52070001	52120000	0.0301524	BBX
22	16740001	16790000	0.0301445	TADA3
22	16740001	16790000	0.0301445	OGG1
22	16740001	16790000	0.0301445	ARPC4
22	16740001	16790000	0.0301445	BRPF1
22	16740001	16790000	0.0301445	CAMK1

8	10780001	10830000	0.0300805	PBK
8	10780001	10830000	0.0300805	SCARA5
12	13110001	13160000	0.0300677	UGGT2
29	31910001	31960000	0.0300486	BARX2
10	49900001	49950000	0.0300411	TCF12
1	145990001	146040000	0.0300242	PCNT
7	41410001	41460000	0.0299897	NIPAL4
7	41410001	41460000	0.0299897	ADAM19
1	52150001	52200000	0.0299531	BBX
10	49850001	49900000	0.0299309	TCF12
5	26760001	26810000	0.029924	KRT8
5	26760001	26810000	0.029924	LOC108635996
11	61170001	61220000	0.0299073	EHBP1
3	7030001	7080000	0.0298908	SPP2
22	11450001	11500000	0.0298839	ACAA1
22	11450001	11500000	0.0298839	MYD88
22	11450001	11500000	0.0298839	DLEC1
13	8970001	9020000	0.0298777	MACROD2
12	10340001	10390000	0.0298492	TRNAS-GGA-68
12	10340001	10390000	0.0298492	DOCK9
12	55190001	55240000	0.0298483	LOC102188230
1	96720001	96770000	0.0298326	PHC3
10	49890001	49940000	0.0298247	TCF12
19	27450001	27500000	0.0298193	LOC102172190
19	27450001	27500000	0.0298193	TRNAK-UUU-24
19	27450001	27500000	0.0298193	TRNAQ-CUG-12
19	27450001	27500000	0.0298193	TRNAL-UAG-3
19	27450001	27500000	0.0298193	HES7
19	27450001	27500000	0.0298193	TRNAR-UCU-8
19	27450001	27500000	0.0298193	ALOX12B
19	27450001	27500000	0.0298193	ALOXE3
19	27450001	27500000	0.0298193	LOC108638269
29	31830001	31880000	0.029801	ARHGAP32
7	58270001	58320000	0.0297736	LOC102188767
7	58270001	58320000	0.0297736	LOC102188497
7	58270001	58320000	0.0297736	LOC102188217
1	52100001	52150000	0.0297706	BBX
4	96670001	96720000	0.0297529	AGMO
29	31890001	31940000	0.0297401	BARX2
15	4370001	4420000	0.0297291	LOC102183417
15	4370001	4420000	0.0297291	LOC102183146
15	4370001	4420000	0.0297291	LOC108637601
15	4370001	4420000	0.0297291	LOC102190601
18	56300001	56350000	0.0297271	DBP
18	56300001	56350000	0.0297271	TRNAE-UUC-76
18	56300001	56350000	0.0297271	NTN5
18	56300001	56350000	0.0297271	LOC102178850
18	56300001	56350000	0.0297271	LOC102185028
18	56300001	56350000	0.0297271	CA11
18	12220001	12270000	0.0297252	KLHL36
18	12220001	12270000	0.0297252	USP10
19	62340001	62390000	0.029716	HELZ
2	109100001	109150000	0.0297111	ABCB11
4	100870001	100920000	0.0296906	THSD7A
16	45070001	45120000	0.0296529	CAMTA1
8	39090001	39140000	0.0296224	CD274
8	39090001	39140000	0.0296224	PLGRKT
11	61200001	61250000	0.0296181	OTX1

11	61200001	61250000	0.0296181	EHBP1
8	39120001	39170000	0.0296154	PLGRKT
6	117590001	117640000	0.0295934	PIGG
16	45090001	45140000	0.0295787	CAMTA1
3	13640001	13690000	0.0295607	LOC106501986
3	13640001	13690000	0.0295607	GJA9
3	13640001	13690000	0.0295607	RRAGC
3	13640001	13690000	0.0295607	MYCBP
14	46170001	46220000	0.0295597	KCNB2
16	45120001	45170000	0.0295378	CAMTA1
10	49870001	49920000	0.0295274	TCF12
19	27830001	27880000	0.0295235	MYH10
16	45080001	45130000	0.0295177	CAMTA1
14	12900001	12950000	0.0294607	NDUFAF6
1	52110001	52160000	0.0294591	BBX
8	38680001	38730000	0.0294569	ERMP1
18	12230001	12280000	0.0294347	KLHL36
18	12230001	12280000	0.0294347	USP10
28	10200001	10250000	0.0294163	LOC102175466
28	10200001	10250000	0.0294163	SFTPD
28	15130001	15180000	0.0294068	ADK
7	41400001	41450000	0.0294036	ADAM19
1	1380001	1430000	0.0294027	C1H21orf59
6	108850001	108900000	0.029276	RAB28
12	8080001	8130000	0.0292449	FGF14
6	117560001	117610000	0.029206	LOC106502244
6	117560001	117610000	0.029206	PIGG
28	17410001	17460000	0.0292026	SPOCK2
28	17410001	17460000	0.0292026	ASCC1
12	8070001	8120000	0.0292018	FGF14
22	12330001	12380000	0.0291938	LOC108638642
22	12330001	12380000	0.0291938	XIRP1
22	12330001	12380000	0.0291938	CSRNP1
4	70500001	70550000	0.0291817	EPDR1
1	52170001	52220000	0.0291748	BBX
12	50400001	50450000	0.0291538	MPHOSPH8
12	50400001	50450000	0.0291538	PSPC1
8	38690001	38740000	0.0291385	ERMP1
19	62350001	62400000	0.029135	HELZ
10	49920001	49970000	0.0291271	TCF12
8	39290001	39340000	0.0291167	JAK2
1	118530001	118580000	0.0290328	CP
14	91190001	91240000	0.0290127	FAM135A
14	91200001	91250000	0.0290097	FAM135A
10	65470001	65520000	0.0289209	VPS39
10	65470001	65520000	0.0289209	PLA2G4F
12	13100001	13150000	0.0289072	UGGT2
10	49860001	49910000	0.0289046	TCF12
28	15120001	15170000	0.0288873	ADK
6	110450001	110500000	0.0288621	CPEB2
3	13660001	13710000	0.0288399	LOC106501986
3	13660001	13710000	0.0288399	GJA9
3	13660001	13710000	0.0288399	RRAGC
3	13660001	13710000	0.0288399	MYCBP
3	13660001	13710000	0.0288399	RHBDL2
2	83930001	83980000	0.0288156	GTDC1
24	42780001	42830000	0.0288076	PIEZO2
14	91170001	91220000	0.0288024	FAM135A

14	13080001	13130000	0.0288017	C14H8orf37
8	39660001	39710000	0.0287874	PLPP6
8	39660001	39710000	0.0287874	CDC37L1
8	39670001	39720000	0.028762	PLPP6
8	39670001	39720000	0.028762	CDC37L1
8	39670001	39720000	0.028762	LOC108636555
18	56330001	56380000	0.0287605	LOC102178850
18	56330001	56380000	0.0287605	LOC102185028
18	56330001	56380000	0.0287605	MAMSTR
12	10390001	10440000	0.0287554	DOCK9
22	16750001	16800000	0.0287458	TADA3
22	16750001	16800000	0.0287458	OGG1
22	16750001	16800000	0.0287458	BRPF1
22	16750001	16800000	0.0287458	CAMK1
26	28150001	28200000	0.0287333	CNNM2
19	27440001	27490000	0.0287059	LOC102169702
19	27440001	27490000	0.0287059	LOC102172658
19	27440001	27490000	0.0287059	LOC102172190
19	27440001	27490000	0.0287059	ALOX12B
19	27440001	27490000	0.0287059	ALOXE3
5	25640001	25690000	0.0286662	LOC106502088
28	15140001	15190000	0.0286261	ADK
13	7360001	7410000	0.0286234	MACROD2
29	41290001	41340000	0.0285817	B3GAT3
29	41290001	41340000	0.0285817	INTS5
29	41290001	41340000	0.0285817	LBHD1
29	41290001	41340000	0.0285817	METTL12
29	41290001	41340000	0.0285817	LOC106503742
29	41290001	41340000	0.0285817	GANAB
29	41290001	41340000	0.0285817	UBXN1
11	61220001	61270000	0.0285716	OTX1
11	61220001	61270000	0.0285716	EHBP1
22	10200001	10250000	0.0285685	LRRFIP2
12	10310001	10360000	0.0284936	TRNAS-GGA-68
12	10310001	10360000	0.0284936	DOCK9
19	62360001	62410000	0.028488	HELZ
19	62330001	62380000	0.0284497	HELZ
3	7020001	7070000	0.0283575	SPP2
15	8730001	8780000	0.0283471	EXT2
18	64850001	64900000	0.0283228	TFPT
18	64850001	64900000	0.0283228	NDUFA3
18	64850001	64900000	0.0283228	LOC102171541
18	64850001	64900000	0.0283228	PRPF31
18	64850001	64900000	0.0283228	OSCAR
18	64850001	64900000	0.0283228	TARM1
11	61140001	61190000	0.0283134	EHBP1
12	50410001	50460000	0.0283083	MPHOSPH8
12	50410001	50460000	0.0283083	PSPC1
4	100850001	100900000	0.0282022	THSD7A
4	86970001	87020000	0.0281992	KIAA1324L
28	17420001	17470000	0.0281787	SPOCK2
28	17420001	17470000	0.0281787	ASCC1
7	68910001	68960000	0.0281544	LOC102171865
7	68910001	68960000	0.0281544	LOC102171598
29	31060001	31110000	0.0281544	LOC106503726
1	52160001	52210000	0.0281343	BBX
29	31920001	31970000	0.0281328	BARX2
11	61230001	61280000	0.0281225	OTX1

8	39130001	39180000	0.0281208	PLGRKT
14	91180001	91230000	0.0280825	FAM135A
7	83710001	83760000	0.0280633	3-Mar
7	83710001	83760000	0.0280633	C7H5orf63
18	56290001	56340000	0.0280604	RPL18
18	56290001	56340000	0.0280604	DBP
18	56290001	56340000	0.0280604	TRNAE-UUC-76
18	56290001	56340000	0.0280604	NTN5
18	56290001	56340000	0.0280604	LOC102178850
18	56290001	56340000	0.0280604	SPHK2
18	56290001	56340000	0.0280604	CA11
6	114820001	114870000	0.0280572	ACOX3
15	39590001	39640000	0.0280474	SBF2
22	16720001	16770000	0.0280053	TADA3
22	16720001	16770000	0.0280053	LOC102184009
22	16720001	16770000	0.0280053	ARPC4
4	100860001	100910000	0.0279599	THSD7A
22	12310001	12360000	0.0279514	LOC108638642
22	12310001	12360000	0.0279514	XIRP1
22	12310001	12360000	0.0279514	CSRNP1
22	12310001	12360000	0.0279514	TTC21A
2	83910001	83960000	0.0279349	GTDC1
4	7750001	7800000	0.0279232	GIMAP8
8	39080001	39130000	0.0279123	CD274
8	39080001	39130000	0.0279123	PLGRKT
24	42470001	42520000	0.0278955	APCDD1
3	119700001	119750000	0.027892	TIPRL
4	86980001	87030000	0.0278919	KIAA1324L
10	65480001	65530000	0.0278764	VPS39
10	65480001	65530000	0.0278764	PLA2G4F
12	50420001	50470000	0.0278647	PSPC1
18	12240001	12290000	0.0278573	KLHL36
18	12240001	12290000	0.0278573	USP10
14	13020001	13070000	0.0278275	LOC106502853
14	13020001	13070000	0.0278275	PLEKHF2
9	1840001	1890000	0.0278224	COL12A1
12	10330001	10380000	0.0277865	TRNAS-GGA-68
12	10330001	10380000	0.0277865	DOCK9
6	110440001	110490000	0.0277798	CPEB2
28	15110001	15160000	0.0277775	ADK
5	25630001	25680000	0.0277773	LOC106502088
6	106880001	106930000	0.0277642	TRNAC-GCA-105
22	17160001	17210000	0.0277399	SRGAP3
2	6630001	6680000	0.0277251	ID3
8	39650001	39700000	0.0277083	AK3
8	39650001	39700000	0.0277083	CDC37L1
16	49810001	49860000	0.0276906	MIR429
16	49810001	49860000	0.0276906	MIR200A
16	49810001	49860000	0.0276906	MIR200B
16	49810001	49860000	0.0276906	TTLL10
3	9170001	9220000	0.0276733	C3H1orf94
10	97120001	97170000	0.0276502	PGGT1B
24	43450001	43500000	0.0276376	PTPN2
5	22530001	22580000	0.0276375	EEA1
1	52120001	52170000	0.0276206	BBX
22	16730001	16780000	0.0276097	TADA3
22	16730001	16780000	0.0276097	LOC102184009
22	16730001	16780000	0.0276097	ARPC4

22	16730001	16780000	0.0276097	CAMK1
2	111020001	111070000	0.0275996	TLK1
16	78860001	78910000	0.027583	LGR6
16	78860001	78910000	0.027583	LOC102168445
16	78860001	78910000	0.027583	PTPN7
7	41390001	41440000	0.0275484	ADAM19
7	83720001	83770000	0.0275319	C7H5orf63
29	31950001	32000000	0.0275301	BARX2
4	86990001	87040000	0.0275055	KIAA1324L
10	65460001	65510000	0.0274935	TMEM87A
10	65460001	65510000	0.0274935	VPS39
5	68710001	68760000	0.0274917	RFX4
5	68710001	68760000	0.0274917	RIC8B
4	1560001	1610000	0.027489	LMBR1
26	15090001	15140000	0.0274719	PNLIPRP3
16	32470001	32520000	0.0274509	CEP170
12	8090001	8140000	0.027439	FGF14
14	4030001	4080000	0.0274369	RALYL
12	63190001	63240000	0.0274348	FREM2
3	7040001	7090000	0.0274224	SPP2
12	13090001	13140000	0.0273603	UGGT2
10	49840001	49890000	0.0273601	TCF12
12	50390001	50440000	0.027352	LOC108637296
12	50390001	50440000	0.027352	MPHOSPH8
12	50390001	50440000	0.027352	PSPC1
1	52130001	52180000	0.0273447	BBX
8	39220001	39270000	0.0273037	INSL6
8	39220001	39270000	0.0273037	JAK2
8	12840001	12890000	0.0272703	TRNAW-CCA-44
16	49820001	49870000	0.0272701	MIR429
16	49820001	49870000	0.0272701	MIR200A
16	49820001	49870000	0.0272701	MIR200B
16	49820001	49870000	0.0272701	TTLL10
10	97110001	97160000	0.0272676	CCDC112
10	97110001	97160000	0.0272676	PGGT1B
1	118580001	118630000	0.0272301	CP
1	118580001	118630000	0.0272301	HPS3
5	22540001	22590000	0.0272067	EEA1
2	14310001	14360000	0.0271891	KPNA6
24	42750001	42800000	0.0271284	PIEZO2
6	108940001	108990000	0.0271238	RAB28
16	32480001	32530000	0.0271186	CEP170
12	13180001	13230000	0.0271033	DNAJC3
2	111010001	111060000	0.027058	TLK1
14	13090001	13140000	0.0270182	C14H8orf37
15	39580001	39630000	0.0270098	SBF2
4	70530001	70580000	0.0269907	EPDR1
4	70530001	70580000	0.0269907	SFRP4
10	66230001	66280000	0.0269829	NDUFAF1
10	66230001	66280000	0.0269829	NUSAP1
10	66230001	66280000	0.0269829	OIP5
19	18990001	19040000	0.0269776	LOC106503912
19	18990001	19040000	0.0269776	LOC108638256
19	18990001	19040000	0.0269776	LOC102189615
15	60390001	60440000	0.0269656	SIK2
19	62370001	62420000	0.0269436	HELZ
9	1910001	1960000	0.0269424	COL12A1
19	27460001	27510000	0.0269224	TRNAK-UUU-24

19	27460001	27510000	0.0269224	TRNAQ-CUG-12
19	27460001	27510000	0.0269224	TRNAL-UAG-3
19	27460001	27510000	0.0269224	HES7
19	27460001	27510000	0.0269224	TRNAR-UCU-8
19	27460001	27510000	0.0269224	TRNAG-GCC-17
19	27460001	27510000	0.0269224	ALOX12B
19	27460001	27510000	0.0269224	ALOXE3
19	27460001	27510000	0.0269224	LOC108638269
29	32030001	32080000	0.0269084	LOC102170585
29	32030001	32080000	0.0269084	LOC108634249
12	63220001	63270000	0.0268981	FREM2
12	8100001	8150000	0.0268768	FGF14
14	91230001	91280000	0.026875	FAM135A
24	43440001	43490000	0.026864	PTPN2
1	152070001	152120000	0.0268496	CAPN7
6	116700001	116750000	0.0268399	MFSD10
6	116700001	116750000	0.0268399	LOC108636244
6	116700001	116750000	0.0268399	NOP14
6	116700001	116750000	0.0268399	ADD1
8	39140001	39190000	0.0268278	PLGRKT
19	23190001	23240000	0.0268241	METTL16
12	10280001	10330000	0.026809	DOCK9
5	81950001	82000000	0.0268054	ITPR2
6	114830001	114880000	0.0267815	ACOX3
19	27430001	27480000	0.0267806	LOC102169702
19	27430001	27480000	0.0267806	LOC102172658
19	27430001	27480000	0.0267806	LOC102172190
19	27430001	27480000	0.0267806	ALOX12B
19	27430001	27480000	0.0267806	ALOXE3
4	100880001	100930000	0.0267735	THSD7A
22	11430001	11480000	0.0267707	ACAA1
22	11430001	11480000	0.0267707	DLEC1
26	35730001	35780000	0.0267671	TRNAE-UUC-97
26	35730001	35780000	0.0267671	NOC3L
26	35730001	35780000	0.0267671	PLCE1
12	55200001	55250000	0.0267422	LOC102188230
12	55200001	55250000	0.0267422	POMP
26	15100001	15150000	0.0267406	PNLIPRP3
19	62400001	62450000	0.0267176	HELZ
19	62380001	62430000	0.0266733	HELZ
1	101790001	101840000	0.0266583	SI
16	49830001	49880000	0.0266507	C16H1orf159
19	23200001	23250000	0.026633	LOC108638230
19	23200001	23250000	0.026633	METTL16
14	91210001	91260000	0.0266324	FAM135A
6	114790001	114840000	0.0266056	ACOX3
5	25650001	25700000	0.0265875	LOC106502088
13	69730001	69780000	0.0265835	CHD6
12	16360001	16410000	0.0265784	ABCC4
4	1570001	1620000	0.0265762	LMBR1
1	51290001	51340000	0.026549	LOC108636831
4	100840001	100890000	0.0265284	THSD7A
4	34700001	34750000	0.0265055	ING3
16	45060001	45110000	0.0264852	CAMTA1
4	94650001	94700000	0.0264639	AHR
2	23770001	23820000	0.0264466	AP1S3
22	10230001	10280000	0.026417	LRRFIP2
14	91160001	91210000	0.0264139	FAM135A

15	8740001	8790000	0.0264021	EXT2
5	22520001	22570000	0.026388	EEA1
4	94660001	94710000	0.0263812	TRNAW-CCA-22
4	94660001	94710000	0.0263812	AHR
19	23180001	23230000	0.0263703	METTL16
14	91220001	91270000	0.0263586	FAM135A
19	49170001	49220000	0.0263313	METRNL
29	45680001	45730000	0.0263296	POLR2L
29	45680001	45730000	0.0263296	CD151
29	45680001	45730000	0.0263296	PNPLA2
29	45680001	45730000	0.0263296	RPLP2
29	45680001	45730000	0.0263296	PIDD1
29	45680001	45730000	0.0263296	SLC25A22
29	45680001	45730000	0.0263296	PPP1CA
29	45680001	45730000	0.0263296	CRACR2B
29	45680001	45730000	0.0263296	CEND1
13	69710001	69760000	0.0263186	CHD6
6	114390001	114440000	0.0263105	AFAP1
11	65500001	65550000	0.0262966	ETAA1
14	12090001	12140000	0.026291	CDH17
5	31320001	31370000	0.0262905	LOC108636097
10	49930001	49980000	0.0262893	TCF12
6	114380001	114430000	0.0262883	AFAP1
29	32020001	32070000	0.026273	LOC102170585
29	32020001	32070000	0.026273	LOC108634249
4	92830001	92880000	0.0262674	HDAC9
13	7370001	7420000	0.0262495	MACROD2
12	63230001	63280000	0.0261889	FREM2
8	10770001	10820000	0.0261857	PBK
8	10770001	10820000	0.0261857	SCARA5
25	9920001	9970000	0.0261747	LOC102173909
4	115130001	115180000	0.0261129	DDC
4	115130001	115180000	0.0261129	GRB10
12	10170001	10220000	0.026104	GPR18
12	10170001	10220000	0.026104	UBAC2
14	12890001	12940000	0.0260912	NDUFAF6
19	23060001	23110000	0.0260849	TSR1
19	23060001	23110000	0.0260849	SRR
19	23060001	23110000	0.0260849	SGSM2
19	23060001	23110000	0.0260849	SMG6
1	52060001	52110000	0.0260717	BBX
25	9840001	9890000	0.0260636	RMI2
6	117610001	117660000	0.0260608	PIGG
29	31800001	31850000	0.0260559	ARHGAP32
4	87000001	87050000	0.0260479	KIAA1324L
9	74610001	74660000	0.026046	PPP1R14C
24	42480001	42530000	0.0260386	APCDD1
22	19040001	19090000	0.0260348	GRM7
2	115530001	115580000	0.0260298	HOXD9
2	115530001	115580000	0.0260298	HOXD8
2	115530001	115580000	0.0260298	LOC108638283
2	115530001	115580000	0.0260298	MIR10B
2	115530001	115580000	0.0260298	HOXD3
26	15060001	15110000	0.0260279	PNLIPRP3
8	10790001	10840000	0.0260242	PBK
16	69470001	69520000	0.0260237	BATF3
2	23780001	23830000	0.026017	AP1S3
13	69720001	69770000	0.0260074	CHD6

2	23790001	23840000	0.0260022	AP1S3
7	90060001	90110000	0.0260009	NFIC
14	12880001	12930000	0.0260004	NDUFAF6
10	97130001	97180000	0.0259546	PGGT1B
21	5230001	5280000	0.0259305	LINS1
21	5230001	5280000	0.0259305	ASB7
8	37800001	37850000	0.0259269	KDM4C
15	8710001	8760000	0.0259205	EXT2
28	17390001	17440000	0.0259105	ASCC1
4	70490001	70540000	0.0258969	EPDR1
29	41300001	41350000	0.0258828	INTS5
29	41300001	41350000	0.0258828	LBHD1
29	41300001	41350000	0.0258828	METTL12
29	41300001	41350000	0.0258828	LOC106503742
29	41300001	41350000	0.0258828	LRRN4CL
29	41300001	41350000	0.0258828	GANAB
29	41300001	41350000	0.0258828	UBXN1
29	41300001	41350000	0.0258828	BSCL2
1	118520001	118570000	0.025881	CP
1	118520001	118570000	0.025881	LOC102172204
6	70080001	70130000	0.0258768	CHIC2
4	94640001	94690000	0.0258638	AHR
29	32040001	32090000	0.025861	LOC102170585
29	32040001	32090000	0.025861	LOC108634249
4	96640001	96690000	0.0258434	AGMO
7	67640001	67690000	0.0258389	LOC102168822
7	67640001	67690000	0.0258389	LOC102168535
7	41420001	41470000	0.025835	NIPAL4
7	41420001	41470000	0.025835	ADAM19
16	78870001	78920000	0.0258344	ARL8A
16	78870001	78920000	0.0258344	LOC102168445
16	78870001	78920000	0.0258344	PTPN7
8	39570001	39620000	0.0258098	RCL1
2	14290001	14340000	0.025809	KPNA6
2	14290001	14340000	0.025809	TMEM39B
3	119680001	119730000	0.0258063	TIPRL
4	96690001	96740000	0.0258023	AGMO
8	37790001	37840000	0.0257858	KDM4C
12	50040001	50090000	0.0257734	ATP12A
22	16760001	16810000	0.0257707	TADA3
22	16760001	16810000	0.0257707	OGG1
22	16760001	16810000	0.0257707	BRPF1
22	16760001	16810000	0.0257707	CPNE9
22	16760001	16810000	0.0257707	CAMK1
15	39570001	39620000	0.0257637	SBF2
4	86940001	86990000	0.0257448	KIAA1324L
24	42460001	42510000	0.0257201	APCDD1
1	112940001	112990000	0.0257185	TRNAE-UUC-2
1	112940001	112990000	0.0257185	GPR149
1	112940001	112990000	0.0257185	DHX36
7	67650001	67700000	0.0257085	LOC102168822
7	67650001	67700000	0.0257085	LOC102168535
2	14300001	14350000	0.0257003	KPNA6
12	10160001	10210000	0.0256927	GPR18
12	10160001	10210000	0.0256927	UBAC2
5	68990001	69040000	0.0256556	CRY1
14	91260001	91310000	0.0256537	SDHAF4
14	91260001	91310000	0.0256537	FAM135A

14	4040001	4090000	0.0256466	RALYL
8	39300001	39350000	0.0256411	JAK2
11	61150001	61200000	0.0256269	EHBP1
5	68970001	69020000	0.0256043	CRY1
26	45940001	45990000	0.025603	PCDH15
7	58320001	58370000	0.0255897	PCDHB1
14	91240001	91290000	0.025577	FAM135A
22	17180001	17230000	0.025552	SRGAP3
3	9180001	9230000	0.0255237	C3H1orf94
5	68980001	69030000	0.0255205	CRY1
12	13080001	13130000	0.0254916	UGGT2
5	68720001	68770000	0.0254753	RFX4
5	68720001	68770000	0.0254753	RIC8B
15	5610001	5660000	0.0254726	PTPMT1
15	5610001	5660000	0.0254726	CELF1
11	72670001	72720000	0.0254683	CIB4
11	72670001	72720000	0.0254683	C11H2orf70
26	45950001	46000000	0.0254634	PCDH15
12	33880001	33930000	0.0254619	FBXL3
12	33880001	33930000	0.0254619	CLN5
12	33880001	33930000	0.0254619	MYCBP2
26	28160001	28210000	0.0254612	AS3MT
26	28160001	28210000	0.0254612	CNNM2
10	66150001	66200000	0.0254396	TRNAW-CCA-59
10	66150001	66200000	0.0254396	RTF1
14	91250001	91300000	0.0254389	SDHAF4
14	91250001	91300000	0.0254389	FAM135A
15	39600001	39650000	0.0254087	SBF2
8	10760001	10810000	0.0254011	PBK
8	10760001	10810000	0.0254011	SCARA5
24	42980001	43030000	0.0253936	GNAL
19	62390001	62440000	0.025388	HELZ
12	50430001	50480000	0.0253715	PSPC1
1	51670001	51720000	0.0253672	LOC102171918
29	31840001	31890000	0.0253647	ARHGAP32
22	17150001	17200000	0.0253306	SRGAP3
5	68730001	68780000	0.0253272	RFX4
5	68730001	68780000	0.0253272	RIC8B
14	46160001	46210000	0.0253122	KCNB2
4	34690001	34740000	0.0252937	ING3
24	42990001	43040000	0.025261	GNAL
19	23170001	23220000	0.0252609	METTL16
15	60380001	60430000	0.0252562	SIK2
12	50460001	50510000	0.0252093	ZMYM5
12	50460001	50510000	0.0252093	PSPC1
10	49790001	49840000	0.0251799	TCF12
10	75910001	75960000	0.0251775	LOC102183694
10	75910001	75960000	0.0251775	LOC102176964
10	75910001	75960000	0.0251775	LOC108636865
1	1390001	1440000	0.0251665	C1H21orf59
1	1390001	1440000	0.0251665	EVA1C
6	111450001	111500000	0.0251517	PROM1
7	90070001	90120000	0.0251498	NFIC
12	50440001	50490000	0.0251409	PSPC1
14	12080001	12130000	0.0251242	CDH17
1	65140001	65190000	0.025102	GTF2E1
24	43460001	43510000	0.0250979	PTPN2
12	33890001	33940000	0.0250966	FBXL3

12	33890001	33940000	0.0250966	CLN5
1	101700001	101750000	0.0250782	SLITRK3
16	45130001	45180000	0.0250645	CAMTA1
1	112950001	113000000	0.0250498	GPR149
1	112950001	113000000	0.0250498	DHX36
3	12680001	12730000	0.0250111	MANEAL
3	12680001	12730000	0.0250111	YRDC
3	12680001	12730000	0.0250111	MTF1
5	81960001	82010000	0.0250054	ITPR2
16	28110001	28160000	0.0249832	STUM
8	39560001	39610000	0.0249805	RCL1
22	17130001	17180000	0.0249471	SRGAP3
8	39580001	39630000	0.0249352	RCL1
22	16710001	16760000	0.0249312	TADA3
22	16710001	16760000	0.0249312	RPUSD3
22	16710001	16760000	0.0249312	LOC102184009
22	16710001	16760000	0.0249312	ARPC4
15	39560001	39610000	0.0248867	SBF2
19	62410001	62460000	0.0248859	HELZ
12	50470001	50520000	0.0248818	ZMYM5
12	50470001	50520000	0.0248818	PSPC1
1	96780001	96830000	0.0248764	LOC102179814
1	96780001	96830000	0.0248764	PHC3
1	96780001	96830000	0.0248764	GPR160
29	45670001	45720000	0.0248691	RAD9A
29	45670001	45720000	0.0248691	POLR2L
29	45670001	45720000	0.0248691	CD151
29	45670001	45720000	0.0248691	PNPLA2
29	45670001	45720000	0.0248691	RPLP2
29	45670001	45720000	0.0248691	PIDD1
29	45670001	45720000	0.0248691	SLC25A22
29	45670001	45720000	0.0248691	PPP1CA
29	45670001	45720000	0.0248691	CRACR2B
24	43000001	43050000	0.0248575	GNAL
1	51280001	51330000	0.024848	LOC108636831
4	34710001	34760000	0.0248466	ING3
13	7380001	7430000	0.0248335	MACROD2
8	22110001	22160000	0.0248324	CDKN2B
8	22110001	22160000	0.0248324	LOC102173187
8	22110001	22160000	0.0248324	LOC108636541
12	10320001	10370000	0.0248138	TRNAS-GGA-68
12	10320001	10370000	0.0248138	DOCK9
4	1550001	1600000	0.0248099	LMBR1
8	39680001	39730000	0.0248096	PLPP6
8	39680001	39730000	0.0248096	LOC108636555
6	116570001	116620000	0.0247916	TNIP2
19	27840001	27890000	0.0247877	MYH10
6	114840001	114890000	0.0247779	TRMT44
6	114840001	114890000	0.0247779	ACOX3
5	69000001	69050000	0.0247723	CRY1
10	49780001	49830000	0.0247584	TCF12
10	66220001	66270000	0.0247471	LOC102182981
10	66220001	66270000	0.0247471	NDUFAF1
10	66220001	66270000	0.0247471	NUSAP1
3	7050001	7100000	0.024745	SPP2
10	75950001	76000000	0.024741	LOC108636865
10	75950001	76000000	0.024741	LOC102177429
10	75950001	76000000	0.024741	LOC102177709

1	152090001	152140000	0.024734	CAPN7
1	152090001	152140000	0.024734	SH3BP5
6	108840001	108890000	0.0247151	RAB28
4	97700001	97750000	0.024714	DGKB
2	6620001	6670000	0.024707	E2F2
2	6620001	6670000	0.024707	ID3
8	39150001	39200000	0.024682	PLGRKT
1	96710001	96760000	0.024675	PHC3
12	8350001	8400000	0.0246662	ITGBL1
28	10190001	10240000	0.0246421	LOC102175466
28	10190001	10240000	0.0246421	SFTPD
28	10190001	10240000	0.0246421	LOC102175746
22	42310001	42360000	0.0246324	C22H3orf67
18	39730001	39780000	0.0246278	LOC108637979
15	39750001	39800000	0.0246068	SBF2
19	27470001	27520000	0.0246011	TRNAK-UUU-24
19	27470001	27520000	0.0246011	TRNAQ-CUG-12
19	27470001	27520000	0.0246011	TRNAL-UAG-3
19	27470001	27520000	0.0246011	HES7
19	27470001	27520000	0.0246011	TRNAR-UCU-8
19	27470001	27520000	0.0246011	TRNAG-GCC-17
19	27470001	27520000	0.0246011	TRNAS-CGA-2
19	27470001	27520000	0.0246011	TRNAT-AGU-3
19	27470001	27520000	0.0246011	ALOXE3
19	27470001	27520000	0.0246011	LOC108638269
19	27470001	27520000	0.0246011	PER1
5	33000001	33050000	0.0245955	SLC38A4
7	67630001	67680000	0.0245859	LOC102168822
15	39760001	39810000	0.0245841	SBF2
7	60580001	60630000	0.0245762	HSPA9
7	60580001	60630000	0.0245762	ETF1
7	66930001	66980000	0.0245712	TMEM259
7	66930001	66980000	0.0245712	ABCA7
7	66930001	66980000	0.0245712	CNN2
7	66930001	66980000	0.0245712	GRIN3B
7	66930001	66980000	0.0245712	WDR18
22	17120001	17170000	0.0245612	SRGAP3
12	37450001	37500000	0.0245465	KLF12
28	15100001	15150000	0.0245407	ADK
19	27380001	27430000	0.0245164	GUCY2D
19	62190001	62240000	0.0245012	CACNG4
1	45220001	45270000	0.0244905	ABI3BP
16	78880001	78930000	0.0244879	ARL8A
16	78880001	78930000	0.0244879	LOC102168445
16	78880001	78930000	0.0244879	GPR37L1
16	78880001	78930000	0.0244879	PTPN7
3	119710001	119760000	0.0244501	TIPRL
11	61130001	61180000	0.0244432	EHBP1
12	10180001	10230000	0.024439	GPR18
12	10180001	10230000	0.024439	UBAC2
15	8140001	8190000	0.0244237	TSPAN18
21	5240001	5290000	0.0244223	LINS1
21	5240001	5290000	0.0244223	ASB7
1	53190001	53240000	0.0244112	KIAA1524
1	53190001	53240000	0.0244112	DZIP3
22	51350001	51400000	0.0244074	UCN2
22	51350001	51400000	0.0244074	PFKFB4
22	51350001	51400000	0.0244074	COL7A1

4	92820001	92870000	0.0243989	HDAC9
12	33870001	33920000	0.0243892	FBXL3
12	33870001	33920000	0.0243892	MYCBP2
22	42300001	42350000	0.0243847	C22H3orf67
5	68700001	68750000	0.0243818	RFX4
3	7010001	7060000	0.0243786	SPP2
5	81940001	81990000	0.0243747	ITPR2
20	9110001	9160000	0.0243427	ZNF366
26	35740001	35790000	0.0243175	TRNAE-UUC-97
26	35740001	35790000	0.0243175	NOC3L
26	35740001	35790000	0.0243175	PLCE1
14	46200001	46250000	0.024303	LOC108637480
14	46200001	46250000	0.024303	KCNB2
1	65100001	65150000	0.0242947	RABL3
1	65100001	65150000	0.0242947	GTF2E1
10	49800001	49850000	0.0242811	TCF12
15	39740001	39790000	0.0242767	SBF2
24	14120001	14170000	0.0242746	PIK3C3
15	5600001	5650000	0.0242716	KBTBD4
15	5600001	5650000	0.0242716	PTPMT1
15	5600001	5650000	0.0242716	NDUFS3
15	5600001	5650000	0.0242716	CELF1
8	38600001	38650000	0.0242686	MLANA
8	38600001	38650000	0.0242686	KIAA2026
8	12860001	12910000	0.0242683	TRNAW-CCA-44
29	31850001	31900000	0.024242	ARHGAP32
8	12870001	12920000	0.0242226	TRNAW-CCA-44
14	91410001	91460000	0.0242185	SMAP1
16	49840001	49890000	0.0242143	C16H1orf159
1	53180001	53230000	0.0242028	KIAA1524
1	53180001	53230000	0.0242028	DZIP3
12	10200001	10250000	0.0241909	UBAC2
6	108990001	109040000	0.0241856	NKX3-2
16	49800001	49850000	0.0241695	MIR429
16	49800001	49850000	0.0241695	MIR200A
16	49800001	49850000	0.0241695	MIR200B
16	49800001	49850000	0.0241695	TTLL10
4	87010001	87060000	0.0241497	KIAA1324L
2	111000001	111050000	0.0241417	TLK1
18	39720001	39770000	0.0241357	LOC108637979
8	39070001	39120000	0.0241016	CD274
8	39070001	39120000	0.0241016	PLGRKT
8	10750001	10800000	0.0240959	SCARA5
19	61870001	61920000	0.0240749	PRKCA
24	43430001	43480000	0.0240716	PTPN2
8	39590001	39640000	0.0240698	AK3
13	7350001	7400000	0.0240617	MACROD2
7	90040001	90090000	0.0240551	NFIC
5	25660001	25710000	0.0240522	LOC106502088
10	65490001	65540000	0.0240293	VPS39
10	65490001	65540000	0.0240293	PLA2G4F
6	117550001	117600000	0.0240152	LOC106502244
6	117550001	117600000	0.0240152	PIGG
3	12670001	12720000	0.0240104	MANEAL
3	12670001	12720000	0.0240104	YRDC
3	12670001	12720000	0.0240104	MTF1
1	51330001	51380000	0.0240024	LOC108636831
20	9120001	9170000	0.0239973	ZNF366

3	13630001	13680000	0.0239879	LOC106501986
3	13630001	13680000	0.0239879	RRAGC
3	13630001	13680000	0.0239879	MYCBP
19	27390001	27440000	0.0239849	LOC102169702
19	27390001	27440000	0.0239849	GUCY2D
19	27780001	27830000	0.0239751	NDEL1
19	27780001	27830000	0.0239751	MYH10
7	64210001	64260000	0.023962	DDX46
19	27420001	27470000	0.0239503	LOC102169702
19	27420001	27470000	0.0239503	LOC102172658
19	27420001	27470000	0.0239503	LOC102172190
19	27420001	27470000	0.0239503	ALOX12B
12	33860001	33910000	0.0239476	FBXL3
12	33860001	33910000	0.0239476	MYCBP2
5	33010001	33060000	0.0239467	SLC38A4
3	9890001	9940000	0.0239304	GJB5
3	9890001	9940000	0.0239304	GJB3
3	9890001	9940000	0.0239304	GJA4
3	9890001	9940000	0.0239304	GJB4
2	112260001	112310000	0.0239183	PDK1
10	49810001	49860000	0.0239133	TCF12
12	50080001	50130000	0.0239076	ATP12A
12	50080001	50130000	0.0239076	RNF17
12	10190001	10240000	0.0239054	UBAC2
24	43660001	43710000	0.0238967	LDLRAD4
7	41430001	41480000	0.0238915	NIPAL4
7	41430001	41480000	0.0238915	ADAM19
19	23160001	23210000	0.0238784	MNT
19	23160001	23210000	0.0238784	METTL16
29	41320001	41370000	0.0238649	LBHD1
29	41320001	41370000	0.0238649	METTL12
29	41320001	41370000	0.0238649	LOC106503742
29	41320001	41370000	0.0238649	LRRN4CL
29	41320001	41370000	0.0238649	GNG3
29	41320001	41370000	0.0238649	UBXN1
29	41320001	41370000	0.0238649	BSCL2
29	41320001	41370000	0.0238649	HNRNPUL2
13	7330001	7380000	0.0238567	SEL1L2
13	7330001	7380000	0.0238567	MACROD2
1	112960001	113010000	0.023849	DHX36
29	41310001	41360000	0.0238378	INTS5
29	41310001	41360000	0.0238378	LBHD1
29	41310001	41360000	0.0238378	METTL12
29	41310001	41360000	0.0238378	LOC106503742
29	41310001	41360000	0.0238378	LRRN4CL
29	41310001	41360000	0.0238378	GNG3
29	41310001	41360000	0.0238378	GANAB
29	41310001	41360000	0.0238378	UBXN1
29	41310001	41360000	0.0238378	BSCL2
9	74600001	74650000	0.0238323	PPP1R14C
24	14130001	14180000	0.0238302	PIK3C3
24	14140001	14190000	0.0238286	PIK3C3
1	51270001	51320000	0.023826	LOC108636831
5	33470001	33520000	0.0238175	SLC38A1
10	97140001	97190000	0.0238156	PGGT1B
12	10270001	10320000	0.023797	DOCK9
7	60590001	60640000	0.0237904	HSPA9
7	60590001	60640000	0.0237904	ETF1

1	152080001	152130000	0.0237837	CAPN7
1	152080001	152130000	0.0237837	SH3BP5
10	97100001	97150000	0.0237774	CCDC112
10	97100001	97150000	0.0237774	PGGT1B
12	50450001	50500000	0.0237757	ZMYM5
12	50450001	50500000	0.0237757	PSPC1
19	23050001	23100000	0.0237737	TSR1
19	23050001	23100000	0.0237737	SRR
19	23050001	23100000	0.0237737	SMG6
13	8980001	9030000	0.0237713	MACROD2
4	112200001	112250000	0.0237696	CDK14
10	49820001	49870000	0.0237692	TCF12
18	23350001	23400000	0.023766	RPGRIP1L
26	28130001	28180000	0.0237556	CNNM2
11	72660001	72710000	0.0237498	CIB4
11	72660001	72710000	0.0237498	C11H2orf70
4	97710001	97760000	0.023748	DGKB
26	35710001	35760000	0.0237182	TRNAE-UUC-97
26	35710001	35760000	0.0237182	NOC3L
26	35710001	35760000	0.0237182	PLCE1
13	65310001	65360000	0.023696	NDRG3
24	14110001	14160000	0.0236949	PIK3C3
8	37780001	37830000	0.0236924	KDM4C
24	43010001	43060000	0.0236831	GNAL
12	28390001	28440000	0.023666	TRNAC-GCA-166
1	146020001	146070000	0.0236579	PCNT
14	12070001	12120000	0.0236579	CDH17
26	35720001	35770000	0.0236484	TRNAE-UUC-97
26	35720001	35770000	0.0236484	NOC3L
26	35720001	35770000	0.0236484	PLCE1
18	12250001	12300000	0.0236293	USP10
9	80990001	81040000	0.0236247	ARID1B
4	7690001	7740000	0.0236171	LOC106501766
4	7690001	7740000	0.0236171	LOC102168236
14	91270001	91320000	0.02361	SDHAF4
14	91270001	91320000	0.02361	FAM135A
8	12880001	12930000	0.0236067	TRNAW-CCA-44
9	81000001	81050000	0.0235958	ARID1B
5	86880001	86930000	0.023594	ABCC9
18	39740001	39790000	0.0235821	LOC108637979
4	7760001	7810000	0.0235813	GIMAP8
22	17140001	17190000	0.0235796	SRGAP3
22	16060001	16110000	0.0235717	TOPAZ1
22	16060001	16110000	0.0235717	TCAIM
2	110320001	110370000	0.0235548	MYO3B
5	26770001	26820000	0.0235505	KRT8
5	26770001	26820000	0.0235505	LOC108635996
5	26770001	26820000	0.0235505	KRT78
6	110460001	110510000	0.0235439	CPEB2
29	31110001	31160000	0.0235424	ETS1
7	92940001	92990000	0.023539	ADGRE1
22	16050001	16100000	0.0235372	TOPAZ1
22	16050001	16100000	0.0235372	TCAIM
2	111670001	111720000	0.0235293	HAT1
13	7390001	7440000	0.0235263	MACROD2
13	69740001	69790000	0.0235196	CHD6
18	64900001	64950000	0.0235153	LOC102168291
18	64900001	64950000	0.0235153	LOC102171271

18	64900001	64950000	0.0235153	LOC102191644
18	64900001	64950000	0.0235153	LOC102171004
18	64900001	64950000	0.0235153	LOC108638097
19	23070001	23120000	0.02351	TSR1
19	23070001	23120000	0.02351	TRNAG-CCC-91
19	23070001	23120000	0.02351	SRR
19	23070001	23120000	0.02351	SGSM2
19	23070001	23120000	0.02351	SMG6
24	42490001	42540000	0.0235043	NAPG
24	42490001	42540000	0.0235043	APCDD1
24	42490001	42540000	0.0235043	LOC106503540
8	370001	420000	0.0235007	ANXA10
16	45050001	45100000	0.0234998	CAMTA1
19	49180001	49230000	0.0234866	METRNL
19	49180001	49230000	0.0234866	B3GNTL1
22	12340001	12390000	0.0234805	LOC108638642
22	12340001	12390000	0.0234805	XIRP1
22	12340001	12390000	0.0234805	CSRNP1
12	50070001	50120000	0.0234796	ATP12A
12	50070001	50120000	0.0234796	RNF17
15	4360001	4410000	0.0234699	LOC108637624
15	4360001	4410000	0.0234699	LOC108637625
15	4360001	4410000	0.0234699	LOC102183417
15	4360001	4410000	0.0234699	LOC102190601
12	50480001	50530000	0.0234652	ZMYM5
12	50480001	50530000	0.0234652	PSPC1
6	103980001	104030000	0.0234609	EVC2
8	360001	410000	0.02345	ANXA10
7	83740001	83790000	0.0234475	C7H5orf63
7	83700001	83750000	0.0234417	3-Mar
7	83700001	83750000	0.0234417	C7H5orf63
26	45930001	45980000	0.0234151	PCDH15
6	72820001	72870000	0.0234083	LOC102180384
6	72820001	72870000	0.0234083	LOC102183584
14	12870001	12920000	0.0234026	NDUFAF6
14	12870001	12920000	0.0234026	LOC102184166
5	69010001	69060000	0.0233978	CRY1
18	23360001	23410000	0.0233801	RPGRIP1L
7	90050001	90100000	0.0233784	NFIC
10	49770001	49820000	0.0233619	LOC108636854
10	49770001	49820000	0.0233619	TCF12
19	34270001	34320000	0.02336	MIR33B
19	34270001	34320000	0.02336	SREBF1
19	34270001	34320000	0.02336	RAI1
29	27000001	27050000	0.0233527	LOC102178703
29	27000001	27050000	0.0233527	LOC102179269
18	15870001	15920000	0.0233497	SPG7
18	15870001	15920000	0.0233497	RPL13
18	15870001	15920000	0.0233497	CPNE7
13	2290001	2340000	0.0233457	PAK5
2	97300001	97350000	0.0233368	ACVR1C
6	109000001	109050000	0.0233259	NKX3-2
1	101710001	101760000	0.0233192	SLITRK3
10	49830001	49880000	0.0233142	TCF12
15	5620001	5670000	0.0232999	CELF1
7	93120001	93170000	0.0232978	LOC102175946
9	83460001	83510000	0.0232902	IGF2R
18	14700001	14750000	0.0232872	SLC7A5

18	14700001	14750000	0.0232872	CA5A
9	20460001	20510000	0.0232426	DCBLD1
2	110330001	110380000	0.0232392	MYO3B
6	110410001	110460000	0.023234	TRNAW-CCA-37
6	110410001	110460000	0.023234	CPEB2
15	39730001	39780000	0.0232321	SBF2
21	6070001	6120000	0.0232226	MEF2A
15	8750001	8800000	0.0232179	EXT2
13	7340001	7390000	0.0232045	SEL1L2
13	7340001	7390000	0.0232045	MACROD2
15	8350001	8400000	0.0232033	CD82
9	32470001	32520000	0.0232011	HACE1
10	75690001	75740000	0.0231837	LOC102184528
10	75690001	75740000	0.0231837	LOC102180319
10	75690001	75740000	0.0231837	LOC102180047
10	75690001	75740000	0.0231837	LOC102179772
4	1540001	1590000	0.0231821	LMBR1
7	90080001	90130000	0.0231735	SMIM24
7	90080001	90130000	0.0231735	NFIC
11	20250001	20300000	0.0231616	RMDN2
2	97310001	97360000	0.0231598	ACVR1C
20	62110001	62160000	0.0231585	CTNND2
21	390001	440000	0.023155	MAGEL2
16	71710001	71760000	0.023153	LOC108637792
16	71710001	71760000	0.023153	SERTAD4
16	71710001	71760000	0.023153	LOC108637749
10	66500001	66550000	0.0231295	INO80
29	32010001	32060000	0.0231292	LOC102170585
29	32010001	32060000	0.0231292	BARX2
14	91420001	91470000	0.0231246	SMAP1
8	12850001	12900000	0.0231138	TRNAW-CCA-44
2	14320001	14370000	0.0231118	KPNA6
2	14320001	14370000	0.0231118	TXLNA
2	110340001	110390000	0.0231102	MYO3B
11	61210001	61260000	0.0231088	OTX1
11	61210001	61260000	0.0231088	EHBP1
2	114000001	114050000	0.023106	WIPF1
22	41820001	41870000	0.0230978	LOC106503441
16	32460001	32510000	0.0230777	CEP170
20	62100001	62150000	0.0230727	CTNND2
18	39710001	39760000	0.0230685	LOC108637979
29	31960001	32010000	0.0230554	BARX2
22	19050001	19100000	0.0230414	GRM7
5	33480001	33530000	0.0230375	SLC38A1
22	17340001	17390000	0.0230284	SRGAP3
1	52180001	52230000	0.0230152	BBX
7	71020001	71070000	0.0230142	COL23A1
8	38780001	38830000	0.0230047	RIC1
2	14280001	14330000	0.0230043	KPNA6
2	14280001	14330000	0.0230043	TMEM39B
25	22590001	22640000	0.0230039	ARHGAP17
25	22590001	22640000	0.0230039	LCMT1
7	71050001	71100000	0.0230008	COL23A1
22	17200001	17250000	0.0229953	SRGAP3
7	45300001	45350000	0.0229933	GRIA1
18	63110001	63160000	0.0229915	LOC102174057
18	63110001	63160000	0.0229915	LOC102173463
1	111250001	111300000	0.0229894	GMPS

4	1590001	1640000	0.0229843	LMBR1
10	75680001	75730000	0.0229659	LOC102180597
10	75680001	75730000	0.0229659	LOC102184528
10	75680001	75730000	0.0229659	LOC102180319
10	75680001	75730000	0.0229659	LOC102180047
10	75680001	75730000	0.0229659	LOC102179772
6	111460001	111510000	0.0229646	PROM1
8	39690001	39740000	0.0229614	PLPP6
8	39690001	39740000	0.0229614	SPATA6L
8	39690001	39740000	0.0229614	LOC108636555
7	64200001	64250000	0.0229483	DDX46
1	53170001	53220000	0.0229328	KIAA1524
1	53170001	53220000	0.0229328	DZIP3
3	12690001	12740000	0.0229288	MANEAL
3	12690001	12740000	0.0229288	YRDC
3	12690001	12740000	0.0229288	MTF1
19	27410001	27460000	0.0229257	LOC102169702
19	27410001	27460000	0.0229257	LOC102172658
19	27410001	27460000	0.0229257	LOC102172190
19	27410001	27460000	0.0229257	GUCY2D
19	27410001	27460000	0.0229257	ALOX12B
15	39610001	39660000	0.0229237	SBF2
19	27400001	27450000	0.0229204	LOC102169702
19	27400001	27450000	0.0229204	LOC102172658
19	27400001	27450000	0.0229204	GUCY2D
6	116730001	116780000	0.0229167	MFSD10
6	116730001	116780000	0.0229167	LOC108636244
6	116730001	116780000	0.0229167	NOP14
6	116730001	116780000	0.0229167	ADD1
6	116730001	116780000	0.0229167	GRK4
19	31500001	31550000	0.0229043	HS3ST3A1
11	93690001	93740000	0.0228938	GPR21
11	93690001	93740000	0.0228938	RABGAP1
18	23370001	23420000	0.0228926	RPGRIP1L
18	23370001	23420000	0.0228926	FTO
16	69460001	69510000	0.0228881	BATF3
26	45960001	46010000	0.0228859	PCDH15
8	82300001	82350000	0.0228858	ERCC6L2
15	39770001	39820000	0.0228823	SBF2
6	106870001	106920000	0.0228807	TRNAC-GCA-105
4	90110001	90160000	0.0228785	DNAH11
22	10190001	10240000	0.0228686	LRRFIP2
15	39690001	39740000	0.0228643	SBF2
9	32480001	32530000	0.0228629	HACE1
29	32050001	32100000	0.0228522	LOC102170585
29	32050001	32100000	0.0228522	LOC108634249
7	67620001	67670000	0.0228509	LOC102168822
5	22510001	22560000	0.022819	EEA1
2	36070001	36120000	0.0228111	ERBB4
6	116860001	116910000	0.0228032	HTT
19	61840001	61890000	0.0227941	PRKCA
26	18670001	18720000	0.0227769	VTI1A
25	25950001	26000000	0.022749	IL27
25	25950001	26000000	0.022749	NUPR1
25	25950001	26000000	0.022749	APOBR
25	25950001	26000000	0.022749	CLN3
10	50130001	50180000	0.022744	TCF12
6	114780001	114830000	0.0227304	HTRA3

6	114780001	114830000	0.0227304	ACOX3
15	39700001	39750000	0.0227173	SBF2
1	51340001	51390000	0.0227164	LOC108636831
18	56430001	56480000	0.0227127	BCAT2
18	56430001	56480000	0.0227127	LOC106503135
18	56430001	56480000	0.0227127	HSD17B14
18	56430001	56480000	0.0227127	PLEKHA4
12	35850001	35900000	0.022704	TBC1D4
8	82310001	82360000	0.0226948	ERCC6L2
1	93970001	94020000	0.0226942	SPATA16
21	18930001	18980000	0.0226928	LOC108633276
21	18930001	18980000	0.0226928	LOC102177379
3	10600001	10650000	0.0226864	NCDN
3	10600001	10650000	0.0226864	TFAP2E
3	10600001	10650000	0.0226864	KIAA0319L
18	15840001	15890000	0.0226737	ANKRD11
18	15840001	15890000	0.0226737	SPG7
9	20470001	20520000	0.0226625	DCBLD1
12	12230001	12280000	0.0226588	HS6ST3
10	49940001	49990000	0.0226579	TCF12
7	44660001	44710000	0.022657	GALNT10
7	67660001	67710000	0.0226501	LOC102168822
7	67660001	67710000	0.0226501	LOC102168535
7	66940001	66990000	0.0226481	TMEM259
7	66940001	66990000	0.0226481	CNN2
7	66940001	66990000	0.0226481	GRIN3B
7	66940001	66990000	0.0226481	ARID3A
7	66940001	66990000	0.0226481	WDR18
18	56340001	56390000	0.0226432	LOC102185028
18	56340001	56390000	0.0226432	MAMSTR
15	60360001	60410000	0.0226417	SIK2
15	5590001	5640000	0.0226314	C1QTNF4
15	5590001	5640000	0.0226314	FAM180B
15	5590001	5640000	0.0226314	KBTBD4
15	5590001	5640000	0.0226314	PTPMT1
15	5590001	5640000	0.0226314	NDUFS3
15	5590001	5640000	0.0226314	CELF1
2	23800001	23850000	0.0226228	AP1S3
8	38670001	38720000	0.0226178	ERMP1
12	55060001	55110000	0.0226132	FLT1
12	42030001	42080000	0.0226092	KLHL1
15	39620001	39670000	0.0225964	SBF2
12	55070001	55120000	0.0225908	FLT1
3	9900001	9950000	0.0225889	GJB3
3	9900001	9950000	0.0225889	GJA4
22	50720001	50770000	0.022584	NICN1
22	50720001	50770000	0.022584	AMT
22	50720001	50770000	0.022584	DAG1
21	3390001	3440000	0.0225804	GABRA5
4	97720001	97770000	0.0225799	DGKB
5	32990001	33040000	0.0225798	SLC38A4
8	22120001	22170000	0.0225752	CDKN2B
8	22120001	22170000	0.0225752	LOC102173187
8	22120001	22170000	0.0225752	LOC108636541
22	10240001	10290000	0.0225685	LRRFIP2
16	78890001	78940000	0.0225545	ARL8A
16	78890001	78940000	0.0225545	LOC102168445
16	78890001	78940000	0.0225545	GPR37L1

16	78890001	78940000	0.0225545	PTPN7
7	44670001	44720000	0.0225538	GALNT10
22	17330001	17380000	0.0225486	SRGAP3
6	117540001	117590000	0.0225257	LOC106502244
6	117540001	117590000	0.0225257	PDE6B
6	117540001	117590000	0.0225257	PIGG
8	350001	400000	0.0225202	ANXA10
17	30360001	30410000	0.0225163	TMEM144
8	22250001	22300000	0.0225125	MTAP
12	13070001	13120000	0.0225121	UGGT2
12	37440001	37490000	0.0224954	KLF12
21	380001	430000	0.0224932	MAGEL2
2	23160001	23210000	0.0224884	CUL3
14	91150001	91200000	0.0224867	FAM135A
1	51320001	51370000	0.0224767	LOC108636831
11	61120001	61170000	0.0224709	EHBP1
15	60370001	60420000	0.0224648	SIK2
9	81010001	81060000	0.0224626	ARID1B
9	81020001	81070000	0.0224524	ARID1B
6	72830001	72880000	0.0224449	LOC102183584
12	50380001	50430000	0.0224367	LOC108637296
12	50380001	50430000	0.0224367	MPHOSPH8
7	93110001	93160000	0.0224359	LOC102178529
6	114400001	114450000	0.0224342	AFAP1
22	19030001	19080000	0.022431	GRM7
1	101780001	101830000	0.0224281	SI
24	42450001	42500000	0.0224194	APCDD1
24	42190001	42240000	0.0224158	TXNDC2
24	42190001	42240000	0.0224158	RAB31
16	71700001	71750000	0.0224142	LOC108637792
16	71700001	71750000	0.0224142	SERTAD4
16	71700001	71750000	0.0224142	LOC108637749
1	112930001	112980000	0.0224029	TRNAE-UUC-2
1	112930001	112980000	0.0224029	GPR149
1	112930001	112980000	0.0224029	DHX36
5	32980001	33030000	0.0224012	SLC38A4
5	68740001	68790000	0.0223924	RFX4
5	68740001	68790000	0.0223924	RIC8B
26	35700001	35750000	0.0223833	NOC3L
26	35700001	35750000	0.0223833	PLCE1
14	91400001	91450000	0.0223739	SMAP1
19	61860001	61910000	0.022349	PRKCA
24	43020001	43070000	0.0223435	GNAL
2	20690001	20740000	0.0223417	IRS1
2	20690001	20740000	0.0223417	RHBDD1
7	71010001	71060000	0.0223394	COL23A1
22	16040001	16090000	0.0223389	TOPAZ1
22	16040001	16090000	0.0223389	TCAIM
22	16840001	16890000	0.0223213	MTMR14
4	112210001	112260000	0.0223177	CDK14
13	8930001	8980000	0.0223171	TRNAW-CCA-67
13	8930001	8980000	0.0223171	MACROD2
5	32970001	33020000	0.0223169	SLC38A4
7	66920001	66970000	0.0223153	TMEM259
7	66920001	66970000	0.0223153	ARHGAP45
7	66920001	66970000	0.0223153	ABCA7
7	66920001	66970000	0.0223153	CNN2
7	66920001	66970000	0.0223153	GRIN3B

25	25960001	26010000	0.0223109	IL27
25	25960001	26010000	0.0223109	NUPR1
25	25960001	26010000	0.0223109	APOBR
25	25960001	26010000	0.0223109	SGF29
25	25960001	26010000	0.0223109	CLN3
10	55440001	55490000	0.0223067	TLN2
4	1600001	1650000	0.0223041	LMBR1
7	44690001	44740000	0.0223037	GALNT10
18	14690001	14740000	0.0223031	SLC7A5
7	89080001	89130000	0.0222927	IZUMO4
7	89080001	89130000	0.0222927	MKNK2
7	89080001	89130000	0.0222927	MOB3A
7	89080001	89130000	0.0222927	AP3D1
4	97690001	97740000	0.0222896	DGKB
22	50700001	50750000	0.0222868	DAG1
15	8130001	8180000	0.0222731	TSPAN18
5	81930001	81980000	0.0222691	ITPR2
18	27260001	27310000	0.022253	LOC108637972
18	27260001	27310000	0.022253	LOC108637973
18	27260001	27310000	0.022253	MMP15
18	27260001	27310000	0.022253	USB1
26	21310001	21360000	0.0222492	ADD3
21	4460001	4510000	0.0222465	VIMP
18	15860001	15910000	0.0222377	SPG7
18	15860001	15910000	0.0222377	RPL13
18	15860001	15910000	0.0222377	CPNE7
26	15050001	15100000	0.0222275	PNLIPRP3
3	12700001	12750000	0.0222223	INPP5B
3	12700001	12750000	0.0222223	MTF1
6	116870001	116920000	0.0222206	HTT
24	42180001	42230000	0.0222004	TXNDC2
24	42180001	42230000	0.0222004	RAB31
14	26490001	26540000	0.0221955	LOC108637522
8	22270001	22320000	0.0221954	MTAP
6	116880001	116930000	0.0221953	HTT
22	17190001	17240000	0.0221937	SRGAP3
26	19310001	19360000	0.0221903	GPAM
5	31070001	31120000	0.0221902	LOC102173162
15	39720001	39770000	0.0221869	SBF2
7	44680001	44730000	0.0221787	GALNT10
16	49710001	49760000	0.0221669	PUSL1
16	49710001	49760000	0.0221669	SCNN1D
16	49710001	49760000	0.0221669	CPSF3L
16	49710001	49760000	0.0221669	ACAP3
16	49710001	49760000	0.0221669	UBE2J2
16	49710001	49760000	0.0221669	FAM132A
2	6640001	6690000	0.0221648	ID3
14	17910001	17960000	0.0221633	FBXO43
14	17910001	17960000	0.0221633	RGS22
6	65080001	65130000	0.0221543	GABRG1
3	12710001	12760000	0.0221505	INPP5B
3	12710001	12760000	0.0221505	MTF1
15	4830001	4880000	0.022138	LOC102168280
15	4830001	4880000	0.022138	LOC102177548
15	4830001	4880000	0.022138	LOC108637605
2	22190001	22240000	0.0221342	NYAP2
25	29280001	29330000	0.0221302	WBSCR17
22	17350001	17400000	0.0221298	SRGAP3

23	19890001	19940000	0.022129	TRNAF-GAA-23
23	19890001	19940000	0.022129	TRNAE-CUC-37
23	19890001	19940000	0.022129	TRNAL-AAG-9
23	19890001	19940000	0.022129	ZNF311
16	45040001	45090000	0.0221288	CAMTA1
19	49160001	49210000	0.0221053	LOC106503289
19	49160001	49210000	0.0221053	METRNL
7	92930001	92980000	0.0221033	ADGRE1
23	30100001	30150000	0.0221021	RUNX2
1	149500001	149550000	0.0220938	DYRK1A
5	31080001	31130000	0.0220891	LOC102173162
4	99780001	99830000	0.0220842	ARL4A
24	42500001	42550000	0.0220674	NAPG
24	42500001	42550000	0.0220674	APCDD1
24	42500001	42550000	0.0220674	LOC106503540
6	108960001	109010000	0.0220598	RAB28
7	45310001	45360000	0.0220585	GRIA1
18	12920001	12970000	0.0220478	LOC108637957
18	12920001	12970000	0.0220478	GSE1
24	42740001	42790000	0.0220456	PIEZO2
29	50030001	50080000	0.0220362	KCNQ1
14	91280001	91330000	0.0220319	SDHAF4
14	91280001	91330000	0.0220319	FAM135A
5	23340001	23390000	0.02203	CRADD
9	32490001	32540000	0.0220245	HACE1
10	66510001	66560000	0.0220176	INO80
7	89070001	89120000	0.0220022	MKNK2
7	89070001	89120000	0.0220022	MOB3A
16	49720001	49770000	0.0219943	SCNN1D
16	49720001	49770000	0.0219943	B3GALT6
16	49720001	49770000	0.0219943	ACAP3
16	49720001	49770000	0.0219943	UBE2J2
16	49720001	49770000	0.0219943	FAM132A
6	117470001	117520000	0.0219933	ATP5I
6	117470001	117520000	0.0219933	LOC106502243
6	117470001	117520000	0.0219933	MFSD7
6	117470001	117520000	0.0219933	PDE6B
6	117470001	117520000	0.0219933	PCGF3
26	47150001	47200000	0.0219929	LOC102181467
12	35870001	35920000	0.0219913	TBC1D4
10	50250001	50300000	0.0219858	CGNL1
24	42790001	42840000	0.0219845	PIEZO2
17	30370001	30420000	0.0219841	TMEM144
22	11460001	11510000	0.0219586	ACAA1
22	11460001	11510000	0.0219586	MYD88
22	11460001	11510000	0.0219586	DLEC1
22	12530001	12580000	0.0219565	LOC100861209
22	12530001	12580000	0.0219565	RPSA
10	65450001	65500000	0.0219454	TMEM87A
10	65450001	65500000	0.0219454	VPS39
22	42320001	42370000	0.0219357	C22H3orf67
4	70340001	70390000	0.0219194	LOC108635915
7	60620001	60670000	0.0219171	EGR1
7	60620001	60670000	0.0219171	ETF1
8	22880001	22930000	0.0219146	KLHL9
8	22880001	22930000	0.0219146	LOC108636545
8	22880001	22930000	0.0219146	LOC102178623
5	68790001	68840000	0.0219094	RIC8B

10	75700001	75750000	0.0219084	LOC102180319
10	75700001	75750000	0.0219084	LOC102180047
10	75700001	75750000	0.0219084	LOC102179772
10	75700001	75750000	0.0219084	LOC102179480
5	68760001	68810000	0.0219045	RIC8B
2	22200001	22250000	0.0218998	NYAP2
5	86930001	86980000	0.0218933	ABCC9
6	117480001	117530000	0.0218909	ATP5I
6	117480001	117530000	0.0218909	LOC106502243
6	117480001	117530000	0.0218909	MFSD7
6	117480001	117530000	0.0218909	PDE6B
10	66490001	66540000	0.0218879	INO80
3	119720001	119770000	0.0218744	SFT2D2
5	33020001	33070000	0.0218537	SLC38A4
21	5610001	5660000	0.0218301	ADAMTS17
10	50140001	50190000	0.0218231	TCF12
7	89090001	89140000	0.0218123	IZUMO4
7	89090001	89140000	0.0218123	MOB3A
7	89090001	89140000	0.0218123	AP3D1
13	65320001	65370000	0.021809	NDRG3
1	117060001	117110000	0.0218031	LOC102169223
1	117060001	117110000	0.0218031	ERICH6
28	15150001	15200000	0.0217979	ADK
7	45290001	45340000	0.0217957	GRIA1
5	68780001	68830000	0.0217953	RIC8B
6	116850001	116900000	0.0217816	HTT
15	39780001	39830000	0.0217813	SBF2
6	117490001	117540000	0.0217695	ATP5I
6	117490001	117540000	0.0217695	LOC106502243
6	117490001	117540000	0.0217695	MFSD7
6	117490001	117540000	0.0217695	PDE6B
1	53160001	53210000	0.0217672	KIAA1524
1	53160001	53210000	0.0217672	DZIP3
6	111470001	111520000	0.0217648	PROM1
11	72650001	72700000	0.0217614	TRNAE-UUC-46
11	72650001	72700000	0.0217614	CIB4
14	12110001	12160000	0.0217589	GEM
12	35860001	35910000	0.0217523	TBC1D4
22	41830001	41880000	0.0217446	LOC106503441
5	68750001	68800000	0.0217444	RIC8B
22	41810001	41860000	0.0217339	LOC106503441
7	60570001	60620000	0.0217013	HSPA9
7	60570001	60620000	0.0217013	ETF1
7	60570001	60620000	0.0217013	LOC108636363
4	1530001	1580000	0.0217003	NOM1
15	8830001	8880000	0.0216995	ACCSL
22	42290001	42340000	0.0216989	C22H3orf67
4	1580001	1630000	0.0216918	LMBR1
22	14930001	14980000	0.0216815	LOC102173615
22	14930001	14980000	0.0216815	LOC102173976
12	55210001	55260000	0.0216812	LOC102188230
12	55210001	55260000	0.0216812	POMP
15	8840001	8890000	0.0216747	ACCSL
4	87020001	87070000	0.0216659	KIAA1324L
24	43650001	43700000	0.0216651	LDLRAD4
9	74620001	74670000	0.0216518	PPP1R14C
18	39690001	39740000	0.0216436	LOC108637979
24	14150001	14200000	0.0216378	PIK3C3

8	22260001	22310000	0.0216377	MTAP
18	39700001	39750000	0.0216352	LOC108637979
5	26740001	26790000	0.0216311	LOC108636128
5	26740001	26790000	0.0216311	KRT8
13	9220001	9270000	0.0216218	MACROD2
2	83940001	83990000	0.0216199	GTDC1
22	51340001	51390000	0.0216199	UCN2
22	51340001	51390000	0.0216199	LOC102188264
22	51340001	51390000	0.0216199	PFKFB4
22	51340001	51390000	0.0216199	COL7A1
16	10620001	10670000	0.0216191	CDC73
16	10620001	10670000	0.0216191	B3GALT2
20	62090001	62140000	0.0216185	CTNND2
22	17360001	17410000	0.0216117	SRGAP3
17	30350001	30400000	0.0216095	TMEM144
1	149490001	149540000	0.0215966	DYRK1A
11	20260001	20310000	0.0215861	RMDN2
4	5050001	5100000	0.0215842	KMT2C
6	72840001	72890000	0.0215828	LOC102183584
1	117070001	117120000	0.0215764	LOC102169223
13	70920001	70970000	0.021571	PTPRT
4	115140001	115190000	0.0215633	DDC
4	115140001	115190000	0.0215633	GRB10
10	68830001	68880000	0.0215631	RASGRP1
6	65410001	65460000	0.0215585	GABRA2
12	28400001	28450000	0.0215487	TRNAC-GCA-166
5	68800001	68850000	0.0215458	RIC8B
29	50020001	50070000	0.0215452	KCNQ1
2	6610001	6660000	0.0215401	E2F2
2	6610001	6660000	0.0215401	ID3
29	45690001	45740000	0.0215388	CD151
29	45690001	45740000	0.0215388	PNPLA2
29	45690001	45740000	0.0215388	RPLP2
29	45690001	45740000	0.0215388	PIDD1
29	45690001	45740000	0.0215388	SLC25A22
29	45690001	45740000	0.0215388	PDDC1
29	45690001	45740000	0.0215388	CRACR2B
29	45690001	45740000	0.0215388	CEND1
20	9130001	9180000	0.0215262	ZNF366
6	106860001	106910000	0.0215235	TRNAC-GCA-105
12	50320001	50370000	0.0215157	MPHOSPH8
12	50320001	50370000	0.0215157	PARP4
12	17690001	17740000	0.0215128	GPC6
18	27250001	27300000	0.0215057	ZNF319
18	27250001	27300000	0.0215057	LOC108637972
18	27250001	27300000	0.0215057	LOC108637973
18	27250001	27300000	0.0215057	MMP15
18	27250001	27300000	0.0215057	USB1
12	33850001	33900000	0.0214934	FBXL3
12	33850001	33900000	0.0214934	MYCBP2
21	4450001	4500000	0.0214933	VIMP
4	92090001	92140000	0.0214916	TMEM196
4	92090001	92140000	0.0214916	TWISTNB
9	83470001	83520000	0.0214868	IGF2R
10	75670001	75720000	0.0214835	LOC102180597
10	75670001	75720000	0.0214835	LOC102184528
10	75670001	75720000	0.0214835	LOC102180319
10	75670001	75720000	0.0214835	LOC102180047

24	43640001	43690000	0.0214835	LDLRAD4
7	71040001	71090000	0.021483	COL23A1
14	52000001	52050000	0.0214809	LOC108637530
14	52000001	52050000	0.0214809	PDE7A
14	52000001	52050000	0.0214809	MTFR1
10	97090001	97140000	0.0214684	CCDC112
10	97090001	97140000	0.0214684	PGGT1B
29	580001	630000	0.0214668	MIR2404
29	580001	630000	0.0214668	C29H11orf54
29	580001	630000	0.0214668	TAF1D
29	580001	630000	0.0214668	CEP295
5	31060001	31110000	0.0214599	LOC102173162
5	82030001	82080000	0.0214459	ITPR2
16	69450001	69500000	0.0214448	NSL1
16	69450001	69500000	0.0214448	BATF3
8	37660001	37710000	0.0214432	KDM4C
7	58330001	58380000	0.0214355	PCDHB1
22	12300001	12350000	0.0214283	CSRNP1
22	12300001	12350000	0.0214283	TTC21A
5	68770001	68820000	0.0214263	RIC8B
17	36480001	36530000	0.0214256	SPATA5
22	19020001	19070000	0.0214188	GRM7
21	5220001	5270000	0.0214129	ASB7
8	4200001	4250000	0.0213971	GALNTL6
24	57050001	57100000	0.0213934	NARS
24	57050001	57100000	0.0213934	ATP8B1
6	100700001	100750000	0.0213897	LOC102189022
6	100700001	100750000	0.0213897	ARHGAP24
12	16370001	16420000	0.0213876	ABCC4
14	4050001	4100000	0.0213874	RALYL
12	16350001	16400000	0.0213808	ABCC4
4	92100001	92150000	0.0213805	TMEM196
4	92100001	92150000	0.0213805	TWISTNB
22	17170001	17220000	0.0213774	SRGAP3
24	42200001	42250000	0.0213695	TXNDC2
24	42200001	42250000	0.0213695	RAB31
12	33900001	33950000	0.0213472	FBXL3
12	33900001	33950000	0.0213472	CLN5
17	30380001	30430000	0.0213414	TMEM144
15	8260001	8310000	0.0213391	CD82
23	19900001	19950000	0.0213349	TRNAL-AAG-9
23	19900001	19950000	0.0213349	LOC102174177
23	19900001	19950000	0.0213349	ZNF311
2	111660001	111710000	0.0213329	HAT1
21	5200001	5250000	0.0213118	ASB7
3	9190001	9240000	0.0213049	C3H1orf94
1	51360001	51410000	0.0212809	LOC108636831
22	50710001	50760000	0.0212746	DAG1
11	82560001	82610000	0.0212744	NBAS
1	65300001	65350000	0.0212738	STXBP5L
12	50330001	50380000	0.0212721	MPHOSPH8
12	50330001	50380000	0.0212721	PARP4
29	42180001	42230000	0.0212719	LOC108633235
26	28170001	28220000	0.0212636	AS3MT
26	28170001	28220000	0.0212636	CNNM2
2	20700001	20750000	0.0212625	IRS1
13	37430001	37480000	0.021254	LOC108637351
13	37430001	37480000	0.021254	RRBP1

12	60010001	60060000	0.0212535	LOC102178917
1	53200001	53250000	0.0212495	KIAA1524
1	53200001	53250000	0.0212495	DZIP3
15	8760001	8810000	0.0212423	LOC108637652
15	8760001	8810000	0.0212423	ACCS
15	8760001	8810000	0.0212423	EXT2
5	81970001	82020000	0.0212421	ITPR2
2	88140001	88190000	0.0212367	LOC106502907
4	96440001	96490000	0.0212322	MEOX2
7	41380001	41430000	0.02123	ADAM19
4	91660001	91710000	0.0212295	MACC1
25	22580001	22630000	0.0212269	ARHGAP17
25	22580001	22630000	0.0212269	LCMT1
3	9880001	9930000	0.0212257	GJB5
3	9880001	9930000	0.0212257	GJB3
3	9880001	9930000	0.0212257	GJB4
4	90120001	90170000	0.021225	DNAH11
13	71190001	71240000	0.0212244	PTPRT
17	36490001	36540000	0.0212229	SPATA5
15	39710001	39760000	0.0212186	SBF2
8	37770001	37820000	0.0212111	KDM4C
18	54120001	54170000	0.021204	PPM1N
18	54120001	54170000	0.021204	LOC108638001
18	54120001	54170000	0.021204	RTN2
18	54120001	54170000	0.021204	VASP
18	54120001	54170000	0.021204	FOSB
12	10400001	10450000	0.0211919	DOCK9
23	30090001	30140000	0.0211684	TRNAE-UUC-88
23	30090001	30140000	0.0211684	RUNX2
11	22260001	22310000	0.0211683	SLC8A1
4	90140001	90190000	0.021168	DNAH11
2	36060001	36110000	0.0211659	ERBB4
26	15040001	15090000	0.0211634	PNLIPRP3
9	20450001	20500000	0.0211626	DCBLD1
18	26920001	26970000	0.0211589	LOC108637971
18	26920001	26970000	0.0211589	ADGRG1
5	25540001	25590000	0.0211401	CBX5
9	36630001	36680000	0.0211393	ASCC3
18	56440001	56490000	0.0211391	BCAT2
18	56440001	56490000	0.0211391	LOC106503135
18	56440001	56490000	0.0211391	HSD17B14
18	56440001	56490000	0.0211391	PLEKHA4
18	56440001	56490000	0.0211391	PPP1R15A
22	50690001	50740000	0.0211298	DAG1
1	113150001	113200000	0.0211272	ARHGEF26
1	51260001	51310000	0.0211194	LOC108636831
9	32460001	32510000	0.0211193	HACE1
10	66480001	66530000	0.0211164	INO80
18	12940001	12990000	0.0211023	LOC108637957
18	12940001	12990000	0.0211023	GSE1
6	65090001	65140000	0.021102	GABRG1
22	38890001	38940000	0.0210976	FEZF2
3	4960001	5010000	0.0210926	IQCA1
29	50040001	50090000	0.0210926	KCNQ1
16	10630001	10680000	0.0210909	CDC73
16	10630001	10680000	0.0210909	B3GALT2
3	3390001	3440000	0.0210877	RAMP1
4	14190001	14240000	0.0210844	LOC108635908

4	14190001	14240000	0.0210844	LOC108635909
4	14190001	14240000	0.0210844	LOC102181470
5	68690001	68740000	0.0210763	RFX4
22	15290001	15340000	0.0210736	ANO10
19	34280001	34330000	0.0210718	MIR33B
19	34280001	34330000	0.0210718	SREBF1
19	34280001	34330000	0.0210718	RAI1
20	9100001	9150000	0.0210701	ZNF366
19	61850001	61900000	0.0210456	PRKCA
24	43580001	43630000	0.0210391	CEP192
1	1370001	1420000	0.0210246	C1H21orf59
1	1370001	1420000	0.0210246	SYNJ1
5	33460001	33510000	0.0210057	SLC38A1
17	70260001	70310000	0.0210026	TLL1
26	5940001	5990000	0.0210008	C26H10orf90
18	12930001	12980000	0.0209982	LOC108637957
18	12930001	12980000	0.0209982	GSE1
2	23090001	23140000	0.0209952	CUL3
10	50160001	50210000	0.0209936	TCF12
19	23040001	23090000	0.0209924	SRR
19	23040001	23090000	0.0209924	SMG6
16	28090001	28140000	0.0209833	STUM
4	91650001	91700000	0.0209828	MACC1
14	17890001	17940000	0.0209814	RGS22
12	13540001	13590000	0.0209731	LOC102187779
4	10250001	10300000	0.0209713	CNTNAP2
29	32060001	32110000	0.0209602	LOC108634249
22	17220001	17270000	0.0209579	SRGAP3
5	68960001	69010000	0.0209575	CRY1
1	152100001	152150000	0.0209573	CAPN7
1	152100001	152150000	0.0209573	SH3BP5
18	23340001	23390000	0.0209548	RPGRIP1L
18	12430001	12480000	0.0209542	CRISPLD2
11	20230001	20280000	0.020952	RMDN2
4	96510001	96560000	0.0209518	AGMO
29	31790001	31840000	0.0209452	ARHGAP32
2	111710001	111760000	0.0209396	HAT1
2	111710001	111760000	0.0209396	METAP1D
10	50120001	50170000	0.0209396	TCF12
16	10640001	10690000	0.0209382	CDC73
16	10640001	10690000	0.0209382	B3GALT2
12	55080001	55130000	0.0209226	FLT1
11	28110001	28160000	0.0209205	PRKCE
2	10100001	10150000	0.0209159	WASF2
3	54200001	54250000	0.0209152	ZZZ3
3	54200001	54250000	0.0209152	USP33
15	39680001	39730000	0.0209109	SBF2
11	13430001	13480000	0.020905	CD207
11	13430001	13480000	0.020905	CLEC4F
11	13430001	13480000	0.020905	FIGLA
9	6560001	6610000	0.0208797	SH3BGRL2
3	4950001	5000000	0.0208762	IQCA1
22	23970001	24020000	0.0208723	LOC108633370
4	97730001	97780000	0.0208679	DGKB
13	9230001	9280000	0.020843	MACROD2
9	32500001	32550000	0.0208342	HACE1
2	83900001	83950000	0.0208311	GTDC1
2	23150001	23200000	0.0208295	CUL3

6	103990001	104040000	0.0208287	EVC2
14	17900001	17950000	0.0208278	RGS22
19	62420001	62470000	0.0208218	LOC102172100
19	62420001	62470000	0.0208218	HELZ
6	108950001	109000000	0.0208077	RAB28
13	39980001	40030000	0.020803	XRN2
4	90990001	91040000	0.020799	SP8
8	22240001	22290000	0.0207912	MTAP
22	11420001	11470000	0.0207905	ACAA1
22	11420001	11470000	0.0207905	DLEC1
16	49790001	49840000	0.0207898	TNFRSF18
16	49790001	49840000	0.0207898	MIR429
16	49790001	49840000	0.0207898	MIR200A
16	49790001	49840000	0.0207898	MIR200B
16	49790001	49840000	0.0207898	TTLL10
19	28290001	28340000	0.020789	NTN1
16	49570001	49620000	0.0207813	TMEM240
16	49570001	49620000	0.0207813	TMEM88B
16	49570001	49620000	0.0207813	SSU72
16	49570001	49620000	0.0207813	LOC102189890
16	49570001	49620000	0.0207813	VWA1
13	10690001	10740000	0.020781	FBXO18
13	10690001	10740000	0.020781	IL15RA
3	3380001	3430000	0.0207794	UBE2F
3	3380001	3430000	0.0207794	RAMP1
19	31660001	31710000	0.0207788	TRNAR-CCG
14	91430001	91480000	0.0207781	SMAP1
25	29290001	29340000	0.0207705	WBSCR17
4	99740001	99790000	0.0207609	ARL4A
29	32080001	32130000	0.0207585	LOC108634249
8	39550001	39600000	0.0207553	RCL1
2	113980001	114030000	0.0207529	GPR155
22	12520001	12570000	0.0207445	LOC100861209
22	12520001	12570000	0.0207445	RPSA
22	50240001	50290000	0.0207338	GNAT1
22	50240001	50290000	0.0207338	SEMA3F
13	7400001	7450000	0.0207267	MACROD2
24	43670001	43720000	0.0207261	LDLRAD4
24	43030001	43080000	0.0207222	CHMP1B
24	43030001	43080000	0.0207222	GNAL
24	43570001	43620000	0.0207204	CEP192
12	12240001	12290000	0.0207154	HS6ST3
13	2280001	2330000	0.0207121	PAK5
22	11840001	11890000	0.0207115	SCN5A
16	10610001	10660000	0.0207059	CDC73
6	116690001	116740000	0.0207045	ADD1
2	23050001	23100000	0.0207039	CUL3
15	8850001	8900000	0.0207018	LOC106502911
5	29840001	29890000	0.0206985	FMNL3
5	29840001	29890000	0.0206985	PRPF40B
5	29840001	29890000	0.0206985	FAM186B
7	93130001	93180000	0.0206942	LOC102175946
7	93130001	93180000	0.0206942	ACTL9
18	15880001	15930000	0.0206903	SPG7
18	15880001	15930000	0.0206903	RPL13
18	15880001	15930000	0.0206903	CPNE7
10	78780001	78830000	0.0206854	LOC102182508
4	92840001	92890000	0.0206852	HDAC9

5	25180001	25230000	0.0206742	GLYCAM1
2	88150001	88200000	0.0206737	LOC106502907
19	49190001	49240000	0.0206732	METRNL
19	49190001	49240000	0.0206732	B3GNTL1
9	81030001	81080000	0.0206676	ARID1B
4	91640001	91690000	0.0206641	MACC1
11	82570001	82620000	0.0206606	NBAS
4	90130001	90180000	0.0206569	DNAH11
1	53070001	53120000	0.020654	MYH15
12	17700001	17750000	0.020654	TRNAE-UUC-49
12	17700001	17750000	0.020654	GPC6
19	23080001	23130000	0.0206533	TSR1
19	23080001	23130000	0.0206533	TRNAG-CCC-91
19	23080001	23130000	0.0206533	SRR
19	23080001	23130000	0.0206533	SGSM2
4	70350001	70400000	0.0206515	LOC108635915
29	45650001	45700000	0.0206481	CLCF1
29	45650001	45700000	0.0206481	RAD9A
29	45650001	45700000	0.0206481	POLR2L
29	45650001	45700000	0.0206481	CD151
29	45650001	45700000	0.0206481	PNPLA2
29	45650001	45700000	0.0206481	PPP1CA
29	45650001	45700000	0.0206481	CRACR2B
3	54190001	54240000	0.0206398	ZZZ3
3	54190001	54240000	0.0206398	USP33
15	60400001	60450000	0.0206379	SIK2
6	85660001	85710000	0.0206309	LOC102168522
24	43560001	43610000	0.0205906	CEP192
11	72640001	72690000	0.0205883	TRNAE-UUC-46
11	72640001	72690000	0.0205883	CIB4
7	60630001	60680000	0.0205839	EGR1
7	60630001	60680000	0.0205839	REEP2
7	60630001	60680000	0.0205839	ETF1
10	18030001	18080000	0.020583	DNAL1
10	18030001	18080000	0.020583	ACOT6
10	18030001	18080000	0.020583	LOC102184912
14	12060001	12110000	0.020579	CDH17
1	97090001	97140000	0.0205726	ACTRT3
1	97090001	97140000	0.0205726	TRNAV-AAC
1	97090001	97140000	0.0205726	LRRC34
1	97090001	97140000	0.0205726	MYNN
13	2300001	2350000	0.0205715	PAK5
15	8720001	8770000	0.0205709	EXT2
2	110350001	110400000	0.0205707	MYO3B
16	43230001	43280000	0.0205689	RERE
4	94670001	94720000	0.0205653	TRNAW-CCA-22
4	94670001	94720000	0.0205653	AHR
2	23080001	23130000	0.0205651	CUL3
7	64490001	64540000	0.020558	CDKN2AIPNL
8	39600001	39650000	0.0205522	AK3
22	9330001	9380000	0.0205503	TRNAQ-CUG-13
9	150001	200000	0.0205464	MTO1
9	150001	200000	0.0205464	EEF1A1
23	30080001	30130000	0.0205454	TRNAE-UUC-88
23	30080001	30130000	0.0205454	RUNX2
1	52050001	52100000	0.0205403	BBX
1	53500001	53550000	0.0205336	GUCA1C
9	36640001	36690000	0.0205252	ASCC3

7	60600001	60650000	0.0205198	ETF1
12	8060001	8110000	0.0205194	FGF14
3	4930001	4980000	0.0205116	IQCA1
18	23380001	23430000	0.0205108	RPGRIP1L
18	23380001	23430000	0.0205108	FTO
3	3410001	3460000	0.0205061	RAMP1
1	111240001	111290000	0.0205021	GMPS
26	21300001	21350000	0.0205008	ADD3
6	108970001	109020000	0.0204988	RAB28
6	106850001	106900000	0.020498	TRNAC-GCA-105
21	6060001	6110000	0.0204971	MEF2A
3	4940001	4990000	0.0204938	IQCA1
26	18680001	18730000	0.0204876	VTI1A
18	27270001	27320000	0.0204865	LOC108637974
18	27270001	27320000	0.0204865	LOC108637973
18	27270001	27320000	0.0204865	MMP15
18	27270001	27320000	0.0204865	USB1
15	7970001	8020000	0.0204828	TP53I11
15	7970001	8020000	0.0204828	TSPAN18
12	50310001	50360000	0.0204807	PARP4
19	35450001	35500000	0.0204711	SPAG9
1	53490001	53540000	0.0204699	GUCA1C
9	36620001	36670000	0.020467	ASCC3
1	118590001	118640000	0.0204478	CP
1	118590001	118640000	0.0204478	HPS3
29	590001	640000	0.0204417	MIR2404
29	590001	640000	0.0204417	TAF1D
29	590001	640000	0.0204417	CEP295
15	8770001	8820000	0.0204405	LOC108637652
15	8770001	8820000	0.0204405	ACCS
15	8770001	8820000	0.0204405	EXT2
8	22870001	22920000	0.0204397	KLHL9
8	22870001	22920000	0.0204397	LOC102178623
4	92810001	92860000	0.0204292	HDAC9
19	37380001	37430000	0.0204276	HOXB13
19	37380001	37430000	0.0204276	TTLL6
10	50150001	50200000	0.0204267	TCF12
9	49450001	49500000	0.020425	TRNAE-UUC-37
2	109160001	109210000	0.0204247	ABCB11
2	109160001	109210000	0.0204247	DHRS9
6	116560001	116610000	0.0204243	TNIP2
16	32990001	33040000	0.0204148	PLD5
4	96480001	96530000	0.0204109	MEOX2
11	49920001	49970000	0.0204041	DNAH6
16	28070001	28120000	0.0204023	STUM
2	88160001	88210000	0.0203856	LOC106502907
3	13620001	13670000	0.020377	RRAGC
26	45920001	45970000	0.0203762	PCDH15
9	20480001	20530000	0.0203758	DCBLD1
16	78900001	78950000	0.0203708	ARL8A
16	78900001	78950000	0.0203708	GPR37L1
16	78900001	78950000	0.0203708	PTPN7
22	16030001	16080000	0.0203706	TOPAZ1
19	23210001	23260000	0.0203694	LOC108638230
19	23210001	23260000	0.0203694	METTL16
6	114370001	114420000	0.0203655	AFAP1
29	390001	440000	0.0203645	LOC106503712
26	15110001	15160000	0.0203557	PNLIPRP3

8	60390001	60440000	0.0203528	RNF38
1	112970001	113020000	0.0203519	DHX36
1	112970001	113020000	0.0203519	ARHGEF26
4	99770001	99820000	0.0203381	ARL4A
26	28120001	28170000	0.0203353	CNNM2
1	1400001	1450000	0.0203332	EVA1C
6	117460001	117510000	0.0203328	MFSD7
6	117460001	117510000	0.0203328	PCGF3
9	88980001	89030000	0.0203269	RPS6KA2
1	53040001	53090000	0.0203255	MYH15
2	110310001	110360000	0.0203182	MYO3B
3	3370001	3420000	0.0203156	UBE2F
3	3370001	3420000	0.0203156	RAMP1
7	67670001	67720000	0.0203127	LOC102168535
7	67670001	67720000	0.0203127	LOC102191610
6	117500001	117550000	0.0203121	ATP5I
6	117500001	117550000	0.0203121	LOC106502243
6	117500001	117550000	0.0203121	MFSD7
6	117500001	117550000	0.0203121	PDE6B
12	10220001	10270000	0.0203067	UBAC2
4	5060001	5110000	0.0203036	KMT2C
22	19370001	19420000	0.0203029	GRM7
16	49550001	49600000	0.0203027	TMEM240
16	49550001	49600000	0.0203027	SSU72
16	49550001	49600000	0.0203027	LOC102189890
4	115150001	115200000	0.0203012	DDC
4	115150001	115200000	0.0203012	GRB10
16	28080001	28130000	0.0202912	STUM
6	117530001	117580000	0.0202893	LOC106502244
6	117530001	117580000	0.0202893	PDE6B
7	90090001	90140000	0.0202841	SMIM24
7	90090001	90140000	0.0202841	LOC102178997
7	90090001	90140000	0.0202841	NFIC
21	5190001	5240000	0.0202833	ASB7
2	10110001	10160000	0.0202775	WASF2
24	49680001	49730000	0.0202719	MYO5B
12	8360001	8410000	0.0202695	ITGBL1
15	8320001	8370000	0.0202646	CD82
4	57970001	58020000	0.0202592	NPSR1
2	23070001	23120000	0.0202576	CUL3
4	68080001	68130000	0.0202546	TES
15	39790001	39840000	0.0202502	SBF2
16	49560001	49610000	0.0202487	TMEM240
16	49560001	49610000	0.0202487	SSU72
16	49560001	49610000	0.0202487	LOC102189890
16	49560001	49610000	0.0202487	VWA1
16	43220001	43270000	0.0202468	RERE
16	49530001	49580000	0.0202467	FNDC10
16	49530001	49580000	0.0202467	TMEM240
16	49530001	49580000	0.0202467	SSU72
18	57560001	57610000	0.0202352	NR1H2
18	57560001	57610000	0.0202352	POLD1
18	57560001	57610000	0.0202352	SPIB
18	57560001	57610000	0.0202352	MYBPC2
6	70090001	70140000	0.0202298	CHIC2
1	99160001	99210000	0.0202203	SERPINI1
13	69750001	69800000	0.0202078	CHD6
18	21820001	21870000	0.020204	LOC108637964

22	16120001	16170000	0.0202039	LOC102176467
22	16120001	16170000	0.0202039	ZNF445
8	60400001	60450000	0.0202019	RNF38
6	85650001	85700000	0.0202008	LOC102168522
8	60380001	60430000	0.0201961	RNF38
1	53510001	53560000	0.0201919	GUCA1C
29	45660001	45710000	0.0201908	RAD9A
29	45660001	45710000	0.0201908	POLR2L
29	45660001	45710000	0.0201908	CD151
29	45660001	45710000	0.0201908	PNPLA2
29	45660001	45710000	0.0201908	RPLP2
29	45660001	45710000	0.0201908	PPP1CA
29	45660001	45710000	0.0201908	CRACR2B
22	19010001	19060000	0.0201905	GRM7
8	46200001	46250000	0.0201877	LOC102187766
8	46200001	46250000	0.0201877	SMC5
2	88130001	88180000	0.0201859	LOC106502907
2	88130001	88180000	0.0201859	ORC4
13	6910001	6960000	0.0201754	TASP1
9	20490001	20540000	0.0201725	DCBLD1
18	56420001	56470000	0.020168	BCAT2
18	56420001	56470000	0.020168	LOC106503135
18	56420001	56470000	0.020168	HSD17B14
18	56420001	56470000	0.020168	PLEKHA4
2	23140001	23190000	0.0201536	CUL3
8	37650001	37700000	0.0201511	KDM4C
1	51350001	51400000	0.0201422	LOC108636831
12	60000001	60050000	0.0201386	LOC102178917
4	91670001	91720000	0.0201359	MACC1
6	72810001	72860000	0.0201267	LOC102180384
6	72810001	72860000	0.0201267	LOC102183584
11	93700001	93750000	0.020126	RABGAP1
12	12220001	12270000	0.0201177	HS6ST3
1	101720001	101770000	0.0200754	SLITRK3
6	117520001	117570000	0.020071	LOC106502244
6	117520001	117570000	0.020071	PDE6B
17	23810001	23860000	0.0200708	TMEM132D
4	90150001	90200000	0.0200679	DNAH11
1	96700001	96750000	0.0200673	PRKCI
1	96700001	96750000	0.0200673	PHC3
22	16090001	16140000	0.0200595	LOC102176467
22	16090001	16140000	0.0200595	TCAIM
8	37810001	37860000	0.0200576	KDM4C
10	32280001	32330000	0.0200544	DACT1
4	111480001	111530000	0.0200474	LOC108635860
22	51360001	51410000	0.0200469	UCN2
22	51360001	51410000	0.0200469	PFKFB4
22	51360001	51410000	0.0200469	COL7A1
7	71030001	71080000	0.0200464	COL23A1
11	13440001	13490000	0.0200454	CD207
11	13440001	13490000	0.0200454	CLEC4F
11	13440001	13490000	0.0200454	FIGLA
4	96540001	96590000	0.0200398	AGMO
10	65500001	65550000	0.0200306	VPS39
10	65500001	65550000	0.0200306	PLA2G4F
13	10680001	10730000	0.0200285	FBXO18
14	12660001	12710000	0.0200231	DPY19L4
14	12660001	12710000	0.0200231	INTS8

12	42020001	42070000	0.0200218	KLHL1
21	4470001	4520000	0.0200211	VIMP
21	4470001	4520000	0.0200211	CHSY1
8	22890001	22940000	0.0200198	KLHL9
8	22890001	22940000	0.0200198	LOC108636545
8	22890001	22940000	0.0200198	IFNA
8	22890001	22940000	0.0200198	LOC102178623
16	49850001	49900000	0.0200156	C16H1orf159
12	10210001	10260000	0.0200153	UBAC2
9	6550001	6600000	0.0200147	SH3BGRL2
23	30110001	30160000	0.020006	RUNX2
22	23960001	24010000	0.0200027	LOC108633370
3	7060001	7110000	0.0199968	TRPM8
3	7060001	7110000	0.0199968	SPP2
22	17210001	17260000	0.0199966	SRGAP3
10	50260001	50310000	0.0199911	CGNL1
7	93100001	93150000	0.0199827	LOC102178529
16	69440001	69490000	0.0199803	NSL1
7	45070001	45120000	0.0199713	FAM114A2
28	17430001	17480000	0.0199696	SPOCK2
28	17430001	17480000	0.0199696	ASCC1
18	54110001	54160000	0.0199657	PPM1N
18	54110001	54160000	0.0199657	LOC108638001
18	54110001	54160000	0.0199657	RTN2
18	54110001	54160000	0.0199657	VASP
18	54110001	54160000	0.0199657	FOSB
24	14100001	14150000	0.019965	PIK3C3
18	14680001	14730000	0.0199639	SLC7A5
25	21240001	21290000	0.0199629	DCTN5
25	21240001	21290000	0.0199629	PLK1
25	21240001	21290000	0.0199629	ERN2
26	18690001	18740000	0.0199587	VTI1A
13	37440001	37490000	0.0199541	LOC108637351
13	37440001	37490000	0.0199541	RRBP1
10	87920001	87970000	0.0199528	LOC106502512
10	87920001	87970000	0.0199528	SNAPC5
10	87920001	87970000	0.0199528	MAP2K1
13	37770001	37820000	0.0199505	PET117
13	37770001	37820000	0.0199505	KAT14
16	49690001	49740000	0.0199502	TAS1R3
16	49690001	49740000	0.0199502	CPTP
16	49690001	49740000	0.0199502	PUSL1
16	49690001	49740000	0.0199502	SCNN1D
16	49690001	49740000	0.0199502	DVL1
16	49690001	49740000	0.0199502	CPSF3L
16	49690001	49740000	0.0199502	ACAP3
13	37780001	37830000	0.0199408	PET117
13	37780001	37830000	0.0199408	KAT14
22	16770001	16820000	0.0199369	OGG1
22	16770001	16820000	0.0199369	BRPF1
22	16770001	16820000	0.0199369	CPNE9
22	16770001	16820000	0.0199369	CAMK1
1	101800001	101850000	0.0199301	SI
14	26500001	26550000	0.0199299	LOC108637522
18	56350001	56400000	0.0199293	LOC102185028
18	56350001	56400000	0.0199293	IZUMO1
18	56350001	56400000	0.0199293	FUT1
18	56350001	56400000	0.0199293	MAMSTR

4	99800001	99850000	0.0199223	SCIN
8	39640001	39690000	0.0199197	AK3
8	39640001	39690000	0.0199197	CDC37L1
11	3040001	3090000	0.0199108	TMEM131
9	83490001	83540000	0.0199102	IGF2R
18	63100001	63150000	0.0199041	LOC102174057
18	63100001	63150000	0.0199041	LOC102173463
17	3340001	3390000	0.019902	TTC28
22	19000001	19050000	0.0198949	GRM7
2	20680001	20730000	0.0198901	RHBDD1
8	38610001	38660000	0.019887	MLANA
8	38610001	38660000	0.019887	KIAA2026
17	36470001	36520000	0.019887	SPATA5
1	110510001	110560000	0.019886	TIPARP
4	1610001	1660000	0.019882	LMBR1
10	65440001	65490000	0.0198794	TMEM87A
10	65440001	65490000	0.0198794	VPS39
3	25680001	25730000	0.0198785	C3H1orf185
4	96520001	96570000	0.0198746	AGMO
17	36510001	36560000	0.0198727	SPATA5
19	31650001	31700000	0.0198667	TRNAR-CCG
22	50230001	50280000	0.0198633	GNAT1
22	50230001	50280000	0.0198633	SEMA3F
12	50300001	50350000	0.0198631	PARP4
20	37500001	37550000	0.0198593	SLC1A3
16	49740001	49790000	0.0198507	B3GALT6
16	49740001	49790000	0.0198507	TNFRSF4
16	49740001	49790000	0.0198507	UBE2J2
16	49740001	49790000	0.0198507	FAM132A
16	49740001	49790000	0.0198507	SDF4
22	16070001	16120000	0.0198474	TOPAZ1
22	16070001	16120000	0.0198474	TCAIM
12	35880001	35930000	0.019846	TBC1D4
6	65390001	65440000	0.0198419	GABRA2
1	149510001	149560000	0.0198335	DYRK1A
6	65400001	65450000	0.0198302	GABRA2
26	5930001	5980000	0.0198284	C26H10orf90
20	62120001	62170000	0.019828	CTNND2
5	84530001	84580000	0.0198265	SOX5
1	53080001	53130000	0.0198256	MYH15
10	49760001	49810000	0.019825	LOC108636854
10	49760001	49810000	0.019825	TCF12
17	35980001	36030000	0.0198166	IL21
17	35980001	36030000	0.0198166	LOC108637875
7	92950001	93000000	0.0198159	ADGRE1
10	66130001	66180000	0.0198159	LTK
10	66130001	66180000	0.0198159	LOC108636994
10	66130001	66180000	0.0198159	ITPKA
10	66130001	66180000	0.0198159	RTF1
26	17360001	17410000	0.0198158	CCDC186
19	27850001	27900000	0.0198134	MYH10
29	41280001	41330000	0.0198065	EML3
29	41280001	41330000	0.0198065	B3GAT3
29	41280001	41330000	0.0198065	INTS5
29	41280001	41330000	0.0198065	LBHD1
29	41280001	41330000	0.0198065	METTL12
29	41280001	41330000	0.0198065	GANAB
29	41280001	41330000	0.0198065	ROM1

19	19000001	19050000	0.0198059	LOC106503912
19	19000001	19050000	0.0198059	LOC108638256
17	36500001	36550000	0.0198032	SPATA5
2	36050001	36100000	0.0198028	ERBB4
11	61110001	61160000	0.0197923	EHBP1
22	19060001	19110000	0.0197873	GRM7
5	68810001	68860000	0.0197851	RIC8B
22	6260001	6310000	0.0197833	OSBPL10
4	68110001	68160000	0.0197822	TES
12	13230001	13280000	0.0197756	DZIP1
3	3360001	3410000	0.0197749	UBE2F
7	64430001	64480000	0.0197747	JADE2
22	17240001	17290000	0.0197693	SRGAP3
18	14070001	14120000	0.0197658	LOC106503074
24	43470001	43520000	0.0197585	PTPN2
11	20240001	20290000	0.0197516	RMDN2
26	18660001	18710000	0.0197469	VTI1A
4	96500001	96550000	0.0197446	AGMO
1	53150001	53200000	0.0197251	KIAA1524
12	13060001	13110000	0.0197191	UGGT2
8	38640001	38690000	0.0197082	MLANA
1	145980001	146030000	0.0196973	PCNT
18	15850001	15900000	0.0196893	SPG7
18	15850001	15900000	0.0196893	RPL13
22	38900001	38950000	0.0196874	FEZF2
10	66120001	66170000	0.0196846	LTK
10	66120001	66170000	0.0196846	LOC108636994
10	66120001	66170000	0.0196846	RPAP1
10	66120001	66170000	0.0196846	ITPKA
10	66120001	66170000	0.0196846	RTF1
16	49700001	49750000	0.0196835	CPTP
16	49700001	49750000	0.0196835	PUSL1
16	49700001	49750000	0.0196835	SCNN1D
16	49700001	49750000	0.0196835	CPSF3L
16	49700001	49750000	0.0196835	ACAP3
16	49700001	49750000	0.0196835	UBE2J2
7	44700001	44750000	0.0196769	GALNT10
13	9240001	9290000	0.0196624	MACROD2
7	68870001	68920000	0.0196513	LOC102172144
7	68870001	68920000	0.0196513	LOC102171865
22	23950001	24000000	0.0196486	LOC108633370
19	37370001	37420000	0.0196345	TTLL6
14	91140001	91190000	0.0196322	FAM135A
6	114410001	114460000	0.0196258	AFAP1
8	60420001	60470000	0.0196236	MELK
7	66950001	67000000	0.0196215	TMEM259
7	66950001	67000000	0.0196215	GRIN3B
7	66950001	67000000	0.0196215	ARID3A
7	66950001	67000000	0.0196215	WDR18
13	70440001	70490000	0.0196136	PTPRT
2	23100001	23150000	0.0196077	CUL3
24	42440001	42490000	0.019606	APCDD1
11	28100001	28150000	0.0195965	PRKCE
8	57640001	57690000	0.0195875	LOC106501875
14	13100001	13150000	0.0195859	C14H8orf37
4	70980001	71030000	0.0195855	NRCAM
7	58260001	58310000	0.0195836	LOC102189032
7	58260001	58310000	0.0195836	LOC102188767

7	58260001	58310000	0.0195836	LOC102188497
3	3350001	3400000	0.0195773	UBE2F
26	17370001	17420000	0.0195759	CCDC186
3	54180001	54230000	0.0195722	ZZZ3
10	66110001	66160000	0.0195707	LTK
10	66110001	66160000	0.0195707	LOC108636994
10	66110001	66160000	0.0195707	RPAP1
10	66110001	66160000	0.0195707	ITPKA
10	66110001	66160000	0.0195707	RTF1
13	7320001	7370000	0.0195667	SEL1L2
13	7320001	7370000	0.0195667	MACROD2
7	56160001	56210000	0.0195619	NR3C1
22	6250001	6300000	0.0195615	OSBPL10
10	94560001	94610000	0.0195605	ANKRD31
6	117510001	117560000	0.0195548	ATP5I
6	117510001	117560000	0.0195548	LOC106502243
6	117510001	117560000	0.0195548	PDE6B
7	71000001	71050000	0.0195544	COL23A1
5	82020001	82070000	0.0195538	ITPR2
26	28180001	28230000	0.0195484	AS3MT
26	28180001	28230000	0.0195484	CNNM2
22	14940001	14990000	0.0195458	LOC102173615
22	14940001	14990000	0.0195458	LOC102173976
12	11890001	11940000	0.0195417	MBNL2
26	47130001	47180000	0.0195387	LOC102181467
8	84750001	84800000	0.0195304	PHF2
24	42170001	42220000	0.0195285	RAB31
16	49730001	49780000	0.0195255	SCNN1D
16	49730001	49780000	0.0195255	B3GALT6
16	49730001	49780000	0.0195255	ACAP3
16	49730001	49780000	0.0195255	UBE2J2
16	49730001	49780000	0.0195255	FAM132A
16	49730001	49780000	0.0195255	SDF4
21	18940001	18990000	0.0195232	LOC108633276
21	18940001	18990000	0.0195232	LOC102177379
3	3400001	3450000	0.0195089	RAMP1
15	39800001	39850000	0.019505	SBF2
18	63080001	63130000	0.0194998	LOC102173498
18	63080001	63130000	0.0194998	LOC102174057
12	33840001	33890000	0.0194961	MYCBP2
