# Supplementary material for: Whole-genome selection signatures identified candidate genes associated with cashmere traits in Inner Mongolia cashmere goats
Source: Anim Biosci. 2025 Jul 11;38(12):2597–611. doi: 10.5713/ab.25.0252 (PMC12580777; doi:10.5713/ab.25.0252)
Supplement: Supplementary file 7 [file ab-25-0252-Supplementary-7.pdf]

Supplement 7. Detected and gene annotation of candidate regions by θπ ratios (LYCG VS HYCG)

Chr	Start	End	θπ ratios	Gene name
12	15260001	15310000	2.889173826	LOC102180583
6	116720001	116770000	2.030257177	MFSD10
6	116720001	116770000	2.030257177	LOC108636244
6	116720001	116770000	2.030257177	NOP14
6	116720001	116770000	2.030257177	ADD1
6	116720001	116770000	2.030257177	GRK4
6	116710001	116760000	1.972634006	MFSD10
6	116710001	116760000	1.972634006	LOC108636244
6	116710001	116760000	1.972634006	NOP14
6	116710001	116760000	1.972634006	ADD1
6	116700001	116750000	1.841001826	MFSD10
6	116700001	116750000	1.841001826	LOC108636244
6	116700001	116750000	1.841001826	NOP14
6	116700001	116750000	1.841001826	ADD1
6	116730001	116780000	1.818367727	MFSD10
6	116730001	116780000	1.818367727	LOC108636244
6	116730001	116780000	1.818367727	NOP14
6	116730001	116780000	1.818367727	ADD1
6	116730001	116780000	1.818367727	GRK4
6	116690001	116740000	1.688840946	ADD1
6	116740001	116790000	1.627862353	MFSD10
6	116740001	116790000	1.627862353	LOC108636244
6	116740001	116790000	1.627862353	NOP14
6	116740001	116790000	1.627862353	ADD1
6	116740001	116790000	1.627862353	GRK4
12	13840001	13890000	1.59080892	LOC108637251
12	13850001	13900000	1.59080892	LOC108637251
12	13860001	13910000	1.59080892	LOC108637251
12	13870001	13920000	1.59080892	LOC108637251
12	13880001	13930000	1.59080892	LOC108637252
12	13880001	13930000	1.59080892	LOC108637251
15	34090001	34140000	1.534076275	LOC102182894
15	34090001	34140000	1.534076275	LOC102175317
15	34090001	34140000	1.534076275	HBBC
15	34090001	34140000	1.534076275	LOC102182615
15	34090001	34140000	1.534076275	LOC102176442
15	34090001	34140000	1.534076275	LOC102174495
25	34940001	34990000	1.496682221	LOC102181832
25	34950001	35000000	1.485727205	LOC102187393
25	34950001	35000000	1.485727205	LOC102181832
15	32020001	32070000	1.483573659	NUP98
25	34960001	35010000	1.481064405	LOC102187393
25	34960001	35010000	1.481064405	LOC102181832
15	32000001	32050000	1.465039901	NUP98
15	31980001	32030000	1.464148443	NUP98
25	34930001	34980000	1.463456422	LOC102181832
10	80590001	80640000	1.461506411	RIPK3
10	80590001	80640000	1.461506411	LOC108637011
10	80590001	80640000	1.461506411	ADCY4
10	80590001	80640000	1.461506411	NFATC4
26	44170001	44220000	1.459348485	PRKG1
15	32010001	32060000	1.459045307	NUP98
6	116750001	116800000	1.454965168	NOP14
6	116750001	116800000	1.454965168	GRK4
1	113150001	113200000	1.454433291	ARHGEF26
15	31990001	32040000	1.445796509	NUP98

10	80600001	80650000	1.443778306	RIPK3
10	80600001	80650000	1.443778306	LOC108637011
10	80600001	80650000	1.443778306	NFATC4
17	66820001	66870000	1.437071572	TLR2
17	66820001	66870000	1.437071572	KIAA0922
25	34920001	34970000	1.435259161	LOC102181832
10	80580001	80630000	1.431349712	RIPK3
10	80580001	80630000	1.431349712	LOC108637011
10	80580001	80630000	1.431349712	LTB4R
10	80580001	80630000	1.431349712	ADCY4
10	80580001	80630000	1.431349712	NFATC4
15	31940001	31990000	1.424605387	LOC102178093
15	31940001	31990000	1.424605387	LOC102177821
15	31940001	31990000	1.424605387	LOC108637670
15	31940001	31990000	1.424605387	LOC102169116
15	31940001	31990000	1.424605387	ART1
15	31940001	31990000	1.424605387	NUP98
15	32030001	32080000	1.42407383	NUP98
6	95450001	95500000	1.423932301	C6H4orf22
25	34900001	34950000	1.423920755	LOC102181832
25	34910001	34960000	1.420182078	LOC102181832
6	116660001	116710000	1.4199526	ADD1
1	41010001	41060000	1.415334368	CLIC6
8	370001	420000	1.412809481	ANXA10
15	31970001	32020000	1.41062794	LOC102177821
15	31970001	32020000	1.41062794	LOC108637670
15	31970001	32020000	1.41062794	ART1
15	31970001	32020000	1.41062794	NUP98
6	95460001	95510000	1.409945679	C6H4orf22
4	87870001	87920000	1.407592547	LOC102171502
6	95540001	95590000	1.405582125	C6H4orf22
8	360001	410000	1.404940811	ANXA10
26	44160001	44210000	1.403237445	PRKG1
6	116650001	116700000	1.402476103	ADD1
1	52120001	52170000	1.401553834	BBX
15	31960001	32010000	1.400562445	LOC102177821
15	31960001	32010000	1.400562445	LOC108637670
15	31960001	32010000	1.400562445	ART1
15	31960001	32010000	1.400562445	NUP98
6	95530001	95580000	1.399961111	C6H4orf22
6	95550001	95600000	1.399587012	C6H4orf22
15	31930001	31980000	1.399236044	LOC102178093
15	31930001	31980000	1.399236044	LOC102177821
15	31930001	31980000	1.399236044	LOC108637670
15	31930001	31980000	1.399236044	LOC102169399
15	31930001	31980000	1.399236044	LOC102169116
15	31930001	31980000	1.399236044	ART1
17	66830001	66880000	1.395952799	TLR2
17	66830001	66880000	1.395952799	RNF175
15	34100001	34150000	1.393117774	LOC102175317
15	34100001	34150000	1.393117774	HBBC
15	34100001	34150000	1.393117774	LOC102182615
15	34100001	34150000	1.393117774	LOC102176442
15	34100001	34150000	1.393117774	LOC102174495
6	95520001	95570000	1.391475149	C6H4orf22
4	87860001	87910000	1.391404482	LOC102171502
3	25680001	25730000	1.390069359	C3H1orf185
15	31950001	32000000	1.385185792	LOC102178093

15	31950001	32000000	1.385185792	LOC102177821
15	31950001	32000000	1.385185792	LOC108637670
15	31950001	32000000	1.385185792	LOC102169116
15	31950001	32000000	1.385185792	ART1
15	31950001	32000000	1.385185792	NUP98
6	95470001	95520000	1.383709523	C6H4orf22
11	105400001	105450000	1.381262208	CACNA1B
2	115530001	115580000	1.379954136	HOXD9
2	115530001	115580000	1.379954136	HOXD8
2	115530001	115580000	1.379954136	LOC108638283
2	115530001	115580000	1.379954136	MIR10B
2	115530001	115580000	1.379954136	HOXD3
6	95510001	95560000	1.377921753	C6H4orf22
12	60120001	60170000	1.376584002	TRNAC-GCA-168
12	60120001	60170000	1.376584002	LOC102178917
8	350001	400000	1.375007556	ANXA10
6	116680001	116730000	1.371965377	ADD1
8	380001	430000	1.368773131	ANXA10
25	34890001	34940000	1.365353214	LOC102181832
11	14110001	14160000	1.364310195	LOC108637103
15	32040001	32090000	1.362367248	PGAP2
15	32040001	32090000	1.362367248	NUP98
11	105410001	105460000	1.362219649	CACNA1B
12	60130001	60180000	1.361458848	TRNAC-GCA-168
12	60130001	60180000	1.361458848	LOC102178917
5	23340001	23390000	1.36145101	CRADD
11	105390001	105440000	1.360908997	CACNA1B
1	113160001	113210000	1.3588461	ARHGEF26
3	25660001	25710000	1.354159703	C3H1orf185
6	95670001	95720000	1.353420158	C6H4orf22
6	95480001	95530000	1.353339938	C6H4orf22
6	116760001	116810000	1.351890959	NOP14
6	116760001	116810000	1.351890959	GRK4
25	34970001	35020000	1.351522445	LOC102187393
25	34970001	35020000	1.351522445	LOC102181832
1	45140001	45190000	1.349950461	ABI3BP
8	390001	440000	1.349530542	ANXA10
1	41020001	41070000	1.348934893	CLIC6
6	95660001	95710000	1.348047053	C6H4orf22
11	105420001	105470000	1.346707344	CACNA1B
1	157310001	157360000	1.344820767	LOC102184668
12	60110001	60160000	1.344170246	TRNAC-GCA-168
12	60110001	60160000	1.344170246	LOC102178917
11	14120001	14170000	1.34245779	LOC108637103
11	14120001	14170000	1.34245779	SRD5A2
6	95490001	95540000	1.340858931	C6H4orf22
26	44180001	44230000	1.340435262	PRKG1
23	19550001	19600000	1.339569723	ZSCAN31
23	19550001	19600000	1.339569723	ZSCAN12
4	87880001	87930000	1.339323967	LOC102171502
15	32050001	32100000	1.338894801	PGAP2
15	32050001	32100000	1.338894801	NUP98
15	32050001	32100000	1.338894801	RHOG
12	60140001	60190000	1.338556018	LOC102178917
13	37810001	37860000	1.337376945	ZNF133
15	31920001	31970000	1.337054561	LOC102178093
15	31920001	31970000	1.337054561	LOC102169399
15	31920001	31970000	1.337054561	LOC102169116

15	31920001	31970000	1.337054561	ART1
13	63260001	63310000	1.335730961	AHCY
7	27600001	27650000	1.335599319	XRCC4
7	27600001	27650000	1.335599319	TMEM167A
13	37820001	37870000	1.333612779	ZNF133
6	95500001	95550000	1.330724125	C6H4orf22
3	25670001	25720000	1.330196744	C3H1orf185
16	43230001	43280000	1.328765759	RERE
6	116670001	116720000	1.328016518	ADD1
24	43900001	43950000	1.32791945	MC2R
6	95650001	95700000	1.327209487	C6H4orf22
6	116770001	116820000	1.326320292	GRK4
5	84530001	84580000	1.326049171	SOX5
1	113140001	113190000	1.325065041	ARHGEF26
15	34080001	34130000	1.323624869	LOC102175600
15	34080001	34130000	1.323624869	LOC102182894
15	34080001	34130000	1.323624869	LOC102175317
15	34080001	34130000	1.323624869	HBBC
15	34080001	34130000	1.323624869	LOC102182615
24	43890001	43940000	1.321580814	MC2R
12	13700001	13750000	1.320902884	LOC108637249
6	95440001	95490000	1.320824922	C6H4orf22
6	95440001	95490000	1.320824922	FGF5
11	105430001	105480000	1.318281202	CACNA1B
10	30510001	30560000	1.318123593	C10H14orf39
2	115540001	115590000	1.31612566	LOC108638283
2	115540001	115590000	1.31612566	MIR10B
2	115540001	115590000	1.31612566	HOXD3
8	39250001	39300000	1.316027464	JAK2
1	112950001	113000000	1.315661488	GPR149
1	112950001	113000000	1.315661488	DHX36
5	23350001	23400000	1.315148568	CRADD
24	43880001	43930000	1.314981464	MC2R
18	54110001	54160000	1.314453933	PPM1N
18	54110001	54160000	1.314453933	LOC108638001
18	54110001	54160000	1.314453933	RTN2
18	54110001	54160000	1.314453933	VASP
18	54110001	54160000	1.314453933	FOSB
12	15220001	15270000	1.313498119	LOC102181111
12	15230001	15280000	1.313498119	LOC102181111
12	15240001	15290000	1.313498119	LOC102181111
12	15240001	15290000	1.313498119	LOC102180583
12	15250001	15300000	1.313498119	LOC102180583
16	43240001	43290000	1.313016158	RERE
16	43220001	43270000	1.312820328	RERE
1	45130001	45180000	1.311699996	ABI3BP
7	27590001	27640000	1.310783623	XRCC4
5	84540001	84590000	1.310561467	SOX5
13	63250001	63300000	1.309830089	AHCY
18	54120001	54170000	1.307197835	PPM1N
18	54120001	54170000	1.307197835	LOC108638001
18	54120001	54170000	1.307197835	RTN2
18	54120001	54170000	1.307197835	VASP
18	54120001	54170000	1.307197835	FOSB
4	5040001	5090000	1.306978118	KMT2C
15	32060001	32110000	1.305164471	PGAP2
15	32060001	32110000	1.305164471	NUP98
15	32060001	32110000	1.305164471	RHOG

18	56300001	56350000	1.304876203	DBP
18	56300001	56350000	1.304876203	TRNAE-UUC-76
18	56300001	56350000	1.304876203	NTN5
18	56300001	56350000	1.304876203	LOC102178850
18	56300001	56350000	1.304876203	LOC102185028
18	56300001	56350000	1.304876203	CA11
1	52110001	52160000	1.304708969	BBX
13	37830001	37880000	1.30362784	LOC108637388
13	37830001	37880000	1.30362784	ZNF133
13	37830001	37880000	1.30362784	DZANK1
13	42970001	43020000	1.303381538	LOC102189109
24	43910001	43960000	1.302177482	MC2R
9	56780001	56830000	1.301256408	ARG1
17	66810001	66860000	1.300705516	TRNAC-GCA-206
17	66810001	66860000	1.300705516	TLR2
17	66810001	66860000	1.300705516	KIAA0922
13	2300001	2350000	1.299908013	PAK5
18	56310001	56360000	1.299583611	TRNAE-UUC-76
18	56310001	56360000	1.299583611	NTN5
18	56310001	56360000	1.299583611	LOC102178850
18	56310001	56360000	1.299583611	LOC102185028
18	56310001	56360000	1.299583611	CA11
6	95560001	95610000	1.299017735	C6H4orf22
13	37850001	37900000	1.298993429	LOC108637388
13	37850001	37900000	1.298993429	LOC102189769
13	37850001	37900000	1.298993429	ZNF133
13	37850001	37900000	1.298993429	DZANK1
1	45150001	45200000	1.298808709	ABI3BP
18	56290001	56340000	1.298308109	RPL18
18	56290001	56340000	1.298308109	DBP
18	56290001	56340000	1.298308109	TRNAE-UUC-76
18	56290001	56340000	1.298308109	NTN5
18	56290001	56340000	1.298308109	LOC102178850
18	56290001	56340000	1.298308109	SPHK2
18	56290001	56340000	1.298308109	CA11
7	37390001	37440000	1.29778846	TRNAS-GGA-38
7	37390001	37440000	1.29778846	GABRB2
5	110380001	110430000	1.297648762	TNRC6B
1	112940001	112990000	1.297083235	TRNAE-UUC-2
1	112940001	112990000	1.297083235	GPR149
1	112940001	112990000	1.297083235	DHX36
5	110370001	110420000	1.296794209	TNRC6B
3	95340001	95390000	1.296097583	SPAG17
13	37800001	37850000	1.295555959	KAT14
13	2290001	2340000	1.295149373	PAK5
4	7680001	7730000	1.294749304	LOC106501766
7	27580001	27630000	1.294510951	XRCC4
18	56320001	56370000	1.293869779	NTN5
18	56320001	56370000	1.293869779	LOC102178850
18	56320001	56370000	1.293869779	LOC102185028
25	34880001	34930000	1.293860439	LOC102181832
1	51290001	51340000	1.293752214	LOC108636831
4	5050001	5100000	1.293423685	KMT2C
8	100900001	100950000	1.293217509	C8H9orf84
2	110640001	110690000	1.291834195	GAD1
2	110640001	110690000	1.291834195	SP5
6	116640001	116690000	1.291374395	SH3BP2
6	116640001	116690000	1.291374395	ADD1

29	420001	470000	1.291360242	LOC106503712
29	580001	630000	1.291349593	MIR2404
29	580001	630000	1.291349593	C29H11orf54
29	580001	630000	1.291349593	TAF1D
29	580001	630000	1.291349593	CEP295
18	56330001	56380000	1.291305687	LOC102178850
18	56330001	56380000	1.291305687	LOC102185028
18	56330001	56380000	1.291305687	MAMSTR
2	110630001	110680000	1.29054178	GAD1
2	110630001	110680000	1.29054178	SP5
29	590001	640000	1.290231281	MIR2404
29	590001	640000	1.290231281	TAF1D
29	590001	640000	1.290231281	CEP295
23	19520001	19570000	1.289830923	ZSCAN31
23	19520001	19570000	1.289830923	PGBD1
26	34400001	34450000	1.289309316	TCTN3
26	34400001	34450000	1.289309316	ENTPD1
8	100960001	101010000	1.288951834	UGCG
8	39260001	39310000	1.288882652	JAK2
3	25650001	25700000	1.288633688	C3H1orf185
4	5030001	5080000	1.287641329	KMT2C
11	105380001	105430000	1.286584545	CACNA1B
10	94660001	94710000	1.286281269	LOC108636845
10	94660001	94710000	1.286281269	GCNT4
10	94660001	94710000	1.286281269	ANKRD31
13	37840001	37890000	1.285455241	LOC108637388
13	37840001	37890000	1.285455241	LOC102189769
13	37840001	37890000	1.285455241	ZNF133
13	37840001	37890000	1.285455241	DZANK1
15	6220001	6270000	1.284868677	CKAP5
11	105370001	105420000	1.284851275	CACNA1B
16	59810001	59860000	1.284806345	CEP350
14	12700001	12750000	1.284725693	CCNE2
14	12700001	12750000	1.284725693	INTS8
16	43260001	43310000	1.28450993	RERE
8	39240001	39290000	1.284393946	JAK2
5	46900001	46950000	1.284051624	IRAK3
13	37790001	37840000	1.284012348	KAT14
14	16040001	16090000	1.283837746	RPL30
14	16040001	16090000	1.283837746	ERICH5
14	16040001	16090000	1.283837746	MATN2
26	44150001	44200000	1.283830565	PRKG1
1	157300001	157350000	1.283739238	LOC102184668
12	13140001	13190000	1.282428788	DNAJC3
12	13140001	13190000	1.282428788	UGGT2
3	95350001	95400000	1.281743203	SPAG17
26	47150001	47200000	1.281645198	LOC102181467
15	34110001	34160000	1.281293211	HBBC
15	34110001	34160000	1.281293211	LOC102182615
15	34110001	34160000	1.281293211	LOC102176442
15	34110001	34160000	1.281293211	LOC102174495
13	63270001	63320000	1.281165318	AHCY
16	43210001	43260000	1.279351981	RERE
16	43250001	43300000	1.279153514	RERE
4	87850001	87900000	1.279064808	LOC102171502
26	34410001	34460000	1.278838238	TCTN3
3	95330001	95380000	1.276023298	SPAG17
14	16060001	16110000	1.275992049	RIDA

14	16060001	16110000	1.275992049	ERICH5
14	16060001	16110000	1.275992049	POP1
18	36250001	36300000	1.275752668	TPPP3
18	36250001	36300000	1.275752668	LRRC36
18	36250001	36300000	1.275752668	ZDHHC1
9	1890001	1940000	1.2754524	COL12A1
5	46910001	46960000	1.275405175	IRAK3
3	95430001	95480000	1.275352412	SPAG17
16	43200001	43250000	1.275140906	RERE
26	34390001	34440000	1.274956065	TCTN3
26	34390001	34440000	1.274956065	ENTPD1
6	116790001	116840000	1.274770159	GRK4
6	116790001	116840000	1.274770159	HTT
13	42980001	43030000	1.2743313	LOC102189109
15	6210001	6260000	1.27423336	CKAP5
12	13680001	13730000	1.273044705	LOC108637249
12	13690001	13740000	1.273044705	LOC108637249
18	54130001	54180000	1.272997805	PPM1N
18	54130001	54180000	1.272997805	RTN2
18	54130001	54180000	1.272997805	VASP
18	54130001	54180000	1.272997805	OPA3
18	54130001	54180000	1.272997805	FOSB
18	36240001	36290000	1.272805714	TPPP3
18	36240001	36290000	1.272805714	LRRC36
18	36240001	36290000	1.272805714	ZDHHC1
10	94560001	94610000	1.271965052	ANKRD31
6	95680001	95730000	1.271874002	C6H4orf22
2	115520001	115570000	1.271868391	HOXD10
2	115520001	115570000	1.271868391	HOXD9
2	115520001	115570000	1.271868391	HOXD8
2	115520001	115570000	1.271868391	LOC108638283
2	115520001	115570000	1.271868391	MIR10B
2	115520001	115570000	1.271868391	HOXD3
13	42960001	43010000	1.271335148	LOC102187204
13	42960001	43010000	1.271335148	LOC102189109
29	430001	480000	1.270967435	LOC106503712
29	430001	480000	1.270967435	VSTM5
21	15230001	15280000	1.270791396	SV2B
18	56970001	57020000	1.270740789	FCGRT
18	56970001	57020000	1.270740789	PRRG2
18	56970001	57020000	1.270740789	RCN3
18	56970001	57020000	1.270740789	NOSIP
18	56970001	57020000	1.270740789	PRR12
10	30520001	30570000	1.270627118	C10H14orf39
8	400001	450000	1.270262458	ANXA10
12	13710001	13760000	1.270247894	LOC108637249
12	60150001	60200000	1.270053237	LOC102178917
14	16070001	16120000	1.269835326	RIDA
14	16070001	16120000	1.269835326	ERICH5
14	16070001	16120000	1.269835326	POP1
23	19540001	19590000	1.269729093	ZSCAN31
23	19540001	19590000	1.269729093	ZSCAN12
23	19540001	19590000	1.269729093	PGBD1
21	15240001	15290000	1.269635232	SV2B
3	95360001	95410000	1.269136659	SPAG17
6	95430001	95480000	1.269129368	FGF5
14	16000001	16050000	1.269081135	RPL30
14	16000001	16050000	1.269081135	MATN2

1	157270001	157320000	1.268894844	LOC108636388
1	157270001	157320000	1.268894844	LOC102184668
5	110360001	110410000	1.268628645	TNRC6B
13	37780001	37830000	1.268547494	PET117
13	37780001	37830000	1.268547494	KAT14
29	570001	620000	1.268233517	MIR2404
29	570001	620000	1.268233517	C29H11orf54
29	570001	620000	1.268233517	TAF1D
29	570001	620000	1.268233517	CEP295
1	96710001	96760000	1.26772669	PHC3
1	51280001	51330000	1.267713001	LOC108636831
21	64490001	64540000	1.266923987	MIR345
21	64490001	64540000	1.266923987	SLC25A47
21	64490001	64540000	1.266923987	WARS
21	64490001	64540000	1.266923987	SLC25A29
4	7670001	7720000	1.266893761	LOC106501766
1	93980001	94030000	1.266862817	SPATA16
12	63190001	63240000	1.266652409	FREM2
12	60100001	60150000	1.266387254	TRNAC-GCA-168
12	60100001	60150000	1.266387254	LOC102178917
2	110620001	110670000	1.266202918	SP5
25	18070001	18120000	1.265934615	ACSM1
14	61010001	61060000	1.265873234	PCMTD1
12	15210001	15260000	1.265588692	LOC102181111
5	33470001	33520000	1.265327296	SLC38A1
10	94650001	94700000	1.264746783	ANKRD31
1	52130001	52180000	1.263697767	BBX
29	560001	610000	1.263451571	MIR2404
29	560001	610000	1.263451571	C29H11orf54
29	560001	610000	1.263451571	TAF1D
29	560001	610000	1.263451571	CEP295
16	59820001	59870000	1.26292425	CEP350
4	5060001	5110000	1.262324998	KMT2C
4	7660001	7710000	1.261685365	LOC102186385
4	7660001	7710000	1.261685365	LOC106501766
16	43270001	43320000	1.2615693	RERE
14	16050001	16100000	1.261292899	RPL30
14	16050001	16100000	1.261292899	RIDA
14	16050001	16100000	1.261292899	ERICH5
25	210001	260000	1.26079116	RGS11
25	210001	260000	1.26079116	ARHGDIG
25	210001	260000	1.26079116	FAM234A
25	210001	260000	1.26079116	PDIA2
25	210001	260000	1.26079116	AXIN1
12	63210001	63260000	1.260511385	FREM2
24	50750001	50800000	1.260199866	SMAD4
14	16030001	16080000	1.26006763	RPL30
14	16030001	16080000	1.26006763	ERICH5
14	16030001	16080000	1.26006763	MATN2
22	16220001	16270000	1.259941757	LOC102177570
2	110610001	110660000	1.259873305	SP5
22	16230001	16280000	1.259789199	LOC102177570
16	45050001	45100000	1.259779942	CAMTA1
3	68940001	68990000	1.259359638	TGFBR3
11	14090001	14140000	1.259082882	XDH
5	23330001	23380000	1.25893927	CRADD
5	14540001	14590000	1.258625977	LRRIQ1
13	37860001	37910000	1.258619055	LOC108637388

13	37860001	37910000	1.258619055	LOC102189769
13	37860001	37910000	1.258619055	ZNF133
13	37860001	37910000	1.258619055	DZANK1
11	48950001	49000000	1.258511713	VAMP8
11	48950001	49000000	1.258511713	GGCX
11	48950001	49000000	1.258511713	MAT2A
1	118540001	118590000	1.258375197	CP
16	45040001	45090000	1.258281262	CAMTA1
15	6230001	6280000	1.258188934	TRNAE-CUC-23
15	6230001	6280000	1.258188934	CKAP5
1	93970001	94020000	1.257989977	SPATA16
5	33480001	33530000	1.25776993	SLC38A1
14	12710001	12760000	1.257762279	CCNE2
14	12710001	12760000	1.257762279	INTS8
25	200001	250000	1.257750939	RGS11
25	200001	250000	1.257750939	ARHGDIG
25	200001	250000	1.257750939	LUC7L
25	200001	250000	1.257750939	FAM234A
25	200001	250000	1.257750939	PDIA2
25	200001	250000	1.257750939	AXIN1
15	6240001	6290000	1.257465041	TRNAE-CUC-23
15	6240001	6290000	1.257465041	CKAP5
12	63200001	63250000	1.257432501	FREM2
6	116780001	116830000	1.257383388	GRK4
6	116780001	116830000	1.257383388	HTT
1	112960001	113010000	1.257091476	DHX36
3	95320001	95370000	1.256403031	SPAG17
23	19510001	19560000	1.255925969	ZSCAN31
23	19510001	19560000	1.255925969	ZSCAN26
23	19510001	19560000	1.255925969	PGBD1
19	5310001	5360000	1.255867983	LOC102176553
19	5310001	5360000	1.255867983	MMD
14	12690001	12740000	1.255825563	INTS8
10	94540001	94590000	1.255795772	ANKRD31
11	28140001	28190000	1.255540073	PRKCE
29	410001	460000	1.255509016	LOC106503712
4	5070001	5120000	1.255376209	KMT2C
15	32360001	32410000	1.255229325	LOC102177547
15	32360001	32410000	1.255229325	LOC102177261
15	32360001	32410000	1.255229325	TRNAG-CCC-64
4	75740001	75790000	1.25445723	PMPCB
4	75740001	75790000	1.25445723	DNAJC2
21	64480001	64530000	1.254449223	MIR345
21	64480001	64530000	1.254449223	SLC25A47
21	64480001	64530000	1.254449223	WARS
21	64480001	64530000	1.254449223	SLC25A29
2	7130001	7180000	1.254214493	MYOM3
2	7130001	7180000	1.254214493	IL22RA1
11	14050001	14100000	1.253853914	XDH
15	6250001	6300000	1.253638354	TRNAE-CUC-23
15	6250001	6300000	1.253638354	CKAP5
23	19560001	19610000	1.253550595	ZSCAN31
23	19560001	19610000	1.253550595	ZSCAN12
7	37400001	37450000	1.25285844	TRNAS-GGA-38
7	37400001	37450000	1.25285844	GABRB2
1	51270001	51320000	1.252781241	LOC108636831
2	88140001	88190000	1.252453003	LOC106502907
11	74660001	74710000	1.252246307	PFN4

11	74660001	74710000	1.252246307	TP53I3
11	74660001	74710000	1.252246307	SF3B6
11	74660001	74710000	1.252246307	FKBP1B
11	74660001	74710000	1.252246307	FAM228B
18	56960001	57010000	1.252221934	RPS11
18	56960001	57010000	1.252221934	MIR150
18	56960001	57010000	1.252221934	FCGRT
18	56960001	57010000	1.252221934	PRRG2
18	56960001	57010000	1.252221934	RCN3
18	56960001	57010000	1.252221934	NOSIP
15	6200001	6250000	1.252186967	LRP4
15	6200001	6250000	1.252186967	CKAP5
24	43860001	43910000	1.251965346	MC5R
24	43860001	43910000	1.251965346	MC2R
16	45080001	45130000	1.251051209	CAMTA1
10	30500001	30550000	1.250923603	C10H14orf39
6	116630001	116680000	1.250796904	SH3BP2
6	116630001	116680000	1.250796904	ADD1
5	31320001	31370000	1.250604889	LOC108636097
26	45900001	45950000	1.2498435	PCDH15
3	95440001	95490000	1.24974838	SPAG17
18	36260001	36310000	1.249622355	TPPP3
18	36260001	36310000	1.249622355	LRRC36
18	36260001	36310000	1.249622355	ZDHHC1
18	36260001	36310000	1.249622355	HSD11B2
11	14100001	14150000	1.249494025	LOC108637103
4	5080001	5130000	1.249408074	KMT2C
5	14530001	14580000	1.249271369	TSPAN19
5	14530001	14580000	1.249271369	LRRIQ1
21	57090001	57140000	1.249105344	ITPK1
22	16210001	16260000	1.248951562	LOC102177570
25	18080001	18130000	1.248931776	ACSM1
1	51260001	51310000	1.248413595	LOC108636831
23	19530001	19580000	1.248327823	ZSCAN31
23	19530001	19580000	1.248327823	PGBD1
14	16010001	16060000	1.248124869	RPL30
14	16010001	16060000	1.248124869	MATN2
19	20520001	20570000	1.24782756	TAOK1
26	34420001	34470000	1.247320254	TCTN3
26	34420001	34470000	1.247320254	ALDH18A1
1	96720001	96770000	1.247274053	PHC3
2	18420001	18470000	1.247100313	PID1
3	95310001	95360000	1.246996738	SPAG17
22	16240001	16290000	1.246585251	ZNF502
22	16240001	16290000	1.246585251	LOC102177570
11	48960001	49010000	1.246506745	GGCX
11	48960001	49010000	1.246506745	MAT2A
5	34460001	34510000	1.246179349	TRNAS-GGA-29
5	34460001	34510000	1.246179349	ANO6
1	110510001	110560000	1.246090729	TIPARP
4	75730001	75780000	1.245939806	PSMC2
4	75730001	75780000	1.245939806	PMPCB
4	75730001	75780000	1.245939806	DNAJC2
4	28480001	28530000	1.245890485	ZNF800
2	7120001	7170000	1.245418923	MYOM3
2	7120001	7170000	1.245418923	IL22RA1
29	390001	440000	1.245297198	LOC106503712
3	68950001	69000000	1.244546954	TGFBR3

13	2280001	2330000	1.244224378	PAK5
22	11550001	11600000	1.244162775	OXSR1
16	45070001	45120000	1.243990598	CAMTA1
10	87830001	87880000	1.243906058	LCTL
10	87830001	87880000	1.243906058	ZWILCH
16	59800001	59850000	1.243733624	CEP350
18	56350001	56400000	1.243714309	LOC102185028
18	56350001	56400000	1.243714309	IZUMO1
18	56350001	56400000	1.243714309	FUT1
18	56350001	56400000	1.243714309	MAMSTR
5	46930001	46980000	1.24364226	TMBIM4
5	46930001	46980000	1.24364226	IRAK3
19	20580001	20630000	1.24327062	TAOK1
11	74650001	74700000	1.242952896	PFN4
11	74650001	74700000	1.242952896	TP53I3
11	74650001	74700000	1.242952896	SF3B6
11	74650001	74700000	1.242952896	FKBP1B
11	74650001	74700000	1.242952896	FAM228B
3	41140001	41190000	1.242913264	LEPR
16	53640001	53690000	1.242902198	KLHL20
1	51250001	51300000	1.242887066	LOC108636831
4	28490001	28540000	1.242667157	ZNF800
5	46980001	47030000	1.242301805	LLPH
5	46980001	47030000	1.242301805	TMBIM4
3	95370001	95420000	1.242177984	SPAG17
26	47160001	47210000	1.241705276	LOC102181467
10	34190001	34240000	1.241648068	TMEM260
10	34210001	34260000	1.241299966	TMEM260
10	34200001	34250000	1.240237251	TMEM260
2	83910001	83960000	1.24010087	GTDC1
25	18440001	18490000	1.239945397	LOC102184112
10	34220001	34270000	1.239725255	TMEM260
2	115510001	115560000	1.239606007	HOXD11
2	115510001	115560000	1.239606007	HOXD10
2	115510001	115560000	1.239606007	HOXD9
2	115510001	115560000	1.239606007	HOXD8
2	115510001	115560000	1.239606007	LOC108638283
2	115510001	115560000	1.239606007	MIR10B
2	115510001	115560000	1.239606007	HOXD3
5	46920001	46970000	1.239515947	IRAK3
1	40970001	41020000	1.239434245	LOC108636218
1	40970001	41020000	1.239434245	LOC106504018
25	220001	270000	1.239230825	RGS11
25	220001	270000	1.239230825	ARHGDIG
25	220001	270000	1.239230825	FAM234A
25	220001	270000	1.239230825	PDIA2
25	220001	270000	1.239230825	AXIN1
7	91560001	91610000	1.239102745	PTPRS
22	11540001	11590000	1.238883895	OXSR1
3	41150001	41200000	1.238869454	LEPR
15	31910001	31960000	1.238408558	LOC102178093
15	31910001	31960000	1.238408558	LOC102169978
15	31910001	31960000	1.238408558	ART5
15	31910001	31960000	1.238408558	LOC102169399
15	31910001	31960000	1.238408558	LOC102169116
25	34870001	34920000	1.238134341	LOC102181832
29	450001	500000	1.238075357	VSTM5
5	23360001	23410000	1.238045976	CRADD

10	94640001	94690000	1.237889475	ANKRD31
11	14130001	14180000	1.237399019	LOC108637103
11	14130001	14180000	1.237399019	SRD5A2
24	62240001	62290000	1.237225249	LOC102181552
24	62240001	62290000	1.237225249	LOC102181826
12	14310001	14360000	1.23717769	LOC108637254
1	157320001	157370000	1.237083043	LOC102184668
13	42990001	43040000	1.23680256	LOC102189109
6	67510001	67560000	1.23661509	TEC
4	12280001	12330000	1.236515957	TRNAS-GGA-21
4	12280001	12330000	1.236515957	TRNAC-GCA-71
19	20540001	20590000	1.236493994	TAOK1
2	6640001	6690000	1.236184046	ID3
5	33260001	33310000	1.235690616	LOC102190664
8	340001	390000	1.235681226	ANXA10
19	20530001	20580000	1.235671512	TAOK1
16	53630001	53680000	1.235563735	KLHL20
22	28830001	28880000	1.235538114	SHQ1
11	14080001	14130000	1.235183021	XDH
14	16020001	16070000	1.2350561	RPL30
14	16020001	16070000	1.2350561	ERICH5
14	16020001	16070000	1.2350561	MATN2
2	88130001	88180000	1.23473989	LOC106502907
2	88130001	88180000	1.23473989	ORC4
1	118530001	118580000	1.234660085	CP
22	16260001	16310000	1.234574471	ZNF502
22	16260001	16310000	1.234574471	ZNF501
22	16260001	16310000	1.234574471	LOC102177570
16	53660001	53710000	1.234434461	KLHL20
16	53660001	53710000	1.234434461	CENPL
14	12680001	12730000	1.234144835	INTS8
19	20590001	20640000	1.234137199	ABHD15
19	20590001	20640000	1.234137199	TAOK1
16	45060001	45110000	1.234062264	CAMTA1
1	96700001	96750000	1.233518357	PRKCI
1	96700001	96750000	1.233518357	PHC3
5	84520001	84570000	1.233306418	SOX5
16	43280001	43330000	1.233014654	RERE
20	31360001	31410000	1.232994006	CCL28
15	6260001	6310000	1.232687296	TRNAE-CUC-23
15	6260001	6310000	1.232687296	F2
15	6260001	6310000	1.232687296	CKAP5
1	118550001	118600000	1.232239828	CP
1	118550001	118600000	1.232239828	HPS3
8	64250001	64300000	1.232051829	LOC106502417
10	94550001	94600000	1.231964259	ANKRD31
10	30910001	30960000	1.231833187	LOC102176425
26	47170001	47220000	1.231633599	LOC102181467
16	59830001	59880000	1.231594881	CEP350
13	65310001	65360000	1.231273058	NDRG3
10	26600001	26650000	1.231102531	LOC102175585
11	105360001	105410000	1.230985368	RXRA
11	105360001	105410000	1.230985368	CACNA1B
16	43190001	43240000	1.230928357	RERE
16	45090001	45140000	1.23084515	CAMTA1
24	62230001	62280000	1.230822975	LOC102181552
4	7640001	7690000	1.230618687	LOC102186385
2	83900001	83950000	1.230303972	GTDC1

1	27970001	28020000	1.230219557	GBE1
2	83890001	83940000	1.230112229	GTDC1
4	7650001	7700000	1.230074389	LOC102186385
4	7650001	7700000	1.230074389	LOC106501766
6	85650001	85700000	1.229547877	LOC102168522
7	70610001	70660000	1.229518728	LOC102170224
7	70610001	70660000	1.229518728	MGAT1
6	85660001	85710000	1.229483487	LOC102168522
25	230001	280000	1.229406102	RGS11
25	230001	280000	1.229406102	ARHGDIG
25	230001	280000	1.229406102	FAM234A
25	230001	280000	1.229406102	PDIA2
25	230001	280000	1.229406102	AXIN1
24	50740001	50790000	1.229300592	SMAD4
4	14180001	14230000	1.228798431	LOC102183208
4	14180001	14230000	1.228798431	LOC108635908
4	14180001	14230000	1.228798431	LOC102181470
22	28840001	28890000	1.228728376	SHQ1
3	68930001	68980000	1.228638984	TGFBR3
4	28500001	28550000	1.228606586	ZNF800
8	39230001	39280000	1.228554517	JAK2
14	12670001	12720000	1.228105501	DPY19L4
14	12670001	12720000	1.228105501	INTS8
2	114250001	114300000	1.227867543	CHN1
26	34480001	34530000	1.227832226	ALDH18A1
28	120001	170000	1.227823081	LOC108634156
28	120001	170000	1.227823081	TFAM
16	45110001	45160000	1.227687185	CAMTA1
25	18480001	18530000	1.227687172	DCUN1D3
25	18480001	18530000	1.227687172	LOC102184112
29	600001	650000	1.227046422	CEP295
24	43870001	43920000	1.227006362	MC5R
24	43870001	43920000	1.227006362	MC2R
4	75750001	75800000	1.226902848	PMPCB
4	75750001	75800000	1.226902848	DNAJC2
9	1900001	1950000	1.226733084	COL12A1
23	19580001	19630000	1.226727214	ZSCAN12
23	19580001	19630000	1.226727214	ZSCAN23
23	19580001	19630000	1.226727214	TRNAF-GAA-18
23	19580001	19630000	1.226727214	LOC102168401
15	6190001	6240000	1.226413281	LRP4
15	6190001	6240000	1.226413281	CKAP5
9	82470001	82520000	1.226281548	TRNAE-UUC-39
9	82470001	82520000	1.226281548	EZR
18	36270001	36320000	1.225597819	TPPP3
18	36270001	36320000	1.225597819	ZDHHC1
18	36270001	36320000	1.225597819	ATP6V0D1
18	36270001	36320000	1.225597819	HSD11B2
15	32070001	32120000	1.225248029	PGAP2
15	32070001	32120000	1.225248029	NUP98
15	32070001	32120000	1.225248029	RHOG
15	32070001	32120000	1.225248029	STIM1
29	440001	490000	1.225232154	LOC106503712
29	440001	490000	1.225232154	VSTM5
11	105440001	105490000	1.22518561	CACNA1B
9	1880001	1930000	1.225127218	COL12A1
26	34470001	34520000	1.225000521	ALDH18A1
22	16250001	16300000	1.224965948	ZNF502

22	16250001	16300000	1.224965948	LOC102177570
24	62150001	62200000	1.224725422	SERPINB7
11	48940001	48990000	1.224518831	VAMP8
11	48940001	48990000	1.224518831	RNF181
11	48940001	48990000	1.224518831	VAMP5
11	48940001	48990000	1.224518831	GGCX
11	48940001	48990000	1.224518831	MAT2A
4	75600001	75650000	1.224240038	RELN
19	20550001	20600000	1.224182291	TAOK1
4	72970001	73020000	1.224150483	NAMPT
1	95400001	95450000	1.223954933	PLD1
20	23250001	23300000	1.223627105	IL6ST
29	550001	600000	1.223322786	MIR2404
29	550001	600000	1.223322786	C29H11orf54
29	550001	600000	1.223322786	TAF1D
29	550001	600000	1.223322786	CEP295
6	95640001	95690000	1.22297845	C6H4orf22
5	111990001	112040000	1.222921337	SMDT1
5	111990001	112040000	1.222921337	LOC102169285
5	111990001	112040000	1.222921337	NAGA
5	111990001	112040000	1.222921337	FAM109B
5	111990001	112040000	1.222921337	NDUFA6
26	45910001	45960000	1.222868763	PCDH15
14	94400001	94450000	1.222786734	KHDC3L
14	94400001	94450000	1.222786734	LOC102180236
14	94400001	94450000	1.222786734	DDX43
29	400001	450000	1.222725481	LOC106503712
24	62160001	62210000	1.222671027	SERPINB7
1	52140001	52190000	1.2225976	BBX
7	27610001	27660000	1.222551409	XRCC4
7	27610001	27660000	1.222551409	TMEM167A
13	37870001	37920000	1.222480243	LOC108637388
13	37870001	37920000	1.222480243	LOC102189769
13	37870001	37920000	1.222480243	DZANK1
11	28150001	28200000	1.222447698	PRKCE
9	1870001	1920000	1.22228579	COL12A1
18	36210001	36260000	1.222267217	LRRC36
1	157090001	157140000	1.222231112	KAT2B
22	28850001	28900000	1.222094475	SHQ1
16	53670001	53720000	1.222035412	KLHL20
16	53670001	53720000	1.222035412	CENPL
13	37770001	37820000	1.22181148	PET117
13	37770001	37820000	1.22181148	KAT14
4	28750001	28800000	1.221798134	GRM8
26	34380001	34430000	1.221676956	ENTPD1
17	34480001	34530000	1.221600688	FSTL5
25	18430001	18480000	1.221515722	LOC102184112
10	87820001	87870000	1.221433609	LCTL
10	87820001	87870000	1.221433609	ZWILCH
10	94530001	94580000	1.221158094	ANKRD31
18	54100001	54150000	1.221085516	PPM1N
18	54100001	54150000	1.221085516	LOC108638001
18	54100001	54150000	1.221085516	RTN2
18	54100001	54150000	1.221085516	FOSB
18	36220001	36270000	1.220916095	TPPP3
18	36220001	36270000	1.220916095	LRRC36
25	18090001	18140000	1.220636108	ACSM1
3	41160001	41210000	1.220586528	LEPR

21	19040001	19090000	1.220514988	LOC102175848
21	19040001	19090000	1.220514988	LOC102176116
21	19040001	19090000	1.220514988	LOC108638532
21	15220001	15270000	1.220409532	SV2B
8	64260001	64310000	1.220132438	LOC106502417
11	74670001	74720000	1.2200808	PFN4
11	74670001	74720000	1.2200808	TP53I3
11	74670001	74720000	1.2200808	SF3B6
11	74670001	74720000	1.2200808	FKBP1B
11	74670001	74720000	1.2200808	WDCP
11	74670001	74720000	1.2200808	FAM228B
18	56340001	56390000	1.219828828	LOC102185028
18	56340001	56390000	1.219828828	MAMSTR
18	36230001	36280000	1.219459353	TPPP3
18	36230001	36280000	1.219459353	LRRC36
18	36230001	36280000	1.219459353	ZDHHC1
25	18450001	18500000	1.219225179	DCUN1D3
25	18450001	18500000	1.219225179	LOC102184112
3	25640001	25690000	1.21917869	C3H1orf185
16	53680001	53730000	1.219067601	KLHL20
16	53680001	53730000	1.219067601	CENPL
16	53680001	53730000	1.219067601	DARS2
10	76040001	76090000	1.219043107	LOC108636866
10	76040001	76090000	1.219043107	LOC102183422
10	76040001	76090000	1.219043107	LOC108636909
10	76050001	76100000	1.219033198	LOC102183422
10	76050001	76100000	1.219033198	TRNAT-UGU-2
10	76050001	76100000	1.219033198	LOC108636909
18	36200001	36250000	1.219008373	KCTD19
18	36200001	36250000	1.219008373	LRRC36
4	5090001	5140000	1.218989922	KMT2C
24	43850001	43900000	1.218963926	MC5R
24	43850001	43900000	1.218963926	RNMT
24	43850001	43900000	1.218963926	MC2R
5	110350001	110400000	1.218879855	TNRC6B
4	75720001	75770000	1.218829567	PSMC2
4	75720001	75770000	1.218829567	DNAJC2
4	87890001	87940000	1.218482094	LOC102171502
2	18430001	18480000	1.218319532	PID1
1	157120001	157170000	1.218247514	KAT2B
16	45120001	45170000	1.218213033	CAMTA1
16	43290001	43340000	1.21818738	RERE
4	14190001	14240000	1.21817097	LOC108635908
4	14190001	14240000	1.21817097	LOC108635909
4	14190001	14240000	1.21817097	LOC102181470
14	94380001	94430000	1.218072691	KHDC3L
14	94380001	94430000	1.218072691	LOC102180236
13	53550001	53600000	1.218061483	CHRNA4
13	53550001	53600000	1.218061483	COL20A1
14	12060001	12110000	1.218042871	CDH17
10	80570001	80620000	1.217892165	CIDEB
10	80570001	80620000	1.217892165	LTB4R2
10	80570001	80620000	1.217892165	RIPK3
10	80570001	80620000	1.217892165	LTB4R
10	80570001	80620000	1.217892165	ADCY4
9	77600001	77650000	1.217887992	LOC102190323
21	64500001	64550000	1.217845619	SLC25A47
21	64500001	64550000	1.217845619	WARS

21	64500001	64550000	1.217845619	WDR25
21	57100001	57150000	1.217793277	ITPK1
3	110910001	110960000	1.217767925	SLAMF1
18	56980001	57030000	1.217382997	PRRG2
18	56980001	57030000	1.217382997	RCN3
18	56980001	57030000	1.217382997	NOSIP
18	56980001	57030000	1.217382997	PRR12
15	39660001	39710000	1.217294926	SBF2
19	25960001	26010000	1.217123262	RABEP1
19	25960001	26010000	1.217123262	NUP88
5	33250001	33300000	1.216907112	LOC102190664
8	60880001	60930000	1.216844486	ZCCHC7
1	157290001	157340000	1.216780538	LOC102184668
10	87840001	87890000	1.216745609	LCTL
10	87840001	87890000	1.216745609	ZWILCH
14	16080001	16130000	1.216655589	RIDA
14	16080001	16130000	1.216655589	ERICH5
14	16080001	16130000	1.216655589	POP1
1	157080001	157130000	1.216397451	KAT2B
19	20560001	20610000	1.21638774	TAOK1
16	45100001	45150000	1.215938866	CAMTA1
20	23230001	23280000	1.215543249	IL6ST
24	62170001	62220000	1.215460686	SERPINB7
6	80530001	80580000	1.215456155	TECRL
7	89100001	89150000	1.215454543	IZUMO4
7	89100001	89150000	1.215454543	MOB3A
7	89100001	89150000	1.215454543	AP3D1
12	13150001	13200000	1.215333555	DNAJC3
12	13150001	13200000	1.215333555	UGGT2
12	63220001	63270000	1.214945442	FREM2
20	31370001	31420000	1.214650325	HMGCS1
9	82480001	82530000	1.214586468	EZR
29	460001	510000	1.214426963	VSTM5
2	83920001	83970000	1.214336249	GTDC1
1	120890001	120940000	1.214136408	LOC106502391
5	20750001	20800000	1.213860274	KERA
5	20750001	20800000	1.213860274	LUM
4	65180001	65230000	1.213816502	TRNAG-CCC-25
12	17910001	17960000	1.213812358	GPC6
16	59780001	59830000	1.21365626	CEP350
6	65100001	65150000	1.213637886	GABRG1
3	41170001	41220000	1.213471721	LEPR
5	112010001	112060000	1.213454644	LOC102169285
5	112010001	112060000	1.213454644	NDUFA6
5	112010001	112060000	1.213454644	LOC102177333
27	39120001	39170000	1.213352906	MCPH1
20	33700001	33750000	1.213223599	PTGER4
20	33700001	33750000	1.213223599	TTC33
16	59790001	59840000	1.213119662	CEP350
16	59840001	59890000	1.212988531	CEP350
5	33460001	33510000	1.212780434	SLC38A1
2	114260001	114310000	1.212721578	CHN1
5	58850001	58900000	1.212691158	SNRPF
14	61000001	61050000	1.212582342	PCMTD1
19	20570001	20620000	1.212401548	TAOK1
7	70620001	70670000	1.212273207	LOC102170224
7	70620001	70670000	1.212273207	MGAT1
13	53560001	53610000	1.212101212	CHRNA4

13	53560001	53610000	1.212101212	COL20A1
19	25970001	26020000	1.212052863	RABEP1
1	118520001	118570000	1.212029703	CP
1	118520001	118570000	1.212029703	LOC102172204
2	7110001	7160000	1.211701305	MYOM3
2	7110001	7160000	1.211701305	IL22RA1
25	240001	290000	1.211691238	ARHGDIG
25	240001	290000	1.211691238	PDIA2
25	240001	290000	1.211691238	AXIN1
24	62220001	62270000	1.211615211	LOC102181552
4	7630001	7680000	1.211438371	LOC102186105
4	7630001	7680000	1.211438371	LOC102186385
13	65300001	65350000	1.211334049	SLA2
13	65300001	65350000	1.211334049	NDRG3
21	15250001	15300000	1.211168648	SV2B
25	18460001	18510000	1.210913744	DCUN1D3
25	18460001	18510000	1.210913744	LOC102184112
1	95380001	95430000	1.210697353	PLD1
4	99740001	99790000	1.210629968	ARL4A
14	15990001	16040000	1.210487631	MATN2
16	45130001	45180000	1.210427542	CAMTA1
27	39110001	39160000	1.210346887	MCPH1
8	55360001	55410000	1.209989703	TLE4
20	23240001	23290000	1.209863885	IL6ST
16	53650001	53700000	1.209772168	KLHL20
5	112000001	112050000	1.209742815	SMDT1
5	112000001	112050000	1.209742815	LOC102169285
5	112000001	112050000	1.209742815	FAM109B
5	112000001	112050000	1.209742815	NDUFA6
5	112000001	112050000	1.209742815	LOC102177333
15	6280001	6330000	1.209621218	F2
15	6280001	6330000	1.209621218	CKAP5
24	26860001	26910000	1.209545192	LOC108633784
12	14500001	14550000	1.20941465	LOC108637255
23	19480001	19530000	1.209401397	ZKSCAN4
23	19480001	19530000	1.209401397	NKAPL
23	19480001	19530000	1.209401397	ZSCAN26
23	19480001	19530000	1.209401397	PGBD1
14	4030001	4080000	1.209115971	RALYL
23	22400001	22450000	1.20910213	SAPCD1
23	22400001	22450000	1.20910213	VARS
23	22400001	22450000	1.20910213	LSM2
23	22400001	22450000	1.20910213	HSP70.1
23	22400001	22450000	1.20910213	MSH5
23	22400001	22450000	1.20910213	LOC102177850
23	22400001	22450000	1.20910213	VWA7
10	94520001	94570000	1.208992582	ANKRD31
23	22410001	22460000	1.208950847	VARS
23	22410001	22460000	1.208950847	LSM2
23	22410001	22460000	1.208950847	HSP70.1
23	22410001	22460000	1.208950847	LOC102178315
23	22410001	22460000	1.208950847	LOC102177673
23	22410001	22460000	1.208950847	LOC102177850
23	22410001	22460000	1.208950847	VWA7
6	22320001	22370000	1.20894172	UBE2D3
6	22320001	22370000	1.20894172	MANBA
4	7690001	7740000	1.208874878	LOC106501766
4	7690001	7740000	1.208874878	LOC102168236

12	14490001	14540000	1.208685795	LOC108637255
20	33710001	33760000	1.208607562	PTGER4
20	33710001	33760000	1.208607562	TTC33
9	14320001	14370000	1.208593753	NKAIN2
1	157260001	157310000	1.208492528	LOC108636388
1	157260001	157310000	1.208492528	LOC102184668
4	75380001	75430000	1.208475469	RELN
1	118560001	118610000	1.208202483	CP
1	118560001	118610000	1.208202483	HPS3
1	50060001	50110000	1.208166446	ALCAM
5	23410001	23460000	1.208164953	CRADD
5	23410001	23460000	1.208164953	LOC106502085
12	60160001	60210000	1.208082001	LOC102178917
6	65090001	65140000	1.208058425	GABRG1
1	146470001	146520000	1.207948637	FBXO25
1	146470001	146520000	1.207948637	TDRP
8	55370001	55420000	1.207842208	TLE4
1	45740001	45790000	1.207730527	ZBTB11
1	45740001	45790000	1.207730527	PCNP
22	16270001	16320000	1.207721565	ZNF502
22	16270001	16320000	1.207721565	ZNF501
22	16270001	16320000	1.207721565	KIAA1143
22	16270001	16320000	1.207721565	LOC102177570
15	34120001	34170000	1.207664822	LOC102182615
15	34120001	34170000	1.207664822	LOC102176442
15	34120001	34170000	1.207664822	LOC102174495
1	157110001	157160000	1.207474564	KAT2B
13	65320001	65370000	1.207366279	NDRG3
26	44190001	44240000	1.207356204	PRKG1
1	112930001	112980000	1.207218412	TRNAE-UUC-2
1	112930001	112980000	1.207218412	GPR149
1	112930001	112980000	1.207218412	DHX36
4	7620001	7670000	1.207080593	LOC102186105
4	7620001	7670000	1.207080593	LOC102186385
2	88120001	88170000	1.207067878	LOC106502907
2	88120001	88170000	1.207067878	ORC4
13	65290001	65340000	1.206986711	SLA2
13	65290001	65340000	1.206986711	NDRG3
1	93990001	94040000	1.206929218	SPATA16
3	64190001	64240000	1.206908563	HS2ST1
3	85650001	85700000	1.206865704	LOC102187823
26	29860001	29910000	1.206660612	SEMA4G
26	29860001	29910000	1.206660612	SLF2
13	37880001	37930000	1.206653912	LOC102189769
13	37880001	37930000	1.206653912	POLR3F
13	37880001	37930000	1.206653912	DZANK1
14	94390001	94440000	1.206652327	KHDC3L
14	94390001	94440000	1.206652327	LOC102180236
14	94390001	94440000	1.206652327	DDX43
11	10740001	10790000	1.206269633	TPRKB
11	10740001	10790000	1.206269633	NAT8
11	10740001	10790000	1.206269633	ALMS1
21	64470001	64520000	1.206141705	MIR345
21	64470001	64520000	1.206141705	SLC25A47
21	64470001	64520000	1.206141705	YY1
21	64470001	64520000	1.206141705	WARS
21	64470001	64520000	1.206141705	SLC25A29
16	78990001	79040000	1.206050786	IPO9

16	78990001	79040000	1.206050786	NAV1
16	78990001	79040000	1.206050786	LOC108637801
7	91550001	91600000	1.205772018	PTPRS
16	43300001	43350000	1.205731797	RERE
11	48930001	48980000	1.205645314	TMEM150A
11	48930001	48980000	1.205645314	VAMP8
11	48930001	48980000	1.205645314	RNF181
11	48930001	48980000	1.205645314	VAMP5
11	48930001	48980000	1.205645314	GGCX
17	66800001	66850000	1.205492854	TRNAC-GCA-206
17	66800001	66850000	1.205492854	KIAA0922
1	40490001	40540000	1.205401323	EPHA6
15	34070001	34120000	1.205341935	LOC102175876
15	34070001	34120000	1.205341935	LOC102175600
15	34070001	34120000	1.205341935	LOC102182894
15	34070001	34120000	1.205341935	LOC102175317
15	34070001	34120000	1.205341935	HBBC
20	20070001	20120000	1.205256932	LOC102184195
20	20070001	20120000	1.205256932	PDE4D
24	14040001	14090000	1.205202048	PIK3C3
1	112970001	113020000	1.20513858	DHX36
1	112970001	113020000	1.20513858	ARHGEF26
16	43180001	43230000	1.204880364	RERE
20	31350001	31400000	1.204874745	CCL28
19	20510001	20560000	1.204829087	TAOK1
5	31330001	31380000	1.204771955	LOC108636097
14	12660001	12710000	1.204560732	DPY19L4
14	12660001	12710000	1.204560732	INTS8
18	16170001	16220000	1.20418925	DBNDD1
18	16170001	16220000	1.20418925	LOC102172001
18	16170001	16220000	1.20418925	GAS8
4	7610001	7660000	1.20415697	LOC102186105
4	7610001	7660000	1.20415697	LOC102186385
25	18470001	18520000	1.204132483	DCUN1D3
25	18470001	18520000	1.204132483	LOC102184112
5	14550001	14600000	1.20401785	LRRIQ1
10	26590001	26640000	1.203896804	LOC102175585
23	22420001	22470000	1.203753601	VARS
23	22420001	22470000	1.203753601	LSM2
23	22420001	22470000	1.203753601	HSP70.1
23	22420001	22470000	1.203753601	LOC102178315
23	22420001	22470000	1.203753601	LOC102177673
23	22420001	22470000	1.203753601	LOC102177850
6	85670001	85720000	1.203489059	LOC102168522
1	157100001	157150000	1.203151857	KAT2B
4	28760001	28810000	1.203065516	MIR592
4	28760001	28810000	1.203065516	GRM8
9	82460001	82510000	1.203041896	TRNAE-UUC-39
9	82460001	82510000	1.203041896	EZR
6	37060001	37110000	1.202958222	ABCG2
26	28140001	28190000	1.202912566	CNNM2
6	21730001	21780000	1.202911955	TACR3
11	105450001	105500000	1.202883246	CACNA1B
5	110390001	110440000	1.202774667	TNRC6B
6	85640001	85690000	1.202528875	LOC102168522
1	157250001	157300000	1.202397339	LOC108636388
1	157250001	157300000	1.202397339	LOC102184668
24	50730001	50780000	1.202373994	SMAD4

3	41180001	41230000	1.202367234	LEPR
13	42950001	43000000	1.202289347	LOC102187204
13	42950001	43000000	1.202289347	LOC102189109
10	87930001	87980000	1.20218023	LOC106502512
10	87930001	87980000	1.20218023	SNAPC5
10	87930001	87980000	1.20218023	MAP2K1
22	60180001	60230000	1.202000634	LOC106503466
22	60180001	60230000	1.202000634	KLF15
10	94670001	94720000	1.201944149	LOC108636845
10	94670001	94720000	1.201944149	GCNT4
10	94670001	94720000	1.201944149	ANKRD31
4	65160001	65210000	1.201765304	TRNAG-CCC-25
19	25980001	26030000	1.201412016	RABEP1
18	36280001	36330000	1.201074031	ZDHHC1
18	36280001	36330000	1.201074031	ATP6V0D1
18	36280001	36330000	1.201074031	HSD11B2
20	3140001	3190000	1.201030384	RANBP17
1	157130001	157180000	1.201013396	KAT2B
5	23420001	23470000	1.200814449	CRADD
5	23420001	23470000	1.200814449	LOC106502085
15	39670001	39720000	1.200696086	SBF2
16	43170001	43220000	1.200399268	RERE
24	43690001	43740000	1.200053233	LDLRAD4
7	91570001	91620000	1.199878408	PTPRS
14	40170001	40220000	1.199876061	ZC2HC1A
14	94370001	94420000	1.19961184	KHDC3L
14	94370001	94420000	1.19961184	LOC102180236
7	41400001	41450000	1.199446581	ADAM19
7	91540001	91590000	1.199269255	PTPRS
4	7600001	7650000	1.199124975	LOC102186105
23	19570001	19620000	1.199044931	ZSCAN12
23	19570001	19620000	1.199044931	ZSCAN23
6	65080001	65130000	1.198967809	GABRG1
8	100970001	101020000	1.198963208	UGCG
10	98790001	98840000	1.198908283	YTHDC2
3	64180001	64230000	1.198870379	HS2ST1
15	39650001	39700000	1.198863306	SBF2
24	14100001	14150000	1.198706629	PIK3C3
21	19030001	19080000	1.198610512	LOC102175848
21	19030001	19080000	1.198610512	LOC108638532
24	43700001	43750000	1.198522701	LDLRAD4
13	37890001	37940000	1.198308542	LOC102189769
13	37890001	37940000	1.198308542	POLR3F
13	37890001	37940000	1.198308542	RBBP9
13	37890001	37940000	1.198308542	DZANK1
3	95450001	95500000	1.198094206	SPAG17
23	19490001	19540000	1.197742155	ZKSCAN4
23	19490001	19540000	1.197742155	NKAPL
23	19490001	19540000	1.197742155	ZSCAN26
23	19490001	19540000	1.197742155	PGBD1
16	30800001	30850000	1.197738211	EFCAB2
12	13130001	13180000	1.197667105	DNAJC3
12	13130001	13180000	1.197667105	UGGT2
24	14050001	14100000	1.19751976	PIK3C3
1	45160001	45210000	1.19714433	ABI3BP
1	94000001	94050000	1.196979184	SPATA16
10	97070001	97120000	1.196769932	CCDC112
10	97070001	97120000	1.196769932	PGGT1B

5	112020001	112070000	1.19663907	LOC102169285
5	112020001	112070000	1.19663907	LOC102177333
5	112020001	112070000	1.19663907	LOC102169002
11	105460001	105510000	1.196482882	CACNA1B
13	71660001	71710000	1.196366105	L3MBTL1
13	71660001	71710000	1.196366105	SGK2
15	6270001	6320000	1.19633745	TRNAE-CUC-23
15	6270001	6320000	1.19633745	F2
15	6270001	6320000	1.19633745	CKAP5
7	41390001	41440000	1.196209886	ADAM19
15	39680001	39730000	1.196089584	SBF2
12	60090001	60140000	1.196010924	TRNAC-GCA-168
12	60090001	60140000	1.196010924	LOC102178917
26	47140001	47190000	1.195991186	LOC102181467
11	14140001	14190000	1.195906368	LOC108637103
11	14140001	14190000	1.195906368	SRD5A2
10	26610001	26660000	1.195737337	LOC102175585
3	46630001	46680000	1.195486084	PTGER3
24	14070001	14120000	1.195478508	PIK3C3
23	22430001	22480000	1.195295392	LSM2
23	22430001	22480000	1.195295392	HSP70.1
23	22430001	22480000	1.195295392	LOC102178315
23	22430001	22480000	1.195295392	LOC102177673
23	22430001	22480000	1.195295392	LOC102177850
23	22430001	22480000	1.195295392	NEU1
1	95410001	95460000	1.195234136	PLD1
24	14080001	14130000	1.195228276	PIK3C3
20	20080001	20130000	1.195217563	LOC102184195
20	20080001	20130000	1.195217563	PDE4D
2	6630001	6680000	1.195142117	ID3
10	26580001	26630000	1.195111513	LOC102175585
16	45030001	45080000	1.194878096	CAMTA1
1	52200001	52250000	1.194823436	BBX
16	30790001	30840000	1.194810217	EFCAB2
21	19070001	19120000	1.194594633	LOC102176116
21	19070001	19120000	1.194594633	LOC108638532
21	19070001	19120000	1.194594633	LOC102174020
22	11530001	11580000	1.19457955	OXSR1
26	44140001	44190000	1.194446061	PRKG1
16	43310001	43360000	1.194376469	RERE
11	14060001	14110000	1.194299414	XDH
1	68330001	68380000	1.1942579	LOC102173333
15	39640001	39690000	1.19424748	SBF2
7	26760001	26810000	1.194200506	EDIL3
21	57080001	57130000	1.194019073	ITPK1
4	7590001	7640000	1.193918327	LOC102186105
19	51570001	51620000	1.193860358	RNF213
14	12650001	12700000	1.193835773	DPY19L4
14	12650001	12700000	1.193835773	INTS8
4	48580001	48630000	1.193802019	NPY
5	87650001	87700000	1.193705186	SLCO1C1
24	14060001	14110000	1.193627993	PIK3C3
16	53620001	53670000	1.193618451	KLHL20
22	28860001	28910000	1.193574907	SHQ1
23	22390001	22440000	1.193537168	SAPCD1
23	22390001	22440000	1.193537168	VARS
23	22390001	22440000	1.193537168	LSM2
23	22390001	22440000	1.193537168	MSH5

23	22390001	22440000	1.193537168	LOC102177850
23	22390001	22440000	1.193537168	VWA7
24	62180001	62230000	1.193410113	SERPINB7
25	34980001	35030000	1.1933378	LOC102187393
25	34980001	35030000	1.1933378	LOC102181832
2	7140001	7190000	1.19332581	MYOM3
2	7140001	7190000	1.19332581	IL22RA1
2	41200001	41250000	1.193289203	ADAM23
6	1920001	1970000	1.193174129	1-Mar
14	94410001	94460000	1.193162259	LOC102180236
14	94410001	94460000	1.193162259	DDX43
14	12720001	12770000	1.193058186	CCNE2
14	12720001	12770000	1.193058186	INTS8
8	55390001	55440000	1.1928382	TLE4
5	39870001	39920000	1.192667595	MUC19
1	95390001	95440000	1.19242653	PLD1
17	66840001	66890000	1.192327378	TLR2
17	66840001	66890000	1.192327378	RNF175
29	42970001	43020000	1.192231069	PPP1R14B
29	42970001	43020000	1.192231069	GPR137
29	42970001	43020000	1.192231069	KCNK4
29	42970001	43020000	1.192231069	BAD
29	42970001	43020000	1.192231069	PLCB3
25	250001	300000	1.192209792	MRPL28
25	250001	300000	1.192209792	TMEM8A
25	250001	300000	1.192209792	AXIN1
26	34460001	34510000	1.192112695	ALDH18A1
6	95570001	95620000	1.192026854	TRNAG-CCC-33
6	95570001	95620000	1.192026854	C6H4orf22
6	4600001	4650000	1.192018045	TNIP3
7	27570001	27620000	1.191992328	XRCC4
1	96730001	96780000	1.191924436	PHC3
7	37410001	37460000	1.19180745	TRNAS-GGA-38
7	37410001	37460000	1.19180745	GABRB2
1	45120001	45170000	1.191637867	ABI3BP
24	14030001	14080000	1.191568212	PIK3C3
5	87640001	87690000	1.191419894	SLCO1C1
4	7700001	7750000	1.191145513	LOC106501766
4	7700001	7750000	1.191145513	LOC102168236
24	14090001	14140000	1.191135609	PIK3C3
11	28130001	28180000	1.191048617	PRKCE
26	29830001	29880000	1.190947332	MRPL43
26	29830001	29880000	1.190947332	SEMA4G
26	29830001	29880000	1.190947332	PDZD7
26	29830001	29880000	1.190947332	LZTS2
26	29830001	29880000	1.190947332	C26H10orf2
26	29830001	29880000	1.190947332	SLF2
1	40480001	40530000	1.190942581	EPHA6
26	29870001	29920000	1.190855475	SLF2
11	14070001	14120000	1.190788203	XDH
23	28220001	28270000	1.190576185	CD2AP
15	25810001	25860000	1.190484698	ANO3
10	34230001	34280000	1.190441144	TMEM260
1	52210001	52260000	1.190415859	BBX
5	72640001	72690000	1.190415248	RBFOX2
11	44390001	44440000	1.190411185	LIMS1
11	44390001	44440000	1.190411185	GCC2
8	64270001	64320000	1.190367035	LOC106502417

14	12730001	12780000	1.190086016	CCNE2
14	12730001	12780000	1.190086016	INTS8
16	45020001	45070000	1.190028468	CAMTA1
6	4610001	4660000	1.190013143	TNIP3
4	5110001	5160000	1.189957196	KMT2C
3	93160001	93210000	1.189927118	SLC22A15
9	88980001	89030000	1.189903294	RPS6KA2
13	2270001	2320000	1.189764352	PAK5
4	72960001	73010000	1.189730137	NAMPT
22	60190001	60240000	1.189674797	KLF15
2	114270001	114320000	1.189652472	CHN1
17	8690001	8740000	1.189524417	TRNAG-CCC-73
17	8690001	8740000	1.189524417	BICDL1
1	50050001	50100000	1.189473181	ALCAM
13	53570001	53620000	1.189398239	ARFGAP1
13	53570001	53620000	1.189398239	CHRNA4
13	53570001	53620000	1.189398239	COL20A1
1	157240001	157290000	1.189377278	LOC108636388
18	14560001	14610000	1.189368199	JPH3
4	28740001	28790000	1.189349452	GRM8
26	28150001	28200000	1.189347656	CNNM2
2	88150001	88200000	1.18909196	LOC106502907
17	34490001	34540000	1.189078105	FSTL5
5	46940001	46990000	1.189038918	TMBIM4
5	46940001	46990000	1.189038918	IRAK3
1	51240001	51290000	1.189014865	LOC108636831
26	47130001	47180000	1.188928108	LOC102181467
9	77590001	77640000	1.188495518	LOC102190323
6	95690001	95740000	1.188435713	C6H4orf22
5	33370001	33420000	1.188389798	LOC106502098
5	33370001	33420000	1.188389798	SLC38A2
21	57110001	57160000	1.188370399	ITPK1
9	14330001	14380000	1.188257907	NKAIN2
18	54140001	54190000	1.188167593	PPM1N
18	54140001	54190000	1.188167593	RTN2
18	54140001	54190000	1.188167593	VASP
18	54140001	54190000	1.188167593	OPA3
24	43680001	43730000	1.188053968	LDLRAD4
4	65190001	65240000	1.187765764	TRNAG-CCC-25
16	79000001	79050000	1.187577634	IPO9
16	79000001	79050000	1.187577634	NAV1
16	79000001	79050000	1.187577634	LOC108637801
11	44380001	44430000	1.187549461	LIMS1
11	44380001	44430000	1.187549461	GCC2
12	60170001	60220000	1.187429025	LOC102178917
15	32080001	32130000	1.187354577	PGAP2
15	32080001	32130000	1.187354577	RHOG
15	32080001	32130000	1.187354577	STIM1
18	64750001	64800000	1.187352152	TSEN34
18	64750001	64800000	1.187352152	LOC106501822
18	64750001	64800000	1.187352152	RPS9
18	64750001	64800000	1.187352152	MBOAT7
28	110001	160000	1.187258065	LOC108634156
28	110001	160000	1.187258065	TFAM
9	31130001	31180000	1.187132187	PRDM1
13	54320001	54370000	1.187062244	MTG2
13	54320001	54370000	1.187062244	HRH3
13	54320001	54370000	1.187062244	SS18L1

16	59770001	59820000	1.186880001	CEP350
10	80610001	80660000	1.186838766	LOC108637011
10	80610001	80660000	1.186838766	NFATC4
10	80610001	80660000	1.186838766	NYNRIN
24	43560001	43610000	1.186785469	CEP192
16	24510001	24560000	1.186713945	TRNAT-UGU-6
16	24510001	24560000	1.186713945	HHIPL2
16	24510001	24560000	1.186713945	TAF1A
10	87940001	87990000	1.186669338	LOC106502512
10	87940001	87990000	1.186669338	SNAPC5
10	87940001	87990000	1.186669338	MAP2K1
5	84550001	84600000	1.186496662	SOX5
12	63230001	63280000	1.186358943	FREM2
23	28200001	28250000	1.186307037	CD2AP
5	33380001	33430000	1.186142801	LOC106502098
5	33380001	33430000	1.186142801	SLC38A2
13	53350001	53400000	1.186138224	SLC2A4RG
13	53350001	53400000	1.186138224	LIME1
13	53350001	53400000	1.186138224	ARFRP1
13	53350001	53400000	1.186138224	TRNAS-GGA-73
13	53350001	53400000	1.186138224	TNFRSF6B
13	53350001	53400000	1.186138224	ZGPAT
13	53350001	53400000	1.186138224	RTEL1
13	53350001	53400000	1.186138224	ZBTB46
11	72330001	72380000	1.186118232	MAPRE3
2	41190001	41240000	1.186039259	ADAM23
15	28940001	28990000	1.185893948	RNF169
13	37900001	37950000	1.18582192	POLR3F
13	37900001	37950000	1.18582192	RBBP9
13	37900001	37950000	1.18582192	DZANK1
23	22380001	22430000	1.185805897	CLIC1
23	22380001	22430000	1.185805897	SAPCD1
23	22380001	22430000	1.185805897	VARS
23	22380001	22430000	1.185805897	DDAH2
23	22380001	22430000	1.185805897	MSH5
23	22380001	22430000	1.185805897	VWA7
14	12550001	12600000	1.185196812	ESRP1
5	23320001	23370000	1.185153722	CRADD
24	43550001	43600000	1.184914022	SEH1L
24	43550001	43600000	1.184914022	CEP192
22	28820001	28870000	1.184743136	SHQ1
2	115500001	115550000	1.184665078	HOXD13
2	115500001	115550000	1.184665078	HOXD11
2	115500001	115550000	1.184665078	HOXD10
2	115500001	115550000	1.184665078	HOXD9
2	115500001	115550000	1.184665078	HOXD8
2	115500001	115550000	1.184665078	LOC108638283
2	115500001	115550000	1.184665078	HOXD12
25	9590001	9640000	1.184645958	CLEC16A
24	43570001	43620000	1.184593778	CEP192
25	34860001	34910000	1.184547142	LOC102181832
21	15300001	15350000	1.184503564	SV2B
2	6650001	6700000	1.184477727	ID3
24	43540001	43590000	1.184467399	SEH1L
24	43540001	43590000	1.184467399	CEP192
24	14020001	14070000	1.18439551	PIK3C3
26	34490001	34540000	1.183858644	ALDH18A1
22	60170001	60220000	1.183831355	LOC106503466

22	60170001	60220000	1.183831355	KLF15
22	60170001	60220000	1.183831355	ALDH1L1
27	30380001	30430000	1.18380242	CENPU
10	94620001	94670000	1.18378355	ANKRD31
13	54330001	54380000	1.183714842	MTG2
13	54330001	54380000	1.183714842	HRH3
13	54330001	54380000	1.183714842	SS18L1
13	54330001	54380000	1.183714842	PSMA7
12	14480001	14530000	1.183695792	LOC108637255
10	94630001	94680000	1.183689237	ANKRD31
19	25950001	26000000	1.183629745	RABEP1
19	25950001	26000000	1.183629745	NUP88
5	80450001	80500000	1.183592093	PTHLH
15	39630001	39680000	1.183570416	SBF2
24	14110001	14160000	1.183518428	PIK3C3
2	110600001	110650000	1.183351102	SP5
4	12270001	12320000	1.183254789	TRNAS-GGA-21
17	34470001	34520000	1.183232728	FSTL5
26	32290001	32340000	1.183166559	TRNAE-UUC-96
26	32290001	32340000	1.183166559	R3HCC1L
26	30960001	31010000	1.183152712	LOC102179346
26	30960001	31010000	1.183152712	ENTPD7
26	30960001	31010000	1.183152712	CUTC
2	88160001	88210000	1.183102698	LOC106502907
16	45010001	45060000	1.183088596	CAMTA1
7	26750001	26800000	1.182914317	EDIL3
5	75040001	75090000	1.182872591	SYT10
28	18820001	18870000	1.182742313	ADAMTS14
1	118570001	118620000	1.182610258	CP
1	118570001	118620000	1.182610258	HPS3
22	6710001	6760000	1.18241068	CMTM7
22	6710001	6760000	1.18241068	CMTM8
18	53380001	53430000	1.182360498	CEACAM20
10	87920001	87970000	1.182346945	LOC106502512
10	87920001	87970000	1.182346945	SNAPC5
10	87920001	87970000	1.182346945	MAP2K1
4	28470001	28520000	1.182257638	ZNF800
24	43580001	43630000	1.182180104	CEP192
6	108900001	108950000	1.182174464	RAB28
11	74640001	74690000	1.182123644	PFN4
11	74640001	74690000	1.182123644	TP53I3
11	74640001	74690000	1.182123644	SF3B6
11	74640001	74690000	1.182123644	FAM228B
16	53690001	53740000	1.182109986	CENPL
16	53690001	53740000	1.182109986	DARS2
1	157150001	157200000	1.182019649	KAT2B
19	5300001	5350000	1.181977929	LOC102176553
19	5300001	5350000	1.181977929	MMD
5	72650001	72700000	1.181946941	RBFOX2
7	70630001	70680000	1.181494045	MGAT1
10	94570001	94620000	1.181293833	ANKRD31
8	55380001	55430000	1.181271328	TLE4
4	5100001	5150000	1.181108025	KMT2C
13	53340001	53390000	1.181088383	SLC2A4RG
13	53340001	53390000	1.181088383	LIME1
13	53340001	53390000	1.181088383	ARFRP1
13	53340001	53390000	1.181088383	TRNAS-GGA-73
13	53340001	53390000	1.181088383	TNFRSF6B

13	53340001	53390000	1.181088383	ZGPAT
13	53340001	53390000	1.181088383	RTEL1
13	53340001	53390000	1.181088383	ZBTB46
4	5020001	5070000	1.181027143	KMT2C
5	109450001	109500000	1.180997118	LOC106502146
5	109450001	109500000	1.180997118	LOC102184324
18	56360001	56410000	1.180996008	IZUMO1
18	56360001	56410000	1.180996008	FUT1
18	56360001	56410000	1.180996008	FGF21
18	56360001	56410000	1.180996008	MAMSTR
20	33690001	33740000	1.180938376	PTGER4
20	33690001	33740000	1.180938376	TTC33
20	33690001	33740000	1.180938376	LOC108638402
20	33690001	33740000	1.180938376	PRKAA1
11	14040001	14090000	1.18087333	XDH
23	28210001	28260000	1.180792519	CD2AP
6	37050001	37100000	1.180783407	ABCG2
7	70640001	70690000	1.180688666	LOC102181492
7	70640001	70690000	1.180688666	MGAT1
8	55350001	55400000	1.180590721	TLE4
24	50760001	50810000	1.18054853	SMAD4
9	82450001	82500000	1.180427343	TRNAE-UUC-39
9	82450001	82500000	1.180427343	EZR
9	82450001	82500000	1.180427343	SYTL3
3	95420001	95470000	1.180363622	SPAG17
9	54770001	54820000	1.180272357	LAMA2
14	12570001	12620000	1.180192505	TRNAG-CCC-57
14	12570001	12620000	1.180192505	DPY19L4
14	12570001	12620000	1.180192505	ESRP1
2	19490001	19540000	1.180041702	SPHKAP
1	27980001	28030000	1.17998403	GBE1
16	30810001	30860000	1.179969859	EFCAB2
20	20090001	20140000	1.179905221	PDE4D
3	41130001	41180000	1.179880194	LEPR
4	65150001	65200000	1.179725688	TRNAG-CCC-25
18	64740001	64790000	1.179685376	TSEN34
18	64740001	64790000	1.179685376	LOC102172431
18	64740001	64790000	1.179685376	LOC106501822
18	64740001	64790000	1.179685376	RPS9
10	31410001	31460000	1.179649411	CCDC175
10	31410001	31460000	1.179649411	JKAMP
11	105470001	105520000	1.179643324	CACNA1B
1	110500001	110550000	1.179441807	TIPARP
4	75760001	75810000	1.179373552	PMPCB
4	75760001	75810000	1.179373552	DNAJC2
10	30530001	30580000	1.179339011	C10H14orf39
17	70300001	70350000	1.179256747	TLL1
5	33240001	33290000	1.17921662	LOC102190664
22	6700001	6750000	1.179208798	CMTM7
22	6700001	6750000	1.179208798	CMTM8
19	41310001	41360000	1.179180045	KRT35
19	41310001	41360000	1.179180045	KRT36
19	41310001	41360000	1.179180045	KRT32
19	41310001	41360000	1.179180045	LOC102179515
16	43160001	43210000	1.179122196	RERE
13	63670001	63720000	1.179112791	NCOA6
5	97820001	97870000	1.179048754	LOC108636058
2	115550001	115600000	1.179036338	MIR10B

2	115550001	115600000	1.179036338	HOXD1
2	115550001	115600000	1.179036338	HOXD3
3	85660001	85710000	1.178990905	TRNAE-UUC-12
3	85660001	85710000	1.178990905	LOC102187823
20	59300001	59350000	1.178961152	DNAH5
1	157140001	157190000	1.178908811	KAT2B
10	87810001	87860000	1.178891082	LCTL
4	12290001	12340000	1.178723956	TRNAC-GCA-71
4	115590001	115640000	1.178636863	COBL
7	60210001	60260000	1.178591293	SIL1
7	60210001	60260000	1.178591293	CTNNA1
4	48570001	48620000	1.178578996	NPY
11	71610001	71660000	1.178498401	MRPL33
11	71610001	71660000	1.178498401	RBKS
10	31420001	31470000	1.178495494	CCDC175
10	31420001	31470000	1.178495494	JKAMP
7	27620001	27670000	1.17845382	XRCC4
7	27620001	27670000	1.17845382	TMEM167A
4	48590001	48640000	1.17808985	NPY
8	39220001	39270000	1.177995119	INSL6
8	39220001	39270000	1.177995119	JAK2
18	64860001	64910000	1.177917715	NDUFA3
18	64860001	64910000	1.177917715	LOC102171541
18	64860001	64910000	1.177917715	LOC102168291
18	64860001	64910000	1.177917715	OSCAR
18	64860001	64910000	1.177917715	TARM1
20	2940001	2990000	1.177900508	RANBP17
23	19470001	19520000	1.177874813	ZSCAN9
23	19470001	19520000	1.177874813	ZKSCAN4
23	19470001	19520000	1.177874813	NKAPL
23	19470001	19520000	1.177874813	ZSCAN26
23	19470001	19520000	1.177874813	PGBD1
10	76060001	76110000	1.177735075	LOC102183422
10	76060001	76110000	1.177735075	TRNAT-UGU-2
10	76060001	76110000	1.177735075	LOC108636909
3	46620001	46670000	1.177613472	PTGER3
14	12770001	12820000	1.177604674	TP53INP1
14	12770001	12820000	1.177604674	LOC102184166
23	19590001	19640000	1.177600632	ZSCAN12
23	19590001	19640000	1.177600632	ZSCAN23
23	19590001	19640000	1.177600632	TRNAF-GAA-18
23	19590001	19640000	1.177600632	LOC102168401
23	19590001	19640000	1.177600632	TRNAT-UGU-9
23	19590001	19640000	1.177600632	TRNAL-AAG-6
23	19590001	19640000	1.177600632	TRNAM-CAU-30
19	34260001	34310000	1.1774905	MIR33B
19	34260001	34310000	1.1774905	SREBF1
19	34260001	34310000	1.1774905	RAI1
2	19500001	19550000	1.177459993	SPHKAP
4	75390001	75440000	1.177386097	RELN
18	64760001	64810000	1.17733215	TSEN34
18	64760001	64810000	1.17733215	LOC106501822
18	64760001	64810000	1.17733215	RPS9
18	64760001	64810000	1.17733215	MBOAT7
18	64760001	64810000	1.17733215	TMC4
8	410001	460000	1.177313203	ANXA10
14	12560001	12610000	1.177208553	TRNAG-CCC-57
14	12560001	12610000	1.177208553	ESRP1

6	67520001	67570000	1.177192693	TEC
24	43710001	43760000	1.177151469	LDLRAD4
7	58880001	58930000	1.177132775	TRNAC-GCA-118
7	58880001	58930000	1.177132775	SLC4A9
7	58880001	58930000	1.177132775	LOC102175120
26	28160001	28210000	1.177075686	AS3MT
26	28160001	28210000	1.177075686	CNNM2
22	40370001	40420000	1.177038341	FHIT
14	12640001	12690000	1.177033006	DPY19L4
23	22340001	22390000	1.176893676	LY6G5C
23	22340001	22390000	1.176893676	LOC102180547
23	22340001	22390000	1.176893676	LY6G6D
23	22340001	22390000	1.176893676	LY6G6C
23	22340001	22390000	1.176893676	C23H6orf25
23	22340001	22390000	1.176893676	CLIC1
23	22340001	22390000	1.176893676	ABHD16A
23	22340001	22390000	1.176893676	LY6G6F
23	22340001	22390000	1.176893676	DDAH2
22	60200001	60250000	1.176861889	KLF15
18	39720001	39770000	1.17684158	LOC108637979
10	34640001	34690000	1.176763562	PELI2
4	75590001	75640000	1.176670939	RELN
6	108910001	108960000	1.17658091	RAB28
26	45890001	45940000	1.176517685	PCDH15
13	9660001	9710000	1.176369695	MACROD2
8	55400001	55450000	1.176282381	TLE4
24	14140001	14190000	1.176201131	PIK3C3
25	18490001	18540000	1.176173192	DCUN1D3
5	23370001	23420000	1.176153677	CRADD
1	157210001	157260000	1.176114862	SGO1
12	17920001	17970000	1.176064842	GPC6
5	58860001	58910000	1.176053457	SNRPF
5	58860001	58910000	1.176053457	CCDC38
14	12580001	12630000	1.176007016	TRNAG-CCC-57
14	12580001	12630000	1.176007016	DPY19L4
14	12580001	12630000	1.176007016	ESRP1
9	14310001	14360000	1.175845533	NKAIN2
1	146480001	146530000	1.175843632	FBXO25
1	146480001	146530000	1.175843632	TDRP
7	48640001	48690000	1.175808151	RPS14
7	48640001	48690000	1.175808151	CD74
7	48640001	48690000	1.175808151	TCOF1
7	48640001	48690000	1.175808151	LOC108636348
3	25630001	25680000	1.175792349	C3H1orf185
13	63680001	63730000	1.175694196	LOC108637356
13	63680001	63730000	1.175694196	NCOA6
5	46890001	46940000	1.175652752	IRAK3
5	33490001	33540000	1.175517203	SLC38A1
12	15430001	15480000	1.175381828	LOC102180583
12	15440001	15490000	1.175381828	LOC102180583
12	15440001	15490000	1.175381828	LOC102180841
12	15450001	15500000	1.175381828	LOC102180583
12	15450001	15500000	1.175381828	LOC102180841
23	24030001	24080000	1.175306594	LOC102174170
23	24030001	24080000	1.175306594	LOC102173702
15	6180001	6230000	1.175298526	LRP4
15	6180001	6230000	1.175298526	CKAP5
24	50720001	50770000	1.175259873	SMAD4

21	28350001	28400000	1.175247823	TARSL2
1	112920001	112970000	1.17510608	TRNAE-UUC-2
1	112920001	112970000	1.17510608	GPR149
1	112920001	112970000	1.17510608	DHX36
23	28230001	28280000	1.175099759	CD2AP
1	35900001	35950000	1.175075968	EPHA3
4	75410001	75460000	1.174998429	RELN
6	95420001	95470000	1.174937366	FGF5
4	65170001	65220000	1.174929641	TRNAG-CCC-25
15	39620001	39670000	1.174782219	SBF2
29	540001	590000	1.174672405	C29H11orf54
29	540001	590000	1.174672405	TAF1D
29	540001	590000	1.174672405	MED17
11	72340001	72390000	1.174641445	LOC108637155
11	72340001	72390000	1.174641445	MAPRE3
12	33720001	33770000	1.17443932	MYCBP2
13	71640001	71690000	1.174433547	SRSF6
13	71640001	71690000	1.174433547	L3MBTL1
16	78900001	78950000	1.174430902	ARL8A
16	78900001	78950000	1.174430902	GPR37L1
16	78900001	78950000	1.174430902	PTPN7
1	21370001	21420000	1.174373227	HSPA13
1	21370001	21420000	1.174373227	LOC102181811
7	27500001	27550000	1.174330136	XRCC4
14	12760001	12810000	1.174180537	TP53INP1
10	94580001	94630000	1.174073191	ANKRD31
13	53360001	53410000	1.173947228	ARFRP1
13	53360001	53410000	1.173947228	TRNAS-GGA-73
13	53360001	53410000	1.173947228	TNFRSF6B
13	53360001	53410000	1.173947228	STMN3
13	53360001	53410000	1.173947228	ZGPAT
13	53360001	53410000	1.173947228	RTEL1
5	72630001	72680000	1.173926672	RBFOX2
10	30490001	30540000	1.17385049	SIX6
10	30490001	30540000	1.17385049	C10H14orf39
10	88620001	88670000	1.173817729	DENND4A
28	8160001	8210000	1.173762533	NRG3
28	18810001	18860000	1.173746774	ADAMTS14
21	15310001	15360000	1.173510628	SV2B
4	5120001	5170000	1.173366907	KMT2C
27	39130001	39180000	1.173294357	MCPH1
1	93960001	94010000	1.173264465	SPATA16
20	2930001	2980000	1.173202936	RANBP17
1	70620001	70670000	1.173200276	PCYT1A
1	70620001	70670000	1.173200276	TM4SF19
1	70620001	70670000	1.173200276	TCTEX1D2
7	60220001	60270000	1.173191729	SIL1
7	60220001	60270000	1.173191729	CTNNA1
2	83930001	83980000	1.173095574	GTDC1
18	10050001	10100000	1.172960291	MPHOSPH6
11	23710001	23760000	1.172957081	TRNAG-UCC-23
8	55420001	55470000	1.172951244	TLE4
23	37930001	37980000	1.172950035	C23H6orf89
23	37930001	37980000	1.172950035	PPIL1
7	48160001	48210000	1.172946577	ZNF300
10	87960001	88010000	1.172935796	MAP2K1
24	43780001	43830000	1.172541683	FAM210A
24	43780001	43830000	1.172541683	RNMT

24	43780001	43830000	1.172541683	LDLRAD4
24	43600001	43650000	1.172342953	CEP192
24	43600001	43650000	1.172342953	LDLRAD4
26	29850001	29900000	1.172334667	MRPL43
26	29850001	29900000	1.172334667	SEMA4G
26	29850001	29900000	1.172334667	C26H10orf2
26	29850001	29900000	1.172334667	SLF2
23	21510001	21560000	1.17232933	RPP21
23	21510001	21560000	1.17232933	LOC102189637
23	21510001	21560000	1.17232933	LOC102188814
24	43530001	43580000	1.172301776	SEH1L
24	43530001	43580000	1.172301776	CEP192
12	59050001	59100000	1.172288477	STARD13
15	32370001	32420000	1.172262786	LOC102177261
15	32370001	32420000	1.172262786	TRNAG-CCC-64
9	15730001	15780000	1.172258736	PKIB
2	97300001	97350000	1.172220488	ACVR1C
2	23110001	23160000	1.172169958	CUL3
29	610001	660000	1.172036002	CEP295
16	76180001	76230000	1.171967851	PTPRC
11	71600001	71650000	1.171958003	RBKS
23	22370001	22420000	1.171914836	LY6G6D
23	22370001	22420000	1.171914836	LY6G6C
23	22370001	22420000	1.171914836	C23H6orf25
23	22370001	22420000	1.171914836	CLIC1
23	22370001	22420000	1.171914836	SAPCD1
23	22370001	22420000	1.171914836	VARS
23	22370001	22420000	1.171914836	DDAH2
23	22370001	22420000	1.171914836	MSH5
23	22370001	22420000	1.171914836	VWA7
23	22360001	22410000	1.171866958	LOC102180547
23	22360001	22410000	1.171866958	LY6G6D
23	22360001	22410000	1.171866958	LY6G6C
23	22360001	22410000	1.171866958	C23H6orf25
23	22360001	22410000	1.171866958	CLIC1
23	22360001	22410000	1.171866958	SAPCD1
23	22360001	22410000	1.171866958	LY6G6F
23	22360001	22410000	1.171866958	DDAH2
23	22360001	22410000	1.171866958	MSH5
23	22360001	22410000	1.171866958	VWA7
5	80410001	80460000	1.171759657	PTHLH
5	23400001	23450000	1.171656428	CRADD
5	23400001	23450000	1.171656428	LOC106502085
26	29880001	29930000	1.171563743	SLF2
23	22350001	22400000	1.171278756	LOC102180547
23	22350001	22400000	1.171278756	LY6G6D
23	22350001	22400000	1.171278756	LY6G6C
23	22350001	22400000	1.171278756	C23H6orf25
23	22350001	22400000	1.171278756	CLIC1
23	22350001	22400000	1.171278756	ABHD16A
23	22350001	22400000	1.171278756	LY6G6F
23	22350001	22400000	1.171278756	DDAH2
23	22350001	22400000	1.171278756	MSH5
16	76170001	76220000	1.171252642	PTPRC
1	70610001	70660000	1.171249692	PCYT1A
1	70610001	70660000	1.171249692	TCTEX1D2
7	89110001	89160000	1.171090057	IZUMO4
7	89110001	89160000	1.171090057	MOB3A

7	89110001	89160000	1.171090057	AP3D1
3	95380001	95430000	1.171075061	SPAG17
8	100980001	101030000	1.171026981	UGCG
18	35410001	35460000	1.171010548	CDH5
20	33720001	33770000	1.170929212	PTGER4
20	33720001	33770000	1.170929212	TTC33
7	27560001	27610000	1.17071553	XRCC4
4	99890001	99940000	1.170692818	SCIN
24	43840001	43890000	1.170664252	MC5R
24	43840001	43890000	1.170664252	RNMT
7	58870001	58920000	1.170629497	LOC102175120
3	95410001	95460000	1.170591621	SPAG17
5	63680001	63730000	1.170591024	ANO4
5	63680001	63730000	1.170591024	SLC5A8
5	58840001	58890000	1.170534178	NTN4
6	59390001	59440000	1.17052097	UBE2K
14	4040001	4090000	1.170498467	RALYL
24	43590001	43640000	1.170486316	CEP192
24	14120001	14170000	1.170296816	PIK3C3
7	91530001	91580000	1.170138889	PTPRS
18	39630001	39680000	1.170123358	LOC108637979
4	180001	230000	1.170116595	VIPR2
9	82440001	82490000	1.169983137	TRNAE-UUC-39
9	82440001	82490000	1.169983137	EZR
9	82440001	82490000	1.169983137	SYTL3
10	76030001	76080000	1.169960619	LOC108636866
10	76030001	76080000	1.169960619	LOC102183422
12	59060001	59110000	1.169942962	STARD13
15	25820001	25870000	1.169924303	ANO3
11	69740001	69790000	1.16991656	ALK
22	16200001	16250000	1.169807324	ZNF852
22	16200001	16250000	1.169807324	LOC102177570
4	111470001	111520000	1.169770194	LOC108635860
20	2900001	2950000	1.169712232	RANBP17
14	61020001	61070000	1.169704863	PCMTD1
11	72350001	72400000	1.169656237	LOC108637155
11	72350001	72400000	1.169656237	MAPRE3
12	15610001	15660000	1.16959213	LOC102180841
14	60990001	61040000	1.16957296	PCMTD1
14	16090001	16140000	1.169572051	RIDA
14	16090001	16140000	1.169572051	POP1
24	43670001	43720000	1.16949201	LDLRAD4
6	22330001	22380000	1.169405865	MANBA
11	85640001	85690000	1.169397755	LPIN1
5	20740001	20790000	1.16937803	KERA
5	20740001	20790000	1.16937803	LUM
6	80520001	80570000	1.169366645	TECRL
27	25560001	25610000	1.169257186	VPS37A
27	25560001	25610000	1.169257186	CNOT7
27	25560001	25610000	1.169257186	MTMR7
14	12040001	12090000	1.16923582	CDH17
22	28810001	28860000	1.169233345	SHQ1
7	89090001	89140000	1.169214774	IZUMO4
7	89090001	89140000	1.169214774	MOB3A
7	89090001	89140000	1.169214774	AP3D1
1	157280001	157330000	1.169211676	LOC108636388
1	157280001	157330000	1.169211676	LOC102184668
21	31550001	31600000	1.169069189	SCAPER

8	60870001	60920000	1.169017122	ZCCHC7
18	64850001	64900000	1.16894319	TFPT
18	64850001	64900000	1.16894319	NDUFA3
18	64850001	64900000	1.16894319	LOC102171541
18	64850001	64900000	1.16894319	PRPF31
18	64850001	64900000	1.16894319	OSCAR
18	64850001	64900000	1.16894319	TARM1
23	37500001	37550000	1.168900364	RNF8
5	46950001	47000000	1.168889984	LLPH
5	46950001	47000000	1.168889984	TMBIM4
5	46950001	47000000	1.168889984	IRAK3
24	43760001	43810000	1.16883375	FAM210A
24	43760001	43810000	1.16883375	LDLRAD4
12	59040001	59090000	1.168800257	STARD13
13	53540001	53590000	1.168528369	KCNQ2
13	53540001	53590000	1.168528369	CHRNA4
13	53540001	53590000	1.168528369	COL20A1
9	14340001	14390000	1.168417388	NKAIN2
4	75400001	75450000	1.168316692	RELN
18	10040001	10090000	1.168256935	MPHOSPH6
24	42230001	42280000	1.168245839	TXNDC2
24	42230001	42280000	1.168245839	VAPA
23	28240001	28290000	1.168111425	LOC108633483
23	28240001	28290000	1.168111425	CD2AP
16	53700001	53750000	1.167969205	CENPL
16	53700001	53750000	1.167969205	DARS2
8	60890001	60940000	1.167956821	ZCCHC7
2	23160001	23210000	1.167934422	CUL3
5	68710001	68760000	1.167921286	RFX4
5	68710001	68760000	1.167921286	RIC8B
7	27510001	27560000	1.167916265	XRCC4
11	27430001	27480000	1.167909955	SRBD1
10	99670001	99720000	1.167882071	EPB41L4A
2	18440001	18490000	1.16782953	PID1
16	30820001	30870000	1.16782152	EFCAB2
8	12840001	12890000	1.167797356	TRNAW-CCA-44
8	39270001	39320000	1.167791899	JAK2
2	18410001	18460000	1.16770381	PID1
21	28340001	28390000	1.167694453	TRNAS-GCU-9
21	28340001	28390000	1.167694453	TARSL2
12	33730001	33780000	1.167691322	MYCBP2
18	56280001	56330000	1.16763818	SPACA4
18	56280001	56330000	1.16763818	RPL18
18	56280001	56330000	1.16763818	DBP
18	56280001	56330000	1.16763818	TRNAE-UUC-76
18	56280001	56330000	1.16763818	NTN5
18	56280001	56330000	1.16763818	LOC102178850
18	56280001	56330000	1.16763818	FAM83E
18	56280001	56330000	1.16763818	SPHK2
18	56280001	56330000	1.16763818	CA11
21	19020001	19070000	1.167625081	LOC102175848
21	19020001	19070000	1.167625081	LOC108638532
14	40150001	40200000	1.167623747	ZC2HC1A
8	64280001	64330000	1.16755239	LOC106502417
20	2910001	2960000	1.167428564	RANBP17
24	43770001	43820000	1.167391767	FAM210A
24	43770001	43820000	1.167391767	LDLRAD4
14	12740001	12790000	1.167384828	CCNE2

14	12740001	12790000	1.167384828	INTS8
26	30940001	30990000	1.167316337	LOC102179346
26	30940001	30990000	1.167316337	ENTPD7
26	30940001	30990000	1.167316337	CUTC
10	22250001	22300000	1.167152803	DCAF5
5	14800001	14850000	1.167099755	ALX1
11	71620001	71670000	1.166913675	MRPL33
11	71620001	71670000	1.166913675	RBKS
14	14810001	14860000	1.166884365	TRNAC-GCA-178
14	14810001	14860000	1.166884365	CPQ
1	157220001	157270000	1.166883258	SGO1
14	12750001	12800000	1.166859122	CCNE2
14	12750001	12800000	1.166859122	TP53INP1
5	75050001	75100000	1.166704683	SYT10
18	39730001	39780000	1.166629223	LOC108637979
18	64870001	64920000	1.16652665	LOC102171541
18	64870001	64920000	1.16652665	LOC102168291
18	64870001	64920000	1.16652665	LOC102171271
18	64870001	64920000	1.16652665	OSCAR
18	64870001	64920000	1.16652665	TARM1
24	14130001	14180000	1.16651269	PIK3C3
6	4620001	4670000	1.166356252	TNIP3
7	26740001	26790000	1.166221914	EDIL3
23	37940001	37990000	1.166078739	C23H6orf89
23	37940001	37990000	1.166078739	PPIL1
24	14010001	14060000	1.166048237	PIK3C3
21	18940001	18990000	1.166027194	LOC108633276
21	18940001	18990000	1.166027194	LOC102177379
4	4830001	4880000	1.165995798	XRCC2
7	27520001	27570000	1.165971666	XRCC4
7	107300001	107350000	1.16593161	LOC102172985
7	107300001	107350000	1.16593161	LOC102172703
7	107300001	107350000	1.16593161	LOC102172529
16	43380001	43430000	1.165886359	RERE
10	98780001	98830000	1.165842532	YTHDC2
9	88970001	89020000	1.16583749	RPS6KA2
5	81060001	81110000	1.165702012	ARNTL2
2	39310001	39360000	1.16558841	PIKFYVE
7	107270001	107320000	1.165577759	LTC4S
7	107270001	107320000	1.165577759	MGAT4B
7	107270001	107320000	1.165577759	LOC102172703
4	4820001	4870000	1.165399758	XRCC2
15	39690001	39740000	1.165399661	SBF2
12	42030001	42080000	1.165390996	KLHL1
1	42140001	42190000	1.165377054	LOC102172099
1	42140001	42190000	1.165377054	LOC102188254
1	42140001	42190000	1.165377054	LOC102172388
11	27440001	27490000	1.165329421	SRBD1
13	71670001	71720000	1.165274292	L3MBTL1
13	71670001	71720000	1.165274292	SGK2
9	15720001	15770000	1.165192864	PKIB
3	95300001	95350000	1.165167286	WDR3
3	95300001	95350000	1.165167286	SPAG17
12	42020001	42070000	1.164938968	KLHL1
20	20100001	20150000	1.164891622	PDE4D
10	88610001	88660000	1.164797306	DENND4A
5	23380001	23430000	1.164766065	CRADD
6	21720001	21770000	1.164619124	TACR3

5	87660001	87710000	1.164586357	SLCO1C1
6	67500001	67550000	1.164527364	TEC
12	33710001	33760000	1.164477441	MYCBP2
17	22280001	22330000	1.164433072	TMEM132C
9	30600001	30650000	1.16441751	QRSL1
9	30600001	30650000	1.16441751	RTN4IP1
19	25990001	26040000	1.164416678	RABEP1
18	10030001	10080000	1.164279347	MPHOSPH6
18	10030001	10080000	1.164279347	HSD17B2
10	98800001	98850000	1.1641496	YTHDC2
6	37070001	37120000	1.164088123	ABCG2
4	75420001	75470000	1.164015594	RELN
5	97810001	97860000	1.16388727	LOC108636058
5	97810001	97860000	1.16388727	LOC102182573
3	7030001	7080000	1.163813785	SPP2
21	6280001	6330000	1.163563524	MEF2A
18	10060001	10110000	1.163531239	MPHOSPH6
11	72360001	72410000	1.163517736	LOC108637155
11	72360001	72410000	1.163517736	DPYSL5
11	72360001	72410000	1.163517736	MAPRE3
18	64730001	64780000	1.163469539	LOC102172431
18	64730001	64780000	1.163469539	LOC106501822
5	80400001	80450000	1.16340314	PTHLH
12	17900001	17950000	1.163331917	GPC6
10	87850001	87900000	1.163323203	LCTL
10	87850001	87900000	1.163323203	ZWILCH
16	36050001	36100000	1.163239143	SCYL3
9	54760001	54810000	1.163228713	LAMA2
26	34450001	34500000	1.163199768	TCTN3
26	34450001	34500000	1.163199768	ALDH18A1
11	72690001	72740000	1.163182764	CIB4
11	72690001	72740000	1.163182764	C11H2orf70
11	72690001	72740000	1.163182764	OTOF
1	152700001	152750000	1.163139912	ANKRD28
10	94600001	94650000	1.163127137	ANKRD31
26	16140001	16190000	1.163061574	ATRNL1
1	135650001	135700000	1.163045146	TMEM108
7	81600001	81650000	1.162991736	ZNF608
25	18420001	18470000	1.162968084	ERI2
25	18420001	18470000	1.162968084	LOC102184112
24	43740001	43790000	1.162910671	LDLRAD4
3	31160001	31210000	1.162901612	PLPP3
24	43750001	43800000	1.162890275	LDLRAD4
18	58810001	58860000	1.162858089	LOC108638020
18	58810001	58860000	1.162858089	ZNF613
18	58810001	58860000	1.162858089	ZNF432
18	58810001	58860000	1.162858089	ZNF614
1	40500001	40550000	1.162661593	EPHA6
21	36330001	36380000	1.162653658	NOVA1
11	78240001	78290000	1.162643963	PUM2
21	18930001	18980000	1.162629404	LOC108633276
21	18930001	18980000	1.162629404	LOC102177379
11	10730001	10780000	1.1625967	TPRKB
11	10730001	10780000	1.1625967	NAT8
11	10730001	10780000	1.1625967	ALMS1
20	3150001	3200000	1.162552444	RANBP17
19	20600001	20650000	1.16230173	TP53I13
19	20600001	20650000	1.16230173	ABHD15

19	20600001	20650000	1.16230173	TAOK1
2	23120001	23170000	1.162234601	CUL3
6	116620001	116670000	1.162206404	SH3BP2
6	116620001	116670000	1.162206404	ADD1
6	65110001	65160000	1.16215938	GABRG1
1	52220001	52270000	1.162033864	BBX
11	44360001	44410000	1.161808111	LIMS1
11	44360001	44410000	1.161808111	GCC2
12	33740001	33790000	1.161792128	MYCBP2
18	39640001	39690000	1.161786671	LOC108637979
13	71650001	71700000	1.161761092	SRSF6
13	71650001	71700000	1.161761092	L3MBTL1
23	37510001	37560000	1.161759311	RNF8
23	37510001	37560000	1.161759311	TBC1D22B
4	7710001	7760000	1.161756677	LOC106501766
4	7710001	7760000	1.161756677	LOC102168236
28	100001	150000	1.161683808	LOC108634156
4	7750001	7800000	1.16143982	GIMAP8
6	22340001	22390000	1.161422772	MANBA
3	7010001	7060000	1.161369022	SPP2
7	107280001	107330000	1.161339777	LOC102172985
7	107280001	107330000	1.161339777	LTC4S
7	107280001	107330000	1.161339777	MGAT4B
7	107280001	107330000	1.161339777	LOC102172703
1	96690001	96740000	1.161301469	PRKCI
1	96690001	96740000	1.161301469	PHC3
4	7740001	7790000	1.161263131	GIMAP8
24	42240001	42290000	1.161259876	VAPA
25	190001	240000	1.161123327	RGS11
25	190001	240000	1.161123327	ARHGDIG
25	190001	240000	1.161123327	LUC7L
25	190001	240000	1.161123327	FAM234A
14	12050001	12100000	1.161035574	CDH17
7	36930001	36980000	1.160837317	GABRA1
13	37910001	37960000	1.160823696	POLR3F
13	37910001	37960000	1.160823696	RBBP9
13	37910001	37960000	1.160823696	SEC23B
13	37910001	37960000	1.160823696	DZANK1
19	50790001	50840000	1.160768918	SLC38A10
7	48150001	48200000	1.160759474	ZNF300
2	19480001	19530000	1.160684818	SPHKAP
7	19730001	19780000	1.160557346	LOC108636454
25	9580001	9630000	1.160498175	CLEC16A
18	39650001	39700000	1.160462414	LOC108637979
11	45460001	45510000	1.16029885	NCK2
18	56990001	57040000	1.160289997	PRRG2
18	56990001	57040000	1.160289997	NOSIP
18	56990001	57040000	1.160289997	PRR12
22	28870001	28920000	1.160274599	SHQ1
16	78890001	78940000	1.160270865	ARL8A
16	78890001	78940000	1.160270865	LOC102168445
16	78890001	78940000	1.160270865	GPR37L1
16	78890001	78940000	1.160270865	PTPN7
9	88950001	89000000	1.160242728	RPS6KA2
19	12400001	12450000	1.160178454	CA4
19	12400001	12450000	1.160178454	USP32
14	12540001	12590000	1.160167953	ESRP1
25	18500001	18550000	1.160073389	DCUN1D3

25	18500001	18550000	1.160073389	LYRM1
4	6480001	6530000	1.16003978	TMEM176B
4	6480001	6530000	1.16003978	TMEM176A
29	42980001	43030000	1.16001605	GPR137
29	42980001	43030000	1.16001605	KCNK4
29	42980001	43030000	1.16001605	ESRRA
29	42980001	43030000	1.16001605	BAD
29	42980001	43030000	1.16001605	TEX40
29	42980001	43030000	1.16001605	PLCB3
18	39710001	39760000	1.159852675	LOC108637979
11	23720001	23770000	1.159773674	TRNAG-UCC-23
5	18670001	18720000	1.159761172	TRNAC-GCA-83
3	54070001	54120000	1.15969014	AK5
3	54070001	54120000	1.15969014	ZZZ3
11	44350001	44400000	1.159669622	LIMS1
5	35040001	35090000	1.1595899	NELL2
21	6270001	6320000	1.159445776	MEF2A
5	33450001	33500000	1.159324472	SLC38A1
3	68920001	68970000	1.159292651	TGFBR3
16	40510001	40560000	1.159277353	FBXO2
16	40510001	40560000	1.159277353	LOC102184360
16	40510001	40560000	1.159277353	LOC102183714
25	9890001	9940000	1.15920843	LOC102173909
18	14500001	14550000	1.159116459	JPH3
10	31400001	31450000	1.159033879	CCDC175
18	35420001	35470000	1.158980088	CDH5
19	34270001	34320000	1.158978986	MIR33B
19	34270001	34320000	1.158978986	SREBF1
19	34270001	34320000	1.158978986	RAI1
3	100500001	100550000	1.158957253	TNFAIP8L2
3	100500001	100550000	1.158957253	LYSMD1
3	100500001	100550000	1.158957253	SCNM1
3	100500001	100550000	1.158957253	VPS72
3	100500001	100550000	1.158957253	TMOD4
3	100500001	100550000	1.158957253	PIP5K1A
7	70600001	70650000	1.158943838	LOC102170224
7	70600001	70650000	1.158943838	MGAT1
21	19010001	19060000	1.158935555	LOC108638488
21	19010001	19060000	1.158935555	LOC108638532
16	40520001	40570000	1.15889524	FBXO2
24	43610001	43660000	1.158777033	CEP192
24	43610001	43660000	1.158777033	LDLRAD4
6	108890001	108940000	1.158770015	RAB28
14	40160001	40210000	1.158744017	ZC2HC1A
23	24020001	24070000	1.15873851	LOC102174170
23	24020001	24070000	1.15873851	LOC102173702
11	78230001	78280000	1.158715722	PUM2
21	20060001	20110000	1.158645411	TRNAR-UCG-3
21	20060001	20110000	1.158645411	POLG
21	20060001	20110000	1.158645411	FANCI
20	3130001	3180000	1.158634613	RANBP17
1	52230001	52280000	1.158598892	BBX
2	7150001	7200000	1.158569539	IL22RA1
2	7150001	7200000	1.158569539	IFNLR1
12	20000001	20050000	1.158563884	GPC5
4	75370001	75420000	1.158549697	RELN
17	11040001	11090000	1.158549656	TBX3
17	11040001	11090000	1.158549656	LOC106503025

24	43520001	43570000	1.158541303	SEH1L
24	43520001	43570000	1.158541303	CEP192
15	28930001	28980000	1.158471376	RNF169
10	87950001	88000000	1.158337413	MAP2K1
11	72680001	72730000	1.158287009	CIB4
11	72680001	72730000	1.158287009	C11H2orf70
23	24190001	24240000	1.158262432	TRAM2
8	55410001	55460000	1.15824009	TLE4
4	99900001	99950000	1.158238158	SCIN
4	7730001	7780000	1.158221519	LOC102168236
26	28130001	28180000	1.158089957	CNNM2
23	28190001	28240000	1.158078373	CD2AP
5	23190001	23240000	1.157981247	CRADD
23	24040001	24090000	1.157721712	LOC102173702
23	24040001	24090000	1.157721712	TMEM14A
12	15040001	15090000	1.157720775	LOC102181111
2	18400001	18450000	1.157717801	PID1
5	45830001	45880000	1.157700418	LOC108636007
5	45830001	45880000	1.157700418	CAND1
27	30390001	30440000	1.157682755	PRIMPOL
27	30390001	30440000	1.157682755	CENPU
5	112030001	112080000	1.157642707	LOC102169285
5	112030001	112080000	1.157642707	TCF20
5	112030001	112080000	1.157642707	LOC102177333
5	112030001	112080000	1.157642707	LOC102169002
4	9640001	9690000	1.157546304	CNTNAP2
9	54780001	54830000	1.157538913	LAMA2
6	108920001	108970000	1.157532571	RAB28
24	43730001	43780000	1.157510197	LDLRAD4
15	39610001	39660000	1.157504311	SBF2
10	94680001	94730000	1.157456395	LOC108636845
10	94680001	94730000	1.157456395	GCNT4
10	94680001	94730000	1.157456395	ANKRD31
20	2920001	2970000	1.157429547	RANBP17
10	43950001	44000000	1.15740302	LOC102172726
16	24500001	24550000	1.157377418	TRNAT-UGU-6
16	24500001	24550000	1.157377418	HHIPL2
10	97770001	97820000	1.157319512	KCNN2
1	96740001	96790000	1.157246498	LOC102179814
1	96740001	96790000	1.157246498	PHC3
9	76900001	76950000	1.157234498	MYCT1
11	68240001	68290000	1.157230998	TRNAG-CCC-51
11	68240001	68290000	1.157230998	C11H2orf42
11	68240001	68290000	1.157230998	TIA1
9	88960001	89010000	1.157182628	RPS6KA2
5	109440001	109490000	1.157109456	LOC106502146
5	109440001	109490000	1.157109456	LOC102184324
7	48650001	48700000	1.157091026	RPS14
7	48650001	48700000	1.157091026	CD74
7	48650001	48700000	1.157091026	TCOF1
11	85630001	85680000	1.15707268	LPIN1
19	25870001	25920000	1.157027347	LOC106503287
19	25870001	25920000	1.157027347	MIS12
19	25870001	25920000	1.157027347	DERL2
19	25870001	25920000	1.157027347	NLRP1
19	25870001	25920000	1.157027347	DHX33
18	62660001	62710000	1.157008127	LOC108638088
18	62670001	62720000	1.157008127	LOC108638088

16	79010001	79060000	1.156935921	IPO9
16	79010001	79060000	1.156935921	NAV1
16	79010001	79060000	1.156935921	LOC108637801
3	41120001	41170000	1.156919039	LEPR
10	34650001	34700000	1.156888439	PELI2
2	88110001	88160000	1.156861697	LOC106502907
2	88110001	88160000	1.156861697	ORC4
19	50780001	50830000	1.156860039	SLC38A10
18	39740001	39790000	1.156842013	LOC108637979
6	59400001	59450000	1.156764225	UBE2K
1	94010001	94060000	1.156742147	SPATA16
3	95400001	95450000	1.156728671	SPAG17
4	28510001	28560000	1.156456196	ZNF800
18	14570001	14620000	1.156443084	JPH3
13	55950001	56000000	1.156389041	SYCP2
5	81990001	82040000	1.156371811	ITPR2
16	43360001	43410000	1.156260855	RERE
8	44280001	44330000	1.156248917	PGM5
25	260001	310000	1.156165122	MRPL28
25	260001	310000	1.156165122	TMEM8A
25	260001	310000	1.156165122	AXIN1
6	46760001	46810000	1.156139845	STIM2
9	30590001	30640000	1.156137255	QRSL1
9	30590001	30640000	1.156137255	RTN4IP1
7	26730001	26780000	1.156035189	EDIL3
2	23150001	23200000	1.156014543	CUL3
1	116820001	116870000	1.156000048	FAM188B2
9	54750001	54800000	1.155999494	LAMA2
1	51230001	51280000	1.155995381	LOC108636831
6	63460001	63510000	1.15594693	KCTD8
6	114620001	114670000	1.155766158	ABLIM2
1	157190001	157240000	1.155584459	SGO1
20	23260001	23310000	1.155551476	IL6ST
26	30950001	31000000	1.155543457	LOC102179346
26	30950001	31000000	1.155543457	ENTPD7
26	30950001	31000000	1.155543457	CUTC
14	45050001	45100000	1.155438865	STAU2
4	114660001	114710000	1.155254915	C4H7orf72
4	114660001	114710000	1.155254915	ZPBP
3	85640001	85690000	1.155219984	LOC102187823
23	37920001	37970000	1.155205009	C23H6orf89
23	37920001	37970000	1.155205009	PPIL1
13	55960001	56010000	1.155179512	SYCP2
7	107290001	107340000	1.155131201	LOC102172985
7	107290001	107340000	1.155131201	MGAT4B
7	107290001	107340000	1.155131201	LOC102172703
7	107290001	107340000	1.155131201	LOC102172529
5	84510001	84560000	1.155054925	SOX5
19	49850001	49900000	1.155029701	LOC108638312
21	31560001	31610000	1.15502947	SCAPER
14	94360001	94410000	1.15497672	KHDC3L
14	94360001	94410000	1.15497672	LOC102180236
22	28800001	28850000	1.154825499	SHQ1
12	15600001	15650000	1.154800344	LOC102180841
24	43720001	43770000	1.154784031	LDLRAD4
1	42210001	42260000	1.154756001	LOC102188532
4	5150001	5200000	1.154600073	KMT2C
9	35710001	35760000	1.15454433	GRIK2

19	39150001	39200000	1.154514653	LOC106503226
19	39150001	39200000	1.154514653	FBXO47
23	19500001	19550000	1.15448039	NKAPL
23	19500001	19550000	1.15448039	ZSCAN26
23	19500001	19550000	1.15448039	PGBD1
3	34520001	34570000	1.154469915	FGGY
13	37190001	37240000	1.154456862	LOC102171702
13	37190001	37240000	1.154456862	LOC102171968
13	37190001	37240000	1.154456862	PCSK2
8	60860001	60910000	1.154440415	ZCCHC7
8	60860001	60910000	1.154440415	PAX5
10	26620001	26670000	1.154378515	ESR2
2	39200001	39250000	1.154345876	PTH2R
3	46610001	46660000	1.154319241	PTGER3
29	21770001	21820000	1.154103046	SLC17A6
5	80440001	80490000	1.154102398	PTHLH
26	28170001	28220000	1.154101545	AS3MT
26	28170001	28220000	1.154101545	CNNM2
10	97060001	97110000	1.154100795	CCDC112
26	32280001	32330000	1.154093925	TRNAE-UUC-96
26	32280001	32330000	1.154093925	R3HCC1L
2	114280001	114330000	1.15408387	CHN1
22	6720001	6770000	1.15408268	CMTM7
25	9900001	9950000	1.154081231	LOC102173909
1	157160001	157210000	1.154067535	SGO1
1	157160001	157210000	1.154067535	KAT2B
4	75770001	75820000	1.154034393	PMPCB
4	75770001	75820000	1.154034393	DNAJC2
21	64460001	64510000	1.154009882	MIR345
21	64460001	64510000	1.154009882	SLC25A47
21	64460001	64510000	1.154009882	YY1
21	64460001	64510000	1.154009882	SLC25A29
13	63690001	63740000	1.153951917	LOC108637356
13	63690001	63740000	1.153951917	GGT7
13	63690001	63740000	1.153951917	NCOA6
14	12630001	12680000	1.153922316	DPY19L4
9	57010001	57060000	1.15391987	ENPP1
16	36040001	36090000	1.153907874	LOC106502954
16	36040001	36090000	1.153907874	C16H1orf112
16	36040001	36090000	1.153907874	SCYL3
19	49880001	49930000	1.153867811	LOC108638312
19	49880001	49930000	1.153867811	LOC108638313
19	49880001	49930000	1.153867811	LOC108638314
19	49880001	49930000	1.153867811	CD7
15	6290001	6340000	1.153835321	ZNF408
15	6290001	6340000	1.153835321	F2
15	6290001	6340000	1.153835321	ARHGAP1
15	6290001	6340000	1.153835321	CKAP5
6	4590001	4640000	1.153811863	TNIP3
19	26840001	26890000	1.153595369	LOC102184370
19	26840001	26890000	1.153595369	KCTD11
19	26840001	26890000	1.153595369	TMEM95
19	26840001	26890000	1.153595369	TNK1
19	26840001	26890000	1.153595369	NEURL4
19	26840001	26890000	1.153595369	ACAP1
19	26840001	26890000	1.153595369	PLSCR3
12	59030001	59080000	1.153539262	STARD13
10	87900001	87950000	1.153430135	RPL4

10	87900001	87950000	1.153430135	LOC106502512
10	87900001	87950000	1.153430135	ZWILCH
10	87900001	87950000	1.153430135	SNAPC5
10	87900001	87950000	1.153430135	MAP2K1
5	33350001	33400000	1.153338543	LOC106502098
5	33350001	33400000	1.153338543	SLC38A2
13	37200001	37250000	1.153310078	LOC102171702
13	37200001	37250000	1.153310078	LOC102171968
13	37200001	37250000	1.153310078	PCSK2
27	29860001	29910000	1.153281012	CCDC110
27	29860001	29910000	1.153281012	C27H4orf47
27	29860001	29910000	1.153281012	LRP2BP
27	29860001	29910000	1.153281012	UFSP2
27	29860001	29910000	1.153281012	ANKRD37
26	32300001	32350000	1.153135117	R3HCC1L
1	157200001	157250000	1.153129224	SGO1
17	60420001	60470000	1.153081196	PRMT9
16	78870001	78920000	1.153080183	ARL8A
16	78870001	78920000	1.153080183	LOC102168445
16	78870001	78920000	1.153080183	PTPN7
22	23410001	23460000	1.152996472	CNTN4
11	44370001	44420000	1.152983221	LIMS1
11	44370001	44420000	1.152983221	GCC2
2	39190001	39240000	1.152962372	PTH2R
4	75580001	75630000	1.152876169	RELN
14	14790001	14840000	1.152872137	TRNAC-GCA-178
14	14790001	14840000	1.152872137	CPQ
13	33610001	33660000	1.15286703	ZNF438
27	25570001	25620000	1.152858541	VPS37A
27	25570001	25620000	1.152858541	MTMR7
19	47260001	47310000	1.152823877	TANC2
19	47270001	47320000	1.152811658	TANC2
19	39140001	39190000	1.152806124	LOC106503226
19	39140001	39190000	1.152806124	LASP1
19	39140001	39190000	1.152806124	FBXO47
10	43960001	44010000	1.152800829	LOC102172726
10	43960001	44010000	1.152800829	CYP19A1
12	15420001	15470000	1.152789426	LOC102180583
26	29890001	29940000	1.152736201	SLF2
4	7720001	7770000	1.152716623	LOC102168236
24	14150001	14200000	1.152708211	PIK3C3
1	70600001	70650000	1.152685536	PCYT1A
1	70600001	70650000	1.152685536	TCTEX1D2
5	14790001	14840000	1.152677066	ALX1
15	7950001	8000000	1.152675654	TP53I11
8	41540001	41590000	1.152659391	PUM3
18	35400001	35450000	1.152621679	CDH5
7	37380001	37430000	1.152513931	TRNAS-GGA-38
7	37380001	37430000	1.152513931	GABRB2
9	1860001	1910000	1.152472977	COL12A1
19	41300001	41350000	1.152463001	KRT35
19	41300001	41350000	1.152463001	KRT36
19	41300001	41350000	1.152463001	KRT32
4	48600001	48650000	1.152391536	NPY
26	45880001	45930000	1.152381562	PCDH15
25	9910001	9960000	1.152298608	LOC102173909
13	53370001	53420000	1.152208641	ARFRP1
13	53370001	53420000	1.152208641	TRNAS-GGA-73

13	53370001	53420000	1.152208641	TNFRSF6B
13	53370001	53420000	1.152208641	STMN3
13	53370001	53420000	1.152208641	RTEL1
13	53370001	53420000	1.152208641	GMEB2
9	69220001	69270000	1.152186213	UTRN
3	7020001	7070000	1.152181374	SPP2
16	43340001	43390000	1.152150115	RERE
1	14440001	14490000	1.152079651	NCAM2
12	57640001	57690000	1.15206411	FRY
19	49860001	49910000	1.152043811	LOC108638312
19	49860001	49910000	1.152043811	LOC108638313
19	49860001	49910000	1.152043811	LOC108638314
10	22240001	22290000	1.151990085	DCAF5
9	34540001	34590000	1.151938986	TRNAS-GGA-49
21	31580001	31630000	1.151903507	RCN2
21	31580001	31630000	1.151903507	SCAPER
28	8150001	8200000	1.151880045	NRG3
10	99650001	99700000	1.151751795	EPB41L4A
21	20050001	20100000	1.151741613	POLG
21	20050001	20100000	1.151741613	FANCI
11	105480001	105530000	1.151735259	CACNA1B
10	31430001	31480000	1.15172235	CCDC175
10	31430001	31480000	1.15172235	L3HYPDH
10	31430001	31480000	1.15172235	JKAMP
6	69370001	69420000	1.151706017	SCFD2
21	36320001	36370000	1.151684029	NOVA1
5	33360001	33410000	1.151597037	LOC106502098
5	33360001	33410000	1.151597037	SLC38A2
28	8170001	8220000	1.151468877	NRG3
10	87860001	87910000	1.151416686	RPL4
10	87860001	87910000	1.151416686	LCTL
10	87860001	87910000	1.151416686	ZWILCH
1	32630001	32680000	1.151374976	CADM2
5	97800001	97850000	1.15128356	LOC102182573
17	70290001	70340000	1.151231065	TLL1
13	53580001	53630000	1.151141148	ARFGAP1
13	53580001	53630000	1.151141148	COL20A1
4	64640001	64690000	1.151135214	BMT2
7	41380001	41430000	1.151095878	ADAM19
10	43940001	43990000	1.151089617	LOC102172726
18	58820001	58870000	1.151058954	ZNF613
18	58820001	58870000	1.151058954	ZNF432
18	58820001	58870000	1.151058954	ZNF614
29	470001	520000	1.151006633	VSTM5
29	470001	520000	1.151006633	MED17
3	100490001	100540000	1.150986356	TNFAIP8L2
3	100490001	100540000	1.150986356	LYSMD1
3	100490001	100540000	1.150986356	SCNM1
3	100490001	100540000	1.150986356	VPS72
3	100490001	100540000	1.150986356	SEMA6C
3	100490001	100540000	1.150986356	TMOD4
12	42010001	42060000	1.150945291	KLHL1
18	56950001	57000000	1.150941058	RPS11
18	56950001	57000000	1.150941058	MIR150
18	56950001	57000000	1.150941058	FCGRT
18	56950001	57000000	1.150941058	RPL13A
18	56950001	57000000	1.150941058	RCN3
18	56950001	57000000	1.150941058	NOSIP

10	99660001	99710000	1.150870342	EPB41L4A
16	41540001	41590000	1.150809097	PEX14
19	26830001	26880000	1.150805976	EIF5A
19	26830001	26880000	1.150805976	GPS2
19	26830001	26880000	1.150805976	LOC102184370
19	26830001	26880000	1.150805976	KCTD11
19	26830001	26880000	1.150805976	TMEM95
19	26830001	26880000	1.150805976	TNK1
19	26830001	26880000	1.150805976	NEURL4
19	26830001	26880000	1.150805976	ACAP1
5	63690001	63740000	1.150761917	SLC5A8
10	69070001	69120000	1.150752309	SPRED1
4	64630001	64680000	1.15074402	BMT2
4	64630001	64680000	1.15074402	TMEM168
6	46770001	46820000	1.15063937	STIM2
26	16150001	16200000	1.150637288	ATRNL1
12	33700001	33750000	1.150612934	MYCBP2
5	46970001	47020000	1.150526511	LLPH
5	46970001	47020000	1.150526511	TMBIM4
2	23140001	23190000	1.150525075	CUL3
11	60640001	60690000	1.150507491	TMEM17
19	47250001	47300000	1.15050436	TANC2
5	68700001	68750000	1.150477525	RFX4
16	78880001	78930000	1.150462622	ARL8A
16	78880001	78930000	1.150462622	LOC102168445
16	78880001	78930000	1.150462622	GPR37L1
16	78880001	78930000	1.150462622	PTPN7
12	60020001	60070000	1.150374908	LOC102178917
11	60660001	60710000	1.150371815	TMEM17
9	89330001	89380000	1.1503441	CCR6
10	43970001	44020000	1.150301898	LOC102172726
10	43970001	44020000	1.150301898	CYP19A1
2	23100001	23150000	1.150295286	CUL3
22	28790001	28840000	1.150277987	SHQ1
5	72620001	72670000	1.150203024	RBFOX2
5	81080001	81130000	1.150174762	ARNTL2
7	96350001	96400000	1.150096892	ADGRE2
15	28950001	29000000	1.150056812	CHRDL2
15	28950001	29000000	1.150056812	RNF169
25	9600001	9650000	1.150049408	CLEC16A
5	63670001	63720000	1.15003699	ANO4
5	63670001	63720000	1.15003699	SLC5A8
5	81070001	81120000	1.149994954	ARNTL2
4	28730001	28780000	1.149965232	GRM8
5	58870001	58920000	1.149883403	SNRPF
5	58870001	58920000	1.149883403	CCDC38
12	19380001	19430000	1.149877867	GPC5
10	89530001	89580000	1.149851525	TXNDC16
21	15320001	15370000	1.149837493	SV2B
14	12030001	12080000	1.149815532	CDH17
9	57000001	57050000	1.149762297	ENPP1
10	43980001	44030000	1.149728829	LOC102172726
10	43980001	44030000	1.149728829	CYP19A1
2	19470001	19520000	1.149715826	SPHKAP
24	14160001	14210000	1.149649868	PIK3C3
1	157330001	157380000	1.149616548	LOC102184668
16	53610001	53660000	1.149607633	KLHL20
16	53610001	53660000	1.149607633	ANKRD45

26	30540001	30590000	1.149591175	ERLIN1
26	30540001	30590000	1.149591175	CHUK
22	3830001	3880000	1.149523018	RBMS3
21	15210001	15260000	1.149472633	SV2B
21	55480001	55530000	1.149435507	GPR68
26	32070001	32120000	1.149431739	HPS1
26	32070001	32120000	1.149431739	HPSE2
9	77580001	77630000	1.149367025	LOC102190323
7	57430001	57480000	1.149346617	GNPDA1
7	57430001	57480000	1.149346617	RNF14
13	55940001	55990000	1.1493399	SYCP2
5	98650001	98700000	1.149303856	KLRF1
11	78260001	78310000	1.149301391	PUM2
2	39210001	39260000	1.149265208	PTH2R
18	53570001	53620000	1.149261799	BCL3
18	53570001	53620000	1.149261799	LOC108638000
18	53570001	53620000	1.149261799	CBLC
5	80420001	80470000	1.149248873	PTHLH
1	152690001	152740000	1.149211456	ANKRD28
1	70630001	70680000	1.149199271	TM4SF19
1	70630001	70680000	1.149199271	UBXN7
1	70630001	70680000	1.149199271	TCTEX1D2
3	110920001	110970000	1.149137346	SLAMF1
4	75430001	75480000	1.148951893	RELN
3	64200001	64250000	1.14894492	HS2ST1
17	8840001	8890000	1.148937273	MAPKAPK5
17	8840001	8890000	1.148937273	LOC108637850
12	60010001	60060000	1.14893481	LOC102178917
8	83370001	83420000	1.148914801	LOC102189675
26	30520001	30570000	1.14886877	CWF19L1
26	30520001	30570000	1.14886877	ERLIN1
26	30520001	30570000	1.14886877	CHUK
28	18830001	18880000	1.148836768	ADAMTS14
28	18830001	18880000	1.148836768	PRF1
5	18660001	18710000	1.148799979	TRNAC-GCA-83
28	15150001	15200000	1.148770018	ADK
16	26890001	26940000	1.148753364	DNAH14
14	4050001	4100000	1.148729797	RALYL
25	860001	910000	1.148696699	LOC108633901
25	860001	910000	1.148696699	LOC102177581
24	62250001	62300000	1.148673667	LOC102181552
24	62250001	62300000	1.148673667	LOC102181826
8	44290001	44340000	1.148631335	PGM5
24	43790001	43840000	1.148620111	FAM210A
24	43790001	43840000	1.148620111	RNMT
24	43790001	43840000	1.148620111	LDLRAD4
2	39300001	39350000	1.148574347	PIKFYVE
21	28360001	28410000	1.148418992	TARSL2
2	130190001	130240000	1.148389902	TRNAE-UUC-7
2	130190001	130240000	1.148389902	MSTN
13	65280001	65330000	1.14837963	SLA2
13	65280001	65330000	1.14837963	NDRG3
18	39620001	39670000	1.14837262	ZFHX3
18	39620001	39670000	1.14837262	LOC108637979
11	78250001	78300000	1.148316595	PUM2
1	50110001	50160000	1.148300947	CBLB
5	75060001	75110000	1.148258019	SYT10
1	51650001	51700000	1.148141807	LOC102171918

9	30270001	30320000	1.148139907	CD24
5	46990001	47040000	1.148119132	LLPH
5	46990001	47040000	1.148119132	TMBIM4
16	43390001	43440000	1.148117485	RERE
1	21380001	21430000	1.148097284	HSPA13
1	21380001	21430000	1.148097284	LOC102181811
10	30900001	30950000	1.148077638	PCNX4
10	30900001	30950000	1.148077638	LOC102176425
9	14300001	14350000	1.148073341	NKAIN2
3	41110001	41160000	1.148046295	LEPR
4	6470001	6520000	1.148036225	TMEM176B
4	6470001	6520000	1.148036225	TMEM176A
13	33600001	33650000	1.147854324	ZNF438
12	19990001	20040000	1.147707266	GPC5
11	78220001	78270000	1.147690614	PUM2
1	44150001	44200000	1.147685159	CMSS1
1	44150001	44200000	1.147685159	FILIP1L
17	69370001	69420000	1.147668573	CPE
5	109430001	109480000	1.147641522	LOC106502146
8	10060001	10110000	1.147601627	FBXO16
8	10060001	10110000	1.147601627	FZD3
4	39260001	39310000	1.147599873	SUGCT
4	7580001	7630000	1.147589498	LOC102186105
19	49870001	49920000	1.14756172	LOC108638312
19	49870001	49920000	1.14756172	LOC108638313
19	49870001	49920000	1.14756172	LOC108638314
19	49870001	49920000	1.14756172	CD7
7	60230001	60280000	1.147526868	SIL1
7	60230001	60280000	1.147526868	CTNNA1
17	26690001	26740000	1.147425889	PXMP2
17	26690001	26740000	1.147425889	PGAM5
17	26690001	26740000	1.147425889	ANKLE2
17	26690001	26740000	1.147425889	GOLGA3
25	2390001	2440000	1.147341615	LOC106503617
25	2390001	2440000	1.147341615	IL32
25	2390001	2440000	1.147341615	ZSCAN10
25	2390001	2440000	1.147341615	LOC108633915
25	2390001	2440000	1.147341615	ZNF205
10	94590001	94640000	1.147291959	ANKRD31
12	19370001	19420000	1.147289891	GPC5
4	12260001	12310000	1.147289581	TRNAS-GGA-21
6	85630001	85680000	1.147270059	LOC102168522
19	51560001	51610000	1.147243414	RNF213
5	82000001	82050000	1.147231221	ITPR2
4	114650001	114700000	1.1472089	ZPBP
10	88600001	88650000	1.147156144	DENND4A
21	31570001	31620000	1.147103474	RCN2
21	31570001	31620000	1.147103474	SCAPER
8	41530001	41580000	1.147066355	PUM3
6	46750001	46800000	1.147028949	STIM2
12	34000001	34050000	1.147021613	KCTD12
28	90001	140000	1.146865605	AGT
20	61790001	61840000	1.14678339	CTNND2
22	60160001	60210000	1.146760922	LOC106503466
22	60160001	60210000	1.146760922	ALDH1L1
5	19300001	19350000	1.146664023	ATP2B1
10	69080001	69130000	1.146655549	SPRED1
13	54340001	54390000	1.146610583	MTG2

13	54340001	54390000	1.146610583	LSM14B
13	54340001	54390000	1.146610583	SS18L1
13	54340001	54390000	1.146610583	PSMA7
13	37920001	37970000	1.146569834	POLR3F
13	37920001	37970000	1.146569834	RBBP9
13	37920001	37970000	1.146569834	SEC23B
13	37920001	37970000	1.146569834	DZANK1
11	71590001	71640000	1.146569673	RBKS
6	36860001	36910000	1.146483368	HERC6
1	148310001	148360000	1.146432821	CBR3
1	148310001	148360000	1.146432821	DOPEY2
17	29250001	29300000	1.146374702	GRIA2
11	40390001	40440000	1.146356389	VRK2
11	40390001	40440000	1.146356389	FANCL
5	86920001	86970000	1.146295118	ABCC9
1	68340001	68390000	1.146279102	LOC102173333
2	23130001	23180000	1.1462749	CUL3
1	120880001	120930000	1.146268489	LOC106502391
23	10490001	10540000	1.146258438	LOC102172503
23	10490001	10540000	1.146258438	FAM8A1
23	10490001	10540000	1.146258438	CAP2
26	47180001	47230000	1.146188074	LOC102181467
3	70180001	70230000	1.146133627	MTF2
2	6620001	6670000	1.14610587	E2F2
2	6620001	6670000	1.14610587	ID3
7	27480001	27530000	1.146064235	XRCC4
22	11560001	11610000	1.146037473	SLC22A13
22	11560001	11610000	1.146037473	OXSR1
16	43150001	43200000	1.146029689	RERE
17	22290001	22340000	1.14601567	TMEM132C
2	83880001	83930000	1.145948632	GTDC1
5	82270001	82320000	1.145897172	SSPN
18	58830001	58880000	1.145883613	ZNF613
18	58830001	58880000	1.145883613	ZNF432
18	58830001	58880000	1.145883613	ZNF614
1	118580001	118630000	1.145838915	CP
1	118580001	118630000	1.145838915	HPS3
11	28950001	29000000	1.145822139	MCFD2
11	28950001	29000000	1.145822139	TTC7A
14	12590001	12640000	1.145815473	TRNAG-CCC-57
14	12590001	12640000	1.145815473	DPY19L4
14	12590001	12640000	1.145815473	ESRP1
15	27400001	27450000	1.145804889	UVRAG
22	57290001	57340000	1.145596265	NR2C2
19	47640001	47690000	1.14559518	FTSJ3
19	47640001	47690000	1.14559518	CCDC47
19	47640001	47690000	1.14559518	DDX42
19	47640001	47690000	1.14559518	PSMC5
19	49890001	49940000	1.145539273	LOC108638312
19	49890001	49940000	1.145539273	LOC108638313
19	49890001	49940000	1.145539273	LOC108638314
19	49890001	49940000	1.145539273	CD7
5	20770001	20820000	1.145536939	LUM
6	1930001	1980000	1.145528407	1-Mar
25	870001	920000	1.145499312	LOC102177855
25	870001	920000	1.145499312	LOC102178129
25	870001	920000	1.145499312	LOC102177581
14	22730001	22780000	1.145495016	TRNAS-GGA-76

21	28370001	28420000	1.145475837	TARSL2
18	39700001	39750000	1.145394859	LOC108637979
14	49630001	49680000	1.14534338	C14H8orf34
16	36060001	36110000	1.145328372	SCYL3
16	36060001	36110000	1.145328372	KIFAP3
12	7250001	7300000	1.145301724	KDELC1
12	7250001	7300000	1.145301724	BIVM
12	7250001	7300000	1.145301724	TEX30
12	7250001	7300000	1.145301724	CCDC168
3	70160001	70210000	1.145298997	MTF2
6	46780001	46830000	1.145189214	STIM2
14	16100001	16150000	1.145108742	RIDA
14	16100001	16150000	1.145108742	POP1
1	40470001	40520000	1.145104815	EPHA6
23	24010001	24060000	1.145088182	LOC102174444
23	24010001	24060000	1.145088182	LOC102174170
3	85630001	85680000	1.145066169	LOC102187823
8	3410001	3460000	1.145018787	LOC108636588
1	148480001	148530000	1.145012236	MORC3
24	26850001	26900000	1.144964446	LOC108633784
6	114190001	114240000	1.144953413	SORCS2
18	64720001	64770000	1.144951592	LOC102175389
18	64720001	64770000	1.144951592	LOC102172431
18	64720001	64770000	1.144951592	LOC106501822
26	32080001	32130000	1.144926331	PYROXD2
26	32080001	32130000	1.144926331	HPS1
26	32080001	32130000	1.144926331	HPSE2
1	80090001	80140000	1.144905595	ADIPOQ
1	80090001	80140000	1.144905595	RFC4
3	68960001	69010000	1.144858003	TGFBR3
2	39180001	39230000	1.144766761	PTH2R
