# Supplementary material for: Whole-genome selection signatures identified candidate genes associated with cashmere traits in Inner Mongolia cashmere goats
Source: Anim Biosci. 2025 Jul 11;38(12):2597–611. doi: 10.5713/ab.25.0252 (PMC12580777; doi:10.5713/ab.25.0252)
Supplement: Supplementary file 8 [file ab-25-0252-Supplementary-8-19.pdf]

Supplement 8. Detected and gene annotation of candidate regions by XP-EHH (HYCG VS LYCG)

Chr	Start	End	XP-EHH	Gene name
1	45365	145365	0.351104	TRNAC-GCA
1	45365	145365	0.351104	ATP5O
1	45365	145365	0.351104	ITSN1
1	45540	145540	0.358739	TRNAC-GCA
1	45540	145540	0.358739	ATP5O
1	45540	145540	0.358739	ITSN1
1	63353	163353	0.345003	ATP5O
1	63353	163353	0.345003	ITSN1
1	63436	163436	0.342531	ATP5O
1	63436	163436	0.342531	ITSN1
1	63592	163592	0.343492	ATP5O
1	63592	163592	0.343492	ITSN1
1	63627	163627	0.348321	ATP5O
1	63627	163627	0.348321	ITSN1
1	63683	163683	0.341821	ATP5O
1	63683	163683	0.341821	ITSN1
1	63729	163729	0.359166	ATP5O
1	63729	163729	0.359166	ITSN1
1	63735	163735	0.352032	ATP5O
1	63735	163735	0.352032	ITSN1
1	63870	163870	0.361647	ATP5O
1	63870	163870	0.361647	ITSN1
1	63953	163953	0.364712	ATP5O
1	63953	163953	0.364712	ITSN1
1	63958	163958	0.349898	ATP5O
1	63958	163958	0.349898	ITSN1
1	64207	164207	0.343399	ATP5O
1	64207	164207	0.343399	ITSN1
1	64380	164380	0.343346	ATP5O
1	64380	164380	0.343346	ITSN1
1	64650	164650	0.343957	ATP5O
1	64650	164650	0.343957	ITSN1
1	64756	164756	0.367206	ATP5O
1	64756	164756	0.367206	ITSN1
1	65120	165120	0.343611	ATP5O
1	65120	165120	0.343611	ITSN1
1	65539	165539	0.353157	ATP5O
1	65539	165539	0.353157	ITSN1
1	65677	165677	0.354504	ATP5O
1	65677	165677	0.354504	ITSN1
1	65732	165732	0.347076	ATP5O
1	65732	165732	0.347076	ITSN1
1	65907	165907	0.35187	ATP5O
1	65907	165907	0.35187	ITSN1
1	65918	165918	0.355685	ATP5O
1	65918	165918	0.355685	ITSN1
1	66230	166230	0.350218	ATP5O
1	66230	166230	0.350218	ITSN1
1	66377	166377	0.383278	ATP5O
1	66377	166377	0.383278	ITSN1
1	66436	166436	0.390977	ATP5O
1	66436	166436	0.390977	ITSN1
1	66484	166484	0.40167	ATP5O
1	66484	166484	0.40167	ITSN1
1	66490	166490	0.393734	ATP5O
1	66490	166490	0.393734	ITSN1

1	66560	166560	0.390036	ATP5O
1	66560	166560	0.390036	ITSN1
1	66563	166563	0.390036	ATP5O
1	66563	166563	0.390036	ITSN1
1	66610	166610	0.390036	ATP5O
1	66610	166610	0.390036	ITSN1
1	66658	166658	0.400793	ATP5O
1	66658	166658	0.400793	ITSN1
1	66722	166722	0.386277	ATP5O
1	66722	166722	0.386277	ITSN1
1	66745	166745	0.386277	ATP5O
1	66745	166745	0.386277	ITSN1
1	66746	166746	0.386277	ATP5O
1	66746	166746	0.386277	ITSN1
1	66970	166970	0.386277	ATP5O
1	66970	166970	0.386277	ITSN1
1	763922	863922	0.342906	IFNAR2
1	764160	864160	0.344231	IFNAR2
1	764171	864171	0.344231	IFNAR2
1	764332	864332	0.343202	IFNAR2
1	764832	864832	0.342206	IFNAR2
1	765050	865050	0.342606	IFNAR2
1	765089	865089	0.342606	IFNAR2
1	765129	865129	0.345104	IFNAR2
1	765150	865150	0.343497	IFNAR2
1	766658	866658	0.349673	IFNAR2
1	767142	867142	0.342871	IFNAR2
1	770505	870505	0.342247	IFNAR2
1	770757	870757	0.346252	IFNAR2
1	776164	876164	0.34187	IFNAR2
1	776329	876329	0.343019	IFNAR2
1	950217	1050217	0.345374	OLIG2
1	951312	1051312	0.361821	OLIG2
1	951483	1051483	0.34576	OLIG2
1	980023	1080023	0.349209	OLIG2
1	980035	1080035	0.356432	OLIG2
1	980050	1080050	0.370012	OLIG2
1	1519410	1619410	0.349338	EVA1C
1	1519410	1619410	0.349338	URB1
1	1519619	1619619	0.349495	EVA1C
1	1519619	1619619	0.349495	URB1
1	1519647	1619647	0.359962	EVA1C
1	1519647	1619647	0.359962	URB1
1	1519775	1619775	0.353583	EVA1C
1	1519775	1619775	0.353583	URB1
1	1519864	1619864	0.365946	EVA1C
1	1519864	1619864	0.365946	URB1
1	1519996	1619996	0.377926	EVA1C
1	1519996	1619996	0.377926	URB1
1	1520142	1620142	0.364814	EVA1C
1	1520142	1620142	0.364814	URB1
1	1520176	1620176	0.365538	EVA1C
1	1520176	1620176	0.365538	URB1
1	1520190	1620190	0.366206	EVA1C
1	1520190	1620190	0.366206	URB1
1	1520237	1620237	0.355475	EVA1C
1	1520237	1620237	0.355475	URB1
1	1520366	1620366	0.350856	EVA1C

1	1520366	1620366	0.350856	URB1
1	1520476	1620476	0.374731	EVA1C
1	1520476	1620476	0.374731	URB1
1	1520611	1620611	0.373327	EVA1C
1	1520611	1620611	0.373327	URB1
1	1520779	1620779	0.369692	EVA1C
1	1520779	1620779	0.369692	URB1
1	1520841	1620841	0.383556	EVA1C
1	1520841	1620841	0.383556	URB1
1	1520851	1620851	0.375813	EVA1C
1	1520851	1620851	0.375813	URB1
1	1520856	1620856	0.379703	EVA1C
1	1520856	1620856	0.379703	URB1
1	1520913	1620913	0.36645	EVA1C
1	1520913	1620913	0.36645	URB1
1	1520936	1620936	0.356088	EVA1C
1	1520936	1620936	0.356088	URB1
1	1521047	1621047	0.356088	EVA1C
1	1521047	1621047	0.356088	URB1
1	1521079	1621079	0.370994	EVA1C
1	1521079	1621079	0.370994	URB1
1	1521105	1621105	0.365806	EVA1C
1	1521105	1621105	0.365806	URB1
1	1521178	1621178	0.370311	EVA1C
1	1521178	1621178	0.370311	URB1
1	1521195	1621195	0.363954	EVA1C
1	1521195	1621195	0.363954	URB1
1	1521252	1621252	0.365999	EVA1C
1	1521252	1621252	0.365999	URB1
1	1521291	1621291	0.372768	EVA1C
1	1521291	1621291	0.372768	URB1
1	1521300	1621300	0.369631	EVA1C
1	1521300	1621300	0.369631	URB1
1	1521369	1621369	0.367554	EVA1C
1	1521369	1621369	0.367554	URB1
1	1521436	1621436	0.367554	EVA1C
1	1521436	1621436	0.367554	URB1
1	1521478	1621478	0.376624	EVA1C
1	1521478	1621478	0.376624	URB1
1	1521486	1621486	0.371202	EVA1C
1	1521486	1621486	0.371202	URB1
1	1521513	1621513	0.353153	EVA1C
1	1521513	1621513	0.353153	URB1
1	1521577	1621577	0.352578	EVA1C
1	1521577	1621577	0.352578	URB1
1	1521762	1621762	0.357301	EVA1C
1	1521762	1621762	0.357301	URB1
1	1521806	1621806	0.350996	EVA1C
1	1521806	1621806	0.350996	URB1
1	1522086	1622086	0.344	EVA1C
1	1522086	1622086	0.344	URB1
1	1522130	1622130	0.349507	EVA1C
1	1522130	1622130	0.349507	URB1
1	1522427	1622427	0.350586	EVA1C
1	1522427	1622427	0.350586	URB1
1	1522725	1622725	0.361106	EVA1C
1	1522725	1622725	0.361106	URB1
1	1522733	1622733	0.347759	EVA1C

1	1522733	1622733	0.347759	URB1
1	1522845	1622845	0.342785	EVA1C
1	1522845	1622845	0.342785	URB1
1	1522904	1622904	0.344128	EVA1C
1	1522904	1622904	0.344128	URB1
1	1522940	1622940	0.344128	EVA1C
1	1522940	1622940	0.344128	URB1
1	1523212	1623212	0.362119	EVA1C
1	1523212	1623212	0.362119	URB1
1	1523239	1623239	0.370071	EVA1C
1	1523239	1623239	0.370071	URB1
1	1523256	1623256	0.366229	EVA1C
1	1523256	1623256	0.366229	URB1
1	1523409	1623409	0.365114	EVA1C
1	1523409	1623409	0.365114	URB1
1	1523566	1623566	0.364023	EVA1C
1	1523566	1623566	0.364023	URB1
1	1523567	1623567	0.364023	EVA1C
1	1523567	1623567	0.364023	URB1
1	1523600	1623600	0.367104	EVA1C
1	1523600	1623600	0.367104	URB1
1	1523606	1623606	0.36908	EVA1C
1	1523606	1623606	0.36908	URB1
1	1523620	1623620	0.364699	EVA1C
1	1523620	1623620	0.364699	URB1
1	1523635	1623635	0.364699	EVA1C
1	1523635	1623635	0.364699	URB1
1	1523659	1623659	0.361621	EVA1C
1	1523659	1623659	0.361621	URB1
1	1523734	1623734	0.368905	EVA1C
1	1523734	1623734	0.368905	URB1
1	1523755	1623755	0.372762	EVA1C
1	1523755	1623755	0.372762	URB1
1	1523794	1623794	0.373086	EVA1C
1	1523794	1623794	0.373086	URB1
1	1523810	1623810	0.373086	EVA1C
1	1523810	1623810	0.373086	URB1
1	1523933	1623933	0.370794	EVA1C
1	1523933	1623933	0.370794	URB1
1	1523960	1623960	0.377852	EVA1C
1	1523960	1623960	0.377852	URB1
1	1524028	1624028	0.363425	EVA1C
1	1524028	1624028	0.363425	URB1
1	1524082	1624082	0.352395	EVA1C
1	1524082	1624082	0.352395	URB1
1	1524168	1624168	0.356858	EVA1C
1	1524168	1624168	0.356858	URB1
1	1529886	1629886	0.343632	EVA1C
1	1529886	1629886	0.343632	URB1
1	1530182	1630182	0.351262	EVA1C
1	1530182	1630182	0.351262	URB1
1	1531094	1631094	0.360813	EVA1C
1	1531094	1631094	0.360813	URB1
1	1531112	1631112	0.381817	EVA1C
1	1531112	1631112	0.381817	URB1
1	1533133	1633133	0.359926	EVA1C
1	1533133	1633133	0.359926	URB1
1	1533881	1633881	0.387565	EVA1C

1	1533881	1633881	0.387565	URB1
1	1533984	1633984	0.392338	EVA1C
1	1533984	1633984	0.392338	URB1
1	1534054	1634054	0.405671	EVA1C
1	1534054	1634054	0.405671	URB1
1	1534061	1634061	0.402589	EVA1C
1	1534061	1634061	0.402589	URB1
1	1534082	1634082	0.380032	EVA1C
1	1534082	1634082	0.380032	URB1
1	1534346	1634346	0.415683	EVA1C
1	1534346	1634346	0.415683	URB1
1	1534383	1634383	0.43145	EVA1C
1	1534383	1634383	0.43145	URB1
1	1534407	1634407	0.422195	EVA1C
1	1534407	1634407	0.422195	URB1
1	1534446	1634446	0.426003	EVA1C
1	1534446	1634446	0.426003	URB1
1	1534494	1634494	0.436928	EVA1C
1	1534494	1634494	0.436928	URB1
1	1534575	1634575	0.438126	EVA1C
1	1534575	1634575	0.438126	URB1
1	1534633	1634633	0.450545	EVA1C
1	1534633	1634633	0.450545	URB1
1	1534642	1634642	0.449407	EVA1C
1	1534642	1634642	0.449407	URB1
1	1534644	1634644	0.450088	EVA1C
1	1534644	1634644	0.450088	URB1
1	1534681	1634681	0.446031	EVA1C
1	1534681	1634681	0.446031	URB1
1	1534712	1634712	0.442713	EVA1C
1	1534712	1634712	0.442713	URB1
1	1534752	1634752	0.413421	EVA1C
1	1534752	1634752	0.413421	URB1
1	1534777	1634777	0.415558	EVA1C
1	1534777	1634777	0.415558	URB1
1	1534782	1634782	0.381881	EVA1C
1	1534782	1634782	0.381881	URB1
1	1534822	1634822	0.385098	EVA1C
1	1534822	1634822	0.385098	URB1
1	1534841	1634841	0.37704	EVA1C
1	1534841	1634841	0.37704	URB1
1	1534921	1634921	0.37704	EVA1C
1	1534921	1634921	0.37704	URB1
1	1534947	1634947	0.367498	EVA1C
1	1534947	1634947	0.367498	URB1
1	1534953	1634953	0.367498	EVA1C
1	1534953	1634953	0.367498	URB1
1	1535030	1635030	0.365813	EVA1C
1	1535030	1635030	0.365813	URB1
1	1535307	1635307	0.357267	EVA1C
1	1535307	1635307	0.357267	URB1
1	1536341	1636341	0.382474	EVA1C
1	1536341	1636341	0.382474	URB1
1	1550453	1650453	0.347557	MRAP
1	1550453	1650453	0.347557	URB1
1	1550840	1650840	0.354436	MRAP
1	1550840	1650840	0.354436	URB1
1	1551152	1651152	0.344058	MRAP

1	1551152	1651152	0.344058	URB1
1	1551622	1651622	0.343264	MRAP
1	1551622	1651622	0.343264	URB1
1	1551757	1651757	0.341698	MRAP
1	1551757	1651757	0.341698	URB1
1	1551802	1651802	0.344549	MRAP
1	1551802	1651802	0.344549	URB1
1	1553132	1653132	0.342809	MRAP
1	1553132	1653132	0.342809	URB1
1	1553162	1653162	0.356524	MRAP
1	1553162	1653162	0.356524	URB1
1	1553185	1653185	0.349876	MRAP
1	1553185	1653185	0.349876	URB1
1	1553359	1653359	0.355029	MRAP
1	1553359	1653359	0.355029	URB1
1	1553380	1653380	0.355029	MRAP
1	1553380	1653380	0.355029	URB1
1	1553431	1653431	0.359522	MRAP
1	1553431	1653431	0.359522	URB1
1	1553432	1653432	0.359522	MRAP
1	1553432	1653432	0.359522	URB1
1	1553438	1653438	0.359522	MRAP
1	1553438	1653438	0.359522	URB1
1	1553443	1653443	0.359522	MRAP
1	1553443	1653443	0.359522	URB1
1	1553447	1653447	0.373934	MRAP
1	1553447	1653447	0.373934	URB1
1	1553448	1653448	0.376532	MRAP
1	1553448	1653448	0.376532	URB1
1	1553456	1653456	0.376532	MRAP
1	1553456	1653456	0.376532	URB1
1	1553464	1653464	0.378721	MRAP
1	1553464	1653464	0.378721	URB1
1	1553564	1653564	0.397766	MRAP
1	1553564	1653564	0.397766	URB1
1	1553593	1653593	0.388159	MRAP
1	1553593	1653593	0.388159	URB1
1	1553621	1653621	0.419604	MRAP
1	1553621	1653621	0.419604	URB1
1	1553622	1653622	0.419604	MRAP
1	1553622	1653622	0.419604	URB1
1	1553666	1653666	0.414641	MRAP
1	1553666	1653666	0.414641	URB1
1	1553675	1653675	0.431785	MRAP
1	1553675	1653675	0.431785	URB1
1	1553708	1653708	0.423776	MRAP
1	1553708	1653708	0.423776	URB1
1	1553721	1653721	0.423776	MRAP
1	1553721	1653721	0.423776	URB1
1	1553778	1653778	0.405267	MRAP
1	1553778	1653778	0.405267	URB1
1	1553813	1653813	0.404273	MRAP
1	1553813	1653813	0.404273	URB1
1	1553973	1653973	0.412964	MRAP
1	1553973	1653973	0.412964	URB1
1	1553976	1653976	0.412964	MRAP
1	1553976	1653976	0.412964	URB1
1	1553979	1653979	0.419577	MRAP

1	1553979	1653979	0.419577	URB1
1	1554007	1654007	0.421691	MRAP
1	1554007	1654007	0.421691	URB1
1	1554010	1654010	0.403382	MRAP
1	1554010	1654010	0.403382	URB1
1	1554060	1654060	0.385974	MRAP
1	1554060	1654060	0.385974	URB1
1	1554141	1654141	0.37919	MRAP
1	1554141	1654141	0.37919	URB1
1	1554324	1654324	0.387176	MRAP
1	1554324	1654324	0.387176	URB1
1	1554551	1654551	0.342706	MRAP
1	1554551	1654551	0.342706	URB1
1	1554722	1654722	0.369832	MRAP
1	1554722	1654722	0.369832	URB1
1	1554739	1654739	0.410835	MRAP
1	1554739	1654739	0.410835	URB1
1	1554776	1654776	0.457007	MRAP
1	1554776	1654776	0.457007	URB1
1	1554781	1654781	0.44566	MRAP
1	1554781	1654781	0.44566	URB1
1	1554782	1654782	0.449814	MRAP
1	1554782	1654782	0.449814	URB1
1	1554798	1654798	0.412216	MRAP
1	1554798	1654798	0.412216	URB1
1	1554800	1654800	0.413283	MRAP
1	1554800	1654800	0.413283	URB1
1	1554807	1654807	0.413283	MRAP
1	1554807	1654807	0.413283	URB1
1	1554808	1654808	0.42242	MRAP
1	1554808	1654808	0.42242	URB1
1	1554813	1654813	0.378612	MRAP
1	1554813	1654813	0.378612	URB1
1	1554821	1654821	0.378831	MRAP
1	1554821	1654821	0.378831	URB1
1	1554840	1654840	0.378891	MRAP
1	1554840	1654840	0.378891	URB1
1	1554847	1654847	0.381316	MRAP
1	1554847	1654847	0.381316	URB1
1	1554854	1654854	0.382877	MRAP
1	1554854	1654854	0.382877	URB1
1	1554874	1654874	0.344342	MRAP
1	1554874	1654874	0.344342	URB1
1	1554931	1654931	0.351194	MRAP
1	1554931	1654931	0.351194	URB1
1	1554966	1654966	0.365273	MRAP
1	1554966	1654966	0.365273	URB1
1	1642256	1742256	0.358026	MRAP
1	1642256	1742256	0.358026	MIS18A
1	1642256	1742256	0.358026	URB1
1	1642795	1742795	0.344207	MRAP
1	1642795	1742795	0.344207	MIS18A
1	1642795	1742795	0.344207	URB1
1	1642948	1742948	0.344722	MRAP
1	1642948	1742948	0.344722	MIS18A
1	1642948	1742948	0.344722	URB1
1	1643136	1743136	0.347959	MRAP
1	1643136	1743136	0.347959	MIS18A

1	1643136	1743136	0.347959	URB1
1	1643138	1743138	0.347959	MRAP
1	1643138	1743138	0.347959	MIS18A
1	1643138	1743138	0.347959	URB1
1	1643146	1743146	0.36975	MRAP
1	1643146	1743146	0.36975	MIS18A
1	1643146	1743146	0.36975	URB1
1	1643170	1743170	0.341897	MRAP
1	1643170	1743170	0.341897	MIS18A
1	1643170	1743170	0.341897	URB1
1	1953289	2053289	0.350876	LOC102177836
1	1953289	2053289	0.350876	HUNK
1	2869779	2969779	0.398465	TIAM1
1	2869791	2969791	0.403523	TIAM1
1	2869809	2969809	0.413141	TIAM1
1	2869861	2969861	0.390484	TIAM1
1	2869874	2969874	0.37679	TIAM1
1	2869918	2969918	0.392673	TIAM1
1	2869928	2969928	0.392748	TIAM1
1	2869935	2969935	0.409866	TIAM1
1	2869939	2969939	0.375324	TIAM1
1	2870027	2970027	0.361081	TIAM1
1	2870045	2970045	0.362	TIAM1
1	2870058	2970058	0.363524	TIAM1
1	2870100	2970100	0.357771	TIAM1
1	3140107	3240107	0.356731	LOC100861181
1	3140107	3240107	0.356731	KAP8
1	3149435	3249435	0.348062	LOC100861181
1	3149435	3249435	0.348062	KAP8
1	3149508	3249508	0.363174	LOC100861181
1	3149508	3249508	0.363174	KAP8
1	3149522	3249522	0.362859	LOC100861181
1	3149522	3249522	0.362859	KAP8
1	3149554	3249554	0.362859	LOC100861181
1	3149554	3249554	0.362859	KAP8
1	3149555	3249555	0.362859	LOC100861181
1	3149555	3249555	0.362859	KAP8
1	3149598	3249598	0.364121	LOC100861181
1	3149598	3249598	0.364121	KAP8
1	3149622	3249622	0.357774	LOC100861181
1	3149622	3249622	0.357774	KAP8
1	3149669	3249669	0.354202	LOC100861181
1	3149669	3249669	0.354202	KAP8
1	3149672	3249672	0.363453	LOC100861181
1	3149672	3249672	0.363453	KAP8
1	3149680	3249680	0.363453	LOC100861181
1	3149680	3249680	0.363453	KAP8
1	3149866	3249866	0.363709	LOC100861181
1	3149866	3249866	0.363709	KAP8
1	3150031	3250031	0.357493	LOC100861181
1	3150031	3250031	0.357493	KAP8
1	3150058	3250058	0.346185	LOC100861181
1	3150058	3250058	0.346185	KAP8
1	3150094	3250094	0.360994	LOC100861181
1	3150094	3250094	0.360994	KAP8
1	3150187	3250187	0.364943	LOC100861181
1	3150187	3250187	0.364943	KAP8
1	3227301	3327301	0.365466	LOC100861168

1	3227321	3327321	0.354466	LOC100861168
1	3227382	3327382	0.359236	LOC100861168
1	3227395	3327395	0.359236	LOC100861168
1	3227426	3327426	0.365265	LOC100861168
1	3227507	3327507	0.362609	LOC100861168
1	3227625	3327625	0.358893	LOC100861168
1	3227717	3327717	0.37695	LOC100861168
1	3227924	3327924	0.351501	LOC100861168
1	3229044	3329044	0.346931	LOC100861168
1	3229887	3329887	0.353892	LOC100861168
1	3229905	3329905	0.357596	LOC100861168
1	3229998	3329998	0.366697	LOC100861168
1	3230320	3330320	0.343851	LOC100861168
1	3230472	3330472	0.341797	LOC100861168
1	3233294	3333294	0.494871	LOC100861168
1	3233505	3333505	0.500242	LOC100861168
1	3233731	3333731	0.399036	LOC100861168
1	3233732	3333732	0.413449	LOC100861168
1	3233755	3333755	0.395916	LOC100861168
1	3233758	3333758	0.395169	LOC100861168
1	3233820	3333820	0.400858	LOC100861168
1	3233999	3333999	0.461129	LOC100861168
1	3234001	3334001	0.461758	LOC100861168
1	3234013	3334013	0.34477	LOC100861168
1	3818673	3918673	0.344492	KRTAP15-1
1	3818673	3918673	0.344492	LOC100861175
1	3818673	3918673	0.344492	LOC102180888
1	3818673	3918673	0.344492	LOC102181160
1	3818673	3918673	0.344492	LOC102181431
1	3818721	3918721	0.343858	KRTAP15-1
1	3818721	3918721	0.343858	LOC100861175
1	3818721	3918721	0.343858	LOC102180888
1	3818721	3918721	0.343858	LOC102181160
1	3818721	3918721	0.343858	LOC102181431
1	3826265	3926265	0.343997	KRTAP15-1
1	3826265	3926265	0.343997	LOC100861175
1	3826265	3926265	0.343997	LOC102180888
1	3826265	3926265	0.343997	LOC102181160
1	3826265	3926265	0.343997	LOC102181431
1	27724032	27824032	0.40308	GBE1
1	27724055	27824055	0.401454	GBE1
1	27724394	27824394	0.400848	GBE1
1	27724446	27824446	0.406748	GBE1
1	27724474	27824474	0.393786	GBE1
1	27724594	27824594	0.393786	GBE1
1	27724863	27824863	0.38835	GBE1
1	27725257	27825257	0.385059	GBE1
1	27725296	27825296	0.399314	GBE1
1	27725301	27825301	0.399314	GBE1
1	27725392	27825392	0.390764	GBE1
1	27725597	27825597	0.436702	GBE1
1	27725624	27825624	0.408171	GBE1
1	27725850	27825850	0.357022	GBE1
1	27726184	27826184	0.370111	GBE1
1	27726193	27826193	0.392747	GBE1
1	27729748	27829748	0.353231	GBE1
1	27730866	27830866	0.343642	GBE1
1	27731112	27831112	0.358291	GBE1

1	27731262	27831262	0.35878	GBE1
1	27731307	27831307	0.35856	GBE1
1	27731356	27831356	0.345757	GBE1
1	27731377	27831377	0.343941	GBE1
1	32591777	32691777	0.353912	CADM2
1	32591894	32691894	0.353912	CADM2
1	32692217	32792217	0.348131	CADM2
1	32692270	32792270	0.351348	CADM2
1	32692498	32792498	0.348691	CADM2
1	32692630	32792630	0.350951	CADM2
1	32692671	32792671	0.347918	CADM2
1	32692704	32792704	0.345278	CADM2
1	32692730	32792730	0.345278	CADM2
1	32692807	32792807	0.34453	CADM2
1	32693187	32793187	0.345072	CADM2
1	32693272	32793272	0.352314	CADM2
1	32693322	32793322	0.353177	CADM2
1	35830147	35930147	0.391895	EPHA3
1	35896927	35996927	0.383449	EPHA3
1	35924556	36024556	0.343459	EPHA3
1	40472173	40572173	0.344958	EPHA6
1	41001299	41101299	0.3431	CLIC6
1	41001734	41101734	0.34936	CLIC6
1	41395626	41495626	0.351488	C1H21orf140
1	41395626	41495626	0.351488	LOC108637287
1	41395626	41495626	0.351488	KCNE2
1	41395626	41495626	0.351488	SMIM11A
1	41396297	41496297	0.350265	C1H21orf140
1	41396297	41496297	0.350265	LOC108637287
1	41396297	41496297	0.350265	KCNE2
1	41396297	41496297	0.350265	SMIM11A
1	41396319	41496319	0.35231	C1H21orf140
1	41396319	41496319	0.35231	LOC108637287
1	41396319	41496319	0.35231	KCNE2
1	41396319	41496319	0.35231	SMIM11A
1	42704404	42804404	0.347538	LOC102168768
1	42704404	42804404	0.347538	ST3GAL6
1	42704561	42804561	0.34415	LOC102168768
1	42704561	42804561	0.34415	ST3GAL6
1	42704646	42804646	0.347853	LOC102168768
1	42704646	42804646	0.347853	ST3GAL6
1	42704673	42804673	0.343131	LOC102168768
1	42704673	42804673	0.343131	ST3GAL6
1	42704674	42804674	0.343131	LOC102168768
1	42704674	42804674	0.343131	ST3GAL6
1	42704682	42804682	0.343131	LOC102168768
1	42704682	42804682	0.343131	ST3GAL6
1	42704733	42804733	0.343131	LOC102168768
1	42704733	42804733	0.343131	ST3GAL6
1	42704743	42804743	0.342473	LOC102168768
1	42704743	42804743	0.342473	ST3GAL6
1	44066822	44166822	0.384243	CMSS1
1	44066822	44166822	0.384243	FILIP1L
1	44066846	44166846	0.383999	CMSS1
1	44066846	44166846	0.383999	FILIP1L
1	44066926	44166926	0.365005	CMSS1
1	44066926	44166926	0.365005	FILIP1L
1	44067373	44167373	0.360443	CMSS1

1	44067373	44167373	0.360443	FILIP1L
1	44215698	44315698	0.401592	LOC102177192
1	44215698	44315698	0.401592	CMSS1
1	44215856	44315856	0.391058	LOC102177192
1	44215856	44315856	0.391058	CMSS1
1	44245826	44345826	0.355034	LOC102177192
1	44245826	44345826	0.355034	CMSS1
1	44245841	44345841	0.366274	LOC102177192
1	44245841	44345841	0.366274	CMSS1
1	44246066	44346066	0.341797	LOC102177192
1	44246066	44346066	0.341797	CMSS1
1	44246377	44346377	0.350935	LOC102177192
1	44246377	44346377	0.350935	CMSS1
1	44246449	44346449	0.347168	LOC102177192
1	44246449	44346449	0.347168	CMSS1
1	44247701	44347701	0.34901	LOC102177192
1	44247701	44347701	0.34901	CMSS1
1	44248295	44348295	0.359168	LOC102177192
1	44248295	44348295	0.359168	CMSS1
1	44248464	44348464	0.389448	LOC102177192
1	44248464	44348464	0.389448	CMSS1
1	44248753	44348753	0.387191	LOC102177192
1	44248753	44348753	0.387191	CMSS1
1	44248763	44348763	0.392813	LOC102177192
1	44248763	44348763	0.392813	CMSS1
1	44248794	44348794	0.364105	LOC102177192
1	44248794	44348794	0.364105	CMSS1
1	44248925	44348925	0.371517	LOC102177192
1	44248925	44348925	0.371517	CMSS1
1	44248948	44348948	0.35116	LOC102177192
1	44248948	44348948	0.35116	CMSS1
1	44249188	44349188	0.347802	LOC102177192
1	44249188	44349188	0.347802	CMSS1
1	44249235	44349235	0.347324	LOC102177192
1	44249235	44349235	0.347324	CMSS1
1	44249723	44349723	0.352962	LOC102177192
1	44249723	44349723	0.352962	CMSS1
1	44250220	44350220	0.353692	LOC102177192
1	44250220	44350220	0.353692	CMSS1
1	44250311	44350311	0.353692	LOC102177192
1	44250311	44350311	0.353692	CMSS1
1	44250643	44350643	0.344096	LOC102177192
1	44250643	44350643	0.344096	CMSS1
1	44251139	44351139	0.351988	LOC102177192
1	44251139	44351139	0.351988	CMSS1
1	44251646	44351646	0.356426	LOC102177192
1	44251646	44351646	0.356426	CMSS1
1	44251705	44351705	0.363001	LOC102177192
1	44251705	44351705	0.363001	CMSS1
1	44251714	44351714	0.352103	LOC102177192
1	44251714	44351714	0.352103	CMSS1
1	44257069	44357069	0.350347	TBC1D23
1	44257069	44357069	0.350347	LOC102177192
1	44257069	44357069	0.350347	CMSS1
1	44257070	44357070	0.350347	TBC1D23
1	44257070	44357070	0.350347	LOC102177192
1	44257070	44357070	0.350347	CMSS1
1	44257169	44357169	0.351231	TBC1D23

1	44257169	44357169	0.351231	LOC102177192
1	44257169	44357169	0.351231	CMSS1
1	44257435	44357435	0.343652	TBC1D23
1	44257435	44357435	0.343652	LOC102177192
1	44257435	44357435	0.343652	CMSS1
1	44257904	44357904	0.353672	TBC1D23
1	44257904	44357904	0.353672	LOC102177192
1	44257904	44357904	0.353672	CMSS1
1	44257926	44357926	0.348851	TBC1D23
1	44257926	44357926	0.348851	LOC102177192
1	44257926	44357926	0.348851	CMSS1
1	44257944	44357944	0.344412	TBC1D23
1	44257944	44357944	0.344412	LOC102177192
1	44257944	44357944	0.344412	CMSS1
1	44263791	44363791	0.342595	TBC1D23
1	44263791	44363791	0.342595	LOC102177192
1	44263791	44363791	0.342595	CMSS1
1	44263868	44363868	0.353005	TBC1D23
1	44263868	44363868	0.353005	LOC102177192
1	44263868	44363868	0.353005	CMSS1
1	44263897	44363897	0.363691	TBC1D23
1	44263897	44363897	0.363691	LOC102177192
1	44263897	44363897	0.363691	CMSS1
1	44264233	44364233	0.353123	TBC1D23
1	44264233	44364233	0.353123	LOC102177192
1	44264233	44364233	0.353123	CMSS1
1	44264245	44364245	0.348155	TBC1D23
1	44264245	44364245	0.348155	LOC102177192
1	44264245	44364245	0.348155	CMSS1
1	44264291	44364291	0.356794	TBC1D23
1	44264291	44364291	0.356794	LOC102177192
1	44264291	44364291	0.356794	CMSS1
1	44264739	44364739	0.352825	TBC1D23
1	44264739	44364739	0.352825	LOC102177192
1	44264739	44364739	0.352825	CMSS1
1	44265152	44365152	0.368548	TBC1D23
1	44265152	44365152	0.368548	LOC102177192
1	44265152	44365152	0.368548	CMSS1
1	44265526	44365526	0.35312	TBC1D23
1	44265526	44365526	0.35312	LOC102177192
1	44265526	44365526	0.35312	CMSS1
1	44265868	44365868	0.374004	TBC1D23
1	44265868	44365868	0.374004	LOC102177192
1	44265868	44365868	0.374004	CMSS1
1	44266078	44366078	0.385691	TBC1D23
1	44266078	44366078	0.385691	LOC102177192
1	44266078	44366078	0.385691	CMSS1
1	44266323	44366323	0.37896	TBC1D23
1	44266323	44366323	0.37896	LOC102177192
1	44266323	44366323	0.37896	CMSS1
1	44266496	44366496	0.38222	TBC1D23
1	44266496	44366496	0.38222	LOC102177192
1	44266496	44366496	0.38222	CMSS1
1	44266590	44366590	0.373735	TBC1D23
1	44266590	44366590	0.373735	LOC102177192
1	44266590	44366590	0.373735	CMSS1
1	44266631	44366631	0.346529	TBC1D23
1	44266631	44366631	0.346529	LOC102177192

1	44266631	44366631	0.346529	CMSS1
1	44266821	44366821	0.352342	TBC1D23
1	44266821	44366821	0.352342	LOC102177192
1	44266821	44366821	0.352342	CMSS1
1	44267169	44367169	0.363748	TBC1D23
1	44267169	44367169	0.363748	LOC102177192
1	44267169	44367169	0.363748	CMSS1
1	44267241	44367241	0.363748	TBC1D23
1	44267241	44367241	0.363748	LOC102177192
1	44267241	44367241	0.363748	CMSS1
1	44267430	44367430	0.364001	TBC1D23
1	44267430	44367430	0.364001	LOC102177192
1	44267430	44367430	0.364001	CMSS1
1	44267881	44367881	0.378978	TBC1D23
1	44267881	44367881	0.378978	LOC102177192
1	44267881	44367881	0.378978	CMSS1
1	44267933	44367933	0.36656	TBC1D23
1	44267933	44367933	0.36656	LOC102177192
1	44267933	44367933	0.36656	CMSS1
1	44268581	44368581	0.356844	TBC1D23
1	44268581	44368581	0.356844	LOC102177192
1	44268581	44368581	0.356844	CMSS1
1	44268757	44368757	0.365263	TBC1D23
1	44268757	44368757	0.365263	LOC102177192
1	44268757	44368757	0.365263	CMSS1
1	44269174	44369174	0.366778	TBC1D23
1	44269174	44369174	0.366778	LOC102177192
1	44269174	44369174	0.366778	CMSS1
1	44269454	44369454	0.391767	TBC1D23
1	44269454	44369454	0.391767	LOC102177192
1	44269454	44369454	0.391767	CMSS1
1	44269545	44369545	0.404314	TBC1D23
1	44269545	44369545	0.404314	LOC102177192
1	44269545	44369545	0.404314	CMSS1
1	44269758	44369758	0.404875	TBC1D23
1	44269758	44369758	0.404875	LOC102177192
1	44269758	44369758	0.404875	CMSS1
1	44269833	44369833	0.404152	TBC1D23
1	44269833	44369833	0.404152	LOC102177192
1	44269833	44369833	0.404152	CMSS1
1	44269927	44369927	0.398744	TBC1D23
1	44269927	44369927	0.398744	LOC102177192
1	44269927	44369927	0.398744	CMSS1
1	44269928	44369928	0.398744	TBC1D23
1	44269928	44369928	0.398744	LOC102177192
1	44269928	44369928	0.398744	CMSS1
1	44269977	44369977	0.416696	TBC1D23
1	44269977	44369977	0.416696	LOC102177192
1	44269977	44369977	0.416696	CMSS1
1	44270017	44370017	0.417999	TBC1D23
1	44270017	44370017	0.417999	LOC102177192
1	44270017	44370017	0.417999	CMSS1
1	44270028	44370028	0.404789	TBC1D23
1	44270028	44370028	0.404789	LOC102177192
1	44270028	44370028	0.404789	CMSS1
1	44270039	44370039	0.412613	TBC1D23
1	44270039	44370039	0.412613	LOC102177192
1	44270039	44370039	0.412613	CMSS1

1	44270040	44370040	0.412613	TBC1D23
1	44270040	44370040	0.412613	LOC102177192
1	44270040	44370040	0.412613	CMSS1
1	44270172	44370172	0.394545	TBC1D23
1	44270172	44370172	0.394545	LOC102177192
1	44270172	44370172	0.394545	CMSS1
1	44270198	44370198	0.381279	TBC1D23
1	44270198	44370198	0.381279	LOC102177192
1	44270198	44370198	0.381279	CMSS1
1	44271542	44371542	0.364364	TBC1D23
1	44271542	44371542	0.364364	LOC102177192
1	44271542	44371542	0.364364	CMSS1
1	44271875	44371875	0.37148	TBC1D23
1	44271875	44371875	0.37148	LOC102177192
1	44271875	44371875	0.37148	CMSS1
1	44272129	44372129	0.388686	TBC1D23
1	44272129	44372129	0.388686	LOC102177192
1	44272129	44372129	0.388686	CMSS1
1	44272332	44372332	0.374242	TBC1D23
1	44272332	44372332	0.374242	LOC102177192
1	44272332	44372332	0.374242	CMSS1
1	44272361	44372361	0.368645	TBC1D23
1	44272361	44372361	0.368645	LOC102177192
1	44272361	44372361	0.368645	CMSS1
1	44272455	44372455	0.379481	TBC1D23
1	44272455	44372455	0.379481	LOC102177192
1	44272455	44372455	0.379481	CMSS1
1	44273292	44373292	0.380417	TBC1D23
1	44273292	44373292	0.380417	LOC102177192
1	44273292	44373292	0.380417	CMSS1
1	44273296	44373296	0.390826	TBC1D23
1	44273296	44373296	0.390826	LOC102177192
1	44273296	44373296	0.390826	CMSS1
1	44274022	44374022	0.390544	TBC1D23
1	44274022	44374022	0.390544	LOC102177192
1	44274022	44374022	0.390544	CMSS1
1	44274314	44374314	0.394094	TBC1D23
1	44274314	44374314	0.394094	LOC102177192
1	44274314	44374314	0.394094	CMSS1
1	44275124	44375124	0.376448	TBC1D23
1	44275124	44375124	0.376448	LOC102177192
1	44275124	44375124	0.376448	CMSS1
1	44275385	44375385	0.383156	TBC1D23
1	44275385	44375385	0.383156	LOC102177192
1	44275385	44375385	0.383156	CMSS1
1	44275882	44375882	0.392904	TBC1D23
1	44275882	44375882	0.392904	LOC102177192
1	44275882	44375882	0.392904	CMSS1
1	44276025	44376025	0.39339	TBC1D23
1	44276025	44376025	0.39339	LOC102177192
1	44276025	44376025	0.39339	CMSS1
1	44276088	44376088	0.389271	TBC1D23
1	44276088	44376088	0.389271	LOC102177192
1	44276088	44376088	0.389271	CMSS1
1	44276350	44376350	0.388848	TBC1D23
1	44276350	44376350	0.388848	LOC102177192
1	44276350	44376350	0.388848	CMSS1
1	44276807	44376807	0.405466	TBC1D23

1	44276807	44376807	0.405466	LOC102177192
1	44276807	44376807	0.405466	CMSS1
1	44277043	44377043	0.400654	TBC1D23
1	44277043	44377043	0.400654	LOC102177192
1	44277043	44377043	0.400654	CMSS1
1	44277114	44377114	0.40878	TBC1D23
1	44277114	44377114	0.40878	LOC102177192
1	44277114	44377114	0.40878	CMSS1
1	44277117	44377117	0.408933	TBC1D23
1	44277117	44377117	0.408933	LOC102177192
1	44277117	44377117	0.408933	CMSS1
1	44277318	44377318	0.363475	TBC1D23
1	44277318	44377318	0.363475	LOC102177192
1	44277318	44377318	0.363475	CMSS1
1	44277518	44377518	0.372556	TBC1D23
1	44277518	44377518	0.372556	LOC102177192
1	44277518	44377518	0.372556	CMSS1
1	44277995	44377995	0.345529	TBC1D23
1	44277995	44377995	0.345529	LOC102177192
1	44277995	44377995	0.345529	CMSS1
1	44278506	44378506	0.361522	TBC1D23
1	44278506	44378506	0.361522	LOC102177192
1	44278808	44378808	0.350786	TBC1D23
1	44278808	44378808	0.350786	LOC102177192
1	44278908	44378908	0.362086	TBC1D23
1	44278908	44378908	0.362086	LOC102177192
1	44278938	44378938	0.366382	TBC1D23
1	44278938	44378938	0.366382	LOC102177192
1	44279202	44379202	0.364688	TBC1D23
1	44279202	44379202	0.364688	LOC102177192
1	44279319	44379319	0.364688	TBC1D23
1	44279319	44379319	0.364688	LOC102177192
1	44279402	44379402	0.35753	TBC1D23
1	44279402	44379402	0.35753	LOC102177192
1	44280033	44380033	0.3547	TBC1D23
1	44280033	44380033	0.3547	LOC102177192
1	44280360	44380360	0.359345	TBC1D23
1	44280360	44380360	0.359345	LOC102177192
1	44280481	44380481	0.359345	TBC1D23
1	44280481	44380481	0.359345	LOC102177192
1	44281066	44381066	0.416675	TBC1D23
1	44281066	44381066	0.416675	LOC102177192
1	44281231	44381231	0.414083	TBC1D23
1	44281231	44381231	0.414083	LOC102177192
1	44281291	44381291	0.430745	TBC1D23
1	44281291	44381291	0.430745	LOC102177192
1	44281296	44381296	0.431084	TBC1D23
1	44281296	44381296	0.431084	LOC102177192
1	44281308	44381308	0.460855	TBC1D23
1	44281308	44381308	0.460855	LOC102177192
1	44281315	44381315	0.456078	TBC1D23
1	44281315	44381315	0.456078	LOC102177192
1	44281452	44381452	0.45476	TBC1D23
1	44281452	44381452	0.45476	LOC102177192
1	44281553	44381553	0.459404	TBC1D23
1	44281553	44381553	0.459404	LOC102177192
1	44281605	44381605	0.452141	TBC1D23
1	44281605	44381605	0.452141	LOC102177192

1	44281697	44381697	0.43844	TBC1D23
1	44281697	44381697	0.43844	LOC102177192
1	44281809	44381809	0.43465	TBC1D23
1	44281809	44381809	0.43465	LOC102177192
1	44282740	44382740	0.433995	TBC1D23
1	44282740	44382740	0.433995	LOC102177192
1	44282843	44382843	0.438354	TBC1D23
1	44282843	44382843	0.438354	LOC102177192
1	44283014	44383014	0.440115	TBC1D23
1	44283014	44383014	0.440115	LOC102177192
1	44283015	44383015	0.437163	TBC1D23
1	44283015	44383015	0.437163	LOC102177192
1	44283168	44383168	0.423863	TBC1D23
1	44283168	44383168	0.423863	LOC102177192
1	44283323	44383323	0.428378	TBC1D23
1	44283323	44383323	0.428378	LOC102177192
1	44284173	44384173	0.401354	TBC1D23
1	44284173	44384173	0.401354	LOC102177192
1	44284199	44384199	0.406871	TBC1D23
1	44284199	44384199	0.406871	LOC102177192
1	44284543	44384543	0.365183	TBC1D23
1	44284543	44384543	0.365183	LOC102177192
1	44284644	44384644	0.360913	TBC1D23
1	44284644	44384644	0.360913	LOC102177192
1	44287159	44387159	0.39086	TBC1D23
1	44287159	44387159	0.39086	LOC102177192
1	44287432	44387432	0.367612	TBC1D23
1	44287432	44387432	0.367612	LOC102177192
1	44288250	44388250	0.367612	TBC1D23
1	44288250	44388250	0.367612	LOC102177192
1	44288303	44388303	0.402304	TBC1D23
1	44288303	44388303	0.402304	LOC102177192
1	44288659	44388659	0.385367	TBC1D23
1	44288659	44388659	0.385367	LOC102177192
1	44288660	44388660	0.385367	TBC1D23
1	44288660	44388660	0.385367	LOC102177192
1	44288776	44388776	0.385367	TBC1D23
1	44288776	44388776	0.385367	LOC102177192
1	44289429	44389429	0.404514	TBC1D23
1	44289429	44389429	0.404514	LOC102177192
1	44289484	44389484	0.399203	TBC1D23
1	44289484	44389484	0.399203	LOC102177192
1	44290047	44390047	0.396652	TBC1D23
1	44290047	44390047	0.396652	LOC102177192
1	44290200	44390200	0.42	TBC1D23
1	44290200	44390200	0.42	LOC102177192
1	44290695	44390695	0.411652	TBC1D23
1	44290695	44390695	0.411652	LOC102177192
1	44290701	44390701	0.420925	TBC1D23
1	44290701	44390701	0.420925	LOC102177192
1	44290965	44390965	0.436078	TBC1D23
1	44290965	44390965	0.436078	LOC102177192
1	44291312	44391312	0.455346	TBC1D23
1	44291312	44391312	0.455346	LOC102177192
1	44291322	44391322	0.455543	TBC1D23
1	44291322	44391322	0.455543	LOC102177192
1	44291339	44391339	0.440164	TBC1D23
1	44291339	44391339	0.440164	LOC102177192

1	44291412	44391412	0.43668	TBC1D23
1	44291412	44391412	0.43668	LOC102177192
1	44291456	44391456	0.434865	TBC1D23
1	44291456	44391456	0.434865	LOC102177192
1	44294965	44394965	0.343359	TBC1D23
1	44294965	44394965	0.343359	LOC102177192
1	44294990	44394990	0.350468	TBC1D23
1	44294990	44394990	0.350468	LOC102177192
1	44295029	44395029	0.345684	TBC1D23
1	44295029	44395029	0.345684	LOC102177192
1	44295041	44395041	0.351942	TBC1D23
1	44295041	44395041	0.351942	LOC102177192
1	44305500	44405500	0.343348	TBC1D23
1	44305500	44405500	0.343348	LOC102177192
1	44305536	44405536	0.343348	TBC1D23
1	44305536	44405536	0.343348	LOC102177192
1	44305965	44405965	0.343654	TBC1D23
1	44305965	44405965	0.343654	LOC102177192
1	44306064	44406064	0.34542	TBC1D23
1	44306064	44406064	0.34542	LOC102177192
1	44306090	44406090	0.348242	TBC1D23
1	44306090	44406090	0.348242	LOC102177192
1	44306158	44406158	0.359869	TBC1D23
1	44306158	44406158	0.359869	LOC102177192
1	44306161	44406161	0.359419	TBC1D23
1	44306161	44406161	0.359419	LOC102177192
1	44306227	44406227	0.356836	TBC1D23
1	44306227	44406227	0.356836	LOC102177192
1	44307956	44407956	0.342009	TBC1D23
1	44308059	44408059	0.364872	TBC1D23
1	45087135	45187135	0.358104	ABI3BP
1	45087478	45187478	0.368133	ABI3BP
1	45087565	45187565	0.410533	ABI3BP
1	45087600	45187600	0.400277	ABI3BP
1	45087804	45187804	0.383115	ABI3BP
1	45087809	45187809	0.383359	ABI3BP
1	45087811	45187811	0.354475	ABI3BP
1	45088215	45188215	0.346409	ABI3BP
1	45088453	45188453	0.353586	ABI3BP
1	45088558	45188558	0.353622	ABI3BP
1	45088569	45188569	0.357501	ABI3BP
1	45088746	45188746	0.361199	ABI3BP
1	45089203	45189203	0.362266	ABI3BP
1	45089219	45189219	0.368335	ABI3BP
1	45089566	45189566	0.368366	ABI3BP
1	45089569	45189569	0.354002	ABI3BP
1	45090227	45190227	0.361415	ABI3BP
1	45090923	45190923	0.343458	ABI3BP
1	45091049	45191049	0.348389	ABI3BP
1	45091393	45191393	0.36454	ABI3BP
1	45096225	45196225	0.345005	ABI3BP
1	45096229	45196229	0.345005	ABI3BP
1	45096565	45196565	0.343417	ABI3BP
1	45123932	45223932	0.351845	ABI3BP
1	45127125	45227125	0.341811	ABI3BP
1	45432115	45532115	0.343743	IMPG2
1	45432145	45532145	0.3604	IMPG2
1	45432182	45532182	0.356029	IMPG2

1	45432189	45532189	0.364309	IMPG2
1	45432203	45532203	0.374286	IMPG2
1	45432205	45532205	0.375889	IMPG2
1	45432338	45532338	0.369874	IMPG2
1	45432398	45532398	0.359335	IMPG2
1	45432441	45532441	0.359686	IMPG2
1	45432455	45532455	0.359686	IMPG2
1	45432567	45532567	0.359898	IMPG2
1	45432658	45532658	0.365359	IMPG2
1	45432766	45532766	0.344941	IMPG2
1	45432863	45532863	0.344941	IMPG2
1	45432914	45532914	0.352876	IMPG2
1	45433048	45533048	0.35606	IMPG2
1	45433183	45533183	0.345379	IMPG2
1	45433241	45533241	0.348443	IMPG2
1	45433545	45533545	0.34476	IMPG2
1	45433709	45533709	0.385491	IMPG2
1	45433867	45533867	0.363862	IMPG2
1	45434086	45534086	0.359541	IMPG2
1	45434224	45534224	0.36722	IMPG2
1	45434225	45534225	0.36722	IMPG2
1	45434312	45534312	0.376017	IMPG2
1	45434375	45534375	0.375408	IMPG2
1	45434427	45534427	0.372059	IMPG2
1	45434474	45534474	0.381622	IMPG2
1	45434484	45534484	0.368434	IMPG2
1	45434702	45534702	0.371913	IMPG2
1	45434868	45534868	0.342326	IMPG2
1	45440157	45540157	0.34547	IMPG2
1	45445785	45545785	0.346579	IMPG2
1	45445927	45545927	0.362551	IMPG2
1	45445987	45545987	0.365219	IMPG2
1	45448028	45548028	0.344595	IMPG2
1	45449654	45549654	0.344279	IMPG2
1	45450083	45550083	0.344304	IMPG2
1	45450088	45550088	0.342193	IMPG2
1	45450097	45550097	0.370394	IMPG2
1	45450743	45550743	0.365856	IMPG2
1	45450846	45550846	0.353686	IMPG2
1	45451166	45551166	0.354609	IMPG2
1	45451180	45551180	0.387585	IMPG2
1	45451226	45551226	0.358849	IMPG2
1	45452088	45552088	0.372479	IMPG2
1	45452819	45552819	0.385635	IMPG2
1	45452958	45552958	0.390394	IMPG2
1	45453234	45553234	0.385208	IMPG2
1	45453323	45553323	0.380111	IMPG2
1	45453681	45553681	0.391789	IMPG2
1	45453727	45553727	0.391789	IMPG2
1	45453952	45553952	0.394506	IMPG2
1	45454007	45554007	0.408569	IMPG2
1	45454097	45554097	0.38444	IMPG2
1	45454461	45554461	0.369931	IMPG2
1	45454552	45554552	0.376986	IMPG2
1	45454631	45554631	0.375666	IMPG2
1	45454633	45554633	0.382787	IMPG2
1	45454899	45554899	0.361428	IMPG2
1	45454913	45554913	0.358833	IMPG2

1	45455386	45555386	0.356912	IMPG2
1	45456127	45556127	0.373892	IMPG2
1	45456143	45556143	0.376099	IMPG2
1	45456825	45556825	0.346919	IMPG2
1	45456837	45556837	0.346919	IMPG2
1	45456890	45556890	0.359696	IMPG2
1	45456907	45556907	0.347218	IMPG2
1	45457644	45557644	0.350551	IMPG2
1	45458688	45558688	0.361715	IMPG2
1	45458744	45558744	0.360412	IMPG2
1	45459060	45559060	0.361526	IMPG2
1	45459148	45559148	0.365406	IMPG2
1	45459165	45559165	0.360719	IMPG2
1	45459482	45559482	0.366989	IMPG2
1	45459519	45559519	0.367038	IMPG2
1	45459527	45559527	0.3693	IMPG2
1	45459842	45559842	0.369427	IMPG2
1	45459895	45559895	0.393329	IMPG2
1	45459896	45559896	0.376132	IMPG2
1	45459993	45559993	0.402855	IMPG2
1	45459995	45559995	0.402798	IMPG2
1	45460019	45560019	0.41296	IMPG2
1	45460256	45560256	0.366209	IMPG2
1	45460391	45560391	0.377141	IMPG2
1	45460505	45560505	0.379041	IMPG2
1	45460506	45560506	0.378848	IMPG2
1	45460522	45560522	0.378848	IMPG2
1	45460553	45560553	0.377026	IMPG2
1	45460616	45560616	0.376659	IMPG2
1	45460635	45560635	0.375247	IMPG2
1	45460795	45560795	0.384346	IMPG2
1	45460808	45560808	0.38635	IMPG2
1	45460837	45560837	0.386355	IMPG2
1	45460884	45560884	0.388785	IMPG2
1	45460884	45560884	0.388785	SENP7
1	45460929	45560929	0.381747	IMPG2
1	45460929	45560929	0.381747	SENP7
1	45461098	45561098	0.381747	IMPG2
1	45461098	45561098	0.381747	SENP7
1	45461152	45561152	0.376235	IMPG2
1	45461152	45561152	0.376235	SENP7
1	45461188	45561188	0.388279	IMPG2
1	45461188	45561188	0.388279	SENP7
1	45461249	45561249	0.391723	IMPG2
1	45461249	45561249	0.391723	SENP7
1	45461405	45561405	0.386938	IMPG2
1	45461405	45561405	0.386938	SENP7
1	45461591	45561591	0.388377	IMPG2
1	45461591	45561591	0.388377	SENP7
1	45461667	45561667	0.380729	IMPG2
1	45461667	45561667	0.380729	SENP7
1	45461723	45561723	0.381891	IMPG2
1	45461723	45561723	0.381891	SENP7
1	45461738	45561738	0.397883	IMPG2
1	45461738	45561738	0.397883	SENP7
1	45461849	45561849	0.381788	IMPG2
1	45461849	45561849	0.381788	SENP7
1	45461873	45561873	0.379027	IMPG2

1	45461873	45561873	0.379027	SENP7
1	45462312	45562312	0.38669	IMPG2
1	45462312	45562312	0.38669	SENP7
1	45462323	45562323	0.388754	IMPG2
1	45462323	45562323	0.388754	SENP7
1	45462330	45562330	0.382662	IMPG2
1	45462330	45562330	0.382662	SENP7
1	45462430	45562430	0.388305	IMPG2
1	45462430	45562430	0.388305	SENP7
1	45462477	45562477	0.397994	IMPG2
1	45462477	45562477	0.397994	SENP7
1	45462540	45562540	0.38606	IMPG2
1	45462540	45562540	0.38606	SENP7
1	45462736	45562736	0.381774	IMPG2
1	45462736	45562736	0.381774	SENP7
1	45462858	45562858	0.371054	IMPG2
1	45462858	45562858	0.371054	SENP7
1	45463813	45563813	0.353689	IMPG2
1	45463813	45563813	0.353689	SENP7
1	45464170	45564170	0.35746	IMPG2
1	45464170	45564170	0.35746	SENP7
1	45464323	45564323	0.3751	IMPG2
1	45464323	45564323	0.3751	SENP7
1	45464390	45564390	0.385113	IMPG2
1	45464390	45564390	0.385113	SENP7
1	45464428	45564428	0.382748	IMPG2
1	45464428	45564428	0.382748	SENP7
1	45464777	45564777	0.382171	IMPG2
1	45464777	45564777	0.382171	SENP7
1	45464968	45564968	0.392038	IMPG2
1	45464968	45564968	0.392038	SENP7
1	45465067	45565067	0.396779	IMPG2
1	45465067	45565067	0.396779	SENP7
1	45465160	45565160	0.386617	IMPG2
1	45465160	45565160	0.386617	SENP7
1	45465337	45565337	0.390149	IMPG2
1	45465337	45565337	0.390149	SENP7
1	45465532	45565532	0.367574	IMPG2
1	45465532	45565532	0.367574	SENP7
1	45465553	45565553	0.367574	IMPG2
1	45465553	45565553	0.367574	SENP7
1	45465589	45565589	0.371571	IMPG2
1	45465589	45565589	0.371571	SENP7
1	45465636	45565636	0.36413	IMPG2
1	45465636	45565636	0.36413	SENP7
1	45465814	45565814	0.362326	IMPG2
1	45465814	45565814	0.362326	SENP7
1	45466557	45566557	0.384713	IMPG2
1	45466557	45566557	0.384713	SENP7
1	45466780	45566780	0.410847	IMPG2
1	45466780	45566780	0.410847	SENP7
1	45466858	45566858	0.396559	IMPG2
1	45466858	45566858	0.396559	SENP7
1	45466955	45566955	0.399444	IMPG2
1	45466955	45566955	0.399444	SENP7
1	45467006	45567006	0.366263	IMPG2
1	45467006	45567006	0.366263	SENP7
1	45467216	45567216	0.342714	IMPG2

1	45467216	45567216	0.342714	SENP7
1	45467851	45567851	0.35528	IMPG2
1	45467851	45567851	0.35528	SENP7
1	45467879	45567879	0.345584	IMPG2
1	45467879	45567879	0.345584	SENP7
1	45468018	45568018	0.344778	IMPG2
1	45468018	45568018	0.344778	SENP7
1	45468168	45568168	0.394537	IMPG2
1	45468168	45568168	0.394537	SENP7
1	45468561	45568561	0.394441	IMPG2
1	45468561	45568561	0.394441	SENP7
1	45468710	45568710	0.402685	IMPG2
1	45468710	45568710	0.402685	SENP7
1	45468959	45568959	0.393237	IMPG2
1	45468959	45568959	0.393237	SENP7
1	45469123	45569123	0.392528	IMPG2
1	45469123	45569123	0.392528	SENP7
1	45469185	45569185	0.403366	IMPG2
1	45469185	45569185	0.403366	SENP7
1	45469203	45569203	0.395671	IMPG2
1	45469203	45569203	0.395671	SENP7
1	45469212	45569212	0.410147	IMPG2
1	45469212	45569212	0.410147	SENP7
1	45469266	45569266	0.403357	IMPG2
1	45469266	45569266	0.403357	SENP7
1	45469509	45569509	0.412519	IMPG2
1	45469509	45569509	0.412519	SENP7
1	45469710	45569710	0.404955	IMPG2
1	45469710	45569710	0.404955	SENP7
1	45469739	45569739	0.400665	IMPG2
1	45469739	45569739	0.400665	SENP7
1	45469831	45569831	0.414194	IMPG2
1	45469831	45569831	0.414194	SENP7
1	45469927	45569927	0.386125	IMPG2
1	45469927	45569927	0.386125	SENP7
1	45470037	45570037	0.389901	IMPG2
1	45470037	45570037	0.389901	SENP7
1	45470229	45570229	0.392473	IMPG2
1	45470229	45570229	0.392473	SENP7
1	45470568	45570568	0.380079	IMPG2
1	45470568	45570568	0.380079	SENP7
1	45470585	45570585	0.386829	IMPG2
1	45470585	45570585	0.386829	SENP7
1	45470595	45570595	0.398068	IMPG2
1	45470595	45570595	0.398068	SENP7
1	45470776	45570776	0.378066	IMPG2
1	45470776	45570776	0.378066	SENP7
1	45471144	45571144	0.372114	IMPG2
1	45471144	45571144	0.372114	SENP7
1	45471470	45571470	0.356964	IMPG2
1	45471470	45571470	0.356964	SENP7
1	45471814	45571814	0.372262	IMPG2
1	45471814	45571814	0.372262	SENP7
1	45471937	45571937	0.36616	IMPG2
1	45471937	45571937	0.36616	SENP7
1	45472028	45572028	0.371436	IMPG2
1	45472028	45572028	0.371436	SENP7
1	45472094	45572094	0.3657	IMPG2

1	45472094	45572094	0.3657	SENP7
1	45472141	45572141	0.3657	IMPG2
1	45472141	45572141	0.3657	SENP7
1	45472245	45572245	0.367697	IMPG2
1	45472245	45572245	0.367697	SENP7
1	45472405	45572405	0.367697	IMPG2
1	45472405	45572405	0.367697	SENP7
1	45472532	45572532	0.374392	IMPG2
1	45472532	45572532	0.374392	SENP7
1	45472803	45572803	0.362498	IMPG2
1	45472803	45572803	0.362498	SENP7
1	45472982	45572982	0.344078	IMPG2
1	45472982	45572982	0.344078	SENP7
1	45473042	45573042	0.354625	IMPG2
1	45473042	45573042	0.354625	SENP7
1	45473688	45573688	0.377323	IMPG2
1	45473688	45573688	0.377323	SENP7
1	45473972	45573972	0.373858	IMPG2
1	45473972	45573972	0.373858	SENP7
1	45474022	45574022	0.355484	IMPG2
1	45474022	45574022	0.355484	SENP7
1	45474084	45574084	0.379645	IMPG2
1	45474084	45574084	0.379645	SENP7
1	45474632	45574632	0.382636	IMPG2
1	45474632	45574632	0.382636	SENP7
1	45474697	45574697	0.402429	IMPG2
1	45474697	45574697	0.402429	SENP7
1	45474846	45574846	0.374674	IMPG2
1	45474846	45574846	0.374674	SENP7
1	45475127	45575127	0.380625	IMPG2
1	45475127	45575127	0.380625	SENP7
1	45475356	45575356	0.349951	IMPG2
1	45475356	45575356	0.349951	SENP7
1	45477805	45577805	0.384325	IMPG2
1	45477805	45577805	0.384325	SENP7
1	45478706	45578706	0.402048	IMPG2
1	45478706	45578706	0.402048	SENP7
1	45479095	45579095	0.371099	IMPG2
1	45479095	45579095	0.371099	SENP7
1	45479310	45579310	0.411658	IMPG2
1	45479310	45579310	0.411658	SENP7
1	45479398	45579398	0.421494	IMPG2
1	45479398	45579398	0.421494	SENP7
1	45479628	45579628	0.398264	IMPG2
1	45479628	45579628	0.398264	SENP7
1	45479948	45579948	0.408242	IMPG2
1	45479948	45579948	0.408242	SENP7
1	45480398	45580398	0.406147	IMPG2
1	45480398	45580398	0.406147	SENP7
1	45480562	45580562	0.414444	IMPG2
1	45480562	45580562	0.414444	SENP7
1	45480662	45580662	0.412563	IMPG2
1	45480662	45580662	0.412563	SENP7
1	45480737	45580737	0.41403	IMPG2
1	45480737	45580737	0.41403	SENP7
1	45481247	45581247	0.419165	IMPG2
1	45481247	45581247	0.419165	SENP7
1	45481279	45581279	0.422844	IMPG2

1	45481279	45581279	0.422844	SENP7
1	45481491	45581491	0.430861	IMPG2
1	45481491	45581491	0.430861	SENP7
1	45481686	45581686	0.434635	IMPG2
1	45481686	45581686	0.434635	SENP7
1	45481760	45581760	0.430992	IMPG2
1	45481760	45581760	0.430992	SENP7
1	45481888	45581888	0.422509	IMPG2
1	45481888	45581888	0.422509	SENP7
1	45481965	45581965	0.424742	IMPG2
1	45481965	45581965	0.424742	SENP7
1	45482129	45582129	0.422339	IMPG2
1	45482129	45582129	0.422339	SENP7
1	45482445	45582445	0.404913	IMPG2
1	45482445	45582445	0.404913	SENP7
1	45482513	45582513	0.418864	IMPG2
1	45482513	45582513	0.418864	SENP7
1	45482737	45582737	0.41803	IMPG2
1	45482737	45582737	0.41803	SENP7
1	45482883	45582883	0.449792	IMPG2
1	45482883	45582883	0.449792	SENP7
1	45482918	45582918	0.458607	IMPG2
1	45482918	45582918	0.458607	SENP7
1	45483038	45583038	0.457216	IMPG2
1	45483038	45583038	0.457216	SENP7
1	45483153	45583153	0.46588	IMPG2
1	45483153	45583153	0.46588	SENP7
1	45483682	45583682	0.457276	IMPG2
1	45483682	45583682	0.457276	SENP7
1	45483725	45583725	0.47459	IMPG2
1	45483725	45583725	0.47459	SENP7
1	45483763	45583763	0.490719	IMPG2
1	45483763	45583763	0.490719	SENP7
1	45483819	45583819	0.490719	IMPG2
1	45483819	45583819	0.490719	SENP7
1	45483860	45583860	0.49351	IMPG2
1	45483860	45583860	0.49351	SENP7
1	45483958	45583958	0.540401	IMPG2
1	45483958	45583958	0.540401	SENP7
1	45483971	45583971	0.538838	IMPG2
1	45483971	45583971	0.538838	SENP7
1	45483981	45583981	0.55488	IMPG2
1	45483981	45583981	0.55488	SENP7
1	45483982	45583982	0.55488	IMPG2
1	45483982	45583982	0.55488	SENP7
1	45483989	45583989	0.599111	IMPG2
1	45483989	45583989	0.599111	SENP7
1	45484000	45584000	0.52308	IMPG2
1	45484000	45584000	0.52308	SENP7
1	45484122	45584122	0.514182	IMPG2
1	45484122	45584122	0.514182	SENP7
1	45484193	45584193	0.507414	IMPG2
1	45484193	45584193	0.507414	SENP7
1	45484232	45584232	0.523121	IMPG2
1	45484232	45584232	0.523121	SENP7
1	45484398	45584398	0.522833	IMPG2
1	45484398	45584398	0.522833	SENP7
1	45484487	45584487	0.519585	IMPG2

1	45484487	45584487	0.519585	SENP7
1	45484496	45584496	0.509326	IMPG2
1	45484496	45584496	0.509326	SENP7
1	45484558	45584558	0.509326	IMPG2
1	45484558	45584558	0.509326	SENP7
1	45484596	45584596	0.503405	IMPG2
1	45484596	45584596	0.503405	SENP7
1	45484620	45584620	0.493911	IMPG2
1	45484620	45584620	0.493911	SENP7
1	45484847	45584847	0.486996	IMPG2
1	45484847	45584847	0.486996	SENP7
1	45485165	45585165	0.494522	IMPG2
1	45485165	45585165	0.494522	SENP7
1	45485182	45585182	0.503584	IMPG2
1	45485182	45585182	0.503584	SENP7
1	45485420	45585420	0.501167	IMPG2
1	45485420	45585420	0.501167	SENP7
1	45485498	45585498	0.493378	IMPG2
1	45485498	45585498	0.493378	SENP7
1	45485585	45585585	0.501036	IMPG2
1	45485585	45585585	0.501036	SENP7
1	45485593	45585593	0.503611	IMPG2
1	45485593	45585593	0.503611	SENP7
1	45485596	45585596	0.513554	IMPG2
1	45485596	45585596	0.513554	SENP7
1	45485718	45585718	0.514256	IMPG2
1	45485718	45585718	0.514256	SENP7
1	45485804	45585804	0.507187	IMPG2
1	45485804	45585804	0.507187	SENP7
1	45485966	45585966	0.493046	IMPG2
1	45485966	45585966	0.493046	SENP7
1	45486160	45586160	0.488294	IMPG2
1	45486160	45586160	0.488294	SENP7
1	45486499	45586499	0.479728	IMPG2
1	45486499	45586499	0.479728	SENP7
1	45486687	45586687	0.446055	IMPG2
1	45486687	45586687	0.446055	SENP7
1	45486816	45586816	0.441475	IMPG2
1	45486816	45586816	0.441475	SENP7
1	45487373	45587373	0.468876	IMPG2
1	45487373	45587373	0.468876	SENP7
1	45487393	45587393	0.479292	IMPG2
1	45487393	45587393	0.479292	SENP7
1	45487604	45587604	0.473403	IMPG2
1	45487604	45587604	0.473403	SENP7
1	45487610	45587610	0.484152	IMPG2
1	45487610	45587610	0.484152	SENP7
1	45487787	45587787	0.464804	IMPG2
1	45487787	45587787	0.464804	SENP7
1	45487998	45587998	0.463045	IMPG2
1	45487998	45587998	0.463045	SENP7
1	45488248	45588248	0.46577	IMPG2
1	45488248	45588248	0.46577	SENP7
1	45488499	45588499	0.460655	IMPG2
1	45488499	45588499	0.460655	SENP7
1	45488574	45588574	0.451663	IMPG2
1	45488574	45588574	0.451663	SENP7
1	45488616	45588616	0.474946	IMPG2

1	45488616	45588616	0.474946	SENP7
1	45488905	45588905	0.464214	IMPG2
1	45488905	45588905	0.464214	SENP7
1	45489265	45589265	0.446764	IMPG2
1	45489265	45589265	0.446764	SENP7
1	45489281	45589281	0.422194	IMPG2
1	45489281	45589281	0.422194	SENP7
1	45489499	45589499	0.422194	IMPG2
1	45489499	45589499	0.422194	SENP7
1	45489609	45589609	0.422194	IMPG2
1	45489609	45589609	0.422194	SENP7
1	45489731	45589731	0.430985	IMPG2
1	45489731	45589731	0.430985	SENP7
1	45489946	45589946	0.460702	IMPG2
1	45489946	45589946	0.460702	SENP7
1	45490177	45590177	0.479775	IMPG2
1	45490177	45590177	0.479775	SENP7
1	45490658	45590658	0.484344	IMPG2
1	45490658	45590658	0.484344	SENP7
1	45490667	45590667	0.516475	IMPG2
1	45490667	45590667	0.516475	SENP7
1	45490709	45590709	0.533704	IMPG2
1	45490709	45590709	0.533704	SENP7
1	45490730	45590730	0.492865	IMPG2
1	45490730	45590730	0.492865	SENP7
1	45490859	45590859	0.470445	IMPG2
1	45490859	45590859	0.470445	SENP7
1	45490864	45590864	0.478789	IMPG2
1	45490864	45590864	0.478789	SENP7
1	45490885	45590885	0.486033	IMPG2
1	45490885	45590885	0.486033	SENP7
1	45490947	45590947	0.465539	IMPG2
1	45490947	45590947	0.465539	SENP7
1	45491153	45591153	0.4315	IMPG2
1	45491153	45591153	0.4315	SENP7
1	45491163	45591163	0.433414	IMPG2
1	45491163	45591163	0.433414	SENP7
1	45492237	45592237	0.437215	IMPG2
1	45492237	45592237	0.437215	SENP7
1	45492345	45592345	0.427501	IMPG2
1	45492345	45592345	0.427501	SENP7
1	45492421	45592421	0.435981	IMPG2
1	45492421	45592421	0.435981	SENP7
1	45492621	45592621	0.419818	IMPG2
1	45492621	45592621	0.419818	SENP7
1	45492668	45592668	0.39919	IMPG2
1	45492668	45592668	0.39919	SENP7
1	45492678	45592678	0.388369	IMPG2
1	45492678	45592678	0.388369	SENP7
1	45492859	45592859	0.395327	IMPG2
1	45492859	45592859	0.395327	SENP7
1	45493329	45593329	0.374244	IMPG2
1	45493329	45593329	0.374244	SENP7
1	45494225	45594225	0.387376	IMPG2
1	45494225	45594225	0.387376	SENP7
1	45494670	45594670	0.35619	IMPG2
1	45494670	45594670	0.35619	SENP7
1	45494675	45594675	0.36997	IMPG2

1	45494675	45594675	0.36997	SENP7
1	45494704	45594704	0.378752	IMPG2
1	45494704	45594704	0.378752	SENP7
1	45494962	45594962	0.386018	IMPG2
1	45494962	45594962	0.386018	SENP7
1	45495256	45595256	0.380233	IMPG2
1	45495256	45595256	0.380233	SENP7
1	45495293	45595293	0.383675	IMPG2
1	45495293	45595293	0.383675	SENP7
1	45495622	45595622	0.37569	IMPG2
1	45495622	45595622	0.37569	SENP7
1	45495646	45595646	0.407979	IMPG2
1	45495646	45595646	0.407979	SENP7
1	45495685	45595685	0.378825	IMPG2
1	45495685	45595685	0.378825	SENP7
1	45496035	45596035	0.378715	IMPG2
1	45496035	45596035	0.378715	SENP7
1	45496083	45596083	0.372816	IMPG2
1	45496083	45596083	0.372816	SENP7
1	45496338	45596338	0.35088	IMPG2
1	45496338	45596338	0.35088	SENP7
1	45573239	45673239	0.358304	SENP7
1	45573302	45673302	0.357145	SENP7
1	45778757	45878757	0.344432	LOC108637877
1	45778757	45878757	0.344432	RPL24
1	45778757	45878757	0.344432	ZBTB11
1	45778757	45878757	0.344432	CEP97
1	45778757	45878757	0.344432	NXPE3
1	45778836	45878836	0.351099	LOC108637877
1	45778836	45878836	0.351099	RPL24
1	45778836	45878836	0.351099	ZBTB11
1	45778836	45878836	0.351099	CEP97
1	45778836	45878836	0.351099	NXPE3
1	45779042	45879042	0.351099	LOC108637877
1	45779042	45879042	0.351099	RPL24
1	45779042	45879042	0.351099	ZBTB11
1	45779042	45879042	0.351099	CEP97
1	45779042	45879042	0.351099	NXPE3
1	45779266	45879266	0.364477	LOC108637877
1	45779266	45879266	0.364477	RPL24
1	45779266	45879266	0.364477	ZBTB11
1	45779266	45879266	0.364477	CEP97
1	45779266	45879266	0.364477	NXPE3
1	45779289	45879289	0.353697	LOC108637877
1	45779289	45879289	0.353697	RPL24
1	45779289	45879289	0.353697	ZBTB11
1	45779289	45879289	0.353697	CEP97
1	45779289	45879289	0.353697	NXPE3
1	45779308	45879308	0.345433	LOC108637877
1	45779308	45879308	0.345433	RPL24
1	45779308	45879308	0.345433	ZBTB11
1	45779308	45879308	0.345433	CEP97
1	45779308	45879308	0.345433	NXPE3
1	45779357	45879357	0.343313	LOC108637877
1	45779357	45879357	0.343313	RPL24
1	45779357	45879357	0.343313	ZBTB11
1	45779357	45879357	0.343313	CEP97
1	45779357	45879357	0.343313	NXPE3

1	45779948	45879948	0.344079	LOC108637877
1	45779948	45879948	0.344079	RPL24
1	45779948	45879948	0.344079	ZBTB11
1	45779948	45879948	0.344079	CEP97
1	45779948	45879948	0.344079	NXPE3
1	45779954	45879954	0.342962	LOC108637877
1	45779954	45879954	0.342962	RPL24
1	45779954	45879954	0.342962	ZBTB11
1	45779954	45879954	0.342962	CEP97
1	45779954	45879954	0.342962	NXPE3
1	45780119	45880119	0.351522	LOC108637877
1	45780119	45880119	0.351522	RPL24
1	45780119	45880119	0.351522	ZBTB11
1	45780119	45880119	0.351522	CEP97
1	45780119	45880119	0.351522	NXPE3
1	45780144	45880144	0.349611	LOC108637877
1	45780144	45880144	0.349611	RPL24
1	45780144	45880144	0.349611	ZBTB11
1	45780144	45880144	0.349611	CEP97
1	45780144	45880144	0.349611	NXPE3
1	45780163	45880163	0.352244	LOC108637877
1	45780163	45880163	0.352244	RPL24
1	45780163	45880163	0.352244	ZBTB11
1	45780163	45880163	0.352244	CEP97
1	45780163	45880163	0.352244	NXPE3
1	45780328	45880328	0.352244	LOC108637877
1	45780328	45880328	0.352244	RPL24
1	45780328	45880328	0.352244	ZBTB11
1	45780328	45880328	0.352244	CEP97
1	45780328	45880328	0.352244	NXPE3
1	45780331	45880331	0.352052	LOC108637877
1	45780331	45880331	0.352052	RPL24
1	45780331	45880331	0.352052	ZBTB11
1	45780331	45880331	0.352052	CEP97
1	45780331	45880331	0.352052	NXPE3
1	45780417	45880417	0.342666	LOC108637877
1	45780417	45880417	0.342666	RPL24
1	45780417	45880417	0.342666	ZBTB11
1	45780417	45880417	0.342666	CEP97
1	45780417	45880417	0.342666	NXPE3
1	45780720	45880720	0.372016	LOC108637877
1	45780720	45880720	0.372016	RPL24
1	45780720	45880720	0.372016	ZBTB11
1	45780720	45880720	0.372016	CEP97
1	45780720	45880720	0.372016	NXPE3
1	45780999	45880999	0.358206	LOC108637877
1	45780999	45880999	0.358206	RPL24
1	45780999	45880999	0.358206	ZBTB11
1	45780999	45880999	0.358206	CEP97
1	45780999	45880999	0.358206	NXPE3
1	45781399	45881399	0.364788	LOC108637877
1	45781399	45881399	0.364788	RPL24
1	45781399	45881399	0.364788	ZBTB11
1	45781399	45881399	0.364788	CEP97
1	45781399	45881399	0.364788	NXPE3
1	46599042	46699042	0.356847	ZPLD1
1	46599051	46699051	0.364046	ZPLD1
1	46599079	46699079	0.350242	ZPLD1

1	46599099	46699099	0.350242	ZPLD1
1	46599123	46699123	0.346672	ZPLD1
1	46599124	46699124	0.384506	ZPLD1
1	46599139	46699139	0.363828	ZPLD1
1	46599170	46699170	0.369922	ZPLD1
1	46599182	46699182	0.368585	ZPLD1
1	46599232	46699232	0.367463	ZPLD1
1	46599238	46699238	0.363121	ZPLD1
1	46599248	46699248	0.363121	ZPLD1
1	46599249	46699249	0.361367	ZPLD1
1	46599257	46699257	0.359417	ZPLD1
1	46599381	46699381	0.376072	ZPLD1
1	46599404	46699404	0.391085	ZPLD1
1	46599405	46699405	0.386099	ZPLD1
1	46599467	46699467	0.391747	ZPLD1
1	46599663	46699663	0.407857	ZPLD1
1	46599779	46699779	0.4132	ZPLD1
1	46599780	46699780	0.385824	ZPLD1
1	46599786	46699786	0.385824	ZPLD1
1	46599812	46699812	0.386216	ZPLD1
1	46599841	46699841	0.369816	ZPLD1
1	46600076	46700076	0.365368	ZPLD1
1	46600084	46700084	0.365368	ZPLD1
1	46600135	46700135	0.365368	ZPLD1
1	50050517	50150517	0.343238	ALCAM
1	50050518	50150518	0.343238	ALCAM
1	50050588	50150588	0.342604	ALCAM
1	50052146	50152146	0.347594	ALCAM
1	50052150	50152150	0.347594	ALCAM
1	50052191	50152191	0.356905	ALCAM
1	50052200	50152200	0.350573	ALCAM
1	50052202	50152202	0.350573	ALCAM
1	50052222	50152222	0.350573	ALCAM
1	50052239	50152239	0.347594	ALCAM
1	50052269	50152269	0.347594	ALCAM
1	50052302	50152302	0.359874	ALCAM
1	50052335	50152335	0.343476	ALCAM
1	50052475	50152475	0.343739	ALCAM
1	50052533	50152533	0.347799	ALCAM
1	50053132	50153132	0.346858	ALCAM
1	50053178	50153178	0.347966	ALCAM
1	50053185	50153185	0.348377	ALCAM
1	50055207	50155207	0.377272	ALCAM
1	50055417	50155417	0.381921	ALCAM
1	50055604	50155604	0.388216	ALCAM
1	50055659	50155659	0.384524	ALCAM
1	50055834	50155834	0.393168	ALCAM
1	50055842	50155842	0.393168	ALCAM
1	50055844	50155844	0.382843	ALCAM
1	50055859	50155859	0.391417	ALCAM
1	50056104	50156104	0.401774	ALCAM
1	50056224	50156224	0.388576	ALCAM
1	50056231	50156231	0.392921	ALCAM
1	50056271	50156271	0.389661	ALCAM
1	50056284	50156284	0.395739	ALCAM
1	50056344	50156344	0.394505	ALCAM
1	50056363	50156363	0.39379	ALCAM
1	50056381	50156381	0.392783	ALCAM

1	50056397	50156397	0.390877	ALCAM
1	50056427	50156427	0.409557	ALCAM
1	50056460	50156460	0.406176	ALCAM
1	50056463	50156463	0.406176	ALCAM
1	50056532	50156532	0.406176	ALCAM
1	50056604	50156604	0.406042	ALCAM
1	50056604	50156604	0.406042	CBLB
1	50056607	50156607	0.406042	ALCAM
1	50056607	50156607	0.406042	CBLB
1	50056683	50156683	0.408475	ALCAM
1	50056683	50156683	0.408475	CBLB
1	50056688	50156688	0.402917	ALCAM
1	50056688	50156688	0.402917	CBLB
1	50056863	50156863	0.406547	ALCAM
1	50056863	50156863	0.406547	CBLB
1	50056872	50156872	0.403794	ALCAM
1	50056872	50156872	0.403794	CBLB
1	50056923	50156923	0.409143	ALCAM
1	50056923	50156923	0.409143	CBLB
1	50056989	50156989	0.401233	ALCAM
1	50056989	50156989	0.401233	CBLB
1	50056995	50156995	0.400158	ALCAM
1	50056995	50156995	0.400158	CBLB
1	50057132	50157132	0.405453	ALCAM
1	50057132	50157132	0.405453	CBLB
1	50057166	50157166	0.405453	ALCAM
1	50057166	50157166	0.405453	CBLB
1	50057173	50157173	0.420034	ALCAM
1	50057173	50157173	0.420034	CBLB
1	50057249	50157249	0.393271	ALCAM
1	50057249	50157249	0.393271	CBLB
1	50057285	50157285	0.391932	ALCAM
1	50057285	50157285	0.391932	CBLB
1	50057305	50157305	0.387656	ALCAM
1	50057305	50157305	0.387656	CBLB
1	50057318	50157318	0.386288	ALCAM
1	50057318	50157318	0.386288	CBLB
1	50057363	50157363	0.390683	ALCAM
1	50057363	50157363	0.390683	CBLB
1	50057479	50157479	0.377079	ALCAM
1	50057479	50157479	0.377079	CBLB
1	50057526	50157526	0.378859	ALCAM
1	50057526	50157526	0.378859	CBLB
1	50057543	50157543	0.375299	ALCAM
1	50057543	50157543	0.375299	CBLB
1	50057567	50157567	0.368374	ALCAM
1	50057567	50157567	0.368374	CBLB
1	50057597	50157597	0.347744	ALCAM
1	50057597	50157597	0.347744	CBLB
1	50057631	50157631	0.352152	ALCAM
1	50057631	50157631	0.352152	CBLB
1	50057676	50157676	0.349259	ALCAM
1	50057676	50157676	0.349259	CBLB
1	50057705	50157705	0.350465	ALCAM
1	50057705	50157705	0.350465	CBLB
1	50057719	50157719	0.350605	ALCAM
1	50057719	50157719	0.350605	CBLB
1	50057733	50157733	0.349646	ALCAM

1	50057733	50157733	0.349646	CBLB
1	50057751	50157751	0.349646	ALCAM
1	50057751	50157751	0.349646	CBLB
1	50057756	50157756	0.353253	ALCAM
1	50057756	50157756	0.353253	CBLB
1	50059485	50159485	0.345067	ALCAM
1	50059485	50159485	0.345067	CBLB
1	50063022	50163022	0.368012	ALCAM
1	50063022	50163022	0.368012	CBLB
1	50063081	50163081	0.375127	ALCAM
1	50063081	50163081	0.375127	CBLB
1	50063362	50163362	0.374723	ALCAM
1	50063362	50163362	0.374723	CBLB
1	50063465	50163465	0.3699	ALCAM
1	50063465	50163465	0.3699	CBLB
1	50063521	50163521	0.443855	ALCAM
1	50063521	50163521	0.443855	CBLB
1	50063528	50163528	0.445205	ALCAM
1	50063528	50163528	0.445205	CBLB
1	50063541	50163541	0.434188	ALCAM
1	50063541	50163541	0.434188	CBLB
1	50063781	50163781	0.463933	ALCAM
1	50063781	50163781	0.463933	CBLB
1	50063815	50163815	0.462497	ALCAM
1	50063815	50163815	0.462497	CBLB
1	50064026	50164026	0.45013	ALCAM
1	50064026	50164026	0.45013	CBLB
1	50064191	50164191	0.450054	ALCAM
1	50064191	50164191	0.450054	CBLB
1	50064211	50164211	0.458446	ALCAM
1	50064211	50164211	0.458446	CBLB
1	50064322	50164322	0.458446	ALCAM
1	50064322	50164322	0.458446	CBLB
1	50064331	50164331	0.458446	ALCAM
1	50064331	50164331	0.458446	CBLB
1	50064393	50164393	0.468005	ALCAM
1	50064393	50164393	0.468005	CBLB
1	50064668	50164668	0.463676	ALCAM
1	50064668	50164668	0.463676	CBLB
1	50064740	50164740	0.468313	ALCAM
1	50064740	50164740	0.468313	CBLB
1	50064833	50164833	0.45365	ALCAM
1	50064833	50164833	0.45365	CBLB
1	50065672	50165672	0.413731	ALCAM
1	50065672	50165672	0.413731	CBLB
1	50065914	50165914	0.407128	ALCAM
1	50065914	50165914	0.407128	CBLB
1	50066364	50166364	0.397757	ALCAM
1	50066364	50166364	0.397757	CBLB
1	50066382	50166382	0.397757	ALCAM
1	50066382	50166382	0.397757	CBLB
1	50066456	50166456	0.403196	ALCAM
1	50066456	50166456	0.403196	CBLB
1	50066661	50166661	0.405363	ALCAM
1	50066661	50166661	0.405363	CBLB
1	50066788	50166788	0.404318	ALCAM
1	50066788	50166788	0.404318	CBLB
1	50066884	50166884	0.405778	ALCAM

1	50066884	50166884	0.405778	CBLB
1	50066910	50166910	0.405778	ALCAM
1	50066910	50166910	0.405778	CBLB
1	50067033	50167033	0.401264	ALCAM
1	50067033	50167033	0.401264	CBLB
1	50067321	50167321	0.402172	ALCAM
1	50067321	50167321	0.402172	CBLB
1	50067692	50167692	0.425894	ALCAM
1	50067692	50167692	0.425894	CBLB
1	50067796	50167796	0.441391	ALCAM
1	50067796	50167796	0.441391	CBLB
1	50067923	50167923	0.422611	ALCAM
1	50067923	50167923	0.422611	CBLB
1	50067995	50167995	0.422611	ALCAM
1	50067995	50167995	0.422611	CBLB
1	50068058	50168058	0.412009	ALCAM
1	50068058	50168058	0.412009	CBLB
1	50068266	50168266	0.456034	CBLB
1	50068416	50168416	0.446045	CBLB
1	50068620	50168620	0.474325	CBLB
1	50068640	50168640	0.472198	CBLB
1	50068814	50168814	0.466376	CBLB
1	50068867	50168867	0.457606	CBLB
1	50069386	50169386	0.459911	CBLB
1	50069542	50169542	0.458208	CBLB
1	50069612	50169612	0.443985	CBLB
1	50070390	50170390	0.4487	CBLB
1	50070554	50170554	0.443668	CBLB
1	50070824	50170824	0.455785	CBLB
1	50070899	50170899	0.435778	CBLB
1	50071383	50171383	0.436443	CBLB
1	50071535	50171535	0.443543	CBLB
1	50071747	50171747	0.457608	CBLB
1	50071838	50171838	0.44759	CBLB
1	50071947	50171947	0.444856	CBLB
1	50072058	50172058	0.451271	CBLB
1	50072720	50172720	0.453586	CBLB
1	50072927	50172927	0.452258	CBLB
1	50072928	50172928	0.440783	CBLB
1	50073146	50173146	0.458194	CBLB
1	50073268	50173268	0.437563	CBLB
1	50073510	50173510	0.4431	CBLB
1	50073525	50173525	0.427419	CBLB
1	50073584	50173584	0.426454	CBLB
1	50073702	50173702	0.416694	CBLB
1	50073910	50173910	0.443979	CBLB
1	50073927	50173927	0.384305	CBLB
1	50074445	50174445	0.357093	CBLB
1	50076118	50176118	0.358884	CBLB
1	50076119	50176119	0.40263	CBLB
1	50076192	50176192	0.389978	CBLB
1	50076790	50176790	0.403866	CBLB
1	50077252	50177252	0.396547	CBLB
1	50077486	50177486	0.418282	CBLB
1	50077624	50177624	0.439474	CBLB
1	50077936	50177936	0.403505	CBLB
1	50077939	50177939	0.412975	CBLB
1	50078089	50178089	0.391254	CBLB

1	50078151	50178151	0.390637	CBLB
1	50078162	50178162	0.390637	CBLB
1	50078319	50178319	0.390637	CBLB
1	50078405	50178405	0.390414	CBLB
1	50078981	50178981	0.390515	CBLB
1	50079202	50179202	0.413191	CBLB
1	50079360	50179360	0.414721	CBLB
1	50079367	50179367	0.405209	CBLB
1	50079606	50179606	0.415939	CBLB
1	50079735	50179735	0.424445	CBLB
1	50079738	50179738	0.425417	CBLB
1	50079781	50179781	0.422569	CBLB
1	50079946	50179946	0.406768	CBLB
1	50080952	50180952	0.360316	CBLB
1	50081099	50181099	0.346364	CBLB
1	50081516	50181516	0.346289	CBLB
1	50081901	50181901	0.369305	CBLB
1	50082275	50182275	0.377319	CBLB
1	50082468	50182468	0.395921	CBLB
1	50082622	50182622	0.407632	CBLB
1	50082852	50182852	0.408438	CBLB
1	50082860	50182860	0.442408	CBLB
1	50082898	50182898	0.429069	CBLB
1	50083003	50183003	0.457139	CBLB
1	50083004	50183004	0.435049	CBLB
1	50083018	50183018	0.429143	CBLB
1	50083218	50183218	0.415822	CBLB
1	50083379	50183379	0.41084	CBLB
1	50083949	50183949	0.415116	CBLB
1	50083957	50183957	0.420142	CBLB
1	50084046	50184046	0.420142	CBLB
1	50084190	50184190	0.421807	CBLB
1	50084202	50184202	0.418651	CBLB
1	50084726	50184726	0.415451	CBLB
1	50084763	50184763	0.438427	CBLB
1	50084852	50184852	0.425175	CBLB
1	50085110	50185110	0.425662	CBLB
1	50085510	50185510	0.412252	CBLB
1	50085553	50185553	0.434995	CBLB
1	50085627	50185627	0.423387	CBLB
1	50085636	50185636	0.415302	CBLB
1	50085812	50185812	0.400583	CBLB
1	50085893	50185893	0.405724	CBLB
1	50085941	50185941	0.390896	CBLB
1	50086997	50186997	0.377352	CBLB
1	50087027	50187027	0.366964	CBLB
1	50087415	50187415	0.366964	CBLB
1	50088275	50188275	0.366011	CBLB
1	50088699	50188699	0.368134	CBLB
1	50089404	50189404	0.347001	CBLB
1	50089878	50189878	0.356915	CBLB
1	50090014	50190014	0.366752	CBLB
1	50091019	50191019	0.371413	CBLB
1	50091086	50191086	0.376254	CBLB
1	50091215	50191215	0.395955	CBLB
1	50091525	50191525	0.393172	CBLB
1	50091693	50191693	0.396858	CBLB
1	50092093	50192093	0.392001	CBLB

1	50092127	50192127	0.393933	CBLB
1	50092578	50192578	0.394256	CBLB
1	50092625	50192625	0.351742	CBLB
1	50092724	50192724	0.342553	CBLB
1	50092763	50192763	0.352971	CBLB
1	50092764	50192764	0.352747	CBLB
1	50092765	50192765	0.352747	CBLB
1	50092769	50192769	0.357304	CBLB
1	50092779	50192779	0.349927	CBLB
1	50092783	50192783	0.343451	CBLB
1	50092970	50192970	0.373184	CBLB
1	50093975	50193975	0.35389	CBLB
1	50094161	50194161	0.396226	CBLB
1	50094480	50194480	0.380204	CBLB
1	50094981	50194981	0.357789	CBLB
1	50095077	50195077	0.387576	CBLB
1	50095169	50195169	0.40213	CBLB
1	50095381	50195381	0.402689	CBLB
1	50095394	50195394	0.393078	CBLB
1	50095657	50195657	0.393078	CBLB
1	50095714	50195714	0.398071	CBLB
1	50095797	50195797	0.42105	CBLB
1	50096061	50196061	0.465412	CBLB
1	50096511	50196511	0.458616	CBLB
1	50096610	50196610	0.461975	CBLB
1	50096739	50196739	0.485902	CBLB
1	50096746	50196746	0.518218	CBLB
1	50096755	50196755	0.498663	CBLB
1	50096951	50196951	0.498187	CBLB
1	50097094	50197094	0.50256	CBLB
1	50097285	50197285	0.485208	CBLB
1	50097286	50197286	0.463567	CBLB
1	50098223	50198223	0.479827	CBLB
1	50098281	50198281	0.495307	CBLB
1	50098412	50198412	0.497308	CBLB
1	50098424	50198424	0.510058	CBLB
1	50098498	50198498	0.511147	CBLB
1	50098774	50198774	0.424597	CBLB
1	50098854	50198854	0.425847	CBLB
1	50099034	50199034	0.458517	CBLB
1	50099095	50199095	0.44761	CBLB
1	50099356	50199356	0.45159	CBLB
1	50099451	50199451	0.453892	CBLB
1	50100102	50200102	0.428363	CBLB
1	50100109	50200109	0.409091	CBLB
1	50100203	50200203	0.401003	CBLB
1	50100402	50200402	0.381967	CBLB
1	50100807	50200807	0.413906	CBLB
1	50101245	50201245	0.435376	CBLB
1	50101303	50201303	0.470607	CBLB
1	50101617	50201617	0.461257	CBLB
1	50101799	50201799	0.380583	CBLB
1	50101868	50201868	0.41828	CBLB
1	50101870	50201870	0.399566	CBLB
1	50101938	50201938	0.376235	CBLB
1	50102195	50202195	0.352048	CBLB
1	50102630	50202630	0.36151	CBLB
1	50102820	50202820	0.377549	CBLB

1	50102960	50202960	0.380594	CBLB
1	50103020	50203020	0.36589	CBLB
1	50103152	50203152	0.354276	CBLB
1	50103216	50203216	0.371738	CBLB
1	50105477	50205477	0.383358	CBLB
1	50105556	50205556	0.38886	CBLB
1	50105632	50205632	0.409598	CBLB
1	50105669	50205669	0.407821	CBLB
1	50105762	50205762	0.399374	CBLB
1	50105979	50205979	0.379937	CBLB
1	50106243	50206243	0.370996	CBLB
1	50106479	50206479	0.370996	CBLB
1	50106499	50206499	0.363685	CBLB
1	50106511	50206511	0.361222	CBLB
1	50106654	50206654	0.355662	CBLB
1	51200149	51300149	0.353721	LOC108636831
1	51200150	51300150	0.3626	LOC108636831
1	51204152	51304152	0.453594	LOC108636831
1	51204414	51304414	0.442628	LOC108636831
1	51204459	51304459	0.442628	LOC108636831
1	51204470	51304470	0.442628	LOC108636831
1	51204548	51304548	0.442628	LOC108636831
1	51204726	51304726	0.442628	LOC108636831
1	51204892	51304892	0.442628	LOC108636831
1	51205014	51305014	0.442628	LOC108636831
1	51205075	51305075	0.442628	LOC108636831
1	51205115	51305115	0.442628	LOC108636831
1	51205162	51305162	0.443391	LOC108636831
1	51205193	51305193	0.443391	LOC108636831
1	51205200	51305200	0.443391	LOC108636831
1	51205250	51305250	0.443391	LOC108636831
1	51205318	51305318	0.442784	LOC108636831
1	51205419	51305419	0.449205	LOC108636831
1	51205421	51305421	0.444734	LOC108636831
1	51205564	51305564	0.444734	LOC108636831
1	51205635	51305635	0.444734	LOC108636831
1	51205671	51305671	0.444734	LOC108636831
1	51206069	51306069	0.451761	LOC108636831
1	51206321	51306321	0.455591	LOC108636831
1	51206364	51306364	0.461744	LOC108636831
1	51206608	51306608	0.467431	LOC108636831
1	51206742	51306742	0.461865	LOC108636831
1	51206770	51306770	0.460863	LOC108636831
1	51206970	51306970	0.393802	LOC108636831
1	51207040	51307040	0.548513	LOC108636831
1	51207406	51307406	0.532589	LOC108636831
1	51207431	51307431	0.527386	LOC108636831
1	51207511	51307511	0.52803	LOC108636831
1	51207625	51307625	0.523587	LOC108636831
1	51207972	51307972	0.52576	LOC108636831
1	51209139	51309139	0.523197	LOC108636831
1	51209356	51309356	0.53131	LOC108636831
1	51209630	51309630	0.534329	LOC108636831
1	51209946	51309946	0.520375	LOC108636831
1	51209975	51309975	0.523631	LOC108636831
1	51210055	51310055	0.518342	LOC108636831
1	51210194	51310194	0.520537	LOC108636831
1	51210392	51310392	0.519036	LOC108636831

1	51210678	51310678	0.515981	LOC108636831
1	51210888	51310888	0.530913	LOC108636831
1	51210970	51310970	0.514153	LOC108636831
1	51211186	51311186	0.513659	LOC108636831
1	51211392	51311392	0.512535	LOC108636831
1	51211942	51311942	0.512132	LOC108636831
1	51212043	51312043	0.512553	LOC108636831
1	51212165	51312165	0.513213	LOC108636831
1	51212349	51312349	0.517196	LOC108636831
1	51212350	51312350	0.517196	LOC108636831
1	51212549	51312549	0.519629	LOC108636831
1	51212669	51312669	0.542991	LOC108636831
1	51212716	51312716	0.544068	LOC108636831
1	51212736	51312736	0.544188	LOC108636831
1	51212739	51312739	0.544124	LOC108636831
1	51212775	51312775	0.54368	LOC108636831
1	51212804	51312804	0.552089	LOC108636831
1	51212805	51312805	0.536617	LOC108636831
1	51212988	51312988	0.525367	LOC108636831
1	51213172	51313172	0.527775	LOC108636831
1	51213265	51313265	0.517116	LOC108636831
1	51213416	51313416	0.516748	LOC108636831
1	51213496	51313496	0.516748	LOC108636831
1	51213523	51313523	0.516748	LOC108636831
1	51213693	51313693	0.515934	LOC108636831
1	51213759	51313759	0.515233	LOC108636831
1	51214040	51314040	0.515132	LOC108636831
1	51214085	51314085	0.517076	LOC108636831
1	51214385	51314385	0.495288	LOC108636831
1	51214861	51314861	0.49566	LOC108636831
1	51215581	51315581	0.497398	LOC108636831
1	51215975	51315975	0.494769	LOC108636831
1	51216088	51316088	0.495887	LOC108636831
1	51216090	51316090	0.495887	LOC108636831
1	51216256	51316256	0.495887	LOC108636831
1	51216267	51316267	0.496224	LOC108636831
1	51216345	51316345	0.494291	LOC108636831
1	51216638	51316638	0.487899	LOC108636831
1	51216924	51316924	0.501029	LOC108636831
1	51216927	51316927	0.505959	LOC108636831
1	51216994	51316994	0.503029	LOC108636831
1	51217047	51317047	0.51926	LOC108636831
1	51218233	51318233	0.505828	LOC108636831
1	51218234	51318234	0.513039	LOC108636831
1	51218549	51318549	0.505423	LOC108636831
1	51218556	51318556	0.51095	LOC108636831
1	51218708	51318708	0.504596	LOC108636831
1	51219199	51319199	0.506591	LOC108636831
1	51219654	51319654	0.50802	LOC108636831
1	51219840	51319840	0.52302	LOC108636831
1	51220072	51320072	0.518427	LOC108636831
1	51220320	51320320	0.522572	LOC108636831
1	51220392	51320392	0.520248	LOC108636831
1	51220453	51320453	0.521478	LOC108636831
1	51220642	51320642	0.515876	LOC108636831
1	51220707	51320707	0.498352	LOC108636831
1	51221196	51321196	0.530435	LOC108636831
1	51221892	51321892	0.498078	LOC108636831

1	51222894	51322894	0.502434	LOC108636831
1	51223391	51323391	0.502434	LOC108636831
1	51223392	51323392	0.501581	LOC108636831
1	51223793	51323793	0.503411	LOC108636831
1	51224448	51324448	0.503019	LOC108636831
1	51224461	51324461	0.51376	LOC108636831
1	51224494	51324494	0.49959	LOC108636831
1	51224504	51324504	0.513625	LOC108636831
1	51224813	51324813	0.514311	LOC108636831
1	51225145	51325145	0.513107	LOC108636831
1	51225270	51325270	0.513771	LOC108636831
1	51225634	51325634	0.54004	LOC108636831
1	51225802	51325802	0.536325	LOC108636831
1	51226170	51326170	0.540127	LOC108636831
1	51226174	51326174	0.556359	LOC108636831
1	51226229	51326229	0.552047	LOC108636831
1	51226235	51326235	0.552047	LOC108636831
1	51226258	51326258	0.549599	LOC108636831
1	51226548	51326548	0.55037	LOC108636831
1	51226782	51326782	0.541902	LOC108636831
1	51227334	51327334	0.544839	LOC108636831
1	51227664	51327664	0.544839	LOC108636831
1	51227739	51327739	0.555273	LOC108636831
1	51227745	51327745	0.548841	LOC108636831
1	51228031	51328031	0.546045	LOC108636831
1	51228059	51328059	0.546387	LOC108636831
1	51228866	51328866	0.546387	LOC108636831
1	51229232	51329232	0.554886	LOC108636831
1	51229301	51329301	0.556804	LOC108636831
1	51229455	51329455	0.563327	LOC108636831
1	51229536	51329536	0.563127	LOC108636831
1	51229728	51329728	0.563608	LOC108636831
1	51229741	51329741	0.562637	LOC108636831
1	51229789	51329789	0.595953	LOC108636831
1	51229819	51329819	0.583445	LOC108636831
1	51229913	51329913	0.580357	LOC108636831
1	51230027	51330027	0.572898	LOC108636831
1	51230038	51330038	0.572935	LOC108636831
1	51230091	51330091	0.570955	LOC108636831
1	51230720	51330720	0.554967	LOC108636831
1	51231083	51331083	0.559813	LOC108636831
1	51231172	51331172	0.559883	LOC108636831
1	51231393	51331393	0.559755	LOC108636831
1	51231402	51331402	0.559226	LOC108636831
1	51231420	51331420	0.559226	LOC108636831
1	51231431	51331431	0.559226	LOC108636831
1	51231641	51331641	0.559226	LOC108636831
1	51231756	51331756	0.559226	LOC108636831
1	51231890	51331890	0.559355	LOC108636831
1	51231983	51331983	0.553689	LOC108636831
1	51231992	51331992	0.553689	LOC108636831
1	51232131	51332131	0.570759	LOC108636831
1	51232139	51332139	0.566475	LOC108636831
1	51232211	51332211	0.566475	LOC108636831
1	51232275	51332275	0.566475	LOC108636831
1	51232403	51332403	0.566475	LOC108636831
1	51232589	51332589	0.566475	LOC108636831
1	51232663	51332663	0.567311	LOC108636831

1	51232893	51332893	0.568273	LOC108636831
1	51232898	51332898	0.569174	LOC108636831
1	51232946	51332946	0.56727	LOC108636831
1	51233004	51333004	0.559692	LOC108636831
1	51233128	51333128	0.58702	LOC108636831
1	51233253	51333253	0.58628	LOC108636831
1	51233571	51333571	0.578928	LOC108636831
1	51233683	51333683	0.578928	LOC108636831
1	51234050	51334050	0.57375	LOC108636831
1	51234317	51334317	0.573428	LOC108636831
1	51234435	51334435	0.573748	LOC108636831
1	51234733	51334733	0.582717	LOC108636831
1	51235089	51335089	0.5871	LOC108636831
1	51235104	51335104	0.58843	LOC108636831
1	51235120	51335120	0.589971	LOC108636831
1	51235122	51335122	0.583315	LOC108636831
1	51235156	51335156	0.58316	LOC108636831
1	51235223	51335223	0.582819	LOC108636831
1	51235511	51335511	0.583199	LOC108636831
1	51235633	51335633	0.585211	LOC108636831
1	51235920	51335920	0.584899	LOC108636831
1	51235931	51335931	0.585927	LOC108636831
1	51236065	51336065	0.586744	LOC108636831
1	51236084	51336084	0.587074	LOC108636831
1	51236105	51336105	0.591547	LOC108636831
1	51236114	51336114	0.58951	LOC108636831
1	51236190	51336190	0.586125	LOC108636831
1	51236346	51336346	0.583062	LOC108636831
1	51236360	51336360	0.585582	LOC108636831
1	51236484	51336484	0.584199	LOC108636831
1	51236679	51336679	0.586397	LOC108636831
1	51236823	51336823	0.584956	LOC108636831
1	51237349	51337349	0.594578	LOC108636831
1	51237532	51337532	0.597714	LOC108636831
1	51237722	51337722	0.594323	LOC108636831
1	51237767	51337767	0.598662	LOC108636831
1	51238026	51338026	0.599239	LOC108636831
1	51238151	51338151	0.599349	LOC108636831
1	51238186	51338186	0.597447	LOC108636831
1	51238363	51338363	0.601167	LOC108636831
1	51238523	51338523	0.621422	LOC108636831
1	51238665	51338665	0.62053	LOC108636831
1	51238680	51338680	0.618164	LOC108636831
1	51238898	51338898	0.620082	LOC108636831
1	51239053	51339053	0.617318	LOC108636831
1	51239246	51339246	0.617278	LOC108636831
1	51239247	51339247	0.559299	LOC108636831
1	51239842	51339842	0.565549	LOC108636831
1	51240048	51340048	0.565549	LOC108636831
1	51240412	51340412	0.571707	LOC108636831
1	51240467	51340467	0.559881	LOC108636831
1	51240581	51340581	0.559881	LOC108636831
1	51241258	51341258	0.560039	LOC108636831
1	51241328	51341328	0.559383	LOC108636831
1	51241508	51341508	0.559383	LOC108636831
1	51241533	51341533	0.56252	LOC108636831
1	51242020	51342020	0.560498	LOC108636831
1	51242022	51342022	0.57378	LOC108636831

1	51242133	51342133	0.574025	LOC108636831
1	51242159	51342159	0.582793	LOC108636831
1	51242160	51342160	0.582793	LOC108636831
1	51242896	51342896	0.558803	LOC108636831
1	51242963	51342963	0.565541	LOC108636831
1	51243240	51343240	0.566185	LOC108636831
1	51243247	51343247	0.566185	LOC108636831
1	51243305	51343305	0.566185	LOC108636831
1	51243308	51343308	0.566185	LOC108636831
1	51243439	51343439	0.566185	LOC108636831
1	51243494	51343494	0.566058	LOC108636831
1	51243495	51343495	0.566058	LOC108636831
1	51243500	51343500	0.566058	LOC108636831
1	51243506	51343506	0.566058	LOC108636831
1	51243827	51343827	0.579143	LOC108636831
1	51243830	51343830	0.591358	LOC108636831
1	51243974	51343974	0.591558	LOC108636831
1	51244252	51344252	0.591558	LOC108636831
1	51244253	51344253	0.591558	LOC108636831
1	51244360	51344360	0.595075	LOC108636831
1	51244412	51344412	0.592252	LOC108636831
1	51244550	51344550	0.591806	LOC108636831
1	51244590	51344590	0.592103	LOC108636831
1	51244728	51344728	0.596318	LOC108636831
1	51244773	51344773	0.599849	LOC108636831
1	51244885	51344885	0.599631	LOC108636831
1	51245256	51345256	0.600186	LOC108636831
1	51245306	51345306	0.600653	LOC108636831
1	51245333	51345333	0.597672	LOC108636831
1	51245427	51345427	0.597672	LOC108636831
1	51245570	51345570	0.597672	LOC108636831
1	51245603	51345603	0.601168	LOC108636831
1	51245669	51345669	0.60025	LOC108636831
1	51245837	51345837	0.58198	LOC108636831
1	51245936	51345936	0.58198	LOC108636831
1	51246066	51346066	0.57929	LOC108636831
1	51246619	51346619	0.577217	LOC108636831
1	51246715	51346715	0.579052	LOC108636831
1	51246743	51346743	0.582661	LOC108636831
1	51246770	51346770	0.578729	LOC108636831
1	51247192	51347192	0.582481	LOC108636831
1	51247277	51347277	0.581245	LOC108636831
1	51247278	51347278	0.580999	LOC108636831
1	51247825	51347825	0.576424	LOC108636831
1	51247826	51347826	0.576424	LOC108636831
1	51248254	51348254	0.581098	LOC108636831
1	51248326	51348326	0.575832	LOC108636831
1	51248587	51348587	0.558465	LOC108636831
1	51249934	51349934	0.56015	LOC108636831
1	51249992	51349992	0.56015	LOC108636831
1	51250057	51350057	0.577783	LOC108636831
1	51250639	51350639	0.57662	LOC108636831
1	51250878	51350878	0.590046	LOC108636831
1	51251084	51351084	0.587127	LOC108636831
1	51251461	51351461	0.587348	LOC108636831
1	51251933	51351933	0.592321	LOC108636831
1	51252024	51352024	0.595245	LOC108636831
1	51252056	51352056	0.594272	LOC108636831

1	51252176	51352176	0.591943	LOC108636831
1	51252585	51352585	0.589012	LOC108636831
1	51252636	51352636	0.589112	LOC108636831
1	51252752	51352752	0.589112	LOC108636831
1	51252911	51352911	0.589838	LOC108636831
1	51253059	51353059	0.588139	LOC108636831
1	51253538	51353538	0.59027	LOC108636831
1	51253670	51353670	0.590555	LOC108636831
1	51253797	51353797	0.593792	LOC108636831
1	51253861	51353861	0.594012	LOC108636831
1	51254097	51354097	0.594297	LOC108636831
1	51254215	51354215	0.593423	LOC108636831
1	51254399	51354399	0.595146	LOC108636831
1	51254593	51354593	0.596158	LOC108636831
1	51254728	51354728	0.597978	LOC108636831
1	51254907	51354907	0.59535	LOC108636831
1	51255151	51355151	0.606211	LOC108636831
1	51255171	51355171	0.590968	LOC108636831
1	51255584	51355584	0.592234	LOC108636831
1	51255725	51355725	0.589019	LOC108636831
1	51255959	51355959	0.595031	LOC108636831
1	51256494	51356494	0.595031	LOC108636831
1	51256531	51356531	0.595439	LOC108636831
1	51256532	51356532	0.585099	LOC108636831
1	51256755	51356755	0.585099	LOC108636831
1	51256815	51356815	0.600982	LOC108636831
1	51256894	51356894	0.602687	LOC108636831
1	51256937	51356937	0.605987	LOC108636831
1	51257078	51357078	0.600798	LOC108636831
1	51257170	51357170	0.618926	LOC108636831
1	51257177	51357177	0.611013	LOC108636831
1	51257796	51357796	0.601465	LOC108636831
1	51257799	51357799	0.601465	LOC108636831
1	51257884	51357884	0.609225	LOC108636831
1	51258053	51358053	0.612479	LOC108636831
1	51258175	51358175	0.610665	LOC108636831
1	51258179	51358179	0.607722	LOC108636831
1	51258237	51358237	0.616597	LOC108636831
1	51258854	51358854	0.612248	LOC108636831
1	51258990	51358990	0.606152	LOC108636831
1	51259150	51359150	0.608433	LOC108636831
1	51259626	51359626	0.598861	LOC108636831
1	51259894	51359894	0.604917	LOC108636831
1	51260029	51360029	0.607354	LOC108636831
1	51260182	51360182	0.6067	LOC108636831
1	51260402	51360402	0.60779	LOC108636831
1	51260706	51360706	0.604331	LOC108636831
1	51260793	51360793	0.607605	LOC108636831
1	51260996	51360996	0.611618	LOC108636831
1	51260998	51360998	0.611859	LOC108636831
1	51261109	51361109	0.608414	LOC108636831
1	51261110	51361110	0.608918	LOC108636831
1	51261226	51361226	0.607717	LOC108636831
1	51261293	51361293	0.610053	LOC108636831
1	51261682	51361682	0.60728	LOC108636831
1	51261761	51361761	0.602296	LOC108636831
1	51262168	51362168	0.603246	LOC108636831
1	51262169	51362169	0.608358	LOC108636831

1	51262470	51362470	0.609496	LOC108636831
1	51262472	51362472	0.609496	LOC108636831
1	51262483	51362483	0.604961	LOC108636831
1	51262564	51362564	0.609461	LOC108636831
1	51262592	51362592	0.604471	LOC108636831
1	51262860	51362860	0.604471	LOC108636831
1	51262942	51362942	0.595671	LOC108636831
1	51263843	51363843	0.59909	LOC108636831
1	51264094	51364094	0.59909	LOC108636831
1	51264207	51364207	0.601627	LOC108636831
1	51264477	51364477	0.608891	LOC108636831
1	51264850	51364850	0.60962	LOC108636831
1	51264853	51364853	0.610815	LOC108636831
1	51264859	51364859	0.609813	LOC108636831
1	51265221	51365221	0.610263	LOC108636831
1	51265539	51365539	0.619281	LOC108636831
1	51265582	51365582	0.616898	LOC108636831
1	51265673	51365673	0.62086	LOC108636831
1	51265700	51365700	0.621257	LOC108636831
1	51265736	51365736	0.621448	LOC108636831
1	51265774	51365774	0.621357	LOC108636831
1	51266291	51366291	0.620649	LOC108636831
1	51266360	51366360	0.613593	LOC108636831
1	51266423	51366423	0.614959	LOC108636831
1	51266459	51366459	0.613726	LOC108636831
1	51266504	51366504	0.609404	LOC108636831
1	51266609	51366609	0.608417	LOC108636831
1	51266945	51366945	0.608075	LOC108636831
1	51267082	51367082	0.606089	LOC108636831
1	51267480	51367480	0.622386	LOC108636831
1	51267615	51367615	0.615922	LOC108636831
1	51267684	51367684	0.616188	LOC108636831
1	51267706	51367706	0.620463	LOC108636831
1	51267708	51367708	0.618004	LOC108636831
1	51267993	51367993	0.614824	LOC108636831
1	51268157	51368157	0.586728	LOC108636831
1	51268307	51368307	0.596384	LOC108636831
1	51268662	51368662	0.591893	LOC108636831
1	51269068	51369068	0.594749	LOC108636831
1	51269120	51369120	0.593453	LOC108636831
1	51269517	51369517	0.639101	LOC108636831
1	51269622	51369622	0.614297	LOC108636831
1	51269996	51369996	0.617688	LOC108636831
1	51270029	51370029	0.617934	LOC108636831
1	51270249	51370249	0.619708	LOC108636831
1	51270261	51370261	0.61188	LOC108636831
1	51270445	51370445	0.611357	LOC108636831
1	51270824	51370824	0.612993	LOC108636831
1	51271199	51371199	0.61075	LOC108636831
1	51271499	51371499	0.611516	LOC108636831
1	51271787	51371787	0.614188	LOC108636831
1	51272121	51372121	0.605629	LOC108636831
1	51272587	51372587	0.6052	LOC108636831
1	51272666	51372666	0.657977	LOC108636831
1	51272668	51372668	0.654005	LOC108636831
1	51272774	51372774	0.619822	LOC108636831
1	51273449	51373449	0.623515	LOC108636831
1	51273785	51373785	0.623567	LOC108636831

1	51273850	51373850	0.624451	LOC108636831
1	51274271	51374271	0.620624	LOC108636831
1	51275225	51375225	0.620624	LOC108636831
1	51275242	51375242	0.628521	LOC108636831
1	51275309	51375309	0.634806	LOC108636831
1	51275633	51375633	0.615365	LOC108636831
1	51276164	51376164	0.610578	LOC108636831
1	51276221	51376221	0.609837	LOC108636831
1	51277200	51377200	0.626137	LOC108636831
1	51278184	51378184	0.546446	LOC108636831
1	51279236	51379236	0.572718	LOC108636831
1	51279258	51379258	0.60211	LOC108636831
1	51279283	51379283	0.592544	LOC108636831
1	51279286	51379286	0.592544	LOC108636831
1	51279314	51379314	0.596887	LOC108636831
1	51279495	51379495	0.569071	LOC108636831
1	51280066	51380066	0.572569	LOC108636831
1	51280320	51380320	0.578892	LOC108636831
1	51280570	51380570	0.574303	LOC108636831
1	51280635	51380635	0.580569	LOC108636831
1	51281061	51381061	0.580569	LOC108636831
1	51281222	51381222	0.580569	LOC108636831
1	51281520	51381520	0.582107	LOC108636831
1	51281634	51381634	0.576425	LOC108636831
1	51282147	51382147	0.580617	LOC108636831
1	51282158	51382158	0.580617	LOC108636831
1	51282191	51382191	0.57557	LOC108636831
1	51282773	51382773	0.57616	LOC108636831
1	51282989	51382989	0.57616	LOC108636831
1	51283344	51383344	0.577248	LOC108636831
1	51284152	51384152	0.573612	LOC108636831
1	51284864	51384864	0.570273	LOC108636831
1	51285120	51385120	0.573898	LOC108636831
1	51285288	51385288	0.57095	LOC108636831
1	51285610	51385610	0.607422	LOC108636831
1	51285617	51385617	0.61173	LOC108636831
1	51285704	51385704	0.598073	LOC108636831
1	51286169	51386169	0.598411	LOC108636831
1	51286672	51386672	0.561985	LOC108636831
1	51286975	51386975	0.565362	LOC108636831
1	51287079	51387079	0.5781	LOC108636831
1	51287081	51387081	0.56908	LOC108636831
1	51287170	51387170	0.593539	LOC108636831
1	51287578	51387578	0.581171	LOC108636831
1	51287876	51387876	0.599159	LOC108636831
1	51288001	51388001	0.591461	LOC108636831
1	51288165	51388165	0.601954	LOC108636831
1	51288640	51388640	0.594062	LOC108636831
1	51289258	51389258	0.594062	LOC108636831
1	51289691	51389691	0.57224	LOC108636831
1	51289749	51389749	0.57224	LOC108636831
1	51290046	51390046	0.57224	LOC108636831
1	51290282	51390282	0.574157	LOC108636831
1	51290509	51390509	0.586136	LOC108636831
1	51290596	51390596	0.631719	LOC108636831
1	51291355	51391355	0.631719	LOC108636831
1	51291364	51391364	0.644044	LOC108636831
1	51291517	51391517	0.649974	LOC108636831

1	51291595	51391595	0.66366	LOC108636831
1	51291644	51391644	0.668083	LOC108636831
1	51291668	51391668	0.668512	LOC108636831
1	51291729	51391729	0.669642	LOC108636831
1	51292046	51392046	0.660195	LOC108636831
1	51292057	51392057	0.653958	LOC108636831
1	51292183	51392183	0.654112	LOC108636831
1	51292248	51392248	0.650033	LOC108636831
1	51292377	51392377	0.646393	LOC108636831
1	51292466	51392466	0.646393	LOC108636831
1	51292639	51392639	0.646393	LOC108636831
1	51292676	51392676	0.646393	LOC108636831
1	51292735	51392735	0.646393	LOC108636831
1	51292775	51392775	0.646393	LOC108636831
1	51292887	51392887	0.649539	LOC108636831
1	51292893	51392893	0.638685	LOC108636831
1	51293070	51393070	0.638685	LOC108636831
1	51293180	51393180	0.648201	LOC108636831
1	51293223	51393223	0.648167	LOC108636831
1	51293279	51393279	0.630474	LOC108636831
1	51293424	51393424	0.617636	LOC108636831
1	51293475	51393475	0.621811	LOC108636831
1	51293536	51393536	0.627694	LOC108636831
1	51293792	51393792	0.622418	LOC108636831
1	51293810	51393810	0.629481	LOC108636831
1	51293926	51393926	0.626546	LOC108636831
1	51293985	51393985	0.626546	LOC108636831
1	51294089	51394089	0.626546	LOC108636831
1	51294091	51394091	0.629124	LOC108636831
1	51294348	51394348	0.616882	LOC108636831
1	51294410	51394410	0.616882	LOC108636831
1	51294436	51394436	0.618959	LOC108636831
1	51294603	51394603	0.616829	LOC108636831
1	51294639	51394639	0.616829	LOC108636831
1	51294746	51394746	0.616829	LOC108636831
1	51295041	51395041	0.616829	LOC108636831
1	51295141	51395141	0.616829	LOC108636831
1	51295211	51395211	0.616829	LOC108636831
1	51295242	51395242	0.626851	LOC108636831
1	51295603	51395603	0.629911	LOC108636831
1	51295648	51395648	0.628796	LOC108636831
1	51295654	51395654	0.631334	LOC108636831
1	51295687	51395687	0.631811	LOC108636831
1	51295742	51395742	0.630734	LOC108636831
1	51295833	51395833	0.633976	LOC108636831
1	51295875	51395875	0.628618	LOC108636831
1	51295996	51395996	0.628618	LOC108636831
1	51296047	51396047	0.628618	LOC108636831
1	51296052	51396052	0.631518	LOC108636831
1	51296056	51396056	0.630553	LOC108636831
1	51296092	51396092	0.630553	LOC108636831
1	51296264	51396264	0.630926	LOC108636831
1	51296306	51396306	0.631878	LOC108636831
1	51296346	51396346	0.628252	LOC108636831
1	51296349	51396349	0.628252	LOC108636831
1	51296382	51396382	0.631951	LOC108636831
1	51296406	51396406	0.616202	LOC108636831
1	51296528	51396528	0.620654	LOC108636831

1	51296595	51396595	0.472867	LOC108636831
1	51296599	51396599	0.509945	LOC108636831
1	51296606	51396606	0.509945	LOC108636831
1	51296655	51396655	0.516725	LOC108636831
1	51296684	51396684	0.516725	LOC108636831
1	51296739	51396739	0.516725	LOC108636831
1	51296746	51396746	0.521528	LOC108636831
1	51296747	51396747	0.510356	LOC108636831
1	51296799	51396799	0.493537	LOC108636831
1	51296814	51396814	0.493537	LOC108636831
1	51296861	51396861	0.50076	LOC108636831
1	51296884	51396884	0.50076	LOC108636831
1	51296907	51396907	0.50076	LOC108636831
1	51297010	51397010	0.489678	LOC108636831
1	51297255	51397255	0.496279	LOC108636831
1	51297434	51397434	0.493492	LOC108636831
1	51297508	51397508	0.49458	LOC108636831
1	51297607	51397607	0.505122	LOC108636831
1	51297614	51397614	0.505892	LOC108636831
1	51297941	51397941	0.513262	LOC108636831
1	51297962	51397962	0.511631	LOC108636831
1	51298202	51398202	0.511697	LOC108636831
1	51298403	51398403	0.507648	LOC108636831
1	51298423	51398423	0.507648	LOC108636831
1	51298651	51398651	0.519975	LOC108636831
1	51298653	51398653	0.518386	LOC108636831
1	51298763	51398763	0.519198	LOC108636831
1	51298778	51398778	0.521768	LOC108636831
1	51298948	51398948	0.510807	LOC108636831
1	51299763	51399763	0.529996	LOC108636831
1	51299815	51399815	0.519576	LOC108636831
1	51300050	51400050	0.524677	LOC108636831
1	51300362	51400362	0.51791	LOC108636831
1	51300464	51400464	0.537561	LOC108636831
1	51300508	51400508	0.548179	LOC108636831
1	51300770	51400770	0.54722	LOC108636831
1	51300797	51400797	0.552926	LOC108636831
1	51300836	51400836	0.549625	LOC108636831
1	51300837	51400837	0.549625	LOC108636831
1	51301287	51401287	0.551742	LOC108636831
1	51301384	51401384	0.558552	LOC108636831
1	51301387	51401387	0.568857	LOC108636831
1	51301406	51401406	0.567479	LOC108636831
1	51301412	51401412	0.547004	LOC108636831
1	51301505	51401505	0.535758	LOC108636831
1	51301921	51401921	0.540631	LOC108636831
1	51302036	51402036	0.542349	LOC108636831
1	51302061	51402061	0.545769	LOC108636831
1	51302085	51402085	0.542512	LOC108636831
1	51302302	51402302	0.538943	LOC108636831
1	51302389	51402389	0.542674	LOC108636831
1	51302411	51402411	0.489578	LOC108636831
1	51302625	51402625	0.495625	LOC108636831
1	51302667	51402667	0.495625	LOC108636831
1	51302736	51402736	0.495322	LOC108636831
1	51302842	51402842	0.495616	LOC108636831
1	51302946	51402946	0.493464	LOC108636831
1	51302998	51402998	0.517367	LOC108636831

1	51303356	51403356	0.508802	LOC108636831
1	51303428	51403428	0.52532	LOC108636831
1	51303456	51403456	0.526546	LOC108636831
1	51303516	51403516	0.525759	LOC108636831
1	51303641	51403641	0.522879	LOC108636831
1	51303705	51403705	0.512156	LOC108636831
1	51303823	51403823	0.517729	LOC108636831
1	51303824	51403824	0.513347	LOC108636831
1	51304439	51404439	0.51763	LOC108636831
1	51304583	51404583	0.519538	LOC108636831
1	51305057	51405057	0.515714	LOC108636831
1	51305121	51405121	0.518293	LOC108636831
1	51305340	51405340	0.516953	LOC108636831
1	51305409	51405409	0.525769	LOC108636831
1	51305431	51405431	0.523499	LOC108636831
1	51305435	51405435	0.524839	LOC108636831
1	51305583	51405583	0.527344	LOC108636831
1	51305626	51405626	0.520151	LOC108636831
1	51305679	51405679	0.505334	LOC108636831
1	51306723	51406723	0.495489	LOC108636831
1	51306791	51406791	0.504857	LOC108636831
1	51307047	51407047	0.51251	LOC108636831
1	51307085	51407085	0.51941	LOC108636831
1	51307514	51407514	0.51941	LOC108636831
1	51307571	51407571	0.513258	LOC108636831
1	51308151	51408151	0.519255	LOC108636831
1	51308255	51408255	0.529129	LOC108636831
1	51308302	51408302	0.534404	LOC108636831
1	51308348	51408348	0.536291	LOC108636831
1	51308389	51408389	0.532056	LOC108636831
1	51308622	51408622	0.531692	LOC108636831
1	51308922	51408922	0.522602	LOC108636831
1	51308927	51408927	0.533186	LOC108636831
1	51308984	51408984	0.531398	LOC108636831
1	51308988	51408988	0.521789	LOC108636831
1	51309286	51409286	0.473284	LOC108636831
1	51309302	51409302	0.474702	LOC108636831
1	51309333	51409333	0.462914	LOC108636831
1	51309522	51409522	0.454919	LOC108636831
1	51309930	51409930	0.45491	LOC108636831
1	51310139	51410139	0.42446	LOC108636831
1	51310361	51410361	0.42446	LOC108636831
1	51310522	51410522	0.425329	LOC108636831
1	51310620	51410620	0.435956	LOC108636831
1	51310647	51410647	0.430729	LOC108636831
1	51310670	51410670	0.440043	LOC108636831
1	51310921	51410921	0.440005	LOC108636831
1	51310959	51410959	0.423094	LOC108636831
1	51311040	51411040	0.418898	LOC108636831
1	51311056	51411056	0.418139	LOC108636831
1	51311089	51411089	0.418396	LOC108636831
1	51311115	51411115	0.419041	LOC108636831
1	51311167	51411167	0.378201	LOC108636831
1	51311407	51411407	0.362611	LOC108636831
1	51311479	51411479	0.364828	LOC108636831
1	51311543	51411543	0.364729	LOC108636831
1	51311729	51411729	0.36503	LOC108636831
1	51311783	51411783	0.374635	LOC108636831

1	51311825	51411825	0.375729	LOC108636831
1	51311902	51411902	0.373318	LOC108636831
1	51312004	51412004	0.370703	LOC108636831
1	51312119	51412119	0.374134	LOC108636831
1	51312185	51412185	0.375338	LOC108636831
1	51312237	51412237	0.376311	LOC108636831
1	51312243	51412243	0.375342	LOC108636831
1	51312297	51412297	0.375803	LOC108636831
1	51312299	51412299	0.371623	LOC108636831
1	51312329	51412329	0.371623	LOC108636831
1	51312355	51412355	0.370852	LOC108636831
1	51312412	51412412	0.371555	LOC108636831
1	51312570	51412570	0.37121	LOC108636831
1	51312613	51412613	0.37542	LOC108636831
1	51312743	51412743	0.387509	LOC108636831
1	51312761	51412761	0.381949	LOC108636831
1	51312788	51412788	0.381949	LOC108636831
1	51312796	51412796	0.381184	LOC108636831
1	51312863	51412863	0.379888	LOC108636831
1	51312871	51412871	0.379936	LOC108636831
1	51312895	51412895	0.381738	LOC108636831
1	51312927	51412927	0.381738	LOC108636831
1	51312930	51412930	0.381522	LOC108636831
1	51313149	51413149	0.379993	LOC108636831
1	51313178	51413178	0.382591	LOC108636831
1	51313195	51413195	0.383244	LOC108636831
1	51313208	51413208	0.382634	LOC108636831
1	51313226	51413226	0.38116	LOC108636831
1	51313329	51413329	0.382357	LOC108636831
1	51313345	51413345	0.38116	LOC108636831
1	51313352	51413352	0.382338	LOC108636831
1	51313376	51413376	0.383174	LOC108636831
1	51313405	51413405	0.38292	LOC108636831
1	51313431	51413431	0.38292	LOC108636831
1	51313495	51413495	0.382083	LOC108636831
1	51313511	51413511	0.382083	LOC108636831
1	51313512	51413512	0.381113	LOC108636831
1	51313550	51413550	0.386698	LOC108636831
1	51313562	51413562	0.38679	LOC108636831
1	51313567	51413567	0.387646	LOC108636831
1	51313599	51413599	0.387254	LOC108636831
1	51313750	51413750	0.382405	LOC108636831
1	51313771	51413771	0.382318	LOC108636831
1	51313783	51413783	0.385486	LOC108636831
1	51313816	51413816	0.384597	LOC108636831
1	51313910	51413910	0.428714	LOC108636831
1	51313964	51413964	0.399664	LOC108636831
1	51314016	51414016	0.400181	LOC108636831
1	51314084	51414084	0.400013	LOC108636831
1	51314103	51414103	0.399967	LOC108636831
1	51314226	51414226	0.403566	LOC108636831
1	51314230	51414230	0.403861	LOC108636831
1	51314244	51414244	0.405244	LOC108636831
1	51314249	51414249	0.404814	LOC108636831
1	51314256	51414256	0.404229	LOC108636831
1	51314263	51414263	0.402291	LOC108636831
1	51314264	51414264	0.402648	LOC108636831
1	51314282	51414282	0.400128	LOC108636831

1	51314533	51414533	0.400128	LOC108636831
1	51314571	51414571	0.403668	LOC108636831
1	51314572	51414572	0.403221	LOC108636831
1	51314614	51414614	0.403221	LOC108636831
1	51314616	51414616	0.346647	LOC108636831
1	51314719	51414719	0.346224	LOC108636831
1	51314769	51414769	0.358201	LOC108636831
1	51314832	51414832	0.352641	LOC108636831
1	51314839	51414839	0.349255	LOC108636831
1	51315366	51415366	0.344286	LOC108636831
1	51315592	51415592	0.369216	LOC108636831
1	51315669	51415669	0.370177	LOC108636831
1	51315678	51415678	0.370946	LOC108636831
1	51315692	51415692	0.380137	LOC108636831
1	51315697	51415697	0.37345	LOC108636831
1	51315734	51415734	0.369316	LOC108636831
1	51315770	51415770	0.368907	LOC108636831
1	51315803	51415803	0.375864	LOC108636831
1	51315844	51415844	0.376554	LOC108636831
1	51315852	51415852	0.388095	LOC108636831
1	51315856	51415856	0.376395	LOC108636831
1	51315871	51415871	0.377048	LOC108636831
1	51315923	51415923	0.377678	LOC108636831
1	51315930	51415930	0.406614	LOC108636831
1	51315965	51415965	0.396814	LOC108636831
1	51316057	51416057	0.399504	LOC108636831
1	51316164	51416164	0.384884	LOC108636831
1	51316183	51416183	0.39191	LOC108636831
1	51316235	51416235	0.389303	LOC108636831
1	51316316	51416316	0.389096	LOC108636831
1	51316318	51416318	0.394125	LOC108636831
1	51316322	51416322	0.391281	LOC108636831
1	51316345	51416345	0.38254	LOC108636831
1	51316357	51416357	0.381242	LOC108636831
1	51316412	51416412	0.383314	LOC108636831
1	51316460	51416460	0.383314	LOC108636831
1	51316516	51416516	0.383294	LOC108636831
1	51316541	51416541	0.383294	LOC108636831
1	51316553	51416553	0.383043	LOC108636831
1	51316563	51416563	0.383043	LOC108636831
1	51316615	51416615	0.376902	LOC108636831
1	51316781	51416781	0.377615	LOC108636831
1	51316804	51416804	0.370243	LOC108636831
1	51316821	51416821	0.374444	LOC108636831
1	51316832	51416832	0.373491	LOC108636831
1	51316923	51416923	0.366682	LOC108636831
1	51316943	51416943	0.36216	LOC108636831
1	51317021	51417021	0.36481	LOC108636831
1	51317069	51417069	0.36481	LOC108636831
1	51317071	51417071	0.36481	LOC108636831
1	51317197	51417197	0.367087	LOC108636831
1	51317252	51417252	0.365089	LOC108636831
1	51317369	51417369	0.378781	LOC108636831
1	51317492	51417492	0.367239	LOC108636831
1	51317516	51417516	0.365951	LOC108636831
1	51317552	51417552	0.363936	LOC108636831
1	51317669	51417669	0.365793	LOC108636831
1	51317686	51417686	0.36797	LOC108636831

1	51317689	51417689	0.369471	LOC108636831
1	51317730	51417730	0.368778	LOC108636831
1	51317808	51417808	0.37965	LOC108636831
1	51317843	51417843	0.38233	LOC108636831
1	51317907	51417907	0.365187	LOC108636831
1	51318205	51418205	0.362895	LOC108636831
1	51318232	51418232	0.360427	LOC108636831
1	51318376	51418376	0.357485	LOC108636831
1	51318420	51418420	0.357246	LOC108636831
1	51318474	51418474	0.360338	LOC108636831
1	51318563	51418563	0.360599	LOC108636831
1	51318637	51418637	0.364949	LOC108636831
1	51318689	51418689	0.360987	LOC108636831
1	51318784	51418784	0.364105	LOC108636831
1	51318786	51418786	0.363485	LOC108636831
1	51318791	51418791	0.368162	LOC108636831
1	51318811	51418811	0.366756	LOC108636831
1	51318812	51418812	0.365847	LOC108636831
1	51318818	51418818	0.358506	LOC108636831
1	51318843	51418843	0.358632	LOC108636831
1	51318909	51418909	0.342864	LOC108636831
1	51318924	51418924	0.343422	LOC108636831
1	51318962	51418962	0.380911	LOC108636831
1	51318995	51418995	0.354227	LOC108636831
1	51319091	51419091	0.353265	LOC108636831
1	51319323	51419323	0.359534	LOC108636831
1	51319383	51419383	0.351617	LOC108636831
1	51319475	51419475	0.358678	LOC108636831
1	51319506	51419506	0.351992	LOC108636831
1	51319693	51419693	0.35796	LOC108636831
1	51319878	51419878	0.353934	LOC108636831
1	51319890	51419890	0.361723	LOC108636831
1	51319891	51419891	0.355594	LOC108636831
1	51319898	51419898	0.349342	LOC108636831
1	51319899	51419899	0.358147	LOC108636831
1	51320100	51420100	0.342737	LOC108636831
1	51320123	51420123	0.344558	LOC108636831
1	51320198	51420198	0.342957	LOC108636831
1	51320251	51420251	0.342957	LOC108636831
1	51320279	51420279	0.342957	LOC108636831
1	51320309	51420309	0.344651	LOC108636831
1	51321704	51421704	0.343866	LOC108636831
1	51321917	51421917	0.347082	LOC108636831
1	51322186	51422186	0.353861	LOC108636831
1	51322329	51422329	0.358855	LOC108636831
1	51322355	51422355	0.355804	LOC108636831
1	51322491	51422491	0.357727	LOC108636831
1	51322674	51422674	0.351963	LOC108636831
1	51322735	51422735	0.342431	LOC108636831
1	51327106	51427106	0.356603	LOC108636831
1	51327289	51427289	0.35744	LOC108636831
1	51327341	51427341	0.348812	LOC108636831
1	51327400	51427400	0.358	LOC108636831
1	51327447	51427447	0.357928	LOC108636831
1	51327494	51427494	0.362675	LOC108636831
1	51327495	51427495	0.366502	LOC108636831
1	51327517	51427517	0.370847	LOC108636831
1	51327534	51427534	0.37032	LOC108636831

1	51327542	51427542	0.374296	LOC108636831
1	51327584	51427584	0.373696	LOC108636831
1	51327612	51427612	0.374681	LOC108636831
1	51327619	51427619	0.374681	LOC108636831
1	51327660	51427660	0.37305	LOC108636831
1	51327679	51427679	0.374154	LOC108636831
1	51327698	51427698	0.375023	LOC108636831
1	51327709	51427709	0.375023	LOC108636831
1	51327773	51427773	0.375023	LOC108636831
1	51327796	51427796	0.375023	LOC108636831
1	51327813	51427813	0.374414	LOC108636831
1	51327820	51427820	0.374945	LOC108636831
1	51327837	51427837	0.374732	LOC108636831
1	51327855	51427855	0.374732	LOC108636831
1	51327857	51427857	0.375319	LOC108636831
1	51327873	51427873	0.37271	LOC108636831
1	51327877	51427877	0.373383	LOC108636831
1	51327979	51427979	0.377487	LOC108636831
1	51327988	51427988	0.374648	LOC108636831
1	51328029	51428029	0.375238	LOC108636831
1	51328102	51428102	0.375722	LOC108636831
1	51328109	51428109	0.356248	LOC108636831
1	51328144	51428144	0.358927	LOC108636831
1	51328203	51428203	0.361665	LOC108636831
1	51328215	51428215	0.361665	LOC108636831
1	51328253	51428253	0.365253	LOC108636831
1	51328374	51428374	0.361352	LOC108636831
1	51328421	51428421	0.361892	LOC108636831
1	51328529	51428529	0.351579	LOC108636831
1	51328537	51428537	0.350096	LOC108636831
1	51328544	51428544	0.351978	LOC108636831
1	51328602	51428602	0.369218	LOC108636831
1	51328632	51428632	0.365688	LOC108636831
1	51328650	51428650	0.355198	LOC108636831
1	51328679	51428679	0.372277	LOC108636831
1	51328721	51428721	0.369714	LOC108636831
1	51328725	51428725	0.357297	LOC108636831
1	51328727	51428727	0.35467	LOC108636831
1	51328765	51428765	0.354657	LOC108636831
1	51328767	51428767	0.354707	LOC108636831
1	51328821	51428821	0.357197	LOC108636831
1	51328853	51428853	0.355263	LOC108636831
1	51328854	51428854	0.355263	LOC108636831
1	51328875	51428875	0.356985	LOC108636831
1	51328921	51428921	0.356985	LOC108636831
1	51328925	51428925	0.357169	LOC108636831
1	51328990	51428990	0.355291	LOC108636831
1	51329134	51429134	0.356047	LOC108636831
1	51329175	51429175	0.348459	LOC108636831
1	51329276	51429276	0.354266	LOC108636831
1	51329510	51429510	0.34423	LOC108636831
1	51329563	51429563	0.343939	LOC108636831
1	51329696	51429696	0.345226	LOC108636831
1	51329743	51429743	0.347259	LOC108636831
1	51329772	51429772	0.354757	LOC108636831
1	51329823	51429823	0.379187	LOC108636831
1	51329874	51429874	0.369986	LOC108636831
1	51330014	51430014	0.369342	LOC108636831

1	51330015	51430015	0.369342	LOC108636831
1	51330028	51430028	0.375868	LOC108636831
1	51330033	51430033	0.373713	LOC108636831
1	51330051	51430051	0.375452	LOC108636831
1	51330092	51430092	0.371996	LOC108636831
1	51330109	51430109	0.373505	LOC108636831
1	51330151	51430151	0.374294	LOC108636831
1	51330226	51430226	0.374015	LOC108636831
1	51330297	51430297	0.357938	LOC108636831
1	51330383	51430383	0.357938	LOC108636831
1	51330390	51430390	0.362329	LOC108636831
1	51330467	51430467	0.356805	LOC108636831
1	51330513	51430513	0.357846	LOC108636831
1	51330614	51430614	0.357846	LOC108636831
1	51330641	51430641	0.357846	LOC108636831
1	51330673	51430673	0.365222	LOC108636831
1	51330679	51430679	0.359274	LOC108636831
1	51330701	51430701	0.358455	LOC108636831
1	51330711	51430711	0.358481	LOC108636831
1	51330712	51430712	0.358317	LOC108636831
1	51330747	51430747	0.358056	LOC108636831
1	51330786	51430786	0.355493	LOC108636831
1	51330791	51430791	0.355493	LOC108636831
1	51330834	51430834	0.354423	LOC108636831
1	51330847	51430847	0.364127	LOC108636831
1	51330863	51430863	0.364067	LOC108636831
1	51330889	51430889	0.365793	LOC108636831
1	51330939	51430939	0.36329	LOC108636831
1	51330974	51430974	0.36329	LOC108636831
1	51330975	51430975	0.364344	LOC108636831
1	51330977	51430977	0.365215	LOC108636831
1	51330998	51430998	0.370308	LOC108636831
1	51331110	51431110	0.359274	LOC108636831
1	51331134	51431134	0.359274	LOC108636831
1	51331136	51431136	0.359274	LOC108636831
1	51331153	51431153	0.359274	LOC108636831
1	51331167	51431167	0.359274	LOC108636831
1	51331186	51431186	0.359274	LOC108636831
1	51331276	51431276	0.372818	LOC108636831
1	51331277	51431277	0.370865	LOC108636831
1	51331295	51431295	0.380419	LOC108636831
1	51331298	51431298	0.369968	LOC108636831
1	51331383	51431383	0.442473	LOC108636831
1	51331384	51431384	0.438441	LOC108636831
1	51331420	51431420	0.441411	LOC108636831
1	51331499	51431499	0.441552	LOC108636831
1	51331507	51431507	0.467842	LOC108636831
1	51331512	51431512	0.428941	LOC108636831
1	51331679	51431679	0.434221	LOC108636831
1	51331687	51431687	0.439105	LOC108636831
1	51331723	51431723	0.409824	LOC108636831
1	51332043	51432043	0.407517	LOC108636831
1	51332327	51432327	0.408811	LOC108636831
1	51332362	51432362	0.419026	LOC108636831
1	51332396	51432396	0.412125	LOC108636831
1	51332464	51432464	0.412071	LOC108636831
1	51332561	51432561	0.413263	LOC108636831
1	51332584	51432584	0.410168	LOC108636831

1	51332648	51432648	0.426059	LOC108636831
1	51332669	51432669	0.393517	LOC108636831
1	51332848	51432848	0.392319	LOC108636831
1	51333576	51433576	0.397802	LOC108636831
1	51333800	51433800	0.38157	LOC108636831
1	51334335	51434335	0.37925	LOC108636831
1	51334446	51434446	0.37925	LOC108636831
1	51334542	51434542	0.386885	LOC108636831
1	51334723	51434723	0.38291	LOC108636831
1	51334793	51434793	0.386264	LOC108636831
1	51334795	51434795	0.380826	LOC108636831
1	51334833	51434833	0.380899	LOC108636831
1	51335167	51435167	0.373737	LOC108636831
1	51335255	51435255	0.374763	LOC108636831
1	51335629	51435629	0.398427	LOC108636831
1	51336132	51436132	0.366532	LOC108636831
1	51336762	51436762	0.422257	LOC108636831
1	51336993	51436993	0.422171	LOC108636831
1	51337001	51437001	0.426009	LOC108636831
1	51337016	51437016	0.416138	LOC108636831
1	51337199	51437199	0.416062	LOC108636831
1	51337226	51437226	0.427313	LOC108636831
1	51337245	51437245	0.444347	LOC108636831
1	51337268	51437268	0.442088	LOC108636831
1	51337382	51437382	0.42978	LOC108636831
1	51337488	51437488	0.437456	LOC108636831
1	51337580	51437580	0.376056	LOC108636831
1	51337589	51437589	0.55841	LOC108636831
1	51337962	51437962	0.510364	LOC108636831
1	51338103	51438103	0.5108	LOC108636831
1	51338323	51438323	0.505253	LOC108636831
1	51338625	51438625	0.515098	LOC108636831
1	51339058	51439058	0.503274	LOC108636831
1	51339164	51439164	0.534044	LOC108636831
1	51339165	51439165	0.553528	LOC108636831
1	51339336	51439336	0.553175	LOC108636831
1	51339721	51439721	0.548804	LOC108636831
1	51339756	51439756	0.549458	LOC108636831
1	51339813	51439813	0.548842	LOC108636831
1	51339904	51439904	0.540704	LOC108636831
1	51340003	51440003	0.592924	LOC108636831
1	51340065	51440065	0.586812	LOC108636831
1	51340178	51440178	0.581963	LOC108636831
1	51340311	51440311	0.600032	LOC108636831
1	51340421	51440421	0.595783	LOC108636831
1	51341425	51441425	0.574801	LOC108636831
1	51341510	51441510	0.587818	LOC108636831
1	51341596	51441596	0.544026	LOC108636831
1	51341676	51441676	0.545626	LOC108636831
1	51341861	51441861	0.539986	LOC108636831
1	51341996	51441996	0.531443	LOC108636831
1	51342769	51442769	0.553938	LOC108636831
1	51342775	51442775	0.553938	LOC108636831
1	51342784	51442784	0.55535	LOC108636831
1	51342827	51442827	0.570452	LOC108636831
1	51342897	51442897	0.566451	LOC108636831
1	51342899	51442899	0.574821	LOC108636831
1	51342926	51442926	0.573719	LOC108636831

1	51343009	51443009	0.573719	LOC108636831
1	51343021	51443021	0.576704	LOC108636831
1	51343037	51443037	0.575831	LOC108636831
1	51343091	51443091	0.575074	LOC108636831
1	51343230	51443230	0.577576	LOC108636831
1	51343258	51443258	0.575358	LOC108636831
1	51343259	51443259	0.575504	LOC108636831
1	51343309	51443309	0.573218	LOC108636831
1	51343450	51443450	0.583206	LOC108636831
1	51343456	51443456	0.566788	LOC108636831
1	51343607	51443607	0.567918	LOC108636831
1	51343716	51443716	0.567593	LOC108636831
1	51343726	51443726	0.56708	LOC108636831
1	51343798	51443798	0.569584	LOC108636831
1	51343817	51443817	0.563823	LOC108636831
1	51343829	51443829	0.567005	LOC108636831
1	51344002	51444002	0.564918	LOC108636831
1	51344064	51444064	0.564646	LOC108636831
1	51344097	51444097	0.561564	LOC108636831
1	51344407	51444407	0.561677	LOC108636831
1	51344651	51444651	0.554412	LOC108636831
1	51344677	51444677	0.55834	LOC108636831
1	51344678	51444678	0.55834	LOC108636831
1	51344717	51444717	0.556858	LOC108636831
1	51344856	51444856	0.556068	LOC108636831
1	51345049	51445049	0.550501	LOC108636831
1	51345141	51445141	0.550501	LOC108636831
1	51345258	51445258	0.534922	LOC108636831
1	51345348	51445348	0.538552	LOC108636831
1	51345350	51445350	0.5154	LOC108636831
1	51346607	51446607	0.520877	LOC108636831
1	51346854	51446854	0.53931	LOC108636831
1	51346874	51446874	0.55349	LOC108636831
1	51346914	51446914	0.570638	LOC108636831
1	51346965	51446965	0.559442	LOC108636831
1	51346966	51446966	0.560226	LOC108636831
1	51346999	51446999	0.565563	LOC108636831
1	51347162	51447162	0.547378	LOC108636831
1	51347191	51447191	0.544811	LOC108636831
1	51347192	51447192	0.547509	LOC108636831
1	51347259	51447259	0.544732	LOC108636831
1	51347336	51447336	0.535075	LOC108636831
1	51347483	51447483	0.535536	LOC108636831
1	51347576	51447576	0.535347	LOC108636831
1	51347691	51447691	0.52691	LOC108636831
1	51347983	51447983	0.530404	LOC108636831
1	51348089	51448089	0.537454	LOC108636831
1	51348118	51448118	0.534489	LOC108636831
1	51348201	51448201	0.534489	LOC108636831
1	51348307	51448307	0.536237	LOC108636831
1	51348360	51448360	0.528466	LOC108636831
1	51348368	51448368	0.528466	LOC108636831
1	51348389	51448389	0.528466	LOC108636831
1	51348422	51448422	0.528466	LOC108636831
1	51348517	51448517	0.528466	LOC108636831
1	51348560	51448560	0.528466	LOC108636831
1	51348561	51448561	0.532051	LOC108636831
1	51348706	51448706	0.519592	LOC108636831

1	51348720	51448720	0.519592	LOC108636831
1	51348824	51448824	0.519423	LOC108636831
1	51348841	51448841	0.519514	LOC108636831
1	51349056	51449056	0.530336	LOC108636831
1	51349126	51449126	0.526059	LOC108636831
1	51349228	51449228	0.524413	LOC108636831
1	51349368	51449368	0.52586	LOC108636831
1	51349703	51449703	0.52892	LOC108636831
1	51349778	51449778	0.548726	LOC108636831
1	51349786	51449786	0.543122	LOC108636831
1	51349854	51449854	0.542093	LOC108636831
1	51349858	51449858	0.542561	LOC108636831
1	51349926	51449926	0.54204	LOC108636831
1	51349967	51449967	0.542599	LOC108636831
1	51350226	51450226	0.535488	LOC108636831
1	51350493	51450493	0.544766	LOC108636831
1	51350560	51450560	0.553389	LOC108636831
1	51350592	51450592	0.564997	LOC108636831
1	51350712	51450712	0.564997	LOC108636831
1	51350763	51450763	0.557701	LOC108636831
1	51350799	51450799	0.56057	LOC108636831
1	51350802	51450802	0.56164	LOC108636831
1	51350981	51450981	0.54664	LOC108636831
1	51351121	51451121	0.54664	LOC108636831
1	51351269	51451269	0.545562	LOC108636831
1	51351283	51451283	0.546798	LOC108636831
1	51351301	51451301	0.546798	LOC108636831
1	51351349	51451349	0.546798	LOC108636831
1	51351352	51451352	0.546798	LOC108636831
1	51351432	51451432	0.546798	LOC108636831
1	51351592	51451592	0.557802	LOC108636831
1	51351688	51451688	0.558419	LOC108636831
1	51351779	51451779	0.55455	LOC108636831
1	51351809	51451809	0.55455	LOC108636831
1	51351847	51451847	0.55455	LOC108636831
1	51351934	51451934	0.554002	LOC108636831
1	51351948	51451948	0.554925	LOC108636831
1	51352088	51452088	0.556751	LOC108636831
1	51352122	51452122	0.564955	LOC108636831
1	51352166	51452166	0.556948	LOC108636831
1	51352269	51452269	0.559281	LOC108636831
1	51352275	51452275	0.559281	LOC108636831
1	51352295	51452295	0.554669	LOC108636831
1	51352316	51452316	0.554669	LOC108636831
1	51352321	51452321	0.554669	LOC108636831
1	51352379	51452379	0.575968	LOC108636831
1	51352389	51452389	0.572151	LOC108636831
1	51352398	51452398	0.572151	LOC108636831
1	51352427	51452427	0.569674	LOC108636831
1	51352499	51452499	0.57047	LOC108636831
1	51352602	51452602	0.568996	LOC108636831
1	51352630	51452630	0.569153	LOC108636831
1	51352769	51452769	0.575113	LOC108636831
1	51352781	51452781	0.576092	LOC108636831
1	51352787	51452787	0.576092	LOC108636831
1	51352814	51452814	0.576092	LOC108636831
1	51352844	51452844	0.571088	LOC108636831
1	51352908	51452908	0.571088	LOC108636831

1	51352982	51452982	0.581723	LOC108636831
1	51353028	51453028	0.585248	LOC108636831
1	51353078	51453078	0.575107	LOC108636831
1	51353089	51453089	0.584179	LOC108636831
1	51353163	51453163	0.583853	LOC108636831
1	51353174	51453174	0.579275	LOC108636831
1	51353297	51453297	0.57481	LOC108636831
1	51353369	51453369	0.570465	LOC108636831
1	51353460	51453460	0.57825	LOC108636831
1	51353524	51453524	0.577852	LOC108636831
1	51353576	51453576	0.577852	LOC108636831
1	51353689	51453689	0.581569	LOC108636831
1	51353799	51453799	0.581329	LOC108636831
1	51353805	51453805	0.58511	LOC108636831
1	51353837	51453837	0.576791	LOC108636831
1	51353842	51453842	0.589862	LOC108636831
1	51353844	51453844	0.586593	LOC108636831
1	51354020	51454020	0.587069	LOC108636831
1	51354034	51454034	0.588089	LOC108636831
1	51354075	51454075	0.553189	LOC108636831
1	51354081	51454081	0.554857	LOC108636831
1	51354104	51454104	0.554857	LOC108636831
1	51354134	51454134	0.565039	LOC108636831
1	51354139	51454139	0.58118	LOC108636831
1	51354143	51454143	0.567899	LOC108636831
1	51354172	51454172	0.565215	LOC108636831
1	51354283	51454283	0.561943	LOC108636831
1	51354372	51454372	0.555922	LOC108636831
1	51354405	51454405	0.56001	LOC108636831
1	51354447	51454447	0.553943	LOC108636831
1	51354489	51454489	0.573897	LOC108636831
1	51354526	51454526	0.574912	LOC108636831
1	51354565	51454565	0.58506	LOC108636831
1	51354576	51454576	0.58506	LOC108636831
1	51354715	51454715	0.574654	LOC108636831
1	51354842	51454842	0.569749	LOC108636831
1	51354911	51454911	0.575438	LOC108636831
1	51354932	51454932	0.569093	LOC108636831
1	51354977	51454977	0.564407	LOC108636831
1	51355427	51455427	0.517252	LOC108636831
1	51355889	51455889	0.517814	LOC108636831
1	51355970	51455970	0.543831	LOC108636831
1	51356303	51456303	0.524419	LOC108636831
1	51356685	51456685	0.494972	LOC108636831
1	51357277	51457277	0.494972	LOC108636831
1	51357426	51457426	0.494138	LOC108636831
1	51357830	51457830	0.498416	LOC108636831
1	51357978	51457978	0.522343	LOC108636831
1	51358079	51458079	0.520856	LOC108636831
1	51358804	51458804	0.503169	LOC108636831
1	51358841	51458841	0.49986	LOC108636831
1	51358902	51458902	0.499833	LOC108636831
1	51359081	51459081	0.499913	LOC108636831
1	51359086	51459086	0.527489	LOC108636831
1	51359219	51459219	0.500177	LOC108636831
1	51359276	51459276	0.506921	LOC108636831
1	51359439	51459439	0.524609	LOC108636831
1	51359787	51459787	0.510971	LOC108636831

1	51359834	51459834	0.510895	LOC108636831
1	51359850	51459850	0.525756	LOC108636831
1	51360416	51460416	0.503058	LOC108636831
1	51360499	51460499	0.522384	LOC108636831
1	51360536	51460536	0.528495	LOC108636831
1	51360774	51460774	0.506917	LOC108636831
1	51361049	51461049	0.506917	LOC108636831
1	51361084	51461084	0.506917	LOC108636831
1	51361277	51461277	0.506917	LOC108636831
1	51362029	51462029	0.506917	LOC108636831
1	51362237	51462237	0.517689	LOC108636831
1	51362358	51462358	0.518662	LOC108636831
1	51362483	51462483	0.557711	LOC108636831
1	51362521	51462521	0.547447	LOC108636831
1	51362618	51462618	0.545067	LOC108636831
1	51362709	51462709	0.545842	LOC108636831
1	51362714	51462714	0.545842	LOC108636831
1	51362791	51462791	0.547551	LOC108636831
1	51362824	51462824	0.547396	LOC108636831
1	51362959	51462959	0.547429	LOC108636831
1	51363098	51463098	0.554247	LOC108636831
1	51363165	51463165	0.544478	LOC108636831
1	51363483	51463483	0.544478	LOC108636831
1	51363491	51463491	0.544478	LOC108636831
1	51363573	51463573	0.556186	LOC108636831
1	51363601	51463601	0.541338	LOC108636831
1	51363637	51463637	0.568684	LOC108636831
1	51363831	51463831	0.536061	LOC108636831
1	51364059	51464059	0.532552	LOC108636831
1	51364075	51464075	0.532552	LOC108636831
1	51364210	51464210	0.534191	LOC108636831
1	51364574	51464574	0.547552	LOC108636831
1	51364575	51464575	0.536038	LOC108636831
1	51364679	51464679	0.547627	LOC108636831
1	51364814	51464814	0.559896	LOC108636831
1	51364855	51464855	0.558484	LOC108636831
1	51364882	51464882	0.560256	LOC108636831
1	51364897	51464897	0.556797	LOC108636831
1	51365053	51465053	0.559396	LOC108636831
1	51365268	51465268	0.556984	LOC108636831
1	51365281	51465281	0.558286	LOC108636831
1	51365409	51465409	0.559457	LOC108636831
1	51365425	51465425	0.580099	LOC108636831
1	51365594	51465594	0.550796	LOC108636831
1	51365678	51465678	0.549937	LOC108636831
1	51366082	51466082	0.53995	LOC108636831
1	51366487	51466487	0.539194	LOC108636831
1	51366570	51466570	0.539194	LOC108636831
1	51366977	51466977	0.551984	LOC108636831
1	51367023	51467023	0.543586	LOC108636831
1	51367400	51467400	0.543787	LOC108636831
1	51367610	51467610	0.543787	LOC108636831
1	51367794	51467794	0.551315	LOC108636831
1	51367830	51467830	0.551014	LOC108636831
1	51368000	51468000	0.547057	LOC108636831
1	51368029	51468029	0.547057	LOC108636831
1	51368146	51468146	0.566534	LOC108636831
1	51368164	51468164	0.569452	LOC108636831

1	51368165	51468165	0.569452	LOC108636831
1	51368183	51468183	0.575634	LOC108636831
1	51368353	51468353	0.559298	LOC108636831
1	51368373	51468373	0.559298	LOC108636831
1	51368396	51468396	0.559298	LOC108636831
1	51368590	51468590	0.562021	LOC108636831
1	51368597	51468597	0.560345	LOC108636831
1	51368683	51468683	0.552929	LOC108636831
1	51368707	51468707	0.552929	LOC108636831
1	51368861	51468861	0.543472	LOC108636831
1	51369267	51469267	0.548867	LOC108636831
1	51369334	51469334	0.560467	LOC108636831
1	51369407	51469407	0.55556	LOC108636831
1	51588770	51688770	0.737719	LOC102171918
1	51588900	51688900	0.738335	LOC102171918
1	51589045	51689045	0.747032	LOC102171918
1	51589053	51689053	0.703384	LOC102171918
1	51589658	51689658	0.702448	LOC102171918
1	51589831	51689831	0.715781	LOC102171918
1	51590032	51690032	0.719283	LOC102171918
1	51590157	51690157	0.717283	LOC102171918
1	51590412	51690412	0.70205	LOC102171918
1	51590654	51690654	0.702294	LOC102171918
1	51590895	51690895	0.695852	LOC102171918
1	51591370	51691370	0.697454	LOC102171918
1	51591547	51691547	0.688275	LOC102171918
1	51591903	51691903	0.655445	LOC102171918
1	51592617	51692617	0.655445	LOC102171918
1	51593423	51693423	0.661174	LOC102171918
1	51593757	51693757	0.655203	LOC102171918
1	51594010	51694010	0.69667	LOC102171918
1	51594075	51694075	0.687842	LOC102171918
1	51594105	51694105	0.683045	LOC102171918
1	51594521	51694521	0.714623	LOC102171918
1	51594586	51694586	0.71025	LOC102171918
1	51595000	51695000	0.703825	LOC102171918
1	51595089	51695089	0.717951	LOC102171918
1	51595249	51695249	0.569902	LOC102171918
1	51595526	51695526	0.568943	LOC102171918
1	51595641	51695641	0.577554	LOC102171918
1	51596097	51696097	0.577171	LOC102171918
1	51596114	51696114	0.577882	LOC102171918
1	51596341	51696341	0.578937	LOC102171918
1	51596386	51696386	0.57441	LOC102171918
1	51596770	51696770	0.577277	LOC102171918
1	51597026	51697026	0.570502	LOC102171918
1	51597051	51697051	0.563174	LOC102171918
1	51597279	51697279	0.56576	LOC102171918
1	51597392	51697392	0.574601	LOC102171918
1	51597449	51697449	0.574601	LOC102171918
1	51597568	51697568	0.564686	LOC102171918
1	51598257	51698257	0.558238	LOC102171918
1	51598453	51698453	0.579051	LOC102171918
1	51598513	51698513	0.581111	LOC102171918
1	51598830	51698830	0.586512	LOC102171918
1	51598909	51698909	0.573687	LOC102171918
1	51598953	51698953	0.578812	LOC102171918
1	51599015	51699015	0.577515	LOC102171918

1	51599057	51699057	0.594846	LOC102171918
1	51599153	51699153	0.594846	LOC102171918
1	51599180	51699180	0.59365	LOC102171918
1	51599274	51699274	0.597753	LOC102171918
1	51599466	51699466	0.595992	LOC102171918
1	51599552	51699552	0.596312	LOC102171918
1	51599659	51699659	0.596239	LOC102171918
1	51599848	51699848	0.598782	LOC102171918
1	51599888	51699888	0.598873	LOC102171918
1	51599911	51699911	0.595269	LOC102171918
1	51600120	51700120	0.599222	LOC102171918
1	51600164	51700164	0.566352	LOC102171918
1	51600413	51700413	0.561863	LOC102171918
1	51600646	51700646	0.565588	LOC102171918
1	51600770	51700770	0.568898	LOC102171918
1	51600774	51700774	0.569315	LOC102171918
1	51600818	51700818	0.569315	LOC102171918
1	51600889	51700889	0.568426	LOC102171918
1	51600943	51700943	0.569329	LOC102171918
1	51600975	51700975	0.571481	LOC102171918
1	51601072	51701072	0.569347	LOC102171918
1	51601386	51701386	0.569654	LOC102171918
1	51601392	51701392	0.564327	LOC102171918
1	51601586	51701586	0.565031	LOC102171918
1	51601678	51701678	0.54372	LOC102171918
1	51601791	51701791	0.54372	LOC102171918
1	51601887	51701887	0.54372	LOC102171918
1	51602003	51702003	0.54372	LOC102171918
1	51602007	51702007	0.54372	LOC102171918
1	51602223	51702223	0.543642	LOC102171918
1	51602431	51702431	0.543708	LOC102171918
1	51602615	51702615	0.540966	LOC102171918
1	51602645	51702645	0.541852	LOC102171918
1	51602649	51702649	0.539174	LOC102171918
1	51603161	51703161	0.542728	LOC102171918
1	51603441	51703441	0.543074	LOC102171918
1	51603515	51703515	0.543074	LOC102171918
1	51603531	51703531	0.543074	LOC102171918
1	51603628	51703628	0.545039	LOC102171918
1	51603666	51703666	0.544936	LOC102171918
1	51603872	51703872	0.544936	LOC102171918
1	51603948	51703948	0.544929	LOC102171918
1	51604030	51704030	0.566913	LOC102171918
1	51604052	51704052	0.566578	LOC102171918
1	51604110	51704110	0.570231	LOC102171918
1	51604191	51704191	0.549695	LOC102171918
1	51604285	51704285	0.55344	LOC102171918
1	51604293	51704293	0.55344	LOC102171918
1	51604815	51704815	0.549652	LOC102171918
1	51605141	51705141	0.535525	LOC102171918
1	51605474	51705474	0.557208	LOC102171918
1	51606036	51706036	0.56996	LOC102171918
1	51606139	51706139	0.553407	LOC102171918
1	51606392	51706392	0.557545	LOC102171918
1	51606977	51706977	0.539057	LOC102171918
1	51607422	51707422	0.540788	LOC102171918
1	51607425	51707425	0.541165	LOC102171918
1	51607453	51707453	0.582827	LOC102171918

1	51607458	51707458	0.582827	LOC102171918
1	51608017	51708017	0.569691	LOC102171918
1	51608823	51708823	0.573161	LOC102171918
1	51609112	51709112	0.573161	LOC102171918
1	51609291	51709291	0.592435	LOC102171918
1	51609676	51709676	0.60105	LOC102171918
1	51610023	51710023	0.604216	LOC102171918
1	51610045	51710045	0.605361	LOC102171918
1	51610053	51710053	0.605225	LOC102171918
1	51610109	51710109	0.61264	LOC102171918
1	51610167	51710167	0.602913	LOC102171918
1	51610212	51710212	0.603829	LOC102171918
1	51610328	51710328	0.588263	LOC102171918
1	51610615	51710615	0.589268	LOC102171918
1	51610658	51710658	0.589512	LOC102171918
1	51611461	51711461	0.586172	LOC102171918
1	51611597	51711597	0.597054	LOC102171918
1	51611674	51711674	0.585877	LOC102171918
1	51612610	51712610	0.583665	LOC102171918
1	51612724	51712724	0.579674	LOC102171918
1	51612811	51712811	0.592511	LOC102171918
1	51613155	51713155	0.681482	LOC102171918
1	51613197	51713197	0.678434	LOC102171918
1	51613221	51713221	0.658146	LOC102171918
1	51613271	51713271	0.651201	LOC102171918
1	51613852	51713852	0.639642	LOC102171918
1	51614113	51714113	0.639423	LOC102171918
1	51614265	51714265	0.64351	LOC102171918
1	51614724	51714724	0.634532	LOC102171918
1	51615178	51715178	0.586108	LOC102171918
1	51615315	51715315	0.58571	LOC102171918
1	51615324	51715324	0.587048	LOC102171918
1	51615472	51715472	0.586914	LOC102171918
1	51615485	51715485	0.589147	LOC102171918
1	51616016	51716016	0.587947	LOC102171918
1	51616070	51716070	0.57832	LOC102171918
1	51616155	51716155	0.566199	LOC102171918
1	51616793	51716793	0.564197	LOC102171918
1	51617198	51717198	0.562117	LOC102171918
1	51617484	51717484	0.560451	LOC102171918
1	51617886	51717886	0.561655	LOC102171918
1	51618180	51718180	0.565448	LOC102171918
1	51618243	51718243	0.55913	LOC102171918
1	51618965	51718965	0.55585	LOC102171918
1	51619095	51719095	0.552536	LOC102171918
1	51619312	51719312	0.563638	LOC102171918
1	51620212	51720212	0.54929	LOC102171918
1	51620284	51720284	0.552962	LOC102171918
1	51620425	51720425	0.544604	LOC102171918
1	51620616	51720616	0.570241	LOC102171918
1	51621004	51721004	0.54682	LOC102171918
1	51622276	51722276	0.544024	LOC102171918
1	51622732	51722732	0.534409	LOC102171918
1	51624270	51724270	0.534409	LOC102171918
1	51624505	51724505	0.545206	LOC102171918
1	51624735	51724735	0.543369	LOC102171918
1	51624956	51724956	0.570187	LOC102171918
1	51625301	51725301	0.573214	LOC102171918

1	51625466	51725466	0.572196	LOC102171918
1	51625471	51725471	0.627181	LOC102171918
1	51625684	51725684	0.582058	LOC102171918
1	51625865	51725865	0.578727	LOC102171918
1	51626055	51726055	0.579933	LOC102171918
1	51626056	51726056	0.579622	LOC102171918
1	51626183	51726183	0.587138	LOC102171918
1	51626801	51726801	0.565356	LOC102171918
1	51627540	51727540	0.570212	LOC102171918
1	51628213	51728213	0.567069	LOC102171918
1	51628223	51728223	0.565671	LOC102171918
1	51628428	51728428	0.565671	LOC102171918
1	51628917	51728917	0.563917	LOC102171918
1	51629064	51729064	0.572497	LOC102171918
1	51629260	51729260	0.574918	LOC102171918
1	51629270	51729270	0.57546	LOC102171918
1	51629507	51729507	0.573523	LOC102171918
1	51629517	51729517	0.574512	LOC102171918
1	51629647	51729647	0.537957	LOC102171918
1	51630241	51730241	0.518054	LOC102171918
1	51630597	51730597	0.522123	LOC102171918
1	51630668	51730668	0.521259	LOC102171918
1	51630737	51730737	0.520575	LOC102171918
1	51631062	51731062	0.518178	LOC102171918
1	51631381	51731381	0.518178	LOC102171918
1	51631582	51731582	0.520807	LOC102171918
1	51631829	51731829	0.516776	LOC102171918
1	51632439	51732439	0.516134	LOC102171918
1	51632468	51732468	0.516284	LOC102171918
1	51632572	51732572	0.519899	LOC102171918
1	51632609	51732609	0.52576	LOC102171918
1	51633042	51733042	0.504646	LOC102171918
1	51633085	51733085	0.505603	LOC102171918
1	51633092	51733092	0.503509	LOC102171918
1	51633443	51733443	0.50487	LOC102171918
1	51634082	51734082	0.502873	LOC102171918
1	51634776	51734776	0.502873	LOC102171918
1	51635048	51735048	0.502993	LOC102171918
1	51635642	51735642	0.503022	LOC102171918
1	51635666	51735666	0.506217	LOC102171918
1	51636216	51736216	0.53072	LOC102171918
1	51636242	51736242	0.520041	LOC102171918
1	51636667	51736667	0.520041	LOC102171918
1	51636744	51736744	0.520041	LOC102171918
1	51637039	51737039	0.519082	LOC102171918
1	51637062	51737062	0.55903	LOC102171918
1	51637620	51737620	0.502074	LOC102171918
1	51637644	51737644	0.50409	LOC102171918
1	51637899	51737899	0.501292	LOC102171918
1	51638007	51738007	0.513621	LOC102171918
1	51638038	51738038	0.513621	LOC102171918
1	51638044	51738044	0.513621	LOC102171918
1	51638274	51738274	0.494036	LOC102171918
1	51639023	51739023	0.493521	LOC102171918
1	51639586	51739586	0.49021	LOC102171918
1	51639934	51739934	0.49021	LOC102171918
1	51640379	51740379	0.50505	LOC102171918
1	51641087	51741087	0.501382	LOC102171918

1	51641363	51741363	0.516844	LOC102171918
1	51641376	51741376	0.516844	LOC102171918
1	51641550	51741550	0.563313	LOC102171918
1	51641627	51741627	0.564072	LOC102171918
1	51641635	51741635	0.564072	LOC102171918
1	51641668	51741668	0.549221	LOC102171918
1	51641731	51741731	0.547601	LOC102171918
1	51641748	51741748	0.547757	LOC102171918
1	51641827	51741827	0.545864	LOC102171918
1	51642046	51742046	0.552391	LOC102171918
1	51642128	51742128	0.531307	LOC102171918
1	51642130	51742130	0.531307	LOC102171918
1	51642273	51742273	0.538132	LOC102171918
1	51642533	51742533	0.538155	LOC102171918
1	51642625	51742625	0.524135	LOC102171918
1	51643568	51743568	0.537552	LOC102171918
1	51643692	51743692	0.531076	LOC102171918
1	51643855	51743855	0.547702	LOC102171918
1	51643941	51743941	0.547529	LOC102171918
1	51643990	51743990	0.546774	LOC102171918
1	51644172	51744172	0.544284	LOC102171918
1	51644252	51744252	0.544284	LOC102171918
1	51644263	51744263	0.544517	LOC102171918
1	51644337	51744337	0.541371	LOC102171918
1	51644355	51744355	0.541269	LOC102171918
1	51644528	51744528	0.537224	LOC102171918
1	51645121	51745121	0.539098	LOC102171918
1	51645451	51745451	0.55098	LOC102171918
1	51645618	51745618	0.55273	LOC102171918
1	51645701	51745701	0.55273	LOC102171918
1	51646058	51746058	0.546997	LOC102171918
1	51646186	51746186	0.542337	LOC102171918
1	51647163	51747163	0.533524	LOC102171918
1	51647346	51747346	0.529302	LOC102171918
1	51647623	51747623	0.531472	LOC102171918
1	51647744	51747744	0.530932	LOC102171918
1	51647778	51747778	0.531148	LOC102171918
1	51647942	51747942	0.527733	LOC102171918
1	51648010	51748010	0.537798	LOC102171918
1	51648024	51748024	0.537798	LOC102171918
1	51648299	51748299	0.537798	LOC102171918
1	51648507	51748507	0.528698	LOC102171918
1	51649556	51749556	0.505428	LOC102171918
1	51649868	51749868	0.516028	LOC102171918
1	51650111	51750111	0.529353	LOC102171918
1	51650464	51750464	0.513052	LOC102171918
1	51650578	51750578	0.50397	LOC102171918
1	51650671	51750671	0.48533	LOC102171918
1	51652138	51752138	0.484499	LOC102171918
1	51653333	51753333	0.484499	LOC102171918
1	51656282	51756282	0.487513	LOC102171918
1	51656724	51756724	0.521651	LOC102171918
1	51656725	51756725	0.521651	LOC102171918
1	51657026	51757026	0.524309	LOC102171918
1	51657096	51757096	0.53369	LOC102171918
1	51657578	51757578	0.527172	LOC102171918
1	51657615	51757615	0.528239	LOC102171918
1	51657716	51757716	0.475085	LOC102171918

1	51658018	51758018	0.494302	LOC102171918
1	51658805	51758805	0.49103	LOC102171918
1	51658842	51758842	0.525593	LOC102171918
1	51659188	51759188	0.519529	LOC102171918
1	51659377	51759377	0.510674	LOC102171918
1	51659595	51759595	0.497848	LOC102171918
1	51659969	51759969	0.517283	LOC102171918
1	51660006	51760006	0.517099	LOC102171918
1	51660546	51760546	0.494821	LOC102171918
1	51661336	51761336	0.485258	LOC102171918
1	51661337	51761337	0.487287	LOC102171918
1	51661492	51761492	0.475876	LOC102171918
1	51662295	51762295	0.475172	LOC102171918
1	51662496	51762496	0.475172	LOC102171918
1	51662504	51762504	0.475172	LOC102171918
1	51662967	51762967	0.475172	LOC102171918
1	51663055	51763055	0.483135	LOC102171918
1	51663319	51763319	0.478353	LOC102171918
1	51663507	51763507	0.477113	LOC102171918
1	51663778	51763778	0.48191	LOC102171918
1	51664638	51764638	0.48191	LOC102171918
1	51664641	51764641	0.497525	LOC102171918
1	51664859	51764859	0.496726	LOC102171918
1	51664919	51764919	0.496746	LOC102171918
1	51664944	51764944	0.518246	LOC102171918
1	51664946	51764946	0.515439	LOC102171918
1	51665083	51765083	0.515439	LOC102171918
1	51665113	51765113	0.52071	LOC102171918
1	51665371	51765371	0.522932	LOC102171918
1	51665500	51765500	0.539258	LOC102171918
1	51665518	51765518	0.539258	LOC102171918
1	51665669	51765669	0.537334	LOC102171918
1	51665709	51765709	0.533053	LOC102171918
1	51666027	51766027	0.531395	LOC102171918
1	51666651	51766651	0.530376	LOC102171918
1	51666684	51766684	0.543688	LOC102171918
1	51666819	51766819	0.536352	LOC102171918
1	51667102	51767102	0.536871	LOC102171918
1	51667213	51767213	0.535288	LOC102171918
1	51667978	51767978	0.55623	LOC102171918
1	51668566	51768566	0.563558	LOC102171918
1	51669018	51769018	0.559286	LOC102171918
1	51669028	51769028	0.565854	LOC102171918
1	51669141	51769141	0.582272	LOC102171918
1	51669289	51769289	0.578793	LOC102171918
1	51669403	51769403	0.577361	LOC102171918
1	51669510	51769510	0.575978	LOC102171918
1	51669541	51769541	0.594622	LOC102171918
1	51670073	51770073	0.588817	LOC102171918
1	51670321	51770321	0.58837	LOC102171918
1	51670460	51770460	0.589675	LOC102171918
1	51670462	51770462	0.582813	LOC102171918
1	51671136	51771136	0.576018	LOC102171918
1	51671231	51771231	0.581184	LOC102171918
1	51671529	51771529	0.590979	LOC102171918
1	51671671	51771671	0.593521	LOC102171918
1	51671717	51771717	0.620364	LOC102171918
1	51671749	51771749	0.610045	LOC102171918

1	51671822	51771822	0.60851	LOC102171918
1	51671924	51771924	0.606578	LOC102171918
1	51672140	51772140	0.607461	LOC102171918
1	51672271	51772271	0.603269	LOC102171918
1	51672442	51772442	0.601725	LOC102171918
1	51672692	51772692	0.598006	LOC102171918
1	51672737	51772737	0.585543	LOC102171918
1	51672808	51772808	0.584291	LOC102171918
1	51673862	51773862	0.582858	LOC102171918
1	51673924	51773924	0.586718	LOC102171918
1	51674332	51774332	0.58223	LOC102171918
1	51674451	51774451	0.582118	LOC102171918
1	51674787	51774787	0.582118	LOC102171918
1	51674801	51774801	0.582118	LOC102171918
1	51675093	51775093	0.581866	LOC102171918
1	51675585	51775585	0.572069	LOC102171918
1	51675769	51775769	0.574055	LOC102171918
1	51675850	51775850	0.552693	LOC102171918
1	51675948	51775948	0.556683	LOC102171918
1	51676453	51776453	0.570342	LOC102171918
1	51677158	51777158	0.563523	LOC102171918
1	51677546	51777546	0.560124	LOC102171918
1	51677958	51777958	0.547591	LOC102171918
1	51678234	51778234	0.549695	LOC102171918
1	51678759	51778759	0.544283	LOC102171918
1	51679192	51779192	0.548969	LOC102171918
1	51679289	51779289	0.555091	LOC102171918
1	51679400	51779400	0.55322	LOC102171918
1	51679501	51779501	0.544256	LOC102171918
1	51679978	51779978	0.53997	LOC102171918
1	51680180	51780180	0.387476	LOC102171918
1	51680193	51780193	0.383404	LOC102171918
1	51680980	51780980	0.383333	LOC102171918
1	51681359	51781359	0.383783	LOC102171918
1	51681561	51781561	0.383783	LOC102171918
1	51681595	51781595	0.390003	LOC102171918
1	51681947	51781947	0.389112	LOC102171918
1	51682220	51782220	0.410687	LOC102171918
1	51682534	51782534	0.408058	LOC102171918
1	51682544	51782544	0.407589	LOC102171918
1	51682591	51782591	0.412562	LOC102171918
1	51682667	51782667	0.396083	LOC102171918
1	51682737	51782737	0.381858	LOC102171918
1	51682938	51782938	0.381858	LOC102171918
1	51683673	51783673	0.381858	LOC102171918
1	51683706	51783706	0.381858	LOC102171918
1	51684125	51784125	0.381858	LOC102171918
1	51684402	51784402	0.399191	LOC102171918
1	51684681	51784681	0.392675	LOC102171918
1	51684721	51784721	0.411633	LOC102171918
1	51685106	51785106	0.411633	LOC102171918
1	51685209	51785209	0.413985	LOC102171918
1	51685511	51785511	0.420609	LOC102171918
1	51685662	51785662	0.421481	LOC102171918
1	51685962	51785962	0.410897	LOC102171918
1	51686108	51786108	0.41852	LOC102171918
1	51686292	51786292	0.416909	LOC102171918
1	51686347	51786347	0.416909	LOC102171918

1	51686479	51786479	0.415155	LOC102171918
1	51686682	51786682	0.420527	LOC102171918
1	51687138	51787138	0.421831	LOC102171918
1	51687141	51787141	0.42563	LOC102171918
1	51687399	51787399	0.42576	LOC102171918
1	51687423	51787423	0.4254	LOC102171918
1	51687718	51787718	0.420392	LOC102171918
1	51687824	51787824	0.421488	LOC102171918
1	51688016	51788016	0.421488	LOC102171918
1	51688101	51788101	0.416382	LOC102171918
1	51688300	51788300	0.413854	LOC102171918
1	51688767	51788767	0.415476	LOC102171918
1	51688955	51788955	0.42226	LOC102171918
1	51688958	51788958	0.428969	LOC102171918
1	51689032	51789032	0.425973	LOC102171918
1	51689038	51789038	0.425973	LOC102171918
1	51689054	51789054	0.424942	LOC102171918
1	51689287	51789287	0.424955	LOC102171918
1	51689302	51789302	0.426302	LOC102171918
1	51689472	51789472	0.424087	LOC102171918
1	51689627	51789627	0.424646	LOC102171918
1	51689789	51789789	0.422875	LOC102171918
1	51689890	51789890	0.422925	LOC102171918
1	51689909	51789909	0.422925	LOC102171918
1	51690026	51790026	0.423401	LOC102171918
1	51690099	51790099	0.423889	LOC102171918
1	51690242	51790242	0.412858	LOC102171918
1	51691142	51791142	0.406717	LOC102171918
1	51691523	51791523	0.412513	LOC102171918
1	51691600	51791600	0.414094	LOC102171918
1	51691735	51791735	0.423025	LOC102171918
1	51691889	51791889	0.42956	LOC102171918
1	51692024	51792024	0.410495	LOC102171918
1	51692125	51792125	0.411012	LOC102171918
1	51692799	51792799	0.397486	LOC102171918
1	51693114	51793114	0.400683	LOC102171918
1	51693823	51793823	0.393205	LOC102171918
1	51693861	51793861	0.422576	LOC102171918
1	51694108	51794108	0.429534	LOC102171918
1	51694127	51794127	0.410016	LOC102171918
1	51741000	51841000	0.349693	CCDC54
1	51741282	51841282	0.348303	CCDC54
1	51768529	51868529	0.34503	CCDC54
1	51768629	51868629	0.409488	CCDC54
1	51768635	51868635	0.412826	CCDC54
1	51768636	51868636	0.412398	CCDC54
1	51768645	51868645	0.40141	CCDC54
1	51768647	51868647	0.384369	CCDC54
1	51768733	51868733	0.37516	CCDC54
1	51768736	51868736	0.360713	CCDC54
1	51768743	51868743	0.361977	CCDC54
1	51768748	51868748	0.362259	CCDC54
1	51768751	51868751	0.362259	CCDC54
1	51768950	51868950	0.364843	CCDC54
1	51769645	51869645	0.367724	CCDC54
1	51770357	51870357	0.364941	CCDC54
1	51770558	51870558	0.362949	CCDC54
1	51771034	51871034	0.353056	CCDC54

1	51771057	51871057	0.359307	CCDC54
1	51771113	51871113	0.348857	CCDC54
1	51772374	51872374	0.34353	CCDC54
1	52097310	52197310	0.390092	BBX
1	52097314	52197314	0.384659	BBX
1	52097602	52197602	0.427219	BBX
1	52097893	52197893	0.422013	BBX
1	52097913	52197913	0.421814	BBX
1	52097959	52197959	0.412614	BBX
1	52097974	52197974	0.421243	BBX
1	52097999	52197999	0.418256	BBX
1	52098070	52198070	0.418256	BBX
1	52098865	52198865	0.418256	BBX
1	52098928	52198928	0.424798	BBX
1	52099167	52199167	0.4317	BBX
1	52099314	52199314	0.440221	BBX
1	52099523	52199523	0.416593	BBX
1	52099541	52199541	0.411755	BBX
1	52099665	52199665	0.411755	BBX
1	52099698	52199698	0.482799	BBX
1	52099806	52199806	0.445838	BBX
1	52099887	52199887	0.445838	BBX
1	52100137	52200137	0.445838	BBX
1	52100146	52200146	0.445838	BBX
1	52100398	52200398	0.445838	BBX
1	52100426	52200426	0.445838	BBX
1	52100758	52200758	0.447755	BBX
1	52100775	52200775	0.48267	BBX
1	52100934	52200934	0.540534	BBX
1	52101256	52201256	0.519069	BBX
1	52101734	52201734	0.540598	BBX
1	52101893	52201893	0.561266	BBX
1	52102013	52202013	0.556481	BBX
1	52102053	52202053	0.570148	BBX
1	52102293	52202293	0.557303	BBX
1	52102620	52202620	0.568928	BBX
1	52102666	52202666	0.564239	BBX
1	52102691	52202691	0.557467	BBX
1	52102810	52202810	0.547201	BBX
1	52102896	52202896	0.548113	BBX
1	52102958	52202958	0.547412	BBX
1	52103011	52203011	0.549263	BBX
1	52103045	52203045	0.545406	BBX
1	52103064	52203064	0.548807	BBX
1	52103100	52203100	0.548598	BBX
1	52103179	52203179	0.548598	BBX
1	52103430	52203430	0.562262	BBX
1	52103573	52203573	0.51961	BBX
1	52103582	52203582	0.51961	BBX
1	52103613	52203613	0.51961	BBX
1	52103614	52203614	0.51961	BBX
1	52103816	52203816	0.518842	BBX
1	52104062	52204062	0.520265	BBX
1	52104067	52204067	0.520265	BBX
1	52104074	52204074	0.520265	BBX
1	52104092	52204092	0.531666	BBX
1	52104094	52204094	0.531666	BBX
1	52104116	52204116	0.526219	BBX

1	52104357	52204357	0.508485	BBX
1	52104470	52204470	0.517208	BBX
1	52104473	52204473	0.517208	BBX
1	52104591	52204591	0.483142	BBX
1	52104623	52204623	0.504055	BBX
1	52104798	52204798	0.483803	BBX
1	52104929	52204929	0.479908	BBX
1	52105205	52205205	0.474871	BBX
1	52105399	52205399	0.474871	BBX
1	52106054	52206054	0.473547	BBX
1	52106191	52206191	0.481235	BBX
1	52106505	52206505	0.481235	BBX
1	52106602	52206602	0.476129	BBX
1	52106675	52206675	0.486494	BBX
1	52106676	52206676	0.486494	BBX
1	52106817	52206817	0.479308	BBX
1	52107442	52207442	0.502478	BBX
1	52107501	52207501	0.491478	BBX
1	52107728	52207728	0.507671	BBX
1	52108077	52208077	0.468442	BBX
1	52108163	52208163	0.507475	BBX
1	52108242	52208242	0.47531	BBX
1	52108338	52208338	0.47501	BBX
1	52108434	52208434	0.482374	BBX
1	52108461	52208461	0.483942	BBX
1	52108565	52208565	0.484118	BBX
1	52108588	52208588	0.484118	BBX
1	52108600	52208600	0.484118	BBX
1	52108854	52208854	0.482136	BBX
1	52108938	52208938	0.481233	BBX
1	52108974	52208974	0.502617	BBX
1	52108983	52208983	0.495453	BBX
1	52108997	52208997	0.495453	BBX
1	52109020	52209020	0.495453	BBX
1	52109058	52209058	0.495453	BBX
1	52109149	52209149	0.495453	BBX
1	52109368	52209368	0.495453	BBX
1	52109494	52209494	0.516382	BBX
1	52109531	52209531	0.488731	BBX
1	52109609	52209609	0.488731	BBX
1	52109668	52209668	0.489377	BBX
1	52109728	52209728	0.487851	BBX
1	52110240	52210240	0.489526	BBX
1	52110304	52210304	0.477537	BBX
1	52110311	52210311	0.477537	BBX
1	52110330	52210330	0.484656	BBX
1	52110412	52210412	0.450432	BBX
1	52110586	52210586	0.458556	BBX
1	52110694	52210694	0.452271	BBX
1	52110731	52210731	0.452271	BBX
1	52110880	52210880	0.452271	BBX
1	52110962	52210962	0.46403	BBX
1	52110982	52210982	0.458184	BBX
1	52111079	52211079	0.471103	BBX
1	52111430	52211430	0.43608	BBX
1	52111467	52211467	0.434972	BBX
1	52111705	52211705	0.441317	BBX
1	52111713	52211713	0.414478	BBX

1	52111952	52211952	0.414478	BBX
1	52112122	52212122	0.414478	BBX
1	52112166	52212166	0.414478	BBX
1	52112359	52212359	0.414478	BBX
1	52112377	52212377	0.414478	BBX
1	52112422	52212422	0.414478	BBX
1	52112850	52212850	0.419382	BBX
1	52112878	52212878	0.413546	BBX
1	52113197	52213197	0.413546	BBX
1	52113291	52213291	0.413546	BBX
1	52113301	52213301	0.424186	BBX
1	52113377	52213377	0.423605	BBX
1	52113489	52213489	0.413812	BBX
1	52113862	52213862	0.413812	BBX
1	52113888	52213888	0.423059	BBX
1	52113926	52213926	0.423059	BBX
1	52114201	52214201	0.390072	BBX
1	52114221	52214221	0.390072	BBX
1	52114393	52214393	0.390072	BBX
1	52114471	52214471	0.390072	BBX
1	52114711	52214711	0.390072	BBX
1	52114766	52214766	0.390072	BBX
1	52114817	52214817	0.390072	BBX
1	52114902	52214902	0.398202	BBX
1	52115073	52215073	0.393527	BBX
1	52115412	52215412	0.418771	BBX
1	52115864	52215864	0.394983	BBX
1	52116102	52216102	0.394983	BBX
1	52116279	52216279	0.394983	BBX
1	52116368	52216368	0.394983	BBX
1	52116540	52216540	0.394983	BBX
1	52116723	52216723	0.391167	BBX
1	52116891	52216891	0.387182	BBX
1	52116979	52216979	0.387182	BBX
1	52117692	52217692	0.388453	BBX
1	52117978	52217978	0.401866	BBX
1	52118026	52218026	0.399574	BBX
1	52118091	52218091	0.398931	BBX
1	52118604	52218604	0.427699	BBX
1	52119347	52219347	0.423443	BBX
1	52119705	52219705	0.426379	BBX
1	52120090	52220090	0.419906	BBX
1	52120215	52220215	0.417721	BBX
1	52120508	52220508	0.417594	BBX
1	52120640	52220640	0.421111	BBX
1	52120684	52220684	0.421262	BBX
1	52120708	52220708	0.411788	BBX
1	52120714	52220714	0.411788	BBX
1	52120810	52220810	0.439602	BBX
1	52121576	52221576	0.434277	BBX
1	52121703	52221703	0.446113	BBX
1	52121779	52221779	0.432511	BBX
1	52121803	52221803	0.432511	BBX
1	52122093	52222093	0.433476	BBX
1	52122699	52222699	0.436703	BBX
1	52122792	52222792	0.416599	BBX
1	52122974	52222974	0.415998	BBX
1	52123147	52223147	0.415998	BBX

1	52123477	52223477	0.43097	BBX
1	52123810	52223810	0.437112	BBX
1	52123841	52223841	0.458101	BBX
1	52123924	52223924	0.471985	BBX
1	52123942	52223942	0.438801	BBX
1	52124063	52224063	0.431582	BBX
1	52124255	52224255	0.443337	BBX
1	52124272	52224272	0.442558	BBX
1	52124289	52224289	0.442558	BBX
1	52124404	52224404	0.442558	BBX
1	52124417	52224417	0.435257	BBX
1	52124531	52224531	0.444189	BBX
1	52124554	52224554	0.434941	BBX
1	52124729	52224729	0.433295	BBX
1	52124836	52224836	0.443901	BBX
1	52125029	52225029	0.443815	BBX
1	52125186	52225186	0.443815	BBX
1	52125500	52225500	0.45244	BBX
1	52125567	52225567	0.456816	BBX
1	52125688	52225688	0.443151	BBX
1	52126001	52226001	0.443151	BBX
1	52126022	52226022	0.443151	BBX
1	52126052	52226052	0.442997	BBX
1	52126106	52226106	0.466829	BBX
1	52126209	52226209	0.454822	BBX
1	52126682	52226682	0.454822	BBX
1	52126815	52226815	0.466711	BBX
1	52126935	52226935	0.432621	BBX
1	52127061	52227061	0.463562	BBX
1	52127108	52227108	0.46963	BBX
1	52127304	52227304	0.469642	BBX
1	52127413	52227413	0.448752	BBX
1	52127492	52227492	0.448752	BBX
1	52127646	52227646	0.456815	BBX
1	52127651	52227651	0.456815	BBX
1	52127709	52227709	0.448246	BBX
1	52127730	52227730	0.448246	BBX
1	52127750	52227750	0.453926	BBX
1	52128031	52228031	0.396027	BBX
1	52128320	52228320	0.396027	BBX
1	52128522	52228522	0.404189	BBX
1	52128637	52228637	0.403267	BBX
1	52128647	52228647	0.403613	BBX
1	52128837	52228837	0.39925	BBX
1	52129440	52229440	0.397303	BBX
1	52130009	52230009	0.41179	BBX
1	52130080	52230080	0.403796	BBX
1	52130535	52230535	0.435957	BBX
1	52130800	52230800	0.423717	BBX
1	52130828	52230828	0.429293	BBX
1	52130922	52230922	0.429293	BBX
1	52130929	52230929	0.415934	BBX
1	52131062	52231062	0.415934	BBX
1	52131106	52231106	0.412702	BBX
1	52131110	52231110	0.41287	BBX
1	52131190	52231190	0.41287	BBX
1	52131273	52231273	0.41287	BBX
1	52131420	52231420	0.402998	BBX

1	52131434	52231434	0.402998	BBX
1	52131969	52231969	0.402398	BBX
1	52132170	52232170	0.406133	BBX
1	52132189	52232189	0.407473	BBX
1	52132201	52232201	0.407473	BBX
1	52132285	52232285	0.405382	BBX
1	52132434	52232434	0.407789	BBX
1	52132659	52232659	0.407789	BBX
1	52132767	52232767	0.402766	BBX
1	52133075	52233075	0.419917	BBX
1	52133079	52233079	0.427037	BBX
1	52133154	52233154	0.420841	BBX
1	52133655	52233655	0.420841	BBX
1	52133737	52233737	0.437357	BBX
1	52133810	52233810	0.437947	BBX
1	52133813	52233813	0.436578	BBX
1	52134015	52234015	0.421807	BBX
1	52134060	52234060	0.430831	BBX
1	52134959	52234959	0.455128	BBX
1	52135062	52235062	0.438596	BBX
1	52135160	52235160	0.438596	BBX
1	52135189	52235189	0.447608	BBX
1	52135197	52235197	0.438749	BBX
1	52135289	52235289	0.438116	BBX
1	52135944	52235944	0.431469	BBX
1	52136427	52236427	0.431993	BBX
1	52136435	52236435	0.431993	BBX
1	52136452	52236452	0.431993	BBX
1	52136535	52236535	0.445584	BBX
1	52136646	52236646	0.445584	BBX
1	52136711	52236711	0.438661	BBX
1	52136721	52236721	0.446552	BBX
1	52136793	52236793	0.406287	BBX
1	52136915	52236915	0.415196	BBX
1	52137655	52237655	0.439698	BBX
1	52137865	52237865	0.444359	BBX
1	52138163	52238163	0.38742	BBX
1	52138331	52238331	0.39257	BBX
1	52138885	52238885	0.39029	BBX
1	52138965	52238965	0.385431	BBX
1	52138966	52238966	0.385431	BBX
1	52139277	52239277	0.385431	BBX
1	52139312	52239312	0.386142	BBX
1	52139961	52239961	0.374097	BBX
1	52140098	52240098	0.354719	BBX
1	52140099	52240099	0.354719	BBX
1	52140125	52240125	0.360124	BBX
1	52140342	52240342	0.367264	BBX
1	52140380	52240380	0.367264	BBX
1	52140396	52240396	0.360049	BBX
1	52140847	52240847	0.360822	BBX
1	52140915	52240915	0.367628	BBX
1	52140924	52240924	0.367598	BBX
1	52141031	52241031	0.381426	BBX
1	52141247	52241247	0.377167	BBX
1	52141281	52241281	0.427005	BBX
1	52141289	52241289	0.418716	BBX
1	52141327	52241327	0.418716	BBX

1	52141475	52241475	0.418716	BBX
1	52141775	52241775	0.413645	BBX
1	52141931	52241931	0.452092	BBX
1	52142059	52242059	0.463914	BBX
1	52142145	52242145	0.476137	BBX
1	52142201	52242201	0.482724	BBX
1	52142204	52242204	0.482724	BBX
1	52142207	52242207	0.488444	BBX
1	52142224	52242224	0.483447	BBX
1	52142227	52242227	0.483447	BBX
1	52142319	52242319	0.468793	BBX
1	52142343	52242343	0.444388	BBX
1	52142426	52242426	0.447997	BBX
1	52142507	52242507	0.435822	BBX
1	52142511	52242511	0.435822	BBX
1	52142625	52242625	0.427873	BBX
1	52142683	52242683	0.430653	BBX
1	52143071	52243071	0.430653	BBX
1	52143107	52243107	0.420834	BBX
1	52143122	52243122	0.428539	BBX
1	52143176	52243176	0.44223	BBX
1	52143272	52243272	0.429572	BBX
1	52143311	52243311	0.431831	BBX
1	52143357	52243357	0.4494	BBX
1	52143414	52243414	0.44237	BBX
1	52143471	52243471	0.435062	BBX
1	52143562	52243562	0.437748	BBX
1	52143726	52243726	0.4339	BBX
1	52143735	52243735	0.428279	BBX
1	52143890	52243890	0.425837	BBX
1	52143937	52243937	0.425837	BBX
1	52144057	52244057	0.425868	BBX
1	52144068	52244068	0.395841	BBX
1	52144443	52244443	0.395841	BBX
1	52144979	52244979	0.408118	BBX
1	52145007	52245007	0.407702	BBX
1	52145076	52245076	0.407975	BBX
1	52145123	52245123	0.410129	BBX
1	52145132	52245132	0.40349	BBX
1	52145203	52245203	0.402793	BBX
1	52145376	52245376	0.408004	BBX
1	52146588	52246588	0.371414	BBX
1	52146937	52246937	0.393689	BBX
1	52146939	52246939	0.395113	BBX
1	52146983	52246983	0.410579	BBX
1	52147189	52247189	0.406974	BBX
1	52147276	52247276	0.423827	BBX
1	52147313	52247313	0.414803	BBX
1	52147414	52247414	0.421437	BBX
1	52148021	52248021	0.427167	BBX
1	52148077	52248077	0.420953	BBX
1	52148098	52248098	0.428286	BBX
1	52148146	52248146	0.414386	BBX
1	52148376	52248376	0.422294	BBX
1	52148627	52248627	0.404332	BBX
1	52148676	52248676	0.401436	BBX
1	52148821	52248821	0.406896	BBX
1	52149182	52249182	0.381142	BBX

1	52149394	52249394	0.381142	BBX
1	52149410	52249410	0.381142	BBX
1	52149457	52249457	0.381142	BBX
1	52149519	52249519	0.381142	BBX
1	52149579	52249579	0.381142	BBX
1	52149798	52249798	0.381142	BBX
1	52149898	52249898	0.38229	BBX
1	52150034	52250034	0.381454	BBX
1	52150106	52250106	0.381454	BBX
1	52150129	52250129	0.381454	BBX
1	52150137	52250137	0.381548	BBX
1	52150385	52250385	0.381548	BBX
1	52150436	52250436	0.397314	BBX
1	52150462	52250462	0.396521	BBX
1	52150464	52250464	0.402806	BBX
1	52150587	52250587	0.419895	BBX
1	52150638	52250638	0.413395	BBX
1	52150855	52250855	0.438692	BBX
1	52151059	52251059	0.439558	BBX
1	52151225	52251225	0.426593	BBX
1	52151318	52251318	0.432094	BBX
1	52151561	52251561	0.444243	BBX
1	52151587	52251587	0.459421	BBX
1	52151612	52251612	0.462775	BBX
1	52151854	52251854	0.4636	BBX
1	52151909	52251909	0.474946	BBX
1	52151943	52251943	0.463576	BBX
1	52152061	52252061	0.463342	BBX
1	52152148	52252148	0.465817	BBX
1	52152300	52252300	0.46203	BBX
1	52152457	52252457	0.457017	BBX
1	52152472	52252472	0.482574	BBX
1	52152550	52252550	0.48279	BBX
1	52152661	52252661	0.471136	BBX
1	52152733	52252733	0.465037	BBX
1	52152765	52252765	0.469049	BBX
1	52152845	52252845	0.472886	BBX
1	52152870	52252870	0.467336	BBX
1	52153004	52253004	0.457557	BBX
1	52153121	52253121	0.432037	BBX
1	52153127	52253127	0.424916	BBX
1	52153553	52253553	0.40397	BBX
1	52154336	52254336	0.374002	BBX
1	52154344	52254344	0.386445	BBX
1	52154548	52254548	0.370374	BBX
1	52154856	52254856	0.371849	BBX
1	52155209	52255209	0.378822	BBX
1	52155253	52255253	0.371064	BBX
1	52155459	52255459	0.422983	BBX
1	52155532	52255532	0.400029	BBX
1	52155655	52255655	0.411774	BBX
1	52156284	52256284	0.404465	BBX
1	52156603	52256603	0.392848	BBX
1	52156796	52256796	0.392974	BBX
1	52157165	52257165	0.402743	BBX
1	52157532	52257532	0.379202	BBX
1	52157584	52257584	0.374034	BBX
1	52157589	52257589	0.385642	BBX

1	52157952	52257952	0.349328	BBX
1	52159334	52259334	0.350793	BBX
1	52159406	52259406	0.346349	BBX
1	52159840	52259840	0.342067	BBX
1	52160455	52260455	0.344303	BBX
1	52161849	52261849	0.360324	BBX
1	52165611	52265611	0.346471	BBX
1	52165711	52265711	0.349691	BBX
1	52166006	52266006	0.349691	BBX
1	52166018	52266018	0.354994	BBX
1	52166122	52266122	0.353102	BBX
1	52166337	52266337	0.353102	BBX
1	52166784	52266784	0.358787	BBX
1	52166880	52266880	0.344747	BBX
1	52167087	52267087	0.344635	BBX
1	52167253	52267253	0.355962	BBX
1	52167353	52267353	0.356952	BBX
1	52170256	52270256	0.344557	BBX
1	52171253	52271253	0.346653	BBX
1	52171310	52271310	0.348893	BBX
1	52171315	52271315	0.348893	BBX
1	52171355	52271355	0.356973	BBX
1	52171413	52271413	0.342467	BBX
1	52172985	52272985	0.343465	BBX
1	52173033	52273033	0.343465	BBX
1	52173036	52273036	0.343465	BBX
1	52173040	52273040	0.346305	BBX
1	52173075	52273075	0.346906	BBX
1	52173085	52273085	0.345578	BBX
1	52173110	52273110	0.345578	BBX
1	52173123	52273123	0.346019	BBX
1	52173345	52273345	0.34682	BBX
1	52174186	52274186	0.342903	BBX
1	52174791	52274791	0.365866	BBX
1	52174883	52274883	0.355342	BBX
1	52174897	52274897	0.349437	BBX
1	52175774	52275774	0.347842	BBX
1	52175864	52275864	0.348543	BBX
1	52175891	52275891	0.354683	BBX
1	52175926	52275926	0.35566	BBX
1	52175993	52275993	0.344445	BBX
1	52175994	52275994	0.344445	BBX
1	52176047	52276047	0.344445	BBX
1	52176091	52276091	0.35409	BBX
1	52176092	52276092	0.349186	BBX
1	52176251	52276251	0.349646	BBX
1	52176497	52276497	0.343042	BBX
1	52177113	52277113	0.346663	BBX
1	52177198	52277198	0.344842	BBX
1	52178827	52278827	0.35933	BBX
1	52178948	52278948	0.36274	BBX
1	52179020	52279020	0.358444	BBX
1	52179093	52279093	0.371249	BBX
1	52179095	52279095	0.358132	BBX
1	52179192	52279192	0.352	BBX
1	52179365	52279365	0.361551	BBX
1	52179458	52279458	0.362193	BBX
1	52179929	52279929	0.364634	BBX

1	52180015	52280015	0.364634	BBX
1	52180362	52280362	0.370781	BBX
1	52180418	52280418	0.359797	BBX
1	52181590	52281590	0.345164	BBX
1	52181766	52281766	0.354668	BBX
1	52181875	52281875	0.348893	BBX
1	52181904	52281904	0.364098	BBX
1	52181912	52281912	0.364098	BBX
1	52182183	52282183	0.344159	BBX
1	52182353	52282353	0.355243	BBX
1	52182386	52282386	0.358986	BBX
1	52182496	52282496	0.362314	BBX
1	52182592	52282592	0.364672	BBX
1	52182639	52282639	0.364586	BBX
1	52182677	52282677	0.364437	BBX
1	52182682	52282682	0.354009	BBX
1	52182754	52282754	0.351099	BBX
1	52182778	52282778	0.349534	BBX
1	52182779	52282779	0.348272	BBX
1	52182971	52282971	0.348281	BBX
1	52182989	52282989	0.347405	BBX
1	52183238	52283238	0.357786	BBX
1	52183353	52283353	0.362885	BBX
1	52183368	52283368	0.36549	BBX
1	52183416	52283416	0.36223	BBX
1	52183603	52283603	0.361997	BBX
1	52183648	52283648	0.347453	BBX
1	52183684	52283684	0.348666	BBX
1	52183948	52283948	0.350252	BBX
1	52184013	52284013	0.348997	BBX
1	52184043	52284043	0.34666	BBX
1	52184236	52284236	0.344448	BBX
1	52184321	52284321	0.346286	BBX
1	52184493	52284493	0.347806	BBX
1	52184512	52284512	0.343383	BBX
1	52184650	52284650	0.345909	BBX
1	52185547	52285547	0.346755	BBX
1	52185584	52285584	0.343326	BBX
1	52185692	52285692	0.343326	BBX
1	52188309	52288309	0.369438	BBX
1	52188410	52288410	0.366489	BBX
1	52188457	52288457	0.368862	BBX
1	52188564	52288564	0.362666	BBX
1	52188655	52288655	0.375994	BBX
1	52189033	52289033	0.361361	BBX
1	52189589	52289589	0.366286	BBX
1	52189890	52289890	0.344408	BBX
1	52190010	52290010	0.344408	BBX
1	52190332	52290332	0.344408	BBX
1	52191891	52291891	0.35141	BBX
1	52191946	52291946	0.347344	BBX
1	52191947	52291947	0.363357	BBX
1	52192040	52292040	0.357801	BBX
1	52192043	52292043	0.357801	BBX
1	52192104	52292104	0.357801	BBX
1	52192129	52292129	0.360423	BBX
1	52192169	52292169	0.35323	BBX
1	52192337	52292337	0.35323	BBX

1	52192347	52292347	0.35348	BBX
1	52192489	52292489	0.368155	BBX
1	52192696	52292696	0.352906	BBX
1	52192826	52292826	0.352673	BBX
1	52193078	52293078	0.352822	BBX
1	52193145	52293145	0.352822	BBX
1	52193239	52293239	0.352822	BBX
1	52193347	52293347	0.352822	BBX
1	52193348	52293348	0.36612	BBX
1	52193371	52293371	0.36358	BBX
1	52193393	52293393	0.36358	BBX
1	52193409	52293409	0.368898	BBX
1	52193537	52293537	0.367935	BBX
1	52193553	52293553	0.370068	BBX
1	52193563	52293563	0.371008	BBX
1	52193591	52293591	0.36281	BBX
1	52193607	52293607	0.36281	BBX
1	52193608	52293608	0.36281	BBX
1	52193638	52293638	0.36281	BBX
1	52193755	52293755	0.364347	BBX
1	52193821	52293821	0.36201	BBX
1	52193839	52293839	0.36201	BBX
1	52193942	52293942	0.36201	BBX
1	52193948	52293948	0.361588	BBX
1	52194031	52294031	0.372777	BBX
1	52194328	52294328	0.373094	BBX
1	52194332	52294332	0.379392	BBX
1	52194667	52294667	0.374737	BBX
1	52194774	52294774	0.374737	BBX
1	52194833	52294833	0.379709	BBX
1	52194929	52294929	0.3776	BBX
1	52194965	52294965	0.378217	BBX
1	52195239	52295239	0.386536	BBX
1	52195291	52295291	0.367082	BBX
1	52195907	52295907	0.343982	BBX
1	52196280	52296280	0.343982	BBX
1	52196757	52296757	0.361671	BBX
1	52196845	52296845	0.352793	BBX
1	52196959	52296959	0.353024	BBX
1	52197092	52297092	0.366035	BBX
1	52197238	52297238	0.35063	BBX
1	52197472	52297472	0.35831	BBX
1	52200097	52300097	0.348455	BBX
1	52200238	52300238	0.343234	BBX
1	52200482	52300482	0.34553	BBX
1	52200568	52300568	0.349923	BBX
1	52200574	52300574	0.355199	BBX
1	52203271	52303271	0.347512	BBX
1	52203356	52303356	0.366018	BBX
1	52203495	52303495	0.370547	BBX
1	52203526	52303526	0.37032	BBX
1	52203589	52303589	0.361905	BBX
1	52204061	52304061	0.359904	BBX
1	52204127	52304127	0.373305	BBX
1	52204274	52304274	0.360829	BBX
1	52204473	52304473	0.368462	BBX
1	52204523	52304523	0.363116	BBX
1	52204675	52304675	0.363525	BBX

1	52205003	52305003	0.361226	BBX
1	52205325	52305325	0.354436	BBX
1	52205437	52305437	0.362746	BBX
1	52205469	52305469	0.365999	BBX
1	52205508	52305508	0.372204	BBX
1	52205638	52305638	0.3418	BBX
1	52206015	52306015	0.350893	BBX
1	52206182	52306182	0.362432	BBX
1	52206504	52306504	0.366554	BBX
1	52206584	52306584	0.372047	BBX
1	52206592	52306592	0.3768	BBX
1	52206866	52306866	0.384337	BBX
1	52206919	52306919	0.433231	BBX
1	52703905	52803905	0.344014	IFT57
1	52703942	52803942	0.357947	IFT57
1	53459776	53559776	0.349777	GUCA1C
1	53459776	53559776	0.349777	LOC102175896
1	53459807	53559807	0.350905	GUCA1C
1	53459807	53559807	0.350905	LOC102175896
1	53460624	53560624	0.350381	GUCA1C
1	53460624	53560624	0.350381	LOC102175896
1	53461020	53561020	0.348546	GUCA1C
1	53461020	53561020	0.348546	LOC102175896
1	53461087	53561087	0.34336	GUCA1C
1	53461087	53561087	0.34336	LOC102175896
1	53461502	53561502	0.344071	GUCA1C
1	53461502	53561502	0.344071	LOC102175896
1	53461694	53561694	0.359568	GUCA1C
1	53461694	53561694	0.359568	LOC102175896
1	53461719	53561719	0.357965	GUCA1C
1	53461719	53561719	0.357965	LOC102175896
1	53461763	53561763	0.349459	GUCA1C
1	53461763	53561763	0.349459	LOC102175896
1	53462080	53562080	0.353607	GUCA1C
1	53462080	53562080	0.353607	LOC102175896
1	53462108	53562108	0.357451	GUCA1C
1	53462108	53562108	0.357451	LOC102175896
1	53462253	53562253	0.359586	GUCA1C
1	53462253	53562253	0.359586	LOC102175896
1	53462444	53562444	0.367498	GUCA1C
1	53462444	53562444	0.367498	LOC102175896
1	53462470	53562470	0.385429	GUCA1C
1	53462470	53562470	0.385429	LOC102175896
1	53462554	53562554	0.354824	GUCA1C
1	53462554	53562554	0.354824	LOC102175896
1	53462685	53562685	0.347182	GUCA1C
1	53462685	53562685	0.347182	LOC102175896
1	53462936	53562936	0.347182	GUCA1C
1	53462936	53562936	0.347182	LOC102175896
1	53463024	53563024	0.347182	GUCA1C
1	53463024	53563024	0.347182	LOC102175896
1	53463109	53563109	0.347182	GUCA1C
1	53463109	53563109	0.347182	LOC102175896
1	53463133	53563133	0.363028	GUCA1C
1	53463133	53563133	0.363028	LOC102175896
1	53463134	53563134	0.380372	GUCA1C
1	53463134	53563134	0.380372	LOC102175896
1	53463138	53563138	0.378089	GUCA1C

1	53463138	53563138	0.378089	LOC102175896
1	53463174	53563174	0.366888	GUCA1C
1	53463174	53563174	0.366888	LOC102175896
1	53463182	53563182	0.368132	GUCA1C
1	53463182	53563182	0.368132	LOC102175896
1	53463202	53563202	0.366529	GUCA1C
1	53463202	53563202	0.366529	LOC102175896
1	53463282	53563282	0.36126	GUCA1C
1	53463282	53563282	0.36126	LOC102175896
1	53463294	53563294	0.355678	GUCA1C
1	53463294	53563294	0.355678	LOC102175896
1	53463344	53563344	0.355678	GUCA1C
1	53463344	53563344	0.355678	LOC102175896
1	53463487	53563487	0.346814	GUCA1C
1	53463487	53563487	0.346814	LOC102175896
1	53463609	53563609	0.346814	GUCA1C
1	53463609	53563609	0.346814	LOC102175896
1	53463621	53563621	0.360148	GUCA1C
1	53463621	53563621	0.360148	LOC102175896
1	53463741	53563741	0.352742	GUCA1C
1	53463741	53563741	0.352742	LOC102175896
1	53463755	53563755	0.363232	GUCA1C
1	53463755	53563755	0.363232	LOC102175896
1	53463761	53563761	0.363958	GUCA1C
1	53463761	53563761	0.363958	LOC102175896
1	53463788	53563788	0.370679	GUCA1C
1	53463788	53563788	0.370679	LOC102175896
1	53463914	53563914	0.364345	GUCA1C
1	53463914	53563914	0.364345	LOC102175896
1	53464065	53564065	0.364568	GUCA1C
1	53464065	53564065	0.364568	LOC102175896
1	53464201	53564201	0.356325	GUCA1C
1	53464201	53564201	0.356325	LOC102175896
1	53464925	53564925	0.342657	GUCA1C
1	53464925	53564925	0.342657	LOC102175896
1	53464936	53564936	0.347564	GUCA1C
1	53464936	53564936	0.347564	LOC102175896
1	53465127	53565127	0.348888	GUCA1C
1	53465127	53565127	0.348888	LOC102175896
1	53465210	53565210	0.360719	GUCA1C
1	53465210	53565210	0.360719	LOC102175896
1	53465272	53565272	0.352458	GUCA1C
1	53465272	53565272	0.352458	LOC102175896
1	53465298	53565298	0.359544	GUCA1C
1	53465298	53565298	0.359544	LOC102175896
1	53465344	53565344	0.34847	GUCA1C
1	53465344	53565344	0.34847	LOC102175896
1	53465351	53565351	0.355168	GUCA1C
1	53465351	53565351	0.355168	LOC102175896
1	53465495	53565495	0.353619	GUCA1C
1	53465495	53565495	0.353619	LOC102175896
1	53465603	53565603	0.353771	GUCA1C
1	53465603	53565603	0.353771	LOC102175896
1	53465630	53565630	0.357484	GUCA1C
1	53465630	53565630	0.357484	LOC102175896
1	53465658	53565658	0.357032	GUCA1C
1	53465658	53565658	0.357032	LOC102175896
1	53465662	53565662	0.354317	GUCA1C

1	53465662	53565662	0.354317	LOC102175896
1	53465779	53565779	0.357652	GUCA1C
1	53465779	53565779	0.357652	LOC102175896
1	53465902	53565902	0.346912	GUCA1C
1	53465902	53565902	0.346912	LOC102175896
1	53466016	53566016	0.353158	GUCA1C
1	53466016	53566016	0.353158	LOC102175896
1	53467050	53567050	0.350673	GUCA1C
1	53467050	53567050	0.350673	LOC102175896
1	53467099	53567099	0.363761	GUCA1C
1	53467099	53567099	0.363761	LOC102175896
1	53467138	53567138	0.345304	GUCA1C
1	53467138	53567138	0.345304	LOC102175896
1	53467345	53567345	0.345086	GUCA1C
1	53467345	53567345	0.345086	LOC102175896
1	53467455	53567455	0.345086	GUCA1C
1	53467455	53567455	0.345086	LOC102175896
1	53467525	53567525	0.345086	GUCA1C
1	53467525	53567525	0.345086	LOC102175896
1	53467557	53567557	0.345086	GUCA1C
1	53467557	53567557	0.345086	LOC102175896
1	53467565	53567565	0.359845	GUCA1C
1	53467565	53567565	0.359845	LOC102175896
1	53467596	53567596	0.348666	GUCA1C
1	53467596	53567596	0.348666	LOC102175896
1	53467911	53567911	0.354364	GUCA1C
1	53467911	53567911	0.354364	LOC102175896
1	53467942	53567942	0.368181	GUCA1C
1	53467942	53567942	0.368181	LOC102175896
1	53467947	53567947	0.367363	GUCA1C
1	53467947	53567947	0.367363	LOC102175896
1	53468106	53568106	0.367497	GUCA1C
1	53468106	53568106	0.367497	LOC102175896
1	53468147	53568147	0.366636	GUCA1C
1	53468147	53568147	0.366636	LOC102175896
1	53468187	53568187	0.363792	GUCA1C
1	53468187	53568187	0.363792	LOC102175896
1	53468225	53568225	0.383419	GUCA1C
1	53468225	53568225	0.383419	LOC102175896
1	53468328	53568328	0.385647	GUCA1C
1	53468328	53568328	0.385647	LOC102175896
1	53468386	53568386	0.386282	GUCA1C
1	53468386	53568386	0.386282	LOC102175896
1	53468392	53568392	0.386282	GUCA1C
1	53468392	53568392	0.386282	LOC102175896
1	53468418	53568418	0.389466	GUCA1C
1	53468418	53568418	0.389466	LOC102175896
1	53468436	53568436	0.385343	GUCA1C
1	53468436	53568436	0.385343	LOC102175896
1	53468495	53568495	0.387009	GUCA1C
1	53468495	53568495	0.387009	LOC102175896
1	53468496	53568496	0.391392	GUCA1C
1	53468496	53568496	0.391392	LOC102175896
1	53468530	53568530	0.394341	GUCA1C
1	53468530	53568530	0.394341	LOC102175896
1	53468536	53568536	0.388395	GUCA1C
1	53468536	53568536	0.388395	LOC102175896
1	53468539	53568539	0.388395	GUCA1C

1	53468539	53568539	0.388395	LOC102175896
1	53468552	53568552	0.375069	GUCA1C
1	53468552	53568552	0.375069	LOC102175896
1	53468574	53568574	0.383729	GUCA1C
1	53468574	53568574	0.383729	LOC102175896
1	53468611	53568611	0.381392	GUCA1C
1	53468611	53568611	0.381392	LOC102175896
1	53468634	53568634	0.386282	GUCA1C
1	53468634	53568634	0.386282	LOC102175896
1	53468746	53568746	0.3788	GUCA1C
1	53468746	53568746	0.3788	LOC102175896
1	53468750	53568750	0.37437	GUCA1C
1	53468750	53568750	0.37437	LOC102175896
1	53468829	53568829	0.370696	GUCA1C
1	53468829	53568829	0.370696	LOC102175896
1	53468875	53568875	0.362712	GUCA1C
1	53468875	53568875	0.362712	LOC102175896
1	53468891	53568891	0.370983	GUCA1C
1	53468891	53568891	0.370983	LOC102175896
1	53468950	53568950	0.361744	GUCA1C
1	53468950	53568950	0.361744	LOC102175896
1	53469076	53569076	0.361744	GUCA1C
1	53469076	53569076	0.361744	LOC102175896
1	53469171	53569171	0.366526	GUCA1C
1	53469171	53569171	0.366526	LOC102175896
1	53469207	53569207	0.366248	GUCA1C
1	53469207	53569207	0.366248	LOC102175896
1	53469360	53569360	0.359855	GUCA1C
1	53469360	53569360	0.359855	LOC102175896
1	53469527	53569527	0.368305	GUCA1C
1	53469527	53569527	0.368305	LOC102175896
1	53469573	53569573	0.369454	GUCA1C
1	53469573	53569573	0.369454	LOC102175896
1	53469647	53569647	0.369454	GUCA1C
1	53469647	53569647	0.369454	LOC102175896
1	53469688	53569688	0.355368	GUCA1C
1	53469688	53569688	0.355368	LOC102175896
1	53469770	53569770	0.363705	GUCA1C
1	53469770	53569770	0.363705	LOC102175896
1	53469771	53569771	0.363705	GUCA1C
1	53469771	53569771	0.363705	LOC102175896
1	53469826	53569826	0.359233	GUCA1C
1	53469826	53569826	0.359233	LOC102175896
1	53469900	53569900	0.361708	GUCA1C
1	53469900	53569900	0.361708	LOC102175896
1	53469968	53569968	0.364134	GUCA1C
1	53469968	53569968	0.364134	LOC102175896
1	53469993	53569993	0.3673	GUCA1C
1	53469993	53569993	0.3673	LOC102175896
1	53470040	53570040	0.360333	GUCA1C
1	53470040	53570040	0.360333	LOC102175896
1	53470097	53570097	0.349547	GUCA1C
1	53470097	53570097	0.349547	LOC102175896
1	53470189	53570189	0.355701	GUCA1C
1	53470189	53570189	0.355701	LOC102175896
1	53470286	53570286	0.345688	GUCA1C
1	53470286	53570286	0.345688	LOC102175896
1	53470550	53570550	0.349347	GUCA1C

1	53470550	53570550	0.349347	LOC102175896
1	53470718	53570718	0.342331	GUCA1C
1	53470718	53570718	0.342331	LOC102175896
1	53470779	53570779	0.342331	GUCA1C
1	53470779	53570779	0.342331	LOC102175896
1	53470807	53570807	0.361547	GUCA1C
1	53470807	53570807	0.361547	LOC102175896
1	53470831	53570831	0.352176	GUCA1C
1	53470831	53570831	0.352176	LOC102175896
1	53470883	53570883	0.354489	GUCA1C
1	53470883	53570883	0.354489	LOC102175896
1	53470913	53570913	0.362883	GUCA1C
1	53470913	53570913	0.362883	LOC102175896
1	53470944	53570944	0.362872	GUCA1C
1	53470944	53570944	0.362872	LOC102175896
1	53470997	53570997	0.442663	GUCA1C
1	53470997	53570997	0.442663	LOC102175896
1	53470999	53570999	0.433936	GUCA1C
1	53470999	53570999	0.433936	LOC102175896
1	53471037	53571037	0.437569	GUCA1C
1	53471037	53571037	0.437569	LOC102175896
1	53471067	53571067	0.42948	GUCA1C
1	53471067	53571067	0.42948	LOC102175896
1	53471120	53571120	0.421453	GUCA1C
1	53471120	53571120	0.421453	LOC102175896
1	53471121	53571121	0.421271	GUCA1C
1	53471121	53571121	0.421271	LOC102175896
1	53471123	53571123	0.42672	GUCA1C
1	53471123	53571123	0.42672	LOC102175896
1	53471191	53571191	0.430208	GUCA1C
1	53471191	53571191	0.430208	LOC102175896
1	53471288	53571288	0.420081	GUCA1C
1	53471288	53571288	0.420081	LOC102175896
1	53471437	53571437	0.402198	GUCA1C
1	53471437	53571437	0.402198	LOC102175896
1	53471469	53571469	0.36871	GUCA1C
1	53471469	53571469	0.36871	LOC102175896
1	53471490	53571490	0.392534	GUCA1C
1	53471490	53571490	0.392534	LOC102175896
1	53471509	53571509	0.362539	GUCA1C
1	53471509	53571509	0.362539	LOC102175896
1	53471615	53571615	0.368309	GUCA1C
1	53471615	53571615	0.368309	LOC102175896
1	53471861	53571861	0.368309	GUCA1C
1	53471861	53571861	0.368309	LOC102175896
1	53471897	53571897	0.393945	GUCA1C
1	53471897	53571897	0.393945	LOC102175896
1	53471972	53571972	0.34611	GUCA1C
1	53471972	53571972	0.34611	LOC102175896
1	53472069	53572069	0.347375	GUCA1C
1	53472069	53572069	0.347375	LOC102175896
1	53472098	53572098	0.347375	GUCA1C
1	53472098	53572098	0.347375	LOC102175896
1	53472197	53572197	0.35803	GUCA1C
1	53472197	53572197	0.35803	LOC102175896
1	53472480	53572480	0.360831	GUCA1C
1	53472480	53572480	0.360831	LOC102175896
1	53472667	53572667	0.346552	GUCA1C

1	53472667	53572667	0.346552	LOC102175896
1	53473717	53573717	0.349439	GUCA1C
1	53473717	53573717	0.349439	LOC102175896
1	53473901	53573901	0.343499	GUCA1C
1	53473901	53573901	0.343499	LOC102175896
1	53473929	53573929	0.374104	GUCA1C
1	53473929	53573929	0.374104	LOC102175896
1	53473975	53573975	0.347989	GUCA1C
1	53473975	53573975	0.347989	LOC102175896
1	53473978	53573978	0.357877	GUCA1C
1	53473978	53573978	0.357877	LOC102175896
1	53474142	53574142	0.357573	GUCA1C
1	53474142	53574142	0.357573	LOC102175896
1	53474300	53574300	0.357628	GUCA1C
1	53474300	53574300	0.357628	LOC102175896
1	53474301	53574301	0.349844	GUCA1C
1	53474301	53574301	0.349844	LOC102175896
1	53474381	53574381	0.365727	GUCA1C
1	53474381	53574381	0.365727	LOC102175896
1	53474442	53574442	0.372457	GUCA1C
1	53474442	53574442	0.372457	LOC102175896
1	53474453	53574453	0.373555	GUCA1C
1	53474453	53574453	0.373555	LOC102175896
1	53474467	53574467	0.369345	GUCA1C
1	53474467	53574467	0.369345	LOC102175896
1	53474472	53574472	0.369345	GUCA1C
1	53474472	53574472	0.369345	LOC102175896
1	53474481	53574481	0.378162	GUCA1C
1	53474481	53574481	0.378162	LOC102175896
1	53474586	53574586	0.376445	GUCA1C
1	53474586	53574586	0.376445	LOC102175896
1	53474617	53574617	0.377308	GUCA1C
1	53474617	53574617	0.377308	LOC102175896
1	53474618	53574618	0.377308	GUCA1C
1	53474618	53574618	0.377308	LOC102175896
1	53474641	53574641	0.374841	GUCA1C
1	53474641	53574641	0.374841	LOC102175896
1	53474689	53574689	0.411723	GUCA1C
1	53474689	53574689	0.411723	LOC102175896
1	53474730	53574730	0.397137	GUCA1C
1	53474730	53574730	0.397137	LOC102175896
1	53474733	53574733	0.40209	GUCA1C
1	53474733	53574733	0.40209	LOC102175896
1	53474778	53574778	0.404354	GUCA1C
1	53474778	53574778	0.404354	LOC102175896
1	53474889	53574889	0.42107	GUCA1C
1	53474889	53574889	0.42107	LOC102175896
1	53474942	53574942	0.422435	GUCA1C
1	53474942	53574942	0.422435	LOC102175896
1	53475316	53575316	0.393762	GUCA1C
1	53475316	53575316	0.393762	LOC102175896
1	53475332	53575332	0.393762	GUCA1C
1	53475332	53575332	0.393762	LOC102175896
1	53476023	53576023	0.343218	GUCA1C
1	53476023	53576023	0.343218	LOC102175896
1	55665657	55765657	0.425396	NECTIN3
1	55665659	55765659	0.425396	NECTIN3
1	55665706	55765706	0.430733	NECTIN3

1	55665869	55765869	0.430733	NECTIN3
1	55665990	55765990	0.441161	NECTIN3
1	55666011	55766011	0.4475	NECTIN3
1	55666023	55766023	0.43004	NECTIN3
1	55666144	55766144	0.433749	NECTIN3
1	55666150	55766150	0.431649	NECTIN3
1	55666220	55766220	0.426273	NECTIN3
1	55666624	55766624	0.410049	NECTIN3
1	55666983	55766983	0.410049	NECTIN3
1	55667038	55767038	0.420826	NECTIN3
1	55667159	55767159	0.419754	NECTIN3
1	55667352	55767352	0.407841	NECTIN3
1	55667372	55767372	0.407841	NECTIN3
1	55667421	55767421	0.407841	NECTIN3
1	55667494	55767494	0.407841	NECTIN3
1	55667514	55767514	0.407531	NECTIN3
1	55667691	55767691	0.408011	NECTIN3
1	55667701	55767701	0.422442	NECTIN3
1	55668380	55768380	0.414704	NECTIN3
1	55668402	55768402	0.414039	NECTIN3
1	55668597	55768597	0.427004	NECTIN3
1	55668671	55768671	0.432251	NECTIN3
1	55668829	55768829	0.416565	NECTIN3
1	55668852	55768852	0.418099	NECTIN3
1	55669142	55769142	0.411911	NECTIN3
1	55669532	55769532	0.419355	NECTIN3
1	55669607	55769607	0.419456	NECTIN3
1	55669645	55769645	0.419226	NECTIN3
1	55669833	55769833	0.421863	NECTIN3
1	55670135	55770135	0.413799	NECTIN3
1	55670171	55770171	0.426544	NECTIN3
1	55670676	55770676	0.423137	NECTIN3
1	55670753	55770753	0.425656	NECTIN3
1	55670916	55770916	0.411173	NECTIN3
1	55671364	55771364	0.411173	NECTIN3
1	55671424	55771424	0.420589	NECTIN3
1	55671815	55771815	0.421836	NECTIN3
1	55672515	55772515	0.411415	NECTIN3
1	55672648	55772648	0.396992	NECTIN3
1	55672701	55772701	0.398735	NECTIN3
1	55672890	55772890	0.398903	NECTIN3
1	55673081	55773081	0.397413	NECTIN3
1	55673285	55773285	0.411908	NECTIN3
1	55673517	55773517	0.419643	NECTIN3
1	55673584	55773584	0.411372	NECTIN3
1	55674000	55774000	0.409819	NECTIN3
1	55674052	55774052	0.483204	NECTIN3
1	55674205	55774205	0.490346	NECTIN3
1	55674273	55774273	0.486509	NECTIN3
1	55674599	55774599	0.495649	NECTIN3
1	55674652	55774652	0.4909	NECTIN3
1	55674697	55774697	0.494637	NECTIN3
1	55674875	55774875	0.479605	NECTIN3
1	55675037	55775037	0.489587	NECTIN3
1	55675164	55775164	0.484507	NECTIN3
1	55675388	55775388	0.48465	NECTIN3
1	55675632	55775632	0.481103	NECTIN3
1	55675692	55775692	0.446961	NECTIN3

1	55675755	55775755	0.45405	NECTIN3
1	55676384	55776384	0.454198	NECTIN3
1	55676832	55776832	0.446185	NECTIN3
1	55677077	55777077	0.446185	NECTIN3
1	55677807	55777807	0.462504	NECTIN3
1	55677961	55777961	0.460393	NECTIN3
1	55678860	55778860	0.453201	NECTIN3
1	55679030	55779030	0.470239	NECTIN3
1	55679085	55779085	0.477752	NECTIN3
1	55679184	55779184	0.500932	NECTIN3
1	55679457	55779457	0.500132	NECTIN3
1	55679702	55779702	0.492866	NECTIN3
1	55679764	55779764	0.473755	NECTIN3
1	55679769	55779769	0.455471	NECTIN3
1	55679843	55779843	0.455471	NECTIN3
1	55680659	55780659	0.455471	NECTIN3
1	55680660	55780660	0.455637	NECTIN3
1	55680774	55780774	0.457093	NECTIN3
1	55681401	55781401	0.463523	NECTIN3
1	55681500	55781500	0.483964	NECTIN3
1	55681990	55781990	0.479407	NECTIN3
1	55682013	55782013	0.477002	NECTIN3
1	55682033	55782033	0.49716	NECTIN3
1	55682043	55782043	0.562399	NECTIN3
1	55682076	55782076	0.574362	NECTIN3
1	55682251	55782251	0.584529	NECTIN3
1	55682275	55782275	0.563853	NECTIN3
1	55682764	55782764	0.559527	NECTIN3
1	55682822	55782822	0.561626	NECTIN3
1	55683028	55783028	0.564335	NECTIN3
1	55683125	55783125	0.567045	NECTIN3
1	55683705	55783705	0.560288	NECTIN3
1	55683725	55783725	0.556913	NECTIN3
1	55684036	55784036	0.56277	NECTIN3
1	55684258	55784258	0.557674	NECTIN3
1	55684272	55784272	0.56317	NECTIN3
1	55684339	55784339	0.558343	NECTIN3
1	55684680	55784680	0.552268	NECTIN3
1	55684693	55784693	0.552268	NECTIN3
1	55684695	55784695	0.553452	NECTIN3
1	55684806	55784806	0.549318	NECTIN3
1	55684850	55784850	0.56197	NECTIN3
1	55684929	55784929	0.56383	NECTIN3
1	55684956	55784956	0.55752	NECTIN3
1	55685000	55785000	0.558948	NECTIN3
1	55685181	55785181	0.540876	NECTIN3
1	55685448	55785448	0.53959	NECTIN3
1	55685663	55785663	0.540907	NECTIN3
1	55685763	55785763	0.559583	NECTIN3
1	55685867	55785867	0.446246	NECTIN3
1	55685881	55785881	0.452989	NECTIN3
1	55685969	55785969	0.452977	NECTIN3
1	55686020	55786020	0.45799	NECTIN3
1	55686096	55786096	0.451247	NECTIN3
1	55686695	55786695	0.451247	NECTIN3
1	55686722	55786722	0.455577	NECTIN3
1	55687416	55787416	0.441124	NECTIN3
1	55687435	55787435	0.435843	NECTIN3

1	55687459	55787459	0.435806	NECTIN3
1	55687819	55787819	0.436032	NECTIN3
1	55688074	55788074	0.443013	NECTIN3
1	55689160	55789160	0.436811	NECTIN3
1	55689344	55789344	0.446947	NECTIN3
1	55689400	55789400	0.44237	NECTIN3
1	55689711	55789711	0.447581	NECTIN3
1	55689752	55789752	0.447663	NECTIN3
1	55689754	55789754	0.444877	NECTIN3
1	55690221	55790221	0.443207	NECTIN3
1	55690382	55790382	0.451074	NECTIN3
1	55690562	55790562	0.450144	NECTIN3
1	55691182	55791182	0.448656	NECTIN3
1	55691238	55791238	0.457808	NECTIN3
1	55691260	55791260	0.444326	NECTIN3
1	55691630	55791630	0.446204	NECTIN3
1	55691897	55791897	0.445344	NECTIN3
1	55692021	55792021	0.445324	NECTIN3
1	55692023	55792023	0.445412	NECTIN3
1	55692204	55792204	0.443004	NECTIN3
1	55692527	55792527	0.448007	NECTIN3
1	55692556	55792556	0.447312	NECTIN3
1	55692600	55792600	0.444333	NECTIN3
1	55692879	55792879	0.467232	NECTIN3
1	55693456	55793456	0.405841	NECTIN3
1	55693467	55793467	0.406469	NECTIN3
1	55693609	55793609	0.402087	NECTIN3
1	55694184	55794184	0.403915	NECTIN3
1	55694437	55794437	0.409195	NECTIN3
1	55694439	55794439	0.399363	NECTIN3
1	55694598	55794598	0.399666	NECTIN3
1	55694860	55794860	0.408463	NECTIN3
1	55694905	55794905	0.410419	NECTIN3
1	55694981	55794981	0.394702	NECTIN3
1	55695111	55795111	0.393385	NECTIN3
1	55695154	55795154	0.397121	NECTIN3
1	55695313	55795313	0.388532	NECTIN3
1	55695792	55795792	0.389104	NECTIN3
1	55696104	55796104	0.38835	NECTIN3
1	55696274	55796274	0.387469	NECTIN3
1	55696353	55796353	0.387815	NECTIN3
1	55696522	55796522	0.387799	NECTIN3
1	55697105	55797105	0.391115	NECTIN3
1	55697158	55797158	0.393624	NECTIN3
1	55697310	55797310	0.390229	NECTIN3
1	55697350	55797350	0.38042	NECTIN3
1	55697551	55797551	0.35951	NECTIN3
1	55697811	55797811	0.353314	NECTIN3
1	55697902	55797902	0.365387	NECTIN3
1	55697920	55797920	0.365387	NECTIN3
1	55698412	55798412	0.365387	NECTIN3
1	55698800	55798800	0.365387	NECTIN3
1	55699157	55799157	0.365387	NECTIN3
1	55699361	55799361	0.386789	NECTIN3
1	55699725	55799725	0.378916	NECTIN3
1	55699736	55799736	0.363853	NECTIN3
1	55699763	55799763	0.363853	NECTIN3
1	55699867	55799867	0.363853	NECTIN3

1	55701065	55801065	0.363853	NECTIN3
1	55701595	55801595	0.380737	NECTIN3
1	55702519	55802519	0.363936	NECTIN3
1	55702619	55802619	0.3666	NECTIN3
1	55702723	55802723	0.383576	NECTIN3
1	55702793	55802793	0.376719	NECTIN3
1	55702956	55802956	0.385084	NECTIN3
1	55703336	55803336	0.375403	NECTIN3
1	55703350	55803350	0.372598	NECTIN3
1	55703779	55803779	0.384868	NECTIN3
1	55704942	55804942	0.389506	NECTIN3
1	55705061	55805061	0.377391	NECTIN3
1	55705325	55805325	0.379916	NECTIN3
1	55705576	55805576	0.379424	NECTIN3
1	55705635	55805635	0.353478	NECTIN3
1	55706441	55806441	0.367541	NECTIN3
1	55707016	55807016	0.36868	NECTIN3
1	55707287	55807287	0.356363	NECTIN3
1	55708596	55808596	0.368979	NECTIN3
1	55708710	55808710	0.36701	NECTIN3
1	55709086	55809086	0.368922	NECTIN3
1	55709553	55809553	0.364567	NECTIN3
1	55709766	55809766	0.364567	NECTIN3
1	55709886	55809886	0.369368	NECTIN3
1	55710124	55810124	0.363664	NECTIN3
1	55710145	55810145	0.363664	NECTIN3
1	55710393	55810393	0.378025	NECTIN3
1	55710753	55810753	0.383745	NECTIN3
1	55711004	55811004	0.380912	NECTIN3
1	55711130	55811130	0.381454	NECTIN3
1	55711428	55811428	0.387529	NECTIN3
1	55711479	55811479	0.386815	NECTIN3
1	55711692	55811692	0.374108	NECTIN3
1	55712838	55812838	0.370069	NECTIN3
1	55712940	55812940	0.383303	NECTIN3
1	55712971	55812971	0.373468	NECTIN3
1	55713014	55813014	0.390046	NECTIN3
1	55713062	55813062	0.380915	NECTIN3
1	55713287	55813287	0.384966	NECTIN3
1	55713379	55813379	0.365945	NECTIN3
1	55714878	55814878	0.370534	NECTIN3
1	55714903	55814903	0.372472	NECTIN3
1	55717319	55817319	0.376937	NECTIN3
1	55717705	55817705	0.38219	NECTIN3
1	55718124	55818124	0.377922	NECTIN3
1	55718176	55818176	0.387731	NECTIN3
1	55718330	55818330	0.369975	NECTIN3
1	55718992	55818992	0.371222	NECTIN3
1	55719209	55819209	0.386871	NECTIN3
1	55719351	55819351	0.407564	NECTIN3
1	55719693	55819693	0.396298	NECTIN3
1	55719732	55819732	0.391181	NECTIN3
1	55719791	55819791	0.396898	NECTIN3
1	55720336	55820336	0.389264	NECTIN3
1	55720462	55820462	0.39448	NECTIN3
1	55720500	55820500	0.397188	NECTIN3
1	55721165	55821165	0.398141	NECTIN3
1	55721185	55821185	0.396149	NECTIN3

1	55721360	55821360	0.3972	NECTIN3
1	55721660	55821660	0.392369	NECTIN3
1	55721707	55821707	0.39492	NECTIN3
1	55722051	55822051	0.393509	NECTIN3
1	55722225	55822225	0.39477	NECTIN3
1	55722451	55822451	0.387557	NECTIN3
1	55722613	55822613	0.385472	NECTIN3
1	55723287	55823287	0.394314	NECTIN3
1	55723460	55823460	0.399045	NECTIN3
1	55723617	55823617	0.395614	NECTIN3
1	55723626	55823626	0.396535	NECTIN3
1	55723753	55823753	0.389257	NECTIN3
1	55723836	55823836	0.389257	NECTIN3
1	55723927	55823927	0.389257	NECTIN3
1	55723971	55823971	0.387105	NECTIN3
1	55723997	55823997	0.392567	NECTIN3
1	55724166	55824166	0.389376	NECTIN3
1	55724194	55824194	0.394294	NECTIN3
1	55724686	55824686	0.394294	NECTIN3
1	55724777	55824777	0.393656	NECTIN3
1	55724830	55824830	0.385358	NECTIN3
1	55725128	55825128	0.3898	NECTIN3
1	55725281	55825281	0.388913	NECTIN3
1	55725545	55825545	0.388909	NECTIN3
1	55726025	55826025	0.379439	NECTIN3
1	55726268	55826268	0.382698	NECTIN3
1	55726297	55826297	0.383532	NECTIN3
1	55726445	55826445	0.383532	NECTIN3
1	55726533	55826533	0.383532	NECTIN3
1	55726538	55826538	0.388166	NECTIN3
1	55727044	55827044	0.388166	NECTIN3
1	55727538	55827538	0.388514	NECTIN3
1	55727624	55827624	0.404391	NECTIN3
1	55727689	55827689	0.402729	NECTIN3
1	55727780	55827780	0.399071	NECTIN3
1	55727855	55827855	0.401056	NECTIN3
1	55727969	55827969	0.396623	NECTIN3
1	55728406	55828406	0.401407	NECTIN3
1	55728513	55828513	0.397035	NECTIN3
1	55728770	55828770	0.393991	NECTIN3
1	55729017	55829017	0.401228	NECTIN3
1	55729427	55829427	0.401228	NECTIN3
1	55729428	55829428	0.398236	NECTIN3
1	55729625	55829625	0.401273	NECTIN3
1	55729793	55829793	0.408496	NECTIN3
1	55729804	55829804	0.407653	NECTIN3
1	55729856	55829856	0.413261	NECTIN3
1	55729880	55829880	0.412806	NECTIN3
1	55729906	55829906	0.412946	NECTIN3
1	55729934	55829934	0.410513	NECTIN3
1	55730059	55830059	0.410919	NECTIN3
1	55730079	55830079	0.401838	NECTIN3
1	55730109	55830109	0.395459	NECTIN3
1	55730236	55830236	0.389799	NECTIN3
1	55730420	55830420	0.398429	NECTIN3
1	55731277	55831277	0.397695	NECTIN3
1	55731432	55831432	0.386503	NECTIN3
1	55731551	55831551	0.392217	NECTIN3

1	55731926	55831926	0.388939	NECTIN3
1	55732421	55832421	0.390933	NECTIN3
1	55732533	55832533	0.39665	NECTIN3
1	55732833	55832833	0.400666	NECTIN3
1	55733174	55833174	0.403201	NECTIN3
1	55733239	55833239	0.399992	NECTIN3
1	55733276	55833276	0.399992	NECTIN3
1	55733446	55833446	0.404491	NECTIN3
1	55733877	55833877	0.403872	NECTIN3
1	55733915	55833915	0.402118	NECTIN3
1	55733982	55833982	0.402118	NECTIN3
1	55734095	55834095	0.404191	NECTIN3
1	55734380	55834380	0.397696	NECTIN3
1	55734601	55834601	0.366203	NECTIN3
1	55734776	55834776	0.379079	NECTIN3
1	55734796	55834796	0.374552	NECTIN3
1	55735146	55835146	0.383423	NECTIN3
1	55735633	55835633	0.380959	NECTIN3
1	55735972	55835972	0.381899	NECTIN3
1	55736067	55836067	0.366135	NECTIN3
1	55736072	55836072	0.351247	NECTIN3
1	55736140	55836140	0.358635	NECTIN3
1	55736590	55836590	0.351522	NECTIN3
1	55737078	55837078	0.351522	NECTIN3
1	55737088	55837088	0.365344	NECTIN3
1	55737230	55837230	0.364144	NECTIN3
1	55737291	55837291	0.365344	NECTIN3
1	55737348	55837348	0.358521	NECTIN3
1	55737569	55837569	0.358521	NECTIN3
1	55737620	55837620	0.364041	NECTIN3
1	55737662	55837662	0.360311	NECTIN3
1	55737882	55837882	0.360311	NECTIN3
1	55737920	55837920	0.360311	NECTIN3
1	55738099	55838099	0.363227	NECTIN3
1	55738407	55838407	0.363227	NECTIN3
1	55738504	55838504	0.364792	NECTIN3
1	55738526	55838526	0.372046	NECTIN3
1	55738844	55838844	0.351305	NECTIN3
1	55738920	55838920	0.355832	NECTIN3
1	55739206	55839206	0.362839	NECTIN3
1	55739266	55839266	0.354836	NECTIN3
1	55739408	55839408	0.345587	NECTIN3
1	55739581	55839581	0.345587	NECTIN3
1	55739657	55839657	0.345587	NECTIN3
1	55739897	55839897	0.344966	NECTIN3
1	55740055	55840055	0.343255	NECTIN3
1	55740184	55840184	0.347943	NECTIN3
1	55741434	55841434	0.353059	NECTIN3
1	55741452	55841452	0.358561	NECTIN3
1	55741494	55841494	0.357214	NECTIN3
1	55741634	55841634	0.359148	NECTIN3
1	55741789	55841789	0.353423	NECTIN3
1	55741880	55841880	0.348967	NECTIN3
1	55741921	55841921	0.348967	NECTIN3
1	55741927	55841927	0.348967	NECTIN3
1	55742418	55842418	0.360908	NECTIN3
1	55742589	55842589	0.363594	NECTIN3
1	55742640	55842640	0.358542	NECTIN3

1	55742968	55842968	0.363068	NECTIN3
1	55742977	55842977	0.355315	NECTIN3
1	55743044	55843044	0.355039	NECTIN3
1	55743069	55843069	0.354794	NECTIN3
1	55743118	55843118	0.354926	NECTIN3
1	55743162	55843162	0.355384	NECTIN3
1	55743565	55843565	0.355384	NECTIN3
1	55743685	55843685	0.383334	NECTIN3
1	55743686	55843686	0.383334	NECTIN3
1	55743719	55843719	0.36874	NECTIN3
1	55743774	55843774	0.360618	NECTIN3
1	55743866	55843866	0.358074	NECTIN3
1	55744465	55844465	0.392291	NECTIN3
1	55744893	55844893	0.389272	NECTIN3
1	55744924	55844924	0.39195	NECTIN3
1	55745144	55845144	0.386989	NECTIN3
1	55745321	55845321	0.389264	NECTIN3
1	55745346	55845346	0.385901	NECTIN3
1	55745529	55845529	0.392103	NECTIN3
1	55745713	55845713	0.39912	NECTIN3
1	55745743	55845743	0.386511	NECTIN3
1	55745801	55845801	0.386865	NECTIN3
1	55745833	55845833	0.386035	NECTIN3
1	55745877	55845877	0.386035	NECTIN3
1	55746029	55846029	0.388946	NECTIN3
1	55746122	55846122	0.386942	NECTIN3
1	55746546	55846546	0.388145	NECTIN3
1	55746700	55846700	0.385209	NECTIN3
1	55746762	55846762	0.387119	NECTIN3
1	55746774	55846774	0.400323	NECTIN3
1	55747068	55847068	0.399338	NECTIN3
1	55747135	55847135	0.392456	NECTIN3
1	55747200	55847200	0.394857	NECTIN3
1	55747532	55847532	0.373131	NECTIN3
1	55747535	55847535	0.371413	NECTIN3
1	55747537	55847537	0.371633	NECTIN3
1	55747680	55847680	0.371633	NECTIN3
1	55747761	55847761	0.371186	NECTIN3
1	55748017	55848017	0.371376	NECTIN3
1	55748161	55848161	0.371376	NECTIN3
1	55748456	55848456	0.371376	NECTIN3
1	55748478	55848478	0.371376	NECTIN3
1	55748605	55848605	0.39371	NECTIN3
1	55748702	55848702	0.380459	NECTIN3
1	55749063	55849063	0.38813	NECTIN3
1	55749141	55849141	0.382398	NECTIN3
1	55749162	55849162	0.390867	NECTIN3
1	55749203	55849203	0.390572	NECTIN3
1	55749330	55849330	0.39075	NECTIN3
1	55749366	55849366	0.373951	NECTIN3
1	55749439	55849439	0.37517	NECTIN3
1	55749443	55849443	0.37517	NECTIN3
1	55749516	55849516	0.37517	NECTIN3
1	55749552	55849552	0.37517	NECTIN3
1	55749560	55849560	0.37517	NECTIN3
1	55749642	55849642	0.37517	NECTIN3
1	55749670	55849670	0.380866	NECTIN3
1	55749908	55849908	0.342049	NECTIN3

1	55750031	55850031	0.341684	NECTIN3
1	55750278	55850278	0.357505	NECTIN3
1	55750373	55850373	0.357387	NECTIN3
1	55753410	55853410	0.372194	NECTIN3
1	58563835	58663835	0.35204	DRD3
1	58901471	59001471	0.34888	ZBTB20
1	58902080	59002080	0.346438	ZBTB20
1	58902186	59002186	0.355796	ZBTB20
1	58902357	59002357	0.359992	ZBTB20
1	58902454	59002454	0.360718	ZBTB20
1	58902594	59002594	0.360884	ZBTB20
1	58902629	59002629	0.360864	ZBTB20
1	58902829	59002829	0.365613	ZBTB20
1	58902848	59002848	0.368736	ZBTB20
1	58903000	59003000	0.348828	ZBTB20
1	58903220	59003220	0.348828	ZBTB20
1	58903226	59003226	0.343648	ZBTB20
1	58903593	59003593	0.348066	ZBTB20
1	58903711	59003711	0.348045	ZBTB20
1	58903756	59003756	0.347911	ZBTB20
1	58903845	59003845	0.347616	ZBTB20
1	58904143	59004143	0.344102	ZBTB20
1	58905078	59005078	0.347562	ZBTB20
1	66007136	66107136	0.345305	EAF2
1	66007136	66107136	0.345305	LOC102184002
1	66007136	66107136	0.345305	LOC102184282
1	66007595	66107595	0.343813	EAF2
1	66007595	66107595	0.343813	LOC102184002
1	66007595	66107595	0.343813	LOC102184282
1	66007596	66107596	0.342802	EAF2
1	66007596	66107596	0.342802	LOC102184002
1	66007596	66107596	0.342802	LOC102184282
1	66007631	66107631	0.346368	EAF2
1	66007631	66107631	0.346368	LOC102184002
1	66007631	66107631	0.346368	LOC102184282
1	66007922	66107922	0.346368	EAF2
1	66007922	66107922	0.346368	LOC102184002
1	66007922	66107922	0.346368	LOC102184282
1	66008033	66108033	0.342695	EAF2
1	66008033	66108033	0.342695	LOC102184002
1	66008033	66108033	0.342695	LOC102184282
1	66008152	66108152	0.346932	EAF2
1	66008152	66108152	0.346932	LOC102184002
1	66008152	66108152	0.346932	LOC102184282
1	66008387	66108387	0.343765	EAF2
1	66008387	66108387	0.343765	LOC102184002
1	66008387	66108387	0.343765	LOC102184282
1	66008414	66108414	0.345783	EAF2
1	66008414	66108414	0.345783	LOC102184002
1	66008414	66108414	0.345783	LOC102184282
1	66008418	66108418	0.346715	EAF2
1	66008418	66108418	0.346715	LOC102184002
1	66008418	66108418	0.346715	LOC102184282
1	66011350	66111350	0.345867	EAF2
1	66011350	66111350	0.345867	LOC102184002
1	66011350	66111350	0.345867	LOC102184282
1	66011419	66111419	0.345867	EAF2
1	66011419	66111419	0.345867	LOC102184002

1	66011419	66111419	0.345867	LOC102184282
1	66011442	66111442	0.34632	EAF2
1	66011442	66111442	0.34632	LOC102184002
1	66011442	66111442	0.34632	LOC102184282
1	66011920	66111920	0.343111	EAF2
1	66011920	66111920	0.343111	LOC102184002
1	66011920	66111920	0.343111	LOC102184282
1	66013238	66113238	0.34565	EAF2
1	66013238	66113238	0.34565	LOC102184002
1	66013238	66113238	0.34565	LOC102184282
1	66013744	66113744	0.343013	EAF2
1	66013744	66113744	0.343013	LOC102184002
1	66013744	66113744	0.343013	LOC102184282
1	66014766	66114766	0.348586	EAF2
1	66014766	66114766	0.348586	LOC102184002
1	66014766	66114766	0.348586	LOC102184282
1	66015080	66115080	0.345299	EAF2
1	66015080	66115080	0.345299	LOC102184002
1	66015080	66115080	0.345299	LOC102184282
1	66015187	66115187	0.343973	EAF2
1	66015187	66115187	0.343973	LOC102184002
1	66015187	66115187	0.343973	LOC102184282
1	66017405	66117405	0.342894	LOC102184002
1	66017405	66117405	0.342894	LOC102184282
1	66017444	66117444	0.342764	LOC102184002
1	66017444	66117444	0.342764	LOC102184282
1	66017577	66117577	0.342373	LOC102184002
1	66017577	66117577	0.342373	LOC102184282
1	66017655	66117655	0.342884	LOC102184002
1	66017655	66117655	0.342884	LOC102184282
1	66017676	66117676	0.342884	LOC102184002
1	66017676	66117676	0.342884	LOC102184282
1	66017926	66117926	0.344619	LOC102184002
1	66017926	66117926	0.344619	LOC102184282
1	66017933	66117933	0.344216	LOC102184002
1	66017933	66117933	0.344216	LOC102184282
1	66018066	66118066	0.348617	LOC102184002
1	66018066	66118066	0.348617	LOC102184282
1	66018323	66118323	0.347445	LOC102184002
1	66018323	66118323	0.347445	LOC102184282
1	66018498	66118498	0.349604	LOC102184002
1	66018498	66118498	0.349604	LOC102184282
1	66020063	66120063	0.35428	LOC102184002
1	66020063	66120063	0.35428	LOC102184282
1	66020064	66120064	0.35428	LOC102184002
1	66020064	66120064	0.35428	LOC102184282
1	66020202	66120202	0.351433	LOC102184002
1	66020202	66120202	0.351433	LOC102184282
1	66020603	66120603	0.349746	LOC102184002
1	66020603	66120603	0.349746	LOC102184282
1	66020856	66120856	0.343285	LOC102184002
1	66020856	66120856	0.343285	LOC102184282
1	66020937	66120937	0.343301	LOC102184002
1	66020937	66120937	0.343301	LOC102184282
1	66021311	66121311	0.342867	LOC102184002
1	66021311	66121311	0.342867	LOC102184282
1	66021615	66121615	0.345034	LOC102184002
1	66021615	66121615	0.345034	LOC102184282

1	66021684	66121684	0.34256	LOC102184002
1	66021684	66121684	0.34256	LOC102184282
1	66021723	66121723	0.342617	LOC102184002
1	66021723	66121723	0.342617	LOC102184282
1	66021895	66121895	0.354623	LOC102184002
1	66021895	66121895	0.354623	LOC102184282
1	66021917	66121917	0.34832	LOC102184002
1	66021917	66121917	0.34832	LOC102184282
1	66022043	66122043	0.343787	LOC102184002
1	66022043	66122043	0.343787	LOC102184282
1	66022709	66122709	0.342749	LOC102184002
1	66022709	66122709	0.342749	LOC102184282
1	66022841	66122841	0.344396	LOC102184002
1	66022841	66122841	0.344396	LOC102184282
1	66023071	66123071	0.343907	LOC102184002
1	66023071	66123071	0.343907	LOC102184282
1	66023072	66123072	0.343907	LOC102184002
1	66023072	66123072	0.343907	LOC102184282
1	66023316	66123316	0.343907	LOC102184002
1	66023316	66123316	0.343907	LOC102184282
1	66023360	66123360	0.350221	LOC102184002
1	66023360	66123360	0.350221	LOC102184282
1	66023452	66123452	0.349616	LOC102184002
1	66023452	66123452	0.349616	LOC102184282
1	66023460	66123460	0.349616	LOC102184002
1	66023460	66123460	0.349616	LOC102184282
1	66023615	66123615	0.349801	LOC102184002
1	66023615	66123615	0.349801	LOC102184282
1	66023728	66123728	0.347055	LOC102184002
1	66023728	66123728	0.347055	LOC102184282
1	66024135	66124135	0.351819	LOC102184002
1	66024135	66124135	0.351819	LOC102184282
1	66024171	66124171	0.351489	LOC102184002
1	66024171	66124171	0.351489	LOC102184282
1	66024190	66124190	0.351489	LOC102184002
1	66024190	66124190	0.351489	LOC102184282
1	66024431	66124431	0.351362	LOC102184002
1	66024431	66124431	0.351362	LOC102184282
1	66024479	66124479	0.354465	LOC102184002
1	66024479	66124479	0.354465	LOC102184282
1	66024586	66124586	0.354538	LOC102184002
1	66024586	66124586	0.354538	LOC102184282
1	66024595	66124595	0.351108	LOC102184002
1	66024595	66124595	0.351108	LOC102184282
1	66024861	66124861	0.350962	LOC102184002
1	66024861	66124861	0.350962	LOC102184282
1	66025172	66125172	0.351085	LOC102184002
1	66025172	66125172	0.351085	LOC102184282
1	66025195	66125195	0.351085	LOC102184002
1	66025195	66125195	0.351085	LOC102184282
1	66025332	66125332	0.355176	LOC102184002
1	66025332	66125332	0.355176	LOC102184282
1	66025513	66125513	0.354015	LOC102184002
1	66025513	66125513	0.354015	LOC102184282
1	66025540	66125540	0.353689	LOC102184002
1	66025540	66125540	0.353689	LOC102184282
1	66025555	66125555	0.357442	LOC102184002
1	66025555	66125555	0.357442	LOC102184282

1	66025647	66125647	0.350278	LOC102184002
1	66025647	66125647	0.350278	LOC102184282
1	66025764	66125764	0.356189	LOC102184002
1	66025764	66125764	0.356189	LOC102184282
1	66025934	66125934	0.350498	LOC102184002
1	66025934	66125934	0.350498	LOC102184282
1	66026097	66126097	0.349852	LOC102184002
1	66026097	66126097	0.349852	LOC102184282
1	66026103	66126103	0.350763	LOC102184002
1	66026103	66126103	0.350763	LOC102184282
1	66026214	66126214	0.350763	LOC102184002
1	66026214	66126214	0.350763	LOC102184282
1	66026229	66126229	0.350763	LOC102184002
1	66026229	66126229	0.350763	LOC102184282
1	66026347	66126347	0.350763	LOC102184002
1	66026347	66126347	0.350763	LOC102184282
1	66026409	66126409	0.353282	LOC102184002
1	66026409	66126409	0.353282	LOC102184282
1	66026492	66126492	0.349798	LOC102184002
1	66026492	66126492	0.349798	LOC102184282
1	66026518	66126518	0.347496	LOC102184002
1	66026518	66126518	0.347496	LOC102184282
1	66026684	66126684	0.349575	LOC102184002
1	66026684	66126684	0.349575	LOC102184282
1	66027082	66127082	0.347614	LOC102184002
1	66027082	66127082	0.347614	LOC102184282
1	66027216	66127216	0.356516	LOC102184002
1	66027216	66127216	0.356516	LOC102184282
1	66027242	66127242	0.342511	LOC102184002
1	66027242	66127242	0.342511	LOC102184282
1	66027244	66127244	0.342511	LOC102184002
1	66027244	66127244	0.342511	LOC102184282
1	66027245	66127245	0.342511	LOC102184002
1	66027245	66127245	0.342511	LOC102184282
1	66027522	66127522	0.358826	LOC102184002
1	66027522	66127522	0.358826	LOC102184282
1	66027577	66127577	0.357641	LOC102184002
1	66027577	66127577	0.357641	LOC102184282
1	66027729	66127729	0.358337	LOC102184002
1	66027729	66127729	0.358337	LOC102184282
1	66027999	66127999	0.361547	LOC102184002
1	66027999	66127999	0.361547	LOC102184282
1	66028190	66128190	0.359166	LOC102184002
1	66028190	66128190	0.359166	LOC102184282
1	66028468	66128468	0.359166	LOC102184002
1	66028468	66128468	0.359166	LOC102184282
1	66028632	66128632	0.359166	LOC102184002
1	66028632	66128632	0.359166	LOC102184282
1	66028783	66128783	0.359166	LOC102184002
1	66028783	66128783	0.359166	LOC102184282
1	66028845	66128845	0.359166	LOC102184002
1	66028845	66128845	0.359166	LOC102184282
1	66028846	66128846	0.359166	LOC102184002
1	66028846	66128846	0.359166	LOC102184282
1	66029014	66129014	0.361038	LOC102184002
1	66029014	66129014	0.361038	LOC102184282
1	66029455	66129455	0.359006	LOC102184002
1	66029455	66129455	0.359006	LOC102184282

1	66029710	66129710	0.359006	LOC102184002
1	66029710	66129710	0.359006	LOC102184282
1	66029935	66129935	0.363529	LOC102184002
1	66029935	66129935	0.363529	LOC102184282
1	66029958	66129958	0.365355	LOC102184002
1	66029958	66129958	0.365355	LOC102184282
1	66029998	66129998	0.365072	LOC102184002
1	66029998	66129998	0.365072	LOC102184282
1	66030033	66130033	0.375406	LOC102184002
1	66030033	66130033	0.375406	LOC102184282
1	66030119	66130119	0.372024	LOC102184002
1	66030119	66130119	0.372024	LOC102184282
1	66030239	66130239	0.377998	LOC102184002
1	66030239	66130239	0.377998	LOC102184282
1	66030407	66130407	0.37924	LOC102184002
1	66030407	66130407	0.37924	LOC102184282
1	66030469	66130469	0.377853	LOC102184002
1	66030469	66130469	0.377853	LOC102184282
1	66030539	66130539	0.377227	LOC102184002
1	66030539	66130539	0.377227	LOC102184282
1	66030670	66130670	0.382743	LOC102184002
1	66030670	66130670	0.382743	LOC102184282
1	66030753	66130753	0.376857	LOC102184002
1	66030753	66130753	0.376857	LOC102184282
1	66031023	66131023	0.377491	LOC102184002
1	66031023	66131023	0.377491	LOC102184282
1	66031084	66131084	0.377023	LOC102184002
1	66031084	66131084	0.377023	LOC102184282
1	66031138	66131138	0.377023	LOC102184002
1	66031138	66131138	0.377023	LOC102184282
1	66031198	66131198	0.378522	LOC102184002
1	66031198	66131198	0.378522	LOC102184282
1	66031203	66131203	0.377212	LOC102184002
1	66031203	66131203	0.377212	LOC102184282
1	66031408	66131408	0.377346	LOC102184002
1	66031408	66131408	0.377346	LOC102184282
1	66031464	66131464	0.37771	LOC102184002
1	66031464	66131464	0.37771	LOC102184282
1	66031515	66131515	0.377437	LOC102184002
1	66031515	66131515	0.377437	LOC102184282
1	66031533	66131533	0.3795	LOC102184002
1	66031533	66131533	0.3795	LOC102184282
1	66031542	66131542	0.367684	LOC102184002
1	66031542	66131542	0.367684	LOC102184282
1	66031565	66131565	0.385218	LOC102184002
1	66031565	66131565	0.385218	LOC102184282
1	66031647	66131647	0.381904	LOC102184002
1	66031647	66131647	0.381904	LOC102184282
1	66031689	66131689	0.382281	LOC102184002
1	66031689	66131689	0.382281	LOC102184282
1	66031761	66131761	0.382405	LOC102184002
1	66031761	66131761	0.382405	LOC102184282
1	66031786	66131786	0.383514	LOC102184002
1	66031786	66131786	0.383514	LOC102184282
1	66031937	66131937	0.383885	LOC102184002
1	66031937	66131937	0.383885	LOC102184282
1	66032153	66132153	0.382534	LOC102184002
1	66032153	66132153	0.382534	LOC102184282

1	66032162	66132162	0.384364	LOC102184002
1	66032162	66132162	0.384364	LOC102184282
1	66032212	66132212	0.383629	LOC102184002
1	66032212	66132212	0.383629	LOC102184282
1	66032371	66132371	0.38365	LOC102184002
1	66032371	66132371	0.38365	LOC102184282
1	66032392	66132392	0.384038	LOC102184002
1	66032392	66132392	0.384038	LOC102184282
1	66032550	66132550	0.387088	LOC102184002
1	66032550	66132550	0.387088	LOC102184282
1	66032569	66132569	0.381541	LOC102184002
1	66032569	66132569	0.381541	LOC102184282
1	66032621	66132621	0.381163	LOC102184002
1	66032621	66132621	0.381163	LOC102184282
1	66032707	66132707	0.381518	LOC102184002
1	66032707	66132707	0.381518	LOC102184282
1	66032719	66132719	0.375406	LOC102184002
1	66032719	66132719	0.375406	LOC102184282
1	66032773	66132773	0.374529	LOC102184002
1	66032773	66132773	0.374529	LOC102184282
1	66032871	66132871	0.372403	LOC102184002
1	66032871	66132871	0.372403	LOC102184282
1	66032954	66132954	0.370289	LOC102184002
1	66032954	66132954	0.370289	LOC102184282
1	66033091	66133091	0.366153	LOC102184002
1	66033091	66133091	0.366153	LOC102184282
1	66033181	66133181	0.36735	LOC102184002
1	66033181	66133181	0.36735	LOC102184282
1	66033183	66133183	0.36735	LOC102184002
1	66033183	66133183	0.36735	LOC102184282
1	66033244	66133244	0.369084	LOC102184002
1	66033244	66133244	0.369084	LOC102184282
1	66033301	66133301	0.368335	LOC102184002
1	66033301	66133301	0.368335	LOC102184282
1	66033340	66133340	0.368335	LOC102184002
1	66033340	66133340	0.368335	LOC102184282
1	66033473	66133473	0.370393	LOC102184002
1	66033473	66133473	0.370393	LOC102184282
1	66033475	66133475	0.371441	LOC102184002
1	66033475	66133475	0.371441	LOC102184282
1	66033482	66133482	0.369308	LOC102184002
1	66033482	66133482	0.369308	LOC102184282
1	66033834	66133834	0.369432	LOC102184002
1	66033834	66133834	0.369432	LOC102184282
1	66033864	66133864	0.361505	LOC102184002
1	66033864	66133864	0.361505	LOC102184282
1	66033871	66133871	0.363319	LOC102184002
1	66033871	66133871	0.363319	LOC102184282
1	66033907	66133907	0.356982	LOC102184002
1	66033907	66133907	0.356982	LOC102184282
1	66033953	66133953	0.356982	LOC102184002
1	66033953	66133953	0.356982	LOC102184282
1	66034584	66134584	0.365618	LOC102184002
1	66034584	66134584	0.365618	LOC102184282
1	66034716	66134716	0.365618	LOC102184002
1	66034716	66134716	0.365618	LOC102184282
1	66035359	66135359	0.372165	LOC102184002
1	66035359	66135359	0.372165	LOC102184282

1	66035482	66135482	0.371819	LOC102184002
1	66035482	66135482	0.371819	LOC102184282
1	66035630	66135630	0.366855	LOC102184002
1	66035630	66135630	0.366855	LOC102184282
1	66035712	66135712	0.364524	LOC102184002
1	66035712	66135712	0.364524	LOC102184282
1	66035747	66135747	0.3658	LOC102184002
1	66035747	66135747	0.3658	LOC102184282
1	66035830	66135830	0.361887	LOC102184002
1	66035830	66135830	0.361887	LOC102184282
1	66036100	66136100	0.361576	LOC102184002
1	66036100	66136100	0.361576	LOC102184282
1	66036122	66136122	0.361962	LOC102184002
1	66036122	66136122	0.361962	LOC102184282
1	66036458	66136458	0.360458	LOC102184002
1	66036458	66136458	0.360458	LOC102184282
1	66036849	66136849	0.361583	LOC102184002
1	66036849	66136849	0.361583	LOC102184282
1	66036906	66136906	0.362267	LOC102184002
1	66036906	66136906	0.362267	LOC102184282
1	66037216	66137216	0.36162	LOC102184002
1	66037216	66137216	0.36162	LOC102184282
1	66037387	66137387	0.363144	LOC102184002
1	66037387	66137387	0.363144	LOC102184282
1	66037771	66137771	0.361567	LOC102184002
1	66037771	66137771	0.361567	LOC102184282
1	66037869	66137869	0.354008	LOC102184002
1	66037869	66137869	0.354008	LOC102184282
1	66038184	66138184	0.353927	LOC102184002
1	66038184	66138184	0.353927	LOC102184282
1	66038209	66138209	0.35036	LOC102184002
1	66038209	66138209	0.35036	LOC102184282
1	66038408	66138408	0.346268	LOC102184002
1	66038408	66138408	0.346268	LOC102184282
1	66038927	66138927	0.347499	LOC102184002
1	66038927	66138927	0.347499	LOC102184282
1	66039026	66139026	0.34682	LOC102184002
1	66039026	66139026	0.34682	LOC102184282
1	66039313	66139313	0.345546	LOC102184002
1	66039313	66139313	0.345546	LOC102184282
1	66039912	66139912	0.342859	LOC102184002
1	66039912	66139912	0.342859	LOC102184282
1	66040337	66140337	0.345623	LOC102184002
1	66040337	66140337	0.345623	ILDR1
1	66040337	66140337	0.345623	LOC102184282
1	66040599	66140599	0.356155	LOC102184002
1	66040599	66140599	0.356155	ILDR1
1	66040599	66140599	0.356155	LOC102184282
1	66040987	66140987	0.358465	LOC102184002
1	66040987	66140987	0.358465	ILDR1
1	66040987	66140987	0.358465	LOC102184282
1	66041014	66141014	0.36127	LOC102184002
1	66041014	66141014	0.36127	ILDR1
1	66041014	66141014	0.36127	LOC102184282
1	66041021	66141021	0.364175	LOC102184002
1	66041021	66141021	0.364175	ILDR1
1	66041021	66141021	0.364175	LOC102184282
1	66041047	66141047	0.364175	LOC102184002

1	66041047	66141047	0.364175	ILDR1
1	66041047	66141047	0.364175	LOC102184282
1	66041115	66141115	0.360832	LOC102184002
1	66041115	66141115	0.360832	ILDR1
1	66041115	66141115	0.360832	LOC102184282
1	66041493	66141493	0.36271	LOC102184002
1	66041493	66141493	0.36271	ILDR1
1	66041493	66141493	0.36271	LOC102184282
1	66041546	66141546	0.360499	LOC102184002
1	66041546	66141546	0.360499	ILDR1
1	66041546	66141546	0.360499	LOC102184282
1	66041671	66141671	0.40384	LOC102184002
1	66041671	66141671	0.40384	ILDR1
1	66041671	66141671	0.40384	LOC102184282
1	66041948	66141948	0.389795	LOC102184002
1	66041948	66141948	0.389795	ILDR1
1	66041948	66141948	0.389795	LOC102184282
1	66041958	66141958	0.38971	LOC102184002
1	66041958	66141958	0.38971	ILDR1
1	66041958	66141958	0.38971	LOC102184282
1	66042065	66142065	0.386911	LOC102184002
1	66042065	66142065	0.386911	ILDR1
1	66042065	66142065	0.386911	LOC102184282
1	66042226	66142226	0.386383	LOC102184002
1	66042226	66142226	0.386383	ILDR1
1	66042226	66142226	0.386383	LOC102184282
1	66042269	66142269	0.393166	LOC102184002
1	66042269	66142269	0.393166	ILDR1
1	66042269	66142269	0.393166	LOC102184282
1	66042296	66142296	0.387337	LOC102184002
1	66042296	66142296	0.387337	ILDR1
1	66042296	66142296	0.387337	LOC102184282
1	66042413	66142413	0.387309	LOC102184002
1	66042413	66142413	0.387309	ILDR1
1	66042413	66142413	0.387309	LOC102184282
1	66042439	66142439	0.388174	LOC102184002
1	66042439	66142439	0.388174	ILDR1
1	66042439	66142439	0.388174	LOC102184282
1	66042582	66142582	0.388174	LOC102184002
1	66042582	66142582	0.388174	ILDR1
1	66042582	66142582	0.388174	LOC102184282
1	66042777	66142777	0.388174	LOC102184002
1	66042777	66142777	0.388174	ILDR1
1	66042777	66142777	0.388174	LOC102184282
1	66043029	66143029	0.393027	LOC102184002
1	66043029	66143029	0.393027	ILDR1
1	66043029	66143029	0.393027	LOC102184282
1	66043206	66143206	0.388241	LOC102184002
1	66043206	66143206	0.388241	ILDR1
1	66043206	66143206	0.388241	LOC102184282
1	66043403	66143403	0.388241	LOC102184002
1	66043403	66143403	0.388241	ILDR1
1	66043403	66143403	0.388241	LOC102184282
1	66043639	66143639	0.388241	LOC102184002
1	66043639	66143639	0.388241	ILDR1
1	66043639	66143639	0.388241	LOC102184282
1	66043661	66143661	0.393503	LOC102184002
1	66043661	66143661	0.393503	ILDR1

1	66043661	66143661	0.393503	LOC102184282
1	66043662	66143662	0.395504	LOC102184002
1	66043662	66143662	0.395504	ILDR1
1	66043662	66143662	0.395504	LOC102184282
1	66043840	66143840	0.393534	LOC102184002
1	66043840	66143840	0.393534	ILDR1
1	66043840	66143840	0.393534	LOC102184282
1	66043962	66143962	0.397723	LOC102184002
1	66043962	66143962	0.397723	ILDR1
1	66043962	66143962	0.397723	LOC102184282
1	66043972	66143972	0.394912	LOC102184002
1	66043972	66143972	0.394912	ILDR1
1	66043972	66143972	0.394912	LOC102184282
1	66044253	66144253	0.414686	LOC102184002
1	66044253	66144253	0.414686	ILDR1
1	66044253	66144253	0.414686	LOC102184282
1	66044344	66144344	0.411025	LOC102184002
1	66044344	66144344	0.411025	ILDR1
1	66044344	66144344	0.411025	LOC102184282
1	66044432	66144432	0.407276	LOC102184002
1	66044432	66144432	0.407276	ILDR1
1	66044432	66144432	0.407276	LOC102184282
1	66044520	66144520	0.408308	LOC102184002
1	66044520	66144520	0.408308	ILDR1
1	66044520	66144520	0.408308	LOC102184282
1	66044580	66144580	0.408859	LOC102184002
1	66044580	66144580	0.408859	ILDR1
1	66044580	66144580	0.408859	LOC102184282
1	66044585	66144585	0.409163	LOC102184002
1	66044585	66144585	0.409163	ILDR1
1	66044585	66144585	0.409163	LOC102184282
1	66044604	66144604	0.408821	LOC102184002
1	66044604	66144604	0.408821	ILDR1
1	66044604	66144604	0.408821	LOC102184282
1	66044633	66144633	0.409803	LOC102184002
1	66044633	66144633	0.409803	ILDR1
1	66044633	66144633	0.409803	LOC102184282
1	66044734	66144734	0.410642	LOC102184002
1	66044734	66144734	0.410642	ILDR1
1	66044734	66144734	0.410642	LOC102184282
1	66045188	66145188	0.409858	LOC102184002
1	66045188	66145188	0.409858	ILDR1
1	66045188	66145188	0.409858	LOC102184282
1	66045291	66145291	0.400574	LOC102184002
1	66045291	66145291	0.400574	ILDR1
1	66045291	66145291	0.400574	LOC102184282
1	66045409	66145409	0.425329	LOC102184002
1	66045409	66145409	0.425329	ILDR1
1	66045409	66145409	0.425329	LOC102184282
1	66045564	66145564	0.42184	LOC102184002
1	66045564	66145564	0.42184	ILDR1
1	66045564	66145564	0.42184	LOC102184282
1	66045750	66145750	0.424189	LOC102184002
1	66045750	66145750	0.424189	ILDR1
1	66045750	66145750	0.424189	LOC102184282
1	66045982	66145982	0.423233	LOC102184002
1	66045982	66145982	0.423233	ILDR1
1	66045982	66145982	0.423233	LOC102184282

1	66046085	66146085	0.417999	LOC102184002
1	66046085	66146085	0.417999	ILDR1
1	66046085	66146085	0.417999	LOC102184282
1	66046294	66146294	0.419788	LOC102184002
1	66046294	66146294	0.419788	ILDR1
1	66046294	66146294	0.419788	LOC102184282
1	66046342	66146342	0.419788	LOC102184002
1	66046342	66146342	0.419788	ILDR1
1	66046342	66146342	0.419788	LOC102184282
1	66046417	66146417	0.418268	LOC102184002
1	66046417	66146417	0.418268	ILDR1
1	66046417	66146417	0.418268	LOC102184282
1	66046684	66146684	0.433752	LOC102184002
1	66046684	66146684	0.433752	ILDR1
1	66046684	66146684	0.433752	LOC102184282
1	66046756	66146756	0.448052	LOC102184002
1	66046756	66146756	0.448052	ILDR1
1	66046756	66146756	0.448052	LOC102184282
1	66046790	66146790	0.464383	LOC102184002
1	66046790	66146790	0.464383	ILDR1
1	66046790	66146790	0.464383	LOC102184282
1	66046798	66146798	0.416628	LOC102184002
1	66046798	66146798	0.416628	ILDR1
1	66046798	66146798	0.416628	LOC102184282
1	66047032	66147032	0.37533	LOC102184002
1	66047032	66147032	0.37533	ILDR1
1	66047032	66147032	0.37533	LOC102184282
1	66047111	66147111	0.37533	LOC102184002
1	66047111	66147111	0.37533	ILDR1
1	66047111	66147111	0.37533	LOC102184282
1	66047708	66147708	0.373518	LOC102184002
1	66047708	66147708	0.373518	ILDR1
1	66047708	66147708	0.373518	LOC102184282
1	66047937	66147937	0.372604	LOC102184002
1	66047937	66147937	0.372604	ILDR1
1	66047937	66147937	0.372604	LOC102184282
1	66048191	66148191	0.373324	LOC102184002
1	66048191	66148191	0.373324	ILDR1
1	66048191	66148191	0.373324	LOC102184282
1	66048323	66148323	0.373324	LOC102184002
1	66048323	66148323	0.373324	ILDR1
1	66048323	66148323	0.373324	LOC102184282
1	66048340	66148340	0.374002	LOC102184002
1	66048340	66148340	0.374002	ILDR1
1	66048340	66148340	0.374002	LOC102184282
1	66048602	66148602	0.369795	LOC102184002
1	66048602	66148602	0.369795	ILDR1
1	66048602	66148602	0.369795	LOC102184282
1	66048731	66148731	0.37505	LOC102184002
1	66048731	66148731	0.37505	ILDR1
1	66048731	66148731	0.37505	LOC102184282
1	66048761	66148761	0.376346	LOC102184002
1	66048761	66148761	0.376346	ILDR1
1	66048761	66148761	0.376346	LOC102184282
1	66048820	66148820	0.378793	LOC102184002
1	66048820	66148820	0.378793	ILDR1
1	66048820	66148820	0.378793	LOC102184282
1	66049066	66149066	0.349878	LOC102184002

1	66049066	66149066	0.349878	ILDR1
1	66049066	66149066	0.349878	LOC102184282
1	66049193	66149193	0.37674	LOC102184002
1	66049193	66149193	0.37674	ILDR1
1	66049193	66149193	0.37674	LOC102184282
1	66049251	66149251	0.36069	LOC102184002
1	66049251	66149251	0.36069	ILDR1
1	66049251	66149251	0.36069	LOC102184282
1	66049357	66149357	0.381389	LOC102184002
1	66049357	66149357	0.381389	ILDR1
1	66049357	66149357	0.381389	LOC102184282
1	66049411	66149411	0.381719	LOC102184002
1	66049411	66149411	0.381719	ILDR1
1	66049411	66149411	0.381719	LOC102184282
1	66049419	66149419	0.383316	LOC102184002
1	66049419	66149419	0.383316	ILDR1
1	66049419	66149419	0.383316	LOC102184282
1	66049522	66149522	0.381639	LOC102184002
1	66049522	66149522	0.381639	ILDR1
1	66049522	66149522	0.381639	LOC102184282
1	66049666	66149666	0.379649	LOC102184002
1	66049666	66149666	0.379649	ILDR1
1	66049666	66149666	0.379649	LOC102184282
1	66049889	66149889	0.382045	LOC102184002
1	66049889	66149889	0.382045	ILDR1
1	66049889	66149889	0.382045	LOC102184282
1	66049942	66149942	0.379482	LOC102184002
1	66049942	66149942	0.379482	ILDR1
1	66049942	66149942	0.379482	LOC102184282
1	66050089	66150089	0.378921	LOC102184002
1	66050089	66150089	0.378921	ILDR1
1	66050089	66150089	0.378921	LOC102184282
1	66050096	66150096	0.378921	LOC102184002
1	66050096	66150096	0.378921	ILDR1
1	66050096	66150096	0.378921	LOC102184282
1	66050171	66150171	0.378921	LOC102184002
1	66050171	66150171	0.378921	ILDR1
1	66050171	66150171	0.378921	LOC102184282
1	66050270	66150270	0.376476	LOC102184002
1	66050270	66150270	0.376476	ILDR1
1	66050270	66150270	0.376476	LOC102184282
1	66050369	66150369	0.369077	LOC102184002
1	66050369	66150369	0.369077	ILDR1
1	66050369	66150369	0.369077	LOC102184282
1	66050492	66150492	0.3703	LOC102184002
1	66050492	66150492	0.3703	ILDR1
1	66050492	66150492	0.3703	LOC102184282
1	66050530	66150530	0.379773	LOC102184002
1	66050530	66150530	0.379773	ILDR1
1	66050530	66150530	0.379773	LOC102184282
1	66050621	66150621	0.352812	LOC102184002
1	66050621	66150621	0.352812	ILDR1
1	66050621	66150621	0.352812	LOC102184282
1	66050690	66150690	0.349078	LOC102184002
1	66050690	66150690	0.349078	ILDR1
1	66050690	66150690	0.349078	LOC102184282
1	66051979	66151979	0.353701	LOC102184002
1	66051979	66151979	0.353701	ILDR1

1	66051979	66151979	0.353701	LOC102184282
1	66052110	66152110	0.353149	LOC102184002
1	66052110	66152110	0.353149	ILDR1
1	66052110	66152110	0.353149	LOC102184282
1	66052180	66152180	0.355365	LOC102184002
1	66052180	66152180	0.355365	ILDR1
1	66052180	66152180	0.355365	LOC102184282
1	66052572	66152572	0.354629	LOC102184002
1	66052572	66152572	0.354629	ILDR1
1	66052572	66152572	0.354629	LOC102184282
1	66052690	66152690	0.353425	LOC102184002
1	66052690	66152690	0.353425	ILDR1
1	66052690	66152690	0.353425	LOC102184282
1	66053181	66153181	0.353421	LOC102184002
1	66053181	66153181	0.353421	ILDR1
1	66053181	66153181	0.353421	LOC102184282
1	66053254	66153254	0.353297	LOC102184002
1	66053254	66153254	0.353297	ILDR1
1	66053254	66153254	0.353297	LOC102184282
1	66053515	66153515	0.362266	LOC102184002
1	66053515	66153515	0.362266	ILDR1
1	66053515	66153515	0.362266	LOC102184282
1	66053534	66153534	0.362025	LOC102184002
1	66053534	66153534	0.362025	ILDR1
1	66053534	66153534	0.362025	LOC102184282
1	66053614	66153614	0.365585	LOC102184002
1	66053614	66153614	0.365585	ILDR1
1	66053614	66153614	0.365585	LOC102184282
1	66053615	66153615	0.371259	LOC102184002
1	66053615	66153615	0.371259	ILDR1
1	66053615	66153615	0.371259	LOC102184282
1	66053815	66153815	0.3735	LOC102184002
1	66053815	66153815	0.3735	ILDR1
1	66053815	66153815	0.3735	LOC102184282
1	66053821	66153821	0.3735	LOC102184002
1	66053821	66153821	0.3735	ILDR1
1	66053821	66153821	0.3735	LOC102184282
1	66053948	66153948	0.371028	LOC102184002
1	66053948	66153948	0.371028	ILDR1
1	66053948	66153948	0.371028	LOC102184282
1	66054243	66154243	0.36546	LOC102184002
1	66054243	66154243	0.36546	ILDR1
1	66054243	66154243	0.36546	LOC102184282
1	66055058	66155058	0.366085	LOC102184002
1	66055058	66155058	0.366085	ILDR1
1	66055058	66155058	0.366085	LOC102184282
1	66055221	66155221	0.366493	LOC102184002
1	66055221	66155221	0.366493	ILDR1
1	66055221	66155221	0.366493	LOC102184282
1	66055296	66155296	0.365232	LOC102184002
1	66055296	66155296	0.365232	ILDR1
1	66055296	66155296	0.365232	LOC102184282
1	66055558	66155558	0.352711	LOC102184002
1	66055558	66155558	0.352711	ILDR1
1	66055558	66155558	0.352711	LOC102184282
1	66056420	66156420	0.344233	LOC102184002
1	66056420	66156420	0.344233	ILDR1
1	66056420	66156420	0.344233	LOC102184282

1	69507817	69607817	0.343644	ZNF148
1	69508874	69608874	0.362739	ZNF148
1	69509294	69609294	0.365247	ZNF148
1	69509438	69609438	0.34471	ZNF148
1	69509823	69609823	0.356664	ZNF148
1	69510021	69610021	0.385583	ZNF148
1	69510082	69610082	0.354735	ZNF148
1	69510103	69610103	0.357382	ZNF148
1	69510296	69610296	0.365025	ZNF148
1	69510447	69610447	0.360027	ZNF148
1	69510507	69610507	0.361514	ZNF148
1	69510684	69610684	0.378564	ZNF148
1	69511168	69611168	0.36333	ZNF148
1	69511485	69611485	0.375269	ZNF148
1	69511534	69611534	0.36257	ZNF148
1	69511650	69611650	0.36326	ZNF148
1	69511907	69611907	0.370533	ZNF148
1	69511912	69611912	0.373212	ZNF148
1	69512381	69612381	0.372948	ZNF148
1	69512388	69612388	0.381588	ZNF148
1	69512395	69612395	0.376349	ZNF148
1	69512684	69612684	0.373379	ZNF148
1	69512685	69612685	0.375856	ZNF148
1	69512686	69612686	0.375856	ZNF148
1	69512823	69612823	0.376876	ZNF148
1	69512906	69612906	0.360907	ZNF148
1	69512929	69612929	0.361043	ZNF148
1	69513062	69613062	0.372288	ZNF148
1	69513100	69613100	0.378958	ZNF148
1	69513111	69613111	0.358277	ZNF148
1	69513717	69613717	0.353326	ZNF148
1	69513745	69613745	0.359064	ZNF148
1	69513796	69613796	0.359064	ZNF148
1	69513943	69613943	0.365818	ZNF148
1	69513944	69613944	0.365818	ZNF148
1	69514231	69614231	0.370785	ZNF148
1	69514417	69614417	0.376951	ZNF148
1	69514589	69614589	0.36681	ZNF148
1	69514676	69614676	0.36681	ZNF148
1	69514949	69614949	0.374816	ZNF148
1	69515208	69615208	0.369126	ZNF148
1	69515254	69615254	0.374944	ZNF148
1	69515355	69615355	0.349502	ZNF148
1	69515495	69615495	0.364357	ZNF148
1	69516213	69616213	0.3493	ZNF148
1	69528624	69628624	0.344955	ZNF148
1	69549774	69649774	0.343389	ZNF148
1	69550779	69650779	0.351593	ZNF148
1	69550839	69650839	0.346483	ZNF148
1	69551869	69651869	0.358585	ZNF148
1	69551925	69651925	0.369684	ZNF148
1	69552036	69652036	0.381141	ZNF148
1	69552092	69652092	0.379307	ZNF148
1	69552100	69652100	0.377751	ZNF148
1	69552124	69652124	0.377803	ZNF148
1	69552125	69652125	0.37779	ZNF148
1	69552159	69652159	0.374551	ZNF148
1	69552189	69652189	0.396712	ZNF148

1	69552197	69652197	0.399438	ZNF148
1	69552231	69652231	0.402988	ZNF148
1	69552253	69652253	0.456367	ZNF148
1	69552287	69652287	0.438005	ZNF148
1	69574606	69674606	0.38596	SNX4
1	69574606	69674606	0.38596	ZNF148
1	69574649	69674649	0.384511	SNX4
1	69574649	69674649	0.384511	ZNF148
1	69574737	69674737	0.374201	SNX4
1	69574737	69674737	0.374201	ZNF148
1	69574973	69674973	0.377266	SNX4
1	69574973	69674973	0.377266	ZNF148
1	69575123	69675123	0.370369	SNX4
1	69575123	69675123	0.370369	ZNF148
1	69575151	69675151	0.450689	SNX4
1	69575151	69675151	0.450689	ZNF148
1	69575232	69675232	0.427042	SNX4
1	69575232	69675232	0.427042	ZNF148
1	69575288	69675288	0.429191	SNX4
1	69575288	69675288	0.429191	ZNF148
1	69575786	69675786	0.426124	SNX4
1	69575786	69675786	0.426124	ZNF148
1	69575885	69675885	0.427777	SNX4
1	69575885	69675885	0.427777	ZNF148
1	69576053	69676053	0.431053	SNX4
1	69576053	69676053	0.431053	ZNF148
1	69576283	69676283	0.435094	SNX4
1	69576283	69676283	0.435094	ZNF148
1	69576293	69676293	0.434198	SNX4
1	69576293	69676293	0.434198	ZNF148
1	69576367	69676367	0.427726	SNX4
1	69576367	69676367	0.427726	ZNF148
1	69577124	69677124	0.423028	SNX4
1	69577124	69677124	0.423028	ZNF148
1	69577218	69677218	0.415228	SNX4
1	69577218	69677218	0.415228	ZNF148
1	69577245	69677245	0.416388	SNX4
1	69577245	69677245	0.416388	ZNF148
1	69577485	69677485	0.400574	SNX4
1	69577485	69677485	0.400574	ZNF148
1	69577944	69677944	0.395013	SNX4
1	69577944	69677944	0.395013	ZNF148
1	69578429	69678429	0.395159	SNX4
1	69578429	69678429	0.395159	ZNF148
1	69579554	69679554	0.395159	SNX4
1	69579554	69679554	0.395159	ZNF148
1	69579586	69679586	0.401807	SNX4
1	69579586	69679586	0.401807	ZNF148
1	69580032	69680032	0.400542	SNX4
1	69580032	69680032	0.400542	ZNF148
1	69580850	69680850	0.416594	SNX4
1	69580850	69680850	0.416594	ZNF148
1	69580885	69680885	0.420039	SNX4
1	69580885	69680885	0.420039	ZNF148
1	69580906	69680906	0.397956	SNX4
1	69580906	69680906	0.397956	ZNF148
1	69582227	69682227	0.394398	SNX4
1	69582227	69682227	0.394398	ZNF148

1	69582520	69682520	0.404259	SNX4
1	69582520	69682520	0.404259	ZNF148
1	69582600	69682600	0.403976	SNX4
1	69582600	69682600	0.403976	ZNF148
1	69582634	69682634	0.403666	SNX4
1	69582634	69682634	0.403666	ZNF148
1	69582643	69682643	0.403666	SNX4
1	69582643	69682643	0.403666	ZNF148
1	69582651	69682651	0.422711	SNX4
1	69582651	69682651	0.422711	ZNF148
1	69582652	69682652	0.423087	SNX4
1	69582652	69682652	0.423087	ZNF148
1	69582673	69682673	0.415736	SNX4
1	69582673	69682673	0.415736	ZNF148
1	69582708	69682708	0.415736	SNX4
1	69582708	69682708	0.415736	ZNF148
1	69582730	69682730	0.415736	SNX4
1	69582730	69682730	0.415736	ZNF148
1	69582880	69682880	0.405731	SNX4
1	69582880	69682880	0.405731	ZNF148
1	69582884	69682884	0.405731	SNX4
1	69582884	69682884	0.405731	ZNF148
1	69583594	69683594	0.413063	SNX4
1	69583594	69683594	0.413063	ZNF148
1	69583656	69683656	0.427356	SNX4
1	69583656	69683656	0.427356	ZNF148
1	69583661	69683661	0.433799	SNX4
1	69583661	69683661	0.433799	ZNF148
1	69583968	69683968	0.433954	SNX4
1	69583968	69683968	0.433954	ZNF148
1	69584164	69684164	0.429519	SNX4
1	69584164	69684164	0.429519	ZNF148
1	69584341	69684341	0.433663	SNX4
1	69584341	69684341	0.433663	ZNF148
1	69584809	69684809	0.468531	SNX4
1	69584809	69684809	0.468531	ZNF148
1	69584837	69684837	0.401221	SNX4
1	69584837	69684837	0.401221	ZNF148
1	69584996	69684996	0.398125	SNX4
1	69584996	69684996	0.398125	ZNF148
1	69585333	69685333	0.39213	SNX4
1	69585333	69685333	0.39213	ZNF148
1	69585577	69685577	0.383356	SNX4
1	69585577	69685577	0.383356	ZNF148
1	69586081	69686081	0.388753	SNX4
1	69586081	69686081	0.388753	ZNF148
1	69586099	69686099	0.393046	SNX4
1	69586099	69686099	0.393046	ZNF148
1	69586242	69686242	0.392627	SNX4
1	69586242	69686242	0.392627	ZNF148
1	69586617	69686617	0.395819	SNX4
1	69586617	69686617	0.395819	ZNF148
1	69586621	69686621	0.395903	SNX4
1	69586621	69686621	0.395903	ZNF148
1	69586729	69686729	0.406406	SNX4
1	69586729	69686729	0.406406	ZNF148
1	69586784	69686784	0.406414	SNX4
1	69586784	69686784	0.406414	ZNF148

1	69586892	69686892	0.410903	SNX4
1	69586929	69686929	0.42082	SNX4
1	69587082	69687082	0.417459	SNX4
1	69587148	69687148	0.417459	SNX4
1	69587214	69687214	0.417574	SNX4
1	69587508	69687508	0.422211	SNX4
1	69587546	69687546	0.423238	SNX4
1	69587818	69687818	0.413867	SNX4
1	69587912	69687912	0.411048	SNX4
1	69587963	69687963	0.413157	SNX4
1	69588067	69688067	0.413442	SNX4
1	69588070	69688070	0.415897	SNX4
1	69588173	69688173	0.40883	SNX4
1	69588185	69688185	0.400732	SNX4
1	69588684	69688684	0.402443	SNX4
1	69588820	69688820	0.406021	SNX4
1	69588833	69688833	0.4021	SNX4
1	69589112	69689112	0.395425	SNX4
1	69589261	69689261	0.397727	SNX4
1	69589640	69689640	0.401644	SNX4
1	69589857	69689857	0.396717	SNX4
1	69590020	69690020	0.392045	SNX4
1	69590050	69690050	0.386325	SNX4
1	69590387	69690387	0.390132	SNX4
1	69590440	69690440	0.393084	SNX4
1	69590596	69690596	0.391893	SNX4
1	69590731	69690731	0.390259	SNX4
1	69590820	69690820	0.383825	SNX4
1	69590836	69690836	0.383673	SNX4
1	69591180	69691180	0.382945	SNX4
1	69591448	69691448	0.377623	SNX4
1	69592179	69692179	0.37796	SNX4
1	69592217	69692217	0.385436	SNX4
1	69592244	69692244	0.385774	SNX4
1	69592395	69692395	0.379081	SNX4
1	69592724	69692724	0.380077	SNX4
1	69592829	69692829	0.380359	SNX4
1	69593070	69693070	0.382616	SNX4
1	69593145	69693145	0.385613	SNX4
1	69593150	69693150	0.385556	SNX4
1	69593203	69693203	0.385757	SNX4
1	69594090	69694090	0.387417	SNX4
1	69594188	69694188	0.388409	SNX4
1	69594482	69694482	0.384788	SNX4
1	69594581	69694581	0.394039	SNX4
1	69594739	69694739	0.383425	SNX4
1	69594744	69694744	0.382601	SNX4
1	69595044	69695044	0.38355	SNX4
1	69595277	69695277	0.382083	SNX4
1	69595372	69695372	0.381783	SNX4
1	69595776	69695776	0.377301	SNX4
1	69596320	69696320	0.384575	SNX4
1	69596350	69696350	0.378877	SNX4
1	69596402	69696402	0.378877	SNX4
1	69596609	69696609	0.383349	SNX4
1	69596803	69696803	0.379022	SNX4
1	69597480	69697480	0.385907	SNX4
1	69597633	69697633	0.380479	SNX4

1	69597704	69697704	0.379732	SNX4
1	69597831	69697831	0.377509	SNX4
1	69597867	69697867	0.36421	SNX4
1	69598141	69698141	0.368056	SNX4
1	69598849	69698849	0.35739	SNX4
1	69599031	69699031	0.361209	SNX4
1	69599105	69699105	0.364383	SNX4
1	69599818	69699818	0.373779	SNX4
1	69599931	69699931	0.441027	SNX4
1	69600089	69700089	0.437621	SNX4
1	69600251	69700251	0.443714	SNX4
1	69600310	69700310	0.44405	SNX4
1	69600324	69700324	0.451982	SNX4
1	69600411	69700411	0.464146	SNX4
1	69600418	69700418	0.434234	SNX4
1	69600425	69700425	0.454886	SNX4
1	69600514	69700514	0.450418	SNX4
1	69600682	69700682	0.448692	SNX4
1	69600971	69700971	0.445222	SNX4
1	69601379	69701379	0.457141	SNX4
1	69601393	69701393	0.453465	SNX4
1	69601573	69701573	0.453043	SNX4
1	69602005	69702005	0.447995	SNX4
1	69602015	69702015	0.457508	SNX4
1	69602142	69702142	0.445379	SNX4
1	69602159	69702159	0.45901	SNX4
1	69602264	69702264	0.439607	SNX4
1	69602516	69702516	0.420575	SNX4
1	69602703	69702703	0.419988	SNX4
1	69602739	69702739	0.400819	SNX4
1	69602798	69702798	0.373026	SNX4
1	69603344	69703344	0.369661	SNX4
1	69603495	69703495	0.370549	SNX4
1	69603851	69703851	0.38317	SNX4
1	69603965	69703965	0.365907	SNX4
1	69604006	69704006	0.359344	SNX4
1	69604652	69704652	0.35976	SNX4
1	69605154	69705154	0.359139	SNX4
1	69605355	69705355	0.359139	SNX4
1	69605467	69705467	0.359139	SNX4
1	69605509	69705509	0.364971	SNX4
1	69605591	69705591	0.372116	SNX4
1	69605699	69705699	0.369943	SNX4
1	69605921	69705921	0.369943	SNX4
1	69605993	69705993	0.369943	SNX4
1	69606116	69706116	0.374373	SNX4
1	69606206	69706206	0.372107	SNX4
1	69606303	69706303	0.401198	SNX4
1	69606308	69706308	0.366839	SNX4
1	69606315	69706315	0.358475	SNX4
1	69606553	69706553	0.351305	SNX4
1	69606652	69706652	0.351305	SNX4
1	69607647	69707647	0.343306	SNX4
1	69610794	69710794	0.34681	SNX4
1	69612645	69712645	0.373494	SNX4
1	69612716	69712716	0.410934	SNX4
1	69612917	69712917	0.434343	SNX4
1	69612984	69712984	0.434282	SNX4

1	69613637	69713637	0.432204	SNX4
1	69613643	69713643	0.429699	SNX4
1	69613678	69713678	0.429265	SNX4
1	69613795	69713795	0.444975	SNX4
1	69613821	69713821	0.444001	SNX4
1	69613894	69713894	0.440796	SNX4
1	69614056	69714056	0.440269	SNX4
1	69614242	69714242	0.439748	SNX4
1	69614495	69714495	0.430737	SNX4
1	69614787	69714787	0.440855	SNX4
1	69614817	69714817	0.432871	SNX4
1	69615234	69715234	0.426087	SNX4
1	69615246	69715246	0.42314	SNX4
1	69615256	69715256	0.41081	SNX4
1	69615261	69715261	0.408632	SNX4
1	69615711	69715711	0.408632	SNX4
1	69615896	69715896	0.408459	SNX4
1	69616477	69716477	0.410836	SNX4
1	69616506	69716506	0.420846	SNX4
1	69616648	69716648	0.391351	SNX4
1	69616958	69716958	0.395359	SNX4
1	69616979	69716979	0.392137	SNX4
1	69617365	69717365	0.393624	SNX4
1	69617710	69717710	0.39303	SNX4
1	69617755	69717755	0.388246	SNX4
1	69617765	69717765	0.388246	SNX4
1	69618097	69718097	0.388246	SNX4
1	69618316	69718316	0.387452	SNX4
1	69618600	69718600	0.391297	SNX4
1	69619092	69719092	0.402522	SNX4
1	69619118	69719118	0.409606	SNX4
1	69619184	69719184	0.408297	SNX4
1	69619493	69719493	0.391533	SNX4
1	69619574	69719574	0.392351	SNX4
1	69620316	69720316	0.391237	SNX4
1	69620379	69720379	0.404237	SNX4
1	69620381	69720381	0.404237	SNX4
1	69620520	69720520	0.410803	SNX4
1	69620651	69720651	0.403611	SNX4
1	69620879	69720879	0.401814	SNX4
1	69621119	69721119	0.403177	SNX4
1	69621144	69721144	0.398145	SNX4
1	69621744	69721744	0.395981	SNX4
1	69621816	69721816	0.393283	SNX4
1	69621980	69721980	0.393283	SNX4
1	69622238	69722238	0.40303	SNX4
1	69622478	69722478	0.416384	SNX4
1	69622658	69722658	0.404555	SNX4
1	69622734	69722734	0.402663	SNX4
1	69623056	69723056	0.411376	SNX4
1	69623236	69723236	0.407294	SNX4
1	69623339	69723339	0.394078	SNX4
1	69623633	69723633	0.407498	SNX4
1	69624063	69724063	0.395795	SNX4
1	69624321	69724321	0.396987	SNX4
1	69624343	69724343	0.394476	SNX4
1	69624727	69724727	0.396467	SNX4
1	69625342	69725342	0.392277	SNX4

1	69625362	69725362	0.383251	SNX4
1	69625594	69725594	0.401649	SNX4
1	69625707	69725707	0.388791	SNX4
1	69625783	69725783	0.392502	SNX4
1	69625883	69725883	0.351422	SNX4
1	69626788	69726788	0.351422	SNX4
1	69627290	69727290	0.350879	SNX4
1	69627290	69727290	0.350879	OSBPL11
1	69627497	69727497	0.369089	SNX4
1	69627497	69727497	0.369089	OSBPL11
1	69628068	69728068	0.347648	SNX4
1	69628068	69728068	0.347648	OSBPL11
1	69628473	69728473	0.354387	SNX4
1	69628473	69728473	0.354387	OSBPL11
1	69629207	69729207	0.3473	SNX4
1	69629207	69729207	0.3473	OSBPL11
1	69629482	69729482	0.350035	SNX4
1	69629482	69729482	0.350035	OSBPL11
1	69630182	69730182	0.34765	SNX4
1	69630182	69730182	0.34765	OSBPL11
1	69630265	69730265	0.373038	SNX4
1	69630265	69730265	0.373038	OSBPL11
1	69630515	69730515	0.382654	SNX4
1	69630515	69730515	0.382654	OSBPL11
1	69631524	69731524	0.37311	SNX4
1	69631524	69731524	0.37311	OSBPL11
1	69631822	69731822	0.357689	SNX4
1	69631822	69731822	0.357689	OSBPL11
1	69633382	69733382	0.364373	SNX4
1	69633382	69733382	0.364373	OSBPL11
1	69633501	69733501	0.383358	SNX4
1	69633501	69733501	0.383358	OSBPL11
1	69633509	69733509	0.383358	SNX4
1	69633509	69733509	0.383358	OSBPL11
1	69633510	69733510	0.388396	SNX4
1	69633510	69733510	0.388396	OSBPL11
1	69633533	69733533	0.409999	SNX4
1	69633533	69733533	0.409999	OSBPL11
1	69633708	69733708	0.42161	SNX4
1	69633708	69733708	0.42161	OSBPL11
1	69633781	69733781	0.42578	SNX4
1	69633781	69733781	0.42578	OSBPL11
1	69633789	69733789	0.425121	SNX4
1	69633789	69733789	0.425121	OSBPL11
1	69635379	69735379	0.411415	SNX4
1	69635379	69735379	0.411415	OSBPL11
1	69635685	69735685	0.421384	SNX4
1	69635685	69735685	0.421384	OSBPL11
1	69635690	69735690	0.422674	SNX4
1	69635690	69735690	0.422674	OSBPL11
1	69635726	69735726	0.425652	SNX4
1	69635726	69735726	0.425652	OSBPL11
1	69635828	69735828	0.41712	SNX4
1	69635828	69735828	0.41712	OSBPL11
1	69635924	69735924	0.43061	SNX4
1	69635924	69735924	0.43061	OSBPL11
1	69636368	69736368	0.427087	SNX4
1	69636368	69736368	0.427087	OSBPL11

1	69636668	69736668	0.433043	SNX4
1	69636668	69736668	0.433043	OSBPL11
1	69636970	69736970	0.435693	SNX4
1	69636970	69736970	0.435693	OSBPL11
1	69637403	69737403	0.431782	SNX4
1	69637403	69737403	0.431782	OSBPL11
1	69637424	69737424	0.437792	SNX4
1	69637424	69737424	0.437792	OSBPL11
1	69637613	69737613	0.432691	SNX4
1	69637613	69737613	0.432691	OSBPL11
1	69637740	69737740	0.432691	SNX4
1	69637740	69737740	0.432691	OSBPL11
1	69637978	69737978	0.44554	SNX4
1	69637978	69737978	0.44554	OSBPL11
1	69637991	69737991	0.378295	SNX4
1	69637991	69737991	0.378295	OSBPL11
1	69637993	69737993	0.417797	SNX4
1	69637993	69737993	0.417797	OSBPL11
1	69638131	69738131	0.386321	SNX4
1	69638131	69738131	0.386321	OSBPL11
1	69638466	69738466	0.374759	SNX4
1	69638466	69738466	0.374759	OSBPL11
1	69638891	69738891	0.374014	SNX4
1	69638891	69738891	0.374014	OSBPL11
1	69638981	69738981	0.355339	SNX4
1	69638981	69738981	0.355339	OSBPL11
1	69639289	69739289	0.356064	SNX4
1	69639289	69739289	0.356064	OSBPL11
1	69641802	69741802	0.347376	SNX4
1	69641802	69741802	0.347376	OSBPL11
1	69642185	69742185	0.34339	SNX4
1	69642185	69742185	0.34339	OSBPL11
1	69642573	69742573	0.353479	SNX4
1	69642573	69742573	0.353479	OSBPL11
1	69642980	69742980	0.361278	SNX4
1	69642980	69742980	0.361278	OSBPL11
1	69643063	69743063	0.389795	SNX4
1	69643063	69743063	0.389795	OSBPL11
1	69643064	69743064	0.385661	SNX4
1	69643064	69743064	0.385661	OSBPL11
1	69643138	69743138	0.381649	SNX4
1	69643138	69743138	0.381649	OSBPL11
1	69643491	69743491	0.386054	SNX4
1	69643491	69743491	0.386054	OSBPL11
1	69643761	69743761	0.39268	SNX4
1	69643761	69743761	0.39268	OSBPL11
1	69643777	69743777	0.391225	SNX4
1	69643777	69743777	0.391225	OSBPL11
1	69644022	69744022	0.390924	SNX4
1	69644022	69744022	0.390924	OSBPL11
1	69644027	69744027	0.392173	SNX4
1	69644027	69744027	0.392173	OSBPL11
1	69644087	69744087	0.389636	SNX4
1	69644087	69744087	0.389636	OSBPL11
1	69644479	69744479	0.38928	SNX4
1	69644479	69744479	0.38928	OSBPL11
1	69644680	69744680	0.377423	SNX4
1	69644680	69744680	0.377423	OSBPL11

1	69644724	69744724	0.378946	SNX4
1	69644724	69744724	0.378946	OSBPL11
1	69645002	69745002	0.37578	SNX4
1	69645002	69745002	0.37578	OSBPL11
1	69645350	69745350	0.370933	SNX4
1	69645350	69745350	0.370933	OSBPL11
1	69645708	69745708	0.377546	SNX4
1	69645708	69745708	0.377546	OSBPL11
1	69645836	69745836	0.378077	SNX4
1	69645836	69745836	0.378077	OSBPL11
1	69645908	69745908	0.355019	SNX4
1	69645908	69745908	0.355019	OSBPL11
1	69646416	69746416	0.354205	SNX4
1	69646416	69746416	0.354205	OSBPL11
1	69648425	69748425	0.354477	SNX4
1	69648425	69748425	0.354477	OSBPL11
1	69648821	69748821	0.348082	SNX4
1	69648821	69748821	0.348082	OSBPL11
1	69648927	69748927	0.344546	SNX4
1	69648927	69748927	0.344546	OSBPL11
1	69648947	69748947	0.344546	SNX4
1	69648947	69748947	0.344546	OSBPL11
1	69649421	69749421	0.375095	SNX4
1	69649421	69749421	0.375095	OSBPL11
1	69649638	69749638	0.377095	SNX4
1	69649638	69749638	0.377095	OSBPL11
1	69649709	69749709	0.380521	SNX4
1	69649709	69749709	0.380521	OSBPL11
1	69649837	69749837	0.369276	SNX4
1	69649837	69749837	0.369276	OSBPL11
1	69651769	69751769	0.359836	SNX4
1	69651769	69751769	0.359836	OSBPL11
1	69652005	69752005	0.349747	SNX4
1	69652005	69752005	0.349747	OSBPL11
1	69652231	69752231	0.346841	SNX4
1	69652231	69752231	0.346841	OSBPL11
1	69653096	69753096	0.347861	SNX4
1	69653096	69753096	0.347861	OSBPL11
1	69653300	69753300	0.349665	SNX4
1	69653300	69753300	0.349665	OSBPL11
1	69653880	69753880	0.368632	SNX4
1	69653880	69753880	0.368632	OSBPL11
1	69653941	69753941	0.372732	SNX4
1	69653941	69753941	0.372732	OSBPL11
1	69653953	69753953	0.367097	SNX4
1	69653953	69753953	0.367097	OSBPL11
1	69653986	69753986	0.378361	SNX4
1	69653986	69753986	0.378361	OSBPL11
1	69654473	69754473	0.378309	SNX4
1	69654473	69754473	0.378309	OSBPL11
1	69654544	69754544	0.391423	SNX4
1	69654544	69754544	0.391423	OSBPL11
1	69654937	69754937	0.383515	SNX4
1	69654937	69754937	0.383515	OSBPL11
1	69654971	69754971	0.357362	SNX4
1	69654971	69754971	0.357362	OSBPL11
1	69654981	69754981	0.357362	SNX4
1	69654981	69754981	0.357362	OSBPL11

1	69655217	69755217	0.345752	SNX4
1	69655217	69755217	0.345752	OSBPL11
1	69899911	69999911	0.362958	RPL35A
1	69899911	69999911	0.362958	LMLN
1	69899911	69999911	0.362958	IQCG
1	69899911	69999911	0.362958	LRCH3
1	69900324	70000324	0.359013	RPL35A
1	69900324	70000324	0.359013	LMLN
1	69900324	70000324	0.359013	IQCG
1	69900324	70000324	0.359013	LRCH3
1	69900384	70000384	0.378757	RPL35A
1	69900384	70000384	0.378757	LMLN
1	69900384	70000384	0.378757	IQCG
1	69900384	70000384	0.378757	LRCH3
1	69900835	70000835	0.372949	RPL35A
1	69900835	70000835	0.372949	LMLN
1	69900835	70000835	0.372949	IQCG
1	69900835	70000835	0.372949	LRCH3
1	69900922	70000922	0.360432	RPL35A
1	69900922	70000922	0.360432	LMLN
1	69900922	70000922	0.360432	IQCG
1	69900922	70000922	0.360432	LRCH3
1	69901492	70001492	0.370838	RPL35A
1	69901492	70001492	0.370838	LMLN
1	69901492	70001492	0.370838	IQCG
1	69901492	70001492	0.370838	LRCH3
1	69902017	70002017	0.363813	RPL35A
1	69902017	70002017	0.363813	LMLN
1	69902017	70002017	0.363813	IQCG
1	69902017	70002017	0.363813	LRCH3
1	69902028	70002028	0.36548	RPL35A
1	69902028	70002028	0.36548	LMLN
1	69902028	70002028	0.36548	IQCG
1	69902028	70002028	0.36548	LRCH3
1	69902044	70002044	0.373061	RPL35A
1	69902044	70002044	0.373061	LMLN
1	69902044	70002044	0.373061	IQCG
1	69902044	70002044	0.373061	LRCH3
1	69902074	70002074	0.36815	RPL35A
1	69902074	70002074	0.36815	LMLN
1	69902074	70002074	0.36815	IQCG
1	69902074	70002074	0.36815	LRCH3
1	69902412	70002412	0.360764	RPL35A
1	69902412	70002412	0.360764	LMLN
1	69902412	70002412	0.360764	IQCG
1	69902412	70002412	0.360764	LRCH3
1	69902498	70002498	0.377591	RPL35A
1	69902498	70002498	0.377591	LMLN
1	69902498	70002498	0.377591	IQCG
1	69902498	70002498	0.377591	LRCH3
1	69902918	70002918	0.360091	RPL35A
1	69902918	70002918	0.360091	LMLN
1	69902918	70002918	0.360091	IQCG
1	69902918	70002918	0.360091	LRCH3
1	69902954	70002954	0.362219	RPL35A
1	69902954	70002954	0.362219	LMLN
1	69902954	70002954	0.362219	IQCG
1	69902954	70002954	0.362219	LRCH3

1	69903044	70003044	0.362219	RPL35A
1	69903044	70003044	0.362219	LMLN
1	69903044	70003044	0.362219	IQCG
1	69903044	70003044	0.362219	LRCH3
1	69903628	70003628	0.36084	RPL35A
1	69903628	70003628	0.36084	LMLN
1	69903628	70003628	0.36084	IQCG
1	69903628	70003628	0.36084	LRCH3
1	77169608	77269608	0.349351	TP63
1	80034368	80134368	0.348483	TRNAK-UUU-3
1	80034368	80134368	0.348483	ADIPOQ
1	80034368	80134368	0.348483	RFC4
1	80080048	80180048	0.352995	EIF4A2
1	80080048	80180048	0.352995	MIR1248
1	80080048	80180048	0.352995	ADIPOQ
1	80080048	80180048	0.352995	RFC4
1	80080048	80180048	0.352995	KNG1
1	80080134	80180134	0.369622	EIF4A2
1	80080134	80180134	0.369622	MIR1248
1	80080134	80180134	0.369622	ADIPOQ
1	80080134	80180134	0.369622	RFC4
1	80080134	80180134	0.369622	KNG1
1	80080188	80180188	0.349004	EIF4A2
1	80080188	80180188	0.349004	MIR1248
1	80080188	80180188	0.349004	ADIPOQ
1	80080188	80180188	0.349004	RFC4
1	80080188	80180188	0.349004	KNG1
1	80080278	80180278	0.368781	EIF4A2
1	80080278	80180278	0.368781	MIR1248
1	80080278	80180278	0.368781	ADIPOQ
1	80080278	80180278	0.368781	RFC4
1	80080278	80180278	0.368781	KNG1
1	80105432	80205432	0.345323	EIF4A2
1	80105432	80205432	0.345323	MIR1248
1	80105432	80205432	0.345323	ADIPOQ
1	80105432	80205432	0.345323	RFC4
1	80105432	80205432	0.345323	KNG1
1	80105787	80205787	0.358202	EIF4A2
1	80105787	80205787	0.358202	MIR1248
1	80105787	80205787	0.358202	RFC4
1	80105787	80205787	0.358202	KNG1
1	80106125	80206125	0.356569	EIF4A2
1	80106125	80206125	0.356569	MIR1248
1	80106125	80206125	0.356569	RFC4
1	80106125	80206125	0.356569	KNG1
1	80106246	80206246	0.346875	EIF4A2
1	80106246	80206246	0.346875	MIR1248
1	80106246	80206246	0.346875	RFC4
1	80106246	80206246	0.346875	KNG1
1	80109396	80209396	0.342236	EIF4A2
1	80109396	80209396	0.342236	MIR1248
1	80109396	80209396	0.342236	HRG
1	80109396	80209396	0.342236	RFC4
1	80109396	80209396	0.342236	KNG1
1	80109479	80209479	0.345169	EIF4A2
1	80109479	80209479	0.345169	MIR1248
1	80109479	80209479	0.345169	HRG
1	80109479	80209479	0.345169	RFC4

1	80109479	80209479	0.345169	KNG1
1	80109730	80209730	0.342832	EIF4A2
1	80109730	80209730	0.342832	MIR1248
1	80109730	80209730	0.342832	HRG
1	80109730	80209730	0.342832	RFC4
1	80109730	80209730	0.342832	KNG1
1	80710937	80810937	0.395911	ETV5
1	80710937	80810937	0.395911	DGKG
1	80711155	80811155	0.342245	ETV5
1	80711155	80811155	0.342245	DGKG
1	80941379	81041379	0.344696	LOC102178398
1	80941379	81041379	0.344696	LOC102179418
1	80941379	81041379	0.344696	LOC108635834
1	80941388	81041388	0.34712	LOC102178398
1	80941388	81041388	0.34712	LOC102179418
1	80941388	81041388	0.34712	LOC108635834
1	80941458	81041458	0.343726	LOC102178398
1	80941458	81041458	0.343726	LOC102179418
1	80941458	81041458	0.343726	LOC108635834
1	80941604	81041604	0.344049	LOC102178398
1	80941604	81041604	0.344049	LOC102179418
1	80941604	81041604	0.344049	LOC108635834
1	80941613	81041613	0.342429	LOC102178398
1	80941613	81041613	0.342429	LOC102179418
1	80941613	81041613	0.342429	LOC108635834
1	80941655	81041655	0.343856	LOC102178398
1	80941655	81041655	0.343856	LOC102179418
1	80941655	81041655	0.343856	LOC108635834
1	80941848	81041848	0.343856	LOC102178398
1	80941848	81041848	0.343856	LOC102179418
1	80941848	81041848	0.343856	LOC108635834
1	80942154	81042154	0.345364	LOC102178398
1	80942154	81042154	0.345364	LOC102179418
1	80942154	81042154	0.345364	LOC108635834
1	80942176	81042176	0.350859	LOC102178398
1	80942176	81042176	0.350859	LOC102179418
1	80942176	81042176	0.350859	LOC108635834
1	80942243	81042243	0.344124	LOC102178398
1	80942243	81042243	0.344124	LOC102179418
1	80942243	81042243	0.344124	LOC108635834
1	80942279	81042279	0.361743	LOC102178398
1	80942279	81042279	0.361743	LOC102179418
1	80942279	81042279	0.361743	LOC108635834
1	80942303	81042303	0.36047	LOC102178398
1	80942303	81042303	0.36047	LOC102179418
1	80942303	81042303	0.36047	LOC108635834
1	80942528	81042528	0.360246	LOC102178398
1	80942528	81042528	0.360246	LOC102179418
1	80942528	81042528	0.360246	LOC108635834
1	85097581	85197581	0.344233	TRNAS-GCU
1	86754382	86854382	0.345912	PEX5L
1	86754414	86854414	0.345105	PEX5L
1	86755832	86855832	0.34728	PEX5L
1	86756915	86856915	0.383047	PEX5L
1	86757225	86857225	0.382361	PEX5L
1	86757282	86857282	0.414479	PEX5L
1	86757355	86857355	0.442664	PEX5L
1	86757396	86857396	0.417784	PEX5L

1	86757401	86857401	0.386904	PEX5L
1	86757402	86857402	0.360174	PEX5L
1	86757481	86857481	0.346498	PEX5L
1	90965597	91065597	0.363645	NAALADL2
1	91051773	91151773	0.348258	NAALADL2
1	91052026	91152026	0.348258	NAALADL2
1	91052614	91152614	0.345174	NAALADL2
1	91052679	91152679	0.348275	NAALADL2
1	91052680	91152680	0.348275	NAALADL2
1	91053369	91153369	0.392479	NAALADL2
1	91053508	91153508	0.391533	NAALADL2
1	91053531	91153531	0.394165	NAALADL2
1	91053533	91153533	0.391674	NAALADL2
1	91053652	91153652	0.388514	NAALADL2
1	91053695	91153695	0.393268	NAALADL2
1	91053756	91153756	0.392026	NAALADL2
1	91053759	91153759	0.391268	NAALADL2
1	91053766	91153766	0.391313	NAALADL2
1	91053772	91153772	0.391726	NAALADL2
1	91053824	91153824	0.387942	NAALADL2
1	91053985	91153985	0.393942	NAALADL2
1	91053996	91153996	0.392614	NAALADL2
1	91054287	91154287	0.386757	NAALADL2
1	91054372	91154372	0.368003	NAALADL2
1	91054433	91154433	0.355181	NAALADL2
1	91054737	91154737	0.347223	NAALADL2
1	91054959	91154959	0.354349	NAALADL2
1	91055012	91155012	0.372239	NAALADL2
1	91055023	91155023	0.366199	NAALADL2
1	91055237	91155237	0.350361	NAALADL2
1	91056303	91156303	0.361927	NAALADL2
1	91056386	91156386	0.361495	NAALADL2
1	91056508	91156508	0.365958	NAALADL2
1	91056628	91156628	0.363222	NAALADL2
1	91056913	91156913	0.358218	NAALADL2
1	91057240	91157240	0.35441	NAALADL2
1	91057406	91157406	0.369944	NAALADL2
1	91057595	91157595	0.369775	NAALADL2
1	91057619	91157619	0.360744	NAALADL2
1	91057665	91157665	0.3625	NAALADL2
1	91058024	91158024	0.361225	NAALADL2
1	91058210	91158210	0.373457	NAALADL2
1	91058253	91158253	0.370338	NAALADL2
1	91058264	91158264	0.375177	NAALADL2
1	91058338	91158338	0.379422	NAALADL2
1	91058364	91158364	0.378432	NAALADL2
1	91058373	91158373	0.376549	NAALADL2
1	91058541	91158541	0.37481	NAALADL2
1	91058655	91158655	0.378983	NAALADL2
1	91058663	91158663	0.387347	NAALADL2
1	91058749	91158749	0.384553	NAALADL2
1	91058776	91158776	0.383654	NAALADL2
1	91058802	91158802	0.385789	NAALADL2
1	91058806	91158806	0.386694	NAALADL2
1	91058869	91158869	0.386025	NAALADL2
1	91058915	91158915	0.387387	NAALADL2
1	91058923	91158923	0.385809	NAALADL2
1	91059024	91159024	0.386668	NAALADL2

1	91059037	91159037	0.386668	NAALADL2
1	91059053	91159053	0.385563	NAALADL2
1	91059054	91159054	0.385563	NAALADL2
1	91059077	91159077	0.388003	NAALADL2
1	91059085	91159085	0.387132	NAALADL2
1	91059165	91159165	0.386907	NAALADL2
1	91059209	91159209	0.395673	NAALADL2
1	91059407	91159407	0.396121	NAALADL2
1	91059415	91159415	0.390211	NAALADL2
1	91059559	91159559	0.387426	NAALADL2
1	91059610	91159610	0.383775	NAALADL2
1	91059620	91159620	0.385287	NAALADL2
1	91059687	91159687	0.37904	NAALADL2
1	91059714	91159714	0.37756	NAALADL2
1	91059798	91159798	0.363021	NAALADL2
1	91059960	91159960	0.371951	NAALADL2
1	91060201	91160201	0.371951	NAALADL2
1	91060264	91160264	0.369948	NAALADL2
1	91060340	91160340	0.374371	NAALADL2
1	91060364	91160364	0.374463	NAALADL2
1	91060425	91160425	0.373913	NAALADL2
1	91060576	91160576	0.370929	NAALADL2
1	91060611	91160611	0.373911	NAALADL2
1	91060641	91160641	0.370584	NAALADL2
1	91060871	91160871	0.370584	NAALADL2
1	91060927	91160927	0.362612	NAALADL2
1	91060930	91160930	0.365541	NAALADL2
1	91061023	91161023	0.369521	NAALADL2
1	91061070	91161070	0.366111	NAALADL2
1	91061090	91161090	0.366111	NAALADL2
1	91061274	91161274	0.366295	NAALADL2
1	91061399	91161399	0.3674	NAALADL2
1	91061430	91161430	0.384131	NAALADL2
1	91061457	91161457	0.37211	NAALADL2
1	91061650	91161650	0.37396	NAALADL2
1	91061761	91161761	0.372232	NAALADL2
1	91061770	91161770	0.369989	NAALADL2
1	91061820	91161820	0.368412	NAALADL2
1	91062088	91162088	0.368412	NAALADL2
1	91062162	91162162	0.373308	NAALADL2
1	91062256	91162256	0.359536	NAALADL2
1	91062380	91162380	0.358682	NAALADL2
1	91062543	91162543	0.345937	NAALADL2
1	91062585	91162585	0.346729	NAALADL2
1	91062713	91162713	0.353691	NAALADL2
1	91062815	91162815	0.360454	NAALADL2
1	91062866	91162866	0.359736	NAALADL2
1	91062915	91162915	0.359736	NAALADL2
1	91062942	91162942	0.359736	NAALADL2
1	91062974	91162974	0.354306	NAALADL2
1	91062980	91162980	0.353695	NAALADL2
1	91063254	91163254	0.3515	NAALADL2
1	91063320	91163320	0.355747	NAALADL2
1	91063329	91163329	0.347893	NAALADL2
1	91063650	91163650	0.357895	NAALADL2
1	91063913	91163913	0.362468	NAALADL2
1	91063963	91163963	0.361656	NAALADL2
1	91064012	91164012	0.357544	NAALADL2

1	91064082	91164082	0.358728	NAALADL2
1	91064109	91164109	0.371741	NAALADL2
1	91064161	91164161	0.370089	NAALADL2
1	91064256	91164256	0.360979	NAALADL2
1	91064396	91164396	0.355068	NAALADL2
1	91064461	91164461	0.357007	NAALADL2
1	91064598	91164598	0.357148	NAALADL2
1	91064661	91164661	0.365526	NAALADL2
1	91064665	91164665	0.368001	NAALADL2
1	91064699	91164699	0.36639	NAALADL2
1	91064881	91164881	0.36639	NAALADL2
1	91065035	91165035	0.371356	NAALADL2
1	91065147	91165147	0.369025	NAALADL2
1	91065150	91165150	0.37074	NAALADL2
1	91065397	91165397	0.358257	NAALADL2
1	91065451	91165451	0.363315	NAALADL2
1	91065646	91165646	0.373263	NAALADL2
1	91065670	91165670	0.379524	NAALADL2
1	91065746	91165746	0.373222	NAALADL2
1	91065823	91165823	0.382276	NAALADL2
1	91065882	91165882	0.380391	NAALADL2
1	91065892	91165892	0.385462	NAALADL2
1	91066014	91166014	0.388033	NAALADL2
1	91066103	91166103	0.382392	NAALADL2
1	91066219	91166219	0.374827	NAALADL2
1	91066412	91166412	0.357635	NAALADL2
1	91066605	91166605	0.360089	NAALADL2
1	91066658	91166658	0.360089	NAALADL2
1	91066729	91166729	0.362977	NAALADL2
1	91066757	91166757	0.354672	NAALADL2
1	91067284	91167284	0.354672	NAALADL2
1	91067395	91167395	0.354672	NAALADL2
1	91067716	91167716	0.344507	NAALADL2
1	91067831	91167831	0.362222	NAALADL2
1	91067838	91167838	0.364881	NAALADL2
1	91067849	91167849	0.364881	NAALADL2
1	91068087	91168087	0.358523	NAALADL2
1	91068359	91168359	0.361493	NAALADL2
1	91068362	91168362	0.376845	NAALADL2
1	91068566	91168566	0.34383	NAALADL2
1	91068666	91168666	0.345152	NAALADL2
1	91068746	91168746	0.344948	NAALADL2
1	91069363	91169363	0.349511	NAALADL2
1	91069399	91169399	0.349511	NAALADL2
1	91069666	91169666	0.344076	NAALADL2
1	91069812	91169812	0.349635	NAALADL2
1	91069996	91169996	0.359092	NAALADL2
1	91070076	91170076	0.354897	NAALADL2
1	91070128	91170128	0.349379	NAALADL2
1	91070172	91170172	0.35958	NAALADL2
1	91070250	91170250	0.378161	NAALADL2
1	91070260	91170260	0.378161	NAALADL2
1	91070264	91170264	0.36038	NAALADL2
1	91070390	91170390	0.349279	NAALADL2
1	91070391	91170391	0.348233	NAALADL2
1	91070404	91170404	0.351111	NAALADL2
1	91070408	91170408	0.348665	NAALADL2
1	91070661	91170661	0.345013	NAALADL2

1	91070692	91170692	0.348784	NAALADL2
1	91070699	91170699	0.348784	NAALADL2
1	91070788	91170788	0.348784	NAALADL2
1	91070915	91170915	0.348784	NAALADL2
1	91071138	91171138	0.347034	NAALADL2
1	91071259	91171259	0.347034	NAALADL2
1	91071455	91171455	0.347034	NAALADL2
1	91071467	91171467	0.347034	NAALADL2
1	91071485	91171485	0.364052	NAALADL2
1	91071663	91171663	0.344097	NAALADL2
1	91071851	91171851	0.345787	NAALADL2
1	91072317	91172317	0.345719	NAALADL2
1	91072389	91172389	0.356534	NAALADL2
1	91072478	91172478	0.353308	NAALADL2
1	91779247	91879247	0.418709	NAALADL2
1	91779254	91879254	0.399756	NAALADL2
1	91779261	91879261	0.411596	NAALADL2
1	91779269	91879269	0.389639	NAALADL2
1	91779297	91879297	0.352659	NAALADL2
1	91779330	91879330	0.347696	NAALADL2
1	92484592	92584592	0.362356	NAALADL2
1	92484629	92584629	0.360981	NAALADL2
1	92484643	92584643	0.360981	NAALADL2
1	92484660	92584660	0.353826	NAALADL2
1	92484661	92584661	0.353826	NAALADL2
1	92484704	92584704	0.366888	NAALADL2
1	92484824	92584824	0.365483	NAALADL2
1	92484894	92584894	0.3651	NAALADL2
1	92484913	92584913	0.368071	NAALADL2
1	92485128	92585128	0.360566	NAALADL2
1	92485172	92585172	0.341808	NAALADL2
1	92514427	92614427	0.349994	NAALADL2
1	92514740	92614740	0.346408	NAALADL2
1	93302082	93402082	0.383341	NLGN1
1	93302096	93402096	0.387538	NLGN1
1	93378256	93478256	0.354496	NLGN1
1	93378266	93478266	0.378347	NLGN1
1	93378391	93478391	0.354233	NLGN1
1	93378864	93478864	0.369264	NLGN1
1	93379668	93479668	0.403451	NLGN1
1	93379727	93479727	0.427801	NLGN1
1	93380464	93480464	0.406346	NLGN1
1	93380503	93480503	0.407549	NLGN1
1	93380605	93480605	0.345722	NLGN1
1	93605846	93705846	0.349046	TRNAC-GCA-8
1	93605870	93705870	0.349046	TRNAC-GCA-8
1	93612207	93712207	0.346078	TRNAC-GCA-8
1	93612250	93712250	0.343142	TRNAC-GCA-8
1	93612474	93712474	0.341939	TRNAC-GCA-8
1	93612622	93712622	0.342374	TRNAC-GCA-8
1	93612732	93712732	0.368685	TRNAC-GCA-8
1	93612737	93712737	0.369288	TRNAC-GCA-8
1	93612759	93712759	0.394984	TRNAC-GCA-8
1	93612836	93712836	0.374867	TRNAC-GCA-8
1	93612873	93712873	0.374365	TRNAC-GCA-8
1	93613169	93713169	0.374365	TRNAC-GCA-8
1	93613267	93713267	0.379972	TRNAC-GCA-8
1	93613369	93713369	0.384564	TRNAC-GCA-8

1	93613420	93713420	0.384564	TRNAC-GCA-8
1	93613431	93713431	0.373621	TRNAC-GCA-8
1	93613520	93713520	0.374517	TRNAC-GCA-8
1	93613622	93713622	0.368431	TRNAC-GCA-8
1	93613705	93713705	0.381298	TRNAC-GCA-8
1	93613755	93713755	0.383376	TRNAC-GCA-8
1	93613757	93713757	0.383376	TRNAC-GCA-8
1	93613803	93713803	0.388055	TRNAC-GCA-8
1	93614047	93714047	0.359545	TRNAC-GCA-8
1	93614121	93714121	0.359948	TRNAC-GCA-8
1	93614975	93714975	0.366762	TRNAC-GCA-8
1	93614976	93714976	0.366762	TRNAC-GCA-8
1	93614982	93714982	0.40133	TRNAC-GCA-8
1	93615178	93715178	0.400921	TRNAC-GCA-8
1	93615192	93715192	0.365112	TRNAC-GCA-8
1	93619008	93719008	0.347452	TRNAC-GCA-8
1	93619055	93719055	0.346956	TRNAC-GCA-8
1	93619391	93719391	0.351911	TRNAC-GCA-8
1	93619512	93719512	0.352604	TRNAC-GCA-8
1	93623043	93723043	0.34716	TRNAC-GCA-8
1	93623179	93723179	0.34822	TRNAC-GCA-8
1	93623216	93723216	0.37876	TRNAC-GCA-8
1	93623548	93723548	0.345515	TRNAC-GCA-8
1	93906380	94006380	0.346151	SPATA16
1	93908160	94008160	0.388494	SPATA16
1	93908454	94008454	0.390046	SPATA16
1	93908727	94008727	0.369807	SPATA16
1	93908767	94008767	0.35124	SPATA16
1	93908768	94008768	0.35124	SPATA16
1	93908771	94008771	0.351481	SPATA16
1	93908977	94008977	0.344808	SPATA16
1	93909351	94009351	0.388941	SPATA16
1	93909358	94009358	0.386977	SPATA16
1	93909359	94009359	0.38533	SPATA16
1	93909362	94009362	0.391643	SPATA16
1	93909367	94009367	0.396346	SPATA16
1	93909373	94009373	0.399288	SPATA16
1	93909374	94009374	0.399288	SPATA16
1	93909450	94009450	0.395788	SPATA16
1	93909624	94009624	0.393506	SPATA16
1	93910287	94010287	0.372816	SPATA16
1	93910400	94010400	0.397837	SPATA16
1	93910750	94010750	0.401686	SPATA16
1	93910808	94010808	0.409465	SPATA16
1	93910876	94010876	0.409465	SPATA16
1	93910991	94010991	0.409332	SPATA16
1	93911187	94011187	0.422481	SPATA16
1	93911202	94011202	0.426437	SPATA16
1	93911204	94011204	0.422075	SPATA16
1	93911230	94011230	0.426177	SPATA16
1	93911233	94011233	0.433932	SPATA16
1	93911245	94011245	0.427985	SPATA16
1	93911300	94011300	0.412685	SPATA16
1	93911315	94011315	0.409797	SPATA16
1	93911321	94011321	0.409797	SPATA16
1	93911430	94011430	0.394493	SPATA16
1	93911519	94011519	0.394278	SPATA16
1	93911524	94011524	0.399885	SPATA16

1	93911533	94011533	0.39187	SPATA16
1	93911666	94011666	0.390004	SPATA16
1	93911672	94011672	0.410519	SPATA16
1	94809444	94909444	0.367036	FNDC3B
1	94809728	94909728	0.343612	FNDC3B
1	94809812	94909812	0.343612	FNDC3B
1	94812217	94912217	0.342472	FNDC3B
1	94819688	94919688	0.346041	FNDC3B
1	94819960	94919960	0.346814	FNDC3B
1	94820077	94920077	0.346814	FNDC3B
1	94820421	94920421	0.348012	FNDC3B
1	94820732	94920732	0.344931	FNDC3B
1	94820748	94920748	0.348931	FNDC3B
1	94820772	94920772	0.349528	FNDC3B
1	94820860	94920860	0.342464	FNDC3B
1	94821187	94921187	0.345404	FNDC3B
1	94823820	94923820	0.348961	FNDC3B
1	94823822	94923822	0.352986	FNDC3B
1	94823843	94923843	0.36812	FNDC3B
1	94823972	94923972	0.370876	FNDC3B
1	94825085	94925085	0.350722	FNDC3B
1	94825119	94925119	0.34635	FNDC3B
1	94825163	94925163	0.343541	FNDC3B
1	94825498	94925498	0.343541	FNDC3B
1	94827386	94927386	0.345305	FNDC3B
1	94827415	94927415	0.343988	FNDC3B
1	94827450	94927450	0.348539	FNDC3B
1	94827476	94927476	0.348539	FNDC3B
1	94827531	94927531	0.363023	FNDC3B
1	94829537	94929537	0.359003	FNDC3B
1	94829667	94929667	0.361304	FNDC3B
1	94829822	94929822	0.342498	FNDC3B
1	94830164	94930164	0.346866	FNDC3B
1	94830915	94930915	0.368865	FNDC3B
1	94831472	94931472	0.341911	FNDC3B
1	94831549	94931549	0.348361	FNDC3B
1	94832879	94932879	0.344438	FNDC3B
1	94834571	94934571	0.342254	FNDC3B
1	94834705	94934705	0.345882	FNDC3B
1	94834706	94934706	0.345882	FNDC3B
1	94837086	94937086	0.365476	FNDC3B
1	94837173	94937173	0.368179	FNDC3B
1	94837354	94937354	0.364218	FNDC3B
1	94837800	94937800	0.351239	FNDC3B
1	94838208	94938208	0.359161	FNDC3B
1	94838234	94938234	0.359161	FNDC3B
1	94838262	94938262	0.365799	FNDC3B
1	94838758	94938758	0.350518	FNDC3B
1	94840517	94940517	0.343825	FNDC3B
1	94840694	94940694	0.344081	FNDC3B
1	94841410	94941410	0.342949	FNDC3B
1	94844211	94944211	0.346221	FNDC3B
1	94844224	94944224	0.348287	FNDC3B
1	94844271	94944271	0.376889	FNDC3B
1	94844306	94944306	0.377103	FNDC3B
1	94844310	94944310	0.378848	FNDC3B
1	94844353	94944353	0.371163	FNDC3B
1	94844707	94944707	0.372423	FNDC3B

1	94844763	94944763	0.382705	FNDC3B
1	94844939	94944939	0.381846	FNDC3B
1	94844944	94944944	0.384188	FNDC3B
1	94845060	94945060	0.381524	FNDC3B
1	94845145	94945145	0.382783	FNDC3B
1	94845186	94945186	0.381873	FNDC3B
1	94845845	94945845	0.367072	FNDC3B
1	94845874	94945874	0.376053	FNDC3B
1	94845890	94945890	0.384519	FNDC3B
1	94845932	94945932	0.379297	FNDC3B
1	94846363	94946363	0.393547	FNDC3B
1	94846364	94946364	0.393547	FNDC3B
1	94846407	94946407	0.363274	FNDC3B
1	94846711	94946711	0.365322	FNDC3B
1	94846754	94946754	0.377636	FNDC3B
1	94847089	94947089	0.374445	FNDC3B
1	94847097	94947097	0.354411	FNDC3B
1	94847144	94947144	0.361435	FNDC3B
1	94847220	94947220	0.364556	FNDC3B
1	94847357	94947357	0.365831	FNDC3B
1	94847413	94947413	0.36628	FNDC3B
1	94847434	94947434	0.365173	FNDC3B
1	94847590	94947590	0.360534	FNDC3B
1	94847597	94947597	0.350092	FNDC3B
1	94847622	94947622	0.349068	FNDC3B
1	94847941	94947941	0.345687	FNDC3B
1	95235522	95335522	0.348786	PLD1
1	95235587	95335587	0.362274	PLD1
1	95235647	95335647	0.360715	PLD1
1	95235737	95335737	0.361527	PLD1
1	95235738	95335738	0.357555	PLD1
1	95235761	95335761	0.354797	PLD1
1	95235775	95335775	0.345788	PLD1
1	95235821	95335821	0.345788	PLD1
1	95235855	95335855	0.346074	PLD1
1	95236126	95336126	0.345359	PLD1
1	95236178	95336178	0.354362	PLD1
1	95236285	95336285	0.353121	PLD1
1	95236325	95336325	0.352797	PLD1
1	95236547	95336547	0.355086	PLD1
1	95236611	95336611	0.350995	PLD1
1	95236927	95336927	0.343895	PLD1
1	95237110	95337110	0.353858	PLD1
1	95237212	95337212	0.351704	PLD1
1	95237239	95337239	0.351704	PLD1
1	95237279	95337279	0.351995	PLD1
1	95237321	95337321	0.352329	PLD1
1	95237378	95337378	0.352147	PLD1
1	95237423	95337423	0.35203	PLD1
1	95237438	95337438	0.35896	PLD1
1	95237490	95337490	0.357822	PLD1
1	95237539	95337539	0.37892	PLD1
1	95237543	95337543	0.376232	PLD1
1	95237563	95337563	0.371267	PLD1
1	95884525	95984525	0.342901	TNIK
1	95885377	95985377	0.34901	TNIK
1	95885647	95985647	0.349216	TNIK
1	95885659	95985659	0.349216	TNIK

1	95885796	95985796	0.349777	TNIK
1	95886482	95986482	0.363698	TNIK
1	95886550	95986550	0.369072	TNIK
1	95886790	95986790	0.369178	TNIK
1	95886918	95986918	0.366245	TNIK
1	95886928	95986928	0.366245	TNIK
1	95887471	95987471	0.374675	TNIK
1	95887558	95987558	0.372468	TNIK
1	95887980	95987980	0.369433	TNIK
1	95887996	95987996	0.364219	TNIK
1	95888188	95988188	0.358389	TNIK
1	95888326	95988326	0.344936	TNIK
1	95894096	95994096	0.344715	TNIK
1	95894118	95994118	0.348613	TNIK
1	95894386	95994386	0.348206	TNIK
1	95894471	95994471	0.348645	TNIK
1	95894483	95994483	0.349448	TNIK
1	96557197	96657197	0.387262	SKIL
1	96557197	96657197	0.387262	PRKCI
1	96557292	96657292	0.37401	SKIL
1	96557292	96657292	0.37401	PRKCI
1	96558404	96658404	0.413533	SKIL
1	96558404	96658404	0.413533	PRKCI
1	96558537	96658537	0.421928	SKIL
1	96558537	96658537	0.421928	PRKCI
1	96558712	96658712	0.402077	SKIL
1	96558712	96658712	0.402077	PRKCI
1	96559010	96659010	0.397704	SKIL
1	96559010	96659010	0.397704	PRKCI
1	96559291	96659291	0.403022	SKIL
1	96559291	96659291	0.403022	PRKCI
1	96559580	96659580	0.390798	SKIL
1	96559580	96659580	0.390798	PRKCI
1	96560413	96660413	0.390333	SKIL
1	96560413	96660413	0.390333	PRKCI
1	96560462	96660462	0.408997	SKIL
1	96560462	96660462	0.408997	PRKCI
1	96561396	96661396	0.444396	SKIL
1	96561396	96661396	0.444396	PRKCI
1	96561493	96661493	0.448287	SKIL
1	96561493	96661493	0.448287	PRKCI
1	96561623	96661623	0.457573	SKIL
1	96561623	96661623	0.457573	PRKCI
1	96561785	96661785	0.4574	SKIL
1	96561785	96661785	0.4574	PRKCI
1	96561818	96661818	0.464753	SKIL
1	96561818	96661818	0.464753	PRKCI
1	96561890	96661890	0.45753	SKIL
1	96561890	96661890	0.45753	PRKCI
1	96561902	96661902	0.45753	SKIL
1	96561902	96661902	0.45753	PRKCI
1	96561990	96661990	0.454924	SKIL
1	96561990	96661990	0.454924	PRKCI
1	96562015	96662015	0.452203	SKIL
1	96562015	96662015	0.452203	PRKCI
1	96562329	96662329	0.457117	SKIL
1	96562329	96662329	0.457117	PRKCI
1	96562620	96662620	0.445115	SKIL

1	96562620	96662620	0.445115	PRKCI
1	96562651	96662651	0.42985	SKIL
1	96562651	96662651	0.42985	PRKCI
1	96562768	96662768	0.44083	SKIL
1	96562768	96662768	0.44083	PRKCI
1	96562821	96662821	0.44157	SKIL
1	96562821	96662821	0.44157	PRKCI
1	96562875	96662875	0.409419	SKIL
1	96562875	96662875	0.409419	PRKCI
1	96562876	96662876	0.408526	SKIL
1	96562876	96662876	0.408526	PRKCI
1	96563009	96663009	0.410997	SKIL
1	96563009	96663009	0.410997	PRKCI
1	96563069	96663069	0.408729	SKIL
1	96563069	96663069	0.408729	PRKCI
1	96563569	96663569	0.386667	SKIL
1	96563569	96663569	0.386667	PRKCI
1	96563671	96663671	0.383865	SKIL
1	96563671	96663671	0.383865	PRKCI
1	96563734	96663734	0.386884	SKIL
1	96563734	96663734	0.386884	PRKCI
1	96564568	96664568	0.386884	SKIL
1	96564568	96664568	0.386884	PRKCI
1	96564946	96664946	0.429769	SKIL
1	96564946	96664946	0.429769	PRKCI
1	96565011	96665011	0.437974	SKIL
1	96565011	96665011	0.437974	PRKCI
1	96565031	96665031	0.431107	SKIL
1	96565031	96665031	0.431107	PRKCI
1	96565175	96665175	0.451029	SKIL
1	96565175	96665175	0.451029	PRKCI
1	96565222	96665222	0.474457	SKIL
1	96565222	96665222	0.474457	PRKCI
1	96565381	96665381	0.385463	SKIL
1	96565381	96665381	0.385463	PRKCI
1	96565587	96665587	0.385363	SKIL
1	96565587	96665587	0.385363	PRKCI
1	96565789	96665789	0.391496	SKIL
1	96565789	96665789	0.391496	PRKCI
1	96565945	96665945	0.383029	SKIL
1	96565945	96665945	0.383029	PRKCI
1	96566243	96666243	0.368163	SKIL
1	96566243	96666243	0.368163	PRKCI
1	96566370	96666370	0.359575	SKIL
1	96566370	96666370	0.359575	PRKCI
1	96568202	96668202	0.35262	SKIL
1	96568202	96668202	0.35262	PRKCI
1	96568593	96668593	0.387872	SKIL
1	96568593	96668593	0.387872	PRKCI
1	96568825	96668825	0.416629	SKIL
1	96568825	96668825	0.416629	PRKCI
1	96568862	96668862	0.415364	SKIL
1	96568862	96668862	0.415364	PRKCI
1	96568958	96668958	0.415688	SKIL
1	96568958	96668958	0.415688	PRKCI
1	96568983	96668983	0.419099	SKIL
1	96568983	96668983	0.419099	PRKCI
1	96569018	96669018	0.420671	SKIL

1	96569018	96669018	0.420671	PRKCI
1	96569325	96669325	0.413388	SKIL
1	96569325	96669325	0.413388	PRKCI
1	96569513	96669513	0.358964	SKIL
1	96569513	96669513	0.358964	PRKCI
1	96569515	96669515	0.358964	SKIL
1	96569515	96669515	0.358964	PRKCI
1	96569766	96669766	0.397	SKIL
1	96569766	96669766	0.397	PRKCI
1	96570008	96670008	0.400301	SKIL
1	96570008	96670008	0.400301	PRKCI
1	96570123	96670123	0.401282	SKIL
1	96570123	96670123	0.401282	PRKCI
1	96570604	96670604	0.400593	SKIL
1	96570604	96670604	0.400593	PRKCI
1	96570687	96670687	0.352444	SKIL
1	96570687	96670687	0.352444	PRKCI
1	96571329	96671329	0.351245	SKIL
1	96571329	96671329	0.351245	PRKCI
1	96655301	96755301	0.354233	PRKCI
1	96655301	96755301	0.354233	PHC3
1	96655431	96755431	0.350573	PRKCI
1	96655431	96755431	0.350573	PHC3
1	96658153	96758153	0.345151	PRKCI
1	96658153	96758153	0.345151	PHC3
1	96659179	96759179	0.359249	PRKCI
1	96659179	96759179	0.359249	PHC3
1	96661689	96761689	0.358684	PRKCI
1	96661689	96761689	0.358684	PHC3
1	96663947	96763947	0.363278	PRKCI
1	96663947	96763947	0.363278	PHC3
1	96664025	96764025	0.344005	PRKCI
1	96664025	96764025	0.344005	PHC3
1	96664043	96764043	0.359499	PRKCI
1	96664043	96764043	0.359499	PHC3
1	96664469	96764469	0.352033	PRKCI
1	96664469	96764469	0.352033	PHC3
1	96664651	96764651	0.361365	PRKCI
1	96664651	96764651	0.361365	PHC3
1	96665403	96765403	0.380574	PRKCI
1	96665403	96765403	0.380574	PHC3
1	96665857	96765857	0.386281	PRKCI
1	96665857	96765857	0.386281	PHC3
1	96665997	96765997	0.389279	PRKCI
1	96665997	96765997	0.389279	PHC3
1	96666248	96766248	0.387992	PRKCI
1	96666248	96766248	0.387992	PHC3
1	96666668	96766668	0.384767	PRKCI
1	96666668	96766668	0.384767	PHC3
1	96667503	96767503	0.367056	PRKCI
1	96667503	96767503	0.367056	PHC3
1	96667791	96767791	0.367056	PRKCI
1	96667791	96767791	0.367056	PHC3
1	96668481	96768481	0.378675	PRKCI
1	96668481	96768481	0.378675	PHC3
1	96668903	96768903	0.3885	PRKCI
1	96668903	96768903	0.3885	PHC3
1	96668950	96768950	0.418438	PRKCI

1	96668950	96768950	0.418438	PHC3
1	96669266	96769266	0.398541	PRKCI
1	96669266	96769266	0.398541	PHC3
1	96669488	96769488	0.384948	PRKCI
1	96669488	96769488	0.384948	PHC3
1	96669996	96769996	0.384948	PRKCI
1	96669996	96769996	0.384948	PHC3
1	96670174	96770174	0.384948	PRKCI
1	96670174	96770174	0.384948	PHC3
1	96670696	96770696	0.384948	PRKCI
1	96670696	96770696	0.384948	PHC3
1	96671008	96771008	0.381849	PRKCI
1	96671008	96771008	0.381849	PHC3
1	96671133	96771133	0.424781	PRKCI
1	96671133	96771133	0.424781	PHC3
1	96671228	96771228	0.424781	PRKCI
1	96671228	96771228	0.424781	PHC3
1	96671371	96771371	0.424781	PRKCI
1	96671371	96771371	0.424781	PHC3
1	96671452	96771452	0.441379	PRKCI
1	96671452	96771452	0.441379	PHC3
1	96671489	96771489	0.442664	PRKCI
1	96671489	96771489	0.442664	PHC3
1	96671538	96771538	0.453365	PRKCI
1	96671538	96771538	0.453365	PHC3
1	96671690	96771690	0.450047	PRKCI
1	96671690	96771690	0.450047	PHC3
1	96671726	96771726	0.445953	PRKCI
1	96671726	96771726	0.445953	PHC3
1	96671735	96771735	0.442472	PRKCI
1	96671735	96771735	0.442472	PHC3
1	96672196	96772196	0.419293	PRKCI
1	96672196	96772196	0.419293	PHC3
1	96673238	96773238	0.409075	PRKCI
1	96673238	96773238	0.409075	PHC3
1	96673912	96773912	0.409075	PRKCI
1	96673912	96773912	0.409075	PHC3
1	96674010	96774010	0.448429	PRKCI
1	96674010	96774010	0.448429	PHC3
1	96674022	96774022	0.454019	PRKCI
1	96674022	96774022	0.454019	PHC3
1	96674026	96774026	0.468657	PRKCI
1	96674026	96774026	0.468657	PHC3
1	96674035	96774035	0.4509	PRKCI
1	96674035	96774035	0.4509	PHC3
1	96674038	96774038	0.439188	PRKCI
1	96674038	96774038	0.439188	PHC3
1	96674104	96774104	0.469683	PRKCI
1	96674104	96774104	0.469683	PHC3
1	96674109	96774109	0.464121	PRKCI
1	96674109	96774109	0.464121	PHC3
1	96674114	96774114	0.467487	PRKCI
1	96674114	96774114	0.467487	PHC3
1	96674139	96774139	0.432497	PRKCI
1	96674139	96774139	0.432497	PHC3
1	96674143	96774143	0.439287	PRKCI
1	96674143	96774143	0.439287	PHC3
1	96674157	96774157	0.43538	PRKCI

1	96674157	96774157	0.43538	PHC3
1	96674158	96774158	0.436088	PRKCI
1	96674158	96774158	0.436088	PHC3
1	96674167	96774167	0.418655	PRKCI
1	96674167	96774167	0.418655	PHC3
1	96674170	96774170	0.40023	PRKCI
1	96674170	96774170	0.40023	PHC3
1	96674174	96774174	0.460004	PRKCI
1	96674174	96774174	0.460004	PHC3
1	96674433	96774433	0.440183	PRKCI
1	96674433	96774433	0.440183	PHC3
1	96674654	96774654	0.441119	PRKCI
1	96674654	96774654	0.441119	PHC3
1	96674880	96774880	0.441119	PRKCI
1	96674880	96774880	0.441119	PHC3
1	96674919	96774919	0.448334	PRKCI
1	96674919	96774919	0.448334	PHC3
1	96675012	96775012	0.450028	PRKCI
1	96675012	96775012	0.450028	PHC3
1	96675120	96775120	0.448895	PRKCI
1	96675120	96775120	0.448895	PHC3
1	96675125	96775125	0.43133	PRKCI
1	96675125	96775125	0.43133	PHC3
1	96675127	96775127	0.408222	PRKCI
1	96675127	96775127	0.408222	PHC3
1	96676076	96776076	0.381788	PRKCI
1	96676076	96776076	0.381788	PHC3
1	96676085	96776085	0.383126	PRKCI
1	96676085	96776085	0.383126	PHC3
1	96676108	96776108	0.384421	PRKCI
1	96676108	96776108	0.384421	PHC3
1	96676299	96776299	0.407137	PRKCI
1	96676299	96776299	0.407137	PHC3
1	96676868	96776868	0.400281	PRKCI
1	96676868	96776868	0.400281	PHC3
1	96677578	96777578	0.416793	PRKCI
1	96677578	96777578	0.416793	PHC3
1	96678232	96778232	0.413112	PRKCI
1	96678232	96778232	0.413112	PHC3
1	96678529	96778529	0.374916	PRKCI
1	96678529	96778529	0.374916	PHC3
1	96678631	96778631	0.392144	PRKCI
1	96678631	96778631	0.392144	PHC3
1	96678670	96778670	0.392138	PRKCI
1	96678670	96778670	0.392138	PHC3
1	96679005	96779005	0.406471	PRKCI
1	96679005	96779005	0.406471	PHC3
1	96679046	96779046	0.403348	PRKCI
1	96679046	96779046	0.403348	PHC3
1	96679333	96779333	0.399552	PRKCI
1	96679333	96779333	0.399552	PHC3
1	96679635	96779635	0.399552	PRKCI
1	96679635	96779635	0.399552	PHC3
1	96679792	96779792	0.393758	PRKCI
1	96679792	96779792	0.393758	PHC3
1	96680561	96780561	0.397966	PRKCI
1	96680561	96780561	0.397966	PHC3
1	96680740	96780740	0.395847	PRKCI

1	96680740	96780740	0.395847	PHC3
1	96681954	96781954	0.370755	PRKCI
1	96681954	96781954	0.370755	PHC3
1	96682096	96782096	0.375843	PRKCI
1	96682096	96782096	0.375843	PHC3
1	96682291	96782291	0.369637	PRKCI
1	96682291	96782291	0.369637	PHC3
1	96682733	96782733	0.369637	LOC102179814
1	96682733	96782733	0.369637	PRKCI
1	96682733	96782733	0.369637	PHC3
1	96683256	96783256	0.369637	LOC102179814
1	96683256	96783256	0.369637	PRKCI
1	96683256	96783256	0.369637	PHC3
1	96684226	96784226	0.370212	LOC102179814
1	96684226	96784226	0.370212	PRKCI
1	96684226	96784226	0.370212	PHC3
1	96684239	96784239	0.373471	LOC102179814
1	96684239	96784239	0.373471	PRKCI
1	96684239	96784239	0.373471	PHC3
1	96684510	96784510	0.374454	LOC102179814
1	96684510	96784510	0.374454	PRKCI
1	96684510	96784510	0.374454	PHC3
1	96685283	96785283	0.357555	LOC102179814
1	96685283	96785283	0.357555	PRKCI
1	96685283	96785283	0.357555	PHC3
1	96685537	96785537	0.35583	LOC102179814
1	96685537	96785537	0.35583	PRKCI
1	96685537	96785537	0.35583	PHC3
1	96685669	96785669	0.35583	LOC102179814
1	96685669	96785669	0.35583	PRKCI
1	96685669	96785669	0.35583	PHC3
1	96685760	96785760	0.359068	LOC102179814
1	96685760	96785760	0.359068	PRKCI
1	96685760	96785760	0.359068	PHC3
1	96685826	96785826	0.355023	LOC102179814
1	96685826	96785826	0.355023	PRKCI
1	96685826	96785826	0.355023	PHC3
1	96686826	96786826	0.347236	LOC102179814
1	96686826	96786826	0.347236	PRKCI
1	96686826	96786826	0.347236	PHC3
1	96688110	96788110	0.474229	LOC102179814
1	96688110	96788110	0.474229	PRKCI
1	96688110	96788110	0.474229	PHC3
1	96688187	96788187	0.482716	LOC102179814
1	96688187	96788187	0.482716	PRKCI
1	96688187	96788187	0.482716	PHC3
1	96688758	96788758	0.474344	LOC102179814
1	96688758	96788758	0.474344	PRKCI
1	96688758	96788758	0.474344	PHC3
1	96689149	96789149	0.474344	LOC102179814
1	96689149	96789149	0.474344	PRKCI
1	96689149	96789149	0.474344	PHC3
1	96689385	96789385	0.474344	LOC102179814
1	96689385	96789385	0.474344	PRKCI
1	96689385	96789385	0.474344	PHC3
1	96690910	96790910	0.49169	LOC102179814
1	96690910	96790910	0.49169	PRKCI
1	96690910	96790910	0.49169	PHC3

1	96692420	96792420	0.482555	LOC102179814
1	96692420	96792420	0.482555	PRKCI
1	96692420	96792420	0.482555	PHC3
1	96692456	96792456	0.486102	LOC102179814
1	96692456	96792456	0.486102	PRKCI
1	96692456	96792456	0.486102	PHC3
1	96692915	96792915	0.495208	LOC102179814
1	96692915	96792915	0.495208	PRKCI
1	96692915	96792915	0.495208	PHC3
1	96694378	96794378	0.495429	LOC102179814
1	96694378	96794378	0.495429	PRKCI
1	96694378	96794378	0.495429	PHC3
1	96694574	96794574	0.492165	LOC102179814
1	96694574	96794574	0.492165	PRKCI
1	96694574	96794574	0.492165	PHC3
1	96694742	96794742	0.487048	LOC102179814
1	96694742	96794742	0.487048	PRKCI
1	96694742	96794742	0.487048	PHC3
1	96697507	96797507	0.479406	LOC102179814
1	96697507	96797507	0.479406	PRKCI
1	96697507	96797507	0.479406	PHC3
1	96697648	96797648	0.480567	LOC102179814
1	96697648	96797648	0.480567	PRKCI
1	96697648	96797648	0.480567	PHC3
1	96697846	96797846	0.484264	LOC102179814
1	96697846	96797846	0.484264	PRKCI
1	96697846	96797846	0.484264	PHC3
1	96699160	96799160	0.484435	LOC102179814
1	96699160	96799160	0.484435	PRKCI
1	96699160	96799160	0.484435	PHC3
1	96699932	96799932	0.490207	LOC102179814
1	96699932	96799932	0.490207	PRKCI
1	96699932	96799932	0.490207	PHC3
1	96699953	96799953	0.488351	LOC102179814
1	96699953	96799953	0.488351	PRKCI
1	96699953	96799953	0.488351	PHC3
1	96699954	96799954	0.489528	LOC102179814
1	96699954	96799954	0.489528	PRKCI
1	96699954	96799954	0.489528	PHC3
1	96700312	96800312	0.478831	LOC102179814
1	96700312	96800312	0.478831	PRKCI
1	96700312	96800312	0.478831	PHC3
1	96701908	96801908	0.47579	LOC102179814
1	96701908	96801908	0.47579	PRKCI
1	96701908	96801908	0.47579	PHC3
1	96702080	96802080	0.417769	LOC102179814
1	96702080	96802080	0.417769	PRKCI
1	96702080	96802080	0.417769	PHC3
1	96703155	96803155	0.441496	LOC102179814
1	96703155	96803155	0.441496	PRKCI
1	96703155	96803155	0.441496	PHC3
1	96705665	96805665	0.442265	LOC102179814
1	96705665	96805665	0.442265	PHC3
1	96705880	96805880	0.441928	LOC102179814
1	96705880	96805880	0.441928	PHC3
1	96706695	96806695	0.444812	LOC102179814
1	96706695	96806695	0.444812	PHC3
1	96707657	96807657	0.467479	LOC102179814

1	96707657	96807657	0.467479	PHC3
1	96707904	96807904	0.478146	LOC102179814
1	96707904	96807904	0.478146	PHC3
1	96708274	96808274	0.460081	LOC102179814
1	96708274	96808274	0.460081	PHC3
1	96708644	96808644	0.457252	LOC102179814
1	96708644	96808644	0.457252	PHC3
1	96709285	96809285	0.454965	LOC102179814
1	96709285	96809285	0.454965	PHC3
1	96709298	96809298	0.45722	LOC102179814
1	96709298	96809298	0.45722	PHC3
1	96711133	96811133	0.431986	LOC102179814
1	96711133	96811133	0.431986	PHC3
1	96711379	96811379	0.442635	LOC102179814
1	96711379	96811379	0.442635	PHC3
1	96712493	96812493	0.411119	LOC102179814
1	96712493	96812493	0.411119	PHC3
1	96713313	96813313	0.392788	LOC102179814
1	96713313	96813313	0.392788	PHC3
1	96713426	96813426	0.392788	LOC102179814
1	96713426	96813426	0.392788	PHC3
1	96717620	96817620	0.402595	LOC102179814
1	96717620	96817620	0.402595	PHC3
1	96717620	96817620	0.402595	GPR160
1	96717755	96817755	0.378306	LOC102179814
1	96717755	96817755	0.378306	PHC3
1	96717755	96817755	0.378306	GPR160
1	96718318	96818318	0.378306	LOC102179814
1	96718318	96818318	0.378306	PHC3
1	96718318	96818318	0.378306	GPR160
1	96718620	96818620	0.376545	LOC102179814
1	96718620	96818620	0.376545	PHC3
1	96718620	96818620	0.376545	GPR160
1	96723546	96823546	0.390513	LOC102179814
1	96723546	96823546	0.390513	PHC3
1	96723546	96823546	0.390513	GPR160
1	96723585	96823585	0.406865	LOC102179814
1	96723585	96823585	0.406865	PHC3
1	96723585	96823585	0.406865	GPR160
1	96723732	96823732	0.42977	LOC102179814
1	96723732	96823732	0.42977	PHC3
1	96723732	96823732	0.42977	GPR160
1	96724206	96824206	0.467456	LOC102179814
1	96724206	96824206	0.467456	PHC3
1	96724206	96824206	0.467456	GPR160
1	96724783	96824783	0.474548	LOC102179814
1	96724783	96824783	0.474548	PHC3
1	96724783	96824783	0.474548	GPR160
1	96725270	96825270	0.474049	LOC102179814
1	96725270	96825270	0.474049	PHC3
1	96725270	96825270	0.474049	GPR160
1	96725309	96825309	0.467558	LOC102179814
1	96725309	96825309	0.467558	PHC3
1	96725309	96825309	0.467558	GPR160
1	96725854	96825854	0.45975	LOC102179814
1	96725854	96825854	0.45975	PHC3
1	96725854	96825854	0.45975	GPR160
1	96727184	96827184	0.448376	LOC102179814

1	96727184	96827184	0.448376	PHC3
1	96727184	96827184	0.448376	GPR160
1	96727217	96827217	0.449402	LOC102179814
1	96727217	96827217	0.449402	PHC3
1	96727217	96827217	0.449402	GPR160
1	96727343	96827343	0.459627	LOC102179814
1	96727343	96827343	0.459627	PHC3
1	96727343	96827343	0.459627	GPR160
1	96728111	96828111	0.462736	LOC102179814
1	96728111	96828111	0.462736	PHC3
1	96728111	96828111	0.462736	GPR160
1	96728668	96828668	0.463972	LOC102179814
1	96728668	96828668	0.463972	PHC3
1	96728668	96828668	0.463972	GPR160
1	96729523	96829523	0.466107	LOC102179814
1	96729523	96829523	0.466107	PHC3
1	96729523	96829523	0.466107	GPR160
1	96729570	96829570	0.477622	LOC102179814
1	96729570	96829570	0.477622	PHC3
1	96729570	96829570	0.477622	GPR160
1	96729704	96829704	0.471544	LOC102179814
1	96729704	96829704	0.471544	PHC3
1	96729704	96829704	0.471544	GPR160
1	96729782	96829782	0.452414	LOC102179814
1	96729782	96829782	0.452414	PHC3
1	96729782	96829782	0.452414	GPR160
1	96730903	96830903	0.456639	LOC102179814
1	96730903	96830903	0.456639	PHC3
1	96730903	96830903	0.456639	GPR160
1	96731698	96831698	0.455223	LOC102179814
1	96731698	96831698	0.455223	PHC3
1	96731698	96831698	0.455223	GPR160
1	96732579	96832579	0.476253	LOC102179814
1	96732579	96832579	0.476253	PHC3
1	96732579	96832579	0.476253	GPR160
1	96732686	96832686	0.469982	LOC102179814
1	96732686	96832686	0.469982	PHC3
1	96732686	96832686	0.469982	GPR160
1	96732861	96832861	0.465605	LOC102179814
1	96732861	96832861	0.465605	PHC3
1	96732861	96832861	0.465605	GPR160
1	96733284	96833284	0.465502	LOC102179814
1	96733284	96833284	0.465502	PHC3
1	96733284	96833284	0.465502	GPR160
1	96735724	96835724	0.459698	LOC102179814
1	96735724	96835724	0.459698	PHC3
1	96735724	96835724	0.459698	GPR160
1	96736357	96836357	0.477396	LOC102179814
1	96736357	96836357	0.477396	PHC3
1	96736357	96836357	0.477396	GPR160
1	96736393	96836393	0.477639	LOC102179814
1	96736393	96836393	0.477639	PHC3
1	96736393	96836393	0.477639	GPR160
1	96737392	96837392	0.477639	LOC102179814
1	96737392	96837392	0.477639	PHC3
1	96737392	96837392	0.477639	GPR160
1	96737911	96837911	0.460178	LOC102179814
1	96737911	96837911	0.460178	PHC3

1	96737911	96837911	0.460178	GPR160
1	96738568	96838568	0.473712	LOC102179814
1	96738568	96838568	0.473712	PHC3
1	96738568	96838568	0.473712	GPR160
1	96738658	96838658	0.475276	LOC102179814
1	96738658	96838658	0.475276	PHC3
1	96738658	96838658	0.475276	GPR160
1	96739029	96839029	0.469369	LOC102179814
1	96739029	96839029	0.469369	PHC3
1	96739029	96839029	0.469369	GPR160
1	96739091	96839091	0.406708	LOC102179814
1	96739091	96839091	0.406708	PHC3
1	96739091	96839091	0.406708	GPR160
1	96741516	96841516	0.417792	LOC102179814
1	96741516	96841516	0.417792	PHC3
1	96741516	96841516	0.417792	GPR160
1	96741832	96841832	0.416442	LOC102179814
1	96741832	96841832	0.416442	PHC3
1	96741832	96841832	0.416442	GPR160
1	96741969	96841969	0.433316	LOC102179814
1	96741969	96841969	0.433316	PHC3
1	96741969	96841969	0.433316	GPR160
1	96743674	96843674	0.41337	LOC102179814
1	96743674	96843674	0.41337	PHC3
1	96743674	96843674	0.41337	GPR160
1	96744509	96844509	0.440448	LOC102179814
1	96744509	96844509	0.440448	PHC3
1	96744509	96844509	0.440448	GPR160
1	96744837	96844837	0.41684	LOC102179814
1	96744837	96844837	0.41684	PHC3
1	96744837	96844837	0.41684	GPR160
1	96745649	96845649	0.359691	LOC102179814
1	96745649	96845649	0.359691	PHC3
1	96745649	96845649	0.359691	GPR160
1	96746711	96846711	0.350836	LOC102179814
1	96746711	96846711	0.350836	PHC3
1	96746711	96846711	0.350836	GPR160
1	96749197	96849197	0.376483	LOC102179814
1	96749197	96849197	0.376483	PHC3
1	96749197	96849197	0.376483	GPR160
1	96749362	96849362	0.383729	LOC102179814
1	96749362	96849362	0.383729	PHC3
1	96749362	96849362	0.383729	GPR160
1	96750235	96850235	0.37364	LOC102179814
1	96750235	96850235	0.37364	PHC3
1	96750235	96850235	0.37364	GPR160
1	96751132	96851132	0.37451	LOC102179814
1	96751132	96851132	0.37451	PHC3
1	96751132	96851132	0.37451	GPR160
1	96752145	96852145	0.370707	LOC102179814
1	96752145	96852145	0.370707	PHC3
1	96752145	96852145	0.370707	GPR160
1	96752854	96852854	0.370974	LOC102179814
1	96752854	96852854	0.370974	PHC3
1	96752854	96852854	0.370974	GPR160
1	96752975	96852975	0.370974	LOC102179814
1	96752975	96852975	0.370974	PHC3
1	96752975	96852975	0.370974	GPR160

1	96753167	96853167	0.375217	LOC102179814
1	96753167	96853167	0.375217	PHC3
1	96753167	96853167	0.375217	GPR160
1	96756065	96856065	0.369409	LOC102179814
1	96756065	96856065	0.369409	PHC3
1	96756065	96856065	0.369409	GPR160
1	96756938	96856938	0.407367	LOC102179814
1	96756938	96856938	0.407367	PHC3
1	96756938	96856938	0.407367	GPR160
1	96758539	96858539	0.406427	LOC102179814
1	96758539	96858539	0.406427	PHC3
1	96758539	96858539	0.406427	GPR160
1	96758578	96858578	0.433725	LOC102179814
1	96758578	96858578	0.433725	PHC3
1	96758578	96858578	0.433725	GPR160
1	96758841	96858841	0.42409	LOC102179814
1	96758841	96858841	0.42409	PHC3
1	96758841	96858841	0.42409	GPR160
1	96759574	96859574	0.419643	LOC102179814
1	96759574	96859574	0.419643	PHC3
1	96759574	96859574	0.419643	GPR160
1	96761355	96861355	0.426698	LOC102179814
1	96761355	96861355	0.426698	PHC3
1	96761355	96861355	0.426698	GPR160
1	96762952	96862952	0.421518	LOC102179814
1	96762952	96862952	0.421518	PHC3
1	96762952	96862952	0.421518	GPR160
1	96762988	96862988	0.456519	LOC102179814
1	96762988	96862988	0.456519	PHC3
1	96762988	96862988	0.456519	GPR160
1	96763479	96863479	0.435048	LOC102179814
1	96763479	96863479	0.435048	PHC3
1	96763479	96863479	0.435048	GPR160
1	96763938	96863938	0.430602	LOC102179814
1	96763938	96863938	0.430602	PHC3
1	96763938	96863938	0.430602	GPR160
1	96764149	96864149	0.430602	LOC102179814
1	96764149	96864149	0.430602	PHC3
1	96764149	96864149	0.430602	GPR160
1	96764232	96864232	0.433012	LOC102179814
1	96764232	96864232	0.433012	PHC3
1	96764232	96864232	0.433012	GPR160
1	96764783	96864783	0.419373	LOC102179814
1	96764783	96864783	0.419373	PHC3
1	96764783	96864783	0.419373	GPR160
1	96765434	96865434	0.419373	LOC102179814
1	96765434	96865434	0.419373	PHC3
1	96765434	96865434	0.419373	GPR160
1	96765589	96865589	0.418181	LOC102179814
1	96765589	96865589	0.418181	PHC3
1	96765589	96865589	0.418181	GPR160
1	96767017	96867017	0.401289	LOC102179814
1	96767017	96867017	0.401289	PHC3
1	96767017	96867017	0.401289	GPR160
1	96768926	96868926	0.367845	LOC102179814
1	96768926	96868926	0.367845	PHC3
1	96768926	96868926	0.367845	GPR160
1	96769092	96869092	0.39201	LOC102179814

1	96769092	96869092	0.39201	PHC3
1	96769092	96869092	0.39201	GPR160
1	96769627	96869627	0.413113	LOC102179814
1	96769627	96869627	0.413113	PHC3
1	96769627	96869627	0.413113	GPR160
1	96770730	96870730	0.391488	LOC102179814
1	96770730	96870730	0.391488	PHC3
1	96770730	96870730	0.391488	GPR160
1	96777435	96877435	0.35272	LOC102179814
1	96777435	96877435	0.35272	LOC108636005
1	96777435	96877435	0.35272	PHC3
1	96777435	96877435	0.35272	GPR160
1	96777629	96877629	0.357707	LOC102179814
1	96777629	96877629	0.357707	LOC108636005
1	96777629	96877629	0.357707	PHC3
1	96777629	96877629	0.357707	GPR160
1	96777701	96877701	0.401745	LOC102179814
1	96777701	96877701	0.401745	LOC108636005
1	96777701	96877701	0.401745	PHC3
1	96777701	96877701	0.401745	GPR160
1	96777738	96877738	0.38079	LOC102179814
1	96777738	96877738	0.38079	LOC108636005
1	96777738	96877738	0.38079	PHC3
1	96777738	96877738	0.38079	GPR160
1	97018051	97118051	0.474877	TRNAY-GUA-3
1	97018051	97118051	0.474877	LRRC31
1	97018051	97118051	0.474877	LRRIQ4
1	97018051	97118051	0.474877	LRRC34
1	97018051	97118051	0.474877	MYNN
1	97018565	97118565	0.468254	TRNAY-GUA-3
1	97018565	97118565	0.468254	LRRC31
1	97018565	97118565	0.468254	LRRIQ4
1	97018565	97118565	0.468254	LRRC34
1	97018565	97118565	0.468254	MYNN
1	97018925	97118925	0.470671	TRNAY-GUA-3
1	97018925	97118925	0.470671	LRRC31
1	97018925	97118925	0.470671	LRRIQ4
1	97018925	97118925	0.470671	LRRC34
1	97018925	97118925	0.470671	MYNN
1	97019158	97119158	0.470663	TRNAY-GUA-3
1	97019158	97119158	0.470663	LRRC31
1	97019158	97119158	0.470663	LRRIQ4
1	97019158	97119158	0.470663	LRRC34
1	97019158	97119158	0.470663	MYNN
1	97019217	97119217	0.470663	TRNAY-GUA-3
1	97019217	97119217	0.470663	LRRC31
1	97019217	97119217	0.470663	LRRIQ4
1	97019217	97119217	0.470663	LRRC34
1	97019217	97119217	0.470663	MYNN
1	97019359	97119359	0.479472	TRNAY-GUA-3
1	97019359	97119359	0.479472	LRRC31
1	97019359	97119359	0.479472	LRRIQ4
1	97019359	97119359	0.479472	LRRC34
1	97019359	97119359	0.479472	MYNN
1	97019654	97119654	0.484013	TRNAY-GUA-3
1	97019654	97119654	0.484013	LRRC31
1	97019654	97119654	0.484013	LRRIQ4
1	97019654	97119654	0.484013	LRRC34

1	97019654	97119654	0.484013	MYNN
1	97019768	97119768	0.482446	TRNAY-GUA-3
1	97019768	97119768	0.482446	LRRC31
1	97019768	97119768	0.482446	LRRIQ4
1	97019768	97119768	0.482446	LRRC34
1	97019768	97119768	0.482446	MYNN
1	97019795	97119795	0.483344	TRNAY-GUA-3
1	97019795	97119795	0.483344	LRRC31
1	97019795	97119795	0.483344	LRRIQ4
1	97019795	97119795	0.483344	LRRC34
1	97019795	97119795	0.483344	MYNN
1	97019835	97119835	0.483657	TRNAY-GUA-3
1	97019835	97119835	0.483657	LRRC31
1	97019835	97119835	0.483657	LRRIQ4
1	97019835	97119835	0.483657	LRRC34
1	97019835	97119835	0.483657	MYNN
1	97020134	97120134	0.478077	TRNAY-GUA-3
1	97020134	97120134	0.478077	LRRC31
1	97020134	97120134	0.478077	LRRIQ4
1	97020134	97120134	0.478077	LRRC34
1	97020134	97120134	0.478077	MYNN
1	97020570	97120570	0.479938	TRNAY-GUA-3
1	97020570	97120570	0.479938	LRRC31
1	97020570	97120570	0.479938	LRRIQ4
1	97020570	97120570	0.479938	LRRC34
1	97020570	97120570	0.479938	MYNN
1	97020703	97120703	0.48004	TRNAY-GUA-3
1	97020703	97120703	0.48004	LRRC31
1	97020703	97120703	0.48004	LRRIQ4
1	97020703	97120703	0.48004	LRRC34
1	97020703	97120703	0.48004	MYNN
1	97020725	97120725	0.480149	TRNAY-GUA-3
1	97020725	97120725	0.480149	LRRC31
1	97020725	97120725	0.480149	LRRIQ4
1	97020725	97120725	0.480149	LRRC34
1	97020725	97120725	0.480149	MYNN
1	97020870	97120870	0.491819	TRNAY-GUA-3
1	97020870	97120870	0.491819	LRRC31
1	97020870	97120870	0.491819	LRRIQ4
1	97020870	97120870	0.491819	LRRC34
1	97020870	97120870	0.491819	MYNN
1	97020951	97120951	0.440396	TRNAY-GUA-3
1	97020951	97120951	0.440396	LRRC31
1	97020951	97120951	0.440396	LRRIQ4
1	97020951	97120951	0.440396	LRRC34
1	97020951	97120951	0.440396	MYNN
1	97020952	97120952	0.43154	TRNAY-GUA-3
1	97020952	97120952	0.43154	LRRC31
1	97020952	97120952	0.43154	LRRIQ4
1	97020952	97120952	0.43154	LRRC34
1	97020952	97120952	0.43154	MYNN
1	97021488	97121488	0.434713	TRNAY-GUA-3
1	97021488	97121488	0.434713	LRRC31
1	97021488	97121488	0.434713	LRRIQ4
1	97021488	97121488	0.434713	LRRC34
1	97021488	97121488	0.434713	MYNN
1	97021489	97121489	0.436476	TRNAY-GUA-3
1	97021489	97121489	0.436476	LRRC31

1	97021489	97121489	0.436476	LRRIQ4
1	97021489	97121489	0.436476	LRRC34
1	97021489	97121489	0.436476	MYNN
1	97021490	97121490	0.437289	TRNAY-GUA-3
1	97021490	97121490	0.437289	LRRC31
1	97021490	97121490	0.437289	LRRIQ4
1	97021490	97121490	0.437289	LRRC34
1	97021490	97121490	0.437289	MYNN
1	97021508	97121508	0.436621	TRNAY-GUA-3
1	97021508	97121508	0.436621	LRRC31
1	97021508	97121508	0.436621	LRRIQ4
1	97021508	97121508	0.436621	LRRC34
1	97021508	97121508	0.436621	MYNN
1	97021602	97121602	0.428571	TRNAY-GUA-3
1	97021602	97121602	0.428571	LRRC31
1	97021602	97121602	0.428571	LRRIQ4
1	97021602	97121602	0.428571	LRRC34
1	97021602	97121602	0.428571	MYNN
1	97021803	97121803	0.427477	TRNAY-GUA-3
1	97021803	97121803	0.427477	LRRC31
1	97021803	97121803	0.427477	LRRIQ4
1	97021803	97121803	0.427477	LRRC34
1	97021803	97121803	0.427477	MYNN
1	97022072	97122072	0.427477	TRNAY-GUA-3
1	97022072	97122072	0.427477	LRRC31
1	97022072	97122072	0.427477	LRRIQ4
1	97022072	97122072	0.427477	LRRC34
1	97022072	97122072	0.427477	MYNN
1	97022388	97122388	0.431846	TRNAY-GUA-3
1	97022388	97122388	0.431846	LRRC31
1	97022388	97122388	0.431846	LRRIQ4
1	97022388	97122388	0.431846	LRRC34
1	97022388	97122388	0.431846	MYNN
1	97022433	97122433	0.42711	TRNAY-GUA-3
1	97022433	97122433	0.42711	LRRC31
1	97022433	97122433	0.42711	LRRIQ4
1	97022433	97122433	0.42711	LRRC34
1	97022433	97122433	0.42711	MYNN
1	97023370	97123370	0.449465	TRNAY-GUA-3
1	97023370	97123370	0.449465	LRRC31
1	97023370	97123370	0.449465	LRRIQ4
1	97023370	97123370	0.449465	LRRC34
1	97023370	97123370	0.449465	MYNN
1	97023430	97123430	0.411797	TRNAY-GUA-3
1	97023430	97123430	0.411797	LRRC31
1	97023430	97123430	0.411797	LRRIQ4
1	97023430	97123430	0.411797	LRRC34
1	97023430	97123430	0.411797	MYNN
1	97023747	97123747	0.413453	TRNAY-GUA-3
1	97023747	97123747	0.413453	LRRC31
1	97023747	97123747	0.413453	LRRIQ4
1	97023747	97123747	0.413453	LRRC34
1	97023747	97123747	0.413453	MYNN
1	97023769	97123769	0.414475	TRNAY-GUA-3
1	97023769	97123769	0.414475	LRRC31
1	97023769	97123769	0.414475	LRRIQ4
1	97023769	97123769	0.414475	LRRC34
1	97023769	97123769	0.414475	MYNN

1	97023937	97123937	0.411637	TRNAY-GUA-3
1	97023937	97123937	0.411637	LRRC31
1	97023937	97123937	0.411637	LRRIQ4
1	97023937	97123937	0.411637	LRRC34
1	97023937	97123937	0.411637	MYNN
1	97024305	97124305	0.411637	TRNAY-GUA-3
1	97024305	97124305	0.411637	LRRC31
1	97024305	97124305	0.411637	LRRIQ4
1	97024305	97124305	0.411637	LRRC34
1	97024305	97124305	0.411637	MYNN
1	97024685	97124685	0.414534	TRNAY-GUA-3
1	97024685	97124685	0.414534	LRRC31
1	97024685	97124685	0.414534	LRRIQ4
1	97024685	97124685	0.414534	LRRC34
1	97024685	97124685	0.414534	MYNN
1	97024794	97124794	0.399077	TRNAY-GUA-3
1	97024794	97124794	0.399077	LRRC31
1	97024794	97124794	0.399077	LRRIQ4
1	97024794	97124794	0.399077	LRRC34
1	97024794	97124794	0.399077	MYNN
1	97025198	97125198	0.399673	TRNAY-GUA-3
1	97025198	97125198	0.399673	LRRC31
1	97025198	97125198	0.399673	LRRIQ4
1	97025198	97125198	0.399673	LRRC34
1	97025198	97125198	0.399673	MYNN
1	97025516	97125516	0.401429	TRNAY-GUA-3
1	97025516	97125516	0.401429	LRRC31
1	97025516	97125516	0.401429	LRRIQ4
1	97025516	97125516	0.401429	LRRC34
1	97025516	97125516	0.401429	MYNN
1	97026589	97126589	0.397331	TRNAY-GUA-3
1	97026589	97126589	0.397331	LRRC31
1	97026589	97126589	0.397331	LRRIQ4
1	97026589	97126589	0.397331	LRRC34
1	97026589	97126589	0.397331	MYNN
1	97026830	97126830	0.399433	TRNAY-GUA-3
1	97026830	97126830	0.399433	LRRC31
1	97026830	97126830	0.399433	LRRIQ4
1	97026830	97126830	0.399433	LRRC34
1	97026830	97126830	0.399433	MYNN
1	97026881	97126881	0.412089	TRNAY-GUA-3
1	97026881	97126881	0.412089	LRRC31
1	97026881	97126881	0.412089	LRRIQ4
1	97026881	97126881	0.412089	LRRC34
1	97026881	97126881	0.412089	MYNN
1	97026922	97126922	0.407821	TRNAY-GUA-3
1	97026922	97126922	0.407821	ACTRT3
1	97026922	97126922	0.407821	LRRC31
1	97026922	97126922	0.407821	LRRIQ4
1	97026922	97126922	0.407821	LRRC34
1	97026922	97126922	0.407821	MYNN
1	97026980	97126980	0.41193	TRNAY-GUA-3
1	97026980	97126980	0.41193	ACTRT3
1	97026980	97126980	0.41193	LRRC31
1	97026980	97126980	0.41193	LRRIQ4
1	97026980	97126980	0.41193	LRRC34
1	97026980	97126980	0.41193	MYNN
1	97026983	97126983	0.403964	TRNAY-GUA-3

1	97026983	97126983	0.403964	ACTRT3
1	97026983	97126983	0.403964	LRRC31
1	97026983	97126983	0.403964	LRRIQ4
1	97026983	97126983	0.403964	LRRC34
1	97026983	97126983	0.403964	MYNN
1	97027173	97127173	0.408017	TRNAY-GUA-3
1	97027173	97127173	0.408017	ACTRT3
1	97027173	97127173	0.408017	LRRC31
1	97027173	97127173	0.408017	LRRIQ4
1	97027173	97127173	0.408017	LRRC34
1	97027173	97127173	0.408017	MYNN
1	97027485	97127485	0.4026	TRNAY-GUA-3
1	97027485	97127485	0.4026	ACTRT3
1	97027485	97127485	0.4026	LRRC31
1	97027485	97127485	0.4026	LRRIQ4
1	97027485	97127485	0.4026	LRRC34
1	97027485	97127485	0.4026	MYNN
1	97027779	97127779	0.407962	TRNAY-GUA-3
1	97027779	97127779	0.407962	ACTRT3
1	97027779	97127779	0.407962	LRRC31
1	97027779	97127779	0.407962	LRRIQ4
1	97027779	97127779	0.407962	LRRC34
1	97027779	97127779	0.407962	MYNN
1	97027897	97127897	0.404018	TRNAY-GUA-3
1	97027897	97127897	0.404018	ACTRT3
1	97027897	97127897	0.404018	LRRC31
1	97027897	97127897	0.404018	LRRIQ4
1	97027897	97127897	0.404018	LRRC34
1	97027897	97127897	0.404018	MYNN
1	97027951	97127951	0.406557	TRNAY-GUA-3
1	97027951	97127951	0.406557	ACTRT3
1	97027951	97127951	0.406557	LRRC31
1	97027951	97127951	0.406557	LRRIQ4
1	97027951	97127951	0.406557	LRRC34
1	97027951	97127951	0.406557	MYNN
1	97028396	97128396	0.406557	TRNAY-GUA-3
1	97028396	97128396	0.406557	ACTRT3
1	97028396	97128396	0.406557	LRRC31
1	97028396	97128396	0.406557	LRRIQ4
1	97028396	97128396	0.406557	LRRC34
1	97028396	97128396	0.406557	MYNN
1	97028763	97128763	0.408289	TRNAY-GUA-3
1	97028763	97128763	0.408289	ACTRT3
1	97028763	97128763	0.408289	LRRC31
1	97028763	97128763	0.408289	LRRIQ4
1	97028763	97128763	0.408289	LRRC34
1	97028763	97128763	0.408289	MYNN
1	97029273	97129273	0.40519	TRNAY-GUA-3
1	97029273	97129273	0.40519	ACTRT3
1	97029273	97129273	0.40519	TRNAV-AAC
1	97029273	97129273	0.40519	LRRC31
1	97029273	97129273	0.40519	LRRIQ4
1	97029273	97129273	0.40519	LRRC34
1	97029273	97129273	0.40519	MYNN
1	97029341	97129341	0.40519	TRNAY-GUA-3
1	97029341	97129341	0.40519	ACTRT3
1	97029341	97129341	0.40519	TRNAV-AAC
1	97029341	97129341	0.40519	LRRC31

1	97029341	97129341	0.40519	LRRIQ4
1	97029341	97129341	0.40519	LRRC34
1	97029341	97129341	0.40519	MYNN
1	97029479	97129479	0.40519	TRNAY-GUA-3
1	97029479	97129479	0.40519	ACTRT3
1	97029479	97129479	0.40519	TRNAV-AAC
1	97029479	97129479	0.40519	LRRC31
1	97029479	97129479	0.40519	LRRIQ4
1	97029479	97129479	0.40519	LRRC34
1	97029479	97129479	0.40519	MYNN
1	97029573	97129573	0.409643	TRNAY-GUA-3
1	97029573	97129573	0.409643	ACTRT3
1	97029573	97129573	0.409643	TRNAV-AAC
1	97029573	97129573	0.409643	LRRC31
1	97029573	97129573	0.409643	LRRIQ4
1	97029573	97129573	0.409643	LRRC34
1	97029573	97129573	0.409643	MYNN
1	97029704	97129704	0.409643	TRNAY-GUA-3
1	97029704	97129704	0.409643	ACTRT3
1	97029704	97129704	0.409643	TRNAV-AAC
1	97029704	97129704	0.409643	LRRC31
1	97029704	97129704	0.409643	LRRIQ4
1	97029704	97129704	0.409643	LRRC34
1	97029704	97129704	0.409643	MYNN
1	97029816	97129816	0.401774	TRNAY-GUA-3
1	97029816	97129816	0.401774	ACTRT3
1	97029816	97129816	0.401774	TRNAV-AAC
1	97029816	97129816	0.401774	LRRC31
1	97029816	97129816	0.401774	LRRIQ4
1	97029816	97129816	0.401774	LRRC34
1	97029816	97129816	0.401774	MYNN
1	97029855	97129855	0.395811	TRNAY-GUA-3
1	97029855	97129855	0.395811	ACTRT3
1	97029855	97129855	0.395811	TRNAV-AAC
1	97029855	97129855	0.395811	LRRC31
1	97029855	97129855	0.395811	LRRIQ4
1	97029855	97129855	0.395811	LRRC34
1	97029855	97129855	0.395811	MYNN
1	97030581	97130581	0.387319	TRNAY-GUA-3
1	97030581	97130581	0.387319	ACTRT3
1	97030581	97130581	0.387319	TRNAV-AAC
1	97030581	97130581	0.387319	LRRC31
1	97030581	97130581	0.387319	LRRIQ4
1	97030581	97130581	0.387319	LRRC34
1	97030581	97130581	0.387319	MYNN
1	97030756	97130756	0.40374	TRNAY-GUA-3
1	97030756	97130756	0.40374	ACTRT3
1	97030756	97130756	0.40374	TRNAV-AAC
1	97030756	97130756	0.40374	LRRC31
1	97030756	97130756	0.40374	LRRIQ4
1	97030756	97130756	0.40374	LRRC34
1	97030756	97130756	0.40374	MYNN
1	97031688	97131688	0.383496	TRNAY-GUA-3
1	97031688	97131688	0.383496	ACTRT3
1	97031688	97131688	0.383496	TRNAV-AAC
1	97031688	97131688	0.383496	LRRC31
1	97031688	97131688	0.383496	LRRIQ4
1	97031688	97131688	0.383496	LRRC34

1	97031688	97131688	0.383496	MYNN
1	97031887	97131887	0.391716	TRNAY-GUA-3
1	97031887	97131887	0.391716	ACTRT3
1	97031887	97131887	0.391716	TRNAV-AAC
1	97031887	97131887	0.391716	LRRC31
1	97031887	97131887	0.391716	LRRIQ4
1	97031887	97131887	0.391716	LRRC34
1	97031887	97131887	0.391716	MYNN
1	97032163	97132163	0.391716	TRNAY-GUA-3
1	97032163	97132163	0.391716	ACTRT3
1	97032163	97132163	0.391716	TRNAV-AAC
1	97032163	97132163	0.391716	LRRC31
1	97032163	97132163	0.391716	LRRIQ4
1	97032163	97132163	0.391716	LRRC34
1	97032163	97132163	0.391716	MYNN
1	97032284	97132284	0.400989	TRNAY-GUA-3
1	97032284	97132284	0.400989	ACTRT3
1	97032284	97132284	0.400989	TRNAV-AAC
1	97032284	97132284	0.400989	LRRC31
1	97032284	97132284	0.400989	LRRIQ4
1	97032284	97132284	0.400989	LRRC34
1	97032284	97132284	0.400989	MYNN
1	97032421	97132421	0.394391	TRNAY-GUA-3
1	97032421	97132421	0.394391	ACTRT3
1	97032421	97132421	0.394391	TRNAV-AAC
1	97032421	97132421	0.394391	LRRC31
1	97032421	97132421	0.394391	LRRIQ4
1	97032421	97132421	0.394391	LRRC34
1	97032421	97132421	0.394391	MYNN
1	97032699	97132699	0.380633	TRNAY-GUA-3
1	97032699	97132699	0.380633	ACTRT3
1	97032699	97132699	0.380633	TRNAV-AAC
1	97032699	97132699	0.380633	LRRC31
1	97032699	97132699	0.380633	LRRIQ4
1	97032699	97132699	0.380633	LRRC34
1	97032699	97132699	0.380633	MYNN
1	97032899	97132899	0.382388	TRNAY-GUA-3
1	97032899	97132899	0.382388	ACTRT3
1	97032899	97132899	0.382388	TRNAV-AAC
1	97032899	97132899	0.382388	LRRC31
1	97032899	97132899	0.382388	LRRIQ4
1	97032899	97132899	0.382388	LRRC34
1	97032899	97132899	0.382388	MYNN
1	97033750	97133750	0.391903	TRNAY-GUA-3
1	97033750	97133750	0.391903	ACTRT3
1	97033750	97133750	0.391903	TRNAV-AAC
1	97033750	97133750	0.391903	LRRC31
1	97033750	97133750	0.391903	LRRIQ4
1	97033750	97133750	0.391903	LRRC34
1	97033750	97133750	0.391903	MYNN
1	97033829	97133829	0.391903	TRNAY-GUA-3
1	97033829	97133829	0.391903	ACTRT3
1	97033829	97133829	0.391903	TRNAV-AAC
1	97033829	97133829	0.391903	LRRC31
1	97033829	97133829	0.391903	LRRIQ4
1	97033829	97133829	0.391903	LRRC34
1	97033829	97133829	0.391903	MYNN
1	97034255	97134255	0.393179	TRNAY-GUA-3

1	97034255	97134255	0.393179	ACTRT3
1	97034255	97134255	0.393179	TRNAV-AAC
1	97034255	97134255	0.393179	LRRC31
1	97034255	97134255	0.393179	LRRIQ4
1	97034255	97134255	0.393179	LRRC34
1	97034255	97134255	0.393179	MYNN
1	97034266	97134266	0.403651	TRNAY-GUA-3
1	97034266	97134266	0.403651	ACTRT3
1	97034266	97134266	0.403651	TRNAV-AAC
1	97034266	97134266	0.403651	LRRC31
1	97034266	97134266	0.403651	LRRIQ4
1	97034266	97134266	0.403651	LRRC34
1	97034266	97134266	0.403651	MYNN
1	97034644	97134644	0.398417	TRNAY-GUA-3
1	97034644	97134644	0.398417	ACTRT3
1	97034644	97134644	0.398417	TRNAV-AAC
1	97034644	97134644	0.398417	LRRC31
1	97034644	97134644	0.398417	LRRIQ4
1	97034644	97134644	0.398417	LRRC34
1	97034644	97134644	0.398417	MYNN
1	97035040	97135040	0.401798	TRNAY-GUA-3
1	97035040	97135040	0.401798	ACTRT3
1	97035040	97135040	0.401798	TRNAV-AAC
1	97035040	97135040	0.401798	LRRC31
1	97035040	97135040	0.401798	LRRIQ4
1	97035040	97135040	0.401798	LRRC34
1	97035040	97135040	0.401798	MYNN
1	97036015	97136015	0.391417	TRNAY-GUA-3
1	97036015	97136015	0.391417	ACTRT3
1	97036015	97136015	0.391417	TRNAV-AAC
1	97036015	97136015	0.391417	LRRC31
1	97036015	97136015	0.391417	LRRIQ4
1	97036015	97136015	0.391417	LRRC34
1	97036015	97136015	0.391417	MYNN
1	97036162	97136162	0.390645	TRNAY-GUA-3
1	97036162	97136162	0.390645	ACTRT3
1	97036162	97136162	0.390645	TRNAV-AAC
1	97036162	97136162	0.390645	LRRC31
1	97036162	97136162	0.390645	LRRIQ4
1	97036162	97136162	0.390645	LRRC34
1	97036162	97136162	0.390645	MYNN
1	97036186	97136186	0.39301	TRNAY-GUA-3
1	97036186	97136186	0.39301	ACTRT3
1	97036186	97136186	0.39301	TRNAV-AAC
1	97036186	97136186	0.39301	LRRC31
1	97036186	97136186	0.39301	LRRIQ4
1	97036186	97136186	0.39301	LRRC34
1	97036186	97136186	0.39301	MYNN
1	97036471	97136471	0.396922	TRNAY-GUA-3
1	97036471	97136471	0.396922	ACTRT3
1	97036471	97136471	0.396922	TRNAV-AAC
1	97036471	97136471	0.396922	LRRC31
1	97036471	97136471	0.396922	LRRIQ4
1	97036471	97136471	0.396922	LRRC34
1	97036471	97136471	0.396922	MYNN
1	97037410	97137410	0.387027	TRNAY-GUA-3
1	97037410	97137410	0.387027	ACTRT3
1	97037410	97137410	0.387027	TRNAV-AAC

1	97037410	97137410	0.387027	LRRC31
1	97037410	97137410	0.387027	LRRIQ4
1	97037410	97137410	0.387027	LRRC34
1	97037410	97137410	0.387027	MYNN
1	97037461	97137461	0.388865	TRNAY-GUA-3
1	97037461	97137461	0.388865	ACTRT3
1	97037461	97137461	0.388865	TRNAV-AAC
1	97037461	97137461	0.388865	LRRC31
1	97037461	97137461	0.388865	LRRIQ4
1	97037461	97137461	0.388865	LRRC34
1	97037461	97137461	0.388865	MYNN
1	97037563	97137563	0.395254	TRNAY-GUA-3
1	97037563	97137563	0.395254	ACTRT3
1	97037563	97137563	0.395254	TRNAV-AAC
1	97037563	97137563	0.395254	LRRC31
1	97037563	97137563	0.395254	LRRIQ4
1	97037563	97137563	0.395254	LRRC34
1	97037563	97137563	0.395254	MYNN
1	97037679	97137679	0.389781	TRNAY-GUA-3
1	97037679	97137679	0.389781	ACTRT3
1	97037679	97137679	0.389781	TRNAV-AAC
1	97037679	97137679	0.389781	LRRC31
1	97037679	97137679	0.389781	LRRIQ4
1	97037679	97137679	0.389781	LRRC34
1	97037679	97137679	0.389781	MYNN
1	97037680	97137680	0.389781	TRNAY-GUA-3
1	97037680	97137680	0.389781	ACTRT3
1	97037680	97137680	0.389781	TRNAV-AAC
1	97037680	97137680	0.389781	LRRC31
1	97037680	97137680	0.389781	LRRIQ4
1	97037680	97137680	0.389781	LRRC34
1	97037680	97137680	0.389781	MYNN
1	97037742	97137742	0.403123	TRNAY-GUA-3
1	97037742	97137742	0.403123	ACTRT3
1	97037742	97137742	0.403123	TRNAV-AAC
1	97037742	97137742	0.403123	LRRC31
1	97037742	97137742	0.403123	LRRIQ4
1	97037742	97137742	0.403123	LRRC34
1	97037742	97137742	0.403123	MYNN
1	97037800	97137800	0.399457	TRNAY-GUA-3
1	97037800	97137800	0.399457	ACTRT3
1	97037800	97137800	0.399457	TRNAV-AAC
1	97037800	97137800	0.399457	LRRC31
1	97037800	97137800	0.399457	LRRIQ4
1	97037800	97137800	0.399457	LRRC34
1	97037800	97137800	0.399457	MYNN
1	97037927	97137927	0.397511	TRNAY-GUA-3
1	97037927	97137927	0.397511	ACTRT3
1	97037927	97137927	0.397511	TRNAV-AAC
1	97037927	97137927	0.397511	LRRC31
1	97037927	97137927	0.397511	LRRIQ4
1	97037927	97137927	0.397511	LRRC34
1	97037927	97137927	0.397511	MYNN
1	97037939	97137939	0.39053	TRNAY-GUA-3
1	97037939	97137939	0.39053	ACTRT3
1	97037939	97137939	0.39053	TRNAV-AAC
1	97037939	97137939	0.39053	LRRC31
1	97037939	97137939	0.39053	LRRIQ4

1	97037939	97137939	0.39053	LRRC34
1	97037939	97137939	0.39053	MYNN
1	97038179	97138179	0.389588	TRNAY-GUA-3
1	97038179	97138179	0.389588	ACTRT3
1	97038179	97138179	0.389588	TRNAV-AAC
1	97038179	97138179	0.389588	LRRC31
1	97038179	97138179	0.389588	LRRIQ4
1	97038179	97138179	0.389588	LRRC34
1	97038179	97138179	0.389588	MYNN
1	97038581	97138581	0.397143	TRNAY-GUA-3
1	97038581	97138581	0.397143	ACTRT3
1	97038581	97138581	0.397143	TRNAV-AAC
1	97038581	97138581	0.397143	LRRC31
1	97038581	97138581	0.397143	LRRIQ4
1	97038581	97138581	0.397143	LRRC34
1	97038581	97138581	0.397143	MYNN
1	97038621	97138621	0.397302	TRNAY-GUA-3
1	97038621	97138621	0.397302	ACTRT3
1	97038621	97138621	0.397302	TRNAV-AAC
1	97038621	97138621	0.397302	LRRC31
1	97038621	97138621	0.397302	LRRIQ4
1	97038621	97138621	0.397302	LRRC34
1	97038621	97138621	0.397302	MYNN
1	97038796	97138796	0.388288	TRNAY-GUA-3
1	97038796	97138796	0.388288	ACTRT3
1	97038796	97138796	0.388288	TRNAV-AAC
1	97038796	97138796	0.388288	LRRC31
1	97038796	97138796	0.388288	LRRIQ4
1	97038796	97138796	0.388288	LRRC34
1	97038796	97138796	0.388288	MYNN
1	97038803	97138803	0.38844	TRNAY-GUA-3
1	97038803	97138803	0.38844	ACTRT3
1	97038803	97138803	0.38844	TRNAV-AAC
1	97038803	97138803	0.38844	LRRC31
1	97038803	97138803	0.38844	LRRIQ4
1	97038803	97138803	0.38844	LRRC34
1	97038803	97138803	0.38844	MYNN
1	97039165	97139165	0.389177	TRNAY-GUA-3
1	97039165	97139165	0.389177	ACTRT3
1	97039165	97139165	0.389177	TRNAV-AAC
1	97039165	97139165	0.389177	LRRC31
1	97039165	97139165	0.389177	LRRIQ4
1	97039165	97139165	0.389177	LRRC34
1	97039165	97139165	0.389177	MYNN
1	97040544	97140544	0.389502	TRNAY-GUA-3
1	97040544	97140544	0.389502	ACTRT3
1	97040544	97140544	0.389502	TRNAV-AAC
1	97040544	97140544	0.389502	LRRC31
1	97040544	97140544	0.389502	LRRIQ4
1	97040544	97140544	0.389502	LRRC34
1	97040544	97140544	0.389502	MYNN
1	97040607	97140607	0.389324	TRNAY-GUA-3
1	97040607	97140607	0.389324	ACTRT3
1	97040607	97140607	0.389324	TRNAV-AAC
1	97040607	97140607	0.389324	LRRC31
1	97040607	97140607	0.389324	LRRIQ4
1	97040607	97140607	0.389324	LRRC34
1	97040607	97140607	0.389324	MYNN

1	97040616	97140616	0.390385	TRNAY-GUA-3
1	97040616	97140616	0.390385	ACTRT3
1	97040616	97140616	0.390385	TRNAV-AAC
1	97040616	97140616	0.390385	LRRC31
1	97040616	97140616	0.390385	LRRIQ4
1	97040616	97140616	0.390385	LRRC34
1	97040616	97140616	0.390385	MYNN
1	97040697	97140697	0.389908	TRNAY-GUA-3
1	97040697	97140697	0.389908	ACTRT3
1	97040697	97140697	0.389908	TRNAV-AAC
1	97040697	97140697	0.389908	LRRC31
1	97040697	97140697	0.389908	LRRIQ4
1	97040697	97140697	0.389908	LRRC34
1	97040697	97140697	0.389908	MYNN
1	97041525	97141525	0.388228	TRNAY-GUA-3
1	97041525	97141525	0.388228	ACTRT3
1	97041525	97141525	0.388228	TRNAV-AAC
1	97041525	97141525	0.388228	LRRC31
1	97041525	97141525	0.388228	LRRIQ4
1	97041525	97141525	0.388228	LRRC34
1	97041525	97141525	0.388228	MYNN
1	97041526	97141526	0.396704	TRNAY-GUA-3
1	97041526	97141526	0.396704	ACTRT3
1	97041526	97141526	0.396704	TRNAV-AAC
1	97041526	97141526	0.396704	LRRC31
1	97041526	97141526	0.396704	LRRIQ4
1	97041526	97141526	0.396704	LRRC34
1	97041526	97141526	0.396704	MYNN
1	97041527	97141527	0.394495	TRNAY-GUA-3
1	97041527	97141527	0.394495	ACTRT3
1	97041527	97141527	0.394495	TRNAV-AAC
1	97041527	97141527	0.394495	LRRC31
1	97041527	97141527	0.394495	LRRIQ4
1	97041527	97141527	0.394495	LRRC34
1	97041527	97141527	0.394495	MYNN
1	97042300	97142300	0.397811	TRNAY-GUA-3
1	97042300	97142300	0.397811	ACTRT3
1	97042300	97142300	0.397811	TRNAV-AAC
1	97042300	97142300	0.397811	LRRC31
1	97042300	97142300	0.397811	LRRIQ4
1	97042300	97142300	0.397811	LRRC34
1	97042300	97142300	0.397811	MYNN
1	97042841	97142841	0.395786	TRNAY-GUA-3
1	97042841	97142841	0.395786	ACTRT3
1	97042841	97142841	0.395786	TRNAV-AAC
1	97042841	97142841	0.395786	LRRC31
1	97042841	97142841	0.395786	LRRIQ4
1	97042841	97142841	0.395786	LRRC34
1	97042841	97142841	0.395786	MYNN
1	97043364	97143364	0.389079	TRNAY-GUA-3
1	97043364	97143364	0.389079	ACTRT3
1	97043364	97143364	0.389079	TRNAV-AAC
1	97043364	97143364	0.389079	LRRC31
1	97043364	97143364	0.389079	LRRIQ4
1	97043364	97143364	0.389079	LRRC34
1	97043364	97143364	0.389079	MYNN
1	97043378	97143378	0.404663	TRNAY-GUA-3
1	97043378	97143378	0.404663	ACTRT3

1	97043378	97143378	0.404663	TRNAV-AAC
1	97043378	97143378	0.404663	LRRC31
1	97043378	97143378	0.404663	LRRIQ4
1	97043378	97143378	0.404663	LRRC34
1	97043378	97143378	0.404663	MYNN
1	97043379	97143379	0.404663	TRNAY-GUA-3
1	97043379	97143379	0.404663	ACTRT3
1	97043379	97143379	0.404663	TRNAV-AAC
1	97043379	97143379	0.404663	LRRC31
1	97043379	97143379	0.404663	LRRIQ4
1	97043379	97143379	0.404663	LRRC34
1	97043379	97143379	0.404663	MYNN
1	97043646	97143646	0.408803	TRNAY-GUA-3
1	97043646	97143646	0.408803	ACTRT3
1	97043646	97143646	0.408803	TRNAV-AAC
1	97043646	97143646	0.408803	LRRC31
1	97043646	97143646	0.408803	LRRIQ4
1	97043646	97143646	0.408803	LRRC34
1	97043646	97143646	0.408803	MYNN
1	97044311	97144311	0.398665	TRNAY-GUA-3
1	97044311	97144311	0.398665	ACTRT3
1	97044311	97144311	0.398665	TRNAV-AAC
1	97044311	97144311	0.398665	LRRC31
1	97044311	97144311	0.398665	LRRIQ4
1	97044311	97144311	0.398665	LRRC34
1	97044311	97144311	0.398665	MYNN
1	97044440	97144440	0.402079	TRNAY-GUA-3
1	97044440	97144440	0.402079	ACTRT3
1	97044440	97144440	0.402079	TRNAV-AAC
1	97044440	97144440	0.402079	LRRC31
1	97044440	97144440	0.402079	LRRIQ4
1	97044440	97144440	0.402079	LRRC34
1	97044440	97144440	0.402079	MYNN
1	97045048	97145048	0.392407	TRNAY-GUA-3
1	97045048	97145048	0.392407	ACTRT3
1	97045048	97145048	0.392407	TRNAV-AAC
1	97045048	97145048	0.392407	LRRC31
1	97045048	97145048	0.392407	LRRIQ4
1	97045048	97145048	0.392407	LRRC34
1	97045048	97145048	0.392407	MYNN
1	97045801	97145801	0.392673	TRNAY-GUA-3
1	97045801	97145801	0.392673	ACTRT3
1	97045801	97145801	0.392673	TRNAV-AAC
1	97045801	97145801	0.392673	LRRC31
1	97045801	97145801	0.392673	LRRIQ4
1	97045801	97145801	0.392673	LRRC34
1	97045801	97145801	0.392673	MYNN
1	97045982	97145982	0.411441	TRNAY-GUA-3
1	97045982	97145982	0.411441	ACTRT3
1	97045982	97145982	0.411441	TRNAV-AAC
1	97045982	97145982	0.411441	LRRC31
1	97045982	97145982	0.411441	LRRIQ4
1	97045982	97145982	0.411441	LRRC34
1	97045982	97145982	0.411441	MYNN
1	97046182	97146182	0.405613	TRNAY-GUA-3
1	97046182	97146182	0.405613	ACTRT3
1	97046182	97146182	0.405613	TRNAV-AAC
1	97046182	97146182	0.405613	LRRC31

1	97046182	97146182	0.405613	LRRIQ4
1	97046182	97146182	0.405613	LRRC34
1	97046182	97146182	0.405613	MYNN
1	97046258	97146258	0.407557	TRNAY-GUA-3
1	97046258	97146258	0.407557	ACTRT3
1	97046258	97146258	0.407557	TRNAV-AAC
1	97046258	97146258	0.407557	LRRC31
1	97046258	97146258	0.407557	LRRIQ4
1	97046258	97146258	0.407557	LRRC34
1	97046258	97146258	0.407557	MYNN
1	97046310	97146310	0.404245	TRNAY-GUA-3
1	97046310	97146310	0.404245	ACTRT3
1	97046310	97146310	0.404245	TRNAV-AAC
1	97046310	97146310	0.404245	LRRC31
1	97046310	97146310	0.404245	LRRIQ4
1	97046310	97146310	0.404245	LRRC34
1	97046310	97146310	0.404245	MYNN
1	97046348	97146348	0.405687	TRNAY-GUA-3
1	97046348	97146348	0.405687	ACTRT3
1	97046348	97146348	0.405687	TRNAV-AAC
1	97046348	97146348	0.405687	LRRC31
1	97046348	97146348	0.405687	LRRIQ4
1	97046348	97146348	0.405687	LRRC34
1	97046348	97146348	0.405687	MYNN
1	97046603	97146603	0.400754	TRNAY-GUA-3
1	97046603	97146603	0.400754	ACTRT3
1	97046603	97146603	0.400754	TRNAV-AAC
1	97046603	97146603	0.400754	LRRC31
1	97046603	97146603	0.400754	LRRIQ4
1	97046603	97146603	0.400754	LRRC34
1	97046603	97146603	0.400754	MYNN
1	97046791	97146791	0.404881	TRNAY-GUA-3
1	97046791	97146791	0.404881	ACTRT3
1	97046791	97146791	0.404881	TRNAV-AAC
1	97046791	97146791	0.404881	LRRC31
1	97046791	97146791	0.404881	LRRIQ4
1	97046791	97146791	0.404881	LRRC34
1	97046791	97146791	0.404881	MYNN
1	97047177	97147177	0.389368	TRNAY-GUA-3
1	97047177	97147177	0.389368	ACTRT3
1	97047177	97147177	0.389368	TRNAV-AAC
1	97047177	97147177	0.389368	LRRC31
1	97047177	97147177	0.389368	LRRIQ4
1	97047177	97147177	0.389368	LRRC34
1	97047177	97147177	0.389368	MYNN
1	97048629	97148629	0.381368	TRNAY-GUA-3
1	97048629	97148629	0.381368	ACTRT3
1	97048629	97148629	0.381368	TRNAV-AAC
1	97048629	97148629	0.381368	LRRC31
1	97048629	97148629	0.381368	LRRIQ4
1	97048629	97148629	0.381368	LRRC34
1	97048629	97148629	0.381368	MYNN
1	97048705	97148705	0.381883	TRNAY-GUA-3
1	97048705	97148705	0.381883	ACTRT3
1	97048705	97148705	0.381883	TRNAV-AAC
1	97048705	97148705	0.381883	LRRC31
1	97048705	97148705	0.381883	LRRIQ4
1	97048705	97148705	0.381883	LRRC34

1	97048705	97148705	0.381883	MYNN
1	97048810	97148810	0.384879	TRNAY-GUA-3
1	97048810	97148810	0.384879	ACTRT3
1	97048810	97148810	0.384879	TRNAV-AAC
1	97048810	97148810	0.384879	LRRC31
1	97048810	97148810	0.384879	LRRIQ4
1	97048810	97148810	0.384879	LRRC34
1	97048810	97148810	0.384879	MYNN
1	97048994	97148994	0.392707	TRNAY-GUA-3
1	97048994	97148994	0.392707	ACTRT3
1	97048994	97148994	0.392707	TRNAV-AAC
1	97048994	97148994	0.392707	LRRC31
1	97048994	97148994	0.392707	LRRIQ4
1	97048994	97148994	0.392707	LRRC34
1	97048994	97148994	0.392707	MYNN
1	97049114	97149114	0.392638	TRNAY-GUA-3
1	97049114	97149114	0.392638	ACTRT3
1	97049114	97149114	0.392638	TRNAV-AAC
1	97049114	97149114	0.392638	LRRC31
1	97049114	97149114	0.392638	LRRIQ4
1	97049114	97149114	0.392638	LRRC34
1	97049114	97149114	0.392638	MYNN
1	97049218	97149218	0.398792	TRNAY-GUA-3
1	97049218	97149218	0.398792	ACTRT3
1	97049218	97149218	0.398792	TRNAV-AAC
1	97049218	97149218	0.398792	LRRC31
1	97049218	97149218	0.398792	LRRIQ4
1	97049218	97149218	0.398792	LRRC34
1	97049218	97149218	0.398792	MYNN
1	97049230	97149230	0.386322	TRNAY-GUA-3
1	97049230	97149230	0.386322	ACTRT3
1	97049230	97149230	0.386322	TRNAV-AAC
1	97049230	97149230	0.386322	LRRC31
1	97049230	97149230	0.386322	LRRIQ4
1	97049230	97149230	0.386322	LRRC34
1	97049230	97149230	0.386322	MYNN
1	97050001	97150001	0.399311	TRNAY-GUA-3
1	97050001	97150001	0.399311	ACTRT3
1	97050001	97150001	0.399311	TRNAV-AAC
1	97050001	97150001	0.399311	LRRC31
1	97050001	97150001	0.399311	LRRIQ4
1	97050001	97150001	0.399311	LRRC34
1	97050001	97150001	0.399311	MYNN
1	97050095	97150095	0.390892	TRNAY-GUA-3
1	97050095	97150095	0.390892	ACTRT3
1	97050095	97150095	0.390892	TRNAV-AAC
1	97050095	97150095	0.390892	LRRC31
1	97050095	97150095	0.390892	LRRIQ4
1	97050095	97150095	0.390892	LRRC34
1	97050095	97150095	0.390892	MYNN
1	97050184	97150184	0.387004	TRNAY-GUA-3
1	97050184	97150184	0.387004	ACTRT3
1	97050184	97150184	0.387004	TRNAV-AAC
1	97050184	97150184	0.387004	LRRC31
1	97050184	97150184	0.387004	LRRIQ4
1	97050184	97150184	0.387004	LRRC34
1	97050184	97150184	0.387004	MYNN
1	97050412	97150412	0.390863	TRNAY-GUA-3

1	97050412	97150412	0.390863	ACTRT3
1	97050412	97150412	0.390863	TRNAV-AAC
1	97050412	97150412	0.390863	LRRC31
1	97050412	97150412	0.390863	LRRIQ4
1	97050412	97150412	0.390863	LRRC34
1	97050412	97150412	0.390863	MYNN
1	97050435	97150435	0.409198	TRNAY-GUA-3
1	97050435	97150435	0.409198	ACTRT3
1	97050435	97150435	0.409198	TRNAV-AAC
1	97050435	97150435	0.409198	LRRC31
1	97050435	97150435	0.409198	LRRIQ4
1	97050435	97150435	0.409198	LRRC34
1	97050435	97150435	0.409198	MYNN
1	97050659	97150659	0.398897	TRNAY-GUA-3
1	97050659	97150659	0.398897	ACTRT3
1	97050659	97150659	0.398897	TRNAV-AAC
1	97050659	97150659	0.398897	LRRC31
1	97050659	97150659	0.398897	LRRIQ4
1	97050659	97150659	0.398897	LRRC34
1	97050659	97150659	0.398897	MYNN
1	97051204	97151204	0.386963	TRNAY-GUA-3
1	97051204	97151204	0.386963	ACTRT3
1	97051204	97151204	0.386963	TRNAV-AAC
1	97051204	97151204	0.386963	LRRC31
1	97051204	97151204	0.386963	LRRIQ4
1	97051204	97151204	0.386963	LRRC34
1	97051204	97151204	0.386963	MYNN
1	97051358	97151358	0.395871	TRNAY-GUA-3
1	97051358	97151358	0.395871	ACTRT3
1	97051358	97151358	0.395871	TRNAV-AAC
1	97051358	97151358	0.395871	LRRC31
1	97051358	97151358	0.395871	LRRIQ4
1	97051358	97151358	0.395871	LRRC34
1	97051358	97151358	0.395871	MYNN
1	97053175	97153175	0.395871	TRNAY-GUA-3
1	97053175	97153175	0.395871	ACTRT3
1	97053175	97153175	0.395871	TRNAV-AAC
1	97053175	97153175	0.395871	LRRC31
1	97053175	97153175	0.395871	LRRIQ4
1	97053175	97153175	0.395871	LRRC34
1	97053175	97153175	0.395871	MYNN
1	97053252	97153252	0.392697	TRNAY-GUA-3
1	97053252	97153252	0.392697	ACTRT3
1	97053252	97153252	0.392697	TRNAV-AAC
1	97053252	97153252	0.392697	LRRC31
1	97053252	97153252	0.392697	LRRIQ4
1	97053252	97153252	0.392697	LRRC34
1	97053252	97153252	0.392697	MYNN
1	97053646	97153646	0.385065	TRNAY-GUA-3
1	97053646	97153646	0.385065	ACTRT3
1	97053646	97153646	0.385065	TRNAV-AAC
1	97053646	97153646	0.385065	LRRC31
1	97053646	97153646	0.385065	LRRIQ4
1	97053646	97153646	0.385065	LRRC34
1	97053646	97153646	0.385065	MYNN
1	97054335	97154335	0.386752	TRNAY-GUA-3
1	97054335	97154335	0.386752	ACTRT3
1	97054335	97154335	0.386752	TRNAV-AAC

1	97054335	97154335	0.386752	LRRC31
1	97054335	97154335	0.386752	LRRIQ4
1	97054335	97154335	0.386752	LRRC34
1	97054335	97154335	0.386752	MYNN
1	97054426	97154426	0.450909	TRNAY-GUA-3
1	97054426	97154426	0.450909	ACTRT3
1	97054426	97154426	0.450909	TRNAV-AAC
1	97054426	97154426	0.450909	LRRC31
1	97054426	97154426	0.450909	LRRIQ4
1	97054426	97154426	0.450909	LRRC34
1	97054426	97154426	0.450909	MYNN
1	97054631	97154631	0.388538	TRNAY-GUA-3
1	97054631	97154631	0.388538	ACTRT3
1	97054631	97154631	0.388538	TRNAV-AAC
1	97054631	97154631	0.388538	LRRC31
1	97054631	97154631	0.388538	LRRIQ4
1	97054631	97154631	0.388538	LRRC34
1	97054631	97154631	0.388538	MYNN
1	97054712	97154712	0.390039	TRNAY-GUA-3
1	97054712	97154712	0.390039	ACTRT3
1	97054712	97154712	0.390039	TRNAV-AAC
1	97054712	97154712	0.390039	LRRC31
1	97054712	97154712	0.390039	LRRIQ4
1	97054712	97154712	0.390039	LRRC34
1	97054712	97154712	0.390039	MYNN
1	97054753	97154753	0.389446	TRNAY-GUA-3
1	97054753	97154753	0.389446	ACTRT3
1	97054753	97154753	0.389446	TRNAV-AAC
1	97054753	97154753	0.389446	LRRC31
1	97054753	97154753	0.389446	LRRIQ4
1	97054753	97154753	0.389446	LRRC34
1	97054753	97154753	0.389446	MYNN
1	97055508	97155508	0.381665	TRNAY-GUA-3
1	97055508	97155508	0.381665	ACTRT3
1	97055508	97155508	0.381665	TRNAV-AAC
1	97055508	97155508	0.381665	LRRC31
1	97055508	97155508	0.381665	LRRIQ4
1	97055508	97155508	0.381665	LRRC34
1	97055508	97155508	0.381665	MYNN
1	97056572	97156572	0.381646	TRNAY-GUA-3
1	97056572	97156572	0.381646	ACTRT3
1	97056572	97156572	0.381646	TRNAV-AAC
1	97056572	97156572	0.381646	LRRC31
1	97056572	97156572	0.381646	LRRIQ4
1	97056572	97156572	0.381646	LRRC34
1	97056572	97156572	0.381646	MYNN
1	97056765	97156765	0.385937	TRNAY-GUA-3
1	97056765	97156765	0.385937	ACTRT3
1	97056765	97156765	0.385937	TRNAV-AAC
1	97056765	97156765	0.385937	LRRC31
1	97056765	97156765	0.385937	LRRIQ4
1	97056765	97156765	0.385937	LRRC34
1	97056765	97156765	0.385937	MYNN
1	97056824	97156824	0.390274	TRNAY-GUA-3
1	97056824	97156824	0.390274	ACTRT3
1	97056824	97156824	0.390274	TRNAV-AAC
1	97056824	97156824	0.390274	LRRC31
1	97056824	97156824	0.390274	LRRIQ4

1	97056824	97156824	0.390274	LRRC34
1	97056824	97156824	0.390274	MYNN
1	97056857	97156857	0.389269	TRNAY-GUA-3
1	97056857	97156857	0.389269	ACTRT3
1	97056857	97156857	0.389269	TRNAV-AAC
1	97056857	97156857	0.389269	LRRC31
1	97056857	97156857	0.389269	LRRIQ4
1	97056857	97156857	0.389269	LRRC34
1	97056857	97156857	0.389269	MYNN
1	97057449	97157449	0.37924	TRNAY-GUA-3
1	97057449	97157449	0.37924	ACTRT3
1	97057449	97157449	0.37924	TRNAV-AAC
1	97057449	97157449	0.37924	LRRC31
1	97057449	97157449	0.37924	LRRIQ4
1	97057449	97157449	0.37924	LRRC34
1	97057449	97157449	0.37924	MYNN
1	97058053	97158053	0.395268	TRNAY-GUA-3
1	97058053	97158053	0.395268	ACTRT3
1	97058053	97158053	0.395268	TRNAV-AAC
1	97058053	97158053	0.395268	LRRC31
1	97058053	97158053	0.395268	LRRIQ4
1	97058053	97158053	0.395268	LRRC34
1	97058053	97158053	0.395268	MYNN
1	97059686	97159686	0.393011	TRNAY-GUA-3
1	97059686	97159686	0.393011	ACTRT3
1	97059686	97159686	0.393011	TRNAV-AAC
1	97059686	97159686	0.393011	LRRC31
1	97059686	97159686	0.393011	LRRIQ4
1	97059686	97159686	0.393011	LRRC34
1	97059686	97159686	0.393011	MYNN
1	97060432	97160432	0.375125	TRNAY-GUA-3
1	97060432	97160432	0.375125	ACTRT3
1	97060432	97160432	0.375125	TRNAV-AAC
1	97060432	97160432	0.375125	LRRC31
1	97060432	97160432	0.375125	LRRIQ4
1	97060432	97160432	0.375125	LRRC34
1	97060432	97160432	0.375125	MYNN
1	97060650	97160650	0.387833	TRNAY-GUA-3
1	97060650	97160650	0.387833	ACTRT3
1	97060650	97160650	0.387833	TRNAV-AAC
1	97060650	97160650	0.387833	LRRC31
1	97060650	97160650	0.387833	LRRIQ4
1	97060650	97160650	0.387833	LRRC34
1	97060650	97160650	0.387833	MYNN
1	97060879	97160879	0.387833	TRNAY-GUA-3
1	97060879	97160879	0.387833	ACTRT3
1	97060879	97160879	0.387833	TRNAV-AAC
1	97060879	97160879	0.387833	LRRC31
1	97060879	97160879	0.387833	LRRIQ4
1	97060879	97160879	0.387833	LRRC34
1	97060879	97160879	0.387833	MYNN
1	97061827	97161827	0.378313	TRNAY-GUA-3
1	97061827	97161827	0.378313	ACTRT3
1	97061827	97161827	0.378313	TRNAV-AAC
1	97061827	97161827	0.378313	LRRC31
1	97061827	97161827	0.378313	LRRIQ4
1	97061827	97161827	0.378313	LRRC34
1	97061827	97161827	0.378313	MYNN

1	97061877	97161877	0.380078	TRNAY-GUA-3
1	97061877	97161877	0.380078	ACTRT3
1	97061877	97161877	0.380078	TRNAV-AAC
1	97061877	97161877	0.380078	LRRC31
1	97061877	97161877	0.380078	LRRIQ4
1	97061877	97161877	0.380078	LRRC34
1	97061877	97161877	0.380078	MYNN
1	97062424	97162424	0.39815	TRNAY-GUA-3
1	97062424	97162424	0.39815	ACTRT3
1	97062424	97162424	0.39815	TRNAV-AAC
1	97062424	97162424	0.39815	LRRC31
1	97062424	97162424	0.39815	LRRIQ4
1	97062424	97162424	0.39815	LRRC34
1	97062424	97162424	0.39815	MYNN
1	97063286	97163286	0.397672	TRNAY-GUA-3
1	97063286	97163286	0.397672	ACTRT3
1	97063286	97163286	0.397672	TRNAV-AAC
1	97063286	97163286	0.397672	LRRC31
1	97063286	97163286	0.397672	LRRIQ4
1	97063286	97163286	0.397672	LRRC34
1	97063286	97163286	0.397672	MYNN
1	97063310	97163310	0.380002	TRNAY-GUA-3
1	97063310	97163310	0.380002	ACTRT3
1	97063310	97163310	0.380002	TRNAV-AAC
1	97063310	97163310	0.380002	LRRC31
1	97063310	97163310	0.380002	LRRIQ4
1	97063310	97163310	0.380002	LRRC34
1	97063310	97163310	0.380002	MYNN
1	97064091	97164091	0.380565	TRNAY-GUA-3
1	97064091	97164091	0.380565	ACTRT3
1	97064091	97164091	0.380565	TRNAV-AAC
1	97064091	97164091	0.380565	LRRIQ4
1	97064091	97164091	0.380565	LRRC34
1	97064091	97164091	0.380565	MYNN
1	97064097	97164097	0.379264	TRNAY-GUA-3
1	97064097	97164097	0.379264	ACTRT3
1	97064097	97164097	0.379264	TRNAV-AAC
1	97064097	97164097	0.379264	LRRIQ4
1	97064097	97164097	0.379264	LRRC34
1	97064097	97164097	0.379264	MYNN
1	97064383	97164383	0.383475	TRNAY-GUA-3
1	97064383	97164383	0.383475	ACTRT3
1	97064383	97164383	0.383475	TRNAV-AAC
1	97064383	97164383	0.383475	LRRIQ4
1	97064383	97164383	0.383475	LRRC34
1	97064383	97164383	0.383475	MYNN
1	97065268	97165268	0.397656	TRNAY-GUA-3
1	97065268	97165268	0.397656	ACTRT3
1	97065268	97165268	0.397656	TRNAV-AAC
1	97065268	97165268	0.397656	LRRIQ4
1	97065268	97165268	0.397656	LRRC34
1	97065268	97165268	0.397656	MYNN
1	97065640	97165640	0.38786	TRNAY-GUA-3
1	97065640	97165640	0.38786	ACTRT3
1	97065640	97165640	0.38786	TRNAV-AAC
1	97065640	97165640	0.38786	LRRIQ4
1	97065640	97165640	0.38786	LRRC34
1	97065640	97165640	0.38786	MYNN

1	97066634	97166634	0.383621	TRNAY-GUA-3
1	97066634	97166634	0.383621	ACTRT3
1	97066634	97166634	0.383621	TRNAV-AAC
1	97066634	97166634	0.383621	LRRIQ4
1	97066634	97166634	0.383621	LRRC34
1	97066634	97166634	0.383621	MYNN
1	97067239	97167239	0.365423	TRNAY-GUA-3
1	97067239	97167239	0.365423	ACTRT3
1	97067239	97167239	0.365423	TRNAV-AAC
1	97067239	97167239	0.365423	LRRIQ4
1	97067239	97167239	0.365423	LRRC34
1	97067239	97167239	0.365423	MYNN
1	97067619	97167619	0.370528	TRNAY-GUA-3
1	97067619	97167619	0.370528	ACTRT3
1	97067619	97167619	0.370528	TRNAV-AAC
1	97067619	97167619	0.370528	LRRIQ4
1	97067619	97167619	0.370528	LRRC34
1	97067619	97167619	0.370528	MYNN
1	97067856	97167856	0.370071	TRNAY-GUA-3
1	97067856	97167856	0.370071	ACTRT3
1	97067856	97167856	0.370071	TRNAV-AAC
1	97067856	97167856	0.370071	LRRIQ4
1	97067856	97167856	0.370071	LRRC34
1	97067856	97167856	0.370071	MYNN
1	97068303	97168303	0.371158	TRNAY-GUA-3
1	97068303	97168303	0.371158	ACTRT3
1	97068303	97168303	0.371158	TRNAV-AAC
1	97068303	97168303	0.371158	LRRIQ4
1	97068303	97168303	0.371158	LRRC34
1	97068303	97168303	0.371158	MYNN
1	97069115	97169115	0.39032	TRNAY-GUA-3
1	97069115	97169115	0.39032	ACTRT3
1	97069115	97169115	0.39032	TRNAV-AAC
1	97069115	97169115	0.39032	LRRIQ4
1	97069115	97169115	0.39032	LRRC34
1	97069115	97169115	0.39032	MYNN
1	97069153	97169153	0.392824	TRNAY-GUA-3
1	97069153	97169153	0.392824	ACTRT3
1	97069153	97169153	0.392824	TRNAV-AAC
1	97069153	97169153	0.392824	LRRIQ4
1	97069153	97169153	0.392824	LRRC34
1	97069153	97169153	0.392824	MYNN
1	97069387	97169387	0.390623	TRNAY-GUA-3
1	97069387	97169387	0.390623	ACTRT3
1	97069387	97169387	0.390623	TRNAV-AAC
1	97069387	97169387	0.390623	LRRIQ4
1	97069387	97169387	0.390623	LRRC34
1	97069387	97169387	0.390623	MYNN
1	97069434	97169434	0.407518	TRNAY-GUA-3
1	97069434	97169434	0.407518	ACTRT3
1	97069434	97169434	0.407518	TRNAV-AAC
1	97069434	97169434	0.407518	LRRIQ4
1	97069434	97169434	0.407518	LRRC34
1	97069434	97169434	0.407518	MYNN
1	97071229	97171229	0.356591	TRNAY-GUA-3
1	97071229	97171229	0.356591	ACTRT3
1	97071229	97171229	0.356591	TRNAV-AAC
1	97071229	97171229	0.356591	LRRIQ4

1	97071229	97171229	0.356591	LRRC34
1	97071229	97171229	0.356591	MYNN
1	97072740	97172740	0.357665	TRNAY-GUA-3
1	97072740	97172740	0.357665	ACTRT3
1	97072740	97172740	0.357665	TRNAV-AAC
1	97072740	97172740	0.357665	LRRIQ4
1	97072740	97172740	0.357665	LRRC34
1	97072740	97172740	0.357665	MYNN
1	97072741	97172741	0.363423	TRNAY-GUA-3
1	97072741	97172741	0.363423	ACTRT3
1	97072741	97172741	0.363423	TRNAV-AAC
1	97072741	97172741	0.363423	LRRIQ4
1	97072741	97172741	0.363423	LRRC34
1	97072741	97172741	0.363423	MYNN
1	97072932	97172932	0.363245	TRNAY-GUA-3
1	97072932	97172932	0.363245	ACTRT3
1	97072932	97172932	0.363245	TRNAV-AAC
1	97072932	97172932	0.363245	LRRIQ4
1	97072932	97172932	0.363245	LRRC34
1	97072932	97172932	0.363245	MYNN
1	97073184	97173184	0.368089	TRNAY-GUA-3
1	97073184	97173184	0.368089	ACTRT3
1	97073184	97173184	0.368089	TRNAV-AAC
1	97073184	97173184	0.368089	LRRIQ4
1	97073184	97173184	0.368089	LRRC34
1	97073184	97173184	0.368089	MYNN
1	97073818	97173818	0.36368	TRNAY-GUA-3
1	97073818	97173818	0.36368	ACTRT3
1	97073818	97173818	0.36368	TRNAV-AAC
1	97073818	97173818	0.36368	LRRIQ4
1	97073818	97173818	0.36368	LRRC34
1	97073818	97173818	0.36368	MYNN
1	97074157	97174157	0.367815	TRNAY-GUA-3
1	97074157	97174157	0.367815	ACTRT3
1	97074157	97174157	0.367815	TRNAV-AAC
1	97074157	97174157	0.367815	LRRIQ4
1	97074157	97174157	0.367815	LRRC34
1	97074157	97174157	0.367815	MYNN
1	97074882	97174882	0.360767	TRNAY-GUA-3
1	97074882	97174882	0.360767	ACTRT3
1	97074882	97174882	0.360767	TRNAV-AAC
1	97074882	97174882	0.360767	LRRIQ4
1	97074882	97174882	0.360767	LRRC34
1	97074882	97174882	0.360767	MYNN
1	97074883	97174883	0.360767	TRNAY-GUA-3
1	97074883	97174883	0.360767	ACTRT3
1	97074883	97174883	0.360767	TRNAV-AAC
1	97074883	97174883	0.360767	LRRIQ4
1	97074883	97174883	0.360767	LRRC34
1	97074883	97174883	0.360767	MYNN
1	97075165	97175165	0.360767	TRNAY-GUA-3
1	97075165	97175165	0.360767	ACTRT3
1	97075165	97175165	0.360767	TRNAV-AAC
1	97075165	97175165	0.360767	LRRIQ4
1	97075165	97175165	0.360767	LRRC34
1	97075165	97175165	0.360767	MYNN
1	97075327	97175327	0.365028	TRNAY-GUA-3
1	97075327	97175327	0.365028	ACTRT3

1	97075327	97175327	0.365028	TRNAV-AAC
1	97075327	97175327	0.365028	LRRIQ4
1	97075327	97175327	0.365028	LRRC34
1	97075327	97175327	0.365028	MYNN
1	97075536	97175536	0.368077	TRNAY-GUA-3
1	97075536	97175536	0.368077	ACTRT3
1	97075536	97175536	0.368077	TRNAV-AAC
1	97075536	97175536	0.368077	LRRIQ4
1	97075536	97175536	0.368077	LRRC34
1	97075536	97175536	0.368077	MYNN
1	97075948	97175948	0.362917	TRNAY-GUA-3
1	97075948	97175948	0.362917	ACTRT3
1	97075948	97175948	0.362917	TRNAV-AAC
1	97075948	97175948	0.362917	LRRIQ4
1	97075948	97175948	0.362917	LRRC34
1	97075948	97175948	0.362917	MYNN
1	97076011	97176011	0.388907	TRNAY-GUA-3
1	97076011	97176011	0.388907	ACTRT3
1	97076011	97176011	0.388907	TRNAV-AAC
1	97076011	97176011	0.388907	LRRIQ4
1	97076011	97176011	0.388907	LRRC34
1	97076011	97176011	0.388907	MYNN
1	97076119	97176119	0.38675	TRNAY-GUA-3
1	97076119	97176119	0.38675	ACTRT3
1	97076119	97176119	0.38675	TRNAV-AAC
1	97076119	97176119	0.38675	LRRIQ4
1	97076119	97176119	0.38675	LRRC34
1	97076119	97176119	0.38675	MYNN
1	97076296	97176296	0.381978	TRNAY-GUA-3
1	97076296	97176296	0.381978	ACTRT3
1	97076296	97176296	0.381978	TRNAV-AAC
1	97076296	97176296	0.381978	LRRIQ4
1	97076296	97176296	0.381978	LRRC34
1	97076296	97176296	0.381978	MYNN
1	97076477	97176477	0.3747	TRNAY-GUA-3
1	97076477	97176477	0.3747	ACTRT3
1	97076477	97176477	0.3747	TRNAV-AAC
1	97076477	97176477	0.3747	LRRIQ4
1	97076477	97176477	0.3747	LRRC34
1	97076477	97176477	0.3747	MYNN
1	97076663	97176663	0.379735	TRNAY-GUA-3
1	97076663	97176663	0.379735	ACTRT3
1	97076663	97176663	0.379735	TRNAV-AAC
1	97076663	97176663	0.379735	LRRIQ4
1	97076663	97176663	0.379735	LRRC34
1	97076663	97176663	0.379735	MYNN
1	97076674	97176674	0.383369	TRNAY-GUA-3
1	97076674	97176674	0.383369	ACTRT3
1	97076674	97176674	0.383369	TRNAV-AAC
1	97076674	97176674	0.383369	LRRIQ4
1	97076674	97176674	0.383369	LRRC34
1	97076674	97176674	0.383369	MYNN
1	97076900	97176900	0.392111	TRNAY-GUA-3
1	97076900	97176900	0.392111	ACTRT3
1	97076900	97176900	0.392111	TRNAV-AAC
1	97076900	97176900	0.392111	LRRIQ4
1	97076900	97176900	0.392111	LRRC34
1	97076900	97176900	0.392111	MYNN

1	97077004	97177004	0.38588	TRNAY-GUA-3
1	97077004	97177004	0.38588	ACTRT3
1	97077004	97177004	0.38588	TRNAV-AAC
1	97077004	97177004	0.38588	LRRIQ4
1	97077004	97177004	0.38588	LRRC34
1	97077004	97177004	0.38588	MYNN
1	97077361	97177361	0.387263	TRNAY-GUA-3
1	97077361	97177361	0.387263	ACTRT3
1	97077361	97177361	0.387263	TRNAV-AAC
1	97077361	97177361	0.387263	LRRIQ4
1	97077361	97177361	0.387263	LRRC34
1	97077361	97177361	0.387263	MYNN
1	97077663	97177663	0.385059	TRNAY-GUA-3
1	97077663	97177663	0.385059	ACTRT3
1	97077663	97177663	0.385059	TRNAV-AAC
1	97077663	97177663	0.385059	LRRIQ4
1	97077663	97177663	0.385059	LRRC34
1	97077663	97177663	0.385059	MYNN
1	97078296	97178296	0.372408	TRNAY-GUA-3
1	97078296	97178296	0.372408	ACTRT3
1	97078296	97178296	0.372408	TRNAV-AAC
1	97078296	97178296	0.372408	LRRIQ4
1	97078296	97178296	0.372408	LRRC34
1	97078296	97178296	0.372408	MYNN
1	97079046	97179046	0.37044	TRNAY-GUA-3
1	97079046	97179046	0.37044	ACTRT3
1	97079046	97179046	0.37044	TRNAV-AAC
1	97079046	97179046	0.37044	LRRIQ4
1	97079046	97179046	0.37044	LRRC34
1	97079046	97179046	0.37044	MYNN
1	97080089	97180089	0.37044	TRNAY-GUA-3
1	97080089	97180089	0.37044	ACTRT3
1	97080089	97180089	0.37044	TRNAV-AAC
1	97080089	97180089	0.37044	LRRIQ4
1	97080089	97180089	0.37044	LRRC34
1	97080089	97180089	0.37044	MYNN
1	97080308	97180308	0.371167	TRNAY-GUA-3
1	97080308	97180308	0.371167	ACTRT3
1	97080308	97180308	0.371167	TRNAV-AAC
1	97080308	97180308	0.371167	LRRIQ4
1	97080308	97180308	0.371167	LRRC34
1	97080308	97180308	0.371167	MYNN
1	97080455	97180455	0.372552	TRNAY-GUA-3
1	97080455	97180455	0.372552	ACTRT3
1	97080455	97180455	0.372552	TRNAV-AAC
1	97080455	97180455	0.372552	LRRIQ4
1	97080455	97180455	0.372552	LRRC34
1	97080455	97180455	0.372552	MYNN
1	97080598	97180598	0.372817	TRNAY-GUA-3
1	97080598	97180598	0.372817	ACTRT3
1	97080598	97180598	0.372817	TRNAV-AAC
1	97080598	97180598	0.372817	LRRIQ4
1	97080598	97180598	0.372817	LRRC34
1	97080598	97180598	0.372817	MYNN
1	97080639	97180639	0.384406	TRNAY-GUA-3
1	97080639	97180639	0.384406	ACTRT3
1	97080639	97180639	0.384406	TRNAV-AAC
1	97080639	97180639	0.384406	LRRIQ4

1	97080639	97180639	0.384406	LRRC34
1	97080639	97180639	0.384406	MYNN
1	97080702	97180702	0.397826	TRNAY-GUA-3
1	97080702	97180702	0.397826	ACTRT3
1	97080702	97180702	0.397826	TRNAV-AAC
1	97080702	97180702	0.397826	LRRIQ4
1	97080702	97180702	0.397826	LRRC34
1	97080702	97180702	0.397826	MYNN
1	97080874	97180874	0.424845	TRNAY-GUA-3
1	97080874	97180874	0.424845	ACTRT3
1	97080874	97180874	0.424845	TRNAV-AAC
1	97080874	97180874	0.424845	LRRIQ4
1	97080874	97180874	0.424845	LRRC34
1	97080874	97180874	0.424845	MYNN
1	97080882	97180882	0.420914	TRNAY-GUA-3
1	97080882	97180882	0.420914	ACTRT3
1	97080882	97180882	0.420914	TRNAV-AAC
1	97080882	97180882	0.420914	LRRIQ4
1	97080882	97180882	0.420914	LRRC34
1	97080882	97180882	0.420914	MYNN
1	97080941	97180941	0.42483	TRNAY-GUA-3
1	97080941	97180941	0.42483	ACTRT3
1	97080941	97180941	0.42483	TRNAV-AAC
1	97080941	97180941	0.42483	LRRIQ4
1	97080941	97180941	0.42483	LRRC34
1	97080941	97180941	0.42483	MYNN
1	97080946	97180946	0.421099	TRNAY-GUA-3
1	97080946	97180946	0.421099	ACTRT3
1	97080946	97180946	0.421099	TRNAV-AAC
1	97080946	97180946	0.421099	LRRIQ4
1	97080946	97180946	0.421099	LRRC34
1	97080946	97180946	0.421099	MYNN
1	97081047	97181047	0.407883	TRNAY-GUA-3
1	97081047	97181047	0.407883	ACTRT3
1	97081047	97181047	0.407883	TRNAV-AAC
1	97081047	97181047	0.407883	LRRIQ4
1	97081047	97181047	0.407883	LRRC34
1	97081047	97181047	0.407883	MYNN
1	97081057	97181057	0.407893	TRNAY-GUA-3
1	97081057	97181057	0.407893	ACTRT3
1	97081057	97181057	0.407893	TRNAV-AAC
1	97081057	97181057	0.407893	LRRIQ4
1	97081057	97181057	0.407893	LRRC34
1	97081057	97181057	0.407893	MYNN
1	97081474	97181474	0.40661	TRNAY-GUA-3
1	97081474	97181474	0.40661	ACTRT3
1	97081474	97181474	0.40661	TRNAV-AAC
1	97081474	97181474	0.40661	LRRC34
1	97081474	97181474	0.40661	MYNN
1	97081569	97181569	0.407655	TRNAY-GUA-3
1	97081569	97181569	0.407655	ACTRT3
1	97081569	97181569	0.407655	TRNAV-AAC
1	97081569	97181569	0.407655	LRRC34
1	97081569	97181569	0.407655	MYNN
1	97081665	97181665	0.407655	TRNAY-GUA-3
1	97081665	97181665	0.407655	ACTRT3
1	97081665	97181665	0.407655	TRNAV-AAC
1	97081665	97181665	0.407655	LRRC34

1	97081665	97181665	0.407655	MYNN
1	97081764	97181764	0.414705	TRNAY-GUA-3
1	97081764	97181764	0.414705	ACTRT3
1	97081764	97181764	0.414705	TRNAV-AAC
1	97081764	97181764	0.414705	LRRC34
1	97081764	97181764	0.414705	MYNN
1	97081920	97181920	0.41078	TRNAY-GUA-3
1	97081920	97181920	0.41078	ACTRT3
1	97081920	97181920	0.41078	TRNAV-AAC
1	97081920	97181920	0.41078	LRRC34
1	97081920	97181920	0.41078	MYNN
1	97081944	97181944	0.409268	TRNAY-GUA-3
1	97081944	97181944	0.409268	ACTRT3
1	97081944	97181944	0.409268	TRNAV-AAC
1	97081944	97181944	0.409268	LRRC34
1	97081944	97181944	0.409268	MYNN
1	97082065	97182065	0.409374	TRNAY-GUA-3
1	97082065	97182065	0.409374	ACTRT3
1	97082065	97182065	0.409374	TRNAV-AAC
1	97082065	97182065	0.409374	LRRC34
1	97082065	97182065	0.409374	MYNN
1	97082073	97182073	0.411178	TRNAY-GUA-3
1	97082073	97182073	0.411178	ACTRT3
1	97082073	97182073	0.411178	TRNAV-AAC
1	97082073	97182073	0.411178	LRRC34
1	97082073	97182073	0.411178	MYNN
1	97082318	97182318	0.40748	TRNAY-GUA-3
1	97082318	97182318	0.40748	ACTRT3
1	97082318	97182318	0.40748	TRNAV-AAC
1	97082318	97182318	0.40748	LRRC34
1	97082318	97182318	0.40748	MYNN
1	97082444	97182444	0.404102	TRNAY-GUA-3
1	97082444	97182444	0.404102	ACTRT3
1	97082444	97182444	0.404102	TRNAV-AAC
1	97082444	97182444	0.404102	LRRC34
1	97082444	97182444	0.404102	MYNN
1	97082590	97182590	0.409943	TRNAY-GUA-3
1	97082590	97182590	0.409943	ACTRT3
1	97082590	97182590	0.409943	TRNAV-AAC
1	97082590	97182590	0.409943	LRRC34
1	97082590	97182590	0.409943	MYNN
1	97082692	97182692	0.387272	TRNAY-GUA-3
1	97082692	97182692	0.387272	ACTRT3
1	97082692	97182692	0.387272	TRNAV-AAC
1	97082692	97182692	0.387272	LRRC34
1	97082692	97182692	0.387272	MYNN
1	97082892	97182892	0.387204	TRNAY-GUA-3
1	97082892	97182892	0.387204	ACTRT3
1	97082892	97182892	0.387204	TRNAV-AAC
1	97082892	97182892	0.387204	LRRC34
1	97082892	97182892	0.387204	MYNN
1	97083426	97183426	0.372649	TRNAY-GUA-3
1	97083426	97183426	0.372649	ACTRT3
1	97083426	97183426	0.372649	TRNAV-AAC
1	97083426	97183426	0.372649	LRRC34
1	97083426	97183426	0.372649	MYNN
1	97084434	97184434	0.376528	ACTRT3
1	97084434	97184434	0.376528	TRNAV-AAC

1	97084434	97184434	0.376528	LRRC34
1	97084434	97184434	0.376528	MYNN
1	97085958	97185958	0.3628	ACTRT3
1	97085958	97185958	0.3628	TRNAV-AAC
1	97085958	97185958	0.3628	LRRC34
1	97085958	97185958	0.3628	MYNN
1	97086220	97186220	0.379927	ACTRT3
1	97086220	97186220	0.379927	TRNAV-AAC
1	97086220	97186220	0.379927	LRRC34
1	97086220	97186220	0.379927	MYNN
1	97086533	97186533	0.383285	ACTRT3
1	97086533	97186533	0.383285	TRNAV-AAC
1	97086533	97186533	0.383285	LRRC34
1	97086533	97186533	0.383285	MYNN
1	97087308	97187308	0.374963	ACTRT3
1	97087308	97187308	0.374963	TRNAV-AAC
1	97087308	97187308	0.374963	LRRC34
1	97087308	97187308	0.374963	MYNN
1	97087732	97187732	0.374963	ACTRT3
1	97087732	97187732	0.374963	TRNAV-AAC
1	97087732	97187732	0.374963	LRRC34
1	97087732	97187732	0.374963	MYNN
1	97101165	97201165	0.415435	ACTRT3
1	97101165	97201165	0.415435	TRNAV-AAC
1	97101165	97201165	0.415435	LRRC34
1	97101165	97201165	0.415435	MYNN
1	97101182	97201182	0.412497	ACTRT3
1	97101182	97201182	0.412497	TRNAV-AAC
1	97101182	97201182	0.412497	LRRC34
1	97101182	97201182	0.412497	MYNN
1	97101279	97201279	0.43754	ACTRT3
1	97101279	97201279	0.43754	TRNAV-AAC
1	97101279	97201279	0.43754	LRRC34
1	97101279	97201279	0.43754	MYNN
1	97101306	97201306	0.436645	ACTRT3
1	97101306	97201306	0.436645	TRNAV-AAC
1	97101306	97201306	0.436645	LRRC34
1	97101306	97201306	0.436645	MYNN
1	97101387	97201387	0.424499	ACTRT3
1	97101387	97201387	0.424499	TRNAV-AAC
1	97101387	97201387	0.424499	LRRC34
1	97101387	97201387	0.424499	MYNN
1	97101396	97201396	0.434347	ACTRT3
1	97101396	97201396	0.434347	TRNAV-AAC
1	97101396	97201396	0.434347	LRRC34
1	97101396	97201396	0.434347	MYNN
1	97101554	97201554	0.434368	ACTRT3
1	97101554	97201554	0.434368	TRNAV-AAC
1	97101554	97201554	0.434368	LRRC34
1	97101554	97201554	0.434368	MYNN
1	97101723	97201723	0.432965	ACTRT3
1	97101723	97201723	0.432965	TRNAV-AAC
1	97101723	97201723	0.432965	LRRC34
1	97101723	97201723	0.432965	MYNN
1	97101815	97201815	0.424868	ACTRT3
1	97101815	97201815	0.424868	TRNAV-AAC
1	97101815	97201815	0.424868	LRRC34
1	97101815	97201815	0.424868	MYNN

1	98899989	98999989	0.367276	GOLIM4
1	99089508	99189508	0.453686	SERPINI1
1	99089527	99189527	0.444117	SERPINI1
1	99089530	99189530	0.443844	SERPINI1
1	99089585	99189585	0.353936	SERPINI1
1	99310577	99410577	0.472163	WDR49
1	99310605	99410605	0.502932	WDR49
1	99310609	99410609	0.5001	WDR49
1	99310757	99410757	0.500189	WDR49
1	99310837	99410837	0.500966	WDR49
1	99310900	99410900	0.478857	WDR49
1	99310947	99410947	0.48023	WDR49
1	99310959	99410959	0.48023	WDR49
1	99311057	99411057	0.479128	WDR49
1	99311085	99411085	0.479128	WDR49
1	99311153	99411153	0.480152	WDR49
1	99311180	99411180	0.484029	WDR49
1	99311226	99411226	0.503355	WDR49
1	99311248	99411248	0.47375	WDR49
1	99311444	99411444	0.414844	WDR49
1	99311530	99411530	0.421654	WDR49
1	99311595	99411595	0.43438	WDR49
1	99311596	99411596	0.408664	WDR49
1	99311760	99411760	0.347213	WDR49
1	99312139	99412139	0.347213	WDR49
1	99312300	99412300	0.355974	WDR49
1	99312305	99412305	0.356965	WDR49
1	99312570	99412570	0.354525	WDR49
1	99312634	99412634	0.354525	WDR49
1	99312681	99412681	0.354525	WDR49
1	99313238	99413238	0.412341	WDR49
1	99313331	99413331	0.425099	WDR49
1	99313417	99413417	0.424492	WDR49
1	99313472	99413472	0.453311	WDR49
1	99313605	99413605	0.441095	WDR49
1	99313615	99413615	0.453592	WDR49
1	99313674	99413674	0.43018	WDR49
1	99313794	99413794	0.423193	WDR49
1	99313824	99413824	0.42329	WDR49
1	99314096	99414096	0.391326	WDR49
1	99314140	99414140	0.415541	WDR49
1	99314215	99414215	0.400003	WDR49
1	99314631	99414631	0.435405	WDR49
1	99314717	99414717	0.453227	WDR49
1	99314797	99414797	0.434028	WDR49
1	99314828	99414828	0.437664	WDR49
1	99314833	99414833	0.405427	WDR49
1	99314943	99414943	0.376654	WDR49
1	99315574	99415574	0.383927	WDR49
1	99315803	99415803	0.346031	WDR49
1	99315901	99415901	0.405495	WDR49
1	99316272	99416272	0.424158	WDR49
1	99316364	99416364	0.444075	WDR49
1	99316623	99416623	0.415603	WDR49
1	99316978	99416978	0.356601	WDR49
1	99325432	99425432	0.381715	WDR49
1	99325520	99425520	0.378239	WDR49
1	99325949	99425949	0.370565	WDR49

1	99326192	99426192	0.363247	WDR49
1	99326193	99426193	0.356922	WDR49
1	99326223	99426223	0.364806	WDR49
1	99326351	99426351	0.373173	WDR49
1	99326451	99426451	0.358743	WDR49
1	99326461	99426461	0.358743	WDR49
1	99326560	99426560	0.373936	WDR49
1	99326589	99426589	0.40334	WDR49
1	99326734	99426734	0.407283	WDR49
1	99326754	99426754	0.390384	WDR49
1	99326757	99426757	0.397815	WDR49
1	99326789	99426789	0.395747	WDR49
1	99326860	99426860	0.394809	WDR49
1	99326943	99426943	0.390472	WDR49
1	99327034	99427034	0.374465	WDR49
1	99327056	99427056	0.365695	WDR49
1	99327151	99427151	0.383164	WDR49
1	99327171	99427171	0.384609	WDR49
1	99327181	99427181	0.365493	WDR49
1	99327460	99427460	0.365493	WDR49
1	99327524	99427524	0.382303	WDR49
1	99327627	99427627	0.385807	WDR49
1	99327722	99427722	0.35954	WDR49
1	99327921	99427921	0.359046	WDR49
1	99328015	99428015	0.358349	WDR49
1	99328016	99428016	0.35966	WDR49
1	99328026	99428026	0.35966	WDR49
1	99328191	99428191	0.35966	WDR49
1	99328220	99428220	0.35966	WDR49
1	99328255	99428255	0.384139	WDR49
1	99328418	99428418	0.3769	WDR49
1	99328506	99428506	0.374475	WDR49
1	99328513	99428513	0.380017	WDR49
1	99328528	99428528	0.382635	WDR49
1	99328554	99428554	0.381329	WDR49
1	99328657	99428657	0.392483	WDR49
1	99328809	99428809	0.361952	WDR49
1	99328825	99428825	0.361952	WDR49
1	99328900	99428900	0.358139	WDR49
1	99328946	99428946	0.358139	WDR49
1	99329155	99429155	0.358139	WDR49
1	99329238	99429238	0.358139	WDR49
1	99329304	99429304	0.358139	WDR49
1	99329307	99429307	0.403398	WDR49
1	99329451	99429451	0.409325	WDR49
1	99329457	99429457	0.413738	WDR49
1	99329534	99429534	0.400969	WDR49
1	99329536	99429536	0.412192	WDR49
1	99329540	99429540	0.389553	WDR49
1	99329541	99429541	0.389553	WDR49
1	99329762	99429762	0.389553	WDR49
1	99329967	99429967	0.392528	WDR49
1	99329992	99429992	0.446044	WDR49
1	99330018	99430018	0.445324	WDR49
1	99330191	99430191	0.432221	WDR49
1	99330359	99430359	0.429053	WDR49
1	99330403	99430403	0.438623	WDR49
1	99330407	99430407	0.420438	WDR49

1	99330541	99430541	0.420438	WDR49
1	99330599	99430599	0.420438	WDR49
1	99330633	99430633	0.427317	WDR49
1	99330749	99430749	0.427317	WDR49
1	99330771	99430771	0.425856	WDR49
1	99330876	99430876	0.425443	WDR49
1	99330980	99430980	0.430899	WDR49
1	99330981	99430981	0.430899	WDR49
1	99330983	99430983	0.430899	WDR49
1	99331023	99431023	0.438014	WDR49
1	99331183	99431183	0.427035	WDR49
1	99331191	99431191	0.439579	WDR49
1	99331232	99431232	0.426397	WDR49
1	99331374	99431374	0.441451	WDR49
1	99331470	99431470	0.422645	WDR49
1	99331472	99431472	0.422645	WDR49
1	99331474	99431474	0.422645	WDR49
1	99331736	99431736	0.401656	WDR49
1	99331818	99431818	0.403476	WDR49
1	99331910	99431910	0.408522	WDR49
1	99332006	99432006	0.417573	WDR49
1	99332009	99432009	0.417573	WDR49
1	99332087	99432087	0.412259	WDR49
1	99332100	99432100	0.395145	WDR49
1	99332101	99432101	0.395145	WDR49
1	99332121	99432121	0.395145	WDR49
1	99332250	99432250	0.412812	WDR49
1	99332350	99432350	0.380785	WDR49
1	99332490	99432490	0.398321	WDR49
1	99332575	99432575	0.351079	WDR49
1	99332610	99432610	0.350572	WDR49
1	99332767	99432767	0.350572	WDR49
1	99332772	99432772	0.350572	WDR49
1	99332821	99432821	0.355135	WDR49
1	99332930	99432930	0.351098	WDR49
1	99333065	99433065	0.351098	WDR49
1	99333193	99433193	0.371971	WDR49
1	99333263	99433263	0.366149	WDR49
1	99333368	99433368	0.352828	WDR49
1	99333510	99433510	0.401299	WDR49
1	99333537	99433537	0.36929	WDR49
1	99333688	99433688	0.371314	WDR49
1	99333821	99433821	0.374236	WDR49
1	99333834	99433834	0.374236	WDR49
1	99333848	99433848	0.371876	WDR49
1	99333959	99433959	0.38713	WDR49
1	99333990	99433990	0.382286	WDR49
1	99334155	99434155	0.383607	WDR49
1	99334177	99434177	0.390897	WDR49
1	99334340	99434340	0.390866	WDR49
1	99334388	99434388	0.389737	WDR49
1	99334412	99434412	0.389458	WDR49
1	99334524	99434524	0.374933	WDR49
1	99334531	99434531	0.434434	WDR49
1	99334538	99434538	0.431587	WDR49
1	99334783	99434783	0.40258	WDR49
1	99334915	99434915	0.40258	WDR49
1	99334916	99434916	0.40258	WDR49

1	99334947	99434947	0.403384	WDR49
1	99334960	99434960	0.40322	WDR49
1	99334976	99434976	0.417775	WDR49
1	99334977	99434977	0.417775	WDR49
1	99334993	99434993	0.417775	WDR49
1	99334994	99434994	0.417775	WDR49
1	99335000	99435000	0.417775	WDR49
1	99335012	99435012	0.415628	WDR49
1	99335039	99435039	0.409501	WDR49
1	99335239	99435239	0.404536	WDR49
1	99335251	99435251	0.406786	WDR49
1	99335336	99435336	0.390848	WDR49
1	99335507	99435507	0.388155	WDR49
1	99335512	99435512	0.388568	WDR49
1	99335589	99435589	0.389232	WDR49
1	99335753	99435753	0.406947	WDR49
1	99335774	99435774	0.366609	WDR49
1	99335817	99435817	0.367713	WDR49
1	99335835	99435835	0.367713	WDR49
1	99335842	99435842	0.367713	WDR49
1	99336082	99436082	0.367713	WDR49
1	99336086	99436086	0.368068	WDR49
1	99336123	99436123	0.366457	WDR49
1	99336146	99436146	0.374738	WDR49
1	99336281	99436281	0.372946	WDR49
1	99336299	99436299	0.372946	WDR49
1	99336302	99436302	0.372946	WDR49
1	99336367	99436367	0.372946	WDR49
1	99336370	99436370	0.372946	WDR49
1	99336417	99436417	0.372946	WDR49
1	99336430	99436430	0.372946	WDR49
1	99336516	99436516	0.372946	WDR49
1	99336649	99436649	0.372946	WDR49
1	99336651	99436651	0.372946	WDR49
1	99336735	99436735	0.376135	WDR49
1	99336769	99436769	0.382685	WDR49
1	99336810	99436810	0.382685	WDR49
1	99336873	99436873	0.424396	WDR49
1	99336881	99436881	0.381504	WDR49
1	99336905	99436905	0.381504	WDR49
1	99336919	99436919	0.381504	WDR49
1	99337018	99437018	0.384547	WDR49
1	99337149	99437149	0.384547	WDR49
1	99337339	99437339	0.385036	WDR49
1	99337388	99437388	0.384892	WDR49
1	99337455	99437455	0.435243	WDR49
1	99337760	99437760	0.41882	WDR49
1	99337820	99437820	0.418433	WDR49
1	99337845	99437845	0.434236	WDR49
1	99337980	99437980	0.443131	WDR49
1	99338078	99438078	0.424428	WDR49
1	99338448	99438448	0.362672	WDR49
1	99338497	99438497	0.472046	WDR49
1	99339165	99439165	0.342269	WDR49
1	99361543	99461543	0.342349	WDR49
1	99377524	99477524	0.353191	WDR49
1	99377524	99477524	0.353191	SERPINI2
1	99469726	99569726	0.342843	WDR49

1	99469726	99569726	0.342843	SERPINI2
1	99469858	99569858	0.373734	WDR49
1	99469858	99569858	0.373734	SERPINI2
1	99469925	99569925	0.373496	WDR49
1	99469925	99569925	0.373496	SERPINI2
1	99470013	99570013	0.362308	WDR49
1	99470013	99570013	0.362308	SERPINI2
1	99470119	99570119	0.348366	WDR49
1	99470119	99570119	0.348366	SERPINI2
1	99470444	99570444	0.457105	WDR49
1	99470444	99570444	0.457105	SERPINI2
1	99470517	99570517	0.411859	WDR49
1	99470517	99570517	0.411859	SERPINI2
1	99470578	99570578	0.4263	WDR49
1	99470578	99570578	0.4263	SERPINI2
1	99470624	99570624	0.423299	WDR49
1	99470624	99570624	0.423299	SERPINI2
1	99470673	99570673	0.414071	WDR49
1	99470673	99570673	0.414071	SERPINI2
1	99470751	99570751	0.450733	WDR49
1	99470751	99570751	0.450733	SERPINI2
1	99470752	99570752	0.465224	WDR49
1	99470752	99570752	0.465224	SERPINI2
1	99470774	99570774	0.457165	WDR49
1	99470774	99570774	0.457165	SERPINI2
1	99470800	99570800	0.458067	WDR49
1	99470800	99570800	0.458067	SERPINI2
1	99470945	99570945	0.433512	WDR49
1	99470945	99570945	0.433512	SERPINI2
1	99470977	99570977	0.437165	WDR49
1	99470977	99570977	0.437165	SERPINI2
1	99471225	99571225	0.447677	WDR49
1	99471225	99571225	0.447677	SERPINI2
1	99471226	99571226	0.431933	WDR49
1	99471226	99571226	0.431933	SERPINI2
1	99471309	99571309	0.433009	WDR49
1	99471309	99571309	0.433009	SERPINI2
1	99471473	99571473	0.449207	SERPINI2
1	99471486	99571486	0.450032	SERPINI2
1	99471500	99571500	0.419887	SERPINI2
1	99471535	99571535	0.422575	SERPINI2
1	99471536	99571536	0.422575	SERPINI2
1	99471634	99571634	0.434511	SERPINI2
1	99471640	99571640	0.354275	SERPINI2
1	99471666	99571666	0.354275	SERPINI2
1	99472189	99572189	0.350404	SERPINI2
1	99472204	99572204	0.374563	SERPINI2
1	99472209	99572209	0.35831	SERPINI2
1	99472237	99572237	0.380631	SERPINI2
1	99472593	99572593	0.342689	SERPINI2
1	99472642	99572642	0.343258	SERPINI2
1	99472815	99572815	0.345244	SERPINI2
1	99473577	99573577	0.358565	SERPINI2
1	99473870	99573870	0.386054	SERPINI2
1	99473921	99573921	0.397377	SERPINI2
1	99473931	99573931	0.390447	SERPINI2
1	99474012	99574012	0.390447	SERPINI2
1	99474082	99574082	0.400627	SERPINI2

1	99474083	99574083	0.381742	SERPINI2
1	99474156	99574156	0.372501	SERPINI2
1	99474323	99574323	0.378787	SERPINI2
1	99474372	99574372	0.378409	SERPINI2
1	99474466	99574466	0.375654	SERPINI2
1	99474477	99574477	0.367077	SERPINI2
1	99474595	99574595	0.390748	SERPINI2
1	99474598	99574598	0.349702	SERPINI2
1	99474951	99574951	0.352969	SERPINI2
1	99475066	99575066	0.357056	SERPINI2
1	99475099	99575099	0.376114	SERPINI2
1	99475359	99575359	0.387555	SERPINI2
1	99475498	99575498	0.386281	SERPINI2
1	99475515	99575515	0.383557	SERPINI2
1	99475680	99575680	0.367217	SERPINI2
1	99475956	99575956	0.39914	SERPINI2
1	99476185	99576185	0.38592	SERPINI2
1	99476186	99576186	0.387509	SERPINI2
1	99476262	99576262	0.352667	SERPINI2
1	99476275	99576275	0.409832	SERPINI2
1	99476311	99576311	0.389051	SERPINI2
1	99476686	99576686	0.369691	SERPINI2
1	99476695	99576695	0.398887	SERPINI2
1	99476751	99576751	0.398377	SERPINI2
1	99477267	99577267	0.386468	SERPINI2
1	99477302	99577302	0.401298	SERPINI2
1	99477384	99577384	0.395629	SERPINI2
1	99477517	99577517	0.387631	SERPINI2
1	99477604	99577604	0.373828	SERPINI2
1	99477629	99577629	0.398148	SERPINI2
1	99477667	99577667	0.396708	SERPINI2
1	99477669	99577669	0.403234	SERPINI2
1	99477729	99577729	0.395696	SERPINI2
1	99477759	99577759	0.424172	SERPINI2
1	99477917	99577917	0.40028	SERPINI2
1	99477917	99577917	0.40028	ZBBX
1	99478015	99578015	0.398688	SERPINI2
1	99478015	99578015	0.398688	ZBBX
1	99478028	99578028	0.407998	SERPINI2
1	99478028	99578028	0.407998	ZBBX
1	99478043	99578043	0.397551	SERPINI2
1	99478043	99578043	0.397551	ZBBX
1	99478055	99578055	0.398843	SERPINI2
1	99478055	99578055	0.398843	ZBBX
1	99478061	99578061	0.38829	SERPINI2
1	99478061	99578061	0.38829	ZBBX
1	99478101	99578101	0.374367	SERPINI2
1	99478101	99578101	0.374367	ZBBX
1	99478174	99578174	0.381323	SERPINI2
1	99478174	99578174	0.381323	ZBBX
1	99478223	99578223	0.387382	SERPINI2
1	99478223	99578223	0.387382	ZBBX
1	99478374	99578374	0.419382	SERPINI2
1	99478374	99578374	0.419382	ZBBX
1	99478377	99578377	0.419167	SERPINI2
1	99478377	99578377	0.419167	ZBBX
1	99478424	99578424	0.399524	SERPINI2
1	99478424	99578424	0.399524	ZBBX

1	99478494	99578494	0.422975	SERPINI2
1	99478494	99578494	0.422975	ZBBX
1	99478524	99578524	0.427892	SERPINI2
1	99478524	99578524	0.427892	ZBBX
1	99478563	99578563	0.475279	SERPINI2
1	99478563	99578563	0.475279	ZBBX
1	99478714	99578714	0.458675	SERPINI2
1	99478714	99578714	0.458675	ZBBX
1	99478718	99578718	0.462949	SERPINI2
1	99478718	99578718	0.462949	ZBBX
1	99478782	99578782	0.450453	SERPINI2
1	99478782	99578782	0.450453	ZBBX
1	99478845	99578845	0.450045	SERPINI2
1	99478845	99578845	0.450045	ZBBX
1	99478873	99578873	0.433151	SERPINI2
1	99478873	99578873	0.433151	ZBBX
1	99478874	99578874	0.460476	SERPINI2
1	99478874	99578874	0.460476	ZBBX
1	99478976	99578976	0.426245	SERPINI2
1	99478976	99578976	0.426245	ZBBX
1	99478984	99578984	0.465634	SERPINI2
1	99478984	99578984	0.465634	ZBBX
1	99478985	99578985	0.447381	SERPINI2
1	99478985	99578985	0.447381	ZBBX
1	99479063	99579063	0.436559	SERPINI2
1	99479063	99579063	0.436559	ZBBX
1	99479153	99579153	0.457818	SERPINI2
1	99479153	99579153	0.457818	ZBBX
1	99479185	99579185	0.45483	SERPINI2
1	99479185	99579185	0.45483	ZBBX
1	99479209	99579209	0.474992	SERPINI2
1	99479209	99579209	0.474992	ZBBX
1	99479285	99579285	0.468132	SERPINI2
1	99479285	99579285	0.468132	ZBBX
1	99479355	99579355	0.476857	SERPINI2
1	99479355	99579355	0.476857	ZBBX
1	99479379	99579379	0.444101	SERPINI2
1	99479379	99579379	0.444101	ZBBX
1	99479456	99579456	0.441602	SERPINI2
1	99479456	99579456	0.441602	ZBBX
1	99479562	99579562	0.44912	SERPINI2
1	99479562	99579562	0.44912	ZBBX
1	99479638	99579638	0.417702	SERPINI2
1	99479638	99579638	0.417702	ZBBX
1	99479814	99579814	0.406587	SERPINI2
1	99479814	99579814	0.406587	ZBBX
1	99479821	99579821	0.412922	SERPINI2
1	99479821	99579821	0.412922	ZBBX
1	99479848	99579848	0.41237	SERPINI2
1	99479848	99579848	0.41237	ZBBX
1	99479963	99579963	0.405041	SERPINI2
1	99479963	99579963	0.405041	ZBBX
1	99479978	99579978	0.427343	SERPINI2
1	99479978	99579978	0.427343	ZBBX
1	99480074	99580074	0.413234	SERPINI2
1	99480074	99580074	0.413234	ZBBX
1	99480079	99580079	0.405963	SERPINI2
1	99480079	99580079	0.405963	ZBBX

1	99480100	99580100	0.412689	SERPINI2
1	99480100	99580100	0.412689	ZBBX
1	99480105	99580105	0.383996	SERPINI2
1	99480105	99580105	0.383996	ZBBX
1	99480106	99580106	0.383996	SERPINI2
1	99480106	99580106	0.383996	ZBBX
1	99480136	99580136	0.355007	SERPINI2
1	99480136	99580136	0.355007	ZBBX
1	99480137	99580137	0.355007	SERPINI2
1	99480137	99580137	0.355007	ZBBX
1	99480225	99580225	0.384135	SERPINI2
1	99480225	99580225	0.384135	ZBBX
1	99480296	99580296	0.422252	SERPINI2
1	99480296	99580296	0.422252	ZBBX
1	99480348	99580348	0.390894	SERPINI2
1	99480348	99580348	0.390894	ZBBX
1	99480361	99580361	0.393251	SERPINI2
1	99480361	99580361	0.393251	ZBBX
1	99480533	99580533	0.391998	SERPINI2
1	99480533	99580533	0.391998	ZBBX
1	99480547	99580547	0.393531	SERPINI2
1	99480547	99580547	0.393531	ZBBX
1	99480548	99580548	0.390342	SERPINI2
1	99480548	99580548	0.390342	ZBBX
1	99480664	99580664	0.38794	SERPINI2
1	99480664	99580664	0.38794	ZBBX
1	99480695	99580695	0.38618	SERPINI2
1	99480695	99580695	0.38618	ZBBX
1	99480733	99580733	0.385465	SERPINI2
1	99480733	99580733	0.385465	ZBBX
1	99480743	99580743	0.358588	SERPINI2
1	99480743	99580743	0.358588	ZBBX
1	99480939	99580939	0.360162	SERPINI2
1	99480939	99580939	0.360162	ZBBX
1	99480960	99580960	0.354868	SERPINI2
1	99480960	99580960	0.354868	ZBBX
1	99481092	99581092	0.346138	SERPINI2
1	99481092	99581092	0.346138	ZBBX
1	99481095	99581095	0.347819	SERPINI2
1	99481095	99581095	0.347819	ZBBX
1	99481198	99581198	0.349546	SERPINI2
1	99481198	99581198	0.349546	ZBBX
1	99481202	99581202	0.343496	SERPINI2
1	99481202	99581202	0.343496	ZBBX
1	99481214	99581214	0.368417	SERPINI2
1	99481214	99581214	0.368417	ZBBX
1	99481263	99581263	0.359256	SERPINI2
1	99481263	99581263	0.359256	ZBBX
1	99481305	99581305	0.358088	SERPINI2
1	99481305	99581305	0.358088	ZBBX
1	99481392	99581392	0.364167	SERPINI2
1	99481392	99581392	0.364167	ZBBX
1	99481488	99581488	0.348205	SERPINI2
1	99481488	99581488	0.348205	ZBBX
1	99481495	99581495	0.366281	SERPINI2
1	99481495	99581495	0.366281	ZBBX
1	99481580	99581580	0.375051	SERPINI2
1	99481580	99581580	0.375051	ZBBX

1	99481583	99581583	0.371745	SERPINI2
1	99481583	99581583	0.371745	ZBBX
1	99481656	99581656	0.366615	SERPINI2
1	99481656	99581656	0.366615	ZBBX
1	99481711	99581711	0.366615	SERPINI2
1	99481711	99581711	0.366615	ZBBX
1	99481738	99581738	0.366168	SERPINI2
1	99481738	99581738	0.366168	ZBBX
1	99481756	99581756	0.368897	SERPINI2
1	99481756	99581756	0.368897	ZBBX
1	99481766	99581766	0.378689	SERPINI2
1	99481766	99581766	0.378689	ZBBX
1	99481802	99581802	0.380275	SERPINI2
1	99481802	99581802	0.380275	ZBBX
1	99481807	99581807	0.377706	SERPINI2
1	99481807	99581807	0.377706	ZBBX
1	99481812	99581812	0.376012	SERPINI2
1	99481812	99581812	0.376012	ZBBX
1	99481842	99581842	0.376012	SERPINI2
1	99481842	99581842	0.376012	ZBBX
1	99481843	99581843	0.368406	SERPINI2
1	99481843	99581843	0.368406	ZBBX
1	99481884	99581884	0.368406	SERPINI2
1	99481884	99581884	0.368406	ZBBX
1	99482002	99582002	0.369495	SERPINI2
1	99482002	99582002	0.369495	ZBBX
1	99482005	99582005	0.354657	SERPINI2
1	99482005	99582005	0.354657	ZBBX
1	99482011	99582011	0.358187	SERPINI2
1	99482011	99582011	0.358187	ZBBX
1	99482012	99582012	0.358187	SERPINI2
1	99482012	99582012	0.358187	ZBBX
1	99482030	99582030	0.350305	SERPINI2
1	99482030	99582030	0.350305	ZBBX
1	99482477	99582477	0.348628	SERPINI2
1	99482477	99582477	0.348628	ZBBX
1	99482509	99582509	0.35129	SERPINI2
1	99482509	99582509	0.35129	ZBBX
1	99483287	99583287	0.34508	SERPINI2
1	99483287	99583287	0.34508	ZBBX
1	99496026	99596026	0.385383	SERPINI2
1	99496026	99596026	0.385383	ZBBX
1	99496053	99596053	0.347248	SERPINI2
1	99496053	99596053	0.347248	ZBBX
1	99496071	99596071	0.347248	SERPINI2
1	99496071	99596071	0.347248	ZBBX
1	99496205	99596205	0.356123	SERPINI2
1	99496205	99596205	0.356123	ZBBX
1	99496341	99596341	0.353054	SERPINI2
1	99496341	99596341	0.353054	ZBBX
1	99505201	99605201	0.361809	SERPINI2
1	99505201	99605201	0.361809	ZBBX
1	99506338	99606338	0.350032	SERPINI2
1	99506338	99606338	0.350032	ZBBX
1	99506471	99606471	0.359668	SERPINI2
1	99506471	99606471	0.359668	ZBBX
1	99506496	99606496	0.359668	SERPINI2
1	99506496	99606496	0.359668	ZBBX

1	99506497	99606497	0.359668	SERPINI2
1	99506497	99606497	0.359668	ZBBX
1	99506498	99606498	0.359668	SERPINI2
1	99506498	99606498	0.359668	ZBBX
1	99506971	99606971	0.386533	SERPINI2
1	99506971	99606971	0.386533	ZBBX
1	99507153	99607153	0.387427	SERPINI2
1	99507153	99607153	0.387427	ZBBX
1	99507179	99607179	0.417481	SERPINI2
1	99507179	99607179	0.417481	ZBBX
1	99507238	99607238	0.373056	SERPINI2
1	99507238	99607238	0.373056	ZBBX
1	99507506	99607506	0.361485	SERPINI2
1	99507506	99607506	0.361485	ZBBX
1	99508080	99608080	0.359634	SERPINI2
1	99508080	99608080	0.359634	ZBBX
1	99508373	99608373	0.421301	SERPINI2
1	99508373	99608373	0.421301	ZBBX
1	99508529	99608529	0.399393	SERPINI2
1	99508529	99608529	0.399393	ZBBX
1	99509580	99609580	0.365971	SERPINI2
1	99509580	99609580	0.365971	ZBBX
1	99509950	99609950	0.405246	SERPINI2
1	99509950	99609950	0.405246	ZBBX
1	99509955	99609955	0.386271	SERPINI2
1	99509955	99609955	0.386271	ZBBX
1	99510065	99610065	0.386271	SERPINI2
1	99510065	99610065	0.386271	ZBBX
1	99510085	99610085	0.408623	SERPINI2
1	99510085	99610085	0.408623	ZBBX
1	99510170	99610170	0.364681	SERPINI2
1	99510170	99610170	0.364681	ZBBX
1	99510265	99610265	0.364549	SERPINI2
1	99510265	99610265	0.364549	ZBBX
1	99510266	99610266	0.364549	SERPINI2
1	99510266	99610266	0.364549	ZBBX
1	99513897	99613897	0.355373	ZBBX
1	99513906	99613906	0.34178	ZBBX
1	99513988	99613988	0.3442	ZBBX
1	99514004	99614004	0.345173	ZBBX
1	99514046	99614046	0.355535	ZBBX
1	99514105	99614105	0.357198	ZBBX
1	99514111	99614111	0.357198	ZBBX
1	99514119	99614119	0.356143	ZBBX
1	99514786	99614786	0.348654	ZBBX
1	99515050	99615050	0.346719	ZBBX
1	99515121	99615121	0.359013	ZBBX
1	99515316	99615316	0.365778	ZBBX
1	99515342	99615342	0.365778	ZBBX
1	99515348	99615348	0.362008	ZBBX
1	99516285	99616285	0.347075	ZBBX
1	99517413	99617413	0.341764	ZBBX
1	99525484	99625484	0.355691	ZBBX
1	99526185	99626185	0.341687	ZBBX
1	99526302	99626302	0.381671	ZBBX
1	99526337	99626337	0.377781	ZBBX
1	99526339	99626339	0.371295	ZBBX
1	99526371	99626371	0.369459	ZBBX

1	99526482	99626482	0.372658	ZBBX
1	99526555	99626555	0.39367	ZBBX
1	99526648	99626648	0.415438	ZBBX
1	99526792	99626792	0.409114	ZBBX
1	99526938	99626938	0.421509	ZBBX
1	99527222	99627222	0.359577	ZBBX
1	99527244	99627244	0.364833	ZBBX
1	99527310	99627310	0.351748	ZBBX
1	99527522	99627522	0.343818	ZBBX
1	99527612	99627612	0.35515	ZBBX
1	99527641	99627641	0.345977	ZBBX
1	99527689	99627689	0.345977	ZBBX
1	99527976	99627976	0.364976	ZBBX
1	99528010	99628010	0.359519	ZBBX
1	99528055	99628055	0.371953	ZBBX
1	99528074	99628074	0.36026	ZBBX
1	99528147	99628147	0.372289	ZBBX
1	99528196	99628196	0.366733	ZBBX
1	99528244	99628244	0.350478	ZBBX
1	99528328	99628328	0.369285	ZBBX
1	99528338	99628338	0.369285	ZBBX
1	99528340	99628340	0.362297	ZBBX
1	99528424	99628424	0.378051	ZBBX
1	99528480	99628480	0.365295	ZBBX
1	99528488	99628488	0.364851	ZBBX
1	99528562	99628562	0.365623	ZBBX
1	99528618	99628618	0.345821	ZBBX
1	99528654	99628654	0.356481	ZBBX
1	99528671	99628671	0.349413	ZBBX
1	99529034	99629034	0.362433	ZBBX
1	99529036	99629036	0.360782	ZBBX
1	99529045	99629045	0.375191	ZBBX
1	99529085	99629085	0.387396	ZBBX
1	99529126	99629126	0.373643	ZBBX
1	99529130	99629130	0.373643	ZBBX
1	99529170	99629170	0.365925	ZBBX
1	99529489	99629489	0.351826	ZBBX
1	99529839	99629839	0.345785	ZBBX
1	99529925	99629925	0.357087	ZBBX
1	99529958	99629958	0.349882	ZBBX
1	99529996	99629996	0.362123	ZBBX
1	99530307	99630307	0.351324	ZBBX
1	99530341	99630341	0.389652	ZBBX
1	99530470	99630470	0.376893	ZBBX
1	99530504	99630504	0.383675	ZBBX
1	99530582	99630582	0.364146	ZBBX
1	99530647	99630647	0.37817	ZBBX
1	99530685	99630685	0.370026	ZBBX
1	99530823	99630823	0.357011	ZBBX
1	99531681	99631681	0.369812	ZBBX
1	99531694	99631694	0.343868	ZBBX
1	99531720	99631720	0.356956	ZBBX
1	99533532	99633532	0.38136	ZBBX
1	99533547	99633547	0.362351	ZBBX
1	99533703	99633703	0.353265	ZBBX
1	99534722	99634722	0.368921	ZBBX
1	99534769	99634769	0.354803	ZBBX
1	99534820	99634820	0.38239	ZBBX

1	99535031	99635031	0.387231	ZBBX
1	99535141	99635141	0.372276	ZBBX
1	99535298	99635298	0.367476	ZBBX
1	99535682	99635682	0.371397	ZBBX
1	99535771	99635771	0.385618	ZBBX
1	99535794	99635794	0.391484	ZBBX
1	99535840	99635840	0.366508	ZBBX
1	99535889	99635889	0.386162	ZBBX
1	99535917	99635917	0.354514	ZBBX
1	99535996	99635996	0.354514	ZBBX
1	99537028	99637028	0.36935	ZBBX
1	99537138	99637138	0.369045	ZBBX
1	99537295	99637295	0.349818	ZBBX
1	99537304	99637304	0.348132	ZBBX
1	99537528	99637528	0.344629	ZBBX
1	99537900	99637900	0.358181	ZBBX
1	99538057	99638057	0.368539	ZBBX
1	99538632	99638632	0.349618	ZBBX
1	99538787	99638787	0.372702	ZBBX
1	99539050	99639050	0.378396	ZBBX
1	99539289	99639289	0.365748	ZBBX
1	99539331	99639331	0.380132	ZBBX
1	99539463	99639463	0.380025	ZBBX
1	99539504	99639504	0.375493	ZBBX
1	99539505	99639505	0.393219	ZBBX
1	99539646	99639646	0.391884	ZBBX
1	99539690	99639690	0.393212	ZBBX
1	99539723	99639723	0.395637	ZBBX
1	99539857	99639857	0.395075	ZBBX
1	99539912	99639912	0.415047	ZBBX
1	99539989	99639989	0.420451	ZBBX
1	99540041	99640041	0.416796	ZBBX
1	99540047	99640047	0.416796	ZBBX
1	99540077	99640077	0.394709	ZBBX
1	99540214	99640214	0.387695	ZBBX
1	99540356	99640356	0.417929	ZBBX
1	99540411	99640411	0.440692	ZBBX
1	99540412	99640412	0.441602	ZBBX
1	99540478	99640478	0.348894	ZBBX
1	99540632	99640632	0.420941	ZBBX
1	99540646	99640646	0.438868	ZBBX
1	99540675	99640675	0.462529	ZBBX
1	99540703	99640703	0.441439	ZBBX
1	99540799	99640799	0.47574	ZBBX
1	99540934	99640934	0.593004	ZBBX
1	99540990	99640990	0.578846	ZBBX
1	99541048	99641048	0.461035	ZBBX
1	99541095	99641095	0.474525	ZBBX
1	99541101	99641101	0.390457	ZBBX
1	99563005	99663005	0.352937	ZBBX
1	99563134	99663134	0.376561	ZBBX
1	99563298	99663298	0.354426	ZBBX
1	99563395	99663395	0.349597	ZBBX
1	99563583	99663583	0.365277	ZBBX
1	99563592	99663592	0.356178	ZBBX
1	99563634	99663634	0.355595	ZBBX
1	99563736	99663736	0.373628	ZBBX
1	99563755	99663755	0.373628	ZBBX

1	99564012	99664012	0.348079	ZBBX
1	99564034	99664034	0.346061	ZBBX
1	99564108	99664108	0.349086	ZBBX
1	99564161	99664161	0.350166	ZBBX
1	99564174	99664174	0.35025	ZBBX
1	99564217	99664217	0.351864	ZBBX
1	99564235	99664235	0.359925	ZBBX
1	99565505	99665505	0.355795	ZBBX
1	99565638	99665638	0.356597	ZBBX
1	99566148	99666148	0.362345	ZBBX
1	99566241	99666241	0.351394	ZBBX
1	99566312	99666312	0.342328	ZBBX
1	99566320	99666320	0.347863	ZBBX
1	99566348	99666348	0.348903	ZBBX
1	99566351	99666351	0.348903	ZBBX
1	99566367	99666367	0.355434	ZBBX
1	99566372	99666372	0.35434	ZBBX
1	99566388	99666388	0.35434	ZBBX
1	99566401	99666401	0.349551	ZBBX
1	99566440	99666440	0.349551	ZBBX
1	99566461	99666461	0.346924	ZBBX
1	99566483	99666483	0.356081	ZBBX
1	99567514	99667514	0.387262	ZBBX
1	99567559	99667559	0.375404	ZBBX
1	99567864	99667864	0.389381	ZBBX
1	99567946	99667946	0.419458	ZBBX
1	99568008	99668008	0.421812	ZBBX
1	99568038	99668038	0.425631	ZBBX
1	99568039	99668039	0.425143	ZBBX
1	99568042	99668042	0.417427	ZBBX
1	99568138	99668138	0.434583	ZBBX
1	99568245	99668245	0.414585	ZBBX
1	99568360	99668360	0.424237	ZBBX
1	99568425	99668425	0.424237	ZBBX
1	99568460	99668460	0.419169	ZBBX
1	99568603	99668603	0.416241	ZBBX
1	99568688	99668688	0.418247	ZBBX
1	99568713	99668713	0.412992	ZBBX
1	99568739	99668739	0.413505	ZBBX
1	99568847	99668847	0.447885	ZBBX
1	99568900	99668900	0.442213	ZBBX
1	99568901	99668901	0.429778	ZBBX
1	99569101	99669101	0.404004	ZBBX
1	99569278	99669278	0.406702	ZBBX
1	99569327	99669327	0.41462	ZBBX
1	99569436	99669436	0.427891	ZBBX
1	99569502	99669502	0.412059	ZBBX
1	99569545	99669545	0.409263	ZBBX
1	99569555	99669555	0.409263	ZBBX
1	99569689	99669689	0.413841	ZBBX
1	99569722	99669722	0.411756	ZBBX
1	99569771	99669771	0.462281	ZBBX
1	99569801	99669801	0.452303	ZBBX
1	99569835	99669835	0.450887	ZBBX
1	99569864	99669864	0.452256	ZBBX
1	99569893	99669893	0.477477	ZBBX
1	99569958	99669958	0.476692	ZBBX
1	99570133	99670133	0.477416	ZBBX

1	99570134	99670134	0.497623	ZBBX
1	99570142	99670142	0.482027	ZBBX
1	99570334	99670334	0.494894	ZBBX
1	99570358	99670358	0.499312	ZBBX
1	99570506	99670506	0.463213	ZBBX
1	99570629	99670629	0.469234	ZBBX
1	99570630	99670630	0.491433	ZBBX
1	99570757	99670757	0.491059	ZBBX
1	99570909	99670909	0.496848	ZBBX
1	99570946	99670946	0.481217	ZBBX
1	99570953	99670953	0.446691	ZBBX
1	99571067	99671067	0.440988	ZBBX
1	99571127	99671127	0.440988	ZBBX
1	99571151	99671151	0.445835	ZBBX
1	99571461	99671461	0.484274	ZBBX
1	99571519	99671519	0.507585	ZBBX
1	99571533	99671533	0.511155	ZBBX
1	99571598	99671598	0.487618	ZBBX
1	99571704	99671704	0.489385	ZBBX
1	99571795	99671795	0.497305	ZBBX
1	99571864	99671864	0.497	ZBBX
1	99571972	99671972	0.517956	ZBBX
1	99572079	99672079	0.540402	ZBBX
1	99572126	99672126	0.424883	ZBBX
1	99572136	99672136	0.417224	ZBBX
1	99587190	99687190	0.347924	ZBBX
1	99587191	99687191	0.348315	ZBBX
1	99597164	99697164	0.39785	ZBBX
1	99597383	99697383	0.402226	ZBBX
1	99597430	99697430	0.40194	ZBBX
1	99597432	99697432	0.40194	ZBBX
1	99597443	99697443	0.404493	ZBBX
1	99597473	99697473	0.438912	ZBBX
1	99597484	99697484	0.437139	ZBBX
1	99597492	99697492	0.437139	ZBBX
1	99597538	99697538	0.437139	ZBBX
1	99597603	99697603	0.44536	ZBBX
1	99597612	99697612	0.426414	ZBBX
1	99597698	99697698	0.431922	ZBBX
1	99597704	99697704	0.439718	ZBBX
1	99597716	99697716	0.425568	ZBBX
1	99597803	99697803	0.426821	ZBBX
1	99597840	99697840	0.363863	ZBBX
1	99597920	99697920	0.366883	ZBBX
1	99597949	99697949	0.361915	ZBBX
1	99598280	99698280	0.368085	ZBBX
1	99598549	99698549	0.368085	ZBBX
1	99598556	99698556	0.369345	ZBBX
1	99598658	99698658	0.367335	ZBBX
1	99598659	99698659	0.367335	ZBBX
1	99598698	99698698	0.367335	ZBBX
1	99598747	99698747	0.369344	ZBBX
1	99598754	99698754	0.376627	ZBBX
1	99598848	99698848	0.376574	ZBBX
1	99598865	99698865	0.371923	ZBBX
1	99598963	99698963	0.372001	ZBBX
1	99598980	99698980	0.375983	ZBBX
1	99599100	99699100	0.37117	ZBBX

1	99599146	99699146	0.36909	ZBBX
1	99599356	99699356	0.360368	ZBBX
1	99599535	99699535	0.369872	ZBBX
1	99599541	99699541	0.367394	ZBBX
1	99599593	99699593	0.376099	ZBBX
1	99599602	99699602	0.378932	ZBBX
1	99599625	99699625	0.431483	ZBBX
1	99599710	99699710	0.451572	ZBBX
1	99599738	99699738	0.44243	ZBBX
1	99599816	99699816	0.440687	ZBBX
1	99599876	99699876	0.444715	ZBBX
1	99600023	99700023	0.444715	ZBBX
1	99600059	99700059	0.422957	ZBBX
1	99600200	99700200	0.445129	ZBBX
1	99600298	99700298	0.444915	ZBBX
1	99600334	99700334	0.447986	ZBBX
1	99600444	99700444	0.434591	ZBBX
1	99600599	99700599	0.434591	ZBBX
1	99600658	99700658	0.470433	ZBBX
1	99600669	99700669	0.470536	ZBBX
1	99600747	99700747	0.471852	ZBBX
1	99600762	99700762	0.468911	ZBBX
1	99600779	99700779	0.473061	ZBBX
1	99600801	99700801	0.478996	ZBBX
1	99600919	99700919	0.538947	ZBBX
1	99601057	99701057	0.525866	ZBBX
1	99601071	99701071	0.45879	ZBBX
1	99601175	99701175	0.466171	ZBBX
1	99601231	99701231	0.357563	ZBBX
1	99601535	99701535	0.353688	ZBBX
1	99601583	99701583	0.352645	ZBBX
1	99601665	99701665	0.345865	ZBBX
1	99601674	99701674	0.345915	ZBBX
1	99601916	99701916	0.353602	ZBBX
1	99652688	99752688	0.34696	ZBBX
1	99652735	99752735	0.347753	ZBBX
1	99652763	99752763	0.348029	ZBBX
1	99652837	99752837	0.34873	ZBBX
1	99652873	99752873	0.343531	ZBBX
1	99652909	99752909	0.35541	ZBBX
1	99652950	99752950	0.342957	ZBBX
1	99652953	99752953	0.342957	ZBBX
1	99652971	99752971	0.351821	ZBBX
1	99652975	99752975	0.343973	ZBBX
1	99653200	99753200	0.352762	ZBBX
1	99653403	99753403	0.34754	ZBBX
1	99653821	99753821	0.348434	ZBBX
1	99653909	99753909	0.3551	ZBBX
1	99653997	99753997	0.345122	ZBBX
1	99654005	99754005	0.345122	ZBBX
1	99654068	99754068	0.347165	ZBBX
1	99654220	99754220	0.341805	ZBBX
1	99654291	99754291	0.3435	ZBBX
1	99654301	99754301	0.343761	ZBBX
1	99654328	99754328	0.348562	ZBBX
1	99654336	99754336	0.355436	ZBBX
1	99654361	99754361	0.347964	ZBBX
1	99654362	99754362	0.361885	ZBBX

1	99654420	99754420	0.362984	ZBBX
1	99654423	99754423	0.362869	ZBBX
1	99654428	99754428	0.359758	ZBBX
1	99654451	99754451	0.360082	ZBBX
1	99654499	99754499	0.360245	ZBBX
1	99654506	99754506	0.363819	ZBBX
1	99654512	99754512	0.363819	ZBBX
1	99654597	99754597	0.342824	ZBBX
1	99655861	99755861	0.380959	ZBBX
1	99655904	99755904	0.351019	ZBBX
1	99656330	99756330	0.34308	ZBBX
1	99657166	99757166	0.343324	ZBBX
1	99657228	99757228	0.350138	ZBBX
1	99678891	99778891	0.381094	ZBBX
1	99678927	99778927	0.378306	ZBBX
1	99679066	99779066	0.384918	ZBBX
1	99679096	99779096	0.377424	ZBBX
1	99679103	99779103	0.360617	ZBBX
1	99679126	99779126	0.362368	ZBBX
1	99679470	99779470	0.359582	ZBBX
1	99679652	99779652	0.34604	ZBBX
1	99679951	99779951	0.365724	ZBBX
1	99679967	99779967	0.351965	ZBBX
1	99680087	99780087	0.355612	ZBBX
1	99680110	99780110	0.355937	ZBBX
1	99680142	99780142	0.373687	ZBBX
1	99680207	99780207	0.353637	ZBBX
1	99680280	99780280	0.353053	ZBBX
1	99680324	99780324	0.353315	ZBBX
1	99680339	99780339	0.353174	ZBBX
1	99680945	99780945	0.342564	ZBBX
1	99680954	99780954	0.347134	ZBBX
1	99681388	99781388	0.347134	ZBBX
1	99681518	99781518	0.354909	ZBBX
1	99681713	99781713	0.352939	ZBBX
1	99681930	99781930	0.353297	ZBBX
1	99681997	99781997	0.348468	ZBBX
1	99682550	99782550	0.343673	ZBBX
1	99682634	99782634	0.346172	ZBBX
1	99683243	99783243	0.345629	ZBBX
1	99684251	99784251	0.344668	ZBBX
1	99684632	99784632	0.343602	ZBBX
1	99684639	99784639	0.343602	ZBBX
1	99684703	99784703	0.349487	ZBBX
1	99684771	99784771	0.349579	ZBBX
1	99684778	99784778	0.356114	ZBBX
1	99684909	99784909	0.343248	ZBBX
1	99685065	99785065	0.341749	ZBBX
1	99688953	99788953	0.354784	ZBBX
1	99689429	99789429	0.346866	ZBBX
1	100931442	101031442	0.361077	BCHE
1	100931586	101031586	0.345686	BCHE
1	100931765	101031765	0.373919	BCHE
1	100931836	101031836	0.397545	BCHE
1	100931838	101031838	0.382014	BCHE
1	100931844	101031844	0.38502	BCHE
1	100932059	101032059	0.38502	BCHE
1	100932156	101032156	0.38644	BCHE

1	100932178	101032178	0.365815	BCHE
1	100932516	101032516	0.365786	BCHE
1	100932899	101032899	0.370693	BCHE
1	100932972	101032972	0.366783	BCHE
1	100933048	101033048	0.366783	BCHE
1	100933252	101033252	0.366083	BCHE
1	100933383	101033383	0.360749	BCHE
1	100933417	101033417	0.360749	BCHE
1	100933749	101033749	0.366863	BCHE
1	100933930	101033930	0.366863	BCHE
1	100933937	101033937	0.401557	BCHE
1	100933960	101033960	0.434735	BCHE
1	100934003	101034003	0.429948	BCHE
1	100934027	101034027	0.425909	BCHE
1	100934042	101034042	0.425909	BCHE
1	100934094	101034094	0.429472	BCHE
1	100934261	101034261	0.434641	BCHE
1	100934269	101034269	0.41451	BCHE
1	100934496	101034496	0.381768	BCHE
1	100934544	101034544	0.381768	BCHE
1	100934624	101034624	0.341792	BCHE
1	100934630	101034630	0.361347	BCHE
1	100934698	101034698	0.396848	BCHE
1	100934719	101034719	0.424947	BCHE
1	100934720	101034720	0.422359	BCHE
1	100934857	101034857	0.427268	BCHE
1	100934921	101034921	0.403676	BCHE
1	100934935	101034935	0.385012	BCHE
1	100935198	101035198	0.385012	BCHE
1	100935285	101035285	0.382006	BCHE
1	100935286	101035286	0.377456	BCHE
1	100935544	101035544	0.380509	BCHE
1	100935553	101035553	0.393684	BCHE
1	100935595	101035595	0.413483	BCHE
1	100935626	101035626	0.387783	BCHE
1	100935650	101035650	0.387783	BCHE
1	100935734	101035734	0.387783	BCHE
1	100935821	101035821	0.387783	BCHE
1	100935849	101035849	0.402041	BCHE
1	100935857	101035857	0.41662	BCHE
1	100935953	101035953	0.382887	BCHE
1	100936381	101036381	0.382887	BCHE
1	100936589	101036589	0.384513	BCHE
1	100936852	101036852	0.40163	BCHE
1	100936970	101036970	0.390882	BCHE
1	100937056	101037056	0.414053	BCHE
1	100937069	101037069	0.41921	BCHE
1	100937092	101037092	0.413109	BCHE
1	100937103	101037103	0.413109	BCHE
1	100937226	101037226	0.400939	BCHE
1	100937318	101037318	0.399101	BCHE
1	100937319	101037319	0.399101	BCHE
1	100937342	101037342	0.389423	BCHE
1	100937522	101037522	0.384001	BCHE
1	100937629	101037629	0.405062	BCHE
1	100937655	101037655	0.409243	BCHE
1	100937753	101037753	0.399341	BCHE
1	100937755	101037755	0.399341	BCHE

1	100937913	101037913	0.413317	BCHE
1	100938152	101038152	0.423074	BCHE
1	100938431	101038431	0.418973	BCHE
1	100938464	101038464	0.400515	BCHE
1	100938633	101038633	0.388356	BCHE
1	100938769	101038769	0.391695	BCHE
1	100938791	101038791	0.405371	BCHE
1	100938827	101038827	0.402893	BCHE
1	100938851	101038851	0.416681	BCHE
1	100938865	101038865	0.419483	BCHE
1	100939040	101039040	0.419483	BCHE
1	100939130	101039130	0.411316	BCHE
1	100939173	101039173	0.415629	BCHE
1	100939180	101039180	0.413933	BCHE
1	100939279	101039279	0.420806	BCHE
1	100939389	101039389	0.41379	BCHE
1	100939405	101039405	0.403222	BCHE
1	100939439	101039439	0.404102	BCHE
1	100939534	101039534	0.394156	BCHE
1	100939568	101039568	0.410593	BCHE
1	100939577	101039577	0.435502	BCHE
1	100939690	101039690	0.422828	BCHE
1	100939728	101039728	0.408729	BCHE
1	100939971	101039971	0.419107	BCHE
1	100940035	101040035	0.419107	BCHE
1	100940103	101040103	0.419107	BCHE
1	100940241	101040241	0.419107	BCHE
1	100940319	101040319	0.417207	BCHE
1	100940333	101040333	0.417207	BCHE
1	100940410	101040410	0.407392	BCHE
1	100940507	101040507	0.407392	BCHE
1	100940538	101040538	0.421166	BCHE
1	100940641	101040641	0.417074	BCHE
1	100940694	101040694	0.419352	BCHE
1	100940780	101040780	0.409025	BCHE
1	100940798	101040798	0.426693	BCHE
1	100940903	101040903	0.426693	BCHE
1	100940998	101040998	0.426693	BCHE
1	100941054	101041054	0.426693	BCHE
1	100941188	101041188	0.416705	BCHE
1	100941324	101041324	0.415829	BCHE
1	100941338	101041338	0.415829	BCHE
1	100941344	101041344	0.425689	BCHE
1	100941531	101041531	0.403766	BCHE
1	100941639	101041639	0.436592	BCHE
1	100941694	101041694	0.433512	BCHE
1	100941869	101041869	0.433512	BCHE
1	100941931	101041931	0.445333	BCHE
1	100942050	101042050	0.446169	BCHE
1	100942067	101042067	0.435256	BCHE
1	100942488	101042488	0.40747	BCHE
1	100942665	101042665	0.418767	BCHE
1	100942752	101042752	0.448566	BCHE
1	100942773	101042773	0.448566	BCHE
1	100942825	101042825	0.373507	BCHE
1	100942883	101042883	0.42107	BCHE
1	100942887	101042887	0.42107	BCHE
1	100942934	101042934	0.42979	BCHE

1	100943020	101043020	0.398887	BCHE
1	100943063	101043063	0.409778	BCHE
1	100943126	101043126	0.390344	BCHE
1	100943165	101043165	0.397941	BCHE
1	100943213	101043213	0.392249	BCHE
1	100943249	101043249	0.371306	BCHE
1	100943449	101043449	0.362976	BCHE
1	100943453	101043453	0.365322	BCHE
1	100943951	101043951	0.359179	BCHE
1	100944082	101044082	0.359179	BCHE
1	100944217	101044217	0.376687	BCHE
1	100944304	101044304	0.342479	BCHE
1	100944356	101044356	0.342479	BCHE
1	100944709	101044709	0.341895	BCHE
1	100944774	101044774	0.367335	BCHE
1	100944953	101044953	0.376131	BCHE
1	100945030	101045030	0.385605	BCHE
1	100945053	101045053	0.388381	BCHE
1	100945217	101045217	0.39435	BCHE
1	100945375	101045375	0.39435	BCHE
1	100945402	101045402	0.393944	BCHE
1	100945429	101045429	0.369024	BCHE
1	100945513	101045513	0.373136	BCHE
1	100945529	101045529	0.543321	BCHE
1	100945600	101045600	0.508211	BCHE
1	100945667	101045667	0.507337	BCHE
1	100945839	101045839	0.507815	BCHE
1	100945899	101045899	0.485255	BCHE
1	100945903	101045903	0.501729	BCHE
1	100945904	101045904	0.501729	BCHE
1	100945930	101045930	0.500383	BCHE
1	100945972	101045972	0.518962	BCHE
1	100945981	101045981	0.489746	BCHE
1	100945991	101045991	0.537104	BCHE
1	100945997	101045997	0.504131	BCHE
1	100946041	101046041	0.470179	BCHE
1	100946107	101046107	0.454677	BCHE
1	100946160	101046160	0.429713	BCHE
1	100946365	101046365	0.434675	BCHE
1	100946468	101046468	0.416732	BCHE
1	100946721	101046721	0.417405	BCHE
1	100946777	101046777	0.43078	BCHE
1	100946869	101046869	0.442109	BCHE
1	100946917	101046917	0.442109	BCHE
1	100947018	101047018	0.442109	BCHE
1	100947020	101047020	0.442837	BCHE
1	100947070	101047070	0.450411	BCHE
1	100947332	101047332	0.434378	BCHE
1	100947365	101047365	0.434378	BCHE
1	100947366	101047366	0.434378	BCHE
1	100947373	101047373	0.434378	BCHE
1	100947420	101047420	0.434378	BCHE
1	100947513	101047513	0.434378	BCHE
1	100947543	101047543	0.443475	BCHE
1	100947579	101047579	0.404006	BCHE
1	100947630	101047630	0.414977	BCHE
1	100947736	101047736	0.411342	BCHE
1	100947737	101047737	0.411342	BCHE

1	100947738	101047738	0.410161	BCHE
1	100947834	101047834	0.412522	BCHE
1	100947858	101047858	0.420606	BCHE
1	100947897	101047897	0.420606	BCHE
1	100948044	101048044	0.420606	BCHE
1	100948091	101048091	0.420606	BCHE
1	100948354	101048354	0.41686	BCHE
1	100948459	101048459	0.410016	BCHE
1	100948460	101048460	0.366716	BCHE
1	100948658	101048658	0.366716	BCHE
1	100948826	101048826	0.370406	BCHE
1	100948916	101048916	0.358739	BCHE
1	100948979	101048979	0.358739	BCHE
1	100949094	101049094	0.374589	BCHE
1	100954804	101054804	0.389006	BCHE
1	100954819	101054819	0.387336	BCHE
1	100954885	101054885	0.387336	BCHE
1	100954927	101054927	0.385571	BCHE
1	100954970	101054970	0.387273	BCHE
1	100955016	101055016	0.387892	BCHE
1	100955129	101055129	0.385284	BCHE
1	100955172	101055172	0.385284	BCHE
1	100955192	101055192	0.386302	BCHE
1	100955238	101055238	0.386302	BCHE
1	100955290	101055290	0.412342	BCHE
1	100955294	101055294	0.407502	BCHE
1	100955396	101055396	0.392879	BCHE
1	100955439	101055439	0.385381	BCHE
1	100955482	101055482	0.387899	BCHE
1	100955483	101055483	0.367829	BCHE
1	100955513	101055513	0.357543	BCHE
1	100955539	101055539	0.385508	BCHE
1	100955563	101055563	0.400693	BCHE
1	100955724	101055724	0.400693	BCHE
1	100955842	101055842	0.400693	BCHE
1	100955858	101055858	0.407798	BCHE
1	100955864	101055864	0.407932	BCHE
1	100955923	101055923	0.418572	BCHE
1	100955960	101055960	0.412242	BCHE
1	100956054	101056054	0.408514	BCHE
1	100956094	101056094	0.393966	BCHE
1	100956628	101056628	0.391303	BCHE
1	100956629	101056629	0.407322	BCHE
1	100956631	101056631	0.400697	BCHE
1	100956689	101056689	0.369866	BCHE
1	100956690	101056690	0.372183	BCHE
1	100956705	101056705	0.372185	BCHE
1	100956724	101056724	0.372185	BCHE
1	100956770	101056770	0.372185	BCHE
1	100956937	101056937	0.372185	BCHE
1	100956939	101056939	0.370585	BCHE
1	100956975	101056975	0.368558	BCHE
1	100957010	101057010	0.368558	BCHE
1	100957259	101057259	0.362359	BCHE
1	100957426	101057426	0.358389	BCHE
1	100957465	101057465	0.358389	BCHE
1	100957619	101057619	0.361168	BCHE
1	100957790	101057790	0.369085	BCHE

1	100957814	101057814	0.369284	BCHE
1	100957822	101057822	0.372022	BCHE
1	100957911	101057911	0.378343	BCHE
1	100957923	101057923	0.372899	BCHE
1	100958010	101058010	0.379998	BCHE
1	100958062	101058062	0.377014	BCHE
1	100958079	101058079	0.418106	BCHE
1	100958100	101058100	0.434023	BCHE
1	100958151	101058151	0.427138	BCHE
1	100958170	101058170	0.427138	BCHE
1	100958189	101058189	0.427138	BCHE
1	100958192	101058192	0.431772	BCHE
1	100958225	101058225	0.423311	BCHE
1	100958255	101058255	0.423311	BCHE
1	100958351	101058351	0.422528	BCHE
1	100958472	101058472	0.40267	BCHE
1	100958618	101058618	0.404845	BCHE
1	100958629	101058629	0.403378	BCHE
1	100958778	101058778	0.417858	BCHE
1	100959126	101059126	0.417069	BCHE
1	100959164	101059164	0.417069	BCHE
1	100959174	101059174	0.417069	BCHE
1	100959213	101059213	0.432699	BCHE
1	100959279	101059279	0.421309	BCHE
1	100959301	101059301	0.424552	BCHE
1	100959304	101059304	0.41571	BCHE
1	100959403	101059403	0.41522	BCHE
1	100959452	101059452	0.422655	BCHE
1	100959464	101059464	0.420181	BCHE
1	100959488	101059488	0.421696	BCHE
1	100959519	101059519	0.423847	BCHE
1	100959739	101059739	0.424737	BCHE
1	100959820	101059820	0.439433	BCHE
1	100959966	101059966	0.439433	BCHE
1	100960325	101060325	0.460492	BCHE
1	100960329	101060329	0.458861	BCHE
1	100960335	101060335	0.450739	BCHE
1	100960406	101060406	0.456142	BCHE
1	100960422	101060422	0.437906	BCHE
1	100960485	101060485	0.411896	BCHE
1	100960901	101060901	0.411896	BCHE
1	100960943	101060943	0.411896	BCHE
1	100960952	101060952	0.424335	BCHE
1	100961136	101061136	0.424335	BCHE
1	100961204	101061204	0.42211	BCHE
1	100961223	101061223	0.441151	BCHE
1	100961570	101061570	0.425997	BCHE
1	100961577	101061577	0.447041	BCHE
1	100961612	101061612	0.460529	BCHE
1	100961613	101061613	0.445021	BCHE
1	100961674	101061674	0.446558	BCHE
1	100961789	101061789	0.446496	BCHE
1	100961895	101061895	0.446496	BCHE
1	100961972	101061972	0.441181	BCHE
1	100961985	101061985	0.435059	BCHE
1	100962174	101062174	0.416758	BCHE
1	100962236	101062236	0.418191	BCHE
1	100962276	101062276	0.396187	BCHE

1	100962407	101062407	0.396187	BCHE
1	100962448	101062448	0.396187	BCHE
1	100962480	101062480	0.394208	BCHE
1	100962679	101062679	0.397577	BCHE
1	100962774	101062774	0.392805	BCHE
1	100962790	101062790	0.392805	BCHE
1	100962848	101062848	0.392805	BCHE
1	100962862	101062862	0.392836	BCHE
1	100962865	101062865	0.418609	BCHE
1	100962866	101062866	0.415067	BCHE
1	100962870	101062870	0.415067	BCHE
1	100962878	101062878	0.418202	BCHE
1	100962879	101062879	0.418202	BCHE
1	100962925	101062925	0.409644	BCHE
1	100962931	101062931	0.409644	BCHE
1	100962959	101062959	0.408193	BCHE
1	100962999	101062999	0.412383	BCHE
1	100963021	101063021	0.426641	BCHE
1	100963050	101063050	0.426641	BCHE
1	100963062	101063062	0.426641	BCHE
1	100963116	101063116	0.426641	BCHE
1	100963146	101063146	0.425737	BCHE
1	100963286	101063286	0.420716	BCHE
1	100963301	101063301	0.409262	BCHE
1	100963363	101063363	0.416247	BCHE
1	100963547	101063547	0.393447	BCHE
1	100963575	101063575	0.412129	BCHE
1	100963600	101063600	0.412443	BCHE
1	100963602	101063602	0.412443	BCHE
1	100963647	101063647	0.419461	BCHE
1	100963722	101063722	0.419461	BCHE
1	100963736	101063736	0.419461	BCHE
1	100963883	101063883	0.413905	BCHE
1	100964034	101064034	0.413905	BCHE
1	100964125	101064125	0.420333	BCHE
1	100964126	101064126	0.417479	BCHE
1	100964153	101064153	0.417479	BCHE
1	100964246	101064246	0.422639	BCHE
1	100964263	101064263	0.422639	BCHE
1	100964267	101064267	0.422639	BCHE
1	100964311	101064311	0.415422	BCHE
1	100964436	101064436	0.416907	BCHE
1	100964466	101064466	0.39503	BCHE
1	100964503	101064503	0.398498	BCHE
1	100964527	101064527	0.420151	BCHE
1	100964636	101064636	0.420151	BCHE
1	100964732	101064732	0.419108	BCHE
1	100964782	101064782	0.428459	BCHE
1	100964785	101064785	0.432101	BCHE
1	100964798	101064798	0.432101	BCHE
1	100965037	101065037	0.432101	BCHE
1	100965056	101065056	0.432101	BCHE
1	100965137	101065137	0.421464	BCHE
1	100965181	101065181	0.418332	BCHE
1	100965362	101065362	0.390761	BCHE
1	100965426	101065426	0.347347	BCHE
1	100965427	101065427	0.347347	BCHE
1	100965433	101065433	0.354451	BCHE

1	100965453	101065453	0.354451	BCHE
1	100965467	101065467	0.345096	BCHE
1	100965564	101065564	0.354439	BCHE
1	100965685	101065685	0.354439	BCHE
1	100965802	101065802	0.354439	BCHE
1	100965838	101065838	0.354451	BCHE
1	100965895	101065895	0.354451	BCHE
1	100966041	101066041	0.343133	BCHE
1	100966553	101066553	0.371238	BCHE
1	100966695	101066695	0.342375	BCHE
1	100966696	101066696	0.342375	BCHE
1	100966902	101066902	0.342375	BCHE
1	100967416	101067416	0.342375	BCHE
1	100967443	101067443	0.342371	BCHE
1	100967466	101067466	0.342371	BCHE
1	100967620	101067620	0.357209	BCHE
1	100968024	101068024	0.366466	BCHE
1	100970060	101070060	0.352208	BCHE
1	100971116	101071116	0.38131	BCHE
1	100971190	101071190	0.346583	BCHE
1	100971294	101071294	0.346583	BCHE
1	100971307	101071307	0.353415	BCHE
1	100971361	101071361	0.353522	BCHE
1	100971378	101071378	0.348508	BCHE
1	100971379	101071379	0.348645	BCHE
1	100971537	101071537	0.359641	BCHE
1	100971619	101071619	0.356475	BCHE
1	100972215	101072215	0.342792	BCHE
1	100972217	101072217	0.348883	BCHE
1	100976079	101076079	0.342322	BCHE
1	101000869	101100869	0.344304	BCHE
1	101007286	101107286	0.362684	BCHE
1	101007311	101107311	0.390461	BCHE
1	101007331	101107331	0.382361	BCHE
1	105414164	105514164	0.345608	LOC108636899
1	105414164	105514164	0.345608	OTOL1
1	105414322	105514322	0.360746	LOC108636899
1	105414322	105514322	0.360746	OTOL1
1	105414351	105514351	0.346946	LOC108636899
1	105414351	105514351	0.346946	OTOL1
1	105414613	105514613	0.352985	LOC108636899
1	105414613	105514613	0.352985	OTOL1
1	105414686	105514686	0.353737	LOC108636899
1	105414686	105514686	0.353737	OTOL1
1	105414764	105514764	0.342078	LOC108636899
1	105414764	105514764	0.342078	OTOL1
1	105415135	105515135	0.358276	LOC108636899
1	105415135	105515135	0.358276	OTOL1
1	105415194	105515194	0.34467	LOC108636899
1	105415194	105515194	0.34467	OTOL1
1	105417262	105517262	0.348245	LOC108636899
1	105417262	105517262	0.348245	OTOL1
1	105417444	105517444	0.342128	LOC108636899
1	105417444	105517444	0.342128	OTOL1
1	105420952	105520952	0.349341	LOC108636899
1	105420952	105520952	0.349341	OTOL1
1	105420992	105520992	0.348044	LOC108636899
1	105420992	105520992	0.348044	OTOL1

1	105421686	105521686	0.345566	LOC108636899
1	105421686	105521686	0.345566	OTOL1
1	105421694	105521694	0.341989	LOC108636899
1	105421694	105521694	0.341989	OTOL1
1	105421708	105521708	0.341989	LOC108636899
1	105421708	105521708	0.341989	OTOL1
1	105421718	105521718	0.341989	LOC108636899
1	105421718	105521718	0.341989	OTOL1
1	105421719	105521719	0.341989	LOC108636899
1	105421719	105521719	0.341989	OTOL1
1	105421749	105521749	0.345602	LOC108636899
1	105421749	105521749	0.345602	OTOL1
1	105423716	105523716	0.348206	LOC108636899
1	105423716	105523716	0.348206	OTOL1
1	105423731	105523731	0.344955	LOC108636899
1	105423731	105523731	0.344955	OTOL1
1	105423740	105523740	0.35324	LOC108636899
1	105423740	105523740	0.35324	OTOL1
1	105423860	105523860	0.357647	LOC108636899
1	105423860	105523860	0.357647	OTOL1
1	105424017	105524017	0.349728	LOC108636899
1	105424017	105524017	0.349728	OTOL1
1	105424070	105524070	0.353122	LOC108636899
1	105424070	105524070	0.353122	OTOL1
1	105424149	105524149	0.350074	LOC108636899
1	105424149	105524149	0.350074	OTOL1
1	105424150	105524150	0.347935	LOC108636899
1	105424150	105524150	0.347935	OTOL1
1	105424182	105524182	0.347935	LOC108636899
1	105424182	105524182	0.347935	OTOL1
1	105424433	105524433	0.342353	LOC108636899
1	105424433	105524433	0.342353	OTOL1
1	105424773	105524773	0.349164	LOC108636899
1	105424773	105524773	0.349164	OTOL1
1	105424954	105524954	0.348625	LOC108636899
1	105424954	105524954	0.348625	OTOL1
1	105425095	105525095	0.348625	LOC108636899
1	105425095	105525095	0.348625	OTOL1
1	105425112	105525112	0.36872	LOC108636899
1	105425112	105525112	0.36872	OTOL1
1	105425164	105525164	0.34891	LOC108636899
1	105425164	105525164	0.34891	OTOL1
1	105425201	105525201	0.347219	LOC108636899
1	105425201	105525201	0.347219	OTOL1
1	105425837	105525837	0.342418	LOC108636899
1	105425837	105525837	0.342418	OTOL1
1	105428125	105528125	0.345872	LOC108636899
1	105428125	105528125	0.345872	OTOL1
1	105811712	105911712	0.4037	B3GALNT1
1	105811830	105911830	0.372648	B3GALNT1
1	106832204	106932204	0.349477	LOC102180430
1	106832261	106932261	0.349477	LOC102180430
1	106832631	106932631	0.384137	LOC102180430
1	106832817	106932817	0.390551	LOC102180430
1	106832845	106932845	0.442646	LOC102180430
1	106832859	106932859	0.400348	LOC102180430
1	106832933	106932933	0.404984	LOC102180430
1	106833004	106933004	0.390888	LOC102180430

1	106833034	106933034	0.408232	LOC102180430
1	106833345	106933345	0.357552	LOC102180430
1	106833710	106933710	0.349843	LOC102180430
1	106833713	106933713	0.348434	LOC102180430
1	108613867	108713867	0.353812	RSRC1
1	110480299	110580299	0.369516	TIPARP
1	110480509	110580509	0.354988	TIPARP
1	110483261	110583261	0.352238	TIPARP
1	110483459	110583459	0.35025	TIPARP
1	110483682	110583682	0.354281	TIPARP
1	110484117	110584117	0.356582	TIPARP
1	110484493	110584493	0.347642	TIPARP
1	110484807	110584807	0.351781	TIPARP
1	110484818	110584818	0.359426	TIPARP
1	110484852	110584852	0.36291	TIPARP
1	110484988	110584988	0.359086	TIPARP
1	110485060	110585060	0.359086	TIPARP
1	110485473	110585473	0.358031	TIPARP
1	110485920	110585920	0.358252	TIPARP
1	110486061	110586061	0.356161	TIPARP
1	110486393	110586393	0.358296	TIPARP
1	110486509	110586509	0.35153	TIPARP
1	110486659	110586659	0.35153	TIPARP
1	110487197	110587197	0.35153	TIPARP
1	110487310	110587310	0.348519	TIPARP
1	110487971	110587971	0.349236	TIPARP
1	110488069	110588069	0.354247	TIPARP
1	110488756	110588756	0.351881	TIPARP
1	110488893	110588893	0.354028	TIPARP
1	110489143	110589143	0.360829	TIPARP
1	110489465	110589465	0.360107	TIPARP
1	110490024	110590024	0.351541	TIPARP
1	110490398	110590398	0.348125	TIPARP
1	110490789	110590789	0.350588	TIPARP
1	110491546	110591546	0.34878	TIPARP
1	110491824	110591824	0.351287	TIPARP
1	110492102	110592102	0.353438	TIPARP
1	110492261	110592261	0.359847	TIPARP
1	110492713	110592713	0.355589	TIPARP
1	110493219	110593219	0.359399	TIPARP
1	110493341	110593341	0.358831	TIPARP
1	110493489	110593489	0.356114	TIPARP
1	110494390	110594390	0.353812	TIPARP
1	110495294	110595294	0.349428	TIPARP
1	110495551	110595551	0.349377	TIPARP
1	110495715	110595715	0.37338	TIPARP
1	110496060	110596060	0.37338	TIPARP
1	110496182	110596182	0.365414	TIPARP
1	110496984	110596984	0.364169	TIPARP
1	110497211	110597211	0.376152	TIPARP
1	110497578	110597578	0.372676	TIPARP
1	110497627	110597627	0.378107	TIPARP
1	110497857	110597857	0.377853	TIPARP
1	110498475	110598475	0.378305	TIPARP
1	110498484	110598484	0.380452	TIPARP
1	110498492	110598492	0.370953	TIPARP
1	110498520	110598520	0.370942	TIPARP
1	110498678	110598678	0.371746	TIPARP

1	110498711	110598711	0.381164	TIPARP
1	110499013	110599013	0.381297	TIPARP
1	110499062	110599062	0.377766	TIPARP
1	110500116	110600116	0.375279	TIPARP
1	110500237	110600237	0.373128	TIPARP
1	110500572	110600572	0.374759	TIPARP
1	110500779	110600779	0.362513	TIPARP
1	110500879	110600879	0.357311	TIPARP
1	110500962	110600962	0.357807	TIPARP
1	110500980	110600980	0.354795	TIPARP
1	110500987	110600987	0.354795	TIPARP
1	110501083	110601083	0.343338	TIPARP
1	110501970	110601970	0.343338	TIPARP
1	110504251	110604251	0.353301	TIPARP
1	110505486	110605486	0.343472	TIPARP
1	110505985	110605985	0.345272	TIPARP
1	110506712	110606712	0.342589	TIPARP
1	110542724	110642724	0.361416	SSR3
1	110543796	110643796	0.365022	SSR3
1	110543811	110643811	0.366932	SSR3
1	110544201	110644201	0.358351	SSR3
1	110544286	110644286	0.358085	SSR3
1	110545482	110645482	0.351166	SSR3
1	110545504	110645504	0.354941	SSR3
1	110545530	110645530	0.354845	SSR3
1	110545533	110645533	0.354212	SSR3
1	110545907	110645907	0.366678	SSR3
1	110546507	110646507	0.363834	SSR3
1	110546752	110646752	0.366667	SSR3
1	110547011	110647011	0.366667	SSR3
1	110547758	110647758	0.388406	SSR3
1	110547792	110647792	0.387078	SSR3
1	110547799	110647799	0.389019	SSR3
1	110547836	110647836	0.395983	SSR3
1	110547892	110647892	0.3952	SSR3
1	110547993	110647993	0.396131	SSR3
1	110548007	110648007	0.396566	SSR3
1	110548370	110648370	0.406592	SSR3
1	110548630	110648630	0.404379	SSR3
1	110549002	110649002	0.407578	SSR3
1	110549096	110649096	0.401885	SSR3
1	110549199	110649199	0.407472	SSR3
1	110549348	110649348	0.402043	SSR3
1	110549464	110649464	0.400936	SSR3
1	110549971	110649971	0.400909	SSR3
1	110550316	110650316	0.40373	SSR3
1	110550572	110650572	0.394672	SSR3
1	110550973	110650973	0.399707	SSR3
1	110551463	110651463	0.395021	SSR3
1	110551775	110651775	0.394336	SSR3
1	110553791	110653791	0.404135	SSR3
1	110553963	110653963	0.405827	SSR3
1	110554048	110654048	0.408556	SSR3
1	110554087	110654087	0.39992	SSR3
1	110554641	110654641	0.404198	SSR3
1	110554677	110654677	0.406287	SSR3
1	110555202	110655202	0.409352	SSR3
1	110555234	110655234	0.403384	SSR3

1	110555442	110655442	0.397613	SSR3
1	110555640	110655640	0.388096	SSR3
1	110555679	110655679	0.404692	SSR3
1	110556772	110656772	0.454121	SSR3
1	110556824	110656824	0.462281	SSR3
1	110557279	110657279	0.472204	SSR3
1	110557391	110657391	0.466953	SSR3
1	110557391	110657391	0.466953	KCNAB1
1	110557505	110657505	0.464697	SSR3
1	110557505	110657505	0.464697	KCNAB1
1	110557950	110657950	0.481607	SSR3
1	110557950	110657950	0.481607	KCNAB1
1	110558485	110658485	0.466616	SSR3
1	110558485	110658485	0.466616	KCNAB1
1	110558952	110658952	0.474856	SSR3
1	110558952	110658952	0.474856	KCNAB1
1	110559009	110659009	0.481894	SSR3
1	110559009	110659009	0.481894	KCNAB1
1	110559359	110659359	0.491159	SSR3
1	110559359	110659359	0.491159	KCNAB1
1	110559800	110659800	0.490026	SSR3
1	110559800	110659800	0.490026	KCNAB1
1	110560041	110660041	0.490494	SSR3
1	110560041	110660041	0.490494	KCNAB1
1	110560235	110660235	0.503352	SSR3
1	110560235	110660235	0.503352	KCNAB1
1	110560335	110660335	0.503352	SSR3
1	110560335	110660335	0.503352	KCNAB1
1	110560627	110660627	0.505402	SSR3
1	110560627	110660627	0.505402	KCNAB1
1	110560698	110660698	0.509673	SSR3
1	110560698	110660698	0.509673	KCNAB1
1	110560817	110660817	0.497611	SSR3
1	110560817	110660817	0.497611	KCNAB1
1	110560982	110660982	0.509826	SSR3
1	110560982	110660982	0.509826	KCNAB1
1	110561081	110661081	0.505232	SSR3
1	110561081	110661081	0.505232	KCNAB1
1	110561625	110661625	0.505171	SSR3
1	110561625	110661625	0.505171	KCNAB1
1	110561677	110661677	0.508546	SSR3
1	110561677	110661677	0.508546	KCNAB1
1	110561786	110661786	0.512848	SSR3
1	110561786	110661786	0.512848	KCNAB1
1	110561888	110661888	0.515237	SSR3
1	110561888	110661888	0.515237	KCNAB1
1	110561957	110661957	0.515237	SSR3
1	110561957	110661957	0.515237	KCNAB1
1	110561959	110661959	0.510122	SSR3
1	110561959	110661959	0.510122	KCNAB1
1	110562074	110662074	0.51916	SSR3
1	110562074	110662074	0.51916	KCNAB1
1	110562196	110662196	0.499169	SSR3
1	110562196	110662196	0.499169	KCNAB1
1	110562515	110662515	0.499969	SSR3
1	110562515	110662515	0.499969	KCNAB1
1	110562538	110662538	0.50684	SSR3
1	110562538	110662538	0.50684	KCNAB1

1	110562545	110662545	0.505531	SSR3
1	110562545	110662545	0.505531	KCNAB1
1	110562577	110662577	0.509821	SSR3
1	110562577	110662577	0.509821	KCNAB1
1	110563152	110663152	0.50347	SSR3
1	110563152	110663152	0.50347	KCNAB1
1	110563431	110663431	0.495921	SSR3
1	110563431	110663431	0.495921	KCNAB1
1	110563660	110663660	0.501553	SSR3
1	110563660	110663660	0.501553	KCNAB1
1	110563768	110663768	0.494177	SSR3
1	110563768	110663768	0.494177	KCNAB1
1	110564198	110664198	0.487283	SSR3
1	110564198	110664198	0.487283	KCNAB1
1	110564617	110664617	0.488112	SSR3
1	110564617	110664617	0.488112	KCNAB1
1	110564989	110664989	0.495163	SSR3
1	110564989	110664989	0.495163	KCNAB1
1	110565642	110665642	0.489869	SSR3
1	110565642	110665642	0.489869	KCNAB1
1	110565885	110665885	0.493752	SSR3
1	110565885	110665885	0.493752	KCNAB1
1	110565988	110665988	0.485213	SSR3
1	110565988	110665988	0.485213	KCNAB1
1	110566321	110666321	0.467261	SSR3
1	110566321	110666321	0.467261	KCNAB1
1	110567173	110667173	0.477624	SSR3
1	110567173	110667173	0.477624	KCNAB1
1	110567196	110667196	0.481718	SSR3
1	110567196	110667196	0.481718	KCNAB1
1	110567325	110667325	0.481718	SSR3
1	110567325	110667325	0.481718	KCNAB1
1	110567767	110667767	0.493704	SSR3
1	110567767	110667767	0.493704	KCNAB1
1	110567803	110667803	0.489984	SSR3
1	110567803	110667803	0.489984	KCNAB1
1	110567885	110667885	0.488173	SSR3
1	110567885	110667885	0.488173	KCNAB1
1	110568993	110668993	0.494551	SSR3
1	110568993	110668993	0.494551	KCNAB1
1	110569015	110669015	0.491788	SSR3
1	110569015	110669015	0.491788	KCNAB1
1	110569061	110669061	0.488738	SSR3
1	110569061	110669061	0.488738	KCNAB1
1	110570060	110670060	0.474721	SSR3
1	110570060	110670060	0.474721	KCNAB1
1	110570119	110670119	0.4454	SSR3
1	110570119	110670119	0.4454	KCNAB1
1	110570722	110670722	0.450555	SSR3
1	110570722	110670722	0.450555	KCNAB1
1	110570816	110670816	0.466659	SSR3
1	110570816	110670816	0.466659	KCNAB1
1	110571166	110671166	0.443542	SSR3
1	110571166	110671166	0.443542	KCNAB1
1	110571429	110671429	0.43514	SSR3
1	110571429	110671429	0.43514	KCNAB1
1	110571819	110671819	0.435761	SSR3
1	110571819	110671819	0.435761	KCNAB1

1	110572523	110672523	0.43843	SSR3
1	110572523	110672523	0.43843	KCNAB1
1	110572793	110672793	0.43843	SSR3
1	110572793	110672793	0.43843	KCNAB1
1	110573298	110673298	0.42386	SSR3
1	110573298	110673298	0.42386	KCNAB1
1	110575376	110675376	0.426572	SSR3
1	110575376	110675376	0.426572	KCNAB1
1	110575377	110675377	0.426572	SSR3
1	110575377	110675377	0.426572	KCNAB1
1	110575767	110675767	0.456176	SSR3
1	110575767	110675767	0.456176	KCNAB1
1	110576031	110676031	0.459156	SSR3
1	110576031	110676031	0.459156	KCNAB1
1	110576358	110676358	0.484968	SSR3
1	110576358	110676358	0.484968	KCNAB1
1	110576655	110676655	0.492527	SSR3
1	110576655	110676655	0.492527	KCNAB1
1	110577019	110677019	0.496255	SSR3
1	110577019	110677019	0.496255	KCNAB1
1	110577092	110677092	0.498827	SSR3
1	110577092	110677092	0.498827	KCNAB1
1	110577232	110677232	0.498827	SSR3
1	110577232	110677232	0.498827	KCNAB1
1	110577393	110677393	0.501278	SSR3
1	110577393	110677393	0.501278	KCNAB1
1	110577413	110677413	0.506177	SSR3
1	110577413	110677413	0.506177	KCNAB1
1	110577444	110677444	0.4956	SSR3
1	110577444	110677444	0.4956	KCNAB1
1	110577627	110677627	0.500224	SSR3
1	110577627	110677627	0.500224	KCNAB1
1	110577678	110677678	0.500224	SSR3
1	110577678	110677678	0.500224	KCNAB1
1	110577704	110677704	0.509861	SSR3
1	110577704	110677704	0.509861	KCNAB1
1	110577819	110677819	0.511817	SSR3
1	110577819	110677819	0.511817	KCNAB1
1	110577918	110677918	0.526756	SSR3
1	110577918	110677918	0.526756	KCNAB1
1	110577931	110677931	0.520584	SSR3
1	110577931	110677931	0.520584	KCNAB1
1	110578085	110678085	0.517128	SSR3
1	110578085	110678085	0.517128	KCNAB1
1	110578087	110678087	0.515751	SSR3
1	110578087	110678087	0.515751	KCNAB1
1	110578375	110678375	0.50577	SSR3
1	110578375	110678375	0.50577	KCNAB1
1	110578579	110678579	0.504411	SSR3
1	110578579	110678579	0.504411	KCNAB1
1	110578752	110678752	0.503685	SSR3
1	110578752	110678752	0.503685	KCNAB1
1	110578882	110678882	0.509281	SSR3
1	110578882	110678882	0.509281	KCNAB1
1	110579155	110679155	0.508909	SSR3
1	110579155	110679155	0.508909	KCNAB1
1	110579301	110679301	0.51244	SSR3
1	110579301	110679301	0.51244	KCNAB1

1	110579386	110679386	0.510542	SSR3
1	110579386	110679386	0.510542	KCNAB1
1	110579387	110679387	0.513118	SSR3
1	110579387	110679387	0.513118	KCNAB1
1	110579399	110679399	0.50209	SSR3
1	110579399	110679399	0.50209	KCNAB1
1	110579553	110679553	0.493475	SSR3
1	110579553	110679553	0.493475	KCNAB1
1	110579605	110679605	0.509029	SSR3
1	110579605	110679605	0.509029	KCNAB1
1	110579608	110679608	0.489733	SSR3
1	110579608	110679608	0.489733	KCNAB1
1	110579731	110679731	0.489733	SSR3
1	110579731	110679731	0.489733	KCNAB1
1	110579817	110679817	0.489733	SSR3
1	110579817	110679817	0.489733	KCNAB1
1	110580074	110680074	0.478874	SSR3
1	110580074	110680074	0.478874	KCNAB1
1	110580353	110680353	0.501082	SSR3
1	110580353	110680353	0.501082	KCNAB1
1	110581001	110681001	0.484204	SSR3
1	110581001	110681001	0.484204	KCNAB1
1	110581143	110681143	0.481643	SSR3
1	110581143	110681143	0.481643	KCNAB1
1	110581962	110681962	0.4851	SSR3
1	110581962	110681962	0.4851	KCNAB1
1	110581976	110681976	0.509123	SSR3
1	110581976	110681976	0.509123	KCNAB1
1	110581981	110681981	0.519192	SSR3
1	110581981	110681981	0.519192	KCNAB1
1	110582047	110682047	0.513719	SSR3
1	110582047	110682047	0.513719	KCNAB1
1	110582121	110682121	0.513972	SSR3
1	110582121	110682121	0.513972	KCNAB1
1	110582428	110682428	0.509859	SSR3
1	110582428	110682428	0.509859	KCNAB1
1	110582690	110682690	0.510095	SSR3
1	110582690	110682690	0.510095	KCNAB1
1	110582797	110682797	0.510095	SSR3
1	110582797	110682797	0.510095	KCNAB1
1	110582841	110682841	0.524641	SSR3
1	110582841	110682841	0.524641	KCNAB1
1	110582910	110682910	0.52179	SSR3
1	110582910	110682910	0.52179	KCNAB1
1	110582962	110682962	0.518021	SSR3
1	110582962	110682962	0.518021	KCNAB1
1	110583276	110683276	0.518495	SSR3
1	110583276	110683276	0.518495	KCNAB1
1	110583522	110683522	0.51951	SSR3
1	110583522	110683522	0.51951	KCNAB1
1	110583529	110683529	0.527917	SSR3
1	110583529	110683529	0.527917	KCNAB1
1	110583651	110683651	0.530877	SSR3
1	110583651	110683651	0.530877	KCNAB1
1	110583692	110683692	0.535597	SSR3
1	110583692	110683692	0.535597	KCNAB1
1	110583782	110683782	0.535098	SSR3
1	110583782	110683782	0.535098	KCNAB1

1	110583822	110683822	0.527025	SSR3
1	110583822	110683822	0.527025	KCNAB1
1	110584008	110684008	0.5272	SSR3
1	110584008	110684008	0.5272	KCNAB1
1	110584052	110684052	0.526518	SSR3
1	110584052	110684052	0.526518	KCNAB1
1	110584095	110684095	0.525317	SSR3
1	110584095	110684095	0.525317	KCNAB1
1	110584297	110684297	0.511147	SSR3
1	110584297	110684297	0.511147	KCNAB1
1	110584401	110684401	0.501524	SSR3
1	110584401	110684401	0.501524	KCNAB1
1	110584757	110684757	0.486068	SSR3
1	110584757	110684757	0.486068	KCNAB1
1	110585013	110685013	0.480918	SSR3
1	110585013	110685013	0.480918	KCNAB1
1	110585554	110685554	0.478953	SSR3
1	110585554	110685554	0.478953	KCNAB1
1	110585848	110685848	0.496354	SSR3
1	110585848	110685848	0.496354	KCNAB1
1	110585859	110685859	0.498964	SSR3
1	110585859	110685859	0.498964	KCNAB1
1	110585971	110685971	0.501926	SSR3
1	110585971	110685971	0.501926	KCNAB1
1	110586070	110686070	0.494064	SSR3
1	110586070	110686070	0.494064	KCNAB1
1	110586072	110686072	0.494889	SSR3
1	110586072	110686072	0.494889	KCNAB1
1	110586489	110686489	0.480479	SSR3
1	110586489	110686489	0.480479	KCNAB1
1	110586837	110686837	0.481541	SSR3
1	110586837	110686837	0.481541	KCNAB1
1	110586949	110686949	0.502388	SSR3
1	110586949	110686949	0.502388	KCNAB1
1	110587343	110687343	0.489038	SSR3
1	110587343	110687343	0.489038	KCNAB1
1	110587868	110687868	0.469005	SSR3
1	110587868	110687868	0.469005	KCNAB1
1	110588501	110688501	0.482708	SSR3
1	110588501	110688501	0.482708	KCNAB1
1	110589711	110689711	0.470438	SSR3
1	110589711	110689711	0.470438	KCNAB1
1	110590187	110690187	0.495905	SSR3
1	110590187	110690187	0.495905	KCNAB1
1	110590343	110690343	0.496268	SSR3
1	110590343	110690343	0.496268	KCNAB1
1	110590353	110690353	0.497412	SSR3
1	110590353	110690353	0.497412	KCNAB1
1	110590368	110690368	0.491069	SSR3
1	110590368	110690368	0.491069	KCNAB1
1	110590518	110690518	0.493681	SSR3
1	110590518	110690518	0.493681	KCNAB1
1	110591179	110691179	0.499163	SSR3
1	110591179	110691179	0.499163	KCNAB1
1	110591557	110691557	0.498235	SSR3
1	110591557	110691557	0.498235	KCNAB1
1	110591758	110691758	0.506384	SSR3
1	110591758	110691758	0.506384	KCNAB1

1	110591786	110691786	0.525161	SSR3
1	110591786	110691786	0.525161	KCNAB1
1	110591800	110691800	0.522241	SSR3
1	110591800	110691800	0.522241	KCNAB1
1	110592022	110692022	0.541225	SSR3
1	110592022	110692022	0.541225	KCNAB1
1	110592081	110692081	0.529403	SSR3
1	110592081	110692081	0.529403	KCNAB1
1	110592353	110692353	0.535308	SSR3
1	110592353	110692353	0.535308	KCNAB1
1	110592674	110692674	0.530746	SSR3
1	110592674	110692674	0.530746	KCNAB1
1	110592881	110692881	0.549355	SSR3
1	110592881	110692881	0.549355	KCNAB1
1	110592933	110692933	0.556468	SSR3
1	110592933	110692933	0.556468	KCNAB1
1	110593116	110693116	0.546685	SSR3
1	110593116	110693116	0.546685	KCNAB1
1	110593361	110693361	0.527018	SSR3
1	110593361	110693361	0.527018	KCNAB1
1	110593791	110693791	0.524332	SSR3
1	110593791	110693791	0.524332	KCNAB1
1	110593848	110693848	0.52509	SSR3
1	110593848	110693848	0.52509	KCNAB1
1	110593939	110693939	0.52875	SSR3
1	110593939	110693939	0.52875	KCNAB1
1	110594313	110694313	0.550231	SSR3
1	110594313	110694313	0.550231	KCNAB1
1	110594914	110694914	0.548527	SSR3
1	110594914	110694914	0.548527	KCNAB1
1	110595009	110695009	0.568392	SSR3
1	110595009	110695009	0.568392	KCNAB1
1	110595054	110695054	0.565212	SSR3
1	110595054	110695054	0.565212	KCNAB1
1	110595082	110695082	0.565212	SSR3
1	110595082	110695082	0.565212	KCNAB1
1	110595167	110695167	0.566291	SSR3
1	110595167	110695167	0.566291	KCNAB1
1	110595175	110695175	0.587556	SSR3
1	110595175	110695175	0.587556	KCNAB1
1	110595257	110695257	0.554224	SSR3
1	110595257	110695257	0.554224	KCNAB1
1	110595444	110695444	0.546745	SSR3
1	110595444	110695444	0.546745	KCNAB1
1	110595614	110695614	0.523336	SSR3
1	110595614	110695614	0.523336	KCNAB1
1	110596071	110696071	0.499973	SSR3
1	110596071	110696071	0.499973	KCNAB1
1	110596546	110696546	0.492769	SSR3
1	110596546	110696546	0.492769	KCNAB1
1	110596613	110696613	0.488033	SSR3
1	110596613	110696613	0.488033	KCNAB1
1	110597811	110697811	0.500336	SSR3
1	110597811	110697811	0.500336	KCNAB1
1	110598029	110698029	0.516862	SSR3
1	110598029	110698029	0.516862	KCNAB1
1	110598210	110698210	0.439838	SSR3
1	110598210	110698210	0.439838	KCNAB1

1	110599210	110699210	0.436015	SSR3
1	110599210	110699210	0.436015	KCNAB1
1	110599601	110699601	0.459833	SSR3
1	110599601	110699601	0.459833	KCNAB1
1	110599919	110699919	0.481527	SSR3
1	110599919	110699919	0.481527	KCNAB1
1	110600076	110700076	0.47714	SSR3
1	110600076	110700076	0.47714	KCNAB1
1	110600108	110700108	0.439074	SSR3
1	110600108	110700108	0.439074	KCNAB1
1	110601196	110701196	0.44353	SSR3
1	110601196	110701196	0.44353	KCNAB1
1	110601286	110701286	0.419915	SSR3
1	110601286	110701286	0.419915	KCNAB1
1	110601750	110701750	0.418398	SSR3
1	110601750	110701750	0.418398	KCNAB1
1	110601839	110701839	0.429152	SSR3
1	110601839	110701839	0.429152	KCNAB1
1	110601950	110701950	0.406322	SSR3
1	110601950	110701950	0.406322	KCNAB1
1	110604394	110704394	0.405308	SSR3
1	110604394	110704394	0.405308	KCNAB1
1	110604454	110704454	0.424308	SSR3
1	110604454	110704454	0.424308	KCNAB1
1	110605704	110705704	0.392774	SSR3
1	110605704	110705704	0.392774	KCNAB1
1	110605996	110705996	0.375132	SSR3
1	110605996	110705996	0.375132	KCNAB1
1	110606277	110706277	0.370049	SSR3
1	110606277	110706277	0.370049	KCNAB1
1	110607232	110707232	0.367797	SSR3
1	110607232	110707232	0.367797	KCNAB1
1	110607320	110707320	0.379238	SSR3
1	110607320	110707320	0.379238	KCNAB1
1	110607671	110707671	0.384126	SSR3
1	110607671	110707671	0.384126	KCNAB1
1	110607949	110707949	0.407801	SSR3
1	110607949	110707949	0.407801	KCNAB1
1	110608248	110708248	0.407784	SSR3
1	110608248	110708248	0.407784	KCNAB1
1	110608472	110708472	0.389055	SSR3
1	110608472	110708472	0.389055	KCNAB1
1	110608722	110708722	0.388365	SSR3
1	110608722	110708722	0.388365	KCNAB1
1	110608735	110708735	0.388365	SSR3
1	110608735	110708735	0.388365	KCNAB1
1	110609051	110709051	0.39038	SSR3
1	110609051	110709051	0.39038	KCNAB1
1	110609207	110709207	0.392069	SSR3
1	110609207	110709207	0.392069	KCNAB1
1	110609549	110709549	0.405419	SSR3
1	110609549	110709549	0.405419	KCNAB1
1	110609585	110709585	0.408956	SSR3
1	110609585	110709585	0.408956	KCNAB1
1	110609664	110709664	0.409405	SSR3
1	110609664	110709664	0.409405	KCNAB1
1	110609898	110709898	0.419083	SSR3
1	110609898	110709898	0.419083	KCNAB1

1	110609923	110709923	0.409866	SSR3
1	110609923	110709923	0.409866	KCNAB1
1	110609934	110709934	0.409866	SSR3
1	110609934	110709934	0.409866	KCNAB1
1	110609999	110709999	0.399689	SSR3
1	110609999	110709999	0.399689	KCNAB1
1	110610187	110710187	0.389215	SSR3
1	110610187	110710187	0.389215	KCNAB1
1	110610445	110710445	0.372729	SSR3
1	110610445	110710445	0.372729	KCNAB1
1	110610961	110710961	0.374815	SSR3
1	110610961	110710961	0.374815	KCNAB1
1	110611262	110711262	0.377028	SSR3
1	110611262	110711262	0.377028	KCNAB1
1	110611289	110711289	0.371179	SSR3
1	110611289	110711289	0.371179	KCNAB1
1	110611849	110711849	0.371814	SSR3
1	110611849	110711849	0.371814	KCNAB1
1	110612320	110712320	0.371814	SSR3
1	110612320	110712320	0.371814	KCNAB1
1	110612607	110712607	0.369611	SSR3
1	110612607	110712607	0.369611	KCNAB1
1	110612705	110712705	0.369611	SSR3
1	110612705	110712705	0.369611	KCNAB1
1	110612916	110712916	0.422505	SSR3
1	110612916	110712916	0.422505	KCNAB1
1	111200547	111300547	0.343215	LOC102177841
1	111200547	111300547	0.343215	GMPS
1	111200830	111300830	0.351896	LOC102177841
1	111200830	111300830	0.351896	GMPS
1	111200914	111300914	0.351586	LOC102177841
1	111200914	111300914	0.351586	GMPS
1	111201075	111301075	0.361568	LOC102177841
1	111201075	111301075	0.361568	GMPS
1	111201352	111301352	0.352462	LOC102177841
1	111201352	111301352	0.352462	GMPS
1	111201600	111301600	0.342322	LOC102177841
1	111201600	111301600	0.342322	GMPS
1	111202180	111302180	0.346748	LOC102177841
1	111202180	111302180	0.346748	GMPS
1	111213091	111313091	0.404664	LOC102177841
1	111213091	111313091	0.404664	GMPS
1	111213148	111313148	0.410114	LOC102177841
1	111213148	111313148	0.410114	GMPS
1	111213337	111313337	0.397042	LOC102177841
1	111213337	111313337	0.397042	GMPS
1	111213538	111313538	0.408159	LOC102177841
1	111213538	111313538	0.408159	GMPS
1	111213900	111313900	0.403782	LOC102177841
1	111213900	111313900	0.403782	GMPS
1	111214025	111314025	0.395979	LOC102177841
1	111214025	111314025	0.395979	GMPS
1	111214271	111314271	0.381537	LOC102177841
1	111214271	111314271	0.381537	GMPS
1	111215046	111315046	0.391711	LOC102177841
1	111215046	111315046	0.391711	GMPS
1	111215187	111315187	0.376092	LOC102177841
1	111215187	111315187	0.376092	GMPS

1	111215990	111315990	0.370521	LOC102177841
1	111215990	111315990	0.370521	GMPS
1	111216060	111316060	0.370068	LOC102177841
1	111216060	111316060	0.370068	GMPS
1	111216332	111316332	0.414229	LOC102177841
1	111216332	111316332	0.414229	GMPS
1	111216507	111316507	0.407199	LOC102177841
1	111216507	111316507	0.407199	GMPS
1	111216508	111316508	0.407199	LOC102177841
1	111216508	111316508	0.407199	GMPS
1	111216530	111316530	0.432684	LOC102177841
1	111216530	111316530	0.432684	GMPS
1	111216582	111316582	0.432568	LOC102177841
1	111216582	111316582	0.432568	GMPS
1	111216810	111316810	0.429325	LOC102177841
1	111216810	111316810	0.429325	GMPS
1	111216822	111316822	0.424354	LOC102177841
1	111216822	111316822	0.424354	GMPS
1	111216936	111316936	0.429408	LOC102177841
1	111216936	111316936	0.429408	GMPS
1	111217020	111317020	0.419903	LOC102177841
1	111217020	111317020	0.419903	GMPS
1	111217033	111317033	0.420948	LOC102177841
1	111217033	111317033	0.420948	GMPS
1	111217206	111317206	0.420948	LOC102177841
1	111217206	111317206	0.420948	GMPS
1	111217434	111317434	0.409211	LOC102177841
1	111217434	111317434	0.409211	GMPS
1	111217672	111317672	0.416224	LOC102177841
1	111217672	111317672	0.416224	GMPS
1	111217712	111317712	0.420606	LOC102177841
1	111217712	111317712	0.420606	GMPS
1	111217780	111317780	0.418794	LOC102177841
1	111217780	111317780	0.418794	GMPS
1	111217801	111317801	0.422039	LOC102177841
1	111217801	111317801	0.422039	GMPS
1	111217857	111317857	0.419625	LOC102177841
1	111217857	111317857	0.419625	GMPS
1	111218038	111318038	0.415809	LOC102177841
1	111218038	111318038	0.415809	GMPS
1	111218068	111318068	0.415809	LOC102177841
1	111218068	111318068	0.415809	GMPS
1	111218114	111318114	0.415872	LOC102177841
1	111218114	111318114	0.415872	GMPS
1	111218321	111318321	0.416303	LOC102177841
1	111218321	111318321	0.416303	GMPS
1	111218405	111318405	0.415041	LOC102177841
1	111218405	111318405	0.415041	GMPS
1	111218480	111318480	0.35984	LOC102177841
1	111218480	111318480	0.35984	GMPS
1	111218578	111318578	0.357398	LOC102177841
1	111218578	111318578	0.357398	GMPS
1	111218689	111318689	0.359778	LOC102177841
1	111218689	111318689	0.359778	GMPS
1	111218748	111318748	0.349057	LOC102177841
1	111218748	111318748	0.349057	GMPS
1	111219733	111319733	0.345176	LOC102177841
1	111219733	111319733	0.345176	GMPS

1	111219771	111319771	0.345176	LOC102177841
1	111219771	111319771	0.345176	GMPS
1	111219775	111319775	0.345176	LOC102177841
1	111219775	111319775	0.345176	GMPS
1	111219789	111319789	0.345176	LOC102177841
1	111219789	111319789	0.345176	GMPS
1	111219794	111319794	0.345176	LOC102177841
1	111219794	111319794	0.345176	GMPS
1	111220338	111320338	0.3496	LOC102177841
1	111220338	111320338	0.3496	GMPS
1	111220380	111320380	0.345645	LOC102177841
1	111220380	111320380	0.345645	GMPS
1	111220414	111320414	0.346314	LOC102177841
1	111220414	111320414	0.346314	GMPS
1	111221336	111321336	0.351815	GMPS
1	111221469	111321469	0.347624	GMPS
1	111221525	111321525	0.357799	GMPS
1	111221969	111321969	0.359523	GMPS
1	111222004	111322004	0.356207	GMPS
1	111222005	111322005	0.365746	GMPS
1	111222213	111322213	0.360366	GMPS
1	111222252	111322252	0.358646	GMPS
1	111222525	111322525	0.365699	GMPS
1	111222602	111322602	0.362201	GMPS
1	111222948	111322948	0.363295	GMPS
1	111222952	111322952	0.344818	GMPS
1	111223962	111323962	0.346967	GMPS
1	111225526	111325526	0.350092	GMPS
1	111225939	111325939	0.35652	GMPS
1	111226431	111326431	0.349225	GMPS
1	111230096	111330096	0.349295	GMPS
1	111230171	111330171	0.357817	GMPS
1	111230313	111330313	0.461151	GMPS
1	111230390	111330390	0.429558	GMPS
1	111231026	111331026	0.428886	GMPS
1	111231202	111331202	0.43613	GMPS
1	111231254	111331254	0.427037	GMPS
1	111231329	111331329	0.426029	GMPS
1	111231581	111331581	0.417752	GMPS
1	111231842	111331842	0.427005	GMPS
1	111231949	111331949	0.398348	GMPS
1	111232848	111332848	0.401703	GMPS
1	111233155	111333155	0.383631	GMPS
1	111233520	111333520	0.399309	GMPS
1	111233529	111333529	0.403796	GMPS
1	111233727	111333727	0.42682	GMPS
1	111233732	111333732	0.42682	GMPS
1	111233799	111333799	0.42682	GMPS
1	111233851	111333851	0.415483	GMPS
1	111233918	111333918	0.433363	GMPS
1	111234083	111334083	0.410946	GMPS
1	111234228	111334228	0.410946	GMPS
1	111234278	111334278	0.376496	GMPS
1	111234486	111334486	0.376528	GMPS
1	111234905	111334905	0.358453	GMPS
1	111235118	111335118	0.367141	GMPS
1	111235560	111335560	0.365927	GMPS
1	111235580	111335580	0.386683	GMPS

1	111236064	111336064	0.386683		GMPS
1	111236214	111336214	0.403655		GMPS
1	111236365	111336365	0.352479		GMPS
1	111236437	111336437	0.34744		GMPS
1	111237240	111337240	0.346109		GMPS
1	111237492	111337492	0.346109		GMPS
1	111238243	111338243	0.352671		GMPS
1	111238256	111338256	0.388682		GMPS
1	111238345	111338345	0.373749		GMPS
1	111238471	111338471	0.3697		GMPS
1	111238652	111338652	0.369036		GMPS
1	111238811	111338811	0.380145		GMPS
1	111238929	111338929	0.378442		GMPS
1	111239493	111339493	0.370161		GMPS
1	111239620	111339620	0.361365		GMPS
1	111239941	111339941	0.362848		GMPS
1	111240209	111340209	0.370127		GMPS
1	111240469	111340469	0.378485		GMPS
1	111240529	111340529	0.389901		GMPS
1	111240585	111340585	0.418551		GMPS
1	111240646	111340646	0.41038		GMPS
1	111240770	111340770	0.411391		GMPS
1	111240993	111340993	0.40939		GMPS
1	111241131	111341131	0.394882		GMPS
1	111241158	111341158	0.387683		GMPS
1	111241422	111341422	0.376411		GMPS
1	111241945	111341945	0.374367		GMPS
1	111242422	111342422	0.363135		GMPS
1	111242509	111342509	0.382734		GMPS
1	111242717	111342717	0.3917		GMPS
1	111242871	111342871	0.383999		GMPS
1	111243082	111343082	0.393783		GMPS
1	111243132	111343132	0.393471		GMPS
1	111243133	111343133	0.378129		GMPS
1	111243847	111343847	0.372497		GMPS
1	111243885	111343885	0.378733		GMPS
1	111244100	111344100	0.3916		GMPS
1	111244148	111344148	0.364027		GMPS
1	111245062	111345062	0.394177		GMPS
1	111245390	111345390	0.4018		GMPS
1	111245410	111345410	0.379817		GMPS
1	111245577	111345577	0.382943		GMPS
1	111246644	111346644	0.379039		GMPS
1	111246775	111346775	0.409833		GMPS
1	111247025	111347025	0.406267		GMPS
1	111247286	111347286	0.393384		GMPS
1	111247373	111347373	0.390822		GMPS
1	111247597	111347597	0.396069		GMPS
1	111247706	111347706	0.412503		GMPS
1	111247791	111347791	0.395169		GMPS
1	111248258	111348258	0.396267		GMPS
1	111248356	111348356	0.394651		GMPS
1	111248575	111348575	0.394651		GMPS
1	111248931	111348931	0.408917		GMPS
1	111249023	111349023	0.408463		GMPS
1	111249347	111349347	0.40652		GMPS
1	111249564	111349564	0.405925		GMPS
1	111249962	111349962	0.412581		GMPS

1	111250027	111350027	0.406583	GMPS
1	111250387	111350387	0.388681	GMPS
1	111250807	111350807	0.394233	GMPS
1	111251005	111351005	0.399307	GMPS
1	111251162	111351162	0.39007	GMPS
1	111251283	111351283	0.384988	GMPS
1	111251573	111351573	0.36937	GMPS
1	111252335	111352335	0.38043	GMPS
1	111253012	111353012	0.375979	GMPS
1	111253340	111353340	0.379945	GMPS
1	111253385	111353385	0.393351	GMPS
1	111253394	111353394	0.393351	GMPS
1	111253566	111353566	0.392129	GMPS
1	111253588	111353588	0.399623	GMPS
1	111253613	111353613	0.427482	GMPS
1	111253616	111353616	0.426026	GMPS
1	111253663	111353663	0.427571	GMPS
1	111253668	111353668	0.427571	GMPS
1	111253726	111353726	0.426893	GMPS
1	111253952	111353952	0.42566	GMPS
1	111254015	111354015	0.431387	GMPS
1	111254132	111354132	0.408923	GMPS
1	111254351	111354351	0.410075	GMPS
1	111255110	111355110	0.407201	GMPS
1	111255288	111355288	0.401939	GMPS
1	111255556	111355556	0.362244	GMPS
1	111255796	111355796	0.407675	GMPS
1	111256639	111356639	0.435748	GMPS
1	111256669	111356669	0.442191	GMPS
1	111256731	111356731	0.438034	GMPS
1	111257158	111357158	0.429909	GMPS
1	111257335	111357335	0.429034	GMPS
1	111257444	111357444	0.435906	GMPS
1	111257499	111357499	0.454149	GMPS
1	111257608	111357608	0.433305	GMPS
1	111257629	111357629	0.418787	GMPS
1	111258127	111358127	0.440213	GMPS
1	111259306	111359306	0.446886	GMPS
1	111260401	111360401	0.458851	GMPS
1	111260565	111360565	0.448759	GMPS
1	111260815	111360815	0.444919	GMPS
1	111260903	111360903	0.450441	GMPS
1	111261603	111361603	0.454317	GMPS
1	111261927	111361927	0.470168	GMPS
1	111262258	111362258	0.459017	GMPS
1	111262322	111362322	0.472382	GMPS
1	111262338	111362338	0.466902	GMPS
1	111262568	111362568	0.481161	GMPS
1	111262945	111362945	0.484783	GMPS
1	111263078	111363078	0.484783	GMPS
1	111263126	111363126	0.479112	GMPS
1	111263374	111363374	0.479215	GMPS
1	111263380	111363380	0.479153	GMPS
1	111263637	111363637	0.467869	GMPS
1	111263688	111363688	0.4484	GMPS
1	111263742	111363742	0.424564	GMPS
1	111264785	111364785	0.424564	GMPS
1	111265049	111365049	0.443439	GMPS

1	111265679	111365679	0.443373	GMPS
1	111265945	111365945	0.443373	GMPS
1	111266118	111366118	0.472065	GMPS
1	111266239	111366239	0.466789	GMPS
1	111266770	111366770	0.456161	GMPS
1	111266822	111366822	0.429064	GMPS
1	111266889	111366889	0.424829	GMPS
1	111267079	111367079	0.424829	GMPS
1	111267939	111367939	0.429423	GMPS
1	111268415	111368415	0.449686	GMPS
1	111268630	111368630	0.461054	GMPS
1	111268652	111368652	0.461054	GMPS
1	111269765	111369765	0.486215	GMPS
1	111269899	111369899	0.461726	GMPS
1	111270052	111370052	0.461208	GMPS
1	111270237	111370237	0.473488	GMPS
1	111270631	111370631	0.468289	GMPS
1	111270771	111370771	0.489798	GMPS
1	111271370	111371370	0.470498	GMPS
1	111271759	111371759	0.505425	GMPS
1	111271790	111371790	0.512561	GMPS
1	111272148	111372148	0.517985	GMPS
1	111272547	111372547	0.517985	GMPS
1	111272694	111372694	0.535891	GMPS
1	111272735	111372735	0.535891	GMPS
1	111272933	111372933	0.491357	GMPS
1	111272934	111372934	0.497173	GMPS
1	111273266	111373266	0.497173	GMPS
1	111273325	111373325	0.464427	GMPS
1	111273353	111373353	0.362967	GMPS
1	111273453	111373453	0.351767	GMPS
1	111273660	111373660	0.35723	GMPS
1	111287938	111387938	0.395208	GMPS
1	111287938	111387938	0.395208	SLC33A1
1	111288028	111388028	0.378927	GMPS
1	111288028	111388028	0.378927	SLC33A1
1	111288134	111388134	0.380485	GMPS
1	111288134	111388134	0.380485	SLC33A1
1	111288178	111388178	0.382291	GMPS
1	111288178	111388178	0.382291	SLC33A1
1	111288191	111388191	0.377972	GMPS
1	111288191	111388191	0.377972	SLC33A1
1	111288482	111388482	0.347562	GMPS
1	111288482	111388482	0.347562	SLC33A1
1	111289506	111389506	0.352252	GMPS
1	111289506	111389506	0.352252	SLC33A1
1	111289554	111389554	0.352252	GMPS
1	111289554	111389554	0.352252	SLC33A1
1	111289606	111389606	0.359499	GMPS
1	111289606	111389606	0.359499	SLC33A1
1	111289651	111389651	0.351069	GMPS
1	111289651	111389651	0.351069	SLC33A1
1	111290504	111390504	0.377925	GMPS
1	111290504	111390504	0.377925	SLC33A1
1	111290720	111390720	0.388249	GMPS
1	111290720	111390720	0.388249	SLC33A1
1	111290767	111390767	0.363814	GMPS
1	111290767	111390767	0.363814	SLC33A1

1	111291043	111391043	0.356479	GMPS
1	111291043	111391043	0.356479	SLC33A1
1	111291096	111391096	0.365845	GMPS
1	111291096	111391096	0.365845	SLC33A1
1	111291798	111391798	0.354231	GMPS
1	111291798	111391798	0.354231	SLC33A1
1	111292483	111392483	0.353995	GMPS
1	111292483	111392483	0.353995	SLC33A1
1	111292561	111392561	0.352926	GMPS
1	111292561	111392561	0.352926	SLC33A1
1	111292651	111392651	0.343568	GMPS
1	111292651	111392651	0.343568	SLC33A1
1	111293132	111393132	0.343568	GMPS
1	111293132	111393132	0.343568	SLC33A1
1	111296758	111396758	0.342479	GMPS
1	111296758	111396758	0.342479	SLC33A1
1	111296918	111396918	0.382135	GMPS
1	111296918	111396918	0.382135	SLC33A1
1	111296981	111396981	0.378941	GMPS
1	111296981	111396981	0.378941	SLC33A1
1	111297195	111397195	0.356797	GMPS
1	111297195	111397195	0.356797	SLC33A1
1	111298056	111398056	0.359251	GMPS
1	111298056	111398056	0.359251	SLC33A1
1	111298377	111398377	0.351126	GMPS
1	111298377	111398377	0.351126	SLC33A1
1	111298405	111398405	0.3666	GMPS
1	111298405	111398405	0.3666	SLC33A1
1	111298522	111398522	0.378072	GMPS
1	111298522	111398522	0.378072	SLC33A1
1	111298588	111398588	0.39315	GMPS
1	111298588	111398588	0.39315	SLC33A1
1	111298634	111398634	0.402337	GMPS
1	111298634	111398634	0.402337	SLC33A1
1	111298808	111398808	0.419985	GMPS
1	111298808	111398808	0.419985	SLC33A1
1	111299043	111399043	0.404477	GMPS
1	111299043	111399043	0.404477	SLC33A1
1	111299184	111399184	0.401467	GMPS
1	111299184	111399184	0.401467	SLC33A1
1	111299465	111399465	0.382482	GMPS
1	111299465	111399465	0.382482	SLC33A1
1	111299530	111399530	0.36766	GMPS
1	111299530	111399530	0.36766	SLC33A1
1	111299650	111399650	0.389179	GMPS
1	111299650	111399650	0.389179	SLC33A1
1	111299909	111399909	0.351119	GMPS
1	111299909	111399909	0.351119	SLC33A1
1	111300037	111400037	0.355524	GMPS
1	111300037	111400037	0.355524	SLC33A1
1	111300242	111400242	0.355524	GMPS
1	111300242	111400242	0.355524	SLC33A1
1	111300374	111400374	0.360153	GMPS
1	111300374	111400374	0.360153	SLC33A1
1	111301488	111401488	0.342017	GMPS
1	111301488	111401488	0.342017	SLC33A1
1	111305025	111405025	0.34937	GMPS
1	111305025	111405025	0.34937	SLC33A1

1	111305065	111405065	0.358115	GMPS
1	111305065	111405065	0.358115	SLC33A1
1	111306473	111406473	0.382404	GMPS
1	111306473	111406473	0.382404	SLC33A1
1	111306474	111406474	0.361181	GMPS
1	111306474	111406474	0.361181	SLC33A1
1	111307199	111407199	0.400327	GMPS
1	111307199	111407199	0.400327	SLC33A1
1	111307252	111407252	0.395587	GMPS
1	111307252	111407252	0.395587	SLC33A1
1	111307385	111407385	0.395619	GMPS
1	111307385	111407385	0.395619	SLC33A1
1	111307393	111407393	0.397639	GMPS
1	111307393	111407393	0.397639	SLC33A1
1	111307444	111407444	0.394191	GMPS
1	111307444	111407444	0.394191	SLC33A1
1	111307722	111407722	0.384563	GMPS
1	111307722	111407722	0.384563	SLC33A1
1	111307726	111407726	0.418128	GMPS
1	111307726	111407726	0.418128	SLC33A1
1	111307926	111407926	0.37927	GMPS
1	111307926	111407926	0.37927	SLC33A1
1	111308284	111408284	0.383947	GMPS
1	111308284	111408284	0.383947	SLC33A1
1	111308629	111408629	0.385777	GMPS
1	111308629	111408629	0.385777	SLC33A1
1	111308753	111408753	0.401635	GMPS
1	111308753	111408753	0.401635	SLC33A1
1	111308812	111408812	0.379322	GMPS
1	111308812	111408812	0.379322	SLC33A1
1	111308813	111408813	0.374776	GMPS
1	111308813	111408813	0.374776	SLC33A1
1	111308825	111408825	0.354845	GMPS
1	111308825	111408825	0.354845	SLC33A1
1	111308856	111408856	0.357994	GMPS
1	111308856	111408856	0.357994	SLC33A1
1	111308930	111408930	0.364239	GMPS
1	111308930	111408930	0.364239	SLC33A1
1	111310891	111410891	0.35288	GMPS
1	111310891	111410891	0.35288	SLC33A1
1	111310916	111410916	0.376106	GMPS
1	111310916	111410916	0.376106	SLC33A1
1	111314906	111414906	0.350831	GMPS
1	111314906	111414906	0.350831	SLC33A1
1	111325567	111425567	0.342929	GMPS
1	111325567	111425567	0.342929	SLC33A1
1	111325796	111425796	0.341814	GMPS
1	111325796	111425796	0.341814	SLC33A1
1	111325866	111425866	0.358672	GMPS
1	111325866	111425866	0.358672	SLC33A1
1	111326041	111426041	0.357529	GMPS
1	111326041	111426041	0.357529	SLC33A1
1	111326064	111426064	0.343596	GMPS
1	111326064	111426064	0.343596	SLC33A1
1	111326323	111426323	0.342801	GMPS
1	111326323	111426323	0.342801	SLC33A1
1	111326776	111426776	0.346177	GMPS
1	111326776	111426776	0.346177	SLC33A1

1	111331451	111431451	0.364095	GMPS
1	111331451	111431451	0.364095	SLC33A1
1	111331482	111431482	0.358802	GMPS
1	111331482	111431482	0.358802	SLC33A1
1	111331484	111431484	0.358802	GMPS
1	111331484	111431484	0.358802	SLC33A1
1	111331744	111431744	0.350256	GMPS
1	111331744	111431744	0.350256	SLC33A1
1	111331821	111431821	0.347249	GMPS
1	111331821	111431821	0.347249	SLC33A1
1	111331845	111431845	0.379644	GMPS
1	111331845	111431845	0.379644	SLC33A1
1	111331982	111431982	0.35057	GMPS
1	111331982	111431982	0.35057	SLC33A1
1	111332141	111432141	0.36555	GMPS
1	111332141	111432141	0.36555	SLC33A1
1	111332183	111432183	0.368577	GMPS
1	111332183	111432183	0.368577	SLC33A1
1	111332439	111432439	0.362762	GMPS
1	111332439	111432439	0.362762	SLC33A1
1	111332986	111432986	0.360391	GMPS
1	111332986	111432986	0.360391	SLC33A1
1	111333003	111433003	0.357666	GMPS
1	111333003	111433003	0.357666	SLC33A1
1	111333033	111433033	0.363552	GMPS
1	111333033	111433033	0.363552	SLC33A1
1	111333218	111433218	0.353209	GMPS
1	111333218	111433218	0.353209	SLC33A1
1	111333347	111433347	0.354851	GMPS
1	111333347	111433347	0.354851	SLC33A1
1	111950573	112050573	0.348517	LOC102184663
1	111952580	112052580	0.356138	LOC102184663
1	112204105	112304105	0.350464	MME
1	112204128	112304128	0.34927	MME
1	112204144	112304144	0.349814	MME
1	112204275	112304275	0.346658	MME
1	112204365	112304365	0.346658	MME
1	112204379	112304379	0.344781	MME
1	112204651	112304651	0.343912	MME
1	112204724	112304724	0.343912	MME
1	112204730	112304730	0.34875	MME
1	112204735	112304735	0.34875	MME
1	112204751	112304751	0.34875	MME
1	112205814	112305814	0.345951	MME
1	112205889	112305889	0.359851	MME
1	112206053	112306053	0.353626	MME
1	112206120	112306120	0.366281	MME
1	112206130	112306130	0.366281	MME
1	112206131	112306131	0.366281	MME
1	112206208	112306208	0.34618	MME
1	112206242	112306242	0.345373	MME
1	112206947	112306947	0.345917	MME
1	112206963	112306963	0.342821	MME
1	112207935	112307935	0.34466	MME
1	112208217	112308217	0.365634	MME
1	112208267	112308267	0.380781	MME
1	112208297	112308297	0.366863	MME
1	112208403	112308403	0.347862	MME

1	112208941	112308941	0.348999	MME
1	112208969	112308969	0.38814	MME
1	112209006	112309006	0.370745	MME
1	112209021	112309021	0.370629	MME
1	112209100	112309100	0.361902	MME
1	112209131	112309131	0.343535	MME
1	112209846	112309846	0.367348	MME
1	112209856	112309856	0.388133	MME
1	112209862	112309862	0.361877	MME
1	112209890	112309890	0.361877	MME
1	112209891	112309891	0.361877	MME
1	112209923	112309923	0.346621	MME
1	112209976	112309976	0.347581	MME
1	112210005	112310005	0.343406	MME
1	112210065	112310065	0.343406	MME
1	112852816	112952816	0.374287	TRNAC-GCA-10
1	112852816	112952816	0.374287	TRNAE-UUC-2
1	112852816	112952816	0.374287	GPR149
1	112878744	112978744	0.365797	TRNAC-GCA-10
1	112878744	112978744	0.365797	TRNAE-UUC-2
1	112878744	112978744	0.365797	GPR149
1	112878744	112978744	0.365797	DHX36
1	113097495	113197495	0.34274	ARHGEF26
1	113097808	113197808	0.350486	ARHGEF26
1	113103033	113203033	0.380755	ARHGEF26
1	113103335	113203335	0.428349	ARHGEF26
1	113103353	113203353	0.366658	ARHGEF26
1	113109839	113209839	0.367024	ARHGEF26
1	113110223	113210223	0.355736	ARHGEF26
1	113110296	113210296	0.358162	ARHGEF26
1	113113381	113213381	0.367707	ARHGEF26
1	113113421	113213421	0.355615	ARHGEF26
1	113113431	113213431	0.355615	ARHGEF26
1	113113587	113213587	0.371279	ARHGEF26
1	113113816	113213816	0.370838	ARHGEF26
1	113114117	113214117	0.370838	ARHGEF26
1	113114176	113214176	0.370838	ARHGEF26
1	113114238	113214238	0.388849	ARHGEF26
1	113114334	113214334	0.350437	ARHGEF26
1	113114717	113214717	0.350437	ARHGEF26
1	113114934	113214934	0.372725	ARHGEF26
1	113115111	113215111	0.372725	ARHGEF26
1	113115114	113215114	0.374284	ARHGEF26
1	113115132	113215132	0.374284	ARHGEF26
1	113115155	113215155	0.406063	ARHGEF26
1	113115177	113215177	0.380072	ARHGEF26
1	113115486	113215486	0.372793	ARHGEF26
1	113115529	113215529	0.369293	ARHGEF26
1	113115608	113215608	0.375657	ARHGEF26
1	113115663	113215663	0.366139	ARHGEF26
1	113115665	113215665	0.379508	ARHGEF26
1	113115875	113215875	0.379508	ARHGEF26
1	113116057	113216057	0.359888	ARHGEF26
1	113116272	113216272	0.359888	ARHGEF26
1	113116431	113216431	0.359888	ARHGEF26
1	113116461	113216461	0.359888	ARHGEF26
1	113116499	113216499	0.359888	ARHGEF26
1	113116511	113216511	0.359888	ARHGEF26

1	113116641	113216641	0.359888	ARHGEF26
1	113116681	113216681	0.359888	ARHGEF26
1	113116836	113216836	0.354461	ARHGEF26
1	113116902	113216902	0.376291	ARHGEF26
1	113116975	113216975	0.401988	ARHGEF26
1	113117908	113217908	0.359253	ARHGEF26
1	113118218	113218218	0.366254	ARHGEF26
1	113118445	113218445	0.359955	ARHGEF26
1	113118460	113218460	0.342336	ARHGEF26
1	113118523	113218523	0.342336	ARHGEF26
1	113118669	113218669	0.342336	ARHGEF26
1	113118719	113218719	0.383238	ARHGEF26
1	113118733	113218733	0.393705	ARHGEF26
1	113118739	113218739	0.385849	ARHGEF26
1	113118836	113218836	0.401385	ARHGEF26
1	113118928	113218928	0.406653	ARHGEF26
1	113118998	113218998	0.406653	ARHGEF26
1	113119102	113219102	0.406653	ARHGEF26
1	113119294	113219294	0.408804	ARHGEF26
1	113119421	113219421	0.359477	ARHGEF26
1	113119445	113219445	0.379821	ARHGEF26
1	113119581	113219581	0.367789	ARHGEF26
1	113119813	113219813	0.367789	ARHGEF26
1	113119816	113219816	0.389918	ARHGEF26
1	113120066	113220066	0.362188	ARHGEF26
1	113120084	113220084	0.362188	ARHGEF26
1	113120342	113220342	0.362188	ARHGEF26
1	113120680	113220680	0.362611	ARHGEF26
1	113120921	113220921	0.363244	ARHGEF26
1	113120999	113220999	0.363244	ARHGEF26
1	113121030	113221030	0.391857	ARHGEF26
1	113121039	113221039	0.414036	ARHGEF26
1	113121236	113221236	0.41309	ARHGEF26
1	113121263	113221263	0.417424	ARHGEF26
1	113121287	113221287	0.404197	ARHGEF26
1	113121304	113221304	0.397116	ARHGEF26
1	113121338	113221338	0.40084	ARHGEF26
1	113121429	113221429	0.400919	ARHGEF26
1	113121437	113221437	0.362532	ARHGEF26
1	113121544	113221544	0.3502	ARHGEF26
1	113121602	113221602	0.361327	ARHGEF26
1	113121647	113221647	0.393186	ARHGEF26
1	113121678	113221678	0.385153	ARHGEF26
1	113121959	113221959	0.385153	ARHGEF26
1	113122180	113222180	0.385153	ARHGEF26
1	113122313	113222313	0.384845	ARHGEF26
1	113122469	113222469	0.394241	ARHGEF26
1	113122850	113222850	0.3946	ARHGEF26
1	113122914	113222914	0.3946	ARHGEF26
1	113122969	113222969	0.3946	ARHGEF26
1	113123214	113223214	0.394766	ARHGEF26
1	113123338	113223338	0.392614	ARHGEF26
1	113123443	113223443	0.42193	ARHGEF26
1	113123488	113223488	0.346659	ARHGEF26
1	113124113	113224113	0.358512	ARHGEF26
1	113124128	113224128	0.358512	ARHGEF26
1	113124176	113224176	0.354941	ARHGEF26
1	113124240	113224240	0.391447	ARHGEF26

1	113124650	113224650	0.380212	ARHGEF26
1	113124857	113224857	0.438092	ARHGEF26
1	113124899	113224899	0.444163	ARHGEF26
1	113124924	113224924	0.444163	ARHGEF26
1	113125086	113225086	0.434027	ARHGEF26
1	113125398	113225398	0.41904	ARHGEF26
1	113125666	113225666	0.41904	ARHGEF26
1	113125818	113225818	0.430347	ARHGEF26
1	113125830	113225830	0.427379	ARHGEF26
1	113125860	113225860	0.461661	ARHGEF26
1	113125882	113225882	0.39769	ARHGEF26
1	113125894	113225894	0.43321	ARHGEF26
1	113126073	113226073	0.393658	ARHGEF26
1	113126507	113226507	0.393658	ARHGEF26
1	113126591	113226591	0.393658	ARHGEF26
1	113126592	113226592	0.393658	ARHGEF26
1	113126758	113226758	0.391572	ARHGEF26
1	113126925	113226925	0.389058	ARHGEF26
1	113127038	113227038	0.390574	ARHGEF26
1	113127215	113227215	0.390574	ARHGEF26
1	113127262	113227262	0.395815	ARHGEF26
1	113127320	113227320	0.390381	ARHGEF26
1	113127504	113227504	0.390381	ARHGEF26
1	113127620	113227620	0.431148	ARHGEF26
1	113127711	113227711	0.431148	ARHGEF26
1	113127746	113227746	0.434945	ARHGEF26
1	113127784	113227784	0.434414	ARHGEF26
1	113127803	113227803	0.434414	ARHGEF26
1	113127932	113227932	0.44926	ARHGEF26
1	113128099	113228099	0.447119	ARHGEF26
1	113128118	113228118	0.447119	ARHGEF26
1	113128142	113228142	0.448376	ARHGEF26
1	113128174	113228174	0.434057	ARHGEF26
1	113128176	113228176	0.434057	ARHGEF26
1	113128229	113228229	0.434057	ARHGEF26
1	113128358	113228358	0.459129	ARHGEF26
1	113128375	113228375	0.448592	ARHGEF26
1	113128399	113228399	0.434112	ARHGEF26
1	113128444	113228444	0.433633	ARHGEF26
1	113128630	113228630	0.45504	ARHGEF26
1	113128643	113228643	0.442634	ARHGEF26
1	113128688	113228688	0.436415	ARHGEF26
1	113128708	113228708	0.436778	ARHGEF26
1	113128769	113228769	0.437265	ARHGEF26
1	113128783	113228783	0.441135	ARHGEF26
1	113128942	113228942	0.447958	ARHGEF26
1	113128944	113228944	0.455186	ARHGEF26
1	113129099	113229099	0.35201	ARHGEF26
1	113129100	113229100	0.35201	ARHGEF26
1	113129148	113229148	0.409175	ARHGEF26
1	113129316	113229316	0.398151	ARHGEF26
1	113129442	113229442	0.410012	ARHGEF26
1	113129527	113229527	0.393792	ARHGEF26
1	113129558	113229558	0.393792	ARHGEF26
1	113129684	113229684	0.393792	ARHGEF26
1	113129697	113229697	0.409894	ARHGEF26
1	113129803	113229803	0.380708	ARHGEF26
1	113129896	113229896	0.375911	ARHGEF26

1	113130125	113230125	0.391037	ARHGEF26
1	113130299	113230299	0.390582	ARHGEF26
1	113130364	113230364	0.398781	ARHGEF26
1	113130669	113230669	0.394123	ARHGEF26
1	113130757	113230757	0.400008	ARHGEF26
1	113131088	113231088	0.400008	ARHGEF26
1	113131144	113231144	0.410518	ARHGEF26
1	113133138	113233138	0.360854	ARHGEF26
1	113134518	113234518	0.357748	ARHGEF26
1	113134542	113234542	0.357855	ARHGEF26
1	113134565	113234565	0.418285	ARHGEF26
1	113134607	113234607	0.379588	ARHGEF26
1	113134654	113234654	0.379588	ARHGEF26
1	113134660	113234660	0.379588	ARHGEF26
1	113134700	113234700	0.446746	ARHGEF26
1	113134705	113234705	0.401965	ARHGEF26
1	113134730	113234730	0.401965	ARHGEF26
1	113134734	113234734	0.402071	ARHGEF26
1	113134760	113234760	0.402071	ARHGEF26
1	113134776	113234776	0.395623	ARHGEF26
1	113134782	113234782	0.391899	ARHGEF26
1	113134819	113234819	0.391899	ARHGEF26
1	113134917	113234917	0.391899	ARHGEF26
1	113135063	113235063	0.391899	ARHGEF26
1	113135067	113235067	0.38834	ARHGEF26
1	113135121	113235121	0.379594	ARHGEF26
1	113135122	113235122	0.374093	ARHGEF26
1	113135364	113235364	0.38053	ARHGEF26
1	113135375	113235375	0.38053	ARHGEF26
1	113135418	113235418	0.379409	ARHGEF26
1	113135419	113235419	0.379409	ARHGEF26
1	113135491	113235491	0.372373	ARHGEF26
1	113135567	113235567	0.372373	ARHGEF26
1	113135700	113235700	0.372373	ARHGEF26
1	113135725	113235725	0.372373	ARHGEF26
1	113135944	113235944	0.397924	ARHGEF26
1	113135988	113235988	0.362211	ARHGEF26
1	113136041	113236041	0.365404	ARHGEF26
1	113136086	113236086	0.365404	ARHGEF26
1	113136093	113236093	0.367661	ARHGEF26
1	113136222	113236222	0.362937	ARHGEF26
1	113136310	113236310	0.364981	ARHGEF26
1	113136402	113236402	0.364352	ARHGEF26
1	113136471	113236471	0.364302	ARHGEF26
1	113136472	113236472	0.364302	ARHGEF26
1	113136474	113236474	0.363783	ARHGEF26
1	113136803	113236803	0.347965	ARHGEF26
1	113137060	113237060	0.352781	ARHGEF26
1	113137091	113237091	0.352781	ARHGEF26
1	113137127	113237127	0.352781	ARHGEF26
1	113137293	113237293	0.356139	ARHGEF26
1	113137351	113237351	0.356139	ARHGEF26
1	113137385	113237385	0.350882	ARHGEF26
1	113137463	113237463	0.350849	ARHGEF26
1	113137769	113237769	0.350098	ARHGEF26
1	113137989	113237989	0.356065	ARHGEF26
1	113138022	113238022	0.356065	ARHGEF26
1	113138163	113238163	0.354171	ARHGEF26

1	113138204	113238204	0.354171	ARHGEF26
1	113138334	113238334	0.354171	ARHGEF26
1	113138436	113238436	0.392439	ARHGEF26
1	113138581	113238581	0.373868	ARHGEF26
1	113138806	113238806	0.374973	ARHGEF26
1	113138932	113238932	0.374973	ARHGEF26
1	113138941	113238941	0.374973	ARHGEF26
1	113139071	113239071	0.374973	ARHGEF26
1	113139449	113239449	0.368862	ARHGEF26
1	113139645	113239645	0.382436	ARHGEF26
1	113139691	113239691	0.427811	ARHGEF26
1	113139777	113239777	0.414291	ARHGEF26
1	113139850	113239850	0.459788	ARHGEF26
1	113139856	113239856	0.437171	ARHGEF26
1	113139876	113239876	0.454479	ARHGEF26
1	113140149	113240149	0.454479	ARHGEF26
1	113140261	113240261	0.454666	ARHGEF26
1	113140378	113240378	0.441255	ARHGEF26
1	113140616	113240616	0.429415	ARHGEF26
1	113140645	113240645	0.42293	ARHGEF26
1	113140646	113240646	0.42293	ARHGEF26
1	113140702	113240702	0.423276	ARHGEF26
1	113140703	113240703	0.423288	ARHGEF26
1	113140911	113240911	0.420457	ARHGEF26
1	113140914	113240914	0.420457	ARHGEF26
1	113140918	113240918	0.42639	ARHGEF26
1	114144569	114244569	0.36181	RAP2B
1	114144611	114244611	0.365151	RAP2B
1	114144832	114244832	0.375384	RAP2B
1	114144866	114244866	0.375384	RAP2B
1	114145107	114245107	0.382829	RAP2B
1	114145111	114245111	0.39944	RAP2B
1	114145275	114245275	0.400586	RAP2B
1	114145283	114245283	0.403052	RAP2B
1	114145363	114245363	0.381767	RAP2B
1	114145540	114245540	0.380756	RAP2B
1	114145581	114245581	0.385939	RAP2B
1	114145688	114245688	0.385939	RAP2B
1	114145757	114245757	0.385939	RAP2B
1	114145792	114245792	0.40396	RAP2B
1	114145825	114245825	0.398668	RAP2B
1	114145883	114245883	0.397257	RAP2B
1	114145990	114245990	0.391761	RAP2B
1	114146023	114246023	0.386344	RAP2B
1	114146667	114246667	0.361213	RAP2B
1	114147042	114247042	0.379743	RAP2B
1	114147208	114247208	0.369497	RAP2B
1	114147371	114247371	0.361481	RAP2B
1	114147774	114247774	0.36179	RAP2B
1	114148045	114248045	0.374566	RAP2B
1	114148224	114248224	0.367369	RAP2B
1	114148241	114248241	0.365567	RAP2B
1	114149404	114249404	0.381119	RAP2B
1	114149415	114249415	0.388758	RAP2B
1	114149416	114249416	0.361763	RAP2B
1	114149471	114249471	0.342653	RAP2B
1	114149938	114249938	0.355887	RAP2B
1	114150515	114250515	0.354267	RAP2B

1	114150836	114250836	0.362681	RAP2B
1	114151053	114251053	0.356881	RAP2B
1	114151277	114251277	0.359207	RAP2B
1	114151282	114251282	0.342172	RAP2B
1	114151452	114251452	0.342015	RAP2B
1	114151477	114251477	0.342015	RAP2B
1	114152281	114252281	0.350733	RAP2B
1	114152329	114252329	0.367232	RAP2B
1	114152362	114252362	0.350733	RAP2B
1	114152391	114252391	0.344889	RAP2B
1	114152572	114252572	0.344889	RAP2B
1	114152575	114252575	0.374211	RAP2B
1	114152612	114252612	0.365747	RAP2B
1	114152693	114252693	0.362861	RAP2B
1	114152853	114252853	0.354535	RAP2B
1	114154107	114254107	0.351933	RAP2B
1	114156599	114256599	0.358728	RAP2B
1	114156886	114256886	0.35839	RAP2B
1	114156888	114256888	0.361206	RAP2B
1	114156930	114256930	0.361206	RAP2B
1	114157100	114257100	0.362608	RAP2B
1	114157169	114257169	0.355534	RAP2B
1	114157284	114257284	0.353192	RAP2B
1	114157347	114257347	0.353192	RAP2B
1	114157366	114257366	0.352931	RAP2B
1	114157425	114257425	0.356242	RAP2B
1	114157466	114257466	0.351345	RAP2B
1	114158511	114258511	0.354259	RAP2B
1	114171845	114271845	0.35912	RAP2B
1	114171994	114271994	0.363558	RAP2B
1	114172017	114272017	0.374134	RAP2B
1	114172090	114272090	0.38286	RAP2B
1	114172218	114272218	0.382361	RAP2B
1	114172527	114272527	0.37528	RAP2B
1	114172619	114272619	0.37528	RAP2B
1	114172630	114272630	0.37528	RAP2B
1	114172782	114272782	0.37528	RAP2B
1	114173129	114273129	0.368711	RAP2B
1	114173223	114273223	0.368711	RAP2B
1	114173336	114273336	0.368711	RAP2B
1	114173864	114273864	0.353193	RAP2B
1	114173923	114273923	0.367273	RAP2B
1	114174013	114274013	0.354493	RAP2B
1	114174720	114274720	0.357098	RAP2B
1	114175933	114275933	0.355369	RAP2B
1	114176361	114276361	0.372858	RAP2B
1	114176520	114276520	0.366259	RAP2B
1	114176572	114276572	0.373178	RAP2B
1	114176622	114276622	0.377715	RAP2B
1	114176657	114276657	0.387884	RAP2B
1	114177009	114277009	0.402296	RAP2B
1	114177028	114277028	0.399215	RAP2B
1	114177314	114277314	0.399558	RAP2B
1	114177372	114277372	0.385708	RAP2B
1	114177388	114277388	0.377252	RAP2B
1	114177700	114277700	0.377252	RAP2B
1	114177787	114277787	0.384082	RAP2B
1	114178179	114278179	0.39221	RAP2B

1	114178180	114278180	0.378894	RAP2B
1	114178815	114278815	0.374785	RAP2B
1	114179174	114279174	0.382419	RAP2B
1	114179285	114279285	0.385742	RAP2B
1	114179349	114279349	0.399598	RAP2B
1	114179393	114279393	0.398887	RAP2B
1	114179441	114279441	0.373557	RAP2B
1	114179671	114279671	0.382089	RAP2B
1	114179717	114279717	0.382034	RAP2B
1	114179859	114279859	0.384953	RAP2B
1	114180178	114280178	0.395979	RAP2B
1	114180179	114280179	0.395979	RAP2B
1	114180525	114280525	0.37402	RAP2B
1	114180570	114280570	0.345713	RAP2B
1	114180644	114280644	0.394037	RAP2B
1	114180679	114280679	0.395506	RAP2B
1	114180709	114280709	0.405728	RAP2B
1	114180889	114280889	0.392569	RAP2B
1	114181215	114281215	0.365172	RAP2B
1	114181590	114281590	0.364682	RAP2B
1	114181691	114281691	0.369148	RAP2B
1	114182346	114282346	0.372793	RAP2B
1	114182358	114282358	0.363668	RAP2B
1	114182378	114282378	0.355642	RAP2B
1	114183046	114283046	0.363218	RAP2B
1	114184084	114284084	0.345895	RAP2B
1	114184187	114284187	0.345979	RAP2B
1	114184477	114284477	0.345868	RAP2B
1	114184565	114284565	0.359835	RAP2B
1	114184992	114284992	0.379285	RAP2B
1	114185055	114285055	0.379285	RAP2B
1	114185394	114285394	0.374671	RAP2B
1	114186055	114286055	0.345071	RAP2B
1	114186133	114286133	0.346962	RAP2B
1	114186511	114286511	0.343146	RAP2B
1	114186867	114286867	0.343146	RAP2B
1	114187750	114287750	0.348341	RAP2B
1	114187821	114287821	0.372836	RAP2B
1	114187836	114287836	0.379849	RAP2B
1	114187837	114287837	0.373812	RAP2B
1	114187898	114287898	0.37215	RAP2B
1	114188003	114288003	0.372798	RAP2B
1	114188281	114288281	0.37191	RAP2B
1	114188441	114288441	0.365536	RAP2B
1	114188512	114288512	0.378801	RAP2B
1	114188753	114288753	0.381084	RAP2B
1	114188800	114288800	0.386415	RAP2B
1	114188813	114288813	0.388921	RAP2B
1	114189382	114289382	0.374555	RAP2B
1	114189493	114289493	0.358512	RAP2B
1	114189805	114289805	0.356199	RAP2B
1	114190513	114290513	0.348046	RAP2B
1	114190600	114290600	0.376302	RAP2B
1	114190787	114290787	0.379294	RAP2B
1	114191500	114291500	0.365877	RAP2B
1	114191778	114291778	0.353734	RAP2B
1	114191968	114291968	0.36944	RAP2B
1	114192320	114292320	0.367406	RAP2B

1	114192578	114292578	0.371182	RAP2B
1	114192833	114292833	0.378903	RAP2B
1	114193006	114293006	0.377236	RAP2B
1	114193048	114293048	0.346345	RAP2B
1	114193673	114293673	0.344619	RAP2B
1	114193807	114293807	0.409417	RAP2B
1	114193852	114293852	0.409902	RAP2B
1	114194034	114294034	0.410158	RAP2B
1	114194087	114294087	0.38741	RAP2B
1	114194094	114294094	0.499513	RAP2B
1	114194096	114294096	0.396161	RAP2B
1	114194186	114294186	0.39178	RAP2B
1	116972434	117072434	0.344335	SIAH2
1	116972434	117072434	0.344335	ERICH6
1	117129717	117229717	0.362884	SERP1
1	117129717	117229717	0.362884	EIF2A
1	118048683	118148683	0.353823	WWTR1
1	118048907	118148907	0.351174	WWTR1
1	118049135	118149135	0.349948	WWTR1
1	118050033	118150033	0.346328	WWTR1
1	118050277	118150277	0.343429	WWTR1
1	118050293	118150293	0.348506	WWTR1
1	118050652	118150652	0.348622	WWTR1
1	118050658	118150658	0.360749	WWTR1
1	118050682	118150682	0.359509	WWTR1
1	118050824	118150824	0.359509	WWTR1
1	118050833	118150833	0.359509	WWTR1
1	118050875	118150875	0.371621	WWTR1
1	118051115	118151115	0.3648	WWTR1
1	118052921	118152921	0.349167	WWTR1
1	118052924	118152924	0.368094	WWTR1
1	118053150	118153150	0.356728	WWTR1
1	118053399	118153399	0.344467	WWTR1
1	118053400	118153400	0.344467	WWTR1
1	118054331	118154331	0.3446	WWTR1
1	118055639	118155639	0.343483	WWTR1
1	118056282	118156282	0.342992	WWTR1
1	118056854	118156854	0.34239	WWTR1
1	118056988	118156988	0.341688	WWTR1
1	118057003	118157003	0.343011	WWTR1
1	118057074	118157074	0.354235	WWTR1
1	118057266	118157266	0.351735	WWTR1
1	118057341	118157341	0.356849	WWTR1
1	118057349	118157349	0.346543	WWTR1
1	118058607	118158607	0.346176	WWTR1
1	118058771	118158771	0.346176	WWTR1
1	118058854	118158854	0.347299	WWTR1
1	118058863	118158863	0.347328	WWTR1
1	118058891	118158891	0.347328	WWTR1
1	118059105	118159105	0.347328	WWTR1
1	118059115	118159115	0.347328	WWTR1
1	118059125	118159125	0.346934	WWTR1
1	118059175	118159175	0.346934	WWTR1
1	118059217	118159217	0.346934	WWTR1
1	118059239	118159239	0.347094	WWTR1
1	118059287	118159287	0.345774	WWTR1
1	118059323	118159323	0.343544	WWTR1
1	118059613	118159613	0.341851	WWTR1

1	118059799	118159799	0.342578	WWTR1
1	118060081	118160081	0.35037	WWTR1
1	118060082	118160082	0.352502	WWTR1
1	118060120	118160120	0.35212	WWTR1
1	118060312	118160312	0.356238	WWTR1
1	118060317	118160317	0.361092	WWTR1
1	118060324	118160324	0.361092	WWTR1
1	118060401	118160401	0.352317	WWTR1
1	118060566	118160566	0.350522	WWTR1
1	118060649	118160649	0.358434	WWTR1
1	118060659	118160659	0.346884	WWTR1
1	118060715	118160715	0.354394	WWTR1
1	118060791	118160791	0.358135	WWTR1
1	118060926	118160926	0.355324	WWTR1
1	118061198	118161198	0.343121	WWTR1
1	118061989	118161989	0.357536	WWTR1
1	118062009	118162009	0.358053	WWTR1
1	118062078	118162078	0.356435	WWTR1
1	118062734	118162734	0.353452	WWTR1
1	118062883	118162883	0.343013	WWTR1
1	118063530	118163530	0.344425	WWTR1
1	118063707	118163707	0.348935	WWTR1
1	118063805	118163805	0.347619	WWTR1
1	118063998	118163998	0.347619	WWTR1
1	118064103	118164103	0.351894	WWTR1
1	118064121	118164121	0.34527	WWTR1
1	118064275	118164275	0.345424	WWTR1
1	118064478	118164478	0.341823	WWTR1
1	118064524	118164524	0.351267	WWTR1
1	118064723	118164723	0.341844	WWTR1
1	118065802	118165802	0.345271	WWTR1
1	118066075	118166075	0.353309	WWTR1
1	118066086	118166086	0.352417	WWTR1
1	118066202	118166202	0.358247	WWTR1
1	118066380	118166380	0.347221	WWTR1
1	118066445	118166445	0.345723	WWTR1
1	118066869	118166869	0.348911	WWTR1
1	118067011	118167011	0.34577	WWTR1
1	118067135	118167135	0.34577	WWTR1
1	118067363	118167363	0.34577	WWTR1
1	118067531	118167531	0.353892	WWTR1
1	118067640	118167640	0.353892	WWTR1
1	118067703	118167703	0.356867	WWTR1
1	118067858	118167858	0.363456	WWTR1
1	118067909	118167909	0.383086	WWTR1
1	118067916	118167916	0.381032	WWTR1
1	118068462	118168462	0.37676	WWTR1
1	118068782	118168782	0.377252	WWTR1
1	118068910	118168910	0.398529	WWTR1
1	118068960	118168960	0.413116	WWTR1
1	118069018	118169018	0.404013	WWTR1
1	118069711	118169711	0.401037	WWTR1
1	118069786	118169786	0.391108	WWTR1
1	118069922	118169922	0.397565	WWTR1
1	118070120	118170120	0.389037	WWTR1
1	118070122	118170122	0.389037	WWTR1
1	118070718	118170718	0.391145	WWTR1
1	118070749	118170749	0.400032	WWTR1

1	118071985	118171985	0.384751	WWTR1
1	118072326	118172326	0.382835	WWTR1
1	118072336	118172336	0.369424	WWTR1
1	118072408	118172408	0.376271	WWTR1
1	118072748	118172748	0.371776	WWTR1
1	118072769	118172769	0.37217	WWTR1
1	118072788	118172788	0.36893	WWTR1
1	118072789	118172789	0.36893	WWTR1
1	118073065	118173065	0.36893	WWTR1
1	118073904	118173904	0.365729	WWTR1
1	118073940	118173940	0.3919	WWTR1
1	118073995	118173995	0.37534	WWTR1
1	118074107	118174107	0.386426	WWTR1
1	118074299	118174299	0.393662	WWTR1
1	118074312	118174312	0.382144	WWTR1
1	118074433	118174433	0.346558	WWTR1
1	118074613	118174613	0.368686	WWTR1
1	118074812	118174812	0.346193	WWTR1
1	118077657	118177657	0.363015	WWTR1
1	118077782	118177782	0.358619	WWTR1
1	118077786	118177786	0.35986	WWTR1
1	118077849	118177849	0.356731	WWTR1
1	118077880	118177880	0.342739	WWTR1
1	118077885	118177885	0.349331	WWTR1
1	118081627	118181627	0.35174	WWTR1
1	118082029	118182029	0.34781	WWTR1
1	118082087	118182087	0.34781	WWTR1
1	118083388	118183388	0.34303	WWTR1
1	118092843	118192843	0.362233	WWTR1
1	118092896	118192896	0.370353	WWTR1
1	118275624	118375624	0.389633	TM4SF1
1	118275681	118375681	0.356263	TM4SF1
1	118275744	118375744	0.356263	TM4SF1
1	118275861	118375861	0.389312	TM4SF1
1	118276011	118376011	0.394518	TM4SF1
1	118276060	118376060	0.380814	TM4SF1
1	118276198	118376198	0.362728	TM4SF1
1	118276857	118376857	0.341688	TM4SF1
1	118276926	118376926	0.342705	TM4SF1
1	118294881	118394881	0.371934	TM4SF1
1	118678186	118778186	0.370968	HLTF
1	118678186	118778186	0.370968	GYG1
1	118678238	118778238	0.350769	HLTF
1	118678238	118778238	0.350769	GYG1
1	118678260	118778260	0.350769	HLTF
1	118678260	118778260	0.350769	GYG1
1	118678353	118778353	0.361692	HLTF
1	118678353	118778353	0.361692	GYG1
1	118678412	118778412	0.342466	HLTF
1	118678412	118778412	0.342466	GYG1
1	118680825	118780825	0.342612	HLTF
1	118680825	118780825	0.342612	GYG1
1	118680935	118780935	0.366522	HLTF
1	118680935	118780935	0.366522	GYG1
1	118680943	118780943	0.366522	HLTF
1	118680943	118780943	0.366522	GYG1
1	118680947	118780947	0.366522	HLTF
1	118680947	118780947	0.366522	GYG1

1	118681067	118781067	0.357931	HLTF
1	118681067	118781067	0.357931	GYG1
1	118681152	118781152	0.344952	HLTF
1	118681152	118781152	0.344952	GYG1
1	118681267	118781267	0.349761	HLTF
1	118681267	118781267	0.349761	GYG1
1	118681280	118781280	0.349761	HLTF
1	118681280	118781280	0.349761	GYG1
1	118681295	118781295	0.352501	HLTF
1	118681295	118781295	0.352501	GYG1
1	118681468	118781468	0.347744	HLTF
1	118681468	118781468	0.347744	GYG1
1	118681510	118781510	0.347744	HLTF
1	118681510	118781510	0.347744	GYG1
1	118681514	118781514	0.347744	HLTF
1	118681514	118781514	0.347744	GYG1
1	118681620	118781620	0.351946	HLTF
1	118681620	118781620	0.351946	GYG1
1	118681622	118781622	0.349986	HLTF
1	118681622	118781622	0.349986	GYG1
1	118682003	118782003	0.34378	HLTF
1	118682003	118782003	0.34378	GYG1
1	118682677	118782677	0.34378	HLTF
1	118682677	118782677	0.34378	GYG1
1	118682752	118782752	0.344903	HLTF
1	118682752	118782752	0.344903	GYG1
1	118683616	118783616	0.345664	HLTF
1	118683616	118783616	0.345664	GYG1
1	118683652	118783652	0.34851	HLTF
1	118683652	118783652	0.34851	GYG1
1	118683811	118783811	0.349076	HLTF
1	118683811	118783811	0.349076	GYG1
1	118684004	118784004	0.34669	HLTF
1	118684004	118784004	0.34669	GYG1
1	118684714	118784714	0.357118	HLTF
1	118684714	118784714	0.357118	GYG1
1	118684751	118784751	0.361158	HLTF
1	118684751	118784751	0.361158	GYG1
1	118684788	118784788	0.361913	HLTF
1	118684788	118784788	0.361913	GYG1
1	118684808	118784808	0.361913	HLTF
1	118684808	118784808	0.361913	GYG1
1	118684843	118784843	0.361913	HLTF
1	118684843	118784843	0.361913	GYG1
1	118684895	118784895	0.35369	HLTF
1	118684895	118784895	0.35369	GYG1
1	118684957	118784957	0.352804	HLTF
1	118684957	118784957	0.352804	GYG1
1	118685230	118785230	0.352804	HLTF
1	118685230	118785230	0.352804	GYG1
1	118685272	118785272	0.352804	HLTF
1	118685272	118785272	0.352804	GYG1
1	118685364	118785364	0.352804	HLTF
1	118685364	118785364	0.352804	GYG1
1	118685401	118785401	0.352804	HLTF
1	118685401	118785401	0.352804	GYG1
1	118685462	118785462	0.352804	HLTF
1	118685462	118785462	0.352804	GYG1

1	118685561	118785561	0.352804	HLTF
1	118685561	118785561	0.352804	GYG1
1	118685579	118785579	0.352804	HLTF
1	118685579	118785579	0.352804	GYG1
1	118685605	118785605	0.352804	HLTF
1	118685605	118785605	0.352804	GYG1
1	118685613	118785613	0.352804	HLTF
1	118685613	118785613	0.352804	GYG1
1	118685668	118785668	0.352804	HLTF
1	118685668	118785668	0.352804	GYG1
1	118685793	118785793	0.352804	HLTF
1	118685793	118785793	0.352804	GYG1
1	118685803	118785803	0.352804	HLTF
1	118685803	118785803	0.352804	GYG1
1	118685945	118785945	0.352804	HLTF
1	118685945	118785945	0.352804	GYG1
1	118686070	118786070	0.352804	HLTF
1	118686070	118786070	0.352804	GYG1
1	118686074	118786074	0.35369	HLTF
1	118686074	118786074	0.35369	GYG1
1	118686134	118786134	0.351919	HLTF
1	118686134	118786134	0.351919	GYG1
1	118686149	118786149	0.361865	HLTF
1	118686149	118786149	0.361865	GYG1
1	118686196	118786196	0.347315	HLTF
1	118686196	118786196	0.347315	GYG1
1	118686383	118786383	0.347315	HLTF
1	118686383	118786383	0.347315	GYG1
1	118686893	118786893	0.353537	HLTF
1	118686893	118786893	0.353537	GYG1
1	118687382	118787382	0.352608	HLTF
1	118687382	118787382	0.352608	GYG1
1	118687772	118787772	0.352608	HLTF
1	118687772	118787772	0.352608	GYG1
1	118687919	118787919	0.352608	HLTF
1	118687919	118787919	0.352608	GYG1
1	118688102	118788102	0.353062	HLTF
1	118688102	118788102	0.353062	GYG1
1	118688187	118788187	0.352717	HLTF
1	118688187	118788187	0.352717	GYG1
1	118688225	118788225	0.35345	HLTF
1	118688225	118788225	0.35345	GYG1
1	118688327	118788327	0.408509	HLTF
1	118688327	118788327	0.408509	GYG1
1	118688333	118788333	0.391826	HLTF
1	118688333	118788333	0.391826	GYG1
1	118688341	118788341	0.391826	HLTF
1	118688341	118788341	0.391826	GYG1
1	118688428	118788428	0.388154	HLTF
1	118688428	118788428	0.388154	GYG1
1	118688505	118788505	0.387291	HLTF
1	118688505	118788505	0.387291	GYG1
1	118688571	118788571	0.457603	HLTF
1	118688571	118788571	0.457603	GYG1
1	118688621	118788621	0.428935	HLTF
1	118688621	118788621	0.428935	GYG1
1	118689211	118789211	0.428935	HLTF
1	118689211	118789211	0.428935	GYG1

1	118689287	118789287	0.428935	HLTF
1	118689287	118789287	0.428935	GYG1
1	118689702	118789702	0.428935	HLTF
1	118689702	118789702	0.428935	GYG1
1	118689733	118789733	0.428935	HLTF
1	118689733	118789733	0.428935	GYG1
1	118689736	118789736	0.428935	HLTF
1	118689736	118789736	0.428935	GYG1
1	118689858	118789858	0.436396	HLTF
1	118689858	118789858	0.436396	GYG1
1	118689997	118789997	0.44725	HLTF
1	118689997	118789997	0.44725	GYG1
1	118690423	118790423	0.414388	HLTF
1	118690423	118790423	0.414388	GYG1
1	118691478	118791478	0.414989	HLTF
1	118691478	118791478	0.414989	GYG1
1	118691773	118791773	0.43593	HLTF
1	118691773	118791773	0.43593	GYG1
1	118691973	118791973	0.420536	HLTF
1	118691973	118791973	0.420536	GYG1
1	118692574	118792574	0.437582	HLTF
1	118692574	118792574	0.437582	GYG1
1	118692577	118792577	0.437582	HLTF
1	118692577	118792577	0.437582	GYG1
1	118692776	118792776	0.464604	HLTF
1	118692776	118792776	0.464604	GYG1
1	118693138	118793138	0.442988	HLTF
1	118693138	118793138	0.442988	GYG1
1	118693190	118793190	0.442988	HLTF
1	118693190	118793190	0.442988	GYG1
1	118693833	118793833	0.440814	HLTF
1	118693833	118793833	0.440814	GYG1
1	118694110	118794110	0.463155	HLTF
1	118694110	118794110	0.463155	GYG1
1	118694328	118794328	0.475844	HLTF
1	118694328	118794328	0.475844	GYG1
1	118694526	118794526	0.445501	HLTF
1	118694526	118794526	0.445501	GYG1
1	118694846	118794846	0.441837	HLTF
1	118694846	118794846	0.441837	GYG1
1	118695184	118795184	0.440094	HLTF
1	118695184	118795184	0.440094	GYG1
1	118696216	118796216	0.436809	HLTF
1	118696216	118796216	0.436809	GYG1
1	118696256	118796256	0.449127	HLTF
1	118696256	118796256	0.449127	GYG1
1	118696437	118796437	0.433007	HLTF
1	118696437	118796437	0.433007	GYG1
1	118696456	118796456	0.433007	HLTF
1	118696456	118796456	0.433007	GYG1
1	118696530	118796530	0.433007	HLTF
1	118696530	118796530	0.433007	GYG1
1	118696563	118796563	0.434392	HLTF
1	118696563	118796563	0.434392	GYG1
1	118696786	118796786	0.434158	HLTF
1	118696786	118796786	0.434158	GYG1
1	118696903	118796903	0.445957	HLTF
1	118696903	118796903	0.445957	GYG1

1	118696913	118796913	0.4572	HLTF
1	118696913	118796913	0.4572	GYG1
1	118696923	118796923	0.443314	HLTF
1	118696923	118796923	0.443314	GYG1
1	118696992	118796992	0.442661	HLTF
1	118696992	118796992	0.442661	GYG1
1	118697095	118797095	0.442661	HLTF
1	118697095	118797095	0.442661	GYG1
1	118697187	118797187	0.440252	HLTF
1	118697187	118797187	0.440252	GYG1
1	118697403	118797403	0.415455	HLTF
1	118697403	118797403	0.415455	GYG1
1	118697537	118797537	0.408505	HLTF
1	118697537	118797537	0.408505	GYG1
1	118697803	118797803	0.394575	HLTF
1	118697803	118797803	0.394575	GYG1
1	118697935	118797935	0.391219	HLTF
1	118697935	118797935	0.391219	GYG1
1	118698759	118798759	0.369436	HLTF
1	118698759	118798759	0.369436	GYG1
1	118698763	118798763	0.372012	HLTF
1	118698763	118798763	0.372012	GYG1
1	118698774	118798774	0.397846	HLTF
1	118698774	118798774	0.397846	GYG1
1	118698776	118798776	0.401312	HLTF
1	118698776	118798776	0.401312	GYG1
1	118698790	118798790	0.402619	HLTF
1	118698790	118798790	0.402619	GYG1
1	118698949	118798949	0.398255	HLTF
1	118698949	118798949	0.398255	GYG1
1	118699030	118799030	0.397826	HLTF
1	118699030	118799030	0.397826	GYG1
1	118699050	118799050	0.397826	HLTF
1	118699050	118799050	0.397826	GYG1
1	118699095	118799095	0.397826	HLTF
1	118699095	118799095	0.397826	GYG1
1	118699126	118799126	0.397826	HLTF
1	118699126	118799126	0.397826	GYG1
1	118699131	118799131	0.397826	HLTF
1	118699131	118799131	0.397826	GYG1
1	118699136	118799136	0.384157	HLTF
1	118699136	118799136	0.384157	GYG1
1	118699182	118799182	0.384157	HLTF
1	118699182	118799182	0.384157	GYG1
1	118699313	118799313	0.384157	HLTF
1	118699313	118799313	0.384157	GYG1
1	118699362	118799362	0.384157	HLTF
1	118699362	118799362	0.384157	GYG1
1	118699427	118799427	0.3777	HLTF
1	118699427	118799427	0.3777	GYG1
1	118699434	118799434	0.3777	HLTF
1	118699434	118799434	0.3777	GYG1
1	118699443	118799443	0.3777	HLTF
1	118699443	118799443	0.3777	GYG1
1	118699452	118799452	0.3777	HLTF
1	118699452	118799452	0.3777	GYG1
1	118699534	118799534	0.37821	HLTF
1	118699534	118799534	0.37821	GYG1

1	118699556	118799556	0.391231	HLTF
1	118699556	118799556	0.391231	GYG1
1	118699570	118799570	0.391231	HLTF
1	118699570	118799570	0.391231	GYG1
1	118699588	118799588	0.391231	HLTF
1	118699588	118799588	0.391231	GYG1
1	118699645	118799645	0.389273	HLTF
1	118699645	118799645	0.389273	GYG1
1	118699653	118799653	0.385368	HLTF
1	118699653	118799653	0.385368	GYG1
1	118699741	118799741	0.388378	HLTF
1	118699741	118799741	0.388378	GYG1
1	118699766	118799766	0.386985	HLTF
1	118699766	118799766	0.386985	GYG1
1	118699880	118799880	0.391281	HLTF
1	118699880	118799880	0.391281	GYG1
1	118940776	119040776	0.353634	CPB1
1	118940972	119040972	0.345623	CPB1
1	118941120	119041120	0.345623	CPB1
1	118941318	119041318	0.343301	CPB1
1	118941324	119041324	0.343301	CPB1
1	118941609	119041609	0.346429	CPB1
1	118941994	119041994	0.360806	CPB1
1	118947423	119047423	0.34399	CPB1
1	118947457	119047457	0.395375	CPB1
1	118947569	119047569	0.394681	CPB1
1	118947584	119047584	0.408506	CPB1
1	118947587	119047587	0.405272	CPB1
1	118947588	119047588	0.405272	CPB1
1	118947718	119047718	0.396826	CPB1
1	118947724	119047724	0.396826	CPB1
1	118947969	119047969	0.372499	CPB1
1	118948131	119048131	0.374207	CPB1
1	118948258	119048258	0.355967	CPB1
1	118948320	119048320	0.355973	CPB1
1	118948465	119048465	0.354498	CPB1
1	118948506	119048506	0.353423	CPB1
1	118948606	119048606	0.35692	CPB1
1	118948608	119048608	0.35692	CPB1
1	118948877	119048877	0.356526	CPB1
1	118948955	119048955	0.356347	CPB1
1	118949220	119049220	0.356433	CPB1
1	118949679	119049679	0.344522	CPB1
1	118949768	119049768	0.343563	CPB1
1	118949834	119049834	0.343563	CPB1
1	118949978	119049978	0.346919	CPB1
1	118950316	119050316	0.374311	CPB1
1	118950561	119050561	0.349105	CPB1
1	118951042	119051042	0.352064	CPB1
1	118951158	119051158	0.344063	CPB1
1	118951669	119051669	0.352909	CPB1
1	118951709	119051709	0.356065	CPB1
1	118952331	119052331	0.346128	CPB1
1	118953096	119053096	0.376533	CPB1
1	118953434	119053434	0.365798	CPB1
1	118953705	119053705	0.384513	CPB1
1	118953748	119053748	0.375491	CPB1
1	118953809	119053809	0.373685	CPB1

1	118953872	119053872	0.364813	CPB1
1	118953992	119053992	0.358478	CPB1
1	118954186	119054186	0.358478	CPB1
1	118954276	119054276	0.351585	CPB1
1	118954309	119054309	0.351585	CPB1
1	118954383	119054383	0.351585	CPB1
1	118968841	119068841	0.344505	AGTR1
1	118968927	119068927	0.359593	AGTR1
1	118969285	119069285	0.349206	AGTR1
1	118969414	119069414	0.349206	AGTR1
1	118969735	119069735	0.352153	AGTR1
1	118969814	119069814	0.363444	AGTR1
1	118969932	119069932	0.393417	AGTR1
1	118970083	119070083	0.407252	AGTR1
1	118970289	119070289	0.396059	AGTR1
1	118970581	119070581	0.397189	AGTR1
1	118970673	119070673	0.374049	AGTR1
1	118970798	119070798	0.392031	AGTR1
1	118971180	119071180	0.373479	AGTR1
1	118971568	119071568	0.364182	AGTR1
1	118972382	119072382	0.391206	AGTR1
1	118972383	119072383	0.351858	AGTR1
1	118972688	119072688	0.365016	AGTR1
1	118972711	119072711	0.36119	AGTR1
1	118973192	119073192	0.383326	AGTR1
1	118973206	119073206	0.364566	AGTR1
1	118973452	119073452	0.363446	AGTR1
1	118973497	119073497	0.353142	AGTR1
1	118973916	119073916	0.363209	AGTR1
1	118974136	119074136	0.370442	AGTR1
1	118974202	119074202	0.378748	AGTR1
1	118974426	119074426	0.365072	AGTR1
1	118974676	119074676	0.35492	AGTR1
1	118975064	119075064	0.346387	AGTR1
1	118977556	119077556	0.34256	AGTR1
1	118978706	119078706	0.343859	AGTR1
1	119025170	119125170	0.413426	AGTR1
1	119025331	119125331	0.404856	AGTR1
1	119025389	119125389	0.414472	AGTR1
1	119025492	119125492	0.408005	AGTR1
1	119025536	119125536	0.427418	AGTR1
1	119025560	119125560	0.440331	AGTR1
1	119025619	119125619	0.434637	AGTR1
1	119025677	119125677	0.447737	AGTR1
1	119025716	119125716	0.490978	AGTR1
1	119025718	119125718	0.34472	AGTR1
1	119025797	119125797	0.344418	AGTR1
1	119025855	119125855	0.35455	AGTR1
1	119025876	119125876	0.347644	AGTR1
1	121418225	121518225	0.36062	PLSCR5
1	121418231	121518231	0.360686	PLSCR5
1	124827672	124927672	0.343175	TRNAK-UUU-4
1	124827672	124927672	0.343175	SLC9A9
1	124827694	124927694	0.34354	TRNAK-UUU-4
1	124827694	124927694	0.34354	SLC9A9
1	124833470	124933470	0.343528	TRNAK-UUU-4
1	124833470	124933470	0.343528	SLC9A9
1	128315484	128415484	0.357779	CLSTN2

1	128315485	128415485	0.400749	CLSTN2
1	128315733	128415733	0.365572	CLSTN2
1	128316130	128416130	0.351638	CLSTN2
1	128316176	128416176	0.363684	CLSTN2
1	128316768	128416768	0.372358	CLSTN2
1	128316780	128416780	0.40309	CLSTN2
1	128316891	128416891	0.433515	CLSTN2
1	128317096	128417096	0.435785	CLSTN2
1	128317290	128417290	0.423441	CLSTN2
1	128317321	128417321	0.414318	CLSTN2
1	128317733	128417733	0.384855	CLSTN2
1	128317769	128417769	0.380288	CLSTN2
1	128317782	128417782	0.378612	CLSTN2
1	128317919	128417919	0.387959	CLSTN2
1	128318048	128418048	0.376642	CLSTN2
1	128318297	128418297	0.353271	CLSTN2
1	128318546	128418546	0.361777	CLSTN2
1	128318762	128418762	0.374547	CLSTN2
1	128318801	128418801	0.387419	CLSTN2
1	128318864	128418864	0.400126	CLSTN2
1	128322586	128422586	0.386166	CLSTN2
1	128322724	128422724	0.381761	CLSTN2
1	128322900	128422900	0.370214	CLSTN2
1	128323019	128423019	0.401623	CLSTN2
1	128323043	128423043	0.366695	CLSTN2
1	128323072	128423072	0.346284	CLSTN2
1	128324082	128424082	0.37957	CLSTN2
1	128324220	128424220	0.351462	CLSTN2
1	128326547	128426547	0.34426	CLSTN2
1	128327701	128427701	0.362042	CLSTN2
1	128327755	128427755	0.353899	CLSTN2
1	128327855	128427855	0.353899	CLSTN2
1	128328068	128428068	0.353899	CLSTN2
1	128328155	128428155	0.37371	CLSTN2
1	128328194	128428194	0.352901	CLSTN2
1	128328202	128428202	0.353574	CLSTN2
1	128332826	128432826	0.355054	CLSTN2
1	128333418	128433418	0.345755	CLSTN2
1	128333782	128433782	0.351778	CLSTN2
1	128333816	128433816	0.341747	CLSTN2
1	132992828	133092828	0.520152	LOC102168581
1	132992830	133092830	0.526639	LOC102168581
1	132992832	133092832	0.471966	LOC102168581
1	135507240	135607240	0.392038	BFSP2
1	135507240	135607240	0.392038	TMEM108
1	135507241	135607241	0.3725	BFSP2
1	135507241	135607241	0.3725	TMEM108
1	135507251	135607251	0.406081	BFSP2
1	135507251	135607251	0.406081	TMEM108
1	135507325	135607325	0.354102	BFSP2
1	135507325	135607325	0.354102	TMEM108
1	136992086	137092086	0.356395	TRNAC-GCA-14
1	136992120	137092120	0.355623	TRNAC-GCA-14
1	137217311	137317311	0.346904	CPNE4
1	137217659	137317659	0.349859	CPNE4
1	137217669	137317669	0.350941	CPNE4
1	137217776	137317776	0.350941	CPNE4
1	137217895	137317895	0.354027	CPNE4

1	137217932	137317932	0.344955	CPNE4
1	137217969	137317969	0.343626	CPNE4
1	137218096	137318096	0.343428	CPNE4
1	137218264	137318264	0.347968	CPNE4
1	137219702	137319702	0.342667	CPNE4
1	137219833	137319833	0.341907	CPNE4
1	137220887	137320887	0.34959	CPNE4
1	137221818	137321818	0.348634	CPNE4
1	137228868	137328868	0.3489	CPNE4
1	137228987	137328987	0.346756	CPNE4
1	137229306	137329306	0.342878	CPNE4
1	137229563	137329563	0.347719	CPNE4
1	137230020	137330020	0.343118	CPNE4
1	137231803	137331803	0.343615	CPNE4
1	137231863	137331863	0.357808	CPNE4
1	137231870	137331870	0.349954	CPNE4
1	137232882	137332882	0.350495	CPNE4
1	137232887	137332887	0.349071	CPNE4
1	137232888	137332888	0.349981	CPNE4
1	139181945	139281945	0.369212	SH3BGR
1	139181950	139281950	0.376161	SH3BGR
1	139181959	139281959	0.376161	SH3BGR
1	139181968	139281968	0.376161	SH3BGR
1	139182025	139282025	0.378215	SH3BGR
1	139182067	139282067	0.377607	SH3BGR
1	139182111	139282111	0.377607	SH3BGR
1	139182153	139282153	0.377607	SH3BGR
1	139182196	139282196	0.37775	SH3BGR
1	139182203	139282203	0.37775	SH3BGR
1	139182208	139282208	0.37775	SH3BGR
1	139182210	139282210	0.378036	SH3BGR
1	139182266	139282266	0.378036	SH3BGR
1	139182310	139282310	0.368935	SH3BGR
1	139182315	139282315	0.373534	SH3BGR
1	139182393	139282393	0.359946	SH3BGR
1	139182416	139282416	0.349671	SH3BGR
1	139182428	139282428	0.349671	SH3BGR
1	139182452	139282452	0.349671	SH3BGR
1	139182666	139282666	0.351115	SH3BGR
1	139182723	139282723	0.366931	SH3BGR
1	139182831	139282831	0.370787	SH3BGR
1	139182861	139282861	0.370787	SH3BGR
1	139182981	139282981	0.378469	SH3BGR
1	139183022	139283022	0.392032	SH3BGR
1	139183049	139283049	0.378545	SH3BGR
1	139183051	139283051	0.37857	SH3BGR
1	141306288	141406288	0.342897	TMPRSS2
1	141353990	141453990	0.342388	LOC108636430
1	141353990	141453990	0.342388	LOC100861174
1	141353990	141453990	0.342388	LOC108636431
1	141354009	141454009	0.342388	LOC108636430
1	141354009	141454009	0.342388	LOC100861174
1	141354009	141454009	0.342388	LOC108636431
1	142171887	142271887	0.356425	TFF2
1	142171887	142271887	0.356425	TMPRSS3
1	142171887	142271887	0.356425	TFF1
1	142172017	142272017	0.350689	TFF2
1	142172017	142272017	0.350689	TMPRSS3

1	142172017	142272017	0.350689	TFF1
1	142172085	142272085	0.351344	TFF2
1	142172085	142272085	0.351344	TMPRSS3
1	142172085	142272085	0.351344	TFF1
1	142172133	142272133	0.35205	TFF2
1	142172133	142272133	0.35205	TMPRSS3
1	142172133	142272133	0.35205	TFF1
1	142172235	142272235	0.34199	TFF2
1	142172235	142272235	0.34199	TMPRSS3
1	142172235	142272235	0.34199	TFF1
1	143321185	143421185	0.34419	LOC102186527
1	143321185	143421185	0.34419	RRP1B
1	143321185	143421185	0.34419	PDXK
1	143321263	143421263	0.343141	LOC102186527
1	143321263	143421263	0.343141	RRP1B
1	143321263	143421263	0.343141	PDXK
1	143321278	143421278	0.347177	LOC102186527
1	143321278	143421278	0.347177	RRP1B
1	143321278	143421278	0.347177	PDXK
1	143321328	143421328	0.350914	LOC102186527
1	143321328	143421328	0.350914	RRP1B
1	143321328	143421328	0.350914	PDXK
1	143321658	143421658	0.357363	LOC102186527
1	143321658	143421658	0.357363	RRP1B
1	143321658	143421658	0.357363	PDXK
1	143321682	143421682	0.366577	LOC102186527
1	143321682	143421682	0.366577	RRP1B
1	143321682	143421682	0.366577	PDXK
1	143321758	143421758	0.379896	LOC102186527
1	143321758	143421758	0.379896	RRP1B
1	143321758	143421758	0.379896	PDXK
1	143321782	143421782	0.375221	LOC102186527
1	143321782	143421782	0.375221	RRP1B
1	143321782	143421782	0.375221	PDXK
1	143321783	143421783	0.375227	LOC102186527
1	143321783	143421783	0.375227	RRP1B
1	143321783	143421783	0.375227	PDXK
1	143321869	143421869	0.37877	LOC102186527
1	143321869	143421869	0.37877	RRP1B
1	143321869	143421869	0.37877	PDXK
1	143321870	143421870	0.378446	LOC102186527
1	143321870	143421870	0.378446	RRP1B
1	143321870	143421870	0.378446	PDXK
1	143321894	143421894	0.385377	LOC102186527
1	143321894	143421894	0.385377	RRP1B
1	143321894	143421894	0.385377	PDXK
1	143322025	143422025	0.385937	LOC102186527
1	143322025	143422025	0.385937	RRP1B
1	143322025	143422025	0.385937	PDXK
1	143322179	143422179	0.36515	LOC102186527
1	143322179	143422179	0.36515	RRP1B
1	143322179	143422179	0.36515	PDXK
1	143322305	143422305	0.363401	LOC102186527
1	143322305	143422305	0.363401	RRP1B
1	143322305	143422305	0.363401	PDXK
1	143322470	143422470	0.363401	LOC102186527
1	143322470	143422470	0.363401	RRP1B
1	143322470	143422470	0.363401	PDXK

1	143322488	143422488	0.363401	LOC102186527
1	143322488	143422488	0.363401	RRP1B
1	143322488	143422488	0.363401	PDXK
1	143322556	143422556	0.343467	LOC102186527
1	143322556	143422556	0.343467	RRP1B
1	143322556	143422556	0.343467	PDXK
1	143322622	143422622	0.357398	LOC102186527
1	143322622	143422622	0.357398	RRP1B
1	143322622	143422622	0.357398	PDXK
1	143322690	143422690	0.359898	LOC102186527
1	143322690	143422690	0.359898	RRP1B
1	143322690	143422690	0.359898	PDXK
1	143322703	143422703	0.352303	LOC102186527
1	143322703	143422703	0.352303	RRP1B
1	143322703	143422703	0.352303	PDXK
1	143322741	143422741	0.355873	LOC102186527
1	143322741	143422741	0.355873	RRP1B
1	143322741	143422741	0.355873	PDXK
1	143322770	143422770	0.355707	LOC102186527
1	143322770	143422770	0.355707	RRP1B
1	143322770	143422770	0.355707	PDXK
1	143322774	143422774	0.364529	LOC102186527
1	143322774	143422774	0.364529	RRP1B
1	143322774	143422774	0.364529	PDXK
1	143322786	143422786	0.381641	LOC102186527
1	143322786	143422786	0.381641	RRP1B
1	143322786	143422786	0.381641	PDXK
1	143322859	143422859	0.397806	LOC102186527
1	143322859	143422859	0.397806	RRP1B
1	143322859	143422859	0.397806	PDXK
1	143322885	143422885	0.396449	LOC102186527
1	143322885	143422885	0.396449	RRP1B
1	143322885	143422885	0.396449	PDXK
1	143322984	143422984	0.413343	LOC102186527
1	143322984	143422984	0.413343	RRP1B
1	143322984	143422984	0.413343	PDXK
1	143322991	143422991	0.398619	LOC102186527
1	143322991	143422991	0.398619	RRP1B
1	143322991	143422991	0.398619	PDXK
1	143323071	143423071	0.396179	LOC102186527
1	143323071	143423071	0.396179	RRP1B
1	143323071	143423071	0.396179	PDXK
1	143323076	143423076	0.396179	LOC102186527
1	143323076	143423076	0.396179	RRP1B
1	143323076	143423076	0.396179	PDXK
1	143323167	143423167	0.372773	LOC102186527
1	143323167	143423167	0.372773	RRP1B
1	143323167	143423167	0.372773	PDXK
1	143323168	143423168	0.377477	LOC102186527
1	143323168	143423168	0.377477	RRP1B
1	143323168	143423168	0.377477	PDXK
1	143323240	143423240	0.367839	LOC102186527
1	143323240	143423240	0.367839	RRP1B
1	143323240	143423240	0.367839	PDXK
1	143893642	143993642	0.350005	AIRE
1	143893642	143993642	0.350005	PFKL
1	143893677	143993677	0.398312	AIRE
1	143893677	143993677	0.398312	PFKL

1	143893686	143993686	0.398312	AIRE
1	143893686	143993686	0.398312	PFKL
1	143893725	143993725	0.376549	AIRE
1	143893725	143993725	0.376549	PFKL
1	143893731	143993731	0.360667	AIRE
1	143893731	143993731	0.360667	PFKL
1	143902061	144002061	0.345977	AIRE
1	143902061	144002061	0.345977	PFKL
1	143902065	144002065	0.345977	AIRE
1	143902065	144002065	0.345977	PFKL
1	143902301	144002301	0.405384	AIRE
1	143902301	144002301	0.405384	PFKL
1	143902445	144002445	0.345905	AIRE
1	143902445	144002445	0.345905	PFKL
1	143902488	144002488	0.343265	AIRE
1	143902488	144002488	0.343265	PFKL
1	143902688	144002688	0.380425	AIRE
1	143902688	144002688	0.380425	PFKL
1	143904034	144004034	0.432883	AIRE
1	143904034	144004034	0.432883	PFKL
1	143904100	144004100	0.386425	AIRE
1	143904100	144004100	0.386425	PFKL
1	143904159	144004159	0.504087	AIRE
1	143904159	144004159	0.504087	PFKL
1	143904215	144004215	0.476608	AIRE
1	143904215	144004215	0.476608	PFKL
1	143904220	144004220	0.495258	AIRE
1	143904220	144004220	0.495258	PFKL
1	143911217	144011217	0.364204	PFKL
1	143912924	144012924	0.379195	PFKL
1	143913070	144013070	0.344617	PFKL
1	143913206	144013206	0.487929	PFKL
1	143913223	144013223	0.527395	PFKL
1	143913253	144013253	0.512982	PFKL
1	143913254	144013254	0.513571	PFKL
1	143913306	144013306	0.483318	PFKL
1	143913335	144013335	0.428497	PFKL
1	143913344	144013344	0.430635	PFKL
1	143913366	144013366	0.398487	PFKL
1	143913382	144013382	0.356523	PFKL
1	143914790	144014790	0.373025	PFKL
1	143915637	144015637	0.373068	PFKL
1	143915983	144015983	0.417783	PFKL
1	143916243	144016243	0.369554	PFKL
1	143916845	144016845	0.352062	PFKL
1	143917227	144017227	0.399969	PFKL
1	143917233	144017233	0.391633	PFKL
1	143917245	144017245	0.386558	PFKL
1	143918430	144018430	0.35318	PFKL
1	144102185	144202185	0.445855	LOC108636542
1	144102265	144202265	0.378488	LOC108636542
1	144102426	144202426	0.398342	LOC108636542
1	144110337	144210337	0.369653	LOC108636542
1	144158821	144258821	0.342639	LOC108636542
1	144158821	144258821	0.342639	TRNAC-GCA-15
1	144158821	144258821	0.342639	LOC108636378
1	144158821	144258821	0.342639	C1H21orf2
1	144158843	144258843	0.369554	LOC108636542

1	144158843	144258843	0.369554	TRNAC-GCA-15
1	144158843	144258843	0.369554	LOC108636378
1	144158843	144258843	0.369554	C1H21orf2
1	144158849	144258849	0.416455	LOC108636542
1	144158849	144258849	0.416455	TRNAC-GCA-15
1	144158849	144258849	0.416455	LOC108636378
1	144158849	144258849	0.416455	C1H21orf2
1	144623524	144723524	0.349793	PTTG1IP
1	144623524	144723524	0.349793	ITGB2
1	145222421	145322421	0.360178	SLC19A1
1	145222421	145322421	0.360178	COL18A1
1	146038961	146138961	0.345732	LOC108636576
1	146038961	146138961	0.345732	PCNT
1	146038961	146138961	0.345732	DIP2A
1	146038973	146138973	0.347188	LOC108636576
1	146038973	146138973	0.347188	PCNT
1	146038973	146138973	0.347188	DIP2A
1	146039049	146139049	0.348968	LOC108636576
1	146039049	146139049	0.348968	PCNT
1	146039049	146139049	0.348968	DIP2A
1	146039126	146139126	0.344594	LOC108636576
1	146039126	146139126	0.344594	PCNT
1	146039126	146139126	0.344594	DIP2A
1	146039163	146139163	0.344594	LOC108636576
1	146039163	146139163	0.344594	PCNT
1	146039163	146139163	0.344594	DIP2A
1	146039238	146139238	0.344793	LOC108636576
1	146039238	146139238	0.344793	PCNT
1	146039238	146139238	0.344793	DIP2A
1	146051844	146151844	0.354903	LOC108636576
1	146051844	146151844	0.354903	PCNT
1	146051844	146151844	0.354903	DIP2A
1	146051922	146151922	0.350391	LOC108636576
1	146051922	146151922	0.350391	PCNT
1	146051922	146151922	0.350391	DIP2A
1	146051997	146151997	0.35315	LOC108636576
1	146051997	146151997	0.35315	PCNT
1	146051997	146151997	0.35315	DIP2A
1	146052964	146152964	0.349655	LOC108636576
1	146052964	146152964	0.349655	PCNT
1	146052964	146152964	0.349655	DIP2A
1	146053004	146153004	0.347814	LOC108636576
1	146053004	146153004	0.347814	PCNT
1	146053004	146153004	0.347814	DIP2A
1	146053960	146153960	0.351523	LOC108636576
1	146053960	146153960	0.351523	PCNT
1	146053960	146153960	0.351523	DIP2A
1	146054863	146154863	0.345814	LOC108636576
1	146054863	146154863	0.345814	PCNT
1	146054863	146154863	0.345814	DIP2A
1	146054916	146154916	0.345814	LOC108636576
1	146054916	146154916	0.345814	PCNT
1	146054916	146154916	0.345814	DIP2A
1	146055257	146155257	0.345814	LOC108636576
1	146055257	146155257	0.345814	PCNT
1	146055257	146155257	0.345814	DIP2A
1	146055303	146155303	0.345814	LOC108636576
1	146055303	146155303	0.345814	PCNT

1	146055303	146155303	0.345814	DIP2A
1	146055304	146155304	0.345814	LOC108636576
1	146055304	146155304	0.345814	PCNT
1	146055304	146155304	0.345814	DIP2A
1	146055567	146155567	0.34505	LOC108636576
1	146055567	146155567	0.34505	PCNT
1	146055567	146155567	0.34505	DIP2A
1	146059089	146159089	0.341983	LOC108636576
1	146059089	146159089	0.341983	PCNT
1	146059089	146159089	0.341983	DIP2A
1	146059120	146159120	0.343011	LOC108636576
1	146059120	146159120	0.343011	PCNT
1	146059120	146159120	0.343011	DIP2A
1	146059744	146159744	0.346156	LOC108636576
1	146059744	146159744	0.346156	PCNT
1	146059744	146159744	0.346156	DIP2A
1	146059883	146159883	0.379517	LOC108636576
1	146059883	146159883	0.379517	PCNT
1	146059883	146159883	0.379517	DIP2A
1	146059938	146159938	0.380584	LOC108636576
1	146059938	146159938	0.380584	PCNT
1	146059938	146159938	0.380584	DIP2A
1	146059945	146159945	0.380584	LOC108636576
1	146059945	146159945	0.380584	PCNT
1	146059945	146159945	0.380584	DIP2A
1	146059947	146159947	0.376891	LOC108636576
1	146059947	146159947	0.376891	PCNT
1	146059947	146159947	0.376891	DIP2A
1	146060017	146160017	0.378725	LOC108636576
1	146060017	146160017	0.378725	PCNT
1	146060017	146160017	0.378725	DIP2A
1	146060047	146160047	0.378725	LOC108636576
1	146060047	146160047	0.378725	PCNT
1	146060047	146160047	0.378725	DIP2A
1	146060158	146160158	0.378148	LOC108636576
1	146060158	146160158	0.378148	PCNT
1	146060158	146160158	0.378148	DIP2A
1	146060185	146160185	0.378148	LOC108636576
1	146060185	146160185	0.378148	PCNT
1	146060185	146160185	0.378148	DIP2A
1	146060253	146160253	0.381945	LOC108636576
1	146060253	146160253	0.381945	PCNT
1	146060253	146160253	0.381945	DIP2A
1	146060279	146160279	0.371087	LOC108636576
1	146060279	146160279	0.371087	PCNT
1	146060279	146160279	0.371087	DIP2A
1	146060439	146160439	0.364	LOC108636576
1	146060439	146160439	0.364	PCNT
1	146060439	146160439	0.364	DIP2A
1	146060551	146160551	0.368229	LOC108636576
1	146060551	146160551	0.368229	PCNT
1	146060551	146160551	0.368229	DIP2A
1	146060591	146160591	0.379277	LOC108636576
1	146060591	146160591	0.379277	PCNT
1	146060591	146160591	0.379277	DIP2A
1	146060594	146160594	0.373811	LOC108636576
1	146060594	146160594	0.373811	PCNT
1	146060594	146160594	0.373811	DIP2A

1	146060626	146160626	0.381006	LOC108636576
1	146060626	146160626	0.381006	PCNT
1	146060626	146160626	0.381006	DIP2A
1	146060716	146160716	0.382118	LOC108636576
1	146060716	146160716	0.382118	PCNT
1	146060716	146160716	0.382118	DIP2A
1	146060744	146160744	0.373111	LOC108636576
1	146060744	146160744	0.373111	PCNT
1	146060744	146160744	0.373111	DIP2A
1	146060750	146160750	0.353514	LOC108636576
1	146060750	146160750	0.353514	PCNT
1	146060750	146160750	0.353514	DIP2A
1	146060897	146160897	0.353514	LOC108636576
1	146060897	146160897	0.353514	PCNT
1	146060897	146160897	0.353514	DIP2A
1	146061018	146161018	0.353514	LOC108636576
1	146061018	146161018	0.353514	PCNT
1	146061018	146161018	0.353514	DIP2A
1	146061116	146161116	0.35522	LOC108636576
1	146061116	146161116	0.35522	PCNT
1	146061116	146161116	0.35522	DIP2A
1	146061146	146161146	0.35522	LOC108636576
1	146061146	146161146	0.35522	PCNT
1	146061146	146161146	0.35522	DIP2A
1	146061152	146161152	0.35522	LOC108636576
1	146061152	146161152	0.35522	PCNT
1	146061152	146161152	0.35522	DIP2A
1	146061626	146161626	0.35522	LOC108636576
1	146061626	146161626	0.35522	PCNT
1	146061626	146161626	0.35522	DIP2A
1	146061770	146161770	0.35522	LOC108636576
1	146061770	146161770	0.35522	PCNT
1	146061770	146161770	0.35522	DIP2A
1	146062145	146162145	0.379255	LOC108636576
1	146062145	146162145	0.379255	PCNT
1	146062145	146162145	0.379255	DIP2A
1	146062251	146162251	0.379255	LOC108636576
1	146062251	146162251	0.379255	PCNT
1	146062251	146162251	0.379255	DIP2A
1	146062323	146162323	0.38096	LOC108636576
1	146062323	146162323	0.38096	PCNT
1	146062323	146162323	0.38096	DIP2A
1	146062358	146162358	0.367979	LOC108636576
1	146062358	146162358	0.367979	PCNT
1	146062358	146162358	0.367979	DIP2A
1	146062403	146162403	0.360489	LOC108636576
1	146062403	146162403	0.360489	PCNT
1	146062403	146162403	0.360489	DIP2A
1	146063040	146163040	0.365852	LOC108636576
1	146063040	146163040	0.365852	PCNT
1	146063040	146163040	0.365852	DIP2A
1	146063326	146163326	0.356137	LOC108636576
1	146063326	146163326	0.356137	PCNT
1	146063326	146163326	0.356137	DIP2A
1	146063608	146163608	0.343097	LOC108636576
1	146063608	146163608	0.343097	PCNT
1	146063608	146163608	0.343097	DIP2A
1	146063654	146163654	0.345522	LOC108636576

1	146063654	146163654	0.345522	PCNT
1	146063654	146163654	0.345522	DIP2A
1	146064262	146164262	0.370063	LOC108636576
1	146064262	146164262	0.370063	PCNT
1	146064262	146164262	0.370063	DIP2A
1	146064302	146164302	0.370063	LOC108636576
1	146064302	146164302	0.370063	PCNT
1	146064302	146164302	0.370063	DIP2A
1	146064440	146164440	0.361442	LOC108636576
1	146064440	146164440	0.361442	PCNT
1	146064440	146164440	0.361442	DIP2A
1	146064857	146164857	0.356111	LOC108636576
1	146064857	146164857	0.356111	PCNT
1	146064857	146164857	0.356111	DIP2A
1	146064867	146164867	0.362788	LOC108636576
1	146064867	146164867	0.362788	PCNT
1	146064867	146164867	0.362788	DIP2A
1	146065148	146165148	0.375334	LOC108636576
1	146065148	146165148	0.375334	PCNT
1	146065148	146165148	0.375334	DIP2A
1	146065235	146165235	0.379207	LOC108636576
1	146065235	146165235	0.379207	PCNT
1	146065235	146165235	0.379207	DIP2A
1	146065274	146165274	0.438571	LOC108636576
1	146065274	146165274	0.438571	PCNT
1	146065274	146165274	0.438571	DIP2A
1	146065286	146165286	0.437341	LOC108636576
1	146065286	146165286	0.437341	PCNT
1	146065286	146165286	0.437341	DIP2A
1	146065323	146165323	0.432327	LOC108636576
1	146065323	146165323	0.432327	PCNT
1	146065323	146165323	0.432327	DIP2A
1	146065340	146165340	0.430974	LOC108636576
1	146065340	146165340	0.430974	PCNT
1	146065340	146165340	0.430974	DIP2A
1	146065350	146165350	0.389787	LOC108636576
1	146065350	146165350	0.389787	PCNT
1	146065350	146165350	0.389787	DIP2A
1	146065815	146165815	0.442637	LOC108636576
1	146065815	146165815	0.442637	PCNT
1	146065815	146165815	0.442637	DIP2A
1	146066731	146166731	0.390738	LOC108636576
1	146066731	146166731	0.390738	PCNT
1	146066731	146166731	0.390738	DIP2A
1	146067359	146167359	0.418972	LOC108636576
1	146067359	146167359	0.418972	PCNT
1	146067359	146167359	0.418972	DIP2A
1	146067413	146167413	0.418972	LOC108636576
1	146067413	146167413	0.418972	PCNT
1	146067413	146167413	0.418972	DIP2A
1	146067664	146167664	0.364719	LOC108636576
1	146067664	146167664	0.364719	PCNT
1	146067664	146167664	0.364719	DIP2A
1	146067710	146167710	0.380923	LOC108636576
1	146067710	146167710	0.380923	PCNT
1	146067710	146167710	0.380923	DIP2A
1	146068083	146168083	0.374368	LOC108636576
1	146068083	146168083	0.374368	PCNT

1	146068083	146168083	0.374368	DIP2A
1	146069332	146169332	0.378664	LOC108636576
1	146069332	146169332	0.378664	PCNT
1	146069332	146169332	0.378664	DIP2A
1	146069634	146169634	0.377616	LOC108636576
1	146069634	146169634	0.377616	PCNT
1	146069634	146169634	0.377616	DIP2A
1	146070865	146170865	0.367061	LOC108636576
1	146070865	146170865	0.367061	PCNT
1	146070865	146170865	0.367061	DIP2A
1	146070948	146170948	0.361479	LOC108636576
1	146070948	146170948	0.361479	PCNT
1	146070948	146170948	0.361479	DIP2A
1	146071880	146171880	0.374536	LOC108636576
1	146071880	146171880	0.374536	PCNT
1	146071880	146171880	0.374536	DIP2A
1	146072107	146172107	0.36549	LOC108636576
1	146072107	146172107	0.36549	PCNT
1	146072107	146172107	0.36549	DIP2A
1	146072361	146172361	0.367246	LOC108636576
1	146072361	146172361	0.367246	DIP2A
1	146072791	146172791	0.380659	LOC108636576
1	146072791	146172791	0.380659	DIP2A
1	146072798	146172798	0.380659	LOC108636576
1	146072798	146172798	0.380659	DIP2A
1	146072881	146172881	0.38174	LOC108636576
1	146072881	146172881	0.38174	DIP2A
1	146073001	146173001	0.399367	LOC108636576
1	146073001	146173001	0.399367	DIP2A
1	146073199	146173199	0.39475	LOC108636576
1	146073199	146173199	0.39475	DIP2A
1	146073256	146173256	0.38677	LOC108636576
1	146073256	146173256	0.38677	DIP2A
1	146073318	146173318	0.364179	LOC108636576
1	146073318	146173318	0.364179	DIP2A
1	146073491	146173491	0.372232	LOC108636576
1	146073491	146173491	0.372232	DIP2A
1	146073513	146173513	0.373151	LOC108636576
1	146073513	146173513	0.373151	DIP2A
1	146073841	146173841	0.373914	LOC108636576
1	146073841	146173841	0.373914	DIP2A
1	146074016	146174016	0.37457	LOC108636576
1	146074016	146174016	0.37457	DIP2A
1	146074223	146174223	0.372987	LOC108636576
1	146074223	146174223	0.372987	DIP2A
1	146074253	146174253	0.352562	LOC108636576
1	146074253	146174253	0.352562	DIP2A
1	146075306	146175306	0.345813	LOC108636576
1	146075306	146175306	0.345813	DIP2A
1	146075720	146175720	0.36529	LOC108636576
1	146075720	146175720	0.36529	DIP2A
1	146075995	146175995	0.36529	LOC108636576
1	146075995	146175995	0.36529	DIP2A
1	146076052	146176052	0.360751	LOC108636576
1	146076052	146176052	0.360751	DIP2A
1	146076134	146176134	0.360751	LOC108636576
1	146076134	146176134	0.360751	DIP2A
1	146077375	146177375	0.360751	LOC108636576

1	146077375	146177375	0.360751	DIP2A
1	146077391	146177391	0.367085	LOC108636576
1	146077391	146177391	0.367085	DIP2A
1	146077711	146177711	0.36975	LOC108636576
1	146077711	146177711	0.36975	DIP2A
1	146077718	146177718	0.377171	LOC108636576
1	146077718	146177718	0.377171	DIP2A
1	146077965	146177965	0.381021	LOC108636576
1	146077965	146177965	0.381021	DIP2A
1	146078211	146178211	0.381957	LOC108636576
1	146078211	146178211	0.381957	DIP2A
1	146078276	146178276	0.377048	LOC108636576
1	146078276	146178276	0.377048	DIP2A
1	146078495	146178495	0.377396	LOC108636576
1	146078495	146178495	0.377396	DIP2A
1	146078719	146178719	0.370039	LOC108636576
1	146078719	146178719	0.370039	DIP2A
1	146079007	146179007	0.370039	LOC108636576
1	146079007	146179007	0.370039	DIP2A
1	146079183	146179183	0.365217	LOC108636576
1	146079183	146179183	0.365217	DIP2A
1	146079237	146179237	0.365957	LOC108636576
1	146079237	146179237	0.365957	DIP2A
1	146079677	146179677	0.364369	LOC108636576
1	146079677	146179677	0.364369	DIP2A
1	146079728	146179728	0.364369	LOC108636576
1	146079728	146179728	0.364369	DIP2A
1	146079751	146179751	0.364369	LOC108636576
1	146079751	146179751	0.364369	DIP2A
1	146354604	146454604	0.413396	FBXO25
1	146354655	146454655	0.419269	FBXO25
1	146354669	146454669	0.434083	FBXO25
1	146354670	146454670	0.435653	FBXO25
1	146354673	146454673	0.44284	FBXO25
1	146354691	146454691	0.454779	FBXO25
1	146354692	146454692	0.45443	FBXO25
1	146354697	146454697	0.453498	FBXO25
1	146354701	146454701	0.432627	FBXO25
1	146354750	146454750	0.438592	FBXO25
1	146355063	146455063	0.436134	FBXO25
1	146355150	146455150	0.437095	FBXO25
1	146355226	146455226	0.439665	FBXO25
1	146355244	146455244	0.432565	FBXO25
1	146355267	146455267	0.437976	FBXO25
1	146355342	146455342	0.430649	FBXO25
1	146355495	146455495	0.404271	FBXO25
1	146355498	146455498	0.392713	FBXO25
1	146355542	146455542	0.393666	FBXO25
1	146355565	146455565	0.392352	FBXO25
1	146355641	146455641	0.42758	FBXO25
1	146355647	146455647	0.437792	FBXO25
1	146355655	146455655	0.440676	FBXO25
1	146355667	146455667	0.45118	FBXO25
1	146355702	146455702	0.449078	FBXO25
1	146355719	146455719	0.443674	FBXO25
1	146355753	146455753	0.437778	FBXO25
1	146355760	146455760	0.440508	FBXO25
1	146355822	146455822	0.431045	FBXO25

1	146355860	146455860	0.431045	FBXO25
1	146355896	146455896	0.423991	FBXO25
1	146355924	146455924	0.393055	FBXO25
1	146355993	146455993	0.437058	FBXO25
1	146356134	146456134	0.467509	FBXO25
1	146356190	146456190	0.41952	FBXO25
1	146356200	146456200	0.404085	FBXO25
1	146356272	146456272	0.408471	FBXO25
1	146356351	146456351	0.396572	FBXO25
1	146356457	146456457	0.396572	FBXO25
1	146356511	146456511	0.393528	FBXO25
1	146356606	146456606	0.410467	FBXO25
1	146356612	146456612	0.412242	FBXO25
1	146356635	146456635	0.44192	FBXO25
1	146356645	146456645	0.384766	FBXO25
1	146356699	146456699	0.386708	FBXO25
1	146356700	146456700	0.399633	FBXO25
1	146356876	146456876	0.402627	FBXO25
1	146356885	146456885	0.408119	FBXO25
1	146356886	146456886	0.398297	FBXO25
1	146356924	146456924	0.401015	FBXO25
1	146356927	146456927	0.401015	FBXO25
1	146356932	146456932	0.401015	FBXO25
1	146356963	146456963	0.405828	FBXO25
1	146356984	146456984	0.386567	FBXO25
1	146357120	146457120	0.458784	FBXO25
1	146357150	146457150	0.455074	FBXO25
1	146357162	146457162	0.449319	FBXO25
1	146357189	146457189	0.445607	FBXO25
1	146357190	146457190	0.448302	FBXO25
1	146357261	146457261	0.44144	FBXO25
1	146357442	146457442	0.44144	FBXO25
1	146357448	146457448	0.44144	FBXO25
1	146357499	146457499	0.421503	FBXO25
1	146357557	146457557	0.386326	FBXO25
1	146357568	146457568	0.440117	FBXO25
1	146357571	146457571	0.440117	FBXO25
1	146357642	146457642	0.440273	FBXO25
1	146357663	146457663	0.455656	FBXO25
1	146357749	146457749	0.447867	FBXO25
1	146357758	146457758	0.446478	FBXO25
1	146357815	146457815	0.447044	FBXO25
1	146357820	146457820	0.447618	FBXO25
1	146357968	146457968	0.494807	FBXO25
1	146357976	146457976	0.508522	FBXO25
1	146358125	146458125	0.496078	FBXO25
1	146358126	146458126	0.469168	FBXO25
1	146358127	146458127	0.470465	FBXO25
1	146358200	146458200	0.470465	FBXO25
1	146358261	146458261	0.440218	FBXO25
1	146358296	146458296	0.440218	FBXO25
1	146358328	146458328	0.406694	FBXO25
1	146358390	146458390	0.409638	FBXO25
1	146358540	146458540	0.407608	FBXO25
1	146358563	146458563	0.429944	FBXO25
1	146358605	146458605	0.484958	FBXO25
1	146358658	146458658	0.468596	FBXO25
1	146358739	146458739	0.474362	FBXO25

1	146358763	146458763	0.457414	FBXO25
1	146358774	146458774	0.457414	FBXO25
1	146358775	146458775	0.455412	FBXO25
1	146358903	146458903	0.44673	FBXO25
1	146358923	146458923	0.456028	FBXO25
1	146358955	146458955	0.418437	FBXO25
1	146359045	146459045	0.417354	FBXO25
1	146359437	146459437	0.419562	FBXO25
1	146359469	146459469	0.381247	FBXO25
1	146359599	146459599	0.360054	FBXO25
1	146359615	146459615	0.357738	FBXO25
1	146359622	146459622	0.401538	FBXO25
1	146359690	146459690	0.431806	FBXO25
1	146359827	146459827	0.476847	FBXO25
1	146359878	146459878	0.469296	FBXO25
1	146359882	146459882	0.469296	FBXO25
1	146360032	146460032	0.444625	FBXO25
1	146360045	146460045	0.441362	FBXO25
1	146360317	146460317	0.454253	FBXO25
1	146360346	146460346	0.405723	FBXO25
1	146360417	146460417	0.358419	FBXO25
1	146360497	146460497	0.352367	FBXO25
1	146360567	146460567	0.354197	FBXO25
1	146360656	146460656	0.394098	FBXO25
1	146360703	146460703	0.402978	FBXO25
1	146360776	146460776	0.432966	FBXO25
1	146360785	146460785	0.45073	FBXO25
1	146360869	146460869	0.453994	FBXO25
1	146360929	146460929	0.444166	FBXO25
1	146360989	146460989	0.444166	FBXO25
1	146361066	146461066	0.408878	FBXO25
1	146361133	146461133	0.410714	FBXO25
1	146361231	146461231	0.406503	FBXO25
1	146361309	146461309	0.403435	FBXO25
1	146361356	146461356	0.355239	FBXO25
1	146361381	146461381	0.355239	FBXO25
1	146361514	146461514	0.347742	FBXO25
1	146361552	146461552	0.359015	FBXO25
1	146361625	146461625	0.360623	FBXO25
1	146361803	146461803	0.388451	FBXO25
1	146361816	146461816	0.403887	FBXO25
1	146361843	146461843	0.405606	FBXO25
1	146362200	146462200	0.395131	FBXO25
1	146362228	146462228	0.361537	FBXO25
1	146362532	146462532	0.421805	FBXO25
1	146362586	146462586	0.440558	FBXO25
1	146362607	146462607	0.440558	FBXO25
1	146362786	146462786	0.495699	FBXO25
1	146362917	146462917	0.463832	FBXO25
1	146363313	146463313	0.463832	FBXO25
1	146363323	146463323	0.455691	FBXO25
1	146363363	146463363	0.466388	FBXO25
1	146363371	146463371	0.462616	FBXO25
1	146363399	146463399	0.392404	FBXO25
1	146363602	146463602	0.397803	FBXO25
1	146363672	146463672	0.412249	FBXO25
1	146363719	146463719	0.408533	FBXO25
1	146363786	146463786	0.43597	FBXO25

1	146363838	146463838	0.436839	FBXO25
1	146363839	146463839	0.436839	FBXO25
1	146363984	146463984	0.471731	FBXO25
1	146364043	146464043	0.430131	FBXO25
1	146364051	146464051	0.413332	FBXO25
1	146364104	146464104	0.413332	FBXO25
1	146364376	146464376	0.376041	FBXO25
1	146364590	146464590	0.432539	FBXO25
1	146364677	146464677	0.465268	FBXO25
1	146364752	146464752	0.458843	FBXO25
1	146364952	146464952	0.496322	FBXO25
1	146365181	146465181	0.498317	FBXO25
1	146365314	146465314	0.471553	FBXO25
1	146365377	146465377	0.463521	FBXO25
1	146365620	146465620	0.501881	FBXO25
1	146365673	146465673	0.522957	FBXO25
1	146365814	146465814	0.538813	FBXO25
1	146365844	146465844	0.539814	FBXO25
1	146365876	146465876	0.541677	FBXO25
1	146365914	146465914	0.539541	FBXO25
1	146365952	146465952	0.546449	FBXO25
1	146365969	146465969	0.529265	FBXO25
1	146365992	146465992	0.513499	FBXO25
1	146366122	146466122	0.513499	FBXO25
1	146366124	146466124	0.510524	FBXO25
1	146366168	146466168	0.510524	FBXO25
1	146366290	146466290	0.509624	FBXO25
1	146366308	146466308	0.499998	FBXO25
1	146366524	146466524	0.462813	FBXO25
1	146366695	146466695	0.485674	FBXO25
1	146366841	146466841	0.474745	FBXO25
1	146366936	146466936	0.478	FBXO25
1	146367170	146467170	0.47622	FBXO25
1	146367184	146467184	0.476343	FBXO25
1	146367192	146467192	0.480452	FBXO25
1	146367206	146467206	0.476683	FBXO25
1	146367210	146467210	0.460939	FBXO25
1	146367236	146467236	0.460939	FBXO25
1	146367376	146467376	0.454802	FBXO25
1	146367482	146467482	0.426725	FBXO25
1	146367834	146467834	0.406389	FBXO25
1	146367873	146467873	0.431913	FBXO25
1	146367880	146467880	0.46196	FBXO25
1	146368095	146468095	0.455732	FBXO25
1	146368222	146468222	0.455608	FBXO25
1	146368232	146468232	0.460953	FBXO25
1	146368249	146468249	0.45618	FBXO25
1	146368279	146468279	0.451696	FBXO25
1	146368420	146468420	0.438793	FBXO25
1	146368613	146468613	0.405708	FBXO25
1	146368814	146468814	0.405708	FBXO25
1	146368855	146468855	0.405408	FBXO25
1	146369024	146469024	0.426224	FBXO25
1	146369117	146469117	0.442286	FBXO25
1	146369195	146469195	0.438282	FBXO25
1	146369246	146469246	0.425232	FBXO25
1	146369297	146469297	0.427622	FBXO25
1	146369413	146469413	0.414336	FBXO25

1	146369433	146469433	0.414336	FBXO25
1	146369807	146469807	0.441851	FBXO25
1	146369934	146469934	0.452416	FBXO25
1	146370045	146470045	0.454894	FBXO25
1	146370092	146470092	0.454894	FBXO25
1	146370110	146470110	0.456059	FBXO25
1	146370148	146470148	0.456059	FBXO25
1	146370350	146470350	0.441054	FBXO25
1	146370508	146470508	0.445058	FBXO25
1	146370520	146470520	0.452103	FBXO25
1	146370602	146470602	0.453727	FBXO25
1	146370669	146470669	0.461945	FBXO25
1	146370697	146470697	0.461473	FBXO25
1	146370791	146470791	0.461473	FBXO25
1	146370829	146470829	0.471787	FBXO25
1	146370838	146470838	0.470087	FBXO25
1	146370845	146470845	0.47016	FBXO25
1	146370848	146470848	0.47016	FBXO25
1	146370933	146470933	0.511558	FBXO25
1	146370941	146470941	0.509142	FBXO25
1	146371105	146471105	0.5081	FBXO25
1	146371261	146471261	0.522059	FBXO25
1	146371266	146471266	0.511616	FBXO25
1	146371328	146471328	0.517986	FBXO25
1	146371424	146471424	0.500899	FBXO25
1	146371429	146471429	0.500899	FBXO25
1	146371495	146471495	0.486908	FBXO25
1	146371529	146471529	0.458885	FBXO25
1	146371572	146471572	0.508582	FBXO25
1	146371579	146471579	0.518053	FBXO25
1	146371656	146471656	0.518142	FBXO25
1	146371884	146471884	0.516893	FBXO25
1	146371908	146471908	0.478841	FBXO25
1	146372029	146472029	0.405056	FBXO25
1	146372113	146472113	0.429874	FBXO25
1	146372280	146472280	0.41777	FBXO25
1	146372377	146472377	0.402101	FBXO25
1	146372456	146472456	0.361038	FBXO25
1	146372518	146472518	0.356415	FBXO25
1	146372526	146472526	0.393468	FBXO25
1	146373022	146473022	0.423147	FBXO25
1	146373406	146473406	0.406085	FBXO25
1	146373413	146473413	0.381056	FBXO25
1	146373561	146473561	0.350842	FBXO25
1	146373602	146473602	0.350842	FBXO25
1	146373621	146473621	0.350842	FBXO25
1	146373676	146473676	0.341799	FBXO25
1	146374259	146474259	0.367879	FBXO25
1	146374463	146474463	0.371641	FBXO25
1	146374590	146474590	0.375061	FBXO25
1	146374631	146474631	0.410852	FBXO25
1	146374801	146474801	0.409313	FBXO25
1	146374878	146474878	0.409313	FBXO25
1	146374945	146474945	0.409313	FBXO25
1	146375304	146475304	0.424536	FBXO25
1	146375331	146475331	0.41317	FBXO25
1	146375445	146475445	0.393559	FBXO25
1	146375523	146475523	0.397316	FBXO25

1	146375788	146475788	0.401317	FBXO25
1	146376044	146476044	0.409123	FBXO25
1	146376062	146476062	0.429499	FBXO25
1	146376064	146476064	0.404057	FBXO25
1	146376144	146476144	0.466246	FBXO25
1	146376241	146476241	0.431134	FBXO25
1	146376361	146476361	0.419714	FBXO25
1	146376432	146476432	0.416312	FBXO25
1	146376699	146476699	0.394796	FBXO25
1	146376702	146476702	0.37336	FBXO25
1	146376793	146476793	0.394995	FBXO25
1	146376839	146476839	0.412333	FBXO25
1	146376870	146476870	0.40763	FBXO25
1	146377261	146477261	0.39375	FBXO25
1	146377298	146477298	0.357785	FBXO25
1	146377478	146477478	0.343193	FBXO25
1	146377527	146477527	0.357695	FBXO25
1	146379599	146479599	0.357185	FBXO25
1	146385915	146485915	0.351527	FBXO25
1	146385915	146485915	0.351527	TDRP
1	146389983	146489983	0.368497	FBXO25
1	146389983	146489983	0.368497	TDRP
1	146391178	146491178	0.344861	FBXO25
1	146391178	146491178	0.344861	TDRP
1	146393090	146493090	0.391143	FBXO25
1	146393090	146493090	0.391143	TDRP
1	146393105	146493105	0.384681	FBXO25
1	146393105	146493105	0.384681	TDRP
1	146393127	146493127	0.346725	FBXO25
1	146393127	146493127	0.346725	TDRP
1	146393128	146493128	0.347634	FBXO25
1	146393128	146493128	0.347634	TDRP
1	146393310	146493310	0.357873	FBXO25
1	146393310	146493310	0.357873	TDRP
1	146393466	146493466	0.347957	FBXO25
1	146393466	146493466	0.347957	TDRP
1	146687051	146787051	0.393051	LOC102177758
1	148429188	148529188	0.369572	DOPEY2
1	148429188	148529188	0.369572	MORC3
1	148429527	148529527	0.366381	DOPEY2
1	148429527	148529527	0.366381	MORC3
1	148430019	148530019	0.370111	DOPEY2
1	148430019	148530019	0.370111	MORC3
1	148430320	148530320	0.36644	DOPEY2
1	148430320	148530320	0.36644	MORC3
1	148430323	148530323	0.365822	DOPEY2
1	148430323	148530323	0.365822	MORC3
1	148430531	148530531	0.365292	DOPEY2
1	148430531	148530531	0.365292	MORC3
1	148430532	148530532	0.368768	DOPEY2
1	148430532	148530532	0.368768	MORC3
1	148430870	148530870	0.361607	DOPEY2
1	148430870	148530870	0.361607	MORC3
1	148432113	148532113	0.349162	DOPEY2
1	148432113	148532113	0.349162	MORC3
1	148433161	148533161	0.362767	DOPEY2
1	148433161	148533161	0.362767	MORC3
1	148433336	148533336	0.362397	DOPEY2

1	148433336	148533336	0.362397	MORC3
1	148433403	148533403	0.383173	DOPEY2
1	148433403	148533403	0.383173	MORC3
1	148433405	148533405	0.385476	DOPEY2
1	148433405	148533405	0.385476	MORC3
1	148433496	148533496	0.380388	DOPEY2
1	148433496	148533496	0.380388	MORC3
1	148433789	148533789	0.378407	DOPEY2
1	148433789	148533789	0.378407	MORC3
1	148433977	148533977	0.379311	DOPEY2
1	148433977	148533977	0.379311	MORC3
1	148434212	148534212	0.3816	DOPEY2
1	148434212	148534212	0.3816	MORC3
1	148434521	148534521	0.381637	DOPEY2
1	148434521	148534521	0.381637	MORC3
1	148435325	148535325	0.38193	DOPEY2
1	148435325	148535325	0.38193	MORC3
1	148435463	148535463	0.381901	DOPEY2
1	148435463	148535463	0.381901	MORC3
1	148435464	148535464	0.376132	DOPEY2
1	148435464	148535464	0.376132	MORC3
1	148435685	148535685	0.376869	DOPEY2
1	148435685	148535685	0.376869	MORC3
1	148436064	148536064	0.376738	DOPEY2
1	148436064	148536064	0.376738	MORC3
1	148436340	148536340	0.379739	DOPEY2
1	148436340	148536340	0.379739	MORC3
1	148436706	148536706	0.377575	DOPEY2
1	148436706	148536706	0.377575	MORC3
1	148436728	148536728	0.372803	DOPEY2
1	148436728	148536728	0.372803	MORC3
1	148437562	148537562	0.364445	DOPEY2
1	148437562	148537562	0.364445	MORC3
1	148437662	148537662	0.378408	DOPEY2
1	148437662	148537662	0.378408	MORC3
1	148437688	148537688	0.377373	DOPEY2
1	148437688	148537688	0.377373	MORC3
1	148437824	148537824	0.384114	DOPEY2
1	148437824	148537824	0.384114	MORC3
1	148437868	148537868	0.384396	DOPEY2
1	148437868	148537868	0.384396	MORC3
1	148438073	148538073	0.37206	DOPEY2
1	148438073	148538073	0.37206	MORC3
1	148438398	148538398	0.367857	DOPEY2
1	148438398	148538398	0.367857	MORC3
1	148438570	148538570	0.367891	DOPEY2
1	148438570	148538570	0.367891	MORC3
1	148438690	148538690	0.385508	DOPEY2
1	148438690	148538690	0.385508	MORC3
1	148438751	148538751	0.374177	DOPEY2
1	148438751	148538751	0.374177	MORC3
1	148439079	148539079	0.372668	CHAF1B
1	148439079	148539079	0.372668	DOPEY2
1	148439079	148539079	0.372668	MORC3
1	148439724	148539724	0.371934	CHAF1B
1	148439724	148539724	0.371934	DOPEY2
1	148439724	148539724	0.371934	MORC3
1	148439847	148539847	0.36844	CHAF1B

1	148439847	148539847	0.36844	DOPEY2
1	148439847	148539847	0.36844	MORC3
1	148440216	148540216	0.367175	CHAF1B
1	148440216	148540216	0.367175	DOPEY2
1	148440216	148540216	0.367175	MORC3
1	148440441	148540441	0.359967	CHAF1B
1	148440441	148540441	0.359967	DOPEY2
1	148440441	148540441	0.359967	MORC3
1	148440554	148540554	0.355791	CHAF1B
1	148440554	148540554	0.355791	DOPEY2
1	148440554	148540554	0.355791	MORC3
1	148440741	148540741	0.36331	CHAF1B
1	148440741	148540741	0.36331	DOPEY2
1	148440741	148540741	0.36331	MORC3
1	148442544	148542544	0.36437	CHAF1B
1	148442544	148542544	0.36437	DOPEY2
1	148442544	148542544	0.36437	MORC3
1	148442910	148542910	0.374944	CHAF1B
1	148442910	148542910	0.374944	DOPEY2
1	148442910	148542910	0.374944	MORC3
1	148443391	148543391	0.367652	CHAF1B
1	148443391	148543391	0.367652	DOPEY2
1	148443391	148543391	0.367652	MORC3
1	148443629	148543629	0.363825	CHAF1B
1	148443629	148543629	0.363825	DOPEY2
1	148443629	148543629	0.363825	MORC3
1	148443817	148543817	0.392692	CHAF1B
1	148443817	148543817	0.392692	DOPEY2
1	148443817	148543817	0.392692	MORC3
1	148443888	148543888	0.381298	CHAF1B
1	148443888	148543888	0.381298	DOPEY2
1	148443888	148543888	0.381298	MORC3
1	148444274	148544274	0.383102	CHAF1B
1	148444274	148544274	0.383102	DOPEY2
1	148444274	148544274	0.383102	MORC3
1	148444492	148544492	0.367326	CHAF1B
1	148444492	148544492	0.367326	DOPEY2
1	148444492	148544492	0.367326	MORC3
1	148446178	148546178	0.343987	CHAF1B
1	148446178	148546178	0.343987	DOPEY2
1	148446178	148546178	0.343987	MORC3
1	148446194	148546194	0.343982	CHAF1B
1	148446194	148546194	0.343982	DOPEY2
1	148446194	148546194	0.343982	MORC3
1	148447837	148547837	0.345711	CHAF1B
1	148447837	148547837	0.345711	DOPEY2
1	148447837	148547837	0.345711	MORC3
1	148448367	148548367	0.346103	CHAF1B
1	148448367	148548367	0.346103	DOPEY2
1	148448367	148548367	0.346103	MORC3
1	148448562	148548562	0.388525	CHAF1B
1	148448562	148548562	0.388525	DOPEY2
1	148448562	148548562	0.388525	MORC3
1	148448873	148548873	0.375725	CHAF1B
1	148448873	148548873	0.375725	DOPEY2
1	148448873	148548873	0.375725	MORC3
1	148449111	148549111	0.379571	CHAF1B
1	148449111	148549111	0.379571	DOPEY2

1	148449111	148549111	0.379571	MORC3
1	148449257	148549257	0.38232	CHAF1B
1	148449257	148549257	0.38232	DOPEY2
1	148449257	148549257	0.38232	MORC3
1	148449277	148549277	0.397608	CHAF1B
1	148449277	148549277	0.397608	DOPEY2
1	148449277	148549277	0.397608	MORC3
1	148449326	148549326	0.376356	CHAF1B
1	148449326	148549326	0.376356	DOPEY2
1	148449326	148549326	0.376356	MORC3
1	148449675	148549675	0.368692	CHAF1B
1	148449675	148549675	0.368692	DOPEY2
1	148449675	148549675	0.368692	MORC3
1	148449676	148549676	0.375334	CHAF1B
1	148449676	148549676	0.375334	DOPEY2
1	148449676	148549676	0.375334	MORC3
1	148450111	148550111	0.350081	CHAF1B
1	148450111	148550111	0.350081	DOPEY2
1	148450111	148550111	0.350081	MORC3
1	148450694	148550694	0.37579	CHAF1B
1	148450694	148550694	0.37579	DOPEY2
1	148450694	148550694	0.37579	MORC3
1	148451198	148551198	0.373035	CHAF1B
1	148451198	148551198	0.373035	DOPEY2
1	148451198	148551198	0.373035	MORC3
1	148451277	148551277	0.375377	CHAF1B
1	148451277	148551277	0.375377	DOPEY2
1	148451277	148551277	0.375377	MORC3
1	148451376	148551376	0.360073	CHAF1B
1	148451376	148551376	0.360073	DOPEY2
1	148451376	148551376	0.360073	MORC3
1	148453521	148553521	0.371435	CHAF1B
1	148453521	148553521	0.371435	DOPEY2
1	148453521	148553521	0.371435	MORC3
1	148453662	148553662	0.373225	CHAF1B
1	148453662	148553662	0.373225	DOPEY2
1	148453662	148553662	0.373225	MORC3
1	148453672	148553672	0.37545	CHAF1B
1	148453672	148553672	0.37545	DOPEY2
1	148453672	148553672	0.37545	MORC3
1	148453843	148553843	0.371818	CHAF1B
1	148453843	148553843	0.371818	DOPEY2
1	148453843	148553843	0.371818	MORC3
1	148454093	148554093	0.364358	CHAF1B
1	148454093	148554093	0.364358	DOPEY2
1	148454093	148554093	0.364358	MORC3
1	148454487	148554487	0.362652	CHAF1B
1	148454487	148554487	0.362652	DOPEY2
1	148454487	148554487	0.362652	MORC3
1	148454546	148554546	0.362652	CHAF1B
1	148454546	148554546	0.362652	DOPEY2
1	148454546	148554546	0.362652	MORC3
1	148454823	148554823	0.362788	CHAF1B
1	148454823	148554823	0.362788	DOPEY2
1	148454823	148554823	0.362788	MORC3
1	148454936	148554936	0.362358	CHAF1B
1	148454936	148554936	0.362358	DOPEY2
1	148454936	148554936	0.362358	MORC3

1	148456310	148556310	0.362358	CHAF1B
1	148456310	148556310	0.362358	DOPEY2
1	148456310	148556310	0.362358	MORC3
1	148456717	148556717	0.370202	CHAF1B
1	148456717	148556717	0.370202	DOPEY2
1	148456717	148556717	0.370202	MORC3
1	148457070	148557070	0.369725	CHAF1B
1	148457070	148557070	0.369725	DOPEY2
1	148457070	148557070	0.369725	MORC3
1	148457079	148557079	0.366418	CHAF1B
1	148457079	148557079	0.366418	DOPEY2
1	148457079	148557079	0.366418	MORC3
1	148457163	148557163	0.3675	CHAF1B
1	148457163	148557163	0.3675	DOPEY2
1	148457163	148557163	0.3675	MORC3
1	148457268	148557268	0.372631	CHAF1B
1	148457268	148557268	0.372631	DOPEY2
1	148457268	148557268	0.372631	MORC3
1	148457546	148557546	0.366173	CHAF1B
1	148457546	148557546	0.366173	DOPEY2
1	148457546	148557546	0.366173	MORC3
1	148457856	148557856	0.383737	CHAF1B
1	148457856	148557856	0.383737	DOPEY2
1	148457856	148557856	0.383737	MORC3
1	148458521	148558521	0.393054	CHAF1B
1	148458521	148558521	0.393054	DOPEY2
1	148458521	148558521	0.393054	MORC3
1	148458679	148558679	0.385952	CHAF1B
1	148458679	148558679	0.385952	DOPEY2
1	148458679	148558679	0.385952	MORC3
1	148459290	148559290	0.388067	CHAF1B
1	148459290	148559290	0.388067	DOPEY2
1	148459290	148559290	0.388067	MORC3
1	148459569	148559569	0.375607	CHAF1B
1	148459569	148559569	0.375607	DOPEY2
1	148459569	148559569	0.375607	MORC3
1	148460269	148560269	0.373195	CHAF1B
1	148460269	148560269	0.373195	DOPEY2
1	148460269	148560269	0.373195	MORC3
1	148460438	148560438	0.351697	CHAF1B
1	148460438	148560438	0.351697	DOPEY2
1	148460438	148560438	0.351697	MORC3
1	148460493	148560493	0.344813	CHAF1B
1	148460493	148560493	0.344813	DOPEY2
1	148460493	148560493	0.344813	MORC3
1	148460895	148560895	0.345172	CHAF1B
1	148460895	148560895	0.345172	DOPEY2
1	148460895	148560895	0.345172	MORC3
1	148461080	148561080	0.351358	CHAF1B
1	148461080	148561080	0.351358	DOPEY2
1	148461080	148561080	0.351358	MORC3
1	148461506	148561506	0.344886	CHAF1B
1	148461506	148561506	0.344886	DOPEY2
1	148461506	148561506	0.344886	MORC3
1	148461948	148561948	0.354831	CHAF1B
1	148461948	148561948	0.354831	DOPEY2
1	148461948	148561948	0.354831	MORC3
1	148462064	148562064	0.348213	CHAF1B

1	148462064	148562064	0.348213	DOPEY2
1	148462064	148562064	0.348213	MORC3
1	148462303	148562303	0.348201	CHAF1B
1	148462303	148562303	0.348201	DOPEY2
1	148462303	148562303	0.348201	MORC3
1	148462887	148562887	0.34705	CHAF1B
1	148462887	148562887	0.34705	DOPEY2
1	148462887	148562887	0.34705	MORC3
1	148463982	148563982	0.34562	CHAF1B
1	148463982	148563982	0.34562	DOPEY2
1	148463982	148563982	0.34562	MORC3
1	148464180	148564180	0.348234	CHAF1B
1	148464180	148564180	0.348234	DOPEY2
1	148464180	148564180	0.348234	MORC3
1	148464355	148564355	0.350618	CHAF1B
1	148464355	148564355	0.350618	DOPEY2
1	148464355	148564355	0.350618	MORC3
1	148470085	148570085	0.341962	CHAF1B
1	148470085	148570085	0.341962	DOPEY2
1	148470085	148570085	0.341962	MORC3
1	148471837	148571837	0.350049	CHAF1B
1	148471837	148571837	0.350049	DOPEY2
1	148471837	148571837	0.350049	MORC3
1	149543511	149643511	0.344231	DYRK1A
1	150702805	150802805	0.351631	TRNAV-CAC
1	150702805	150802805	0.351631	ERG
1	150702812	150802812	0.369015	TRNAV-CAC
1	150702812	150802812	0.369015	ERG
1	150702839	150802839	0.354034	TRNAV-CAC
1	150702839	150802839	0.354034	ERG
1	150702851	150802851	0.354034	TRNAV-CAC
1	150702851	150802851	0.354034	ERG
1	150702900	150802900	0.354164	TRNAV-CAC
1	150702900	150802900	0.354164	ERG
1	150703072	150803072	0.362004	TRNAV-CAC
1	150703072	150803072	0.362004	ERG
1	150703207	150803207	0.367987	TRNAV-CAC
1	150703207	150803207	0.367987	ERG
1	150703211	150803211	0.360171	TRNAV-CAC
1	150703211	150803211	0.360171	ERG
1	150703399	150803399	0.3592	TRNAV-CAC
1	150703399	150803399	0.3592	ERG
1	150703671	150803671	0.365005	TRNAV-CAC
1	150703671	150803671	0.365005	ERG
1	150703739	150803739	0.343388	TRNAV-CAC
1	150703739	150803739	0.343388	ERG
1	150704727	150804727	0.351415	TRNAV-CAC
1	150704727	150804727	0.351415	ERG
1	150704743	150804743	0.342648	TRNAV-CAC
1	150704743	150804743	0.342648	ERG
1	150704819	150804819	0.358516	TRNAV-CAC
1	150704819	150804819	0.358516	ERG
1	150705159	150805159	0.363937	TRNAV-CAC
1	150705159	150805159	0.363937	ERG
1	150705166	150805166	0.364194	TRNAV-CAC
1	150705166	150805166	0.364194	ERG
1	150705212	150805212	0.358077	TRNAV-CAC
1	150705212	150805212	0.358077	ERG

1	150705373	150805373	0.345298	TRNAV-CAC
1	150705373	150805373	0.345298	ERG
1	150705526	150805526	0.346222	TRNAV-CAC
1	150705526	150805526	0.346222	ERG
1	150705546	150805546	0.34864	TRNAV-CAC
1	150705546	150805546	0.34864	ERG
1	150705585	150805585	0.347954	TRNAV-CAC
1	150705585	150805585	0.347954	ERG
1	150705741	150805741	0.379458	TRNAV-CAC
1	150705741	150805741	0.379458	ERG
1	150705759	150805759	0.379347	TRNAV-CAC
1	150705759	150805759	0.379347	ERG
1	150705898	150805898	0.379347	TRNAV-CAC
1	150705898	150805898	0.379347	ERG
1	150705948	150805948	0.379347	TRNAV-CAC
1	150705948	150805948	0.379347	ERG
1	150705953	150805953	0.379347	TRNAV-CAC
1	150705953	150805953	0.379347	ERG
1	150706004	150806004	0.379347	TRNAV-CAC
1	150706004	150806004	0.379347	ERG
1	150706037	150806037	0.378338	TRNAV-CAC
1	150706037	150806037	0.378338	ERG
1	150706038	150806038	0.377732	TRNAV-CAC
1	150706038	150806038	0.377732	ERG
1	150706039	150806039	0.377732	TRNAV-CAC
1	150706039	150806039	0.377732	ERG
1	150706052	150806052	0.377732	TRNAV-CAC
1	150706052	150806052	0.377732	ERG
1	150706087	150806087	0.377732	TRNAV-CAC
1	150706087	150806087	0.377732	ERG
1	150706098	150806098	0.377732	TRNAV-CAC
1	150706098	150806098	0.377732	ERG
1	150706130	150806130	0.377732	TRNAV-CAC
1	150706130	150806130	0.377732	ERG
1	150706135	150806135	0.376229	TRNAV-CAC
1	150706135	150806135	0.376229	ERG
1	150706143	150806143	0.380912	TRNAV-CAC
1	150706143	150806143	0.380912	ERG
1	150706145	150806145	0.372318	TRNAV-CAC
1	150706145	150806145	0.372318	ERG
1	150706327	150806327	0.372318	TRNAV-CAC
1	150706327	150806327	0.372318	ERG
1	150706365	150806365	0.380424	TRNAV-CAC
1	150706365	150806365	0.380424	ERG
1	150706494	150806494	0.355705	TRNAV-CAC
1	150706494	150806494	0.355705	ERG
1	150706513	150806513	0.357755	TRNAV-CAC
1	150706513	150806513	0.357755	ERG
1	150706738	150806738	0.372208	TRNAV-CAC
1	150706738	150806738	0.372208	ERG
1	150706756	150806756	0.372065	TRNAV-CAC
1	150706756	150806756	0.372065	ERG
1	150706808	150806808	0.346014	TRNAV-CAC
1	150706808	150806808	0.346014	ERG
1	150707018	150807018	0.35475	TRNAV-CAC
1	150707018	150807018	0.35475	ERG
1	150707844	150807844	0.34619	TRNAV-CAC
1	150707844	150807844	0.34619	ERG

1	150707898	150807898	0.344258	TRNAV-CAC
1	150707898	150807898	0.344258	ERG
1	150707928	150807928	0.363577	TRNAV-CAC
1	150707928	150807928	0.363577	ERG
1	150708294	150808294	0.365493	TRNAV-CAC
1	150708294	150808294	0.365493	ERG
1	150708304	150808304	0.368436	TRNAV-CAC
1	150708304	150808304	0.368436	ERG
1	150708430	150808430	0.36806	TRNAV-CAC
1	150708430	150808430	0.36806	ERG
1	150708597	150808597	0.344658	TRNAV-CAC
1	150708597	150808597	0.344658	ERG
1	150708618	150808618	0.347659	TRNAV-CAC
1	150708618	150808618	0.347659	ERG
1	150708625	150808625	0.365189	TRNAV-CAC
1	150708625	150808625	0.365189	ERG
1	150708626	150808626	0.373201	TRNAV-CAC
1	150708626	150808626	0.373201	ERG
1	150708789	150808789	0.344492	TRNAV-CAC
1	150708789	150808789	0.344492	ERG
1	150709166	150809166	0.344492	TRNAV-CAC
1	150709166	150809166	0.344492	ERG
1	150709316	150809316	0.342174	TRNAV-CAC
1	150709316	150809316	0.342174	ERG
1	150709323	150809323	0.343838	TRNAV-CAC
1	150709323	150809323	0.343838	ERG
1	150711134	150811134	0.350014	TRNAV-CAC
1	150711134	150811134	0.350014	ERG
1	150758190	150858190	0.342114	TRNAV-CAC
1	150758190	150858190	0.342114	ERG
1	150758594	150858594	0.356266	TRNAV-CAC
1	150758594	150858594	0.356266	ERG
1	150758639	150858639	0.353232	TRNAV-CAC
1	150758639	150858639	0.353232	ERG
1	150758758	150858758	0.362828	TRNAV-CAC
1	150758758	150858758	0.362828	ERG
1	150758812	150858812	0.384236	TRNAV-CAC
1	150758812	150858812	0.384236	ERG
1	150758903	150858903	0.386934	TRNAV-CAC
1	150758903	150858903	0.386934	ERG
1	150759241	150859241	0.373066	TRNAV-CAC
1	150759241	150859241	0.373066	ERG
1	150759295	150859295	0.373066	TRNAV-CAC
1	150759295	150859295	0.373066	ERG
1	150759319	150859319	0.373066	TRNAV-CAC
1	150759319	150859319	0.373066	ERG
1	150759323	150859323	0.373066	TRNAV-CAC
1	150759323	150859323	0.373066	ERG
1	150759451	150859451	0.373236	TRNAV-CAC
1	150759451	150859451	0.373236	ERG
1	150759467	150859467	0.373236	TRNAV-CAC
1	150759467	150859467	0.373236	ERG
1	150759684	150859684	0.343323	TRNAV-CAC
1	150759684	150859684	0.343323	ERG
1	150759960	150859960	0.351384	TRNAV-CAC
1	150759960	150859960	0.351384	ERG
1	150759965	150859965	0.346139	TRNAV-CAC
1	150759965	150859965	0.346139	ERG

1	150760058	150860058	0.345993	TRNAV-CAC
1	150760058	150860058	0.345993	ERG
1	150760087	150860087	0.361563	TRNAV-CAC
1	150760087	150860087	0.361563	ERG
1	150760094	150860094	0.358117	TRNAV-CAC
1	150760094	150860094	0.358117	ERG
1	150760385	150860385	0.34527	TRNAV-CAC
1	150760385	150860385	0.34527	ERG
1	150765162	150865162	0.343557	TRNAV-CAC
1	150765162	150865162	0.343557	ERG
1	150765221	150865221	0.343557	TRNAV-CAC
1	150765221	150865221	0.343557	ERG
1	150767812	150867812	0.349998	TRNAV-CAC
1	150767812	150867812	0.349998	ERG
1	150767913	150867913	0.354873	TRNAV-CAC
1	150767913	150867913	0.354873	ERG
1	150960196	151060196	0.347108	ETS2
1	150961142	151061142	0.352677	ETS2
1	150961303	151061303	0.373636	ETS2
1	150963462	151063462	0.352212	ETS2
1	150963578	151063578	0.402534	ETS2
1	150963642	151063642	0.399571	ETS2
1	150963740	151063740	0.414573	ETS2
1	150964093	151064093	0.391586	ETS2
1	150964140	151064140	0.380025	ETS2
1	150964194	151064194	0.38536	ETS2
1	150964428	151064428	0.38536	ETS2
1	150964440	151064440	0.38536	ETS2
1	150964815	151064815	0.374219	ETS2
1	150965158	151065158	0.440503	ETS2
1	150965170	151065170	0.4653	ETS2
1	150965277	151065277	0.445561	ETS2
1	150965357	151065357	0.447802	ETS2
1	150965395	151065395	0.40837	ETS2
1	150965532	151065532	0.415383	ETS2
1	150965537	151065537	0.44861	ETS2
1	150965699	151065699	0.46018	ETS2
1	150965748	151065748	0.462066	ETS2
1	150965753	151065753	0.462251	ETS2
1	150965793	151065793	0.466449	ETS2
1	150965827	151065827	0.46869	ETS2
1	150965828	151065828	0.462554	ETS2
1	150965837	151065837	0.470978	ETS2
1	150966051	151066051	0.470978	ETS2
1	150966061	151066061	0.479629	ETS2
1	150966148	151066148	0.478506	ETS2
1	150966253	151066253	0.478069	ETS2
1	150966268	151066268	0.465163	ETS2
1	150966272	151066272	0.458318	ETS2
1	150966277	151066277	0.442194	ETS2
1	150966396	151066396	0.425354	ETS2
1	150966400	151066400	0.441894	ETS2
1	150966511	151066511	0.432059	ETS2
1	150966605	151066605	0.448205	ETS2
1	150966691	151066691	0.465158	ETS2
1	150966771	151066771	0.465158	ETS2
1	150966948	151066948	0.465158	ETS2
1	150966956	151066956	0.465158	ETS2

1	150967123	151067123	0.462373	ETS2
1	150967185	151067185	0.442777	ETS2
1	150967286	151067286	0.448213	ETS2
1	150967290	151067290	0.45042	ETS2
1	150967321	151067321	0.436713	ETS2
1	150967489	151067489	0.38837	ETS2
1	150967502	151067502	0.38837	ETS2
1	150967527	151067527	0.431307	ETS2
1	150967572	151067572	0.425245	ETS2
1	150967840	151067840	0.44311	ETS2
1	150967907	151067907	0.43452	ETS2
1	150968469	151068469	0.431131	ETS2
1	150968572	151068572	0.429792	ETS2
1	150968580	151068580	0.455197	ETS2
1	150968626	151068626	0.436173	ETS2
1	150968627	151068627	0.436173	ETS2
1	150968772	151068772	0.44063	ETS2
1	150968919	151068919	0.449711	ETS2
1	150968920	151068920	0.432494	ETS2
1	150969007	151069007	0.433116	ETS2
1	150969016	151069016	0.433116	ETS2
1	150969075	151069075	0.433116	ETS2
1	150969122	151069122	0.433162	ETS2
1	150969367	151069367	0.433312	ETS2
1	150969369	151069369	0.434107	ETS2
1	150969453	151069453	0.434894	ETS2
1	150969498	151069498	0.42159	ETS2
1	150969501	151069501	0.410901	ETS2
1	150969515	151069515	0.423416	ETS2
1	150969530	151069530	0.42387	ETS2
1	150969600	151069600	0.439737	ETS2
1	150969609	151069609	0.432474	ETS2
1	150969627	151069627	0.425928	ETS2
1	150969914	151069914	0.428547	ETS2
1	150969977	151069977	0.430389	ETS2
1	150970025	151070025	0.408609	ETS2
1	150970094	151070094	0.395449	ETS2
1	150970548	151070548	0.39317	ETS2
1	150970560	151070560	0.409136	ETS2
1	150970628	151070628	0.409136	ETS2
1	150970631	151070631	0.422186	ETS2
1	150970633	151070633	0.423047	ETS2
1	150970647	151070647	0.401899	ETS2
1	150970873	151070873	0.401899	ETS2
1	150970888	151070888	0.401899	ETS2
1	150970985	151070985	0.401905	ETS2
1	150971159	151071159	0.401905	ETS2
1	150971306	151071306	0.402791	ETS2
1	150971311	151071311	0.409119	ETS2
1	150971383	151071383	0.401744	ETS2
1	150971433	151071433	0.365952	ETS2
1	150971532	151071532	0.365952	ETS2
1	150971599	151071599	0.365952	ETS2
1	150971647	151071647	0.365952	ETS2
1	150971665	151071665	0.358891	ETS2
1	150971708	151071708	0.375295	ETS2
1	150971787	151071787	0.367493	ETS2
1	150971812	151071812	0.371326	ETS2

1	150971888	151071888	0.3994	ETS2
1	150971907	151071907	0.378736	ETS2
1	150972106	151072106	0.378736	ETS2
1	150972284	151072284	0.381249	ETS2
1	150972360	151072360	0.385903	ETS2
1	150972475	151072475	0.385903	ETS2
1	150972682	151072682	0.408948	ETS2
1	150972757	151072757	0.405809	ETS2
1	150972780	151072780	0.410094	ETS2
1	150972809	151072809	0.410094	ETS2
1	150972935	151072935	0.410094	ETS2
1	150972989	151072989	0.410094	ETS2
1	150973035	151073035	0.439408	ETS2
1	150973058	151073058	0.434616	ETS2
1	150973067	151073067	0.434616	ETS2
1	150973159	151073159	0.437389	ETS2
1	150973204	151073204	0.427681	ETS2
1	150973286	151073286	0.430106	ETS2
1	150973300	151073300	0.429483	ETS2
1	150973302	151073302	0.437728	ETS2
1	150973402	151073402	0.430166	ETS2
1	150973488	151073488	0.40544	ETS2
1	150973496	151073496	0.404854	ETS2
1	150973501	151073501	0.40972	ETS2
1	150973539	151073539	0.42165	ETS2
1	150973562	151073562	0.438461	ETS2
1	150973601	151073601	0.431632	ETS2
1	150973608	151073608	0.431894	ETS2
1	150973631	151073631	0.418818	ETS2
1	150973649	151073649	0.406476	ETS2
1	150973687	151073687	0.415091	ETS2
1	150973699	151073699	0.409129	ETS2
1	150973811	151073811	0.41934	ETS2
1	150973840	151073840	0.423056	ETS2
1	150973925	151073925	0.438907	ETS2
1	150973954	151073954	0.408552	ETS2
1	150974067	151074067	0.378709	ETS2
1	150974249	151074249	0.378709	ETS2
1	150974293	151074293	0.378709	ETS2
1	150974313	151074313	0.378709	ETS2
1	150974329	151074329	0.378709	ETS2
1	150974386	151074386	0.378668	ETS2
1	150974394	151074394	0.39266	ETS2
1	150974559	151074559	0.39266	ETS2
1	150974820	151074820	0.42225	ETS2
1	150974821	151074821	0.42225	ETS2
1	150974836	151074836	0.444878	ETS2
1	150974895	151074895	0.445424	ETS2
1	150974960	151074960	0.45318	ETS2
1	150974971	151074971	0.442997	ETS2
1	150975244	151075244	0.403243	ETS2
1	150975298	151075298	0.398487	ETS2
1	150975303	151075303	0.398487	ETS2
1	150975329	151075329	0.398487	ETS2
1	150975334	151075334	0.398487	ETS2
1	150975687	151075687	0.404268	ETS2
1	150975695	151075695	0.406081	ETS2
1	150976149	151076149	0.390358	ETS2

1	150976205	151076205	0.409397	ETS2
1	150976245	151076245	0.406886	ETS2
1	150976313	151076313	0.400368	ETS2
1	150976314	151076314	0.37864	ETS2
1	150976921	151076921	0.37864	ETS2
1	150977029	151077029	0.387658	ETS2
1	150977314	151077314	0.358944	ETS2
1	150977509	151077509	0.360618	ETS2
1	150978104	151078104	0.388192	ETS2
1	150978530	151078530	0.366913	ETS2
1	150978560	151078560	0.370828	ETS2
1	150978561	151078561	0.370828	ETS2
1	150979032	151079032	0.37021	ETS2
1	150979116	151079116	0.392727	ETS2
1	150979158	151079158	0.407326	ETS2
1	150979174	151079174	0.407442	ETS2
1	150979431	151079431	0.401181	ETS2
1	150979548	151079548	0.382476	ETS2
1	150979554	151079554	0.40488	ETS2
1	150979980	151079980	0.398636	ETS2
1	150979987	151079987	0.420062	ETS2
1	150980065	151080065	0.407339	ETS2
1	150980202	151080202	0.411988	ETS2
1	150980289	151080289	0.388947	ETS2
1	150980290	151080290	0.388947	ETS2
1	150980330	151080330	0.388947	ETS2
1	150980331	151080331	0.388947	ETS2
1	150980416	151080416	0.388947	ETS2
1	150980638	151080638	0.386291	ETS2
1	150980678	151080678	0.409556	ETS2
1	150980729	151080729	0.43604	ETS2
1	150980759	151080759	0.448124	ETS2
1	150980815	151080815	0.4442	ETS2
1	150980900	151080900	0.436661	ETS2
1	150980949	151080949	0.431929	ETS2
1	150980971	151080971	0.408651	ETS2
1	150981198	151081198	0.38626	ETS2
1	150981573	151081573	0.376275	ETS2
1	150981750	151081750	0.39756	ETS2
1	150981764	151081764	0.411673	ETS2
1	150981796	151081796	0.408011	ETS2
1	150981816	151081816	0.408011	ETS2
1	150981822	151081822	0.408011	ETS2
1	150981859	151081859	0.408011	ETS2
1	150981895	151081895	0.408011	ETS2
1	150981965	151081965	0.408011	ETS2
1	150982057	151082057	0.408011	ETS2
1	150982112	151082112	0.392871	ETS2
1	150982116	151082116	0.399301	ETS2
1	150982122	151082122	0.396297	ETS2
1	150982126	151082126	0.402183	ETS2
1	150982149	151082149	0.399379	ETS2
1	150982196	151082196	0.380349	ETS2
1	150982733	151082733	0.377076	ETS2
1	150982882	151082882	0.386155	ETS2
1	150982921	151082921	0.402136	ETS2
1	150982955	151082955	0.386491	ETS2
1	150983158	151083158	0.386955	ETS2

1	150983186	151083186	0.388984	ETS2
1	150983259	151083259	0.388026	ETS2
1	150983403	151083403	0.385147	ETS2
1	150983447	151083447	0.384454	ETS2
1	150983460	151083460	0.392568	ETS2
1	150983541	151083541	0.39814	ETS2
1	150983560	151083560	0.392515	ETS2
1	150983920	151083920	0.413266	ETS2
1	150983925	151083925	0.435088	ETS2
1	150983975	151083975	0.447901	ETS2
1	150984142	151084142	0.411523	ETS2
1	150984187	151084187	0.400783	ETS2
1	150984493	151084493	0.380938	ETS2
1	150984623	151084623	0.371004	ETS2
1	150984689	151084689	0.386606	ETS2
1	150984824	151084824	0.405941	ETS2
1	150984851	151084851	0.389852	ETS2
1	150985245	151085245	0.380999	ETS2
1	150985558	151085558	0.397707	ETS2
1	150985602	151085602	0.397719	ETS2
1	150985687	151085687	0.414703	ETS2
1	150985925	151085925	0.442816	ETS2
1	150985956	151085956	0.428139	ETS2
1	150986066	151086066	0.424666	ETS2
1	150986104	151086104	0.424093	ETS2
1	150986122	151086122	0.398155	ETS2
1	150986214	151086214	0.398155	ETS2
1	150986257	151086257	0.398155	ETS2
1	150986288	151086288	0.398155	ETS2
1	150986504	151086504	0.378431	ETS2
1	150986685	151086685	0.407302	ETS2
1	150986779	151086779	0.407302	ETS2
1	150986839	151086839	0.407302	ETS2
1	150987024	151087024	0.421546	ETS2
1	150987035	151087035	0.417825	ETS2
1	150987117	151087117	0.411232	ETS2
1	150987147	151087147	0.411232	ETS2
1	150987326	151087326	0.411232	ETS2
1	150987332	151087332	0.4311	ETS2
1	150987385	151087385	0.40662	ETS2
1	150987514	151087514	0.397986	ETS2
1	150987592	151087592	0.389021	ETS2
1	150987636	151087636	0.41257	ETS2
1	150987690	151087690	0.41257	ETS2
1	150987779	151087779	0.422486	ETS2
1	150987805	151087805	0.422486	ETS2
1	150987810	151087810	0.42173	ETS2
1	150987812	151087812	0.398024	ETS2
1	150988112	151088112	0.373619	ETS2
1	150988492	151088492	0.359073	ETS2
1	152445021	152545021	0.345123	BTD
1	152445021	152545021	0.345123	HACL1
1	152445327	152545327	0.347246	BTD
1	152445327	152545327	0.347246	HACL1
1	152445378	152545378	0.344617	BTD
1	152445378	152545378	0.344617	HACL1
1	152445402	152545402	0.344617	BTD
1	152445402	152545402	0.344617	HACL1

1	153678764	153778764	0.34485	PLCL2
1	153678780	153778780	0.348304	PLCL2
1	153678788	153778788	0.353194	PLCL2
1	153703751	153803751	0.354184	PLCL2
1	153703752	153803752	0.354184	PLCL2
1	156623904	156723904	0.357958	KCNH8
1	156623946	156723946	0.351776	KCNH8
1	156876739	156976739	0.348295	EFHB
1	156881007	156981007	0.405367	EFHB
1	156881025	156981025	0.421048	EFHB
1	156881074	156981074	0.420353	EFHB
1	156881090	156981090	0.397667	EFHB
1	156881207	156981207	0.34765	EFHB
1	156881582	156981582	0.34202	EFHB
1	156885155	156985155	0.371302	EFHB
1	156885296	156985296	0.371302	EFHB
1	156885357	156985357	0.369341	EFHB
1	156885370	156985370	0.369341	EFHB
1	156885411	156985411	0.37733	EFHB
1	156885472	156985472	0.363578	EFHB
1	156885582	156985582	0.391787	EFHB
1	156885653	156985653	0.430729	EFHB
1	156885674	156985674	0.497608	EFHB
1	156885787	156985787	0.517213	EFHB
1	156885807	156985807	0.518421	EFHB
1	156886086	156986086	0.525644	EFHB
1	156886126	156986126	0.512484	EFHB
1	156886169	156986169	0.508921	EFHB
1	156886206	156986206	0.508921	EFHB
1	156886226	156986226	0.499922	EFHB
1	156886321	156986321	0.483763	EFHB
1	156886340	156986340	0.483763	EFHB
1	156886342	156986342	0.48181	EFHB
1	156886366	156986366	0.48181	EFHB
1	156886394	156986394	0.373334	EFHB
1	156886395	156986395	0.373334	EFHB
1	156886425	156986425	0.362584	EFHB
1	156886429	156986429	0.363462	EFHB
1	156886435	156986435	0.363462	EFHB
1	156886804	156986804	0.343978	EFHB
1	156886827	156986827	0.360886	EFHB
1	156886964	156986964	0.360886	EFHB
1	156886981	156986981	0.365124	EFHB
1	156887028	156987028	0.420661	EFHB
1	156887058	156987058	0.439862	EFHB
1	156887062	156987062	0.40207	EFHB
1	156887199	156987199	0.376768	EFHB
1	156887517	156987517	0.400564	EFHB
1	156887576	156987576	0.429916	EFHB
1	156887664	156987664	0.476177	EFHB
1	156887722	156987722	0.422357	EFHB
1	156887820	156987820	0.389638	EFHB
1	156889640	156989640	0.382867	EFHB
1	156889846	156989846	0.382375	EFHB
1	156890409	156990409	0.344665	EFHB
1	156890460	156990460	0.352122	EFHB
1	156890576	156990576	0.37577	EFHB
1	156895417	156995417	0.376046	EFHB

1	156895428	156995428	0.413636	EFHB
1	156895468	156995468	0.443404	EFHB
1	156895478	156995478	0.446593	EFHB
1	156895485	156995485	0.414415	EFHB
1	156895488	156995488	0.412229	EFHB
1	156895562	156995562	0.415911	EFHB
1	156895579	156995579	0.466669	EFHB
1	156895780	156995780	0.481696	EFHB
1	156895787	156995787	0.505708	EFHB
1	156895788	156995788	0.505708	EFHB
1	156895845	156995845	0.529503	EFHB
1	156895885	156995885	0.540217	EFHB
1	156895890	156995890	0.542933	EFHB
1	156895921	156995921	0.537475	EFHB
1	156895922	156995922	0.537475	EFHB
1	156895947	156995947	0.541647	EFHB
1	156895958	156995958	0.537633	EFHB
1	156895961	156995961	0.536953	EFHB
1	156895973	156995973	0.508452	EFHB
1	156895990	156995990	0.455435	EFHB
1	156897353	156997353	0.346075	EFHB
1	156925606	157025606	0.378159	EFHB
1	156925606	157025606	0.378159	RAB5A
1	156925609	157025609	0.380551	EFHB
1	156925609	157025609	0.380551	RAB5A
1	156925839	157025839	0.354711	EFHB
1	156925839	157025839	0.354711	RAB5A
1	156925892	157025892	0.348373	EFHB
1	156925892	157025892	0.348373	RAB5A
1	156925988	157025988	0.364123	EFHB
1	156925988	157025988	0.364123	RAB5A
1	156926064	157026064	0.361675	EFHB
1	156926064	157026064	0.361675	RAB5A
1	156926065	157026065	0.361675	EFHB
1	156926065	157026065	0.361675	RAB5A
1	156926190	157026190	0.361501	EFHB
1	156926190	157026190	0.361501	RAB5A
1	156927758	157027758	0.352079	EFHB
1	156927758	157027758	0.352079	RAB5A
1	156927844	157027844	0.349566	EFHB
1	156927844	157027844	0.349566	RAB5A
1	156927875	157027875	0.349025	EFHB
1	156927875	157027875	0.349025	RAB5A
1	156980862	157080862	0.342993	EFHB
1	156980862	157080862	0.342993	PP2D1
1	156980862	157080862	0.342993	RAB5A
1	156984794	157084794	0.345686	EFHB
1	156984794	157084794	0.345686	PP2D1
1	156984794	157084794	0.345686	RAB5A
1	156984795	157084795	0.346971	EFHB
1	156984795	157084795	0.346971	PP2D1
1	156984795	157084795	0.346971	RAB5A
1	156984854	157084854	0.359146	EFHB
1	156984854	157084854	0.359146	PP2D1
1	156984854	157084854	0.359146	RAB5A
1	157031432	157131432	0.366	PP2D1
1	157031432	157131432	0.366	RAB5A
1	157031432	157131432	0.366	KAT2B

1	157031896	157131896	0.371112	PP2D1
1	157031896	157131896	0.371112	RAB5A
1	157031896	157131896	0.371112	KAT2B
1	157031979	157131979	0.392863	PP2D1
1	157031979	157131979	0.392863	RAB5A
1	157031979	157131979	0.392863	KAT2B
1	157032715	157132715	0.401819	PP2D1
1	157032715	157132715	0.401819	RAB5A
1	157032715	157132715	0.401819	KAT2B
1	157032716	157132716	0.427782	PP2D1
1	157032716	157132716	0.427782	RAB5A
1	157032716	157132716	0.427782	KAT2B
1	157032728	157132728	0.425359	PP2D1
1	157032728	157132728	0.425359	RAB5A
1	157032728	157132728	0.425359	KAT2B
1	157033333	157133333	0.415784	PP2D1
1	157033333	157133333	0.415784	RAB5A
1	157033333	157133333	0.415784	KAT2B
1	157033521	157133521	0.460711	PP2D1
1	157033521	157133521	0.460711	RAB5A
1	157033521	157133521	0.460711	KAT2B
1	157033897	157133897	0.454958	PP2D1
1	157033897	157133897	0.454958	RAB5A
1	157033897	157133897	0.454958	KAT2B
1	157034805	157134805	0.410615	PP2D1
1	157034805	157134805	0.410615	RAB5A
1	157034805	157134805	0.410615	KAT2B
1	157034922	157134922	0.410615	PP2D1
1	157034922	157134922	0.410615	RAB5A
1	157034922	157134922	0.410615	KAT2B
1	157036873	157136873	0.439218	PP2D1
1	157036873	157136873	0.439218	RAB5A
1	157036873	157136873	0.439218	KAT2B
1	157037257	157137257	0.354675	PP2D1
1	157037257	157137257	0.354675	RAB5A
1	157037257	157137257	0.354675	KAT2B
1	157037588	157137588	0.349568	PP2D1
1	157037588	157137588	0.349568	RAB5A
1	157037588	157137588	0.349568	KAT2B
1	157038510	157138510	0.349568	PP2D1
1	157038510	157138510	0.349568	RAB5A
1	157038510	157138510	0.349568	KAT2B
1	157039196	157139196	0.401627	PP2D1
1	157039196	157139196	0.401627	RAB5A
1	157039196	157139196	0.401627	KAT2B
1	157039310	157139310	0.401627	PP2D1
1	157039310	157139310	0.401627	RAB5A
1	157039310	157139310	0.401627	KAT2B
1	157039337	157139337	0.390857	PP2D1
1	157039337	157139337	0.390857	RAB5A
1	157039337	157139337	0.390857	KAT2B
1	157039763	157139763	0.390857	PP2D1
1	157039763	157139763	0.390857	RAB5A
1	157039763	157139763	0.390857	KAT2B
1	157039820	157139820	0.390857	PP2D1
1	157039820	157139820	0.390857	RAB5A
1	157039820	157139820	0.390857	KAT2B
1	157040010	157140010	0.390857	PP2D1

1	157040010	157140010	0.390857	RAB5A
1	157040010	157140010	0.390857	KAT2B
1	157040634	157140634	0.38994	PP2D1
1	157040634	157140634	0.38994	RAB5A
1	157040634	157140634	0.38994	KAT2B
1	157041180	157141180	0.407736	PP2D1
1	157041180	157141180	0.407736	RAB5A
1	157041180	157141180	0.407736	KAT2B
1	157041205	157141205	0.406864	PP2D1
1	157041205	157141205	0.406864	RAB5A
1	157041205	157141205	0.406864	KAT2B
1	157041444	157141444	0.421463	PP2D1
1	157041444	157141444	0.421463	KAT2B
1	157041558	157141558	0.405507	PP2D1
1	157041558	157141558	0.405507	KAT2B
1	157041635	157141635	0.434362	PP2D1
1	157041635	157141635	0.434362	KAT2B
1	157041734	157141734	0.379058	PP2D1
1	157041734	157141734	0.379058	KAT2B
1	157042111	157142111	0.352389	PP2D1
1	157042111	157142111	0.352389	KAT2B
1	157042218	157142218	0.372807	PP2D1
1	157042218	157142218	0.372807	KAT2B
1	157046202	157146202	0.344033	PP2D1
1	157046202	157146202	0.344033	KAT2B
1	157292362	157392362	0.357942	LOC102184668
1	157292364	157392364	0.357942	LOC102184668
2	219123	319123	0.347113	LOC108637009
2	219123	319123	0.347113	RSG1
2	219123	319123	0.347113	ARHGEF19
2	219123	319123	0.347113	FBXO42
2	1100075	1200075	0.36614	LOC102173345
2	1100075	1200075	0.36614	LOC108637091
2	1100251	1200251	0.349377	LOC102173345
2	1100251	1200251	0.349377	LOC108637091
2	1100313	1200313	0.479016	LOC102173345
2	1100313	1200313	0.479016	LOC108637091
2	1100314	1200314	0.376811	LOC102173345
2	1100314	1200314	0.376811	LOC108637091
2	1100365	1200365	0.373014	LOC102173345
2	1100365	1200365	0.373014	LOC108637091
2	1100396	1200396	0.372522	LOC102173345
2	1100396	1200396	0.372522	LOC108637091
2	1100428	1200428	0.379573	LOC102173345
2	1100428	1200428	0.379573	LOC108637091
2	1100543	1200543	0.39928	LOC102173345
2	1100543	1200543	0.39928	LOC108637091
2	1100550	1200550	0.347891	LOC102173345
2	1100550	1200550	0.347891	LOC108637091
2	1100650	1200650	0.375173	LOC102173345
2	1100650	1200650	0.375173	LOC108637091
2	1100761	1200761	0.395474	LOC102173345
2	1100761	1200761	0.395474	LOC108637091
2	1100768	1200768	0.358782	LOC102173345
2	1100768	1200768	0.358782	LOC108637091
2	1100769	1200769	0.381122	LOC102173345
2	1100769	1200769	0.381122	LOC108637091
2	1100784	1200784	0.360163	LOC102173345

2	1100784	1200784	0.360163	LOC108637091
2	1100937	1200937	0.360163	LOC102173345
2	1100937	1200937	0.360163	LOC108637091
2	1101373	1201373	0.349485	LOC102173345
2	1101373	1201373	0.349485	LOC108637091
2	1101396	1201396	0.366854	LOC102173345
2	1101396	1201396	0.366854	LOC108637091
2	1101399	1201399	0.346793	LOC102173345
2	1101399	1201399	0.346793	LOC108637091
2	1101456	1201456	0.372657	LOC102173345
2	1101456	1201456	0.372657	LOC108637091
2	1101468	1201468	0.367913	LOC102173345
2	1101468	1201468	0.367913	LOC108637091
2	1101509	1201509	0.372198	LOC102173345
2	1101509	1201509	0.372198	LOC108637091
2	1101568	1201568	0.380521	LOC102173345
2	1101568	1201568	0.380521	LOC108637091
2	1101638	1201638	0.377328	LOC102173345
2	1101638	1201638	0.377328	LOC108637091
2	1101639	1201639	0.381294	LOC102173345
2	1101639	1201639	0.381294	LOC108637091
2	1101676	1201676	0.359889	LOC102173345
2	1101676	1201676	0.359889	LOC108637091
2	1101716	1201716	0.371781	LOC102173345
2	1101716	1201716	0.371781	LOC108637091
2	1101947	1201947	0.34242	LOC102173345
2	1101947	1201947	0.34242	LOC108637091
2	1102160	1202160	0.355927	LOC102173345
2	1102160	1202160	0.355927	LOC108637091
2	2550749	2650749	0.375611	LOC108637120
2	2550749	2650749	0.375611	MRTO4
2	2550749	2650749	0.375611	LOC106503221
2	2550749	2650749	0.375611	UBR4
2	2550749	2650749	0.375611	EMC1
2	2551289	2651289	0.345128	LOC108637120
2	2551289	2651289	0.345128	MRTO4
2	2551289	2651289	0.345128	LOC106503221
2	2551289	2651289	0.345128	UBR4
2	2551289	2651289	0.345128	EMC1
2	2561764	2661764	0.36291	LOC108637120
2	2561764	2661764	0.36291	MRTO4
2	2561764	2661764	0.36291	LOC106503221
2	2561764	2661764	0.36291	UBR4
2	2561764	2661764	0.36291	EMC1
2	2561826	2661826	0.354656	LOC108637120
2	2561826	2661826	0.354656	MRTO4
2	2561826	2661826	0.354656	LOC106503221
2	2561826	2661826	0.354656	UBR4
2	2561826	2661826	0.354656	EMC1
2	2562396	2662396	0.358185	LOC108637120
2	2562396	2662396	0.358185	MRTO4
2	2562396	2662396	0.358185	LOC106503221
2	2562396	2662396	0.358185	UBR4
2	2562396	2662396	0.358185	EMC1
2	2562437	2662437	0.355626	LOC108637120
2	2562437	2662437	0.355626	MRTO4
2	2562437	2662437	0.355626	LOC106503221
2	2562437	2662437	0.355626	UBR4

2	2562437	2662437	0.355626	EMC1
2	2562459	2662459	0.355626	LOC108637120
2	2562459	2662459	0.355626	MRTO4
2	2562459	2662459	0.355626	LOC106503221
2	2562459	2662459	0.355626	UBR4
2	2562459	2662459	0.355626	EMC1
2	2563056	2663056	0.352831	LOC108637120
2	2563056	2663056	0.352831	MRTO4
2	2563056	2663056	0.352831	LOC106503221
2	2563056	2663056	0.352831	UBR4
2	2563056	2663056	0.352831	EMC1
2	2563082	2663082	0.354542	LOC108637120
2	2563082	2663082	0.354542	MRTO4
2	2563082	2663082	0.354542	LOC106503221
2	2563082	2663082	0.354542	UBR4
2	2563082	2663082	0.354542	EMC1
2	2563227	2663227	0.354194	LOC108637120
2	2563227	2663227	0.354194	MRTO4
2	2563227	2663227	0.354194	LOC106503221
2	2563227	2663227	0.354194	UBR4
2	2563227	2663227	0.354194	EMC1
2	2563471	2663471	0.355304	LOC108637120
2	2563471	2663471	0.355304	MRTO4
2	2563471	2663471	0.355304	LOC106503221
2	2563471	2663471	0.355304	UBR4
2	2563471	2663471	0.355304	EMC1
2	2563551	2663551	0.365025	LOC108637120
2	2563551	2663551	0.365025	MRTO4
2	2563551	2663551	0.365025	LOC106503221
2	2563551	2663551	0.365025	UBR4
2	2563551	2663551	0.365025	EMC1
2	2563557	2663557	0.361525	LOC108637120
2	2563557	2663557	0.361525	MRTO4
2	2563557	2663557	0.361525	LOC106503221
2	2563557	2663557	0.361525	UBR4
2	2563557	2663557	0.361525	EMC1
2	2563653	2663653	0.358909	LOC108637120
2	2563653	2663653	0.358909	MRTO4
2	2563653	2663653	0.358909	LOC106503221
2	2563653	2663653	0.358909	UBR4
2	2563653	2663653	0.358909	EMC1
2	2563665	2663665	0.361699	LOC108637120
2	2563665	2663665	0.361699	MRTO4
2	2563665	2663665	0.361699	LOC106503221
2	2563665	2663665	0.361699	UBR4
2	2563665	2663665	0.361699	EMC1
2	2563735	2663735	0.361699	LOC108637120
2	2563735	2663735	0.361699	MRTO4
2	2563735	2663735	0.361699	LOC106503221
2	2563735	2663735	0.361699	UBR4
2	2563735	2663735	0.361699	EMC1
2	2564009	2664009	0.376606	LOC108637120
2	2564009	2664009	0.376606	MRTO4
2	2564009	2664009	0.376606	LOC106503221
2	2564009	2664009	0.376606	UBR4
2	2564009	2664009	0.376606	EMC1
2	2564426	2664426	0.3684	LOC108637120
2	2564426	2664426	0.3684	MRTO4

2	2564426	2664426	0.3684	LOC106503221
2	2564426	2664426	0.3684	UBR4
2	2564426	2664426	0.3684	EMC1
2	2564888	2664888	0.37304	LOC108637120
2	2564888	2664888	0.37304	MRTO4
2	2564888	2664888	0.37304	LOC106503221
2	2564888	2664888	0.37304	UBR4
2	2564888	2664888	0.37304	EMC1
2	2565070	2665070	0.36976	LOC108637120
2	2565070	2665070	0.36976	MRTO4
2	2565070	2665070	0.36976	LOC106503221
2	2565070	2665070	0.36976	UBR4
2	2565070	2665070	0.36976	EMC1
2	2565363	2665363	0.36976	LOC108637120
2	2565363	2665363	0.36976	MRTO4
2	2565363	2665363	0.36976	LOC106503221
2	2565363	2665363	0.36976	UBR4
2	2565363	2665363	0.36976	EMC1
2	2565588	2665588	0.36976	LOC108637120
2	2565588	2665588	0.36976	MRTO4
2	2565588	2665588	0.36976	LOC106503221
2	2565588	2665588	0.36976	UBR4
2	2565588	2665588	0.36976	EMC1
2	2565790	2665790	0.368191	LOC108637120
2	2565790	2665790	0.368191	MRTO4
2	2565790	2665790	0.368191	LOC106503221
2	2565790	2665790	0.368191	UBR4
2	2565790	2665790	0.368191	EMC1
2	2565980	2665980	0.369922	LOC108637120
2	2565980	2665980	0.369922	MRTO4
2	2565980	2665980	0.369922	LOC106503221
2	2565980	2665980	0.369922	UBR4
2	2565980	2665980	0.369922	EMC1
2	2566272	2666272	0.369182	LOC108637120
2	2566272	2666272	0.369182	MRTO4
2	2566272	2666272	0.369182	LOC106503221
2	2566272	2666272	0.369182	UBR4
2	2566272	2666272	0.369182	EMC1
2	2566489	2666489	0.384641	LOC108637120
2	2566489	2666489	0.384641	MRTO4
2	2566489	2666489	0.384641	LOC106503221
2	2566489	2666489	0.384641	UBR4
2	2566489	2666489	0.384641	EMC1
2	2566647	2666647	0.386726	LOC108637120
2	2566647	2666647	0.386726	MRTO4
2	2566647	2666647	0.386726	LOC106503221
2	2566647	2666647	0.386726	UBR4
2	2566647	2666647	0.386726	EMC1
2	2566766	2666766	0.3966	LOC108637120
2	2566766	2666766	0.3966	MRTO4
2	2566766	2666766	0.3966	LOC106503221
2	2566766	2666766	0.3966	UBR4
2	2566766	2666766	0.3966	EMC1
2	2566799	2666799	0.399549	LOC108637120
2	2566799	2666799	0.399549	MRTO4
2	2566799	2666799	0.399549	LOC106503221
2	2566799	2666799	0.399549	UBR4
2	2566799	2666799	0.399549	EMC1

2	2566815	2666815	0.39698	LOC108637120
2	2566815	2666815	0.39698	MRTO4
2	2566815	2666815	0.39698	LOC106503221
2	2566815	2666815	0.39698	UBR4
2	2566815	2666815	0.39698	EMC1
2	2566842	2666842	0.388792	LOC108637120
2	2566842	2666842	0.388792	MRTO4
2	2566842	2666842	0.388792	LOC106503221
2	2566842	2666842	0.388792	UBR4
2	2566842	2666842	0.388792	EMC1
2	2566953	2666953	0.391144	LOC108637120
2	2566953	2666953	0.391144	MRTO4
2	2566953	2666953	0.391144	LOC106503221
2	2566953	2666953	0.391144	UBR4
2	2566953	2666953	0.391144	EMC1
2	2567065	2667065	0.391519	LOC108637120
2	2567065	2667065	0.391519	MRTO4
2	2567065	2667065	0.391519	LOC106503221
2	2567065	2667065	0.391519	UBR4
2	2567065	2667065	0.391519	EMC1
2	2567270	2667270	0.387022	LOC108637120
2	2567270	2667270	0.387022	MRTO4
2	2567270	2667270	0.387022	LOC106503221
2	2567270	2667270	0.387022	UBR4
2	2567270	2667270	0.387022	EMC1
2	2567381	2667381	0.387022	LOC108637120
2	2567381	2667381	0.387022	MRTO4
2	2567381	2667381	0.387022	LOC106503221
2	2567381	2667381	0.387022	UBR4
2	2567381	2667381	0.387022	EMC1
2	2567523	2667523	0.389807	LOC108637120
2	2567523	2667523	0.389807	MRTO4
2	2567523	2667523	0.389807	LOC106503221
2	2567523	2667523	0.389807	UBR4
2	2567523	2667523	0.389807	EMC1
2	2567541	2667541	0.392152	LOC108637120
2	2567541	2667541	0.392152	MRTO4
2	2567541	2667541	0.392152	LOC106503221
2	2567541	2667541	0.392152	UBR4
2	2567541	2667541	0.392152	EMC1
2	2567563	2667563	0.387538	LOC108637120
2	2567563	2667563	0.387538	MRTO4
2	2567563	2667563	0.387538	LOC106503221
2	2567563	2667563	0.387538	UBR4
2	2567563	2667563	0.387538	EMC1
2	2567638	2667638	0.387014	LOC108637120
2	2567638	2667638	0.387014	MRTO4
2	2567638	2667638	0.387014	LOC106503221
2	2567638	2667638	0.387014	UBR4
2	2567638	2667638	0.387014	EMC1
2	2567775	2667775	0.38765	LOC108637120
2	2567775	2667775	0.38765	MRTO4
2	2567775	2667775	0.38765	LOC106503221
2	2567775	2667775	0.38765	UBR4
2	2567775	2667775	0.38765	EMC1
2	2567895	2667895	0.387508	LOC108637120
2	2567895	2667895	0.387508	MRTO4
2	2567895	2667895	0.387508	LOC106503221

2	2567895	2667895	0.387508	UBR4
2	2567895	2667895	0.387508	EMC1
2	2568137	2668137	0.381677	LOC108637120
2	2568137	2668137	0.381677	MRTO4
2	2568137	2668137	0.381677	LOC106503221
2	2568137	2668137	0.381677	UBR4
2	2568137	2668137	0.381677	EMC1
2	2568212	2668212	0.382104	LOC108637120
2	2568212	2668212	0.382104	MRTO4
2	2568212	2668212	0.382104	LOC106503221
2	2568212	2668212	0.382104	UBR4
2	2568212	2668212	0.382104	EMC1
2	2568528	2668528	0.382104	LOC108637120
2	2568528	2668528	0.382104	MRTO4
2	2568528	2668528	0.382104	LOC106503221
2	2568528	2668528	0.382104	UBR4
2	2568528	2668528	0.382104	EMC1
2	2568658	2668658	0.382104	LOC108637120
2	2568658	2668658	0.382104	MRTO4
2	2568658	2668658	0.382104	LOC106503221
2	2568658	2668658	0.382104	UBR4
2	2568658	2668658	0.382104	EMC1
2	2568686	2668686	0.382104	LOC108637120
2	2568686	2668686	0.382104	MRTO4
2	2568686	2668686	0.382104	LOC106503221
2	2568686	2668686	0.382104	UBR4
2	2568686	2668686	0.382104	EMC1
2	2568859	2668859	0.388095	LOC108637120
2	2568859	2668859	0.388095	MRTO4
2	2568859	2668859	0.388095	LOC106503221
2	2568859	2668859	0.388095	UBR4
2	2568859	2668859	0.388095	EMC1
2	2568860	2668860	0.376533	LOC108637120
2	2568860	2668860	0.376533	MRTO4
2	2568860	2668860	0.376533	LOC106503221
2	2568860	2668860	0.376533	UBR4
2	2568860	2668860	0.376533	EMC1
2	2568933	2668933	0.379488	LOC108637120
2	2568933	2668933	0.379488	MRTO4
2	2568933	2668933	0.379488	LOC106503221
2	2568933	2668933	0.379488	UBR4
2	2568933	2668933	0.379488	EMC1
2	2569009	2669009	0.365081	LOC108637120
2	2569009	2669009	0.365081	MRTO4
2	2569009	2669009	0.365081	LOC106503221
2	2569009	2669009	0.365081	UBR4
2	2569009	2669009	0.365081	EMC1
2	2569253	2669253	0.365081	LOC108637120
2	2569253	2669253	0.365081	MRTO4
2	2569253	2669253	0.365081	LOC106503221
2	2569253	2669253	0.365081	UBR4
2	2569253	2669253	0.365081	EMC1
2	2569435	2669435	0.365081	LOC108637120
2	2569435	2669435	0.365081	MRTO4
2	2569435	2669435	0.365081	LOC106503221
2	2569435	2669435	0.365081	UBR4
2	2569435	2669435	0.365081	EMC1
2	2569633	2669633	0.365081	LOC108637120

2	2569633	2669633	0.365081	MRTO4
2	2569633	2669633	0.365081	LOC106503221
2	2569633	2669633	0.365081	UBR4
2	2569633	2669633	0.365081	EMC1
2	2569820	2669820	0.365081	LOC108637120
2	2569820	2669820	0.365081	MRTO4
2	2569820	2669820	0.365081	LOC106503221
2	2569820	2669820	0.365081	UBR4
2	2569820	2669820	0.365081	EMC1
2	2569822	2669822	0.365081	LOC108637120
2	2569822	2669822	0.365081	MRTO4
2	2569822	2669822	0.365081	LOC106503221
2	2569822	2669822	0.365081	UBR4
2	2569822	2669822	0.365081	EMC1
2	2569929	2669929	0.3632	LOC108637120
2	2569929	2669929	0.3632	MRTO4
2	2569929	2669929	0.3632	LOC106503221
2	2569929	2669929	0.3632	UBR4
2	2569929	2669929	0.3632	EMC1
2	2570111	2670111	0.3632	LOC108637120
2	2570111	2670111	0.3632	MRTO4
2	2570111	2670111	0.3632	LOC106503221
2	2570111	2670111	0.3632	UBR4
2	2570111	2670111	0.3632	EMC1
2	2570354	2670354	0.362109	LOC108637120
2	2570354	2670354	0.362109	MRTO4
2	2570354	2670354	0.362109	LOC106503221
2	2570354	2670354	0.362109	UBR4
2	2570354	2670354	0.362109	EMC1
2	2570393	2670393	0.364625	LOC108637120
2	2570393	2670393	0.364625	MRTO4
2	2570393	2670393	0.364625	LOC106503221
2	2570393	2670393	0.364625	UBR4
2	2570393	2670393	0.364625	EMC1
2	2570737	2670737	0.364625	LOC108637120
2	2570737	2670737	0.364625	MRTO4
2	2570737	2670737	0.364625	LOC106503221
2	2570737	2670737	0.364625	UBR4
2	2570737	2670737	0.364625	EMC1
2	2570816	2670816	0.363564	LOC108637120
2	2570816	2670816	0.363564	MRTO4
2	2570816	2670816	0.363564	LOC106503221
2	2570816	2670816	0.363564	UBR4
2	2570816	2670816	0.363564	EMC1
2	2570887	2670887	0.363499	LOC108637120
2	2570887	2670887	0.363499	MRTO4
2	2570887	2670887	0.363499	LOC106503221
2	2570887	2670887	0.363499	UBR4
2	2570887	2670887	0.363499	EMC1
2	2570938	2670938	0.363546	LOC108637120
2	2570938	2670938	0.363546	MRTO4
2	2570938	2670938	0.363546	LOC106503221
2	2570938	2670938	0.363546	UBR4
2	2570938	2670938	0.363546	EMC1
2	2571037	2671037	0.353151	LOC108637120
2	2571037	2671037	0.353151	MRTO4
2	2571037	2671037	0.353151	LOC106503221
2	2571037	2671037	0.353151	UBR4

2	2571037	2671037	0.353151	EMC1
2	2571324	2671324	0.367096	LOC108637120
2	2571324	2671324	0.367096	MRTO4
2	2571324	2671324	0.367096	LOC106503221
2	2571324	2671324	0.367096	UBR4
2	2571324	2671324	0.367096	EMC1
2	2571821	2671821	0.364563	LOC108637120
2	2571821	2671821	0.364563	MRTO4
2	2571821	2671821	0.364563	LOC106503221
2	2571821	2671821	0.364563	UBR4
2	2571821	2671821	0.364563	EMC1
2	2571944	2671944	0.367389	LOC108637120
2	2571944	2671944	0.367389	MRTO4
2	2571944	2671944	0.367389	LOC106503221
2	2571944	2671944	0.367389	UBR4
2	2571944	2671944	0.367389	EMC1
2	2572038	2672038	0.367353	LOC108637120
2	2572038	2672038	0.367353	MRTO4
2	2572038	2672038	0.367353	LOC106503221
2	2572038	2672038	0.367353	UBR4
2	2572038	2672038	0.367353	EMC1
2	2572051	2672051	0.380127	LOC108637120
2	2572051	2672051	0.380127	MRTO4
2	2572051	2672051	0.380127	LOC106503221
2	2572051	2672051	0.380127	UBR4
2	2572051	2672051	0.380127	EMC1
2	2572062	2672062	0.377984	LOC108637120
2	2572062	2672062	0.377984	MRTO4
2	2572062	2672062	0.377984	LOC106503221
2	2572062	2672062	0.377984	UBR4
2	2572062	2672062	0.377984	EMC1
2	2572223	2672223	0.386176	LOC108637120
2	2572223	2672223	0.386176	MRTO4
2	2572223	2672223	0.386176	LOC106503221
2	2572223	2672223	0.386176	UBR4
2	2572223	2672223	0.386176	EMC1
2	2572603	2672603	0.379591	LOC108637120
2	2572603	2672603	0.379591	MRTO4
2	2572603	2672603	0.379591	LOC106503221
2	2572603	2672603	0.379591	UBR4
2	2572603	2672603	0.379591	EMC1
2	2572782	2672782	0.396758	LOC108637120
2	2572782	2672782	0.396758	MRTO4
2	2572782	2672782	0.396758	LOC106503221
2	2572782	2672782	0.396758	UBR4
2	2572782	2672782	0.396758	EMC1
2	2572960	2672960	0.382473	LOC108637120
2	2572960	2672960	0.382473	MRTO4
2	2572960	2672960	0.382473	LOC106503221
2	2572960	2672960	0.382473	UBR4
2	2572960	2672960	0.382473	EMC1
2	2573212	2673212	0.388033	LOC108637120
2	2573212	2673212	0.388033	MRTO4
2	2573212	2673212	0.388033	LOC106503221
2	2573212	2673212	0.388033	UBR4
2	2573212	2673212	0.388033	EMC1
2	2573833	2673833	0.38521	LOC108637120
2	2573833	2673833	0.38521	MRTO4

2	2573833	2673833	0.38521	LOC106503221
2	2573833	2673833	0.38521	UBR4
2	2573833	2673833	0.38521	EMC1
2	2574884	2674884	0.346539	LOC108637120
2	2574884	2674884	0.346539	MRTO4
2	2574884	2674884	0.346539	LOC106503221
2	2574884	2674884	0.346539	UBR4
2	2574884	2674884	0.346539	EMC1
2	2574933	2674933	0.354842	LOC108637120
2	2574933	2674933	0.354842	MRTO4
2	2574933	2674933	0.354842	LOC106503221
2	2574933	2674933	0.354842	UBR4
2	2574933	2674933	0.354842	EMC1
2	2575178	2675178	0.351445	LOC108637120
2	2575178	2675178	0.351445	MRTO4
2	2575178	2675178	0.351445	LOC106503221
2	2575178	2675178	0.351445	UBR4
2	2575178	2675178	0.351445	EMC1
2	2579700	2679700	0.354534	LOC108637120
2	2579700	2679700	0.354534	MRTO4
2	2579700	2679700	0.354534	LOC106503221
2	2579700	2679700	0.354534	UBR4
2	2579700	2679700	0.354534	EMC1
2	2784204	2884204	0.354843	CAPZB
2	2784243	2884243	0.342405	CAPZB
2	2784333	2884333	0.353873	CAPZB
2	2784699	2884699	0.349865	CAPZB
2	2784826	2884826	0.362943	CAPZB
2	2784918	2884918	0.365996	CAPZB
2	2784950	2884950	0.364617	CAPZB
2	2784963	2884963	0.360567	CAPZB
2	2784991	2884991	0.359993	CAPZB
2	2785010	2885010	0.359993	CAPZB
2	2785041	2885041	0.361616	CAPZB
2	2785066	2885066	0.360705	CAPZB
2	2785071	2885071	0.360705	CAPZB
2	2785074	2885074	0.360705	CAPZB
2	2785113	2885113	0.360705	CAPZB
2	2785132	2885132	0.360705	CAPZB
2	2785136	2885136	0.360705	CAPZB
2	2785137	2885137	0.360705	CAPZB
2	2785191	2885191	0.380607	CAPZB
2	2785217	2885217	0.380588	CAPZB
2	2785226	2885226	0.380984	CAPZB
2	2785248	2885248	0.38233	CAPZB
2	2785289	2885289	0.391834	CAPZB
2	2785440	2885440	0.385725	CAPZB
2	2785478	2885478	0.385721	CAPZB
2	2785515	2885515	0.388475	CAPZB
2	2785517	2885517	0.391349	CAPZB
2	2785549	2885549	0.415999	CAPZB
2	2785557	2885557	0.412136	CAPZB
2	2785566	2885566	0.411588	CAPZB
2	2785567	2885567	0.411588	CAPZB
2	2785570	2885570	0.407605	CAPZB
2	2785607	2885607	0.40797	CAPZB
2	2785608	2885608	0.442467	CAPZB
2	2785617	2885617	0.431984	CAPZB

2	2785621	2885621	0.429942	CAPZB
2	2785657	2885657	0.437417	CAPZB
2	2785671	2885671	0.435567	CAPZB
2	2785701	2885701	0.435567	CAPZB
2	2785731	2885731	0.428645	CAPZB
2	2785754	2885754	0.440125	CAPZB
2	2785766	2885766	0.440125	CAPZB
2	2785767	2885767	0.440125	CAPZB
2	2785831	2885831	0.4215	CAPZB
2	2785865	2885865	0.42606	CAPZB
2	2785957	2885957	0.404382	CAPZB
2	2785991	2885991	0.404904	CAPZB
2	2786005	2886005	0.3975	CAPZB
2	2786068	2886068	0.38976	CAPZB
2	2786069	2886069	0.38976	CAPZB
2	2786072	2886072	0.38976	CAPZB
2	2786089	2886089	0.38976	CAPZB
2	2786156	2886156	0.38976	CAPZB
2	2786305	2886305	0.396908	CAPZB
2	2786488	2886488	0.385785	MINOS1
2	2786488	2886488	0.385785	CAPZB
2	2786503	2886503	0.385785	MINOS1
2	2786503	2886503	0.385785	CAPZB
2	2786582	2886582	0.385785	MINOS1
2	2786582	2886582	0.385785	CAPZB
2	2786700	2886700	0.396999	MINOS1
2	2786700	2886700	0.396999	CAPZB
2	2786773	2886773	0.402655	MINOS1
2	2786773	2886773	0.402655	CAPZB
2	2786780	2886780	0.413739	MINOS1
2	2786780	2886780	0.413739	CAPZB
2	2786781	2886781	0.410026	MINOS1
2	2786781	2886781	0.410026	CAPZB
2	2786858	2886858	0.34788	MINOS1
2	2786858	2886858	0.34788	CAPZB
2	2787018	2887018	0.362194	MINOS1
2	2787018	2887018	0.362194	CAPZB
2	2787053	2887053	0.362194	MINOS1
2	2787053	2887053	0.362194	CAPZB
2	2787199	2887199	0.360592	MINOS1
2	2787199	2887199	0.360592	CAPZB
2	2787272	2887272	0.360592	MINOS1
2	2787272	2887272	0.360592	CAPZB
2	3364193	3464193	0.348255	PLA2G2F
2	3364193	3464193	0.348255	LOC102168673
2	3364193	3464193	0.348255	LOC108633234
2	3364589	3464589	0.355184	PLA2G2F
2	3364589	3464589	0.355184	LOC102168673
2	3364589	3464589	0.355184	LOC108633234
2	3551813	3651813	0.346443	VWA5B1
2	3551820	3651820	0.344994	VWA5B1
2	3551933	3651933	0.352377	VWA5B1
2	4493421	4593421	0.344266	ECE1
2	4493517	4593517	0.375467	ECE1
2	4493840	4593840	0.389329	ECE1
2	4493848	4593848	0.366178	ECE1
2	4493949	4593949	0.359956	ECE1
2	4494002	4594002	0.359956	ECE1

2	4494101	4594101	0.361669	ECE1
2	4494135	4594135	0.35875	ECE1
2	4494204	4594204	0.347314	ECE1
2	4494207	4594207	0.347314	ECE1
2	4494470	4594470	0.354705	ECE1
2	4494581	4594581	0.354705	ECE1
2	4494736	4594736	0.351162	ECE1
2	4494864	4594864	0.35844	ECE1
2	4495038	4595038	0.355214	ECE1
2	4495085	4595085	0.359128	ECE1
2	4495511	4595511	0.354437	ECE1
2	4495520	4595520	0.357911	ECE1
2	4495534	4595534	0.357911	ECE1
2	4495626	4595626	0.35882	ECE1
2	4495647	4595647	0.358796	ECE1
2	4495697	4595697	0.357716	ECE1
2	4495725	4595725	0.36576	ECE1
2	4495765	4595765	0.366529	ECE1
2	4495776	4595776	0.373547	ECE1
2	4495797	4595797	0.367626	ECE1
2	4495967	4595967	0.355788	ECE1
2	4496036	4596036	0.355788	ECE1
2	4496080	4596080	0.413697	ECE1
2	4496500	4596500	0.425332	ECE1
2	4496501	4596501	0.445374	ECE1
2	4496606	4596606	0.432013	ECE1
2	4496722	4596722	0.420423	ECE1
2	4496784	4596784	0.402269	ECE1
2	4496788	4596788	0.408582	ECE1
2	4496903	4596903	0.401163	ECE1
2	4497102	4597102	0.345867	ECE1
2	4497507	4597507	0.348554	ECE1
2	4497508	4597508	0.351186	ECE1
2	4497544	4597544	0.345576	ECE1
2	4497550	4597550	0.360155	ECE1
2	4497910	4597910	0.343662	ECE1
2	4497952	4597952	0.355992	ECE1
2	4497990	4597990	0.346754	ECE1
2	4498169	4598169	0.359168	ECE1
2	4498283	4598283	0.349916	ECE1
2	4498736	4598736	0.370615	ECE1
2	4498743	4598743	0.368653	ECE1
2	4499024	4599024	0.368532	ECE1
2	4499131	4599131	0.358225	ECE1
2	4499207	4599207	0.357747	ECE1
2	4499216	4599216	0.356112	ECE1
2	4499242	4599242	0.365403	ECE1
2	4499251	4599251	0.370238	ECE1
2	4499275	4599275	0.37076	ECE1
2	4499279	4599279	0.37076	ECE1
2	4499298	4599298	0.372695	ECE1
2	4505386	4605386	0.359762	ECE1
2	6198886	6298886	0.353591	LUZP1
2	6594205	6694205	0.343245	E2F2
2	6594205	6694205	0.343245	ID3
2	6594795	6694795	0.360059	E2F2
2	6594795	6694795	0.360059	ID3
2	6594932	6694932	0.360059	E2F2

2	6594932	6694932	0.360059	ID3
2	6594977	6694977	0.36054	E2F2
2	6594977	6694977	0.36054	ID3
2	6596587	6696587	0.35473	E2F2
2	6596587	6696587	0.35473	ID3
2	6600799	6700799	0.38312	E2F2
2	6600799	6700799	0.38312	ID3
2	6600862	6700862	0.37965	E2F2
2	6600862	6700862	0.37965	ID3
2	6600906	6700906	0.390235	E2F2
2	6600906	6700906	0.390235	ID3
2	6600942	6700942	0.383544	E2F2
2	6600942	6700942	0.383544	ID3
2	6601371	6701371	0.380826	E2F2
2	6601371	6701371	0.380826	ID3
2	6601385	6701385	0.380826	E2F2
2	6601385	6701385	0.380826	ID3
2	6601655	6701655	0.382638	E2F2
2	6601655	6701655	0.382638	ID3
2	6601953	6701953	0.381126	E2F2
2	6601953	6701953	0.381126	ID3
2	6601972	6701972	0.381126	E2F2
2	6601972	6701972	0.381126	ID3
2	6601982	6701982	0.381126	E2F2
2	6601982	6701982	0.381126	ID3
2	6602813	6702813	0.378937	E2F2
2	6602813	6702813	0.378937	ID3
2	6603338	6703338	0.392699	E2F2
2	6603338	6703338	0.392699	ID3
2	6603341	6703341	0.392699	E2F2
2	6603341	6703341	0.392699	ID3
2	6603434	6703434	0.392699	E2F2
2	6603434	6703434	0.392699	ID3
2	6603832	6703832	0.385068	E2F2
2	6603832	6703832	0.385068	ID3
2	6603997	6703997	0.38498	E2F2
2	6603997	6703997	0.38498	ID3
2	6604344	6704344	0.380072	E2F2
2	6604344	6704344	0.380072	ID3
2	6604510	6704510	0.380072	E2F2
2	6604510	6704510	0.380072	ID3
2	6604837	6704837	0.383332	E2F2
2	6604837	6704837	0.383332	ID3
2	6605788	6705788	0.36795	E2F2
2	6605788	6705788	0.36795	ID3
2	6605811	6705811	0.36795	E2F2
2	6605811	6705811	0.36795	ID3
2	6606190	6706190	0.368137	E2F2
2	6606190	6706190	0.368137	ID3
2	6606607	6706607	0.36888	E2F2
2	6606607	6706607	0.36888	ID3
2	6607778	6707778	0.365265	E2F2
2	6607778	6707778	0.365265	ID3
2	6607959	6707959	0.353969	E2F2
2	6607959	6707959	0.353969	ID3
2	6608327	6708327	0.358458	E2F2
2	6608327	6708327	0.358458	ID3
2	6608387	6708387	0.35018	E2F2

2	6608387	6708387	0.35018	ID3
2	6609908	6709908	0.375874	E2F2
2	6609908	6709908	0.375874	ID3
2	6609978	6709978	0.401196	E2F2
2	6609978	6709978	0.401196	ID3
2	6611455	6711455	0.343869	E2F2
2	6611455	6711455	0.343869	ID3
2	6611496	6711496	0.344542	E2F2
2	6611496	6711496	0.344542	ID3
2	6611751	6711751	0.343064	E2F2
2	6611751	6711751	0.343064	ID3
2	6614726	6714726	0.350449	E2F2
2	6614726	6714726	0.350449	ID3
2	6614774	6714774	0.347923	E2F2
2	6614774	6714774	0.347923	ID3
2	6614775	6714775	0.346779	E2F2
2	6614775	6714775	0.346779	ID3
2	6614872	6714872	0.346779	E2F2
2	6614872	6714872	0.346779	ID3
2	6614878	6714878	0.349822	E2F2
2	6614878	6714878	0.349822	ID3
2	6615146	6715146	0.358633	E2F2
2	6615146	6715146	0.358633	ID3
2	6615268	6715268	0.35247	E2F2
2	6615268	6715268	0.35247	ID3
2	6615375	6715375	0.35988	E2F2
2	6615375	6715375	0.35988	ID3
2	6615416	6715416	0.359052	E2F2
2	6615416	6715416	0.359052	ID3
2	6615475	6715475	0.360054	E2F2
2	6615475	6715475	0.360054	ID3
2	6615490	6715490	0.35774	E2F2
2	6615490	6715490	0.35774	ID3
2	6629589	6729589	0.34232	ID3
2	6629666	6729666	0.342687	ID3
2	6629681	6729681	0.3448	ID3
2	7070380	7170380	0.349839	MYOM3
2	7070380	7170380	0.349839	IL22RA1
2	7070557	7170557	0.35406	MYOM3
2	7070557	7170557	0.35406	IL22RA1
2	7070625	7170625	0.357949	MYOM3
2	7070625	7170625	0.357949	IL22RA1
2	7070670	7170670	0.351328	MYOM3
2	7070670	7170670	0.351328	IL22RA1
2	7070824	7170824	0.365801	MYOM3
2	7070824	7170824	0.365801	IL22RA1
2	7071319	7171319	0.364416	MYOM3
2	7071319	7171319	0.364416	IL22RA1
2	7071584	7171584	0.370921	MYOM3
2	7071584	7171584	0.370921	IL22RA1
2	7071700	7171700	0.371661	MYOM3
2	7071700	7171700	0.371661	IL22RA1
2	7071721	7171721	0.370262	MYOM3
2	7071721	7171721	0.370262	IL22RA1
2	7071785	7171785	0.365842	MYOM3
2	7071785	7171785	0.365842	IL22RA1
2	7071915	7171915	0.368932	MYOM3
2	7071915	7171915	0.368932	IL22RA1

2	7072101	7172101	0.359486	MYOM3
2	7072101	7172101	0.359486	IL22RA1
2	7072109	7172109	0.359486	MYOM3
2	7072109	7172109	0.359486	IL22RA1
2	7072227	7172227	0.359408	MYOM3
2	7072227	7172227	0.359408	IL22RA1
2	7072307	7172307	0.361599	MYOM3
2	7072307	7172307	0.361599	IL22RA1
2	7072591	7172591	0.359367	MYOM3
2	7072591	7172591	0.359367	IL22RA1
2	7073160	7173160	0.364056	MYOM3
2	7073160	7173160	0.364056	IL22RA1
2	7073347	7173347	0.362334	MYOM3
2	7073347	7173347	0.362334	IL22RA1
2	7073528	7173528	0.364112	MYOM3
2	7073528	7173528	0.364112	IL22RA1
2	7074341	7174341	0.364888	MYOM3
2	7074341	7174341	0.364888	IL22RA1
2	7074459	7174459	0.372046	MYOM3
2	7074459	7174459	0.372046	IL22RA1
2	7074470	7174470	0.371257	MYOM3
2	7074470	7174470	0.371257	IL22RA1
2	7074583	7174583	0.372117	MYOM3
2	7074583	7174583	0.372117	IL22RA1
2	7074613	7174613	0.358962	MYOM3
2	7074613	7174613	0.358962	IL22RA1
2	7075971	7175971	0.344699	MYOM3
2	7075971	7175971	0.344699	IL22RA1
2	7077610	7177610	0.342068	MYOM3
2	7077610	7177610	0.342068	IL22RA1
2	7077631	7177631	0.342423	MYOM3
2	7077631	7177631	0.342423	IL22RA1
2	7077707	7177707	0.342423	MYOM3
2	7077707	7177707	0.342423	IL22RA1
2	7077708	7177708	0.342423	MYOM3
2	7077708	7177708	0.342423	IL22RA1
2	7077976	7177976	0.342423	MYOM3
2	7077976	7177976	0.342423	IL22RA1
2	7078281	7178281	0.342258	MYOM3
2	7078281	7178281	0.342258	IL22RA1
2	7078686	7178686	0.353041	MYOM3
2	7078686	7178686	0.353041	IL22RA1
2	7078831	7178831	0.349685	MYOM3
2	7078831	7178831	0.349685	IL22RA1
2	7079590	7179590	0.378872	MYOM3
2	7079590	7179590	0.378872	IL22RA1
2	7079765	7179765	0.388166	MYOM3
2	7079765	7179765	0.388166	IL22RA1
2	7079793	7179793	0.387374	MYOM3
2	7079793	7179793	0.387374	IL22RA1
2	7079955	7179955	0.389144	MYOM3
2	7079955	7179955	0.389144	IL22RA1
2	7080037	7180037	0.387378	MYOM3
2	7080037	7180037	0.387378	IL22RA1
2	7080175	7180175	0.395208	MYOM3
2	7080175	7180175	0.395208	IL22RA1
2	7080289	7180289	0.397115	MYOM3
2	7080289	7180289	0.397115	IL22RA1

2	7080347	7180347	0.404765	MYOM3
2	7080347	7180347	0.404765	IL22RA1
2	7080376	7180376	0.40108	MYOM3
2	7080376	7180376	0.40108	IL22RA1
2	7080428	7180428	0.391937	MYOM3
2	7080428	7180428	0.391937	IL22RA1
2	7080472	7180472	0.395256	MYOM3
2	7080472	7180472	0.395256	IL22RA1
2	7080482	7180482	0.392306	MYOM3
2	7080482	7180482	0.392306	IL22RA1
2	7080582	7180582	0.392306	MYOM3
2	7080582	7180582	0.392306	IL22RA1
2	7080834	7180834	0.385633	MYOM3
2	7080834	7180834	0.385633	IL22RA1
2	7080852	7180852	0.392917	MYOM3
2	7080852	7180852	0.392917	IL22RA1
2	7081520	7181520	0.495879	MYOM3
2	7081520	7181520	0.495879	IL22RA1
2	7081579	7181579	0.4084	MYOM3
2	7081579	7181579	0.4084	IL22RA1
2	7081715	7181715	0.411233	MYOM3
2	7081715	7181715	0.411233	IL22RA1
2	7082181	7182181	0.411255	MYOM3
2	7082181	7182181	0.411255	IL22RA1
2	7082674	7182674	0.411691	MYOM3
2	7082674	7182674	0.411691	IL22RA1
2	7082886	7182886	0.411691	MYOM3
2	7082886	7182886	0.411691	IL22RA1
2	7082889	7182889	0.411691	MYOM3
2	7082889	7182889	0.411691	IL22RA1
2	7082898	7182898	0.411691	MYOM3
2	7082898	7182898	0.411691	IL22RA1
2	7082922	7182922	0.41573	MYOM3
2	7082922	7182922	0.41573	IL22RA1
2	7083001	7183001	0.414278	MYOM3
2	7083001	7183001	0.414278	IL22RA1
2	7083126	7183126	0.414278	MYOM3
2	7083126	7183126	0.414278	IL22RA1
2	7083140	7183140	0.414278	MYOM3
2	7083140	7183140	0.414278	IL22RA1
2	7083207	7183207	0.414278	MYOM3
2	7083207	7183207	0.414278	IL22RA1
2	7083217	7183217	0.414278	MYOM3
2	7083217	7183217	0.414278	IL22RA1
2	7083277	7183277	0.41756	MYOM3
2	7083277	7183277	0.41756	IL22RA1
2	7083314	7183314	0.417265	MYOM3
2	7083314	7183314	0.417265	IL22RA1
2	7083358	7183358	0.416756	MYOM3
2	7083358	7183358	0.416756	IL22RA1
2	7083596	7183596	0.423269	MYOM3
2	7083596	7183596	0.423269	IL22RA1
2	7083597	7183597	0.423269	MYOM3
2	7083597	7183597	0.423269	IL22RA1
2	7083619	7183619	0.423269	MYOM3
2	7083619	7183619	0.423269	IL22RA1
2	7083695	7183695	0.418092	MYOM3
2	7083695	7183695	0.418092	IL22RA1

2	7083753	7183753	0.417947	MYOM3
2	7083753	7183753	0.417947	IL22RA1
2	7083811	7183811	0.42036	MYOM3
2	7083811	7183811	0.42036	IL22RA1
2	7083854	7183854	0.421018	MYOM3
2	7083854	7183854	0.421018	IL22RA1
2	7083962	7183962	0.419424	MYOM3
2	7083962	7183962	0.419424	IL22RA1
2	7083979	7183979	0.419424	MYOM3
2	7083979	7183979	0.419424	IL22RA1
2	7084128	7184128	0.418806	MYOM3
2	7084128	7184128	0.418806	IL22RA1
2	7084276	7184276	0.419261	MYOM3
2	7084276	7184276	0.419261	IL22RA1
2	7084286	7184286	0.416229	MYOM3
2	7084286	7184286	0.416229	IL22RA1
2	7084581	7184581	0.412149	MYOM3
2	7084581	7184581	0.412149	IL22RA1
2	7084692	7184692	0.412149	MYOM3
2	7084692	7184692	0.412149	IL22RA1
2	7084723	7184723	0.412149	MYOM3
2	7084723	7184723	0.412149	IL22RA1
2	7085095	7185095	0.40983	MYOM3
2	7085095	7185095	0.40983	IL22RA1
2	7085278	7185278	0.399239	MYOM3
2	7085278	7185278	0.399239	IL22RA1
2	7085367	7185367	0.407574	MYOM3
2	7085367	7185367	0.407574	IL22RA1
2	7085445	7185445	0.407019	MYOM3
2	7085445	7185445	0.407019	IL22RA1
2	7085462	7185462	0.407019	MYOM3
2	7085462	7185462	0.407019	IL22RA1
2	7085470	7185470	0.407019	MYOM3
2	7085470	7185470	0.407019	IL22RA1
2	7085474	7185474	0.407019	MYOM3
2	7085474	7185474	0.407019	IL22RA1
2	7085483	7185483	0.407019	MYOM3
2	7085483	7185483	0.407019	IL22RA1
2	7085552	7185552	0.406965	MYOM3
2	7085552	7185552	0.406965	IL22RA1
2	7085553	7185553	0.406965	MYOM3
2	7085553	7185553	0.406965	IL22RA1
2	7085554	7185554	0.406503	MYOM3
2	7085554	7185554	0.406503	IL22RA1
2	7085690	7185690	0.406503	MYOM3
2	7085690	7185690	0.406503	IL22RA1
2	7085738	7185738	0.406841	MYOM3
2	7085738	7185738	0.406841	IL22RA1
2	7085760	7185760	0.407138	MYOM3
2	7085760	7185760	0.407138	IL22RA1
2	7085976	7185976	0.406566	MYOM3
2	7085976	7185976	0.406566	IL22RA1
2	7085997	7185997	0.406653	MYOM3
2	7085997	7185997	0.406653	IL22RA1
2	7086027	7186027	0.405926	MYOM3
2	7086027	7186027	0.405926	IL22RA1
2	7086135	7186135	0.408504	MYOM3
2	7086135	7186135	0.408504	IL22RA1

2	7086448	7186448	0.40704	MYOM3
2	7086448	7186448	0.40704	IL22RA1
2	7086480	7186480	0.409602	MYOM3
2	7086480	7186480	0.409602	IL22RA1
2	7086600	7186600	0.408307	MYOM3
2	7086600	7186600	0.408307	IL22RA1
2	7086630	7186630	0.406299	MYOM3
2	7086630	7186630	0.406299	IL22RA1
2	7086853	7186853	0.406299	MYOM3
2	7086853	7186853	0.406299	IL22RA1
2	7087016	7187016	0.423755	MYOM3
2	7087016	7187016	0.423755	IL22RA1
2	7087114	7187114	0.402451	MYOM3
2	7087114	7187114	0.402451	IL22RA1
2	7087115	7187115	0.402451	MYOM3
2	7087115	7187115	0.402451	IL22RA1
2	7087116	7187116	0.402451	MYOM3
2	7087116	7187116	0.402451	IL22RA1
2	7087118	7187118	0.402451	MYOM3
2	7087118	7187118	0.402451	IL22RA1
2	7087119	7187119	0.402451	MYOM3
2	7087119	7187119	0.402451	IL22RA1
2	7087132	7187132	0.402533	MYOM3
2	7087132	7187132	0.402533	IL22RA1
2	7087288	7187288	0.402499	MYOM3
2	7087288	7187288	0.402499	IL22RA1
2	7087305	7187305	0.402499	MYOM3
2	7087305	7187305	0.402499	IL22RA1
2	7087331	7187331	0.393733	MYOM3
2	7087331	7187331	0.393733	IL22RA1
2	7087378	7187378	0.41308	MYOM3
2	7087378	7187378	0.41308	IL22RA1
2	7087493	7187493	0.405706	MYOM3
2	7087493	7187493	0.405706	IL22RA1
2	7087510	7187510	0.405706	MYOM3
2	7087510	7187510	0.405706	IL22RA1
2	7087671	7187671	0.398731	MYOM3
2	7087671	7187671	0.398731	IL22RA1
2	7087814	7187814	0.410919	MYOM3
2	7087814	7187814	0.410919	IL22RA1
2	7087815	7187815	0.410919	MYOM3
2	7087815	7187815	0.410919	IL22RA1
2	7087984	7187984	0.410919	MYOM3
2	7087984	7187984	0.410919	IL22RA1
2	7088029	7188029	0.410919	MYOM3
2	7088029	7188029	0.410919	IL22RA1
2	7088037	7188037	0.410919	MYOM3
2	7088037	7188037	0.410919	IL22RA1
2	7088052	7188052	0.410919	MYOM3
2	7088052	7188052	0.410919	IL22RA1
2	7088078	7188078	0.410919	MYOM3
2	7088078	7188078	0.410919	IL22RA1
2	7088163	7188163	0.41284	MYOM3
2	7088163	7188163	0.41284	IL22RA1
2	7088616	7188616	0.412206	MYOM3
2	7088616	7188616	0.412206	IL22RA1
2	7088666	7188666	0.410433	MYOM3
2	7088666	7188666	0.410433	IL22RA1

2	7089187	7189187	0.408972	MYOM3
2	7089187	7189187	0.408972	IL22RA1
2	7089533	7189533	0.408972	MYOM3
2	7089533	7189533	0.408972	IL22RA1
2	7089541	7189541	0.408972	MYOM3
2	7089541	7189541	0.408972	IL22RA1
2	7089617	7189617	0.412319	MYOM3
2	7089617	7189617	0.412319	IL22RA1
2	7089839	7189839	0.415422	MYOM3
2	7089839	7189839	0.415422	IL22RA1
2	7090023	7190023	0.412379	MYOM3
2	7090023	7190023	0.412379	IL22RA1
2	7090098	7190098	0.412786	MYOM3
2	7090098	7190098	0.412786	IL22RA1
2	7090100	7190100	0.412786	MYOM3
2	7090100	7190100	0.412786	IL22RA1
2	7090114	7190114	0.412683	MYOM3
2	7090114	7190114	0.412683	IL22RA1
2	7090174	7190174	0.410172	MYOM3
2	7090174	7190174	0.410172	IL22RA1
2	7090295	7190295	0.411385	MYOM3
2	7090295	7190295	0.411385	IL22RA1
2	7090514	7190514	0.414084	MYOM3
2	7090514	7190514	0.414084	IL22RA1
2	7090955	7190955	0.411978	MYOM3
2	7090955	7190955	0.411978	IL22RA1
2	7091010	7191010	0.412559	MYOM3
2	7091010	7191010	0.412559	IL22RA1
2	7091028	7191028	0.411715	MYOM3
2	7091028	7191028	0.411715	IL22RA1
2	7091279	7191279	0.41198	MYOM3
2	7091279	7191279	0.41198	IL22RA1
2	7091292	7191292	0.411781	MYOM3
2	7091292	7191292	0.411781	IL22RA1
2	7091317	7191317	0.4126	MYOM3
2	7091317	7191317	0.4126	IL22RA1
2	7091318	7191318	0.473265	MYOM3
2	7091318	7191318	0.473265	IL22RA1
2	7091510	7191510	0.435919	MYOM3
2	7091510	7191510	0.435919	IL22RA1
2	7091532	7191532	0.416191	MYOM3
2	7091532	7191532	0.416191	IL22RA1
2	7091965	7191965	0.41797	MYOM3
2	7091965	7191965	0.41797	IL22RA1
2	7092039	7192039	0.420725	MYOM3
2	7092039	7192039	0.420725	IL22RA1
2	7092162	7192162	0.420779	MYOM3
2	7092162	7192162	0.420779	IL22RA1
2	7092169	7192169	0.421016	MYOM3
2	7092169	7192169	0.421016	IL22RA1
2	7092342	7192342	0.40314	MYOM3
2	7092342	7192342	0.40314	IL22RA1
2	7092360	7192360	0.420677	MYOM3
2	7092360	7192360	0.420677	IL22RA1
2	7092444	7192444	0.421742	MYOM3
2	7092444	7192444	0.421742	IL22RA1
2	7092479	7192479	0.420337	MYOM3
2	7092479	7192479	0.420337	IL22RA1

2	7092657	7192657	0.399203	MYOM3
2	7092657	7192657	0.399203	IL22RA1
2	7092661	7192661	0.397927	MYOM3
2	7092661	7192661	0.397927	IL22RA1
2	7092851	7192851	0.39742	MYOM3
2	7092851	7192851	0.39742	IL22RA1
2	7092909	7192909	0.398459	MYOM3
2	7092909	7192909	0.398459	IL22RA1
2	7092962	7192962	0.396875	MYOM3
2	7092962	7192962	0.396875	IL22RA1
2	7093263	7193263	0.396028	MYOM3
2	7093263	7193263	0.396028	IL22RA1
2	7093465	7193465	0.399352	MYOM3
2	7093465	7193465	0.399352	IL22RA1
2	7093532	7193532	0.394937	MYOM3
2	7093532	7193532	0.394937	IL22RA1
2	7093767	7193767	0.394413	MYOM3
2	7093767	7193767	0.394413	IL22RA1
2	7093967	7193967	0.396446	MYOM3
2	7093967	7193967	0.396446	IL22RA1
2	7094118	7194118	0.396253	MYOM3
2	7094118	7194118	0.396253	IL22RA1
2	7094137	7194137	0.396253	MYOM3
2	7094137	7194137	0.396253	IL22RA1
2	7094145	7194145	0.397494	MYOM3
2	7094145	7194145	0.397494	IL22RA1
2	7094291	7194291	0.418351	MYOM3
2	7094291	7194291	0.418351	IL22RA1
2	7094425	7194425	0.403533	MYOM3
2	7094425	7194425	0.403533	IL22RA1
2	7094473	7194473	0.404296	MYOM3
2	7094473	7194473	0.404296	IL22RA1
2	7094500	7194500	0.404394	MYOM3
2	7094500	7194500	0.404394	IL22RA1
2	7094502	7194502	0.404394	MYOM3
2	7094502	7194502	0.404394	IL22RA1
2	7094677	7194677	0.401539	MYOM3
2	7094677	7194677	0.401539	IL22RA1
2	7094841	7194841	0.419926	MYOM3
2	7094841	7194841	0.419926	IL22RA1
2	7094992	7194992	0.408293	MYOM3
2	7094992	7194992	0.408293	IL22RA1
2	7095069	7195069	0.40766	MYOM3
2	7095069	7195069	0.40766	IL22RA1
2	7095279	7195279	0.407294	MYOM3
2	7095279	7195279	0.407294	IL22RA1
2	7095289	7195289	0.407966	MYOM3
2	7095289	7195289	0.407966	IL22RA1
2	7095297	7195297	0.40902	MYOM3
2	7095297	7195297	0.40902	IL22RA1
2	7095307	7195307	0.40902	MYOM3
2	7095307	7195307	0.40902	IL22RA1
2	7095456	7195456	0.401464	MYOM3
2	7095456	7195456	0.401464	IL22RA1
2	7095467	7195467	0.401464	MYOM3
2	7095467	7195467	0.401464	IL22RA1
2	7095510	7195510	0.401464	MYOM3
2	7095510	7195510	0.401464	IL22RA1

2	7095533	7195533	0.401464	MYOM3
2	7095533	7195533	0.401464	IL22RA1
2	7096650	7196650	0.39667	MYOM3
2	7096650	7196650	0.39667	IL22RA1
2	7096658	7196658	0.410039	MYOM3
2	7096658	7196658	0.410039	IL22RA1
2	7097219	7197219	0.409296	MYOM3
2	7097219	7197219	0.409296	IL22RA1
2	7097219	7197219	0.409296	IFNLR1
2	7097227	7197227	0.409296	MYOM3
2	7097227	7197227	0.409296	IL22RA1
2	7097227	7197227	0.409296	IFNLR1
2	7097264	7197264	0.409296	MYOM3
2	7097264	7197264	0.409296	IL22RA1
2	7097264	7197264	0.409296	IFNLR1
2	7097311	7197311	0.409296	MYOM3
2	7097311	7197311	0.409296	IL22RA1
2	7097311	7197311	0.409296	IFNLR1
2	7097855	7197855	0.421422	MYOM3
2	7097855	7197855	0.421422	IL22RA1
2	7097855	7197855	0.421422	IFNLR1
2	7097870	7197870	0.425603	MYOM3
2	7097870	7197870	0.425603	IL22RA1
2	7097870	7197870	0.425603	IFNLR1
2	7097885	7197885	0.425603	MYOM3
2	7097885	7197885	0.425603	IL22RA1
2	7097885	7197885	0.425603	IFNLR1
2	7097980	7197980	0.425603	MYOM3
2	7097980	7197980	0.425603	IL22RA1
2	7097980	7197980	0.425603	IFNLR1
2	7098025	7198025	0.425394	MYOM3
2	7098025	7198025	0.425394	IL22RA1
2	7098025	7198025	0.425394	IFNLR1
2	7098202	7198202	0.425566	MYOM3
2	7098202	7198202	0.425566	IL22RA1
2	7098202	7198202	0.425566	IFNLR1
2	7098256	7198256	0.429047	MYOM3
2	7098256	7198256	0.429047	IL22RA1
2	7098256	7198256	0.429047	IFNLR1
2	7098302	7198302	0.426545	MYOM3
2	7098302	7198302	0.426545	IL22RA1
2	7098302	7198302	0.426545	IFNLR1
2	7098507	7198507	0.424728	MYOM3
2	7098507	7198507	0.424728	IL22RA1
2	7098507	7198507	0.424728	IFNLR1
2	7098550	7198550	0.424728	MYOM3
2	7098550	7198550	0.424728	IL22RA1
2	7098550	7198550	0.424728	IFNLR1
2	7098909	7198909	0.426051	MYOM3
2	7098909	7198909	0.426051	IL22RA1
2	7098909	7198909	0.426051	IFNLR1
2	7098915	7198915	0.430391	MYOM3
2	7098915	7198915	0.430391	IL22RA1
2	7098915	7198915	0.430391	IFNLR1
2	7098938	7198938	0.430057	MYOM3
2	7098938	7198938	0.430057	IL22RA1
2	7098938	7198938	0.430057	IFNLR1
2	7098979	7198979	0.430057	MYOM3

2	7098979	7198979	0.430057	IL22RA1
2	7098979	7198979	0.430057	IFNLR1
2	7099186	7199186	0.426839	MYOM3
2	7099186	7199186	0.426839	IL22RA1
2	7099186	7199186	0.426839	IFNLR1
2	7099260	7199260	0.43795	MYOM3
2	7099260	7199260	0.43795	IL22RA1
2	7099260	7199260	0.43795	IFNLR1
2	7099440	7199440	0.436829	MYOM3
2	7099440	7199440	0.436829	IL22RA1
2	7099440	7199440	0.436829	IFNLR1
2	7099449	7199449	0.424369	MYOM3
2	7099449	7199449	0.424369	IL22RA1
2	7099449	7199449	0.424369	IFNLR1
2	7099702	7199702	0.423705	MYOM3
2	7099702	7199702	0.423705	IL22RA1
2	7099702	7199702	0.423705	IFNLR1
2	7099960	7199960	0.426827	MYOM3
2	7099960	7199960	0.426827	IL22RA1
2	7099960	7199960	0.426827	IFNLR1
2	7099971	7199971	0.420946	MYOM3
2	7099971	7199971	0.420946	IL22RA1
2	7099971	7199971	0.420946	IFNLR1
2	7100009	7200009	0.413405	MYOM3
2	7100009	7200009	0.413405	IL22RA1
2	7100009	7200009	0.413405	IFNLR1
2	7100024	7200024	0.413064	MYOM3
2	7100024	7200024	0.413064	IL22RA1
2	7100024	7200024	0.413064	IFNLR1
2	7100125	7200125	0.411473	MYOM3
2	7100125	7200125	0.411473	IL22RA1
2	7100125	7200125	0.411473	IFNLR1
2	7100312	7200312	0.398968	MYOM3
2	7100312	7200312	0.398968	IL22RA1
2	7100312	7200312	0.398968	IFNLR1
2	7100372	7200372	0.399017	MYOM3
2	7100372	7200372	0.399017	IL22RA1
2	7100372	7200372	0.399017	IFNLR1
2	7100482	7200482	0.396107	MYOM3
2	7100482	7200482	0.396107	IL22RA1
2	7100482	7200482	0.396107	IFNLR1
2	7100551	7200551	0.385714	MYOM3
2	7100551	7200551	0.385714	IL22RA1
2	7100551	7200551	0.385714	IFNLR1
2	7100756	7200756	0.385714	MYOM3
2	7100756	7200756	0.385714	IL22RA1
2	7100756	7200756	0.385714	IFNLR1
2	7100797	7200797	0.391025	MYOM3
2	7100797	7200797	0.391025	IL22RA1
2	7100797	7200797	0.391025	IFNLR1
2	7100804	7200804	0.343111	MYOM3
2	7100804	7200804	0.343111	IL22RA1
2	7100804	7200804	0.343111	IFNLR1
2	7101983	7201983	0.348181	MYOM3
2	7101983	7201983	0.348181	IL22RA1
2	7101983	7201983	0.348181	IFNLR1
2	7102009	7202009	0.350592	MYOM3
2	7102009	7202009	0.350592	IL22RA1

2	7102009	7202009	0.350592	IFNLR1
2	7102127	7202127	0.350045	MYOM3
2	7102127	7202127	0.350045	IL22RA1
2	7102127	7202127	0.350045	IFNLR1
2	7102148	7202148	0.352951	MYOM3
2	7102148	7202148	0.352951	IL22RA1
2	7102148	7202148	0.352951	IFNLR1
2	7102234	7202234	0.352133	MYOM3
2	7102234	7202234	0.352133	IL22RA1
2	7102234	7202234	0.352133	IFNLR1
2	7102357	7202357	0.34625	MYOM3
2	7102357	7202357	0.34625	IL22RA1
2	7102357	7202357	0.34625	IFNLR1
2	7102532	7202532	0.348321	MYOM3
2	7102532	7202532	0.348321	IL22RA1
2	7102532	7202532	0.348321	IFNLR1
2	7102535	7202535	0.342781	MYOM3
2	7102535	7202535	0.342781	IL22RA1
2	7102535	7202535	0.342781	IFNLR1
2	7102845	7202845	0.367654	MYOM3
2	7102845	7202845	0.367654	IL22RA1
2	7102845	7202845	0.367654	IFNLR1
2	7102985	7202985	0.345587	MYOM3
2	7102985	7202985	0.345587	IL22RA1
2	7102985	7202985	0.345587	IFNLR1
2	7103461	7203461	0.344227	MYOM3
2	7103461	7203461	0.344227	IL22RA1
2	7103461	7203461	0.344227	IFNLR1
2	14890158	14990158	0.350441	HPCA
2	14890158	14990158	0.350441	S100PBP
2	14890158	14990158	0.350441	FNDC5
2	14890158	14990158	0.350441	RNF19B
2	14890158	14990158	0.350441	TMEM54
2	14897480	14997480	0.346172	HPCA
2	14897480	14997480	0.346172	S100PBP
2	14897480	14997480	0.346172	FNDC5
2	14897480	14997480	0.346172	RNF19B
2	14897480	14997480	0.346172	TMEM54
2	14897570	14997570	0.357052	HPCA
2	14897570	14997570	0.357052	S100PBP
2	14897570	14997570	0.357052	FNDC5
2	14897570	14997570	0.357052	RNF19B
2	14897570	14997570	0.357052	TMEM54
2	15588352	15688352	0.372725	LOC102190876
2	15588352	15688352	0.372725	LOC102190919
2	15588352	15688352	0.372725	LOC102190650
2	15588352	15688352	0.372725	LOC102180168
2	15588352	15688352	0.372725	ECEL1
2	17208639	17308639	0.353189	LOC108633356
2	17208639	17308639	0.353189	LOC102186814
2	17208730	17308730	0.378462	LOC108633356
2	17208730	17308730	0.378462	LOC102186814
2	17208793	17308793	0.383082	LOC108633356
2	17208793	17308793	0.383082	LOC102186814
2	17208864	17308864	0.351403	LOC108633356
2	17208864	17308864	0.351403	LOC102186814
2	17209263	17309263	0.365364	LOC108633356
2	17209263	17309263	0.365364	LOC102186814

2	17209280	17309280	0.387612	LOC108633356
2	17209280	17309280	0.387612	LOC102186814
2	17209282	17309282	0.372635	LOC108633356
2	17209282	17309282	0.372635	LOC102186814
2	17209283	17309283	0.393649	LOC108633356
2	17209283	17309283	0.393649	LOC102186814
2	17209408	17309408	0.403849	LOC108633356
2	17209408	17309408	0.403849	LOC102186814
2	17209637	17309637	0.378033	LOC108633356
2	17209637	17309637	0.378033	LOC102186814
2	17209665	17309665	0.378033	LOC108633356
2	17209665	17309665	0.378033	LOC102186814
2	17210127	17310127	0.381165	LOC108633356
2	17210127	17310127	0.381165	LOC102186814
2	17210128	17310128	0.381165	LOC108633356
2	17210128	17310128	0.381165	LOC102186814
2	17210380	17310380	0.360622	LOC108633356
2	17210380	17310380	0.360622	LOC102186814
2	17210392	17310392	0.368373	LOC108633356
2	17210392	17310392	0.368373	LOC102186814
2	17210488	17310488	0.375033	LOC108633356
2	17210488	17310488	0.375033	LOC102186814
2	17210518	17310518	0.366791	LOC108633356
2	17210518	17310518	0.366791	LOC102186814
2	17210579	17310579	0.358019	LOC108633356
2	17210579	17310579	0.358019	LOC102186814
2	17211069	17311069	0.352465	LOC108633356
2	17211069	17311069	0.352465	LOC102186814
2	17211235	17311235	0.394729	LOC108633356
2	17211235	17311235	0.394729	LOC102186814
2	17211291	17311291	0.393402	LOC108633356
2	17211291	17311291	0.393402	LOC102186814
2	17211362	17311362	0.422326	LOC108633356
2	17211362	17311362	0.422326	LOC102186814
2	17211421	17311421	0.421192	LOC108633356
2	17211421	17311421	0.421192	LOC102186814
2	17211547	17311547	0.415274	LOC108633356
2	17211547	17311547	0.415274	LOC102186814
2	17211552	17311552	0.430942	LOC108633356
2	17211552	17311552	0.430942	LOC102186814
2	17211584	17311584	0.425238	LOC108633356
2	17211584	17311584	0.425238	LOC102186814
2	17211639	17311639	0.435184	LOC108633356
2	17211639	17311639	0.435184	LOC102186814
2	17211730	17311730	0.433032	LOC108633356
2	17211730	17311730	0.433032	LOC102186814
2	17211867	17311867	0.424217	LOC108633356
2	17211867	17311867	0.424217	LOC102186814
2	17211898	17311898	0.41753	LOC108633356
2	17211898	17311898	0.41753	LOC102186814
2	17211943	17311943	0.415877	LOC108633356
2	17211943	17311943	0.415877	LOC102186814
2	17212031	17312031	0.408606	LOC108633356
2	17212031	17312031	0.408606	LOC102186814
2	17212064	17312064	0.410528	LOC108633356
2	17212064	17312064	0.410528	LOC102186814
2	17212094	17312094	0.405223	LOC108633356
2	17212094	17312094	0.405223	LOC102186814

2	17212134	17312134	0.405223	LOC108633356
2	17212134	17312134	0.405223	LOC102186814
2	17212177	17312177	0.414161	LOC108633356
2	17212177	17312177	0.414161	LOC102186814
2	17212455	17312455	0.400954	LOC108633356
2	17212455	17312455	0.400954	LOC102186814
2	17212556	17312556	0.386253	LOC108633356
2	17212556	17312556	0.386253	LOC102186814
2	17212575	17312575	0.38748	LOC108633356
2	17212575	17312575	0.38748	LOC102186814
2	17212693	17312693	0.379452	LOC108633356
2	17212693	17312693	0.379452	LOC102186814
2	17212708	17312708	0.383872	LOC108633356
2	17212708	17312708	0.383872	LOC102186814
2	17212739	17312739	0.378039	LOC108633356
2	17212739	17312739	0.378039	LOC102186814
2	17212759	17312759	0.378077	LOC108633356
2	17212759	17312759	0.378077	LOC102186814
2	17212767	17312767	0.378077	LOC108633356
2	17212767	17312767	0.378077	LOC102186814
2	17212827	17312827	0.381847	LOC108633356
2	17212827	17312827	0.381847	LOC102186814
2	17212851	17312851	0.363173	LOC108633356
2	17212851	17312851	0.363173	LOC102186814
2	17212921	17312921	0.363173	LOC108633356
2	17212921	17312921	0.363173	LOC102186814
2	17212961	17312961	0.363173	LOC108633356
2	17212961	17312961	0.363173	LOC102186814
2	17213007	17313007	0.364828	LOC108633356
2	17213007	17313007	0.364828	LOC102186814
2	17214429	17314429	0.343941	LOC108633356
2	17214429	17314429	0.343941	LOC102186814
2	17214474	17314474	0.343388	LOC108633356
2	17214474	17314474	0.343388	LOC102186814
2	17214559	17314559	0.343093	LOC108633356
2	17214559	17314559	0.343093	LOC102186814
2	17214686	17314686	0.346953	LOC108633356
2	17214686	17314686	0.346953	LOC102186814
2	17214706	17314706	0.344159	LOC108633356
2	17214706	17314706	0.344159	LOC102186814
2	17214798	17314798	0.350981	LOC108633356
2	17214798	17314798	0.350981	LOC102186814
2	17214801	17314801	0.349957	LOC108633356
2	17214801	17314801	0.349957	LOC102186814
2	17215632	17315632	0.418165	LOC108633356
2	17215632	17315632	0.418165	LOC102186814
2	17215633	17315633	0.460537	LOC108633356
2	17215633	17315633	0.460537	LOC102186814
2	17215841	17315841	0.417686	LOC108633356
2	17215841	17315841	0.417686	LOC102186814
2	17215987	17315987	0.432804	LOC108633356
2	17215987	17315987	0.432804	LOC102186814
2	17215988	17315988	0.412128	LOC108633356
2	17215988	17315988	0.412128	LOC102186814
2	17216025	17316025	0.412128	LOC108633356
2	17216025	17316025	0.412128	LOC102186814
2	17216038	17316038	0.414145	LOC108633356
2	17216038	17316038	0.414145	LOC102186814

2	17216077	17316077	0.416891	LOC108633356
2	17216077	17316077	0.416891	LOC102186814
2	17216201	17316201	0.413853	LOC108633356
2	17216201	17316201	0.413853	LOC102186814
2	17216240	17316240	0.413853	LOC108633356
2	17216240	17316240	0.413853	LOC102186814
2	17216241	17316241	0.413853	LOC108633356
2	17216241	17316241	0.413853	LOC102186814
2	17216383	17316383	0.413985	LOC108633356
2	17216383	17316383	0.413985	LOC102186814
2	17216642	17316642	0.414107	LOC108633356
2	17216642	17316642	0.414107	LOC102186814
2	17216674	17316674	0.414107	LOC108633356
2	17216674	17316674	0.414107	LOC102186814
2	17216675	17316675	0.414107	LOC108633356
2	17216675	17316675	0.414107	LOC102186814
2	17216719	17316719	0.408113	LOC108633356
2	17216719	17316719	0.408113	LOC102186814
2	17217065	17317065	0.412616	LOC108633356
2	17217065	17317065	0.412616	LOC102186814
2	17217073	17317073	0.415469	LOC108633356
2	17217073	17317073	0.415469	LOC102186814
2	17217102	17317102	0.42939	LOC108633356
2	17217102	17317102	0.42939	LOC102186814
2	17217188	17317188	0.411461	LOC108633356
2	17217188	17317188	0.411461	LOC102186814
2	17217277	17317277	0.396543	LOC108633356
2	17217277	17317277	0.396543	LOC102186814
2	17217476	17317476	0.405071	LOC108633356
2	17217476	17317476	0.405071	LOC102186814
2	17217538	17317538	0.405071	LOC108633356
2	17217538	17317538	0.405071	LOC102186814
2	17217642	17317642	0.405071	LOC108633356
2	17217642	17317642	0.405071	LOC102186814
2	17217668	17317668	0.399378	LOC108633356
2	17217668	17317668	0.399378	LOC102186814
2	17217929	17317929	0.397906	LOC108633356
2	17217929	17317929	0.397906	LOC102186814
2	17217993	17317993	0.397906	LOC108633356
2	17217993	17317993	0.397906	LOC102186814
2	17218103	17318103	0.397906	LOC108633356
2	17218103	17318103	0.397906	LOC102186814
2	17218270	17318270	0.406082	LOC108633356
2	17218270	17318270	0.406082	LOC102186814
2	17218277	17318277	0.406726	LOC108633356
2	17218277	17318277	0.406726	LOC102186814
2	17218321	17318321	0.406404	LOC108633356
2	17218321	17318321	0.406404	LOC102186814
2	17218362	17318362	0.404572	LOC108633356
2	17218362	17318362	0.404572	LOC102186814
2	17218430	17318430	0.408255	LOC108633356
2	17218430	17318430	0.408255	LOC102186814
2	17218435	17318435	0.408255	LOC108633356
2	17218435	17318435	0.408255	LOC102186814
2	17218477	17318477	0.407245	LOC108633356
2	17218477	17318477	0.407245	LOC102186814
2	17218548	17318548	0.40121	LOC108633356
2	17218548	17318548	0.40121	LOC102186814

2	17218553	17318553	0.398805	LOC108633356
2	17218553	17318553	0.398805	LOC102186814
2	17218624	17318624	0.404452	LOC108633356
2	17218624	17318624	0.404452	LOC102186814
2	17218626	17318626	0.404452	LOC108633356
2	17218626	17318626	0.404452	LOC102186814
2	17218709	17318709	0.440854	LOC108633356
2	17218709	17318709	0.440854	LOC102186814
2	17218872	17318872	0.43899	LOC108633356
2	17218872	17318872	0.43899	LOC102186814
2	17218918	17318918	0.433982	LOC108633356
2	17218918	17318918	0.433982	LOC102186814
2	17218943	17318943	0.42684	LOC108633356
2	17218943	17318943	0.42684	LOC102186814
2	17219140	17319140	0.393277	LOC108633356
2	17219140	17319140	0.393277	LOC102186814
2	17219178	17319178	0.400033	LOC108633356
2	17219178	17319178	0.400033	LOC102186814
2	17219182	17319182	0.403263	LOC108633356
2	17219182	17319182	0.403263	LOC102186814
2	17219190	17319190	0.402017	LOC108633356
2	17219190	17319190	0.402017	LOC102186814
2	17219216	17319216	0.402017	LOC108633356
2	17219216	17319216	0.402017	LOC102186814
2	17219260	17319260	0.402017	LOC108633356
2	17219260	17319260	0.402017	LOC102186814
2	17219261	17319261	0.402017	LOC108633356
2	17219261	17319261	0.402017	LOC102186814
2	17219267	17319267	0.403186	LOC108633356
2	17219267	17319267	0.403186	LOC102186814
2	17219337	17319337	0.404852	LOC108633356
2	17219337	17319337	0.404852	LOC102186814
2	17219403	17319403	0.382005	LOC108633356
2	17219403	17319403	0.382005	LOC102186814
2	17219439	17319439	0.383596	LOC108633356
2	17219439	17319439	0.383596	LOC102186814
2	17219441	17319441	0.383596	LOC108633356
2	17219441	17319441	0.383596	LOC102186814
2	17219489	17319489	0.383596	LOC108633356
2	17219489	17319489	0.383596	LOC102186814
2	17219604	17319604	0.380043	LOC108633356
2	17219604	17319604	0.380043	LOC102186814
2	17219660	17319660	0.375778	LOC108633356
2	17219660	17319660	0.375778	LOC102186814
2	17219693	17319693	0.379176	LOC108633356
2	17219693	17319693	0.379176	LOC102186814
2	17219721	17319721	0.354641	LOC108633356
2	17219721	17319721	0.354641	LOC102186814
2	17219748	17319748	0.354641	LOC108633356
2	17219748	17319748	0.354641	LOC102186814
2	17219857	17319857	0.354664	LOC108633356
2	17219857	17319857	0.354664	LOC102186814
2	17219968	17319968	0.365337	LOC108633356
2	17219968	17319968	0.365337	LOC102186814
2	17220037	17320037	0.365337	LOC108633356
2	17220037	17320037	0.365337	LOC102186814
2	17220070	17320070	0.363658	LOC108633356
2	17220070	17320070	0.363658	LOC102186814

2	17220083	17320083	0.361886	LOC108633356
2	17220083	17320083	0.361886	LOC102186814
2	17220121	17320121	0.356451	LOC108633356
2	17220121	17320121	0.356451	LOC102186814
2	17220122	17320122	0.35316	LOC108633356
2	17220122	17320122	0.35316	LOC102186814
2	17220238	17320238	0.353626	LOC102186814
2	17220247	17320247	0.353626	LOC102186814
2	17220464	17320464	0.347422	LOC102186814
2	17220629	17320629	0.342559	LOC102186814
2	17220633	17320633	0.342559	LOC102186814
2	17220711	17320711	0.346898	LOC102186814
2	17220732	17320732	0.346898	LOC102186814
2	17220779	17320779	0.351443	LOC102186814
2	17233980	17333980	0.379757	LOC102186814
2	17908429	18008429	0.349192	DNER
2	17908429	18008429	0.349192	TRIP12
2	17908443	18008443	0.386269	DNER
2	17908443	18008443	0.386269	TRIP12
2	17908665	18008665	0.425051	DNER
2	17908665	18008665	0.425051	TRIP12
2	17908912	18008912	0.3573	DNER
2	17908912	18008912	0.3573	TRIP12
2	17909094	18009094	0.357543	DNER
2	17909094	18009094	0.357543	TRIP12
2	17909109	18009109	0.377748	DNER
2	17909109	18009109	0.377748	TRIP12
2	17909169	18009169	0.35812	DNER
2	17909169	18009169	0.35812	TRIP12
2	17909347	18009347	0.353921	DNER
2	17909347	18009347	0.353921	TRIP12
2	17909349	18009349	0.353921	DNER
2	17909349	18009349	0.353921	TRIP12
2	17909440	18009440	0.354931	DNER
2	17909440	18009440	0.354931	TRIP12
2	17909797	18009797	0.348808	DNER
2	17909797	18009797	0.348808	TRIP12
2	17910262	18010262	0.370117	DNER
2	17910262	18010262	0.370117	TRIP12
2	17910337	18010337	0.370117	DNER
2	17910337	18010337	0.370117	TRIP12
2	17910418	18010418	0.373837	DNER
2	17910418	18010418	0.373837	TRIP12
2	17910652	18010652	0.385162	DNER
2	17910652	18010652	0.385162	TRIP12
2	17910944	18010944	0.361747	DNER
2	17910944	18010944	0.361747	TRIP12
2	17911039	18011039	0.381524	DNER
2	17911039	18011039	0.381524	TRIP12
2	17911234	18011234	0.383541	DNER
2	17911234	18011234	0.383541	TRIP12
2	17911530	18011530	0.405713	DNER
2	17911530	18011530	0.405713	TRIP12
2	17911595	18011595	0.403224	DNER
2	17911595	18011595	0.403224	TRIP12
2	17911629	18011629	0.378812	DNER
2	17911629	18011629	0.378812	TRIP12
2	17911782	18011782	0.352681	DNER

2	17911782	18011782	0.352681	TRIP12
2	17911891	18011891	0.350141	DNER
2	17911891	18011891	0.350141	TRIP12
2	17912203	18012203	0.372632	DNER
2	17912203	18012203	0.372632	TRIP12
2	17912294	18012294	0.372632	DNER
2	17912294	18012294	0.372632	TRIP12
2	17912386	18012386	0.375347	DNER
2	17912386	18012386	0.375347	TRIP12
2	17912545	18012545	0.379743	DNER
2	17912545	18012545	0.379743	TRIP12
2	17912586	18012586	0.370488	DNER
2	17912586	18012586	0.370488	TRIP12
2	17912694	18012694	0.381961	DNER
2	17912694	18012694	0.381961	TRIP12
2	17912740	18012740	0.367506	DNER
2	17912740	18012740	0.367506	TRIP12
2	17912898	18012898	0.363946	DNER
2	17912898	18012898	0.363946	TRIP12
2	17912933	18012933	0.363946	DNER
2	17912933	18012933	0.363946	TRIP12
2	17912940	18012940	0.363946	DNER
2	17912940	18012940	0.363946	TRIP12
2	17912987	18012987	0.366803	DNER
2	17912987	18012987	0.366803	TRIP12
2	17913134	18013134	0.344809	DNER
2	17913134	18013134	0.344809	TRIP12
2	18353581	18453581	0.345666	PID1
2	18361642	18461642	0.341661	PID1
2	18361659	18461659	0.344606	PID1
2	18361666	18461666	0.343757	PID1
2	18361789	18461789	0.347988	PID1
2	18365615	18465615	0.354534	PID1
2	18365723	18465723	0.351167	PID1
2	18365770	18465770	0.347923	PID1
2	18365781	18465781	0.349145	PID1
2	18399902	18499902	0.368427	PID1
2	18399949	18499949	0.400321	PID1
2	18400045	18500045	0.400321	PID1
2	18400068	18500068	0.411763	PID1
2	18400082	18500082	0.424424	PID1
2	18400227	18500227	0.478514	PID1
2	18400310	18500310	0.480055	PID1
2	18400417	18500417	0.467558	PID1
2	18400445	18500445	0.451083	PID1
2	18400484	18500484	0.488018	PID1
2	18400549	18500549	0.513279	PID1
2	18400646	18500646	0.487016	PID1
2	18400755	18500755	0.478489	PID1
2	18400759	18500759	0.473128	PID1
2	18400809	18500809	0.461706	PID1
2	18400849	18500849	0.422732	PID1
2	18400869	18500869	0.43051	PID1
2	18401196	18501196	0.438321	PID1
2	18401490	18501490	0.406941	PID1
2	18401500	18501500	0.393947	PID1
2	18401819	18501819	0.380268	PID1
2	18401856	18501856	0.377656	PID1

2	18401962	18501962	0.403076	PID1
2	18402032	18502032	0.403076	PID1
2	18402151	18502151	0.366287	PID1
2	18402159	18502159	0.362317	PID1
2	18402378	18502378	0.373557	PID1
2	18402404	18502404	0.39282	PID1
2	18402420	18502420	0.39282	PID1
2	18402485	18502485	0.39282	PID1
2	18402538	18502538	0.393665	PID1
2	18402749	18502749	0.393214	PID1
2	18403327	18503327	0.393214	PID1
2	18403571	18503571	0.393214	PID1
2	18403580	18503580	0.393214	PID1
2	18403632	18503632	0.394267	PID1
2	18403739	18503739	0.391555	PID1
2	18403788	18503788	0.403768	PID1
2	18403874	18503874	0.402814	PID1
2	18403880	18503880	0.402814	PID1
2	18404014	18504014	0.403273	PID1
2	18404261	18504261	0.403273	PID1
2	18404393	18504393	0.396081	PID1
2	18404935	18504935	0.411566	PID1
2	18404960	18504960	0.385528	PID1
2	18405324	18505324	0.416699	PID1
2	18405333	18505333	0.362238	PID1
2	18405408	18505408	0.357076	PID1
2	18405649	18505649	0.344239	PID1
2	18405789	18505789	0.353621	PID1
2	18405875	18505875	0.353621	PID1
2	18405942	18505942	0.353621	PID1
2	18405975	18505975	0.353621	PID1
2	18405984	18505984	0.407285	PID1
2	18406163	18506163	0.368523	PID1
2	18406292	18506292	0.368182	PID1
2	18406293	18506293	0.356315	PID1
2	18407242	18507242	0.346063	PID1
2	18407742	18507742	0.356355	PID1
2	18407886	18507886	0.356689	PID1
2	18408003	18508003	0.358915	PID1
2	18408141	18508141	0.426893	PID1
2	18408325	18508325	0.405206	PID1
2	18408407	18508407	0.433305	PID1
2	18408458	18508458	0.421487	PID1
2	18408526	18508526	0.388836	PID1
2	18409362	18509362	0.364648	PID1
2	18409403	18509403	0.38135	PID1
2	18409524	18509524	0.386073	PID1
2	18409547	18509547	0.380006	PID1
2	18409678	18509678	0.41607	PID1
2	18409711	18509711	0.476987	PID1
2	18409712	18509712	0.491549	PID1
2	18409741	18509741	0.482338	PID1
2	18409961	18509961	0.470325	PID1
2	18410020	18510020	0.524371	PID1
2	18410099	18510099	0.439263	PID1
2	18410438	18510438	0.417446	PID1
2	18410484	18510484	0.417446	PID1
2	18410533	18510533	0.412077	PID1

2	18410630	18510630	0.456667	PID1
2	18410795	18510795	0.446455	PID1
2	18410810	18510810	0.461368	PID1
2	18410852	18510852	0.442323	PID1
2	18410967	18510967	0.445679	PID1
2	18410982	18510982	0.445679	PID1
2	18411109	18511109	0.456579	PID1
2	18411217	18511217	0.464575	PID1
2	18411444	18511444	0.464575	PID1
2	18411494	18511494	0.464575	PID1
2	18411546	18511546	0.464575	PID1
2	18411578	18511578	0.464721	PID1
2	18411594	18511594	0.464954	PID1
2	18411739	18511739	0.465692	PID1
2	18411740	18511740	0.465692	PID1
2	18411862	18511862	0.465798	PID1
2	18411869	18511869	0.463836	PID1
2	18411987	18511987	0.498704	PID1
2	18412034	18512034	0.457964	PID1
2	18412092	18512092	0.478829	PID1
2	18412124	18512124	0.382655	PID1
2	18415237	18515237	0.415781	PID1
2	18415297	18515297	0.362053	PID1
2	18415633	18515633	0.357877	PID1
2	18415688	18515688	0.372547	PID1
2	18416085	18516085	0.393455	PID1
2	18416135	18516135	0.378146	PID1
2	18416152	18516152	0.396137	PID1
2	18416223	18516223	0.355067	PID1
2	18416283	18516283	0.364607	PID1
2	18416284	18516284	0.364607	PID1
2	18416464	18516464	0.430342	PID1
2	18416518	18516518	0.406273	PID1
2	18416654	18516654	0.360944	PID1
2	18416923	18516923	0.408954	PID1
2	18417397	18517397	0.465205	PID1
2	18417453	18517453	0.48502	PID1
2	18417456	18517456	0.485098	PID1
2	18417477	18517477	0.493897	PID1
2	18417492	18517492	0.497553	PID1
2	18417493	18517493	0.418049	PID1
2	18417529	18517529	0.483157	PID1
2	18417541	18517541	0.464481	PID1
2	18417646	18517646	0.449426	PID1
2	18417682	18517682	0.445144	PID1
2	18417712	18517712	0.474558	PID1
2	18417804	18517804	0.474558	PID1
2	18417840	18517840	0.425507	PID1
2	18417865	18517865	0.448144	PID1
2	18418053	18518053	0.341965	PID1
2	18418228	18518228	0.344965	PID1
2	18418293	18518293	0.345931	PID1
2	18420226	18520226	0.382247	PID1
2	18420724	18520724	0.381883	PID1
2	18420892	18520892	0.369523	PID1
2	18420910	18520910	0.395401	PID1
2	18426894	18526894	0.37122	PID1
2	18427104	18527104	0.368755	PID1

2	18427124	18527124	0.35137	PID1
2	18427383	18527383	0.34411	PID1
2	18427385	18527385	0.34411	PID1
2	18429072	18529072	0.361995	PID1
2	18563614	18663614	0.357457	PID1
2	18563768	18663768	0.343631	PID1
2	19887759	19987759	0.346018	SLC19A3
2	19887759	19987759	0.346018	LOC108638522
2	19887761	19987761	0.353449	SLC19A3
2	19887761	19987761	0.353449	LOC108638522
2	19887776	19987776	0.352769	SLC19A3
2	19887776	19987776	0.352769	LOC108638522
2	19887850	19987850	0.342786	SLC19A3
2	19887850	19987850	0.342786	LOC108638522
2	19887892	19987892	0.362112	SLC19A3
2	19887892	19987892	0.362112	LOC108638522
2	19887893	19987893	0.363169	SLC19A3
2	19887893	19987893	0.363169	LOC108638522
2	19887917	19987917	0.397221	SLC19A3
2	19887917	19987917	0.397221	LOC108638522
2	19887936	19987936	0.377409	SLC19A3
2	19887936	19987936	0.377409	LOC108638522
2	19887983	19987983	0.383109	SLC19A3
2	19887983	19987983	0.383109	LOC108638522
2	19888008	19988008	0.387355	SLC19A3
2	19888008	19988008	0.387355	LOC108638522
2	19888063	19988063	0.384099	SLC19A3
2	19888063	19988063	0.384099	LOC108638522
2	19888102	19988102	0.357297	SLC19A3
2	19888102	19988102	0.357297	LOC108638522
2	19946132	20046132	0.354195	LOC108638522
2	19946132	20046132	0.354195	AGFG1
2	19946307	20046307	0.347716	LOC108638522
2	19946307	20046307	0.347716	AGFG1
2	20455288	20555288	0.364361	COL4A4
2	20456260	20556260	0.360021	COL4A4
2	20456473	20556473	0.375007	COL4A4
2	20456507	20556507	0.363468	COL4A4
2	20457599	20557599	0.361763	COL4A4
2	20458469	20558469	0.368538	COL4A4
2	20458579	20558579	0.370191	COL4A4
2	20458638	20558638	0.382793	COL4A4
2	20459241	20559241	0.37238	COL4A4
2	20459424	20559424	0.37238	COL4A4
2	20459549	20559549	0.37238	COL4A4
2	20459637	20559637	0.37238	COL4A4
2	20459755	20559755	0.381359	COL4A4
2	20459974	20559974	0.379478	COL4A4
2	20460061	20560061	0.379478	COL4A4
2	20460080	20560080	0.379478	COL4A4
2	20460161	20560161	0.379478	COL4A4
2	20460296	20560296	0.40344	COL4A4
2	20460300	20560300	0.416169	COL4A4
2	20460321	20560321	0.40448	COL4A4
2	20460968	20560968	0.400415	COL4A4
2	20461676	20561676	0.403123	COL4A4
2	20461732	20561732	0.403123	COL4A4
2	20461899	20561899	0.41098	COL4A4

2	20462047	20562047	0.41098	COL4A4
2	20462181	20562181	0.41098	COL4A4
2	20462364	20562364	0.391	COL4A4
2	20462364	20562364	0.391	RHBDD1
2	20462759	20562759	0.406376	COL4A4
2	20462759	20562759	0.406376	RHBDD1
2	20462760	20562760	0.406376	COL4A4
2	20462760	20562760	0.406376	RHBDD1
2	20462933	20562933	0.406376	COL4A4
2	20462933	20562933	0.406376	RHBDD1
2	20462946	20562946	0.41574	COL4A4
2	20462946	20562946	0.41574	RHBDD1
2	20462957	20562957	0.405382	COL4A4
2	20462957	20562957	0.405382	RHBDD1
2	20463086	20563086	0.405382	COL4A4
2	20463086	20563086	0.405382	RHBDD1
2	20463296	20563296	0.405382	COL4A4
2	20463296	20563296	0.405382	RHBDD1
2	20463595	20563595	0.413986	COL4A4
2	20463595	20563595	0.413986	RHBDD1
2	20463670	20563670	0.41024	COL4A4
2	20463670	20563670	0.41024	RHBDD1
2	20463764	20563764	0.419297	COL4A4
2	20463764	20563764	0.419297	RHBDD1
2	20463798	20563798	0.414296	COL4A4
2	20463798	20563798	0.414296	RHBDD1
2	20463864	20563864	0.414296	COL4A4
2	20463864	20563864	0.414296	RHBDD1
2	20463879	20563879	0.438292	COL4A4
2	20463879	20563879	0.438292	RHBDD1
2	20463887	20563887	0.438292	COL4A4
2	20463887	20563887	0.438292	RHBDD1
2	20464313	20564313	0.433402	COL4A4
2	20464313	20564313	0.433402	RHBDD1
2	20464434	20564434	0.45487	COL4A4
2	20464434	20564434	0.45487	RHBDD1
2	20464542	20564542	0.437833	COL4A4
2	20464542	20564542	0.437833	RHBDD1
2	20464881	20564881	0.445411	COL4A4
2	20464881	20564881	0.445411	RHBDD1
2	20465116	20565116	0.440959	COL4A4
2	20465116	20565116	0.440959	RHBDD1
2	20465867	20565867	0.422117	COL4A4
2	20465867	20565867	0.422117	RHBDD1
2	20465961	20565961	0.421386	COL4A4
2	20465961	20565961	0.421386	RHBDD1
2	20466000	20566000	0.437823	COL4A4
2	20466000	20566000	0.437823	RHBDD1
2	20466001	20566001	0.437823	COL4A4
2	20466001	20566001	0.437823	RHBDD1
2	20466021	20566021	0.443811	COL4A4
2	20466021	20566021	0.443811	RHBDD1
2	20466057	20566057	0.43737	COL4A4
2	20466057	20566057	0.43737	RHBDD1
2	20466118	20566118	0.442806	COL4A4
2	20466118	20566118	0.442806	RHBDD1
2	20466363	20566363	0.43233	COL4A4
2	20466363	20566363	0.43233	RHBDD1

2	20466372	20566372	0.434002	COL4A4
2	20466372	20566372	0.434002	RHBDD1
2	20466531	20566531	0.401028	COL4A4
2	20466531	20566531	0.401028	RHBDD1
2	20466905	20566905	0.373892	COL4A4
2	20466905	20566905	0.373892	RHBDD1
2	20466983	20566983	0.365735	COL4A4
2	20466983	20566983	0.365735	RHBDD1
2	20466991	20566991	0.365735	COL4A4
2	20466991	20566991	0.365735	RHBDD1
2	20467150	20567150	0.374535	COL4A4
2	20467150	20567150	0.374535	RHBDD1
2	20467174	20567174	0.374033	COL4A4
2	20467174	20567174	0.374033	RHBDD1
2	20467611	20567611	0.375538	COL4A4
2	20467611	20567611	0.375538	RHBDD1
2	20467707	20567707	0.404807	COL4A4
2	20467707	20567707	0.404807	RHBDD1
2	20468068	20568068	0.375047	COL4A4
2	20468068	20568068	0.375047	RHBDD1
2	20468083	20568083	0.375224	COL4A4
2	20468083	20568083	0.375224	RHBDD1
2	20468093	20568093	0.372532	COL4A4
2	20468093	20568093	0.372532	RHBDD1
2	20468179	20568179	0.37206	COL4A4
2	20468179	20568179	0.37206	RHBDD1
2	20468199	20568199	0.390776	COL4A4
2	20468199	20568199	0.390776	RHBDD1
2	20468220	20568220	0.378451	COL4A4
2	20468220	20568220	0.378451	RHBDD1
2	20468241	20568241	0.379389	COL4A4
2	20468241	20568241	0.379389	RHBDD1
2	20468476	20568476	0.388931	COL4A4
2	20468476	20568476	0.388931	RHBDD1
2	20468507	20568507	0.389146	COL4A4
2	20468507	20568507	0.389146	RHBDD1
2	20468510	20568510	0.398958	COL4A4
2	20468510	20568510	0.398958	RHBDD1
2	20468567	20568567	0.391126	COL4A4
2	20468567	20568567	0.391126	RHBDD1
2	20468680	20568680	0.427796	COL4A4
2	20468680	20568680	0.427796	RHBDD1
2	20468708	20568708	0.420838	COL4A4
2	20468708	20568708	0.420838	RHBDD1
2	20468759	20568759	0.420071	COL4A4
2	20468759	20568759	0.420071	RHBDD1
2	20468782	20568782	0.420071	COL4A4
2	20468782	20568782	0.420071	RHBDD1
2	20468786	20568786	0.420071	COL4A4
2	20468786	20568786	0.420071	RHBDD1
2	20468842	20568842	0.420004	COL4A4
2	20468842	20568842	0.420004	RHBDD1
2	20468923	20568923	0.420023	COL4A4
2	20468923	20568923	0.420023	RHBDD1
2	20469011	20569011	0.44122	COL4A4
2	20469011	20569011	0.44122	RHBDD1
2	20469143	20569143	0.427945	COL4A4
2	20469143	20569143	0.427945	RHBDD1

2	20469225	20569225	0.431559	COL4A4
2	20469225	20569225	0.431559	RHBDD1
2	20469246	20569246	0.415309	COL4A4
2	20469246	20569246	0.415309	RHBDD1
2	20469482	20569482	0.412792	COL4A4
2	20469482	20569482	0.412792	RHBDD1
2	20469559	20569559	0.414932	COL4A4
2	20469559	20569559	0.414932	RHBDD1
2	20469681	20569681	0.416592	COL4A4
2	20469681	20569681	0.416592	RHBDD1
2	20469697	20569697	0.428642	COL4A4
2	20469697	20569697	0.428642	RHBDD1
2	20469698	20569698	0.426238	COL4A4
2	20469698	20569698	0.426238	RHBDD1
2	20469703	20569703	0.423724	COL4A4
2	20469703	20569703	0.423724	RHBDD1
2	20469751	20569751	0.452896	COL4A4
2	20469751	20569751	0.452896	RHBDD1
2	20469826	20569826	0.412886	COL4A4
2	20469826	20569826	0.412886	RHBDD1
2	20469827	20569827	0.41274	COL4A4
2	20469827	20569827	0.41274	RHBDD1
2	20469991	20569991	0.411505	COL4A4
2	20469991	20569991	0.411505	RHBDD1
2	20470005	20570005	0.418108	COL4A4
2	20470005	20570005	0.418108	RHBDD1
2	20470121	20570121	0.408366	COL4A4
2	20470121	20570121	0.408366	RHBDD1
2	20470181	20570181	0.408366	COL4A4
2	20470181	20570181	0.408366	RHBDD1
2	20470199	20570199	0.422674	COL4A4
2	20470199	20570199	0.422674	RHBDD1
2	20470259	20570259	0.41516	COL4A4
2	20470259	20570259	0.41516	RHBDD1
2	20470323	20570323	0.41516	COL4A4
2	20470323	20570323	0.41516	RHBDD1
2	20470385	20570385	0.41516	COL4A4
2	20470385	20570385	0.41516	RHBDD1
2	20470533	20570533	0.414075	COL4A4
2	20470533	20570533	0.414075	RHBDD1
2	20470753	20570753	0.414075	COL4A4
2	20470753	20570753	0.414075	RHBDD1
2	20470860	20570860	0.436123	COL4A4
2	20470860	20570860	0.436123	RHBDD1
2	20470898	20570898	0.444836	COL4A4
2	20470898	20570898	0.444836	RHBDD1
2	20470938	20570938	0.431635	COL4A4
2	20470938	20570938	0.431635	RHBDD1
2	20471037	20571037	0.43203	COL4A4
2	20471037	20571037	0.43203	RHBDD1
2	20471428	20571428	0.398779	COL4A4
2	20471428	20571428	0.398779	RHBDD1
2	20471523	20571523	0.381686	COL4A4
2	20471523	20571523	0.381686	RHBDD1
2	20471970	20571970	0.386609	COL4A4
2	20471970	20571970	0.386609	RHBDD1
2	20474315	20574315	0.405406	COL4A4
2	20474315	20574315	0.405406	RHBDD1

2	20485691	20585691	0.363907	COL4A4
2	20485691	20585691	0.363907	RHBDD1
2	21987795	22087795	0.343066	NYAP2
2	22844734	22944734	0.348047	DOCK10
2	23134687	23234687	0.344383	CUL3
2	23150699	23250699	0.342496	FAM124B
2	23150699	23250699	0.342496	CUL3
2	23153239	23253239	0.345092	FAM124B
2	23153239	23253239	0.345092	CUL3
2	23154113	23254113	0.344395	FAM124B
2	23154113	23254113	0.344395	CUL3
2	23154152	23254152	0.352988	FAM124B
2	23154152	23254152	0.352988	CUL3
2	23154190	23254190	0.352391	FAM124B
2	23154190	23254190	0.352391	CUL3
2	23154447	23254447	0.352158	FAM124B
2	23154447	23254447	0.352158	CUL3
2	23154522	23254522	0.35102	FAM124B
2	23154522	23254522	0.35102	CUL3
2	23154665	23254665	0.348212	FAM124B
2	23154665	23254665	0.348212	CUL3
2	23156218	23256218	0.344564	FAM124B
2	23156218	23256218	0.344564	CUL3
2	23156721	23256721	0.343747	FAM124B
2	23156721	23256721	0.343747	CUL3
2	23157649	23257649	0.38523	FAM124B
2	23157649	23257649	0.38523	CUL3
2	23158570	23258570	0.373925	FAM124B
2	23158570	23258570	0.373925	CUL3
2	23158857	23258857	0.374714	FAM124B
2	23158857	23258857	0.374714	CUL3
2	23158886	23258886	0.359051	FAM124B
2	23158886	23258886	0.359051	CUL3
2	23165475	23265475	0.34553	FAM124B
2	23165475	23265475	0.34553	CUL3
2	23165598	23265598	0.343465	FAM124B
2	23165598	23265598	0.343465	CUL3
2	23165891	23265891	0.342499	FAM124B
2	23165891	23265891	0.342499	CUL3
2	23166484	23266484	0.341764	FAM124B
2	23167614	23267614	0.351996	FAM124B
2	23167696	23267696	0.356112	FAM124B
2	23167777	23267777	0.351596	FAM124B
2	23167803	23267803	0.354013	FAM124B
2	23167849	23267849	0.354995	FAM124B
2	23168314	23268314	0.351986	FAM124B
2	23168435	23268435	0.342719	FAM124B
2	23168572	23268572	0.42346	FAM124B
2	23168743	23268743	0.410674	FAM124B
2	23168791	23268791	0.414586	FAM124B
2	23168843	23268843	0.405528	FAM124B
2	23169016	23269016	0.414108	FAM124B
2	23169443	23269443	0.415064	FAM124B
2	23169932	23269932	0.415139	FAM124B
2	23169998	23269998	0.406418	FAM124B
2	23170241	23270241	0.407889	FAM124B
2	23170257	23270257	0.390925	FAM124B
2	23170270	23270270	0.389921	FAM124B

2	23170365	23270365	0.389226	FAM124B
2	23171015	23271015	0.388441	FAM124B
2	23171243	23271243	0.371068	FAM124B
2	23172167	23272167	0.344213	FAM124B
2	23172168	23272168	0.344386	FAM124B
2	23172175	23272175	0.344386	FAM124B
2	23172232	23272232	0.343861	FAM124B
2	23172262	23272262	0.360307	FAM124B
2	23172564	23272564	0.344185	FAM124B
2	23178110	23278110	0.370419	FAM124B
2	23178251	23278251	0.361257	FAM124B
2	29074331	29174331	0.347216	RNF25
2	29074331	29174331	0.347216	STK36
2	29074331	29174331	0.347216	TTLL4
2	29074983	29174983	0.345642	RNF25
2	29074983	29174983	0.345642	STK36
2	29074983	29174983	0.345642	TTLL4
2	39201498	39301498	0.355689	PTH2R
2	39201498	39301498	0.355689	PIKFYVE
2	40517207	40617207	0.376966	KLF7
2	40517215	40617215	0.37085	KLF7
2	40518807	40618807	0.347886	KLF7
2	43721122	43821122	0.351201	ICOS
2	51523754	51623754	0.349042	SLC39A10
2	51523754	51623754	0.349042	DNAH7
2	51523803	51623803	0.370395	SLC39A10
2	51523803	51623803	0.370395	DNAH7
2	51523804	51623804	0.370395	SLC39A10
2	51523804	51623804	0.370395	DNAH7
2	51523903	51623903	0.362364	SLC39A10
2	51523903	51623903	0.362364	DNAH7
2	65992715	66092715	0.34813	TRNAC-GCA-27
2	65993021	66093021	0.367482	TRNAC-GCA-27
2	65993054	66093054	0.381825	TRNAC-GCA-27
2	65993060	66093060	0.380715	TRNAC-GCA-27
2	65993120	66093120	0.37226	TRNAC-GCA-27
2	65993131	66093131	0.37019	TRNAC-GCA-27
2	65993194	66093194	0.368978	TRNAC-GCA-27
2	65993206	66093206	0.376853	TRNAC-GCA-27
2	65993226	66093226	0.381249	TRNAC-GCA-27
2	65993339	66093339	0.37532	TRNAC-GCA-27
2	65993521	66093521	0.379631	TRNAC-GCA-27
2	65993537	66093537	0.369722	TRNAC-GCA-27
2	65993549	66093549	0.376082	TRNAC-GCA-27
2	65993611	66093611	0.371623	TRNAC-GCA-27
2	65993651	66093651	0.370859	TRNAC-GCA-27
2	65993653	66093653	0.370859	TRNAC-GCA-27
2	65993659	66093659	0.370859	TRNAC-GCA-27
2	65993699	66093699	0.371715	TRNAC-GCA-27
2	65993720	66093720	0.360042	TRNAC-GCA-27
2	65993885	66093885	0.342131	TRNAC-GCA-27
2	65994668	66094668	0.344941	TRNAC-GCA-27
2	65995008	66095008	0.372431	TRNAC-GCA-27
2	65995099	66095099	0.356769	TRNAC-GCA-27
2	71208166	71308166	0.36426	GPR39
2	71208176	71308176	0.36804	GPR39
2	71208206	71308206	0.39221	GPR39
2	72317306	72417306	0.364966	TRNAG-GCC-2

2	72317306	72417306	0.364966	LOC108637976
2	72317306	72417306	0.364966	NCKAP5
2	72317307	72417307	0.364966	TRNAG-GCC-2
2	72317307	72417307	0.364966	LOC108637976
2	72317307	72417307	0.364966	NCKAP5
2	72318004	72418004	0.345724	TRNAG-GCC-2
2	72318004	72418004	0.345724	LOC108637976
2	72318004	72418004	0.345724	NCKAP5
2	72318006	72418006	0.345724	TRNAG-GCC-2
2	72318006	72418006	0.345724	LOC108637976
2	72318006	72418006	0.345724	NCKAP5
2	72328701	72428701	0.342176	TRNAG-GCC-2
2	72328701	72428701	0.342176	NCKAP5
2	72329278	72429278	0.362255	TRNAG-GCC-2
2	72329278	72429278	0.362255	NCKAP5
2	72329279	72429279	0.362255	TRNAG-GCC-2
2	72329279	72429279	0.362255	NCKAP5
2	72329325	72429325	0.35719	TRNAG-GCC-2
2	72329325	72429325	0.35719	NCKAP5
2	72329331	72429331	0.348718	TRNAG-GCC-2
2	72329331	72429331	0.348718	NCKAP5
2	72329333	72429333	0.353949	TRNAG-GCC-2
2	72329333	72429333	0.353949	NCKAP5
2	72329545	72429545	0.344854	TRNAG-GCC-2
2	72329545	72429545	0.344854	NCKAP5
2	72331264	72431264	0.341822	TRNAG-GCC-2
2	72331264	72431264	0.341822	NCKAP5
2	72332274	72432274	0.348643	TRNAG-GCC-2
2	72332274	72432274	0.348643	NCKAP5
2	72332767	72432767	0.352506	TRNAG-GCC-2
2	72332767	72432767	0.352506	NCKAP5
2	72333263	72433263	0.342303	TRNAG-GCC-2
2	72333263	72433263	0.342303	NCKAP5
2	72333346	72433346	0.349413	TRNAG-GCC-2
2	72333346	72433346	0.349413	NCKAP5
2	72338196	72438196	0.401453	TRNAG-GCC-2
2	72338196	72438196	0.401453	NCKAP5
2	72338595	72438595	0.376935	TRNAG-GCC-2
2	72338595	72438595	0.376935	NCKAP5
2	72338744	72438744	0.365539	TRNAG-GCC-2
2	72338744	72438744	0.365539	NCKAP5
2	72338745	72438745	0.368997	TRNAG-GCC-2
2	72338745	72438745	0.368997	NCKAP5
2	72339143	72439143	0.363289	NCKAP5
2	72339358	72439358	0.356037	NCKAP5
2	72341241	72441241	0.356021	NCKAP5
2	81606865	81706865	0.357469	LRP1B
2	81623127	81723127	0.345243	LRP1B
2	81623298	81723298	0.342356	LRP1B
2	81623372	81723372	0.344277	LRP1B
2	81623604	81723604	0.347762	LRP1B
2	81623616	81723616	0.345511	LRP1B
2	81623672	81723672	0.344802	LRP1B
2	81624826	81724826	0.355548	LRP1B
2	81624832	81724832	0.357834	LRP1B
2	81624875	81724875	0.35536	LRP1B
2	81624921	81724921	0.35294	LRP1B
2	81627748	81727748	0.341754	LRP1B

2	81633767	81733767	0.356567	LRP1B
2	81633823	81733823	0.349523	LRP1B
2	81634148	81734148	0.348126	LRP1B
2	81634357	81734357	0.352658	LRP1B
2	81634433	81734433	0.349246	LRP1B
2	97648234	97748234	0.343376	ACVR1
2	97648250	97748250	0.349737	ACVR1
2	97649564	97749564	0.360007	ACVR1
2	97649570	97749570	0.360007	ACVR1
2	107941004	108041004	0.349899	B3GALT1
2	107941004	108041004	0.349899	LOC108633447
2	107941093	108041093	0.348672	B3GALT1
2	107941093	108041093	0.348672	LOC108633447
2	107941128	108041128	0.348672	B3GALT1
2	107941128	108041128	0.348672	LOC108633447
2	107941206	108041206	0.377376	B3GALT1
2	107941206	108041206	0.377376	LOC108633447
2	107946152	108046152	0.366405	B3GALT1
2	107946152	108046152	0.366405	LOC108633447
2	107946591	108046591	0.354281	B3GALT1
2	107946591	108046591	0.354281	LOC108633447
2	107946681	108046681	0.347968	B3GALT1
2	107946681	108046681	0.347968	LOC108633447
2	107946909	108046909	0.359475	B3GALT1
2	107946909	108046909	0.359475	LOC108633447
2	107947015	108047015	0.344658	B3GALT1
2	107947015	108047015	0.344658	LOC108633447
2	107947192	108047192	0.342871	B3GALT1
2	107947192	108047192	0.342871	LOC108633447
2	107947213	108047213	0.348752	B3GALT1
2	107947213	108047213	0.348752	LOC108633447
2	107949247	108049247	0.348227	B3GALT1
2	107949247	108049247	0.348227	LOC108633447
2	107949368	108049368	0.34328	B3GALT1
2	107949368	108049368	0.34328	LOC108633447
2	107949879	108049879	0.369737	B3GALT1
2	107949879	108049879	0.369737	LOC108633447
2	107949895	108049895	0.355697	B3GALT1
2	107949895	108049895	0.355697	LOC108633447
2	107949979	108049979	0.374737	B3GALT1
2	107949979	108049979	0.374737	LOC108633447
2	107949996	108049996	0.377898	B3GALT1
2	107949996	108049996	0.377898	LOC108633447
2	107950169	108050169	0.367456	B3GALT1
2	107950169	108050169	0.367456	LOC108633447
2	107950408	108050408	0.372291	B3GALT1
2	107950408	108050408	0.372291	LOC108633447
2	107950576	108050576	0.391234	B3GALT1
2	107950576	108050576	0.391234	LOC108633447
2	107953784	108053784	0.377264	B3GALT1
2	107953784	108053784	0.377264	LOC108633447
2	111614280	111714280	0.371505	SLC25A12
2	111614280	111714280	0.371505	HAT1
2	111614894	111714894	0.378899	SLC25A12
2	111614894	111714894	0.378899	HAT1
2	111615464	111715464	0.379824	SLC25A12
2	111615464	111715464	0.379824	HAT1
2	111615471	111715471	0.391658	SLC25A12

2	111615471	111715471	0.391658	HAT1
2	111616273	111716273	0.385949	SLC25A12
2	111616273	111716273	0.385949	HAT1
2	111616470	111716470	0.392849	SLC25A12
2	111616470	111716470	0.392849	HAT1
2	111616488	111716488	0.356646	SLC25A12
2	111616488	111716488	0.356646	HAT1
2	111616991	111716991	0.390033	SLC25A12
2	111616991	111716991	0.390033	HAT1
2	111617085	111717085	0.391651	SLC25A12
2	111617085	111717085	0.391651	HAT1
2	111617192	111717192	0.39627	SLC25A12
2	111617192	111717192	0.39627	HAT1
2	111617229	111717229	0.3937	SLC25A12
2	111617229	111717229	0.3937	HAT1
2	111617243	111717243	0.387049	SLC25A12
2	111617243	111717243	0.387049	HAT1
2	111617485	111717485	0.390293	SLC25A12
2	111617485	111717485	0.390293	HAT1
2	111617506	111717506	0.357299	SLC25A12
2	111617506	111717506	0.357299	HAT1
2	111617800	111717800	0.374528	SLC25A12
2	111617800	111717800	0.374528	HAT1
2	111618401	111718401	0.38647	SLC25A12
2	111618401	111718401	0.38647	HAT1
2	111618474	111718474	0.37594	SLC25A12
2	111618474	111718474	0.37594	HAT1
2	111618710	111718710	0.404498	SLC25A12
2	111618710	111718710	0.404498	HAT1
2	111619661	111719661	0.388259	SLC25A12
2	111619661	111719661	0.388259	HAT1
2	111620357	111720357	0.38784	SLC25A12
2	111620357	111720357	0.38784	HAT1
2	111620409	111720409	0.388867	SLC25A12
2	111620409	111720409	0.388867	HAT1
2	111620581	111720581	0.391475	SLC25A12
2	111620581	111720581	0.391475	HAT1
2	111670947	111770947	0.350454	HAT1
2	111670947	111770947	0.350454	METAP1D
2	113368274	113468274	0.34399	SP3
2	113372958	113472958	0.427791	SP3
2	113373122	113473122	0.415792	SP3
2	113373134	113473134	0.420166	SP3
2	113373171	113473171	0.379857	SP3
2	113373356	113473356	0.370682	SP3
2	113373624	113473624	0.343083	SP3
2	113374098	113474098	0.343083	SP3
2	113374104	113474104	0.361066	SP3
2	113374267	113474267	0.374932	SP3
2	113374300	113474300	0.355692	SP3
2	113374602	113474602	0.378967	SP3
2	113374806	113474806	0.379732	SP3
2	113374809	113474809	0.379732	SP3
2	113374864	113474864	0.391667	SP3
2	113374942	113474942	0.466073	SP3
2	113375061	113475061	0.456836	SP3
2	113375104	113475104	0.415163	SP3
2	113375109	113475109	0.401228	SP3

2	113375121	113475121	0.407477	SP3
2	113375122	113475122	0.393401	SP3
2	113375125	113475125	0.403943	SP3
2	113375138	113475138	0.369845	SP3
2	113375514	113475514	0.352129	SP3
2	113375630	113475630	0.346594	SP3
2	113375827	113475827	0.345487	SP3
2	113376063	113476063	0.345986	SP3
2	113376069	113476069	0.345808	SP3
2	113376626	113476626	0.347134	SP3
2	113376743	113476743	0.346204	SP3
2	113376753	113476753	0.347954	SP3
2	113380166	113480166	0.34596	SP3
2	113380986	113480986	0.343891	SP3
2	113381009	113481009	0.344926	SP3
2	113381108	113481108	0.344662	SP3
2	113381116	113481116	0.344662	SP3
2	115479690	115579690	0.361422	HOXD13
2	115479690	115579690	0.361422	HOXD11
2	115479690	115579690	0.361422	HOXD10
2	115479690	115579690	0.361422	HOXD9
2	115479690	115579690	0.361422	HOXD8
2	115479690	115579690	0.361422	LOC108638283
2	115479690	115579690	0.361422	MIR10B
2	115479690	115579690	0.361422	EVX2
2	115479690	115579690	0.361422	HOXD12
2	115479690	115579690	0.361422	HOXD3
2	115479691	115579691	0.379492	HOXD13
2	115479691	115579691	0.379492	HOXD11
2	115479691	115579691	0.379492	HOXD10
2	115479691	115579691	0.379492	HOXD9
2	115479691	115579691	0.379492	HOXD8
2	115479691	115579691	0.379492	LOC108638283
2	115479691	115579691	0.379492	MIR10B
2	115479691	115579691	0.379492	EVX2
2	115479691	115579691	0.379492	HOXD12
2	115479691	115579691	0.379492	HOXD3
2	115481108	115581108	0.368555	HOXD13
2	115481108	115581108	0.368555	HOXD11
2	115481108	115581108	0.368555	HOXD10
2	115481108	115581108	0.368555	HOXD9
2	115481108	115581108	0.368555	HOXD8
2	115481108	115581108	0.368555	LOC108638283
2	115481108	115581108	0.368555	MIR10B
2	115481108	115581108	0.368555	EVX2
2	115481108	115581108	0.368555	HOXD12
2	115481108	115581108	0.368555	HOXD3
2	115483418	115583418	0.396303	HOXD13
2	115483418	115583418	0.396303	HOXD11
2	115483418	115583418	0.396303	HOXD10
2	115483418	115583418	0.396303	HOXD9
2	115483418	115583418	0.396303	HOXD8
2	115483418	115583418	0.396303	LOC108638283
2	115483418	115583418	0.396303	MIR10B
2	115483418	115583418	0.396303	EVX2
2	115483418	115583418	0.396303	HOXD12
2	115483418	115583418	0.396303	HOXD3
2	115483592	115583592	0.397022	HOXD13

2	115483592	115583592	0.397022	HOXD11
2	115483592	115583592	0.397022	HOXD10
2	115483592	115583592	0.397022	HOXD9
2	115483592	115583592	0.397022	HOXD8
2	115483592	115583592	0.397022	LOC108638283
2	115483592	115583592	0.397022	MIR10B
2	115483592	115583592	0.397022	EVX2
2	115483592	115583592	0.397022	HOXD12
2	115483592	115583592	0.397022	HOXD3
2	115483605	115583605	0.400732	HOXD13
2	115483605	115583605	0.400732	HOXD11
2	115483605	115583605	0.400732	HOXD10
2	115483605	115583605	0.400732	HOXD9
2	115483605	115583605	0.400732	HOXD8
2	115483605	115583605	0.400732	LOC108638283
2	115483605	115583605	0.400732	MIR10B
2	115483605	115583605	0.400732	EVX2
2	115483605	115583605	0.400732	HOXD12
2	115483605	115583605	0.400732	HOXD3
2	115483696	115583696	0.398288	HOXD13
2	115483696	115583696	0.398288	HOXD11
2	115483696	115583696	0.398288	HOXD10
2	115483696	115583696	0.398288	HOXD9
2	115483696	115583696	0.398288	HOXD8
2	115483696	115583696	0.398288	LOC108638283
2	115483696	115583696	0.398288	MIR10B
2	115483696	115583696	0.398288	EVX2
2	115483696	115583696	0.398288	HOXD12
2	115483696	115583696	0.398288	HOXD3
2	115483883	115583883	0.398288	HOXD13
2	115483883	115583883	0.398288	HOXD11
2	115483883	115583883	0.398288	HOXD10
2	115483883	115583883	0.398288	HOXD9
2	115483883	115583883	0.398288	HOXD8
2	115483883	115583883	0.398288	LOC108638283
2	115483883	115583883	0.398288	MIR10B
2	115483883	115583883	0.398288	EVX2
2	115483883	115583883	0.398288	HOXD12
2	115483883	115583883	0.398288	HOXD3
2	115484066	115584066	0.396884	HOXD13
2	115484066	115584066	0.396884	HOXD11
2	115484066	115584066	0.396884	HOXD10
2	115484066	115584066	0.396884	HOXD9
2	115484066	115584066	0.396884	HOXD8
2	115484066	115584066	0.396884	LOC108638283
2	115484066	115584066	0.396884	MIR10B
2	115484066	115584066	0.396884	EVX2
2	115484066	115584066	0.396884	HOXD12
2	115484066	115584066	0.396884	HOXD3
2	115484072	115584072	0.404873	HOXD13
2	115484072	115584072	0.404873	HOXD11
2	115484072	115584072	0.404873	HOXD10
2	115484072	115584072	0.404873	HOXD9
2	115484072	115584072	0.404873	HOXD8
2	115484072	115584072	0.404873	LOC108638283
2	115484072	115584072	0.404873	MIR10B
2	115484072	115584072	0.404873	EVX2
2	115484072	115584072	0.404873	HOXD12

2	115484072	115584072	0.404873	HOXD3
2	115484354	115584354	0.401927	HOXD13
2	115484354	115584354	0.401927	HOXD11
2	115484354	115584354	0.401927	HOXD10
2	115484354	115584354	0.401927	HOXD9
2	115484354	115584354	0.401927	HOXD8
2	115484354	115584354	0.401927	LOC108638283
2	115484354	115584354	0.401927	MIR10B
2	115484354	115584354	0.401927	EVX2
2	115484354	115584354	0.401927	HOXD12
2	115484354	115584354	0.401927	HOXD3
2	115484872	115584872	0.405768	HOXD13
2	115484872	115584872	0.405768	HOXD11
2	115484872	115584872	0.405768	HOXD10
2	115484872	115584872	0.405768	HOXD9
2	115484872	115584872	0.405768	HOXD8
2	115484872	115584872	0.405768	LOC108638283
2	115484872	115584872	0.405768	MIR10B
2	115484872	115584872	0.405768	EVX2
2	115484872	115584872	0.405768	HOXD12
2	115484872	115584872	0.405768	HOXD3
2	115485163	115585163	0.401611	HOXD13
2	115485163	115585163	0.401611	HOXD11
2	115485163	115585163	0.401611	HOXD10
2	115485163	115585163	0.401611	HOXD9
2	115485163	115585163	0.401611	HOXD8
2	115485163	115585163	0.401611	LOC108638283
2	115485163	115585163	0.401611	MIR10B
2	115485163	115585163	0.401611	EVX2
2	115485163	115585163	0.401611	HOXD12
2	115485163	115585163	0.401611	HOXD3
2	115485495	115585495	0.403754	HOXD13
2	115485495	115585495	0.403754	HOXD11
2	115485495	115585495	0.403754	HOXD10
2	115485495	115585495	0.403754	HOXD9
2	115485495	115585495	0.403754	HOXD8
2	115485495	115585495	0.403754	LOC108638283
2	115485495	115585495	0.403754	MIR10B
2	115485495	115585495	0.403754	EVX2
2	115485495	115585495	0.403754	HOXD12
2	115485495	115585495	0.403754	HOXD3
2	115485876	115585876	0.375197	HOXD13
2	115485876	115585876	0.375197	HOXD11
2	115485876	115585876	0.375197	HOXD10
2	115485876	115585876	0.375197	HOXD9
2	115485876	115585876	0.375197	HOXD8
2	115485876	115585876	0.375197	LOC108638283
2	115485876	115585876	0.375197	MIR10B
2	115485876	115585876	0.375197	EVX2
2	115485876	115585876	0.375197	HOXD12
2	115485876	115585876	0.375197	HOXD3
2	115485932	115585932	0.375197	HOXD13
2	115485932	115585932	0.375197	HOXD11
2	115485932	115585932	0.375197	HOXD10
2	115485932	115585932	0.375197	HOXD9
2	115485932	115585932	0.375197	HOXD8
2	115485932	115585932	0.375197	LOC108638283
2	115485932	115585932	0.375197	MIR10B

2	115485932	115585932	0.375197	EVX2
2	115485932	115585932	0.375197	HOXD12
2	115485932	115585932	0.375197	HOXD3
2	115486046	115586046	0.371237	HOXD13
2	115486046	115586046	0.371237	HOXD11
2	115486046	115586046	0.371237	HOXD10
2	115486046	115586046	0.371237	HOXD9
2	115486046	115586046	0.371237	HOXD8
2	115486046	115586046	0.371237	LOC108638283
2	115486046	115586046	0.371237	MIR10B
2	115486046	115586046	0.371237	EVX2
2	115486046	115586046	0.371237	HOXD12
2	115486046	115586046	0.371237	HOXD3
2	115486140	115586140	0.365482	HOXD13
2	115486140	115586140	0.365482	HOXD11
2	115486140	115586140	0.365482	HOXD10
2	115486140	115586140	0.365482	HOXD9
2	115486140	115586140	0.365482	HOXD8
2	115486140	115586140	0.365482	LOC108638283
2	115486140	115586140	0.365482	MIR10B
2	115486140	115586140	0.365482	EVX2
2	115486140	115586140	0.365482	HOXD12
2	115486140	115586140	0.365482	HOXD3
2	115486643	115586643	0.374195	HOXD13
2	115486643	115586643	0.374195	HOXD11
2	115486643	115586643	0.374195	HOXD10
2	115486643	115586643	0.374195	HOXD9
2	115486643	115586643	0.374195	HOXD8
2	115486643	115586643	0.374195	LOC108638283
2	115486643	115586643	0.374195	MIR10B
2	115486643	115586643	0.374195	EVX2
2	115486643	115586643	0.374195	HOXD12
2	115486643	115586643	0.374195	HOXD3
2	115488147	115588147	0.361412	HOXD13
2	115488147	115588147	0.361412	HOXD11
2	115488147	115588147	0.361412	HOXD10
2	115488147	115588147	0.361412	HOXD9
2	115488147	115588147	0.361412	HOXD8
2	115488147	115588147	0.361412	LOC108638283
2	115488147	115588147	0.361412	MIR10B
2	115488147	115588147	0.361412	EVX2
2	115488147	115588147	0.361412	HOXD12
2	115488147	115588147	0.361412	HOXD3
2	115488625	115588625	0.361412	HOXD13
2	115488625	115588625	0.361412	HOXD11
2	115488625	115588625	0.361412	HOXD10
2	115488625	115588625	0.361412	HOXD9
2	115488625	115588625	0.361412	HOXD8
2	115488625	115588625	0.361412	LOC108638283
2	115488625	115588625	0.361412	MIR10B
2	115488625	115588625	0.361412	EVX2
2	115488625	115588625	0.361412	HOXD12
2	115488625	115588625	0.361412	HOXD3
2	115490544	115590544	0.370484	HOXD13
2	115490544	115590544	0.370484	HOXD11
2	115490544	115590544	0.370484	HOXD10
2	115490544	115590544	0.370484	HOXD9
2	115490544	115590544	0.370484	HOXD8

2	115490544	115590544	0.370484	LOC108638283
2	115490544	115590544	0.370484	MIR10B
2	115490544	115590544	0.370484	EVX2
2	115490544	115590544	0.370484	HOXD12
2	115490544	115590544	0.370484	HOXD3
2	115490716	115590716	0.362171	HOXD13
2	115490716	115590716	0.362171	HOXD11
2	115490716	115590716	0.362171	HOXD10
2	115490716	115590716	0.362171	HOXD9
2	115490716	115590716	0.362171	HOXD8
2	115490716	115590716	0.362171	LOC108638283
2	115490716	115590716	0.362171	MIR10B
2	115490716	115590716	0.362171	EVX2
2	115490716	115590716	0.362171	HOXD12
2	115490716	115590716	0.362171	HOXD3
2	115491889	115591889	0.362171	HOXD13
2	115491889	115591889	0.362171	HOXD11
2	115491889	115591889	0.362171	HOXD10
2	115491889	115591889	0.362171	HOXD9
2	115491889	115591889	0.362171	HOXD8
2	115491889	115591889	0.362171	LOC108638283
2	115491889	115591889	0.362171	MIR10B
2	115491889	115591889	0.362171	HOXD12
2	115491889	115591889	0.362171	HOXD3
2	115491927	115591927	0.374964	HOXD13
2	115491927	115591927	0.374964	HOXD11
2	115491927	115591927	0.374964	HOXD10
2	115491927	115591927	0.374964	HOXD9
2	115491927	115591927	0.374964	HOXD8
2	115491927	115591927	0.374964	LOC108638283
2	115491927	115591927	0.374964	MIR10B
2	115491927	115591927	0.374964	HOXD12
2	115491927	115591927	0.374964	HOXD3
2	115491950	115591950	0.374964	HOXD13
2	115491950	115591950	0.374964	HOXD11
2	115491950	115591950	0.374964	HOXD10
2	115491950	115591950	0.374964	HOXD9
2	115491950	115591950	0.374964	HOXD8
2	115491950	115591950	0.374964	LOC108638283
2	115491950	115591950	0.374964	MIR10B
2	115491950	115591950	0.374964	HOXD12
2	115491950	115591950	0.374964	HOXD3
2	115492445	115592445	0.373274	HOXD13
2	115492445	115592445	0.373274	HOXD11
2	115492445	115592445	0.373274	HOXD10
2	115492445	115592445	0.373274	HOXD9
2	115492445	115592445	0.373274	HOXD8
2	115492445	115592445	0.373274	LOC108638283
2	115492445	115592445	0.373274	MIR10B
2	115492445	115592445	0.373274	HOXD12
2	115492445	115592445	0.373274	HOXD3
2	115492483	115592483	0.373274	HOXD13
2	115492483	115592483	0.373274	HOXD11
2	115492483	115592483	0.373274	HOXD10
2	115492483	115592483	0.373274	HOXD9
2	115492483	115592483	0.373274	HOXD8
2	115492483	115592483	0.373274	LOC108638283
2	115492483	115592483	0.373274	MIR10B

2	115492483	115592483	0.373274	HOXD12
2	115492483	115592483	0.373274	HOXD3
2	115492545	115592545	0.373274	HOXD13
2	115492545	115592545	0.373274	HOXD11
2	115492545	115592545	0.373274	HOXD10
2	115492545	115592545	0.373274	HOXD9
2	115492545	115592545	0.373274	HOXD8
2	115492545	115592545	0.373274	LOC108638283
2	115492545	115592545	0.373274	MIR10B
2	115492545	115592545	0.373274	HOXD12
2	115492545	115592545	0.373274	HOXD3
2	115492877	115592877	0.374964	HOXD13
2	115492877	115592877	0.374964	HOXD11
2	115492877	115592877	0.374964	HOXD10
2	115492877	115592877	0.374964	HOXD9
2	115492877	115592877	0.374964	HOXD8
2	115492877	115592877	0.374964	LOC108638283
2	115492877	115592877	0.374964	MIR10B
2	115492877	115592877	0.374964	HOXD12
2	115492877	115592877	0.374964	HOXD3
2	115493206	115593206	0.374964	HOXD13
2	115493206	115593206	0.374964	HOXD11
2	115493206	115593206	0.374964	HOXD10
2	115493206	115593206	0.374964	HOXD9
2	115493206	115593206	0.374964	HOXD8
2	115493206	115593206	0.374964	LOC108638283
2	115493206	115593206	0.374964	MIR10B
2	115493206	115593206	0.374964	HOXD12
2	115493206	115593206	0.374964	HOXD3
2	115493312	115593312	0.370587	HOXD13
2	115493312	115593312	0.370587	HOXD11
2	115493312	115593312	0.370587	HOXD10
2	115493312	115593312	0.370587	HOXD9
2	115493312	115593312	0.370587	HOXD8
2	115493312	115593312	0.370587	LOC108638283
2	115493312	115593312	0.370587	MIR10B
2	115493312	115593312	0.370587	HOXD12
2	115493312	115593312	0.370587	HOXD3
2	115494366	115594366	0.373556	HOXD13
2	115494366	115594366	0.373556	HOXD11
2	115494366	115594366	0.373556	HOXD10
2	115494366	115594366	0.373556	HOXD9
2	115494366	115594366	0.373556	HOXD8
2	115494366	115594366	0.373556	LOC108638283
2	115494366	115594366	0.373556	MIR10B
2	115494366	115594366	0.373556	HOXD1
2	115494366	115594366	0.373556	HOXD12
2	115494366	115594366	0.373556	HOXD3
2	115494780	115594780	0.375243	HOXD13
2	115494780	115594780	0.375243	HOXD11
2	115494780	115594780	0.375243	HOXD10
2	115494780	115594780	0.375243	HOXD9
2	115494780	115594780	0.375243	HOXD8
2	115494780	115594780	0.375243	LOC108638283
2	115494780	115594780	0.375243	MIR10B
2	115494780	115594780	0.375243	HOXD1
2	115494780	115594780	0.375243	HOXD12
2	115494780	115594780	0.375243	HOXD3

2	115495027	115595027	0.375632	HOXD13
2	115495027	115595027	0.375632	HOXD11
2	115495027	115595027	0.375632	HOXD10
2	115495027	115595027	0.375632	HOXD9
2	115495027	115595027	0.375632	HOXD8
2	115495027	115595027	0.375632	LOC108638283
2	115495027	115595027	0.375632	MIR10B
2	115495027	115595027	0.375632	HOXD1
2	115495027	115595027	0.375632	HOXD12
2	115495027	115595027	0.375632	HOXD3
2	115495161	115595161	0.376785	HOXD13
2	115495161	115595161	0.376785	HOXD11
2	115495161	115595161	0.376785	HOXD10
2	115495161	115595161	0.376785	HOXD9
2	115495161	115595161	0.376785	HOXD8
2	115495161	115595161	0.376785	LOC108638283
2	115495161	115595161	0.376785	MIR10B
2	115495161	115595161	0.376785	HOXD1
2	115495161	115595161	0.376785	HOXD12
2	115495161	115595161	0.376785	HOXD3
2	115495907	115595907	0.375496	HOXD13
2	115495907	115595907	0.375496	HOXD11
2	115495907	115595907	0.375496	HOXD10
2	115495907	115595907	0.375496	HOXD9
2	115495907	115595907	0.375496	HOXD8
2	115495907	115595907	0.375496	LOC108638283
2	115495907	115595907	0.375496	MIR10B
2	115495907	115595907	0.375496	HOXD1
2	115495907	115595907	0.375496	HOXD12
2	115495907	115595907	0.375496	HOXD3
2	115496012	115596012	0.399239	HOXD13
2	115496012	115596012	0.399239	HOXD11
2	115496012	115596012	0.399239	HOXD10
2	115496012	115596012	0.399239	HOXD9
2	115496012	115596012	0.399239	HOXD8
2	115496012	115596012	0.399239	LOC108638283
2	115496012	115596012	0.399239	MIR10B
2	115496012	115596012	0.399239	HOXD1
2	115496012	115596012	0.399239	HOXD12
2	115496012	115596012	0.399239	HOXD3
2	115496592	115596592	0.357485	HOXD13
2	115496592	115596592	0.357485	HOXD11
2	115496592	115596592	0.357485	HOXD10
2	115496592	115596592	0.357485	HOXD9
2	115496592	115596592	0.357485	HOXD8
2	115496592	115596592	0.357485	LOC108638283
2	115496592	115596592	0.357485	MIR10B
2	115496592	115596592	0.357485	HOXD1
2	115496592	115596592	0.357485	HOXD12
2	115496592	115596592	0.357485	HOXD3
2	115496626	115596626	0.363323	HOXD13
2	115496626	115596626	0.363323	HOXD11
2	115496626	115596626	0.363323	HOXD10
2	115496626	115596626	0.363323	HOXD9
2	115496626	115596626	0.363323	HOXD8
2	115496626	115596626	0.363323	LOC108638283
2	115496626	115596626	0.363323	MIR10B
2	115496626	115596626	0.363323	HOXD1

2	115496626	115596626	0.363323	HOXD12
2	115496626	115596626	0.363323	HOXD3
2	115502931	115602931	0.376508	HOXD11
2	115502931	115602931	0.376508	HOXD10
2	115502931	115602931	0.376508	HOXD9
2	115502931	115602931	0.376508	HOXD8
2	115502931	115602931	0.376508	LOC108638283
2	115502931	115602931	0.376508	MIR10B
2	115502931	115602931	0.376508	HOXD1
2	115502931	115602931	0.376508	HOXD12
2	115502931	115602931	0.376508	HOXD3
2	115502977	115602977	0.358003	HOXD11
2	115502977	115602977	0.358003	HOXD10
2	115502977	115602977	0.358003	HOXD9
2	115502977	115602977	0.358003	HOXD8
2	115502977	115602977	0.358003	LOC108638283
2	115502977	115602977	0.358003	MIR10B
2	115502977	115602977	0.358003	HOXD1
2	115502977	115602977	0.358003	HOXD12
2	115502977	115602977	0.358003	HOXD3
2	115503127	115603127	0.356839	HOXD11
2	115503127	115603127	0.356839	HOXD10
2	115503127	115603127	0.356839	HOXD9
2	115503127	115603127	0.356839	HOXD8
2	115503127	115603127	0.356839	LOC108638283
2	115503127	115603127	0.356839	MIR10B
2	115503127	115603127	0.356839	HOXD1
2	115503127	115603127	0.356839	HOXD12
2	115503127	115603127	0.356839	HOXD3
2	115503973	115603973	0.356839	HOXD11
2	115503973	115603973	0.356839	HOXD10
2	115503973	115603973	0.356839	HOXD9
2	115503973	115603973	0.356839	HOXD8
2	115503973	115603973	0.356839	LOC108638283
2	115503973	115603973	0.356839	MIR10B
2	115503973	115603973	0.356839	HOXD1
2	115503973	115603973	0.356839	HOXD12
2	115503973	115603973	0.356839	HOXD3
2	115504686	115604686	0.357544	HOXD11
2	115504686	115604686	0.357544	HOXD10
2	115504686	115604686	0.357544	HOXD9
2	115504686	115604686	0.357544	HOXD8
2	115504686	115604686	0.357544	LOC108638283
2	115504686	115604686	0.357544	MIR10B
2	115504686	115604686	0.357544	HOXD1
2	115504686	115604686	0.357544	HOXD12
2	115504686	115604686	0.357544	HOXD3
2	115505049	115605049	0.41745	HOXD11
2	115505049	115605049	0.41745	HOXD10
2	115505049	115605049	0.41745	HOXD9
2	115505049	115605049	0.41745	HOXD8
2	115505049	115605049	0.41745	LOC108638283
2	115505049	115605049	0.41745	MIR10B
2	115505049	115605049	0.41745	HOXD1
2	115505049	115605049	0.41745	HOXD12
2	115505049	115605049	0.41745	HOXD3
2	115513634	115613634	0.348886	HOXD11
2	115513634	115613634	0.348886	HOXD10

2	115513634	115613634	0.348886	HOXD9
2	115513634	115613634	0.348886	HOXD8
2	115513634	115613634	0.348886	LOC108638283
2	115513634	115613634	0.348886	MIR10B
2	115513634	115613634	0.348886	HOXD1
2	115513634	115613634	0.348886	HOXD3
2	115513646	115613646	0.346805	HOXD11
2	115513646	115613646	0.346805	HOXD10
2	115513646	115613646	0.346805	HOXD9
2	115513646	115613646	0.346805	HOXD8
2	115513646	115613646	0.346805	LOC108638283
2	115513646	115613646	0.346805	MIR10B
2	115513646	115613646	0.346805	HOXD1
2	115513646	115613646	0.346805	HOXD3
2	115516519	115616519	0.346369	HOXD11
2	115516519	115616519	0.346369	HOXD10
2	115516519	115616519	0.346369	HOXD9
2	115516519	115616519	0.346369	HOXD8
2	115516519	115616519	0.346369	LOC108638283
2	115516519	115616519	0.346369	MIR10B
2	115516519	115616519	0.346369	HOXD1
2	115516519	115616519	0.346369	HOXD3
2	115518605	115618605	0.353504	HOXD10
2	115518605	115618605	0.353504	HOXD9
2	115518605	115618605	0.353504	HOXD8
2	115518605	115618605	0.353504	LOC108638283
2	115518605	115618605	0.353504	MIR10B
2	115518605	115618605	0.353504	HOXD1
2	115518605	115618605	0.353504	HOXD3
2	115519941	115619941	0.409625	HOXD10
2	115519941	115619941	0.409625	HOXD9
2	115519941	115619941	0.409625	HOXD8
2	115519941	115619941	0.409625	LOC108638283
2	115519941	115619941	0.409625	MIR10B
2	115519941	115619941	0.409625	HOXD1
2	115519941	115619941	0.409625	HOXD3
2	115520144	115620144	0.412846	HOXD10
2	115520144	115620144	0.412846	HOXD9
2	115520144	115620144	0.412846	HOXD8
2	115520144	115620144	0.412846	LOC108638283
2	115520144	115620144	0.412846	MIR10B
2	115520144	115620144	0.412846	HOXD1
2	115520144	115620144	0.412846	HOXD3
2	115520200	115620200	0.448647	HOXD10
2	115520200	115620200	0.448647	HOXD9
2	115520200	115620200	0.448647	HOXD8
2	115520200	115620200	0.448647	LOC108638283
2	115520200	115620200	0.448647	MIR10B
2	115520200	115620200	0.448647	HOXD1
2	115520200	115620200	0.448647	HOXD3
2	115520519	115620519	0.433328	HOXD10
2	115520519	115620519	0.433328	HOXD9
2	115520519	115620519	0.433328	HOXD8
2	115520519	115620519	0.433328	LOC108638283
2	115520519	115620519	0.433328	MIR10B
2	115520519	115620519	0.433328	HOXD1
2	115520519	115620519	0.433328	HOXD3
2	115520722	115620722	0.43142	HOXD10

2	115520722	115620722	0.43142	HOXD9
2	115520722	115620722	0.43142	HOXD8
2	115520722	115620722	0.43142	LOC108638283
2	115520722	115620722	0.43142	MIR10B
2	115520722	115620722	0.43142	HOXD1
2	115520722	115620722	0.43142	HOXD3
2	115521140	115621140	0.383346	HOXD10
2	115521140	115621140	0.383346	HOXD9
2	115521140	115621140	0.383346	HOXD8
2	115521140	115621140	0.383346	LOC108638283
2	115521140	115621140	0.383346	MIR10B
2	115521140	115621140	0.383346	HOXD1
2	115521140	115621140	0.383346	HOXD3
2	115521343	115621343	0.38518	HOXD10
2	115521343	115621343	0.38518	HOXD9
2	115521343	115621343	0.38518	HOXD8
2	115521343	115621343	0.38518	LOC108638283
2	115521343	115621343	0.38518	MIR10B
2	115521343	115621343	0.38518	HOXD1
2	115521343	115621343	0.38518	HOXD3
2	115524311	115624311	0.399886	HOXD10
2	115524311	115624311	0.399886	HOXD9
2	115524311	115624311	0.399886	HOXD8
2	115524311	115624311	0.399886	LOC108638283
2	115524311	115624311	0.399886	MIR10B
2	115524311	115624311	0.399886	HOXD1
2	115524311	115624311	0.399886	HOXD3
2	115524476	115624476	0.399886	HOXD10
2	115524476	115624476	0.399886	HOXD9
2	115524476	115624476	0.399886	HOXD8
2	115524476	115624476	0.399886	LOC108638283
2	115524476	115624476	0.399886	MIR10B
2	115524476	115624476	0.399886	HOXD1
2	115524476	115624476	0.399886	HOXD3
2	115524477	115624477	0.398681	HOXD10
2	115524477	115624477	0.398681	HOXD9
2	115524477	115624477	0.398681	HOXD8
2	115524477	115624477	0.398681	LOC108638283
2	115524477	115624477	0.398681	MIR10B
2	115524477	115624477	0.398681	HOXD1
2	115524477	115624477	0.398681	HOXD3
2	115524509	115624509	0.404761	HOXD10
2	115524509	115624509	0.404761	HOXD9
2	115524509	115624509	0.404761	HOXD8
2	115524509	115624509	0.404761	LOC108638283
2	115524509	115624509	0.404761	MIR10B
2	115524509	115624509	0.404761	HOXD1
2	115524509	115624509	0.404761	HOXD3
2	115524970	115624970	0.404761	HOXD10
2	115524970	115624970	0.404761	HOXD9
2	115524970	115624970	0.404761	HOXD8
2	115524970	115624970	0.404761	LOC108638283
2	115524970	115624970	0.404761	MIR10B
2	115524970	115624970	0.404761	HOXD1
2	115524970	115624970	0.404761	HOXD3
2	115525096	115625096	0.404761	HOXD10
2	115525096	115625096	0.404761	HOXD9
2	115525096	115625096	0.404761	HOXD8

2	115525096	115625096	0.404761	LOC108638283
2	115525096	115625096	0.404761	MIR10B
2	115525096	115625096	0.404761	HOXD1
2	115525096	115625096	0.404761	HOXD3
2	115549929	115649929	0.341671	MIR10B
2	115549929	115649929	0.341671	HOXD1
2	115549929	115649929	0.341671	HOXD3
2	115550125	115650125	0.35729	MIR10B
2	115550125	115650125	0.35729	HOXD1
2	115550125	115650125	0.35729	HOXD3
2	115561657	115661657	0.346294	HOXD1
2	115561657	115661657	0.346294	HOXD3
2	115565935	115665935	0.364859	HOXD1
2	115565935	115665935	0.364859	HOXD3
2	115566072	115666072	0.347737	HOXD1
2	115566072	115666072	0.347737	HOXD3
2	115566955	115666955	0.342488	HOXD1
2	115566955	115666955	0.342488	HOXD3
2	115567019	115667019	0.353508	HOXD1
2	115567019	115667019	0.353508	HOXD3
2	115567065	115667065	0.3656	HOXD1
2	115567065	115667065	0.3656	HOXD3
2	116544401	116644401	0.367414	HNRNPA3
2	116545397	116645397	0.341901	HNRNPA3
2	116545872	116645872	0.365167	HNRNPA3
2	116545908	116645908	0.366071	HNRNPA3
2	116546374	116646374	0.365802	HNRNPA3
2	116546377	116646377	0.365802	HNRNPA3
2	116546456	116646456	0.365802	HNRNPA3
2	116546768	116646768	0.403648	HNRNPA3
2	116546793	116646793	0.403648	HNRNPA3
2	116547093	116647093	0.415531	HNRNPA3
2	116547142	116647142	0.393604	HNRNPA3
2	116548658	116648658	0.395213	HNRNPA3
2	116548677	116648677	0.419215	HNRNPA3
2	116548911	116648911	0.429395	HNRNPA3
2	116548969	116648969	0.439216	HNRNPA3
2	116548988	116648988	0.439216	HNRNPA3
2	116549000	116649000	0.442256	HNRNPA3
2	116549342	116649342	0.447821	HNRNPA3
2	116549348	116649348	0.443098	HNRNPA3
2	116549467	116649467	0.428583	HNRNPA3
2	116549502	116649502	0.430387	HNRNPA3
2	116549553	116649553	0.457072	HNRNPA3
2	116549652	116649652	0.423315	HNRNPA3
2	116549668	116649668	0.420116	HNRNPA3
2	116549707	116649707	0.473304	HNRNPA3
2	116549772	116649772	0.411134	HNRNPA3
2	116549864	116649864	0.408093	HNRNPA3
2	116549885	116649885	0.408332	HNRNPA3
2	116549954	116649954	0.408035	HNRNPA3
2	116549981	116649981	0.408099	HNRNPA3
2	116550078	116650078	0.408401	HNRNPA3
2	116550135	116650135	0.394569	HNRNPA3
2	116550493	116650493	0.394956	HNRNPA3
2	116551225	116651225	0.35402	HNRNPA3
2	116551966	116651966	0.359667	HNRNPA3
2	116552233	116652233	0.368211	HNRNPA3

2	116552641	116652641	0.366713	HNRNPA3
2	116552726	116652726	0.353281	HNRNPA3
2	116927359	117027359	0.384349	LOC102176173
2	116927359	117027359	0.384349	AGPS
2	116927431	117027431	0.415606	LOC102176173
2	116927431	117027431	0.415606	AGPS
2	118095037	118195037	0.354717	TTN
2	118095368	118195368	0.351029	TTN
2	118095557	118195557	0.344098	TTN
2	118095644	118195644	0.358626	TTN
2	118095749	118195749	0.367732	TTN
2	118095839	118195839	0.350313	TTN
2	118096051	118196051	0.357551	TTN
2	118294238	118394238	0.348743	CCDC141
2	118294295	118394295	0.345182	CCDC141
2	118294354	118394354	0.343712	CCDC141
2	118295156	118395156	0.364927	CCDC141
2	118295318	118395318	0.400171	CCDC141
2	118295390	118395390	0.358628	CCDC141
2	118295414	118395414	0.386332	CCDC141
2	121651717	121751717	0.345251	SSFA2
2	121651717	121751717	0.345251	LOC102168307
2	121655822	121755822	0.345212	SSFA2
2	121655822	121755822	0.345212	LOC102168307
2	121655822	121755822	0.345212	PPP1R1C
2	121656024	121756024	0.342273	SSFA2
2	121656024	121756024	0.342273	LOC102168307
2	121656024	121756024	0.342273	PPP1R1C
2	122796759	122896759	0.350966	DUSP19
2	122796759	122896759	0.350966	NCKAP1
2	122796777	122896777	0.350896	DUSP19
2	122796777	122896777	0.350896	NCKAP1
2	122796867	122896867	0.344328	DUSP19
2	122796867	122896867	0.344328	NCKAP1
2	125895611	125995611	0.342388	FSIP2
2	125896149	125996149	0.342993	FSIP2
2	126547819	126647819	0.353262	ZC3H15
2	126548023	126648023	0.351399	ZC3H15
2	126574023	126674023	0.348023	ZC3H15
2	126574056	126674056	0.351221	ZC3H15
2	126590663	126690663	0.386833	LOC106502650
2	126590663	126690663	0.386833	ZC3H15
2	126590664	126690664	0.388483	LOC106502650
2	126590664	126690664	0.388483	ZC3H15
2	126590667	126690667	0.388483	LOC106502650
2	126590667	126690667	0.388483	ZC3H15
2	126590670	126690670	0.38022	LOC106502650
2	126590670	126690670	0.38022	ZC3H15
2	126590676	126690676	0.38022	LOC106502650
2	126590676	126690676	0.38022	ZC3H15
2	126590680	126690680	0.372807	LOC106502650
2	126590680	126690680	0.372807	ZC3H15
2	126591281	126691281	0.3425	LOC106502650
2	126591281	126691281	0.3425	ZC3H15
2	126591564	126691564	0.344884	LOC106502650
2	126591564	126691564	0.344884	ZC3H15
2	126591676	126691676	0.382559	LOC106502650
2	126591676	126691676	0.382559	ZC3H15

2	126591735	126691735	0.365422	LOC106502650
2	126591735	126691735	0.365422	ZC3H15
2	126591816	126691816	0.365422	LOC106502650
2	126591816	126691816	0.365422	ZC3H15
2	126591970	126691970	0.378369	LOC106502650
2	126591970	126691970	0.378369	ZC3H15
2	126593911	126693911	0.364065	LOC106502650
2	126593911	126693911	0.364065	ZC3H15
2	126593929	126693929	0.369025	LOC106502650
2	126593929	126693929	0.369025	ZC3H15
2	126593937	126693937	0.369025	LOC106502650
2	126593937	126693937	0.369025	ZC3H15
2	126593960	126693960	0.364538	LOC106502650
2	126593960	126693960	0.364538	ZC3H15
2	126594707	126694707	0.346014	LOC106502650
2	126594707	126694707	0.346014	ZC3H15
2	126594736	126694736	0.351743	LOC106502650
2	126594736	126694736	0.351743	ZC3H15
2	126594885	126694885	0.349088	LOC106502650
2	126594885	126694885	0.349088	ZC3H15
2	126645890	126745890	0.34819	LOC106502650
2	126645890	126745890	0.34819	ITGAV
2	126645935	126745935	0.344862	LOC106502650
2	126645935	126745935	0.344862	ITGAV
2	126645996	126745996	0.401394	LOC106502650
2	126645996	126745996	0.401394	ITGAV
2	126645997	126745997	0.411173	LOC106502650
2	126645997	126745997	0.411173	ITGAV
2	126646042	126746042	0.443201	LOC106502650
2	126646042	126746042	0.443201	ITGAV
2	126646127	126746127	0.418146	LOC106502650
2	126646127	126746127	0.418146	ITGAV
2	126646373	126746373	0.411258	LOC106502650
2	126646373	126746373	0.411258	ITGAV
2	126646379	126746379	0.415667	LOC106502650
2	126646379	126746379	0.415667	ITGAV
2	126646400	126746400	0.381595	LOC106502650
2	126646400	126746400	0.381595	ITGAV
2	126646412	126746412	0.385131	LOC106502650
2	126646412	126746412	0.385131	ITGAV
2	126646472	126746472	0.367321	LOC106502650
2	126646472	126746472	0.367321	ITGAV
2	126646533	126746533	0.371119	LOC106502650
2	126646533	126746533	0.371119	ITGAV
2	126646583	126746583	0.393902	LOC106502650
2	126646583	126746583	0.393902	ITGAV
2	126646593	126746593	0.397292	LOC106502650
2	126646593	126746593	0.397292	ITGAV
2	126646634	126746634	0.393204	LOC106502650
2	126646634	126746634	0.393204	ITGAV
2	126646665	126746665	0.394151	LOC106502650
2	126646665	126746665	0.394151	ITGAV
2	126646717	126746717	0.393992	LOC106502650
2	126646717	126746717	0.393992	ITGAV
2	126646887	126746887	0.393329	LOC106502650
2	126646887	126746887	0.393329	ITGAV
2	126647023	126747023	0.384832	LOC106502650
2	126647023	126747023	0.384832	ITGAV

2	126647241	126747241	0.342399	LOC106502650
2	126647241	126747241	0.342399	ITGAV
2	126648749	126748749	0.356058	LOC106502650
2	126648749	126748749	0.356058	ITGAV
2	126649642	126749642	0.344382	LOC106502650
2	126649642	126749642	0.344382	ITGAV
2	126649738	126749738	0.351709	LOC106502650
2	126649738	126749738	0.351709	ITGAV
2	126649794	126749794	0.356272	LOC106502650
2	126649794	126749794	0.356272	ITGAV
2	126649845	126749845	0.372408	LOC106502650
2	126649845	126749845	0.372408	ITGAV
2	126649860	126749860	0.386084	LOC106502650
2	126649860	126749860	0.386084	ITGAV
2	126649896	126749896	0.393368	LOC106502650
2	126649896	126749896	0.393368	ITGAV
2	126649934	126749934	0.38349	LOC106502650
2	126649934	126749934	0.38349	ITGAV
2	126649966	126749966	0.386091	LOC106502650
2	126649966	126749966	0.386091	ITGAV
2	126649990	126749990	0.404619	LOC106502650
2	126649990	126749990	0.404619	ITGAV
2	126650008	126750008	0.396932	LOC106502650
2	126650008	126750008	0.396932	ITGAV
2	126650029	126750029	0.343593	LOC106502650
2	126650029	126750029	0.343593	ITGAV
2	126650864	126750864	0.346653	LOC106502650
2	126650864	126750864	0.346653	ITGAV
2	127474902	127574902	0.356013	TFPI
2	127474902	127574902	0.356013	CALCRL
2	130315047	130415047	0.342987	C2H2orf88
2	130315092	130415092	0.357982	C2H2orf88
2	130315127	130415127	0.356895	C2H2orf88
2	130315252	130415252	0.342684	C2H2orf88
2	130315313	130415313	0.344515	C2H2orf88
2	130316979	130416979	0.345745	C2H2orf88
2	130317020	130417020	0.35991	C2H2orf88
2	130317072	130417072	0.352637	C2H2orf88
2	130317095	130417095	0.352871	C2H2orf88
2	130317123	130417123	0.353639	C2H2orf88
2	130317160	130417160	0.421443	C2H2orf88
2	130317176	130417176	0.443783	C2H2orf88
2	130317177	130417177	0.473141	C2H2orf88
2	130317219	130417219	0.47661	C2H2orf88
2	130317220	130417220	0.47661	C2H2orf88
2	130317227	130417227	0.480665	C2H2orf88
2	130317272	130417272	0.441777	C2H2orf88
2	130317319	130417319	0.346683	C2H2orf88
2	130355571	130455571	0.40555	HIBCH
2	130355571	130455571	0.40555	C2H2orf88
2	130355573	130455573	0.458041	HIBCH
2	130355573	130455573	0.458041	C2H2orf88
2	130355576	130455576	0.427893	HIBCH
2	130355576	130455576	0.427893	C2H2orf88
2	130355583	130455583	0.361076	HIBCH
2	130355583	130455583	0.361076	C2H2orf88
2	130355584	130455584	0.429488	HIBCH
2	130355584	130455584	0.429488	C2H2orf88

3	637775	737775	0.363436	ANO7
3	637775	737775	0.363436	PPP1R7
3	637775	737775	0.363436	PASK
3	637775	737775	0.363436	HDLBP
3	637796	737796	0.352844	ANO7
3	637796	737796	0.352844	PPP1R7
3	637796	737796	0.352844	PASK
3	637796	737796	0.352844	HDLBP
3	1336741	1436741	0.342788	OTOS
3	1336741	1436741	0.342788	COPS9
3	1398142	1498142	0.388513	OTOS
3	1398142	1498142	0.388513	LOC102184970
3	1398142	1498142	0.388513	LOC102184688
3	1398142	1498142	0.388513	LOC102184399
3	1398142	1498142	0.388513	COPS9
3	1398165	1498165	0.346179	OTOS
3	1398165	1498165	0.346179	LOC102184970
3	1398165	1498165	0.346179	LOC102184688
3	1398165	1498165	0.346179	LOC102184399
3	1398165	1498165	0.346179	COPS9
3	1398169	1498169	0.358719	OTOS
3	1398169	1498169	0.358719	LOC102184970
3	1398169	1498169	0.358719	LOC102184688
3	1398169	1498169	0.358719	LOC102184399
3	1398169	1498169	0.358719	COPS9
3	1398172	1498172	0.368431	OTOS
3	1398172	1498172	0.368431	LOC102184970
3	1398172	1498172	0.368431	LOC102184688
3	1398172	1498172	0.368431	LOC102184399
3	1398172	1498172	0.368431	COPS9
3	1398176	1498176	0.381509	OTOS
3	1398176	1498176	0.381509	LOC102184970
3	1398176	1498176	0.381509	LOC102184688
3	1398176	1498176	0.381509	LOC102184399
3	1398176	1498176	0.381509	COPS9
3	1412311	1512311	0.342053	OTOS
3	1412311	1512311	0.342053	LOC102184970
3	1412311	1512311	0.342053	LOC102184688
3	1412311	1512311	0.342053	LOC102184399
3	1412311	1512311	0.342053	LOC102183846
3	1412311	1512311	0.342053	COPS9
3	1412350	1512350	0.345821	OTOS
3	1412350	1512350	0.345821	LOC102184970
3	1412350	1512350	0.345821	LOC102184688
3	1412350	1512350	0.345821	LOC102184399
3	1412350	1512350	0.345821	LOC102183846
3	1412350	1512350	0.345821	COPS9
3	1412358	1512358	0.345821	OTOS
3	1412358	1512358	0.345821	LOC102184970
3	1412358	1512358	0.345821	LOC102184688
3	1412358	1512358	0.345821	LOC102184399
3	1412358	1512358	0.345821	LOC102183846
3	1412358	1512358	0.345821	COPS9
3	1412592	1512592	0.347641	OTOS
3	1412592	1512592	0.347641	LOC102184970
3	1412592	1512592	0.347641	LOC102184688
3	1412592	1512592	0.347641	LOC102184399
3	1412592	1512592	0.347641	LOC102183846

3	1412592	1512592	0.347641	COPS9
3	1412596	1512596	0.345737	OTOS
3	1412596	1512596	0.345737	LOC102184970
3	1412596	1512596	0.345737	LOC102184688
3	1412596	1512596	0.345737	LOC102184399
3	1412596	1512596	0.345737	LOC102183846
3	1412596	1512596	0.345737	COPS9
3	1412624	1512624	0.349694	OTOS
3	1412624	1512624	0.349694	LOC102184970
3	1412624	1512624	0.349694	LOC102184688
3	1412624	1512624	0.349694	LOC102184399
3	1412624	1512624	0.349694	LOC102183846
3	1412624	1512624	0.349694	COPS9
3	1412632	1512632	0.349694	OTOS
3	1412632	1512632	0.349694	LOC102184970
3	1412632	1512632	0.349694	LOC102184688
3	1412632	1512632	0.349694	LOC102184399
3	1412632	1512632	0.349694	LOC102183846
3	1412632	1512632	0.349694	COPS9
3	1412653	1512653	0.341726	OTOS
3	1412653	1512653	0.341726	LOC102184970
3	1412653	1512653	0.341726	LOC102184688
3	1412653	1512653	0.341726	LOC102184399
3	1412653	1512653	0.341726	LOC102183846
3	1412653	1512653	0.341726	COPS9
3	1436633	1536633	0.354644	LOC102184970
3	1436633	1536633	0.354644	LOC102184688
3	1436633	1536633	0.354644	LOC102184399
3	1436633	1536633	0.354644	LOC102183846
3	1436633	1536633	0.354644	LOC102191600
3	1436687	1536687	0.355501	LOC102184970
3	1436687	1536687	0.355501	LOC102184688
3	1436687	1536687	0.355501	LOC102184399
3	1436687	1536687	0.355501	LOC102183846
3	1436687	1536687	0.355501	LOC102191600
3	1436716	1536716	0.351702	LOC102184970
3	1436716	1536716	0.351702	LOC102184688
3	1436716	1536716	0.351702	LOC102184399
3	1436716	1536716	0.351702	LOC102183846
3	1436716	1536716	0.351702	LOC102191600
3	2363143	2463143	0.342703	HDAC4
3	2363448	2463448	0.354781	HDAC4
3	2363561	2463561	0.354369	HDAC4
3	2363687	2463687	0.354618	HDAC4
3	2364180	2464180	0.342728	HDAC4
3	2364791	2464791	0.354125	HDAC4
3	2366156	2466156	0.358924	HDAC4
3	2366374	2466374	0.364498	HDAC4
3	2366431	2466431	0.401006	HDAC4
3	2366460	2466460	0.394935	HDAC4
3	2366630	2466630	0.388816	HDAC4
3	2366744	2466744	0.400168	HDAC4
3	2366766	2466766	0.435217	HDAC4
3	2366795	2466795	0.43429	HDAC4
3	2366908	2466908	0.432486	HDAC4
3	2367031	2467031	0.432096	HDAC4
3	2367056	2467056	0.423246	HDAC4
3	2367130	2467130	0.423246	HDAC4

3	2367397	2467397	0.426128	HDAC4
3	2367424	2467424	0.44128	HDAC4
3	2367669	2467669	0.432591	HDAC4
3	2367934	2467934	0.428762	HDAC4
3	2367957	2467957	0.442815	HDAC4
3	2368321	2468321	0.444894	HDAC4
3	2368414	2468414	0.443101	HDAC4
3	2368444	2468444	0.468863	HDAC4
3	2368473	2468473	0.458616	HDAC4
3	2368485	2468485	0.467611	HDAC4
3	2368567	2468567	0.461258	HDAC4
3	2368659	2468659	0.475195	HDAC4
3	2368773	2468773	0.471176	HDAC4
3	2368847	2468847	0.470101	HDAC4
3	2368890	2468890	0.472195	HDAC4
3	2368897	2468897	0.454946	HDAC4
3	2369043	2469043	0.451477	HDAC4
3	2369322	2469322	0.458921	HDAC4
3	2369374	2469374	0.444073	HDAC4
3	2369404	2469404	0.440226	HDAC4
3	2369565	2469565	0.441379	HDAC4
3	2369706	2469706	0.436049	HDAC4
3	2369832	2469832	0.439174	HDAC4
3	2370270	2470270	0.418702	HDAC4
3	2370383	2470383	0.445547	HDAC4
3	2370599	2470599	0.394207	HDAC4
3	2370600	2470600	0.394207	HDAC4
3	2370862	2470862	0.419041	HDAC4
3	2370868	2470868	0.387065	HDAC4
3	2370991	2470991	0.36695	HDAC4
3	2371077	2471077	0.349638	HDAC4
3	2378306	2478306	0.343734	HDAC4
3	2378366	2478366	0.344395	HDAC4
3	2378368	2478368	0.345274	HDAC4
3	2378467	2478467	0.357587	HDAC4
3	2378499	2478499	0.357587	HDAC4
3	2638632	2738632	0.367111	LOC102179832
3	2638632	2738632	0.367111	TWIST2
3	3610603	3710603	0.362866	TRNAG-CCC-9
3	3610603	3710603	0.362866	RAB17
3	3610603	3710603	0.362866	LRRFIP1
3	3779527	3879527	0.341637	COL6A3
3	7607886	7707886	0.395436	USP40
3	7607886	7707886	0.395436	DGKD
3	7608131	7708131	0.406478	USP40
3	7608131	7708131	0.406478	DGKD
3	7608663	7708663	0.357696	USP40
3	7608663	7708663	0.357696	DGKD
3	7608673	7708673	0.366959	USP40
3	7608673	7708673	0.366959	DGKD
3	7609130	7709130	0.382307	USP40
3	7609130	7709130	0.382307	DGKD
3	7609432	7709432	0.37292	USP40
3	7609432	7709432	0.37292	DGKD
3	7609884	7709884	0.362121	USP40
3	7609884	7709884	0.362121	DGKD
3	7609940	7709940	0.343112	USP40
3	7609940	7709940	0.343112	DGKD

3	9038085	9138085	0.35391	CSMD2
3	9123074	9223074	0.434057	C3H1orf94
3	9123074	9223074	0.434057	CSMD2
3	9123080	9223080	0.434057	C3H1orf94
3	9123080	9223080	0.434057	CSMD2
3	9123130	9223130	0.350716	C3H1orf94
3	9123130	9223130	0.350716	CSMD2
3	9123261	9223261	0.3511	C3H1orf94
3	9123261	9223261	0.3511	CSMD2
3	9123377	9223377	0.3511	C3H1orf94
3	9123377	9223377	0.3511	CSMD2
3	9123392	9223392	0.387973	C3H1orf94
3	9123392	9223392	0.387973	CSMD2
3	9123435	9223435	0.350362	C3H1orf94
3	9123435	9223435	0.350362	CSMD2
3	9123485	9223485	0.364042	C3H1orf94
3	9123485	9223485	0.364042	CSMD2
3	9123490	9223490	0.364846	C3H1orf94
3	9123490	9223490	0.364846	CSMD2
3	9123491	9223491	0.344581	C3H1orf94
3	9123491	9223491	0.344581	CSMD2
3	9123499	9223499	0.346791	C3H1orf94
3	9123499	9223499	0.346791	CSMD2
3	9123734	9223734	0.345896	C3H1orf94
3	9123734	9223734	0.345896	CSMD2
3	9123995	9223995	0.341834	C3H1orf94
3	9123995	9223995	0.341834	CSMD2
3	9124074	9224074	0.341754	C3H1orf94
3	9124074	9224074	0.341754	CSMD2
3	9124091	9224091	0.356482	C3H1orf94
3	9124091	9224091	0.356482	CSMD2
3	9124169	9224169	0.352911	C3H1orf94
3	9124169	9224169	0.352911	CSMD2
3	9124186	9224186	0.352911	C3H1orf94
3	9124186	9224186	0.352911	CSMD2
3	9124198	9224198	0.342664	C3H1orf94
3	9124198	9224198	0.342664	CSMD2
3	9124206	9224206	0.358007	C3H1orf94
3	9124206	9224206	0.358007	CSMD2
3	9124274	9224274	0.355252	C3H1orf94
3	9124274	9224274	0.355252	CSMD2
3	9124358	9224358	0.344158	C3H1orf94
3	9124358	9224358	0.344158	CSMD2
3	9124387	9224387	0.341912	C3H1orf94
3	9124387	9224387	0.341912	CSMD2
3	9124937	9224937	0.349143	C3H1orf94
3	9124937	9224937	0.349143	CSMD2
3	9125514	9225514	0.34418	C3H1orf94
3	9125514	9225514	0.34418	CSMD2
3	9126172	9226172	0.34418	C3H1orf94
3	9126172	9226172	0.34418	CSMD2
3	9127026	9227026	0.354954	C3H1orf94
3	9127026	9227026	0.354954	CSMD2
3	9127446	9227446	0.355846	C3H1orf94
3	9127446	9227446	0.355846	CSMD2
3	9127580	9227580	0.343112	C3H1orf94
3	9127580	9227580	0.343112	CSMD2
3	9127819	9227819	0.343112	C3H1orf94

3	9127819	9227819	0.343112	CSMD2
3	9127965	9227965	0.377907	C3H1orf94
3	9127965	9227965	0.377907	CSMD2
3	9128082	9228082	0.380738	C3H1orf94
3	9128082	9228082	0.380738	CSMD2
3	9128091	9228091	0.385322	C3H1orf94
3	9128091	9228091	0.385322	CSMD2
3	9128212	9228212	0.371251	C3H1orf94
3	9128212	9228212	0.371251	CSMD2
3	9128421	9228421	0.403545	C3H1orf94
3	9128421	9228421	0.403545	CSMD2
3	9128679	9228679	0.406461	C3H1orf94
3	9128679	9228679	0.406461	CSMD2
3	9128974	9228974	0.410099	C3H1orf94
3	9128974	9228974	0.410099	CSMD2
3	9129020	9229020	0.398378	C3H1orf94
3	9129020	9229020	0.398378	CSMD2
3	9129330	9229330	0.407981	C3H1orf94
3	9129330	9229330	0.407981	CSMD2
3	9129353	9229353	0.410698	C3H1orf94
3	9129353	9229353	0.410698	CSMD2
3	9129626	9229626	0.402301	C3H1orf94
3	9129626	9229626	0.402301	CSMD2
3	9129681	9229681	0.402473	C3H1orf94
3	9129681	9229681	0.402473	CSMD2
3	9129893	9229893	0.419485	C3H1orf94
3	9129893	9229893	0.419485	CSMD2
3	9129984	9229984	0.413836	C3H1orf94
3	9129984	9229984	0.413836	CSMD2
3	9130094	9230094	0.42185	C3H1orf94
3	9130094	9230094	0.42185	CSMD2
3	9130191	9230191	0.425707	C3H1orf94
3	9130191	9230191	0.425707	CSMD2
3	9130210	9230210	0.419761	C3H1orf94
3	9130210	9230210	0.419761	CSMD2
3	9130229	9230229	0.408803	C3H1orf94
3	9130229	9230229	0.408803	CSMD2
3	9130479	9230479	0.414075	C3H1orf94
3	9130479	9230479	0.414075	CSMD2
3	9130519	9230519	0.417237	C3H1orf94
3	9130519	9230519	0.417237	CSMD2
3	9130640	9230640	0.401329	C3H1orf94
3	9130640	9230640	0.401329	CSMD2
3	9130749	9230749	0.401212	C3H1orf94
3	9130749	9230749	0.401212	CSMD2
3	9130753	9230753	0.417268	C3H1orf94
3	9130753	9230753	0.417268	CSMD2
3	9130868	9230868	0.344158	C3H1orf94
3	9130868	9230868	0.344158	CSMD2
3	9136771	9236771	0.370287	C3H1orf94
3	9136801	9236801	0.349586	C3H1orf94
3	9136890	9236890	0.367689	C3H1orf94
3	9137235	9237235	0.376237	C3H1orf94
3	9137749	9237749	0.373057	C3H1orf94
3	9137968	9237968	0.399082	C3H1orf94
3	9138024	9238024	0.400269	C3H1orf94
3	9138093	9238093	0.401913	C3H1orf94
3	9138174	9238174	0.411363	C3H1orf94

3	9138351	9238351	0.406856	C3H1orf94
3	9138428	9238428	0.402449	C3H1orf94
3	9138537	9238537	0.399384	C3H1orf94
3	9138539	9238539	0.410344	C3H1orf94
3	9138652	9238652	0.423253	C3H1orf94
3	9138852	9238852	0.409266	C3H1orf94
3	9138918	9238918	0.419064	C3H1orf94
3	9138939	9238939	0.422347	C3H1orf94
3	9138954	9238954	0.417431	C3H1orf94
3	9139162	9239162	0.426057	C3H1orf94
3	9139208	9239208	0.415967	C3H1orf94
3	9139340	9239340	0.416788	C3H1orf94
3	9139367	9239367	0.416917	C3H1orf94
3	9139390	9239390	0.420451	C3H1orf94
3	9139483	9239483	0.417495	C3H1orf94
3	9139568	9239568	0.418045	C3H1orf94
3	9139795	9239795	0.409989	C3H1orf94
3	9139968	9239968	0.413207	C3H1orf94
3	9139992	9239992	0.416773	C3H1orf94
3	9140097	9240097	0.421201	C3H1orf94
3	9140378	9240378	0.414665	C3H1orf94
3	9140416	9240416	0.415874	C3H1orf94
3	9140566	9240566	0.419328	C3H1orf94
3	9140652	9240652	0.425468	C3H1orf94
3	9140722	9240722	0.41962	C3H1orf94
3	9140859	9240859	0.42114	C3H1orf94
3	9140862	9240862	0.429229	C3H1orf94
3	9140928	9240928	0.418309	C3H1orf94
3	9140995	9240995	0.423856	C3H1orf94
3	9141098	9241098	0.40299	C3H1orf94
3	9141125	9241125	0.402768	C3H1orf94
3	9141181	9241181	0.3997	C3H1orf94
3	9141661	9241661	0.404732	C3H1orf94
3	9141693	9241693	0.407302	C3H1orf94
3	9141699	9241699	0.400513	C3H1orf94
3	9141868	9241868	0.400513	C3H1orf94
3	9143043	9243043	0.410999	C3H1orf94
3	9143565	9243565	0.426711	C3H1orf94
3	9143605	9243605	0.436422	C3H1orf94
3	9143701	9243701	0.426539	C3H1orf94
3	9143950	9243950	0.415901	C3H1orf94
3	9144110	9244110	0.428492	C3H1orf94
3	9144754	9244754	0.398731	C3H1orf94
3	9144921	9244921	0.41302	C3H1orf94
3	9145043	9245043	0.434278	C3H1orf94
3	9145101	9245101	0.420897	C3H1orf94
3	9145227	9245227	0.443932	C3H1orf94
3	9145228	9245228	0.443932	C3H1orf94
3	9145332	9245332	0.448175	C3H1orf94
3	9145343	9245343	0.439747	C3H1orf94
3	9145914	9245914	0.410998	C3H1orf94
3	9146126	9246126	0.410998	C3H1orf94
3	9146257	9246257	0.442575	C3H1orf94
3	9146333	9246333	0.400224	C3H1orf94
3	9146541	9246541	0.412452	C3H1orf94
3	9147093	9247093	0.390783	C3H1orf94
3	9147274	9247274	0.398128	C3H1orf94
3	9147399	9247399	0.40962	C3H1orf94

3	9147497	9247497	0.42545	C3H1orf94
3	9147589	9247589	0.403565	C3H1orf94
3	9147631	9247631	0.409253	C3H1orf94
3	9147852	9247852	0.398637	C3H1orf94
3	9147931	9247931	0.399173	C3H1orf94
3	9148328	9248328	0.393724	C3H1orf94
3	9149065	9249065	0.342	C3H1orf94
3	9149367	9249367	0.342	C3H1orf94
3	9149670	9249670	0.364282	C3H1orf94
3	9150145	9250145	0.341977	C3H1orf94
3	9150780	9250780	0.368727	C3H1orf94
3	9150841	9250841	0.367208	C3H1orf94
3	9150853	9250853	0.358192	C3H1orf94
3	9151210	9251210	0.379467	C3H1orf94
3	9151915	9251915	0.378743	C3H1orf94
3	9152858	9252858	0.352513	C3H1orf94
3	9152987	9252987	0.347545	C3H1orf94
3	9154367	9254367	0.385364	C3H1orf94
3	9156774	9256774	0.428409	C3H1orf94
3	12413701	12513701	0.352537	DNALI1
3	12413701	12513701	0.352537	MEAF6
3	12413701	12513701	0.352537	SNIP1
3	12413701	12513701	0.352537	GNL2
3	12414930	12514930	0.351633	DNALI1
3	12414930	12514930	0.351633	MEAF6
3	12414930	12514930	0.351633	SNIP1
3	12414930	12514930	0.351633	GNL2
3	12414950	12514950	0.347081	DNALI1
3	12414950	12514950	0.347081	MEAF6
3	12414950	12514950	0.347081	SNIP1
3	12414950	12514950	0.347081	GNL2
3	12415007	12515007	0.357495	DNALI1
3	12415007	12515007	0.357495	MEAF6
3	12415007	12515007	0.357495	SNIP1
3	12415007	12515007	0.357495	GNL2
3	12415154	12515154	0.341868	DNALI1
3	12415154	12515154	0.341868	MEAF6
3	12415154	12515154	0.341868	SNIP1
3	12415154	12515154	0.341868	GNL2
3	12418367	12518367	0.353208	DNALI1
3	12418367	12518367	0.353208	MEAF6
3	12418367	12518367	0.353208	SNIP1
3	12418367	12518367	0.353208	GNL2
3	12418732	12518732	0.36954	DNALI1
3	12418732	12518732	0.36954	MEAF6
3	12418732	12518732	0.36954	SNIP1
3	12418732	12518732	0.36954	GNL2
3	12418797	12518797	0.343945	DNALI1
3	12418797	12518797	0.343945	MEAF6
3	12418797	12518797	0.343945	SNIP1
3	12418797	12518797	0.343945	GNL2
3	12418808	12518808	0.353946	DNALI1
3	12418808	12518808	0.353946	MEAF6
3	12418808	12518808	0.353946	SNIP1
3	12418808	12518808	0.353946	GNL2
3	12452544	12552544	0.350917	DNALI1
3	12452544	12552544	0.350917	SNIP1
3	12452544	12552544	0.350917	GNL2

3	12452544	12552544	0.350917	RSPO1
3	12453638	12553638	0.354895	DNALI1
3	12453638	12553638	0.354895	SNIP1
3	12453638	12553638	0.354895	GNL2
3	12453638	12553638	0.354895	RSPO1
3	12454755	12554755	0.374531	DNALI1
3	12454755	12554755	0.374531	SNIP1
3	12454755	12554755	0.374531	GNL2
3	12454755	12554755	0.374531	RSPO1
3	12454824	12554824	0.378834	DNALI1
3	12454824	12554824	0.378834	SNIP1
3	12454824	12554824	0.378834	GNL2
3	12454824	12554824	0.378834	RSPO1
3	12454876	12554876	0.378834	DNALI1
3	12454876	12554876	0.378834	SNIP1
3	12454876	12554876	0.378834	GNL2
3	12454876	12554876	0.378834	RSPO1
3	12455025	12555025	0.381038	DNALI1
3	12455025	12555025	0.381038	SNIP1
3	12455025	12555025	0.381038	GNL2
3	12455025	12555025	0.381038	RSPO1
3	12455063	12555063	0.362753	DNALI1
3	12455063	12555063	0.362753	SNIP1
3	12455063	12555063	0.362753	GNL2
3	12455063	12555063	0.362753	RSPO1
3	12455090	12555090	0.345153	DNALI1
3	12455090	12555090	0.345153	SNIP1
3	12455090	12555090	0.345153	GNL2
3	12455090	12555090	0.345153	RSPO1
3	12455624	12555624	0.349609	DNALI1
3	12455624	12555624	0.349609	SNIP1
3	12455624	12555624	0.349609	GNL2
3	12455624	12555624	0.349609	RSPO1
3	12456129	12556129	0.350177	DNALI1
3	12456129	12556129	0.350177	SNIP1
3	12456129	12556129	0.350177	GNL2
3	12456129	12556129	0.350177	RSPO1
3	12456969	12556969	0.380873	DNALI1
3	12456969	12556969	0.380873	SNIP1
3	12456969	12556969	0.380873	GNL2
3	12456969	12556969	0.380873	RSPO1
3	12457026	12557026	0.377186	DNALI1
3	12457026	12557026	0.377186	SNIP1
3	12457026	12557026	0.377186	GNL2
3	12457026	12557026	0.377186	RSPO1
3	12679842	12779842	0.360679	MANEAL
3	12679842	12779842	0.360679	YRDC
3	12679842	12779842	0.360679	INPP5B
3	12679842	12779842	0.360679	MTF1
3	16513624	16613624	0.380261	HIVEP3
3	16671611	16771611	0.362362	GUCA2B
3	16671611	16771611	0.362362	GUCA2A
3	16671611	16771611	0.362362	FOXJ3
3	16671665	16771665	0.364969	GUCA2B
3	16671665	16771665	0.364969	GUCA2A
3	16671665	16771665	0.364969	FOXJ3
3	16671793	16771793	0.371764	GUCA2B
3	16671793	16771793	0.371764	GUCA2A

3	16671793	16771793	0.371764	FOXJ3
3	16671937	16771937	0.347031	GUCA2B
3	16671937	16771937	0.347031	GUCA2A
3	16671937	16771937	0.347031	FOXJ3
3	16671960	16771960	0.358463	GUCA2B
3	16671960	16771960	0.358463	GUCA2A
3	16671960	16771960	0.358463	FOXJ3
3	16671961	16771961	0.360676	GUCA2B
3	16671961	16771961	0.360676	GUCA2A
3	16671961	16771961	0.360676	FOXJ3
3	16671990	16771990	0.370684	GUCA2B
3	16671990	16771990	0.370684	GUCA2A
3	16671990	16771990	0.370684	FOXJ3
3	16672391	16772391	0.364397	GUCA2B
3	16672391	16772391	0.364397	GUCA2A
3	16672391	16772391	0.364397	FOXJ3
3	16672497	16772497	0.387501	GUCA2B
3	16672497	16772497	0.387501	GUCA2A
3	16672497	16772497	0.387501	FOXJ3
3	16672524	16772524	0.409016	GUCA2B
3	16672524	16772524	0.409016	GUCA2A
3	16672524	16772524	0.409016	FOXJ3
3	16672981	16772981	0.409016	GUCA2B
3	16672981	16772981	0.409016	GUCA2A
3	16672981	16772981	0.409016	FOXJ3
3	16673006	16773006	0.409016	GUCA2B
3	16673006	16773006	0.409016	GUCA2A
3	16673006	16773006	0.409016	FOXJ3
3	16673016	16773016	0.408528	GUCA2B
3	16673016	16773016	0.408528	GUCA2A
3	16673016	16773016	0.408528	FOXJ3
3	16673046	16773046	0.419001	GUCA2B
3	16673046	16773046	0.419001	GUCA2A
3	16673046	16773046	0.419001	FOXJ3
3	16673150	16773150	0.417829	GUCA2B
3	16673150	16773150	0.417829	GUCA2A
3	16673150	16773150	0.417829	FOXJ3
3	16673301	16773301	0.440508	GUCA2B
3	16673301	16773301	0.440508	GUCA2A
3	16673301	16773301	0.440508	FOXJ3
3	16673312	16773312	0.443879	GUCA2B
3	16673312	16773312	0.443879	GUCA2A
3	16673312	16773312	0.443879	FOXJ3
3	16673552	16773552	0.399574	GUCA2B
3	16673552	16773552	0.399574	GUCA2A
3	16673552	16773552	0.399574	FOXJ3
3	16673665	16773665	0.403006	GUCA2B
3	16673665	16773665	0.403006	GUCA2A
3	16673665	16773665	0.403006	FOXJ3
3	16673834	16773834	0.392113	GUCA2B
3	16673834	16773834	0.392113	GUCA2A
3	16673834	16773834	0.392113	FOXJ3
3	16673936	16773936	0.396885	GUCA2B
3	16673936	16773936	0.396885	GUCA2A
3	16673936	16773936	0.396885	FOXJ3
3	16674029	16774029	0.392877	GUCA2B
3	16674029	16774029	0.392877	GUCA2A
3	16674029	16774029	0.392877	FOXJ3

3	16674050	16774050	0.39425	GUCA2B
3	16674050	16774050	0.39425	GUCA2A
3	16674050	16774050	0.39425	FOXJ3
3	16674157	16774157	0.390015	GUCA2B
3	16674157	16774157	0.390015	GUCA2A
3	16674157	16774157	0.390015	FOXJ3
3	16674373	16774373	0.385657	GUCA2B
3	16674373	16774373	0.385657	GUCA2A
3	16674373	16774373	0.385657	FOXJ3
3	16674585	16774585	0.342647	GUCA2B
3	16674585	16774585	0.342647	GUCA2A
3	16674585	16774585	0.342647	FOXJ3
3	16674616	16774616	0.342821	GUCA2B
3	16674616	16774616	0.342821	GUCA2A
3	16674616	16774616	0.342821	FOXJ3
3	16674764	16774764	0.34332	GUCA2B
3	16674764	16774764	0.34332	GUCA2A
3	16674764	16774764	0.34332	FOXJ3
3	16674851	16774851	0.342441	GUCA2B
3	16674851	16774851	0.342441	GUCA2A
3	16674851	16774851	0.342441	FOXJ3
3	16674882	16774882	0.342677	GUCA2B
3	16674882	16774882	0.342677	GUCA2A
3	16674882	16774882	0.342677	FOXJ3
3	16675026	16775026	0.342725	GUCA2B
3	16675026	16775026	0.342725	GUCA2A
3	16675026	16775026	0.342725	FOXJ3
3	16675168	16775168	0.355728	GUCA2B
3	16675168	16775168	0.355728	GUCA2A
3	16675168	16775168	0.355728	FOXJ3
3	16675258	16775258	0.353284	GUCA2B
3	16675258	16775258	0.353284	GUCA2A
3	16675258	16775258	0.353284	FOXJ3
3	16675267	16775267	0.353284	GUCA2B
3	16675267	16775267	0.353284	GUCA2A
3	16675267	16775267	0.353284	FOXJ3
3	16675339	16775339	0.353284	GUCA2B
3	16675339	16775339	0.353284	GUCA2A
3	16675339	16775339	0.353284	FOXJ3
3	16675361	16775361	0.35217	GUCA2B
3	16675361	16775361	0.35217	GUCA2A
3	16675361	16775361	0.35217	FOXJ3
3	16675484	16775484	0.35217	GUCA2B
3	16675484	16775484	0.35217	GUCA2A
3	16675484	16775484	0.35217	FOXJ3
3	16675525	16775525	0.352245	GUCA2B
3	16675525	16775525	0.352245	GUCA2A
3	16675525	16775525	0.352245	FOXJ3
3	16675547	16775547	0.353314	GUCA2B
3	16675547	16775547	0.353314	GUCA2A
3	16675547	16775547	0.353314	FOXJ3
3	16675584	16775584	0.360022	GUCA2B
3	16675584	16775584	0.360022	GUCA2A
3	16675584	16775584	0.360022	FOXJ3
3	16675629	16775629	0.358815	GUCA2B
3	16675629	16775629	0.358815	GUCA2A
3	16675629	16775629	0.358815	FOXJ3
3	16675876	16775876	0.358815	GUCA2B

3	16675876	16775876	0.358815	GUCA2A
3	16675876	16775876	0.358815	FOXJ3
3	16675961	16775961	0.358815	GUCA2B
3	16675961	16775961	0.358815	GUCA2A
3	16675961	16775961	0.358815	FOXJ3
3	16675963	16775963	0.358815	GUCA2B
3	16675963	16775963	0.358815	GUCA2A
3	16675963	16775963	0.358815	FOXJ3
3	16675992	16775992	0.381313	GUCA2B
3	16675992	16775992	0.381313	GUCA2A
3	16675992	16775992	0.381313	FOXJ3
3	16675999	16775999	0.375162	GUCA2B
3	16675999	16775999	0.375162	GUCA2A
3	16675999	16775999	0.375162	FOXJ3
3	16676016	16776016	0.375162	GUCA2B
3	16676016	16776016	0.375162	GUCA2A
3	16676016	16776016	0.375162	FOXJ3
3	16676030	16776030	0.375917	GUCA2B
3	16676030	16776030	0.375917	GUCA2A
3	16676030	16776030	0.375917	FOXJ3
3	16676268	16776268	0.34972	GUCA2B
3	16676268	16776268	0.34972	GUCA2A
3	16676268	16776268	0.34972	FOXJ3
3	16676321	16776321	0.348828	GUCA2B
3	16676321	16776321	0.348828	GUCA2A
3	16676321	16776321	0.348828	FOXJ3
3	16677162	16777162	0.36626	GUCA2B
3	16677162	16777162	0.36626	GUCA2A
3	16677162	16777162	0.36626	FOXJ3
3	16677170	16777170	0.36632	GUCA2B
3	16677170	16777170	0.36632	GUCA2A
3	16677170	16777170	0.36632	FOXJ3
3	16677211	16777211	0.362877	GUCA2B
3	16677211	16777211	0.362877	GUCA2A
3	16677211	16777211	0.362877	FOXJ3
3	16677275	16777275	0.362993	GUCA2B
3	16677275	16777275	0.362993	GUCA2A
3	16677275	16777275	0.362993	FOXJ3
3	16677291	16777291	0.357297	GUCA2B
3	16677291	16777291	0.357297	GUCA2A
3	16677291	16777291	0.357297	FOXJ3
3	16677628	16777628	0.356807	GUCA2B
3	16677628	16777628	0.356807	GUCA2A
3	16677628	16777628	0.356807	FOXJ3
3	16677999	16777999	0.360771	GUCA2B
3	16677999	16777999	0.360771	GUCA2A
3	16677999	16777999	0.360771	FOXJ3
3	16678273	16778273	0.360662	GUCA2B
3	16678273	16778273	0.360662	GUCA2A
3	16678273	16778273	0.360662	FOXJ3
3	23377933	23477933	0.359919	LOC106501971
3	23405132	23505132	0.345025	LOC106501971
3	23406317	23506317	0.350904	LOC106501971
3	23406708	23506708	0.345783	LOC106501971
3	23407000	23507000	0.394539	LOC106501971
3	23407633	23507633	0.388849	LOC106501971
3	23407824	23507824	0.387692	LOC106501971
3	23408036	23508036	0.383058	LOC106501971

3	23408291	23508291	0.402886	LOC106501971
3	23408391	23508391	0.410128	LOC106501971
3	23408711	23508711	0.3812	LOC106501971
3	23408873	23508873	0.391417	LOC106501971
3	23409582	23509582	0.386241	LOC106501971
3	23409693	23509693	0.395179	LOC106501971
3	23409725	23509725	0.385509	LOC106501971
3	23409905	23509905	0.383896	LOC106501971
3	23410285	23510285	0.386895	LOC106501971
3	23410471	23510471	0.386895	LOC106501971
3	23410641	23510641	0.386895	LOC106501971
3	23410796	23510796	0.411931	LOC106501971
3	23411463	23511463	0.388587	LOC106501971
3	23411464	23511464	0.392965	LOC106501971
3	23411483	23511483	0.392998	LOC106501971
3	23412561	23512561	0.383572	LOC106501971
3	23412714	23512714	0.395614	LOC106501971
3	23412899	23512899	0.38766	LOC106501971
3	23416627	23516627	0.350223	LOC106501971
3	23416750	23516750	0.35552	LOC106501971
3	23417076	23517076	0.362689	LOC106501971
3	23417588	23517588	0.359853	LOC106501971
3	23417618	23517618	0.355227	LOC106501971
3	23418161	23518161	0.354931	LOC106501971
3	23418244	23518244	0.376257	LOC106501971
3	26978216	27078216	0.349092	ZCCHC11
3	26978234	27078234	0.369853	ZCCHC11
3	26978240	27078240	0.370201	ZCCHC11
3	26978551	27078551	0.363309	ZCCHC11
3	26978633	27078633	0.371324	ZCCHC11
3	26978642	27078642	0.34266	ZCCHC11
3	26979728	27079728	0.353215	GPX7
3	26979728	27079728	0.353215	ZCCHC11
3	29250796	29350796	0.363656	PARS2
3	29250796	29350796	0.363656	TTC22
3	29250796	29350796	0.363656	LEXM
3	29250796	29350796	0.363656	DHCR24
3	29250827	29350827	0.360455	PARS2
3	29250827	29350827	0.360455	TTC22
3	29250827	29350827	0.360455	LEXM
3	29250827	29350827	0.360455	DHCR24
3	29250843	29350843	0.378975	PARS2
3	29250843	29350843	0.378975	TTC22
3	29250843	29350843	0.378975	LEXM
3	29250843	29350843	0.378975	DHCR24
3	29250853	29350853	0.357304	PARS2
3	29250853	29350853	0.357304	TTC22
3	29250853	29350853	0.357304	LEXM
3	29250853	29350853	0.357304	DHCR24
3	29250882	29350882	0.357137	PARS2
3	29250882	29350882	0.357137	TTC22
3	29250882	29350882	0.357137	LEXM
3	29250882	29350882	0.357137	DHCR24
3	34701552	34801552	0.357979	HOOK1
3	34701552	34801552	0.357979	LOC102184867
3	34701552	34801552	0.357979	LOC102191099
3	34701561	34801561	0.367642	HOOK1
3	34701561	34801561	0.367642	LOC102184867

3	34701561	34801561	0.367642	LOC102191099
3	34701564	34801564	0.367642	HOOK1
3	34701564	34801564	0.367642	LOC102184867
3	34701564	34801564	0.367642	LOC102191099
3	34701570	34801570	0.367162	HOOK1
3	34701570	34801570	0.367162	LOC102184867
3	34701570	34801570	0.367162	LOC102191099
3	34701590	34801590	0.355026	HOOK1
3	34701590	34801590	0.355026	LOC102184867
3	34701590	34801590	0.355026	LOC102191099
3	34701601	34801601	0.355431	HOOK1
3	34701601	34801601	0.355431	LOC102184867
3	34701601	34801601	0.355431	LOC102191099
3	34701605	34801605	0.355431	HOOK1
3	34701605	34801605	0.355431	LOC102184867
3	34701605	34801605	0.355431	LOC102191099
3	34701640	34801640	0.341662	HOOK1
3	34701640	34801640	0.341662	LOC102184867
3	34701640	34801640	0.341662	LOC102191099
3	34701686	34801686	0.352628	HOOK1
3	34701686	34801686	0.352628	LOC102184867
3	34701686	34801686	0.352628	LOC102191099
3	34701723	34801723	0.344122	HOOK1
3	34701723	34801723	0.344122	LOC102184867
3	34701723	34801723	0.344122	LOC102191099
3	34701724	34801724	0.344122	HOOK1
3	34701724	34801724	0.344122	LOC102184867
3	34701724	34801724	0.344122	LOC102191099
3	34702731	34802731	0.358341	HOOK1
3	34702731	34802731	0.358341	LOC102184867
3	34702731	34802731	0.358341	LOC102191099
3	34702802	34802802	0.357208	HOOK1
3	34702802	34802802	0.357208	LOC102184867
3	34702802	34802802	0.357208	LOC102191099
3	34702844	34802844	0.357407	HOOK1
3	34702844	34802844	0.357407	LOC102184867
3	34702844	34802844	0.357407	LOC102191099
3	34702871	34802871	0.357458	HOOK1
3	34702871	34802871	0.357458	LOC102184867
3	34702871	34802871	0.357458	LOC102191099
3	34702914	34802914	0.353799	HOOK1
3	34702914	34802914	0.353799	LOC102184867
3	34702914	34802914	0.353799	LOC102191099
3	34702935	34802935	0.368669	HOOK1
3	34702935	34802935	0.368669	LOC102184867
3	34702935	34802935	0.368669	LOC102191099
3	34702968	34802968	0.35324	HOOK1
3	34702968	34802968	0.35324	LOC102184867
3	34702968	34802968	0.35324	LOC102191099
3	34702974	34802974	0.35324	HOOK1
3	34702974	34802974	0.35324	LOC102184867
3	34702974	34802974	0.35324	LOC102191099
3	34702977	34802977	0.35324	HOOK1
3	34702977	34802977	0.35324	LOC102184867
3	34702977	34802977	0.35324	LOC102191099
3	34703009	34803009	0.348512	HOOK1
3	34703009	34803009	0.348512	LOC102184867
3	34703009	34803009	0.348512	LOC102191099

3	34703010	34803010	0.348512	HOOK1
3	34703010	34803010	0.348512	LOC102184867
3	34703010	34803010	0.348512	LOC102191099
3	34703014	34803014	0.348512	HOOK1
3	34703014	34803014	0.348512	LOC102184867
3	34703014	34803014	0.348512	LOC102191099
3	34703015	34803015	0.348512	HOOK1
3	34703015	34803015	0.348512	LOC102184867
3	34703015	34803015	0.348512	LOC102191099
3	34703029	34803029	0.348512	HOOK1
3	34703029	34803029	0.348512	LOC102184867
3	34703029	34803029	0.348512	LOC102191099
3	34703047	34803047	0.348512	HOOK1
3	34703047	34803047	0.348512	LOC102184867
3	34703047	34803047	0.348512	LOC102191099
3	34703096	34803096	0.361517	HOOK1
3	34703096	34803096	0.361517	LOC102184867
3	34703096	34803096	0.361517	LOC102191099
3	34703102	34803102	0.361517	HOOK1
3	34703102	34803102	0.361517	LOC102184867
3	34703102	34803102	0.361517	LOC102191099
3	34703127	34803127	0.361517	HOOK1
3	34703127	34803127	0.361517	LOC102184867
3	34703127	34803127	0.361517	LOC102191099
3	34703179	34803179	0.360246	HOOK1
3	34703179	34803179	0.360246	LOC102184867
3	34703179	34803179	0.360246	LOC102191099
3	34703185	34803185	0.405223	HOOK1
3	34703185	34803185	0.405223	LOC102184867
3	34703185	34803185	0.405223	LOC102191099
3	36202568	36302568	0.361248	NFIA
3	36234450	36334450	0.343919	NFIA
3	36234481	36334481	0.361484	NFIA
3	38969900	39069900	0.342438	PGM1
3	38971770	39071770	0.348659	PGM1
3	38972203	39072203	0.344165	PGM1
3	38974280	39074280	0.343372	PGM1
3	38974548	39074548	0.347089	PGM1
3	38974909	39074909	0.348385	PGM1
3	38974951	39074951	0.355759	PGM1
3	38975846	39075846	0.342095	PGM1
3	38976614	39076614	0.341658	PGM1
3	38976676	39076676	0.352584	PGM1
3	38977221	39077221	0.353718	PGM1
3	38977343	39077343	0.343017	PGM1
3	38977368	39077368	0.347273	PGM1
3	38977380	39077380	0.34638	PGM1
3	38986723	39086723	0.347679	PGM1
3	38992694	39092694	0.343803	PGM1
3	38992784	39092784	0.341885	PGM1
3	38992854	39092854	0.341885	PGM1
3	38992878	39092878	0.343005	PGM1
3	38992922	39092922	0.342887	PGM1
3	39002757	39102757	0.3459	PGM1
3	39003010	39103010	0.354697	PGM1
3	39020828	39120828	0.342009	ROR1
3	39020848	39120848	0.34789	ROR1
3	39021247	39121247	0.353355	ROR1

3	40562342	40662342	0.344369	MIR101
3	40562342	40662342	0.344369	LOC108635646
3	40567956	40667956	0.346308	MIR101
3	40567956	40667956	0.346308	LOC108635646
3	40569039	40669039	0.344413	MIR101
3	40569039	40669039	0.344413	LOC108635646
3	40569104	40669104	0.342225	MIR101
3	40569104	40669104	0.342225	LOC108635646
3	40574390	40674390	0.357319	MIR101
3	40574390	40674390	0.357319	LOC108635646
3	40574866	40674866	0.344787	MIR101
3	40574866	40674866	0.344787	LOC108635646
3	40575045	40675045	0.345267	MIR101
3	40575045	40675045	0.345267	LOC108635646
3	40575256	40675256	0.348358	MIR101
3	40575256	40675256	0.348358	LOC108635646
3	40575871	40675871	0.342616	MIR101
3	40575871	40675871	0.342616	LOC108635646
3	40575872	40675872	0.347848	MIR101
3	40575872	40675872	0.347848	LOC108635646
3	40579251	40679251	0.380617	MIR101
3	40579251	40679251	0.380617	LOC108635646
3	40579251	40679251	0.380617	AK4
3	40579253	40679253	0.424939	MIR101
3	40579253	40679253	0.424939	LOC108635646
3	40579253	40679253	0.424939	AK4
3	40579255	40679255	0.424939	MIR101
3	40579255	40679255	0.424939	LOC108635646
3	40579255	40679255	0.424939	AK4
3	40579295	40679295	0.40531	MIR101
3	40579295	40679295	0.40531	LOC108635646
3	40579295	40679295	0.40531	AK4
3	40579314	40679314	0.40586	MIR101
3	40579314	40679314	0.40586	LOC108635646
3	40579314	40679314	0.40586	AK4
3	40579374	40679374	0.402094	MIR101
3	40579374	40679374	0.402094	LOC108635646
3	40579374	40679374	0.402094	AK4
3	40579432	40679432	0.355571	MIR101
3	40579432	40679432	0.355571	LOC108635646
3	40579432	40679432	0.355571	AK4
3	47490472	47590472	0.417023	NEGR1
3	47490530	47590530	0.41572	NEGR1
3	47490541	47590541	0.415682	NEGR1
3	47490664	47590664	0.428439	NEGR1
3	47490694	47590694	0.439271	NEGR1
3	47490755	47590755	0.475623	NEGR1
3	47490827	47590827	0.435477	NEGR1
3	47490969	47590969	0.39671	NEGR1
3	47490999	47590999	0.419048	NEGR1
3	47491031	47591031	0.407806	NEGR1
3	47491082	47591082	0.419991	NEGR1
3	47491385	47591385	0.427606	NEGR1
3	47491440	47591440	0.426877	NEGR1
3	47491776	47591776	0.432029	NEGR1
3	47491865	47591865	0.435019	NEGR1
3	47491878	47591878	0.432087	NEGR1
3	47491885	47591885	0.432087	NEGR1

3	47491913	47591913	0.432699	NEGR1
3	47491928	47591928	0.42763	NEGR1
3	47492070	47592070	0.434077	NEGR1
3	47492118	47592118	0.438369	NEGR1
3	47492126	47592126	0.431874	NEGR1
3	47492216	47592216	0.433362	NEGR1
3	47492224	47592224	0.427883	NEGR1
3	47492337	47592337	0.427883	NEGR1
3	47492349	47592349	0.437607	NEGR1
3	47492395	47592395	0.437607	NEGR1
3	47492396	47592396	0.437607	NEGR1
3	47492446	47592446	0.437607	NEGR1
3	47492474	47592474	0.444878	NEGR1
3	47492508	47592508	0.443555	NEGR1
3	47492520	47592520	0.444268	NEGR1
3	47492597	47592597	0.445972	NEGR1
3	47492645	47592645	0.458704	NEGR1
3	47492649	47592649	0.453035	NEGR1
3	47492676	47592676	0.457636	NEGR1
3	47492705	47592705	0.458038	NEGR1
3	47492722	47592722	0.458068	NEGR1
3	47492723	47592723	0.458068	NEGR1
3	47492749	47592749	0.457754	NEGR1
3	47492758	47592758	0.458329	NEGR1
3	47492759	47592759	0.459514	NEGR1
3	47492798	47592798	0.453886	NEGR1
3	47492799	47592799	0.456867	NEGR1
3	47492894	47592894	0.475985	NEGR1
3	47492896	47592896	0.458747	NEGR1
3	47493038	47593038	0.435325	NEGR1
3	47493041	47593041	0.435325	NEGR1
3	47493044	47593044	0.435325	NEGR1
3	47493049	47593049	0.435325	NEGR1
3	47493073	47593073	0.435325	NEGR1
3	47493096	47593096	0.435318	NEGR1
3	47493139	47593139	0.435318	NEGR1
3	47493216	47593216	0.435416	NEGR1
3	47493230	47593230	0.435352	NEGR1
3	47493323	47593323	0.43544	NEGR1
3	47493344	47593344	0.43544	NEGR1
3	47493368	47593368	0.440239	NEGR1
3	47493371	47593371	0.44158	NEGR1
3	47493379	47593379	0.440834	NEGR1
3	47493412	47593412	0.443936	NEGR1
3	47493418	47593418	0.442279	NEGR1
3	47493458	47593458	0.4312	NEGR1
3	47493477	47593477	0.431192	NEGR1
3	47493537	47593537	0.431997	NEGR1
3	47493710	47593710	0.432821	NEGR1
3	47493719	47593719	0.431213	NEGR1
3	47493751	47593751	0.431213	NEGR1
3	47493825	47593825	0.434588	NEGR1
3	47493842	47593842	0.434581	NEGR1
3	47493845	47593845	0.434581	NEGR1
3	47493851	47593851	0.438455	NEGR1
3	47493856	47593856	0.438455	NEGR1
3	47493865	47593865	0.438874	NEGR1
3	47493869	47593869	0.443506	NEGR1

3	47493881	47593881	0.444398	NEGR1
3	47493908	47593908	0.446422	NEGR1
3	47493955	47593955	0.445588	NEGR1
3	47494011	47594011	0.447196	NEGR1
3	47494042	47594042	0.446259	NEGR1
3	47494064	47594064	0.427553	NEGR1
3	47494075	47594075	0.42749	NEGR1
3	47494085	47594085	0.432902	NEGR1
3	47494133	47594133	0.378061	NEGR1
3	47494153	47594153	0.383584	NEGR1
3	47494161	47594161	0.366863	NEGR1
3	47494180	47594180	0.403828	NEGR1
3	47494221	47594221	0.398186	NEGR1
3	47936540	48036540	0.344771	NEGR1
3	47936784	48036784	0.344458	NEGR1
3	47936847	48036847	0.3551	NEGR1
3	47936876	48036876	0.347317	NEGR1
3	47936877	48036877	0.348041	NEGR1
3	47936882	48036882	0.347131	NEGR1
3	47936883	48036883	0.345712	NEGR1
3	47951832	48051832	0.343998	NEGR1
3	47952253	48052253	0.343998	NEGR1
3	47952849	48052849	0.359978	NEGR1
3	47952853	48052853	0.351078	NEGR1
3	50490853	50590853	0.355196	LOC102170378
3	50490880	50590880	0.34555	LOC102170378
3	50490885	50590885	0.379396	LOC102170378
3	50490892	50590892	0.371036	LOC102170378
3	50490919	50590919	0.366822	LOC102170378
3	50490935	50590935	0.370881	LOC102170378
3	50490967	50590967	0.359559	LOC102170378
3	50491107	50591107	0.348977	LOC102170378
3	50491157	50591157	0.372883	LOC102170378
3	50491242	50591242	0.376047	LOC102170378
3	50491275	50591275	0.375443	LOC102170378
3	50491322	50591322	0.369591	LOC102170378
3	50491334	50591334	0.364986	LOC102170378
3	50491350	50591350	0.355404	LOC102170378
3	50491369	50591369	0.364328	LOC102170378
3	50491402	50591402	0.34925	LOC102170378
3	50491567	50591567	0.347216	LOC102170378
3	50491578	50591578	0.352376	LOC102170378
3	50491592	50591592	0.350909	LOC102170378
3	50491613	50591613	0.354159	LOC102170378
3	50491638	50591638	0.347408	LOC102170378
3	50491668	50591668	0.342657	LOC102170378
3	50495462	50595462	0.366563	LOC102170378
3	50495529	50595529	0.354056	LOC102170378
3	51596534	51696534	0.341639	TRNAC-GCA-45
3	51596841	51696841	0.357052	TRNAC-GCA-45
3	54022760	54122760	0.366365	AK5
3	54022760	54122760	0.366365	ZZZ3
3	54022783	54122783	0.422784	AK5
3	54022783	54122783	0.422784	ZZZ3
3	54022787	54122787	0.385212	AK5
3	54022787	54122787	0.385212	ZZZ3
3	54022808	54122808	0.389763	AK5
3	54022808	54122808	0.389763	ZZZ3

3	54022848	54122848	0.361072	AK5
3	54022848	54122848	0.361072	ZZZ3
3	54022850	54122850	0.365516	AK5
3	54022850	54122850	0.365516	ZZZ3
3	54022857	54122857	0.347579	AK5
3	54022857	54122857	0.347579	ZZZ3
3	54022883	54122883	0.342861	AK5
3	54022883	54122883	0.342861	ZZZ3
3	54032992	54132992	0.342416	AK5
3	54032992	54132992	0.342416	ZZZ3
3	54033078	54133078	0.342944	AK5
3	54033078	54133078	0.342944	ZZZ3
3	54033320	54133320	0.342339	AK5
3	54033320	54133320	0.342339	ZZZ3
3	54033642	54133642	0.350779	AK5
3	54033642	54133642	0.350779	ZZZ3
3	54033654	54133654	0.373173	AK5
3	54033654	54133654	0.373173	ZZZ3
3	54033984	54133984	0.342807	AK5
3	54033984	54133984	0.342807	ZZZ3
3	54167692	54267692	0.347546	ZZZ3
3	54167692	54267692	0.347546	USP33
3	54167699	54267699	0.348948	ZZZ3
3	54167699	54267699	0.348948	USP33
3	54167703	54267703	0.346259	ZZZ3
3	54167703	54267703	0.346259	USP33
3	54167704	54267704	0.342597	ZZZ3
3	54167704	54267704	0.342597	USP33
3	54171514	54271514	0.344198	ZZZ3
3	54171514	54271514	0.344198	USP33
3	54172725	54272725	0.353463	ZZZ3
3	54172725	54272725	0.353463	USP33
3	54173770	54273770	0.350967	ZZZ3
3	54173770	54273770	0.350967	USP33
3	54179326	54279326	0.388266	ZZZ3
3	54179326	54279326	0.388266	USP33
3	54179526	54279526	0.35108	ZZZ3
3	54179526	54279526	0.35108	USP33
3	54179632	54279632	0.35123	ZZZ3
3	54179632	54279632	0.35123	USP33
3	54179872	54279872	0.35953	ZZZ3
3	54179872	54279872	0.35953	USP33
3	54180034	54280034	0.345697	ZZZ3
3	54180034	54280034	0.345697	USP33
3	54180425	54280425	0.346513	ZZZ3
3	54180425	54280425	0.346513	USP33
3	54180539	54280539	0.354568	ZZZ3
3	54180539	54280539	0.354568	USP33
3	54180670	54280670	0.354568	ZZZ3
3	54180670	54280670	0.354568	USP33
3	54180738	54280738	0.343546	ZZZ3
3	54180738	54280738	0.343546	USP33
3	54180747	54280747	0.345818	ZZZ3
3	54180747	54280747	0.345818	USP33
3	54180952	54280952	0.34419	ZZZ3
3	54180952	54280952	0.34419	USP33
3	54181561	54281561	0.351281	ZZZ3
3	54181561	54281561	0.351281	USP33

3	54182876	54282876	0.352865	ZZZ3
3	54182876	54282876	0.352865	USP33
3	54183426	54283426	0.347237	ZZZ3
3	54183426	54283426	0.347237	USP33
3	54183433	54283433	0.353616	ZZZ3
3	54183433	54283433	0.353616	USP33
3	54183652	54283652	0.34836	ZZZ3
3	54183652	54283652	0.34836	USP33
3	54191648	54291648	0.363011	ZZZ3
3	54191648	54291648	0.363011	USP33
3	54191668	54291668	0.362356	ZZZ3
3	54191668	54291668	0.362356	USP33
3	54191970	54291970	0.355829	ZZZ3
3	54191970	54291970	0.355829	USP33
3	54192554	54292554	0.371967	ZZZ3
3	54192554	54292554	0.371967	USP33
3	54192555	54292555	0.375188	ZZZ3
3	54192555	54292555	0.375188	USP33
3	54192750	54292750	0.361437	ZZZ3
3	54192750	54292750	0.361437	USP33
3	54193038	54293038	0.372035	ZZZ3
3	54193038	54293038	0.372035	USP33
3	54193471	54293471	0.371138	ZZZ3
3	54193471	54293471	0.371138	USP33
3	54193555	54293555	0.403271	ZZZ3
3	54193555	54293555	0.403271	USP33
3	54193746	54293746	0.404442	ZZZ3
3	54193746	54293746	0.404442	USP33
3	54193932	54293932	0.401958	ZZZ3
3	54193932	54293932	0.401958	USP33
3	54194157	54294157	0.396846	ZZZ3
3	54194157	54294157	0.396846	USP33
3	54194206	54294206	0.386749	ZZZ3
3	54194206	54294206	0.386749	USP33
3	54194207	54294207	0.386749	ZZZ3
3	54194207	54294207	0.386749	USP33
3	54194212	54294212	0.386749	ZZZ3
3	54194212	54294212	0.386749	USP33
3	54194226	54294226	0.386749	ZZZ3
3	54194226	54294226	0.386749	USP33
3	54194230	54294230	0.386749	ZZZ3
3	54194230	54294230	0.386749	USP33
3	54194307	54294307	0.356458	ZZZ3
3	54194307	54294307	0.356458	USP33
3	54194310	54294310	0.351756	ZZZ3
3	54194310	54294310	0.351756	USP33
3	54194321	54294321	0.3992	ZZZ3
3	54194321	54294321	0.3992	USP33
3	54195457	54295457	0.376353	ZZZ3
3	54195457	54295457	0.376353	USP33
3	54195645	54295645	0.376353	ZZZ3
3	54195645	54295645	0.376353	USP33
3	54196008	54296008	0.369415	ZZZ3
3	54196008	54296008	0.369415	USP33
3	54196861	54296861	0.392591	ZZZ3
3	54196861	54296861	0.392591	USP33
3	54197095	54297095	0.397024	ZZZ3
3	54197095	54297095	0.397024	USP33

3	54197462	54297462	0.352626	ZZZ3
3	54197462	54297462	0.352626	USP33
3	54197544	54297544	0.352626	ZZZ3
3	54197544	54297544	0.352626	USP33
3	54198198	54298198	0.359613	ZZZ3
3	54198198	54298198	0.359613	USP33
3	54198441	54298441	0.355997	ZZZ3
3	54198441	54298441	0.355997	USP33
3	54202187	54302187	0.35465	ZZZ3
3	54202187	54302187	0.35465	USP33
3	54202285	54302285	0.36204	ZZZ3
3	54202285	54302285	0.36204	USP33
3	54202903	54302903	0.379584	ZZZ3
3	54202903	54302903	0.379584	USP33
3	54203563	54303563	0.379584	ZZZ3
3	54203563	54303563	0.379584	USP33
3	54203670	54303670	0.398223	ZZZ3
3	54203670	54303670	0.398223	USP33
3	54204072	54304072	0.402785	ZZZ3
3	54204072	54304072	0.402785	USP33
3	54204107	54304107	0.402785	ZZZ3
3	54204107	54304107	0.402785	USP33
3	54204817	54304817	0.40737	ZZZ3
3	54204817	54304817	0.40737	USP33
3	54204825	54304825	0.410582	ZZZ3
3	54204825	54304825	0.410582	USP33
3	54205131	54305131	0.410582	ZZZ3
3	54205131	54305131	0.410582	USP33
3	54205586	54305586	0.416805	ZZZ3
3	54205586	54305586	0.416805	USP33
3	54205632	54305632	0.393366	ZZZ3
3	54205632	54305632	0.393366	USP33
3	54206849	54306849	0.393366	ZZZ3
3	54206849	54306849	0.393366	USP33
3	54207315	54307315	0.415884	ZZZ3
3	54207315	54307315	0.415884	USP33
3	54207612	54307612	0.414055	ZZZ3
3	54207612	54307612	0.414055	USP33
3	54207922	54307922	0.427129	ZZZ3
3	54207922	54307922	0.427129	USP33
3	54207984	54307984	0.424397	ZZZ3
3	54207984	54307984	0.424397	USP33
3	54208104	54308104	0.411996	ZZZ3
3	54208104	54308104	0.411996	USP33
3	54208344	54308344	0.391667	ZZZ3
3	54208344	54308344	0.391667	USP33
3	54209516	54309516	0.41789	ZZZ3
3	54209516	54309516	0.41789	USP33
3	54209728	54309728	0.437702	ZZZ3
3	54209728	54309728	0.437702	USP33
3	54209931	54309931	0.445515	ZZZ3
3	54209931	54309931	0.445515	USP33
3	54210235	54310235	0.405413	ZZZ3
3	54210235	54310235	0.405413	USP33
3	54210431	54310431	0.409441	ZZZ3
3	54210431	54310431	0.409441	USP33
3	54210651	54310651	0.398677	ZZZ3
3	54210651	54310651	0.398677	USP33

3	54211199	54311199	0.376453	ZZZ3
3	54211199	54311199	0.376453	USP33
3	54211686	54311686	0.394523	ZZZ3
3	54211686	54311686	0.394523	USP33
3	54211952	54311952	0.362754	ZZZ3
3	54211952	54311952	0.362754	USP33
3	54212029	54312029	0.364783	ZZZ3
3	54212029	54312029	0.364783	USP33
3	54212153	54312153	0.350012	ZZZ3
3	54212153	54312153	0.350012	USP33
3	54212236	54312236	0.358383	ZZZ3
3	54212236	54312236	0.358383	USP33
3	54212415	54312415	0.367835	ZZZ3
3	54212415	54312415	0.367835	USP33
3	54212585	54312585	0.35286	ZZZ3
3	54212585	54312585	0.35286	USP33
3	54212766	54312766	0.350084	ZZZ3
3	54212766	54312766	0.350084	USP33
3	54213928	54313928	0.341987	ZZZ3
3	54213928	54313928	0.341987	USP33
3	54214366	54314366	0.388879	ZZZ3
3	54214366	54314366	0.388879	USP33
3	54214597	54314597	0.364964	ZZZ3
3	54214597	54314597	0.364964	USP33
3	54215361	54315361	0.371153	ZZZ3
3	54215361	54315361	0.371153	USP33
3	54215446	54315446	0.395311	ZZZ3
3	54215446	54315446	0.395311	USP33
3	54215469	54315469	0.381098	ZZZ3
3	54215469	54315469	0.381098	USP33
3	54216826	54316826	0.342802	ZZZ3
3	54216826	54316826	0.342802	USP33
3	54216913	54316913	0.352884	ZZZ3
3	54216913	54316913	0.352884	USP33
3	54217174	54317174	0.352884	ZZZ3
3	54217174	54317174	0.352884	USP33
3	54217350	54317350	0.346675	ZZZ3
3	54217350	54317350	0.346675	USP33
3	54217783	54317783	0.357771	USP33
3	54218453	54318453	0.390318	USP33
3	54218582	54318582	0.412247	USP33
3	54219028	54319028	0.394616	USP33
3	54219028	54319028	0.394616	MIGA1
3	54219381	54319381	0.427528	USP33
3	54219381	54319381	0.427528	MIGA1
3	54220221	54320221	0.418609	USP33
3	54220221	54320221	0.418609	MIGA1
3	54220267	54320267	0.426884	USP33
3	54220267	54320267	0.426884	MIGA1
3	54220302	54320302	0.429309	USP33
3	54220302	54320302	0.429309	MIGA1
3	54220461	54320461	0.442507	USP33
3	54220461	54320461	0.442507	MIGA1
3	54220495	54320495	0.461204	USP33
3	54220495	54320495	0.461204	MIGA1
3	54220508	54320508	0.452221	USP33
3	54220508	54320508	0.452221	MIGA1
3	54220620	54320620	0.452221	USP33

3	54220620	54320620	0.452221	MIGA1
3	54220755	54320755	0.455194	USP33
3	54220755	54320755	0.455194	MIGA1
3	54220785	54320785	0.467745	USP33
3	54220785	54320785	0.467745	MIGA1
3	54221305	54321305	0.446213	USP33
3	54221305	54321305	0.446213	MIGA1
3	54221651	54321651	0.463688	USP33
3	54221651	54321651	0.463688	MIGA1
3	54221971	54321971	0.472299	USP33
3	54221971	54321971	0.472299	MIGA1
3	54221978	54321978	0.43727	USP33
3	54221978	54321978	0.43727	MIGA1
3	54222080	54322080	0.44226	USP33
3	54222080	54322080	0.44226	MIGA1
3	54222303	54322303	0.451098	USP33
3	54222303	54322303	0.451098	MIGA1
3	54222812	54322812	0.438394	USP33
3	54222812	54322812	0.438394	MIGA1
3	54222845	54322845	0.441963	USP33
3	54222845	54322845	0.441963	MIGA1
3	54222855	54322855	0.441713	USP33
3	54222855	54322855	0.441713	MIGA1
3	54223002	54323002	0.441977	USP33
3	54223002	54323002	0.441977	MIGA1
3	54223209	54323209	0.457233	USP33
3	54223209	54323209	0.457233	MIGA1
3	54223263	54323263	0.444779	USP33
3	54223263	54323263	0.444779	MIGA1
3	54223659	54323659	0.444495	USP33
3	54223659	54323659	0.444495	MIGA1
3	54223775	54323775	0.417601	USP33
3	54223775	54323775	0.417601	MIGA1
3	54223791	54323791	0.417601	USP33
3	54223791	54323791	0.417601	MIGA1
3	54223956	54323956	0.418021	USP33
3	54223956	54323956	0.418021	MIGA1
3	54224125	54324125	0.438231	USP33
3	54224125	54324125	0.438231	MIGA1
3	54224150	54324150	0.412312	USP33
3	54224150	54324150	0.412312	MIGA1
3	54224165	54324165	0.407896	USP33
3	54224165	54324165	0.407896	MIGA1
3	54224428	54324428	0.405373	USP33
3	54224428	54324428	0.405373	MIGA1
3	54224462	54324462	0.370879	USP33
3	54224462	54324462	0.370879	MIGA1
3	54227259	54327259	0.367441	USP33
3	54227259	54327259	0.367441	MIGA1
3	55042655	55142655	0.357815	PTGFR
3	55042678	55142678	0.359717	PTGFR
3	55042962	55142962	0.35961	PTGFR
3	55043028	55143028	0.35961	PTGFR
3	55043047	55143047	0.35961	PTGFR
3	55043118	55143118	0.357492	PTGFR
3	55043155	55143155	0.357455	PTGFR
3	55043156	55143156	0.37863	PTGFR
3	55043466	55143466	0.360196	PTGFR

3	55043664	55143664	0.364593	PTGFR
3	55043910	55143910	0.363984	PTGFR
3	55044362	55144362	0.36966	PTGFR
3	55044454	55144454	0.368456	PTGFR
3	55044719	55144719	0.365288	PTGFR
3	55044935	55144935	0.366895	PTGFR
3	55044964	55144964	0.363885	PTGFR
3	55045340	55145340	0.36612	PTGFR
3	55045573	55145573	0.390438	PTGFR
3	55051623	55151623	0.341797	PTGFR
3	55051645	55151645	0.342333	PTGFR
3	55051710	55151710	0.375059	PTGFR
3	55051807	55151807	0.376623	PTGFR
3	55051883	55151883	0.373891	PTGFR
3	55052001	55152001	0.357703	PTGFR
3	55052453	55152453	0.407802	IFI44L
3	55052453	55152453	0.407802	PTGFR
3	55052456	55152456	0.407308	IFI44L
3	55052456	55152456	0.407308	PTGFR
3	55124117	55224117	0.350949	IFI44L
3	55124117	55224117	0.350949	IFI44
3	55124379	55224379	0.358477	IFI44L
3	55124379	55224379	0.358477	IFI44
3	55124386	55224386	0.359683	IFI44L
3	55124386	55224386	0.359683	IFI44
3	55124427	55224427	0.367592	IFI44L
3	55124427	55224427	0.367592	IFI44
3	55124444	55224444	0.36136	IFI44L
3	55124444	55224444	0.36136	IFI44
3	55124473	55224473	0.368529	IFI44L
3	55124473	55224473	0.368529	IFI44
3	55124529	55224529	0.365558	IFI44L
3	55124529	55224529	0.365558	IFI44
3	55124537	55224537	0.364006	IFI44L
3	55124537	55224537	0.364006	IFI44
3	55124551	55224551	0.360662	IFI44L
3	55124551	55224551	0.360662	IFI44
3	55124684	55224684	0.362902	IFI44L
3	55124684	55224684	0.362902	IFI44
3	55124782	55224782	0.347477	IFI44L
3	55124782	55224782	0.347477	IFI44
3	55124815	55224815	0.355981	IFI44L
3	55124815	55224815	0.355981	IFI44
3	55124926	55224926	0.345096	IFI44L
3	55124926	55224926	0.345096	IFI44
3	55124938	55224938	0.34451	IFI44L
3	55124938	55224938	0.34451	IFI44
3	55124940	55224940	0.344645	IFI44L
3	55124940	55224940	0.344645	IFI44
3	55125284	55225284	0.375236	IFI44L
3	55125284	55225284	0.375236	IFI44
3	55129989	55229989	0.371511	IFI44L
3	55129989	55229989	0.371511	IFI44
3	55130450	55230450	0.342779	IFI44L
3	55130450	55230450	0.342779	IFI44
3	55130745	55230745	0.357445	IFI44L
3	55130745	55230745	0.357445	IFI44
3	55130764	55230764	0.354367	IFI44L

3	55130764	55230764	0.354367	IFI44
3	55130951	55230951	0.37219	IFI44L
3	55130951	55230951	0.37219	IFI44
3	55130990	55230990	0.377105	IFI44L
3	55130990	55230990	0.377105	IFI44
3	55130992	55230992	0.377105	IFI44L
3	55130992	55230992	0.377105	IFI44
3	55131027	55231027	0.379215	IFI44L
3	55131027	55231027	0.379215	IFI44
3	55131052	55231052	0.382364	IFI44L
3	55131052	55231052	0.382364	IFI44
3	55131123	55231123	0.37301	IFI44L
3	55131123	55231123	0.37301	IFI44
3	55131153	55231153	0.357467	IFI44L
3	55131153	55231153	0.357467	IFI44
3	55131372	55231372	0.420918	IFI44L
3	55131372	55231372	0.420918	IFI44
3	55521211	55621211	0.359441	ADGRL4
3	55521260	55621260	0.35141	ADGRL4
3	55521302	55621302	0.35141	ADGRL4
3	55521449	55621449	0.374574	ADGRL4
3	55521458	55621458	0.373298	ADGRL4
3	55521996	55621996	0.348072	ADGRL4
3	55522012	55622012	0.349726	ADGRL4
3	55522227	55622227	0.349365	ADGRL4
3	55522306	55622306	0.341859	ADGRL4
3	55522406	55622406	0.341859	ADGRL4
3	55522537	55622537	0.353876	ADGRL4
3	55522543	55622543	0.353876	ADGRL4
3	55522555	55622555	0.344275	ADGRL4
3	55522708	55622708	0.360176	ADGRL4
3	55523140	55623140	0.368999	ADGRL4
3	55523415	55623415	0.368999	ADGRL4
3	55523552	55623552	0.359985	ADGRL4
3	55523627	55623627	0.408093	ADGRL4
3	55523889	55623889	0.356492	ADGRL4
3	55523898	55623898	0.356492	ADGRL4
3	55524157	55624157	0.348222	ADGRL4
3	55525104	55625104	0.346329	ADGRL4
3	55525158	55625158	0.346329	ADGRL4
3	55525164	55625164	0.348469	ADGRL4
3	57822675	57922675	0.34269	LOC102173355
3	57827011	57927011	0.360295	LOC102173355
3	58729980	58829980	0.346607	ADGRL2
3	58730226	58830226	0.358296	ADGRL2
3	58731385	58831385	0.370745	ADGRL2
3	58731713	58831713	0.368381	ADGRL2
3	58732379	58832379	0.357392	ADGRL2
3	58732402	58832402	0.38105	ADGRL2
3	58732536	58832536	0.395822	ADGRL2
3	58732597	58832597	0.385162	ADGRL2
3	58732702	58832702	0.353309	ADGRL2
3	58732883	58832883	0.364498	ADGRL2
3	58733147	58833147	0.35916	ADGRL2
3	58733381	58833381	0.370421	ADGRL2
3	58733829	58833829	0.350392	ADGRL2
3	58733972	58833972	0.348548	ADGRL2
3	58737358	58837358	0.350036	ADGRL2

3	58737523	58837523	0.366431	ADGRL2
3	58737549	58837549	0.352349	ADGRL2
3	58737670	58837670	0.353296	ADGRL2
3	58737712	58837712	0.354965	ADGRL2
3	58737858	58837858	0.39327	ADGRL2
3	58737926	58837926	0.359661	ADGRL2
3	58737978	58837978	0.354245	ADGRL2
3	58738638	58838638	0.349677	ADGRL2
3	58738815	58838815	0.349677	ADGRL2
3	58739080	58839080	0.347497	ADGRL2
3	58739219	58839219	0.35427	ADGRL2
3	58739392	58839392	0.401098	ADGRL2
3	58739831	58839831	0.348107	ADGRL2
3	58742741	58842741	0.356352	ADGRL2
3	58742781	58842781	0.354306	ADGRL2
3	58742880	58842880	0.345938	ADGRL2
3	66315376	66415376	0.34366	LOC102181554
3	66315376	66415376	0.34366	LOC102170144
3	66315377	66415377	0.34366	LOC102181554
3	66315377	66415377	0.34366	LOC102170144
3	66315391	66415391	0.376613	LOC102181554
3	66315391	66415391	0.376613	LOC102170144
3	66315404	66415404	0.359536	LOC102181554
3	66315404	66415404	0.359536	LOC102170144
3	66315405	66415405	0.365304	LOC102181554
3	66315405	66415405	0.365304	LOC102170144
3	66315406	66415406	0.370206	LOC102181554
3	66315406	66415406	0.370206	LOC102170144
3	66315414	66415414	0.367133	LOC102181554
3	66315414	66415414	0.367133	LOC102170144
3	66315426	66415426	0.397157	LOC102181554
3	66315426	66415426	0.397157	LOC102170144
3	66315445	66415445	0.388014	LOC102181554
3	66315445	66415445	0.388014	LOC102170144
3	66315500	66415500	0.356713	LOC102181554
3	66315500	66415500	0.356713	LOC102170144
3	66316080	66416080	0.416528	LOC102181554
3	66316080	66416080	0.416528	LOC102170144
3	66316110	66416110	0.363323	LOC102181554
3	66316110	66416110	0.363323	LOC102170144
3	66316130	66416130	0.364199	LOC102181554
3	66316130	66416130	0.364199	LOC102170144
3	66316164	66416164	0.36723	LOC102181554
3	66316164	66416164	0.36723	LOC102170144
3	66316165	66416165	0.469569	LOC102181554
3	66316165	66416165	0.469569	LOC102170144
3	66316212	66416212	0.436279	LOC102181554
3	66316212	66416212	0.436279	LOC102170144
3	66316257	66416257	0.436245	LOC102181554
3	66316257	66416257	0.436245	LOC102170144
3	66316264	66416264	0.441033	LOC102181554
3	66316264	66416264	0.441033	LOC102170144
3	66316288	66416288	0.498875	LOC102181554
3	66316288	66416288	0.498875	LOC102170144
3	66316289	66416289	0.498875	LOC102181554
3	66316289	66416289	0.498875	LOC102170144
3	66316319	66416319	0.477286	LOC102181554
3	66316319	66416319	0.477286	LOC102170144

3	66316335	66416335	0.495743	LOC102181554
3	66316335	66416335	0.495743	LOC102170144
3	66316416	66416416	0.523725	LOC102181554
3	66316416	66416416	0.523725	LOC102170144
3	66316432	66416432	0.532881	LOC102181554
3	66316432	66416432	0.532881	LOC102170144
3	66316552	66416552	0.555718	LOC102181554
3	66316552	66416552	0.555718	LOC102170144
3	66316633	66416633	0.519683	LOC102181554
3	66316633	66416633	0.519683	LOC102170144
3	66316713	66416713	0.521388	LOC102181554
3	66316713	66416713	0.521388	LOC102170144
3	66316774	66416774	0.514839	LOC102181554
3	66316774	66416774	0.514839	LOC102170144
3	66316836	66416836	0.5123	LOC102181554
3	66316836	66416836	0.5123	LOC102170144
3	66316957	66416957	0.543496	LOC102181554
3	66316957	66416957	0.543496	LOC102170144
3	66316960	66416960	0.571071	LOC102181554
3	66316960	66416960	0.571071	LOC102170144
3	66316964	66416964	0.594438	LOC102181554
3	66316964	66416964	0.594438	LOC102170144
3	66316986	66416986	0.572292	LOC102181554
3	66316986	66416986	0.572292	LOC102170144
3	66316997	66416997	0.56537	LOC102181554
3	66316997	66416997	0.56537	LOC102170144
3	66317010	66417010	0.564189	LOC102181554
3	66317010	66417010	0.564189	LOC102170144
3	66317028	66417028	0.560763	LOC102181554
3	66317028	66417028	0.560763	LOC102170144
3	66317038	66417038	0.561223	LOC102181554
3	66317038	66417038	0.561223	LOC102170144
3	66317043	66417043	0.561369	LOC102181554
3	66317043	66417043	0.561369	LOC102170144
3	66317072	66417072	0.555031	LOC102181554
3	66317072	66417072	0.555031	LOC102170144
3	66317080	66417080	0.555833	LOC102181554
3	66317080	66417080	0.555833	LOC102170144
3	66317105	66417105	0.554126	LOC102181554
3	66317105	66417105	0.554126	LOC102170144
3	66317148	66417148	0.578836	LOC102181554
3	66317148	66417148	0.578836	LOC102170144
3	66317151	66417151	0.553705	LOC102181554
3	66317151	66417151	0.553705	LOC102170144
3	66317242	66417242	0.698352	LOC102181554
3	66317242	66417242	0.698352	LOC102170144
3	66317282	66417282	0.62941	LOC102181554
3	66317282	66417282	0.62941	LOC102170144
3	66317285	66417285	0.68959	LOC102181554
3	66317285	66417285	0.68959	LOC102170144
3	66317312	66417312	0.674099	LOC102181554
3	66317312	66417312	0.674099	LOC102170144
3	66317345	66417345	0.658645	LOC102181554
3	66317345	66417345	0.658645	LOC102170144
3	66317382	66417382	0.656146	LOC102181554
3	66317382	66417382	0.656146	LOC102170144
3	66317444	66417444	0.693788	LOC102181554
3	66317444	66417444	0.693788	LOC102170144

3	66317486	66417486	0.678682	LOC102181554
3	66317486	66417486	0.678682	LOC102170144
3	66317529	66417529	0.651929	LOC102181554
3	66317529	66417529	0.651929	LOC102170144
3	66317543	66417543	0.645631	LOC102181554
3	66317543	66417543	0.645631	LOC102170144
3	66317553	66417553	0.65006	LOC102181554
3	66317553	66417553	0.65006	LOC102170144
3	66317569	66417569	0.575119	LOC102181554
3	66317569	66417569	0.575119	LOC102170144
3	66317643	66417643	0.588776	LOC102181554
3	66317643	66417643	0.588776	LOC102170144
3	66317658	66417658	0.608631	LOC102181554
3	66317658	66417658	0.608631	LOC102170144
3	66317669	66417669	0.601758	LOC102181554
3	66317669	66417669	0.601758	LOC102170144
3	66317715	66417715	0.542645	LOC102181554
3	66317715	66417715	0.542645	LOC102170144
3	66317720	66417720	0.532984	LOC102181554
3	66317720	66417720	0.532984	LOC102170144
3	66317726	66417726	0.647142	LOC102181554
3	66317726	66417726	0.647142	LOC102170144
3	66317742	66417742	0.584121	LOC102181554
3	66317742	66417742	0.584121	LOC102170144
3	66317777	66417777	0.56967	LOC102181554
3	66317777	66417777	0.56967	LOC102170144
3	66317840	66417840	0.484853	LOC102181554
3	66317840	66417840	0.484853	LOC102170144
3	66317884	66417884	0.45819	LOC102181554
3	66317884	66417884	0.45819	LOC102170144
3	66317919	66417919	0.482745	LOC102181554
3	66317919	66417919	0.482745	LOC102170144
3	66318026	66418026	0.504105	LOC102181554
3	66318026	66418026	0.504105	LOC102170144
3	66318054	66418054	0.572037	LOC102181554
3	66318054	66418054	0.572037	LOC102170144
3	66355426	66455426	0.454445	LOC102181554
3	66355426	66455426	0.454445	LOC102174264
3	66355426	66455426	0.454445	LOC102170144
3	66357282	66457282	0.343079	LOC102181554
3	66357282	66457282	0.343079	LOC102174264
3	66357282	66457282	0.343079	LOC102170144
3	68894025	68994025	0.354988	TGFBR3
3	68894691	68994691	0.365108	TGFBR3
3	68895167	68995167	0.351998	TGFBR3
3	68895840	68995840	0.3675	TGFBR3
3	68896344	68996344	0.35623	TGFBR3
3	68897258	68997258	0.375588	TGFBR3
3	68897515	68997515	0.385928	TGFBR3
3	68897823	68997823	0.382204	TGFBR3
3	68897844	68997844	0.369323	TGFBR3
3	68898545	68998545	0.37492	TGFBR3
3	68898627	68998627	0.375326	TGFBR3
3	68899405	68999405	0.342095	TGFBR3
3	68902228	69002228	0.344817	TGFBR3
3	68902315	69002315	0.343315	TGFBR3
3	68903003	69003003	0.356543	TGFBR3
3	68904861	69004861	0.344392	TGFBR3

3	68904912	69004912	0.344392	TGFBR3
3	68905219	69005219	0.344392	TGFBR3
3	68906131	69006131	0.374449	TGFBR3
3	68906136	69006136	0.369744	TGFBR3
3	68906255	69006255	0.371636	TGFBR3
3	68907063	69007063	0.365647	TGFBR3
3	68907834	69007834	0.380801	TGFBR3
3	68907960	69007960	0.349449	TGFBR3
3	68908020	69008020	0.350963	TGFBR3
3	68908158	69008158	0.346449	TGFBR3
3	68911740	69011740	0.345874	TGFBR3
3	68911811	69011811	0.347275	TGFBR3
3	68912667	69012667	0.375811	TGFBR3
3	68913410	69013410	0.36436	TGFBR3
3	68913509	69013509	0.370238	TGFBR3
3	68913675	69013675	0.350812	TGFBR3
3	68914393	69014393	0.349526	TGFBR3
3	68914507	69014507	0.354665	TGFBR3
3	68914920	69014920	0.352308	TGFBR3
3	68914995	69014995	0.35104	TGFBR3
3	68915084	69015084	0.35104	TGFBR3
3	68917297	69017297	0.386923	TGFBR3
3	68917996	69017996	0.398037	TGFBR3
3	68918157	69018157	0.409859	TGFBR3
3	68918429	69018429	0.407191	TGFBR3
3	68918439	69018439	0.407257	TGFBR3
3	68918592	69018592	0.410536	TGFBR3
3	68918990	69018990	0.402581	TGFBR3
3	68919212	69019212	0.378518	TGFBR3
3	68920035	69020035	0.378438	TGFBR3
3	68920097	69020097	0.386067	TGFBR3
3	68920446	69020446	0.377831	TGFBR3
3	68920735	69020735	0.373734	TGFBR3
3	68921000	69021000	0.370601	TGFBR3
3	68921152	69021152	0.376489	TGFBR3
3	68921527	69021527	0.375437	TGFBR3
3	68922069	69022069	0.375437	TGFBR3
3	68923486	69023486	0.37803	TGFBR3
3	68923651	69023651	0.350996	TGFBR3
3	68923934	69023934	0.379814	TGFBR3
3	68924002	69024002	0.364529	TGFBR3
3	68924566	69024566	0.383899	TGFBR3
3	68924771	69024771	0.368975	TGFBR3
3	68924868	69024868	0.375789	TGFBR3
3	68924983	69024983	0.364057	TGFBR3
3	68925106	69025106	0.364057	TGFBR3
3	68925949	69025949	0.367931	TGFBR3
3	68926403	69026403	0.382405	TGFBR3
3	68926837	69026837	0.38095	TGFBR3
3	68927015	69027015	0.377597	TGFBR3
3	68927231	69027231	0.375445	TGFBR3
3	68927615	69027615	0.381629	TGFBR3
3	68927684	69027684	0.376364	TGFBR3
3	68927841	69027841	0.371361	TGFBR3
3	68928118	69028118	0.376767	TGFBR3
3	68928379	69028379	0.372064	TGFBR3
3	68928403	69028403	0.367175	TGFBR3
3	68930688	69030688	0.355531	TGFBR3

3	68931467	69031467	0.346743	TGFBR3
3	68931901	69031901	0.365591	TGFBR3
3	68932209	69032209	0.369465	TGFBR3
3	68932466	69032466	0.361494	TGFBR3
3	68932865	69032865	0.361494	TGFBR3
3	68933221	69033221	0.379415	TGFBR3
3	68938820	69038820	0.352895	TGFBR3
3	68939233	69039233	0.345712	TGFBR3
3	68939471	69039471	0.356264	TGFBR3
3	68939945	69039945	0.399955	TGFBR3
3	68939979	69039979	0.386248	TGFBR3
3	68940032	69040032	0.393011	TGFBR3
3	68941243	69041243	0.399652	TGFBR3
3	68941672	69041672	0.399652	TGFBR3
3	68941818	69041818	0.387878	TGFBR3
3	68941864	69041864	0.388305	TGFBR3
3	68942295	69042295	0.387537	TGFBR3
3	68942580	69042580	0.387703	TGFBR3
3	68942688	69042688	0.381331	TGFBR3
3	68942914	69042914	0.37367	TGFBR3
3	68942941	69042941	0.390731	TGFBR3
3	68942984	69042984	0.380735	TGFBR3
3	68943006	69043006	0.380735	TGFBR3
3	68943110	69043110	0.39155	TGFBR3
3	68943490	69043490	0.382969	TGFBR3
3	68943920	69043920	0.384156	TGFBR3
3	68943969	69043969	0.361477	TGFBR3
3	68944436	69044436	0.362582	TGFBR3
3	68944509	69044509	0.371569	TGFBR3
3	68944690	69044690	0.382009	TGFBR3
3	68944699	69044699	0.383368	TGFBR3
3	68944782	69044782	0.394442	TGFBR3
3	68944979	69044979	0.4157	TGFBR3
3	68945037	69045037	0.409635	TGFBR3
3	68945042	69045042	0.405107	TGFBR3
3	68945176	69045176	0.407897	TGFBR3
3	68945276	69045276	0.40248	TGFBR3
3	68945515	69045515	0.388428	TGFBR3
3	68945538	69045538	0.389963	TGFBR3
3	68945787	69045787	0.391527	TGFBR3
3	68946931	69046931	0.412767	TGFBR3
3	68946987	69046987	0.417346	TGFBR3
3	68995940	69095940	0.349941	TGFBR3
3	70848070	70948070	0.368294	TRNAR-UCU-2
3	70848070	70948070	0.368294	DNTTIP2
3	70848070	70948070	0.368294	GCLM
3	70848070	70948070	0.368294	BCAR3
3	70848070	70948070	0.368294	LOC108635648
3	70848104	70948104	0.361653	TRNAR-UCU-2
3	70848104	70948104	0.361653	DNTTIP2
3	70848104	70948104	0.361653	GCLM
3	70848104	70948104	0.361653	BCAR3
3	70848104	70948104	0.361653	LOC108635648
3	70871028	70971028	0.342535	TRNAR-UCU-2
3	70871028	70971028	0.342535	TRNAY-AUA-2
3	70871028	70971028	0.342535	DNTTIP2
3	70871028	70971028	0.342535	GCLM
3	70871028	70971028	0.342535	LOC108635648

3	70871273	70971273	0.344007	TRNAR-UCU-2
3	70871273	70971273	0.344007	TRNAY-AUA-2
3	70871273	70971273	0.344007	DNTTIP2
3	70871273	70971273	0.344007	GCLM
3	70871273	70971273	0.344007	LOC108635648
3	70871355	70971355	0.342556	TRNAR-UCU-2
3	70871355	70971355	0.342556	TRNAY-AUA-2
3	70871355	70971355	0.342556	DNTTIP2
3	70871355	70971355	0.342556	GCLM
3	70871355	70971355	0.342556	LOC108635648
3	70871548	70971548	0.346382	TRNAR-UCU-2
3	70871548	70971548	0.346382	TRNAY-AUA-2
3	70871548	70971548	0.346382	DNTTIP2
3	70871548	70971548	0.346382	GCLM
3	70871548	70971548	0.346382	LOC108635648
3	70872423	70972423	0.362814	TRNAR-UCU-2
3	70872423	70972423	0.362814	TRNAY-AUA-2
3	70872423	70972423	0.362814	DNTTIP2
3	70872423	70972423	0.362814	GCLM
3	70872423	70972423	0.362814	LOC108635648
3	70872772	70972772	0.343183	TRNAR-UCU-2
3	70872772	70972772	0.343183	TRNAY-AUA-2
3	70872772	70972772	0.343183	DNTTIP2
3	70872772	70972772	0.343183	GCLM
3	70872772	70972772	0.343183	LOC108635648
3	70873039	70973039	0.34503	TRNAR-UCU-2
3	70873039	70973039	0.34503	TRNAY-AUA-2
3	70873039	70973039	0.34503	DNTTIP2
3	70873039	70973039	0.34503	GCLM
3	70873039	70973039	0.34503	LOC108635648
3	70873229	70973229	0.353164	TRNAR-UCU-2
3	70873229	70973229	0.353164	TRNAY-AUA-2
3	70873229	70973229	0.353164	DNTTIP2
3	70873229	70973229	0.353164	GCLM
3	70873229	70973229	0.353164	LOC108635648
3	70873577	70973577	0.351368	TRNAR-UCU-2
3	70873577	70973577	0.351368	TRNAY-AUA-2
3	70873577	70973577	0.351368	DNTTIP2
3	70873577	70973577	0.351368	GCLM
3	70873577	70973577	0.351368	LOC108635648
3	70873723	70973723	0.354671	TRNAR-UCU-2
3	70873723	70973723	0.354671	TRNAY-AUA-2
3	70873723	70973723	0.354671	DNTTIP2
3	70873723	70973723	0.354671	GCLM
3	70873723	70973723	0.354671	LOC108635648
3	70873937	70973937	0.354434	TRNAR-UCU-2
3	70873937	70973937	0.354434	TRNAY-AUA-2
3	70873937	70973937	0.354434	DNTTIP2
3	70873937	70973937	0.354434	GCLM
3	70873937	70973937	0.354434	LOC108635648
3	70874048	70974048	0.353528	TRNAR-UCU-2
3	70874048	70974048	0.353528	TRNAY-AUA-2
3	70874048	70974048	0.353528	DNTTIP2
3	70874048	70974048	0.353528	GCLM
3	70874048	70974048	0.353528	LOC108635648
3	70874421	70974421	0.35603	TRNAR-UCU-2
3	70874421	70974421	0.35603	TRNAY-AUA-2
3	70874421	70974421	0.35603	DNTTIP2

3	70874421	70974421	0.35603	GCLM
3	70874421	70974421	0.35603	LOC108635648
3	70874553	70974553	0.362824	TRNAR-UCU-2
3	70874553	70974553	0.362824	TRNAY-AUA-2
3	70874553	70974553	0.362824	DNTTIP2
3	70874553	70974553	0.362824	GCLM
3	70874553	70974553	0.362824	LOC108635648
3	70888533	70988533	0.365966	TRNAR-UCU-2
3	70888533	70988533	0.365966	TRNAY-AUA-2
3	70888533	70988533	0.365966	DNTTIP2
3	70888533	70988533	0.365966	GCLM
3	70888533	70988533	0.365966	LOC108635648
3	70888544	70988544	0.351331	TRNAR-UCU-2
3	70888544	70988544	0.351331	TRNAY-AUA-2
3	70888544	70988544	0.351331	DNTTIP2
3	70888544	70988544	0.351331	GCLM
3	70888544	70988544	0.351331	LOC108635648
3	70888681	70988681	0.349713	TRNAR-UCU-2
3	70888681	70988681	0.349713	TRNAY-AUA-2
3	70888681	70988681	0.349713	DNTTIP2
3	70888681	70988681	0.349713	GCLM
3	70888681	70988681	0.349713	LOC108635648
3	70890379	70990379	0.348532	TRNAR-UCU-2
3	70890379	70990379	0.348532	TRNAY-AUA-2
3	70890379	70990379	0.348532	DNTTIP2
3	70890379	70990379	0.348532	GCLM
3	70890379	70990379	0.348532	LOC108635648
3	70891127	70991127	0.346762	TRNAR-UCU-2
3	70891127	70991127	0.346762	TRNAY-AUA-2
3	70891127	70991127	0.346762	DNTTIP2
3	70891127	70991127	0.346762	GCLM
3	70891127	70991127	0.346762	LOC108635648
3	70891221	70991221	0.349733	TRNAR-UCU-2
3	70891221	70991221	0.349733	TRNAY-AUA-2
3	70891221	70991221	0.349733	DNTTIP2
3	70891221	70991221	0.349733	GCLM
3	70891221	70991221	0.349733	LOC108635648
3	70891344	70991344	0.36073	TRNAR-UCU-2
3	70891344	70991344	0.36073	TRNAY-AUA-2
3	70891344	70991344	0.36073	DNTTIP2
3	70891344	70991344	0.36073	GCLM
3	70891344	70991344	0.36073	LOC108635648
3	70891489	70991489	0.355694	TRNAR-UCU-2
3	70891489	70991489	0.355694	TRNAY-AUA-2
3	70891489	70991489	0.355694	DNTTIP2
3	70891489	70991489	0.355694	GCLM
3	70891489	70991489	0.355694	LOC108635648
3	70891495	70991495	0.369058	TRNAR-UCU-2
3	70891495	70991495	0.369058	TRNAY-AUA-2
3	70891495	70991495	0.369058	DNTTIP2
3	70891495	70991495	0.369058	GCLM
3	70891495	70991495	0.369058	LOC108635648
3	70891662	70991662	0.37886	TRNAR-UCU-2
3	70891662	70991662	0.37886	TRNAY-AUA-2
3	70891662	70991662	0.37886	DNTTIP2
3	70891662	70991662	0.37886	GCLM
3	70891662	70991662	0.37886	LOC108635648
3	70891832	70991832	0.345072	TRNAR-UCU-2

3	70891832	70991832	0.345072	TRNAY-AUA-2
3	70891832	70991832	0.345072	DNTTIP2
3	70891832	70991832	0.345072	GCLM
3	70891832	70991832	0.345072	LOC108635648
3	70892200	70992200	0.350056	TRNAR-UCU-2
3	70892200	70992200	0.350056	TRNAY-AUA-2
3	70892200	70992200	0.350056	DNTTIP2
3	70892200	70992200	0.350056	GCLM
3	70892200	70992200	0.350056	LOC108635648
3	70892486	70992486	0.343527	TRNAR-UCU-2
3	70892486	70992486	0.343527	TRNAY-AUA-2
3	70892486	70992486	0.343527	DNTTIP2
3	70892486	70992486	0.343527	GCLM
3	70892486	70992486	0.343527	LOC108635648
3	70892917	70992917	0.346241	TRNAR-UCU-2
3	70892917	70992917	0.346241	TRNAY-AUA-2
3	70892917	70992917	0.346241	DNTTIP2
3	70892917	70992917	0.346241	GCLM
3	70892917	70992917	0.346241	LOC108635648
3	70893097	70993097	0.342256	TRNAR-UCU-2
3	70893097	70993097	0.342256	TRNAY-AUA-2
3	70893097	70993097	0.342256	DNTTIP2
3	70893097	70993097	0.342256	GCLM
3	70893097	70993097	0.342256	LOC108635648
3	70893173	70993173	0.350381	TRNAR-UCU-2
3	70893173	70993173	0.350381	TRNAY-AUA-2
3	70893173	70993173	0.350381	DNTTIP2
3	70893173	70993173	0.350381	GCLM
3	70893173	70993173	0.350381	LOC108635648
3	70894305	70994305	0.344805	TRNAR-UCU-2
3	70894305	70994305	0.344805	TRNAY-AUA-2
3	70894305	70994305	0.344805	DNTTIP2
3	70894305	70994305	0.344805	GCLM
3	70894305	70994305	0.344805	LOC108635648
3	70898181	70998181	0.351822	TRNAR-UCU-2
3	70898181	70998181	0.351822	TRNAY-AUA-2
3	70898181	70998181	0.351822	DNTTIP2
3	70898181	70998181	0.351822	GCLM
3	70898181	70998181	0.351822	LOC108635648
3	70898425	70998425	0.343048	TRNAR-UCU-2
3	70898425	70998425	0.343048	TRNAY-AUA-2
3	70898425	70998425	0.343048	DNTTIP2
3	70898425	70998425	0.343048	GCLM
3	70898425	70998425	0.343048	LOC108635648
3	70898474	70998474	0.342602	TRNAR-UCU-2
3	70898474	70998474	0.342602	TRNAY-AUA-2
3	70898474	70998474	0.342602	DNTTIP2
3	70898474	70998474	0.342602	GCLM
3	70898474	70998474	0.342602	LOC108635648
3	70899803	70999803	0.351391	TRNAR-UCU-2
3	70899803	70999803	0.351391	TRNAY-AUA-2
3	70899803	70999803	0.351391	DNTTIP2
3	70899803	70999803	0.351391	GCLM
3	70899803	70999803	0.351391	LOC108635648
3	70900324	71000324	0.375575	TRNAR-UCU-2
3	70900324	71000324	0.375575	TRNAY-AUA-2
3	70900324	71000324	0.375575	DNTTIP2
3	70900324	71000324	0.375575	GCLM

3	70900324	71000324	0.375575	LOC108635648
3	70900414	71000414	0.368815	TRNAR-UCU-2
3	70900414	71000414	0.368815	TRNAY-AUA-2
3	70900414	71000414	0.368815	DNTTIP2
3	70900414	71000414	0.368815	GCLM
3	70900414	71000414	0.368815	LOC108635648
3	70900486	71000486	0.384292	TRNAR-UCU-2
3	70900486	71000486	0.384292	TRNAY-AUA-2
3	70900486	71000486	0.384292	DNTTIP2
3	70900486	71000486	0.384292	GCLM
3	70900486	71000486	0.384292	LOC108635648
3	70900721	71000721	0.37724	TRNAR-UCU-2
3	70900721	71000721	0.37724	TRNAY-AUA-2
3	70900721	71000721	0.37724	DNTTIP2
3	70900721	71000721	0.37724	GCLM
3	70900721	71000721	0.37724	LOC108635648
3	70900971	71000971	0.38929	TRNAR-UCU-2
3	70900971	71000971	0.38929	TRNAY-AUA-2
3	70900971	71000971	0.38929	DNTTIP2
3	70900971	71000971	0.38929	GCLM
3	70900971	71000971	0.38929	LOC108635648
3	70901374	71001374	0.356529	TRNAR-UCU-2
3	70901374	71001374	0.356529	TRNAY-AUA-2
3	70901374	71001374	0.356529	DNTTIP2
3	70901374	71001374	0.356529	GCLM
3	70901374	71001374	0.356529	LOC108635648
3	70901687	71001687	0.365459	TRNAR-UCU-2
3	70901687	71001687	0.365459	TRNAY-AUA-2
3	70901687	71001687	0.365459	DNTTIP2
3	70901687	71001687	0.365459	GCLM
3	70901687	71001687	0.365459	LOC108635648
3	70901867	71001867	0.353783	TRNAR-UCU-2
3	70901867	71001867	0.353783	TRNAY-AUA-2
3	70901867	71001867	0.353783	DNTTIP2
3	70901867	71001867	0.353783	GCLM
3	70901867	71001867	0.353783	LOC108635648
3	70902550	71002550	0.352985	TRNAR-UCU-2
3	70902550	71002550	0.352985	TRNAY-AUA-2
3	70902550	71002550	0.352985	DNTTIP2
3	70902550	71002550	0.352985	GCLM
3	70902550	71002550	0.352985	LOC108635648
3	70902682	71002682	0.355645	TRNAR-UCU-2
3	70902682	71002682	0.355645	TRNAY-AUA-2
3	70902682	71002682	0.355645	DNTTIP2
3	70902682	71002682	0.355645	GCLM
3	70902682	71002682	0.355645	LOC108635648
3	70906624	71006624	0.354279	TRNAR-UCU-2
3	70906624	71006624	0.354279	TRNAY-AUA-2
3	70906624	71006624	0.354279	DNTTIP2
3	70906624	71006624	0.354279	GCLM
3	70906624	71006624	0.354279	LOC108635648
3	70909299	71009299	0.360173	TRNAR-UCU-2
3	70909299	71009299	0.360173	TRNAY-AUA-2
3	70909299	71009299	0.360173	DNTTIP2
3	70909299	71009299	0.360173	GCLM
3	70909299	71009299	0.360173	LOC108635648
3	70913239	71013239	0.354675	TRNAY-AUA-2
3	70913239	71013239	0.354675	DNTTIP2

3	70913239	71013239	0.354675	GCLM
3	70913239	71013239	0.354675	ABCA4
3	70918091	71018091	0.343986	TRNAY-AUA-2
3	70918091	71018091	0.343986	DNTTIP2
3	70918091	71018091	0.343986	GCLM
3	70918091	71018091	0.343986	ABCA4
3	70920515	71020515	0.360205	TRNAY-AUA-2
3	70920515	71020515	0.360205	DNTTIP2
3	70920515	71020515	0.360205	GCLM
3	70920515	71020515	0.360205	ABCA4
3	70921231	71021231	0.355989	TRNAY-AUA-2
3	70921231	71021231	0.355989	DNTTIP2
3	70921231	71021231	0.355989	GCLM
3	70921231	71021231	0.355989	ABCA4
3	70921455	71021455	0.361937	TRNAY-AUA-2
3	70921455	71021455	0.361937	DNTTIP2
3	70921455	71021455	0.361937	GCLM
3	70921455	71021455	0.361937	ABCA4
3	70923170	71023170	0.361328	TRNAY-AUA-2
3	70923170	71023170	0.361328	DNTTIP2
3	70923170	71023170	0.361328	GCLM
3	70923170	71023170	0.361328	ABCA4
3	70923215	71023215	0.365446	TRNAY-AUA-2
3	70923215	71023215	0.365446	DNTTIP2
3	70923215	71023215	0.365446	GCLM
3	70923215	71023215	0.365446	ABCA4
3	70924589	71024589	0.3721	TRNAY-AUA-2
3	70924589	71024589	0.3721	DNTTIP2
3	70924589	71024589	0.3721	GCLM
3	70924589	71024589	0.3721	ABCA4
3	70924613	71024613	0.366597	TRNAY-AUA-2
3	70924613	71024613	0.366597	DNTTIP2
3	70924613	71024613	0.366597	GCLM
3	70924613	71024613	0.366597	ABCA4
3	70925150	71025150	0.365365	TRNAY-AUA-2
3	70925150	71025150	0.365365	DNTTIP2
3	70925150	71025150	0.365365	GCLM
3	70925150	71025150	0.365365	ABCA4
3	70925220	71025220	0.374732	TRNAY-AUA-2
3	70925220	71025220	0.374732	DNTTIP2
3	70925220	71025220	0.374732	GCLM
3	70925220	71025220	0.374732	ABCA4
3	70925699	71025699	0.35286	TRNAY-AUA-2
3	70925699	71025699	0.35286	DNTTIP2
3	70925699	71025699	0.35286	GCLM
3	70925699	71025699	0.35286	ABCA4
3	70925802	71025802	0.354258	TRNAY-AUA-2
3	70925802	71025802	0.354258	DNTTIP2
3	70925802	71025802	0.354258	GCLM
3	70925802	71025802	0.354258	ABCA4
3	70926040	71026040	0.3624	TRNAY-AUA-2
3	70926040	71026040	0.3624	DNTTIP2
3	70926040	71026040	0.3624	GCLM
3	70926040	71026040	0.3624	ABCA4
3	70926945	71026945	0.349723	TRNAY-AUA-2
3	70926945	71026945	0.349723	DNTTIP2
3	70926945	71026945	0.349723	GCLM
3	70926945	71026945	0.349723	ABCA4

3	70926997	71026997	0.350642	TRNAY-AUA-2
3	70926997	71026997	0.350642	DNTTIP2
3	70926997	71026997	0.350642	GCLM
3	70926997	71026997	0.350642	ABCA4
3	70927035	71027035	0.350856	TRNAY-AUA-2
3	70927035	71027035	0.350856	DNTTIP2
3	70927035	71027035	0.350856	GCLM
3	70927035	71027035	0.350856	ABCA4
3	70927385	71027385	0.354152	TRNAY-AUA-2
3	70927385	71027385	0.354152	DNTTIP2
3	70927385	71027385	0.354152	GCLM
3	70927385	71027385	0.354152	ABCA4
3	70927762	71027762	0.352812	TRNAY-AUA-2
3	70927762	71027762	0.352812	DNTTIP2
3	70927762	71027762	0.352812	GCLM
3	70927762	71027762	0.352812	ABCA4
3	70927987	71027987	0.351478	TRNAY-AUA-2
3	70927987	71027987	0.351478	DNTTIP2
3	70927987	71027987	0.351478	GCLM
3	70927987	71027987	0.351478	ABCA4
3	70928065	71028065	0.350188	TRNAY-AUA-2
3	70928065	71028065	0.350188	DNTTIP2
3	70928065	71028065	0.350188	GCLM
3	70928065	71028065	0.350188	ABCA4
3	70928178	71028178	0.357818	TRNAY-AUA-2
3	70928178	71028178	0.357818	DNTTIP2
3	70928178	71028178	0.357818	GCLM
3	70928178	71028178	0.357818	ABCA4
3	70930138	71030138	0.349842	TRNAY-AUA-2
3	70930138	71030138	0.349842	DNTTIP2
3	70930138	71030138	0.349842	GCLM
3	70930138	71030138	0.349842	ABCA4
3	70930483	71030483	0.343974	TRNAY-AUA-2
3	70930483	71030483	0.343974	DNTTIP2
3	70930483	71030483	0.343974	GCLM
3	70930483	71030483	0.343974	ABCA4
3	70930484	71030484	0.343974	TRNAY-AUA-2
3	70930484	71030484	0.343974	DNTTIP2
3	70930484	71030484	0.343974	GCLM
3	70930484	71030484	0.343974	ABCA4
3	70931070	71031070	0.345213	TRNAY-AUA-2
3	70931070	71031070	0.345213	DNTTIP2
3	70931070	71031070	0.345213	GCLM
3	70931070	71031070	0.345213	ABCA4
3	70931090	71031090	0.347304	TRNAY-AUA-2
3	70931090	71031090	0.347304	DNTTIP2
3	70931090	71031090	0.347304	GCLM
3	70931090	71031090	0.347304	ABCA4
3	70938125	71038125	0.34618	TRNAY-AUA-2
3	70938125	71038125	0.34618	GCLM
3	70938125	71038125	0.34618	ABCA4
3	70938247	71038247	0.343574	TRNAY-AUA-2
3	70938247	71038247	0.343574	GCLM
3	70938247	71038247	0.343574	ABCA4
3	70938333	71038333	0.35758	TRNAY-AUA-2
3	70938333	71038333	0.35758	GCLM
3	70938333	71038333	0.35758	ABCA4
3	70938470	71038470	0.359153	TRNAY-AUA-2

3	70938470	71038470	0.359153	GCLM
3	70938470	71038470	0.359153	ABCA4
3	70938563	71038563	0.354489	TRNAY-AUA-2
3	70938563	71038563	0.354489	GCLM
3	70938563	71038563	0.354489	ABCA4
3	70938906	71038906	0.343067	TRNAY-AUA-2
3	70938906	71038906	0.343067	GCLM
3	70938906	71038906	0.343067	ABCA4
3	70946102	71046102	0.352043	TRNAY-AUA-2
3	70946102	71046102	0.352043	GCLM
3	70946102	71046102	0.352043	ABCA4
3	70946103	71046103	0.352043	TRNAY-AUA-2
3	70946103	71046103	0.352043	GCLM
3	70946103	71046103	0.352043	ABCA4
3	70946215	71046215	0.349612	TRNAY-AUA-2
3	70946215	71046215	0.349612	GCLM
3	70946215	71046215	0.349612	ABCA4
3	70946295	71046295	0.372716	TRNAY-AUA-2
3	70946295	71046295	0.372716	GCLM
3	70946295	71046295	0.372716	ABCA4
3	74054985	74154985	0.373574	DPYD
3	74055017	74155017	0.40249	DPYD
3	74740754	74840754	0.362341	DPYD
3	74740847	74840847	0.361846	DPYD
3	74740896	74840896	0.364068	DPYD
3	74740912	74840912	0.359906	DPYD
3	74741072	74841072	0.37259	DPYD
3	74741075	74841075	0.388997	DPYD
3	74741096	74841096	0.39439	DPYD
3	74741112	74841112	0.386866	DPYD
3	74741193	74841193	0.393277	DPYD
3	74741231	74841231	0.392219	DPYD
3	74741242	74841242	0.382097	DPYD
3	74741244	74841244	0.374873	DPYD
3	74741294	74841294	0.374726	DPYD
3	74741383	74841383	0.374626	DPYD
3	74741413	74841413	0.540783	DPYD
3	74741562	74841562	0.546618	DPYD
3	74741577	74841577	0.555871	DPYD
3	74741689	74841689	0.537397	DPYD
3	74741706	74841706	0.546082	DPYD
3	74741745	74841745	0.49456	DPYD
3	74741886	74841886	0.502579	DPYD
3	74741922	74841922	0.502961	DPYD
3	74741951	74841951	0.506732	DPYD
3	74742103	74842103	0.482509	DPYD
3	74742148	74842148	0.458811	DPYD
3	74742170	74842170	0.454799	DPYD
3	74742323	74842323	0.437116	DPYD
3	74742325	74842325	0.437117	DPYD
3	74742377	74842377	0.443674	DPYD
3	74742469	74842469	0.439027	DPYD
3	74742478	74842478	0.439027	DPYD
3	74742695	74842695	0.439027	DPYD
3	74742777	74842777	0.439027	DPYD
3	74742829	74842829	0.438042	DPYD
3	74742924	74842924	0.442251	DPYD
3	74742953	74842953	0.442251	DPYD

3	74743007	74843007	0.442251	DPYD
3	74743012	74843012	0.442062	DPYD
3	74743030	74843030	0.442058	DPYD
3	74743089	74843089	0.442058	DPYD
3	74743120	74843120	0.448787	DPYD
3	74743159	74843159	0.449039	DPYD
3	74743162	74843162	0.473597	DPYD
3	74743246	74843246	0.45635	DPYD
3	74743298	74843298	0.459744	DPYD
3	74743303	74843303	0.459744	DPYD
3	74743341	74843341	0.459744	DPYD
3	74743373	74843373	0.466927	DPYD
3	74743393	74843393	0.468941	DPYD
3	74743403	74843403	0.463968	DPYD
3	74743721	74843721	0.462196	DPYD
3	74743781	74843781	0.467957	DPYD
3	74743864	74843864	0.472083	DPYD
3	74743947	74843947	0.47255	DPYD
3	74743957	74843957	0.472345	DPYD
3	74744098	74844098	0.440548	DPYD
3	74744114	74844114	0.439238	DPYD
3	74744115	74844115	0.461091	DPYD
3	74744199	74844199	0.475359	DPYD
3	74744227	74844227	0.456372	DPYD
3	74744301	74844301	0.459469	DPYD
3	74744323	74844323	0.459354	DPYD
3	74744345	74844345	0.460256	DPYD
3	74744414	74844414	0.461316	DPYD
3	74744472	74844472	0.432151	DPYD
3	74744698	74844698	0.406382	DPYD
3	74744731	74844731	0.414777	DPYD
3	74744757	74844757	0.414335	DPYD
3	74744851	74844851	0.414276	DPYD
3	74745094	74845094	0.415755	DPYD
3	74745105	74845105	0.387033	DPYD
3	74745337	74845337	0.390967	DPYD
3	74745719	74845719	0.39013	DPYD
3	74745757	74845757	0.408872	DPYD
3	74745796	74845796	0.409068	DPYD
3	74745878	74845878	0.406919	DPYD
3	74745963	74845963	0.405864	DPYD
3	74745968	74845968	0.391299	DPYD
3	74746001	74846001	0.38425	DPYD
3	74746535	74846535	0.386119	DPYD
3	74746579	74846579	0.386119	DPYD
3	74746618	74846618	0.386243	DPYD
3	74746651	74846651	0.386243	DPYD
3	74746657	74846657	0.386243	DPYD
3	74746737	74846737	0.386213	DPYD
3	74746768	74846768	0.41017	DPYD
3	74746907	74846907	0.362596	DPYD
3	74746952	74846952	0.344826	DPYD
3	74746953	74846953	0.344826	DPYD
3	74746960	74846960	0.346061	DPYD
3	74746985	74846985	0.343158	DPYD
3	74746987	74846987	0.343158	DPYD
3	74746991	74846991	0.343158	DPYD
3	74747573	74847573	0.344546	DPYD

3	74750099	74850099	0.367051	DPYD
3	74751119	74851119	0.350761	DPYD
3	74751559	74851559	0.341719	DPYD
3	74751597	74851597	0.341748	DPYD
3	74752117	74852117	0.341692	DPYD
3	74752161	74852161	0.349117	DPYD
3	74752314	74852314	0.343627	DPYD
3	74752396	74852396	0.34377	DPYD
3	74752400	74852400	0.351711	DPYD
3	74752414	74852414	0.351327	DPYD
3	74752461	74852461	0.34457	DPYD
3	74752462	74852462	0.360659	DPYD
3	74752522	74852522	0.352682	DPYD
3	74752579	74852579	0.37382	DPYD
3	74752671	74852671	0.352093	DPYD
3	74752675	74852675	0.352597	DPYD
3	74752791	74852791	0.356063	DPYD
3	74752813	74852813	0.355244	DPYD
3	74752857	74852857	0.348315	DPYD
3	74752932	74852932	0.345697	DPYD
3	74753481	74853481	0.344896	DPYD
3	74754085	74854085	0.361727	DPYD
3	74755395	74855395	0.360072	DPYD
3	74755451	74855451	0.360349	DPYD
3	74755565	74855565	0.358328	DPYD
3	74755999	74855999	0.350671	DPYD
3	74757255	74857255	0.35684	DPYD
3	74757360	74857360	0.369283	DPYD
3	74757385	74857385	0.369251	DPYD
3	74757423	74857423	0.373478	DPYD
3	74757432	74857432	0.38319	DPYD
3	74757488	74857488	0.376373	DPYD
3	74757498	74857498	0.376373	DPYD
3	74757500	74857500	0.376586	DPYD
3	74757555	74857555	0.372796	DPYD
3	74757593	74857593	0.372796	DPYD
3	74757640	74857640	0.372796	DPYD
3	74757729	74857729	0.372796	DPYD
3	74757742	74857742	0.372796	DPYD
3	74757786	74857786	0.377248	DPYD
3	74757854	74857854	0.378394	DPYD
3	74757863	74857863	0.385355	DPYD
3	74758126	74858126	0.380142	DPYD
3	74758264	74858264	0.380879	DPYD
3	74758381	74858381	0.387396	DPYD
3	74758395	74858395	0.387396	DPYD
3	74758442	74858442	0.389876	DPYD
3	74758477	74858477	0.390258	DPYD
3	74758492	74858492	0.389001	DPYD
3	74758623	74858623	0.38394	DPYD
3	74758624	74858624	0.38394	DPYD
3	74758679	74858679	0.409518	DPYD
3	74758682	74858682	0.405308	DPYD
3	74758717	74858717	0.405308	DPYD
3	74758732	74858732	0.41364	DPYD
3	74758733	74858733	0.410977	DPYD
3	74759082	74859082	0.408048	DPYD
3	74759146	74859146	0.411405	DPYD

3	74759163	74859163	0.411405	DPYD
3	74759260	74859260	0.398315	DPYD
3	74759261	74859261	0.398315	DPYD
3	74759314	74859314	0.427049	DPYD
3	74759330	74859330	0.424454	DPYD
3	74759396	74859396	0.432387	DPYD
3	74759465	74859465	0.432387	DPYD
3	74759490	74859490	0.43315	DPYD
3	74759500	74859500	0.425171	DPYD
3	74759527	74859527	0.424581	DPYD
3	74759657	74859657	0.424619	DPYD
3	74759662	74859662	0.428861	DPYD
3	74759696	74859696	0.428497	DPYD
3	74759769	74859769	0.429282	DPYD
3	74759786	74859786	0.429282	DPYD
3	74759817	74859817	0.424367	DPYD
3	74759902	74859902	0.420395	DPYD
3	74759905	74859905	0.420395	DPYD
3	74760109	74860109	0.435736	DPYD
3	74760114	74860114	0.438008	DPYD
3	74760129	74860129	0.41885	DPYD
3	74760130	74860130	0.378042	DPYD
3	74760341	74860341	0.377957	DPYD
3	74760346	74860346	0.367994	DPYD
3	74760355	74860355	0.362918	DPYD
3	74760373	74860373	0.343501	DPYD
3	74912682	75012682	0.358002	DPYD
3	75034312	75134312	0.346501	LOC108635577
3	75034312	75134312	0.346501	DPYD
3	75035296	75135296	0.369733	LOC108635577
3	75035296	75135296	0.369733	DPYD
3	75035510	75135510	0.345283	LOC108635577
3	75035510	75135510	0.345283	DPYD
3	76545702	76645702	0.378496	PLPPR4
3	77742595	77842595	0.354978	GPR88
3	85059006	85159006	0.389137	VAV3
3	85059033	85159033	0.387783	VAV3
3	85059060	85159060	0.390577	VAV3
3	85059083	85159083	0.373408	VAV3
3	85059186	85159186	0.360324	VAV3
3	85059204	85159204	0.35754	VAV3
3	85059370	85159370	0.384753	VAV3
3	85059458	85159458	0.381085	VAV3
3	85059467	85159467	0.394512	VAV3
3	85059516	85159516	0.354452	VAV3
3	85059545	85159545	0.371666	VAV3
3	85059574	85159574	0.36704	VAV3
3	85059889	85159889	0.344857	VAV3
3	85059944	85159944	0.344857	VAV3
3	85059968	85159968	0.344857	VAV3
3	85060337	85160337	0.346258	VAV3
3	85060346	85160346	0.363066	VAV3
3	85060358	85160358	0.367166	VAV3
3	85060392	85160392	0.357509	VAV3
3	85060460	85160460	0.355186	VAV3
3	85060745	85160745	0.344401	VAV3
3	85060911	85160911	0.34345	VAV3
3	85060918	85160918	0.342275	VAV3

3	91013659	91113659	0.344426	RSBN1
3	91013659	91113659	0.344426	PTPN22
3	91013659	91113659	0.344426	AP4B1
3	91013659	91113659	0.344426	BCL2L15
3	98386064	98486064	0.34194	BCL9
3	104435930	104535930	0.361676	PMVK
3	104435930	104535930	0.361676	PYGO2
3	104435930	104535930	0.361676	CKS1B
3	104435930	104535930	0.361676	LENEP
3	104435930	104535930	0.361676	PBXIP1
3	104435930	104535930	0.361676	SHC1
3	104435930	104535930	0.361676	FLAD1
3	104435935	104535935	0.410906	PMVK
3	104435935	104535935	0.410906	PYGO2
3	104435935	104535935	0.410906	CKS1B
3	104435935	104535935	0.410906	LENEP
3	104435935	104535935	0.410906	PBXIP1
3	104435935	104535935	0.410906	SHC1
3	104435935	104535935	0.410906	FLAD1
3	104436027	104536027	0.404595	PMVK
3	104436027	104536027	0.404595	PYGO2
3	104436027	104536027	0.404595	CKS1B
3	104436027	104536027	0.404595	LENEP
3	104436027	104536027	0.404595	PBXIP1
3	104436027	104536027	0.404595	SHC1
3	104436027	104536027	0.404595	FLAD1
3	104436100	104536100	0.386525	PMVK
3	104436100	104536100	0.386525	PYGO2
3	104436100	104536100	0.386525	CKS1B
3	104436100	104536100	0.386525	LENEP
3	104436100	104536100	0.386525	PBXIP1
3	104436100	104536100	0.386525	SHC1
3	104436100	104536100	0.386525	FLAD1
3	104436105	104536105	0.409549	PMVK
3	104436105	104536105	0.409549	PYGO2
3	104436105	104536105	0.409549	CKS1B
3	104436105	104536105	0.409549	LENEP
3	104436105	104536105	0.409549	PBXIP1
3	104436105	104536105	0.409549	SHC1
3	104436105	104536105	0.409549	FLAD1
3	104436121	104536121	0.39154	PMVK
3	104436121	104536121	0.39154	PYGO2
3	104436121	104536121	0.39154	CKS1B
3	104436121	104536121	0.39154	LENEP
3	104436121	104536121	0.39154	PBXIP1
3	104436121	104536121	0.39154	SHC1
3	104436121	104536121	0.39154	FLAD1
3	104436148	104536148	0.382893	PMVK
3	104436148	104536148	0.382893	PYGO2
3	104436148	104536148	0.382893	CKS1B
3	104436148	104536148	0.382893	LENEP
3	104436148	104536148	0.382893	PBXIP1
3	104436148	104536148	0.382893	SHC1
3	104436148	104536148	0.382893	FLAD1
3	104436149	104536149	0.375661	PMVK
3	104436149	104536149	0.375661	PYGO2
3	104436149	104536149	0.375661	CKS1B
3	104436149	104536149	0.375661	LENEP

3	104436149	104536149	0.375661	PBXIP1
3	104436149	104536149	0.375661	SHC1
3	104436149	104536149	0.375661	FLAD1
3	112800154	112900154	0.350061	C3H1orf226
3	113562968	113662968	0.357359	CCDC190
3	113563103	113663103	0.362134	CCDC190
3	113563114	113663114	0.354868	CCDC190
3	113563118	113663118	0.353892	CCDC190
3	113563138	113663138	0.356297	CCDC190
3	113563151	113663151	0.356379	CCDC190
3	113563183	113663183	0.356379	CCDC190
3	113563206	113663206	0.359509	CCDC190
3	113563207	113663207	0.35834	CCDC190
3	113563215	113663215	0.35834	CCDC190
3	113563244	113663244	0.366388	CCDC190
3	113563253	113663253	0.366388	CCDC190
3	113563261	113663261	0.378053	CCDC190
3	113563267	113663267	0.37846	CCDC190
3	113563272	113663272	0.37846	CCDC190
3	113563284	113663284	0.376405	CCDC190
3	113563287	113663287	0.379896	CCDC190
3	113563309	113663309	0.379896	CCDC190
3	113563311	113663311	0.379896	CCDC190
3	113563314	113663314	0.379896	CCDC190
3	113563344	113663344	0.379252	CCDC190
3	113563354	113663354	0.374176	CCDC190
3	113563398	113663398	0.378898	CCDC190
3	113563399	113663399	0.378898	CCDC190
3	113563419	113663419	0.364797	CCDC190
3	113563436	113663436	0.37003	CCDC190
3	113563440	113663440	0.356325	CCDC190
3	113563548	113663548	0.35836	CCDC190
3	113563607	113663607	0.3576	CCDC190
3	113563636	113663636	0.360387	CCDC190
3	113563648	113663648	0.355459	CCDC190
3	115870171	115970171	0.346717	LOC106503647
3	115870171	115970171	0.346717	PBX1
3	115870318	115970318	0.351988	LOC106503647
3	115870318	115970318	0.351988	PBX1
3	115870339	115970339	0.36709	LOC106503647
3	115870339	115970339	0.36709	PBX1
3	115870359	115970359	0.344588	LOC106503647
3	115870359	115970359	0.344588	PBX1
3	115870360	115970360	0.362833	LOC106503647
3	115870360	115970360	0.362833	PBX1
3	115870363	115970363	0.388507	LOC106503647
3	115870363	115970363	0.388507	PBX1
3	115870374	115970374	0.385817	LOC106503647
3	115870374	115970374	0.385817	PBX1
3	115870391	115970391	0.392728	LOC106503647
3	115870391	115970391	0.392728	PBX1
3	115870408	115970408	0.404156	LOC106503647
3	115870408	115970408	0.404156	PBX1
3	115870412	115970412	0.404875	LOC106503647
3	115870412	115970412	0.404875	PBX1
3	115870415	115970415	0.394019	LOC106503647
3	115870415	115970415	0.394019	PBX1
3	115870430	115970430	0.419811	LOC106503647

3	115870430	115970430	0.419811	PBX1
3	115870432	115970432	0.419811	LOC106503647
3	115870432	115970432	0.419811	PBX1
3	115870434	115970434	0.36293	LOC106503647
3	115870434	115970434	0.36293	PBX1
3	115878992	115978992	0.369063	LOC106503647
3	115878992	115978992	0.369063	PBX1
3	115879102	115979102	0.384672	LOC106503647
3	115879102	115979102	0.384672	PBX1
3	115879228	115979228	0.35366	LOC106503647
3	115879228	115979228	0.35366	PBX1
3	116564285	116664285	0.36445	RXRG
3	116564474	116664474	0.35151	RXRG
3	116564509	116664509	0.347396	RXRG
3	116564835	116664835	0.368267	RXRG
3	116564908	116664908	0.405126	RXRG
3	116564945	116664945	0.349363	RXRG
3	116565030	116665030	0.381634	RXRG
3	116613592	116713592	0.355561	RXRG
3	116613625	116713625	0.400975	RXRG
3	116613674	116713674	0.412295	RXRG
3	116613779	116713779	0.399915	RXRG
3	116613917	116713917	0.365337	RXRG
3	116613937	116713937	0.360678	RXRG
3	116613947	116713947	0.359664	RXRG
3	116616728	116716728	0.356184	RXRG
3	116627408	116727408	0.344971	RXRG
3	116627639	116727639	0.360652	RXRG
3	116627978	116727978	0.357071	RXRG
3	116627980	116727980	0.356003	RXRG
3	116627982	116727982	0.368148	RXRG
3	116628071	116728071	0.369607	RXRG
3	116628204	116728204	0.371448	RXRG
3	116628240	116728240	0.371448	RXRG
3	116628280	116728280	0.37213	RXRG
3	116628293	116728293	0.368493	RXRG
3	116628302	116728302	0.368493	RXRG
3	116628311	116728311	0.369473	RXRG
3	116628334	116728334	0.369473	RXRG
3	116628379	116728379	0.378996	RXRG
3	117572845	117672845	0.354333	LOC108635666
3	118339695	118439695	0.354665	GPA33
3	118339825	118439825	0.352861	GPA33
3	118339930	118439930	0.396811	GPA33
3	118340049	118440049	0.378439	GPA33
3	118340224	118440224	0.363989	GPA33
3	118340254	118440254	0.36251	GPA33
3	118340260	118440260	0.359386	GPA33
3	118340285	118440285	0.377077	GPA33
3	118340324	118440324	0.372792	GPA33
3	119669626	119769626	0.384189	SFT2D2
3	119669626	119769626	0.384189	TIPRL
3	119943560	120043560	0.359173	LOC102172500
4	80173	180173	0.35197	VIPR2
4	128768	228768	0.397217	VIPR2
4	662853	762853	0.348376	PTPRN2
4	662982	762982	0.353334	PTPRN2
4	662987	762987	0.352796	PTPRN2

4	662993	762993	0.35448	PTPRN2
4	663000	763000	0.35448	PTPRN2
4	663020	763020	0.343336	PTPRN2
4	663031	763031	0.374212	PTPRN2
4	663140	763140	0.37565	PTPRN2
4	663190	763190	0.368394	PTPRN2
4	663282	763282	0.3417	PTPRN2
4	663350	763350	0.3417	PTPRN2
4	663541	763541	0.365956	PTPRN2
4	663551	763551	0.354416	PTPRN2
4	664442	764442	0.354008	PTPRN2
4	664445	764445	0.354008	PTPRN2
4	664711	764711	0.342758	PTPRN2
4	664835	764835	0.361415	PTPRN2
4	664861	764861	0.36822	PTPRN2
4	666949	766949	0.346397	PTPRN2
4	667096	767096	0.391735	PTPRN2
4	668159	768159	0.344271	PTPRN2
4	668263	768263	0.343818	PTPRN2
4	668323	768323	0.345119	PTPRN2
4	668375	768375	0.349361	PTPRN2
4	1524325	1624325	0.350775	NOM1
4	1524325	1624325	0.350775	LMBR1
4	1524386	1624386	0.372129	NOM1
4	1524386	1624386	0.372129	LMBR1
4	1524452	1624452	0.420294	NOM1
4	1524452	1624452	0.420294	LMBR1
4	1524586	1624586	0.416327	NOM1
4	1524586	1624586	0.416327	LMBR1
4	1524722	1624722	0.433093	NOM1
4	1524722	1624722	0.433093	LMBR1
4	1524724	1624724	0.433093	NOM1
4	1524724	1624724	0.433093	LMBR1
4	1524747	1624747	0.435881	NOM1
4	1524747	1624747	0.435881	LMBR1
4	1524767	1624767	0.421423	NOM1
4	1524767	1624767	0.421423	LMBR1
4	1524919	1624919	0.387379	NOM1
4	1524919	1624919	0.387379	LMBR1
4	1525036	1625036	0.363481	NOM1
4	1525036	1625036	0.363481	LMBR1
4	1525065	1625065	0.367191	NOM1
4	1525065	1625065	0.367191	LMBR1
4	1651584	1751584	0.348209	RNF32
4	1651584	1751584	0.348209	LMBR1
4	1651586	1751586	0.366817	RNF32
4	1651586	1751586	0.366817	LMBR1
4	1651631	1751631	0.401576	RNF32
4	1651631	1751631	0.401576	LMBR1
4	1651641	1751641	0.397514	RNF32
4	1651641	1751641	0.397514	LMBR1
4	1651645	1751645	0.403028	RNF32
4	1651645	1751645	0.403028	LMBR1
4	1651657	1751657	0.385554	RNF32
4	1651657	1751657	0.385554	LMBR1
4	1651860	1751860	0.404369	RNF32
4	1651860	1751860	0.404369	LMBR1
4	1651961	1751961	0.400175	RNF32

4	1651961	1751961	0.400175	LMBR1
4	1651986	1751986	0.400175	RNF32
4	1651986	1751986	0.400175	LMBR1
4	1651995	1751995	0.404315	RNF32
4	1651995	1751995	0.404315	LMBR1
4	1652000	1752000	0.3878	RNF32
4	1652000	1752000	0.3878	LMBR1
4	1652085	1752085	0.387506	RNF32
4	1652085	1752085	0.387506	LMBR1
4	1652099	1752099	0.379891	RNF32
4	1652099	1752099	0.379891	LMBR1
4	1652140	1752140	0.378451	RNF32
4	1652140	1752140	0.378451	LMBR1
4	1652394	1752394	0.385045	RNF32
4	1652394	1752394	0.385045	LMBR1
4	1652424	1752424	0.391035	RNF32
4	1652424	1752424	0.391035	LMBR1
4	1652692	1752692	0.389794	RNF32
4	1652692	1752692	0.389794	LMBR1
4	1652722	1752722	0.363195	RNF32
4	1652722	1752722	0.363195	LMBR1
4	1652747	1752747	0.375121	RNF32
4	1652747	1752747	0.375121	LMBR1
4	1652749	1752749	0.37545	RNF32
4	1652749	1752749	0.37545	LMBR1
4	1652799	1752799	0.379058	RNF32
4	1652799	1752799	0.379058	LMBR1
4	1652818	1752818	0.363133	RNF32
4	1652818	1752818	0.363133	LMBR1
4	1652821	1752821	0.363133	RNF32
4	1652821	1752821	0.363133	LMBR1
4	1652978	1752978	0.34822	RNF32
4	1652978	1752978	0.34822	LMBR1
4	1653181	1753181	0.355535	RNF32
4	1653181	1753181	0.355535	LMBR1
4	1679605	1779605	0.355444	RNF32
4	1679605	1779605	0.355444	LMBR1
4	1679614	1779614	0.344475	RNF32
4	1679614	1779614	0.344475	LMBR1
4	1679653	1779653	0.342115	RNF32
4	1679653	1779653	0.342115	LMBR1
4	1679928	1779928	0.34642	RNF32
4	1679928	1779928	0.34642	LMBR1
4	1680381	1780381	0.368627	RNF32
4	1680381	1780381	0.368627	LMBR1
4	1680855	1780855	0.34584	RNF32
4	1680855	1780855	0.34584	LMBR1
4	2220162	2320162	0.398202	LOC106502075
4	2801134	2901134	0.37034	HTR5A
4	2801160	2901160	0.432214	HTR5A
4	2801184	2901184	0.442328	HTR5A
4	2801406	2901406	0.385187	HTR5A
4	3383053	3483053	0.346001	DPP6
4	3383167	3483167	0.342608	DPP6
4	3383207	3483207	0.369348	DPP6
4	4576976	4676976	0.373904	TRNAW-CCA-16
4	4618070	4718070	0.342814	ACTR3B
4	4618120	4718120	0.367098	ACTR3B

4	4618122	4718122	0.367098	ACTR3B
4	4618137	4718137	0.358767	ACTR3B
4	4831557	4931557	0.392546	XRCC2
4	4831740	4931740	0.376984	XRCC2
4	4831862	4931862	0.385844	XRCC2
4	4831899	4931899	0.38735	XRCC2
4	4832164	4932164	0.383171	XRCC2
4	4832183	4932183	0.378405	XRCC2
4	4832341	4932341	0.368908	XRCC2
4	4833283	4933283	0.461217	XRCC2
4	4833506	4933506	0.488003	XRCC2
4	4833541	4933541	0.509668	XRCC2
4	4833640	4933640	0.511476	XRCC2
4	4833912	4933912	0.513764	XRCC2
4	4833995	4933995	0.515516	XRCC2
4	4834141	4934141	0.510198	XRCC2
4	4834279	4934279	0.509953	XRCC2
4	4834547	4934547	0.492165	XRCC2
4	4834622	4934622	0.49316	XRCC2
4	4834780	4934780	0.497049	XRCC2
4	4835013	4935013	0.473018	XRCC2
4	4835335	4935335	0.458628	XRCC2
4	4835466	4935466	0.465504	XRCC2
4	4835521	4935521	0.436876	XRCC2
4	4835754	4935754	0.436876	XRCC2
4	4836315	4936315	0.474622	XRCC2
4	4836399	4936399	0.476872	XRCC2
4	4836515	4936515	0.477671	XRCC2
4	4836602	4936602	0.456401	XRCC2
4	4836605	4936605	0.456401	XRCC2
4	4836612	4936612	0.438789	XRCC2
4	4837880	4937880	0.438812	XRCC2
4	4837989	4937989	0.443719	XRCC2
4	4838027	4938027	0.455903	XRCC2
4	4838076	4938076	0.453474	XRCC2
4	4838187	4938187	0.411044	XRCC2
4	4838219	4938219	0.411044	XRCC2
4	4838371	4938371	0.41235	XRCC2
4	4838691	4938691	0.417661	XRCC2
4	4838751	4938751	0.435359	XRCC2
4	4838802	4938802	0.429244	XRCC2
4	4839343	4939343	0.42638	XRCC2
4	4922759	5022759	0.342253	CCT8L2
4	4922759	5022759	0.342253	KMT2C
4	4922954	5022954	0.345071	CCT8L2
4	4922954	5022954	0.345071	KMT2C
4	4923817	5023817	0.34329	CCT8L2
4	4923817	5023817	0.34329	KMT2C
4	4930980	5030980	0.343757	CCT8L2
4	4930980	5030980	0.343757	KMT2C
4	4931428	5031428	0.357898	CCT8L2
4	4931428	5031428	0.357898	KMT2C
4	4931447	5031447	0.383252	CCT8L2
4	4931447	5031447	0.383252	KMT2C
4	4931484	5031484	0.380326	CCT8L2
4	4931484	5031484	0.380326	KMT2C
4	4931770	5031770	0.376416	CCT8L2
4	4931770	5031770	0.376416	KMT2C

4	4932555	5032555	0.359555	CCT8L2
4	4932555	5032555	0.359555	KMT2C
4	4933679	5033679	0.354025	CCT8L2
4	4933679	5033679	0.354025	KMT2C
4	4933705	5033705	0.355681	CCT8L2
4	4933705	5033705	0.355681	KMT2C
4	4933725	5033725	0.3468	CCT8L2
4	4933725	5033725	0.3468	KMT2C
4	4936056	5036056	0.352075	CCT8L2
4	4936056	5036056	0.352075	KMT2C
4	4936381	5036381	0.349419	CCT8L2
4	4936381	5036381	0.349419	KMT2C
4	4936767	5036767	0.344976	CCT8L2
4	4936767	5036767	0.344976	KMT2C
4	4936775	5036775	0.356697	CCT8L2
4	4936775	5036775	0.356697	KMT2C
4	4937383	5037383	0.356167	CCT8L2
4	4937383	5037383	0.356167	KMT2C
4	4937390	5037390	0.347027	CCT8L2
4	4937390	5037390	0.347027	KMT2C
4	4937769	5037769	0.412844	CCT8L2
4	4937769	5037769	0.412844	KMT2C
4	4937794	5037794	0.39507	CCT8L2
4	4937794	5037794	0.39507	KMT2C
4	4937829	5037829	0.4148	CCT8L2
4	4937829	5037829	0.4148	KMT2C
4	4937846	5037846	0.438871	CCT8L2
4	4937846	5037846	0.438871	KMT2C
4	4937847	5037847	0.438871	CCT8L2
4	4937847	5037847	0.438871	KMT2C
4	4937848	5037848	0.438871	CCT8L2
4	4937848	5037848	0.438871	KMT2C
4	4937855	5037855	0.422743	CCT8L2
4	4937855	5037855	0.422743	KMT2C
4	4938287	5038287	0.376103	CCT8L2
4	4938287	5038287	0.376103	KMT2C
4	4938747	5038747	0.383218	CCT8L2
4	4938747	5038747	0.383218	KMT2C
4	4938927	5038927	0.383942	CCT8L2
4	4938927	5038927	0.383942	KMT2C
4	4938958	5038958	0.41292	CCT8L2
4	4938958	5038958	0.41292	KMT2C
4	4938971	5038971	0.43169	CCT8L2
4	4938971	5038971	0.43169	KMT2C
4	4939094	5039094	0.39441	CCT8L2
4	4939094	5039094	0.39441	KMT2C
4	4941268	5041268	0.367741	CCT8L2
4	4941268	5041268	0.367741	KMT2C
4	4941280	5041280	0.367741	CCT8L2
4	4941280	5041280	0.367741	KMT2C
4	5013053	5113053	0.364359	KMT2C
4	5092100	5192100	0.360088	KMT2C
4	5092103	5192103	0.369367	KMT2C
4	5092128	5192128	0.367101	KMT2C
4	5395735	5495735	0.348305	TRNAC-GCA-51
4	5395735	5495735	0.348305	GALNTL5
4	5395735	5495735	0.348305	PRKAG2
4	5395808	5495808	0.342553	TRNAC-GCA-51

4	5395808	5495808	0.342553	GALNTL5
4	5395808	5495808	0.342553	PRKAG2
4	5395848	5495848	0.34259	TRNAC-GCA-51
4	5395848	5495848	0.34259	GALNTL5
4	5395848	5495848	0.34259	PRKAG2
4	5395849	5495849	0.34259	TRNAC-GCA-51
4	5395849	5495849	0.34259	GALNTL5
4	5395849	5495849	0.34259	PRKAG2
4	5395850	5495850	0.34259	TRNAC-GCA-51
4	5395850	5495850	0.34259	GALNTL5
4	5395850	5495850	0.34259	PRKAG2
4	5398886	5498886	0.357561	TRNAC-GCA-51
4	5398886	5498886	0.357561	PRKAG2
4	5398917	5498917	0.371228	TRNAC-GCA-51
4	5398917	5498917	0.371228	PRKAG2
4	5460078	5560078	0.386824	TRNAC-GCA-51
4	5460078	5560078	0.386824	PRKAG2
4	5460080	5560080	0.392656	TRNAC-GCA-51
4	5460080	5560080	0.392656	PRKAG2
4	5460119	5560119	0.418042	TRNAC-GCA-51
4	5460119	5560119	0.418042	PRKAG2
4	5460130	5560130	0.347974	TRNAC-GCA-51
4	5460130	5560130	0.347974	PRKAG2
4	5460248	5560248	0.488525	TRNAC-GCA-51
4	5460248	5560248	0.488525	PRKAG2
4	5460323	5560323	0.446156	TRNAC-GCA-51
4	5460323	5560323	0.446156	PRKAG2
4	5460324	5560324	0.479342	TRNAC-GCA-51
4	5460324	5560324	0.479342	PRKAG2
4	5460720	5560720	0.346321	TRNAC-GCA-51
4	5460720	5560720	0.346321	PRKAG2
4	5475953	5575953	0.405399	PRKAG2
4	5476058	5576058	0.350503	PRKAG2
4	5476155	5576155	0.411866	PRKAG2
4	5568507	5668507	0.342018	PRKAG2
4	5574917	5674917	0.345032	PRKAG2
4	5574988	5674988	0.361864	PRKAG2
4	5575002	5675002	0.371603	PRKAG2
4	5575787	5675787	0.352202	PRKAG2
4	5576257	5676257	0.346088	PRKAG2
4	5576391	5676391	0.346186	PRKAG2
4	5579500	5679500	0.347078	PRKAG2
4	5579509	5679509	0.343016	PRKAG2
4	5579571	5679571	0.346171	PRKAG2
4	5581414	5681414	0.345037	PRKAG2
4	5582362	5682362	0.347124	PRKAG2
4	5582514	5682514	0.355517	PRKAG2
4	5582558	5682558	0.356138	PRKAG2
4	5582917	5682917	0.343563	PRKAG2
4	5583354	5683354	0.347586	PRKAG2
4	5583660	5683660	0.362657	PRKAG2
4	5583691	5683691	0.351454	PRKAG2
4	5583783	5683783	0.366304	PRKAG2
4	5584383	5684383	0.346451	PRKAG2
4	5585224	5685224	0.34832	PRKAG2
4	5585239	5685239	0.350529	PRKAG2
4	5585245	5685245	0.351622	PRKAG2
4	5585589	5685589	0.355792	PRKAG2

4	5586150	5686150	0.375719	PRKAG2
4	5586198	5686198	0.355043	PRKAG2
4	5586226	5686226	0.359759	PRKAG2
4	5586261	5686261	0.346908	PRKAG2
4	5586289	5686289	0.348148	PRKAG2
4	5586438	5686438	0.352307	PRKAG2
4	5586513	5686513	0.351987	PRKAG2
4	5586558	5686558	0.352794	PRKAG2
4	5586627	5686627	0.360102	PRKAG2
4	5586655	5686655	0.372419	PRKAG2
4	5586708	5686708	0.348688	PRKAG2
4	5586764	5686764	0.360864	PRKAG2
4	5586934	5686934	0.369402	PRKAG2
4	5587144	5687144	0.354851	PRKAG2
4	5587161	5687161	0.358518	PRKAG2
4	5587163	5687163	0.362538	PRKAG2
4	5587164	5687164	0.359432	PRKAG2
4	5587260	5687260	0.36076	PRKAG2
4	5587308	5687308	0.366025	PRKAG2
4	5587323	5687323	0.382588	PRKAG2
4	5587357	5687357	0.371272	PRKAG2
4	5587438	5687438	0.368423	PRKAG2
4	5587590	5687590	0.347517	PRKAG2
4	5587809	5687809	0.360466	PRKAG2
4	5588033	5688033	0.369348	PRKAG2
4	5588091	5688091	0.363096	PRKAG2
4	5588098	5688098	0.365493	PRKAG2
4	5588117	5688117	0.365507	PRKAG2
4	5588135	5688135	0.370816	PRKAG2
4	5588173	5688173	0.369248	PRKAG2
4	5588227	5688227	0.371873	PRKAG2
4	5588277	5688277	0.377512	PRKAG2
4	5588298	5688298	0.362555	PRKAG2
4	5588435	5688435	0.368362	PRKAG2
4	5588529	5688529	0.356921	PRKAG2
4	5588596	5688596	0.363643	PRKAG2
4	5588669	5688669	0.361984	PRKAG2
4	5588780	5688780	0.360951	PRKAG2
4	5589126	5689126	0.36067	PRKAG2
4	5589209	5689209	0.35594	PRKAG2
4	5589267	5689267	0.36737	PRKAG2
4	5589268	5689268	0.362827	PRKAG2
4	5589381	5689381	0.363453	PRKAG2
4	5589397	5689397	0.367539	PRKAG2
4	5589436	5689436	0.376267	PRKAG2
4	5589643	5689643	0.362481	PRKAG2
4	5589649	5689649	0.366991	PRKAG2
4	5589691	5689691	0.363109	PRKAG2
4	5589822	5689822	0.365348	PRKAG2
4	5589899	5689899	0.363096	PRKAG2
4	5589967	5689967	0.368759	PRKAG2
4	5589971	5689971	0.349396	PRKAG2
4	5590269	5690269	0.348988	PRKAG2
4	5592511	5692511	0.382895	PRKAG2
4	5592925	5692925	0.359796	PRKAG2
4	5592967	5692967	0.373114	PRKAG2
4	5593019	5693019	0.379713	PRKAG2
4	5593022	5693022	0.397061	PRKAG2

4	5593074	5693074	0.395693	PRKAG2
4	5593126	5693126	0.398964	PRKAG2
4	5593164	5693164	0.396522	PRKAG2
4	5593176	5693176	0.389841	PRKAG2
4	5593215	5693215	0.410074	PRKAG2
4	5593260	5693260	0.401676	PRKAG2
4	5593262	5693262	0.401919	PRKAG2
4	5593272	5693272	0.390599	PRKAG2
4	5593274	5693274	0.39309	PRKAG2
4	5593334	5693334	0.407756	PRKAG2
4	5593339	5693339	0.383853	PRKAG2
4	5593447	5693447	0.382214	PRKAG2
4	5593455	5693455	0.379395	PRKAG2
4	5593513	5693513	0.391428	PRKAG2
4	5593517	5693517	0.39363	PRKAG2
4	5593544	5693544	0.392954	PRKAG2
4	5593668	5693668	0.39488	PRKAG2
4	5593735	5693735	0.409442	PRKAG2
4	5593750	5693750	0.407271	PRKAG2
4	5593751	5693751	0.40413	PRKAG2
4	5593778	5693778	0.422596	PRKAG2
4	5593789	5693789	0.418707	PRKAG2
4	5593858	5693858	0.36991	PRKAG2
4	5593940	5693940	0.372105	PRKAG2
4	5593958	5693958	0.372105	PRKAG2
4	5593993	5693993	0.372105	PRKAG2
4	5593995	5693995	0.372105	PRKAG2
4	5594015	5694015	0.372105	PRKAG2
4	5594032	5694032	0.372105	PRKAG2
4	5594054	5694054	0.372105	PRKAG2
4	5594061	5694061	0.372105	PRKAG2
4	5594073	5694073	0.372105	PRKAG2
4	5594088	5694088	0.383919	PRKAG2
4	5594092	5694092	0.374624	PRKAG2
4	5594095	5694095	0.383852	PRKAG2
4	5594113	5694113	0.381298	PRKAG2
4	5594118	5694118	0.381298	PRKAG2
4	5594175	5694175	0.381298	PRKAG2
4	5594200	5694200	0.379964	PRKAG2
4	5594340	5694340	0.378617	PRKAG2
4	5594401	5694401	0.378066	PRKAG2
4	5594418	5694418	0.378066	PRKAG2
4	5594469	5694469	0.378066	PRKAG2
4	5594492	5694492	0.381932	PRKAG2
4	5594540	5694540	0.37971	PRKAG2
4	5594587	5694587	0.391864	PRKAG2
4	5594659	5694659	0.386899	PRKAG2
4	5594699	5694699	0.371629	PRKAG2
4	5594711	5694711	0.371629	PRKAG2
4	5594775	5694775	0.376799	PRKAG2
4	5594982	5694982	0.366719	PRKAG2
4	5594985	5694985	0.362889	PRKAG2
4	5595132	5695132	0.376975	PRKAG2
4	5595168	5695168	0.384827	PRKAG2
4	5595217	5695217	0.392857	PRKAG2
4	5595225	5695225	0.392857	PRKAG2
4	5595468	5695468	0.363182	PRKAG2
4	5595672	5695672	0.370368	PRKAG2

4	5595777	5695777	0.36621	PRKAG2
4	6417966	6517966	0.342857	TMEM176B
4	6417966	6517966	0.342857	AOC1
4	6417966	6517966	0.342857	TMEM176A
4	6418240	6518240	0.382033	TMEM176B
4	6418240	6518240	0.382033	AOC1
4	6418240	6518240	0.382033	TMEM176A
4	6418388	6518388	0.379415	TMEM176B
4	6418388	6518388	0.379415	AOC1
4	6418388	6518388	0.379415	TMEM176A
4	6418412	6518412	0.386154	TMEM176B
4	6418412	6518412	0.386154	AOC1
4	6418412	6518412	0.386154	TMEM176A
4	6418789	6518789	0.377377	TMEM176B
4	6418789	6518789	0.377377	AOC1
4	6418789	6518789	0.377377	TMEM176A
4	6418811	6518811	0.37266	TMEM176B
4	6418811	6518811	0.37266	AOC1
4	6418811	6518811	0.37266	TMEM176A
4	6418879	6518879	0.388786	TMEM176B
4	6418879	6518879	0.388786	AOC1
4	6418879	6518879	0.388786	TMEM176A
4	6418910	6518910	0.39368	TMEM176B
4	6418910	6518910	0.39368	AOC1
4	6418910	6518910	0.39368	TMEM176A
4	6418911	6518911	0.394882	TMEM176B
4	6418911	6518911	0.394882	AOC1
4	6418911	6518911	0.394882	TMEM176A
4	6418941	6518941	0.393512	TMEM176B
4	6418941	6518941	0.393512	AOC1
4	6418941	6518941	0.393512	TMEM176A
4	6418955	6518955	0.388733	TMEM176B
4	6418955	6518955	0.388733	AOC1
4	6418955	6518955	0.388733	TMEM176A
4	6418964	6518964	0.388733	TMEM176B
4	6418964	6518964	0.388733	AOC1
4	6418964	6518964	0.388733	TMEM176A
4	6418994	6518994	0.391799	TMEM176B
4	6418994	6518994	0.391799	AOC1
4	6418994	6518994	0.391799	TMEM176A
4	6419041	6519041	0.385682	TMEM176B
4	6419041	6519041	0.385682	AOC1
4	6419041	6519041	0.385682	TMEM176A
4	6419055	6519055	0.395217	TMEM176B
4	6419055	6519055	0.395217	AOC1
4	6419055	6519055	0.395217	TMEM176A
4	6419076	6519076	0.389419	TMEM176B
4	6419076	6519076	0.389419	AOC1
4	6419076	6519076	0.389419	TMEM176A
4	6419087	6519087	0.356425	TMEM176B
4	6419087	6519087	0.356425	AOC1
4	6419087	6519087	0.356425	TMEM176A
4	6419325	6519325	0.363088	TMEM176B
4	6419325	6519325	0.363088	AOC1
4	6419325	6519325	0.363088	TMEM176A
4	6419333	6519333	0.371587	TMEM176B
4	6419333	6519333	0.371587	AOC1
4	6419333	6519333	0.371587	TMEM176A

4	6443540	6543540	0.356774	TMEM176B
4	6443540	6543540	0.356774	AOC1
4	6443540	6543540	0.356774	TMEM176A
4	6443553	6543553	0.374684	TMEM176B
4	6443553	6543553	0.374684	AOC1
4	6443553	6543553	0.374684	TMEM176A
4	6443569	6543569	0.377307	TMEM176B
4	6443569	6543569	0.377307	AOC1
4	6443569	6543569	0.377307	TMEM176A
4	6443607	6543607	0.391019	TMEM176B
4	6443607	6543607	0.391019	AOC1
4	6443607	6543607	0.391019	TMEM176A
4	6443624	6543624	0.387646	TMEM176B
4	6443624	6543624	0.387646	AOC1
4	6443624	6543624	0.387646	TMEM176A
4	6443625	6543625	0.387646	TMEM176B
4	6443625	6543625	0.387646	AOC1
4	6443625	6543625	0.387646	TMEM176A
4	6443626	6543626	0.387646	TMEM176B
4	6443626	6543626	0.387646	AOC1
4	6443626	6543626	0.387646	TMEM176A
4	6443671	6543671	0.378439	TMEM176B
4	6443671	6543671	0.378439	AOC1
4	6443671	6543671	0.378439	TMEM176A
4	6443676	6543676	0.440596	TMEM176B
4	6443676	6543676	0.440596	AOC1
4	6443676	6543676	0.440596	TMEM176A
4	6443742	6543742	0.365052	TMEM176B
4	6443742	6543742	0.365052	AOC1
4	6443742	6543742	0.365052	TMEM176A
4	6443756	6543756	0.350389	TMEM176B
4	6443756	6543756	0.350389	AOC1
4	6443756	6543756	0.350389	TMEM176A
4	6443837	6543837	0.360917	TMEM176B
4	6443837	6543837	0.360917	AOC1
4	6443837	6543837	0.360917	TMEM176A
4	6443917	6543917	0.361035	TMEM176B
4	6443917	6543917	0.361035	AOC1
4	6443917	6543917	0.361035	TMEM176A
4	7273663	7373663	0.359196	LOC102180028
4	7273663	7373663	0.359196	LOC102178618
4	7273663	7373663	0.359196	LOC102187124
4	7274047	7374047	0.364553	LOC102180028
4	7274047	7374047	0.364553	LOC102178618
4	7274047	7374047	0.364553	LOC102187124
4	7274273	7374273	0.375203	LOC102180028
4	7274273	7374273	0.375203	LOC102178618
4	7274273	7374273	0.375203	LOC102187124
4	7274335	7374335	0.375367	LOC102180028
4	7274335	7374335	0.375367	LOC102178618
4	7274335	7374335	0.375367	LOC102187124
4	7274389	7374389	0.371263	LOC102180028
4	7274389	7374389	0.371263	LOC102178618
4	7274389	7374389	0.371263	LOC102187124
4	7274542	7374542	0.361054	LOC102180028
4	7274542	7374542	0.361054	LOC102178618
4	7274542	7374542	0.361054	LOC102187124
4	7274885	7374885	0.361054	LOC102180028

4	7274885	7374885	0.361054	LOC102178618
4	7274885	7374885	0.361054	LOC102187124
4	7274924	7374924	0.361054	LOC102180028
4	7274924	7374924	0.361054	LOC102178618
4	7274924	7374924	0.361054	LOC102187124
4	7274936	7374936	0.359511	LOC102180028
4	7274936	7374936	0.359511	LOC102178618
4	7274936	7374936	0.359511	LOC102187124
4	7275027	7375027	0.367785	LOC102180028
4	7275027	7375027	0.367785	LOC102178618
4	7275027	7375027	0.367785	LOC102187124
4	7275050	7375050	0.363369	LOC102180028
4	7275050	7375050	0.363369	LOC102178618
4	7275050	7375050	0.363369	LOC102187124
4	7275062	7375062	0.363369	LOC102180028
4	7275062	7375062	0.363369	LOC102178618
4	7275062	7375062	0.363369	LOC102187124
4	7275304	7375304	0.343056	LOC102180028
4	7275304	7375304	0.343056	LOC102178618
4	7275304	7375304	0.343056	LOC102187124
4	7275306	7375306	0.343056	LOC102180028
4	7275306	7375306	0.343056	LOC102178618
4	7275306	7375306	0.343056	LOC102187124
4	7275672	7375672	0.365759	LOC102180028
4	7275672	7375672	0.365759	LOC102178618
4	7275672	7375672	0.365759	LOC102187124
4	7276007	7376007	0.347058	LOC102180028
4	7276007	7376007	0.347058	LOC102178618
4	7276007	7376007	0.347058	LOC102187124
4	7276238	7376238	0.343339	LOC102180028
4	7276238	7376238	0.343339	LOC102178618
4	7276238	7376238	0.343339	LOC102187124
4	7276292	7376292	0.356695	LOC102180028
4	7276292	7376292	0.356695	LOC102178618
4	7276292	7376292	0.356695	LOC102187124
4	7276586	7376586	0.356695	LOC102180028
4	7276586	7376586	0.356695	LOC102178618
4	7276586	7376586	0.356695	LOC102187124
4	7276604	7376604	0.361141	LOC102180028
4	7276604	7376604	0.361141	LOC102178618
4	7276604	7376604	0.361141	LOC102187124
4	7276800	7376800	0.346009	LOC102180028
4	7276800	7376800	0.346009	LOC102178618
4	7276800	7376800	0.346009	LOC102187124
4	7277053	7377053	0.346009	LOC102180028
4	7277053	7377053	0.346009	LOC102178618
4	7277053	7377053	0.346009	LOC102187124
4	7277353	7377353	0.352743	LOC102180028
4	7277353	7377353	0.352743	LOC102178618
4	7277353	7377353	0.352743	LOC102187124
4	7277459	7377459	0.355549	LOC102180028
4	7277459	7377459	0.355549	LOC102178618
4	7277459	7377459	0.355549	LOC102187124
4	7277662	7377662	0.349529	LOC102180028
4	7277662	7377662	0.349529	LOC102178618
4	7277662	7377662	0.349529	LOC102187124
4	7277855	7377855	0.37586	LOC102180028
4	7277855	7377855	0.37586	LOC102178618

4	7277855	7377855	0.37586	LOC102187124
4	7278061	7378061	0.367136	LOC102180028
4	7278061	7378061	0.367136	LOC102178618
4	7278061	7378061	0.367136	LOC102187124
4	7278721	7378721	0.347016	LOC102180028
4	7278721	7378721	0.347016	LOC102178618
4	7278721	7378721	0.347016	LOC102187124
4	7278887	7378887	0.348536	LOC102180028
4	7278887	7378887	0.348536	LOC102178618
4	7278887	7378887	0.348536	LOC102187124
4	7278975	7378975	0.348536	LOC102180028
4	7278975	7378975	0.348536	LOC102178618
4	7278975	7378975	0.348536	LOC102187124
4	7279037	7379037	0.351781	LOC102180028
4	7279037	7379037	0.351781	LOC102178618
4	7279037	7379037	0.351781	LOC102187124
4	7279086	7379086	0.348541	LOC102180028
4	7279086	7379086	0.348541	LOC102178618
4	7279086	7379086	0.348541	LOC102187124
4	7279121	7379121	0.346485	LOC102180028
4	7279121	7379121	0.346485	LOC102178618
4	7279121	7379121	0.346485	LOC102187124
4	7279367	7379367	0.349679	LOC102180028
4	7279367	7379367	0.349679	LOC102178618
4	7279367	7379367	0.349679	LOC102187124
4	7279373	7379373	0.350266	LOC102180028
4	7279373	7379373	0.350266	LOC102178618
4	7279373	7379373	0.350266	LOC102187124
4	7279493	7379493	0.350501	LOC102180028
4	7279493	7379493	0.350501	LOC102178618
4	7279493	7379493	0.350501	LOC102187124
4	7279709	7379709	0.350908	LOC102180028
4	7279709	7379709	0.350908	LOC102178618
4	7279709	7379709	0.350908	LOC102187124
4	7279798	7379798	0.342008	LOC102180028
4	7279798	7379798	0.342008	LOC102178618
4	7279798	7379798	0.342008	LOC102187124
4	7280112	7380112	0.343683	LOC102180028
4	7280112	7380112	0.343683	LOC102178618
4	7280112	7380112	0.343683	LOC102187124
4	7280121	7380121	0.343683	LOC102180028
4	7280121	7380121	0.343683	LOC102178618
4	7280121	7380121	0.343683	LOC102187124
4	7280171	7380171	0.372063	LOC102180028
4	7280171	7380171	0.372063	LOC102178618
4	7280171	7380171	0.372063	LOC102187124
4	7287966	7387966	0.411862	LOC102180028
4	7287966	7387966	0.411862	LOC102178618
4	7288060	7388060	0.34822	LOC102180028
4	7288060	7388060	0.34822	LOC102178618
4	7288125	7388125	0.35444	LOC102180028
4	7288125	7388125	0.35444	LOC102178618
4	7288204	7388204	0.348778	LOC102180028
4	7288204	7388204	0.348778	LOC102178618
4	7288220	7388220	0.349526	LOC102180028
4	7288220	7388220	0.349526	LOC102178618
4	7288272	7388272	0.390211	LOC102180028
4	7288272	7388272	0.390211	LOC102178618

4	7288402	7388402	0.39262	LOC102180028
4	7288402	7388402	0.39262	LOC102178618
4	7288403	7388403	0.39262	LOC102180028
4	7288403	7388403	0.39262	LOC102178618
4	7288465	7388465	0.349142	LOC102180028
4	7288465	7388465	0.349142	LOC102178618
4	7288876	7388876	0.348721	LOC102180028
4	7288876	7388876	0.348721	LOC102178618
4	7289054	7389054	0.352358	LOC102180028
4	7289054	7389054	0.352358	LOC102178618
4	7289647	7389647	0.370911	LOC102180028
4	7289647	7389647	0.370911	LOC102178618
4	7297976	7397976	0.344554	LOC102180028
4	7297976	7397976	0.344554	LOC102178618
4	7297993	7397993	0.360839	LOC102180028
4	7297993	7397993	0.360839	LOC102178618
4	7349130	7449130	0.360068	LOC102178618
4	7349130	7449130	0.360068	LOC102178162
4	7369843	7469843	0.349791	LOC102178162
4	7369851	7469851	0.34369	LOC102178162
4	7369852	7469852	0.34369	LOC102178162
4	7372762	7472762	0.352974	LOC102179185
4	7372762	7472762	0.352974	LOC102178162
4	7372879	7472879	0.391698	LOC102179185
4	7372879	7472879	0.391698	LOC102178162
4	7372981	7472981	0.406873	LOC102179185
4	7372981	7472981	0.406873	LOC102178162
4	7372996	7472996	0.416727	LOC102179185
4	7372996	7472996	0.416727	LOC102178162
4	7373008	7473008	0.408646	LOC102179185
4	7373008	7473008	0.408646	LOC102178162
4	7373046	7473046	0.406021	LOC102179185
4	7373046	7473046	0.406021	LOC102178162
4	7373196	7473196	0.444895	LOC102179185
4	7373196	7473196	0.444895	LOC102178162
4	7373220	7473220	0.427906	LOC102179185
4	7373220	7473220	0.427906	LOC102178162
4	7373221	7473221	0.429195	LOC102179185
4	7373221	7473221	0.429195	LOC102178162
4	7373235	7473235	0.443133	LOC102179185
4	7373235	7473235	0.443133	LOC102178162
4	7373237	7473237	0.443133	LOC102179185
4	7373237	7473237	0.443133	LOC102178162
4	7373316	7473316	0.375959	LOC102179185
4	7373316	7473316	0.375959	LOC102178162
4	7373712	7473712	0.377185	LOC102179185
4	7373712	7473712	0.377185	LOC102178162
4	7373770	7473770	0.376148	LOC102179185
4	7373770	7473770	0.376148	LOC102178162
4	7373833	7473833	0.372566	LOC102179185
4	7373833	7473833	0.372566	LOC102178162
4	7373907	7473907	0.37866	LOC102179185
4	7373907	7473907	0.37866	LOC102178162
4	7373928	7473928	0.37779	LOC102179185
4	7373928	7473928	0.37779	LOC102178162
4	7374010	7474010	0.375866	LOC102179185
4	7374010	7474010	0.375866	LOC102178162
4	7374092	7474092	0.376501	LOC102179185

4	7374092	7474092	0.376501	LOC102178162
4	7374400	7474400	0.377223	LOC102179185
4	7374400	7474400	0.377223	LOC102178162
4	7374675	7474675	0.374399	LOC102179185
4	7374675	7474675	0.374399	LOC102178162
4	7374678	7474678	0.374399	LOC102179185
4	7374678	7474678	0.374399	LOC102178162
4	7374714	7474714	0.377394	LOC102179185
4	7374714	7474714	0.377394	LOC102178162
4	7374748	7474748	0.399629	LOC102179185
4	7374748	7474748	0.399629	LOC102178162
4	7374756	7474756	0.374499	LOC102179185
4	7374756	7474756	0.374499	LOC102178162
4	7374813	7474813	0.34725	LOC102179185
4	7374813	7474813	0.34725	LOC102178162
4	7374831	7474831	0.346407	LOC102179185
4	7374831	7474831	0.346407	LOC102178162
4	7375371	7475371	0.377929	LOC102179185
4	7375371	7475371	0.377929	LOC102178162
4	7375381	7475381	0.36846	LOC102179185
4	7375381	7475381	0.36846	LOC102178162
4	7375385	7475385	0.349358	LOC102179185
4	7375385	7475385	0.349358	LOC102178162
4	7375400	7475400	0.347553	LOC102179185
4	7375400	7475400	0.347553	LOC102178162
4	7375513	7475513	0.396981	LOC102179185
4	7375513	7475513	0.396981	LOC102178162
4	7375686	7475686	0.395226	LOC102179185
4	7375686	7475686	0.395226	LOC102178162
4	7375938	7475938	0.389342	LOC102179185
4	7375938	7475938	0.389342	LOC102178162
4	7376023	7476023	0.398439	LOC102179185
4	7376023	7476023	0.398439	LOC102178162
4	7376224	7476224	0.384269	LOC102179185
4	7376224	7476224	0.384269	LOC102178162
4	7376697	7476697	0.377393	LOC102179185
4	7376697	7476697	0.377393	LOC102178162
4	7376709	7476709	0.39116	LOC102179185
4	7376709	7476709	0.39116	LOC102178162
4	7376717	7476717	0.365687	LOC102179185
4	7376717	7476717	0.365687	LOC102178162
4	7376800	7476800	0.388869	LOC102179185
4	7376800	7476800	0.388869	LOC102178162
4	7376861	7476861	0.384507	LOC102179185
4	7376861	7476861	0.384507	LOC102178162
4	7376917	7476917	0.368909	LOC102179185
4	7376917	7476917	0.368909	LOC102178162
4	7426147	7526147	0.436768	LOC102179185
4	7426499	7526499	0.400845	LOC102179185
4	7426713	7526713	0.401915	LOC102179185
4	7426728	7526728	0.395213	LOC102179185
4	7426878	7526878	0.379335	LOC102179185
4	7426938	7526938	0.35231	LOC102179185
4	7427120	7527120	0.350241	LOC102179185
4	7427121	7527121	0.354645	LOC102179185
4	7438084	7538084	0.37692	LOC102179185
4	7438109	7538109	0.350188	LOC102179185
4	7438197	7538197	0.37276	LOC102179185

4	7438202	7538202	0.378562	LOC102179185
4	7438710	7538710	0.371741	LOC102179185
4	7438745	7538745	0.37222	LOC102179185
4	7439622	7539622	0.404522	LOC102179185
4	7439638	7539638	0.386486	LOC102179185
4	7439816	7539816	0.434442	LOC102179185
4	7439824	7539824	0.414515	LOC102179185
4	7439999	7539999	0.408991	LOC102179185
4	7440175	7540175	0.410373	LOC102179185
4	7440231	7540231	0.404086	LOC102179185
4	7440376	7540376	0.347653	LOC102179185
4	7441913	7541913	0.348655	LOC102179185
4	7441914	7541914	0.353742	LOC102179185
4	7441925	7541925	0.358452	LOC102179185
4	7441944	7541944	0.362002	LOC102179185
4	7441957	7541957	0.36188	LOC102179185
4	7441958	7541958	0.361941	LOC102179185
4	7441978	7541978	0.358375	LOC102179185
4	7443564	7543564	0.344254	LOC102179185
4	7443572	7543572	0.350669	LOC102179185
4	7552894	7652894	0.450714	LOC102179465
4	7552894	7652894	0.450714	LOC102186105
4	7552907	7652907	0.450714	LOC102179465
4	7552907	7652907	0.450714	LOC102186105
4	7552908	7652908	0.457199	LOC102179465
4	7552908	7652908	0.457199	LOC102186105
4	7552920	7652920	0.456115	LOC102179465
4	7552920	7652920	0.456115	LOC102186105
4	7552937	7652937	0.456101	LOC102179465
4	7552937	7652937	0.456101	LOC102186105
4	7552938	7652938	0.471002	LOC102179465
4	7552938	7652938	0.471002	LOC102186105
4	7552940	7652940	0.471241	LOC102179465
4	7552940	7652940	0.471241	LOC102186105
4	7552971	7652971	0.47077	LOC102179465
4	7552971	7652971	0.47077	LOC102186105
4	7552987	7652987	0.443678	LOC102179465
4	7552987	7652987	0.443678	LOC102186105
4	7552989	7652989	0.443678	LOC102179465
4	7552989	7652989	0.443678	LOC102186105
4	7552999	7652999	0.443678	LOC102179465
4	7552999	7652999	0.443678	LOC102186105
4	7553015	7653015	0.443678	LOC102179465
4	7553015	7653015	0.443678	LOC102186105
4	7553047	7653047	0.444522	LOC102179465
4	7553047	7653047	0.444522	LOC102186105
4	7589108	7689108	0.343922	LOC102186105
4	7589108	7689108	0.343922	LOC102186385
4	7589109	7689109	0.343922	LOC102186105
4	7589109	7689109	0.343922	LOC102186385
4	7589470	7689470	0.343065	LOC102186105
4	7589470	7689470	0.343065	LOC102186385
4	7589714	7689714	0.395881	LOC102186105
4	7589714	7689714	0.395881	LOC102186385
4	7589755	7689755	0.388422	LOC102186105
4	7589755	7689755	0.388422	LOC102186385
4	7589775	7689775	0.392154	LOC102186105
4	7589775	7689775	0.392154	LOC102186385

4	7589809	7689809	0.392154	LOC102186105
4	7589809	7689809	0.392154	LOC102186385
4	7589848	7689848	0.392612	LOC102186105
4	7589848	7689848	0.392612	LOC102186385
4	7589867	7689867	0.39227	LOC102186105
4	7589867	7689867	0.39227	LOC102186385
4	7589887	7689887	0.392527	LOC102186105
4	7589887	7689887	0.392527	LOC102186385
4	7590093	7690093	0.394024	LOC102186105
4	7590093	7690093	0.394024	LOC102186385
4	7590139	7690139	0.394252	LOC102186105
4	7590139	7690139	0.394252	LOC102186385
4	7590159	7690159	0.395067	LOC102186105
4	7590159	7690159	0.395067	LOC102186385
4	7590178	7690178	0.395214	LOC102186105
4	7590178	7690178	0.395214	LOC102186385
4	7590244	7690244	0.394855	LOC102186105
4	7590244	7690244	0.394855	LOC102186385
4	7590245	7690245	0.393918	LOC102186105
4	7590245	7690245	0.393918	LOC102186385
4	7590289	7690289	0.394574	LOC102186105
4	7590289	7690289	0.394574	LOC102186385
4	7590397	7690397	0.394443	LOC102186105
4	7590397	7690397	0.394443	LOC102186385
4	7590399	7690399	0.394295	LOC102186105
4	7590399	7690399	0.394295	LOC102186385
4	7590475	7690475	0.394304	LOC102186105
4	7590475	7690475	0.394304	LOC102186385
4	7590492	7690492	0.395341	LOC102186105
4	7590492	7690492	0.395341	LOC102186385
4	7590512	7690512	0.394854	LOC102186105
4	7590512	7690512	0.394854	LOC102186385
4	7590530	7690530	0.394854	LOC102186105
4	7590530	7690530	0.394854	LOC102186385
4	7590583	7690583	0.39483	LOC102186105
4	7590583	7690583	0.39483	LOC102186385
4	7590632	7690632	0.395094	LOC102186105
4	7590632	7690632	0.395094	LOC102186385
4	7590661	7690661	0.394653	LOC102186105
4	7590661	7690661	0.394653	LOC102186385
4	7590727	7690727	0.396076	LOC102186105
4	7590727	7690727	0.396076	LOC102186385
4	7590808	7690808	0.395579	LOC102186105
4	7590808	7690808	0.395579	LOC102186385
4	7590899	7690899	0.39138	LOC102186105
4	7590899	7690899	0.39138	LOC102186385
4	7590939	7690939	0.390064	LOC102186105
4	7590939	7690939	0.390064	LOC102186385
4	7590940	7690940	0.3901	LOC102186105
4	7590940	7690940	0.3901	LOC102186385
4	7590960	7690960	0.389435	LOC102186105
4	7590960	7690960	0.389435	LOC102186385
4	7590969	7690969	0.389782	LOC102186105
4	7590969	7690969	0.389782	LOC102186385
4	7590975	7690975	0.387647	LOC102186105
4	7590975	7690975	0.387647	LOC102186385
4	7590998	7690998	0.387647	LOC102186105
4	7590998	7690998	0.387647	LOC102186385

4	7591024	7691024	0.386647	LOC102186105
4	7591024	7691024	0.386647	LOC102186385
4	7591035	7691035	0.387975	LOC102186105
4	7591035	7691035	0.387975	LOC102186385
4	7592322	7692322	0.402321	LOC102186105
4	7592322	7692322	0.402321	LOC102186385
4	7592333	7692333	0.402886	LOC102186105
4	7592333	7692333	0.402886	LOC102186385
4	7592348	7692348	0.40493	LOC102186105
4	7592348	7692348	0.40493	LOC102186385
4	7592363	7692363	0.406026	LOC102186105
4	7592363	7692363	0.406026	LOC102186385
4	7592367	7692367	0.406014	LOC102186105
4	7592367	7692367	0.406014	LOC102186385
4	7592383	7692383	0.405316	LOC102186105
4	7592383	7692383	0.405316	LOC102186385
4	7592426	7692426	0.406224	LOC102186105
4	7592426	7692426	0.406224	LOC102186385
4	7593180	7693180	0.39353	LOC102186105
4	7593180	7693180	0.39353	LOC102186385
4	7593344	7693344	0.406356	LOC102186105
4	7593344	7693344	0.406356	LOC102186385
4	7593385	7693385	0.409893	LOC102186105
4	7593385	7693385	0.409893	LOC102186385
4	7593429	7693429	0.413589	LOC102186105
4	7593429	7693429	0.413589	LOC102186385
4	7593548	7693548	0.42207	LOC102186105
4	7593548	7693548	0.42207	LOC102186385
4	7593567	7693567	0.420777	LOC102186105
4	7593567	7693567	0.420777	LOC102186385
4	7593631	7693631	0.394329	LOC102186105
4	7593631	7693631	0.394329	LOC102186385
4	7594900	7694900	0.391751	LOC102186105
4	7594900	7694900	0.391751	LOC102186385
4	7594916	7694916	0.402483	LOC102186105
4	7594916	7694916	0.402483	LOC102186385
4	7594944	7694944	0.402867	LOC102186105
4	7594944	7694944	0.402867	LOC102186385
4	7594969	7694969	0.402567	LOC102186105
4	7594969	7694969	0.402567	LOC102186385
4	7594995	7694995	0.403045	LOC102186105
4	7594995	7694995	0.403045	LOC102186385
4	7595002	7695002	0.403551	LOC102186105
4	7595002	7695002	0.403551	LOC102186385
4	7595021	7695021	0.403551	LOC102186105
4	7595021	7695021	0.403551	LOC102186385
4	7595028	7695028	0.403551	LOC102186105
4	7595028	7695028	0.403551	LOC102186385
4	7595067	7695067	0.403753	LOC102186105
4	7595067	7695067	0.403753	LOC102186385
4	7595121	7695121	0.403728	LOC102186105
4	7595121	7695121	0.403728	LOC102186385
4	7595145	7695145	0.405375	LOC102186105
4	7595145	7695145	0.405375	LOC102186385
4	7595219	7695219	0.403214	LOC102186105
4	7595219	7695219	0.403214	LOC102186385
4	7595263	7695263	0.403088	LOC102186105
4	7595263	7695263	0.403088	LOC102186385

4	7595290	7695290	0.403088	LOC102186105
4	7595290	7695290	0.403088	LOC102186385
4	7595306	7695306	0.403088	LOC102186105
4	7595306	7695306	0.403088	LOC102186385
4	7595319	7695319	0.402883	LOC102186105
4	7595319	7695319	0.402883	LOC102186385
4	7595324	7695324	0.402883	LOC102186105
4	7595324	7695324	0.402883	LOC102186385
4	7595339	7695339	0.403337	LOC102186105
4	7595339	7695339	0.403337	LOC102186385
4	7595359	7695359	0.403309	LOC102186105
4	7595359	7695359	0.403309	LOC102186385
4	7595384	7695384	0.403567	LOC102186105
4	7595384	7695384	0.403567	LOC102186385
4	7595389	7695389	0.403567	LOC102186105
4	7595389	7695389	0.403567	LOC102186385
4	7595420	7695420	0.403567	LOC102186105
4	7595420	7695420	0.403567	LOC102186385
4	7595491	7695491	0.403832	LOC102186105
4	7595491	7695491	0.403832	LOC102186385
4	7595491	7695491	0.403832	LOC106501766
4	7595496	7695496	0.403832	LOC102186105
4	7595496	7695496	0.403832	LOC102186385
4	7595496	7695496	0.403832	LOC106501766
4	7595510	7695510	0.403832	LOC102186105
4	7595510	7695510	0.403832	LOC102186385
4	7595510	7695510	0.403832	LOC106501766
4	7595515	7695515	0.403832	LOC102186105
4	7595515	7695515	0.403832	LOC102186385
4	7595515	7695515	0.403832	LOC106501766
4	7595561	7695561	0.403711	LOC102186105
4	7595561	7695561	0.403711	LOC102186385
4	7595561	7695561	0.403711	LOC106501766
4	7595562	7695562	0.404062	LOC102186105
4	7595562	7695562	0.404062	LOC102186385
4	7595562	7695562	0.404062	LOC106501766
4	7595574	7695574	0.403463	LOC102186105
4	7595574	7695574	0.403463	LOC102186385
4	7595574	7695574	0.403463	LOC106501766
4	7595614	7695614	0.403815	LOC102186105
4	7595614	7695614	0.403815	LOC102186385
4	7595614	7695614	0.403815	LOC106501766
4	7595666	7695666	0.403364	LOC102186105
4	7595666	7695666	0.403364	LOC102186385
4	7595666	7695666	0.403364	LOC106501766
4	7595760	7695760	0.403438	LOC102186105
4	7595760	7695760	0.403438	LOC102186385
4	7595760	7695760	0.403438	LOC106501766
4	7595768	7695768	0.403438	LOC102186105
4	7595768	7695768	0.403438	LOC102186385
4	7595768	7695768	0.403438	LOC106501766
4	7595797	7695797	0.403159	LOC102186105
4	7595797	7695797	0.403159	LOC102186385
4	7595797	7695797	0.403159	LOC106501766
4	7595819	7695819	0.403159	LOC102186105
4	7595819	7695819	0.403159	LOC102186385
4	7595819	7695819	0.403159	LOC106501766
4	7595896	7695896	0.404531	LOC102186105

4	7595896	7695896	0.404531	LOC102186385
4	7595896	7695896	0.404531	LOC106501766
4	7596005	7696005	0.404577	LOC102186105
4	7596005	7696005	0.404577	LOC102186385
4	7596005	7696005	0.404577	LOC106501766
4	7596019	7696019	0.404492	LOC102186105
4	7596019	7696019	0.404492	LOC102186385
4	7596019	7696019	0.404492	LOC106501766
4	7596052	7696052	0.404492	LOC102186105
4	7596052	7696052	0.404492	LOC102186385
4	7596052	7696052	0.404492	LOC106501766
4	7596112	7696112	0.404492	LOC102186105
4	7596112	7696112	0.404492	LOC102186385
4	7596112	7696112	0.404492	LOC106501766
4	7596164	7696164	0.404523	LOC102186105
4	7596164	7696164	0.404523	LOC102186385
4	7596164	7696164	0.404523	LOC106501766
4	7596179	7696179	0.404523	LOC102186105
4	7596179	7696179	0.404523	LOC102186385
4	7596179	7696179	0.404523	LOC106501766
4	7596191	7696191	0.404523	LOC102186105
4	7596191	7696191	0.404523	LOC102186385
4	7596191	7696191	0.404523	LOC106501766
4	7596242	7696242	0.404523	LOC102186105
4	7596242	7696242	0.404523	LOC102186385
4	7596242	7696242	0.404523	LOC106501766
4	7596263	7696263	0.404523	LOC102186105
4	7596263	7696263	0.404523	LOC102186385
4	7596263	7696263	0.404523	LOC106501766
4	7596305	7696305	0.40453	LOC102186105
4	7596305	7696305	0.40453	LOC102186385
4	7596305	7696305	0.40453	LOC106501766
4	7596389	7696389	0.404528	LOC102186105
4	7596389	7696389	0.404528	LOC102186385
4	7596389	7696389	0.404528	LOC106501766
4	7596408	7696408	0.405036	LOC102186105
4	7596408	7696408	0.405036	LOC102186385
4	7596408	7696408	0.405036	LOC106501766
4	7596419	7696419	0.404767	LOC102186105
4	7596419	7696419	0.404767	LOC102186385
4	7596419	7696419	0.404767	LOC106501766
4	7596434	7696434	0.404767	LOC102186105
4	7596434	7696434	0.404767	LOC102186385
4	7596434	7696434	0.404767	LOC106501766
4	7596440	7696440	0.404767	LOC102186105
4	7596440	7696440	0.404767	LOC102186385
4	7596440	7696440	0.404767	LOC106501766
4	7596443	7696443	0.404767	LOC102186105
4	7596443	7696443	0.404767	LOC102186385
4	7596443	7696443	0.404767	LOC106501766
4	7596483	7696483	0.407038	LOC102186105
4	7596483	7696483	0.407038	LOC102186385
4	7596483	7696483	0.407038	LOC106501766
4	7596484	7696484	0.407038	LOC102186105
4	7596484	7696484	0.407038	LOC102186385
4	7596484	7696484	0.407038	LOC106501766
4	7596599	7696599	0.40561	LOC102186105
4	7596599	7696599	0.40561	LOC102186385

4	7596599	7696599	0.40561	LOC106501766
4	7596614	7696614	0.40561	LOC102186105
4	7596614	7696614	0.40561	LOC102186385
4	7596614	7696614	0.40561	LOC106501766
4	7596709	7696709	0.405286	LOC102186105
4	7596709	7696709	0.405286	LOC102186385
4	7596709	7696709	0.405286	LOC106501766
4	7596725	7696725	0.405182	LOC102186105
4	7596725	7696725	0.405182	LOC102186385
4	7596725	7696725	0.405182	LOC106501766
4	7596769	7696769	0.405427	LOC102186105
4	7596769	7696769	0.405427	LOC102186385
4	7596769	7696769	0.405427	LOC106501766
4	7596770	7696770	0.405318	LOC102186105
4	7596770	7696770	0.405318	LOC102186385
4	7596770	7696770	0.405318	LOC106501766
4	7596793	7696793	0.405377	LOC102186105
4	7596793	7696793	0.405377	LOC102186385
4	7596793	7696793	0.405377	LOC106501766
4	7596827	7696827	0.405377	LOC102186105
4	7596827	7696827	0.405377	LOC102186385
4	7596827	7696827	0.405377	LOC106501766
4	7596836	7696836	0.405377	LOC102186105
4	7596836	7696836	0.405377	LOC102186385
4	7596836	7696836	0.405377	LOC106501766
4	7596856	7696856	0.405377	LOC102186105
4	7596856	7696856	0.405377	LOC102186385
4	7596856	7696856	0.405377	LOC106501766
4	7596857	7696857	0.405484	LOC102186105
4	7596857	7696857	0.405484	LOC102186385
4	7596857	7696857	0.405484	LOC106501766
4	7596914	7696914	0.405098	LOC102186105
4	7596914	7696914	0.405098	LOC102186385
4	7596914	7696914	0.405098	LOC106501766
4	7596946	7696946	0.405098	LOC102186105
4	7596946	7696946	0.405098	LOC102186385
4	7596946	7696946	0.405098	LOC106501766
4	7596963	7696963	0.407078	LOC102186105
4	7596963	7696963	0.407078	LOC102186385
4	7596963	7696963	0.407078	LOC106501766
4	7597040	7697040	0.406142	LOC102186105
4	7597040	7697040	0.406142	LOC102186385
4	7597040	7697040	0.406142	LOC106501766
4	7597071	7697071	0.416992	LOC102186105
4	7597071	7697071	0.416992	LOC102186385
4	7597071	7697071	0.416992	LOC106501766
4	7597083	7697083	0.406838	LOC102186105
4	7597083	7697083	0.406838	LOC102186385
4	7597083	7697083	0.406838	LOC106501766
4	7597105	7697105	0.406838	LOC102186105
4	7597105	7697105	0.406838	LOC102186385
4	7597105	7697105	0.406838	LOC106501766
4	7597111	7697111	0.406838	LOC102186105
4	7597111	7697111	0.406838	LOC102186385
4	7597111	7697111	0.406838	LOC106501766
4	7597124	7697124	0.406838	LOC102186105
4	7597124	7697124	0.406838	LOC102186385
4	7597124	7697124	0.406838	LOC106501766

4	7597173	7697173	0.406674	LOC102186105
4	7597173	7697173	0.406674	LOC102186385
4	7597173	7697173	0.406674	LOC106501766
4	7597202	7697202	0.406674	LOC102186105
4	7597202	7697202	0.406674	LOC102186385
4	7597202	7697202	0.406674	LOC106501766
4	7597217	7697217	0.407196	LOC102186105
4	7597217	7697217	0.407196	LOC102186385
4	7597217	7697217	0.407196	LOC106501766
4	7597266	7697266	0.407171	LOC102186105
4	7597266	7697266	0.407171	LOC102186385
4	7597266	7697266	0.407171	LOC106501766
4	7597299	7697299	0.407522	LOC102186105
4	7597299	7697299	0.407522	LOC102186385
4	7597299	7697299	0.407522	LOC106501766
4	7597303	7697303	0.407524	LOC102186105
4	7597303	7697303	0.407524	LOC102186385
4	7597303	7697303	0.407524	LOC106501766
4	7597312	7697312	0.407442	LOC102186105
4	7597312	7697312	0.407442	LOC102186385
4	7597312	7697312	0.407442	LOC106501766
4	7597325	7697325	0.407442	LOC102186105
4	7597325	7697325	0.407442	LOC102186385
4	7597325	7697325	0.407442	LOC106501766
4	7597375	7697375	0.407442	LOC102186105
4	7597375	7697375	0.407442	LOC102186385
4	7597375	7697375	0.407442	LOC106501766
4	7597393	7697393	0.407432	LOC102186105
4	7597393	7697393	0.407432	LOC102186385
4	7597393	7697393	0.407432	LOC106501766
4	7597396	7697396	0.407485	LOC102186105
4	7597396	7697396	0.407485	LOC102186385
4	7597396	7697396	0.407485	LOC106501766
4	7597398	7697398	0.407485	LOC102186105
4	7597398	7697398	0.407485	LOC102186385
4	7597398	7697398	0.407485	LOC106501766
4	7597400	7697400	0.407485	LOC102186105
4	7597400	7697400	0.407485	LOC102186385
4	7597400	7697400	0.407485	LOC106501766
4	7597411	7697411	0.412217	LOC102186105
4	7597411	7697411	0.412217	LOC102186385
4	7597411	7697411	0.412217	LOC106501766
4	7597434	7697434	0.412011	LOC102186105
4	7597434	7697434	0.412011	LOC102186385
4	7597434	7697434	0.412011	LOC106501766
4	7597435	7697435	0.411277	LOC102186105
4	7597435	7697435	0.411277	LOC102186385
4	7597435	7697435	0.411277	LOC106501766
4	7597490	7697490	0.410779	LOC102186105
4	7597490	7697490	0.410779	LOC102186385
4	7597490	7697490	0.410779	LOC106501766
4	7597516	7697516	0.41073	LOC102186105
4	7597516	7697516	0.41073	LOC102186385
4	7597516	7697516	0.41073	LOC106501766
4	7597573	7697573	0.41115	LOC102186105
4	7597573	7697573	0.41115	LOC102186385
4	7597573	7697573	0.41115	LOC106501766
4	7597578	7697578	0.41115	LOC102186105

4	7597578	7697578	0.41115	LOC102186385
4	7597578	7697578	0.41115	LOC106501766
4	7597621	7697621	0.410753	LOC102186105
4	7597621	7697621	0.410753	LOC102186385
4	7597621	7697621	0.410753	LOC106501766
4	7597626	7697626	0.410873	LOC102186105
4	7597626	7697626	0.410873	LOC102186385
4	7597626	7697626	0.410873	LOC106501766
4	7597669	7697669	0.410206	LOC102186105
4	7597669	7697669	0.410206	LOC102186385
4	7597669	7697669	0.410206	LOC106501766
4	7597801	7697801	0.413401	LOC102186105
4	7597801	7697801	0.413401	LOC102186385
4	7597801	7697801	0.413401	LOC106501766
4	7597811	7697811	0.413467	LOC102186105
4	7597811	7697811	0.413467	LOC102186385
4	7597811	7697811	0.413467	LOC106501766
4	7597812	7697812	0.413467	LOC102186105
4	7597812	7697812	0.413467	LOC102186385
4	7597812	7697812	0.413467	LOC106501766
4	7597817	7697817	0.414227	LOC102186105
4	7597817	7697817	0.414227	LOC102186385
4	7597817	7697817	0.414227	LOC106501766
4	7597830	7697830	0.414064	LOC102186105
4	7597830	7697830	0.414064	LOC102186385
4	7597830	7697830	0.414064	LOC106501766
4	7597833	7697833	0.414064	LOC102186105
4	7597833	7697833	0.414064	LOC102186385
4	7597833	7697833	0.414064	LOC106501766
4	7597867	7697867	0.413355	LOC102186105
4	7597867	7697867	0.413355	LOC102186385
4	7597867	7697867	0.413355	LOC106501766
4	7597932	7697932	0.412545	LOC102186105
4	7597932	7697932	0.412545	LOC102186385
4	7597932	7697932	0.412545	LOC106501766
4	7597954	7697954	0.403557	LOC102186105
4	7597954	7697954	0.403557	LOC102186385
4	7597954	7697954	0.403557	LOC106501766
4	7598003	7698003	0.420967	LOC102186105
4	7598003	7698003	0.420967	LOC102186385
4	7598003	7698003	0.420967	LOC106501766
4	7598008	7698008	0.420805	LOC102186105
4	7598008	7698008	0.420805	LOC102186385
4	7598008	7698008	0.420805	LOC106501766
4	7598020	7698020	0.420534	LOC102186105
4	7598020	7698020	0.420534	LOC102186385
4	7598020	7698020	0.420534	LOC106501766
4	7598344	7698344	0.409314	LOC102186105
4	7598344	7698344	0.409314	LOC102186385
4	7598344	7698344	0.409314	LOC106501766
4	7598358	7698358	0.410539	LOC102186105
4	7598358	7698358	0.410539	LOC102186385
4	7598358	7698358	0.410539	LOC106501766
4	7598390	7698390	0.410568	LOC102186105
4	7598390	7698390	0.410568	LOC102186385
4	7598390	7698390	0.410568	LOC106501766
4	7598406	7698406	0.410584	LOC102186105
4	7598406	7698406	0.410584	LOC102186385

4	7598406	7698406	0.410584	LOC106501766
4	7598424	7698424	0.410584	LOC102186105
4	7598424	7698424	0.410584	LOC102186385
4	7598424	7698424	0.410584	LOC106501766
4	7598439	7698439	0.409804	LOC102186105
4	7598439	7698439	0.409804	LOC102186385
4	7598439	7698439	0.409804	LOC106501766
4	7598454	7698454	0.409931	LOC102186105
4	7598454	7698454	0.409931	LOC102186385
4	7598454	7698454	0.409931	LOC106501766
4	7598458	7698458	0.409931	LOC102186105
4	7598458	7698458	0.409931	LOC102186385
4	7598458	7698458	0.409931	LOC106501766
4	7598467	7698467	0.40991	LOC102186105
4	7598467	7698467	0.40991	LOC102186385
4	7598467	7698467	0.40991	LOC106501766
4	7598479	7698479	0.409877	LOC102186105
4	7598479	7698479	0.409877	LOC102186385
4	7598479	7698479	0.409877	LOC106501766
4	7598501	7698501	0.409956	LOC102186105
4	7598501	7698501	0.409956	LOC102186385
4	7598501	7698501	0.409956	LOC106501766
4	7598518	7698518	0.41003	LOC102186105
4	7598518	7698518	0.41003	LOC102186385
4	7598518	7698518	0.41003	LOC106501766
4	7598608	7698608	0.409719	LOC102186105
4	7598608	7698608	0.409719	LOC102186385
4	7598608	7698608	0.409719	LOC106501766
4	7598610	7698610	0.409719	LOC102186105
4	7598610	7698610	0.409719	LOC102186385
4	7598610	7698610	0.409719	LOC106501766
4	7598618	7698618	0.410154	LOC102186105
4	7598618	7698618	0.410154	LOC102186385
4	7598618	7698618	0.410154	LOC106501766
4	7598622	7698622	0.410154	LOC102186105
4	7598622	7698622	0.410154	LOC102186385
4	7598622	7698622	0.410154	LOC106501766
4	7598643	7698643	0.405416	LOC102186105
4	7598643	7698643	0.405416	LOC102186385
4	7598643	7698643	0.405416	LOC106501766
4	7598646	7698646	0.405724	LOC102186105
4	7598646	7698646	0.405724	LOC102186385
4	7598646	7698646	0.405724	LOC106501766
4	7598729	7698729	0.402341	LOC102186105
4	7598729	7698729	0.402341	LOC102186385
4	7598729	7698729	0.402341	LOC106501766
4	7598775	7698775	0.401561	LOC102186105
4	7598775	7698775	0.401561	LOC102186385
4	7598775	7698775	0.401561	LOC106501766
4	7598777	7698777	0.401561	LOC102186105
4	7598777	7698777	0.401561	LOC102186385
4	7598777	7698777	0.401561	LOC106501766
4	7598785	7698785	0.401561	LOC102186105
4	7598785	7698785	0.401561	LOC102186385
4	7598785	7698785	0.401561	LOC106501766
4	7598797	7698797	0.401561	LOC102186105
4	7598797	7698797	0.401561	LOC102186385
4	7598797	7698797	0.401561	LOC106501766

4	7598802	7698802	0.401708	LOC102186105
4	7598802	7698802	0.401708	LOC102186385
4	7598802	7698802	0.401708	LOC106501766
4	7598817	7698817	0.402272	LOC102186105
4	7598817	7698817	0.402272	LOC102186385
4	7598817	7698817	0.402272	LOC106501766
4	7598852	7698852	0.402272	LOC102186105
4	7598852	7698852	0.402272	LOC102186385
4	7598852	7698852	0.402272	LOC106501766
4	7598853	7698853	0.402329	LOC102186105
4	7598853	7698853	0.402329	LOC102186385
4	7598853	7698853	0.402329	LOC106501766
4	7598860	7698860	0.40095	LOC102186105
4	7598860	7698860	0.40095	LOC102186385
4	7598860	7698860	0.40095	LOC106501766
4	7598874	7698874	0.40095	LOC102186105
4	7598874	7698874	0.40095	LOC102186385
4	7598874	7698874	0.40095	LOC106501766
4	7598907	7698907	0.40095	LOC102186105
4	7598907	7698907	0.40095	LOC102186385
4	7598907	7698907	0.40095	LOC106501766
4	7598948	7698948	0.400929	LOC102186105
4	7598948	7698948	0.400929	LOC102186385
4	7598948	7698948	0.400929	LOC106501766
4	7598970	7698970	0.400929	LOC102186105
4	7598970	7698970	0.400929	LOC102186385
4	7598970	7698970	0.400929	LOC106501766
4	7599004	7699004	0.400929	LOC102186105
4	7599004	7699004	0.400929	LOC102186385
4	7599004	7699004	0.400929	LOC106501766
4	7599022	7699022	0.402567	LOC102186105
4	7599022	7699022	0.402567	LOC102186385
4	7599022	7699022	0.402567	LOC106501766
4	7599055	7699055	0.399909	LOC102186105
4	7599055	7699055	0.399909	LOC102186385
4	7599055	7699055	0.399909	LOC106501766
4	7599095	7699095	0.399569	LOC102186105
4	7599095	7699095	0.399569	LOC102186385
4	7599095	7699095	0.399569	LOC106501766
4	7599149	7699149	0.400495	LOC102186105
4	7599149	7699149	0.400495	LOC102186385
4	7599149	7699149	0.400495	LOC106501766
4	7599163	7699163	0.400495	LOC102186105
4	7599163	7699163	0.400495	LOC102186385
4	7599163	7699163	0.400495	LOC106501766
4	7599231	7699231	0.400026	LOC102186105
4	7599231	7699231	0.400026	LOC102186385
4	7599231	7699231	0.400026	LOC106501766
4	7599344	7699344	0.400026	LOC102186105
4	7599344	7699344	0.400026	LOC102186385
4	7599344	7699344	0.400026	LOC106501766
4	7599349	7699349	0.409531	LOC102186105
4	7599349	7699349	0.409531	LOC102186385
4	7599349	7699349	0.409531	LOC106501766
4	7599397	7699397	0.399995	LOC102186105
4	7599397	7699397	0.399995	LOC102186385
4	7599397	7699397	0.399995	LOC106501766
4	7599427	7699427	0.399995	LOC102186105

4	7599427	7699427	0.399995	LOC102186385
4	7599427	7699427	0.399995	LOC106501766
4	7599435	7699435	0.400625	LOC102186105
4	7599435	7699435	0.400625	LOC102186385
4	7599435	7699435	0.400625	LOC106501766
4	7599470	7699470	0.401279	LOC102186105
4	7599470	7699470	0.401279	LOC102186385
4	7599470	7699470	0.401279	LOC106501766
4	7599550	7699550	0.400185	LOC102186105
4	7599550	7699550	0.400185	LOC102186385
4	7599550	7699550	0.400185	LOC106501766
4	7599628	7699628	0.400175	LOC102186105
4	7599628	7699628	0.400175	LOC102186385
4	7599628	7699628	0.400175	LOC106501766
4	7599709	7699709	0.40012	LOC102186105
4	7599709	7699709	0.40012	LOC102186385
4	7599709	7699709	0.40012	LOC106501766
4	7599809	7699809	0.400926	LOC102186105
4	7599809	7699809	0.400926	LOC102186385
4	7599809	7699809	0.400926	LOC106501766
4	7599831	7699831	0.401125	LOC102186105
4	7599831	7699831	0.401125	LOC102186385
4	7599831	7699831	0.401125	LOC106501766
4	7599840	7699840	0.401087	LOC102186105
4	7599840	7699840	0.401087	LOC102186385
4	7599840	7699840	0.401087	LOC106501766
4	7599908	7699908	0.401288	LOC102186105
4	7599908	7699908	0.401288	LOC102186385
4	7599908	7699908	0.401288	LOC106501766
4	7599942	7699942	0.401288	LOC102186105
4	7599942	7699942	0.401288	LOC102186385
4	7599942	7699942	0.401288	LOC106501766
4	7599949	7699949	0.401288	LOC102186105
4	7599949	7699949	0.401288	LOC102186385
4	7599949	7699949	0.401288	LOC106501766
4	7599977	7699977	0.401288	LOC102186105
4	7599977	7699977	0.401288	LOC102186385
4	7599977	7699977	0.401288	LOC106501766
4	7600006	7700006	0.401494	LOC102186105
4	7600006	7700006	0.401494	LOC102186385
4	7600006	7700006	0.401494	LOC106501766
4	7600048	7700048	0.40065	LOC102186105
4	7600048	7700048	0.40065	LOC102186385
4	7600048	7700048	0.40065	LOC106501766
4	7600199	7700199	0.40065	LOC102186105
4	7600199	7700199	0.40065	LOC102186385
4	7600199	7700199	0.40065	LOC106501766
4	7600209	7700209	0.40065	LOC102186105
4	7600209	7700209	0.40065	LOC102186385
4	7600209	7700209	0.40065	LOC106501766
4	7600533	7700533	0.401127	LOC102186105
4	7600533	7700533	0.401127	LOC102186385
4	7600533	7700533	0.401127	LOC106501766
4	7600695	7700695	0.399963	LOC102186105
4	7600695	7700695	0.399963	LOC102186385
4	7600695	7700695	0.399963	LOC106501766
4	7600729	7700729	0.399744	LOC102186105
4	7600729	7700729	0.399744	LOC102186385

4	7600729	7700729	0.399744	LOC106501766
4	7600789	7700789	0.399744	LOC102186105
4	7600789	7700789	0.399744	LOC102186385
4	7600789	7700789	0.399744	LOC106501766
4	7600814	7700814	0.399744	LOC102186105
4	7600814	7700814	0.399744	LOC102186385
4	7600814	7700814	0.399744	LOC106501766
4	7600937	7700937	0.400296	LOC102186105
4	7600937	7700937	0.400296	LOC102186385
4	7600937	7700937	0.400296	LOC106501766
4	7600945	7700945	0.400238	LOC102186105
4	7600945	7700945	0.400238	LOC102186385
4	7600945	7700945	0.400238	LOC106501766
4	7600958	7700958	0.400238	LOC102186105
4	7600958	7700958	0.400238	LOC102186385
4	7600958	7700958	0.400238	LOC106501766
4	7600967	7700967	0.402465	LOC102186105
4	7600967	7700967	0.402465	LOC102186385
4	7600967	7700967	0.402465	LOC106501766
4	7601002	7701002	0.402498	LOC102186105
4	7601002	7701002	0.402498	LOC102186385
4	7601002	7701002	0.402498	LOC106501766
4	7601025	7701025	0.402498	LOC102186105
4	7601025	7701025	0.402498	LOC102186385
4	7601025	7701025	0.402498	LOC106501766
4	7601026	7701026	0.402498	LOC102186105
4	7601026	7701026	0.402498	LOC102186385
4	7601026	7701026	0.402498	LOC106501766
4	7601073	7701073	0.402888	LOC102186105
4	7601073	7701073	0.402888	LOC102186385
4	7601073	7701073	0.402888	LOC106501766
4	7601076	7701076	0.402782	LOC102186105
4	7601076	7701076	0.402782	LOC102186385
4	7601076	7701076	0.402782	LOC106501766
4	7601114	7701114	0.402779	LOC102186105
4	7601114	7701114	0.402779	LOC102186385
4	7601114	7701114	0.402779	LOC106501766
4	7601196	7701196	0.402802	LOC102186105
4	7601196	7701196	0.402802	LOC102186385
4	7601196	7701196	0.402802	LOC106501766
4	7601221	7701221	0.400917	LOC102186105
4	7601221	7701221	0.400917	LOC102186385
4	7601221	7701221	0.400917	LOC106501766
4	7601222	7701222	0.400746	LOC102186105
4	7601222	7701222	0.400746	LOC102186385
4	7601222	7701222	0.400746	LOC106501766
4	7601314	7701314	0.397707	LOC102186105
4	7601314	7701314	0.397707	LOC102186385
4	7601314	7701314	0.397707	LOC106501766
4	7601385	7701385	0.397707	LOC102186105
4	7601385	7701385	0.397707	LOC102186385
4	7601385	7701385	0.397707	LOC106501766
4	7601404	7701404	0.397707	LOC102186105
4	7601404	7701404	0.397707	LOC102186385
4	7601404	7701404	0.397707	LOC106501766
4	7601469	7701469	0.397732	LOC102186105
4	7601469	7701469	0.397732	LOC102186385
4	7601469	7701469	0.397732	LOC106501766

4	7601632	7701632	0.39752	LOC102186105
4	7601632	7701632	0.39752	LOC102186385
4	7601632	7701632	0.39752	LOC106501766
4	7601655	7701655	0.397931	LOC102186105
4	7601655	7701655	0.397931	LOC102186385
4	7601655	7701655	0.397931	LOC106501766
4	7601660	7701660	0.397931	LOC102186105
4	7601660	7701660	0.397931	LOC102186385
4	7601660	7701660	0.397931	LOC106501766
4	7601707	7701707	0.397085	LOC102186105
4	7601707	7701707	0.397085	LOC102186385
4	7601707	7701707	0.397085	LOC106501766
4	7601834	7701834	0.39727	LOC102186105
4	7601834	7701834	0.39727	LOC102186385
4	7601834	7701834	0.39727	LOC106501766
4	7601880	7701880	0.397472	LOC102186105
4	7601880	7701880	0.397472	LOC102186385
4	7601880	7701880	0.397472	LOC106501766
4	7601916	7701916	0.387156	LOC102186105
4	7601916	7701916	0.387156	LOC102186385
4	7601916	7701916	0.387156	LOC106501766
4	7601972	7701972	0.393377	LOC102186105
4	7601972	7701972	0.393377	LOC102186385
4	7601972	7701972	0.393377	LOC106501766
4	7602007	7702007	0.393873	LOC102186105
4	7602007	7702007	0.393873	LOC102186385
4	7602007	7702007	0.393873	LOC106501766
4	7602043	7702043	0.394475	LOC102186105
4	7602043	7702043	0.394475	LOC102186385
4	7602043	7702043	0.394475	LOC106501766
4	7602106	7702106	0.391006	LOC102186105
4	7602106	7702106	0.391006	LOC102186385
4	7602106	7702106	0.391006	LOC106501766
4	7602145	7702145	0.391768	LOC102186105
4	7602145	7702145	0.391768	LOC102186385
4	7602145	7702145	0.391768	LOC106501766
4	7602325	7702325	0.391768	LOC102186105
4	7602325	7702325	0.391768	LOC102186385
4	7602325	7702325	0.391768	LOC106501766
4	7602431	7702431	0.392255	LOC102186105
4	7602431	7702431	0.392255	LOC102186385
4	7602431	7702431	0.392255	LOC106501766
4	7602437	7702437	0.392255	LOC102186105
4	7602437	7702437	0.392255	LOC102186385
4	7602437	7702437	0.392255	LOC106501766
4	7602438	7702438	0.392255	LOC102186105
4	7602438	7702438	0.392255	LOC102186385
4	7602438	7702438	0.392255	LOC106501766
4	7602534	7702534	0.391701	LOC102186105
4	7602534	7702534	0.391701	LOC102186385
4	7602534	7702534	0.391701	LOC106501766
4	7602568	7702568	0.393261	LOC102186105
4	7602568	7702568	0.393261	LOC102186385
4	7602568	7702568	0.393261	LOC106501766
4	7602656	7702656	0.389757	LOC102186105
4	7602656	7702656	0.389757	LOC102186385
4	7602656	7702656	0.389757	LOC106501766
4	7602683	7702683	0.389757	LOC102186105

4	7602683	7702683	0.389757	LOC102186385
4	7602683	7702683	0.389757	LOC106501766
4	7602737	7702737	0.389757	LOC102186105
4	7602737	7702737	0.389757	LOC102186385
4	7602737	7702737	0.389757	LOC106501766
4	7602800	7702800	0.389757	LOC102186105
4	7602800	7702800	0.389757	LOC102186385
4	7602800	7702800	0.389757	LOC106501766
4	7602873	7702873	0.389757	LOC102186105
4	7602873	7702873	0.389757	LOC102186385
4	7602873	7702873	0.389757	LOC106501766
4	7603120	7703120	0.389757	LOC102186105
4	7603120	7703120	0.389757	LOC102186385
4	7603120	7703120	0.389757	LOC106501766
4	7603207	7703207	0.382494	LOC102186105
4	7603207	7703207	0.382494	LOC102186385
4	7603207	7703207	0.382494	LOC106501766
4	7603208	7703208	0.382494	LOC102186105
4	7603208	7703208	0.382494	LOC102186385
4	7603208	7703208	0.382494	LOC106501766
4	7603213	7703213	0.362387	LOC102186105
4	7603213	7703213	0.362387	LOC102186385
4	7603213	7703213	0.362387	LOC106501766
4	7603228	7703228	0.376014	LOC102186105
4	7603228	7703228	0.376014	LOC102186385
4	7603228	7703228	0.376014	LOC106501766
4	7603256	7703256	0.37512	LOC102186105
4	7603256	7703256	0.37512	LOC102186385
4	7603256	7703256	0.37512	LOC106501766
4	7603261	7703261	0.37512	LOC102186105
4	7603261	7703261	0.37512	LOC102186385
4	7603261	7703261	0.37512	LOC106501766
4	7603262	7703262	0.37512	LOC102186105
4	7603262	7703262	0.37512	LOC102186385
4	7603262	7703262	0.37512	LOC106501766
4	7603412	7703412	0.374547	LOC102186105
4	7603412	7703412	0.374547	LOC102186385
4	7603412	7703412	0.374547	LOC106501766
4	7603453	7703453	0.374547	LOC102186105
4	7603453	7703453	0.374547	LOC102186385
4	7603453	7703453	0.374547	LOC106501766
4	7603482	7703482	0.374547	LOC102186105
4	7603482	7703482	0.374547	LOC102186385
4	7603482	7703482	0.374547	LOC106501766
4	7603484	7703484	0.374547	LOC102186105
4	7603484	7703484	0.374547	LOC102186385
4	7603484	7703484	0.374547	LOC106501766
4	7603492	7703492	0.377438	LOC102186105
4	7603492	7703492	0.377438	LOC102186385
4	7603492	7703492	0.377438	LOC106501766
4	7603515	7703515	0.376937	LOC102186105
4	7603515	7703515	0.376937	LOC102186385
4	7603515	7703515	0.376937	LOC106501766
4	7603516	7703516	0.376937	LOC102186105
4	7603516	7703516	0.376937	LOC102186385
4	7603516	7703516	0.376937	LOC106501766
4	7603592	7703592	0.377561	LOC102186105
4	7603592	7703592	0.377561	LOC102186385

4	7603592	7703592	0.377561	LOC106501766
4	7603598	7703598	0.377561	LOC102186105
4	7603598	7703598	0.377561	LOC102186385
4	7603598	7703598	0.377561	LOC106501766
4	7603623	7703623	0.377438	LOC102186105
4	7603623	7703623	0.377438	LOC102186385
4	7603623	7703623	0.377438	LOC106501766
4	7603657	7703657	0.378994	LOC102186105
4	7603657	7703657	0.378994	LOC102186385
4	7603657	7703657	0.378994	LOC106501766
4	7604056	7704056	0.379778	LOC102186105
4	7604056	7704056	0.379778	LOC102186385
4	7604056	7704056	0.379778	LOC106501766
4	7604245	7704245	0.373479	LOC102186105
4	7604245	7704245	0.373479	LOC102186385
4	7604245	7704245	0.373479	LOC106501766
4	7604281	7704281	0.373447	LOC102186105
4	7604281	7704281	0.373447	LOC102186385
4	7604281	7704281	0.373447	LOC106501766
4	7604366	7704366	0.373447	LOC102186105
4	7604366	7704366	0.373447	LOC102186385
4	7604366	7704366	0.373447	LOC106501766
4	7604453	7704453	0.373447	LOC102186105
4	7604453	7704453	0.373447	LOC102186385
4	7604453	7704453	0.373447	LOC106501766
4	7604472	7704472	0.373447	LOC102186105
4	7604472	7704472	0.373447	LOC102186385
4	7604472	7704472	0.373447	LOC106501766
4	7604549	7704549	0.37426	LOC102186105
4	7604549	7704549	0.37426	LOC102186385
4	7604549	7704549	0.37426	LOC106501766
4	7604550	7704550	0.37426	LOC102186105
4	7604550	7704550	0.37426	LOC102186385
4	7604550	7704550	0.37426	LOC106501766
4	7604555	7704555	0.37426	LOC102186105
4	7604555	7704555	0.37426	LOC102186385
4	7604555	7704555	0.37426	LOC106501766
4	7604709	7704709	0.37426	LOC102186105
4	7604709	7704709	0.37426	LOC102186385
4	7604709	7704709	0.37426	LOC106501766
4	7604739	7704739	0.372943	LOC102186105
4	7604739	7704739	0.372943	LOC102186385
4	7604739	7704739	0.372943	LOC106501766
4	7604782	7704782	0.374098	LOC102186105
4	7604782	7704782	0.374098	LOC102186385
4	7604782	7704782	0.374098	LOC106501766
4	7604818	7704818	0.373012	LOC102186105
4	7604818	7704818	0.373012	LOC102186385
4	7604818	7704818	0.373012	LOC106501766
4	7604836	7704836	0.373012	LOC102186105
4	7604836	7704836	0.373012	LOC102186385
4	7604836	7704836	0.373012	LOC106501766
4	7604839	7704839	0.373012	LOC102186105
4	7604839	7704839	0.373012	LOC102186385
4	7604839	7704839	0.373012	LOC106501766
4	7604873	7704873	0.373012	LOC102186105
4	7604873	7704873	0.373012	LOC102186385
4	7604873	7704873	0.373012	LOC106501766

4	7604896	7704896	0.373012	LOC102186105
4	7604896	7704896	0.373012	LOC102186385
4	7604896	7704896	0.373012	LOC106501766
4	7604918	7704918	0.373051	LOC102186105
4	7604918	7704918	0.373051	LOC102186385
4	7604918	7704918	0.373051	LOC106501766
4	7604923	7704923	0.373051	LOC102186105
4	7604923	7704923	0.373051	LOC102186385
4	7604923	7704923	0.373051	LOC106501766
4	7604961	7704961	0.373051	LOC102186105
4	7604961	7704961	0.373051	LOC102186385
4	7604961	7704961	0.373051	LOC106501766
4	7604975	7704975	0.373058	LOC102186105
4	7604975	7704975	0.373058	LOC102186385
4	7604975	7704975	0.373058	LOC106501766
4	7604979	7704979	0.373052	LOC102186105
4	7604979	7704979	0.373052	LOC102186385
4	7604979	7704979	0.373052	LOC106501766
4	7605007	7705007	0.372794	LOC102186105
4	7605007	7705007	0.372794	LOC102186385
4	7605007	7705007	0.372794	LOC106501766
4	7605011	7705011	0.375763	LOC102186105
4	7605011	7705011	0.375763	LOC102186385
4	7605011	7705011	0.375763	LOC106501766
4	7605022	7705022	0.375901	LOC102186105
4	7605022	7705022	0.375901	LOC102186385
4	7605022	7705022	0.375901	LOC106501766
4	7605025	7705025	0.375901	LOC102186105
4	7605025	7705025	0.375901	LOC102186385
4	7605025	7705025	0.375901	LOC106501766
4	7605038	7705038	0.376641	LOC102186105
4	7605038	7705038	0.376641	LOC102186385
4	7605038	7705038	0.376641	LOC106501766
4	7605211	7705211	0.374698	LOC102186105
4	7605211	7705211	0.374698	LOC102186385
4	7605211	7705211	0.374698	LOC106501766
4	7605236	7705236	0.374997	LOC102186105
4	7605236	7705236	0.374997	LOC102186385
4	7605236	7705236	0.374997	LOC106501766
4	7605309	7705309	0.374978	LOC102186105
4	7605309	7705309	0.374978	LOC102186385
4	7605309	7705309	0.374978	LOC106501766
4	7605318	7705318	0.374923	LOC102186105
4	7605318	7705318	0.374923	LOC102186385
4	7605318	7705318	0.374923	LOC106501766
4	7605413	7705413	0.369576	LOC102186105
4	7605413	7705413	0.369576	LOC102186385
4	7605413	7705413	0.369576	LOC106501766
4	7605436	7705436	0.369576	LOC102186105
4	7605436	7705436	0.369576	LOC102186385
4	7605436	7705436	0.369576	LOC106501766
4	7605447	7705447	0.369576	LOC102186105
4	7605447	7705447	0.369576	LOC102186385
4	7605447	7705447	0.369576	LOC106501766
4	7605462	7705462	0.369576	LOC102186105
4	7605462	7705462	0.369576	LOC102186385
4	7605462	7705462	0.369576	LOC106501766
4	7605466	7705466	0.369576	LOC102186105

4	7605466	7705466	0.369576	LOC102186385
4	7605466	7705466	0.369576	LOC106501766
4	7605525	7705525	0.369576	LOC102186105
4	7605525	7705525	0.369576	LOC102186385
4	7605525	7705525	0.369576	LOC106501766
4	7605557	7705557	0.369576	LOC102186105
4	7605557	7705557	0.369576	LOC102186385
4	7605557	7705557	0.369576	LOC106501766
4	7605561	7705561	0.369576	LOC102186105
4	7605561	7705561	0.369576	LOC102186385
4	7605561	7705561	0.369576	LOC106501766
4	7605692	7705692	0.369576	LOC102186105
4	7605692	7705692	0.369576	LOC102186385
4	7605692	7705692	0.369576	LOC106501766
4	7605734	7705734	0.369576	LOC102186105
4	7605734	7705734	0.369576	LOC102186385
4	7605734	7705734	0.369576	LOC106501766
4	7605768	7705768	0.370576	LOC102186105
4	7605768	7705768	0.370576	LOC102186385
4	7605768	7705768	0.370576	LOC106501766
4	7605900	7705900	0.369477	LOC102186105
4	7605900	7705900	0.369477	LOC102186385
4	7605900	7705900	0.369477	LOC106501766
4	7605905	7705905	0.369477	LOC102186105
4	7605905	7705905	0.369477	LOC102186385
4	7605905	7705905	0.369477	LOC106501766
4	7605920	7705920	0.369477	LOC102186105
4	7605920	7705920	0.369477	LOC102186385
4	7605920	7705920	0.369477	LOC106501766
4	7605940	7705940	0.369477	LOC102186105
4	7605940	7705940	0.369477	LOC102186385
4	7605940	7705940	0.369477	LOC106501766
4	7605957	7705957	0.369477	LOC102186105
4	7605957	7705957	0.369477	LOC102186385
4	7605957	7705957	0.369477	LOC106501766
4	7605974	7705974	0.369477	LOC102186105
4	7605974	7705974	0.369477	LOC102186385
4	7605974	7705974	0.369477	LOC106501766
4	7606000	7706000	0.369477	LOC102186105
4	7606000	7706000	0.369477	LOC102186385
4	7606000	7706000	0.369477	LOC106501766
4	7606009	7706009	0.369477	LOC102186105
4	7606009	7706009	0.369477	LOC102186385
4	7606009	7706009	0.369477	LOC106501766
4	7606031	7706031	0.369477	LOC102186105
4	7606031	7706031	0.369477	LOC102186385
4	7606031	7706031	0.369477	LOC106501766
4	7606066	7706066	0.369477	LOC102186105
4	7606066	7706066	0.369477	LOC102186385
4	7606066	7706066	0.369477	LOC106501766
4	7606067	7706067	0.369477	LOC102186105
4	7606067	7706067	0.369477	LOC102186385
4	7606067	7706067	0.369477	LOC106501766
4	7606073	7706073	0.369477	LOC102186105
4	7606073	7706073	0.369477	LOC102186385
4	7606073	7706073	0.369477	LOC106501766
4	7606121	7706121	0.369477	LOC102186105
4	7606121	7706121	0.369477	LOC102186385

4	7606121	7706121	0.369477	LOC106501766
4	7606137	7706137	0.369477	LOC102186105
4	7606137	7706137	0.369477	LOC102186385
4	7606137	7706137	0.369477	LOC106501766
4	7606262	7706262	0.369477	LOC102186105
4	7606262	7706262	0.369477	LOC102186385
4	7606262	7706262	0.369477	LOC106501766
4	7606281	7706281	0.369538	LOC102186105
4	7606281	7706281	0.369538	LOC102186385
4	7606281	7706281	0.369538	LOC106501766
4	7606438	7706438	0.369898	LOC102186105
4	7606438	7706438	0.369898	LOC102186385
4	7606438	7706438	0.369898	LOC106501766
4	7606555	7706555	0.370262	LOC102186105
4	7606555	7706555	0.370262	LOC102186385
4	7606555	7706555	0.370262	LOC106501766
4	7606558	7706558	0.370558	LOC102186105
4	7606558	7706558	0.370558	LOC102186385
4	7606558	7706558	0.370558	LOC106501766
4	7606562	7706562	0.370047	LOC102186105
4	7606562	7706562	0.370047	LOC102186385
4	7606562	7706562	0.370047	LOC106501766
4	7606592	7706592	0.369971	LOC102186105
4	7606592	7706592	0.369971	LOC102186385
4	7606592	7706592	0.369971	LOC106501766
4	7606612	7706612	0.369531	LOC102186105
4	7606612	7706612	0.369531	LOC102186385
4	7606612	7706612	0.369531	LOC106501766
4	7606641	7706641	0.369926	LOC102186105
4	7606641	7706641	0.369926	LOC102186385
4	7606641	7706641	0.369926	LOC106501766
4	7606642	7706642	0.369926	LOC102186105
4	7606642	7706642	0.369926	LOC102186385
4	7606642	7706642	0.369926	LOC106501766
4	7606710	7706710	0.37026	LOC102186105
4	7606710	7706710	0.37026	LOC102186385
4	7606710	7706710	0.37026	LOC106501766
4	7606713	7706713	0.370182	LOC102186105
4	7606713	7706713	0.370182	LOC102186385
4	7606713	7706713	0.370182	LOC106501766
4	7606753	7706753	0.370942	LOC102186105
4	7606753	7706753	0.370942	LOC102186385
4	7606753	7706753	0.370942	LOC106501766
4	7606754	7706754	0.370942	LOC102186105
4	7606754	7706754	0.370942	LOC102186385
4	7606754	7706754	0.370942	LOC106501766
4	7606758	7706758	0.369744	LOC102186105
4	7606758	7706758	0.369744	LOC102186385
4	7606758	7706758	0.369744	LOC106501766
4	7606809	7706809	0.369372	LOC102186105
4	7606809	7706809	0.369372	LOC102186385
4	7606809	7706809	0.369372	LOC106501766
4	7606818	7706818	0.36989	LOC102186105
4	7606818	7706818	0.36989	LOC102186385
4	7606818	7706818	0.36989	LOC106501766
4	7606834	7706834	0.369562	LOC102186105
4	7606834	7706834	0.369562	LOC102186385
4	7606834	7706834	0.369562	LOC106501766

4	7606847	7706847	0.369562	LOC102186105
4	7606847	7706847	0.369562	LOC102186385
4	7606847	7706847	0.369562	LOC106501766
4	7606856	7706856	0.369562	LOC102186105
4	7606856	7706856	0.369562	LOC102186385
4	7606856	7706856	0.369562	LOC106501766
4	7606867	7706867	0.369562	LOC102186105
4	7606867	7706867	0.369562	LOC102186385
4	7606867	7706867	0.369562	LOC106501766
4	7606872	7706872	0.369562	LOC102186105
4	7606872	7706872	0.369562	LOC102186385
4	7606872	7706872	0.369562	LOC106501766
4	7606882	7706882	0.369562	LOC102186105
4	7606882	7706882	0.369562	LOC102186385
4	7606882	7706882	0.369562	LOC106501766
4	7606920	7706920	0.37002	LOC102186105
4	7606920	7706920	0.37002	LOC102186385
4	7606920	7706920	0.37002	LOC106501766
4	7606925	7706925	0.369535	LOC102186105
4	7606925	7706925	0.369535	LOC102186385
4	7606925	7706925	0.369535	LOC106501766
4	7606945	7706945	0.369123	LOC102186105
4	7606945	7706945	0.369123	LOC102186385
4	7606945	7706945	0.369123	LOC106501766
4	7606957	7706957	0.369405	LOC102186105
4	7606957	7706957	0.369405	LOC102186385
4	7606957	7706957	0.369405	LOC106501766
4	7606976	7706976	0.369405	LOC102186105
4	7606976	7706976	0.369405	LOC102186385
4	7606976	7706976	0.369405	LOC106501766
4	7606985	7706985	0.368713	LOC102186105
4	7606985	7706985	0.368713	LOC102186385
4	7606985	7706985	0.368713	LOC106501766
4	7606986	7706986	0.368713	LOC102186105
4	7606986	7706986	0.368713	LOC102186385
4	7606986	7706986	0.368713	LOC106501766
4	7607004	7707004	0.369993	LOC102186105
4	7607004	7707004	0.369993	LOC102186385
4	7607004	7707004	0.369993	LOC106501766
4	7607007	7707007	0.369993	LOC102186105
4	7607007	7707007	0.369993	LOC102186385
4	7607007	7707007	0.369993	LOC106501766
4	7607016	7707016	0.371261	LOC102186105
4	7607016	7707016	0.371261	LOC102186385
4	7607016	7707016	0.371261	LOC106501766
4	7607019	7707019	0.367286	LOC102186105
4	7607019	7707019	0.367286	LOC102186385
4	7607019	7707019	0.367286	LOC106501766
4	7611905	7711905	0.380444	LOC102186105
4	7611905	7711905	0.380444	LOC102186385
4	7611905	7711905	0.380444	LOC106501766
4	7611972	7711972	0.378886	LOC102186105
4	7611972	7711972	0.378886	LOC102186385
4	7611972	7711972	0.378886	LOC106501766
4	7612155	7712155	0.374574	LOC102186105
4	7612155	7712155	0.374574	LOC102186385
4	7612155	7712155	0.374574	LOC106501766
4	7612159	7712159	0.374574	LOC102186105

4	7612159	7712159	0.374574	LOC102186385
4	7612159	7712159	0.374574	LOC106501766
4	7612163	7712163	0.374811	LOC102186105
4	7612163	7712163	0.374811	LOC102186385
4	7612163	7712163	0.374811	LOC106501766
4	7612168	7712168	0.369056	LOC102186105
4	7612168	7712168	0.369056	LOC102186385
4	7612168	7712168	0.369056	LOC106501766
4	7612200	7712200	0.363831	LOC102186105
4	7612200	7712200	0.363831	LOC102186385
4	7612200	7712200	0.363831	LOC106501766
4	7612214	7712214	0.363831	LOC102186105
4	7612214	7712214	0.363831	LOC102186385
4	7612214	7712214	0.363831	LOC106501766
4	7612225	7712225	0.363831	LOC102186105
4	7612225	7712225	0.363831	LOC102186385
4	7612225	7712225	0.363831	LOC106501766
4	7612254	7712254	0.363831	LOC102186105
4	7612254	7712254	0.363831	LOC102186385
4	7612254	7712254	0.363831	LOC106501766
4	7612292	7712292	0.363831	LOC102186105
4	7612292	7712292	0.363831	LOC102186385
4	7612292	7712292	0.363831	LOC106501766
4	7612293	7712293	0.363831	LOC102186105
4	7612293	7712293	0.363831	LOC102186385
4	7612293	7712293	0.363831	LOC106501766
4	7612338	7712338	0.363831	LOC102186105
4	7612338	7712338	0.363831	LOC102186385
4	7612338	7712338	0.363831	LOC106501766
4	7612440	7712440	0.363831	LOC102186105
4	7612440	7712440	0.363831	LOC102186385
4	7612440	7712440	0.363831	LOC106501766
4	7612457	7712457	0.363831	LOC102186105
4	7612457	7712457	0.363831	LOC102186385
4	7612457	7712457	0.363831	LOC106501766
4	7612474	7712474	0.363831	LOC102186105
4	7612474	7712474	0.363831	LOC102186385
4	7612474	7712474	0.363831	LOC106501766
4	7612496	7712496	0.363831	LOC102186105
4	7612496	7712496	0.363831	LOC102186385
4	7612496	7712496	0.363831	LOC106501766
4	7612520	7712520	0.363831	LOC102186105
4	7612520	7712520	0.363831	LOC102186385
4	7612520	7712520	0.363831	LOC106501766
4	7612562	7712562	0.363831	LOC102186105
4	7612562	7712562	0.363831	LOC102186385
4	7612562	7712562	0.363831	LOC106501766
4	7612578	7712578	0.363831	LOC102186105
4	7612578	7712578	0.363831	LOC102186385
4	7612578	7712578	0.363831	LOC106501766
4	7612580	7712580	0.363899	LOC102186105
4	7612580	7712580	0.363899	LOC102186385
4	7612580	7712580	0.363899	LOC106501766
4	7612605	7712605	0.363698	LOC102186105
4	7612605	7712605	0.363698	LOC102186385
4	7612605	7712605	0.363698	LOC106501766
4	7612872	7712872	0.36786	LOC102186105
4	7612872	7712872	0.36786	LOC102186385

4	7612872	7712872	0.36786	LOC106501766
4	7612888	7712888	0.364801	LOC102186105
4	7612888	7712888	0.364801	LOC102186385
4	7612888	7712888	0.364801	LOC106501766
4	7612939	7712939	0.364887	LOC102186105
4	7612939	7712939	0.364887	LOC102186385
4	7612939	7712939	0.364887	LOC106501766
4	7612946	7712946	0.354847	LOC102186105
4	7612946	7712946	0.354847	LOC102186385
4	7612946	7712946	0.354847	LOC106501766
4	7612947	7712947	0.354847	LOC102186105
4	7612947	7712947	0.354847	LOC102186385
4	7612947	7712947	0.354847	LOC106501766
4	7612972	7712972	0.354847	LOC102186105
4	7612972	7712972	0.354847	LOC102186385
4	7612972	7712972	0.354847	LOC106501766
4	7613012	7713012	0.354847	LOC102186105
4	7613012	7713012	0.354847	LOC102186385
4	7613012	7713012	0.354847	LOC106501766
4	7613040	7713040	0.354847	LOC102186105
4	7613040	7713040	0.354847	LOC102186385
4	7613040	7713040	0.354847	LOC106501766
4	7613090	7713090	0.354938	LOC102186105
4	7613090	7713090	0.354938	LOC102186385
4	7613090	7713090	0.354938	LOC106501766
4	7613091	7713091	0.354938	LOC102186105
4	7613091	7713091	0.354938	LOC102186385
4	7613091	7713091	0.354938	LOC106501766
4	7613152	7713152	0.354938	LOC102186105
4	7613152	7713152	0.354938	LOC102186385
4	7613152	7713152	0.354938	LOC106501766
4	7613161	7713161	0.354938	LOC102186105
4	7613161	7713161	0.354938	LOC102186385
4	7613161	7713161	0.354938	LOC106501766
4	7613177	7713177	0.354938	LOC102186105
4	7613177	7713177	0.354938	LOC102186385
4	7613177	7713177	0.354938	LOC106501766
4	7613188	7713188	0.354938	LOC102186105
4	7613188	7713188	0.354938	LOC102186385
4	7613188	7713188	0.354938	LOC106501766
4	7613195	7713195	0.354938	LOC102186105
4	7613195	7713195	0.354938	LOC102186385
4	7613195	7713195	0.354938	LOC106501766
4	7613366	7713366	0.357753	LOC102186105
4	7613366	7713366	0.357753	LOC102186385
4	7613366	7713366	0.357753	LOC106501766
4	7613369	7713369	0.355255	LOC102186105
4	7613369	7713369	0.355255	LOC102186385
4	7613369	7713369	0.355255	LOC106501766
4	7613467	7713467	0.355255	LOC102186105
4	7613467	7713467	0.355255	LOC102186385
4	7613467	7713467	0.355255	LOC106501766
4	7613474	7713474	0.355255	LOC102186105
4	7613474	7713474	0.355255	LOC102186385
4	7613474	7713474	0.355255	LOC106501766
4	7613556	7713556	0.355255	LOC102186105
4	7613556	7713556	0.355255	LOC102186385
4	7613556	7713556	0.355255	LOC106501766

4	7613601	7713601	0.355255	LOC102186105
4	7613601	7713601	0.355255	LOC102186385
4	7613601	7713601	0.355255	LOC106501766
4	7613650	7713650	0.355255	LOC102186105
4	7613650	7713650	0.355255	LOC102186385
4	7613650	7713650	0.355255	LOC106501766
4	7613713	7713713	0.357859	LOC102186105
4	7613713	7713713	0.357859	LOC102186385
4	7613713	7713713	0.357859	LOC106501766
4	7613714	7713714	0.348714	LOC102186105
4	7613714	7713714	0.348714	LOC102186385
4	7613714	7713714	0.348714	LOC106501766
4	7613811	7713811	0.348714	LOC102186105
4	7613811	7713811	0.348714	LOC102186385
4	7613811	7713811	0.348714	LOC106501766
4	7613845	7713845	0.348714	LOC102186105
4	7613845	7713845	0.348714	LOC102186385
4	7613845	7713845	0.348714	LOC106501766
4	7614114	7714114	0.348714	LOC102186105
4	7614114	7714114	0.348714	LOC102186385
4	7614114	7714114	0.348714	LOC106501766
4	7614127	7714127	0.348714	LOC102186105
4	7614127	7714127	0.348714	LOC102186385
4	7614127	7714127	0.348714	LOC106501766
4	7614172	7714172	0.348714	LOC102186105
4	7614172	7714172	0.348714	LOC102186385
4	7614172	7714172	0.348714	LOC106501766
4	7614178	7714178	0.348714	LOC102186105
4	7614178	7714178	0.348714	LOC102186385
4	7614178	7714178	0.348714	LOC106501766
4	7614239	7714239	0.348714	LOC102186105
4	7614239	7714239	0.348714	LOC102186385
4	7614239	7714239	0.348714	LOC106501766
4	7614276	7714276	0.348714	LOC102186105
4	7614276	7714276	0.348714	LOC102186385
4	7614276	7714276	0.348714	LOC106501766
4	7614347	7714347	0.348714	LOC102186105
4	7614347	7714347	0.348714	LOC102186385
4	7614347	7714347	0.348714	LOC106501766
4	7614410	7714410	0.348714	LOC102186105
4	7614410	7714410	0.348714	LOC102186385
4	7614410	7714410	0.348714	LOC106501766
4	7614502	7714502	0.348714	LOC102186105
4	7614502	7714502	0.348714	LOC102186385
4	7614502	7714502	0.348714	LOC106501766
4	7614529	7714529	0.348714	LOC102186105
4	7614529	7714529	0.348714	LOC102186385
4	7614529	7714529	0.348714	LOC106501766
4	7614748	7714748	0.348714	LOC102186105
4	7614748	7714748	0.348714	LOC102186385
4	7614748	7714748	0.348714	LOC106501766
4	7614797	7714797	0.348714	LOC102186105
4	7614797	7714797	0.348714	LOC102186385
4	7614797	7714797	0.348714	LOC106501766
4	7614824	7714824	0.350509	LOC102186105
4	7614824	7714824	0.350509	LOC102186385
4	7614824	7714824	0.350509	LOC106501766
4	7614832	7714832	0.346371	LOC102186105

4	7614832	7714832	0.346371	LOC102186385
4	7614832	7714832	0.346371	LOC106501766
4	7614884	7714884	0.346371	LOC102186105
4	7614884	7714884	0.346371	LOC102186385
4	7614884	7714884	0.346371	LOC106501766
4	7614885	7714885	0.346371	LOC102186105
4	7614885	7714885	0.346371	LOC102186385
4	7614885	7714885	0.346371	LOC106501766
4	7614889	7714889	0.346371	LOC102186105
4	7614889	7714889	0.346371	LOC102186385
4	7614889	7714889	0.346371	LOC106501766
4	7614995	7714995	0.346371	LOC102186105
4	7614995	7714995	0.346371	LOC102186385
4	7614995	7714995	0.346371	LOC106501766
4	7615019	7715019	0.346371	LOC102186105
4	7615019	7715019	0.346371	LOC102186385
4	7615019	7715019	0.346371	LOC106501766
4	7615036	7715036	0.346371	LOC102186105
4	7615036	7715036	0.346371	LOC102186385
4	7615036	7715036	0.346371	LOC106501766
4	7615119	7715119	0.346371	LOC102186105
4	7615119	7715119	0.346371	LOC102186385
4	7615119	7715119	0.346371	LOC106501766
4	7615165	7715165	0.346371	LOC102186105
4	7615165	7715165	0.346371	LOC102186385
4	7615165	7715165	0.346371	LOC106501766
4	7615274	7715274	0.346371	LOC102186105
4	7615274	7715274	0.346371	LOC102186385
4	7615274	7715274	0.346371	LOC106501766
4	7615412	7715412	0.346322	LOC102186105
4	7615412	7715412	0.346322	LOC102186385
4	7615412	7715412	0.346322	LOC106501766
4	7615694	7715694	0.352868	LOC102186105
4	7615694	7715694	0.352868	LOC102186385
4	7615694	7715694	0.352868	LOC106501766
4	7615738	7715738	0.351663	LOC102186105
4	7615738	7715738	0.351663	LOC102186385
4	7615738	7715738	0.351663	LOC106501766
4	7615882	7715882	0.353155	LOC102186105
4	7615882	7715882	0.353155	LOC102186385
4	7615882	7715882	0.353155	LOC106501766
4	7615980	7715980	0.352903	LOC102186105
4	7615980	7715980	0.352903	LOC102186385
4	7615980	7715980	0.352903	LOC106501766
4	7616040	7716040	0.349584	LOC102186105
4	7616040	7716040	0.349584	LOC102186385
4	7616040	7716040	0.349584	LOC106501766
4	7616117	7716117	0.349584	LOC102186105
4	7616117	7716117	0.349584	LOC102186385
4	7616117	7716117	0.349584	LOC106501766
4	7616343	7716343	0.349584	LOC102186105
4	7616343	7716343	0.349584	LOC102186385
4	7616343	7716343	0.349584	LOC106501766
4	7616835	7716835	0.349584	LOC102186105
4	7616835	7716835	0.349584	LOC102186385
4	7616835	7716835	0.349584	LOC106501766
4	7617380	7717380	0.394855	LOC102186105
4	7617380	7717380	0.394855	LOC102186385

4	7617380	7717380	0.394855	LOC106501766
4	7617504	7717504	0.376701	LOC102186105
4	7617504	7717504	0.376701	LOC102186385
4	7617504	7717504	0.376701	LOC106501766
4	7617660	7717660	0.454929	LOC102186105
4	7617660	7717660	0.454929	LOC102186385
4	7617660	7717660	0.454929	LOC106501766
4	7617727	7717727	0.399953	LOC102186105
4	7617727	7717727	0.399953	LOC102186385
4	7617727	7717727	0.399953	LOC106501766
4	7617797	7717797	0.401118	LOC102186105
4	7617797	7717797	0.401118	LOC102186385
4	7617797	7717797	0.401118	LOC106501766
4	7617865	7717865	0.401856	LOC102186105
4	7617865	7717865	0.401856	LOC102186385
4	7617865	7717865	0.401856	LOC106501766
4	7617892	7717892	0.401856	LOC102186105
4	7617892	7717892	0.401856	LOC102186385
4	7617892	7717892	0.401856	LOC106501766
4	7617934	7717934	0.401856	LOC102186105
4	7617934	7717934	0.401856	LOC102186385
4	7617934	7717934	0.401856	LOC106501766
4	7618049	7718049	0.402128	LOC102186105
4	7618049	7718049	0.402128	LOC102186385
4	7618049	7718049	0.402128	LOC106501766
4	7618075	7718075	0.402057	LOC102186105
4	7618075	7718075	0.402057	LOC102186385
4	7618075	7718075	0.402057	LOC106501766
4	7618113	7718113	0.40189	LOC102186105
4	7618113	7718113	0.40189	LOC102186385
4	7618113	7718113	0.40189	LOC106501766
4	7618245	7718245	0.40568	LOC102186105
4	7618245	7718245	0.40568	LOC102186385
4	7618245	7718245	0.40568	LOC106501766
4	7618266	7718266	0.402509	LOC102186105
4	7618266	7718266	0.402509	LOC102186385
4	7618266	7718266	0.402509	LOC106501766
4	7618317	7718317	0.402509	LOC102186105
4	7618317	7718317	0.402509	LOC102186385
4	7618317	7718317	0.402509	LOC106501766
4	7618408	7718408	0.402509	LOC102186105
4	7618408	7718408	0.402509	LOC102186385
4	7618408	7718408	0.402509	LOC106501766
4	7618486	7718486	0.402509	LOC102186105
4	7618486	7718486	0.402509	LOC102186385
4	7618486	7718486	0.402509	LOC106501766
4	7618562	7718562	0.402896	LOC102186105
4	7618562	7718562	0.402896	LOC102186385
4	7618562	7718562	0.402896	LOC106501766
4	7618599	7718599	0.402975	LOC102186105
4	7618599	7718599	0.402975	LOC102186385
4	7618599	7718599	0.402975	LOC106501766
4	7618617	7718617	0.401626	LOC102186105
4	7618617	7718617	0.401626	LOC102186385
4	7618617	7718617	0.401626	LOC106501766
4	7618734	7718734	0.401626	LOC102186105
4	7618734	7718734	0.401626	LOC102186385
4	7618734	7718734	0.401626	LOC106501766

4	7618747	7718747	0.402685	LOC102186105
4	7618747	7718747	0.402685	LOC102186385
4	7618747	7718747	0.402685	LOC106501766
4	7618765	7718765	0.404366	LOC102186105
4	7618765	7718765	0.404366	LOC102186385
4	7618765	7718765	0.404366	LOC106501766
4	7618822	7718822	0.404357	LOC102186105
4	7618822	7718822	0.404357	LOC102186385
4	7618822	7718822	0.404357	LOC106501766
4	7618862	7718862	0.406062	LOC102186105
4	7618862	7718862	0.406062	LOC102186385
4	7618862	7718862	0.406062	LOC106501766
4	7618899	7718899	0.406025	LOC102186105
4	7618899	7718899	0.406025	LOC102186385
4	7618899	7718899	0.406025	LOC106501766
4	7619008	7719008	0.406025	LOC102186105
4	7619008	7719008	0.406025	LOC102186385
4	7619008	7719008	0.406025	LOC106501766
4	7619011	7719011	0.406128	LOC102186105
4	7619011	7719011	0.406128	LOC102186385
4	7619011	7719011	0.406128	LOC106501766
4	7619124	7719124	0.406116	LOC102186105
4	7619124	7719124	0.406116	LOC102186385
4	7619124	7719124	0.406116	LOC106501766
4	7619170	7719170	0.405748	LOC102186105
4	7619170	7719170	0.405748	LOC102186385
4	7619170	7719170	0.405748	LOC106501766
4	7619215	7719215	0.406603	LOC102186105
4	7619215	7719215	0.406603	LOC102186385
4	7619215	7719215	0.406603	LOC106501766
4	7619245	7719245	0.40663	LOC102186105
4	7619245	7719245	0.40663	LOC102186385
4	7619245	7719245	0.40663	LOC106501766
4	7619255	7719255	0.406306	LOC102186105
4	7619255	7719255	0.406306	LOC102186385
4	7619255	7719255	0.406306	LOC106501766
4	7619274	7719274	0.406306	LOC102186105
4	7619274	7719274	0.406306	LOC102186385
4	7619274	7719274	0.406306	LOC106501766
4	7619369	7719369	0.417416	LOC102186105
4	7619369	7719369	0.417416	LOC102186385
4	7619369	7719369	0.417416	LOC106501766
4	7619380	7719380	0.418001	LOC102186105
4	7619380	7719380	0.418001	LOC102186385
4	7619380	7719380	0.418001	LOC106501766
4	7619387	7719387	0.418001	LOC102186105
4	7619387	7719387	0.418001	LOC102186385
4	7619387	7719387	0.418001	LOC106501766
4	7619434	7719434	0.418001	LOC102186105
4	7619434	7719434	0.418001	LOC102186385
4	7619434	7719434	0.418001	LOC106501766
4	7619484	7719484	0.416069	LOC102186105
4	7619484	7719484	0.416069	LOC102186385
4	7619484	7719484	0.416069	LOC106501766
4	7619690	7719690	0.416679	LOC102186105
4	7619690	7719690	0.416679	LOC102186385
4	7619690	7719690	0.416679	LOC106501766
4	7619755	7719755	0.408843	LOC102186105

4	7619755	7719755	0.408843	LOC102186385
4	7619755	7719755	0.408843	LOC106501766
4	7619757	7719757	0.408843	LOC102186105
4	7619757	7719757	0.408843	LOC102186385
4	7619757	7719757	0.408843	LOC106501766
4	7619856	7719856	0.40885	LOC102186105
4	7619856	7719856	0.40885	LOC102186385
4	7619856	7719856	0.40885	LOC106501766
4	7619914	7719914	0.40885	LOC102186105
4	7619914	7719914	0.40885	LOC102186385
4	7619914	7719914	0.40885	LOC106501766
4	7620133	7720133	0.408625	LOC102186105
4	7620133	7720133	0.408625	LOC102186385
4	7620133	7720133	0.408625	LOC106501766
4	7620171	7720171	0.409598	LOC102186105
4	7620171	7720171	0.409598	LOC102186385
4	7620171	7720171	0.409598	LOC106501766
4	7620181	7720181	0.409183	LOC102186105
4	7620181	7720181	0.409183	LOC102186385
4	7620181	7720181	0.409183	LOC106501766
4	7620284	7720284	0.409183	LOC102186105
4	7620284	7720284	0.409183	LOC102186385
4	7620284	7720284	0.409183	LOC106501766
4	7620417	7720417	0.409183	LOC102186105
4	7620417	7720417	0.409183	LOC102186385
4	7620417	7720417	0.409183	LOC106501766
4	7620455	7720455	0.409384	LOC102186105
4	7620455	7720455	0.409384	LOC102186385
4	7620455	7720455	0.409384	LOC106501766
4	7620473	7720473	0.409632	LOC102186105
4	7620473	7720473	0.409632	LOC102186385
4	7620473	7720473	0.409632	LOC106501766
4	7620477	7720477	0.409414	LOC102186105
4	7620477	7720477	0.409414	LOC102186385
4	7620477	7720477	0.409414	LOC106501766
4	7620486	7720486	0.409414	LOC102186105
4	7620486	7720486	0.409414	LOC102186385
4	7620486	7720486	0.409414	LOC106501766
4	7620534	7720534	0.409414	LOC102186105
4	7620534	7720534	0.409414	LOC102186385
4	7620534	7720534	0.409414	LOC106501766
4	7620621	7720621	0.409091	LOC102186105
4	7620621	7720621	0.409091	LOC102186385
4	7620621	7720621	0.409091	LOC106501766
4	7620759	7720759	0.409311	LOC102186105
4	7620759	7720759	0.409311	LOC102186385
4	7620759	7720759	0.409311	LOC106501766
4	7620768	7720768	0.409311	LOC102186105
4	7620768	7720768	0.409311	LOC102186385
4	7620768	7720768	0.409311	LOC106501766
4	7620769	7720769	0.409311	LOC102186105
4	7620769	7720769	0.409311	LOC102186385
4	7620769	7720769	0.409311	LOC106501766
4	7620792	7720792	0.409311	LOC102186105
4	7620792	7720792	0.409311	LOC102186385
4	7620792	7720792	0.409311	LOC106501766
4	7620872	7720872	0.409311	LOC102186105
4	7620872	7720872	0.409311	LOC102186385

4	7620872	7720872	0.409311		LOC106501766
4	7620896	7720896	0.409245		LOC102186105
4	7620896	7720896	0.409245		LOC102186385
4	7620896	7720896	0.409245		LOC106501766
4	7620985	7720985	0.409573		LOC102186105
4	7620985	7720985	0.409573		LOC102186385
4	7620985	7720985	0.409573		LOC106501766
4	7621034	7721034	0.409558		LOC102186105
4	7621034	7721034	0.409558		LOC102186385
4	7621034	7721034	0.409558		LOC106501766
4	7621080	7721080	0.409558		LOC102186105
4	7621080	7721080	0.409558		LOC102186385
4	7621080	7721080	0.409558		LOC106501766
4	7621137	7721137	0.409779		LOC102186105
4	7621137	7721137	0.409779		LOC102186385
4	7621137	7721137	0.409779		LOC106501766
4	7621156	7721156	0.40979		LOC102186105
4	7621156	7721156	0.40979		LOC102186385
4	7621156	7721156	0.40979		LOC106501766
4	7621163	7721163	0.409454		LOC102186105
4	7621163	7721163	0.409454		LOC102186385
4	7621163	7721163	0.409454		LOC106501766
4	7621183	7721183	0.409454		LOC102186105
4	7621183	7721183	0.409454		LOC102186385
4	7621183	7721183	0.409454		LOC106501766
4	7621213	7721213	0.40931		LOC102186105
4	7621213	7721213	0.40931		LOC102186385
4	7621213	7721213	0.40931		LOC106501766
4	7621224	7721224	0.409226		LOC102186105
4	7621224	7721224	0.409226		LOC102186385
4	7621224	7721224	0.409226		LOC106501766
4	7621225	7721225	0.409226		LOC102186105
4	7621225	7721225	0.409226		LOC102186385
4	7621225	7721225	0.409226		LOC106501766
4	7621305	7721305	0.412573		LOC102186105
4	7621305	7721305	0.412573		LOC102186385
4	7621305	7721305	0.412573		LOC106501766
4	7621377	7721377	0.408228		LOC102186105
4	7621377	7721377	0.408228		LOC102186385
4	7621377	7721377	0.408228		LOC106501766
4	7621540	7721540	0.408228		LOC102186105
4	7621540	7721540	0.408228		LOC102186385
4	7621540	7721540	0.408228		LOC106501766
4	7621562	7721562	0.408228		LOC102186105
4	7621562	7721562	0.408228		LOC102186385
4	7621562	7721562	0.408228		LOC106501766
4	7621572	7721572	0.408228		LOC102186105
4	7621572	7721572	0.408228		LOC102186385
4	7621572	7721572	0.408228		LOC106501766
4	7621644	7721644	0.408228		LOC102186105
4	7621644	7721644	0.408228		LOC102186385
4	7621644	7721644	0.408228		LOC106501766
4	7621676	7721676	0.408228		LOC102186105
4	7621676	7721676	0.408228		LOC102186385
4	7621676	7721676	0.408228		LOC106501766
4	7621685	7721685	0.408228		LOC102186105
4	7621685	7721685	0.408228		LOC102186385
4	7621685	7721685	0.408228		LOC106501766

4	7621721	7721721	0.408228	LOC102186105
4	7621721	7721721	0.408228	LOC102186385
4	7621721	7721721	0.408228	LOC106501766
4	7621725	7721725	0.408228	LOC102186105
4	7621725	7721725	0.408228	LOC102186385
4	7621725	7721725	0.408228	LOC106501766
4	7621980	7721980	0.40751	LOC102186105
4	7621980	7721980	0.40751	LOC102186385
4	7621980	7721980	0.40751	LOC106501766
4	7622035	7722035	0.4091	LOC102186105
4	7622035	7722035	0.4091	LOC102186385
4	7622035	7722035	0.4091	LOC106501766
4	7622096	7722096	0.409075	LOC102186105
4	7622096	7722096	0.409075	LOC102186385
4	7622096	7722096	0.409075	LOC106501766
4	7622145	7722145	0.409026	LOC102186105
4	7622145	7722145	0.409026	LOC102186385
4	7622145	7722145	0.409026	LOC106501766
4	7622197	7722197	0.409026	LOC102186105
4	7622197	7722197	0.409026	LOC102186385
4	7622197	7722197	0.409026	LOC106501766
4	7622208	7722208	0.409026	LOC102186105
4	7622208	7722208	0.409026	LOC102186385
4	7622208	7722208	0.409026	LOC106501766
4	7622229	7722229	0.408734	LOC102186105
4	7622229	7722229	0.408734	LOC102186385
4	7622229	7722229	0.408734	LOC106501766
4	7622245	7722245	0.408734	LOC102186105
4	7622245	7722245	0.408734	LOC102186385
4	7622245	7722245	0.408734	LOC106501766
4	7622272	7722272	0.409879	LOC102186105
4	7622272	7722272	0.409879	LOC102186385
4	7622272	7722272	0.409879	LOC106501766
4	7622309	7722309	0.409879	LOC102186105
4	7622309	7722309	0.409879	LOC102186385
4	7622309	7722309	0.409879	LOC106501766
4	7622346	7722346	0.409879	LOC102186105
4	7622346	7722346	0.409879	LOC102186385
4	7622346	7722346	0.409879	LOC106501766
4	7622348	7722348	0.409879	LOC102186105
4	7622348	7722348	0.409879	LOC102186385
4	7622348	7722348	0.409879	LOC106501766
4	7622360	7722360	0.409879	LOC102186105
4	7622360	7722360	0.409879	LOC102186385
4	7622360	7722360	0.409879	LOC106501766
4	7622398	7722398	0.409879	LOC102186105
4	7622398	7722398	0.409879	LOC102186385
4	7622398	7722398	0.409879	LOC106501766
4	7622551	7722551	0.407849	LOC102186105
4	7622551	7722551	0.407849	LOC102186385
4	7622551	7722551	0.407849	LOC106501766
4	7622582	7722582	0.408339	LOC102186105
4	7622582	7722582	0.408339	LOC102186385
4	7622582	7722582	0.408339	LOC106501766
4	7622650	7722650	0.407913	LOC102186105
4	7622650	7722650	0.407913	LOC102186385
4	7622650	7722650	0.407913	LOC106501766
4	7622735	7722735	0.40855	LOC102186105

4	7622735	7722735	0.40855	LOC102186385
4	7622735	7722735	0.40855	LOC106501766
4	7622772	7722772	0.40853	LOC102186105
4	7622772	7722772	0.40853	LOC102186385
4	7622772	7722772	0.40853	LOC106501766
4	7622796	7722796	0.40853	LOC102186105
4	7622796	7722796	0.40853	LOC102186385
4	7622796	7722796	0.40853	LOC106501766
4	7622815	7722815	0.408719	LOC102186105
4	7622815	7722815	0.408719	LOC102186385
4	7622815	7722815	0.408719	LOC106501766
4	7622876	7722876	0.407978	LOC102186105
4	7622876	7722876	0.407978	LOC102186385
4	7622876	7722876	0.407978	LOC106501766
4	7622970	7722970	0.408008	LOC102186105
4	7622970	7722970	0.408008	LOC102186385
4	7622970	7722970	0.408008	LOC106501766
4	7622998	7722998	0.408008	LOC102186105
4	7622998	7722998	0.408008	LOC102186385
4	7622998	7722998	0.408008	LOC106501766
4	7623099	7723099	0.408554	LOC102186105
4	7623099	7723099	0.408554	LOC102186385
4	7623099	7723099	0.408554	LOC106501766
4	7623103	7723103	0.408554	LOC102186105
4	7623103	7723103	0.408554	LOC102186385
4	7623103	7723103	0.408554	LOC106501766
4	7623186	7723186	0.408289	LOC102186105
4	7623186	7723186	0.408289	LOC102186385
4	7623186	7723186	0.408289	LOC106501766
4	7623209	7723209	0.408289	LOC102186105
4	7623209	7723209	0.408289	LOC102186385
4	7623209	7723209	0.408289	LOC106501766
4	7623366	7723366	0.408289	LOC102186105
4	7623366	7723366	0.408289	LOC102186385
4	7623366	7723366	0.408289	LOC106501766
4	7623395	7723395	0.409948	LOC102186105
4	7623395	7723395	0.409948	LOC102186385
4	7623395	7723395	0.409948	LOC106501766
4	7623460	7723460	0.410839	LOC102186105
4	7623460	7723460	0.410839	LOC102186385
4	7623460	7723460	0.410839	LOC106501766
4	7623506	7723506	0.408915	LOC102186105
4	7623506	7723506	0.408915	LOC102186385
4	7623506	7723506	0.408915	LOC106501766
4	7623653	7723653	0.407901	LOC102186105
4	7623653	7723653	0.407901	LOC102186385
4	7623653	7723653	0.407901	LOC106501766
4	7623724	7723724	0.409244	LOC102186105
4	7623724	7723724	0.409244	LOC102186385
4	7623724	7723724	0.409244	LOC106501766
4	7623842	7723842	0.407856	LOC102186105
4	7623842	7723842	0.407856	LOC102186385
4	7623842	7723842	0.407856	LOC106501766
4	7623865	7723865	0.407856	LOC102186105
4	7623865	7723865	0.407856	LOC102186385
4	7623865	7723865	0.407856	LOC106501766
4	7623902	7723902	0.407856	LOC102186105
4	7623902	7723902	0.407856	LOC102186385

4	7623902	7723902	0.407856	LOC106501766
4	7623910	7723910	0.417466	LOC102186105
4	7623910	7723910	0.417466	LOC102186385
4	7623910	7723910	0.417466	LOC106501766
4	7623929	7723929	0.413348	LOC102186105
4	7623929	7723929	0.413348	LOC102186385
4	7623929	7723929	0.413348	LOC106501766
4	7624032	7724032	0.418017	LOC102186105
4	7624032	7724032	0.418017	LOC102186385
4	7624032	7724032	0.418017	LOC106501766
4	7624034	7724034	0.418017	LOC102186105
4	7624034	7724034	0.418017	LOC102186385
4	7624034	7724034	0.418017	LOC106501766
4	7624036	7724036	0.418017	LOC102186105
4	7624036	7724036	0.418017	LOC102186385
4	7624036	7724036	0.418017	LOC106501766
4	7624039	7724039	0.41142	LOC102186105
4	7624039	7724039	0.41142	LOC102186385
4	7624039	7724039	0.41142	LOC106501766
4	7624112	7724112	0.412091	LOC102186105
4	7624112	7724112	0.412091	LOC102186385
4	7624112	7724112	0.412091	LOC106501766
4	7624151	7724151	0.411584	LOC102186105
4	7624151	7724151	0.411584	LOC102186385
4	7624151	7724151	0.411584	LOC106501766
4	7624210	7724210	0.411976	LOC102186105
4	7624210	7724210	0.411976	LOC102186385
4	7624210	7724210	0.411976	LOC106501766
4	7624257	7724257	0.411976	LOC102186105
4	7624257	7724257	0.411976	LOC102186385
4	7624257	7724257	0.411976	LOC106501766
4	7624260	7724260	0.413559	LOC102186105
4	7624260	7724260	0.413559	LOC102186385
4	7624260	7724260	0.413559	LOC106501766
4	7624304	7724304	0.411878	LOC102186105
4	7624304	7724304	0.411878	LOC102186385
4	7624304	7724304	0.411878	LOC106501766
4	7624306	7724306	0.411878	LOC102186105
4	7624306	7724306	0.411878	LOC102186385
4	7624306	7724306	0.411878	LOC106501766
4	7624319	7724319	0.411988	LOC102186105
4	7624319	7724319	0.411988	LOC102186385
4	7624319	7724319	0.411988	LOC106501766
4	7624351	7724351	0.411937	LOC102186105
4	7624351	7724351	0.411937	LOC102186385
4	7624351	7724351	0.411937	LOC106501766
4	7624355	7724355	0.411937	LOC102186105
4	7624355	7724355	0.411937	LOC102186385
4	7624355	7724355	0.411937	LOC106501766
4	7624362	7724362	0.411937	LOC102186105
4	7624362	7724362	0.411937	LOC102186385
4	7624362	7724362	0.411937	LOC106501766
4	7624376	7724376	0.411937	LOC102186105
4	7624376	7724376	0.411937	LOC102186385
4	7624376	7724376	0.411937	LOC106501766
4	7624380	7724380	0.412129	LOC102186105
4	7624380	7724380	0.412129	LOC102186385
4	7624380	7724380	0.412129	LOC106501766

4	7624406	7724406	0.413119	LOC102186105
4	7624406	7724406	0.413119	LOC102186385
4	7624406	7724406	0.413119	LOC106501766
4	7624441	7724441	0.412356	LOC102186105
4	7624441	7724441	0.412356	LOC102186385
4	7624441	7724441	0.412356	LOC106501766
4	7624470	7724470	0.412423	LOC102186105
4	7624470	7724470	0.412423	LOC102186385
4	7624470	7724470	0.412423	LOC106501766
4	7624503	7724503	0.412401	LOC102186105
4	7624503	7724503	0.412401	LOC102186385
4	7624503	7724503	0.412401	LOC106501766
4	7624504	7724504	0.412221	LOC102186105
4	7624504	7724504	0.412221	LOC102186385
4	7624504	7724504	0.412221	LOC106501766
4	7624555	7724555	0.413843	LOC102186105
4	7624555	7724555	0.413843	LOC102186385
4	7624555	7724555	0.413843	LOC106501766
4	7624563	7724563	0.413843	LOC102186105
4	7624563	7724563	0.413843	LOC102186385
4	7624563	7724563	0.413843	LOC106501766
4	7624578	7724578	0.413843	LOC102186105
4	7624578	7724578	0.413843	LOC102186385
4	7624578	7724578	0.413843	LOC106501766
4	7624594	7724594	0.415111	LOC102186105
4	7624594	7724594	0.415111	LOC102186385
4	7624594	7724594	0.415111	LOC106501766
4	7624595	7724595	0.415111	LOC102186105
4	7624595	7724595	0.415111	LOC102186385
4	7624595	7724595	0.415111	LOC106501766
4	7624615	7724615	0.415111	LOC102186105
4	7624615	7724615	0.415111	LOC102186385
4	7624615	7724615	0.415111	LOC106501766
4	7624626	7724626	0.415142	LOC102186105
4	7624626	7724626	0.415142	LOC102186385
4	7624626	7724626	0.415142	LOC106501766
4	7624633	7724633	0.41524	LOC102186105
4	7624633	7724633	0.41524	LOC102186385
4	7624633	7724633	0.41524	LOC106501766
4	7624651	7724651	0.414489	LOC102186105
4	7624651	7724651	0.414489	LOC102186385
4	7624651	7724651	0.414489	LOC106501766
4	7624684	7724684	0.413192	LOC102186105
4	7624684	7724684	0.413192	LOC102186385
4	7624684	7724684	0.413192	LOC106501766
4	7624714	7724714	0.411056	LOC102186105
4	7624714	7724714	0.411056	LOC102186385
4	7624714	7724714	0.411056	LOC106501766
4	7624741	7724741	0.411056	LOC102186105
4	7624741	7724741	0.411056	LOC102186385
4	7624741	7724741	0.411056	LOC106501766
4	7624748	7724748	0.411056	LOC102186105
4	7624748	7724748	0.411056	LOC102186385
4	7624748	7724748	0.411056	LOC106501766
4	7624772	7724772	0.411396	LOC102186105
4	7624772	7724772	0.411396	LOC102186385
4	7624772	7724772	0.411396	LOC106501766
4	7624787	7724787	0.411396	LOC102186105

4	7624787	7724787	0.411396	LOC102186385
4	7624787	7724787	0.411396	LOC106501766
4	7624788	7724788	0.411396	LOC102186105
4	7624788	7724788	0.411396	LOC102186385
4	7624788	7724788	0.411396	LOC106501766
4	7624795	7724795	0.411396	LOC102186105
4	7624795	7724795	0.411396	LOC102186385
4	7624795	7724795	0.411396	LOC106501766
4	7624796	7724796	0.412232	LOC102186105
4	7624796	7724796	0.412232	LOC102186385
4	7624796	7724796	0.412232	LOC106501766
4	7624803	7724803	0.413528	LOC102186105
4	7624803	7724803	0.413528	LOC102186385
4	7624803	7724803	0.413528	LOC106501766
4	7624811	7724811	0.413524	LOC102186105
4	7624811	7724811	0.413524	LOC102186385
4	7624811	7724811	0.413524	LOC106501766
4	7624823	7724823	0.413285	LOC102186105
4	7624823	7724823	0.413285	LOC102186385
4	7624823	7724823	0.413285	LOC106501766
4	7624849	7724849	0.413412	LOC102186105
4	7624849	7724849	0.413412	LOC102186385
4	7624849	7724849	0.413412	LOC106501766
4	7624884	7724884	0.413412	LOC102186105
4	7624884	7724884	0.413412	LOC102186385
4	7624884	7724884	0.413412	LOC106501766
4	7624888	7724888	0.413412	LOC102186105
4	7624888	7724888	0.413412	LOC102186385
4	7624888	7724888	0.413412	LOC106501766
4	7624897	7724897	0.413456	LOC102186105
4	7624897	7724897	0.413456	LOC102186385
4	7624897	7724897	0.413456	LOC106501766
4	7624898	7724898	0.412757	LOC102186105
4	7624898	7724898	0.412757	LOC102186385
4	7624898	7724898	0.412757	LOC106501766
4	7624899	7724899	0.4129	LOC102186105
4	7624899	7724899	0.4129	LOC102186385
4	7624899	7724899	0.4129	LOC106501766
4	7624912	7724912	0.412934	LOC102186105
4	7624912	7724912	0.412934	LOC102186385
4	7624912	7724912	0.412934	LOC106501766
4	7624970	7724970	0.413031	LOC102186105
4	7624970	7724970	0.413031	LOC102186385
4	7624970	7724970	0.413031	LOC106501766
4	7625008	7725008	0.404107	LOC102186105
4	7625008	7725008	0.404107	LOC102186385
4	7625008	7725008	0.404107	LOC106501766
4	7625041	7725041	0.401985	LOC102186105
4	7625041	7725041	0.401985	LOC102186385
4	7625041	7725041	0.401985	LOC106501766
4	7625045	7725045	0.414489	LOC102186105
4	7625045	7725045	0.414489	LOC102186385
4	7625045	7725045	0.414489	LOC106501766
4	7625153	7725153	0.403439	LOC102186105
4	7625153	7725153	0.403439	LOC102186385
4	7625153	7725153	0.403439	LOC106501766
4	7625159	7725159	0.416478	LOC102186105
4	7625159	7725159	0.416478	LOC102186385

4	7625159	7725159	0.416478	LOC106501766
4	7625210	7725210	0.41251	LOC102186105
4	7625210	7725210	0.41251	LOC102186385
4	7625210	7725210	0.41251	LOC106501766
4	7625253	7725253	0.412735	LOC102186105
4	7625253	7725253	0.412735	LOC102186385
4	7625253	7725253	0.412735	LOC106501766
4	7625254	7725254	0.412735	LOC102186105
4	7625254	7725254	0.412735	LOC102186385
4	7625254	7725254	0.412735	LOC106501766
4	7625285	7725285	0.412726	LOC102186105
4	7625285	7725285	0.412726	LOC102186385
4	7625285	7725285	0.412726	LOC106501766
4	7625313	7725313	0.412714	LOC102186105
4	7625313	7725313	0.412714	LOC102186385
4	7625313	7725313	0.412714	LOC106501766
4	7625328	7725328	0.412188	LOC102186105
4	7625328	7725328	0.412188	LOC102186385
4	7625328	7725328	0.412188	LOC106501766
4	7625351	7725351	0.412147	LOC102186105
4	7625351	7725351	0.412147	LOC102186385
4	7625351	7725351	0.412147	LOC106501766
4	7625352	7725352	0.412147	LOC102186105
4	7625352	7725352	0.412147	LOC102186385
4	7625352	7725352	0.412147	LOC106501766
4	7625357	7725357	0.412147	LOC102186105
4	7625357	7725357	0.412147	LOC102186385
4	7625357	7725357	0.412147	LOC106501766
4	7625359	7725359	0.412147	LOC102186105
4	7625359	7725359	0.412147	LOC102186385
4	7625359	7725359	0.412147	LOC106501766
4	7625376	7725376	0.412139	LOC102186105
4	7625376	7725376	0.412139	LOC102186385
4	7625376	7725376	0.412139	LOC106501766
4	7625378	7725378	0.412139	LOC102186105
4	7625378	7725378	0.412139	LOC102186385
4	7625378	7725378	0.412139	LOC106501766
4	7625442	7725442	0.410237	LOC102186105
4	7625442	7725442	0.410237	LOC102186385
4	7625442	7725442	0.410237	LOC106501766
4	7625486	7725486	0.410141	LOC102186105
4	7625486	7725486	0.410141	LOC102186385
4	7625486	7725486	0.410141	LOC106501766
4	7625497	7725497	0.408179	LOC102186105
4	7625497	7725497	0.408179	LOC102186385
4	7625497	7725497	0.408179	LOC106501766
4	7625513	7725513	0.408179	LOC102186105
4	7625513	7725513	0.408179	LOC102186385
4	7625513	7725513	0.408179	LOC106501766
4	7625550	7725550	0.408128	LOC102186105
4	7625550	7725550	0.408128	LOC102186385
4	7625550	7725550	0.408128	LOC106501766
4	7625553	7725553	0.408128	LOC102186105
4	7625553	7725553	0.408128	LOC102186385
4	7625553	7725553	0.408128	LOC106501766
4	7625590	7725590	0.411214	LOC102186105
4	7625590	7725590	0.411214	LOC102186385
4	7625590	7725590	0.411214	LOC106501766

4	7625593	7725593	0.410252	LOC102186105
4	7625593	7725593	0.410252	LOC102186385
4	7625593	7725593	0.410252	LOC106501766
4	7625596	7725596	0.425948	LOC102186105
4	7625596	7725596	0.425948	LOC102186385
4	7625596	7725596	0.425948	LOC106501766
4	7625600	7725600	0.425948	LOC102186105
4	7625600	7725600	0.425948	LOC102186385
4	7625600	7725600	0.425948	LOC106501766
4	7625624	7725624	0.412806	LOC102186105
4	7625624	7725624	0.412806	LOC102186385
4	7625624	7725624	0.412806	LOC106501766
4	7625638	7725638	0.413414	LOC102186105
4	7625638	7725638	0.413414	LOC102186385
4	7625638	7725638	0.413414	LOC106501766
4	7625731	7725731	0.419033	LOC102186105
4	7625731	7725731	0.419033	LOC102186385
4	7625731	7725731	0.419033	LOC106501766
4	7625733	7725733	0.413473	LOC102186105
4	7625733	7725733	0.413473	LOC102186385
4	7625733	7725733	0.413473	LOC106501766
4	7625754	7725754	0.413206	LOC102186105
4	7625754	7725754	0.413206	LOC102186385
4	7625754	7725754	0.413206	LOC106501766
4	7625756	7725756	0.413181	LOC102186105
4	7625756	7725756	0.413181	LOC102186385
4	7625756	7725756	0.413181	LOC106501766
4	7625760	7725760	0.413167	LOC102186105
4	7625760	7725760	0.413167	LOC102186385
4	7625760	7725760	0.413167	LOC106501766
4	7625772	7725772	0.413167	LOC102186105
4	7625772	7725772	0.413167	LOC102186385
4	7625772	7725772	0.413167	LOC106501766
4	7625812	7725812	0.414279	LOC102186105
4	7625812	7725812	0.414279	LOC102186385
4	7625812	7725812	0.414279	LOC106501766
4	7625872	7725872	0.414149	LOC102186105
4	7625872	7725872	0.414149	LOC102186385
4	7625872	7725872	0.414149	LOC106501766
4	7625911	7725911	0.415148	LOC102186105
4	7625911	7725911	0.415148	LOC102186385
4	7625911	7725911	0.415148	LOC106501766
4	7625920	7725920	0.415148	LOC102186105
4	7625920	7725920	0.415148	LOC102186385
4	7625920	7725920	0.415148	LOC106501766
4	7625922	7725922	0.415148	LOC102186105
4	7625922	7725922	0.415148	LOC102186385
4	7625922	7725922	0.415148	LOC106501766
4	7625924	7725924	0.415148	LOC102186105
4	7625924	7725924	0.415148	LOC102186385
4	7625924	7725924	0.415148	LOC106501766
4	7625926	7725926	0.415148	LOC102186105
4	7625926	7725926	0.415148	LOC102186385
4	7625926	7725926	0.415148	LOC106501766
4	7625961	7725961	0.415148	LOC102186105
4	7625961	7725961	0.415148	LOC102186385
4	7625961	7725961	0.415148	LOC106501766
4	7626158	7726158	0.414324	LOC102186105

4	7626158	7726158	0.414324	LOC102186385
4	7626158	7726158	0.414324	LOC106501766
4	7626172	7726172	0.416413	LOC102186105
4	7626172	7726172	0.416413	LOC102186385
4	7626172	7726172	0.416413	LOC106501766
4	7626179	7726179	0.406525	LOC102186105
4	7626179	7726179	0.406525	LOC102186385
4	7626179	7726179	0.406525	LOC106501766
4	7626188	7726188	0.406525	LOC102186105
4	7626188	7726188	0.406525	LOC102186385
4	7626188	7726188	0.406525	LOC106501766
4	7626191	7726191	0.406525	LOC102186105
4	7626191	7726191	0.406525	LOC102186385
4	7626191	7726191	0.406525	LOC106501766
4	7626232	7726232	0.406858	LOC102186105
4	7626232	7726232	0.406858	LOC102186385
4	7626232	7726232	0.406858	LOC106501766
4	7626237	7726237	0.406807	LOC102186105
4	7626237	7726237	0.406807	LOC102186385
4	7626237	7726237	0.406807	LOC106501766
4	7626258	7726258	0.406897	LOC102186105
4	7626258	7726258	0.406897	LOC102186385
4	7626258	7726258	0.406897	LOC106501766
4	7626299	7726299	0.406661	LOC102186105
4	7626299	7726299	0.406661	LOC102186385
4	7626299	7726299	0.406661	LOC106501766
4	7626310	7726310	0.406598	LOC102186105
4	7626310	7726310	0.406598	LOC102186385
4	7626310	7726310	0.406598	LOC106501766
4	7626320	7726320	0.406616	LOC102186105
4	7626320	7726320	0.406616	LOC102186385
4	7626320	7726320	0.406616	LOC106501766
4	7626344	7726344	0.406616	LOC102186105
4	7626344	7726344	0.406616	LOC102186385
4	7626344	7726344	0.406616	LOC106501766
4	7626392	7726392	0.40661	LOC102186105
4	7626392	7726392	0.40661	LOC102186385
4	7626392	7726392	0.40661	LOC106501766
4	7626423	7726423	0.406335	LOC102186105
4	7626423	7726423	0.406335	LOC102186385
4	7626423	7726423	0.406335	LOC106501766
4	7626428	7726428	0.406335	LOC102186105
4	7626428	7726428	0.406335	LOC102186385
4	7626428	7726428	0.406335	LOC106501766
4	7626474	7726474	0.406335	LOC102186105
4	7626474	7726474	0.406335	LOC102186385
4	7626474	7726474	0.406335	LOC106501766
4	7626477	7726477	0.406335	LOC102186105
4	7626477	7726477	0.406335	LOC102186385
4	7626477	7726477	0.406335	LOC106501766
4	7626505	7726505	0.406986	LOC102186105
4	7626505	7726505	0.406986	LOC102186385
4	7626505	7726505	0.406986	LOC106501766
4	7626531	7726531	0.405315	LOC102186105
4	7626531	7726531	0.405315	LOC102186385
4	7626531	7726531	0.405315	LOC106501766
4	7626572	7726572	0.404534	LOC102186105
4	7626572	7726572	0.404534	LOC102186385

4	7626572	7726572	0.404534	LOC106501766
4	7626604	7726604	0.404534	LOC102186105
4	7626604	7726604	0.404534	LOC102186385
4	7626604	7726604	0.404534	LOC106501766
4	7626624	7726624	0.404868	LOC102186105
4	7626624	7726624	0.404868	LOC102186385
4	7626624	7726624	0.404868	LOC106501766
4	7626627	7726627	0.40445	LOC102186105
4	7626627	7726627	0.40445	LOC102186385
4	7626627	7726627	0.40445	LOC106501766
4	7626755	7726755	0.404473	LOC102186105
4	7626755	7726755	0.404473	LOC102186385
4	7626755	7726755	0.404473	LOC106501766
4	7626770	7726770	0.404473	LOC102186105
4	7626770	7726770	0.404473	LOC102186385
4	7626770	7726770	0.404473	LOC106501766
4	7626790	7726790	0.404473	LOC102186105
4	7626790	7726790	0.404473	LOC102186385
4	7626790	7726790	0.404473	LOC106501766
4	7626821	7726821	0.40548	LOC102186105
4	7626821	7726821	0.40548	LOC102186385
4	7626821	7726821	0.40548	LOC106501766
4	7626863	7726863	0.404988	LOC102186105
4	7626863	7726863	0.404988	LOC102186385
4	7626863	7726863	0.404988	LOC106501766
4	7626871	7726871	0.404988	LOC102186105
4	7626871	7726871	0.404988	LOC102186385
4	7626871	7726871	0.404988	LOC106501766
4	7626873	7726873	0.405432	LOC102186105
4	7626873	7726873	0.405432	LOC102186385
4	7626873	7726873	0.405432	LOC106501766
4	7626878	7726878	0.405195	LOC102186105
4	7626878	7726878	0.405195	LOC102186385
4	7626878	7726878	0.405195	LOC106501766
4	7626879	7726879	0.405195	LOC102186105
4	7626879	7726879	0.405195	LOC102186385
4	7626879	7726879	0.405195	LOC106501766
4	7626894	7726894	0.405195	LOC102186105
4	7626894	7726894	0.405195	LOC102186385
4	7626894	7726894	0.405195	LOC106501766
4	7626909	7726909	0.405195	LOC102186105
4	7626909	7726909	0.405195	LOC102186385
4	7626909	7726909	0.405195	LOC106501766
4	7627007	7727007	0.405195	LOC102186105
4	7627007	7727007	0.405195	LOC102186385
4	7627007	7727007	0.405195	LOC106501766
4	7627008	7727008	0.405432	LOC102186105
4	7627008	7727008	0.405432	LOC102186385
4	7627008	7727008	0.405432	LOC106501766
4	7627016	7727016	0.40493	LOC102186105
4	7627016	7727016	0.40493	LOC102186385
4	7627016	7727016	0.40493	LOC106501766
4	7627031	7727031	0.40493	LOC102186105
4	7627031	7727031	0.40493	LOC102186385
4	7627031	7727031	0.40493	LOC106501766
4	7627124	7727124	0.40493	LOC102186105
4	7627124	7727124	0.40493	LOC102186385
4	7627124	7727124	0.40493	LOC106501766

4	7627160	7727160	0.40493	LOC102186105
4	7627160	7727160	0.40493	LOC102186385
4	7627160	7727160	0.40493	LOC106501766
4	7627224	7727224	0.40493	LOC102186105
4	7627224	7727224	0.40493	LOC102186385
4	7627224	7727224	0.40493	LOC106501766
4	7627252	7727252	0.40493	LOC102186105
4	7627252	7727252	0.40493	LOC102186385
4	7627252	7727252	0.40493	LOC106501766
4	7627266	7727266	0.40493	LOC102186105
4	7627266	7727266	0.40493	LOC102186385
4	7627266	7727266	0.40493	LOC106501766
4	7627271	7727271	0.40493	LOC102186105
4	7627271	7727271	0.40493	LOC102186385
4	7627271	7727271	0.40493	LOC106501766
4	7627323	7727323	0.404578	LOC102186105
4	7627323	7727323	0.404578	LOC102186385
4	7627323	7727323	0.404578	LOC106501766
4	7627324	7727324	0.4045	LOC102186105
4	7627324	7727324	0.4045	LOC102186385
4	7627324	7727324	0.4045	LOC106501766
4	7627384	7727384	0.405375	LOC102186105
4	7627384	7727384	0.405375	LOC102186385
4	7627384	7727384	0.405375	LOC106501766
4	7627397	7727397	0.405468	LOC102186105
4	7627397	7727397	0.405468	LOC102186385
4	7627397	7727397	0.405468	LOC106501766
4	7627409	7727409	0.405293	LOC102186105
4	7627409	7727409	0.405293	LOC102186385
4	7627409	7727409	0.405293	LOC106501766
4	7627427	7727427	0.405293	LOC102186105
4	7627427	7727427	0.405293	LOC102186385
4	7627427	7727427	0.405293	LOC106501766
4	7627464	7727464	0.405293	LOC102186105
4	7627464	7727464	0.405293	LOC102186385
4	7627464	7727464	0.405293	LOC106501766
4	7627465	7727465	0.405293	LOC102186105
4	7627465	7727465	0.405293	LOC102186385
4	7627465	7727465	0.405293	LOC106501766
4	7627498	7727498	0.405293	LOC102186105
4	7627498	7727498	0.405293	LOC102186385
4	7627498	7727498	0.405293	LOC106501766
4	7627543	7727543	0.405431	LOC102186105
4	7627543	7727543	0.405431	LOC102186385
4	7627543	7727543	0.405431	LOC106501766
4	7627560	7727560	0.405431	LOC102186105
4	7627560	7727560	0.405431	LOC102186385
4	7627560	7727560	0.405431	LOC106501766
4	7627619	7727619	0.401897	LOC102186105
4	7627619	7727619	0.401897	LOC102186385
4	7627619	7727619	0.401897	LOC106501766
4	7627909	7727909	0.401897	LOC102186105
4	7627909	7727909	0.401897	LOC102186385
4	7627909	7727909	0.401897	LOC106501766
4	7627926	7727926	0.399341	LOC102186105
4	7627926	7727926	0.399341	LOC102186385
4	7627926	7727926	0.399341	LOC106501766
4	7627959	7727959	0.40661	LOC102186105

4	7627959	7727959	0.40661	LOC102186385
4	7627959	7727959	0.40661	LOC106501766
4	7628024	7728024	0.406609	LOC102186105
4	7628024	7728024	0.406609	LOC102186385
4	7628024	7728024	0.406609	LOC106501766
4	7628033	7728033	0.406456	LOC102186105
4	7628033	7728033	0.406456	LOC102186385
4	7628033	7728033	0.406456	LOC106501766
4	7628094	7728094	0.406468	LOC102186105
4	7628094	7728094	0.406468	LOC102186385
4	7628094	7728094	0.406468	LOC106501766
4	7628153	7728153	0.406247	LOC102186105
4	7628153	7728153	0.406247	LOC102186385
4	7628153	7728153	0.406247	LOC106501766
4	7628169	7728169	0.404369	LOC102186105
4	7628169	7728169	0.404369	LOC102186385
4	7628169	7728169	0.404369	LOC106501766
4	7628177	7728177	0.404369	LOC102186105
4	7628177	7728177	0.404369	LOC102186385
4	7628177	7728177	0.404369	LOC106501766
4	7628195	7728195	0.404338	LOC102186105
4	7628195	7728195	0.404338	LOC102186385
4	7628195	7728195	0.404338	LOC106501766
4	7628200	7728200	0.404362	LOC102186105
4	7628200	7728200	0.404362	LOC102186385
4	7628200	7728200	0.404362	LOC106501766
4	7628226	7728226	0.404345	LOC102186105
4	7628226	7728226	0.404345	LOC102186385
4	7628226	7728226	0.404345	LOC106501766
4	7628266	7728266	0.404362	LOC102186105
4	7628266	7728266	0.404362	LOC102186385
4	7628266	7728266	0.404362	LOC106501766
4	7628275	7728275	0.404339	LOC102186105
4	7628275	7728275	0.404339	LOC102186385
4	7628275	7728275	0.404339	LOC106501766
4	7628296	7728296	0.404543	LOC102186105
4	7628296	7728296	0.404543	LOC102186385
4	7628296	7728296	0.404543	LOC106501766
4	7628446	7728446	0.396312	LOC102186105
4	7628446	7728446	0.396312	LOC102186385
4	7628446	7728446	0.396312	LOC106501766
4	7628450	7728450	0.400222	LOC102186105
4	7628450	7728450	0.400222	LOC102186385
4	7628450	7728450	0.400222	LOC106501766
4	7628486	7728486	0.4004	LOC102186105
4	7628486	7728486	0.4004	LOC102186385
4	7628486	7728486	0.4004	LOC106501766
4	7628513	7728513	0.4004	LOC102186105
4	7628513	7728513	0.4004	LOC102186385
4	7628513	7728513	0.4004	LOC106501766
4	7628625	7728625	0.4004	LOC102186105
4	7628625	7728625	0.4004	LOC102186385
4	7628625	7728625	0.4004	LOC106501766
4	7628770	7728770	0.403741	LOC102186105
4	7628770	7728770	0.403741	LOC102186385
4	7628770	7728770	0.403741	LOC106501766
4	7628813	7728813	0.402173	LOC102186105
4	7628813	7728813	0.402173	LOC102186385

4	7628813	7728813	0.402173	LOC106501766
4	7628874	7728874	0.401802	LOC102186105
4	7628874	7728874	0.401802	LOC102186385
4	7628874	7728874	0.401802	LOC106501766
4	7628886	7728886	0.40188	LOC102186105
4	7628886	7728886	0.40188	LOC102186385
4	7628886	7728886	0.40188	LOC106501766
4	7629548	7729548	0.344847	LOC102186105
4	7629548	7729548	0.344847	LOC102186385
4	7629548	7729548	0.344847	LOC106501766
4	7629855	7729855	0.34519	LOC102186105
4	7629855	7729855	0.34519	LOC102186385
4	7629855	7729855	0.34519	LOC106501766
4	7629930	7729930	0.347316	LOC102186105
4	7629930	7729930	0.347316	LOC102186385
4	7629930	7729930	0.347316	LOC106501766
4	7630018	7730018	0.346437	LOC102186105
4	7630018	7730018	0.346437	LOC102186385
4	7630018	7730018	0.346437	LOC106501766
4	7630065	7730065	0.346437	LOC102186105
4	7630065	7730065	0.346437	LOC102186385
4	7630065	7730065	0.346437	LOC106501766
4	7630085	7730085	0.347426	LOC102186105
4	7630085	7730085	0.347426	LOC102186385
4	7630085	7730085	0.347426	LOC106501766
4	7630124	7730124	0.342149	LOC102186105
4	7630124	7730124	0.342149	LOC102186385
4	7630124	7730124	0.342149	LOC106501766
4	7630167	7730167	0.342149	LOC102186105
4	7630167	7730167	0.342149	LOC102186385
4	7630167	7730167	0.342149	LOC106501766
4	7630370	7730370	0.342149	LOC102186105
4	7630370	7730370	0.342149	LOC102186385
4	7630370	7730370	0.342149	LOC106501766
4	7630475	7730475	0.342149	LOC102186105
4	7630475	7730475	0.342149	LOC102186385
4	7630475	7730475	0.342149	LOC106501766
4	7630475	7730475	0.342149	LOC102168236
4	7630564	7730564	0.342149	LOC102186105
4	7630564	7730564	0.342149	LOC102186385
4	7630564	7730564	0.342149	LOC106501766
4	7630564	7730564	0.342149	LOC102168236
4	7630677	7730677	0.347335	LOC102186105
4	7630677	7730677	0.347335	LOC102186385
4	7630677	7730677	0.347335	LOC106501766
4	7630677	7730677	0.347335	LOC102168236
4	7630716	7730716	0.344676	LOC102186105
4	7630716	7730716	0.344676	LOC102186385
4	7630716	7730716	0.344676	LOC106501766
4	7630716	7730716	0.344676	LOC102168236
4	7631008	7731008	0.38651	LOC102186105
4	7631008	7731008	0.38651	LOC102186385
4	7631008	7731008	0.38651	LOC106501766
4	7631008	7731008	0.38651	LOC102168236
4	7631121	7731121	0.381976	LOC102186105
4	7631121	7731121	0.381976	LOC102186385
4	7631121	7731121	0.381976	LOC106501766
4	7631121	7731121	0.381976	LOC102168236

4	7631296	7731296	0.378109	LOC102186105
4	7631296	7731296	0.378109	LOC102186385
4	7631296	7731296	0.378109	LOC106501766
4	7631296	7731296	0.378109	LOC102168236
4	7631320	7731320	0.385811	LOC102186105
4	7631320	7731320	0.385811	LOC102186385
4	7631320	7731320	0.385811	LOC106501766
4	7631320	7731320	0.385811	LOC102168236
4	7631633	7731633	0.385484	LOC102186105
4	7631633	7731633	0.385484	LOC102186385
4	7631633	7731633	0.385484	LOC106501766
4	7631633	7731633	0.385484	LOC102168236
4	7631655	7731655	0.385961	LOC102186105
4	7631655	7731655	0.385961	LOC102186385
4	7631655	7731655	0.385961	LOC106501766
4	7631655	7731655	0.385961	LOC102168236
4	7631664	7731664	0.385765	LOC102186105
4	7631664	7731664	0.385765	LOC102186385
4	7631664	7731664	0.385765	LOC106501766
4	7631664	7731664	0.385765	LOC102168236
4	7631722	7731722	0.386617	LOC102186105
4	7631722	7731722	0.386617	LOC102186385
4	7631722	7731722	0.386617	LOC106501766
4	7631722	7731722	0.386617	LOC102168236
4	7631895	7731895	0.379055	LOC102186105
4	7631895	7731895	0.379055	LOC102186385
4	7631895	7731895	0.379055	LOC106501766
4	7631895	7731895	0.379055	LOC102168236
4	7632053	7732053	0.378263	LOC102186105
4	7632053	7732053	0.378263	LOC102186385
4	7632053	7732053	0.378263	LOC106501766
4	7632053	7732053	0.378263	LOC102168236
4	7632057	7732057	0.378263	LOC102186105
4	7632057	7732057	0.378263	LOC102186385
4	7632057	7732057	0.378263	LOC106501766
4	7632057	7732057	0.378263	LOC102168236
4	7632189	7732189	0.378263	LOC102186105
4	7632189	7732189	0.378263	LOC102186385
4	7632189	7732189	0.378263	LOC106501766
4	7632189	7732189	0.378263	LOC102168236
4	7632222	7732222	0.378263	LOC102186105
4	7632222	7732222	0.378263	LOC102186385
4	7632222	7732222	0.378263	LOC106501766
4	7632222	7732222	0.378263	LOC102168236
4	7632261	7732261	0.378669	LOC102186105
4	7632261	7732261	0.378669	LOC102186385
4	7632261	7732261	0.378669	LOC106501766
4	7632261	7732261	0.378669	LOC102168236
4	7632618	7732618	0.373976	LOC102186385
4	7632618	7732618	0.373976	LOC106501766
4	7632618	7732618	0.373976	LOC102168236
4	7632704	7732704	0.373699	LOC102186385
4	7632704	7732704	0.373699	LOC106501766
4	7632704	7732704	0.373699	LOC102168236
4	7632842	7732842	0.373699	LOC102186385
4	7632842	7732842	0.373699	LOC106501766
4	7632842	7732842	0.373699	LOC102168236
4	7632864	7732864	0.373699	LOC102186385

4	7632864	7732864	0.373699	LOC106501766
4	7632864	7732864	0.373699	LOC102168236
4	7632866	7732866	0.373699	LOC102186385
4	7632866	7732866	0.373699	LOC106501766
4	7632866	7732866	0.373699	LOC102168236
4	7632882	7732882	0.373699	LOC102186385
4	7632882	7732882	0.373699	LOC106501766
4	7632882	7732882	0.373699	LOC102168236
4	7633025	7733025	0.375134	LOC102186385
4	7633025	7733025	0.375134	LOC106501766
4	7633025	7733025	0.375134	LOC102168236
4	7633046	7733046	0.375134	LOC102186385
4	7633046	7733046	0.375134	LOC106501766
4	7633046	7733046	0.375134	LOC102168236
4	7633057	7733057	0.380341	LOC102186385
4	7633057	7733057	0.380341	LOC106501766
4	7633057	7733057	0.380341	LOC102168236
4	7633100	7733100	0.375318	LOC102186385
4	7633100	7733100	0.375318	LOC106501766
4	7633100	7733100	0.375318	LOC102168236
4	7633131	7733131	0.375318	LOC102186385
4	7633131	7733131	0.375318	LOC106501766
4	7633131	7733131	0.375318	LOC102168236
4	7633217	7733217	0.375318	LOC102186385
4	7633217	7733217	0.375318	LOC106501766
4	7633217	7733217	0.375318	LOC102168236
4	7633263	7733263	0.375318	LOC102186385
4	7633263	7733263	0.375318	LOC106501766
4	7633263	7733263	0.375318	LOC102168236
4	7633455	7733455	0.375318	LOC102186385
4	7633455	7733455	0.375318	LOC106501766
4	7633455	7733455	0.375318	LOC102168236
4	7633568	7733568	0.375318	LOC102186385
4	7633568	7733568	0.375318	LOC106501766
4	7633568	7733568	0.375318	LOC102168236
4	7633569	7733569	0.375318	LOC102186385
4	7633569	7733569	0.375318	LOC106501766
4	7633569	7733569	0.375318	LOC102168236
4	7633615	7733615	0.375318	LOC102186385
4	7633615	7733615	0.375318	LOC106501766
4	7633615	7733615	0.375318	LOC102168236
4	7633720	7733720	0.38012	LOC102186385
4	7633720	7733720	0.38012	LOC106501766
4	7633720	7733720	0.38012	LOC102168236
4	7633727	7733727	0.375069	LOC102186385
4	7633727	7733727	0.375069	LOC106501766
4	7633727	7733727	0.375069	LOC102168236
4	7633883	7733883	0.375069	LOC102186385
4	7633883	7733883	0.375069	LOC106501766
4	7633883	7733883	0.375069	LOC102168236
4	7633993	7733993	0.375069	LOC102186385
4	7633993	7733993	0.375069	LOC106501766
4	7633993	7733993	0.375069	LOC102168236
4	7634086	7734086	0.375069	LOC102186385
4	7634086	7734086	0.375069	LOC106501766
4	7634086	7734086	0.375069	LOC102168236
4	7634258	7734258	0.376725	LOC102186385
4	7634258	7734258	0.376725	LOC106501766

4	7634258	7734258	0.376725	LOC102168236
4	7634269	7734269	0.375969	LOC102186385
4	7634269	7734269	0.375969	LOC106501766
4	7634269	7734269	0.375969	LOC102168236
4	7634396	7734396	0.383621	LOC102186385
4	7634396	7734396	0.383621	LOC106501766
4	7634396	7734396	0.383621	LOC102168236
4	7634465	7734465	0.382322	LOC102186385
4	7634465	7734465	0.382322	LOC106501766
4	7634465	7734465	0.382322	LOC102168236
4	7634486	7734486	0.382437	LOC102186385
4	7634486	7734486	0.382437	LOC106501766
4	7634486	7734486	0.382437	LOC102168236
4	7634491	7734491	0.382437	LOC102186385
4	7634491	7734491	0.382437	LOC106501766
4	7634491	7734491	0.382437	LOC102168236
4	7634522	7734522	0.382517	LOC102186385
4	7634522	7734522	0.382517	LOC106501766
4	7634522	7734522	0.382517	LOC102168236
4	7634546	7734546	0.381449	LOC102186385
4	7634546	7734546	0.381449	LOC106501766
4	7634546	7734546	0.381449	LOC102168236
4	7634587	7734587	0.381449	LOC102186385
4	7634587	7734587	0.381449	LOC106501766
4	7634587	7734587	0.381449	LOC102168236
4	7634606	7734606	0.381449	LOC102186385
4	7634606	7734606	0.381449	LOC106501766
4	7634606	7734606	0.381449	LOC102168236
4	7634662	7734662	0.381449	LOC102186385
4	7634662	7734662	0.381449	LOC106501766
4	7634662	7734662	0.381449	LOC102168236
4	7634693	7734693	0.382074	LOC102186385
4	7634693	7734693	0.382074	LOC106501766
4	7634693	7734693	0.382074	LOC102168236
4	7634865	7734865	0.376094	LOC102186385
4	7634865	7734865	0.376094	LOC106501766
4	7634865	7734865	0.376094	LOC102168236
4	7634896	7734896	0.376094	LOC102186385
4	7634896	7734896	0.376094	LOC106501766
4	7634896	7734896	0.376094	LOC102168236
4	7634906	7734906	0.376094	LOC102186385
4	7634906	7734906	0.376094	LOC106501766
4	7634906	7734906	0.376094	LOC102168236
4	7634917	7734917	0.376094	LOC102186385
4	7634917	7734917	0.376094	LOC106501766
4	7634917	7734917	0.376094	LOC102168236
4	7635061	7735061	0.376094	LOC102186385
4	7635061	7735061	0.376094	LOC106501766
4	7635061	7735061	0.376094	LOC102168236
4	7635250	7735250	0.376094	LOC102186385
4	7635250	7735250	0.376094	LOC106501766
4	7635250	7735250	0.376094	LOC102168236
4	7635383	7735383	0.376094	LOC102186385
4	7635383	7735383	0.376094	LOC106501766
4	7635383	7735383	0.376094	LOC102168236
4	7635499	7735499	0.376094	LOC102186385
4	7635499	7735499	0.376094	LOC106501766
4	7635499	7735499	0.376094	LOC102168236

4	7635529	7735529	0.385365	LOC102186385
4	7635529	7735529	0.385365	LOC106501766
4	7635529	7735529	0.385365	LOC102168236
4	7635536	7735536	0.384014	LOC102186385
4	7635536	7735536	0.384014	LOC106501766
4	7635536	7735536	0.384014	LOC102168236
4	7635601	7735601	0.401582	LOC102186385
4	7635601	7735601	0.401582	LOC106501766
4	7635601	7735601	0.401582	LOC102168236
4	7635677	7735677	0.392212	LOC102186385
4	7635677	7735677	0.392212	LOC106501766
4	7635677	7735677	0.392212	LOC102168236
4	7635724	7735724	0.392212	LOC102186385
4	7635724	7735724	0.392212	LOC106501766
4	7635724	7735724	0.392212	LOC102168236
4	7635731	7735731	0.392212	LOC102186385
4	7635731	7735731	0.392212	LOC106501766
4	7635731	7735731	0.392212	LOC102168236
4	7635792	7735792	0.393416	LOC102186385
4	7635792	7735792	0.393416	LOC106501766
4	7635792	7735792	0.393416	LOC102168236
4	7635850	7735850	0.384917	LOC102186385
4	7635850	7735850	0.384917	LOC106501766
4	7635850	7735850	0.384917	LOC102168236
4	7635871	7735871	0.384917	LOC102186385
4	7635871	7735871	0.384917	LOC106501766
4	7635871	7735871	0.384917	LOC102168236
4	7635953	7735953	0.384917	LOC102186385
4	7635953	7735953	0.384917	LOC106501766
4	7635953	7735953	0.384917	LOC102168236
4	7636144	7736144	0.384917	LOC102186385
4	7636144	7736144	0.384917	LOC106501766
4	7636144	7736144	0.384917	LOC102168236
4	7636485	7736485	0.384917	LOC102186385
4	7636485	7736485	0.384917	LOC106501766
4	7636485	7736485	0.384917	LOC102168236
4	7636528	7736528	0.384917	LOC102186385
4	7636528	7736528	0.384917	LOC106501766
4	7636528	7736528	0.384917	LOC102168236
4	7636570	7736570	0.384917	LOC102186385
4	7636570	7736570	0.384917	LOC106501766
4	7636570	7736570	0.384917	LOC102168236
4	7636856	7736856	0.394019	LOC102186385
4	7636856	7736856	0.394019	LOC106501766
4	7636856	7736856	0.394019	LOC102168236
4	7636985	7736985	0.381968	LOC102186385
4	7636985	7736985	0.381968	LOC106501766
4	7636985	7736985	0.381968	LOC102168236
4	7636988	7736988	0.381968	LOC102186385
4	7636988	7736988	0.381968	LOC106501766
4	7636988	7736988	0.381968	LOC102168236
4	7636992	7736992	0.381968	LOC102186385
4	7636992	7736992	0.381968	LOC106501766
4	7636992	7736992	0.381968	LOC102168236
4	7637007	7737007	0.381968	LOC102186385
4	7637007	7737007	0.381968	LOC106501766
4	7637007	7737007	0.381968	LOC102168236
4	7637027	7737027	0.381968	LOC102186385

4	7637027	7737027	0.381968		LOC106501766
4	7637027	7737027	0.381968		LOC102168236
4	7637036	7737036	0.381968		LOC102186385
4	7637036	7737036	0.381968		LOC106501766
4	7637036	7737036	0.381968		LOC102168236
4	7637037	7737037	0.381968		LOC102186385
4	7637037	7737037	0.381968		LOC106501766
4	7637037	7737037	0.381968		LOC102168236
4	7637076	7737076	0.381968		LOC102186385
4	7637076	7737076	0.381968		LOC106501766
4	7637076	7737076	0.381968		LOC102168236
4	7637087	7737087	0.381968		LOC102186385
4	7637087	7737087	0.381968		LOC106501766
4	7637087	7737087	0.381968		LOC102168236
4	7637108	7737108	0.381968		LOC102186385
4	7637108	7737108	0.381968		LOC106501766
4	7637108	7737108	0.381968		LOC102168236
4	7637120	7737120	0.381968		LOC102186385
4	7637120	7737120	0.381968		LOC106501766
4	7637120	7737120	0.381968		LOC102168236
4	7637135	7737135	0.381968		LOC102186385
4	7637135	7737135	0.381968		LOC106501766
4	7637135	7737135	0.381968		LOC102168236
4	7637162	7737162	0.381968		LOC102186385
4	7637162	7737162	0.381968		LOC106501766
4	7637162	7737162	0.381968		LOC102168236
4	7637181	7737181	0.381968		LOC102186385
4	7637181	7737181	0.381968		LOC106501766
4	7637181	7737181	0.381968		LOC102168236
4	7637227	7737227	0.381968		LOC102186385
4	7637227	7737227	0.381968		LOC106501766
4	7637227	7737227	0.381968		LOC102168236
4	7637232	7737232	0.381968		LOC102186385
4	7637232	7737232	0.381968		LOC106501766
4	7637232	7737232	0.381968		LOC102168236
4	7637262	7737262	0.381968		LOC102186385
4	7637262	7737262	0.381968		LOC106501766
4	7637262	7737262	0.381968		LOC102168236
4	7637433	7737433	0.385278		LOC102186385
4	7637433	7737433	0.385278		LOC106501766
4	7637433	7737433	0.385278		LOC102168236
4	7637655	7737655	0.380403		LOC102186385
4	7637655	7737655	0.380403		LOC106501766
4	7637655	7737655	0.380403		LOC102168236
4	7637672	7737672	0.399145		LOC102186385
4	7637672	7737672	0.399145		LOC106501766
4	7637672	7737672	0.399145		LOC102168236
4	7637966	7737966	0.386092		LOC102186385
4	7637966	7737966	0.386092		LOC106501766
4	7637966	7737966	0.386092		LOC102168236
4	7638279	7738279	0.387119		LOC102186385
4	7638279	7738279	0.387119		LOC106501766
4	7638279	7738279	0.387119		LOC102168236
4	7638363	7738363	0.384647		LOC102186385
4	7638363	7738363	0.384647		LOC106501766
4	7638363	7738363	0.384647		LOC102168236
4	7638564	7738564	0.384782		LOC102186385
4	7638564	7738564	0.384782		LOC106501766

4	7638564	7738564	0.384782	LOC102168236
4	7638567	7738567	0.384782	LOC102186385
4	7638567	7738567	0.384782	LOC106501766
4	7638567	7738567	0.384782	LOC102168236
4	7638780	7738780	0.384812	LOC102186385
4	7638780	7738780	0.384812	LOC106501766
4	7638780	7738780	0.384812	LOC102168236
4	7639180	7739180	0.373644	LOC102186385
4	7639180	7739180	0.373644	LOC106501766
4	7639180	7739180	0.373644	LOC102168236
4	7639269	7739269	0.35026	LOC102186385
4	7639269	7739269	0.35026	LOC106501766
4	7639269	7739269	0.35026	LOC102168236
4	7639332	7739332	0.35026	LOC102186385
4	7639332	7739332	0.35026	LOC106501766
4	7639332	7739332	0.35026	LOC102168236
4	7639436	7739436	0.35026	LOC102186385
4	7639436	7739436	0.35026	LOC106501766
4	7639436	7739436	0.35026	LOC102168236
4	7639723	7739723	0.35026	LOC102186385
4	7639723	7739723	0.35026	LOC106501766
4	7639723	7739723	0.35026	LOC102168236
4	7639814	7739814	0.35026	LOC102186385
4	7639814	7739814	0.35026	LOC106501766
4	7639814	7739814	0.35026	LOC102168236
4	7640004	7740004	0.35026	LOC102186385
4	7640004	7740004	0.35026	LOC106501766
4	7640004	7740004	0.35026	LOC102168236
4	7640262	7740262	0.35026	LOC102186385
4	7640262	7740262	0.35026	LOC106501766
4	7640262	7740262	0.35026	LOC102168236
4	7640326	7740326	0.35026	LOC102186385
4	7640326	7740326	0.35026	LOC106501766
4	7640326	7740326	0.35026	LOC102168236
4	7640350	7740350	0.35026	LOC102186385
4	7640350	7740350	0.35026	LOC106501766
4	7640350	7740350	0.35026	LOC102168236
4	7640406	7740406	0.35026	LOC102186385
4	7640406	7740406	0.35026	LOC106501766
4	7640406	7740406	0.35026	LOC102168236
4	7640679	7740679	0.361974	LOC102186385
4	7640679	7740679	0.361974	LOC106501766
4	7640679	7740679	0.361974	LOC102168236
4	7640902	7740902	0.360757	LOC102186385
4	7640902	7740902	0.360757	LOC106501766
4	7640902	7740902	0.360757	LOC102168236
4	7640927	7740927	0.360757	LOC102186385
4	7640927	7740927	0.360757	LOC106501766
4	7640927	7740927	0.360757	LOC102168236
4	7641189	7741189	0.362062	LOC102186385
4	7641189	7741189	0.362062	LOC106501766
4	7641189	7741189	0.362062	LOC102168236
4	7641405	7741405	0.36416	LOC102186385
4	7641405	7741405	0.36416	LOC106501766
4	7641405	7741405	0.36416	LOC102168236
4	7662761	7762761	0.415383	LOC106501766
4	7662761	7762761	0.415383	LOC102168236
4	7662801	7762801	0.352834	LOC106501766

4	7662801	7762801	0.352834	LOC102168236
4	7662868	7762868	0.359122	LOC106501766
4	7662868	7762868	0.359122	LOC102168236
4	7662883	7762883	0.358793	LOC106501766
4	7662883	7762883	0.358793	LOC102168236
4	7662896	7762896	0.360866	LOC106501766
4	7662896	7762896	0.360866	LOC102168236
4	7662993	7762993	0.376586	LOC106501766
4	7662993	7762993	0.376586	LOC102168236
4	7663194	7763194	0.375094	LOC106501766
4	7663194	7763194	0.375094	LOC102168236
4	7663198	7763198	0.375094	LOC106501766
4	7663198	7763198	0.375094	LOC102168236
4	7663242	7763242	0.370172	LOC106501766
4	7663242	7763242	0.370172	LOC102168236
4	7663255	7763255	0.406153	LOC106501766
4	7663255	7763255	0.406153	LOC102168236
4	7663279	7763279	0.40148	LOC106501766
4	7663279	7763279	0.40148	LOC102168236
4	7663390	7763390	0.40148	LOC106501766
4	7663390	7763390	0.40148	LOC102168236
4	7663428	7763428	0.400304	LOC106501766
4	7663428	7763428	0.400304	LOC102168236
4	7663454	7763454	0.40629	LOC106501766
4	7663454	7763454	0.40629	LOC102168236
4	7663495	7763495	0.449407	LOC106501766
4	7663495	7763495	0.449407	LOC102168236
4	7663516	7763516	0.449407	LOC106501766
4	7663516	7763516	0.449407	LOC102168236
4	7663647	7763647	0.44115	LOC106501766
4	7663647	7763647	0.44115	LOC102168236
4	7663695	7763695	0.427729	LOC106501766
4	7663695	7763695	0.427729	LOC102168236
4	7663782	7763782	0.427822	LOC106501766
4	7663782	7763782	0.427822	LOC102168236
4	7663833	7763833	0.427736	LOC106501766
4	7663833	7763833	0.427736	LOC102168236
4	7664109	7764109	0.411819	LOC106501766
4	7664109	7764109	0.411819	LOC102168236
4	7664115	7764115	0.40837	LOC106501766
4	7664115	7764115	0.40837	LOC102168236
4	7664122	7764122	0.436231	LOC106501766
4	7664122	7764122	0.436231	LOC102168236
4	7664214	7764214	0.436231	LOC106501766
4	7664214	7764214	0.436231	LOC102168236
4	7664249	7764249	0.436231	LOC106501766
4	7664249	7764249	0.436231	LOC102168236
4	7664369	7764369	0.436231	LOC106501766
4	7664369	7764369	0.436231	LOC102168236
4	7664554	7764554	0.440729	LOC106501766
4	7664554	7764554	0.440729	LOC102168236
4	7664599	7764599	0.440044	LOC106501766
4	7664599	7764599	0.440044	LOC102168236
4	7664608	7764608	0.440044	LOC106501766
4	7664608	7764608	0.440044	LOC102168236
4	7664693	7764693	0.448413	LOC106501766
4	7664693	7764693	0.448413	LOC102168236
4	7664743	7764743	0.445065	LOC106501766

4	7664743	7764743	0.445065	LOC102168236
4	7664843	7764843	0.446367	LOC106501766
4	7664843	7764843	0.446367	LOC102168236
4	7664844	7764844	0.446367	LOC106501766
4	7664844	7764844	0.446367	LOC102168236
4	7664890	7764890	0.452116	LOC106501766
4	7664890	7764890	0.452116	LOC102168236
4	7664898	7764898	0.459262	LOC106501766
4	7664898	7764898	0.459262	LOC102168236
4	7664903	7764903	0.460204	LOC106501766
4	7664903	7764903	0.460204	LOC102168236
4	7664906	7764906	0.450387	LOC106501766
4	7664906	7764906	0.450387	LOC102168236
4	7664928	7764928	0.435196	LOC106501766
4	7664928	7764928	0.435196	LOC102168236
4	7664940	7764940	0.434678	LOC106501766
4	7664940	7764940	0.434678	LOC102168236
4	7665155	7765155	0.419157	LOC106501766
4	7665155	7765155	0.419157	LOC102168236
4	7665455	7765455	0.398068	LOC106501766
4	7665455	7765455	0.398068	LOC102168236
4	7665533	7765533	0.366353	LOC106501766
4	7665533	7765533	0.366353	LOC102168236
4	7665539	7765539	0.35254	LOC106501766
4	7665539	7765539	0.35254	LOC102168236
4	7665591	7765591	0.342353	LOC106501766
4	7665591	7765591	0.342353	LOC102168236
4	7666332	7766332	0.349404	LOC106501766
4	7666332	7766332	0.349404	LOC102168236
4	7666404	7766404	0.351683	LOC106501766
4	7666404	7766404	0.351683	LOC102168236
4	7667083	7767083	0.346863	LOC106501766
4	7667083	7767083	0.346863	LOC102168236
4	7667179	7767179	0.343473	LOC106501766
4	7667179	7767179	0.343473	LOC102168236
4	7667409	7767409	0.34698	LOC106501766
4	7667409	7767409	0.34698	LOC102168236
4	7667424	7767424	0.342105	LOC106501766
4	7667424	7767424	0.342105	LOC102168236
4	7668018	7768018	0.342694	LOC106501766
4	7668018	7768018	0.342694	LOC102168236
4	7668019	7768019	0.353791	LOC106501766
4	7668019	7768019	0.353791	LOC102168236
4	7668026	7768026	0.343528	LOC106501766
4	7668026	7768026	0.343528	LOC102168236
4	7668074	7768074	0.344347	LOC106501766
4	7668074	7768074	0.344347	LOC102168236
4	7668124	7768124	0.343899	LOC106501766
4	7668124	7768124	0.343899	LOC102168236
4	7668377	7768377	0.350228	LOC106501766
4	7668377	7768377	0.350228	LOC102168236
4	7686615	7786615	0.384307	LOC106501766
4	7686615	7786615	0.384307	GIMAP8
4	7686615	7786615	0.384307	LOC102168236
4	7687028	7787028	0.378561	LOC106501766
4	7687028	7787028	0.378561	GIMAP8
4	7687028	7787028	0.378561	LOC102168236
4	7687264	7787264	0.414883	LOC106501766

4	7687264	7787264	0.414883	GIMAP8
4	7687264	7787264	0.414883	LOC102168236
4	7687953	7787953	0.398864	LOC106501766
4	7687953	7787953	0.398864	GIMAP8
4	7687953	7787953	0.398864	LOC102168236
4	7688165	7788165	0.398864	LOC106501766
4	7688165	7788165	0.398864	GIMAP8
4	7688165	7788165	0.398864	LOC102168236
4	7688635	7788635	0.402252	LOC106501766
4	7688635	7788635	0.402252	GIMAP8
4	7688635	7788635	0.402252	LOC102168236
4	7688779	7788779	0.408141	LOC106501766
4	7688779	7788779	0.408141	GIMAP8
4	7688779	7788779	0.408141	LOC102168236
4	7688869	7788869	0.407753	LOC106501766
4	7688869	7788869	0.407753	GIMAP8
4	7688869	7788869	0.407753	LOC102168236
4	7688931	7788931	0.398698	LOC106501766
4	7688931	7788931	0.398698	GIMAP8
4	7688931	7788931	0.398698	LOC102168236
4	7688947	7788947	0.399456	LOC106501766
4	7688947	7788947	0.399456	GIMAP8
4	7688947	7788947	0.399456	LOC102168236
4	7689080	7789080	0.400114	LOC106501766
4	7689080	7789080	0.400114	GIMAP8
4	7689080	7789080	0.400114	LOC102168236
4	7689103	7789103	0.400114	LOC106501766
4	7689103	7789103	0.400114	GIMAP8
4	7689103	7789103	0.400114	LOC102168236
4	7689151	7789151	0.399927	LOC106501766
4	7689151	7789151	0.399927	GIMAP8
4	7689151	7789151	0.399927	LOC102168236
4	7689273	7789273	0.40503	LOC106501766
4	7689273	7789273	0.40503	GIMAP8
4	7689273	7789273	0.40503	LOC102168236
4	7689388	7789388	0.397007	LOC106501766
4	7689388	7789388	0.397007	GIMAP8
4	7689388	7789388	0.397007	LOC102168236
4	7689606	7789606	0.396087	LOC106501766
4	7689606	7789606	0.396087	GIMAP8
4	7689606	7789606	0.396087	LOC102168236
4	7689857	7789857	0.395626	LOC106501766
4	7689857	7789857	0.395626	GIMAP8
4	7689857	7789857	0.395626	LOC102168236
4	7690462	7790462	0.403623	LOC106501766
4	7690462	7790462	0.403623	GIMAP8
4	7690462	7790462	0.403623	LOC102168236
4	7690482	7790482	0.394072	LOC106501766
4	7690482	7790482	0.394072	GIMAP8
4	7690482	7790482	0.394072	LOC102168236
4	7690569	7790569	0.394874	LOC106501766
4	7690569	7790569	0.394874	GIMAP8
4	7690569	7790569	0.394874	LOC102168236
4	7690666	7790666	0.394874	LOC106501766
4	7690666	7790666	0.394874	GIMAP8
4	7690666	7790666	0.394874	LOC102168236
4	7690935	7790935	0.39472	LOC106501766
4	7690935	7790935	0.39472	GIMAP8

4	7690935	7790935	0.39472	LOC102168236
4	7691076	7791076	0.395623	LOC106501766
4	7691076	7791076	0.395623	GIMAP8
4	7691076	7791076	0.395623	LOC102168236
4	7691099	7791099	0.396004	LOC106501766
4	7691099	7791099	0.396004	GIMAP8
4	7691099	7791099	0.396004	LOC102168236
4	7691220	7791220	0.396131	LOC106501766
4	7691220	7791220	0.396131	GIMAP8
4	7691220	7791220	0.396131	LOC102168236
4	7691262	7791262	0.408704	LOC106501766
4	7691262	7791262	0.408704	GIMAP8
4	7691262	7791262	0.408704	LOC102168236
4	7691347	7791347	0.387969	LOC106501766
4	7691347	7791347	0.387969	GIMAP8
4	7691347	7791347	0.387969	LOC102168236
4	7691383	7791383	0.387969	LOC106501766
4	7691383	7791383	0.387969	GIMAP8
4	7691383	7791383	0.387969	LOC102168236
4	7691670	7791670	0.40708	LOC106501766
4	7691670	7791670	0.40708	GIMAP8
4	7691670	7791670	0.40708	LOC102168236
4	7691677	7791677	0.408283	LOC106501766
4	7691677	7791677	0.408283	GIMAP8
4	7691677	7791677	0.408283	LOC102168236
4	7691923	7791923	0.401335	LOC106501766
4	7691923	7791923	0.401335	GIMAP8
4	7691923	7791923	0.401335	LOC102168236
4	7692042	7792042	0.400718	LOC106501766
4	7692042	7792042	0.400718	GIMAP8
4	7692042	7792042	0.400718	LOC102168236
4	7692227	7792227	0.40212	LOC106501766
4	7692227	7792227	0.40212	GIMAP8
4	7692227	7792227	0.40212	LOC102168236
4	7692560	7792560	0.421773	LOC106501766
4	7692560	7792560	0.421773	GIMAP8
4	7692560	7792560	0.421773	LOC102168236
4	7692573	7792573	0.40993	LOC106501766
4	7692573	7792573	0.40993	GIMAP8
4	7692573	7792573	0.40993	LOC102168236
4	7692890	7792890	0.410901	LOC106501766
4	7692890	7792890	0.410901	GIMAP8
4	7692890	7792890	0.410901	LOC102168236
4	7692930	7792930	0.410901	LOC106501766
4	7692930	7792930	0.410901	GIMAP8
4	7692930	7792930	0.410901	LOC102168236
4	7693048	7793048	0.402249	LOC106501766
4	7693048	7793048	0.402249	GIMAP8
4	7693048	7793048	0.402249	LOC102168236
4	7693176	7793176	0.418679	LOC106501766
4	7693176	7793176	0.418679	GIMAP8
4	7693176	7793176	0.418679	LOC102168236
4	7693178	7793178	0.418679	LOC106501766
4	7693178	7793178	0.418679	GIMAP8
4	7693178	7793178	0.418679	LOC102168236
4	7693730	7793730	0.440191	LOC106501766
4	7693730	7793730	0.440191	GIMAP8
4	7693730	7793730	0.440191	LOC102168236

4	7693759	7793759	0.439422	LOC106501766
4	7693759	7793759	0.439422	GIMAP8
4	7693759	7793759	0.439422	LOC102168236
4	7693850	7793850	0.439422	LOC106501766
4	7693850	7793850	0.439422	GIMAP8
4	7693850	7793850	0.439422	LOC102168236
4	7693867	7793867	0.440239	LOC106501766
4	7693867	7793867	0.440239	GIMAP8
4	7693867	7793867	0.440239	LOC102168236
4	7694005	7794005	0.428975	LOC106501766
4	7694005	7794005	0.428975	GIMAP8
4	7694005	7794005	0.428975	LOC102168236
4	7694040	7794040	0.428975	LOC106501766
4	7694040	7794040	0.428975	GIMAP8
4	7694040	7794040	0.428975	LOC102168236
4	7694134	7794134	0.433376	LOC106501766
4	7694134	7794134	0.433376	GIMAP8
4	7694134	7794134	0.433376	LOC102168236
4	7694329	7794329	0.430823	LOC106501766
4	7694329	7794329	0.430823	GIMAP8
4	7694329	7794329	0.430823	LOC102168236
4	7694380	7794380	0.430981	LOC106501766
4	7694380	7794380	0.430981	GIMAP8
4	7694380	7794380	0.430981	LOC102168236
4	7694386	7794386	0.430981	LOC106501766
4	7694386	7794386	0.430981	GIMAP8
4	7694386	7794386	0.430981	LOC102168236
4	7694599	7794599	0.432944	LOC106501766
4	7694599	7794599	0.432944	GIMAP8
4	7694599	7794599	0.432944	LOC102168236
4	7694609	7794609	0.433827	LOC106501766
4	7694609	7794609	0.433827	GIMAP8
4	7694609	7794609	0.433827	LOC102168236
4	7694692	7794692	0.415795	LOC106501766
4	7694692	7794692	0.415795	GIMAP8
4	7694692	7794692	0.415795	LOC102168236
4	7694697	7794697	0.422073	LOC106501766
4	7694697	7794697	0.422073	GIMAP8
4	7694697	7794697	0.422073	LOC102168236
4	7694890	7794890	0.505769	LOC106501766
4	7694890	7794890	0.505769	GIMAP8
4	7694890	7794890	0.505769	LOC102168236
4	7694920	7794920	0.5048	LOC106501766
4	7694920	7794920	0.5048	GIMAP8
4	7694920	7794920	0.5048	LOC102168236
4	7694976	7794976	0.504763	LOC106501766
4	7694976	7794976	0.504763	GIMAP8
4	7694976	7794976	0.504763	LOC102168236
4	7695015	7795015	0.504763	LOC106501766
4	7695015	7795015	0.504763	GIMAP8
4	7695015	7795015	0.504763	LOC102168236
4	7695122	7795122	0.507091	LOC106501766
4	7695122	7795122	0.507091	GIMAP8
4	7695122	7795122	0.507091	LOC102168236
4	7695216	7795216	0.521318	LOC106501766
4	7695216	7795216	0.521318	GIMAP8
4	7695216	7795216	0.521318	LOC102168236
4	7695228	7795228	0.524138	LOC106501766

4	7695228	7795228	0.524138	GIMAP8
4	7695228	7795228	0.524138	LOC102168236
4	7695284	7795284	0.523052	LOC106501766
4	7695284	7795284	0.523052	GIMAP8
4	7695284	7795284	0.523052	LOC102168236
4	7695295	7795295	0.523052	LOC106501766
4	7695295	7795295	0.523052	GIMAP8
4	7695295	7795295	0.523052	LOC102168236
4	7695357	7795357	0.523546	LOC106501766
4	7695357	7795357	0.523546	GIMAP8
4	7695357	7795357	0.523546	LOC102168236
4	7695449	7795449	0.520949	LOC106501766
4	7695449	7795449	0.520949	GIMAP8
4	7695449	7795449	0.520949	LOC102168236
4	7695468	7795468	0.520949	LOC106501766
4	7695468	7795468	0.520949	GIMAP8
4	7695468	7795468	0.520949	LOC102168236
4	7695701	7795701	0.522537	LOC106501766
4	7695701	7795701	0.522537	GIMAP8
4	7695701	7795701	0.522537	LOC102168236
4	7695744	7795744	0.537804	LOC106501766
4	7695744	7795744	0.537804	GIMAP8
4	7695744	7795744	0.537804	LOC102168236
4	7695769	7795769	0.525393	LOC106501766
4	7695769	7795769	0.525393	GIMAP8
4	7695769	7795769	0.525393	LOC102168236
4	7695818	7795818	0.499263	LOC106501766
4	7695818	7795818	0.499263	GIMAP8
4	7695818	7795818	0.499263	LOC102168236
4	7695855	7795855	0.475462	LOC106501766
4	7695855	7795855	0.475462	GIMAP8
4	7695855	7795855	0.475462	LOC102168236
4	7695966	7795966	0.475298	LOC106501766
4	7695966	7795966	0.475298	GIMAP8
4	7695966	7795966	0.475298	LOC102168236
4	7695968	7795968	0.474056	LOC106501766
4	7695968	7795968	0.474056	GIMAP8
4	7695968	7795968	0.474056	LOC102168236
4	7695974	7795974	0.474056	LOC106501766
4	7695974	7795974	0.474056	GIMAP8
4	7695974	7795974	0.474056	LOC102168236
4	7695979	7795979	0.478569	LOC106501766
4	7695979	7795979	0.478569	GIMAP8
4	7695979	7795979	0.478569	LOC102168236
4	7696009	7796009	0.477287	LOC106501766
4	7696009	7796009	0.477287	GIMAP8
4	7696009	7796009	0.477287	LOC102168236
4	7696012	7796012	0.477287	LOC106501766
4	7696012	7796012	0.477287	GIMAP8
4	7696012	7796012	0.477287	LOC102168236
4	7696020	7796020	0.426988	LOC106501766
4	7696020	7796020	0.426988	GIMAP8
4	7696020	7796020	0.426988	LOC102168236
4	7696383	7796383	0.55375	LOC106501766
4	7696383	7796383	0.55375	GIMAP8
4	7696383	7796383	0.55375	LOC102168236
4	7696451	7796451	0.556174	LOC106501766
4	7696451	7796451	0.556174	GIMAP8

4	7696451	7796451	0.556174	LOC102168236
4	7696467	7796467	0.544858	LOC106501766
4	7696467	7796467	0.544858	GIMAP8
4	7696467	7796467	0.544858	LOC102168236
4	7696608	7796608	0.543371	LOC106501766
4	7696608	7796608	0.543371	GIMAP8
4	7696608	7796608	0.543371	LOC102168236
4	7696750	7796750	0.537051	LOC106501766
4	7696750	7796750	0.537051	GIMAP8
4	7696750	7796750	0.537051	LOC102168236
4	7696818	7796818	0.542475	LOC106501766
4	7696818	7796818	0.542475	GIMAP8
4	7696818	7796818	0.542475	LOC102168236
4	7696874	7796874	0.543146	LOC106501766
4	7696874	7796874	0.543146	GIMAP8
4	7696874	7796874	0.543146	LOC102168236
4	7696960	7796960	0.545858	LOC106501766
4	7696960	7796960	0.545858	GIMAP8
4	7696960	7796960	0.545858	LOC102168236
4	7697051	7797051	0.545384	LOC106501766
4	7697051	7797051	0.545384	GIMAP8
4	7697051	7797051	0.545384	LOC102168236
4	7697058	7797058	0.539629	LOC106501766
4	7697058	7797058	0.539629	GIMAP8
4	7697058	7797058	0.539629	LOC102168236
4	7697074	7797074	0.539522	LOC106501766
4	7697074	7797074	0.539522	GIMAP8
4	7697074	7797074	0.539522	LOC102168236
4	7697469	7797469	0.551519	LOC106501766
4	7697469	7797469	0.551519	GIMAP8
4	7697469	7797469	0.551519	LOC102168236
4	7697479	7797479	0.545046	LOC106501766
4	7697479	7797479	0.545046	GIMAP8
4	7697479	7797479	0.545046	LOC102168236
4	7697488	7797488	0.530032	LOC106501766
4	7697488	7797488	0.530032	GIMAP8
4	7697488	7797488	0.530032	LOC102168236
4	7697710	7797710	0.50523	LOC106501766
4	7697710	7797710	0.50523	GIMAP8
4	7697710	7797710	0.50523	LOC102168236
4	7697791	7797791	0.506482	LOC106501766
4	7697791	7797791	0.506482	GIMAP8
4	7697791	7797791	0.506482	LOC102168236
4	7697853	7797853	0.496834	LOC106501766
4	7697853	7797853	0.496834	GIMAP8
4	7697853	7797853	0.496834	LOC102168236
4	7698078	7798078	0.498345	LOC106501766
4	7698078	7798078	0.498345	GIMAP8
4	7698078	7798078	0.498345	LOC102168236
4	7698159	7798159	0.496653	LOC106501766
4	7698159	7798159	0.496653	GIMAP8
4	7698159	7798159	0.496653	LOC102168236
4	7698228	7798228	0.498106	LOC106501766
4	7698228	7798228	0.498106	GIMAP8
4	7698228	7798228	0.498106	LOC102168236
4	7698287	7798287	0.491042	LOC106501766
4	7698287	7798287	0.491042	GIMAP8
4	7698287	7798287	0.491042	LOC102168236

4	7698413	7798413	0.493024	LOC106501766
4	7698413	7798413	0.493024	GIMAP8
4	7698413	7798413	0.493024	LOC102168236
4	7698449	7798449	0.492327	LOC106501766
4	7698449	7798449	0.492327	GIMAP8
4	7698449	7798449	0.492327	LOC102168236
4	7698746	7798746	0.493294	LOC106501766
4	7698746	7798746	0.493294	GIMAP8
4	7698746	7798746	0.493294	LOC102168236
4	7698761	7798761	0.5016	LOC106501766
4	7698761	7798761	0.5016	GIMAP8
4	7698761	7798761	0.5016	LOC102168236
4	7698848	7798848	0.502076	LOC106501766
4	7698848	7798848	0.502076	GIMAP8
4	7698848	7798848	0.502076	LOC102168236
4	7699138	7799138	0.497043	LOC106501766
4	7699138	7799138	0.497043	GIMAP8
4	7699138	7799138	0.497043	LOC102168236
4	7699701	7799701	0.492368	LOC106501766
4	7699701	7799701	0.492368	GIMAP8
4	7699701	7799701	0.492368	LOC102168236
4	7699730	7799730	0.494749	LOC106501766
4	7699730	7799730	0.494749	GIMAP8
4	7699730	7799730	0.494749	LOC102168236
4	7699751	7799751	0.494749	LOC106501766
4	7699751	7799751	0.494749	GIMAP8
4	7699751	7799751	0.494749	LOC102168236
4	7699809	7799809	0.495673	LOC106501766
4	7699809	7799809	0.495673	GIMAP8
4	7699809	7799809	0.495673	LOC102168236
4	7699813	7799813	0.499911	LOC106501766
4	7699813	7799813	0.499911	GIMAP8
4	7699813	7799813	0.499911	LOC102168236
4	7699823	7799823	0.513878	LOC106501766
4	7699823	7799823	0.513878	GIMAP8
4	7699823	7799823	0.513878	LOC102168236
4	7699833	7799833	0.51354	LOC106501766
4	7699833	7799833	0.51354	GIMAP8
4	7699833	7799833	0.51354	LOC102168236
4	7699834	7799834	0.513328	LOC106501766
4	7699834	7799834	0.513328	GIMAP8
4	7699834	7799834	0.513328	LOC102168236
4	7699837	7799837	0.525446	LOC106501766
4	7699837	7799837	0.525446	GIMAP8
4	7699837	7799837	0.525446	LOC102168236
4	7700035	7800035	0.473096	LOC106501766
4	7700035	7800035	0.473096	GIMAP8
4	7700035	7800035	0.473096	LOC102168236
4	7700188	7800188	0.473371	LOC106501766
4	7700188	7800188	0.473371	GIMAP8
4	7700188	7800188	0.473371	LOC102168236
4	7700259	7800259	0.480262	LOC106501766
4	7700259	7800259	0.480262	GIMAP8
4	7700259	7800259	0.480262	LOC102168236
4	7700300	7800300	0.479872	LOC106501766
4	7700300	7800300	0.479872	GIMAP8
4	7700300	7800300	0.479872	LOC102168236
4	7700370	7800370	0.477723	LOC106501766

4	7700370	7800370	0.477723	GIMAP8
4	7700370	7800370	0.477723	LOC102168236
4	7700510	7800510	0.478131	LOC106501766
4	7700510	7800510	0.478131	GIMAP8
4	7700510	7800510	0.478131	LOC102168236
4	7700865	7800865	0.476164	LOC106501766
4	7700865	7800865	0.476164	GIMAP8
4	7700865	7800865	0.476164	LOC102168236
4	7700924	7800924	0.479643	LOC106501766
4	7700924	7800924	0.479643	GIMAP8
4	7700924	7800924	0.479643	LOC102168236
4	7701339	7801339	0.473284	LOC106501766
4	7701339	7801339	0.473284	GIMAP8
4	7701339	7801339	0.473284	LOC102168236
4	7701365	7801365	0.472467	LOC106501766
4	7701365	7801365	0.472467	GIMAP8
4	7701365	7801365	0.472467	LOC102168236
4	7701493	7801493	0.478224	LOC106501766
4	7701493	7801493	0.478224	GIMAP8
4	7701493	7801493	0.478224	LOC102168236
4	7702032	7802032	0.479906	LOC106501766
4	7702032	7802032	0.479906	GIMAP8
4	7702032	7802032	0.479906	LOC102168236
4	7702033	7802033	0.479906	LOC106501766
4	7702033	7802033	0.479906	GIMAP8
4	7702033	7802033	0.479906	LOC102168236
4	7702255	7802255	0.476817	LOC106501766
4	7702255	7802255	0.476817	GIMAP8
4	7702255	7802255	0.476817	LOC102168236
4	7702471	7802471	0.477085	LOC106501766
4	7702471	7802471	0.477085	GIMAP8
4	7702471	7802471	0.477085	LOC102168236
4	7702566	7802566	0.470942	LOC106501766
4	7702566	7802566	0.470942	GIMAP8
4	7702566	7802566	0.470942	LOC102168236
4	7702926	7802926	0.470942	LOC106501766
4	7702926	7802926	0.470942	GIMAP8
4	7702926	7802926	0.470942	LOC102168236
4	7702942	7802942	0.479549	LOC106501766
4	7702942	7802942	0.479549	GIMAP8
4	7702942	7802942	0.479549	LOC102168236
4	7703014	7803014	0.481192	LOC106501766
4	7703014	7803014	0.481192	GIMAP8
4	7703014	7803014	0.481192	LOC102168236
4	7703024	7803024	0.481192	LOC106501766
4	7703024	7803024	0.481192	GIMAP8
4	7703024	7803024	0.481192	LOC102168236
4	7703046	7803046	0.478708	LOC106501766
4	7703046	7803046	0.478708	GIMAP8
4	7703046	7803046	0.478708	LOC102168236
4	7703047	7803047	0.478708	LOC106501766
4	7703047	7803047	0.478708	GIMAP8
4	7703047	7803047	0.478708	LOC102168236
4	7703073	7803073	0.478708	LOC106501766
4	7703073	7803073	0.478708	GIMAP8
4	7703073	7803073	0.478708	LOC102168236
4	7703229	7803229	0.480468	LOC106501766
4	7703229	7803229	0.480468	GIMAP8

4	7703229	7803229	0.480468	LOC102168236
4	7703405	7803405	0.487948	LOC106501766
4	7703405	7803405	0.487948	GIMAP8
4	7703405	7803405	0.487948	LOC102168236
4	7703496	7803496	0.479105	LOC106501766
4	7703496	7803496	0.479105	GIMAP8
4	7703496	7803496	0.479105	LOC102168236
4	7703572	7803572	0.479105	LOC106501766
4	7703572	7803572	0.479105	GIMAP8
4	7703572	7803572	0.479105	LOC102168236
4	7703702	7803702	0.47459	LOC106501766
4	7703702	7803702	0.47459	GIMAP8
4	7703702	7803702	0.47459	LOC102168236
4	7703774	7803774	0.472222	LOC106501766
4	7703774	7803774	0.472222	GIMAP8
4	7703774	7803774	0.472222	LOC102168236
4	7704090	7804090	0.472377	LOC106501766
4	7704090	7804090	0.472377	GIMAP8
4	7704090	7804090	0.472377	LOC102168236
4	7704774	7804774	0.467289	LOC106501766
4	7704774	7804774	0.467289	GIMAP8
4	7704774	7804774	0.467289	LOC102168236
4	7704789	7804789	0.466595	LOC106501766
4	7704789	7804789	0.466595	GIMAP8
4	7704789	7804789	0.466595	LOC102168236
4	7704909	7804909	0.479165	LOC106501766
4	7704909	7804909	0.479165	GIMAP8
4	7704909	7804909	0.479165	LOC102168236
4	7705212	7805212	0.479791	LOC106501766
4	7705212	7805212	0.479791	GIMAP8
4	7705212	7805212	0.479791	LOC102168236
4	7705747	7805747	0.468059	LOC106501766
4	7705747	7805747	0.468059	GIMAP8
4	7705747	7805747	0.468059	LOC102168236
4	7705840	7805840	0.483878	LOC106501766
4	7705840	7805840	0.483878	GIMAP8
4	7705840	7805840	0.483878	LOC102168236
4	7705849	7805849	0.461008	LOC106501766
4	7705849	7805849	0.461008	GIMAP8
4	7705849	7805849	0.461008	LOC102168236
4	7706443	7806443	0.461161	LOC106501766
4	7706443	7806443	0.461161	GIMAP8
4	7706443	7806443	0.461161	LOC102168236
4	7706509	7806509	0.474464	LOC106501766
4	7706509	7806509	0.474464	GIMAP8
4	7706509	7806509	0.474464	LOC102168236
4	7706593	7806593	0.472296	LOC106501766
4	7706593	7806593	0.472296	GIMAP8
4	7706593	7806593	0.472296	LOC102168236
4	7706764	7806764	0.476342	LOC106501766
4	7706764	7806764	0.476342	GIMAP8
4	7706764	7806764	0.476342	LOC102168236
4	7706834	7806834	0.480134	LOC106501766
4	7706834	7806834	0.480134	GIMAP8
4	7706834	7806834	0.480134	LOC102168236
4	7707060	7807060	0.47244	LOC106501766
4	7707060	7807060	0.47244	GIMAP8
4	7707060	7807060	0.47244	LOC102168236

4	7707281	7807281	0.472592	LOC106501766
4	7707281	7807281	0.472592	GIMAP8
4	7707281	7807281	0.472592	LOC102168236
4	7707308	7807308	0.460791	LOC106501766
4	7707308	7807308	0.460791	GIMAP8
4	7707308	7807308	0.460791	LOC102168236
4	7707506	7807506	0.460765	LOC106501766
4	7707506	7807506	0.460765	GIMAP8
4	7707506	7807506	0.460765	LOC102168236
4	7707774	7807774	0.460765	LOC106501766
4	7707774	7807774	0.460765	GIMAP8
4	7707774	7807774	0.460765	LOC102168236
4	7708065	7808065	0.474868	LOC106501766
4	7708065	7808065	0.474868	GIMAP8
4	7708065	7808065	0.474868	LOC102168236
4	7708120	7808120	0.459639	LOC106501766
4	7708120	7808120	0.459639	GIMAP8
4	7708120	7808120	0.459639	LOC102168236
4	7708171	7808171	0.460725	LOC106501766
4	7708171	7808171	0.460725	GIMAP8
4	7708171	7808171	0.460725	LOC102168236
4	7708204	7808204	0.481842	LOC106501766
4	7708204	7808204	0.481842	GIMAP8
4	7708204	7808204	0.481842	LOC102168236
4	7709185	7809185	0.488891	LOC106501766
4	7709185	7809185	0.488891	GIMAP8
4	7709185	7809185	0.488891	LOC102168236
4	7709227	7809227	0.488891	LOC106501766
4	7709227	7809227	0.488891	GIMAP8
4	7709227	7809227	0.488891	LOC102168236
4	7709286	7809286	0.4825	LOC106501766
4	7709286	7809286	0.4825	GIMAP8
4	7709286	7809286	0.4825	LOC102168236
4	7709400	7809400	0.482716	LOC106501766
4	7709400	7809400	0.482716	GIMAP8
4	7709400	7809400	0.482716	LOC102168236
4	7709548	7809548	0.482773	LOC106501766
4	7709548	7809548	0.482773	GIMAP8
4	7709548	7809548	0.482773	LOC102168236
4	7709612	7809612	0.49024	LOC106501766
4	7709612	7809612	0.49024	GIMAP8
4	7709612	7809612	0.49024	LOC102168236
4	7709822	7809822	0.477338	LOC106501766
4	7709822	7809822	0.477338	GIMAP8
4	7709822	7809822	0.477338	LOC102168236
4	7710077	7810077	0.477338	LOC106501766
4	7710077	7810077	0.477338	GIMAP8
4	7710077	7810077	0.477338	LOC102168236
4	7710100	7810100	0.477444	LOC106501766
4	7710100	7810100	0.477444	GIMAP8
4	7710100	7810100	0.477444	LOC102168236
4	7710340	7810340	0.477331	LOC106501766
4	7710340	7810340	0.477331	GIMAP8
4	7710340	7810340	0.477331	LOC102168236
4	7710368	7810368	0.490084	LOC106501766
4	7710368	7810368	0.490084	GIMAP8
4	7710368	7810368	0.490084	LOC102168236
4	7710477	7810477	0.535016	LOC106501766

4	7710477	7810477	0.535016	GIMAP8
4	7710477	7810477	0.535016	LOC102168236
4	7710869	7810869	0.509098	LOC106501766
4	7710869	7810869	0.509098	GIMAP8
4	7710869	7810869	0.509098	LOC102168236
4	7710927	7810927	0.543385	LOC106501766
4	7710927	7810927	0.543385	GIMAP8
4	7710927	7810927	0.543385	LOC102168236
4	7710947	7810947	0.523052	LOC106501766
4	7710947	7810947	0.523052	GIMAP8
4	7710947	7810947	0.523052	LOC102168236
4	7711236	7811236	0.535593	LOC106501766
4	7711236	7811236	0.535593	GIMAP8
4	7711236	7811236	0.535593	LOC102168236
4	7711364	7811364	0.526195	LOC106501766
4	7711364	7811364	0.526195	GIMAP8
4	7711364	7811364	0.526195	LOC102168236
4	7712011	7812011	0.528714	LOC106501766
4	7712011	7812011	0.528714	GIMAP8
4	7712011	7812011	0.528714	LOC102168236
4	7712772	7812772	0.5372	LOC106501766
4	7712772	7812772	0.5372	GIMAP8
4	7712772	7812772	0.5372	LOC102168236
4	7712949	7812949	0.536783	LOC106501766
4	7712949	7812949	0.536783	GIMAP8
4	7712949	7812949	0.536783	LOC102168236
4	7713044	7813044	0.539969	LOC106501766
4	7713044	7813044	0.539969	GIMAP8
4	7713044	7813044	0.539969	LOC102168236
4	7713134	7813134	0.532546	LOC106501766
4	7713134	7813134	0.532546	GIMAP8
4	7713134	7813134	0.532546	LOC102168236
4	7713205	7813205	0.516549	LOC106501766
4	7713205	7813205	0.516549	GIMAP8
4	7713205	7813205	0.516549	LOC102168236
4	7713373	7813373	0.517389	LOC106501766
4	7713373	7813373	0.517389	GIMAP8
4	7713373	7813373	0.517389	LOC102168236
4	7713655	7813655	0.51679	LOC106501766
4	7713655	7813655	0.51679	GIMAP8
4	7713655	7813655	0.51679	LOC102168236
4	7713877	7813877	0.536017	LOC106501766
4	7713877	7813877	0.536017	GIMAP8
4	7713877	7813877	0.536017	LOC102168236
4	7714095	7814095	0.529346	LOC106501766
4	7714095	7814095	0.529346	GIMAP8
4	7714095	7814095	0.529346	LOC102168236
4	7714425	7814425	0.540713	LOC106501766
4	7714425	7814425	0.540713	GIMAP8
4	7714425	7814425	0.540713	LOC102168236
4	7714446	7814446	0.540305	LOC106501766
4	7714446	7814446	0.540305	GIMAP8
4	7714446	7814446	0.540305	LOC102168236
4	7715286	7815286	0.54542	LOC106501766
4	7715286	7815286	0.54542	GIMAP8
4	7715286	7815286	0.54542	LOC102168236
4	7715866	7815866	0.529834	GIMAP8
4	7715866	7815866	0.529834	LOC102168236

4	7715903	7815903	0.52989	GIMAP8
4	7715903	7815903	0.52989	LOC102168236
4	7716156	7816156	0.559916	GIMAP8
4	7716156	7816156	0.559916	LOC102168236
4	7716168	7816168	0.559916	GIMAP8
4	7716168	7816168	0.559916	LOC102168236
4	7716262	7816262	0.532992	GIMAP8
4	7716262	7816262	0.532992	LOC102168236
4	7716305	7816305	0.532992	GIMAP8
4	7716305	7816305	0.532992	LOC102168236
4	7716437	7816437	0.533314	GIMAP8
4	7716437	7816437	0.533314	LOC102168236
4	7716821	7816821	0.533097	GIMAP8
4	7716821	7816821	0.533097	LOC102168236
4	7717065	7817065	0.542079	GIMAP8
4	7717065	7817065	0.542079	LOC102168236
4	7717098	7817098	0.545103	GIMAP8
4	7717098	7817098	0.545103	LOC102168236
4	7717736	7817736	0.556047	GIMAP8
4	7717736	7817736	0.556047	LOC102168236
4	7717760	7817760	0.556095	GIMAP8
4	7717760	7817760	0.556095	LOC102168236
4	7718051	7818051	0.561897	GIMAP8
4	7718051	7818051	0.561897	LOC102168236
4	7718091	7818091	0.558907	GIMAP8
4	7718091	7818091	0.558907	LOC102168236
4	7718284	7818284	0.575382	GIMAP8
4	7718284	7818284	0.575382	LOC102168236
4	7718301	7818301	0.575609	GIMAP8
4	7718301	7818301	0.575609	LOC102168236
4	7718449	7818449	0.612503	GIMAP8
4	7718449	7818449	0.612503	LOC102168236
4	7718550	7818550	0.603601	GIMAP8
4	7718550	7818550	0.603601	LOC102168236
4	7718665	7818665	0.670694	GIMAP8
4	7718665	7818665	0.670694	LOC102168236
4	7719012	7819012	0.629578	GIMAP8
4	7719012	7819012	0.629578	LOC102168236
4	7719612	7819612	0.691913	GIMAP8
4	7719612	7819612	0.691913	LOC102168236
4	7719622	7819622	0.676396	GIMAP8
4	7719622	7819622	0.676396	LOC102168236
4	7719648	7819648	0.649958	GIMAP8
4	7719648	7819648	0.649958	LOC102168236
4	7719681	7819681	0.651887	GIMAP8
4	7719681	7819681	0.651887	LOC102168236
4	7719911	7819911	0.650508	GIMAP8
4	7719911	7819911	0.650508	LOC102168236
4	7719989	7819989	0.65081	GIMAP8
4	7719989	7819989	0.65081	LOC102168236
4	7720259	7820259	0.667971	GIMAP8
4	7720259	7820259	0.667971	LOC102168236
4	7720342	7820342	0.658222	GIMAP8
4	7720342	7820342	0.658222	LOC102168236
4	7720695	7820695	0.664386	GIMAP8
4	7720695	7820695	0.664386	LOC102168236
4	7721503	7821503	0.68703	GIMAP8
4	7721503	7821503	0.68703	LOC102168236

4	7721688	7821688	0.737449	GIMAP8
4	7721688	7821688	0.737449	LOC102168236
4	7721852	7821852	0.694382	GIMAP8
4	7721852	7821852	0.694382	LOC102168236
4	7721938	7821938	0.71698	GIMAP8
4	7721938	7821938	0.71698	LOC102168236
4	7722123	7822123	0.707871	GIMAP8
4	7722123	7822123	0.707871	LOC102168236
4	7722326	7822326	0.715219	GIMAP8
4	7722326	7822326	0.715219	LOC102168236
4	7722329	7822329	0.691463	GIMAP8
4	7722329	7822329	0.691463	LOC102168236
4	7722754	7822754	0.675687	GIMAP8
4	7722754	7822754	0.675687	LOC102168236
4	7723053	7823053	0.718857	GIMAP8
4	7723053	7823053	0.718857	LOC102168236
4	7723353	7823353	0.678382	GIMAP8
4	7723353	7823353	0.678382	LOC102168236
4	7723672	7823672	0.681555	GIMAP8
4	7723672	7823672	0.681555	LOC102168236
4	7723740	7823740	0.682252	GIMAP8
4	7723740	7823740	0.682252	LOC102168236
4	7723888	7823888	0.682252	GIMAP8
4	7723888	7823888	0.682252	LOC102168236
4	7723942	7823942	0.682252	GIMAP8
4	7723942	7823942	0.682252	LOC102168236
4	7724286	7824286	0.705909	GIMAP8
4	7724286	7824286	0.705909	LOC102168236
4	7724371	7824371	0.706656	GIMAP8
4	7724371	7824371	0.706656	LOC102168236
4	7724480	7824480	0.707205	GIMAP8
4	7724480	7824480	0.707205	LOC102168236
4	7724516	7824516	0.715736	GIMAP8
4	7724516	7824516	0.715736	LOC102168236
4	7724527	7824527	0.714884	GIMAP8
4	7724527	7824527	0.714884	LOC102168236
4	7724550	7824550	0.719698	GIMAP8
4	7724550	7824550	0.719698	LOC102168236
4	7724562	7824562	0.718106	GIMAP8
4	7724562	7824562	0.718106	LOC102168236
4	7724583	7824583	0.718127	GIMAP8
4	7724583	7824583	0.718127	LOC102168236
4	7724677	7824677	0.709922	GIMAP8
4	7724677	7824677	0.709922	LOC102168236
4	7724736	7824736	0.711283	GIMAP8
4	7724736	7824736	0.711283	LOC102168236
4	7724762	7824762	0.711129	GIMAP8
4	7724762	7824762	0.711129	LOC102168236
4	7724787	7824787	0.718559	GIMAP8
4	7724787	7824787	0.718559	LOC102168236
4	7724818	7824818	0.751461	GIMAP8
4	7724818	7824818	0.751461	LOC102168236
4	7724843	7824843	0.794814	GIMAP8
4	7724843	7824843	0.794814	LOC102168236
4	7724854	7824854	0.779609	GIMAP8
4	7724854	7824854	0.779609	LOC102168236
4	7724859	7824859	0.782156	GIMAP8
4	7724859	7824859	0.782156	LOC102168236

4	7724864	7824864	0.782775	GIMAP8
4	7724864	7824864	0.782775	LOC102168236
4	7724866	7824866	0.784023	GIMAP8
4	7724866	7824866	0.784023	LOC102168236
4	7724874	7824874	0.78546	GIMAP8
4	7724874	7824874	0.78546	LOC102168236
4	7724885	7824885	0.783598	GIMAP8
4	7724885	7824885	0.783598	LOC102168236
4	7724890	7824890	0.782806	GIMAP8
4	7724890	7824890	0.782806	LOC102168236
4	7724897	7824897	0.782806	GIMAP8
4	7724897	7824897	0.782806	LOC102168236
4	7724942	7824942	0.739859	GIMAP8
4	7724942	7824942	0.739859	LOC102168236
4	7724962	7824962	0.733274	GIMAP8
4	7724962	7824962	0.733274	LOC102168236
4	7724963	7824963	0.719119	GIMAP8
4	7724963	7824963	0.719119	LOC102168236
4	7724965	7824965	0.719119	GIMAP8
4	7724965	7824965	0.719119	LOC102168236
4	7725247	7825247	0.72055	GIMAP8
4	7725247	7825247	0.72055	LOC102168236
4	7725462	7825462	0.719696	GIMAP8
4	7725462	7825462	0.719696	LOC102168236
4	7725485	7825485	0.743751	GIMAP8
4	7725485	7825485	0.743751	LOC102168236
4	7725571	7825571	0.737856	GIMAP8
4	7725571	7825571	0.737856	LOC102168236
4	7725574	7825574	0.747485	GIMAP8
4	7725574	7825574	0.747485	LOC102168236
4	7725668	7825668	0.760166	GIMAP8
4	7725668	7825668	0.760166	LOC102168236
4	7726092	7826092	0.746495	GIMAP8
4	7726092	7826092	0.746495	LOC102168236
4	7726264	7826264	0.745594	GIMAP8
4	7726264	7826264	0.745594	LOC102168236
4	7726477	7826477	0.772315	GIMAP8
4	7726477	7826477	0.772315	LOC102168236
4	7726478	7826478	0.747815	GIMAP8
4	7726478	7826478	0.747815	LOC102168236
4	7726782	7826782	0.749144	GIMAP8
4	7726782	7826782	0.749144	LOC102168236
4	7727041	7827041	0.747636	GIMAP8
4	7727041	7827041	0.747636	LOC102168236
4	7727058	7827058	0.758688	GIMAP8
4	7727058	7827058	0.758688	LOC102168236
4	7727081	7827081	0.748402	GIMAP8
4	7727081	7827081	0.748402	LOC102168236
4	7727213	7827213	0.749193	GIMAP8
4	7727213	7827213	0.749193	LOC102168236
4	7727312	7827312	0.756317	GIMAP8
4	7727312	7827312	0.756317	LOC102168236
4	7727345	7827345	0.755593	GIMAP8
4	7727345	7827345	0.755593	LOC102168236
4	7727417	7827417	0.756803	GIMAP8
4	7727417	7827417	0.756803	LOC102168236
4	7727568	7827568	0.759737	GIMAP8
4	7727568	7827568	0.759737	LOC102168236

4	7727599	7827599	0.759097	GIMAP8
4	7727599	7827599	0.759097	LOC102168236
4	7727649	7827649	0.754477	GIMAP8
4	7727649	7827649	0.754477	LOC102168236
4	7728245	7828245	0.742952	GIMAP8
4	7728245	7828245	0.742952	LOC102168236
4	7728266	7828266	0.757111	GIMAP8
4	7728266	7828266	0.757111	LOC102168236
4	7728337	7828337	0.761519	GIMAP8
4	7728337	7828337	0.761519	LOC102168236
4	7728396	7828396	0.760172	GIMAP8
4	7728396	7828396	0.760172	LOC102168236
4	7728526	7828526	0.668661	GIMAP8
4	7728526	7828526	0.668661	LOC102168236
4	7728538	7828538	0.643265	GIMAP8
4	7728538	7828538	0.643265	LOC102168236
4	7728599	7828599	0.63919	GIMAP8
4	7728599	7828599	0.63919	LOC102168236
4	7728825	7828825	0.639635	GIMAP8
4	7728825	7828825	0.639635	LOC102168236
4	7728830	7828830	0.638866	GIMAP8
4	7728830	7828830	0.638866	LOC102168236
4	7728969	7828969	0.468465	GIMAP8
4	7728969	7828969	0.468465	LOC102168236
4	7729162	7829162	0.448282	GIMAP8
4	7729162	7829162	0.448282	LOC102168236
4	7729165	7829165	0.448282	GIMAP8
4	7729165	7829165	0.448282	LOC102168236
4	7729303	7829303	0.576548	GIMAP8
4	7729303	7829303	0.576548	LOC102168236
4	7730009	7830009	0.577236	GIMAP8
4	7730009	7830009	0.577236	LOC102168236
4	7730436	7830436	0.579205	GIMAP8
4	7730436	7830436	0.579205	LOC102168236
4	7730662	7830662	0.547575	GIMAP8
4	7730662	7830662	0.547575	LOC102168236
4	7730677	7830677	0.506819	GIMAP8
4	7730677	7830677	0.506819	LOC102168236
4	7732312	7832312	0.466712	GIMAP8
4	7732312	7832312	0.466712	LOC102168236
4	7732358	7832358	0.522543	GIMAP8
4	7732358	7832358	0.522543	LOC102168236
4	7733163	7833163	0.480478	GIMAP8
4	7733163	7833163	0.480478	LOC102168236
4	7733467	7833467	0.413073	GIMAP8
4	7733467	7833467	0.413073	LOC102168236
4	7734065	7834065	0.477876	GIMAP8
4	7734065	7834065	0.477876	LOC102168236
4	7734344	7834344	0.480917	GIMAP8
4	7734344	7834344	0.480917	LOC102168236
4	7734474	7834474	0.489555	GIMAP8
4	7734474	7834474	0.489555	LOC102168236
4	7734530	7834530	0.486225	GIMAP8
4	7734530	7834530	0.486225	LOC102168236
4	7734805	7834805	0.488192	GIMAP8
4	7734805	7834805	0.488192	LOC102168236
4	7735104	7835104	0.489182	GIMAP8
4	7735104	7835104	0.489182	LOC102168236

4	7735249	7835249	0.492625	GIMAP8
4	7735249	7835249	0.492625	LOC102168236
4	7735314	7835314	0.492625	GIMAP8
4	7735314	7835314	0.492625	LOC102168236
4	7735339	7835339	0.494648	GIMAP8
4	7735339	7835339	0.494648	LOC102168236
4	7735470	7835470	0.507876	GIMAP8
4	7735470	7835470	0.507876	LOC102168236
4	7735497	7835497	0.469076	GIMAP8
4	7735497	7835497	0.469076	LOC102168236
4	7735732	7835732	0.508163	GIMAP8
4	7735732	7835732	0.508163	LOC102168236
4	7735981	7835981	0.477915	GIMAP8
4	7735981	7835981	0.477915	LOC102168236
4	7735991	7835991	0.477915	GIMAP8
4	7735991	7835991	0.477915	LOC102168236
4	7736411	7836411	0.373054	GIMAP8
4	7736411	7836411	0.373054	LOC102168236
4	8317019	8417019	0.349552	LOC102189828
4	8317019	8417019	0.349552	ZNF783
4	8317019	8417019	0.349552	ZNF212
4	8317019	8417019	0.349552	ZNF282
4	8317019	8417019	0.349552	ZNF398
4	8317035	8417035	0.350276	LOC102189828
4	8317035	8417035	0.350276	ZNF783
4	8317035	8417035	0.350276	ZNF212
4	8317035	8417035	0.350276	ZNF282
4	8317035	8417035	0.350276	ZNF398
4	8317043	8417043	0.349406	LOC102189828
4	8317043	8417043	0.349406	ZNF783
4	8317043	8417043	0.349406	ZNF212
4	8317043	8417043	0.349406	ZNF282
4	8317043	8417043	0.349406	ZNF398
4	8718790	8818790	0.346223	CUL1
4	8718790	8818790	0.346223	LOC102189097
4	8724651	8824651	0.343332	CUL1
4	8724651	8824651	0.343332	LOC102189097
4	8727580	8827580	0.375965	CUL1
4	8727580	8827580	0.375965	LOC102189097
4	8728182	8828182	0.364045	CUL1
4	8728182	8828182	0.364045	LOC102189097
4	8728207	8828207	0.361775	CUL1
4	8728207	8828207	0.361775	LOC102189097
4	8728519	8828519	0.366928	CUL1
4	8728519	8828519	0.366928	LOC102189097
4	8728522	8828522	0.366928	CUL1
4	8728522	8828522	0.366928	LOC102189097
4	8728670	8828670	0.380458	CUL1
4	8728670	8828670	0.380458	LOC102189097
4	8728716	8828716	0.382031	CUL1
4	8728716	8828716	0.382031	LOC102189097
4	8728733	8828733	0.374043	CUL1
4	8728733	8828733	0.374043	LOC102189097
4	8728743	8828743	0.379654	CUL1
4	8728743	8828743	0.379654	LOC102189097
4	8728753	8828753	0.38084	CUL1
4	8728753	8828753	0.38084	LOC102189097
4	8728871	8828871	0.34627	CUL1

4	8728871	8828871	0.34627	LOC102189097
4	9728777	9828777	0.342109	CNTNAP2
4	9728812	9828812	0.436663	CNTNAP2
4	9728821	9828821	0.446137	CNTNAP2
4	9728865	9828865	0.446149	CNTNAP2
4	9728871	9828871	0.446149	CNTNAP2
4	9728905	9828905	0.453499	CNTNAP2
4	9728991	9828991	0.454096	CNTNAP2
4	9728993	9828993	0.405824	CNTNAP2
4	9729006	9829006	0.351383	CNTNAP2
4	9729017	9829017	0.346034	CNTNAP2
4	9729094	9829094	0.34426	CNTNAP2
4	9729102	9829102	0.342306	CNTNAP2
4	9729118	9829118	0.342306	CNTNAP2
4	9729119	9829119	0.342306	CNTNAP2
4	9729120	9829120	0.342306	CNTNAP2
4	9729132	9829132	0.342306	CNTNAP2
4	9729159	9829159	0.341976	CNTNAP2
4	9729171	9829171	0.341976	CNTNAP2
4	9729318	9829318	0.343623	CNTNAP2
4	9729374	9829374	0.346439	CNTNAP2
4	9729378	9829378	0.34877	CNTNAP2
4	9729379	9829379	0.34877	CNTNAP2
4	9729685	9829685	0.346582	CNTNAP2
4	9729691	9829691	0.365498	CNTNAP2
4	9832542	9932542	0.342233	CNTNAP2
4	9832548	9932548	0.346744	CNTNAP2
4	9832568	9932568	0.346744	CNTNAP2
4	9832739	9932739	0.343934	CNTNAP2
4	9832793	9932793	0.345514	CNTNAP2
4	9833112	9933112	0.345468	CNTNAP2
4	9833154	9933154	0.35484	CNTNAP2
4	9833226	9933226	0.35484	CNTNAP2
4	9833227	9933227	0.35484	CNTNAP2
4	9833239	9933239	0.354776	CNTNAP2
4	9833314	9933314	0.356486	CNTNAP2
4	9833320	9933320	0.355809	CNTNAP2
4	9833486	9933486	0.355979	CNTNAP2
4	9833489	9933489	0.35737	CNTNAP2
4	9833491	9933491	0.357212	CNTNAP2
4	9833562	9933562	0.355497	CNTNAP2
4	9833586	9933586	0.352622	CNTNAP2
4	9833597	9933597	0.353011	CNTNAP2
4	9833603	9933603	0.353652	CNTNAP2
4	9833614	9933614	0.353709	CNTNAP2
4	9833691	9933691	0.355204	CNTNAP2
4	9833694	9933694	0.359164	CNTNAP2
4	9833725	9933725	0.357663	CNTNAP2
4	9833752	9933752	0.35113	CNTNAP2
4	9833826	9933826	0.346327	CNTNAP2
4	9833954	9933954	0.341799	CNTNAP2
4	9847184	9947184	0.352141	CNTNAP2
4	9848258	9948258	0.356443	CNTNAP2
4	9849329	9949329	0.345755	CNTNAP2
4	9849756	9949756	0.343658	CNTNAP2
4	9849877	9949877	0.355939	CNTNAP2
4	9849902	9949902	0.358997	CNTNAP2
4	9849952	9949952	0.358997	CNTNAP2

4	9849984	9949984	0.345139	CNTNAP2
4	9849997	9949997	0.347912	CNTNAP2
4	9850002	9950002	0.346302	CNTNAP2
4	9850021	9950021	0.346302	CNTNAP2
4	9850022	9950022	0.346302	CNTNAP2
4	9850024	9950024	0.346302	CNTNAP2
4	9850087	9950087	0.346302	CNTNAP2
4	9850105	9950105	0.357668	CNTNAP2
4	9850120	9950120	0.343209	CNTNAP2
4	9850144	9950144	0.343911	CNTNAP2
4	10164013	10264013	0.396992	CNTNAP2
4	10164051	10264051	0.343584	CNTNAP2
4	10756985	10856985	0.36225	CNTNAP2
4	10757014	10857014	0.360156	CNTNAP2
4	13317812	13417812	0.341652	LOC102186371
4	13317812	13417812	0.341652	LOC102186088
4	13317812	13417812	0.341652	LOC102185809
4	13317812	13417812	0.341652	LOC102185534
4	13317812	13417812	0.341652	TRNAV-AAC-9
4	13318286	13418286	0.344057	LOC102186371
4	13318286	13418286	0.344057	LOC102186088
4	13318286	13418286	0.344057	LOC102185809
4	13318286	13418286	0.344057	LOC102185534
4	13318286	13418286	0.344057	TRNAV-AAC-9
4	13318287	13418287	0.344057	LOC102186371
4	13318287	13418287	0.344057	LOC102186088
4	13318287	13418287	0.344057	LOC102185809
4	13318287	13418287	0.344057	LOC102185534
4	13318287	13418287	0.344057	TRNAV-AAC-9
4	13318334	13418334	0.347471	LOC102186371
4	13318334	13418334	0.347471	LOC102186088
4	13318334	13418334	0.347471	LOC102185809
4	13318334	13418334	0.347471	LOC102185534
4	13318334	13418334	0.347471	TRNAV-AAC-9
4	13318335	13418335	0.346494	LOC102186371
4	13318335	13418335	0.346494	LOC102186088
4	13318335	13418335	0.346494	LOC102185809
4	13318335	13418335	0.346494	LOC102185534
4	13318335	13418335	0.346494	TRNAV-AAC-9
4	13318384	13418384	0.343691	LOC102186371
4	13318384	13418384	0.343691	LOC102186088
4	13318384	13418384	0.343691	LOC102185809
4	13318384	13418384	0.343691	LOC102185534
4	13318384	13418384	0.343691	TRNAV-AAC-9
4	13318423	13418423	0.342891	LOC102186371
4	13318423	13418423	0.342891	LOC102186088
4	13318423	13418423	0.342891	LOC102185809
4	13318423	13418423	0.342891	LOC102185534
4	13318423	13418423	0.342891	TRNAV-AAC-9
4	13318467	13418467	0.34487	LOC102186371
4	13318467	13418467	0.34487	LOC102186088
4	13318467	13418467	0.34487	LOC102185809
4	13318467	13418467	0.34487	LOC102185534
4	13318467	13418467	0.34487	TRNAV-AAC-9
4	13318497	13418497	0.350544	LOC102186371
4	13318497	13418497	0.350544	LOC102186088
4	13318497	13418497	0.350544	LOC102185809
4	13318497	13418497	0.350544	LOC102185534

4	13318497	13418497	0.350544	TRNAV-AAC-9
4	13318556	13418556	0.345455	LOC102186371
4	13318556	13418556	0.345455	LOC102186088
4	13318556	13418556	0.345455	LOC102185809
4	13318556	13418556	0.345455	LOC102185534
4	13318556	13418556	0.345455	TRNAV-AAC-9
4	13318562	13418562	0.342209	LOC102186371
4	13318562	13418562	0.342209	LOC102186088
4	13318562	13418562	0.342209	LOC102185809
4	13318562	13418562	0.342209	LOC102185534
4	13318562	13418562	0.342209	TRNAV-AAC-9
4	13318564	13418564	0.341979	LOC102186371
4	13318564	13418564	0.341979	LOC102186088
4	13318564	13418564	0.341979	LOC102185809
4	13318564	13418564	0.341979	LOC102185534
4	13318564	13418564	0.341979	TRNAV-AAC-9
4	13318861	13418861	0.346223	LOC102186371
4	13318861	13418861	0.346223	LOC102186088
4	13318861	13418861	0.346223	LOC102185809
4	13318861	13418861	0.346223	LOC102185534
4	13318861	13418861	0.346223	TRNAV-AAC-9
4	13318869	13418869	0.354793	LOC102186371
4	13318869	13418869	0.354793	LOC102186088
4	13318869	13418869	0.354793	LOC102185809
4	13318869	13418869	0.354793	LOC102185534
4	13318869	13418869	0.354793	TRNAV-AAC-9
4	13318897	13418897	0.347433	LOC102186371
4	13318897	13418897	0.347433	LOC102186088
4	13318897	13418897	0.347433	LOC102185809
4	13318897	13418897	0.347433	LOC102185534
4	13318897	13418897	0.347433	TRNAV-AAC-9
4	13318961	13418961	0.344776	LOC102186371
4	13318961	13418961	0.344776	LOC102186088
4	13318961	13418961	0.344776	LOC102185809
4	13318961	13418961	0.344776	LOC102185534
4	13318961	13418961	0.344776	TRNAV-AAC-9
4	13318972	13418972	0.344776	LOC102186371
4	13318972	13418972	0.344776	LOC102186088
4	13318972	13418972	0.344776	LOC102185809
4	13318972	13418972	0.344776	LOC102185534
4	13318972	13418972	0.344776	TRNAV-AAC-9
4	13319165	13419165	0.344776	LOC102186371
4	13319165	13419165	0.344776	LOC102186088
4	13319165	13419165	0.344776	LOC102185809
4	13319165	13419165	0.344776	LOC102185534
4	13319165	13419165	0.344776	TRNAV-AAC-9
4	13319192	13419192	0.344932	LOC102186371
4	13319192	13419192	0.344932	LOC102186088
4	13319192	13419192	0.344932	LOC102185809
4	13319192	13419192	0.344932	LOC102185534
4	13319192	13419192	0.344932	TRNAV-AAC-9
4	13319291	13419291	0.344932	LOC102186371
4	13319291	13419291	0.344932	LOC102186088
4	13319291	13419291	0.344932	LOC102185809
4	13319291	13419291	0.344932	LOC102185534
4	13319291	13419291	0.344932	TRNAV-AAC-9
4	13319378	13419378	0.346772	LOC102186371
4	13319378	13419378	0.346772	LOC102186088

4	13319378	13419378	0.346772	LOC102185809
4	13319378	13419378	0.346772	LOC102185534
4	13319378	13419378	0.346772	TRNAV-AAC-9
4	13319411	13419411	0.343056	LOC102186371
4	13319411	13419411	0.343056	LOC102186088
4	13319411	13419411	0.343056	LOC102185809
4	13319411	13419411	0.343056	LOC102185534
4	13319411	13419411	0.343056	TRNAV-AAC-9
4	13319426	13419426	0.355034	LOC102186371
4	13319426	13419426	0.355034	LOC102186088
4	13319426	13419426	0.355034	LOC102185809
4	13319426	13419426	0.355034	LOC102185534
4	13319426	13419426	0.355034	TRNAV-AAC-9
4	13319432	13419432	0.354423	LOC102186371
4	13319432	13419432	0.354423	LOC102186088
4	13319432	13419432	0.354423	LOC102185809
4	13319432	13419432	0.354423	LOC102185534
4	13319432	13419432	0.354423	TRNAV-AAC-9
4	13319516	13419516	0.352309	LOC102186371
4	13319516	13419516	0.352309	LOC102186088
4	13319516	13419516	0.352309	LOC102185809
4	13319516	13419516	0.352309	LOC102185534
4	13319516	13419516	0.352309	TRNAV-AAC-9
4	13319565	13419565	0.352535	LOC102186371
4	13319565	13419565	0.352535	LOC102186088
4	13319565	13419565	0.352535	LOC102185809
4	13319565	13419565	0.352535	LOC102185534
4	13319565	13419565	0.352535	TRNAV-AAC-9
4	13319573	13419573	0.353881	LOC102186371
4	13319573	13419573	0.353881	LOC102186088
4	13319573	13419573	0.353881	LOC102185809
4	13319573	13419573	0.353881	LOC102185534
4	13319573	13419573	0.353881	TRNAV-AAC-9
4	13319604	13419604	0.349329	LOC102186371
4	13319604	13419604	0.349329	LOC102186088
4	13319604	13419604	0.349329	LOC102185809
4	13319604	13419604	0.349329	LOC102185534
4	13319604	13419604	0.349329	TRNAV-AAC-9
4	13319608	13419608	0.349329	LOC102186371
4	13319608	13419608	0.349329	LOC102186088
4	13319608	13419608	0.349329	LOC102185809
4	13319608	13419608	0.349329	LOC102185534
4	13319608	13419608	0.349329	TRNAV-AAC-9
4	13319620	13419620	0.349329	LOC102186371
4	13319620	13419620	0.349329	LOC102186088
4	13319620	13419620	0.349329	LOC102185809
4	13319620	13419620	0.349329	LOC102185534
4	13319620	13419620	0.349329	TRNAV-AAC-9
4	13319701	13419701	0.349329	LOC102186371
4	13319701	13419701	0.349329	LOC102186088
4	13319701	13419701	0.349329	LOC102185809
4	13319701	13419701	0.349329	LOC102185534
4	13319701	13419701	0.349329	TRNAV-AAC-9
4	13319793	13419793	0.348165	LOC102186371
4	13319793	13419793	0.348165	LOC102186088
4	13319793	13419793	0.348165	LOC102185809
4	13319793	13419793	0.348165	LOC102185534
4	13319793	13419793	0.348165	TRNAV-AAC-9

4	13320260	13420260	0.345178	LOC102186371
4	13320260	13420260	0.345178	LOC102186088
4	13320260	13420260	0.345178	LOC102185809
4	13320260	13420260	0.345178	LOC102185534
4	13320260	13420260	0.345178	TRNAV-AAC-9
4	13320272	13420272	0.347387	LOC102186371
4	13320272	13420272	0.347387	LOC102186088
4	13320272	13420272	0.347387	LOC102185809
4	13320272	13420272	0.347387	LOC102185534
4	13320272	13420272	0.347387	TRNAV-AAC-9
4	13320277	13420277	0.344857	LOC102186371
4	13320277	13420277	0.344857	LOC102186088
4	13320277	13420277	0.344857	LOC102185809
4	13320277	13420277	0.344857	LOC102185534
4	13320277	13420277	0.344857	TRNAV-AAC-9
4	13320309	13420309	0.346252	LOC102186371
4	13320309	13420309	0.346252	LOC102186088
4	13320309	13420309	0.346252	LOC102185809
4	13320309	13420309	0.346252	LOC102185534
4	13320309	13420309	0.346252	TRNAV-AAC-9
4	13320485	13420485	0.343786	LOC102186371
4	13320485	13420485	0.343786	LOC102186088
4	13320485	13420485	0.343786	LOC102185809
4	13320485	13420485	0.343786	LOC102185534
4	13320485	13420485	0.343786	TRNAV-AAC-9
4	13320753	13420753	0.343762	LOC102186371
4	13320753	13420753	0.343762	LOC102186088
4	13320753	13420753	0.343762	LOC102185809
4	13320753	13420753	0.343762	LOC102185534
4	13320753	13420753	0.343762	TRNAV-AAC-9
4	13797364	13897364	0.367646	FAM131B
4	13797364	13897364	0.367646	EPHA1
4	13797364	13897364	0.367646	ZYX
4	13797364	13897364	0.367646	CLCN1
4	13797771	13897771	0.369028	FAM131B
4	13797771	13897771	0.369028	EPHA1
4	13797771	13897771	0.369028	ZYX
4	13797771	13897771	0.369028	CLCN1
4	13797991	13897991	0.404377	FAM131B
4	13797991	13897991	0.404377	EPHA1
4	13797991	13897991	0.404377	ZYX
4	13797991	13897991	0.404377	CLCN1
4	13798003	13898003	0.413797	FAM131B
4	13798003	13898003	0.413797	EPHA1
4	13798003	13898003	0.413797	ZYX
4	13798003	13898003	0.413797	CLCN1
4	13798298	13898298	0.360519	FAM131B
4	13798298	13898298	0.360519	EPHA1
4	13798298	13898298	0.360519	ZYX
4	13798298	13898298	0.360519	CLCN1
4	13798298	13898298	0.360519	CASP2
4	13798431	13898431	0.364308	FAM131B
4	13798431	13898431	0.364308	EPHA1
4	13798431	13898431	0.364308	ZYX
4	13798431	13898431	0.364308	CLCN1
4	13798431	13898431	0.364308	CASP2
4	13798496	13898496	0.412252	FAM131B
4	13798496	13898496	0.412252	EPHA1

4	13798496	13898496	0.412252	ZYX
4	13798496	13898496	0.412252	CLCN1
4	13798496	13898496	0.412252	CASP2
4	13798745	13898745	0.39626	FAM131B
4	13798745	13898745	0.39626	EPHA1
4	13798745	13898745	0.39626	ZYX
4	13798745	13898745	0.39626	CLCN1
4	13798745	13898745	0.39626	CASP2
4	13799005	13899005	0.405309	FAM131B
4	13799005	13899005	0.405309	EPHA1
4	13799005	13899005	0.405309	ZYX
4	13799005	13899005	0.405309	CLCN1
4	13799005	13899005	0.405309	CASP2
4	13799048	13899048	0.405309	FAM131B
4	13799048	13899048	0.405309	EPHA1
4	13799048	13899048	0.405309	ZYX
4	13799048	13899048	0.405309	CLCN1
4	13799048	13899048	0.405309	CASP2
4	13799280	13899280	0.398047	FAM131B
4	13799280	13899280	0.398047	EPHA1
4	13799280	13899280	0.398047	ZYX
4	13799280	13899280	0.398047	CLCN1
4	13799280	13899280	0.398047	CASP2
4	13799645	13899645	0.398047	FAM131B
4	13799645	13899645	0.398047	EPHA1
4	13799645	13899645	0.398047	ZYX
4	13799645	13899645	0.398047	CLCN1
4	13799645	13899645	0.398047	CASP2
4	13799659	13899659	0.413481	FAM131B
4	13799659	13899659	0.413481	EPHA1
4	13799659	13899659	0.413481	ZYX
4	13799659	13899659	0.413481	CLCN1
4	13799659	13899659	0.413481	CASP2
4	13799677	13899677	0.394799	FAM131B
4	13799677	13899677	0.394799	EPHA1
4	13799677	13899677	0.394799	ZYX
4	13799677	13899677	0.394799	CLCN1
4	13799677	13899677	0.394799	CASP2
4	13800014	13900014	0.421156	FAM131B
4	13800014	13900014	0.421156	EPHA1
4	13800014	13900014	0.421156	ZYX
4	13800014	13900014	0.421156	CLCN1
4	13800014	13900014	0.421156	CASP2
4	13800184	13900184	0.407699	FAM131B
4	13800184	13900184	0.407699	EPHA1
4	13800184	13900184	0.407699	ZYX
4	13800184	13900184	0.407699	CLCN1
4	13800184	13900184	0.407699	CASP2
4	13800245	13900245	0.442558	FAM131B
4	13800245	13900245	0.442558	EPHA1
4	13800245	13900245	0.442558	ZYX
4	13800245	13900245	0.442558	CLCN1
4	13800245	13900245	0.442558	CASP2
4	13800274	13900274	0.419704	FAM131B
4	13800274	13900274	0.419704	EPHA1
4	13800274	13900274	0.419704	ZYX
4	13800274	13900274	0.419704	CLCN1
4	13800274	13900274	0.419704	CASP2

4	13800370	13900370	0.419704	FAM131B
4	13800370	13900370	0.419704	EPHA1
4	13800370	13900370	0.419704	ZYX
4	13800370	13900370	0.419704	CLCN1
4	13800370	13900370	0.419704	CASP2
4	13800426	13900426	0.421144	FAM131B
4	13800426	13900426	0.421144	EPHA1
4	13800426	13900426	0.421144	ZYX
4	13800426	13900426	0.421144	CLCN1
4	13800426	13900426	0.421144	CASP2
4	13800663	13900663	0.414319	FAM131B
4	13800663	13900663	0.414319	EPHA1
4	13800663	13900663	0.414319	ZYX
4	13800663	13900663	0.414319	CLCN1
4	13800663	13900663	0.414319	CASP2
4	13800830	13900830	0.432151	FAM131B
4	13800830	13900830	0.432151	EPHA1
4	13800830	13900830	0.432151	ZYX
4	13800830	13900830	0.432151	CLCN1
4	13800830	13900830	0.432151	CASP2
4	13800931	13900931	0.412882	FAM131B
4	13800931	13900931	0.412882	EPHA1
4	13800931	13900931	0.412882	ZYX
4	13800931	13900931	0.412882	CLCN1
4	13800931	13900931	0.412882	CASP2
4	13801252	13901252	0.412856	FAM131B
4	13801252	13901252	0.412856	EPHA1
4	13801252	13901252	0.412856	ZYX
4	13801252	13901252	0.412856	CLCN1
4	13801252	13901252	0.412856	CASP2
4	13801311	13901311	0.436851	FAM131B
4	13801311	13901311	0.436851	EPHA1
4	13801311	13901311	0.436851	ZYX
4	13801311	13901311	0.436851	CLCN1
4	13801311	13901311	0.436851	CASP2
4	13801313	13901313	0.436851	FAM131B
4	13801313	13901313	0.436851	EPHA1
4	13801313	13901313	0.436851	ZYX
4	13801313	13901313	0.436851	CLCN1
4	13801313	13901313	0.436851	CASP2
4	13801434	13901434	0.40692	FAM131B
4	13801434	13901434	0.40692	EPHA1
4	13801434	13901434	0.40692	ZYX
4	13801434	13901434	0.40692	CLCN1
4	13801434	13901434	0.40692	CASP2
4	13801613	13901613	0.40692	FAM131B
4	13801613	13901613	0.40692	EPHA1
4	13801613	13901613	0.40692	ZYX
4	13801613	13901613	0.40692	CLCN1
4	13801613	13901613	0.40692	CASP2
4	13801627	13901627	0.384566	FAM131B
4	13801627	13901627	0.384566	EPHA1
4	13801627	13901627	0.384566	ZYX
4	13801627	13901627	0.384566	CLCN1
4	13801627	13901627	0.384566	CASP2
4	13801699	13901699	0.375268	FAM131B
4	13801699	13901699	0.375268	EPHA1
4	13801699	13901699	0.375268	ZYX

4	13801699	13901699	0.375268	CLCN1
4	13801699	13901699	0.375268	CASP2
4	13802061	13902061	0.37023	FAM131B
4	13802061	13902061	0.37023	EPHA1
4	13802061	13902061	0.37023	ZYX
4	13802061	13902061	0.37023	CLCN1
4	13802061	13902061	0.37023	CASP2
4	13802078	13902078	0.380973	FAM131B
4	13802078	13902078	0.380973	EPHA1
4	13802078	13902078	0.380973	ZYX
4	13802078	13902078	0.380973	CLCN1
4	13802078	13902078	0.380973	CASP2
4	13802152	13902152	0.379687	FAM131B
4	13802152	13902152	0.379687	EPHA1
4	13802152	13902152	0.379687	ZYX
4	13802152	13902152	0.379687	CLCN1
4	13802152	13902152	0.379687	CASP2
4	13802350	13902350	0.380449	FAM131B
4	13802350	13902350	0.380449	EPHA1
4	13802350	13902350	0.380449	ZYX
4	13802350	13902350	0.380449	CLCN1
4	13802350	13902350	0.380449	CASP2
4	13802632	13902632	0.371263	FAM131B
4	13802632	13902632	0.371263	EPHA1
4	13802632	13902632	0.371263	ZYX
4	13802632	13902632	0.371263	CLCN1
4	13802632	13902632	0.371263	CASP2
4	13802728	13902728	0.411214	FAM131B
4	13802728	13902728	0.411214	EPHA1
4	13802728	13902728	0.411214	ZYX
4	13802728	13902728	0.411214	CLCN1
4	13802728	13902728	0.411214	CASP2
4	13803013	13903013	0.429209	FAM131B
4	13803013	13903013	0.429209	EPHA1
4	13803013	13903013	0.429209	ZYX
4	13803013	13903013	0.429209	CLCN1
4	13803013	13903013	0.429209	CASP2
4	13803387	13903387	0.42091	FAM131B
4	13803387	13903387	0.42091	EPHA1
4	13803387	13903387	0.42091	ZYX
4	13803387	13903387	0.42091	CLCN1
4	13803387	13903387	0.42091	CASP2
4	13803388	13903388	0.455727	FAM131B
4	13803388	13903388	0.455727	EPHA1
4	13803388	13903388	0.455727	ZYX
4	13803388	13903388	0.455727	CLCN1
4	13803388	13903388	0.455727	CASP2
4	13803692	13903692	0.40241	FAM131B
4	13803692	13903692	0.40241	EPHA1
4	13803692	13903692	0.40241	ZYX
4	13803692	13903692	0.40241	CLCN1
4	13803692	13903692	0.40241	CASP2
4	13803696	13903696	0.403218	FAM131B
4	13803696	13903696	0.403218	EPHA1
4	13803696	13903696	0.403218	ZYX
4	13803696	13903696	0.403218	CLCN1
4	13803696	13903696	0.403218	CASP2
4	13803751	13903751	0.403218	FAM131B

4	13803751	13903751	0.403218	EPHA1
4	13803751	13903751	0.403218	ZYX
4	13803751	13903751	0.403218	CLCN1
4	13803751	13903751	0.403218	CASP2
4	13803822	13903822	0.388787	FAM131B
4	13803822	13903822	0.388787	EPHA1
4	13803822	13903822	0.388787	ZYX
4	13803822	13903822	0.388787	CLCN1
4	13803822	13903822	0.388787	CASP2
4	13803905	13903905	0.388787	FAM131B
4	13803905	13903905	0.388787	EPHA1
4	13803905	13903905	0.388787	ZYX
4	13803905	13903905	0.388787	CLCN1
4	13803905	13903905	0.388787	CASP2
4	13804234	13904234	0.394209	FAM131B
4	13804234	13904234	0.394209	EPHA1
4	13804234	13904234	0.394209	ZYX
4	13804234	13904234	0.394209	CLCN1
4	13804234	13904234	0.394209	CASP2
4	13804278	13904278	0.380401	FAM131B
4	13804278	13904278	0.380401	EPHA1
4	13804278	13904278	0.380401	ZYX
4	13804278	13904278	0.380401	CLCN1
4	13804278	13904278	0.380401	CASP2
4	13804939	13904939	0.380035	FAM131B
4	13804939	13904939	0.380035	EPHA1
4	13804939	13904939	0.380035	ZYX
4	13804939	13904939	0.380035	CLCN1
4	13804939	13904939	0.380035	CASP2
4	13805104	13905104	0.397427	FAM131B
4	13805104	13905104	0.397427	EPHA1
4	13805104	13905104	0.397427	ZYX
4	13805104	13905104	0.397427	CLCN1
4	13805104	13905104	0.397427	CASP2
4	13805107	13905107	0.36843	FAM131B
4	13805107	13905107	0.36843	EPHA1
4	13805107	13905107	0.36843	ZYX
4	13805107	13905107	0.36843	CLCN1
4	13805107	13905107	0.36843	CASP2
4	13805411	13905411	0.369957	FAM131B
4	13805411	13905411	0.369957	EPHA1
4	13805411	13905411	0.369957	ZYX
4	13805411	13905411	0.369957	CLCN1
4	13805411	13905411	0.369957	CASP2
4	13805636	13905636	0.370705	FAM131B
4	13805636	13905636	0.370705	EPHA1
4	13805636	13905636	0.370705	ZYX
4	13805636	13905636	0.370705	CLCN1
4	13805636	13905636	0.370705	CASP2
4	13805666	13905666	0.357548	FAM131B
4	13805666	13905666	0.357548	EPHA1
4	13805666	13905666	0.357548	ZYX
4	13805666	13905666	0.357548	CLCN1
4	13805666	13905666	0.357548	CASP2
4	13805973	13905973	0.376652	FAM131B
4	13805973	13905973	0.376652	EPHA1
4	13805973	13905973	0.376652	ZYX
4	13805973	13905973	0.376652	CLCN1

4	13805973	13905973	0.376652	CASP2
4	13806050	13906050	0.363867	FAM131B
4	13806050	13906050	0.363867	EPHA1
4	13806050	13906050	0.363867	ZYX
4	13806050	13906050	0.363867	CLCN1
4	13806050	13906050	0.363867	CASP2
4	13806125	13906125	0.363867	FAM131B
4	13806125	13906125	0.363867	EPHA1
4	13806125	13906125	0.363867	ZYX
4	13806125	13906125	0.363867	CLCN1
4	13806125	13906125	0.363867	CASP2
4	13806240	13906240	0.347517	FAM131B
4	13806240	13906240	0.347517	EPHA1
4	13806240	13906240	0.347517	ZYX
4	13806240	13906240	0.347517	CLCN1
4	13806240	13906240	0.347517	CASP2
4	13807101	13907101	0.346621	FAM131B
4	13807101	13907101	0.346621	ZYX
4	13807101	13907101	0.346621	CLCN1
4	13807101	13907101	0.346621	CASP2
4	13807177	13907177	0.348471	FAM131B
4	13807177	13907177	0.348471	ZYX
4	13807177	13907177	0.348471	CLCN1
4	13807177	13907177	0.348471	CASP2
4	13807449	13907449	0.351845	FAM131B
4	13807449	13907449	0.351845	ZYX
4	13807449	13907449	0.351845	CLCN1
4	13807449	13907449	0.351845	CASP2
4	14151626	14251626	0.353361	LOC102183208
4	14151626	14251626	0.353361	LOC108635908
4	14151626	14251626	0.353361	LOC108635909
4	14151626	14251626	0.353361	LOC102181187
4	14151626	14251626	0.353361	LOC102181470
4	15148167	15248167	0.345757	TAS2R38
4	15148167	15248167	0.345757	CLEC5A
4	15148167	15248167	0.345757	MGAM
4	19321469	19421469	0.343209	PTN
4	19321469	19421469	0.343209	DGKI
4	19321542	19421542	0.370546	PTN
4	19321542	19421542	0.370546	DGKI
4	19321597	19421597	0.36074	PTN
4	19321597	19421597	0.36074	DGKI
4	19321626	19421626	0.350525	PTN
4	19321626	19421626	0.350525	DGKI
4	19321643	19421643	0.350525	PTN
4	19321643	19421643	0.350525	DGKI
4	19321649	19421649	0.350525	PTN
4	19321649	19421649	0.350525	DGKI
4	19322039	19422039	0.343616	PTN
4	19322039	19422039	0.343616	DGKI
4	19325128	19425128	0.349148	PTN
4	19325128	19425128	0.349148	DGKI
4	19325816	19425816	0.34857	PTN
4	19325816	19425816	0.34857	DGKI
4	19325817	19425817	0.34857	PTN
4	19325817	19425817	0.34857	DGKI
4	19325937	19425937	0.357467	PTN
4	19325937	19425937	0.357467	DGKI

4	19325979	19425979	0.379531	PTN
4	19325979	19425979	0.379531	DGKI
4	19326264	19426264	0.380861	PTN
4	19326264	19426264	0.380861	DGKI
4	19326269	19426269	0.394217	PTN
4	19326269	19426269	0.394217	DGKI
4	19326270	19426270	0.396103	PTN
4	19326270	19426270	0.396103	DGKI
4	19326294	19426294	0.412682	PTN
4	19326294	19426294	0.412682	DGKI
4	19326334	19426334	0.397489	PTN
4	19326334	19426334	0.397489	DGKI
4	19326345	19426345	0.356331	PTN
4	19326345	19426345	0.356331	DGKI
4	19326484	19426484	0.356331	PTN
4	19326484	19426484	0.356331	DGKI
4	19326491	19426491	0.356331	PTN
4	19326491	19426491	0.356331	DGKI
4	19326522	19426522	0.356331	PTN
4	19326522	19426522	0.356331	DGKI
4	19326599	19426599	0.356331	PTN
4	19326599	19426599	0.356331	DGKI
4	19326933	19426933	0.364384	PTN
4	19326933	19426933	0.364384	DGKI
4	19327535	19427535	0.349549	PTN
4	19327535	19427535	0.349549	DGKI
4	19329513	19429513	0.356834	PTN
4	19329513	19429513	0.356834	DGKI
4	19329607	19429607	0.347107	PTN
4	19329607	19429607	0.347107	DGKI
4	19329613	19429613	0.350216	PTN
4	19329613	19429613	0.350216	DGKI
4	19329781	19429781	0.350216	PTN
4	19329781	19429781	0.350216	DGKI
4	19329887	19429887	0.379471	PTN
4	19329887	19429887	0.379471	DGKI
4	19329925	19429925	0.375496	PTN
4	19329925	19429925	0.375496	DGKI
4	19329979	19429979	0.369499	PTN
4	19329979	19429979	0.369499	DGKI
4	19330019	19430019	0.369499	PTN
4	19330019	19430019	0.369499	DGKI
4	19330044	19430044	0.380772	PTN
4	19330044	19430044	0.380772	DGKI
4	19330110	19430110	0.373228	PTN
4	19330110	19430110	0.373228	DGKI
4	19330203	19430203	0.373228	PTN
4	19330203	19430203	0.373228	DGKI
4	19330265	19430265	0.373228	PTN
4	19330265	19430265	0.373228	DGKI
4	19330274	19430274	0.373228	PTN
4	19330274	19430274	0.373228	DGKI
4	19330287	19430287	0.373228	PTN
4	19330287	19430287	0.373228	DGKI
4	19330293	19430293	0.374807	PTN
4	19330293	19430293	0.374807	DGKI
4	19330294	19430294	0.375851	PTN
4	19330294	19430294	0.375851	DGKI

4	19330298	19430298	0.375851	PTN
4	19330298	19430298	0.375851	DGKI
4	19330300	19430300	0.375851	PTN
4	19330300	19430300	0.375851	DGKI
4	19330307	19430307	0.375851	PTN
4	19330307	19430307	0.375851	DGKI
4	19330318	19430318	0.380579	PTN
4	19330318	19430318	0.380579	DGKI
4	19330545	19430545	0.371505	PTN
4	19330545	19430545	0.371505	DGKI
4	19330614	19430614	0.371505	PTN
4	19330614	19430614	0.371505	DGKI
4	19330625	19430625	0.371505	PTN
4	19330625	19430625	0.371505	DGKI
4	19330676	19430676	0.381852	PTN
4	19330676	19430676	0.381852	DGKI
4	19330683	19430683	0.356228	PTN
4	19330683	19430683	0.356228	DGKI
4	19330710	19430710	0.365245	PTN
4	19330710	19430710	0.365245	DGKI
4	19330714	19430714	0.355788	PTN
4	19330714	19430714	0.355788	DGKI
4	19330829	19430829	0.349136	PTN
4	19330829	19430829	0.349136	DGKI
4	19330832	19430832	0.349136	PTN
4	19330832	19430832	0.349136	DGKI
4	19330892	19430892	0.349136	PTN
4	19330892	19430892	0.349136	DGKI
4	19330916	19430916	0.349136	PTN
4	19330916	19430916	0.349136	DGKI
4	19330965	19430965	0.349136	PTN
4	19330965	19430965	0.349136	DGKI
4	19330968	19430968	0.349136	PTN
4	19330968	19430968	0.349136	DGKI
4	19331067	19431067	0.349136	PTN
4	19331067	19431067	0.349136	DGKI
4	19331149	19431149	0.349136	PTN
4	19331149	19431149	0.349136	DGKI
4	19331156	19431156	0.349136	PTN
4	19331156	19431156	0.349136	DGKI
4	19331218	19431218	0.349136	PTN
4	19331218	19431218	0.349136	DGKI
4	19331270	19431270	0.349136	PTN
4	19331270	19431270	0.349136	DGKI
4	19331342	19431342	0.355017	PTN
4	19331342	19431342	0.355017	DGKI
4	19331350	19431350	0.34435	PTN
4	19331350	19431350	0.34435	DGKI
4	19331365	19431365	0.34435	PTN
4	19331365	19431365	0.34435	DGKI
4	19331368	19431368	0.347428	PTN
4	19331368	19431368	0.347428	DGKI
4	19331396	19431396	0.343112	PTN
4	19331396	19431396	0.343112	DGKI
4	19331522	19431522	0.346724	PTN
4	19331522	19431522	0.346724	DGKI
4	19331836	19431836	0.347327	PTN
4	19331836	19431836	0.347327	DGKI

4	19331876	19431876	0.347327	PTN
4	19331876	19431876	0.347327	DGKI
4	19331899	19431899	0.347327	PTN
4	19331899	19431899	0.347327	DGKI
4	19331987	19431987	0.347327	PTN
4	19331987	19431987	0.347327	DGKI
4	19332012	19432012	0.347327	PTN
4	19332012	19432012	0.347327	DGKI
4	19332143	19432143	0.347327	PTN
4	19332143	19432143	0.347327	DGKI
4	19332154	19432154	0.376689	PTN
4	19332154	19432154	0.376689	DGKI
4	19332155	19432155	0.364579	PTN
4	19332155	19432155	0.364579	DGKI
4	19332245	19432245	0.362685	PTN
4	19332245	19432245	0.362685	DGKI
4	19332380	19432380	0.395264	PTN
4	19332380	19432380	0.395264	DGKI
4	19332410	19432410	0.393544	PTN
4	19332410	19432410	0.393544	DGKI
4	19332998	19432998	0.367388	PTN
4	19332998	19432998	0.367388	DGKI
4	19333117	19433117	0.438963	PTN
4	19333117	19433117	0.438963	DGKI
4	19333242	19433242	0.467817	PTN
4	19333242	19433242	0.467817	DGKI
4	19333273	19433273	0.46135	PTN
4	19333273	19433273	0.46135	DGKI
4	19333492	19433492	0.459095	PTN
4	19333492	19433492	0.459095	DGKI
4	19333555	19433555	0.46523	PTN
4	19333555	19433555	0.46523	DGKI
4	19333567	19433567	0.477109	PTN
4	19333567	19433567	0.477109	DGKI
4	19333715	19433715	0.482772	PTN
4	19333715	19433715	0.482772	DGKI
4	19333750	19433750	0.483344	PTN
4	19333750	19433750	0.483344	DGKI
4	19333756	19433756	0.475457	PTN
4	19333756	19433756	0.475457	DGKI
4	19333885	19433885	0.481618	PTN
4	19333885	19433885	0.481618	DGKI
4	19333921	19433921	0.484337	PTN
4	19333921	19433921	0.484337	DGKI
4	19333950	19433950	0.500419	PTN
4	19333950	19433950	0.500419	DGKI
4	19333953	19433953	0.499696	PTN
4	19333953	19433953	0.499696	DGKI
4	19333971	19433971	0.516779	PTN
4	19333971	19433971	0.516779	DGKI
4	19333978	19433978	0.5322	PTN
4	19333978	19433978	0.5322	DGKI
4	19333990	19433990	0.509615	PTN
4	19333990	19433990	0.509615	DGKI
4	19334050	19434050	0.521179	PTN
4	19334050	19434050	0.521179	DGKI
4	19334094	19434094	0.483445	PTN
4	19334094	19434094	0.483445	DGKI

4	19334109	19434109	0.47302	PTN
4	19334109	19434109	0.47302	DGKI
4	19334113	19434113	0.419515	PTN
4	19334113	19434113	0.419515	DGKI
4	19334153	19434153	0.421642	PTN
4	19334153	19434153	0.421642	DGKI
4	19334215	19434215	0.457852	PTN
4	19334215	19434215	0.457852	DGKI
4	19336225	19436225	0.351728	PTN
4	19336225	19436225	0.351728	DGKI
4	19337285	19437285	0.343543	PTN
4	19337285	19437285	0.343543	DGKI
4	19337623	19437623	0.368536	PTN
4	19337623	19437623	0.368536	DGKI
4	19337833	19437833	0.386587	PTN
4	19337833	19437833	0.386587	DGKI
4	19337846	19437846	0.386385	PTN
4	19337846	19437846	0.386385	DGKI
4	19337889	19437889	0.397047	PTN
4	19337889	19437889	0.397047	DGKI
4	19337906	19437906	0.408669	PTN
4	19337906	19437906	0.408669	DGKI
4	19337908	19437908	0.408669	PTN
4	19337908	19437908	0.408669	DGKI
4	19337912	19437912	0.408669	PTN
4	19337912	19437912	0.408669	DGKI
4	19337937	19437937	0.386216	PTN
4	19337937	19437937	0.386216	DGKI
4	19338132	19438132	0.3564	PTN
4	19338132	19438132	0.3564	DGKI
4	19338143	19438143	0.391176	PTN
4	19338143	19438143	0.391176	DGKI
4	19338364	19438364	0.377259	PTN
4	19338364	19438364	0.377259	DGKI
4	19338587	19438587	0.400811	PTN
4	19338587	19438587	0.400811	DGKI
4	19338632	19438632	0.365923	PTN
4	19338632	19438632	0.365923	DGKI
4	19338642	19438642	0.365923	PTN
4	19338642	19438642	0.365923	DGKI
4	19338731	19438731	0.365923	PTN
4	19338731	19438731	0.365923	DGKI
4	19338960	19438960	0.366719	PTN
4	19338960	19438960	0.366719	DGKI
4	19338999	19438999	0.36796	PTN
4	19338999	19438999	0.36796	DGKI
4	19339219	19439219	0.368697	PTN
4	19339219	19439219	0.368697	DGKI
4	19339234	19439234	0.361696	PTN
4	19339234	19439234	0.361696	DGKI
4	19339356	19439356	0.347883	PTN
4	19339356	19439356	0.347883	DGKI
4	19339378	19439378	0.359458	PTN
4	19339378	19439378	0.359458	DGKI
4	19339595	19439595	0.385444	PTN
4	19339595	19439595	0.385444	DGKI
4	19340014	19440014	0.385043	PTN
4	19340014	19440014	0.385043	DGKI

4	19340193	19440193	0.419253	PTN
4	19340193	19440193	0.419253	DGKI
4	19340410	19440410	0.420568	PTN
4	19340410	19440410	0.420568	DGKI
4	19340609	19440609	0.396661	PTN
4	19340609	19440609	0.396661	DGKI
4	19340961	19440961	0.402287	PTN
4	19340961	19440961	0.402287	DGKI
4	19341165	19441165	0.418768	PTN
4	19341165	19441165	0.418768	DGKI
4	19341306	19441306	0.428984	PTN
4	19341306	19441306	0.428984	DGKI
4	19341390	19441390	0.425895	PTN
4	19341390	19441390	0.425895	DGKI
4	19341412	19441412	0.434596	PTN
4	19341412	19441412	0.434596	DGKI
4	19341416	19441416	0.451322	PTN
4	19341416	19441416	0.451322	DGKI
4	19341541	19441541	0.494223	PTN
4	19341541	19441541	0.494223	DGKI
4	19342011	19442011	0.358732	PTN
4	19342011	19442011	0.358732	DGKI
4	19342074	19442074	0.39839	PTN
4	19342074	19442074	0.39839	DGKI
4	20851274	20951274	0.370067	FAM180A
4	20851274	20951274	0.370067	LOC102168973
4	20851274	20951274	0.370067	SLC13A4
4	20851274	20951274	0.370067	C4H7orf73
4	20851274	20951274	0.370067	NUP205
4	20851285	20951285	0.367328	FAM180A
4	20851285	20951285	0.367328	LOC102168973
4	20851285	20951285	0.367328	SLC13A4
4	20851285	20951285	0.367328	C4H7orf73
4	20851285	20951285	0.367328	NUP205
4	20851483	20951483	0.369578	FAM180A
4	20851483	20951483	0.369578	LOC102168973
4	20851483	20951483	0.369578	SLC13A4
4	20851483	20951483	0.369578	C4H7orf73
4	20851483	20951483	0.369578	NUP205
4	20851537	20951537	0.368439	FAM180A
4	20851537	20951537	0.368439	LOC102168973
4	20851537	20951537	0.368439	SLC13A4
4	20851537	20951537	0.368439	C4H7orf73
4	20851537	20951537	0.368439	NUP205
4	20851824	20951824	0.359362	FAM180A
4	20851824	20951824	0.359362	LOC102168973
4	20851824	20951824	0.359362	SLC13A4
4	20851824	20951824	0.359362	C4H7orf73
4	20851824	20951824	0.359362	NUP205
4	20851932	20951932	0.35226	FAM180A
4	20851932	20951932	0.35226	LOC102168973
4	20851932	20951932	0.35226	SLC13A4
4	20851932	20951932	0.35226	C4H7orf73
4	20851932	20951932	0.35226	NUP205
4	20852284	20952284	0.349087	FAM180A
4	20852284	20952284	0.349087	LOC102168973
4	20852284	20952284	0.349087	SLC13A4
4	20852284	20952284	0.349087	C4H7orf73

4	20852284	20952284	0.349087	NUP205
4	20852719	20952719	0.361464	FAM180A
4	20852719	20952719	0.361464	LOC102168973
4	20852719	20952719	0.361464	SLC13A4
4	20852719	20952719	0.361464	C4H7orf73
4	20852719	20952719	0.361464	NUP205
4	20852725	20952725	0.361464	FAM180A
4	20852725	20952725	0.361464	LOC102168973
4	20852725	20952725	0.361464	SLC13A4
4	20852725	20952725	0.361464	C4H7orf73
4	20852725	20952725	0.361464	NUP205
4	20852746	20952746	0.357112	FAM180A
4	20852746	20952746	0.357112	LOC102168973
4	20852746	20952746	0.357112	SLC13A4
4	20852746	20952746	0.357112	C4H7orf73
4	20852746	20952746	0.357112	NUP205
4	20853047	20953047	0.369534	FAM180A
4	20853047	20953047	0.369534	LOC102168973
4	20853047	20953047	0.369534	SLC13A4
4	20853047	20953047	0.369534	C4H7orf73
4	20853047	20953047	0.369534	NUP205
4	20853337	20953337	0.38088	FAM180A
4	20853337	20953337	0.38088	LOC102168973
4	20853337	20953337	0.38088	SLC13A4
4	20853337	20953337	0.38088	C4H7orf73
4	20853337	20953337	0.38088	NUP205
4	20853342	20953342	0.370504	FAM180A
4	20853342	20953342	0.370504	LOC102168973
4	20853342	20953342	0.370504	SLC13A4
4	20853342	20953342	0.370504	C4H7orf73
4	20853342	20953342	0.370504	NUP205
4	20853399	20953399	0.373948	FAM180A
4	20853399	20953399	0.373948	LOC102168973
4	20853399	20953399	0.373948	SLC13A4
4	20853399	20953399	0.373948	C4H7orf73
4	20853399	20953399	0.373948	NUP205
4	20853431	20953431	0.374677	FAM180A
4	20853431	20953431	0.374677	LOC102168973
4	20853431	20953431	0.374677	SLC13A4
4	20853431	20953431	0.374677	C4H7orf73
4	20853431	20953431	0.374677	NUP205
4	20853544	20953544	0.37395	FAM180A
4	20853544	20953544	0.37395	LOC102168973
4	20853544	20953544	0.37395	SLC13A4
4	20853544	20953544	0.37395	C4H7orf73
4	20853544	20953544	0.37395	NUP205
4	20853575	20953575	0.387907	FAM180A
4	20853575	20953575	0.387907	LOC102168973
4	20853575	20953575	0.387907	SLC13A4
4	20853575	20953575	0.387907	C4H7orf73
4	20853575	20953575	0.387907	NUP205
4	20853627	20953627	0.38167	FAM180A
4	20853627	20953627	0.38167	LOC102168973
4	20853627	20953627	0.38167	SLC13A4
4	20853627	20953627	0.38167	C4H7orf73
4	20853627	20953627	0.38167	NUP205
4	20853650	20953650	0.372756	FAM180A
4	20853650	20953650	0.372756	LOC102168973

4	20853650	20953650	0.372756	SLC13A4
4	20853650	20953650	0.372756	C4H7orf73
4	20853650	20953650	0.372756	NUP205
4	20853651	20953651	0.372713	FAM180A
4	20853651	20953651	0.372713	LOC102168973
4	20853651	20953651	0.372713	SLC13A4
4	20853651	20953651	0.372713	C4H7orf73
4	20853651	20953651	0.372713	NUP205
4	20853757	20953757	0.372834	FAM180A
4	20853757	20953757	0.372834	LOC102168973
4	20853757	20953757	0.372834	SLC13A4
4	20853757	20953757	0.372834	C4H7orf73
4	20853757	20953757	0.372834	NUP205
4	20853764	20953764	0.372691	FAM180A
4	20853764	20953764	0.372691	LOC102168973
4	20853764	20953764	0.372691	SLC13A4
4	20853764	20953764	0.372691	C4H7orf73
4	20853764	20953764	0.372691	NUP205
4	20853856	20953856	0.350156	FAM180A
4	20853856	20953856	0.350156	LOC102168973
4	20853856	20953856	0.350156	SLC13A4
4	20853856	20953856	0.350156	C4H7orf73
4	20853856	20953856	0.350156	NUP205
4	20853909	20953909	0.341687	FAM180A
4	20853909	20953909	0.341687	LOC102168973
4	20853909	20953909	0.341687	SLC13A4
4	20853909	20953909	0.341687	C4H7orf73
4	20853909	20953909	0.341687	NUP205
4	20854494	20954494	0.342426	FAM180A
4	20854494	20954494	0.342426	LOC102168973
4	20854494	20954494	0.342426	SLC13A4
4	20854494	20954494	0.342426	C4H7orf73
4	20854494	20954494	0.342426	NUP205
4	20854583	20954583	0.350919	FAM180A
4	20854583	20954583	0.350919	LOC102168973
4	20854583	20954583	0.350919	SLC13A4
4	20854583	20954583	0.350919	C4H7orf73
4	20854583	20954583	0.350919	NUP205
4	20854590	20954590	0.349281	FAM180A
4	20854590	20954590	0.349281	LOC102168973
4	20854590	20954590	0.349281	SLC13A4
4	20854590	20954590	0.349281	C4H7orf73
4	20854590	20954590	0.349281	NUP205
4	20854698	20954698	0.347601	FAM180A
4	20854698	20954698	0.347601	LOC102168973
4	20854698	20954698	0.347601	SLC13A4
4	20854698	20954698	0.347601	C4H7orf73
4	20854698	20954698	0.347601	NUP205
4	20854709	20954709	0.343111	FAM180A
4	20854709	20954709	0.343111	LOC102168973
4	20854709	20954709	0.343111	SLC13A4
4	20854709	20954709	0.343111	C4H7orf73
4	20854709	20954709	0.343111	NUP205
4	20854768	20954768	0.346389	FAM180A
4	20854768	20954768	0.346389	LOC102168973
4	20854768	20954768	0.346389	SLC13A4
4	20854768	20954768	0.346389	C4H7orf73
4	20854768	20954768	0.346389	NUP205

4	20854769	20954769	0.346389	FAM180A
4	20854769	20954769	0.346389	LOC102168973
4	20854769	20954769	0.346389	SLC13A4
4	20854769	20954769	0.346389	C4H7orf73
4	20854769	20954769	0.346389	NUP205
4	20864822	20964822	0.34342	FAM180A
4	20864822	20964822	0.34342	LOC102168973
4	20864822	20964822	0.34342	SLC13A4
4	20864822	20964822	0.34342	C4H7orf73
4	20864822	20964822	0.34342	NUP205
4	20865478	20965478	0.364963	FAM180A
4	20865478	20965478	0.364963	LOC102168973
4	20865478	20965478	0.364963	SLC13A4
4	20865478	20965478	0.364963	C4H7orf73
4	20865478	20965478	0.364963	NUP205
4	20865504	20965504	0.378028	FAM180A
4	20865504	20965504	0.378028	LOC102168973
4	20865504	20965504	0.378028	SLC13A4
4	20865504	20965504	0.378028	C4H7orf73
4	20865504	20965504	0.378028	NUP205
4	20865576	20965576	0.373788	FAM180A
4	20865576	20965576	0.373788	LOC102168973
4	20865576	20965576	0.373788	SLC13A4
4	20865576	20965576	0.373788	C4H7orf73
4	20865576	20965576	0.373788	NUP205
4	20865595	20965595	0.373117	FAM180A
4	20865595	20965595	0.373117	LOC102168973
4	20865595	20965595	0.373117	SLC13A4
4	20865595	20965595	0.373117	C4H7orf73
4	20865595	20965595	0.373117	NUP205
4	20865596	20965596	0.390172	FAM180A
4	20865596	20965596	0.390172	LOC102168973
4	20865596	20965596	0.390172	SLC13A4
4	20865596	20965596	0.390172	C4H7orf73
4	20865596	20965596	0.390172	NUP205
4	20865639	20965639	0.383561	FAM180A
4	20865639	20965639	0.383561	LOC102168973
4	20865639	20965639	0.383561	SLC13A4
4	20865639	20965639	0.383561	C4H7orf73
4	20865639	20965639	0.383561	NUP205
4	20865699	20965699	0.379833	FAM180A
4	20865699	20965699	0.379833	LOC102168973
4	20865699	20965699	0.379833	SLC13A4
4	20865699	20965699	0.379833	C4H7orf73
4	20865699	20965699	0.379833	NUP205
4	20865766	20965766	0.38866	FAM180A
4	20865766	20965766	0.38866	LOC102168973
4	20865766	20965766	0.38866	SLC13A4
4	20865766	20965766	0.38866	C4H7orf73
4	20865766	20965766	0.38866	NUP205
4	20866210	20966210	0.384612	FAM180A
4	20866210	20966210	0.384612	LOC102168973
4	20866210	20966210	0.384612	SLC13A4
4	20866210	20966210	0.384612	C4H7orf73
4	20866210	20966210	0.384612	NUP205
4	20866311	20966311	0.374877	FAM180A
4	20866311	20966311	0.374877	LOC102168973
4	20866311	20966311	0.374877	SLC13A4

4	20866311	20966311	0.374877	C4H7orf73
4	20866311	20966311	0.374877	NUP205
4	20866452	20966452	0.369823	FAM180A
4	20866452	20966452	0.369823	LOC102168973
4	20866452	20966452	0.369823	SLC13A4
4	20866452	20966452	0.369823	C4H7orf73
4	20866452	20966452	0.369823	NUP205
4	20866486	20966486	0.377975	FAM180A
4	20866486	20966486	0.377975	LOC102168973
4	20866486	20966486	0.377975	SLC13A4
4	20866486	20966486	0.377975	C4H7orf73
4	20866486	20966486	0.377975	NUP205
4	20866515	20966515	0.358107	FAM180A
4	20866515	20966515	0.358107	LOC102168973
4	20866515	20966515	0.358107	SLC13A4
4	20866515	20966515	0.358107	C4H7orf73
4	20866515	20966515	0.358107	NUP205
4	20866935	20966935	0.346697	FAM180A
4	20866935	20966935	0.346697	LOC102168973
4	20866935	20966935	0.346697	SLC13A4
4	20866935	20966935	0.346697	C4H7orf73
4	20866935	20966935	0.346697	NUP205
4	20867203	20967203	0.341882	FAM180A
4	20867203	20967203	0.341882	LOC102168973
4	20867203	20967203	0.341882	SLC13A4
4	20867203	20967203	0.341882	C4H7orf73
4	20867203	20967203	0.341882	NUP205
4	20867339	20967339	0.363748	FAM180A
4	20867339	20967339	0.363748	LOC102168973
4	20867339	20967339	0.363748	SLC13A4
4	20867339	20967339	0.363748	C4H7orf73
4	20867339	20967339	0.363748	NUP205
4	20867389	20967389	0.368546	FAM180A
4	20867389	20967389	0.368546	LOC102168973
4	20867389	20967389	0.368546	SLC13A4
4	20867389	20967389	0.368546	C4H7orf73
4	20867389	20967389	0.368546	NUP205
4	20867414	20967414	0.377207	FAM180A
4	20867414	20967414	0.377207	LOC102168973
4	20867414	20967414	0.377207	SLC13A4
4	20867414	20967414	0.377207	C4H7orf73
4	20867414	20967414	0.377207	NUP205
4	20867565	20967565	0.364301	FAM180A
4	20867565	20967565	0.364301	LOC102168973
4	20867565	20967565	0.364301	SLC13A4
4	20867565	20967565	0.364301	C4H7orf73
4	20867565	20967565	0.364301	NUP205
4	20867979	20967979	0.36453	FAM180A
4	20867979	20967979	0.36453	LOC102168973
4	20867979	20967979	0.36453	SLC13A4
4	20867979	20967979	0.36453	C4H7orf73
4	20867979	20967979	0.36453	NUP205
4	20868309	20968309	0.370172	FAM180A
4	20868309	20968309	0.370172	LOC102168973
4	20868309	20968309	0.370172	SLC13A4
4	20868309	20968309	0.370172	C4H7orf73
4	20868309	20968309	0.370172	NUP205
4	20868310	20968310	0.350675	FAM180A

4	20868310	20968310	0.350675	LOC102168973
4	20868310	20968310	0.350675	SLC13A4
4	20868310	20968310	0.350675	C4H7orf73
4	20868310	20968310	0.350675	NUP205
4	20870508	20970508	0.347813	LOC102168973
4	20870508	20970508	0.347813	SLC13A4
4	20870508	20970508	0.347813	C4H7orf73
4	20870508	20970508	0.347813	NUP205
4	20870910	20970910	0.344976	SLC13A4
4	20870910	20970910	0.344976	C4H7orf73
4	20870910	20970910	0.344976	NUP205
4	20871130	20971130	0.373016	SLC13A4
4	20871130	20971130	0.373016	C4H7orf73
4	20871130	20971130	0.373016	NUP205
4	20871436	20971436	0.360247	SLC13A4
4	20871436	20971436	0.360247	C4H7orf73
4	20871436	20971436	0.360247	NUP205
4	20871658	20971658	0.357996	SLC13A4
4	20871658	20971658	0.357996	C4H7orf73
4	20871658	20971658	0.357996	NUP205
4	20871869	20971869	0.369356	SLC13A4
4	20871869	20971869	0.369356	C4H7orf73
4	20871869	20971869	0.369356	NUP205
4	20871986	20971986	0.369356	SLC13A4
4	20871986	20971986	0.369356	C4H7orf73
4	20871986	20971986	0.369356	NUP205
4	20872506	20972506	0.363961	SLC13A4
4	20872506	20972506	0.363961	C4H7orf73
4	20872506	20972506	0.363961	NUP205
4	20872605	20972605	0.343089	SLC13A4
4	20872605	20972605	0.343089	C4H7orf73
4	20872605	20972605	0.343089	NUP205
4	20872839	20972839	0.374181	SLC13A4
4	20872839	20972839	0.374181	C4H7orf73
4	20872839	20972839	0.374181	NUP205
4	20872944	20972944	0.366698	SLC13A4
4	20872944	20972944	0.366698	C4H7orf73
4	20872944	20972944	0.366698	NUP205
4	20873091	20973091	0.382855	SLC13A4
4	20873091	20973091	0.382855	C4H7orf73
4	20873091	20973091	0.382855	NUP205
4	20873203	20973203	0.386325	SLC13A4
4	20873203	20973203	0.386325	C4H7orf73
4	20873203	20973203	0.386325	NUP205
4	20873244	20973244	0.386905	SLC13A4
4	20873244	20973244	0.386905	C4H7orf73
4	20873244	20973244	0.386905	NUP205
4	20873247	20973247	0.372571	SLC13A4
4	20873247	20973247	0.372571	C4H7orf73
4	20873247	20973247	0.372571	NUP205
4	20873448	20973448	0.368419	SLC13A4
4	20873448	20973448	0.368419	C4H7orf73
4	20873448	20973448	0.368419	NUP205
4	20873501	20973501	0.368676	SLC13A4
4	20873501	20973501	0.368676	C4H7orf73
4	20873501	20973501	0.368676	NUP205
4	20873871	20973871	0.383932	SLC13A4
4	20873871	20973871	0.383932	C4H7orf73

4	20873871	20973871	0.383932	NUP205
4	20874007	20974007	0.375183	SLC13A4
4	20874007	20974007	0.375183	C4H7orf73
4	20874007	20974007	0.375183	NUP205
4	20874027	20974027	0.380876	SLC13A4
4	20874027	20974027	0.380876	C4H7orf73
4	20874027	20974027	0.380876	NUP205
4	20874082	20974082	0.379888	SLC13A4
4	20874082	20974082	0.379888	C4H7orf73
4	20874082	20974082	0.379888	NUP205
4	20874350	20974350	0.386842	SLC13A4
4	20874350	20974350	0.386842	C4H7orf73
4	20874350	20974350	0.386842	NUP205
4	20874439	20974439	0.385304	SLC13A4
4	20874439	20974439	0.385304	C4H7orf73
4	20874439	20974439	0.385304	NUP205
4	20874487	20974487	0.387177	SLC13A4
4	20874487	20974487	0.387177	C4H7orf73
4	20874487	20974487	0.387177	NUP205
4	20874518	20974518	0.379309	SLC13A4
4	20874518	20974518	0.379309	C4H7orf73
4	20874518	20974518	0.379309	NUP205
4	20874582	20974582	0.379109	SLC13A4
4	20874582	20974582	0.379109	C4H7orf73
4	20874582	20974582	0.379109	NUP205
4	20874592	20974592	0.379344	SLC13A4
4	20874592	20974592	0.379344	C4H7orf73
4	20874592	20974592	0.379344	NUP205
4	20874795	20974795	0.395561	SLC13A4
4	20874795	20974795	0.395561	C4H7orf73
4	20874795	20974795	0.395561	NUP205
4	20874875	20974875	0.383449	SLC13A4
4	20874875	20974875	0.383449	C4H7orf73
4	20874875	20974875	0.383449	NUP205
4	20874881	20974881	0.380946	SLC13A4
4	20874881	20974881	0.380946	C4H7orf73
4	20874881	20974881	0.380946	NUP205
4	20874894	20974894	0.367985	SLC13A4
4	20874894	20974894	0.367985	C4H7orf73
4	20874894	20974894	0.367985	NUP205
4	20875173	20975173	0.376562	SLC13A4
4	20875173	20975173	0.376562	C4H7orf73
4	20875173	20975173	0.376562	NUP205
4	20875521	20975521	0.355793	SLC13A4
4	20875521	20975521	0.355793	C4H7orf73
4	20875521	20975521	0.355793	NUP205
4	20875601	20975601	0.354756	SLC13A4
4	20875601	20975601	0.354756	C4H7orf73
4	20875601	20975601	0.354756	NUP205
4	20875610	20975610	0.350212	SLC13A4
4	20875610	20975610	0.350212	C4H7orf73
4	20875610	20975610	0.350212	NUP205
4	20875867	20975867	0.369386	SLC13A4
4	20875867	20975867	0.369386	C4H7orf73
4	20875867	20975867	0.369386	NUP205
4	20875973	20975973	0.371397	SLC13A4
4	20875973	20975973	0.371397	C4H7orf73
4	20875973	20975973	0.371397	NUP205

4	20876356	20976356	0.379394	SLC13A4
4	20876356	20976356	0.379394	C4H7orf73
4	20876356	20976356	0.379394	NUP205
4	21592620	21692620	0.343405	CALD1
4	21592621	21692621	0.343405	CALD1
4	21592645	21692645	0.364594	CALD1
4	21592648	21692648	0.364594	CALD1
4	21592669	21692669	0.355965	CALD1
4	21592676	21692676	0.350694	CALD1
4	21592684	21692684	0.352763	CALD1
4	21592688	21692688	0.365479	CALD1
4	21592694	21692694	0.365971	CALD1
4	21592738	21692738	0.351595	CALD1
4	21592798	21692798	0.358719	CALD1
4	21592823	21692823	0.352607	CALD1
4	21592827	21692827	0.349578	CALD1
4	21592833	21692833	0.349607	CALD1
4	21592852	21692852	0.348293	CALD1
4	21624217	21724217	0.347211	CALD1
4	21624239	21724239	0.35598	CALD1
4	21624240	21724240	0.367195	CALD1
4	21749957	21849957	0.369023	BPGM
4	21749957	21849957	0.369023	CALD1
4	21749967	21849967	0.347686	BPGM
4	21749967	21849967	0.347686	CALD1
4	25521551	25621551	0.342638	MIR29A
4	25521551	25621551	0.342638	LOC102184972
4	28533766	28633766	0.344807	GRM8
4	28533829	28633829	0.358546	GRM8
4	28533830	28633830	0.358546	GRM8
4	28533832	28633832	0.344349	GRM8
4	28533898	28633898	0.349466	GRM8
4	28534240	28634240	0.348429	GRM8
4	28797644	28897644	0.342348	MIR592
4	28797644	28897644	0.342348	GRM8
4	28823164	28923164	0.345771	GRM8
4	28823230	28923230	0.344436	GRM8
4	28823275	28923275	0.344866	GRM8
4	28823345	28923345	0.362352	GRM8
4	28823396	28923396	0.346192	GRM8
4	28823583	28923583	0.345284	GRM8
4	28824845	28924845	0.342194	GRM8
4	29195539	29295539	0.362758	GRM8
4	30062970	30162970	0.36356	LOC102169830
4	30062971	30162971	0.36356	LOC102169830
4	30938186	31038186	0.40391	POT1
4	30938438	31038438	0.3943	POT1
4	30939097	31039097	0.374189	POT1
4	30939664	31039664	0.406167	POT1
4	30939712	31039712	0.390153	POT1
4	30939973	31039973	0.394809	POT1
4	30940040	31040040	0.385972	POT1
4	30940620	31040620	0.385972	POT1
4	30940661	31040661	0.399052	POT1
4	30940754	31040754	0.383075	POT1
4	30940872	31040872	0.383075	POT1
4	30940907	31040907	0.387043	POT1
4	30941034	31041034	0.384734	POT1

4	30941257	31041257	0.384734		POT1
4	30941272	31041272	0.384734		POT1
4	30941487	31041487	0.384734		POT1
4	30941643	31041643	0.384734		POT1
4	30941825	31041825	0.385919		POT1
4	30941934	31041934	0.398668		POT1
4	30941942	31041942	0.38905		POT1
4	30942123	31042123	0.369925		POT1
4	30942131	31042131	0.372229		POT1
4	30942232	31042232	0.384211		POT1
4	30942554	31042554	0.384913		POT1
4	30942592	31042592	0.38986		POT1
4	30942686	31042686	0.391486		POT1
4	30942752	31042752	0.382654		POT1
4	30942790	31042790	0.411182		POT1
4	30942832	31042832	0.416546		POT1
4	30942858	31042858	0.418848		POT1
4	30942882	31042882	0.412143		POT1
4	30942887	31042887	0.409178		POT1
4	30943038	31043038	0.38881		POT1
4	30943140	31043140	0.364979		POT1
4	30943787	31043787	0.350574		POT1
4	30943801	31043801	0.349259		POT1
4	30944033	31044033	0.355159		POT1
4	30944409	31044409	0.372004		POT1
4	30945965	31045965	0.365014		POT1
4	30946314	31046314	0.373127		POT1
4	30946437	31046437	0.392995		POT1
4	30946760	31046760	0.381705		POT1
4	30946796	31046796	0.389633		POT1
4	30947180	31047180	0.396461		POT1
4	30947267	31047267	0.403489		POT1
4	30947368	31047368	0.398655		POT1
4	30947401	31047401	0.381081		POT1
4	30947540	31047540	0.380577		POT1
4	30947579	31047579	0.381561		POT1
4	30947581	31047581	0.381561		POT1
4	30947583	31047583	0.381561		POT1
4	30948091	31048091	0.381009		POT1
4	30948296	31048296	0.383742		POT1
4	30948388	31048388	0.392796		POT1
4	30948463	31048463	0.383567		POT1
4	30948631	31048631	0.404722		POT1
4	30948658	31048658	0.384483		POT1
4	30948732	31048732	0.384483		POT1
4	30948807	31048807	0.391913		POT1
4	30948939	31048939	0.381664		POT1
4	30949064	31049064	0.381252		POT1
4	30949330	31049330	0.381252		POT1
4	30949355	31049355	0.382085		POT1
4	30949682	31049682	0.384383		POT1
4	30949802	31049802	0.405515		POT1
4	30949996	31049996	0.402309		POT1
4	30950094	31050094	0.432856		POT1
4	30950121	31050121	0.445739		POT1
4	30950124	31050124	0.438464		POT1
4	30950386	31050386	0.4364		POT1
4	30950422	31050422	0.429505		POT1

4	30950439	31050439	0.429505	POT1
4	30950468	31050468	0.433068	POT1
4	30950665	31050665	0.421107	POT1
4	30950754	31050754	0.427252	POT1
4	30951043	31051043	0.438089	POT1
4	30951298	31051298	0.413763	POT1
4	30951452	31051452	0.391138	POT1
4	30951745	31051745	0.381722	POT1
4	30952214	31052214	0.384817	POT1
4	30952724	31052724	0.361934	POT1
4	30953027	31053027	0.382517	POT1
4	30953154	31053154	0.382517	POT1
4	30953195	31053195	0.385991	POT1
4	30953288	31053288	0.385341	POT1
4	30953324	31053324	0.369275	POT1
4	30953911	31053911	0.374955	POT1
4	30954036	31054036	0.364693	POT1
4	30954057	31054057	0.357861	POT1
4	30954159	31054159	0.364219	POT1
4	30954223	31054223	0.354024	POT1
4	30954567	31054567	0.354024	POT1
4	30955118	31055118	0.380366	POT1
4	30955134	31055134	0.380366	POT1
4	30955672	31055672	0.36758	POT1
4	30955682	31055682	0.373561	POT1
4	30955921	31055921	0.394696	POT1
4	30956044	31056044	0.366915	POT1
4	30956240	31056240	0.377743	POT1
4	30956315	31056315	0.360769	POT1
4	30956913	31056913	0.357717	POT1
4	30957079	31057079	0.356576	POT1
4	30957197	31057197	0.376213	POT1
4	30957231	31057231	0.38323	POT1
4	30957313	31057313	0.383259	POT1
4	30957330	31057330	0.386648	POT1
4	30957476	31057476	0.395164	POT1
4	30957553	31057553	0.390995	POT1
4	30957595	31057595	0.390849	POT1
4	30957602	31057602	0.38925	POT1
4	30957800	31057800	0.360256	POT1
4	30957962	31057962	0.345226	POT1
4	30958028	31058028	0.345305	POT1
4	30959767	31059767	0.352182	POT1
4	30959881	31059881	0.352682	POT1
4	30960121	31060121	0.360876	POT1
4	30960221	31060221	0.372	POT1
4	30960242	31060242	0.376309	POT1
4	30960572	31060572	0.368721	POT1
4	30960622	31060622	0.343925	POT1
4	30962536	31062536	0.361191	POT1
4	30962846	31062846	0.356645	POT1
4	30963049	31063049	0.353977	POT1
4	30963289	31063289	0.360395	POT1
4	30963412	31063412	0.355846	POT1
4	30963467	31063467	0.350584	POT1
4	30963532	31063532	0.351272	POT1
4	30963960	31063960	0.362706	POT1
4	30964109	31064109	0.36289	POT1

4	30964190	31064190	0.36621	POT1
4	30964227	31064227	0.36621	POT1
4	30964404	31064404	0.362336	POT1
4	30964972	31064972	0.359944	POT1
4	30965330	31065330	0.357156	POT1
4	30965525	31065525	0.371876	POT1
4	30965789	31065789	0.395297	POT1
4	30966326	31066326	0.373706	POT1
4	30966470	31066470	0.391891	POT1
4	30966508	31066508	0.393069	POT1
4	30966514	31066514	0.393069	POT1
4	30966541	31066541	0.389393	POT1
4	30966650	31066650	0.393565	POT1
4	30966651	31066651	0.394581	POT1
4	30966818	31066818	0.398563	POT1
4	30966832	31066832	0.400969	POT1
4	30966838	31066838	0.416776	POT1
4	30966850	31066850	0.397268	POT1
4	30966907	31066907	0.396842	POT1
4	30966958	31066958	0.394937	POT1
4	30967155	31067155	0.397738	POT1
4	30967287	31067287	0.394267	POT1
4	30967372	31067372	0.39967	POT1
4	30967442	31067442	0.400992	POT1
4	30967466	31067466	0.399827	POT1
4	30967567	31067567	0.407003	POT1
4	30967607	31067607	0.419687	POT1
4	30967654	31067654	0.405169	POT1
4	30967942	31067942	0.406247	POT1
4	30967963	31067963	0.403493	POT1
4	30968033	31068033	0.403493	POT1
4	30968138	31068138	0.403493	POT1
4	30968198	31068198	0.403493	POT1
4	30968249	31068249	0.403493	POT1
4	30968268	31068268	0.403493	POT1
4	30968301	31068301	0.403493	POT1
4	30968381	31068381	0.403493	POT1
4	30968424	31068424	0.4008	POT1
4	30968541	31068541	0.40244	POT1
4	30968629	31068629	0.40156	POT1
4	30968661	31068661	0.398261	POT1
4	30968828	31068828	0.404473	POT1
4	30968923	31068923	0.408414	POT1
4	30968934	31068934	0.402794	POT1
4	30968971	31068971	0.400112	POT1
4	30969063	31069063	0.400112	POT1
4	30969098	31069098	0.400112	POT1
4	30969099	31069099	0.402433	POT1
4	30969148	31069148	0.399405	POT1
4	30969164	31069164	0.404249	POT1
4	30969195	31069195	0.399515	POT1
4	30969239	31069239	0.405795	POT1
4	30969569	31069569	0.403558	POT1
4	30969915	31069915	0.407808	POT1
4	30970191	31070191	0.407808	POT1
4	30970263	31070263	0.407808	POT1
4	30970336	31070336	0.418382	POT1
4	30970347	31070347	0.418611	POT1

4	30970462	31070462	0.39496	POT1
4	30970530	31070530	0.397121	POT1
4	30970670	31070670	0.400683	POT1
4	30970710	31070710	0.400973	POT1
4	30970755	31070755	0.384131	POT1
4	30970953	31070953	0.404949	POT1
4	30970964	31070964	0.403558	POT1
4	30970985	31070985	0.383903	POT1
4	30971106	31071106	0.385693	POT1
4	30971117	31071117	0.385693	POT1
4	30971210	31071210	0.386158	POT1
4	30971286	31071286	0.388668	POT1
4	30971342	31071342	0.387293	POT1
4	30971450	31071450	0.38403	POT1
4	30971558	31071558	0.388029	POT1
4	30971617	31071617	0.391959	POT1
4	30971633	31071633	0.371263	POT1
4	30971665	31071665	0.371263	POT1
4	30971788	31071788	0.3718	POT1
4	30971839	31071839	0.365787	POT1
4	30972060	31072060	0.363644	POT1
4	30972103	31072103	0.363644	POT1
4	30972157	31072157	0.37511	POT1
4	30972158	31072158	0.37511	POT1
4	30972171	31072171	0.37511	POT1
4	30972410	31072410	0.379561	POT1
4	30972441	31072441	0.371304	POT1
4	30972966	31072966	0.371304	POT1
4	30973494	31073494	0.37253	POT1
4	30973603	31073603	0.37286	POT1
4	30973713	31073713	0.361757	POT1
4	30973725	31073725	0.361757	POT1
4	30973976	31073976	0.361757	POT1
4	30973985	31073985	0.361757	POT1
4	30974231	31074231	0.364888	POT1
4	30974381	31074381	0.368323	POT1
4	30974404	31074404	0.36672	POT1
4	30974505	31074505	0.372097	POT1
4	30974626	31074626	0.359166	POT1
4	30974693	31074693	0.368296	POT1
4	30974726	31074726	0.373816	POT1
4	30974906	31074906	0.366598	POT1
4	30975140	31075140	0.373129	POT1
4	30975191	31075191	0.373129	POT1
4	30975586	31075586	0.364481	POT1
4	30975638	31075638	0.368996	POT1
4	30975737	31075737	0.36887	POT1
4	30975769	31075769	0.366553	POT1
4	30975997	31075997	0.374241	POT1
4	30976166	31076166	0.374241	POT1
4	30976274	31076274	0.374241	POT1
4	30976537	31076537	0.373271	POT1
4	30976587	31076587	0.372474	POT1
4	30976621	31076621	0.372474	POT1
4	30976706	31076706	0.368416	POT1
4	30976789	31076789	0.380823	POT1
4	30977005	31077005	0.360696	POT1
4	30977607	31077607	0.373963	POT1

4	30978222	31078222	0.400491	POT1
4	30978288	31078288	0.396577	POT1
4	30978305	31078305	0.404167	POT1
4	30978400	31078400	0.400546	POT1
4	30978435	31078435	0.404685	POT1
4	30978583	31078583	0.417875	POT1
4	30978746	31078746	0.399241	POT1
4	30978871	31078871	0.398483	POT1
4	30978873	31078873	0.399438	POT1
4	30978886	31078886	0.40004	POT1
4	30978998	31078998	0.408292	POT1
4	30979006	31079006	0.407592	POT1
4	30979167	31079167	0.407592	POT1
4	30979192	31079192	0.406114	POT1
4	30979419	31079419	0.393297	POT1
4	30979427	31079427	0.397003	POT1
4	30979435	31079435	0.408853	POT1
4	30979508	31079508	0.379071	POT1
4	30979532	31079532	0.372194	POT1
4	30979577	31079577	0.37296	POT1
4	30979739	31079739	0.368723	POT1
4	30979792	31079792	0.342918	POT1
4	30980489	31080489	0.347455	POT1
4	30980592	31080592	0.345736	POT1
4	30980630	31080630	0.345603	POT1
4	30980791	31080791	0.34738	POT1
4	30982330	31082330	0.381746	POT1
4	30982420	31082420	0.377493	POT1
4	30982434	31082434	0.364755	POT1
4	30982660	31082660	0.364755	POT1
4	30982778	31082778	0.366315	POT1
4	30983072	31083072	0.369729	POT1
4	30983074	31083074	0.370094	POT1
4	30983281	31083281	0.382874	POT1
4	30986586	31086586	0.347216	POT1
4	30987052	31087052	0.345686	POT1
4	30987138	31087138	0.342563	POT1
4	30987174	31087174	0.342563	POT1
4	30987176	31087176	0.344224	POT1
4	30987692	31087692	0.349515	POT1
4	30987926	31087926	0.358862	POT1
4	30987976	31087976	0.360297	POT1
4	30988006	31088006	0.348831	POT1
4	30988174	31088174	0.347125	POT1
4	30988179	31088179	0.345386	POT1
4	30988215	31088215	0.345386	POT1
4	30988446	31088446	0.410971	POT1
4	30988584	31088584	0.379833	POT1
4	30988629	31088629	0.379833	POT1
4	30988686	31088686	0.379833	POT1
4	30988965	31088965	0.380891	POT1
4	30989220	31089220	0.382316	POT1
4	30989232	31089232	0.39476	POT1
4	30989267	31089267	0.399148	POT1
4	30989344	31089344	0.395053	POT1
4	30989474	31089474	0.357779	POT1
4	30989752	31089752	0.356237	POT1
4	30989998	31089998	0.358279	POT1

4	30990351	31090351	0.357619	POT1
4	30990405	31090405	0.364489	POT1
4	30990445	31090445	0.361542	POT1
4	30990774	31090774	0.370872	POT1
4	30990882	31090882	0.356315	POT1
4	30990910	31090910	0.356315	POT1
4	30990912	31090912	0.355602	POT1
4	30990968	31090968	0.343993	POT1
4	30991038	31091038	0.346101	POT1
4	30991060	31091060	0.344645	POT1
4	30991567	31091567	0.347915	POT1
4	30991624	31091624	0.347661	POT1
4	30991968	31091968	0.348956	POT1
4	30991970	31091970	0.348956	POT1
4	30992011	31092011	0.348093	POT1
4	30992087	31092087	0.348255	POT1
4	30992546	31092546	0.348255	POT1
4	30992618	31092618	0.363983	POT1
4	30992709	31092709	0.379807	POT1
4	30992815	31092815	0.382313	POT1
4	30992974	31092974	0.386348	POT1
4	30993178	31093178	0.435738	POT1
4	30993270	31093270	0.430123	POT1
4	30993374	31093374	0.4294	POT1
4	30993378	31093378	0.4294	POT1
4	30993569	31093569	0.448255	POT1
4	30993570	31093570	0.446847	POT1
4	30993625	31093625	0.43572	POT1
4	30993670	31093670	0.438876	POT1
4	30993707	31093707	0.43563	POT1
4	30993718	31093718	0.434761	POT1
4	30993765	31093765	0.434761	POT1
4	30994041	31094041	0.437428	POT1
4	30994161	31094161	0.449606	POT1
4	30994300	31094300	0.445821	POT1
4	30994406	31094406	0.436657	POT1
4	30994471	31094471	0.444856	POT1
4	30994517	31094517	0.414758	POT1
4	30994641	31094641	0.412547	POT1
4	30994705	31094705	0.41532	POT1
4	30994834	31094834	0.411753	POT1
4	30995000	31095000	0.40614	POT1
4	30995029	31095029	0.40614	POT1
4	30995041	31095041	0.4115	POT1
4	30995078	31095078	0.4115	POT1
4	30995150	31095150	0.411136	POT1
4	30995224	31095224	0.41479	POT1
4	30995234	31095234	0.412848	POT1
4	30995313	31095313	0.412848	POT1
4	30995386	31095386	0.412848	POT1
4	30995405	31095405	0.449417	POT1
4	30995539	31095539	0.459087	POT1
4	30995567	31095567	0.462687	POT1
4	30995570	31095570	0.463798	POT1
4	30995576	31095576	0.463853	POT1
4	30995580	31095580	0.463218	POT1
4	30995675	31095675	0.465569	POT1
4	30996137	31096137	0.451882	POT1

4	30996190	31096190	0.455951	POT1
4	30996330	31096330	0.454433	POT1
4	30996425	31096425	0.450023	POT1
4	30996457	31096457	0.450023	POT1
4	30996801	31096801	0.454407	POT1
4	30996933	31096933	0.450249	POT1
4	30996965	31096965	0.448859	POT1
4	31122893	31222893	0.356737	GPR37
4	31122950	31222950	0.354206	GPR37
4	31123162	31223162	0.363575	GPR37
4	31123166	31223166	0.350734	GPR37
4	31123174	31223174	0.345895	GPR37
4	31126413	31226413	0.356356	GPR37
4	32839172	32939172	0.395089	CADPS2
4	32839175	32939175	0.395089	CADPS2
4	32847859	32947859	0.350014	CADPS2
4	32892921	32992921	0.347601	CADPS2
4	32893062	32993062	0.34884	CADPS2
4	32893084	32993084	0.348974	CADPS2
4	32893204	32993204	0.346052	CADPS2
4	32893265	32993265	0.346052	CADPS2
4	32899811	32999811	0.343327	CADPS2
4	32900161	33000161	0.348218	CADPS2
4	32900284	33000284	0.344238	CADPS2
4	32900325	33000325	0.343429	CADPS2
4	32900606	33000606	0.344369	CADPS2
4	32900628	33000628	0.34254	CADPS2
4	32900631	33000631	0.34254	CADPS2
4	32900671	33000671	0.370285	CADPS2
4	32900734	33000734	0.370515	CADPS2
4	32900743	33000743	0.367369	CADPS2
4	32900924	33000924	0.367369	CADPS2
4	32900954	33000954	0.371086	CADPS2
4	32900955	33000955	0.365583	CADPS2
4	32900987	33000987	0.365583	CADPS2
4	32901008	33001008	0.36769	CADPS2
4	32901029	33001029	0.366709	CADPS2
4	32901159	33001159	0.367478	CADPS2
4	32901201	33001201	0.369182	CADPS2
4	32901221	33001221	0.370132	CADPS2
4	32901235	33001235	0.368397	CADPS2
4	32901435	33001435	0.367475	CADPS2
4	32901443	33001443	0.362868	CADPS2
4	32901470	33001470	0.373692	CADPS2
4	32901531	33001531	0.366395	CADPS2
4	32901534	33001534	0.368042	CADPS2
4	32901666	33001666	0.360919	CADPS2
4	32901813	33001813	0.370838	CADPS2
4	32901849	33001849	0.355142	CADPS2
4	32902529	33002529	0.355142	CADPS2
4	32902539	33002539	0.37166	CADPS2
4	32902569	33002569	0.367608	CADPS2
4	32902611	33002611	0.367796	CADPS2
4	32902618	33002618	0.370155	CADPS2
4	32902838	33002838	0.366495	CADPS2
4	32902853	33002853	0.367822	CADPS2
4	32902882	33002882	0.367339	CADPS2
4	32903009	33003009	0.367339	CADPS2

4	32903010	33003010	0.347732	CADPS2
4	32903095	33003095	0.354242	CADPS2
4	32903303	33003303	0.342531	CADPS2
4	32903563	33003563	0.345836	CADPS2
4	32903802	33003802	0.346227	CADPS2
4	32903869	33003869	0.349469	CADPS2
4	32903928	33003928	0.34722	CADPS2
4	32903972	33003972	0.350404	CADPS2
4	32904251	33004251	0.343451	CADPS2
4	32904399	33004399	0.346535	CADPS2
4	32904457	33004457	0.348406	CADPS2
4	32904479	33004479	0.34667	CADPS2
4	32904607	33004607	0.341715	CADPS2
4	32905093	33005093	0.34909	CADPS2
4	32910218	33010218	0.349566	CADPS2
4	32910355	33010355	0.360498	CADPS2
4	32910463	33010463	0.359911	CADPS2
4	32910531	33010531	0.363709	CADPS2
4	32910548	33010548	0.354931	CADPS2
4	32910673	33010673	0.342456	CADPS2
4	32910774	33010774	0.342456	CADPS2
4	32910939	33010939	0.34696	CADPS2
4	32911000	33011000	0.34696	CADPS2
4	32911037	33011037	0.346796	CADPS2
4	32911096	33011096	0.3497	CADPS2
4	32911126	33011126	0.350479	CADPS2
4	32911140	33011140	0.35	CADPS2
4	32911244	33011244	0.348488	CADPS2
4	32911429	33011429	0.349783	CADPS2
4	32911518	33011518	0.346987	CADPS2
4	32911616	33011616	0.349446	CADPS2
4	32911631	33011631	0.349446	CADPS2
4	32911661	33011661	0.349683	CADPS2
4	32911686	33011686	0.350165	CADPS2
4	32911766	33011766	0.348093	CADPS2
4	32911991	33011991	0.348702	CADPS2
4	32912335	33012335	0.346167	CADPS2
4	33065498	33165498	0.360197	RNF133
4	33065498	33165498	0.360197	CADPS2
4	33601386	33701386	0.343918	AASS
4	33601386	33701386	0.343918	PTPRZ1
4	35150928	35250928	0.342036	KCND2
4	39229026	39329026	0.343177	MPLKIP
4	39229026	39329026	0.343177	SUGCT
4	39254512	39354512	0.401682	SUGCT
4	39254518	39354518	0.401611	SUGCT
4	39254526	39354526	0.417135	SUGCT
4	39254539	39354539	0.408372	SUGCT
4	39254883	39354883	0.35644	SUGCT
4	39255180	39355180	0.352265	SUGCT
4	39255360	39355360	0.342308	SUGCT
4	39255882	39355882	0.342679	SUGCT
4	39255974	39355974	0.342679	SUGCT
4	39256075	39356075	0.343275	SUGCT
4	39256145	39356145	0.342652	SUGCT
4	39256425	39356425	0.369575	SUGCT
4	39256546	39356546	0.377238	SUGCT
4	39256886	39356886	0.369183	SUGCT

4	39256905	39356905	0.352021	SUGCT
4	39257066	39357066	0.347429	SUGCT
4	39257109	39357109	0.347429	SUGCT
4	39257678	39357678	0.348115	SUGCT
4	39258367	39358367	0.349621	SUGCT
4	39258368	39358368	0.349621	SUGCT
4	39258434	39358434	0.367795	SUGCT
4	39259023	39359023	0.364709	SUGCT
4	41051813	41151813	0.37228	GLI3
4	42017833	42117833	0.345413	C4H7orf25
4	42017833	42117833	0.345413	MRPL32
4	42017833	42117833	0.345413	PSMA2
4	42080851	42180851	0.352969	LOC106502047
4	42080941	42180941	0.349155	LOC106502047
4	42080954	42180954	0.356532	LOC106502047
4	42081034	42181034	0.355789	LOC106502047
4	42081093	42181093	0.35688	LOC106502047
4	42081123	42181123	0.347823	LOC106502047
4	42081162	42181162	0.411478	LOC106502047
4	42081223	42181223	0.398673	LOC106502047
4	42081285	42181285	0.396533	LOC106502047
4	42081293	42181293	0.372876	LOC106502047
4	42081335	42181335	0.376	LOC106502047
4	42081412	42181412	0.346884	LOC106502047
4	42081413	42181413	0.346884	LOC106502047
4	42081964	42181964	0.345704	LOC106502047
4	42082136	42182136	0.345704	LOC106502047
4	42082252	42182252	0.345704	LOC106502047
4	42082370	42182370	0.344904	LOC106502047
4	42082571	42182571	0.354937	LOC106502047
4	42082610	42182610	0.350583	LOC106502047
4	42082867	42182867	0.355625	LOC106502047
4	42082894	42182894	0.359658	LOC106502047
4	42082918	42182918	0.354538	LOC106502047
4	42083025	42183025	0.368229	LOC106502047
4	42083050	42183050	0.369391	LOC106502047
4	42083189	42183189	0.353738	LOC106502047
4	42083367	42183367	0.357492	LOC106502047
4	42083536	42183536	0.354293	LOC106502047
4	42083670	42183670	0.350741	LOC106502047
4	42085660	42185660	0.346116	LOC106502047
4	42086411	42186411	0.353208	LOC106502047
4	42086424	42186424	0.348732	LOC106502047
4	42086490	42186490	0.349752	LOC106502047
4	42086497	42186497	0.349752	LOC106502047
4	42086510	42186510	0.345755	LOC106502047
4	42086585	42186585	0.345755	LOC106502047
4	42086592	42186592	0.345755	LOC106502047
4	42086712	42186712	0.345627	LOC106502047
4	42086735	42186735	0.355625	LOC106502047
4	42086745	42186745	0.354751	LOC106502047
4	42086811	42186811	0.374104	LOC106502047
4	42086831	42186831	0.350069	LOC106502047
4	42086915	42186915	0.350069	LOC106502047
4	42086981	42186981	0.350201	LOC106502047
4	42087018	42187018	0.345435	LOC106502047
4	42087038	42187038	0.345435	LOC106502047
4	42087402	42187402	0.352805	LOC106502047

4	42087445	42187445	0.350606	LOC106502047
4	42087452	42187452	0.344614	LOC106502047
4	42087512	42187512	0.34784	LOC106502047
4	42087605	42187605	0.34753	LOC106502047
4	42087882	42187882	0.345399	LOC106502047
4	42088180	42188180	0.352932	LOC106502047
4	42088205	42188205	0.347569	LOC106502047
4	42088269	42188269	0.377939	LOC106502047
4	42088337	42188337	0.346637	LOC106502047
4	42088554	42188554	0.353539	LOC106502047
4	42088687	42188687	0.351673	LOC106502047
4	42088702	42188702	0.351673	LOC106502047
4	42090914	42190914	0.356302	LOC106502047
4	42090918	42190918	0.359338	LOC106502047
4	42091106	42191106	0.354551	LOC106502047
4	42091415	42191415	0.38474	LOC106502047
4	42091464	42191464	0.356135	LOC106502047
4	42092599	42192599	0.355409	LOC106502047
4	42129386	42229386	0.342346	LOC106502047
4	42129553	42229553	0.38077	LOC106502047
4	42129632	42229632	0.395872	LOC106502047
4	42129643	42229643	0.395872	LOC106502047
4	42129645	42229645	0.395872	LOC106502047
4	42129664	42229664	0.372587	LOC106502047
4	42129670	42229670	0.372587	LOC106502047
4	42129755	42229755	0.393881	LOC106502047
4	42129867	42229867	0.392907	LOC106502047
4	42129912	42229912	0.368615	LOC106502047
4	42129921	42229921	0.366643	LOC106502047
4	42129933	42229933	0.36557	LOC106502047
4	42130132	42230132	0.371863	LOC106502047
4	42130150	42230150	0.388589	LOC106502047
4	42130167	42230167	0.377479	LOC106502047
4	42130188	42230188	0.377479	LOC106502047
4	42130201	42230201	0.379982	LOC106502047
4	42130245	42230245	0.377825	LOC106502047
4	42130385	42230385	0.382022	LOC106502047
4	42130391	42230391	0.378633	LOC106502047
4	42130562	42230562	0.363246	LOC106502047
4	42130767	42230767	0.372182	LOC106502047
4	42470160	42570160	0.382689	HECW1
4	42533571	42633571	0.357439	HECW1
4	42533656	42633656	0.358094	HECW1
4	42533678	42633678	0.346462	HECW1
4	42533770	42633770	0.371533	HECW1
4	42533779	42633779	0.36496	HECW1
4	42533819	42633819	0.406703	HECW1
4	42533861	42633861	0.402511	HECW1
4	42533885	42633885	0.407892	HECW1
4	42533938	42633938	0.407892	HECW1
4	42533948	42633948	0.407892	HECW1
4	42533997	42633997	0.407529	HECW1
4	42534044	42634044	0.405126	HECW1
4	42534135	42634135	0.4098	HECW1
4	42534180	42634180	0.39259	HECW1
4	42534253	42634253	0.446042	HECW1
4	42534405	42634405	0.445762	HECW1
4	43133160	43233160	0.346341	CAMK2B

4	43133160	43233160	0.346341	NUDCD3
4	43137345	43237345	0.349236	CAMK2B
4	43137345	43237345	0.349236	NUDCD3
4	43137401	43237401	0.381133	CAMK2B
4	43137401	43237401	0.381133	NUDCD3
4	43137722	43237722	0.383788	CAMK2B
4	43137722	43237722	0.383788	NUDCD3
4	43137799	43237799	0.355837	CAMK2B
4	43137799	43237799	0.355837	NUDCD3
4	43137952	43237952	0.35524	CAMK2B
4	43137952	43237952	0.35524	NUDCD3
4	43137982	43237982	0.366128	CAMK2B
4	43137982	43237982	0.366128	NUDCD3
4	43138036	43238036	0.355694	CAMK2B
4	43138036	43238036	0.355694	NUDCD3
4	43138401	43238401	0.361594	CAMK2B
4	43138401	43238401	0.361594	NUDCD3
4	43138424	43238424	0.359915	CAMK2B
4	43138424	43238424	0.359915	NUDCD3
4	43138586	43238586	0.361508	CAMK2B
4	43138586	43238586	0.361508	NUDCD3
4	43138597	43238597	0.366362	CAMK2B
4	43138597	43238597	0.366362	NUDCD3
4	43138668	43238668	0.366029	CAMK2B
4	43138668	43238668	0.366029	NUDCD3
4	43138823	43238823	0.371661	CAMK2B
4	43138823	43238823	0.371661	NUDCD3
4	43138854	43238854	0.359469	CAMK2B
4	43138854	43238854	0.359469	NUDCD3
4	43138917	43238917	0.364759	CAMK2B
4	43138917	43238917	0.364759	NUDCD3
4	43139082	43239082	0.374767	CAMK2B
4	43139082	43239082	0.374767	NUDCD3
4	43139107	43239107	0.370582	CAMK2B
4	43139107	43239107	0.370582	NUDCD3
4	43139252	43239252	0.368846	CAMK2B
4	43139252	43239252	0.368846	NUDCD3
4	43139324	43239324	0.368614	CAMK2B
4	43139324	43239324	0.368614	NUDCD3
4	43139327	43239327	0.356774	CAMK2B
4	43139327	43239327	0.356774	NUDCD3
4	43139395	43239395	0.358954	CAMK2B
4	43139395	43239395	0.358954	NUDCD3
4	43139590	43239590	0.355924	CAMK2B
4	43139590	43239590	0.355924	NUDCD3
4	43139790	43239790	0.349542	CAMK2B
4	43139790	43239790	0.349542	NUDCD3
4	53116225	53216225	0.370804	CPVL
4	53116265	53216265	0.358169	CPVL
4	53116295	53216295	0.35109	CPVL
4	53116421	53216421	0.351035	CPVL
4	53116422	53216422	0.362659	CPVL
4	53120097	53220097	0.347886	CPVL
4	53123690	53223690	0.359929	CPVL
4	53123847	53223847	0.350417	CPVL
4	53133157	53233157	0.346691	CPVL
4	53133254	53233254	0.353014	CPVL
4	53133835	53233835	0.34997	CPVL

4	53133929	53233929	0.356427	CPVL
4	53133990	53233990	0.365785	CPVL
4	53134509	53234509	0.355708	CPVL
4	53134652	53234652	0.366072	CPVL
4	53134671	53234671	0.374832	CPVL
4	53134860	53234860	0.3696	CPVL
4	53134884	53234884	0.377775	CPVL
4	53134962	53234962	0.362971	CPVL
4	53134987	53234987	0.362971	CPVL
4	53135073	53235073	0.360233	CPVL
4	53135209	53235209	0.37365	CPVL
4	53135298	53235298	0.364601	CPVL
4	53135302	53235302	0.356736	CPVL
4	53135332	53235332	0.349868	CPVL
4	53135338	53235338	0.349868	CPVL
4	53135565	53235565	0.364599	CPVL
4	53135618	53235618	0.342291	CPVL
4	53135708	53235708	0.354307	CPVL
4	53135851	53235851	0.346778	CPVL
4	53136024	53236024	0.360409	CPVL
4	53136064	53236064	0.34772	CPVL
4	53136066	53236066	0.359	CPVL
4	53136153	53236153	0.35994	CPVL
4	53136155	53236155	0.35994	CPVL
4	53136156	53236156	0.35994	CPVL
4	53136169	53236169	0.358703	CPVL
4	53136288	53236288	0.366039	CPVL
4	53136289	53236289	0.366763	CPVL
4	53136372	53236372	0.356859	CPVL
4	53136375	53236375	0.341966	CPVL
4	53136434	53236434	0.341966	CPVL
4	53136586	53236586	0.342372	CPVL
4	53136640	53236640	0.351707	CPVL
4	53137728	53237728	0.352969	CPVL
4	53140140	53240140	0.352335	CPVL
4	53140192	53240192	0.344581	CPVL
4	53140306	53240306	0.345536	CPVL
4	53140406	53240406	0.353797	CPVL
4	53140548	53240548	0.3591	CPVL
4	53140599	53240599	0.366153	CPVL
4	53140805	53240805	0.350822	CPVL
4	53140964	53240964	0.356658	CPVL
4	53141066	53241066	0.356188	CPVL
4	53141203	53241203	0.361542	CPVL
4	53141368	53241368	0.349216	CPVL
4	53141664	53241664	0.359566	CPVL
4	53141714	53241714	0.376105	CPVL
4	53141839	53241839	0.37142	CPVL
4	53141899	53241899	0.379481	CPVL
4	53141922	53241922	0.368577	CPVL
4	53141946	53241946	0.387468	CPVL
4	53142018	53242018	0.377694	CPVL
4	53142020	53242020	0.378743	CPVL
4	53142029	53242029	0.378743	CPVL
4	53142044	53242044	0.350626	CPVL
4	53142132	53242132	0.356934	CPVL
4	53142274	53242274	0.3595	CPVL
4	53142333	53242333	0.352727	CPVL

4	53142924	53242924	0.349633	CPVL
4	53143026	53243026	0.359379	CPVL
4	53143116	53243116	0.373702	CPVL
4	53143174	53243174	0.368428	CPVL
4	53143245	53243245	0.348515	CPVL
4	53143642	53243642	0.343118	CPVL
4	53143753	53243753	0.353546	CPVL
4	53143790	53243790	0.354223	CPVL
4	53143809	53243809	0.354223	CPVL
4	53143854	53243854	0.359715	CPVL
4	53143857	53243857	0.360376	CPVL
4	53143911	53243911	0.359237	CPVL
4	53144130	53244130	0.355998	CPVL
4	53149145	53249145	0.354934	CPVL
4	53149170	53249170	0.3891	CPVL
4	53149329	53249329	0.39816	CPVL
4	53149388	53249388	0.405275	CPVL
4	53149454	53249454	0.414467	CPVL
4	53149590	53249590	0.405771	CPVL
4	53149630	53249630	0.402841	CPVL
4	53149726	53249726	0.394905	CPVL
4	53149784	53249784	0.393471	CPVL
4	53149785	53249785	0.399739	CPVL
4	53149819	53249819	0.396939	CPVL
4	53149906	53249906	0.388932	CPVL
4	53149999	53249999	0.37655	CPVL
4	53150063	53250063	0.378192	CPVL
4	53150072	53250072	0.381286	CPVL
4	53150105	53250105	0.381286	CPVL
4	53150112	53250112	0.377411	CPVL
4	53150243	53250243	0.367953	CPVL
4	53150411	53250411	0.364513	CPVL
4	53150816	53250816	0.370313	CPVL
4	53150932	53250932	0.367916	CPVL
4	53150965	53250965	0.37151	CPVL
4	53150967	53250967	0.37151	CPVL
4	53151000	53251000	0.342953	CPVL
4	53151014	53251014	0.355087	CPVL
4	53151382	53251382	0.391656	CPVL
4	53151531	53251531	0.371301	CPVL
4	53151655	53251655	0.388636	CPVL
4	53151674	53251674	0.372758	CPVL
4	53151907	53251907	0.361339	CPVL
4	53152021	53252021	0.374507	CPVL
4	53152265	53252265	0.368064	CPVL
4	53152321	53252321	0.377437	CPVL
4	53152436	53252436	0.380832	CPVL
4	53152642	53252642	0.37463	CPVL
4	53152666	53252666	0.37463	CPVL
4	53152674	53252674	0.37463	CPVL
4	53152741	53252741	0.381033	CPVL
4	53152906	53252906	0.367183	CPVL
4	53153008	53253008	0.368268	CPVL
4	53153219	53253219	0.349359	CPVL
4	53153392	53253392	0.35535	CPVL
4	53153580	53253580	0.35535	CPVL
4	53153658	53253658	0.356461	CPVL
4	53153931	53253931	0.383233	CPVL

4	53154034	53254034	0.382664	CPVL
4	53154152	53254152	0.382468	CPVL
4	53154211	53254211	0.384993	CPVL
4	53154253	53254253	0.379728	CPVL
4	53154289	53254289	0.410174	CPVL
4	53154301	53254301	0.410174	CPVL
4	53154303	53254303	0.410174	CPVL
4	53154418	53254418	0.416902	CPVL
4	53154547	53254547	0.39566	CPVL
4	53154611	53254611	0.394039	CPVL
4	53154745	53254745	0.405224	CPVL
4	53154770	53254770	0.412292	CPVL
4	53155124	53255124	0.415264	CPVL
4	53155141	53255141	0.418688	CPVL
4	53155159	53255159	0.416727	CPVL
4	53155268	53255268	0.418187	CPVL
4	53155274	53255274	0.418187	CPVL
4	53155367	53255367	0.414008	CPVL
4	53155427	53255427	0.409083	CPVL
4	53155496	53255496	0.408717	CPVL
4	53155690	53255690	0.407677	CPVL
4	53155780	53255780	0.410368	CPVL
4	53155893	53255893	0.402965	CPVL
4	53155912	53255912	0.396212	CPVL
4	53156040	53256040	0.396212	CPVL
4	53156067	53256067	0.409468	CPVL
4	53156125	53256125	0.395571	CPVL
4	53156374	53256374	0.404952	CPVL
4	53156474	53256474	0.407641	CPVL
4	53156488	53256488	0.418592	CPVL
4	53156690	53256690	0.417988	CPVL
4	53156716	53256716	0.419034	CPVL
4	53156749	53256749	0.418359	CPVL
4	53156804	53256804	0.415314	CPVL
4	53156813	53256813	0.412667	CPVL
4	53157068	53257068	0.414258	CPVL
4	53157069	53257069	0.413335	CPVL
4	53157158	53257158	0.426915	CPVL
4	53157192	53257192	0.418391	CPVL
4	53157219	53257219	0.41569	CPVL
4	53157320	53257320	0.415467	CPVL
4	53157377	53257377	0.417923	CPVL
4	53157380	53257380	0.417923	CPVL
4	53157395	53257395	0.411685	CPVL
4	53157405	53257405	0.411462	CPVL
4	53157419	53257419	0.409694	CPVL
4	53157468	53257468	0.411082	CPVL
4	53157546	53257546	0.415779	CPVL
4	53157570	53257570	0.421558	CPVL
4	53157575	53257575	0.420172	CPVL
4	53157683	53257683	0.413685	CPVL
4	53157697	53257697	0.412163	CPVL
4	53157816	53257816	0.412052	CPVL
4	53157862	53257862	0.417063	CPVL
4	53158210	53258210	0.43006	CPVL
4	53158217	53258217	0.441352	CPVL
4	53158410	53258410	0.398167	CPVL
4	53158416	53258416	0.432788	CPVL

4	53158427	53258427	0.433033	CPVL
4	53158515	53258515	0.391189	CPVL
4	53158544	53258544	0.398067	CPVL
4	53158606	53258606	0.409965	CPVL
4	53158712	53258712	0.390775	CPVL
4	53158740	53258740	0.395501	CPVL
4	53158819	53258819	0.367884	CPVL
4	53158964	53258964	0.37547	CPVL
4	53158967	53258967	0.381188	CPVL
4	53158976	53258976	0.373617	CPVL
4	53159109	53259109	0.374566	CPVL
4	53159410	53259410	0.382011	CPVL
4	53159432	53259432	0.36892	CPVL
4	53160916	53260916	0.355428	CPVL
4	53160998	53260998	0.347938	CPVL
4	53161101	53261101	0.353874	CPVL
4	53161337	53261337	0.357372	CPVL
4	53161351	53261351	0.345807	CPVL
4	53161484	53261484	0.34443	CPVL
4	53161587	53261587	0.34443	CPVL
4	53161760	53261760	0.353002	CPVL
4	53161863	53261863	0.370135	CPVL
4	53162116	53262116	0.351436	CPVL
4	53162342	53262342	0.361862	CPVL
4	53162342	53262342	0.361862	CHN2
4	53162526	53262526	0.359517	CPVL
4	53162526	53262526	0.359517	CHN2
4	53162658	53262658	0.363388	CPVL
4	53162658	53262658	0.363388	CHN2
4	53162724	53262724	0.365788	CPVL
4	53162724	53262724	0.365788	CHN2
4	53162836	53262836	0.346316	CPVL
4	53162836	53262836	0.346316	CHN2
4	53162920	53262920	0.369842	CPVL
4	53162920	53262920	0.369842	CHN2
4	53163000	53263000	0.362356	CPVL
4	53163000	53263000	0.362356	CHN2
4	53163157	53263157	0.382284	CPVL
4	53163157	53263157	0.382284	CHN2
4	53163415	53263415	0.369276	CPVL
4	53163415	53263415	0.369276	CHN2
4	53163468	53263468	0.39602	CPVL
4	53163468	53263468	0.39602	CHN2
4	53163529	53263529	0.377664	CPVL
4	53163529	53263529	0.377664	CHN2
4	53163558	53263558	0.372528	CPVL
4	53163558	53263558	0.372528	CHN2
4	53163603	53263603	0.371858	CPVL
4	53163603	53263603	0.371858	CHN2
4	53163638	53263638	0.371782	CPVL
4	53163638	53263638	0.371782	CHN2
4	53163729	53263729	0.371985	CPVL
4	53163729	53263729	0.371985	CHN2
4	53163759	53263759	0.371985	CPVL
4	53163759	53263759	0.371985	CHN2
4	53163845	53263845	0.354906	CPVL
4	53163845	53263845	0.354906	CHN2
4	53163859	53263859	0.386849	CPVL

4	53163859	53263859	0.386849	CHN2
4	53163865	53263865	0.373006	CPVL
4	53163865	53263865	0.373006	CHN2
4	53163999	53263999	0.347067	CPVL
4	53163999	53263999	0.347067	CHN2
4	53169658	53269658	0.347008	CPVL
4	53169658	53269658	0.347008	CHN2
4	53172598	53272598	0.342565	CPVL
4	53172598	53272598	0.342565	CHN2
4	53172620	53272620	0.342893	CPVL
4	53172620	53272620	0.342893	CHN2
4	53172621	53272621	0.34507	CPVL
4	53172621	53272621	0.34507	CHN2
4	53172622	53272622	0.34507	CPVL
4	53172622	53272622	0.34507	CHN2
4	53172700	53272700	0.342378	CPVL
4	53172700	53272700	0.342378	CHN2
4	53174978	53274978	0.351076	CPVL
4	53174978	53274978	0.351076	CHN2
4	53175050	53275050	0.353177	CPVL
4	53175050	53275050	0.353177	CHN2
4	53175080	53275080	0.352062	CPVL
4	53175080	53275080	0.352062	CHN2
4	53175141	53275141	0.353215	CPVL
4	53175141	53275141	0.353215	CHN2
4	53175241	53275241	0.350505	CPVL
4	53175241	53275241	0.350505	CHN2
4	53175291	53275291	0.353629	CPVL
4	53175291	53275291	0.353629	CHN2
4	53175306	53275306	0.353124	CPVL
4	53175306	53275306	0.353124	CHN2
4	53175313	53275313	0.360525	CPVL
4	53175313	53275313	0.360525	CHN2
4	53175328	53275328	0.370157	CPVL
4	53175328	53275328	0.370157	CHN2
4	53175329	53275329	0.360204	CPVL
4	53175329	53275329	0.360204	CHN2
4	53175359	53275359	0.361589	CPVL
4	53175359	53275359	0.361589	CHN2
4	53175465	53275465	0.353179	CPVL
4	53175465	53275465	0.353179	CHN2
4	53175599	53275599	0.356492	CPVL
4	53175599	53275599	0.356492	CHN2
4	53175600	53275600	0.3566	CPVL
4	53175600	53275600	0.3566	CHN2
4	53175628	53275628	0.349311	CPVL
4	53175628	53275628	0.349311	CHN2
4	53175641	53275641	0.349369	CPVL
4	53175641	53275641	0.349369	CHN2
4	53175914	53275914	0.358387	CPVL
4	53175914	53275914	0.358387	CHN2
4	53175963	53275963	0.350548	CPVL
4	53175963	53275963	0.350548	CHN2
4	53175971	53275971	0.350548	CPVL
4	53175971	53275971	0.350548	CHN2
4	53176122	53276122	0.350548	CPVL
4	53176122	53276122	0.350548	CHN2
4	53176253	53276253	0.358477	CPVL

4	53176253	53276253	0.358477	CHN2
4	53176313	53276313	0.356295	CPVL
4	53176313	53276313	0.356295	CHN2
4	53176370	53276370	0.35491	CPVL
4	53176370	53276370	0.35491	CHN2
4	53176407	53276407	0.356379	CPVL
4	53176407	53276407	0.356379	CHN2
4	53176514	53276514	0.350429	CPVL
4	53176514	53276514	0.350429	CHN2
4	53179377	53279377	0.353752	CPVL
4	53179377	53279377	0.353752	CHN2
4	53179395	53279395	0.342522	CPVL
4	53179395	53279395	0.342522	CHN2
4	53179499	53279499	0.344132	CPVL
4	53179499	53279499	0.344132	CHN2
4	53179600	53279600	0.346214	CPVL
4	53179600	53279600	0.346214	CHN2
4	53179636	53279636	0.350464	CPVL
4	53179636	53279636	0.350464	CHN2
4	53179676	53279676	0.350464	CPVL
4	53179676	53279676	0.350464	CHN2
4	53179729	53279729	0.35083	CPVL
4	53179729	53279729	0.35083	CHN2
4	53179874	53279874	0.349047	CPVL
4	53179874	53279874	0.349047	CHN2
4	53179885	53279885	0.363612	CPVL
4	53179885	53279885	0.363612	CHN2
4	53179907	53279907	0.358024	CPVL
4	53179907	53279907	0.358024	CHN2
4	53179931	53279931	0.358024	CPVL
4	53179931	53279931	0.358024	CHN2
4	53179933	53279933	0.358255	CPVL
4	53179933	53279933	0.358255	CHN2
4	53179940	53279940	0.358255	CPVL
4	53179940	53279940	0.358255	CHN2
4	53180028	53280028	0.355732	CPVL
4	53180028	53280028	0.355732	CHN2
4	53180082	53280082	0.356708	CPVL
4	53180082	53280082	0.356708	CHN2
4	53180204	53280204	0.35966	CPVL
4	53180204	53280204	0.35966	CHN2
4	53180209	53280209	0.360007	CPVL
4	53180209	53280209	0.360007	CHN2
4	53180231	53280231	0.352098	CPVL
4	53180231	53280231	0.352098	CHN2
4	53180291	53280291	0.35797	CPVL
4	53180291	53280291	0.35797	CHN2
4	53180292	53280292	0.356513	CPVL
4	53180292	53280292	0.356513	CHN2
4	53180307	53280307	0.358577	CPVL
4	53180307	53280307	0.358577	CHN2
4	53180319	53280319	0.361644	CPVL
4	53180319	53280319	0.361644	CHN2
4	53180330	53280330	0.361644	CPVL
4	53180330	53280330	0.361644	CHN2
4	53180339	53280339	0.360175	CPVL
4	53180339	53280339	0.360175	CHN2
4	53180389	53280389	0.355356	CPVL

4	53180389	53280389	0.355356	CHN2
4	53180448	53280448	0.361478	CPVL
4	53180448	53280448	0.361478	CHN2
4	53180455	53280455	0.362696	CPVL
4	53180455	53280455	0.362696	CHN2
4	53180458	53280458	0.367112	CPVL
4	53180458	53280458	0.367112	CHN2
4	53180505	53280505	0.366477	CPVL
4	53180505	53280505	0.366477	CHN2
4	53180527	53280527	0.3572	CPVL
4	53180527	53280527	0.3572	CHN2
4	53180529	53280529	0.356569	CPVL
4	53180529	53280529	0.356569	CHN2
4	53809172	53909172	0.348528	FKBP14
4	53809172	53909172	0.348528	PLEKHA8
4	53809172	53909172	0.348528	WIPF3
4	53809172	53909172	0.348528	SCRN1
4	53809264	53909264	0.346307	FKBP14
4	53809264	53909264	0.346307	PLEKHA8
4	53809264	53909264	0.346307	WIPF3
4	53809264	53909264	0.346307	SCRN1
4	53809375	53909375	0.35957	FKBP14
4	53809375	53909375	0.35957	PLEKHA8
4	53809375	53909375	0.35957	SCRN1
4	53809391	53909391	0.357638	FKBP14
4	53809391	53909391	0.357638	PLEKHA8
4	53809391	53909391	0.357638	SCRN1
4	53809394	53909394	0.351237	FKBP14
4	53809394	53909394	0.351237	PLEKHA8
4	53809394	53909394	0.351237	SCRN1
4	53809487	53909487	0.358178	FKBP14
4	53809487	53909487	0.358178	PLEKHA8
4	53809487	53909487	0.358178	SCRN1
4	53809515	53909515	0.358178	FKBP14
4	53809515	53909515	0.358178	PLEKHA8
4	53809515	53909515	0.358178	SCRN1
4	53809675	53909675	0.353711	FKBP14
4	53809675	53909675	0.353711	PLEKHA8
4	53809675	53909675	0.353711	SCRN1
4	53809678	53909678	0.348732	FKBP14
4	53809678	53909678	0.348732	PLEKHA8
4	53809678	53909678	0.348732	SCRN1
4	53809756	53909756	0.344208	FKBP14
4	53809756	53909756	0.344208	PLEKHA8
4	53809756	53909756	0.344208	SCRN1
4	53809971	53909971	0.347268	FKBP14
4	53809971	53909971	0.347268	PLEKHA8
4	53809971	53909971	0.347268	SCRN1
4	53810039	53910039	0.342637	FKBP14
4	53810039	53910039	0.342637	PLEKHA8
4	53810039	53910039	0.342637	SCRN1
4	55318224	55418224	0.389158	CCDC129
4	55318270	55418270	0.390231	CCDC129
4	55318369	55418369	0.360992	CCDC129
4	55318495	55418495	0.43683	CCDC129
4	55321169	55421169	0.341922	CCDC129
4	55321170	55421170	0.345707	CCDC129
4	55322707	55422707	0.346844	CCDC129

4	55322957	55422957	0.363923	CCDC129
4	55323041	55423041	0.343138	CCDC129
4	55323083	55423083	0.351754	CCDC129
4	55323111	55423111	0.357781	CCDC129
4	55326874	55426874	0.358168	CCDC129
4	55782887	55882887	0.352718	LOC102180455
4	55782895	55882895	0.366611	LOC102180455
4	55782960	55882960	0.394351	LOC102180455
4	55783170	55883170	0.367322	LOC102180455
4	55783188	55883188	0.359455	LOC102180455
4	55931485	56031485	0.366944	LOC108635899
4	55931731	56031731	0.373977	LOC108635899
4	55931808	56031808	0.388139	LOC108635899
4	55931837	56031837	0.420732	LOC108635899
4	55931875	56031875	0.414511	LOC108635899
4	55931934	56031934	0.409547	LOC108635899
4	55931935	56031935	0.409547	LOC108635899
4	55932019	56032019	0.413564	LOC108635899
4	55932034	56032034	0.413564	LOC108635899
4	55932077	56032077	0.417686	LOC108635899
4	55932121	56032121	0.439828	LOC108635899
4	55932162	56032162	0.42413	LOC108635899
4	55932257	56032257	0.409014	LOC108635899
4	55932355	56032355	0.421241	LOC108635899
4	55932459	56032459	0.419959	LOC108635899
4	55932517	56032517	0.420645	LOC108635899
4	55932643	56032643	0.387594	LOC108635899
4	55932700	56032700	0.404232	LOC108635899
4	55932853	56032853	0.38614	LOC108635899
4	55935189	56035189	0.373922	LOC108635899
4	55936140	56036140	0.348566	LOC108635899
4	55936254	56036254	0.418444	LOC108635899
4	55936827	56036827	0.402668	LOC108635899
4	55937203	56037203	0.409648	LOC108635899
4	55937696	56037696	0.390532	LOC108635899
4	55937713	56037713	0.360876	LOC108635899
4	55937714	56037714	0.360876	LOC108635899
4	55938098	56038098	0.356761	LOC108635899
4	56305623	56405623	0.343487	KBTBD2
4	56305623	56405623	0.343487	FKBP9
4	56305623	56405623	0.343487	LOC102179628
4	56306291	56406291	0.347979	KBTBD2
4	56306291	56406291	0.347979	FKBP9
4	56306291	56406291	0.347979	LOC102179628
4	56306388	56406388	0.36799	KBTBD2
4	56306388	56406388	0.36799	FKBP9
4	56306388	56406388	0.36799	LOC102179628
4	56306416	56406416	0.350343	KBTBD2
4	56306416	56406416	0.350343	FKBP9
4	56306416	56406416	0.350343	LOC102179628
4	56306636	56406636	0.350405	KBTBD2
4	56306636	56406636	0.350405	FKBP9
4	56306636	56406636	0.350405	LOC102179628
4	56306682	56406682	0.350243	KBTBD2
4	56306682	56406682	0.350243	FKBP9
4	56306682	56406682	0.350243	LOC102179628
4	56306687	56406687	0.350682	KBTBD2
4	56306687	56406687	0.350682	FKBP9

4	56306687	56406687	0.350682	LOC102179628
4	56306760	56406760	0.349318	KBTBD2
4	56306760	56406760	0.349318	FKBP9
4	56306760	56406760	0.349318	LOC102179628
4	56307338	56407338	0.370015	KBTBD2
4	56307338	56407338	0.370015	FKBP9
4	56307338	56407338	0.370015	LOC102179628
4	56307460	56407460	0.35116	KBTBD2
4	56307460	56407460	0.35116	FKBP9
4	56307460	56407460	0.35116	LOC102179628
4	56307508	56407508	0.352156	KBTBD2
4	56307508	56407508	0.352156	FKBP9
4	56307508	56407508	0.352156	LOC102179628
4	56307971	56407971	0.347795	KBTBD2
4	56307971	56407971	0.347795	FKBP9
4	56307971	56407971	0.347795	LOC102179628
4	56308312	56408312	0.346461	KBTBD2
4	56308312	56408312	0.346461	FKBP9
4	56308312	56408312	0.346461	LOC102179628
4	56308349	56408349	0.348363	KBTBD2
4	56308349	56408349	0.348363	FKBP9
4	56308349	56408349	0.348363	LOC102179628
4	56308529	56408529	0.348385	KBTBD2
4	56308529	56408529	0.348385	FKBP9
4	56308529	56408529	0.348385	LOC102179628
4	56310082	56410082	0.344795	KBTBD2
4	56310082	56410082	0.344795	FKBP9
4	56310082	56410082	0.344795	LOC102179628
4	56310083	56410083	0.344795	KBTBD2
4	56310083	56410083	0.344795	FKBP9
4	56310083	56410083	0.344795	LOC102179628
4	56310101	56410101	0.344795	KBTBD2
4	56310101	56410101	0.344795	FKBP9
4	56310101	56410101	0.344795	LOC102179628
4	56313644	56413644	0.383358	KBTBD2
4	56313644	56413644	0.383358	FKBP9
4	56313644	56413644	0.383358	LOC102179628
4	56313645	56413645	0.380079	KBTBD2
4	56313645	56413645	0.380079	FKBP9
4	56313645	56413645	0.380079	LOC102179628
4	56313751	56413751	0.362598	KBTBD2
4	56313751	56413751	0.362598	FKBP9
4	56313751	56413751	0.362598	LOC102179628
4	56313883	56413883	0.364674	KBTBD2
4	56313883	56413883	0.364674	FKBP9
4	56313883	56413883	0.364674	LOC102179628
4	56315208	56415208	0.343655	KBTBD2
4	56315208	56415208	0.343655	FKBP9
4	56315208	56415208	0.343655	LOC102179628
4	56315261	56415261	0.345296	KBTBD2
4	56315261	56415261	0.345296	FKBP9
4	56315261	56415261	0.345296	LOC102179628
4	56317151	56417151	0.348476	KBTBD2
4	56317151	56417151	0.348476	FKBP9
4	56317151	56417151	0.348476	LOC102179628
4	56318178	56418178	0.360478	KBTBD2
4	56318178	56418178	0.360478	FKBP9
4	56318178	56418178	0.360478	LOC102179628

4	56318414	56418414	0.37283	KBTBD2
4	56318414	56418414	0.37283	FKBP9
4	56318414	56418414	0.37283	LOC102179628
4	56319003	56419003	0.369593	KBTBD2
4	56319003	56419003	0.369593	FKBP9
4	56319003	56419003	0.369593	LOC102179628
4	56319110	56419110	0.369394	KBTBD2
4	56319110	56419110	0.369394	FKBP9
4	56319110	56419110	0.369394	LOC102179628
4	56319648	56419648	0.368455	KBTBD2
4	56319648	56419648	0.368455	FKBP9
4	56319648	56419648	0.368455	LOC102179628
4	56319808	56419808	0.368455	KBTBD2
4	56319808	56419808	0.368455	FKBP9
4	56319808	56419808	0.368455	LOC102179628
4	56320325	56420325	0.371673	KBTBD2
4	56320325	56420325	0.371673	FKBP9
4	56320325	56420325	0.371673	LOC102179628
4	56320376	56420376	0.371228	KBTBD2
4	56320376	56420376	0.371228	FKBP9
4	56320376	56420376	0.371228	LOC102179628
4	56320546	56420546	0.371908	KBTBD2
4	56320546	56420546	0.371908	FKBP9
4	56320546	56420546	0.371908	LOC102179628
4	56320575	56420575	0.371908	KBTBD2
4	56320575	56420575	0.371908	FKBP9
4	56320575	56420575	0.371908	LOC102179628
4	56320943	56420943	0.375808	KBTBD2
4	56320943	56420943	0.375808	FKBP9
4	56320943	56420943	0.375808	LOC102179628
4	56320952	56420952	0.375808	KBTBD2
4	56320952	56420952	0.375808	FKBP9
4	56320952	56420952	0.375808	LOC102179628
4	56320980	56420980	0.482397	KBTBD2
4	56320980	56420980	0.482397	FKBP9
4	56320980	56420980	0.482397	LOC102179628
4	56321222	56421222	0.39333	KBTBD2
4	56321222	56421222	0.39333	FKBP9
4	56321222	56421222	0.39333	LOC102179628
4	56321242	56421242	0.386763	KBTBD2
4	56321242	56421242	0.386763	FKBP9
4	56321242	56421242	0.386763	LOC102179628
4	56321367	56421367	0.395184	KBTBD2
4	56321367	56421367	0.395184	FKBP9
4	56321367	56421367	0.395184	LOC102179628
4	56321408	56421408	0.356083	KBTBD2
4	56321408	56421408	0.356083	FKBP9
4	56321408	56421408	0.356083	LOC102179628
4	56321437	56421437	0.345433	KBTBD2
4	56321437	56421437	0.345433	FKBP9
4	56321437	56421437	0.345433	LOC102179628
4	56322220	56422220	0.345556	KBTBD2
4	56322220	56422220	0.345556	FKBP9
4	56322220	56422220	0.345556	LOC102179628
4	56322335	56422335	0.345556	KBTBD2
4	56322335	56422335	0.345556	FKBP9
4	56322335	56422335	0.345556	LOC102179628
4	56322524	56422524	0.344328	KBTBD2

4	56322524	56422524	0.344328	FKBP9
4	56322524	56422524	0.344328	LOC102179628
4	56322578	56422578	0.347709	KBTBD2
4	56322578	56422578	0.347709	FKBP9
4	56322578	56422578	0.347709	LOC102179628
4	56322860	56422860	0.347709	KBTBD2
4	56322860	56422860	0.347709	FKBP9
4	56322860	56422860	0.347709	LOC102179628
4	56323821	56423821	0.344818	KBTBD2
4	56323821	56423821	0.344818	FKBP9
4	56323821	56423821	0.344818	LOC102179628
4	56345309	56445309	0.36924	FKBP9
4	56345309	56445309	0.36924	NT5C3A
4	56345309	56445309	0.36924	LOC102179628
4	56345433	56445433	0.350159	FKBP9
4	56345433	56445433	0.350159	NT5C3A
4	56345433	56445433	0.350159	LOC102179628
4	56345750	56445750	0.349301	FKBP9
4	56345750	56445750	0.349301	NT5C3A
4	56345750	56445750	0.349301	LOC102179628
4	56345906	56445906	0.349203	FKBP9
4	56345906	56445906	0.349203	NT5C3A
4	56345906	56445906	0.349203	LOC102179628
4	56345988	56445988	0.349203	FKBP9
4	56345988	56445988	0.349203	NT5C3A
4	56345988	56445988	0.349203	LOC102179628
4	56346212	56446212	0.349203	FKBP9
4	56346212	56446212	0.349203	NT5C3A
4	56346212	56446212	0.349203	LOC102179628
4	56346381	56446381	0.359461	FKBP9
4	56346381	56446381	0.359461	NT5C3A
4	56346381	56446381	0.359461	LOC102179628
4	56346443	56446443	0.359549	FKBP9
4	56346443	56446443	0.359549	NT5C3A
4	56346443	56446443	0.359549	LOC102179628
4	56346635	56446635	0.346849	FKBP9
4	56346635	56446635	0.346849	NT5C3A
4	56346635	56446635	0.346849	LOC102179628
4	56347481	56447481	0.341829	FKBP9
4	56347481	56447481	0.341829	NT5C3A
4	56347481	56447481	0.341829	LOC102179628
4	56797304	56897304	0.349409	BBS9
4	56797432	56897432	0.435783	BBS9
4	56797433	56897433	0.435218	BBS9
4	56797452	56897452	0.438225	BBS9
4	56797601	56897601	0.403096	BBS9
4	59651069	59751069	0.345969	AOAH
4	59655948	59755948	0.353033	AOAH
4	59655948	59755948	0.353033	ELMO1
4	59656216	59756216	0.342084	AOAH
4	59656216	59756216	0.342084	ELMO1
4	59656239	59756239	0.344774	AOAH
4	59656239	59756239	0.344774	ELMO1
4	59656820	59756820	0.346098	ELMO1
4	59656895	59756895	0.419705	ELMO1
4	59670989	59770989	0.348589	ELMO1
4	59676607	59776607	0.34354	ELMO1
4	59676630	59776630	0.356008	ELMO1

4	59677769	59777769	0.352673	ELMO1
4	59677876	59777876	0.353492	ELMO1
4	59677942	59777942	0.35241	ELMO1
4	59677993	59777993	0.352737	ELMO1
4	59678074	59778074	0.357037	ELMO1
4	59678362	59778362	0.350894	ELMO1
4	59678476	59778476	0.35261	ELMO1
4	59678727	59778727	0.358176	ELMO1
4	59678748	59778748	0.375323	ELMO1
4	59678895	59778895	0.362278	ELMO1
4	59679709	59779709	0.36334	ELMO1
4	59679789	59779789	0.37069	ELMO1
4	59679824	59779824	0.358864	ELMO1
4	59679838	59779838	0.354666	ELMO1
4	59679856	59779856	0.376412	ELMO1
4	59679934	59779934	0.372	ELMO1
4	59680215	59780215	0.363746	ELMO1
4	59680216	59780216	0.359657	ELMO1
4	59680844	59780844	0.363016	ELMO1
4	59680871	59780871	0.362172	ELMO1
4	59680944	59780944	0.371514	ELMO1
4	59680952	59780952	0.370532	ELMO1
4	59680965	59780965	0.372324	ELMO1
4	59681214	59781214	0.374137	ELMO1
4	59681528	59781528	0.368161	ELMO1
4	59681678	59781678	0.365564	ELMO1
4	59681691	59781691	0.367866	ELMO1
4	59682145	59782145	0.35849	ELMO1
4	59682298	59782298	0.365787	ELMO1
4	59682453	59782453	0.357536	ELMO1
4	59682525	59782525	0.351476	ELMO1
4	59682644	59782644	0.35622	ELMO1
4	59682747	59782747	0.35622	ELMO1
4	59683130	59783130	0.363277	ELMO1
4	59683395	59783395	0.368047	ELMO1
4	59683505	59783505	0.365999	ELMO1
4	59683521	59783521	0.351983	ELMO1
4	59683560	59783560	0.351983	ELMO1
4	59683906	59783906	0.354286	ELMO1
4	59684047	59784047	0.357496	ELMO1
4	59684196	59784196	0.359271	ELMO1
4	59684365	59784365	0.363992	ELMO1
4	59684465	59784465	0.360283	ELMO1
4	59684574	59784574	0.375044	ELMO1
4	59684719	59784719	0.370275	ELMO1
4	59684905	59784905	0.36265	ELMO1
4	59684926	59784926	0.36547	ELMO1
4	59685151	59785151	0.374388	ELMO1
4	59685344	59785344	0.361867	ELMO1
4	59685430	59785430	0.372235	ELMO1
4	59685446	59785446	0.3713	ELMO1
4	59685447	59785447	0.3713	ELMO1
4	59685470	59785470	0.381119	ELMO1
4	59685625	59785625	0.376551	ELMO1
4	59685656	59785656	0.378641	ELMO1
4	59685900	59785900	0.380604	ELMO1
4	59686192	59786192	0.376314	ELMO1
4	59686300	59786300	0.371141	ELMO1

4	59686347	59786347	0.371816	ELMO1
4	59686353	59786353	0.37623	ELMO1
4	59686413	59786413	0.374419	ELMO1
4	59686513	59786513	0.374223	ELMO1
4	59686570	59786570	0.374343	ELMO1
4	59686652	59786652	0.374022	ELMO1
4	59686946	59786946	0.359814	ELMO1
4	59687083	59787083	0.359814	ELMO1
4	59687204	59787204	0.364327	ELMO1
4	59687256	59787256	0.361253	ELMO1
4	59687367	59787367	0.360642	ELMO1
4	59687436	59787436	0.360263	ELMO1
4	59687551	59787551	0.360284	ELMO1
4	59687695	59787695	0.360284	ELMO1
4	59687743	59787743	0.360284	ELMO1
4	59687804	59787804	0.370622	ELMO1
4	59688163	59788163	0.368885	ELMO1
4	59688387	59788387	0.364073	ELMO1
4	59689089	59789089	0.360698	ELMO1
4	59689180	59789180	0.384114	ELMO1
4	59689198	59789198	0.387858	ELMO1
4	59689227	59789227	0.385453	ELMO1
4	59689737	59789737	0.383993	ELMO1
4	59689767	59789767	0.386321	ELMO1
4	59690096	59790096	0.35367	ELMO1
4	59690438	59790438	0.375064	ELMO1
4	59690513	59790513	0.38101	ELMO1
4	59690738	59790738	0.376928	ELMO1
4	59690810	59790810	0.362844	ELMO1
4	59691026	59791026	0.358913	ELMO1
4	59710627	59810627	0.343075	ELMO1
4	59711114	59811114	0.411808	ELMO1
4	59711276	59811276	0.397809	ELMO1
4	59711345	59811345	0.409065	ELMO1
4	59711802	59811802	0.380292	ELMO1
4	59711905	59811905	0.394364	ELMO1
4	59711996	59811996	0.397083	ELMO1
4	59712038	59812038	0.398106	ELMO1
4	59712199	59812199	0.39438	ELMO1
4	59712214	59812214	0.396017	ELMO1
4	59712776	59812776	0.388235	ELMO1
4	59712810	59812810	0.370917	ELMO1
4	59713004	59813004	0.403977	ELMO1
4	59713100	59813100	0.371126	ELMO1
4	59713447	59813447	0.369052	ELMO1
4	59713900	59813900	0.390701	ELMO1
4	59714313	59814313	0.385124	ELMO1
4	59714541	59814541	0.380004	ELMO1
4	59714582	59814582	0.385393	ELMO1
4	59714795	59814795	0.406818	ELMO1
4	59714848	59814848	0.406818	ELMO1
4	59715291	59815291	0.396922	ELMO1
4	59715416	59815416	0.400647	ELMO1
4	59715427	59815427	0.387636	ELMO1
4	59715709	59815709	0.387636	ELMO1
4	59715752	59815752	0.396999	ELMO1
4	59715865	59815865	0.382256	ELMO1
4	59716334	59816334	0.393362	ELMO1

4	59716491	59816491	0.393362	ELMO1
4	59716740	59816740	0.397609	ELMO1
4	59716798	59816798	0.397609	ELMO1
4	59716869	59816869	0.397984	ELMO1
4	59716911	59816911	0.399104	ELMO1
4	59716912	59816912	0.399104	ELMO1
4	59716957	59816957	0.399104	ELMO1
4	59716968	59816968	0.409827	ELMO1
4	59717032	59817032	0.408874	ELMO1
4	59717177	59817177	0.408039	ELMO1
4	59717203	59817203	0.411927	ELMO1
4	59717204	59817204	0.411927	ELMO1
4	59717223	59817223	0.402031	ELMO1
4	59717253	59817253	0.402031	ELMO1
4	59717268	59817268	0.402031	ELMO1
4	59717284	59817284	0.402031	ELMO1
4	59717298	59817298	0.402031	ELMO1
4	59717318	59817318	0.402031	ELMO1
4	59717341	59817341	0.402031	ELMO1
4	59717517	59817517	0.401547	ELMO1
4	59717599	59817599	0.405957	ELMO1
4	59717741	59817741	0.391403	ELMO1
4	59718097	59818097	0.38753	ELMO1
4	59718247	59818247	0.401305	ELMO1
4	59718389	59818389	0.404277	ELMO1
4	59718408	59818408	0.399908	ELMO1
4	59718482	59818482	0.413911	ELMO1
4	59718559	59818559	0.438591	ELMO1
4	59718560	59818560	0.438591	ELMO1
4	59718572	59818572	0.437238	ELMO1
4	59718585	59818585	0.425785	ELMO1
4	59718604	59818604	0.424884	ELMO1
4	59718635	59818635	0.42305	ELMO1
4	59718744	59818744	0.424562	ELMO1
4	59718748	59818748	0.424562	ELMO1
4	59719143	59819143	0.4242	ELMO1
4	59719202	59819202	0.417214	ELMO1
4	59719280	59819280	0.417214	ELMO1
4	59719419	59819419	0.415294	ELMO1
4	59719425	59819425	0.425849	ELMO1
4	59719634	59819634	0.419841	ELMO1
4	59719760	59819760	0.417212	ELMO1
4	59719774	59819774	0.417212	ELMO1
4	59719786	59819786	0.417212	ELMO1
4	59719804	59819804	0.431915	ELMO1
4	59719932	59819932	0.431915	ELMO1
4	59720150	59820150	0.430565	ELMO1
4	59720276	59820276	0.418251	ELMO1
4	59720282	59820282	0.425998	ELMO1
4	59720589	59820589	0.412775	ELMO1
4	59720652	59820652	0.422584	ELMO1
4	59720833	59820833	0.420383	ELMO1
4	59720945	59820945	0.422795	ELMO1
4	59721021	59821021	0.43441	ELMO1
4	59721046	59821046	0.433754	ELMO1
4	59721054	59821054	0.435987	ELMO1
4	59721058	59821058	0.436334	ELMO1
4	59721106	59821106	0.433788	ELMO1

4	59721118	59821118	0.434534	ELMO1
4	59721129	59821129	0.428299	ELMO1
4	59721293	59821293	0.427334	ELMO1
4	59721325	59821325	0.427334	ELMO1
4	59721331	59821331	0.434217	ELMO1
4	59721339	59821339	0.432137	ELMO1
4	59721385	59821385	0.437674	ELMO1
4	59721390	59821390	0.437674	ELMO1
4	59721416	59821416	0.438599	ELMO1
4	59721643	59821643	0.43599	ELMO1
4	59721659	59821659	0.434643	ELMO1
4	59721760	59821760	0.448491	ELMO1
4	59721812	59821812	0.440701	ELMO1
4	59721819	59821819	0.444262	ELMO1
4	59721831	59821831	0.444262	ELMO1
4	59721880	59821880	0.444262	ELMO1
4	59721908	59821908	0.455659	ELMO1
4	59721919	59821919	0.437068	ELMO1
4	59722167	59822167	0.408232	ELMO1
4	59722279	59822279	0.41384	ELMO1
4	59722342	59822342	0.414817	ELMO1
4	59722351	59822351	0.414036	ELMO1
4	59722369	59822369	0.414036	ELMO1
4	59722373	59822373	0.416536	ELMO1
4	59722420	59822420	0.412532	ELMO1
4	59722481	59822481	0.422416	ELMO1
4	59722575	59822575	0.418871	ELMO1
4	59722614	59822614	0.408559	ELMO1
4	59722721	59822721	0.408559	ELMO1
4	59722802	59822802	0.408559	ELMO1
4	59722854	59822854	0.386629	ELMO1
4	59722895	59822895	0.388423	ELMO1
4	59722973	59822973	0.399913	ELMO1
4	59723010	59823010	0.405479	ELMO1
4	59723105	59823105	0.398636	ELMO1
4	59723269	59823269	0.38511	ELMO1
4	59723364	59823364	0.387124	ELMO1
4	59723448	59823448	0.387886	ELMO1
4	59723486	59823486	0.397703	ELMO1
4	59723730	59823730	0.385986	ELMO1
4	59723772	59823772	0.385723	ELMO1
4	59723850	59823850	0.394855	ELMO1
4	59723947	59823947	0.395032	ELMO1
4	59724011	59824011	0.390204	ELMO1
4	59724097	59824097	0.368261	ELMO1
4	59724176	59824176	0.346798	ELMO1
4	59724233	59824233	0.347567	ELMO1
4	59724265	59824265	0.343105	ELMO1
4	59724376	59824376	0.343105	ELMO1
4	59724396	59824396	0.347681	ELMO1
4	59724450	59824450	0.346415	ELMO1
4	59725578	59825578	0.34526	ELMO1
4	59725618	59825618	0.353963	ELMO1
4	59725674	59825674	0.355793	ELMO1
4	59725741	59825741	0.343508	ELMO1
4	59725786	59825786	0.350027	ELMO1
4	59726345	59826345	0.347161	ELMO1
4	59726373	59826373	0.356734	ELMO1

4	59726604	59826604	0.355315	ELMO1
4	59726671	59826671	0.358177	ELMO1
4	59726849	59826849	0.350948	ELMO1
4	59727374	59827374	0.363504	ELMO1
4	59727529	59827529	0.368489	ELMO1
4	59727597	59827597	0.366561	ELMO1
4	59727665	59827665	0.358319	ELMO1
4	59727872	59827872	0.358319	ELMO1
4	59728031	59828031	0.358319	ELMO1
4	59728082	59828082	0.358319	ELMO1
4	59728119	59828119	0.358319	ELMO1
4	59728517	59828517	0.410101	ELMO1
4	59728655	59828655	0.409989	ELMO1
4	59728718	59828718	0.442707	ELMO1
4	65134912	65234912	0.363837	TRNAG-CCC-25
4	65134917	65234917	0.378445	TRNAG-CCC-25
4	69604522	69704522	0.346594	CTTNBP2
4	69604530	69704530	0.347628	CTTNBP2
4	69604963	69704963	0.363615	CTTNBP2
4	69605069	69705069	0.376252	CTTNBP2
4	69605074	69705074	0.379367	CTTNBP2
4	69605091	69705091	0.382369	CTTNBP2
4	69605107	69705107	0.375906	CTTNBP2
4	69605120	69705120	0.366588	CTTNBP2
4	69605287	69705287	0.349556	CTTNBP2
4	69605437	69705437	0.347525	CTTNBP2
4	69606031	69706031	0.351349	CTTNBP2
4	69606076	69706076	0.361748	CTTNBP2
4	75739820	75839820	0.364761	PMPCB
4	75739820	75839820	0.364761	NAPEPLD
4	75739820	75839820	0.364761	DNAJC2
4	75739835	75839835	0.360639	PMPCB
4	75739835	75839835	0.360639	NAPEPLD
4	75739835	75839835	0.360639	DNAJC2
4	75739976	75839976	0.354095	PMPCB
4	75739976	75839976	0.354095	NAPEPLD
4	75739976	75839976	0.354095	DNAJC2
4	75740141	75840141	0.354673	PMPCB
4	75740141	75840141	0.354673	NAPEPLD
4	75740141	75840141	0.354673	DNAJC2
4	75740643	75840643	0.355326	PMPCB
4	75740643	75840643	0.355326	NAPEPLD
4	75740643	75840643	0.355326	DNAJC2
4	75740808	75840808	0.355163	PMPCB
4	75740808	75840808	0.355163	NAPEPLD
4	75740808	75840808	0.355163	DNAJC2
4	75740818	75840818	0.354934	PMPCB
4	75740818	75840818	0.354934	NAPEPLD
4	75740818	75840818	0.354934	DNAJC2
4	75740896	75840896	0.355599	PMPCB
4	75740896	75840896	0.355599	NAPEPLD
4	75740896	75840896	0.355599	DNAJC2
4	75740961	75840961	0.353832	PMPCB
4	75740961	75840961	0.353832	NAPEPLD
4	75740961	75840961	0.353832	DNAJC2
4	75741839	75841839	0.361525	PMPCB
4	75741839	75841839	0.361525	NAPEPLD
4	75741839	75841839	0.361525	DNAJC2

4	75741895	75841895	0.358759	PMPCB
4	75741895	75841895	0.358759	NAPEPLD
4	75741895	75841895	0.358759	DNAJC2
4	75742217	75842217	0.357386	PMPCB
4	75742217	75842217	0.357386	NAPEPLD
4	75742217	75842217	0.357386	DNAJC2
4	75742227	75842227	0.360646	PMPCB
4	75742227	75842227	0.360646	NAPEPLD
4	75742227	75842227	0.360646	DNAJC2
4	75742687	75842687	0.356652	PMPCB
4	75742687	75842687	0.356652	NAPEPLD
4	75742687	75842687	0.356652	DNAJC2
4	75742722	75842722	0.344389	PMPCB
4	75742722	75842722	0.344389	NAPEPLD
4	75742722	75842722	0.344389	DNAJC2
4	75743016	75843016	0.359324	PMPCB
4	75743016	75843016	0.359324	NAPEPLD
4	75743016	75843016	0.359324	DNAJC2
4	75743926	75843926	0.356461	PMPCB
4	75743926	75843926	0.356461	NAPEPLD
4	75743926	75843926	0.356461	DNAJC2
4	75744056	75844056	0.358103	PMPCB
4	75744056	75844056	0.358103	NAPEPLD
4	75744056	75844056	0.358103	DNAJC2
4	75744340	75844340	0.355151	PMPCB
4	75744340	75844340	0.355151	NAPEPLD
4	75744340	75844340	0.355151	DNAJC2
4	75754495	75854495	0.355369	PMPCB
4	75754495	75854495	0.355369	NAPEPLD
4	75754495	75854495	0.355369	DNAJC2
4	75754514	75854514	0.352258	PMPCB
4	75754514	75854514	0.352258	NAPEPLD
4	75754514	75854514	0.352258	DNAJC2
4	75754558	75854558	0.354708	PMPCB
4	75754558	75854558	0.354708	NAPEPLD
4	75754558	75854558	0.354708	DNAJC2
4	75754580	75854580	0.356988	PMPCB
4	75754580	75854580	0.356988	NAPEPLD
4	75754580	75854580	0.356988	DNAJC2
4	75754587	75854587	0.368575	PMPCB
4	75754587	75854587	0.368575	NAPEPLD
4	75754587	75854587	0.368575	DNAJC2
4	75754704	75854704	0.364026	PMPCB
4	75754704	75854704	0.364026	NAPEPLD
4	75754704	75854704	0.364026	DNAJC2
4	75754735	75854735	0.360584	PMPCB
4	75754735	75854735	0.360584	NAPEPLD
4	75754735	75854735	0.360584	DNAJC2
4	75755064	75855064	0.353614	PMPCB
4	75755064	75855064	0.353614	NAPEPLD
4	75755064	75855064	0.353614	DNAJC2
4	75755316	75855316	0.342329	PMPCB
4	75755316	75855316	0.342329	NAPEPLD
4	75755316	75855316	0.342329	DNAJC2
4	75755534	75855534	0.342282	PMPCB
4	75755534	75855534	0.342282	NAPEPLD
4	75755534	75855534	0.342282	DNAJC2
4	75755641	75855641	0.343456	PMPCB

4	75755641	75855641	0.343456	NAPEPLD
4	75755641	75855641	0.343456	DNAJC2
4	75755736	75855736	0.342325	PMPCB
4	75755736	75855736	0.342325	NAPEPLD
4	75755736	75855736	0.342325	DNAJC2
4	75756070	75856070	0.349258	PMPCB
4	75756070	75856070	0.349258	NAPEPLD
4	75756070	75856070	0.349258	DNAJC2
4	75756133	75856133	0.348416	PMPCB
4	75756133	75856133	0.348416	NAPEPLD
4	75756133	75856133	0.348416	DNAJC2
4	75756158	75856158	0.345931	PMPCB
4	75756158	75856158	0.345931	NAPEPLD
4	75756158	75856158	0.345931	DNAJC2
4	75834087	75934087	0.345853	NAPEPLD
4	75834087	75934087	0.345853	ARMC10
4	87854930	87954930	0.346045	LOC102171502
4	89328839	89428839	0.360525	RAPGEF5
4	89328844	89428844	0.363095	RAPGEF5
4	89328855	89428855	0.363478	RAPGEF5
4	89328856	89428856	0.363478	RAPGEF5
4	89328857	89428857	0.374291	RAPGEF5
4	89328866	89428866	0.357287	RAPGEF5
4	91552540	91652540	0.343888	MACC1
4	91553088	91653088	0.345564	MACC1
4	91553108	91653108	0.342413	MACC1
4	91554358	91654358	0.344533	MACC1
4	91554407	91654407	0.342706	MACC1
4	91554414	91654414	0.342706	MACC1
4	91555823	91655823	0.386003	MACC1
4	91555943	91655943	0.346734	MACC1
4	91556054	91656054	0.348475	MACC1
4	91556365	91656365	0.346492	MACC1
4	91556901	91656901	0.347442	MACC1
4	91557006	91657006	0.347442	MACC1
4	91557078	91657078	0.347442	MACC1
4	91557152	91657152	0.349177	MACC1
4	91557166	91657166	0.349177	MACC1
4	91557244	91657244	0.352438	MACC1
4	91557245	91657245	0.352438	MACC1
4	91557247	91657247	0.352438	MACC1
4	91557311	91657311	0.352433	MACC1
4	91557314	91657314	0.354683	MACC1
4	91557331	91657331	0.354683	MACC1
4	91557376	91657376	0.34621	MACC1
4	91557403	91657403	0.344842	MACC1
4	91557430	91657430	0.351212	MACC1
4	91557461	91657461	0.351212	MACC1
4	91557521	91657521	0.347	MACC1
4	91557569	91657569	0.347	MACC1
4	91557584	91657584	0.347	MACC1
4	91557969	91657969	0.346713	MACC1
4	91557996	91657996	0.346713	MACC1
4	91558079	91658079	0.353985	MACC1
4	99880569	99980569	0.34198	SCIN
4	99880657	99980657	0.373459	SCIN
4	99880659	99980659	0.348844	SCIN
4	101093723	101193723	0.37557	LOC108635920

4	101093919	101193919	0.352253	LOC108635920
4	105204865	105304865	0.34761	COL28A1
4	105204865	105304865	0.34761	LOC108635857
4	105204970	105304970	0.364953	COL28A1
4	105204970	105304970	0.364953	LOC108635857
4	105204999	105304999	0.371548	COL28A1
4	105204999	105304999	0.371548	LOC108635857
4	105205001	105305001	0.374201	COL28A1
4	105205001	105305001	0.374201	LOC108635857
4	105205013	105305013	0.37417	COL28A1
4	105205013	105305013	0.37417	LOC108635857
4	105205062	105305062	0.373876	COL28A1
4	105205062	105305062	0.373876	LOC108635857
4	105205131	105305131	0.359222	COL28A1
4	105205131	105305131	0.359222	LOC108635857
4	105205140	105305140	0.366772	COL28A1
4	105205140	105305140	0.366772	LOC108635857
4	105205163	105305163	0.347254	COL28A1
4	105205163	105305163	0.347254	LOC108635857
4	105205165	105305165	0.347254	COL28A1
4	105205165	105305165	0.347254	LOC108635857
4	105205891	105305891	0.35781	COL28A1
4	105205891	105305891	0.35781	LOC108635857
4	105205895	105305895	0.35781	COL28A1
4	105205895	105305895	0.35781	LOC108635857
4	105205936	105305936	0.357613	COL28A1
4	105205936	105305936	0.357613	LOC108635857
4	105205952	105305952	0.36109	COL28A1
4	105205952	105305952	0.36109	LOC108635857
4	105205953	105305953	0.362622	COL28A1
4	105205953	105305953	0.362622	LOC108635857
4	105205955	105305955	0.350225	COL28A1
4	105205955	105305955	0.350225	LOC108635857
4	105205966	105305966	0.350225	COL28A1
4	105205966	105305966	0.350225	LOC108635857
4	107501331	107601331	0.378313	DYNC1I1
4	107501375	107601375	0.369173	DYNC1I1
4	107501929	107601929	0.347223	DYNC1I1
4	107501954	107601954	0.347223	DYNC1I1
4	107502084	107602084	0.352418	DYNC1I1
4	107502116	107602116	0.347427	DYNC1I1
4	107502256	107602256	0.368412	DYNC1I1
4	107502274	107602274	0.366766	DYNC1I1
4	107502276	107602276	0.366766	DYNC1I1
4	107502378	107602378	0.36851	DYNC1I1
4	107502436	107602436	0.346944	DYNC1I1
4	107502682	107602682	0.342185	DYNC1I1
4	107502692	107602692	0.360404	DYNC1I1
4	107628761	107728761	0.368101	LOC108635922
4	107628761	107728761	0.368101	LOC102190292
4	107628910	107728910	0.360038	LOC108635922
4	107628910	107728910	0.360038	LOC102190292
4	107628949	107728949	0.410099	LOC108635922
4	107628949	107728949	0.410099	LOC102190292
4	107628958	107728958	0.403973	LOC108635922
4	107628958	107728958	0.403973	LOC102190292
4	107629007	107729007	0.405789	LOC108635922
4	107629007	107729007	0.405789	LOC102190292

4	107629046	107729046	0.429484	LOC108635922
4	107629046	107729046	0.429484	LOC102190292
4	107629126	107729126	0.431414	LOC108635922
4	107629126	107729126	0.431414	LOC102190292
4	107629172	107729172	0.41402	LOC108635922
4	107629172	107729172	0.41402	LOC102190292
4	107629252	107729252	0.377406	LOC108635922
4	107629252	107729252	0.377406	LOC102190292
4	107629301	107729301	0.381797	LOC108635922
4	107629301	107729301	0.381797	LOC102190292
4	107629360	107729360	0.378348	LOC108635922
4	107629360	107729360	0.378348	LOC102190292
4	107629497	107729497	0.349231	LOC108635922
4	107629497	107729497	0.349231	LOC102190292
4	107629575	107729575	0.360176	LOC108635922
4	107629575	107729575	0.360176	LOC102190292
4	107629602	107729602	0.367841	LOC108635922
4	107629602	107729602	0.367841	LOC102190292
4	107629738	107729738	0.374373	LOC108635922
4	107629738	107729738	0.374373	LOC102190292
4	107629786	107729786	0.359325	LOC108635922
4	107629786	107729786	0.359325	LOC102190292
4	107629865	107729865	0.359325	LOC108635922
4	107629865	107729865	0.359325	LOC102190292
4	107629912	107729912	0.364209	LOC108635922
4	107629912	107729912	0.364209	LOC102190292
4	107633671	107733671	0.350656	LOC102190292
4	107633751	107733751	0.353271	LOC102190292
4	107633779	107733779	0.353271	LOC102190292
4	107634253	107734253	0.351266	LOC102190292
4	107634254	107734254	0.351266	LOC102190292
4	107634271	107734271	0.355023	LOC102190292
4	107634272	107734272	0.343053	LOC102190292
4	107634298	107734298	0.343053	LOC102190292
4	107634310	107734310	0.343053	LOC102190292
4	107634323	107734323	0.343053	LOC102190292
4	107634411	107734411	0.360978	LOC102190292
4	107634423	107734423	0.348186	LOC102190292
4	107634428	107734428	0.348305	LOC102190292
4	107634440	107734440	0.34714	LOC102190292
4	107634465	107734465	0.348804	LOC102190292
4	107634483	107734483	0.35011	LOC102190292
4	107634523	107734523	0.35435	LOC102190292
4	107634552	107734552	0.347012	LOC102190292
4	107634573	107734573	0.365994	LOC102190292
4	107634679	107734679	0.366089	LOC102190292
4	107634740	107734740	0.359935	LOC102190292
4	107634747	107734747	0.36151	LOC102190292
4	107634930	107734930	0.390489	LOC102190292
4	107634971	107734971	0.351213	LOC102190292
4	107635060	107735060	0.351213	LOC102190292
4	107635071	107735071	0.352504	LOC102190292
4	107635145	107735145	0.352648	LOC102190292
4	107635244	107735244	0.354605	LOC102190292
4	107635251	107735251	0.354605	LOC102190292
4	107635275	107735275	0.342959	LOC102190292
4	107635579	107735579	0.389562	LOC102190292
4	107635597	107735597	0.378635	LOC102190292

4	107635602	107735602	0.396707	LOC102190292
4	107635623	107735623	0.395634	LOC102190292
4	107635624	107735624	0.401831	LOC102190292
4	107635643	107735643	0.399828	LOC102190292
4	115299348	115399348	0.377615	GRB10
4	115299604	115399604	0.377272	GRB10
4	115299786	115399786	0.39034	GRB10
4	115300051	115400051	0.460173	GRB10
4	115300052	115400052	0.417934	GRB10
4	115300312	115400312	0.410905	GRB10
4	115300961	115400961	0.37079	GRB10
4	115301038	115401038	0.371218	GRB10
4	115301174	115401174	0.413919	GRB10
4	115301215	115401215	0.418014	GRB10
4	115301231	115401231	0.378261	GRB10
4	115301279	115401279	0.356676	GRB10
4	115301323	115401323	0.348692	GRB10
4	115301472	115401472	0.348692	GRB10
4	115301559	115401559	0.374065	GRB10
4	115302034	115402034	0.358726	GRB10
4	115302041	115402041	0.399395	GRB10
4	115302057	115402057	0.424516	GRB10
4	115302061	115402061	0.398906	GRB10
4	115302128	115402128	0.351516	GRB10
4	115302977	115402977	0.352419	GRB10
4	115304735	115404735	0.351616	GRB10
4	115307402	115407402	0.349212	GRB10
4	115307706	115407706	0.384078	GRB10
4	115307732	115407732	0.354787	GRB10
4	115351997	115451997	0.343792	GRB10
4	115352184	115452184	0.382417	GRB10
4	115352271	115452271	0.379759	GRB10
4	115359052	115459052	0.342505	GRB10
4	115359078	115459078	0.348318	GRB10
4	115359247	115459247	0.348318	GRB10
4	115359280	115459280	0.351106	GRB10
4	115359633	115459633	0.357832	GRB10
4	115359722	115459722	0.347104	GRB10
4	115359723	115459723	0.357695	GRB10
5	597755	697755	0.383522	TSPAN8
5	597772	697772	0.389646	TSPAN8
5	597798	697798	0.38881	TSPAN8
5	597804	697804	0.375562	TSPAN8
5	597826	697826	0.365336	TSPAN8
5	597890	697890	0.369187	TSPAN8
5	908594	1008594	0.366876	LGR5
5	912907	1012907	0.405067	LGR5
5	913475	1013475	0.427513	LGR5
5	913786	1013786	0.376915	LGR5
5	913810	1013810	0.384077	LGR5
5	913813	1013813	0.379098	LGR5
5	914363	1014363	0.346654	LGR5
5	914550	1014550	0.346654	LGR5
5	914580	1014580	0.392987	LGR5
5	914582	1014582	0.395174	LGR5
5	914620	1014620	0.391142	LGR5
5	914673	1014673	0.389546	LGR5
5	914683	1014683	0.39965	LGR5

5	915006	1015006	0.377107	LGR5
5	915242	1015242	0.377107	LGR5
5	915540	1015540	0.377107	LGR5
5	915589	1015589	0.377107	LGR5
5	916542	1016542	0.343032	LGR5
5	917114	1017114	0.352881	LGR5
5	917228	1017228	0.362473	LGR5
5	917413	1017413	0.348834	LGR5
5	918063	1018063	0.347828	LGR5
5	2044681	2144681	0.37782	TRHDE
5	2044683	2144683	0.381953	TRHDE
5	2044702	2144702	0.377607	TRHDE
5	2044786	2144786	0.363546	TRHDE
5	2044818	2144818	0.368076	TRHDE
5	2044835	2144835	0.366913	TRHDE
5	2044845	2144845	0.365299	TRHDE
5	2044850	2144850	0.365494	TRHDE
5	2044869	2144869	0.366302	TRHDE
5	2044873	2144873	0.371085	TRHDE
5	2045197	2145197	0.342445	TRHDE
5	2045206	2145206	0.34325	TRHDE
5	2045282	2145282	0.347305	TRHDE
5	2046004	2146004	0.342312	TRHDE
5	2046627	2146627	0.353746	TRHDE
5	2046714	2146714	0.354159	TRHDE
5	2046859	2146859	0.364744	TRHDE
5	2046921	2146921	0.404849	TRHDE
5	2046934	2146934	0.388385	TRHDE
5	2046960	2146960	0.390547	TRHDE
5	2046987	2146987	0.391914	TRHDE
5	2047232	2147232	0.372933	TRHDE
5	2047348	2147348	0.391098	TRHDE
5	2047371	2147371	0.386743	TRHDE
5	2047449	2147449	0.400496	TRHDE
5	2047549	2147549	0.424421	TRHDE
5	2047612	2147612	0.416795	TRHDE
5	2047623	2147623	0.397019	TRHDE
5	2047656	2147656	0.400452	TRHDE
5	2047715	2147715	0.398963	TRHDE
5	2047859	2147859	0.388539	TRHDE
5	2047911	2147911	0.389524	TRHDE
5	2047932	2147932	0.389524	TRHDE
5	2047980	2147980	0.423842	TRHDE
5	2048036	2148036	0.400692	TRHDE
5	2048044	2148044	0.398671	TRHDE
5	2048068	2148068	0.397545	TRHDE
5	2048128	2148128	0.386297	TRHDE
5	2048162	2148162	0.390738	TRHDE
5	2048195	2148195	0.39073	TRHDE
5	2048208	2148208	0.394578	TRHDE
5	2048239	2148239	0.404454	TRHDE
5	2048332	2148332	0.40678	TRHDE
5	2048333	2148333	0.416872	TRHDE
5	2048366	2148366	0.409971	TRHDE
5	2048367	2148367	0.393854	TRHDE
5	2048495	2148495	0.396631	TRHDE
5	2048619	2148619	0.404689	TRHDE
5	2048634	2148634	0.355334	TRHDE

5	2048759	2148759	0.354725	TRHDE
5	2048830	2148830	0.368532	TRHDE
5	2048873	2148873	0.35437	TRHDE
5	2048944	2148944	0.359983	TRHDE
5	2048952	2148952	0.360069	TRHDE
5	2049067	2149067	0.351656	TRHDE
5	2049157	2149157	0.344409	TRHDE
5	2049203	2149203	0.369242	TRHDE
5	2049278	2149278	0.34976	TRHDE
5	2049364	2149364	0.34976	TRHDE
5	2049430	2149430	0.3666	TRHDE
5	2049443	2149443	0.365938	TRHDE
5	2049454	2149454	0.366822	TRHDE
5	2049654	2149654	0.36493	TRHDE
5	2049720	2149720	0.364829	TRHDE
5	2049721	2149721	0.368324	TRHDE
5	2049769	2149769	0.359787	TRHDE
5	2049788	2149788	0.362791	TRHDE
5	2049907	2149907	0.35434	TRHDE
5	2050237	2150237	0.349681	TRHDE
5	2050316	2150316	0.345083	TRHDE
5	2050368	2150368	0.366575	TRHDE
5	2050406	2150406	0.36534	TRHDE
5	2050478	2150478	0.357925	TRHDE
5	2050641	2150641	0.343207	TRHDE
5	2051252	2151252	0.344011	TRHDE
5	2051293	2151293	0.365963	TRHDE
5	2052025	2152025	0.359196	TRHDE
5	2052051	2152051	0.362873	TRHDE
5	2052073	2152073	0.361976	TRHDE
5	2052123	2152123	0.354698	TRHDE
5	2052219	2152219	0.358186	TRHDE
5	2052235	2152235	0.357642	TRHDE
5	2052277	2152277	0.35522	TRHDE
5	2052291	2152291	0.3617	TRHDE
5	2052303	2152303	0.358401	TRHDE
5	2052319	2152319	0.359333	TRHDE
5	2052355	2152355	0.351973	TRHDE
5	2052427	2152427	0.357551	TRHDE
5	2052483	2152483	0.364426	TRHDE
5	2052521	2152521	0.366012	TRHDE
5	2052524	2152524	0.348868	TRHDE
5	2052794	2152794	0.354306	TRHDE
5	2052816	2152816	0.377629	TRHDE
5	2052833	2152833	0.39271	TRHDE
5	2052868	2152868	0.350373	TRHDE
5	2052915	2152915	0.368173	TRHDE
5	2053102	2153102	0.384149	TRHDE
5	2053193	2153193	0.388619	TRHDE
5	2053289	2153289	0.386494	TRHDE
5	2053390	2153390	0.391207	TRHDE
5	2053411	2153411	0.366631	TRHDE
5	2053447	2153447	0.362996	TRHDE
5	2053449	2153449	0.350945	TRHDE
5	2053710	2153710	0.350945	TRHDE
5	2053733	2153733	0.344949	TRHDE
5	2054037	2154037	0.358824	TRHDE
5	2054236	2154236	0.347514	TRHDE

5	2054435	2154435	0.345056	TRHDE
5	2054565	2154565	0.345056	TRHDE
5	2054731	2154731	0.353093	TRHDE
5	2054909	2154909	0.359352	TRHDE
5	2054913	2154913	0.354358	TRHDE
5	2055030	2155030	0.352569	TRHDE
5	2055091	2155091	0.346528	TRHDE
5	2055125	2155125	0.356332	TRHDE
5	2055133	2155133	0.34806	TRHDE
5	2055187	2155187	0.34806	TRHDE
5	2055296	2155296	0.346758	TRHDE
5	2055339	2155339	0.346758	TRHDE
5	2055405	2155405	0.345921	TRHDE
5	2055601	2155601	0.347583	TRHDE
5	2055632	2155632	0.356093	TRHDE
5	2055643	2155643	0.353276	TRHDE
5	2055711	2155711	0.342803	TRHDE
5	2055760	2155760	0.343764	TRHDE
5	2055907	2155907	0.347864	TRHDE
5	2056013	2156013	0.347864	TRHDE
5	2056094	2156094	0.361165	TRHDE
5	2056100	2156100	0.362422	TRHDE
5	2056162	2156162	0.36205	TRHDE
5	2056163	2156163	0.362519	TRHDE
5	2056195	2156195	0.362519	TRHDE
5	2056236	2156236	0.359781	TRHDE
5	2056340	2156340	0.360669	TRHDE
5	2056380	2156380	0.362567	TRHDE
5	2056447	2156447	0.361878	TRHDE
5	2056451	2156451	0.359766	TRHDE
5	2056466	2156466	0.356496	TRHDE
5	2056590	2156590	0.359044	TRHDE
5	2056655	2156655	0.361851	TRHDE
5	2056819	2156819	0.350295	TRHDE
5	2056917	2156917	0.363776	TRHDE
5	2056956	2156956	0.36825	TRHDE
5	2056978	2156978	0.355879	TRHDE
5	2057044	2157044	0.415074	TRHDE
5	2057059	2157059	0.417854	TRHDE
5	2057089	2157089	0.394144	TRHDE
5	2057098	2157098	0.391155	TRHDE
5	2057100	2157100	0.391155	TRHDE
5	3858208	3958208	0.392007	ATXN7L3B
5	3858211	3958211	0.3723	ATXN7L3B
5	3858229	3958229	0.367175	ATXN7L3B
5	3858338	3958338	0.394748	ATXN7L3B
5	3858348	3958348	0.396483	ATXN7L3B
5	3858417	3958417	0.387349	ATXN7L3B
5	3858426	3958426	0.392693	ATXN7L3B
5	3858427	3958427	0.381093	ATXN7L3B
5	3858486	3958486	0.408838	ATXN7L3B
5	3858488	3958488	0.41413	ATXN7L3B
5	3858512	3958512	0.414146	ATXN7L3B
5	3858520	3958520	0.389026	ATXN7L3B
5	3858548	3958548	0.396266	ATXN7L3B
5	3858553	3958553	0.367819	ATXN7L3B
5	3858569	3958569	0.343093	ATXN7L3B
5	3858587	3958587	0.345316	ATXN7L3B

5	3858600	3958600	0.345316	ATXN7L3B
5	3858790	3958790	0.365803	ATXN7L3B
5	3858860	3958860	0.396901	ATXN7L3B
5	3858948	3958948	0.352987	ATXN7L3B
5	5133565	5233565	0.354928	LOC108636124
5	5133617	5233617	0.355225	LOC108636124
5	5133842	5233842	0.348248	LOC108636124
5	5133882	5233882	0.357823	LOC108636124
5	5133981	5233981	0.360888	LOC108636124
5	5134059	5234059	0.363695	LOC108636124
5	5134127	5234127	0.362927	LOC108636124
5	5134131	5234131	0.363001	LOC108636124
5	5134231	5234231	0.363499	LOC108636124
5	5134276	5234276	0.364826	LOC108636124
5	5134282	5234282	0.363104	LOC108636124
5	5134350	5234350	0.375728	LOC108636124
5	5134443	5234443	0.357385	LOC108636124
5	5134468	5234468	0.355092	LOC108636124
5	5134474	5234474	0.362554	LOC108636124
5	5134535	5234535	0.380144	LOC108636124
5	5134631	5234631	0.342326	LOC108636124
5	5134632	5234632	0.343249	LOC108636124
5	5136201	5236201	0.344069	LOC108636124
5	5136219	5236219	0.341944	LOC108636124
5	5136575	5236575	0.341861	LOC108636124
5	5137631	5237631	0.358681	LOC108636124
5	5137683	5237683	0.370699	LOC108636124
5	5138732	5238732	0.343413	LOC108636124
5	5140826	5240826	0.346351	LOC108636124
5	5140980	5240980	0.366359	LOC108636124
5	5141017	5241017	0.366359	LOC108636124
5	5141027	5241027	0.354116	LOC108636124
5	5141142	5241142	0.348645	LOC108636124
5	5141145	5241145	0.348645	LOC108636124
5	5141290	5241290	0.345371	LOC108636124
5	5141636	5241636	0.346978	LOC108636124
5	5141653	5241653	0.347907	LOC108636124
5	5141666	5241666	0.343617	LOC108636124
5	5141756	5241756	0.341792	LOC108636124
5	5141876	5241876	0.353192	LOC108636124
5	5141882	5241882	0.348725	LOC108636124
5	5142087	5242087	0.355081	LOC108636124
5	5142098	5242098	0.354445	LOC108636124
5	5142100	5242100	0.356693	LOC108636124
5	5142167	5242167	0.356914	LOC108636124
5	5142289	5242289	0.358798	LOC108636124
5	5142441	5242441	0.352583	LOC108636124
5	5142522	5242522	0.353484	LOC108636124
5	5142584	5242584	0.356141	LOC108636124
5	5142639	5242639	0.35533	LOC108636124
5	5142688	5242688	0.354479	LOC108636124
5	5142705	5242705	0.363314	LOC108636124
5	5142719	5242719	0.35586	LOC108636124
5	5142734	5242734	0.363994	LOC108636124
5	5142761	5242761	0.369076	LOC108636124
5	5142786	5242786	0.369076	LOC108636124
5	5142787	5242787	0.370363	LOC108636124
5	5142804	5242804	0.368169	LOC108636124

5	5142810	5242810	0.368169	LOC108636124
5	5142812	5242812	0.368169	LOC108636124
5	5142814	5242814	0.370667	LOC108636124
5	5142824	5242824	0.370467	LOC108636124
5	5142826	5242826	0.370667	LOC108636124
5	5142829	5242829	0.370399	LOC108636124
5	5142863	5242863	0.365843	LOC108636124
5	5142865	5242865	0.365843	LOC108636124
5	5142874	5242874	0.365843	LOC108636124
5	5142883	5242883	0.361161	LOC108636124
5	5142910	5242910	0.363361	LOC108636124
5	5142933	5242933	0.35513	LOC108636124
5	5143135	5243135	0.350542	LOC108636124
5	5143140	5243140	0.350542	LOC108636124
5	5143196	5243196	0.348435	LOC108636124
5	5143353	5243353	0.351128	LOC108636124
5	5143358	5243358	0.353961	LOC108636124
5	5143501	5243501	0.342857	LOC108636124
5	5143552	5243552	0.345608	LOC108636124
5	5143576	5243576	0.344285	LOC108636124
5	5143632	5243632	0.345418	LOC108636124
5	5143642	5243642	0.346863	LOC108636124
5	5147139	5247139	0.346225	LOC108636124
5	5147142	5247142	0.348212	LOC108636124
5	5147153	5247153	0.348207	LOC108636124
5	5147154	5247154	0.347549	LOC108636124
5	5147625	5247625	0.344089	LOC108636124
5	5147629	5247629	0.34178	LOC108636124
5	6330246	6430246	0.350812	LOC102182100
5	6749140	6849140	0.354276	NAV3
5	6749173	6849173	0.351669	NAV3
5	6749178	6849178	0.351669	NAV3
5	6749184	6849184	0.351669	NAV3
5	6749218	6849218	0.351669	NAV3
5	6749350	6849350	0.349647	NAV3
5	6749665	6849665	0.35145	NAV3
5	6749669	6849669	0.34359	NAV3
5	6749706	6849706	0.342877	NAV3
5	6749900	6849900	0.341703	NAV3
5	6749901	6849901	0.342841	NAV3
5	6749910	6849910	0.345356	NAV3
5	6750967	6850967	0.380762	NAV3
5	6751002	6851002	0.3804	NAV3
5	6751020	6851020	0.388767	NAV3
5	6751123	6851123	0.393088	NAV3
5	6751177	6851177	0.411417	NAV3
5	6751188	6851188	0.41112	NAV3
5	6751248	6851248	0.411901	NAV3
5	6751260	6851260	0.413907	NAV3
5	6751282	6851282	0.42433	NAV3
5	6751325	6851325	0.423821	NAV3
5	6751365	6851365	0.429948	NAV3
5	6751456	6851456	0.42672	NAV3
5	6751471	6851471	0.428062	NAV3
5	6751481	6851481	0.422444	NAV3
5	6751497	6851497	0.422472	NAV3
5	6751508	6851508	0.42862	NAV3
5	6751521	6851521	0.424847	NAV3

5	6751525	6851525	0.424842	NAV3
5	6751536	6851536	0.426763	NAV3
5	6751556	6851556	0.42465	NAV3
5	6751632	6851632	0.417069	NAV3
5	6751674	6851674	0.366894	NAV3
5	6751714	6851714	0.367651	NAV3
5	6751715	6851715	0.366494	NAV3
5	6751727	6851727	0.36365	NAV3
5	6751828	6851828	0.363384	NAV3
5	6751875	6851875	0.359683	NAV3
5	6751962	6851962	0.356157	NAV3
5	6751971	6851971	0.356398	NAV3
5	6751977	6851977	0.356398	NAV3
5	6752027	6852027	0.354899	NAV3
5	6752095	6852095	0.356343	NAV3
5	6752121	6852121	0.344384	NAV3
5	6752138	6852138	0.344384	NAV3
5	6752151	6852151	0.344384	NAV3
5	6752174	6852174	0.344384	NAV3
5	6752187	6852187	0.346536	NAV3
5	6752195	6852195	0.346373	NAV3
5	6752196	6852196	0.346373	NAV3
5	6752208	6852208	0.345762	NAV3
5	6752307	6852307	0.345581	NAV3
5	6752328	6852328	0.345581	NAV3
5	6752331	6852331	0.345581	NAV3
5	6752351	6852351	0.344987	NAV3
5	6752433	6852433	0.345025	NAV3
5	6752459	6852459	0.345025	NAV3
5	6752500	6852500	0.345025	NAV3
5	6753902	6853902	0.347221	NAV3
5	6755045	6855045	0.350235	NAV3
5	6755150	6855150	0.342513	NAV3
5	6755268	6855268	0.342554	NAV3
5	6755290	6855290	0.342491	NAV3
5	6755301	6855301	0.342491	NAV3
5	6755510	6855510	0.347667	NAV3
5	6755538	6855538	0.346091	NAV3
5	6755575	6855575	0.346091	NAV3
5	6755592	6855592	0.346998	NAV3
5	6755775	6855775	0.349387	NAV3
5	6756020	6856020	0.342473	NAV3
5	6756095	6856095	0.341797	NAV3
5	6757189	6857189	0.343809	NAV3
5	6757354	6857354	0.343799	NAV3
5	6757540	6857540	0.344255	NAV3
5	6757542	6857542	0.344913	NAV3
5	6758270	6858270	0.34407	NAV3
5	6758487	6858487	0.343758	NAV3
5	6758506	6858506	0.343574	NAV3
5	6759068	6859068	0.360976	NAV3
5	6761321	6861321	0.343401	NAV3
5	6761742	6861742	0.343954	NAV3
5	6761768	6861768	0.344908	NAV3
5	6761785	6861785	0.345006	NAV3
5	6761892	6861892	0.344307	NAV3
5	6761923	6861923	0.344307	NAV3
5	7098247	7198247	0.343077	NAV3

5	7230510	7330510	0.353701	NAV3
5	9518183	9618183	0.381052	OTOGL
5	9518234	9618234	0.350719	OTOGL
5	9545459	9645459	0.343512	OTOGL
5	9545973	9645973	0.361742	OTOGL
5	9546051	9646051	0.352925	OTOGL
5	9546052	9646052	0.352415	OTOGL
5	9546178	9646178	0.367066	OTOGL
5	9546553	9646553	0.355199	OTOGL
5	9547345	9647345	0.364947	OTOGL
5	9547428	9647428	0.36688	OTOGL
5	9547605	9647605	0.369836	OTOGL
5	9547744	9647744	0.373941	OTOGL
5	9547858	9647858	0.36597	OTOGL
5	9547921	9647921	0.398738	OTOGL
5	9547942	9647942	0.39074	OTOGL
5	9548065	9648065	0.389814	OTOGL
5	9548099	9648099	0.39808	OTOGL
5	9548125	9648125	0.389247	OTOGL
5	9548258	9648258	0.385774	OTOGL
5	9548353	9648353	0.385672	OTOGL
5	9548467	9648467	0.388233	OTOGL
5	9548569	9648569	0.409704	OTOGL
5	9548572	9648572	0.406185	OTOGL
5	9548625	9648625	0.384183	OTOGL
5	9548634	9648634	0.391497	OTOGL
5	9548679	9648679	0.384689	OTOGL
5	9548684	9648684	0.38247	OTOGL
5	9548826	9648826	0.382459	OTOGL
5	9548908	9648908	0.375262	OTOGL
5	9548956	9648956	0.380174	OTOGL
5	9549109	9649109	0.360745	OTOGL
5	9549392	9649392	0.363141	OTOGL
5	9549626	9649626	0.363277	OTOGL
5	9549815	9649815	0.373007	OTOGL
5	9549826	9649826	0.357653	OTOGL
5	9550049	9650049	0.357653	OTOGL
5	9550110	9650110	0.411959	OTOGL
5	9550121	9650121	0.397163	OTOGL
5	9550122	9650122	0.394149	OTOGL
5	9550224	9650224	0.367535	OTOGL
5	9550431	9650431	0.366141	OTOGL
5	9550460	9650460	0.390334	OTOGL
5	9550475	9650475	0.369643	OTOGL
5	9550644	9650644	0.346089	OTOGL
5	9550835	9650835	0.346089	OTOGL
5	9550921	9650921	0.350598	OTOGL
5	9551286	9651286	0.354213	OTOGL
5	9551891	9651891	0.358237	OTOGL
5	9552810	9652810	0.360514	OTOGL
5	9552812	9652812	0.368285	OTOGL
5	9552831	9652831	0.363299	OTOGL
5	9552971	9652971	0.371204	OTOGL
5	9553031	9653031	0.359806	OTOGL
5	9553181	9653181	0.368704	OTOGL
5	9553208	9653208	0.385489	OTOGL
5	9553357	9653357	0.387009	OTOGL
5	9553417	9653417	0.38572	OTOGL

5	9553437	9653437	0.391219	OTOGL
5	9553447	9653447	0.388488	OTOGL
5	9553501	9653501	0.385122	OTOGL
5	9553503	9653503	0.381999	OTOGL
5	9553513	9653513	0.381508	OTOGL
5	9553521	9653521	0.381659	OTOGL
5	9553559	9653559	0.37925	OTOGL
5	9553695	9653695	0.380658	OTOGL
5	9553772	9653772	0.372751	OTOGL
5	9553777	9653777	0.371991	OTOGL
5	9553872	9653872	0.370334	OTOGL
5	9553941	9653941	0.371701	OTOGL
5	9553968	9653968	0.357331	OTOGL
5	9553969	9653969	0.357331	OTOGL
5	9553970	9653970	0.357331	OTOGL
5	9553990	9653990	0.357331	OTOGL
5	9553994	9653994	0.357331	OTOGL
5	9554001	9654001	0.357331	OTOGL
5	9554028	9654028	0.357613	OTOGL
5	9554051	9654051	0.35752	OTOGL
5	9554052	9654052	0.35752	OTOGL
5	9554109	9654109	0.356565	OTOGL
5	9554110	9654110	0.356565	OTOGL
5	9554139	9654139	0.357281	OTOGL
5	9554140	9654140	0.357563	OTOGL
5	9554143	9654143	0.356367	OTOGL
5	9554177	9654177	0.356367	OTOGL
5	9554295	9654295	0.350642	OTOGL
5	9554387	9654387	0.356813	OTOGL
5	9554388	9654388	0.343654	OTOGL
5	9564868	9664868	0.342131	OTOGL
5	9564938	9664938	0.342102	OTOGL
5	9565460	9665460	0.3449	OTOGL
5	9680378	9780378	0.350956	LOC102187742
5	9680378	9780378	0.350956	OTOGL
5	14478022	14578022	0.377	TSPAN19
5	14478022	14578022	0.377	LRRIQ1
5	14478077	14578077	0.474988	TSPAN19
5	14478077	14578077	0.474988	LRRIQ1
5	14479792	14579792	0.383322	TSPAN19
5	14479792	14579792	0.383322	LRRIQ1
5	14480355	14580355	0.385031	TSPAN19
5	14480355	14580355	0.385031	LRRIQ1
5	14480468	14580468	0.381067	TSPAN19
5	14480468	14580468	0.381067	LRRIQ1
5	14480579	14580579	0.353502	TSPAN19
5	14480579	14580579	0.353502	LRRIQ1
5	14480612	14580612	0.354265	TSPAN19
5	14480612	14580612	0.354265	LRRIQ1
5	14480669	14580669	0.356596	TSPAN19
5	14480669	14580669	0.356596	LRRIQ1
5	14481077	14581077	0.389222	TSPAN19
5	14481077	14581077	0.389222	LRRIQ1
5	14481156	14581156	0.422333	TSPAN19
5	14481156	14581156	0.422333	LRRIQ1
5	14494202	14594202	0.348013	TSPAN19
5	14494202	14594202	0.348013	LRRIQ1
5	14496191	14596191	0.354601	TSPAN19

5	14496191	14596191	0.354601	LRRIQ1
5	14496381	14596381	0.422595	TSPAN19
5	14496381	14596381	0.422595	LRRIQ1
5	14496452	14596452	0.458332	TSPAN19
5	14496452	14596452	0.458332	LRRIQ1
5	14496584	14596584	0.384472	TSPAN19
5	14496584	14596584	0.384472	LRRIQ1
5	14496761	14596761	0.421992	TSPAN19
5	14496761	14596761	0.421992	LRRIQ1
5	14496764	14596764	0.428154	TSPAN19
5	14496764	14596764	0.428154	LRRIQ1
5	14496803	14596803	0.414089	TSPAN19
5	14496803	14596803	0.414089	LRRIQ1
5	14496859	14596859	0.413992	TSPAN19
5	14496859	14596859	0.413992	LRRIQ1
5	14496895	14596895	0.415194	TSPAN19
5	14496895	14596895	0.415194	LRRIQ1
5	14496909	14596909	0.415194	TSPAN19
5	14496909	14596909	0.415194	LRRIQ1
5	14496965	14596965	0.40882	TSPAN19
5	14496965	14596965	0.40882	LRRIQ1
5	14497087	14597087	0.409724	TSPAN19
5	14497087	14597087	0.409724	LRRIQ1
5	14497286	14597286	0.408399	TSPAN19
5	14497286	14597286	0.408399	LRRIQ1
5	14497871	14597871	0.405974	TSPAN19
5	14497871	14597871	0.405974	LRRIQ1
5	14497938	14597938	0.410111	TSPAN19
5	14497938	14597938	0.410111	LRRIQ1
5	14497980	14597980	0.413199	TSPAN19
5	14497980	14597980	0.413199	LRRIQ1
5	14498061	14598061	0.410219	TSPAN19
5	14498061	14598061	0.410219	LRRIQ1
5	14498346	14598346	0.407963	TSPAN19
5	14498346	14598346	0.407963	LRRIQ1
5	14498416	14598416	0.410491	TSPAN19
5	14498416	14598416	0.410491	LRRIQ1
5	14498551	14598551	0.404658	TSPAN19
5	14498551	14598551	0.404658	LRRIQ1
5	14499355	14599355	0.404026	TSPAN19
5	14499355	14599355	0.404026	LRRIQ1
5	14499393	14599393	0.404026	TSPAN19
5	14499393	14599393	0.404026	LRRIQ1
5	14499395	14599395	0.404026	TSPAN19
5	14499395	14599395	0.404026	LRRIQ1
5	14499646	14599646	0.404026	TSPAN19
5	14499646	14599646	0.404026	LRRIQ1
5	14499751	14599751	0.405312	TSPAN19
5	14499751	14599751	0.405312	LRRIQ1
5	14499784	14599784	0.405946	TSPAN19
5	14499784	14599784	0.405946	LRRIQ1
5	14500149	14600149	0.405946	TSPAN19
5	14500149	14600149	0.405946	LRRIQ1
5	14500180	14600180	0.413458	TSPAN19
5	14500180	14600180	0.413458	LRRIQ1
5	14500182	14600182	0.414653	TSPAN19
5	14500182	14600182	0.414653	LRRIQ1
5	14500196	14600196	0.398839	TSPAN19

5	14500196	14600196	0.398839	LRRIQ1
5	14500197	14600197	0.398839	TSPAN19
5	14500197	14600197	0.398839	LRRIQ1
5	14500235	14600235	0.398839	TSPAN19
5	14500235	14600235	0.398839	LRRIQ1
5	14500535	14600535	0.385587	TSPAN19
5	14500535	14600535	0.385587	LRRIQ1
5	14500778	14600778	0.387706	TSPAN19
5	14500778	14600778	0.387706	LRRIQ1
5	14501054	14601054	0.387487	TSPAN19
5	14501054	14601054	0.387487	LRRIQ1
5	14501123	14601123	0.396281	TSPAN19
5	14501123	14601123	0.396281	LRRIQ1
5	14501263	14601263	0.394668	TSPAN19
5	14501263	14601263	0.394668	LRRIQ1
5	14501319	14601319	0.380741	TSPAN19
5	14501319	14601319	0.380741	LRRIQ1
5	14501408	14601408	0.387743	TSPAN19
5	14501408	14601408	0.387743	LRRIQ1
5	14501448	14601448	0.386078	TSPAN19
5	14501448	14601448	0.386078	LRRIQ1
5	14501552	14601552	0.386714	TSPAN19
5	14501552	14601552	0.386714	LRRIQ1
5	14501943	14601943	0.400086	TSPAN19
5	14501943	14601943	0.400086	LRRIQ1
5	14502162	14602162	0.389352	TSPAN19
5	14502162	14602162	0.389352	LRRIQ1
5	14502697	14602697	0.384431	TSPAN19
5	14502697	14602697	0.384431	LRRIQ1
5	14502826	14602826	0.372202	TSPAN19
5	14502826	14602826	0.372202	LRRIQ1
5	14502970	14602970	0.392741	TSPAN19
5	14502970	14602970	0.392741	LRRIQ1
5	14503712	14603712	0.38715	TSPAN19
5	14503712	14603712	0.38715	LRRIQ1
5	14504060	14604060	0.403701	TSPAN19
5	14504060	14604060	0.403701	LRRIQ1
5	14504142	14604142	0.398508	TSPAN19
5	14504142	14604142	0.398508	LRRIQ1
5	14504252	14604252	0.396909	TSPAN19
5	14504252	14604252	0.396909	LRRIQ1
5	14504449	14604449	0.389977	TSPAN19
5	14504449	14604449	0.389977	LRRIQ1
5	14505279	14605279	0.395367	TSPAN19
5	14505279	14605279	0.395367	LRRIQ1
5	14505298	14605298	0.393701	TSPAN19
5	14505298	14605298	0.393701	LRRIQ1
5	14505345	14605345	0.399389	TSPAN19
5	14505345	14605345	0.399389	LRRIQ1
5	14505395	14605395	0.399389	TSPAN19
5	14505395	14605395	0.399389	LRRIQ1
5	14505420	14605420	0.38465	TSPAN19
5	14505420	14605420	0.38465	LRRIQ1
5	14505577	14605577	0.385275	TSPAN19
5	14505577	14605577	0.385275	LRRIQ1
5	14505680	14605680	0.390548	TSPAN19
5	14505680	14605680	0.390548	LRRIQ1
5	14506062	14606062	0.38747	TSPAN19

5	14506062	14606062	0.38747	LRRIQ1
5	14506373	14606373	0.387732	TSPAN19
5	14506373	14606373	0.387732	LRRIQ1
5	14506402	14606402	0.384601	TSPAN19
5	14506402	14606402	0.384601	LRRIQ1
5	14506864	14606864	0.387972	TSPAN19
5	14506864	14606864	0.387972	LRRIQ1
5	14506971	14606971	0.387114	TSPAN19
5	14506971	14606971	0.387114	LRRIQ1
5	14507041	14607041	0.387818	TSPAN19
5	14507041	14607041	0.387818	LRRIQ1
5	14507169	14607169	0.388953	TSPAN19
5	14507169	14607169	0.388953	LRRIQ1
5	14507408	14607408	0.383919	TSPAN19
5	14507408	14607408	0.383919	LRRIQ1
5	14507482	14607482	0.383877	TSPAN19
5	14507482	14607482	0.383877	LRRIQ1
5	14507931	14607931	0.369127	TSPAN19
5	14507931	14607931	0.369127	LRRIQ1
5	14508571	14608571	0.363392	TSPAN19
5	14508571	14608571	0.363392	LRRIQ1
5	14508772	14608772	0.363392	TSPAN19
5	14508772	14608772	0.363392	LRRIQ1
5	14509078	14609078	0.362943	TSPAN19
5	14509078	14609078	0.362943	LRRIQ1
5	14509193	14609193	0.368739	TSPAN19
5	14509193	14609193	0.368739	LRRIQ1
5	14509489	14609489	0.380848	TSPAN19
5	14509489	14609489	0.380848	LRRIQ1
5	14515614	14615614	0.356576	TSPAN19
5	14515614	14615614	0.356576	LRRIQ1
5	14515692	14615692	0.34386	TSPAN19
5	14515692	14615692	0.34386	LRRIQ1
5	14515695	14615695	0.343042	TSPAN19
5	14515695	14615695	0.343042	LRRIQ1
5	14518195	14618195	0.349028	TSPAN19
5	14518195	14618195	0.349028	LRRIQ1
5	14518637	14618637	0.343734	TSPAN19
5	14518637	14618637	0.343734	LRRIQ1
5	14518648	14618648	0.343734	TSPAN19
5	14518648	14618648	0.343734	LRRIQ1
5	14519403	14619403	0.384345	TSPAN19
5	14519403	14619403	0.384345	LRRIQ1
5	14519475	14619475	0.383733	TSPAN19
5	14519475	14619475	0.383733	LRRIQ1
5	14519912	14619912	0.41523	TSPAN19
5	14519912	14619912	0.41523	LRRIQ1
5	14520235	14620235	0.407149	TSPAN19
5	14520235	14620235	0.407149	LRRIQ1
5	14520631	14620631	0.410572	TSPAN19
5	14520631	14620631	0.410572	LRRIQ1
5	14520718	14620718	0.407222	TSPAN19
5	14520718	14620718	0.407222	LRRIQ1
5	14521081	14621081	0.405898	TSPAN19
5	14521081	14621081	0.405898	LRRIQ1
5	14521380	14621380	0.404761	TSPAN19
5	14521380	14621380	0.404761	LRRIQ1
5	14521798	14621798	0.404761	TSPAN19

5	14521798	14621798	0.404761	LRRIQ1
5	14521814	14621814	0.404761	TSPAN19
5	14521814	14621814	0.404761	LRRIQ1
5	14521816	14621816	0.404761	TSPAN19
5	14521816	14621816	0.404761	LRRIQ1
5	14521819	14621819	0.409598	TSPAN19
5	14521819	14621819	0.409598	LRRIQ1
5	14522340	14622340	0.38984	TSPAN19
5	14522340	14622340	0.38984	LRRIQ1
5	14522770	14622770	0.342114	TSPAN19
5	14522770	14622770	0.342114	LRRIQ1
5	14523205	14623205	0.359188	TSPAN19
5	14523205	14623205	0.359188	LRRIQ1
5	14523215	14623215	0.369863	TSPAN19
5	14523215	14623215	0.369863	LRRIQ1
5	14523338	14623338	0.368312	TSPAN19
5	14523338	14623338	0.368312	LRRIQ1
5	14523411	14623411	0.362958	TSPAN19
5	14523411	14623411	0.362958	LRRIQ1
5	14523508	14623508	0.367869	TSPAN19
5	14523508	14623508	0.367869	LRRIQ1
5	14523657	14623657	0.369354	TSPAN19
5	14523657	14623657	0.369354	LRRIQ1
5	14523671	14623671	0.368837	TSPAN19
5	14523671	14623671	0.368837	LRRIQ1
5	14523815	14623815	0.367758	TSPAN19
5	14523815	14623815	0.367758	LRRIQ1
5	14524128	14624128	0.36669	TSPAN19
5	14524128	14624128	0.36669	LRRIQ1
5	14524131	14624131	0.368675	TSPAN19
5	14524131	14624131	0.368675	LRRIQ1
5	14524153	14624153	0.368909	TSPAN19
5	14524153	14624153	0.368909	LRRIQ1
5	14524238	14624238	0.376753	TSPAN19
5	14524238	14624238	0.376753	LRRIQ1
5	14524270	14624270	0.351578	TSPAN19
5	14524270	14624270	0.351578	LRRIQ1
5	14524817	14624817	0.358358	TSPAN19
5	14524817	14624817	0.358358	LRRIQ1
5	14525143	14625143	0.358044	TSPAN19
5	14525143	14625143	0.358044	LRRIQ1
5	14525203	14625203	0.357923	TSPAN19
5	14525203	14625203	0.357923	LRRIQ1
5	14525311	14625311	0.378585	TSPAN19
5	14525311	14625311	0.378585	LRRIQ1
5	14525341	14625341	0.375743	TSPAN19
5	14525341	14625341	0.375743	LRRIQ1
5	14525503	14625503	0.378913	TSPAN19
5	14525503	14625503	0.378913	LRRIQ1
5	14525663	14625663	0.369942	TSPAN19
5	14525663	14625663	0.369942	LRRIQ1
5	14525788	14625788	0.36949	TSPAN19
5	14525788	14625788	0.36949	LRRIQ1
5	14526171	14626171	0.370466	TSPAN19
5	14526171	14626171	0.370466	LRRIQ1
5	14526455	14626455	0.371143	TSPAN19
5	14526455	14626455	0.371143	LRRIQ1
5	14526665	14626665	0.378523	TSPAN19

5	14526665	14626665	0.378523	LRRIQ1
5	14526672	14626672	0.377588	TSPAN19
5	14526672	14626672	0.377588	LRRIQ1
5	14526678	14626678	0.381792	TSPAN19
5	14526678	14626678	0.381792	LRRIQ1
5	14526704	14626704	0.383594	TSPAN19
5	14526704	14626704	0.383594	LRRIQ1
5	14526710	14626710	0.352862	TSPAN19
5	14526710	14626710	0.352862	LRRIQ1
5	14526718	14626718	0.355119	TSPAN19
5	14526718	14626718	0.355119	LRRIQ1
5	14526733	14626733	0.376996	TSPAN19
5	14526733	14626733	0.376996	LRRIQ1
5	14526845	14626845	0.376334	TSPAN19
5	14526845	14626845	0.376334	LRRIQ1
5	14526895	14626895	0.377337	TSPAN19
5	14526895	14626895	0.377337	LRRIQ1
5	14526901	14626901	0.377439	TSPAN19
5	14526901	14626901	0.377439	LRRIQ1
5	14526935	14626935	0.365471	TSPAN19
5	14526935	14626935	0.365471	LRRIQ1
5	14527116	14627116	0.372008	TSPAN19
5	14527116	14627116	0.372008	LRRIQ1
5	14527147	14627147	0.373247	TSPAN19
5	14527147	14627147	0.373247	LRRIQ1
5	14527211	14627211	0.373591	TSPAN19
5	14527211	14627211	0.373591	LRRIQ1
5	14527216	14627216	0.403623	TSPAN19
5	14527216	14627216	0.403623	LRRIQ1
5	14527224	14627224	0.396259	TSPAN19
5	14527224	14627224	0.396259	LRRIQ1
5	14527246	14627246	0.380598	TSPAN19
5	14527246	14627246	0.380598	LRRIQ1
5	14527310	14627310	0.379939	TSPAN19
5	14527310	14627310	0.379939	LRRIQ1
5	14527346	14627346	0.373333	TSPAN19
5	14527346	14627346	0.373333	LRRIQ1
5	14527367	14627367	0.375189	TSPAN19
5	14527367	14627367	0.375189	LRRIQ1
5	14527417	14627417	0.395941	TSPAN19
5	14527417	14627417	0.395941	LRRIQ1
5	14527429	14627429	0.395941	TSPAN19
5	14527429	14627429	0.395941	LRRIQ1
5	14527640	14627640	0.408324	TSPAN19
5	14527640	14627640	0.408324	LRRIQ1
5	14527659	14627659	0.395014	TSPAN19
5	14527659	14627659	0.395014	LRRIQ1
5	14527765	14627765	0.395014	TSPAN19
5	14527765	14627765	0.395014	LRRIQ1
5	14527800	14627800	0.390242	TSPAN19
5	14527800	14627800	0.390242	LRRIQ1
5	14527973	14627973	0.390242	TSPAN19
5	14527973	14627973	0.390242	LRRIQ1
5	14527981	14627981	0.390242	TSPAN19
5	14527981	14627981	0.390242	LRRIQ1
5	14528306	14628306	0.393237	TSPAN19
5	14528306	14628306	0.393237	LRRIQ1
5	14528375	14628375	0.395114	TSPAN19

5	14528375	14628375	0.395114	LRRIQ1
5	14528430	14628430	0.394381	TSPAN19
5	14528430	14628430	0.394381	LRRIQ1
5	14528624	14628624	0.392243	TSPAN19
5	14528624	14628624	0.392243	LRRIQ1
5	14528706	14628706	0.395501	TSPAN19
5	14528706	14628706	0.395501	LRRIQ1
5	14528759	14628759	0.398139	TSPAN19
5	14528759	14628759	0.398139	LRRIQ1
5	14528791	14628791	0.392962	TSPAN19
5	14528791	14628791	0.392962	LRRIQ1
5	14528801	14628801	0.393154	TSPAN19
5	14528801	14628801	0.393154	LRRIQ1
5	14528816	14628816	0.390201	TSPAN19
5	14528816	14628816	0.390201	LRRIQ1
5	14528827	14628827	0.388167	TSPAN19
5	14528827	14628827	0.388167	LRRIQ1
5	14528947	14628947	0.388082	TSPAN19
5	14528947	14628947	0.388082	LRRIQ1
5	14529258	14629258	0.38504	TSPAN19
5	14529258	14629258	0.38504	LRRIQ1
5	14529435	14629435	0.380127	TSPAN19
5	14529435	14629435	0.380127	LRRIQ1
5	14529463	14629463	0.382379	TSPAN19
5	14529463	14629463	0.382379	LRRIQ1
5	14530316	14630316	0.450684	TSPAN19
5	14530316	14630316	0.450684	LRRIQ1
5	14530943	14630943	0.370298	LRRIQ1
5	14531027	14631027	0.367954	LRRIQ1
5	14531043	14631043	0.367954	LRRIQ1
5	14531082	14631082	0.367954	LRRIQ1
5	14531245	14631245	0.37037	LRRIQ1
5	14531992	14631992	0.367353	LRRIQ1
5	14532046	14632046	0.377478	LRRIQ1
5	14532356	14632356	0.35616	LRRIQ1
5	14532359	14632359	0.366531	LRRIQ1
5	14532374	14632374	0.383346	LRRIQ1
5	14532568	14632568	0.373405	LRRIQ1
5	14533050	14633050	0.374806	LRRIQ1
5	14533221	14633221	0.371859	LRRIQ1
5	14533399	14633399	0.361578	LRRIQ1
5	14533474	14633474	0.370975	LRRIQ1
5	14533737	14633737	0.370975	LRRIQ1
5	14533779	14633779	0.367619	LRRIQ1
5	14533979	14633979	0.367619	LRRIQ1
5	14534084	14634084	0.368131	LRRIQ1
5	14534308	14634308	0.370399	LRRIQ1
5	14534611	14634611	0.368406	LRRIQ1
5	14534642	14634642	0.375729	LRRIQ1
5	14534649	14634649	0.367116	LRRIQ1
5	14534725	14634725	0.361531	LRRIQ1
5	14535800	14635800	0.354619	LRRIQ1
5	14536055	14636055	0.403736	LRRIQ1
5	14536120	14636120	0.403736	LRRIQ1
5	14536289	14636289	0.403736	LRRIQ1
5	14536562	14636562	0.403736	LRRIQ1
5	14536853	14636853	0.399	LRRIQ1
5	14536869	14636869	0.41103	LRRIQ1

5	14536938	14636938	0.41103	LRRIQ1
5	14537048	14637048	0.412245	LRRIQ1
5	14537131	14637131	0.41128	LRRIQ1
5	14537629	14637629	0.41281	LRRIQ1
5	14538170	14638170	0.416549	LRRIQ1
5	14538368	14638368	0.366708	LRRIQ1
5	14538414	14638414	0.375752	LRRIQ1
5	14538655	14638655	0.370879	LRRIQ1
5	14538687	14638687	0.370879	LRRIQ1
5	14538759	14638759	0.37431	LRRIQ1
5	14538821	14638821	0.36089	LRRIQ1
5	14538822	14638822	0.36089	LRRIQ1
5	14539029	14639029	0.360229	LRRIQ1
5	14539101	14639101	0.406438	LRRIQ1
5	14539186	14639186	0.375561	LRRIQ1
5	14539206	14639206	0.375561	LRRIQ1
5	14539223	14639223	0.375561	LRRIQ1
5	14539282	14639282	0.398844	LRRIQ1
5	14539395	14639395	0.379443	LRRIQ1
5	14539446	14639446	0.382167	LRRIQ1
5	14539462	14639462	0.382167	LRRIQ1
5	14539585	14639585	0.382167	LRRIQ1
5	14539730	14639730	0.382167	LRRIQ1
5	14539751	14639751	0.382167	LRRIQ1
5	14539975	14639975	0.378481	LRRIQ1
5	14540027	14640027	0.383303	LRRIQ1
5	14540028	14640028	0.383303	LRRIQ1
5	14540037	14640037	0.383303	LRRIQ1
5	14540046	14640046	0.385316	LRRIQ1
5	14540078	14640078	0.378323	LRRIQ1
5	14540081	14640081	0.378323	LRRIQ1
5	14540230	14640230	0.376464	LRRIQ1
5	14540293	14640293	0.379486	LRRIQ1
5	14540377	14640377	0.378287	LRRIQ1
5	14540501	14640501	0.378287	LRRIQ1
5	14540514	14640514	0.381457	LRRIQ1
5	14540573	14640573	0.371623	LRRIQ1
5	14540789	14640789	0.388595	LRRIQ1
5	14540806	14640806	0.38287	LRRIQ1
5	14540856	14640856	0.38287	LRRIQ1
5	14540908	14640908	0.385088	LRRIQ1
5	14540963	14640963	0.385099	LRRIQ1
5	14541008	14641008	0.385099	LRRIQ1
5	14541095	14641095	0.387549	LRRIQ1
5	14541158	14641158	0.384304	LRRIQ1
5	14541286	14641286	0.383277	LRRIQ1
5	14541500	14641500	0.383736	LRRIQ1
5	14541763	14641763	0.366553	LRRIQ1
5	14541923	14641923	0.368533	LRRIQ1
5	14541986	14641986	0.368262	LRRIQ1
5	14541987	14641987	0.368262	LRRIQ1
5	14542110	14642110	0.368262	LRRIQ1
5	14542130	14642130	0.368262	LRRIQ1
5	14542229	14642229	0.395152	LRRIQ1
5	14542361	14642361	0.383033	LRRIQ1
5	14542370	14642370	0.402602	LRRIQ1
5	14542434	14642434	0.41063	LRRIQ1
5	14542477	14642477	0.410335	LRRIQ1

5	14542512	14642512	0.413144	LRRIQ1
5	14542549	14642549	0.410173	LRRIQ1
5	14542582	14642582	0.410173	LRRIQ1
5	14542645	14642645	0.410173	LRRIQ1
5	14542667	14642667	0.410173	LRRIQ1
5	14542675	14642675	0.410173	LRRIQ1
5	14542685	14642685	0.410173	LRRIQ1
5	14542717	14642717	0.410173	LRRIQ1
5	14542743	14642743	0.416082	LRRIQ1
5	14542771	14642771	0.409941	LRRIQ1
5	14542788	14642788	0.413394	LRRIQ1
5	14542870	14642870	0.406706	LRRIQ1
5	14542873	14642873	0.406706	LRRIQ1
5	14542923	14642923	0.406706	LRRIQ1
5	14543092	14643092	0.406706	LRRIQ1
5	14543115	14643115	0.406706	LRRIQ1
5	14543190	14643190	0.412212	LRRIQ1
5	14543192	14643192	0.415836	LRRIQ1
5	14543207	14643207	0.410678	LRRIQ1
5	14543219	14643219	0.413276	LRRIQ1
5	14543272	14643272	0.412647	LRRIQ1
5	14543356	14643356	0.412647	LRRIQ1
5	14543505	14643505	0.41706	LRRIQ1
5	14543561	14643561	0.418217	LRRIQ1
5	14543586	14643586	0.408014	LRRIQ1
5	14543607	14643607	0.408014	LRRIQ1
5	14543614	14643614	0.408014	LRRIQ1
5	14543631	14643631	0.408014	LRRIQ1
5	14543669	14643669	0.428084	LRRIQ1
5	14543790	14643790	0.395607	LRRIQ1
5	14544413	14644413	0.397902	LRRIQ1
5	14544486	14644486	0.398475	LRRIQ1
5	14544710	14644710	0.399677	LRRIQ1
5	14544863	14644863	0.396563	LRRIQ1
5	14544903	14644903	0.396563	LRRIQ1
5	14545024	14645024	0.396563	LRRIQ1
5	14545272	14645272	0.3992	LRRIQ1
5	14545309	14645309	0.392692	LRRIQ1
5	14545325	14645325	0.392692	LRRIQ1
5	14545378	14645378	0.392692	LRRIQ1
5	14545384	14645384	0.392692	LRRIQ1
5	14545388	14645388	0.392692	LRRIQ1
5	14545781	14645781	0.401646	LRRIQ1
5	14545842	14645842	0.365064	LRRIQ1
5	14546471	14646471	0.365064	LRRIQ1
5	14546526	14646526	0.378812	LRRIQ1
5	14547114	14647114	0.378384	LRRIQ1
5	14547520	14647520	0.378384	LRRIQ1
5	14547849	14647849	0.39826	LRRIQ1
5	14547853	14647853	0.425837	LRRIQ1
5	14548047	14648047	0.427129	LRRIQ1
5	14548097	14648097	0.414856	LRRIQ1
5	14548257	14648257	0.420729	LRRIQ1
5	14548647	14648647	0.419241	LRRIQ1
5	14548653	14648653	0.425472	LRRIQ1
5	14548658	14648658	0.424852	LRRIQ1
5	14548733	14648733	0.436802	LRRIQ1
5	14548798	14648798	0.381168	LRRIQ1

5	14548835	14648835	0.380957	LRRIQ1
5	14548986	14648986	0.376649	LRRIQ1
5	14549091	14649091	0.377003	LRRIQ1
5	14549337	14649337	0.377003	LRRIQ1
5	14549734	14649734	0.407857	LRRIQ1
5	14550178	14650178	0.415993	LRRIQ1
5	14550680	14650680	0.410583	LRRIQ1
5	14550697	14650697	0.423328	LRRIQ1
5	14551376	14651376	0.403579	LRRIQ1
5	14551641	14651641	0.434787	LRRIQ1
5	14551651	14651651	0.430739	LRRIQ1
5	14551951	14651951	0.458802	LRRIQ1
5	14551984	14651984	0.479087	LRRIQ1
5	14551998	14651998	0.504111	LRRIQ1
5	14552012	14652012	0.484391	LRRIQ1
5	14552013	14652013	0.499033	LRRIQ1
5	14552041	14652041	0.485745	LRRIQ1
5	14552076	14652076	0.478505	LRRIQ1
5	14552163	14652163	0.474923	LRRIQ1
5	14552198	14652198	0.481029	LRRIQ1
5	14552204	14652204	0.478396	LRRIQ1
5	14552212	14652212	0.455233	LRRIQ1
5	14552337	14652337	0.432901	LRRIQ1
5	14552487	14652487	0.43223	LRRIQ1
5	14553145	14653145	0.423124	LRRIQ1
5	14553459	14653459	0.373528	LRRIQ1
5	14554557	14654557	0.381082	LRRIQ1
5	14554888	14654888	0.380379	LRRIQ1
5	14555197	14655197	0.401956	LRRIQ1
5	14555201	14655201	0.445187	LRRIQ1
5	14555433	14655433	0.42951	LRRIQ1
5	14555673	14655673	0.42951	LRRIQ1
5	14555686	14655686	0.435627	LRRIQ1
5	14555807	14655807	0.426114	LRRIQ1
5	14556091	14656091	0.345722	LRRIQ1
5	14556281	14656281	0.374846	LRRIQ1
5	14556290	14656290	0.396132	LRRIQ1
5	14556300	14656300	0.41179	LRRIQ1
5	14556816	14656816	0.391409	LRRIQ1
5	15265007	15365007	0.356208	RASSF9
5	15265012	15365012	0.354234	RASSF9
5	15265110	15365110	0.353094	RASSF9
5	15265127	15365127	0.350578	RASSF9
5	15265228	15365228	0.345698	RASSF9
5	15265336	15365336	0.341725	RASSF9
5	15265402	15365402	0.342555	RASSF9
5	15265485	15365485	0.35425	RASSF9
5	15265504	15365504	0.35425	RASSF9
5	15265516	15365516	0.35425	RASSF9
5	15265527	15365527	0.35425	RASSF9
5	15265535	15365535	0.354087	RASSF9
5	15265551	15365551	0.354087	RASSF9
5	15265554	15365554	0.361898	RASSF9
5	15265584	15365584	0.3606	RASSF9
5	15265585	15365585	0.3606	RASSF9
5	15265645	15365645	0.377912	RASSF9
5	15265646	15365646	0.379723	RASSF9
5	15265653	15365653	0.379723	RASSF9

5	15265659	15365659	0.379723	RASSF9
5	15265670	15365670	0.382585	RASSF9
5	15265724	15365724	0.364523	RASSF9
5	15265735	15365735	0.364523	RASSF9
5	15265742	15365742	0.362804	RASSF9
5	15265753	15365753	0.348661	RASSF9
5	15265771	15365771	0.348998	RASSF9
5	15265781	15365781	0.351215	RASSF9
5	15265792	15365792	0.351219	RASSF9
5	15265807	15365807	0.350958	RASSF9
5	15265824	15365824	0.351224	RASSF9
5	15265849	15365849	0.347197	RASSF9
5	15265882	15365882	0.346536	RASSF9
5	15265906	15365906	0.346536	RASSF9
5	15265925	15365925	0.346635	RASSF9
5	15265937	15365937	0.346635	RASSF9
5	15265942	15365942	0.346635	RASSF9
5	15266223	15366223	0.343808	RASSF9
5	15266318	15366318	0.344043	RASSF9
5	15266482	15366482	0.343656	RASSF9
5	15266559	15366559	0.346285	RASSF9
5	15266565	15366565	0.346285	RASSF9
5	15266587	15366587	0.349837	RASSF9
5	15266588	15366588	0.349837	RASSF9
5	15266589	15366589	0.349837	RASSF9
5	15266624	15366624	0.346615	RASSF9
5	15266626	15366626	0.346615	RASSF9
5	15266635	15366635	0.359052	RASSF9
5	15266644	15366644	0.353862	RASSF9
5	15266652	15366652	0.349228	RASSF9
5	15266675	15366675	0.350745	RASSF9
5	15266683	15366683	0.349722	RASSF9
5	15266711	15366711	0.351306	RASSF9
5	15267867	15367867	0.342554	RASSF9
5	15267892	15367892	0.347977	RASSF9
5	15267893	15367893	0.36125	RASSF9
5	15267918	15367918	0.376666	RASSF9
5	15267935	15367935	0.378936	RASSF9
5	15267951	15367951	0.374444	RASSF9
5	15267972	15367972	0.376168	RASSF9
5	15268006	15368006	0.385108	RASSF9
5	15268111	15368111	0.37641	RASSF9
5	15268120	15368120	0.381414	RASSF9
5	15268127	15368127	0.384438	RASSF9
5	15268143	15368143	0.38084	RASSF9
5	15268196	15368196	0.377209	RASSF9
5	15268323	15368323	0.373806	RASSF9
5	15268575	15368575	0.354595	RASSF9
5	15268600	15368600	0.355828	RASSF9
5	17195927	17295927	0.465054	TRNAC-ACA-24
5	17196096	17296096	0.367138	TRNAC-ACA-24
5	17196429	17296429	0.375162	TRNAC-ACA-24
5	17196448	17296448	0.384544	TRNAC-ACA-24
5	17196923	17296923	0.374335	TRNAC-ACA-24
5	17198034	17298034	0.382907	TRNAC-ACA-24
5	17198442	17298442	0.371784	TRNAC-ACA-24
5	17198461	17298461	0.367266	TRNAC-ACA-24
5	17199029	17299029	0.367266	TRNAC-ACA-24

5	17199125	17299125	0.377779	TRNAC-ACA-24
5	17199275	17299275	0.370561	TRNAC-ACA-24
5	17199329	17299329	0.369363	TRNAC-ACA-24
5	17201659	17301659	0.364623	TRNAC-ACA-24
5	17201785	17301785	0.380702	TRNAC-ACA-24
5	17202004	17302004	0.382751	TRNAC-ACA-24
5	17202807	17302807	0.372341	TRNAC-ACA-24
5	17203025	17303025	0.369986	TRNAC-ACA-24
5	17203422	17303422	0.379001	TRNAC-ACA-24
5	17203544	17303544	0.379001	TRNAC-ACA-24
5	17203864	17303864	0.390842	TRNAC-ACA-24
5	17203990	17303990	0.390934	TRNAC-ACA-24
5	17204218	17304218	0.389405	TRNAC-ACA-24
5	17204348	17304348	0.375596	TRNAC-ACA-24
5	17204414	17304414	0.375596	TRNAC-ACA-24
5	17204514	17304514	0.375073	TRNAC-ACA-24
5	17206843	17306843	0.375946	TRNAC-ACA-24
5	17207154	17307154	0.375946	TRNAC-ACA-24
5	17207317	17307317	0.384797	TRNAC-ACA-24
5	17207394	17307394	0.399291	TRNAC-ACA-24
5	17207396	17307396	0.399291	TRNAC-ACA-24
5	17207397	17307397	0.399291	TRNAC-ACA-24
5	17207541	17307541	0.398866	TRNAC-ACA-24
5	17207655	17307655	0.397276	TRNAC-ACA-24
5	17207817	17307817	0.397705	TRNAC-ACA-24
5	17207864	17307864	0.395906	TRNAC-ACA-24
5	17208026	17308026	0.389654	TRNAC-ACA-24
5	17208589	17308589	0.39152	TRNAC-ACA-24
5	17209377	17309377	0.38818	TRNAC-ACA-24
5	17209726	17309726	0.38602	TRNAC-ACA-24
5	17209806	17309806	0.389451	TRNAC-ACA-24
5	17209994	17309994	0.384767	TRNAC-ACA-24
5	17210000	17310000	0.388396	TRNAC-ACA-24
5	17210150	17310150	0.382428	TRNAC-ACA-24
5	17210335	17310335	0.385055	TRNAC-ACA-24
5	17210363	17310363	0.385394	TRNAC-ACA-24
5	17210447	17310447	0.382381	TRNAC-ACA-24
5	17210678	17310678	0.382115	TRNAC-ACA-24
5	17210805	17310805	0.386058	TRNAC-ACA-24
5	17211114	17311114	0.384324	TRNAC-ACA-24
5	17211202	17311202	0.386943	TRNAC-ACA-24
5	17211251	17311251	0.375754	TRNAC-ACA-24
5	17211256	17311256	0.37705	TRNAC-ACA-24
5	17211494	17311494	0.379518	TRNAC-ACA-24
5	19153928	19253928	0.351096	LOC102172790
5	19153928	19253928	0.351096	POC1B
5	19153928	19253928	0.351096	ATP2B1
5	19154485	19254485	0.358638	LOC102172790
5	19154485	19254485	0.358638	POC1B
5	19154485	19254485	0.358638	ATP2B1
5	19154577	19254577	0.418737	LOC102172790
5	19154577	19254577	0.418737	POC1B
5	19154577	19254577	0.418737	ATP2B1
5	19154579	19254579	0.357218	LOC102172790
5	19154579	19254579	0.357218	POC1B
5	19154579	19254579	0.357218	ATP2B1
5	19160625	19260625	0.349112	LOC102172790
5	19160625	19260625	0.349112	POC1B

5	19160625	19260625	0.349112	ATP2B1
5	19160688	19260688	0.368094	LOC102172790
5	19160688	19260688	0.368094	POC1B
5	19160688	19260688	0.368094	ATP2B1
5	19293725	19393725	0.368093	ATP2B1
5	19463594	19563594	0.354428	LOC108636126
5	22241359	22341359	0.342618	C5H12orf74
5	22241371	22341371	0.342125	C5H12orf74
5	22248879	22348879	0.419458	C5H12orf74
5	22248879	22348879	0.419458	PLEKHG7
5	22249182	22349182	0.40518	C5H12orf74
5	22249182	22349182	0.40518	PLEKHG7
5	22249721	22349721	0.404118	C5H12orf74
5	22249721	22349721	0.404118	PLEKHG7
5	22249869	22349869	0.392426	C5H12orf74
5	22249869	22349869	0.392426	PLEKHG7
5	22250288	22350288	0.366814	C5H12orf74
5	22250288	22350288	0.366814	PLEKHG7
5	22250563	22350563	0.374248	C5H12orf74
5	22250563	22350563	0.374248	PLEKHG7
5	22259021	22359021	0.419449	C5H12orf74
5	22259021	22359021	0.419449	PLEKHG7
5	22259504	22359504	0.416004	C5H12orf74
5	22259504	22359504	0.416004	PLEKHG7
5	22259518	22359518	0.416004	C5H12orf74
5	22259518	22359518	0.416004	PLEKHG7
5	22260270	22360270	0.423582	C5H12orf74
5	22260270	22360270	0.423582	PLEKHG7
5	22260518	22360518	0.426213	C5H12orf74
5	22260518	22360518	0.426213	PLEKHG7
5	22260626	22360626	0.387518	C5H12orf74
5	22260626	22360626	0.387518	PLEKHG7
5	22261072	22361072	0.382013	C5H12orf74
5	22261072	22361072	0.382013	PLEKHG7
5	22261093	22361093	0.368697	C5H12orf74
5	22261093	22361093	0.368697	PLEKHG7
5	22261152	22361152	0.376288	C5H12orf74
5	22261152	22361152	0.376288	PLEKHG7
5	22261441	22361441	0.357351	C5H12orf74
5	22261441	22361441	0.357351	PLEKHG7
5	22261977	22361977	0.355258	C5H12orf74
5	22261977	22361977	0.355258	PLEKHG7
5	22262388	22362388	0.382523	C5H12orf74
5	22262388	22362388	0.382523	PLEKHG7
5	22262416	22362416	0.35944	C5H12orf74
5	22262416	22362416	0.35944	PLEKHG7
5	22262680	22362680	0.350445	C5H12orf74
5	22262680	22362680	0.350445	PLEKHG7
5	22263288	22363288	0.35232	C5H12orf74
5	22263288	22363288	0.35232	PLEKHG7
5	22263327	22363327	0.371011	C5H12orf74
5	22263327	22363327	0.371011	PLEKHG7
5	22263447	22363447	0.35781	C5H12orf74
5	22263447	22363447	0.35781	PLEKHG7
5	22263728	22363728	0.358334	C5H12orf74
5	22263728	22363728	0.358334	PLEKHG7
5	22264035	22364035	0.380439	C5H12orf74
5	22264035	22364035	0.380439	PLEKHG7

5	22264040	22364040	0.380439	C5H12orf74
5	22264040	22364040	0.380439	PLEKHG7
5	22265217	22365217	0.35473	C5H12orf74
5	22265217	22365217	0.35473	PLEKHG7
5	22265245	22365245	0.353282	C5H12orf74
5	22265245	22365245	0.353282	PLEKHG7
5	22265766	22365766	0.374935	C5H12orf74
5	22265766	22365766	0.374935	PLEKHG7
5	22265804	22365804	0.378429	C5H12orf74
5	22265804	22365804	0.378429	PLEKHG7
5	22265955	22365955	0.410664	C5H12orf74
5	22265955	22365955	0.410664	PLEKHG7
5	22266225	22366225	0.410699	C5H12orf74
5	22266225	22366225	0.410699	PLEKHG7
5	22266259	22366259	0.416023	C5H12orf74
5	22266259	22366259	0.416023	PLEKHG7
5	22266333	22366333	0.455515	C5H12orf74
5	22266333	22366333	0.455515	PLEKHG7
5	22266349	22366349	0.439071	C5H12orf74
5	22266349	22366349	0.439071	PLEKHG7
5	22266375	22366375	0.43846	C5H12orf74
5	22266375	22366375	0.43846	PLEKHG7
5	22266400	22366400	0.439442	C5H12orf74
5	22266400	22366400	0.439442	PLEKHG7
5	22266413	22366413	0.435017	C5H12orf74
5	22266413	22366413	0.435017	PLEKHG7
5	22266459	22366459	0.418302	C5H12orf74
5	22266459	22366459	0.418302	PLEKHG7
5	22266587	22366587	0.432982	C5H12orf74
5	22266587	22366587	0.432982	PLEKHG7
5	22266649	22366649	0.41275	C5H12orf74
5	22266649	22366649	0.41275	PLEKHG7
5	22267383	22367383	0.417089	C5H12orf74
5	22267383	22367383	0.417089	PLEKHG7
5	22267920	22367920	0.410322	C5H12orf74
5	22267920	22367920	0.410322	PLEKHG7
5	22267997	22367997	0.428591	C5H12orf74
5	22267997	22367997	0.428591	PLEKHG7
5	22268041	22368041	0.425666	C5H12orf74
5	22268041	22368041	0.425666	PLEKHG7
5	22268060	22368060	0.4279	C5H12orf74
5	22268060	22368060	0.4279	PLEKHG7
5	22268061	22368061	0.430973	C5H12orf74
5	22268061	22368061	0.430973	PLEKHG7
5	22268090	22368090	0.427024	C5H12orf74
5	22268090	22368090	0.427024	PLEKHG7
5	22268142	22368142	0.434826	C5H12orf74
5	22268142	22368142	0.434826	PLEKHG7
5	22268152	22368152	0.448018	C5H12orf74
5	22268152	22368152	0.448018	PLEKHG7
5	22268297	22368297	0.425327	C5H12orf74
5	22268297	22368297	0.425327	PLEKHG7
5	22268366	22368366	0.429181	C5H12orf74
5	22268366	22368366	0.429181	PLEKHG7
5	22268523	22368523	0.383057	C5H12orf74
5	22268523	22368523	0.383057	PLEKHG7
5	22268556	22368556	0.374321	C5H12orf74
5	22268556	22368556	0.374321	PLEKHG7

5	22268566	22368566	0.378562	C5H12orf74
5	22268566	22368566	0.378562	PLEKHG7
5	22268944	22368944	0.421649	C5H12orf74
5	22268944	22368944	0.421649	PLEKHG7
5	22270238	22370238	0.344327	C5H12orf74
5	22270238	22370238	0.344327	PLEKHG7
5	23147613	23247613	0.351866	CRADD
5	23147841	23247841	0.358069	CRADD
5	23147873	23247873	0.359256	CRADD
5	23147923	23247923	0.360357	CRADD
5	23148166	23248166	0.365823	CRADD
5	23148217	23248217	0.36252	CRADD
5	23148286	23248286	0.363792	CRADD
5	23148501	23248501	0.350265	CRADD
5	23148595	23248595	0.358499	CRADD
5	23148697	23248697	0.361632	CRADD
5	23148713	23248713	0.359658	CRADD
5	23148834	23248834	0.361911	CRADD
5	23148861	23248861	0.368248	CRADD
5	23148976	23248976	0.350728	CRADD
5	23149426	23249426	0.507873	CRADD
5	23149430	23249430	0.507873	CRADD
5	23149646	23249646	0.511537	CRADD
5	23149818	23249818	0.498138	CRADD
5	23149950	23249950	0.500705	CRADD
5	23149996	23249996	0.489995	CRADD
5	23150166	23250166	0.491898	CRADD
5	23150573	23250573	0.46668	CRADD
5	23150651	23250651	0.534027	CRADD
5	23151247	23251247	0.533623	CRADD
5	23151385	23251385	0.552118	CRADD
5	23151458	23251458	0.576775	CRADD
5	23151475	23251475	0.543775	CRADD
5	23151545	23251545	0.543775	CRADD
5	23151561	23251561	0.531207	CRADD
5	23152106	23252106	0.530238	CRADD
5	23152443	23252443	0.538731	CRADD
5	23152502	23252502	0.559202	CRADD
5	23152568	23252568	0.570151	CRADD
5	23152649	23252649	0.577256	CRADD
5	23152669	23252669	0.590039	CRADD
5	23152837	23252837	0.57564	CRADD
5	23153070	23253070	0.563014	CRADD
5	23153130	23253130	0.568763	CRADD
5	23153280	23253280	0.564693	CRADD
5	23153319	23253319	0.572103	CRADD
5	23153489	23253489	0.548954	CRADD
5	23153565	23253565	0.549625	CRADD
5	23153595	23253595	0.549625	CRADD
5	23153602	23253602	0.558671	CRADD
5	23153649	23253649	0.549924	CRADD
5	23153677	23253677	0.547798	CRADD
5	23153845	23253845	0.548203	CRADD
5	23153906	23253906	0.548203	CRADD
5	23153957	23253957	0.530948	CRADD
5	23154072	23254072	0.531572	CRADD
5	23154123	23254123	0.531572	CRADD
5	23154315	23254315	0.531572	CRADD

5	23154441	23254441	0.531572	CRADD
5	23154602	23254602	0.548999	CRADD
5	23154694	23254694	0.577207	CRADD
5	23154797	23254797	0.58549	CRADD
5	23154801	23254801	0.577011	CRADD
5	23154860	23254860	0.588912	CRADD
5	23154952	23254952	0.577853	CRADD
5	23154961	23254961	0.578524	CRADD
5	23154962	23254962	0.577455	CRADD
5	23154975	23254975	0.559476	CRADD
5	23155123	23255123	0.555765	CRADD
5	23155132	23255132	0.556173	CRADD
5	23155324	23255324	0.556705	CRADD
5	23155398	23255398	0.556705	CRADD
5	23155499	23255499	0.53422	CRADD
5	23155508	23255508	0.5422	CRADD
5	23155563	23255563	0.545155	CRADD
5	23155590	23255590	0.545601	CRADD
5	23155662	23255662	0.508863	CRADD
5	23155731	23255731	0.509424	CRADD
5	23155778	23255778	0.516426	CRADD
5	23155945	23255945	0.506223	CRADD
5	23156063	23256063	0.510494	CRADD
5	23156143	23256143	0.499882	CRADD
5	23156549	23256549	0.500408	CRADD
5	23156593	23256593	0.505794	CRADD
5	23156990	23256990	0.508841	CRADD
5	23157068	23257068	0.50546	CRADD
5	23157132	23257132	0.505611	CRADD
5	23157240	23257240	0.504694	CRADD
5	23157343	23257343	0.528557	CRADD
5	23157353	23257353	0.517369	CRADD
5	23157408	23257408	0.517369	CRADD
5	23157781	23257781	0.512571	CRADD
5	23157824	23257824	0.519961	CRADD
5	23157831	23257831	0.519253	CRADD
5	23157864	23257864	0.535457	CRADD
5	23157929	23257929	0.530281	CRADD
5	23158157	23258157	0.521155	CRADD
5	23158256	23258256	0.52312	CRADD
5	23158308	23258308	0.529449	CRADD
5	23158330	23258330	0.529449	CRADD
5	23158463	23258463	0.524504	CRADD
5	23158514	23258514	0.526526	CRADD
5	23158547	23258547	0.52547	CRADD
5	23158626	23258626	0.52441	CRADD
5	23158817	23258817	0.519612	CRADD
5	23158897	23258897	0.549683	CRADD
5	23159001	23259001	0.529309	CRADD
5	23159006	23259006	0.526224	CRADD
5	23159457	23259457	0.540669	CRADD
5	23159534	23259534	0.55317	CRADD
5	23160204	23260204	0.57023	CRADD
5	23160275	23260275	0.549506	CRADD
5	23160276	23260276	0.554137	CRADD
5	23160392	23260392	0.53784	CRADD
5	23161268	23261268	0.538236	CRADD
5	23161340	23261340	0.561145	CRADD

5	23161610	23261610	0.564093	CRADD
5	23161653	23261653	0.587649	CRADD
5	23161846	23261846	0.585116	CRADD
5	23161872	23261872	0.590111	CRADD
5	23161932	23261932	0.598395	CRADD
5	23162389	23262389	0.597414	CRADD
5	23162446	23262446	0.592174	CRADD
5	23162505	23262505	0.585789	CRADD
5	23162529	23262529	0.573345	CRADD
5	23162540	23262540	0.576966	CRADD
5	23162560	23262560	0.576983	CRADD
5	23162684	23262684	0.573703	CRADD
5	23162695	23262695	0.575774	CRADD
5	23162698	23262698	0.574317	CRADD
5	23162710	23262710	0.574317	CRADD
5	23162713	23262713	0.574317	CRADD
5	23162727	23262727	0.574317	CRADD
5	23162728	23262728	0.574317	CRADD
5	23162733	23262733	0.577812	CRADD
5	23162745	23262745	0.574852	CRADD
5	23162789	23262789	0.569276	CRADD
5	23162887	23262887	0.563256	CRADD
5	23163292	23263292	0.561519	CRADD
5	23163368	23263368	0.569381	CRADD
5	23163849	23263849	0.566907	CRADD
5	23163882	23263882	0.566907	CRADD
5	23164003	23264003	0.571921	CRADD
5	23164195	23264195	0.56975	CRADD
5	23164216	23264216	0.571052	CRADD
5	23164236	23264236	0.576357	CRADD
5	23164315	23264315	0.57174	CRADD
5	23164352	23264352	0.57174	CRADD
5	23164519	23264519	0.565763	CRADD
5	23164608	23264608	0.554513	CRADD
5	23164611	23264611	0.554513	CRADD
5	23164613	23264613	0.554513	CRADD
5	23164890	23264890	0.547436	CRADD
5	23165422	23265422	0.547912	CRADD
5	23165648	23265648	0.560069	CRADD
5	23165656	23265656	0.559103	CRADD
5	23165847	23265847	0.559103	CRADD
5	23165986	23265986	0.574095	CRADD
5	23166009	23266009	0.557773	CRADD
5	23166156	23266156	0.556781	CRADD
5	23166165	23266165	0.539537	CRADD
5	23166321	23266321	0.544647	CRADD
5	23166579	23266579	0.544647	CRADD
5	23166800	23266800	0.557962	CRADD
5	23166884	23266884	0.497569	CRADD
5	23166893	23266893	0.528489	CRADD
5	23166959	23266959	0.484001	CRADD
5	23167009	23267009	0.515274	CRADD
5	23167367	23267367	0.509768	CRADD
5	23167848	23267848	0.495542	CRADD
5	23168020	23268020	0.510771	CRADD
5	23168096	23268096	0.528954	CRADD
5	23168164	23268164	0.529476	CRADD
5	23168629	23268629	0.497455	CRADD

5	23168916	23268916	0.497455	CRADD
5	23169113	23269113	0.51559	CRADD
5	23169490	23269490	0.528152	CRADD
5	23169656	23269656	0.54892	CRADD
5	23169662	23269662	0.552348	CRADD
5	23169688	23269688	0.538224	CRADD
5	23169928	23269928	0.561609	CRADD
5	23170077	23270077	0.557367	CRADD
5	23170574	23270574	0.555566	CRADD
5	23171094	23271094	0.56133	CRADD
5	23171133	23271133	0.584189	CRADD
5	23171139	23271139	0.558075	CRADD
5	23171290	23271290	0.543155	CRADD
5	23171385	23271385	0.498117	CRADD
5	23171569	23271569	0.507719	CRADD
5	23172575	23272575	0.507719	CRADD
5	23172792	23272792	0.487454	CRADD
5	23172846	23272846	0.486491	CRADD
5	23173919	23273919	0.515933	CRADD
5	23173955	23273955	0.508279	CRADD
5	23174755	23274755	0.520904	CRADD
5	23174795	23274795	0.527127	CRADD
5	23175787	23275787	0.512028	CRADD
5	23175932	23275932	0.528848	CRADD
5	23176025	23276025	0.564512	CRADD
5	23176027	23276027	0.564512	CRADD
5	23176028	23276028	0.564512	CRADD
5	23176120	23276120	0.572571	CRADD
5	23176121	23276121	0.561057	CRADD
5	23176147	23276147	0.5671	CRADD
5	23176457	23276457	0.564472	CRADD
5	23176504	23276504	0.563989	CRADD
5	23176518	23276518	0.555794	CRADD
5	23177519	23277519	0.539844	CRADD
5	23177586	23277586	0.548661	CRADD
5	23177711	23277711	0.558274	CRADD
5	23177841	23277841	0.557195	CRADD
5	23177891	23277891	0.561424	CRADD
5	23177998	23277998	0.561478	CRADD
5	23178005	23278005	0.559343	CRADD
5	23178113	23278113	0.55237	CRADD
5	23178235	23278235	0.55237	CRADD
5	23178258	23278258	0.538191	CRADD
5	23178439	23278439	0.543747	CRADD
5	23178589	23278589	0.557333	CRADD
5	23178635	23278635	0.559986	CRADD
5	23178911	23278911	0.559986	CRADD
5	23178921	23278921	0.559986	CRADD
5	23179465	23279465	0.57579	CRADD
5	23179573	23279573	0.579817	CRADD
5	23179590	23279590	0.596187	CRADD
5	23179600	23279600	0.583558	CRADD
5	23179820	23279820	0.583558	CRADD
5	23179931	23279931	0.590494	CRADD
5	23179958	23279958	0.548001	CRADD
5	23180348	23280348	0.35319	CRADD
5	23180368	23280368	0.400025	CRADD
5	23180551	23280551	0.409309	CRADD

5	23180995	23280995	0.391258	CRADD
5	23181186	23281186	0.394952	CRADD
5	23181551	23281551	0.412846	CRADD
5	23181713	23281713	0.423491	CRADD
5	23181736	23281736	0.424611	CRADD
5	23181820	23281820	0.420657	CRADD
5	23181915	23281915	0.420657	CRADD
5	23181918	23281918	0.427468	CRADD
5	23181936	23281936	0.420898	CRADD
5	23181953	23281953	0.420898	CRADD
5	23181976	23281976	0.42518	CRADD
5	23181979	23281979	0.409555	CRADD
5	23181996	23281996	0.409555	CRADD
5	23182006	23282006	0.409555	CRADD
5	23182041	23282041	0.409555	CRADD
5	23182073	23282073	0.409555	CRADD
5	23182140	23282140	0.409555	CRADD
5	23182225	23282225	0.409555	CRADD
5	23182241	23282241	0.434749	CRADD
5	23182265	23282265	0.418939	CRADD
5	23182277	23282277	0.418939	CRADD
5	23182363	23282363	0.418939	CRADD
5	23182365	23282365	0.418939	CRADD
5	23182437	23282437	0.419363	CRADD
5	23182445	23282445	0.41905	CRADD
5	23182490	23282490	0.405308	CRADD
5	23183002	23283002	0.403081	CRADD
5	23183477	23283477	0.428527	CRADD
5	23183878	23283878	0.49678	CRADD
5	23183912	23283912	0.461284	CRADD
5	23183926	23283926	0.444646	CRADD
5	23183969	23283969	0.346732	CRADD
5	23238416	23338416	0.349298	CRADD
5	23238686	23338686	0.362309	CRADD
5	23238917	23338917	0.359716	CRADD
5	23238943	23338943	0.359716	CRADD
5	23239553	23339553	0.367339	CRADD
5	23239827	23339827	0.363887	CRADD
5	23240085	23340085	0.35117	CRADD
5	23240115	23340115	0.353848	CRADD
5	23240327	23340327	0.344928	CRADD
5	23240544	23340544	0.34936	CRADD
5	23240594	23340594	0.344045	CRADD
5	23240790	23340790	0.344045	CRADD
5	23241015	23341015	0.344045	CRADD
5	23241050	23341050	0.344045	CRADD
5	23241061	23341061	0.344045	CRADD
5	23241080	23341080	0.344045	CRADD
5	23241082	23341082	0.344045	CRADD
5	23241111	23341111	0.344045	CRADD
5	23241271	23341271	0.344045	CRADD
5	23241442	23341442	0.367734	CRADD
5	23241447	23341447	0.367579	CRADD
5	23241462	23341462	0.363375	CRADD
5	23241474	23341474	0.363375	CRADD
5	23242168	23342168	0.366658	CRADD
5	23242394	23342394	0.364416	CRADD
5	23242466	23342466	0.402427	CRADD

5	23242574	23342574	0.404013	CRADD
5	23242577	23342577	0.402427	CRADD
5	23242826	23342826	0.403742	CRADD
5	23242855	23342855	0.406025	CRADD
5	23242861	23342861	0.400056	CRADD
5	23242885	23342885	0.400056	CRADD
5	23242972	23342972	0.402686	CRADD
5	23242991	23342991	0.421323	CRADD
5	23243007	23343007	0.418333	CRADD
5	23243160	23343160	0.409726	CRADD
5	23243265	23343265	0.40674	CRADD
5	23243342	23343342	0.40674	CRADD
5	23243491	23343491	0.41038	CRADD
5	23243671	23343671	0.38943	CRADD
5	23243930	23343930	0.417938	CRADD
5	23244134	23344134	0.405084	CRADD
5	23244301	23344301	0.405833	CRADD
5	23244314	23344314	0.416718	CRADD
5	23244406	23344406	0.414623	CRADD
5	23244457	23344457	0.415497	CRADD
5	23244484	23344484	0.422818	CRADD
5	23244714	23344714	0.408263	CRADD
5	23244964	23344964	0.408263	CRADD
5	23244998	23344998	0.417841	CRADD
5	23245006	23345006	0.408401	CRADD
5	23245007	23345007	0.408401	CRADD
5	23245035	23345035	0.408401	CRADD
5	23245097	23345097	0.408401	CRADD
5	23245133	23345133	0.409986	CRADD
5	23245134	23345134	0.427735	CRADD
5	23245167	23345167	0.414803	CRADD
5	23245172	23345172	0.415146	CRADD
5	23245197	23345197	0.415146	CRADD
5	23245262	23345262	0.415659	CRADD
5	23245513	23345513	0.414058	CRADD
5	23245533	23345533	0.402858	CRADD
5	23245686	23345686	0.394723	CRADD
5	23245926	23345926	0.393984	CRADD
5	23245949	23345949	0.398713	CRADD
5	23246072	23346072	0.384987	CRADD
5	23246265	23346265	0.370717	CRADD
5	23246530	23346530	0.385584	CRADD
5	23246531	23346531	0.385584	CRADD
5	23246581	23346581	0.385584	CRADD
5	23246617	23346617	0.385584	CRADD
5	23246630	23346630	0.398574	CRADD
5	23246632	23346632	0.398574	CRADD
5	23246636	23346636	0.398478	CRADD
5	23246671	23346671	0.396601	CRADD
5	23246750	23346750	0.418771	CRADD
5	23246805	23346805	0.417992	CRADD
5	23247201	23347201	0.408499	CRADD
5	23247221	23347221	0.394042	CRADD
5	23247324	23347324	0.395728	CRADD
5	23247933	23347933	0.371995	CRADD
5	23248036	23348036	0.396183	CRADD
5	23248064	23348064	0.403693	CRADD
5	23248065	23348065	0.403693	CRADD

5	23248102	23348102	0.397646	CRADD
5	23248275	23348275	0.392506	CRADD
5	23248665	23348665	0.395983	CRADD
5	23248751	23348751	0.40588	CRADD
5	23249342	23349342	0.40588	CRADD
5	23249396	23349396	0.40588	CRADD
5	23249407	23349407	0.40588	CRADD
5	23249451	23349451	0.40588	CRADD
5	23249598	23349598	0.40588	CRADD
5	23249716	23349716	0.40588	CRADD
5	23249730	23349730	0.406743	CRADD
5	23249851	23349851	0.406465	CRADD
5	23249915	23349915	0.39259	CRADD
5	23249975	23349975	0.396029	CRADD
5	23250028	23350028	0.398272	CRADD
5	23250042	23350042	0.393983	CRADD
5	23250114	23350114	0.393983	CRADD
5	23250220	23350220	0.390803	CRADD
5	23250270	23350270	0.388867	CRADD
5	23250420	23350420	0.383952	CRADD
5	23250661	23350661	0.383952	CRADD
5	23250994	23350994	0.383952	CRADD
5	23251050	23351050	0.383952	CRADD
5	23251103	23351103	0.387033	CRADD
5	23251597	23351597	0.378516	CRADD
5	23251637	23351637	0.381264	CRADD
5	23251962	23351962	0.390634	CRADD
5	23252214	23352214	0.384039	CRADD
5	23252609	23352609	0.38699	CRADD
5	23252908	23352908	0.38699	CRADD
5	23252957	23352957	0.38699	CRADD
5	23252959	23352959	0.38699	CRADD
5	23252965	23352965	0.38699	CRADD
5	23253004	23353004	0.39255	CRADD
5	23253040	23353040	0.39255	CRADD
5	23253170	23353170	0.387732	CRADD
5	23253174	23353174	0.390884	CRADD
5	23253192	23353192	0.398336	CRADD
5	23253268	23353268	0.392913	CRADD
5	23253278	23353278	0.392913	CRADD
5	23253318	23353318	0.400797	CRADD
5	23253319	23353319	0.393129	CRADD
5	23253391	23353391	0.387081	CRADD
5	23253750	23353750	0.387081	CRADD
5	23253771	23353771	0.387081	CRADD
5	23254035	23354035	0.391233	CRADD
5	23254064	23354064	0.391689	CRADD
5	23254081	23354081	0.391689	CRADD
5	23254140	23354140	0.394876	CRADD
5	23254177	23354177	0.394876	CRADD
5	23254218	23354218	0.39401	CRADD
5	23254396	23354396	0.398434	CRADD
5	23254399	23354399	0.398434	CRADD
5	23254495	23354495	0.394251	CRADD
5	23254625	23354625	0.397009	CRADD
5	23254654	23354654	0.396925	CRADD
5	23255109	23355109	0.410532	CRADD
5	23255227	23355227	0.39793	CRADD

5	23255266	23355266	0.399902	CRADD
5	23255276	23355276	0.403681	CRADD
5	23255342	23355342	0.406846	CRADD
5	23255343	23355343	0.401876	CRADD
5	23255358	23355358	0.40738	CRADD
5	23255526	23355526	0.405455	CRADD
5	23255686	23355686	0.401275	CRADD
5	23255739	23355739	0.396717	CRADD
5	23255929	23355929	0.395033	CRADD
5	23256025	23356025	0.39748	CRADD
5	23256219	23356219	0.39748	CRADD
5	23256333	23356333	0.39748	CRADD
5	23256752	23356752	0.39748	CRADD
5	23256769	23356769	0.39748	CRADD
5	23256907	23356907	0.39748	CRADD
5	23256965	23356965	0.400818	CRADD
5	23256990	23356990	0.39749	CRADD
5	23257019	23357019	0.39749	CRADD
5	23257075	23357075	0.399233	CRADD
5	23257148	23357148	0.399403	CRADD
5	23257155	23357155	0.399403	CRADD
5	23257157	23357157	0.40978	CRADD
5	23257228	23357228	0.400515	CRADD
5	23257785	23357785	0.406337	CRADD
5	23257865	23357865	0.398986	CRADD
5	23257938	23357938	0.39545	CRADD
5	23257947	23357947	0.39545	CRADD
5	23258256	23358256	0.390276	CRADD
5	23258369	23358369	0.39678	CRADD
5	23258455	23358455	0.399055	CRADD
5	23258463	23358463	0.398262	CRADD
5	23258555	23358555	0.423157	CRADD
5	23258652	23358652	0.397474	CRADD
5	23258798	23358798	0.382578	CRADD
5	23258968	23358968	0.382578	CRADD
5	23258992	23358992	0.382578	CRADD
5	23259270	23359270	0.382578	CRADD
5	23259671	23359671	0.382578	CRADD
5	23259788	23359788	0.379745	CRADD
5	23260066	23360066	0.366619	CRADD
5	23260067	23360067	0.366619	CRADD
5	23260120	23360120	0.372783	CRADD
5	23260463	23360463	0.369598	CRADD
5	23260573	23360573	0.369598	CRADD
5	23260628	23360628	0.375474	CRADD
5	23260629	23360629	0.40146	CRADD
5	23260644	23360644	0.357312	CRADD
5	23261170	23361170	0.366354	CRADD
5	23261330	23361330	0.391133	CRADD
5	23261357	23361357	0.38994	CRADD
5	23261364	23361364	0.38994	CRADD
5	23261387	23361387	0.389912	CRADD
5	23261460	23361460	0.392255	CRADD
5	23261629	23361629	0.390318	CRADD
5	23261636	23361636	0.392502	CRADD
5	23261677	23361677	0.396448	CRADD
5	23261732	23361732	0.394963	CRADD
5	23261738	23361738	0.393542	CRADD

5	23261794	23361794	0.38993	CRADD
5	23261880	23361880	0.38993	CRADD
5	23262067	23362067	0.390615	CRADD
5	23262069	23362069	0.391355	CRADD
5	23262082	23362082	0.391355	CRADD
5	23262158	23362158	0.391355	CRADD
5	23262219	23362219	0.38385	CRADD
5	23262318	23362318	0.396512	CRADD
5	23262330	23362330	0.397609	CRADD
5	23262378	23362378	0.394163	CRADD
5	23262402	23362402	0.400076	CRADD
5	23262408	23362408	0.39918	CRADD
5	23262599	23362599	0.401681	CRADD
5	23262661	23362661	0.403789	CRADD
5	23262723	23362723	0.402574	CRADD
5	23262745	23362745	0.397832	CRADD
5	23262827	23362827	0.391807	CRADD
5	23262884	23362884	0.395705	CRADD
5	23263281	23363281	0.387564	CRADD
5	23263485	23363485	0.38912	CRADD
5	23263527	23363527	0.38961	CRADD
5	23263919	23363919	0.394642	CRADD
5	23264253	23364253	0.378438	CRADD
5	23264426	23364426	0.381315	CRADD
5	23264522	23364522	0.379768	CRADD
5	23264618	23364618	0.379287	CRADD
5	23264743	23364743	0.379967	CRADD
5	23264786	23364786	0.375787	CRADD
5	23264816	23364816	0.369955	CRADD
5	23265243	23365243	0.378676	CRADD
5	23265319	23365319	0.378264	CRADD
5	23265455	23365455	0.407963	CRADD
5	23265614	23365614	0.381641	CRADD
5	23265780	23365780	0.381832	CRADD
5	23265798	23365798	0.383741	CRADD
5	23265821	23365821	0.384252	CRADD
5	23265976	23365976	0.384698	CRADD
5	23265997	23365997	0.387405	CRADD
5	23266072	23366072	0.381675	CRADD
5	23266382	23366382	0.381653	CRADD
5	23266438	23366438	0.382805	CRADD
5	23266535	23366535	0.398574	CRADD
5	23266738	23366738	0.388064	CRADD
5	23266856	23366856	0.388716	CRADD
5	23266991	23366991	0.38882	CRADD
5	23266994	23366994	0.395126	CRADD
5	23267043	23367043	0.375632	CRADD
5	23267147	23367147	0.373981	CRADD
5	23267226	23367226	0.370693	CRADD
5	23267579	23367579	0.370693	CRADD
5	23267689	23367689	0.374682	CRADD
5	23267720	23367720	0.374682	CRADD
5	23267937	23367937	0.372857	CRADD
5	23267995	23367995	0.388174	CRADD
5	23268064	23368064	0.387129	CRADD
5	23268066	23368066	0.388719	CRADD
5	23268122	23368122	0.387213	CRADD
5	23268140	23368140	0.387371	CRADD

5	23268451	23368451	0.40188	CRADD
5	23268503	23368503	0.384055	CRADD
5	23268525	23368525	0.384622	CRADD
5	23268729	23368729	0.386519	CRADD
5	23268763	23368763	0.382217	CRADD
5	23268770	23368770	0.382612	CRADD
5	23269112	23369112	0.375619	CRADD
5	23269181	23369181	0.376301	CRADD
5	23269230	23369230	0.387634	CRADD
5	23269337	23369337	0.380226	CRADD
5	23269358	23369358	0.379579	CRADD
5	23269360	23369360	0.379579	CRADD
5	23269392	23369392	0.379579	CRADD
5	23269461	23369461	0.383364	CRADD
5	23269464	23369464	0.38281	CRADD
5	23269540	23369540	0.369116	CRADD
5	23269743	23369743	0.355222	CRADD
5	23269832	23369832	0.356656	CRADD
5	23270266	23370266	0.356656	CRADD
5	23270447	23370447	0.374377	CRADD
5	23270506	23370506	0.384369	CRADD
5	23270548	23370548	0.388426	CRADD
5	23270555	23370555	0.378787	CRADD
5	23270636	23370636	0.378787	CRADD
5	23270643	23370643	0.378787	CRADD
5	23270684	23370684	0.37579	CRADD
5	23270795	23370795	0.392949	CRADD
5	23270853	23370853	0.386849	CRADD
5	23270873	23370873	0.390203	CRADD
5	23270877	23370877	0.389075	CRADD
5	23270906	23370906	0.390529	CRADD
5	23270921	23370921	0.377163	CRADD
5	23270927	23370927	0.381628	CRADD
5	23271079	23371079	0.386693	CRADD
5	23271123	23371123	0.35366	CRADD
5	23271212	23371212	0.41871	CRADD
5	23271292	23371292	0.430944	CRADD
5	23271623	23371623	0.373327	CRADD
5	23271782	23371782	0.367442	CRADD
5	23271888	23371888	0.408984	CRADD
5	23272172	23372172	0.429118	CRADD
5	23273303	23373303	0.431418	CRADD
5	23273362	23373362	0.431418	CRADD
5	23273381	23373381	0.444366	CRADD
5	23273478	23373478	0.457752	CRADD
5	23273496	23373496	0.461373	CRADD
5	23273517	23373517	0.449724	CRADD
5	23273575	23373575	0.446869	CRADD
5	23273951	23373951	0.44184	CRADD
5	23274337	23374337	0.441511	CRADD
5	23274846	23374846	0.445525	CRADD
5	23275425	23375425	0.454831	CRADD
5	23275740	23375740	0.465563	CRADD
5	23276328	23376328	0.465563	CRADD
5	23276518	23376518	0.460107	CRADD
5	23276585	23376585	0.472446	CRADD
5	23276915	23376915	0.469144	CRADD
5	23277109	23377109	0.472268	CRADD

5	23277127	23377127	0.47793	CRADD
5	23277155	23377155	0.47779	CRADD
5	23277471	23377471	0.472588	CRADD
5	23277703	23377703	0.46958	CRADD
5	23277893	23377893	0.470799	CRADD
5	23277930	23377930	0.475779	CRADD
5	23278005	23378005	0.479838	CRADD
5	23278012	23378012	0.483022	CRADD
5	23278023	23378023	0.483022	CRADD
5	23278024	23378024	0.483022	CRADD
5	23278030	23378030	0.481182	CRADD
5	23278061	23378061	0.481182	CRADD
5	23278136	23378136	0.482221	CRADD
5	23278244	23378244	0.465312	CRADD
5	23278402	23378402	0.466602	CRADD
5	23278527	23378527	0.452334	CRADD
5	23278537	23378537	0.452334	CRADD
5	23278571	23378571	0.473988	CRADD
5	23278572	23378572	0.470891	CRADD
5	23278866	23378866	0.491006	CRADD
5	23278868	23378868	0.493729	CRADD
5	23278917	23378917	0.494676	CRADD
5	23278948	23378948	0.488108	CRADD
5	23279044	23379044	0.475706	CRADD
5	23279061	23379061	0.475863	CRADD
5	23279221	23379221	0.475569	CRADD
5	23279343	23379343	0.475569	CRADD
5	23279882	23379882	0.475569	CRADD
5	23279918	23379918	0.475569	CRADD
5	23279945	23379945	0.481715	CRADD
5	23280046	23380046	0.477047	CRADD
5	23280051	23380051	0.482523	CRADD
5	23280156	23380156	0.488197	CRADD
5	23280184	23380184	0.469302	CRADD
5	23280240	23380240	0.469302	CRADD
5	23280328	23380328	0.469832	CRADD
5	23280364	23380364	0.465883	CRADD
5	23280771	23380771	0.465471	CRADD
5	23280815	23380815	0.460358	CRADD
5	23280845	23380845	0.460358	CRADD
5	23281838	23381838	0.460367	CRADD
5	23281869	23381869	0.465908	CRADD
5	23282030	23382030	0.461407	CRADD
5	23282194	23382194	0.457586	CRADD
5	23282225	23382225	0.460152	CRADD
5	23282322	23382322	0.448185	CRADD
5	23282365	23382365	0.469906	CRADD
5	23282378	23382378	0.464359	CRADD
5	23282379	23382379	0.453431	CRADD
5	23282401	23382401	0.453431	CRADD
5	23282404	23382404	0.453431	CRADD
5	23282783	23382783	0.464291	CRADD
5	23282880	23382880	0.460962	CRADD
5	23282964	23382964	0.461074	CRADD
5	23283012	23383012	0.461074	CRADD
5	23283043	23383043	0.461074	CRADD
5	23283090	23383090	0.461074	CRADD
5	23283100	23383100	0.461074	CRADD

5	23283113	23383113	0.461074	CRADD
5	23283326	23383326	0.461074	CRADD
5	23283330	23383330	0.464327	CRADD
5	23283363	23383363	0.466796	CRADD
5	23283686	23383686	0.464917	CRADD
5	23283764	23383764	0.464917	CRADD
5	23283829	23383829	0.465044	CRADD
5	23283925	23383925	0.465981	CRADD
5	23283950	23383950	0.475002	CRADD
5	23283959	23383959	0.45216	CRADD
5	23283974	23383974	0.45216	CRADD
5	23283986	23383986	0.45216	CRADD
5	23284051	23384051	0.463272	CRADD
5	23284061	23384061	0.463272	CRADD
5	23284131	23384131	0.463272	CRADD
5	23284184	23384184	0.463272	CRADD
5	23284214	23384214	0.463356	CRADD
5	23284266	23384266	0.462965	CRADD
5	23285011	23385011	0.465499	CRADD
5	23285106	23385106	0.464849	CRADD
5	23285115	23385115	0.463943	CRADD
5	23285150	23385150	0.45682	CRADD
5	23285205	23385205	0.456937	CRADD
5	23285428	23385428	0.456931	CRADD
5	23285438	23385438	0.456931	CRADD
5	23285721	23385721	0.456796	CRADD
5	23286056	23386056	0.456935	CRADD
5	23286060	23386060	0.463738	CRADD
5	23286243	23386243	0.467349	CRADD
5	23286310	23386310	0.441583	CRADD
5	23286342	23386342	0.435675	CRADD
5	23286533	23386533	0.435675	CRADD
5	23286664	23386664	0.442072	CRADD
5	23286761	23386761	0.445711	CRADD
5	23286836	23386836	0.446381	CRADD
5	23286844	23386844	0.445808	CRADD
5	23286872	23386872	0.445419	CRADD
5	23286879	23386879	0.444215	CRADD
5	23286967	23386967	0.459977	CRADD
5	23286977	23386977	0.446791	CRADD
5	23287299	23387299	0.396679	CRADD
5	23287355	23387355	0.396199	CRADD
5	23287386	23387386	0.396128	CRADD
5	23287555	23387555	0.39824	CRADD
5	23287609	23387609	0.398615	CRADD
5	23287614	23387614	0.403858	CRADD
5	23287615	23387615	0.40156	CRADD
5	23287624	23387624	0.398458	CRADD
5	23287636	23387636	0.39639	CRADD
5	23287973	23387973	0.39157	CRADD
5	23288332	23388332	0.398245	CRADD
5	23288477	23388477	0.396694	CRADD
5	23288492	23388492	0.400221	CRADD
5	23288694	23388694	0.398006	CRADD
5	23288770	23388770	0.393382	CRADD
5	23289028	23389028	0.391165	CRADD
5	23289416	23389416	0.392813	CRADD
5	23289430	23389430	0.406212	CRADD

5	23289438	23389438	0.40089	CRADD
5	23289590	23389590	0.38922	CRADD
5	23289767	23389767	0.392482	CRADD
5	23289818	23389818	0.385806	CRADD
5	23289956	23389956	0.385806	CRADD
5	23290120	23390120	0.38259	CRADD
5	23290125	23390125	0.38259	CRADD
5	23290145	23390145	0.368722	CRADD
5	23290733	23390733	0.356866	CRADD
5	23290868	23390868	0.361381	CRADD
5	23290933	23390933	0.366527	CRADD
5	23291071	23391071	0.353563	CRADD
5	23291618	23391618	0.352412	CRADD
5	23291669	23391669	0.351447	CRADD
5	23291726	23391726	0.355649	CRADD
5	23291904	23391904	0.355649	CRADD
5	23291913	23391913	0.356	CRADD
5	23292001	23392001	0.351473	CRADD
5	23292351	23392351	0.351473	CRADD
5	23292462	23392462	0.351473	CRADD
5	23292543	23392543	0.351473	CRADD
5	23292557	23392557	0.351473	CRADD
5	23292668	23392668	0.351473	CRADD
5	23292773	23392773	0.351473	CRADD
5	23292795	23392795	0.351473	CRADD
5	23292807	23392807	0.351473	CRADD
5	23293073	23393073	0.353368	CRADD
5	23293109	23393109	0.357645	CRADD
5	23293133	23393133	0.354348	CRADD
5	23293653	23393653	0.354024	CRADD
5	23293671	23393671	0.354024	CRADD
5	23293687	23393687	0.354024	CRADD
5	23293741	23393741	0.354024	CRADD
5	23294589	23394589	0.347543	CRADD
5	23294887	23394887	0.355943	CRADD
5	23294983	23394983	0.345133	CRADD
5	23295123	23395123	0.3544	CRADD
5	23295277	23395277	0.352758	CRADD
5	23295368	23395368	0.352665	CRADD
5	23295399	23395399	0.354425	CRADD
5	23295453	23395453	0.350083	CRADD
5	23295496	23395496	0.358897	CRADD
5	23295566	23395566	0.358897	CRADD
5	23295610	23395610	0.358723	CRADD
5	23295611	23395611	0.358723	CRADD
5	23295634	23395634	0.359621	CRADD
5	23295914	23395914	0.352244	CRADD
5	23296306	23396306	0.352244	CRADD
5	23296404	23396404	0.360015	CRADD
5	23296684	23396684	0.35076	CRADD
5	23296734	23396734	0.35076	CRADD
5	23296782	23396782	0.35076	CRADD
5	23296802	23396802	0.35076	CRADD
5	23297043	23397043	0.35022	CRADD
5	23297124	23397124	0.35022	CRADD
5	23297157	23397157	0.350473	CRADD
5	23297280	23397280	0.350473	CRADD
5	23297367	23397367	0.352968	CRADD

5	23297370	23397370	0.352968	CRADD
5	23297450	23397450	0.351575	CRADD
5	23297526	23397526	0.351575	CRADD
5	23297551	23397551	0.351575	CRADD
5	23297572	23397572	0.351575	CRADD
5	23297841	23397841	0.351575	CRADD
5	23297980	23397980	0.351575	CRADD
5	23298006	23398006	0.351575	CRADD
5	23298103	23398103	0.351575	CRADD
5	23298158	23398158	0.351575	CRADD
5	23298238	23398238	0.351575	CRADD
5	23298322	23398322	0.351863	CRADD
5	23298325	23398325	0.351863	CRADD
5	23298341	23398341	0.349573	CRADD
5	23298383	23398383	0.356163	CRADD
5	23298573	23398573	0.35441	CRADD
5	23298786	23398786	0.360206	CRADD
5	23298875	23398875	0.357802	CRADD
5	23299273	23399273	0.352778	CRADD
5	23299377	23399377	0.351307	CRADD
5	23299404	23399404	0.348323	CRADD
5	23299663	23399663	0.348323	CRADD
5	23300079	23400079	0.348398	CRADD
5	23300136	23400136	0.358208	CRADD
5	23300251	23400251	0.345341	CRADD
5	23300407	23400407	0.345818	CRADD
5	23300511	23400511	0.345818	CRADD
5	23300551	23400551	0.348917	CRADD
5	23300980	23400980	0.362577	CRADD
5	23301019	23401019	0.359429	CRADD
5	23301163	23401163	0.359429	CRADD
5	23301322	23401322	0.360746	CRADD
5	23301367	23401367	0.35037	CRADD
5	23301387	23401387	0.352109	CRADD
5	23302029	23402029	0.342845	CRADD
5	23302030	23402030	0.342845	CRADD
5	23302100	23402100	0.342777	CRADD
5	23302110	23402110	0.342777	CRADD
5	23302112	23402112	0.342777	CRADD
5	23303229	23403229	0.343295	CRADD
5	23303487	23403487	0.358815	CRADD
5	23303684	23403684	0.344696	CRADD
5	23304083	23404083	0.356283	CRADD
5	23304133	23404133	0.356283	CRADD
5	23304231	23404231	0.356283	CRADD
5	23304247	23404247	0.356283	CRADD
5	23304254	23404254	0.356283	CRADD
5	23304742	23404742	0.356283	CRADD
5	23304766	23404766	0.362058	CRADD
5	23304797	23404797	0.372184	CRADD
5	23305081	23405081	0.375451	CRADD
5	23305102	23405102	0.375451	CRADD
5	23305123	23405123	0.374319	CRADD
5	23305155	23405155	0.374319	CRADD
5	23305221	23405221	0.376823	CRADD
5	23305236	23405236	0.376823	CRADD
5	23305247	23405247	0.371077	CRADD
5	23305334	23405334	0.373208	CRADD

5	23305371	23405371	0.373208	CRADD
5	23305408	23405408	0.373208	CRADD
5	23305451	23405451	0.373325	CRADD
5	23305464	23405464	0.372402	CRADD
5	23305476	23405476	0.383759	CRADD
5	23305486	23405486	0.383759	CRADD
5	23305528	23405528	0.383642	CRADD
5	23306034	23406034	0.389788	CRADD
5	23306143	23406143	0.390173	CRADD
5	23306431	23406431	0.390173	CRADD
5	23306482	23406482	0.396691	CRADD
5	23306486	23406486	0.385305	CRADD
5	23306576	23406576	0.384453	CRADD
5	23306609	23406609	0.384453	CRADD
5	23306992	23406992	0.384168	CRADD
5	23307075	23407075	0.384168	CRADD
5	23307116	23407116	0.385	CRADD
5	23307274	23407274	0.384792	CRADD
5	23307290	23407290	0.384792	CRADD
5	23307346	23407346	0.384792	CRADD
5	23307350	23407350	0.385105	CRADD
5	23307360	23407360	0.385105	CRADD
5	23307619	23407619	0.382149	CRADD
5	23307986	23407986	0.38007	CRADD
5	23308050	23408050	0.38007	CRADD
5	23308555	23408555	0.38007	CRADD
5	23308731	23408731	0.358706	CRADD
5	23309128	23409128	0.359004	CRADD
5	23309688	23409688	0.359596	CRADD
5	23309692	23409692	0.359596	CRADD
5	23309852	23409852	0.367015	CRADD
5	23310021	23410021	0.384562	CRADD
5	23310640	23410640	0.38347	CRADD
5	23310843	23410843	0.371144	CRADD
5	23311188	23411188	0.388235	CRADD
5	23311660	23411660	0.388235	CRADD
5	23311710	23411710	0.388235	CRADD
5	23311942	23411942	0.39387	CRADD
5	23312352	23412352	0.393547	CRADD
5	23312532	23412532	0.393547	CRADD
5	23312694	23412694	0.399492	CRADD
5	23313071	23413071	0.395552	CRADD
5	23313207	23413207	0.360655	CRADD
5	23313383	23413383	0.393488	CRADD
5	23313392	23413392	0.393488	CRADD
5	23313528	23413528	0.393488	CRADD
5	23313613	23413613	0.393488	CRADD
5	23313984	23413984	0.387008	CRADD
5	23314022	23414022	0.38824	CRADD
5	23314104	23414104	0.389158	CRADD
5	23314158	23414158	0.388887	CRADD
5	23314215	23414215	0.408011	CRADD
5	23314257	23414257	0.352296	CRADD
5	23314286	23414286	0.344914	CRADD
5	23314336	23414336	0.343509	CRADD
5	23314340	23414340	0.343509	CRADD
5	23314413	23414413	0.343479	CRADD
5	23314423	23414423	0.343479	CRADD

5	23314439	23414439	0.343479	CRADD
5	23314473	23414473	0.343479	CRADD
5	23314477	23414477	0.343479	CRADD
5	23314492	23414492	0.343479	CRADD
5	23314493	23414493	0.343479	CRADD
5	23314585	23414585	0.343479	CRADD
5	23314615	23414615	0.343479	CRADD
5	23314652	23414652	0.343479	CRADD
5	23314655	23414655	0.343479	CRADD
5	23314671	23414671	0.343479	CRADD
5	23314686	23414686	0.343479	CRADD
5	23314967	23414967	0.348122	CRADD
5	23315010	23415010	0.344431	CRADD
5	23315011	23415011	0.344431	CRADD
5	23315016	23415016	0.344431	CRADD
5	23315024	23415024	0.344431	CRADD
5	23315031	23415031	0.344431	CRADD
5	23315087	23415087	0.359201	CRADD
5	23315129	23415129	0.342251	CRADD
5	23368004	23468004	0.343632	CRADD
5	23368004	23468004	0.343632	LOC106502085
5	23368035	23468035	0.359149	CRADD
5	23368035	23468035	0.359149	LOC106502085
5	23368069	23468069	0.39249	CRADD
5	23368069	23468069	0.39249	LOC106502085
5	23368084	23468084	0.353702	CRADD
5	23368084	23468084	0.353702	LOC106502085
5	23368093	23468093	0.350558	CRADD
5	23368093	23468093	0.350558	LOC106502085
5	23368151	23468151	0.38448	CRADD
5	23368151	23468151	0.38448	LOC106502085
5	23368239	23468239	0.461336	CRADD
5	23368239	23468239	0.461336	LOC106502085
5	23368438	23468438	0.45473	CRADD
5	23368438	23468438	0.45473	LOC106502085
5	23368453	23468453	0.441437	CRADD
5	23368453	23468453	0.441437	LOC106502085
5	23368583	23468583	0.428057	CRADD
5	23368583	23468583	0.428057	LOC106502085
5	23368775	23468775	0.425127	CRADD
5	23368775	23468775	0.425127	LOC106502085
5	23368937	23468937	0.42677	CRADD
5	23368937	23468937	0.42677	LOC106502085
5	23369016	23469016	0.426192	CRADD
5	23369016	23469016	0.426192	LOC106502085
5	23369054	23469054	0.427304	CRADD
5	23369054	23469054	0.427304	LOC106502085
5	23369055	23469055	0.427304	CRADD
5	23369055	23469055	0.427304	LOC106502085
5	23369127	23469127	0.427015	CRADD
5	23369127	23469127	0.427015	LOC106502085
5	23369246	23469246	0.426093	CRADD
5	23369246	23469246	0.426093	LOC106502085
5	23369426	23469426	0.431868	CRADD
5	23369426	23469426	0.431868	LOC106502085
5	23369433	23469433	0.431589	CRADD
5	23369433	23469433	0.431589	LOC106502085
5	23369439	23469439	0.454238	CRADD

5	23369439	23469439	0.454238	LOC106502085
5	23369491	23469491	0.403956	CRADD
5	23369491	23469491	0.403956	LOC106502085
5	23369549	23469549	0.404025	CRADD
5	23369549	23469549	0.404025	LOC106502085
5	23369558	23469558	0.400216	CRADD
5	23369558	23469558	0.400216	LOC106502085
5	23369583	23469583	0.400216	CRADD
5	23369583	23469583	0.400216	LOC106502085
5	23369626	23469626	0.400216	CRADD
5	23369626	23469626	0.400216	LOC106502085
5	23369627	23469627	0.400481	CRADD
5	23369627	23469627	0.400481	LOC106502085
5	23369865	23469865	0.399459	CRADD
5	23369865	23469865	0.399459	LOC106502085
5	23369883	23469883	0.399459	CRADD
5	23369883	23469883	0.399459	LOC106502085
5	23369892	23469892	0.399459	CRADD
5	23369892	23469892	0.399459	LOC106502085
5	23369933	23469933	0.404431	CRADD
5	23369933	23469933	0.404431	LOC106502085
5	23369939	23469939	0.391284	CRADD
5	23369939	23469939	0.391284	LOC106502085
5	23370063	23470063	0.393219	CRADD
5	23370063	23470063	0.393219	LOC106502085
5	23370100	23470100	0.396009	CRADD
5	23370100	23470100	0.396009	LOC106502085
5	23370103	23470103	0.390285	CRADD
5	23370103	23470103	0.390285	LOC106502085
5	23370170	23470170	0.388484	CRADD
5	23370170	23470170	0.388484	LOC106502085
5	23370191	23470191	0.388484	CRADD
5	23370191	23470191	0.388484	LOC106502085
5	23370214	23470214	0.370293	CRADD
5	23370214	23470214	0.370293	LOC106502085
5	23370297	23470297	0.37916	CRADD
5	23370297	23470297	0.37916	LOC106502085
5	23370355	23470355	0.363093	CRADD
5	23370355	23470355	0.363093	LOC106502085
5	23370436	23470436	0.381096	CRADD
5	23370436	23470436	0.381096	LOC106502085
5	23370538	23470538	0.359085	CRADD
5	23370538	23470538	0.359085	LOC106502085
5	23370806	23470806	0.357727	CRADD
5	23370806	23470806	0.357727	LOC106502085
5	23371011	23471011	0.357072	CRADD
5	23371011	23471011	0.357072	LOC106502085
5	23371315	23471315	0.35768	CRADD
5	23371315	23471315	0.35768	LOC106502085
5	23371556	23471556	0.358872	CRADD
5	23371556	23471556	0.358872	LOC106502085
5	23371592	23471592	0.358512	CRADD
5	23371592	23471592	0.358512	LOC106502085
5	23371908	23471908	0.359139	CRADD
5	23371908	23471908	0.359139	LOC106502085
5	23372124	23472124	0.377378	CRADD
5	23372124	23472124	0.377378	LOC106502085
5	23372291	23472291	0.373169	CRADD

5	23372291	23472291	0.373169	LOC106502085
5	23372653	23472653	0.371436	CRADD
5	23372653	23472653	0.371436	LOC106502085
5	23372682	23472682	0.371436	CRADD
5	23372682	23472682	0.371436	LOC106502085
5	23372890	23472890	0.404616	CRADD
5	23372890	23472890	0.404616	LOC106502085
5	23373309	23473309	0.411184	CRADD
5	23373309	23473309	0.411184	LOC106502085
5	23373372	23473372	0.392051	CRADD
5	23373372	23473372	0.392051	LOC106502085
5	23373430	23473430	0.394914	CRADD
5	23373430	23473430	0.394914	LOC106502085
5	23373492	23473492	0.394914	CRADD
5	23373492	23473492	0.394914	LOC106502085
5	23373957	23473957	0.394914	CRADD
5	23373957	23473957	0.394914	LOC106502085
5	23374178	23474178	0.393752	CRADD
5	23374178	23474178	0.393752	LOC106502085
5	23374417	23474417	0.393752	CRADD
5	23374417	23474417	0.393752	LOC106502085
5	23374505	23474505	0.393752	CRADD
5	23374505	23474505	0.393752	LOC106502085
5	23374593	23474593	0.393752	CRADD
5	23374593	23474593	0.393752	LOC106502085
5	23374601	23474601	0.393752	CRADD
5	23374601	23474601	0.393752	LOC106502085
5	23374641	23474641	0.425079	CRADD
5	23374641	23474641	0.425079	LOC106502085
5	23374750	23474750	0.415519	CRADD
5	23374750	23474750	0.415519	LOC106502085
5	23374790	23474790	0.412342	CRADD
5	23374790	23474790	0.412342	LOC106502085
5	23374865	23474865	0.44321	CRADD
5	23374865	23474865	0.44321	LOC106502085
5	23374868	23474868	0.456096	CRADD
5	23374868	23474868	0.456096	LOC106502085
5	23374869	23474869	0.456096	CRADD
5	23374869	23474869	0.456096	LOC106502085
5	23374885	23474885	0.456096	CRADD
5	23374885	23474885	0.456096	LOC106502085
5	23374959	23474959	0.46473	CRADD
5	23374959	23474959	0.46473	LOC106502085
5	23374962	23474962	0.463907	CRADD
5	23374962	23474962	0.463907	LOC106502085
5	23375010	23475010	0.462343	CRADD
5	23375010	23475010	0.462343	LOC106502085
5	23375082	23475082	0.459645	CRADD
5	23375082	23475082	0.459645	LOC106502085
5	23375225	23475225	0.456155	CRADD
5	23375225	23475225	0.456155	LOC106502085
5	23375405	23475405	0.462424	CRADD
5	23375405	23475405	0.462424	LOC106502085
5	23375406	23475406	0.465916	CRADD
5	23375406	23475406	0.465916	LOC106502085
5	23375461	23475461	0.458767	CRADD
5	23375461	23475461	0.458767	LOC106502085
5	23375469	23475469	0.468997	CRADD

5	23375469	23475469	0.468997	LOC106502085
5	23375670	23475670	0.460329	CRADD
5	23375670	23475670	0.460329	LOC106502085
5	23375701	23475701	0.470734	CRADD
5	23375701	23475701	0.470734	LOC106502085
5	23375755	23475755	0.470734	CRADD
5	23375755	23475755	0.470734	LOC106502085
5	23375806	23475806	0.421966	CRADD
5	23375806	23475806	0.421966	LOC106502085
5	23375807	23475807	0.572079	CRADD
5	23375807	23475807	0.572079	LOC106502085
5	23375870	23475870	0.578754	CRADD
5	23375870	23475870	0.578754	LOC106502085
5	23375879	23475879	0.576042	CRADD
5	23375879	23475879	0.576042	LOC106502085
5	23375905	23475905	0.564191	CRADD
5	23375905	23475905	0.564191	LOC106502085
5	23376013	23476013	0.564191	CRADD
5	23376013	23476013	0.564191	LOC106502085
5	23376303	23476303	0.574707	CRADD
5	23376303	23476303	0.574707	LOC106502085
5	23376417	23476417	0.572296	CRADD
5	23376417	23476417	0.572296	LOC106502085
5	23376515	23476515	0.579973	CRADD
5	23376515	23476515	0.579973	LOC106502085
5	23376533	23476533	0.554158	CRADD
5	23376533	23476533	0.554158	LOC106502085
5	23376534	23476534	0.554158	CRADD
5	23376534	23476534	0.554158	LOC106502085
5	23376607	23476607	0.556508	CRADD
5	23376607	23476607	0.556508	LOC106502085
5	23376703	23476703	0.548716	CRADD
5	23376703	23476703	0.548716	LOC106502085
5	23376724	23476724	0.548716	CRADD
5	23376724	23476724	0.548716	LOC106502085
5	23376783	23476783	0.550095	CRADD
5	23376783	23476783	0.550095	LOC106502085
5	23376793	23476793	0.543837	CRADD
5	23376793	23476793	0.543837	LOC106502085
5	23376899	23476899	0.543982	CRADD
5	23376899	23476899	0.543982	LOC106502085
5	23376961	23476961	0.546204	CRADD
5	23376961	23476961	0.546204	LOC106502085
5	23377122	23477122	0.53611	CRADD
5	23377122	23477122	0.53611	LOC106502085
5	23377211	23477211	0.53611	CRADD
5	23377211	23477211	0.53611	LOC106502085
5	23377413	23477413	0.544337	CRADD
5	23377413	23477413	0.544337	LOC106502085
5	23377448	23477448	0.544337	CRADD
5	23377448	23477448	0.544337	LOC106502085
5	23377449	23477449	0.548213	CRADD
5	23377449	23477449	0.548213	LOC106502085
5	23377536	23477536	0.554862	CRADD
5	23377536	23477536	0.554862	LOC106502085
5	23377724	23477724	0.530659	CRADD
5	23377724	23477724	0.530659	LOC106502085
5	23377746	23477746	0.530659	CRADD

5	23377746	23477746	0.530659	LOC106502085
5	23377783	23477783	0.531758	CRADD
5	23377783	23477783	0.531758	LOC106502085
5	23377813	23477813	0.52864	CRADD
5	23377813	23477813	0.52864	LOC106502085
5	23377877	23477877	0.529767	CRADD
5	23377877	23477877	0.529767	LOC106502085
5	23378076	23478076	0.528999	CRADD
5	23378076	23478076	0.528999	LOC106502085
5	23378224	23478224	0.528999	CRADD
5	23378224	23478224	0.528999	LOC106502085
5	23378268	23478268	0.532454	CRADD
5	23378268	23478268	0.532454	LOC106502085
5	23378278	23478278	0.533382	CRADD
5	23378278	23478278	0.533382	LOC106502085
5	23378305	23478305	0.529678	CRADD
5	23378305	23478305	0.529678	LOC106502085
5	23378365	23478365	0.547116	CRADD
5	23378365	23478365	0.547116	LOC106502085
5	23378724	23478724	0.518419	CRADD
5	23378724	23478724	0.518419	LOC106502085
5	23378783	23478783	0.517252	CRADD
5	23378783	23478783	0.517252	LOC106502085
5	23379029	23479029	0.517252	CRADD
5	23379029	23479029	0.517252	LOC106502085
5	23379121	23479121	0.517252	CRADD
5	23379121	23479121	0.517252	LOC106502085
5	23379130	23479130	0.518748	CRADD
5	23379130	23479130	0.518748	LOC106502085
5	23379281	23479281	0.533062	CRADD
5	23379281	23479281	0.533062	LOC106502085
5	23379391	23479391	0.518494	CRADD
5	23379391	23479391	0.518494	LOC106502085
5	23379732	23479732	0.526761	CRADD
5	23379732	23479732	0.526761	LOC106502085
5	23379767	23479767	0.533979	CRADD
5	23379767	23479767	0.533979	LOC106502085
5	23379855	23479855	0.544867	CRADD
5	23379855	23479855	0.544867	LOC106502085
5	23380047	23480047	0.526819	CRADD
5	23380047	23480047	0.526819	LOC106502085
5	23380149	23480149	0.523114	CRADD
5	23380149	23480149	0.523114	LOC106502085
5	23380214	23480214	0.528018	CRADD
5	23380214	23480214	0.528018	LOC106502085
5	23380224	23480224	0.527817	CRADD
5	23380224	23480224	0.527817	LOC106502085
5	23380260	23480260	0.527118	CRADD
5	23380260	23480260	0.527118	LOC106502085
5	23380320	23480320	0.524186	CRADD
5	23380320	23480320	0.524186	LOC106502085
5	23380399	23480399	0.526351	CRADD
5	23380399	23480399	0.526351	LOC106502085
5	23380420	23480420	0.524076	CRADD
5	23380420	23480420	0.524076	LOC106502085
5	23380601	23480601	0.529514	CRADD
5	23380601	23480601	0.529514	LOC106502085
5	23380835	23480835	0.518612	CRADD

5	23380835	23480835	0.518612	LOC106502085
5	23381213	23481213	0.524206	CRADD
5	23381213	23481213	0.524206	LOC106502085
5	23381214	23481214	0.527578	CRADD
5	23381214	23481214	0.527578	LOC106502085
5	23381225	23481225	0.520939	CRADD
5	23381225	23481225	0.520939	LOC106502085
5	23381263	23481263	0.519175	CRADD
5	23381263	23481263	0.519175	LOC106502085
5	23381428	23481428	0.523479	CRADD
5	23381428	23481428	0.523479	LOC106502085
5	23381463	23481463	0.51977	CRADD
5	23381463	23481463	0.51977	LOC106502085
5	23381852	23481852	0.544605	CRADD
5	23381852	23481852	0.544605	LOC106502085
5	23381857	23481857	0.542718	CRADD
5	23381857	23481857	0.542718	LOC106502085
5	23381861	23481861	0.555225	CRADD
5	23381861	23481861	0.555225	LOC106502085
5	23381905	23481905	0.540659	CRADD
5	23381905	23481905	0.540659	LOC106502085
5	23381935	23481935	0.543117	CRADD
5	23381935	23481935	0.543117	LOC106502085
5	23381953	23481953	0.541733	CRADD
5	23381953	23481953	0.541733	LOC106502085
5	23382051	23482051	0.541182	CRADD
5	23382051	23482051	0.541182	LOC106502085
5	23382182	23482182	0.544694	CRADD
5	23382182	23482182	0.544694	LOC106502085
5	23382313	23482313	0.55792	CRADD
5	23382313	23482313	0.55792	LOC106502085
5	23382499	23482499	0.544432	CRADD
5	23382499	23482499	0.544432	LOC106502085
5	23382510	23482510	0.540489	CRADD
5	23382510	23482510	0.540489	LOC106502085
5	23382648	23482648	0.530021	CRADD
5	23382648	23482648	0.530021	LOC106502085
5	23382657	23482657	0.521873	CRADD
5	23382657	23482657	0.521873	LOC106502085
5	23382680	23482680	0.521873	CRADD
5	23382680	23482680	0.521873	LOC106502085
5	23382682	23482682	0.517151	CRADD
5	23382682	23482682	0.517151	LOC106502085
5	23383129	23483129	0.514688	CRADD
5	23383129	23483129	0.514688	LOC106502085
5	23383221	23483221	0.508707	CRADD
5	23383221	23483221	0.508707	LOC106502085
5	23383327	23483327	0.509404	CRADD
5	23383327	23483327	0.509404	LOC106502085
5	23383364	23483364	0.509404	CRADD
5	23383364	23483364	0.509404	LOC106502085
5	23383383	23483383	0.535267	CRADD
5	23383383	23483383	0.535267	LOC106502085
5	23383432	23483432	0.534492	CRADD
5	23383432	23483432	0.534492	LOC106502085
5	23383457	23483457	0.509042	CRADD
5	23383457	23483457	0.509042	LOC106502085
5	23383578	23483578	0.515844	CRADD

5	23383578	23483578	0.515844	LOC106502085
5	23384003	23484003	0.500752	CRADD
5	23384003	23484003	0.500752	LOC106502085
5	23384312	23484312	0.494709	CRADD
5	23384312	23484312	0.494709	LOC106502085
5	23384961	23484961	0.507448	CRADD
5	23384961	23484961	0.507448	LOC106502085
5	23385498	23485498	0.535613	CRADD
5	23385498	23485498	0.535613	LOC106502085
5	23385599	23485599	0.531038	CRADD
5	23385599	23485599	0.531038	LOC106502085
5	23385651	23485651	0.537659	CRADD
5	23385651	23485651	0.537659	LOC106502085
5	23385723	23485723	0.537814	CRADD
5	23385723	23485723	0.537814	LOC106502085
5	23385926	23485926	0.541405	CRADD
5	23385926	23485926	0.541405	LOC106502085
5	23386061	23486061	0.550172	CRADD
5	23386061	23486061	0.550172	LOC106502085
5	23386101	23486101	0.568719	CRADD
5	23386101	23486101	0.568719	LOC106502085
5	23386110	23486110	0.551335	CRADD
5	23386110	23486110	0.551335	LOC106502085
5	23386152	23486152	0.550512	CRADD
5	23386152	23486152	0.550512	LOC106502085
5	23386320	23486320	0.547817	CRADD
5	23386320	23486320	0.547817	LOC106502085
5	23386332	23486332	0.547817	CRADD
5	23386332	23486332	0.547817	LOC106502085
5	23386349	23486349	0.547817	CRADD
5	23386349	23486349	0.547817	LOC106502085
5	23386472	23486472	0.545252	CRADD
5	23386472	23486472	0.545252	LOC106502085
5	23386684	23486684	0.521972	CRADD
5	23386684	23486684	0.521972	LOC106502085
5	23386903	23486903	0.521972	CRADD
5	23386903	23486903	0.521972	LOC106502085
5	23387287	23487287	0.526195	CRADD
5	23387287	23487287	0.526195	LOC106502085
5	23387567	23487567	0.537194	CRADD
5	23387567	23487567	0.537194	LOC106502085
5	23387597	23487597	0.520406	CRADD
5	23387597	23487597	0.520406	LOC106502085
5	23387625	23487625	0.512123	CRADD
5	23387625	23487625	0.512123	LOC106502085
5	23388569	23488569	0.492461	CRADD
5	23388569	23488569	0.492461	LOC106502085
5	23388815	23488815	0.495538	CRADD
5	23388815	23488815	0.495538	LOC106502085
5	23388879	23488879	0.497452	CRADD
5	23388879	23488879	0.497452	LOC106502085
5	23389020	23489020	0.497452	CRADD
5	23389020	23489020	0.497452	LOC106502085
5	23389380	23489380	0.496393	CRADD
5	23389380	23489380	0.496393	LOC106502085
5	23389534	23489534	0.50367	CRADD
5	23389534	23489534	0.50367	LOC106502085
5	23389714	23489714	0.515817	CRADD

5	23389714	23489714	0.515817	LOC106502085
5	23389886	23489886	0.50363	CRADD
5	23389886	23489886	0.50363	LOC106502085
5	23389995	23489995	0.513364	CRADD
5	23389995	23489995	0.513364	LOC106502085
5	23390516	23490516	0.491233	CRADD
5	23390516	23490516	0.491233	LOC106502085
5	23390643	23490643	0.508807	CRADD
5	23390643	23490643	0.508807	LOC106502085
5	23390819	23490819	0.470967	CRADD
5	23390819	23490819	0.470967	LOC106502085
5	23391056	23491056	0.473142	CRADD
5	23391056	23491056	0.473142	LOC106502085
5	23391353	23491353	0.484482	CRADD
5	23391353	23491353	0.484482	LOC106502085
5	23391507	23491507	0.487678	CRADD
5	23391507	23491507	0.487678	LOC106502085
5	23391884	23491884	0.484883	CRADD
5	23391884	23491884	0.484883	LOC106502085
5	23392620	23492620	0.492069	CRADD
5	23392620	23492620	0.492069	LOC106502085
5	23392657	23492657	0.488928	CRADD
5	23392657	23492657	0.488928	LOC106502085
5	23392809	23492809	0.483848	CRADD
5	23392809	23492809	0.483848	LOC106502085
5	23393439	23493439	0.503863	CRADD
5	23393439	23493439	0.503863	LOC106502085
5	23393466	23493466	0.508671	CRADD
5	23393466	23493466	0.508671	LOC106502085
5	23393527	23493527	0.515981	CRADD
5	23393527	23493527	0.515981	LOC106502085
5	23393755	23493755	0.508799	CRADD
5	23393755	23493755	0.508799	LOC106502085
5	23393781	23493781	0.506231	CRADD
5	23393781	23493781	0.506231	LOC106502085
5	23393989	23493989	0.473621	CRADD
5	23393989	23493989	0.473621	LOC106502085
5	23394092	23494092	0.473621	CRADD
5	23394092	23494092	0.473621	LOC106502085
5	23394484	23494484	0.478963	CRADD
5	23394484	23494484	0.478963	LOC106502085
5	23395221	23495221	0.442306	CRADD
5	23395221	23495221	0.442306	LOC106502085
5	23395448	23495448	0.468633	CRADD
5	23395448	23495448	0.468633	LOC106502085
5	23395461	23495461	0.452134	CRADD
5	23395461	23495461	0.452134	LOC106502085
5	23395538	23495538	0.4462	CRADD
5	23395538	23495538	0.4462	LOC106502085
5	23396410	23496410	0.4462	CRADD
5	23396410	23496410	0.4462	LOC106502085
5	23396465	23496465	0.468986	CRADD
5	23396465	23496465	0.468986	LOC106502085
5	23396858	23496858	0.459442	CRADD
5	23396858	23496858	0.459442	LOC106502085
5	23396956	23496956	0.478018	CRADD
5	23396956	23496956	0.478018	LOC106502085
5	23397093	23497093	0.494891	CRADD

5	23397093	23497093	0.494891	LOC106502085
5	23397200	23497200	0.512846	CRADD
5	23397200	23497200	0.512846	LOC106502085
5	23397201	23497201	0.501849	CRADD
5	23397201	23497201	0.501849	LOC106502085
5	23397246	23497246	0.501849	CRADD
5	23397246	23497246	0.501849	LOC106502085
5	23397262	23497262	0.502546	CRADD
5	23397262	23497262	0.502546	LOC106502085
5	23397444	23497444	0.497864	CRADD
5	23397444	23497444	0.497864	LOC106502085
5	23397562	23497562	0.497864	CRADD
5	23397562	23497562	0.497864	LOC106502085
5	23397615	23497615	0.503362	CRADD
5	23397615	23497615	0.503362	LOC106502085
5	23397723	23497723	0.508928	CRADD
5	23397723	23497723	0.508928	LOC106502085
5	23397845	23497845	0.472691	CRADD
5	23397845	23497845	0.472691	LOC106502085
5	23397907	23497907	0.472691	CRADD
5	23397907	23497907	0.472691	LOC106502085
5	23397914	23497914	0.472691	CRADD
5	23397914	23497914	0.472691	LOC106502085
5	23397995	23497995	0.472691	CRADD
5	23397995	23497995	0.472691	LOC106502085
5	23398004	23498004	0.472691	CRADD
5	23398004	23498004	0.472691	LOC106502085
5	23398362	23498362	0.478248	CRADD
5	23398362	23498362	0.478248	LOC106502085
5	23398507	23498507	0.473356	CRADD
5	23398507	23498507	0.473356	LOC106502085
5	23398514	23498514	0.473356	CRADD
5	23398514	23498514	0.473356	LOC106502085
5	23398523	23498523	0.473356	CRADD
5	23398523	23498523	0.473356	LOC106502085
5	23398604	23498604	0.469672	CRADD
5	23398604	23498604	0.469672	LOC106502085
5	23398921	23498921	0.46607	CRADD
5	23398921	23498921	0.46607	LOC106502085
5	23399338	23499338	0.456598	CRADD
5	23399338	23499338	0.456598	LOC106502085
5	23400156	23500156	0.392692	CRADD
5	23400156	23500156	0.392692	LOC106502085
5	23400433	23500433	0.390558	CRADD
5	23400433	23500433	0.390558	LOC106502085
5	23400670	23500670	0.390558	CRADD
5	23400670	23500670	0.390558	LOC106502085
5	23400848	23500848	0.365328	CRADD
5	23400848	23500848	0.365328	LOC106502085
5	23401917	23501917	0.357722	CRADD
5	23401917	23501917	0.357722	LOC106502085
5	23402119	23502119	0.366849	CRADD
5	23402119	23502119	0.366849	LOC106502085
5	23402165	23502165	0.379768	CRADD
5	23402165	23502165	0.379768	LOC106502085
5	23402260	23502260	0.383389	CRADD
5	23402260	23502260	0.383389	LOC106502085
5	23402262	23502262	0.371315	CRADD

5	23402262	23502262	0.371315	LOC106502085
5	23402433	23502433	0.370433	CRADD
5	23402433	23502433	0.370433	LOC106502085
5	23402582	23502582	0.368416	CRADD
5	23402582	23502582	0.368416	LOC106502085
5	23402664	23502664	0.363449	CRADD
5	23402664	23502664	0.363449	LOC106502085
5	23402907	23502907	0.361928	CRADD
5	23402907	23502907	0.361928	LOC106502085
5	23403092	23503092	0.361928	CRADD
5	23403092	23503092	0.361928	LOC106502085
5	23403100	23503100	0.361928	CRADD
5	23403100	23503100	0.361928	LOC106502085
5	23403180	23503180	0.370472	CRADD
5	23403180	23503180	0.370472	LOC106502085
5	23403193	23503193	0.364946	CRADD
5	23403193	23503193	0.364946	LOC106502085
5	23403327	23503327	0.370251	CRADD
5	23403327	23503327	0.370251	LOC106502085
5	23403766	23503766	0.3672	CRADD
5	23403766	23503766	0.3672	LOC106502085
5	23403970	23503970	0.379675	CRADD
5	23403970	23503970	0.379675	LOC106502085
5	23404179	23504179	0.364058	CRADD
5	23404179	23504179	0.364058	LOC106502085
5	23404330	23504330	0.364058	CRADD
5	23404330	23504330	0.364058	LOC106502085
5	23404334	23504334	0.364058	CRADD
5	23404334	23504334	0.364058	LOC106502085
5	23404401	23504401	0.362673	CRADD
5	23404401	23504401	0.362673	LOC106502085
5	23954378	24054378	0.343839	CEP83
5	23954422	24054422	0.344697	CEP83
5	23954788	24054788	0.351133	CEP83
5	23954791	24054791	0.351133	CEP83
5	23954794	24054794	0.351133	CEP83
5	23955172	24055172	0.347984	CEP83
5	23955253	24055253	0.35305	CEP83
5	23955542	24055542	0.343212	CEP83
5	23955595	24055595	0.359202	CEP83
5	23955728	24055728	0.360745	CEP83
5	23956622	24056622	0.352261	CEP83
5	23956858	24056858	0.359552	CEP83
5	23956873	24056873	0.355082	CEP83
5	23956935	24056935	0.357052	CEP83
5	23956982	24056982	0.355517	CEP83
5	23957017	24057017	0.372464	CEP83
5	23957038	24057038	0.372464	CEP83
5	23957055	24057055	0.372464	CEP83
5	23957072	24057072	0.372464	CEP83
5	23957079	24057079	0.370616	CEP83
5	23957203	24057203	0.379912	CEP83
5	23957211	24057211	0.380314	CEP83
5	23957260	24057260	0.370448	CEP83
5	24136082	24236082	0.36144	TMCC3
5	24136249	24236249	0.344714	TMCC3
5	24136328	24236328	0.368142	TMCC3
5	24136723	24236723	0.360457	TMCC3

5	24136772	24236772	0.368434	TMCC3
5	24136776	24236776	0.35763	TMCC3
5	24136778	24236778	0.380201	TMCC3
5	24136966	24236966	0.404578	TMCC3
5	24136998	24236998	0.398511	TMCC3
5	24137054	24237054	0.423285	TMCC3
5	24137071	24237071	0.410409	TMCC3
5	24137092	24237092	0.410409	TMCC3
5	24137178	24237178	0.456794	TMCC3
5	24137197	24237197	0.459901	TMCC3
5	24137251	24237251	0.401621	TMCC3
5	24137460	24237460	0.41882	TMCC3
5	24137731	24237731	0.441209	TMCC3
5	24137732	24237732	0.441209	TMCC3
5	24137817	24237817	0.451113	TMCC3
5	24137847	24237847	0.445442	TMCC3
5	24137854	24237854	0.436001	TMCC3
5	24138008	24238008	0.396622	TMCC3
5	24138033	24238033	0.438351	TMCC3
5	24138049	24238049	0.430153	TMCC3
5	24138140	24238140	0.435216	TMCC3
5	24138178	24238178	0.355175	TMCC3
5	24138223	24238223	0.346221	TMCC3
5	24138534	24238534	0.348306	TMCC3
5	24138544	24238544	0.350205	TMCC3
5	24936338	25036338	0.351545	LOC102184404
5	24936734	25036734	0.356493	LOC102184404
5	24945551	25045551	0.368665	LOC102184404
5	24945551	25045551	0.368665	MUCL1
5	24946457	25046457	0.344331	LOC102184404
5	24946457	25046457	0.344331	MUCL1
5	24948455	25048455	0.343023	LOC102184404
5	24948455	25048455	0.343023	MUCL1
5	24948480	25048480	0.368816	LOC102184404
5	24948480	25048480	0.368816	MUCL1
5	24954939	25054939	0.405195	LOC102184404
5	24954939	25054939	0.405195	MUCL1
5	24954939	25054939	0.405195	LOC108636093
5	25147246	25247246	0.344585	GLYCAM1
5	25147246	25247246	0.344585	PPP1R1A
5	25147561	25247561	0.3508	GLYCAM1
5	25147561	25247561	0.3508	PPP1R1A
5	25147585	25247585	0.36211	GLYCAM1
5	25147585	25247585	0.36211	PPP1R1A
5	25147587	25247587	0.364764	GLYCAM1
5	25147587	25247587	0.364764	PPP1R1A
5	25149365	25249365	0.342323	GLYCAM1
5	25149365	25249365	0.342323	PPP1R1A
5	25149529	25249529	0.3431	GLYCAM1
5	25149529	25249529	0.3431	PPP1R1A
5	25149667	25249667	0.342687	GLYCAM1
5	25149667	25249667	0.342687	PPP1R1A
5	25149725	25249725	0.342687	GLYCAM1
5	25149725	25249725	0.342687	PPP1R1A
5	25149953	25249953	0.351677	GLYCAM1
5	25149953	25249953	0.351677	PPP1R1A
5	25149965	25249965	0.351881	GLYCAM1
5	25149965	25249965	0.351881	PPP1R1A

5	25150014	25250014	0.351745	GLYCAM1
5	25150014	25250014	0.351745	PPP1R1A
5	25150194	25250194	0.345476	GLYCAM1
5	25150194	25250194	0.345476	PPP1R1A
5	25150389	25250389	0.345476	GLYCAM1
5	25150389	25250389	0.345476	PPP1R1A
5	25150413	25250413	0.352777	GLYCAM1
5	25150413	25250413	0.352777	PPP1R1A
5	25150546	25250546	0.366682	GLYCAM1
5	25150546	25250546	0.366682	PPP1R1A
5	25150602	25250602	0.365645	GLYCAM1
5	25150602	25250602	0.365645	PPP1R1A
5	25150622	25250622	0.367224	GLYCAM1
5	25150622	25250622	0.367224	PPP1R1A
5	25150629	25250629	0.345036	GLYCAM1
5	25150629	25250629	0.345036	PPP1R1A
5	25151060	25251060	0.374905	GLYCAM1
5	25151060	25251060	0.374905	PPP1R1A
5	25151095	25251095	0.374905	GLYCAM1
5	25151095	25251095	0.374905	PPP1R1A
5	25151267	25251267	0.368172	GLYCAM1
5	25151267	25251267	0.368172	PPP1R1A
5	25151290	25251290	0.369024	GLYCAM1
5	25151290	25251290	0.369024	PPP1R1A
5	25151361	25251361	0.369542	GLYCAM1
5	25151361	25251361	0.369542	PPP1R1A
5	25151368	25251368	0.369542	GLYCAM1
5	25151368	25251368	0.369542	PPP1R1A
5	25151372	25251372	0.371112	GLYCAM1
5	25151372	25251372	0.371112	PPP1R1A
5	25151482	25251482	0.380989	GLYCAM1
5	25151482	25251482	0.380989	PPP1R1A
5	25151550	25251550	0.378582	GLYCAM1
5	25151550	25251550	0.378582	PPP1R1A
5	25151659	25251659	0.384814	GLYCAM1
5	25151659	25251659	0.384814	PPP1R1A
5	25151664	25251664	0.384814	GLYCAM1
5	25151664	25251664	0.384814	PPP1R1A
5	25151807	25251807	0.384814	GLYCAM1
5	25151807	25251807	0.384814	PPP1R1A
5	25151808	25251808	0.384814	GLYCAM1
5	25151808	25251808	0.384814	PPP1R1A
5	25151827	25251827	0.384814	GLYCAM1
5	25151827	25251827	0.384814	PPP1R1A
5	25151837	25251837	0.384814	GLYCAM1
5	25151837	25251837	0.384814	PPP1R1A
5	25151852	25251852	0.384814	GLYCAM1
5	25151852	25251852	0.384814	PPP1R1A
5	25151860	25251860	0.384814	GLYCAM1
5	25151860	25251860	0.384814	PPP1R1A
5	25151884	25251884	0.384814	GLYCAM1
5	25151884	25251884	0.384814	PPP1R1A
5	25151894	25251894	0.384814	GLYCAM1
5	25151894	25251894	0.384814	PPP1R1A
5	25151949	25251949	0.384814	GLYCAM1
5	25151949	25251949	0.384814	PPP1R1A
5	25151962	25251962	0.384814	GLYCAM1
5	25151962	25251962	0.384814	PPP1R1A

5	25151992	25251992	0.384814	GLYCAM1
5	25151992	25251992	0.384814	PPP1R1A
5	25151994	25251994	0.384814	GLYCAM1
5	25151994	25251994	0.384814	PPP1R1A
5	25152100	25252100	0.384814	GLYCAM1
5	25152100	25252100	0.384814	PPP1R1A
5	25152170	25252170	0.384814	GLYCAM1
5	25152170	25252170	0.384814	PPP1R1A
5	25152201	25252201	0.386466	GLYCAM1
5	25152201	25252201	0.386466	PPP1R1A
5	25152230	25252230	0.376697	GLYCAM1
5	25152230	25252230	0.376697	PPP1R1A
5	25152236	25252236	0.379143	GLYCAM1
5	25152236	25252236	0.379143	PPP1R1A
5	25152267	25252267	0.376397	GLYCAM1
5	25152267	25252267	0.376397	PPP1R1A
5	25152283	25252283	0.375351	GLYCAM1
5	25152283	25252283	0.375351	PPP1R1A
5	25152299	25252299	0.378373	GLYCAM1
5	25152299	25252299	0.378373	PPP1R1A
5	25152327	25252327	0.372999	GLYCAM1
5	25152327	25252327	0.372999	PPP1R1A
5	25152357	25252357	0.36871	GLYCAM1
5	25152357	25252357	0.36871	PPP1R1A
5	25152371	25252371	0.36871	GLYCAM1
5	25152371	25252371	0.36871	PPP1R1A
5	25152378	25252378	0.363697	GLYCAM1
5	25152378	25252378	0.363697	PPP1R1A
5	25152419	25252419	0.363538	GLYCAM1
5	25152419	25252419	0.363538	PPP1R1A
5	25152464	25252464	0.363538	GLYCAM1
5	25152464	25252464	0.363538	PPP1R1A
5	25152486	25252486	0.363538	GLYCAM1
5	25152486	25252486	0.363538	PPP1R1A
5	25152548	25252548	0.363538	GLYCAM1
5	25152548	25252548	0.363538	PPP1R1A
5	25152554	25252554	0.363538	GLYCAM1
5	25152554	25252554	0.363538	PPP1R1A
5	25152563	25252563	0.363538	GLYCAM1
5	25152563	25252563	0.363538	PPP1R1A
5	25152564	25252564	0.363538	GLYCAM1
5	25152564	25252564	0.363538	PPP1R1A
5	25152585	25252585	0.363538	GLYCAM1
5	25152585	25252585	0.363538	PPP1R1A
5	25152586	25252586	0.363538	GLYCAM1
5	25152586	25252586	0.363538	PPP1R1A
5	25152625	25252625	0.363538	GLYCAM1
5	25152625	25252625	0.363538	PPP1R1A
5	25152643	25252643	0.365122	GLYCAM1
5	25152643	25252643	0.365122	PPP1R1A
5	25152645	25252645	0.365122	GLYCAM1
5	25152645	25252645	0.365122	PPP1R1A
5	25152661	25252661	0.360512	GLYCAM1
5	25152661	25252661	0.360512	PPP1R1A
5	25152685	25252685	0.360512	GLYCAM1
5	25152685	25252685	0.360512	PPP1R1A
5	25152701	25252701	0.360619	GLYCAM1
5	25152701	25252701	0.360619	PPP1R1A

5	25152727	25252727	0.360434	GLYCAM1
5	25152727	25252727	0.360434	PPP1R1A
5	25152734	25252734	0.360434	GLYCAM1
5	25152734	25252734	0.360434	PPP1R1A
5	25152770	25252770	0.361852	GLYCAM1
5	25152770	25252770	0.361852	PPP1R1A
5	25152774	25252774	0.361852	GLYCAM1
5	25152774	25252774	0.361852	PPP1R1A
5	25152784	25252784	0.361852	GLYCAM1
5	25152784	25252784	0.361852	PPP1R1A
5	25152787	25252787	0.362291	GLYCAM1
5	25152787	25252787	0.362291	PPP1R1A
5	25152822	25252822	0.362072	GLYCAM1
5	25152822	25252822	0.362072	PPP1R1A
5	25152834	25252834	0.359165	GLYCAM1
5	25152834	25252834	0.359165	PPP1R1A
5	25152839	25252839	0.36058	GLYCAM1
5	25152839	25252839	0.36058	PPP1R1A
5	25152842	25252842	0.36058	GLYCAM1
5	25152842	25252842	0.36058	PPP1R1A
5	25152865	25252865	0.359249	GLYCAM1
5	25152865	25252865	0.359249	PPP1R1A
5	25152893	25252893	0.360151	GLYCAM1
5	25152893	25252893	0.360151	PPP1R1A
5	25152940	25252940	0.359329	GLYCAM1
5	25152940	25252940	0.359329	PPP1R1A
5	25153058	25253058	0.359329	GLYCAM1
5	25153058	25253058	0.359329	PPP1R1A
5	25153079	25253079	0.358385	GLYCAM1
5	25153079	25253079	0.358385	PPP1R1A
5	25153097	25253097	0.358385	GLYCAM1
5	25153097	25253097	0.358385	PPP1R1A
5	25153108	25253108	0.358385	GLYCAM1
5	25153108	25253108	0.358385	PPP1R1A
5	25153109	25253109	0.358385	GLYCAM1
5	25153109	25253109	0.358385	PPP1R1A
5	25153124	25253124	0.36083	GLYCAM1
5	25153124	25253124	0.36083	PPP1R1A
5	25153185	25253185	0.362573	GLYCAM1
5	25153185	25253185	0.362573	PPP1R1A
5	25153187	25253187	0.362573	GLYCAM1
5	25153187	25253187	0.362573	PPP1R1A
5	25153206	25253206	0.362601	GLYCAM1
5	25153206	25253206	0.362601	PPP1R1A
5	25153259	25253259	0.361855	GLYCAM1
5	25153259	25253259	0.361855	PPP1R1A
5	25153272	25253272	0.362686	GLYCAM1
5	25153272	25253272	0.362686	PPP1R1A
5	25153288	25253288	0.362739	GLYCAM1
5	25153288	25253288	0.362739	PPP1R1A
5	25153499	25253499	0.360436	GLYCAM1
5	25153499	25253499	0.360436	PPP1R1A
5	25153547	25253547	0.361134	GLYCAM1
5	25153547	25253547	0.361134	PPP1R1A
5	25153576	25253576	0.357733	GLYCAM1
5	25153576	25253576	0.357733	PPP1R1A
5	25153625	25253625	0.355684	GLYCAM1
5	25153625	25253625	0.355684	PPP1R1A

5	25153679	25253679	0.355684	GLYCAM1
5	25153679	25253679	0.355684	PPP1R1A
5	25153748	25253748	0.355684	GLYCAM1
5	25153748	25253748	0.355684	PPP1R1A
5	25153765	25253765	0.355684	GLYCAM1
5	25153765	25253765	0.355684	PPP1R1A
5	25153797	25253797	0.355684	GLYCAM1
5	25153797	25253797	0.355684	PPP1R1A
5	25153806	25253806	0.355684	GLYCAM1
5	25153806	25253806	0.355684	PPP1R1A
5	25153807	25253807	0.357852	GLYCAM1
5	25153807	25253807	0.357852	PPP1R1A
5	25153831	25253831	0.356917	GLYCAM1
5	25153831	25253831	0.356917	PPP1R1A
5	25153833	25253833	0.358431	GLYCAM1
5	25153833	25253833	0.358431	PPP1R1A
5	25153839	25253839	0.358431	GLYCAM1
5	25153839	25253839	0.358431	PPP1R1A
5	25153909	25253909	0.358431	GLYCAM1
5	25153909	25253909	0.358431	PPP1R1A
5	25153916	25253916	0.358431	GLYCAM1
5	25153916	25253916	0.358431	PPP1R1A
5	25153916	25253916	0.358431	PDE1B
5	25153967	25253967	0.358431	GLYCAM1
5	25153967	25253967	0.358431	PPP1R1A
5	25153967	25253967	0.358431	PDE1B
5	25153970	25253970	0.358568	GLYCAM1
5	25153970	25253970	0.358568	PPP1R1A
5	25153970	25253970	0.358568	PDE1B
5	25153997	25253997	0.359181	GLYCAM1
5	25153997	25253997	0.359181	PPP1R1A
5	25153997	25253997	0.359181	PDE1B
5	25154027	25254027	0.359181	GLYCAM1
5	25154027	25254027	0.359181	PPP1R1A
5	25154027	25254027	0.359181	PDE1B
5	25154053	25254053	0.359181	GLYCAM1
5	25154053	25254053	0.359181	PPP1R1A
5	25154053	25254053	0.359181	PDE1B
5	25154076	25254076	0.359181	GLYCAM1
5	25154076	25254076	0.359181	PPP1R1A
5	25154076	25254076	0.359181	PDE1B
5	25154090	25254090	0.359181	GLYCAM1
5	25154090	25254090	0.359181	PPP1R1A
5	25154090	25254090	0.359181	PDE1B
5	25154091	25254091	0.359181	GLYCAM1
5	25154091	25254091	0.359181	PPP1R1A
5	25154091	25254091	0.359181	PDE1B
5	25154113	25254113	0.362619	GLYCAM1
5	25154113	25254113	0.362619	PPP1R1A
5	25154113	25254113	0.362619	PDE1B
5	25154145	25254145	0.353859	GLYCAM1
5	25154145	25254145	0.353859	PPP1R1A
5	25154145	25254145	0.353859	PDE1B
5	25154466	25254466	0.353859	GLYCAM1
5	25154466	25254466	0.353859	PPP1R1A
5	25154466	25254466	0.353859	PDE1B
5	25154513	25254513	0.348859	GLYCAM1
5	25154513	25254513	0.348859	PPP1R1A

5	25154513	25254513	0.348859	PDE1B
5	25154566	25254566	0.350428	GLYCAM1
5	25154566	25254566	0.350428	PPP1R1A
5	25154566	25254566	0.350428	PDE1B
5	25154616	25254616	0.347101	GLYCAM1
5	25154616	25254616	0.347101	PPP1R1A
5	25154616	25254616	0.347101	PDE1B
5	25155312	25255312	0.352051	GLYCAM1
5	25155312	25255312	0.352051	PPP1R1A
5	25155312	25255312	0.352051	PDE1B
5	25155313	25255313	0.369131	GLYCAM1
5	25155313	25255313	0.369131	PPP1R1A
5	25155313	25255313	0.369131	PDE1B
5	25155323	25255323	0.35882	GLYCAM1
5	25155323	25255323	0.35882	PPP1R1A
5	25155323	25255323	0.35882	PDE1B
5	25155423	25255423	0.35882	GLYCAM1
5	25155423	25255423	0.35882	PPP1R1A
5	25155423	25255423	0.35882	PDE1B
5	25155432	25255432	0.35882	GLYCAM1
5	25155432	25255432	0.35882	PPP1R1A
5	25155432	25255432	0.35882	PDE1B
5	25155499	25255499	0.35882	GLYCAM1
5	25155499	25255499	0.35882	PPP1R1A
5	25155499	25255499	0.35882	PDE1B
5	25155511	25255511	0.360087	GLYCAM1
5	25155511	25255511	0.360087	PPP1R1A
5	25155511	25255511	0.360087	PDE1B
5	25155546	25255546	0.357602	GLYCAM1
5	25155546	25255546	0.357602	PPP1R1A
5	25155546	25255546	0.357602	PDE1B
5	25155583	25255583	0.357602	GLYCAM1
5	25155583	25255583	0.357602	PPP1R1A
5	25155583	25255583	0.357602	PDE1B
5	25155986	25255986	0.356042	GLYCAM1
5	25155986	25255986	0.356042	PPP1R1A
5	25155986	25255986	0.356042	PDE1B
5	25156137	25256137	0.352577	GLYCAM1
5	25156137	25256137	0.352577	PPP1R1A
5	25156137	25256137	0.352577	PDE1B
5	25156143	25256143	0.402555	GLYCAM1
5	25156143	25256143	0.402555	PPP1R1A
5	25156143	25256143	0.402555	PDE1B
5	25156208	25256208	0.387224	GLYCAM1
5	25156208	25256208	0.387224	PPP1R1A
5	25156208	25256208	0.387224	PDE1B
5	25156273	25256273	0.386655	GLYCAM1
5	25156273	25256273	0.386655	PPP1R1A
5	25156273	25256273	0.386655	PDE1B
5	25156435	25256435	0.388243	GLYCAM1
5	25156435	25256435	0.388243	PPP1R1A
5	25156435	25256435	0.388243	PDE1B
5	25156654	25256654	0.38579	GLYCAM1
5	25156654	25256654	0.38579	PPP1R1A
5	25156654	25256654	0.38579	PDE1B
5	25156707	25256707	0.386171	GLYCAM1
5	25156707	25256707	0.386171	PPP1R1A
5	25156707	25256707	0.386171	PDE1B

5	25157162	25257162	0.380354	GLYCAM1
5	25157162	25257162	0.380354	PPP1R1A
5	25157162	25257162	0.380354	PDE1B
5	25157208	25257208	0.380666	GLYCAM1
5	25157208	25257208	0.380666	PPP1R1A
5	25157208	25257208	0.380666	PDE1B
5	25157699	25257699	0.380666	GLYCAM1
5	25157699	25257699	0.380666	PPP1R1A
5	25157699	25257699	0.380666	PDE1B
5	25157780	25257780	0.380914	GLYCAM1
5	25157780	25257780	0.380914	PPP1R1A
5	25157780	25257780	0.380914	PDE1B
5	25157889	25257889	0.380914	GLYCAM1
5	25157889	25257889	0.380914	PPP1R1A
5	25157889	25257889	0.380914	PDE1B
5	25157895	25257895	0.382526	GLYCAM1
5	25157895	25257895	0.382526	PPP1R1A
5	25157895	25257895	0.382526	PDE1B
5	25158012	25258012	0.382729	GLYCAM1
5	25158012	25258012	0.382729	PPP1R1A
5	25158012	25258012	0.382729	PDE1B
5	25158062	25258062	0.385578	GLYCAM1
5	25158062	25258062	0.385578	PPP1R1A
5	25158062	25258062	0.385578	PDE1B
5	25158071	25258071	0.401773	GLYCAM1
5	25158071	25258071	0.401773	PPP1R1A
5	25158071	25258071	0.401773	PDE1B
5	25158210	25258210	0.390035	GLYCAM1
5	25158210	25258210	0.390035	PPP1R1A
5	25158210	25258210	0.390035	PDE1B
5	25158296	25258296	0.390159	GLYCAM1
5	25158296	25258296	0.390159	PPP1R1A
5	25158296	25258296	0.390159	PDE1B
5	25158474	25258474	0.392573	GLYCAM1
5	25158474	25258474	0.392573	PPP1R1A
5	25158474	25258474	0.392573	PDE1B
5	25158503	25258503	0.385142	GLYCAM1
5	25158503	25258503	0.385142	PPP1R1A
5	25158503	25258503	0.385142	PDE1B
5	25158686	25258686	0.385253	GLYCAM1
5	25158686	25258686	0.385253	PPP1R1A
5	25158686	25258686	0.385253	PDE1B
5	25159066	25259066	0.39813	GLYCAM1
5	25159066	25259066	0.39813	PPP1R1A
5	25159066	25259066	0.39813	PDE1B
5	25159163	25259163	0.398293	GLYCAM1
5	25159163	25259163	0.398293	PPP1R1A
5	25159163	25259163	0.398293	PDE1B
5	25159282	25259282	0.398458	GLYCAM1
5	25159282	25259282	0.398458	PPP1R1A
5	25159282	25259282	0.398458	PDE1B
5	25159311	25259311	0.398381	GLYCAM1
5	25159311	25259311	0.398381	PPP1R1A
5	25159311	25259311	0.398381	PDE1B
5	25159365	25259365	0.397158	GLYCAM1
5	25159365	25259365	0.397158	PPP1R1A
5	25159365	25259365	0.397158	PDE1B
5	25159437	25259437	0.405164	GLYCAM1

5	25159437	25259437	0.405164	PPP1R1A
5	25159437	25259437	0.405164	PDE1B
5	25159475	25259475	0.390219	GLYCAM1
5	25159475	25259475	0.390219	PPP1R1A
5	25159475	25259475	0.390219	PDE1B
5	25159615	25259615	0.376738	GLYCAM1
5	25159615	25259615	0.376738	PPP1R1A
5	25159615	25259615	0.376738	PDE1B
5	25159776	25259776	0.369924	GLYCAM1
5	25159776	25259776	0.369924	PPP1R1A
5	25159776	25259776	0.369924	PDE1B
5	25160246	25260246	0.39094	GLYCAM1
5	25160246	25260246	0.39094	PPP1R1A
5	25160246	25260246	0.39094	PDE1B
5	25160436	25260436	0.393103	GLYCAM1
5	25160436	25260436	0.393103	PPP1R1A
5	25160436	25260436	0.393103	PDE1B
5	25160480	25260480	0.398018	GLYCAM1
5	25160480	25260480	0.398018	PPP1R1A
5	25160480	25260480	0.398018	PDE1B
5	25160860	25260860	0.377602	GLYCAM1
5	25160860	25260860	0.377602	PPP1R1A
5	25160860	25260860	0.377602	PDE1B
5	25161145	25261145	0.381746	GLYCAM1
5	25161145	25261145	0.381746	PPP1R1A
5	25161145	25261145	0.381746	PDE1B
5	25161149	25261149	0.396064	GLYCAM1
5	25161149	25261149	0.396064	PPP1R1A
5	25161149	25261149	0.396064	PDE1B
5	25161579	25261579	0.403789	GLYCAM1
5	25161579	25261579	0.403789	PPP1R1A
5	25161579	25261579	0.403789	PDE1B
5	25161782	25261782	0.386262	GLYCAM1
5	25161782	25261782	0.386262	PPP1R1A
5	25161782	25261782	0.386262	PDE1B
5	25161881	25261881	0.379113	GLYCAM1
5	25161881	25261881	0.379113	PPP1R1A
5	25161881	25261881	0.379113	PDE1B
5	25161909	25261909	0.379152	GLYCAM1
5	25161909	25261909	0.379152	PPP1R1A
5	25161909	25261909	0.379152	PDE1B
5	25162091	25262091	0.376696	GLYCAM1
5	25162091	25262091	0.376696	PPP1R1A
5	25162091	25262091	0.376696	PDE1B
5	25162235	25262235	0.378816	GLYCAM1
5	25162235	25262235	0.378816	PPP1R1A
5	25162235	25262235	0.378816	PDE1B
5	25162384	25262384	0.378816	GLYCAM1
5	25162384	25262384	0.378816	PPP1R1A
5	25162384	25262384	0.378816	PDE1B
5	25162867	25262867	0.381975	GLYCAM1
5	25162867	25262867	0.381975	PPP1R1A
5	25162867	25262867	0.381975	PDE1B
5	25162977	25262977	0.40541	GLYCAM1
5	25162977	25262977	0.40541	PPP1R1A
5	25162977	25262977	0.40541	PDE1B
5	25163000	25263000	0.411156	GLYCAM1
5	25163000	25263000	0.411156	PPP1R1A

5	25163000	25263000	0.411156	PDE1B
5	25163057	25263057	0.397409	GLYCAM1
5	25163057	25263057	0.397409	PPP1R1A
5	25163057	25263057	0.397409	PDE1B
5	25163065	25263065	0.397733	GLYCAM1
5	25163065	25263065	0.397733	PPP1R1A
5	25163065	25263065	0.397733	PDE1B
5	25163124	25263124	0.398501	GLYCAM1
5	25163124	25263124	0.398501	PPP1R1A
5	25163124	25263124	0.398501	PDE1B
5	25172510	25272510	0.382837	GLYCAM1
5	25172510	25272510	0.382837	PPP1R1A
5	25172510	25272510	0.382837	PDE1B
5	25172631	25272631	0.370275	GLYCAM1
5	25172631	25272631	0.370275	PPP1R1A
5	25172631	25272631	0.370275	PDE1B
5	25172790	25272790	0.370506	GLYCAM1
5	25172790	25272790	0.370506	PPP1R1A
5	25172790	25272790	0.370506	PDE1B
5	25172813	25272813	0.370157	GLYCAM1
5	25172813	25272813	0.370157	PPP1R1A
5	25172813	25272813	0.370157	PDE1B
5	25172822	25272822	0.376613	GLYCAM1
5	25172822	25272822	0.376613	PPP1R1A
5	25172822	25272822	0.376613	PDE1B
5	25172869	25272869	0.374451	GLYCAM1
5	25172869	25272869	0.374451	PPP1R1A
5	25172869	25272869	0.374451	PDE1B
5	25172879	25272879	0.374449	GLYCAM1
5	25172879	25272879	0.374449	PPP1R1A
5	25172879	25272879	0.374449	PDE1B
5	25172980	25272980	0.38312	GLYCAM1
5	25172980	25272980	0.38312	PPP1R1A
5	25172980	25272980	0.38312	PDE1B
5	25173064	25273064	0.384611	GLYCAM1
5	25173064	25273064	0.384611	PPP1R1A
5	25173064	25273064	0.384611	PDE1B
5	25173257	25273257	0.371689	GLYCAM1
5	25173257	25273257	0.371689	PPP1R1A
5	25173257	25273257	0.371689	PDE1B
5	25173464	25273464	0.374634	GLYCAM1
5	25173464	25273464	0.374634	PPP1R1A
5	25173464	25273464	0.374634	PDE1B
5	25173507	25273507	0.376043	GLYCAM1
5	25173507	25273507	0.376043	PPP1R1A
5	25173507	25273507	0.376043	PDE1B
5	25173694	25273694	0.374124	GLYCAM1
5	25173694	25273694	0.374124	PPP1R1A
5	25173694	25273694	0.374124	PDE1B
5	25173822	25273822	0.373539	GLYCAM1
5	25173822	25273822	0.373539	PPP1R1A
5	25173822	25273822	0.373539	PDE1B
5	25174210	25274210	0.398793	GLYCAM1
5	25174210	25274210	0.398793	PPP1R1A
5	25174210	25274210	0.398793	PDE1B
5	25174216	25274216	0.399655	GLYCAM1
5	25174216	25274216	0.399655	PPP1R1A
5	25174216	25274216	0.399655	PDE1B

5	25174836	25274836	0.388725	GLYCAM1
5	25174836	25274836	0.388725	PPP1R1A
5	25174836	25274836	0.388725	PDE1B
5	25174853	25274853	0.39087	GLYCAM1
5	25174853	25274853	0.39087	PPP1R1A
5	25174853	25274853	0.39087	PDE1B
5	25174962	25274962	0.390192	GLYCAM1
5	25174962	25274962	0.390192	PPP1R1A
5	25174962	25274962	0.390192	PDE1B
5	25175007	25275007	0.386072	GLYCAM1
5	25175007	25275007	0.386072	PPP1R1A
5	25175007	25275007	0.386072	PDE1B
5	25175287	25275287	0.386658	GLYCAM1
5	25175287	25275287	0.386658	PPP1R1A
5	25175287	25275287	0.386658	PDE1B
5	25175342	25275342	0.387626	GLYCAM1
5	25175342	25275342	0.387626	PPP1R1A
5	25175342	25275342	0.387626	PDE1B
5	25175402	25275402	0.388286	GLYCAM1
5	25175402	25275402	0.388286	PPP1R1A
5	25175402	25275402	0.388286	PDE1B
5	25175499	25275499	0.386025	GLYCAM1
5	25175499	25275499	0.386025	PPP1R1A
5	25175499	25275499	0.386025	PDE1B
5	25175587	25275587	0.386272	GLYCAM1
5	25175587	25275587	0.386272	PPP1R1A
5	25175587	25275587	0.386272	PDE1B
5	25175765	25275765	0.385859	GLYCAM1
5	25175765	25275765	0.385859	PPP1R1A
5	25175765	25275765	0.385859	PDE1B
5	25175877	25275877	0.378299	GLYCAM1
5	25175877	25275877	0.378299	PPP1R1A
5	25175877	25275877	0.378299	PDE1B
5	25175881	25275881	0.382795	GLYCAM1
5	25175881	25275881	0.382795	PPP1R1A
5	25175881	25275881	0.382795	PDE1B
5	25175893	25275893	0.381949	GLYCAM1
5	25175893	25275893	0.381949	PPP1R1A
5	25175893	25275893	0.381949	PDE1B
5	25175916	25275916	0.380987	GLYCAM1
5	25175916	25275916	0.380987	PPP1R1A
5	25175916	25275916	0.380987	PDE1B
5	25176199	25276199	0.385987	GLYCAM1
5	25176199	25276199	0.385987	PPP1R1A
5	25176199	25276199	0.385987	PDE1B
5	25176212	25276212	0.379382	GLYCAM1
5	25176212	25276212	0.379382	PPP1R1A
5	25176212	25276212	0.379382	PDE1B
5	25176238	25276238	0.37935	GLYCAM1
5	25176238	25276238	0.37935	PPP1R1A
5	25176238	25276238	0.37935	PDE1B
5	25176285	25276285	0.37935	GLYCAM1
5	25176285	25276285	0.37935	PPP1R1A
5	25176285	25276285	0.37935	PDE1B
5	25176306	25276306	0.380713	GLYCAM1
5	25176306	25276306	0.380713	PPP1R1A
5	25176306	25276306	0.380713	PDE1B
5	25176330	25276330	0.379747	GLYCAM1

5	25176330	25276330	0.379747	PPP1R1A
5	25176330	25276330	0.379747	PDE1B
5	25176380	25276380	0.379763	GLYCAM1
5	25176380	25276380	0.379763	PPP1R1A
5	25176380	25276380	0.379763	PDE1B
5	25176504	25276504	0.377742	GLYCAM1
5	25176504	25276504	0.377742	PPP1R1A
5	25176504	25276504	0.377742	PDE1B
5	25176737	25276737	0.427946	GLYCAM1
5	25176737	25276737	0.427946	PPP1R1A
5	25176737	25276737	0.427946	PDE1B
5	25176932	25276932	0.42165	GLYCAM1
5	25176932	25276932	0.42165	PPP1R1A
5	25176932	25276932	0.42165	PDE1B
5	25176956	25276956	0.42165	GLYCAM1
5	25176956	25276956	0.42165	PPP1R1A
5	25176956	25276956	0.42165	PDE1B
5	25177032	25277032	0.422562	GLYCAM1
5	25177032	25277032	0.422562	PPP1R1A
5	25177032	25277032	0.422562	PDE1B
5	25177191	25277191	0.418178	GLYCAM1
5	25177191	25277191	0.418178	PPP1R1A
5	25177191	25277191	0.418178	PDE1B
5	25177265	25277265	0.407899	GLYCAM1
5	25177265	25277265	0.407899	PPP1R1A
5	25177265	25277265	0.407899	PDE1B
5	25177266	25277266	0.40799	GLYCAM1
5	25177266	25277266	0.40799	PPP1R1A
5	25177266	25277266	0.40799	PDE1B
5	25177294	25277294	0.427022	GLYCAM1
5	25177294	25277294	0.427022	PPP1R1A
5	25177294	25277294	0.427022	PDE1B
5	25177479	25277479	0.417402	GLYCAM1
5	25177479	25277479	0.417402	PPP1R1A
5	25177479	25277479	0.417402	PDE1B
5	25177502	25277502	0.427521	GLYCAM1
5	25177502	25277502	0.427521	PPP1R1A
5	25177502	25277502	0.427521	PDE1B
5	25177594	25277594	0.424276	GLYCAM1
5	25177594	25277594	0.424276	PPP1R1A
5	25177594	25277594	0.424276	PDE1B
5	25177772	25277772	0.400543	GLYCAM1
5	25177772	25277772	0.400543	PPP1R1A
5	25177772	25277772	0.400543	PDE1B
5	25177792	25277792	0.400543	GLYCAM1
5	25177792	25277792	0.400543	PPP1R1A
5	25177792	25277792	0.400543	PDE1B
5	25177796	25277796	0.400543	GLYCAM1
5	25177796	25277796	0.400543	PPP1R1A
5	25177796	25277796	0.400543	PDE1B
5	25177798	25277798	0.400543	GLYCAM1
5	25177798	25277798	0.400543	PPP1R1A
5	25177798	25277798	0.400543	PDE1B
5	25177838	25277838	0.414956	GLYCAM1
5	25177838	25277838	0.414956	PPP1R1A
5	25177838	25277838	0.414956	PDE1B
5	25178105	25278105	0.379442	GLYCAM1
5	25178105	25278105	0.379442	PPP1R1A

5	25178105	25278105	0.379442	PDE1B
5	25178211	25278211	0.397615	GLYCAM1
5	25178211	25278211	0.397615	PPP1R1A
5	25178211	25278211	0.397615	PDE1B
5	25178328	25278328	0.358435	GLYCAM1
5	25178328	25278328	0.358435	PPP1R1A
5	25178328	25278328	0.358435	PDE1B
5	25178341	25278341	0.357876	GLYCAM1
5	25178341	25278341	0.357876	PPP1R1A
5	25178341	25278341	0.357876	PDE1B
5	25178343	25278343	0.357719	GLYCAM1
5	25178343	25278343	0.357719	PPP1R1A
5	25178343	25278343	0.357719	PDE1B
5	25178434	25278434	0.404676	GLYCAM1
5	25178434	25278434	0.404676	PPP1R1A
5	25178434	25278434	0.404676	PDE1B
5	25178700	25278700	0.399153	GLYCAM1
5	25178700	25278700	0.399153	PPP1R1A
5	25178700	25278700	0.399153	PDE1B
5	25178710	25278710	0.399153	GLYCAM1
5	25178710	25278710	0.399153	PPP1R1A
5	25178710	25278710	0.399153	PDE1B
5	25178866	25278866	0.414184	GLYCAM1
5	25178866	25278866	0.414184	PPP1R1A
5	25178866	25278866	0.414184	PDE1B
5	25178950	25278950	0.398101	GLYCAM1
5	25178950	25278950	0.398101	PPP1R1A
5	25178950	25278950	0.398101	PDE1B
5	25178959	25278959	0.410446	GLYCAM1
5	25178959	25278959	0.410446	PPP1R1A
5	25178959	25278959	0.410446	PDE1B
5	25178986	25278986	0.426681	GLYCAM1
5	25178986	25278986	0.426681	PPP1R1A
5	25178986	25278986	0.426681	PDE1B
5	25179129	25279129	0.423838	GLYCAM1
5	25179129	25279129	0.423838	PPP1R1A
5	25179129	25279129	0.423838	PDE1B
5	25179134	25279134	0.418557	GLYCAM1
5	25179134	25279134	0.418557	PPP1R1A
5	25179134	25279134	0.418557	PDE1B
5	25179169	25279169	0.447647	GLYCAM1
5	25179169	25279169	0.447647	PPP1R1A
5	25179169	25279169	0.447647	PDE1B
5	25179192	25279192	0.428321	GLYCAM1
5	25179192	25279192	0.428321	PPP1R1A
5	25179192	25279192	0.428321	PDE1B
5	25179288	25279288	0.424526	GLYCAM1
5	25179288	25279288	0.424526	PPP1R1A
5	25179288	25279288	0.424526	PDE1B
5	25179626	25279626	0.415808	GLYCAM1
5	25179626	25279626	0.415808	PPP1R1A
5	25179626	25279626	0.415808	PDE1B
5	25179627	25279627	0.415808	GLYCAM1
5	25179627	25279627	0.415808	PPP1R1A
5	25179627	25279627	0.415808	PDE1B
5	25179663	25279663	0.411095	GLYCAM1
5	25179663	25279663	0.411095	PPP1R1A
5	25179663	25279663	0.411095	PDE1B

5	25179746	25279746	0.478983	GLYCAM1
5	25179746	25279746	0.478983	PPP1R1A
5	25179746	25279746	0.478983	PDE1B
5	25179765	25279765	0.470965	GLYCAM1
5	25179765	25279765	0.470965	PPP1R1A
5	25179765	25279765	0.470965	PDE1B
5	25179861	25279861	0.470945	GLYCAM1
5	25179861	25279861	0.470945	PPP1R1A
5	25179861	25279861	0.470945	PDE1B
5	25179976	25279976	0.469378	GLYCAM1
5	25179976	25279976	0.469378	PPP1R1A
5	25179976	25279976	0.469378	PDE1B
5	25180093	25280093	0.469558	GLYCAM1
5	25180093	25280093	0.469558	PPP1R1A
5	25180093	25280093	0.469558	PDE1B
5	25180111	25280111	0.468025	GLYCAM1
5	25180111	25280111	0.468025	PPP1R1A
5	25180111	25280111	0.468025	PDE1B
5	25180299	25280299	0.479816	GLYCAM1
5	25180299	25280299	0.479816	PPP1R1A
5	25180299	25280299	0.479816	PDE1B
5	25180720	25280720	0.467386	GLYCAM1
5	25180720	25280720	0.467386	PPP1R1A
5	25180720	25280720	0.467386	PDE1B
5	25180832	25280832	0.477129	GLYCAM1
5	25180832	25280832	0.477129	PPP1R1A
5	25180832	25280832	0.477129	PDE1B
5	25180958	25280958	0.442194	GLYCAM1
5	25180958	25280958	0.442194	PPP1R1A
5	25180958	25280958	0.442194	PDE1B
5	25181217	25281217	0.444492	GLYCAM1
5	25181217	25281217	0.444492	PPP1R1A
5	25181217	25281217	0.444492	PDE1B
5	25181420	25281420	0.445219	GLYCAM1
5	25181420	25281420	0.445219	PPP1R1A
5	25181420	25281420	0.445219	PDE1B
5	25181468	25281468	0.445219	GLYCAM1
5	25181468	25281468	0.445219	PPP1R1A
5	25181468	25281468	0.445219	PDE1B
5	25181510	25281510	0.445378	GLYCAM1
5	25181510	25281510	0.445378	PPP1R1A
5	25181510	25281510	0.445378	PDE1B
5	25181541	25281541	0.442612	GLYCAM1
5	25181541	25281541	0.442612	PPP1R1A
5	25181541	25281541	0.442612	PDE1B
5	25181650	25281650	0.428917	GLYCAM1
5	25181650	25281650	0.428917	PPP1R1A
5	25181650	25281650	0.428917	PDE1B
5	25181986	25281986	0.43484	GLYCAM1
5	25181986	25281986	0.43484	PPP1R1A
5	25181986	25281986	0.43484	PDE1B
5	25182071	25282071	0.440629	GLYCAM1
5	25182071	25282071	0.440629	PPP1R1A
5	25182071	25282071	0.440629	PDE1B
5	25182106	25282106	0.444507	GLYCAM1
5	25182106	25282106	0.444507	PPP1R1A
5	25182106	25282106	0.444507	PDE1B
5	25182191	25282191	0.439002	GLYCAM1

5	25182191	25282191	0.439002	PPP1R1A
5	25182191	25282191	0.439002	PDE1B
5	25182257	25282257	0.438958	GLYCAM1
5	25182257	25282257	0.438958	PPP1R1A
5	25182257	25282257	0.438958	PDE1B
5	25182299	25282299	0.457033	GLYCAM1
5	25182299	25282299	0.457033	PPP1R1A
5	25182299	25282299	0.457033	PDE1B
5	25182344	25282344	0.443833	GLYCAM1
5	25182344	25282344	0.443833	PPP1R1A
5	25182344	25282344	0.443833	PDE1B
5	25182379	25282379	0.443833	GLYCAM1
5	25182379	25282379	0.443833	PPP1R1A
5	25182379	25282379	0.443833	PDE1B
5	25182402	25282402	0.443833	GLYCAM1
5	25182402	25282402	0.443833	PPP1R1A
5	25182402	25282402	0.443833	PDE1B
5	25182535	25282535	0.445401	GLYCAM1
5	25182535	25282535	0.445401	PPP1R1A
5	25182535	25282535	0.445401	PDE1B
5	25182565	25282565	0.439361	GLYCAM1
5	25182565	25282565	0.439361	PPP1R1A
5	25182565	25282565	0.439361	PDE1B
5	25182623	25282623	0.439361	GLYCAM1
5	25182623	25282623	0.439361	PPP1R1A
5	25182623	25282623	0.439361	PDE1B
5	25182651	25282651	0.439361	GLYCAM1
5	25182651	25282651	0.439361	PPP1R1A
5	25182651	25282651	0.439361	PDE1B
5	25182710	25282710	0.439361	GLYCAM1
5	25182710	25282710	0.439361	PPP1R1A
5	25182710	25282710	0.439361	PDE1B
5	25182911	25282911	0.439361	GLYCAM1
5	25182911	25282911	0.439361	PPP1R1A
5	25182911	25282911	0.439361	PDE1B
5	25182922	25282922	0.442675	GLYCAM1
5	25182922	25282922	0.442675	PPP1R1A
5	25182922	25282922	0.442675	PDE1B
5	25182945	25282945	0.453393	GLYCAM1
5	25182945	25282945	0.453393	PPP1R1A
5	25182945	25282945	0.453393	PDE1B
5	25183053	25283053	0.450814	GLYCAM1
5	25183053	25283053	0.450814	PPP1R1A
5	25183053	25283053	0.450814	PDE1B
5	25183056	25283056	0.450814	GLYCAM1
5	25183056	25283056	0.450814	PPP1R1A
5	25183056	25283056	0.450814	PDE1B
5	25183134	25283134	0.45854	GLYCAM1
5	25183134	25283134	0.45854	PPP1R1A
5	25183134	25283134	0.45854	PDE1B
5	25183146	25283146	0.453217	GLYCAM1
5	25183146	25283146	0.453217	PPP1R1A
5	25183146	25283146	0.453217	PDE1B
5	25183233	25283233	0.453452	GLYCAM1
5	25183233	25283233	0.453452	PPP1R1A
5	25183233	25283233	0.453452	PDE1B
5	25183237	25283237	0.453452	GLYCAM1
5	25183237	25283237	0.453452	PPP1R1A

5	25183237	25283237	0.453452	PDE1B
5	25183253	25283253	0.453452	GLYCAM1
5	25183253	25283253	0.453452	PPP1R1A
5	25183253	25283253	0.453452	PDE1B
5	25183285	25283285	0.453452	GLYCAM1
5	25183285	25283285	0.453452	PPP1R1A
5	25183285	25283285	0.453452	PDE1B
5	25183398	25283398	0.457602	GLYCAM1
5	25183398	25283398	0.457602	PPP1R1A
5	25183398	25283398	0.457602	PDE1B
5	25183410	25283410	0.461259	GLYCAM1
5	25183410	25283410	0.461259	PPP1R1A
5	25183410	25283410	0.461259	PDE1B
5	25183430	25283430	0.456127	GLYCAM1
5	25183430	25283430	0.456127	PPP1R1A
5	25183430	25283430	0.456127	PDE1B
5	25183591	25283591	0.449862	GLYCAM1
5	25183591	25283591	0.449862	PPP1R1A
5	25183591	25283591	0.449862	PDE1B
5	25183625	25283625	0.453403	GLYCAM1
5	25183625	25283625	0.453403	PPP1R1A
5	25183625	25283625	0.453403	PDE1B
5	25183738	25283738	0.443804	GLYCAM1
5	25183738	25283738	0.443804	PPP1R1A
5	25183738	25283738	0.443804	PDE1B
5	25183840	25283840	0.443804	GLYCAM1
5	25183840	25283840	0.443804	PPP1R1A
5	25183840	25283840	0.443804	PDE1B
5	25184062	25284062	0.443804	GLYCAM1
5	25184062	25284062	0.443804	PPP1R1A
5	25184062	25284062	0.443804	PDE1B
5	25184120	25284120	0.452619	GLYCAM1
5	25184120	25284120	0.452619	PPP1R1A
5	25184120	25284120	0.452619	PDE1B
5	25184238	25284238	0.447276	GLYCAM1
5	25184238	25284238	0.447276	PPP1R1A
5	25184238	25284238	0.447276	PDE1B
5	25184243	25284243	0.447517	GLYCAM1
5	25184243	25284243	0.447517	PPP1R1A
5	25184243	25284243	0.447517	PDE1B
5	25184370	25284370	0.449723	GLYCAM1
5	25184370	25284370	0.449723	PPP1R1A
5	25184370	25284370	0.449723	PDE1B
5	25184462	25284462	0.448223	GLYCAM1
5	25184462	25284462	0.448223	PPP1R1A
5	25184462	25284462	0.448223	PDE1B
5	25184649	25284649	0.456134	GLYCAM1
5	25184649	25284649	0.456134	PPP1R1A
5	25184649	25284649	0.456134	PDE1B
5	25184863	25284863	0.455846	GLYCAM1
5	25184863	25284863	0.455846	PPP1R1A
5	25184863	25284863	0.455846	PDE1B
5	25184933	25284933	0.449803	GLYCAM1
5	25184933	25284933	0.449803	PPP1R1A
5	25184933	25284933	0.449803	PDE1B
5	25184967	25284967	0.457415	GLYCAM1
5	25184967	25284967	0.457415	PPP1R1A
5	25184967	25284967	0.457415	PDE1B

5	25185074	25285074	0.446136	GLYCAM1
5	25185074	25285074	0.446136	PPP1R1A
5	25185074	25285074	0.446136	PDE1B
5	25185125	25285125	0.451833	GLYCAM1
5	25185125	25285125	0.451833	PPP1R1A
5	25185125	25285125	0.451833	PDE1B
5	25185399	25285399	0.460406	GLYCAM1
5	25185399	25285399	0.460406	PPP1R1A
5	25185399	25285399	0.460406	PDE1B
5	25185408	25285408	0.463572	GLYCAM1
5	25185408	25285408	0.463572	PPP1R1A
5	25185408	25285408	0.463572	PDE1B
5	25185475	25285475	0.44316	GLYCAM1
5	25185475	25285475	0.44316	PPP1R1A
5	25185475	25285475	0.44316	PDE1B
5	25185598	25285598	0.44328	GLYCAM1
5	25185598	25285598	0.44328	PPP1R1A
5	25185598	25285598	0.44328	PDE1B
5	25185606	25285606	0.44328	GLYCAM1
5	25185606	25285606	0.44328	PPP1R1A
5	25185606	25285606	0.44328	PDE1B
5	25185623	25285623	0.435695	GLYCAM1
5	25185623	25285623	0.435695	PPP1R1A
5	25185623	25285623	0.435695	PDE1B
5	25185634	25285634	0.437259	GLYCAM1
5	25185634	25285634	0.437259	PPP1R1A
5	25185634	25285634	0.437259	PDE1B
5	25185757	25285757	0.435999	GLYCAM1
5	25185757	25285757	0.435999	PPP1R1A
5	25185757	25285757	0.435999	PDE1B
5	25185783	25285783	0.447528	GLYCAM1
5	25185783	25285783	0.447528	PPP1R1A
5	25185783	25285783	0.447528	PDE1B
5	25185805	25285805	0.429249	GLYCAM1
5	25185805	25285805	0.429249	PPP1R1A
5	25185805	25285805	0.429249	PDE1B
5	25185926	25285926	0.429249	GLYCAM1
5	25185926	25285926	0.429249	PPP1R1A
5	25185926	25285926	0.429249	PDE1B
5	25186183	25286183	0.429249	GLYCAM1
5	25186183	25286183	0.429249	PPP1R1A
5	25186183	25286183	0.429249	PDE1B
5	25186273	25286273	0.429366	GLYCAM1
5	25186273	25286273	0.429366	PPP1R1A
5	25186273	25286273	0.429366	PDE1B
5	25186403	25286403	0.440466	GLYCAM1
5	25186403	25286403	0.440466	PPP1R1A
5	25186403	25286403	0.440466	PDE1B
5	25186403	25286403	0.440466	NCKAP1L
5	25186488	25286488	0.420899	GLYCAM1
5	25186488	25286488	0.420899	PPP1R1A
5	25186488	25286488	0.420899	PDE1B
5	25186488	25286488	0.420899	NCKAP1L
5	25186506	25286506	0.420899	GLYCAM1
5	25186506	25286506	0.420899	PPP1R1A
5	25186506	25286506	0.420899	PDE1B
5	25186506	25286506	0.420899	NCKAP1L
5	25186518	25286518	0.420899	GLYCAM1

5	25186518	25286518	0.420899	PPP1R1A
5	25186518	25286518	0.420899	PDE1B
5	25186518	25286518	0.420899	NCKAP1L
5	25186596	25286596	0.420899	GLYCAM1
5	25186596	25286596	0.420899	PPP1R1A
5	25186596	25286596	0.420899	PDE1B
5	25186596	25286596	0.420899	NCKAP1L
5	25186718	25286718	0.420899	GLYCAM1
5	25186718	25286718	0.420899	PPP1R1A
5	25186718	25286718	0.420899	PDE1B
5	25186718	25286718	0.420899	NCKAP1L
5	25186747	25286747	0.420899	GLYCAM1
5	25186747	25286747	0.420899	PPP1R1A
5	25186747	25286747	0.420899	PDE1B
5	25186747	25286747	0.420899	NCKAP1L
5	25186762	25286762	0.420288	GLYCAM1
5	25186762	25286762	0.420288	PPP1R1A
5	25186762	25286762	0.420288	PDE1B
5	25186762	25286762	0.420288	NCKAP1L
5	25186920	25286920	0.421369	GLYCAM1
5	25186920	25286920	0.421369	PPP1R1A
5	25186920	25286920	0.421369	PDE1B
5	25186920	25286920	0.421369	NCKAP1L
5	25186958	25286958	0.421369	GLYCAM1
5	25186958	25286958	0.421369	PPP1R1A
5	25186958	25286958	0.421369	PDE1B
5	25186958	25286958	0.421369	NCKAP1L
5	25186992	25286992	0.421369	GLYCAM1
5	25186992	25286992	0.421369	PPP1R1A
5	25186992	25286992	0.421369	PDE1B
5	25186992	25286992	0.421369	NCKAP1L
5	25187182	25287182	0.421369	GLYCAM1
5	25187182	25287182	0.421369	PPP1R1A
5	25187182	25287182	0.421369	PDE1B
5	25187182	25287182	0.421369	NCKAP1L
5	25187200	25287200	0.421369	GLYCAM1
5	25187200	25287200	0.421369	PPP1R1A
5	25187200	25287200	0.421369	PDE1B
5	25187200	25287200	0.421369	NCKAP1L
5	25187259	25287259	0.422161	GLYCAM1
5	25187259	25287259	0.422161	PPP1R1A
5	25187259	25287259	0.422161	PDE1B
5	25187259	25287259	0.422161	NCKAP1L
5	25187305	25287305	0.411642	GLYCAM1
5	25187305	25287305	0.411642	PPP1R1A
5	25187305	25287305	0.411642	PDE1B
5	25187305	25287305	0.411642	NCKAP1L
5	25187335	25287335	0.418466	GLYCAM1
5	25187335	25287335	0.418466	PPP1R1A
5	25187335	25287335	0.418466	PDE1B
5	25187335	25287335	0.418466	NCKAP1L
5	25187464	25287464	0.418466	GLYCAM1
5	25187464	25287464	0.418466	PPP1R1A
5	25187464	25287464	0.418466	PDE1B
5	25187464	25287464	0.418466	NCKAP1L
5	25187598	25287598	0.416757	GLYCAM1
5	25187598	25287598	0.416757	PPP1R1A
5	25187598	25287598	0.416757	PDE1B

5	25187598	25287598	0.416757	NCKAP1L
5	25187692	25287692	0.432273	GLYCAM1
5	25187692	25287692	0.432273	PPP1R1A
5	25187692	25287692	0.432273	PDE1B
5	25187692	25287692	0.432273	NCKAP1L
5	25187755	25287755	0.463072	GLYCAM1
5	25187755	25287755	0.463072	PPP1R1A
5	25187755	25287755	0.463072	PDE1B
5	25187755	25287755	0.463072	NCKAP1L
5	25187787	25287787	0.490582	GLYCAM1
5	25187787	25287787	0.490582	PPP1R1A
5	25187787	25287787	0.490582	PDE1B
5	25187787	25287787	0.490582	NCKAP1L
5	25187810	25287810	0.460224	GLYCAM1
5	25187810	25287810	0.460224	PPP1R1A
5	25187810	25287810	0.460224	PDE1B
5	25187810	25287810	0.460224	NCKAP1L
5	25188073	25288073	0.450639	GLYCAM1
5	25188073	25288073	0.450639	PPP1R1A
5	25188073	25288073	0.450639	PDE1B
5	25188073	25288073	0.450639	NCKAP1L
5	25188100	25288100	0.450639	GLYCAM1
5	25188100	25288100	0.450639	PPP1R1A
5	25188100	25288100	0.450639	PDE1B
5	25188100	25288100	0.450639	NCKAP1L
5	25188233	25288233	0.453511	GLYCAM1
5	25188233	25288233	0.453511	PPP1R1A
5	25188233	25288233	0.453511	PDE1B
5	25188233	25288233	0.453511	NCKAP1L
5	25188656	25288656	0.439033	GLYCAM1
5	25188656	25288656	0.439033	PPP1R1A
5	25188656	25288656	0.439033	PDE1B
5	25188656	25288656	0.439033	NCKAP1L
5	25188712	25288712	0.439547	GLYCAM1
5	25188712	25288712	0.439547	PPP1R1A
5	25188712	25288712	0.439547	PDE1B
5	25188712	25288712	0.439547	NCKAP1L
5	25189217	25289217	0.448214	GLYCAM1
5	25189217	25289217	0.448214	PPP1R1A
5	25189217	25289217	0.448214	PDE1B
5	25189217	25289217	0.448214	NCKAP1L
5	25189227	25289227	0.447262	GLYCAM1
5	25189227	25289227	0.447262	PPP1R1A
5	25189227	25289227	0.447262	PDE1B
5	25189227	25289227	0.447262	NCKAP1L
5	25189569	25289569	0.435912	GLYCAM1
5	25189569	25289569	0.435912	PPP1R1A
5	25189569	25289569	0.435912	PDE1B
5	25189569	25289569	0.435912	NCKAP1L
5	25189743	25289743	0.42666	GLYCAM1
5	25189743	25289743	0.42666	PPP1R1A
5	25189743	25289743	0.42666	PDE1B
5	25189743	25289743	0.42666	NCKAP1L
5	25190744	25290744	0.427037	GLYCAM1
5	25190744	25290744	0.427037	PPP1R1A
5	25190744	25290744	0.427037	PDE1B
5	25190744	25290744	0.427037	NCKAP1L
5	25191404	25291404	0.414917	GLYCAM1

5	25191404	25291404	0.414917	PPP1R1A
5	25191404	25291404	0.414917	PDE1B
5	25191404	25291404	0.414917	NCKAP1L
5	25192812	25292812	0.432951	GLYCAM1
5	25192812	25292812	0.432951	PPP1R1A
5	25192812	25292812	0.432951	PDE1B
5	25192812	25292812	0.432951	NCKAP1L
5	25192994	25292994	0.464888	GLYCAM1
5	25192994	25292994	0.464888	PPP1R1A
5	25192994	25292994	0.464888	PDE1B
5	25192994	25292994	0.464888	NCKAP1L
5	25193097	25293097	0.464106	GLYCAM1
5	25193097	25293097	0.464106	PPP1R1A
5	25193097	25293097	0.464106	PDE1B
5	25193097	25293097	0.464106	NCKAP1L
5	25193319	25293319	0.459029	GLYCAM1
5	25193319	25293319	0.459029	PPP1R1A
5	25193319	25293319	0.459029	PDE1B
5	25193319	25293319	0.459029	NCKAP1L
5	25193375	25293375	0.460921	GLYCAM1
5	25193375	25293375	0.460921	PPP1R1A
5	25193375	25293375	0.460921	PDE1B
5	25193375	25293375	0.460921	NCKAP1L
5	25193607	25293607	0.466593	GLYCAM1
5	25193607	25293607	0.466593	PPP1R1A
5	25193607	25293607	0.466593	PDE1B
5	25193607	25293607	0.466593	NCKAP1L
5	25194203	25294203	0.449992	GLYCAM1
5	25194203	25294203	0.449992	PPP1R1A
5	25194203	25294203	0.449992	PDE1B
5	25194203	25294203	0.449992	NCKAP1L
5	25194225	25294225	0.461568	GLYCAM1
5	25194225	25294225	0.461568	PPP1R1A
5	25194225	25294225	0.461568	PDE1B
5	25194225	25294225	0.461568	NCKAP1L
5	25194253	25294253	0.468101	GLYCAM1
5	25194253	25294253	0.468101	PPP1R1A
5	25194253	25294253	0.468101	PDE1B
5	25194253	25294253	0.468101	NCKAP1L
5	25194301	25294301	0.45786	GLYCAM1
5	25194301	25294301	0.45786	PPP1R1A
5	25194301	25294301	0.45786	PDE1B
5	25194301	25294301	0.45786	NCKAP1L
5	25194347	25294347	0.455476	GLYCAM1
5	25194347	25294347	0.455476	PPP1R1A
5	25194347	25294347	0.455476	PDE1B
5	25194347	25294347	0.455476	NCKAP1L
5	25194361	25294361	0.454252	GLYCAM1
5	25194361	25294361	0.454252	PPP1R1A
5	25194361	25294361	0.454252	PDE1B
5	25194361	25294361	0.454252	NCKAP1L
5	25194930	25294930	0.424997	GLYCAM1
5	25194930	25294930	0.424997	PPP1R1A
5	25194930	25294930	0.424997	PDE1B
5	25194930	25294930	0.424997	NCKAP1L
5	25195263	25295263	0.427908	GLYCAM1
5	25195263	25295263	0.427908	PPP1R1A
5	25195263	25295263	0.427908	PDE1B

5	25195263	25295263	0.427908	NCKAP1L
5	25195349	25295349	0.422084	GLYCAM1
5	25195349	25295349	0.422084	PPP1R1A
5	25195349	25295349	0.422084	PDE1B
5	25195349	25295349	0.422084	NCKAP1L
5	25195361	25295361	0.443455	GLYCAM1
5	25195361	25295361	0.443455	PPP1R1A
5	25195361	25295361	0.443455	PDE1B
5	25195361	25295361	0.443455	NCKAP1L
5	25195433	25295433	0.405272	GLYCAM1
5	25195433	25295433	0.405272	PPP1R1A
5	25195433	25295433	0.405272	PDE1B
5	25195433	25295433	0.405272	NCKAP1L
5	25196238	25296238	0.395817	GLYCAM1
5	25196238	25296238	0.395817	PPP1R1A
5	25196238	25296238	0.395817	PDE1B
5	25196238	25296238	0.395817	NCKAP1L
5	25196643	25296643	0.40902	GLYCAM1
5	25196643	25296643	0.40902	PPP1R1A
5	25196643	25296643	0.40902	PDE1B
5	25196643	25296643	0.40902	NCKAP1L
5	25196668	25296668	0.404182	GLYCAM1
5	25196668	25296668	0.404182	PPP1R1A
5	25196668	25296668	0.404182	PDE1B
5	25196668	25296668	0.404182	NCKAP1L
5	25197398	25297398	0.397913	GLYCAM1
5	25197398	25297398	0.397913	PPP1R1A
5	25197398	25297398	0.397913	PDE1B
5	25197398	25297398	0.397913	NCKAP1L
5	25197552	25297552	0.39298	GLYCAM1
5	25197552	25297552	0.39298	PPP1R1A
5	25197552	25297552	0.39298	PDE1B
5	25197552	25297552	0.39298	NCKAP1L
5	25198097	25298097	0.396692	GLYCAM1
5	25198097	25298097	0.396692	PPP1R1A
5	25198097	25298097	0.396692	PDE1B
5	25198097	25298097	0.396692	NCKAP1L
5	25198277	25298277	0.397017	GLYCAM1
5	25198277	25298277	0.397017	PPP1R1A
5	25198277	25298277	0.397017	PDE1B
5	25198277	25298277	0.397017	NCKAP1L
5	25198749	25298749	0.399316	GLYCAM1
5	25198749	25298749	0.399316	PPP1R1A
5	25198749	25298749	0.399316	PDE1B
5	25198749	25298749	0.399316	NCKAP1L
5	25198806	25298806	0.40733	GLYCAM1
5	25198806	25298806	0.40733	PPP1R1A
5	25198806	25298806	0.40733	PDE1B
5	25198806	25298806	0.40733	NCKAP1L
5	25198821	25298821	0.405651	GLYCAM1
5	25198821	25298821	0.405651	PPP1R1A
5	25198821	25298821	0.405651	PDE1B
5	25198821	25298821	0.405651	NCKAP1L
5	25198899	25298899	0.404709	GLYCAM1
5	25198899	25298899	0.404709	PPP1R1A
5	25198899	25298899	0.404709	PDE1B
5	25198899	25298899	0.404709	NCKAP1L
5	25199122	25299122	0.433608	GLYCAM1

5	25199122	25299122	0.433608	PPP1R1A
5	25199122	25299122	0.433608	PDE1B
5	25199122	25299122	0.433608	NCKAP1L
5	25199154	25299154	0.433251	GLYCAM1
5	25199154	25299154	0.433251	PPP1R1A
5	25199154	25299154	0.433251	PDE1B
5	25199154	25299154	0.433251	NCKAP1L
5	25199181	25299181	0.434955	GLYCAM1
5	25199181	25299181	0.434955	PPP1R1A
5	25199181	25299181	0.434955	PDE1B
5	25199181	25299181	0.434955	NCKAP1L
5	25199295	25299295	0.45896	GLYCAM1
5	25199295	25299295	0.45896	PPP1R1A
5	25199295	25299295	0.45896	PDE1B
5	25199295	25299295	0.45896	NCKAP1L
5	25199340	25299340	0.452688	GLYCAM1
5	25199340	25299340	0.452688	PPP1R1A
5	25199340	25299340	0.452688	PDE1B
5	25199340	25299340	0.452688	NCKAP1L
5	25199461	25299461	0.455893	GLYCAM1
5	25199461	25299461	0.455893	PPP1R1A
5	25199461	25299461	0.455893	PDE1B
5	25199461	25299461	0.455893	NCKAP1L
5	25199606	25299606	0.43947	GLYCAM1
5	25199606	25299606	0.43947	PPP1R1A
5	25199606	25299606	0.43947	PDE1B
5	25199606	25299606	0.43947	NCKAP1L
5	25199708	25299708	0.438095	GLYCAM1
5	25199708	25299708	0.438095	PPP1R1A
5	25199708	25299708	0.438095	PDE1B
5	25199708	25299708	0.438095	NCKAP1L
5	25199765	25299765	0.438042	GLYCAM1
5	25199765	25299765	0.438042	PPP1R1A
5	25199765	25299765	0.438042	PDE1B
5	25199765	25299765	0.438042	NCKAP1L
5	25199768	25299768	0.438586	GLYCAM1
5	25199768	25299768	0.438586	PPP1R1A
5	25199768	25299768	0.438586	PDE1B
5	25199768	25299768	0.438586	NCKAP1L
5	25199770	25299770	0.438534	GLYCAM1
5	25199770	25299770	0.438534	PPP1R1A
5	25199770	25299770	0.438534	PDE1B
5	25199770	25299770	0.438534	NCKAP1L
5	25199817	25299817	0.433963	GLYCAM1
5	25199817	25299817	0.433963	PPP1R1A
5	25199817	25299817	0.433963	PDE1B
5	25199817	25299817	0.433963	NCKAP1L
5	25199866	25299866	0.432923	GLYCAM1
5	25199866	25299866	0.432923	PPP1R1A
5	25199866	25299866	0.432923	PDE1B
5	25199866	25299866	0.432923	NCKAP1L
5	25199974	25299974	0.437269	GLYCAM1
5	25199974	25299974	0.437269	PPP1R1A
5	25199974	25299974	0.437269	PDE1B
5	25199974	25299974	0.437269	NCKAP1L
5	25200331	25300331	0.408259	GLYCAM1
5	25200331	25300331	0.408259	PPP1R1A
5	25200331	25300331	0.408259	PDE1B

5	25200331	25300331	0.408259	NCKAP1L
5	25200502	25300502	0.409189	GLYCAM1
5	25200502	25300502	0.409189	PPP1R1A
5	25200502	25300502	0.409189	PDE1B
5	25200502	25300502	0.409189	NCKAP1L
5	25200546	25300546	0.407028	GLYCAM1
5	25200546	25300546	0.407028	PPP1R1A
5	25200546	25300546	0.407028	PDE1B
5	25200546	25300546	0.407028	NCKAP1L
5	25200845	25300845	0.402897	GLYCAM1
5	25200845	25300845	0.402897	PPP1R1A
5	25200845	25300845	0.402897	PDE1B
5	25200845	25300845	0.402897	NCKAP1L
5	25200865	25300865	0.406041	GLYCAM1
5	25200865	25300865	0.406041	PPP1R1A
5	25200865	25300865	0.406041	PDE1B
5	25200865	25300865	0.406041	NCKAP1L
5	25202307	25302307	0.38504	GLYCAM1
5	25202307	25302307	0.38504	PPP1R1A
5	25202307	25302307	0.38504	PDE1B
5	25202307	25302307	0.38504	NCKAP1L
5	25202979	25302979	0.38504	GLYCAM1
5	25202979	25302979	0.38504	PPP1R1A
5	25202979	25302979	0.38504	PDE1B
5	25202979	25302979	0.38504	NCKAP1L
5	25203032	25303032	0.38504	GLYCAM1
5	25203032	25303032	0.38504	PPP1R1A
5	25203032	25303032	0.38504	PDE1B
5	25203032	25303032	0.38504	NCKAP1L
5	25203082	25303082	0.385785	GLYCAM1
5	25203082	25303082	0.385785	PPP1R1A
5	25203082	25303082	0.385785	PDE1B
5	25203082	25303082	0.385785	NCKAP1L
5	25203500	25303500	0.413881	GLYCAM1
5	25203500	25303500	0.413881	PPP1R1A
5	25203500	25303500	0.413881	PDE1B
5	25203500	25303500	0.413881	NCKAP1L
5	25203568	25303568	0.411119	GLYCAM1
5	25203568	25303568	0.411119	PPP1R1A
5	25203568	25303568	0.411119	PDE1B
5	25203568	25303568	0.411119	NCKAP1L
5	25203613	25303613	0.393474	GLYCAM1
5	25203613	25303613	0.393474	PPP1R1A
5	25203613	25303613	0.393474	PDE1B
5	25203613	25303613	0.393474	NCKAP1L
5	25204260	25304260	0.396651	GLYCAM1
5	25204260	25304260	0.396651	PPP1R1A
5	25204260	25304260	0.396651	PDE1B
5	25204260	25304260	0.396651	NCKAP1L
5	25204664	25304664	0.397794	GLYCAM1
5	25204664	25304664	0.397794	PPP1R1A
5	25204664	25304664	0.397794	PDE1B
5	25204664	25304664	0.397794	NCKAP1L
5	25204791	25304791	0.405175	GLYCAM1
5	25204791	25304791	0.405175	PPP1R1A
5	25204791	25304791	0.405175	PDE1B
5	25204791	25304791	0.405175	NCKAP1L
5	25204799	25304799	0.405674	GLYCAM1

5	25204799	25304799	0.405674	PPP1R1A
5	25204799	25304799	0.405674	PDE1B
5	25204799	25304799	0.405674	NCKAP1L
5	25204809	25304809	0.400714	GLYCAM1
5	25204809	25304809	0.400714	PPP1R1A
5	25204809	25304809	0.400714	PDE1B
5	25204809	25304809	0.400714	NCKAP1L
5	25205232	25305232	0.406991	GLYCAM1
5	25205232	25305232	0.406991	PPP1R1A
5	25205232	25305232	0.406991	PDE1B
5	25205232	25305232	0.406991	NCKAP1L
5	25205663	25305663	0.387041	GLYCAM1
5	25205663	25305663	0.387041	PPP1R1A
5	25205663	25305663	0.387041	PDE1B
5	25205663	25305663	0.387041	NCKAP1L
5	25205841	25305841	0.387041	GLYCAM1
5	25205841	25305841	0.387041	PPP1R1A
5	25205841	25305841	0.387041	PDE1B
5	25205841	25305841	0.387041	NCKAP1L
5	25205845	25305845	0.38631	GLYCAM1
5	25205845	25305845	0.38631	PPP1R1A
5	25205845	25305845	0.38631	PDE1B
5	25205845	25305845	0.38631	NCKAP1L
5	25205857	25305857	0.39621	GLYCAM1
5	25205857	25305857	0.39621	PPP1R1A
5	25205857	25305857	0.39621	PDE1B
5	25205857	25305857	0.39621	NCKAP1L
5	25205935	25305935	0.392952	GLYCAM1
5	25205935	25305935	0.392952	PPP1R1A
5	25205935	25305935	0.392952	PDE1B
5	25205935	25305935	0.392952	NCKAP1L
5	25205939	25305939	0.392952	GLYCAM1
5	25205939	25305939	0.392952	PPP1R1A
5	25205939	25305939	0.392952	PDE1B
5	25205939	25305939	0.392952	NCKAP1L
5	25206255	25306255	0.395077	GLYCAM1
5	25206255	25306255	0.395077	PPP1R1A
5	25206255	25306255	0.395077	PDE1B
5	25206255	25306255	0.395077	NCKAP1L
5	25206338	25306338	0.394838	GLYCAM1
5	25206338	25306338	0.394838	PPP1R1A
5	25206338	25306338	0.394838	PDE1B
5	25206338	25306338	0.394838	NCKAP1L
5	25206729	25306729	0.39407	GLYCAM1
5	25206729	25306729	0.39407	PPP1R1A
5	25206729	25306729	0.39407	PDE1B
5	25206729	25306729	0.39407	NCKAP1L
5	25206788	25306788	0.401158	GLYCAM1
5	25206788	25306788	0.401158	PPP1R1A
5	25206788	25306788	0.401158	PDE1B
5	25206788	25306788	0.401158	NCKAP1L
5	25206803	25306803	0.401158	GLYCAM1
5	25206803	25306803	0.401158	PPP1R1A
5	25206803	25306803	0.401158	PDE1B
5	25206803	25306803	0.401158	NCKAP1L
5	25206825	25306825	0.401158	GLYCAM1
5	25206825	25306825	0.401158	PPP1R1A
5	25206825	25306825	0.401158	PDE1B

5	25206825	25306825	0.401158	NCKAP1L
5	25207052	25307052	0.387921	GLYCAM1
5	25207052	25307052	0.387921	PPP1R1A
5	25207052	25307052	0.387921	PDE1B
5	25207052	25307052	0.387921	NCKAP1L
5	25207359	25307359	0.403318	GLYCAM1
5	25207359	25307359	0.403318	PPP1R1A
5	25207359	25307359	0.403318	PDE1B
5	25207359	25307359	0.403318	NCKAP1L
5	25207499	25307499	0.39982	GLYCAM1
5	25207499	25307499	0.39982	PPP1R1A
5	25207499	25307499	0.39982	PDE1B
5	25207499	25307499	0.39982	NCKAP1L
5	25207681	25307681	0.405519	GLYCAM1
5	25207681	25307681	0.405519	PPP1R1A
5	25207681	25307681	0.405519	PDE1B
5	25207681	25307681	0.405519	NCKAP1L
5	25207702	25307702	0.399735	GLYCAM1
5	25207702	25307702	0.399735	PPP1R1A
5	25207702	25307702	0.399735	PDE1B
5	25207702	25307702	0.399735	NCKAP1L
5	25208021	25308021	0.399735	GLYCAM1
5	25208021	25308021	0.399735	PPP1R1A
5	25208021	25308021	0.399735	PDE1B
5	25208021	25308021	0.399735	NCKAP1L
5	25208229	25308229	0.403168	GLYCAM1
5	25208229	25308229	0.403168	PPP1R1A
5	25208229	25308229	0.403168	PDE1B
5	25208229	25308229	0.403168	NCKAP1L
5	25208248	25308248	0.420984	GLYCAM1
5	25208248	25308248	0.420984	PPP1R1A
5	25208248	25308248	0.420984	PDE1B
5	25208248	25308248	0.420984	NCKAP1L
5	25208484	25308484	0.415137	GLYCAM1
5	25208484	25308484	0.415137	PPP1R1A
5	25208484	25308484	0.415137	PDE1B
5	25208484	25308484	0.415137	NCKAP1L
5	25209308	25309308	0.408801	GLYCAM1
5	25209308	25309308	0.408801	PPP1R1A
5	25209308	25309308	0.408801	PDE1B
5	25209308	25309308	0.408801	NCKAP1L
5	25209453	25309453	0.406403	GLYCAM1
5	25209453	25309453	0.406403	PPP1R1A
5	25209453	25309453	0.406403	PDE1B
5	25209453	25309453	0.406403	NCKAP1L
5	25209665	25309665	0.406403	GLYCAM1
5	25209665	25309665	0.406403	PPP1R1A
5	25209665	25309665	0.406403	PDE1B
5	25209665	25309665	0.406403	NCKAP1L
5	25210198	25310198	0.404813	GLYCAM1
5	25210198	25310198	0.404813	PPP1R1A
5	25210198	25310198	0.404813	PDE1B
5	25210198	25310198	0.404813	NCKAP1L
5	25210199	25310199	0.438351	GLYCAM1
5	25210199	25310199	0.438351	PPP1R1A
5	25210199	25310199	0.438351	PDE1B
5	25210199	25310199	0.438351	NCKAP1L
5	25210521	25310521	0.417469	GLYCAM1

5	25210521	25310521	0.417469	PPP1R1A
5	25210521	25310521	0.417469	PDE1B
5	25210521	25310521	0.417469	NCKAP1L
5	25210770	25310770	0.418048	GLYCAM1
5	25210770	25310770	0.418048	PPP1R1A
5	25210770	25310770	0.418048	PDE1B
5	25210770	25310770	0.418048	NCKAP1L
5	25210806	25310806	0.417099	GLYCAM1
5	25210806	25310806	0.417099	PPP1R1A
5	25210806	25310806	0.417099	PDE1B
5	25210806	25310806	0.417099	NCKAP1L
5	25210831	25310831	0.417099	GLYCAM1
5	25210831	25310831	0.417099	PPP1R1A
5	25210831	25310831	0.417099	PDE1B
5	25210831	25310831	0.417099	NCKAP1L
5	25210837	25310837	0.416863	GLYCAM1
5	25210837	25310837	0.416863	PPP1R1A
5	25210837	25310837	0.416863	PDE1B
5	25210837	25310837	0.416863	NCKAP1L
5	25210877	25310877	0.419521	GLYCAM1
5	25210877	25310877	0.419521	PPP1R1A
5	25210877	25310877	0.419521	PDE1B
5	25210877	25310877	0.419521	NCKAP1L
5	25210888	25310888	0.436647	GLYCAM1
5	25210888	25310888	0.436647	PPP1R1A
5	25210888	25310888	0.436647	PDE1B
5	25210888	25310888	0.436647	NCKAP1L
5	25211001	25311001	0.436647	GLYCAM1
5	25211001	25311001	0.436647	PPP1R1A
5	25211001	25311001	0.436647	PDE1B
5	25211001	25311001	0.436647	NCKAP1L
5	25211134	25311134	0.426826	GLYCAM1
5	25211134	25311134	0.426826	PPP1R1A
5	25211134	25311134	0.426826	PDE1B
5	25211134	25311134	0.426826	NCKAP1L
5	25211238	25311238	0.429595	GLYCAM1
5	25211238	25311238	0.429595	PPP1R1A
5	25211238	25311238	0.429595	PDE1B
5	25211238	25311238	0.429595	NCKAP1L
5	25211496	25311496	0.426353	GLYCAM1
5	25211496	25311496	0.426353	PPP1R1A
5	25211496	25311496	0.426353	PDE1B
5	25211496	25311496	0.426353	NCKAP1L
5	25211817	25311817	0.426719	GLYCAM1
5	25211817	25311817	0.426719	PPP1R1A
5	25211817	25311817	0.426719	PDE1B
5	25211817	25311817	0.426719	NCKAP1L
5	25211941	25311941	0.428232	GLYCAM1
5	25211941	25311941	0.428232	PPP1R1A
5	25211941	25311941	0.428232	PDE1B
5	25211941	25311941	0.428232	NCKAP1L
5	25212239	25312239	0.407681	GLYCAM1
5	25212239	25312239	0.407681	PPP1R1A
5	25212239	25312239	0.407681	PDE1B
5	25212239	25312239	0.407681	NCKAP1L
5	25212321	25312321	0.416328	GLYCAM1
5	25212321	25312321	0.416328	PPP1R1A
5	25212321	25312321	0.416328	PDE1B

5	25212321	25312321	0.416328	NCKAP1L
5	25212442	25312442	0.416682	GLYCAM1
5	25212442	25312442	0.416682	PPP1R1A
5	25212442	25312442	0.416682	PDE1B
5	25212442	25312442	0.416682	NCKAP1L
5	25212499	25312499	0.401783	GLYCAM1
5	25212499	25312499	0.401783	PPP1R1A
5	25212499	25312499	0.401783	PDE1B
5	25212499	25312499	0.401783	NCKAP1L
5	25212731	25312731	0.40374	GLYCAM1
5	25212731	25312731	0.40374	PPP1R1A
5	25212731	25312731	0.40374	PDE1B
5	25212731	25312731	0.40374	NCKAP1L
5	25212817	25312817	0.392144	GLYCAM1
5	25212817	25312817	0.392144	PPP1R1A
5	25212817	25312817	0.392144	PDE1B
5	25212817	25312817	0.392144	NCKAP1L
5	25213006	25313006	0.391441	GLYCAM1
5	25213006	25313006	0.391441	PPP1R1A
5	25213006	25313006	0.391441	PDE1B
5	25213006	25313006	0.391441	NCKAP1L
5	25213036	25313036	0.398003	GLYCAM1
5	25213036	25313036	0.398003	PPP1R1A
5	25213036	25313036	0.398003	PDE1B
5	25213036	25313036	0.398003	NCKAP1L
5	25213182	25313182	0.393664	GLYCAM1
5	25213182	25313182	0.393664	PPP1R1A
5	25213182	25313182	0.393664	PDE1B
5	25213182	25313182	0.393664	NCKAP1L
5	25213234	25313234	0.393664	GLYCAM1
5	25213234	25313234	0.393664	PPP1R1A
5	25213234	25313234	0.393664	PDE1B
5	25213234	25313234	0.393664	NCKAP1L
5	25213272	25313272	0.393901	GLYCAM1
5	25213272	25313272	0.393901	PPP1R1A
5	25213272	25313272	0.393901	PDE1B
5	25213272	25313272	0.393901	NCKAP1L
5	25213328	25313328	0.394097	GLYCAM1
5	25213328	25313328	0.394097	PPP1R1A
5	25213328	25313328	0.394097	PDE1B
5	25213328	25313328	0.394097	NCKAP1L
5	25213491	25313491	0.398442	GLYCAM1
5	25213491	25313491	0.398442	PPP1R1A
5	25213491	25313491	0.398442	PDE1B
5	25213491	25313491	0.398442	NCKAP1L
5	25213712	25313712	0.38775	GLYCAM1
5	25213712	25313712	0.38775	PPP1R1A
5	25213712	25313712	0.38775	PDE1B
5	25213712	25313712	0.38775	NCKAP1L
5	25213813	25313813	0.391512	GLYCAM1
5	25213813	25313813	0.391512	PPP1R1A
5	25213813	25313813	0.391512	PDE1B
5	25213813	25313813	0.391512	NCKAP1L
5	25213974	25313974	0.37187	GLYCAM1
5	25213974	25313974	0.37187	PPP1R1A
5	25213974	25313974	0.37187	PDE1B
5	25213974	25313974	0.37187	NCKAP1L
5	25214038	25314038	0.37323	GLYCAM1

5	25214038	25314038	0.37323	PPP1R1A
5	25214038	25314038	0.37323	PDE1B
5	25214038	25314038	0.37323	NCKAP1L
5	25214487	25314487	0.372556	GLYCAM1
5	25214487	25314487	0.372556	PPP1R1A
5	25214487	25314487	0.372556	PDE1B
5	25214487	25314487	0.372556	NCKAP1L
5	25214565	25314565	0.376794	GLYCAM1
5	25214565	25314565	0.376794	PPP1R1A
5	25214565	25314565	0.376794	PDE1B
5	25214565	25314565	0.376794	NCKAP1L
5	25214812	25314812	0.396921	GLYCAM1
5	25214812	25314812	0.396921	PPP1R1A
5	25214812	25314812	0.396921	PDE1B
5	25214812	25314812	0.396921	NCKAP1L
5	25215099	25315099	0.397205	GLYCAM1
5	25215099	25315099	0.397205	PPP1R1A
5	25215099	25315099	0.397205	PDE1B
5	25215099	25315099	0.397205	NCKAP1L
5	25215542	25315542	0.383218	GLYCAM1
5	25215542	25315542	0.383218	PPP1R1A
5	25215542	25315542	0.383218	PDE1B
5	25215542	25315542	0.383218	NCKAP1L
5	25215680	25315680	0.383568	GLYCAM1
5	25215680	25315680	0.383568	PPP1R1A
5	25215680	25315680	0.383568	PDE1B
5	25215680	25315680	0.383568	NCKAP1L
5	25215954	25315954	0.387425	GLYCAM1
5	25215954	25315954	0.387425	PPP1R1A
5	25215954	25315954	0.387425	PDE1B
5	25215954	25315954	0.387425	NCKAP1L
5	25216317	25316317	0.394476	GLYCAM1
5	25216317	25316317	0.394476	PPP1R1A
5	25216317	25316317	0.394476	PDE1B
5	25216317	25316317	0.394476	NCKAP1L
5	25216542	25316542	0.404089	GLYCAM1
5	25216542	25316542	0.404089	PPP1R1A
5	25216542	25316542	0.404089	PDE1B
5	25216542	25316542	0.404089	NCKAP1L
5	25216550	25316550	0.400221	GLYCAM1
5	25216550	25316550	0.400221	PPP1R1A
5	25216550	25316550	0.400221	PDE1B
5	25216550	25316550	0.400221	NCKAP1L
5	25216567	25316567	0.400781	GLYCAM1
5	25216567	25316567	0.400781	PPP1R1A
5	25216567	25316567	0.400781	PDE1B
5	25216567	25316567	0.400781	NCKAP1L
5	25216644	25316644	0.400298	GLYCAM1
5	25216644	25316644	0.400298	PPP1R1A
5	25216644	25316644	0.400298	PDE1B
5	25216644	25316644	0.400298	NCKAP1L
5	25216653	25316653	0.397692	GLYCAM1
5	25216653	25316653	0.397692	PPP1R1A
5	25216653	25316653	0.397692	PDE1B
5	25216653	25316653	0.397692	NCKAP1L
5	25216862	25316862	0.392363	GLYCAM1
5	25216862	25316862	0.392363	PPP1R1A
5	25216862	25316862	0.392363	PDE1B

5	25216862	25316862	0.392363	NCKAP1L
5	25217033	25317033	0.392363	GLYCAM1
5	25217033	25317033	0.392363	PPP1R1A
5	25217033	25317033	0.392363	PDE1B
5	25217033	25317033	0.392363	NCKAP1L
5	25217083	25317083	0.398164	GLYCAM1
5	25217083	25317083	0.398164	PPP1R1A
5	25217083	25317083	0.398164	PDE1B
5	25217083	25317083	0.398164	NCKAP1L
5	25217539	25317539	0.398313	GLYCAM1
5	25217539	25317539	0.398313	PPP1R1A
5	25217539	25317539	0.398313	PDE1B
5	25217539	25317539	0.398313	NCKAP1L
5	25217689	25317689	0.403392	GLYCAM1
5	25217689	25317689	0.403392	PPP1R1A
5	25217689	25317689	0.403392	PDE1B
5	25217689	25317689	0.403392	NCKAP1L
5	25217742	25317742	0.403358	GLYCAM1
5	25217742	25317742	0.403358	PPP1R1A
5	25217742	25317742	0.403358	PDE1B
5	25217742	25317742	0.403358	NCKAP1L
5	25217996	25317996	0.410246	GLYCAM1
5	25217996	25317996	0.410246	PPP1R1A
5	25217996	25317996	0.410246	PDE1B
5	25217996	25317996	0.410246	NCKAP1L
5	25217997	25317997	0.410246	GLYCAM1
5	25217997	25317997	0.410246	PPP1R1A
5	25217997	25317997	0.410246	PDE1B
5	25217997	25317997	0.410246	NCKAP1L
5	25218010	25318010	0.387327	GLYCAM1
5	25218010	25318010	0.387327	PPP1R1A
5	25218010	25318010	0.387327	PDE1B
5	25218010	25318010	0.387327	NCKAP1L
5	25218278	25318278	0.387034	GLYCAM1
5	25218278	25318278	0.387034	PPP1R1A
5	25218278	25318278	0.387034	PDE1B
5	25218278	25318278	0.387034	NCKAP1L
5	25218646	25318646	0.393593	GLYCAM1
5	25218646	25318646	0.393593	PPP1R1A
5	25218646	25318646	0.393593	PDE1B
5	25218646	25318646	0.393593	NCKAP1L
5	25218728	25318728	0.391519	GLYCAM1
5	25218728	25318728	0.391519	PPP1R1A
5	25218728	25318728	0.391519	PDE1B
5	25218728	25318728	0.391519	NCKAP1L
5	25218826	25318826	0.39173	GLYCAM1
5	25218826	25318826	0.39173	PPP1R1A
5	25218826	25318826	0.39173	PDE1B
5	25218826	25318826	0.39173	NCKAP1L
5	25218924	25318924	0.363477	GLYCAM1
5	25218924	25318924	0.363477	PPP1R1A
5	25218924	25318924	0.363477	PDE1B
5	25218924	25318924	0.363477	NCKAP1L
5	25219129	25319129	0.365434	GLYCAM1
5	25219129	25319129	0.365434	PPP1R1A
5	25219129	25319129	0.365434	PDE1B
5	25219129	25319129	0.365434	NCKAP1L
5	25219175	25319175	0.365204	GLYCAM1

5	25219175	25319175	0.365204	PPP1R1A
5	25219175	25319175	0.365204	PDE1B
5	25219175	25319175	0.365204	NCKAP1L
5	25219222	25319222	0.368182	GLYCAM1
5	25219222	25319222	0.368182	PPP1R1A
5	25219222	25319222	0.368182	PDE1B
5	25219222	25319222	0.368182	NCKAP1L
5	25219368	25319368	0.362714	GLYCAM1
5	25219368	25319368	0.362714	PPP1R1A
5	25219368	25319368	0.362714	PDE1B
5	25219368	25319368	0.362714	NCKAP1L
5	25220032	25320032	0.36555	GLYCAM1
5	25220032	25320032	0.36555	PPP1R1A
5	25220032	25320032	0.36555	PDE1B
5	25220032	25320032	0.36555	NCKAP1L
5	25220043	25320043	0.369512	GLYCAM1
5	25220043	25320043	0.369512	PPP1R1A
5	25220043	25320043	0.369512	PDE1B
5	25220043	25320043	0.369512	NCKAP1L
5	25220170	25320170	0.356128	GLYCAM1
5	25220170	25320170	0.356128	PPP1R1A
5	25220170	25320170	0.356128	PDE1B
5	25220170	25320170	0.356128	NCKAP1L
5	25220522	25320522	0.370191	PPP1R1A
5	25220522	25320522	0.370191	PDE1B
5	25220522	25320522	0.370191	NCKAP1L
5	25220682	25320682	0.373581	PPP1R1A
5	25220682	25320682	0.373581	PDE1B
5	25220682	25320682	0.373581	NCKAP1L
5	25220698	25320698	0.373581	PPP1R1A
5	25220698	25320698	0.373581	PDE1B
5	25220698	25320698	0.373581	NCKAP1L
5	25220746	25320746	0.371933	PPP1R1A
5	25220746	25320746	0.371933	PDE1B
5	25220746	25320746	0.371933	NCKAP1L
5	25220762	25320762	0.383132	PPP1R1A
5	25220762	25320762	0.383132	PDE1B
5	25220762	25320762	0.383132	NCKAP1L
5	25220774	25320774	0.377151	PPP1R1A
5	25220774	25320774	0.377151	PDE1B
5	25220774	25320774	0.377151	NCKAP1L
5	25221184	25321184	0.378202	PPP1R1A
5	25221184	25321184	0.378202	PDE1B
5	25221184	25321184	0.378202	NCKAP1L
5	25221385	25321385	0.361923	PPP1R1A
5	25221385	25321385	0.361923	PDE1B
5	25221385	25321385	0.361923	NCKAP1L
5	25221900	25321900	0.359825	PPP1R1A
5	25221900	25321900	0.359825	PDE1B
5	25221900	25321900	0.359825	NCKAP1L
5	25222591	25322591	0.35428	PPP1R1A
5	25222591	25322591	0.35428	PDE1B
5	25222591	25322591	0.35428	NCKAP1L
5	25222666	25322666	0.408017	PPP1R1A
5	25222666	25322666	0.408017	PDE1B
5	25222666	25322666	0.408017	NCKAP1L
5	25222858	25322858	0.395752	PPP1R1A
5	25222858	25322858	0.395752	PDE1B

5	25222858	25322858	0.395752	NCKAP1L
5	25223161	25323161	0.395414	PPP1R1A
5	25223161	25323161	0.395414	PDE1B
5	25223161	25323161	0.395414	NCKAP1L
5	25223167	25323167	0.396219	PPP1R1A
5	25223167	25323167	0.396219	PDE1B
5	25223167	25323167	0.396219	NCKAP1L
5	25223254	25323254	0.432542	PPP1R1A
5	25223254	25323254	0.432542	PDE1B
5	25223254	25323254	0.432542	NCKAP1L
5	25223287	25323287	0.400179	PPP1R1A
5	25223287	25323287	0.400179	PDE1B
5	25223287	25323287	0.400179	NCKAP1L
5	25223697	25323697	0.400562	PPP1R1A
5	25223697	25323697	0.400562	PDE1B
5	25223697	25323697	0.400562	NCKAP1L
5	25223831	25323831	0.409155	PPP1R1A
5	25223831	25323831	0.409155	PDE1B
5	25223831	25323831	0.409155	NCKAP1L
5	25223863	25323863	0.397509	PPP1R1A
5	25223863	25323863	0.397509	PDE1B
5	25223863	25323863	0.397509	NCKAP1L
5	25224028	25324028	0.398892	PPP1R1A
5	25224028	25324028	0.398892	PDE1B
5	25224028	25324028	0.398892	NCKAP1L
5	25224166	25324166	0.394004	PPP1R1A
5	25224166	25324166	0.394004	PDE1B
5	25224166	25324166	0.394004	NCKAP1L
5	25224228	25324228	0.394517	PPP1R1A
5	25224228	25324228	0.394517	PDE1B
5	25224228	25324228	0.394517	NCKAP1L
5	25224342	25324342	0.418133	PPP1R1A
5	25224342	25324342	0.418133	PDE1B
5	25224342	25324342	0.418133	NCKAP1L
5	25224484	25324484	0.418747	PPP1R1A
5	25224484	25324484	0.418747	PDE1B
5	25224484	25324484	0.418747	NCKAP1L
5	25224489	25324489	0.418747	PPP1R1A
5	25224489	25324489	0.418747	PDE1B
5	25224489	25324489	0.418747	NCKAP1L
5	25224538	25324538	0.418755	PPP1R1A
5	25224538	25324538	0.418755	PDE1B
5	25224538	25324538	0.418755	NCKAP1L
5	25224799	25324799	0.38565	PPP1R1A
5	25224799	25324799	0.38565	PDE1B
5	25224799	25324799	0.38565	NCKAP1L
5	25225013	25325013	0.404713	PPP1R1A
5	25225013	25325013	0.404713	PDE1B
5	25225013	25325013	0.404713	NCKAP1L
5	25225276	25325276	0.379016	PPP1R1A
5	25225276	25325276	0.379016	PDE1B
5	25225276	25325276	0.379016	NCKAP1L
5	25225288	25325288	0.379016	PPP1R1A
5	25225288	25325288	0.379016	PDE1B
5	25225288	25325288	0.379016	NCKAP1L
5	28247742	28347742	0.35797	POU6F1
5	28247742	28347742	0.35797	CSRNP2
5	28247742	28347742	0.35797	TFCP2

5	28247959	28347959	0.374403	POU6F1
5	28247959	28347959	0.374403	CSRNP2
5	28247959	28347959	0.374403	TFCP2
5	28259125	28359125	0.348318	POU6F1
5	28259125	28359125	0.348318	CSRNP2
5	28259125	28359125	0.348318	LETMD1
5	28259125	28359125	0.348318	TFCP2
5	28259204	28359204	0.347206	POU6F1
5	28259204	28359204	0.347206	CSRNP2
5	28259204	28359204	0.347206	LETMD1
5	28259204	28359204	0.347206	TFCP2
5	28259227	28359227	0.346704	POU6F1
5	28259227	28359227	0.346704	CSRNP2
5	28259227	28359227	0.346704	LETMD1
5	28259227	28359227	0.346704	TFCP2
5	28259573	28359573	0.342632	POU6F1
5	28259573	28359573	0.342632	CSRNP2
5	28259573	28359573	0.342632	LETMD1
5	28259573	28359573	0.342632	TFCP2
5	28260466	28360466	0.357138	POU6F1
5	28260466	28360466	0.357138	CSRNP2
5	28260466	28360466	0.357138	LETMD1
5	28260466	28360466	0.357138	TFCP2
5	30446653	30546653	0.342649	PRKAG1
5	30446653	30546653	0.342649	DDN
5	30446653	30546653	0.342649	WNT10B
5	30446653	30546653	0.342649	CCDC65
5	30446653	30546653	0.342649	WNT1
5	30446653	30546653	0.342649	ARF3
5	30446653	30546653	0.342649	FKBP11
5	30446781	30546781	0.344437	PRKAG1
5	30446781	30546781	0.344437	DDN
5	30446781	30546781	0.344437	WNT10B
5	30446781	30546781	0.344437	CCDC65
5	30446781	30546781	0.344437	WNT1
5	30446781	30546781	0.344437	ARF3
5	30446781	30546781	0.344437	FKBP11
5	30448588	30548588	0.357574	PRKAG1
5	30448588	30548588	0.357574	DDN
5	30448588	30548588	0.357574	WNT10B
5	30448588	30548588	0.357574	CCDC65
5	30448588	30548588	0.357574	WNT1
5	30448588	30548588	0.357574	ARF3
5	30448588	30548588	0.357574	FKBP11
5	30448727	30548727	0.363965	PRKAG1
5	30448727	30548727	0.363965	DDN
5	30448727	30548727	0.363965	WNT10B
5	30448727	30548727	0.363965	CCDC65
5	30448727	30548727	0.363965	WNT1
5	30448727	30548727	0.363965	ARF3
5	30448727	30548727	0.363965	FKBP11
5	30448893	30548893	0.365369	PRKAG1
5	30448893	30548893	0.365369	DDN
5	30448893	30548893	0.365369	WNT10B
5	30448893	30548893	0.365369	CCDC65
5	30448893	30548893	0.365369	WNT1
5	30448893	30548893	0.365369	ARF3
5	30448893	30548893	0.365369	FKBP11

5	30449070	30549070	0.360755	PRKAG1
5	30449070	30549070	0.360755	DDN
5	30449070	30549070	0.360755	WNT10B
5	30449070	30549070	0.360755	CCDC65
5	30449070	30549070	0.360755	WNT1
5	30449070	30549070	0.360755	ARF3
5	30449070	30549070	0.360755	FKBP11
5	30449496	30549496	0.36341	PRKAG1
5	30449496	30549496	0.36341	DDN
5	30449496	30549496	0.36341	WNT10B
5	30449496	30549496	0.36341	CCDC65
5	30449496	30549496	0.36341	WNT1
5	30449496	30549496	0.36341	ARF3
5	30449496	30549496	0.36341	FKBP11
5	30449529	30549529	0.363996	PRKAG1
5	30449529	30549529	0.363996	DDN
5	30449529	30549529	0.363996	WNT10B
5	30449529	30549529	0.363996	CCDC65
5	30449529	30549529	0.363996	WNT1
5	30449529	30549529	0.363996	ARF3
5	30449529	30549529	0.363996	FKBP11
5	30449675	30549675	0.363622	PRKAG1
5	30449675	30549675	0.363622	DDN
5	30449675	30549675	0.363622	WNT10B
5	30449675	30549675	0.363622	CCDC65
5	30449675	30549675	0.363622	WNT1
5	30449675	30549675	0.363622	ARF3
5	30449675	30549675	0.363622	FKBP11
5	30449724	30549724	0.385201	PRKAG1
5	30449724	30549724	0.385201	DDN
5	30449724	30549724	0.385201	WNT10B
5	30449724	30549724	0.385201	CCDC65
5	30449724	30549724	0.385201	WNT1
5	30449724	30549724	0.385201	ARF3
5	30449724	30549724	0.385201	FKBP11
5	30449926	30549926	0.383242	PRKAG1
5	30449926	30549926	0.383242	DDN
5	30449926	30549926	0.383242	WNT10B
5	30449926	30549926	0.383242	CCDC65
5	30449926	30549926	0.383242	WNT1
5	30449926	30549926	0.383242	ARF3
5	30449926	30549926	0.383242	FKBP11
5	30450081	30550081	0.387116	PRKAG1
5	30450081	30550081	0.387116	DDN
5	30450081	30550081	0.387116	WNT10B
5	30450081	30550081	0.387116	CCDC65
5	30450081	30550081	0.387116	WNT1
5	30450081	30550081	0.387116	ARF3
5	30450081	30550081	0.387116	FKBP11
5	30450116	30550116	0.374189	PRKAG1
5	30450116	30550116	0.374189	DDN
5	30450116	30550116	0.374189	WNT10B
5	30450116	30550116	0.374189	CCDC65
5	30450116	30550116	0.374189	WNT1
5	30450116	30550116	0.374189	ARF3
5	30450116	30550116	0.374189	FKBP11
5	30450499	30550499	0.376698	PRKAG1
5	30450499	30550499	0.376698	DDN

5	30450499	30550499	0.376698	WNT10B
5	30450499	30550499	0.376698	CCDC65
5	30450499	30550499	0.376698	WNT1
5	30450499	30550499	0.376698	ARF3
5	30450499	30550499	0.376698	FKBP11
5	30450525	30550525	0.376372	PRKAG1
5	30450525	30550525	0.376372	DDN
5	30450525	30550525	0.376372	WNT10B
5	30450525	30550525	0.376372	CCDC65
5	30450525	30550525	0.376372	WNT1
5	30450525	30550525	0.376372	ARF3
5	30450525	30550525	0.376372	FKBP11
5	30450668	30550668	0.371006	PRKAG1
5	30450668	30550668	0.371006	DDN
5	30450668	30550668	0.371006	WNT10B
5	30450668	30550668	0.371006	CCDC65
5	30450668	30550668	0.371006	WNT1
5	30450668	30550668	0.371006	ARF3
5	30450668	30550668	0.371006	FKBP11
5	30450720	30550720	0.363329	PRKAG1
5	30450720	30550720	0.363329	DDN
5	30450720	30550720	0.363329	WNT10B
5	30450720	30550720	0.363329	CCDC65
5	30450720	30550720	0.363329	WNT1
5	30450720	30550720	0.363329	ARF3
5	30450720	30550720	0.363329	FKBP11
5	30450944	30550944	0.364954	PRKAG1
5	30450944	30550944	0.364954	DDN
5	30450944	30550944	0.364954	WNT10B
5	30450944	30550944	0.364954	CCDC65
5	30450944	30550944	0.364954	WNT1
5	30450944	30550944	0.364954	ARF3
5	30450944	30550944	0.364954	FKBP11
5	30451508	30551508	0.369283	PRKAG1
5	30451508	30551508	0.369283	DDN
5	30451508	30551508	0.369283	WNT10B
5	30451508	30551508	0.369283	CCDC65
5	30451508	30551508	0.369283	WNT1
5	30451508	30551508	0.369283	ARF3
5	30451508	30551508	0.369283	FKBP11
5	30451946	30551946	0.369047	PRKAG1
5	30451946	30551946	0.369047	DDN
5	30451946	30551946	0.369047	WNT10B
5	30451946	30551946	0.369047	CCDC65
5	30451946	30551946	0.369047	WNT1
5	30451946	30551946	0.369047	ARF3
5	30451946	30551946	0.369047	FKBP11
5	30452252	30552252	0.370142	PRKAG1
5	30452252	30552252	0.370142	DDN
5	30452252	30552252	0.370142	WNT10B
5	30452252	30552252	0.370142	CCDC65
5	30452252	30552252	0.370142	WNT1
5	30452252	30552252	0.370142	ARF3
5	30452252	30552252	0.370142	FKBP11
5	30452522	30552522	0.3656	PRKAG1
5	30452522	30552522	0.3656	DDN
5	30452522	30552522	0.3656	WNT10B
5	30452522	30552522	0.3656	CCDC65

5	30452522	30552522	0.3656	WNT1
5	30452522	30552522	0.3656	ARF3
5	30452522	30552522	0.3656	FKBP11
5	30452580	30552580	0.368135	PRKAG1
5	30452580	30552580	0.368135	DDN
5	30452580	30552580	0.368135	WNT10B
5	30452580	30552580	0.368135	CCDC65
5	30452580	30552580	0.368135	WNT1
5	30452580	30552580	0.368135	ARF3
5	30452580	30552580	0.368135	FKBP11
5	30452688	30552688	0.367053	PRKAG1
5	30452688	30552688	0.367053	DDN
5	30452688	30552688	0.367053	WNT10B
5	30452688	30552688	0.367053	CCDC65
5	30452688	30552688	0.367053	WNT1
5	30452688	30552688	0.367053	ARF3
5	30452688	30552688	0.367053	FKBP11
5	30452776	30552776	0.374622	PRKAG1
5	30452776	30552776	0.374622	DDN
5	30452776	30552776	0.374622	WNT10B
5	30452776	30552776	0.374622	CCDC65
5	30452776	30552776	0.374622	WNT1
5	30452776	30552776	0.374622	ARF3
5	30452776	30552776	0.374622	FKBP11
5	30452928	30552928	0.367926	PRKAG1
5	30452928	30552928	0.367926	DDN
5	30452928	30552928	0.367926	WNT10B
5	30452928	30552928	0.367926	CCDC65
5	30452928	30552928	0.367926	WNT1
5	30452928	30552928	0.367926	ARF3
5	30452928	30552928	0.367926	FKBP11
5	30453127	30553127	0.376043	PRKAG1
5	30453127	30553127	0.376043	DDN
5	30453127	30553127	0.376043	WNT10B
5	30453127	30553127	0.376043	CCDC65
5	30453127	30553127	0.376043	WNT1
5	30453127	30553127	0.376043	ARF3
5	30453127	30553127	0.376043	FKBP11
5	30453461	30553461	0.376143	PRKAG1
5	30453461	30553461	0.376143	DDN
5	30453461	30553461	0.376143	WNT10B
5	30453461	30553461	0.376143	CCDC65
5	30453461	30553461	0.376143	WNT1
5	30453461	30553461	0.376143	ARF3
5	30453461	30553461	0.376143	FKBP11
5	30453625	30553625	0.360517	PRKAG1
5	30453625	30553625	0.360517	DDN
5	30453625	30553625	0.360517	WNT10B
5	30453625	30553625	0.360517	CCDC65
5	30453625	30553625	0.360517	WNT1
5	30453625	30553625	0.360517	ARF3
5	30453625	30553625	0.360517	FKBP11
5	30453853	30553853	0.370011	PRKAG1
5	30453853	30553853	0.370011	DDN
5	30453853	30553853	0.370011	WNT10B
5	30453853	30553853	0.370011	CCDC65
5	30453853	30553853	0.370011	WNT1
5	30453853	30553853	0.370011	ARF3

5	30453853	30553853	0.370011	FKBP11
5	30454057	30554057	0.37841	PRKAG1
5	30454057	30554057	0.37841	DDN
5	30454057	30554057	0.37841	WNT10B
5	30454057	30554057	0.37841	CCDC65
5	30454057	30554057	0.37841	WNT1
5	30454057	30554057	0.37841	ARF3
5	30454057	30554057	0.37841	FKBP11
5	30454092	30554092	0.379803	PRKAG1
5	30454092	30554092	0.379803	DDN
5	30454092	30554092	0.379803	WNT10B
5	30454092	30554092	0.379803	CCDC65
5	30454092	30554092	0.379803	WNT1
5	30454092	30554092	0.379803	ARF3
5	30454092	30554092	0.379803	FKBP11
5	30454216	30554216	0.373776	PRKAG1
5	30454216	30554216	0.373776	DDN
5	30454216	30554216	0.373776	WNT10B
5	30454216	30554216	0.373776	CCDC65
5	30454216	30554216	0.373776	WNT1
5	30454216	30554216	0.373776	ARF3
5	30454216	30554216	0.373776	FKBP11
5	30454426	30554426	0.374126	PRKAG1
5	30454426	30554426	0.374126	DDN
5	30454426	30554426	0.374126	WNT10B
5	30454426	30554426	0.374126	CCDC65
5	30454426	30554426	0.374126	WNT1
5	30454426	30554426	0.374126	ARF3
5	30454426	30554426	0.374126	FKBP11
5	30454622	30554622	0.364532	PRKAG1
5	30454622	30554622	0.364532	DDN
5	30454622	30554622	0.364532	WNT10B
5	30454622	30554622	0.364532	CCDC65
5	30454622	30554622	0.364532	WNT1
5	30454622	30554622	0.364532	ARF3
5	30454622	30554622	0.364532	FKBP11
5	30454765	30554765	0.362534	PRKAG1
5	30454765	30554765	0.362534	DDN
5	30454765	30554765	0.362534	WNT10B
5	30454765	30554765	0.362534	CCDC65
5	30454765	30554765	0.362534	WNT1
5	30454765	30554765	0.362534	ARF3
5	30454765	30554765	0.362534	FKBP11
5	30455287	30555287	0.368332	PRKAG1
5	30455287	30555287	0.368332	DDN
5	30455287	30555287	0.368332	WNT10B
5	30455287	30555287	0.368332	CCDC65
5	30455287	30555287	0.368332	WNT1
5	30455287	30555287	0.368332	ARF3
5	30455287	30555287	0.368332	FKBP11
5	30455290	30555290	0.374671	PRKAG1
5	30455290	30555290	0.374671	DDN
5	30455290	30555290	0.374671	WNT10B
5	30455290	30555290	0.374671	CCDC65
5	30455290	30555290	0.374671	WNT1
5	30455290	30555290	0.374671	ARF3
5	30455290	30555290	0.374671	FKBP11
5	30455402	30555402	0.366579	PRKAG1

5	30455402	30555402	0.366579	DDN
5	30455402	30555402	0.366579	WNT10B
5	30455402	30555402	0.366579	CCDC65
5	30455402	30555402	0.366579	WNT1
5	30455402	30555402	0.366579	ARF3
5	30455402	30555402	0.366579	FKBP11
5	30455517	30555517	0.352698	PRKAG1
5	30455517	30555517	0.352698	DDN
5	30455517	30555517	0.352698	WNT10B
5	30455517	30555517	0.352698	CCDC65
5	30455517	30555517	0.352698	WNT1
5	30455517	30555517	0.352698	ARF3
5	30455517	30555517	0.352698	FKBP11
5	30459255	30559255	0.344627	DDN
5	30459255	30559255	0.344627	WNT10B
5	30459255	30559255	0.344627	CCDC65
5	30459255	30559255	0.344627	WNT1
5	30459255	30559255	0.344627	ARF3
5	30459255	30559255	0.344627	FKBP11
5	30467479	30567479	0.35678	WNT10B
5	30467479	30567479	0.35678	CCDC65
5	30467479	30567479	0.35678	RND1
5	30467479	30567479	0.35678	WNT1
5	30467479	30567479	0.35678	ARF3
5	30467479	30567479	0.35678	FKBP11
5	30467589	30567589	0.349884	WNT10B
5	30467589	30567589	0.349884	CCDC65
5	30467589	30567589	0.349884	RND1
5	30467589	30567589	0.349884	WNT1
5	30467589	30567589	0.349884	ARF3
5	30467589	30567589	0.349884	FKBP11
5	30467990	30567990	0.355997	WNT10B
5	30467990	30567990	0.355997	CCDC65
5	30467990	30567990	0.355997	RND1
5	30467990	30567990	0.355997	WNT1
5	30467990	30567990	0.355997	ARF3
5	30467990	30567990	0.355997	FKBP11
5	30483017	30583017	0.342049	WNT10B
5	30483017	30583017	0.342049	CCDC65
5	30483017	30583017	0.342049	RND1
5	30483017	30583017	0.342049	DDX23
5	30483017	30583017	0.342049	ARF3
5	30483017	30583017	0.342049	FKBP11
5	30483323	30583323	0.344871	WNT10B
5	30483323	30583323	0.344871	CCDC65
5	30483323	30583323	0.344871	RND1
5	30483323	30583323	0.344871	DDX23
5	30483323	30583323	0.344871	ARF3
5	30483323	30583323	0.344871	FKBP11
5	30484439	30584439	0.344579	WNT10B
5	30484439	30584439	0.344579	CCDC65
5	30484439	30584439	0.344579	RND1
5	30484439	30584439	0.344579	DDX23
5	30484439	30584439	0.344579	ARF3
5	30484439	30584439	0.344579	FKBP11
5	30491738	30591738	0.346046	CCDC65
5	30491738	30591738	0.346046	RND1
5	30491738	30591738	0.346046	DDX23

5	30491738	30591738	0.346046	ARF3
5	30491738	30591738	0.346046	FKBP11
5	30491738	30591738	0.346046	CACNB3
5	30491820	30591820	0.347709	CCDC65
5	30491820	30591820	0.347709	RND1
5	30491820	30591820	0.347709	DDX23
5	30491820	30591820	0.347709	ARF3
5	30491820	30591820	0.347709	FKBP11
5	30491820	30591820	0.347709	CACNB3
5	30506269	30606269	0.344633	CCDC65
5	30506269	30606269	0.344633	RND1
5	30506269	30606269	0.344633	DDX23
5	30506269	30606269	0.344633	ARF3
5	30506269	30606269	0.344633	FKBP11
5	30506269	30606269	0.344633	CACNB3
5	30506337	30606337	0.343969	CCDC65
5	30506337	30606337	0.343969	RND1
5	30506337	30606337	0.343969	DDX23
5	30506337	30606337	0.343969	ARF3
5	30506337	30606337	0.343969	FKBP11
5	30506337	30606337	0.343969	CACNB3
5	30506462	30606462	0.346597	CCDC65
5	30506462	30606462	0.346597	RND1
5	30506462	30606462	0.346597	DDX23
5	30506462	30606462	0.346597	ARF3
5	30506462	30606462	0.346597	FKBP11
5	30506462	30606462	0.346597	CACNB3
5	30506472	30606472	0.360887	CCDC65
5	30506472	30606472	0.360887	RND1
5	30506472	30606472	0.360887	DDX23
5	30506472	30606472	0.360887	ARF3
5	30506472	30606472	0.360887	FKBP11
5	30506472	30606472	0.360887	CACNB3
5	30506477	30606477	0.356115	CCDC65
5	30506477	30606477	0.356115	RND1
5	30506477	30606477	0.356115	DDX23
5	30506477	30606477	0.356115	ARF3
5	30506477	30606477	0.356115	FKBP11
5	30506477	30606477	0.356115	CACNB3
5	30506482	30606482	0.35002	CCDC65
5	30506482	30606482	0.35002	RND1
5	30506482	30606482	0.35002	DDX23
5	30506482	30606482	0.35002	ARF3
5	30506482	30606482	0.35002	FKBP11
5	30506482	30606482	0.35002	CACNB3
5	30506493	30606493	0.35002	CCDC65
5	30506493	30606493	0.35002	RND1
5	30506493	30606493	0.35002	DDX23
5	30506493	30606493	0.35002	ARF3
5	30506493	30606493	0.35002	FKBP11
5	30506493	30606493	0.35002	CACNB3
5	30506499	30606499	0.35002	CCDC65
5	30506499	30606499	0.35002	RND1
5	30506499	30606499	0.35002	DDX23
5	30506499	30606499	0.35002	ARF3
5	30506499	30606499	0.35002	FKBP11
5	30506499	30606499	0.35002	CACNB3
5	30506510	30606510	0.342738	CCDC65

5	30506510	30606510	0.342738	RND1
5	30506510	30606510	0.342738	DDX23
5	30506510	30606510	0.342738	ARF3
5	30506510	30606510	0.342738	FKBP11
5	30506510	30606510	0.342738	CACNB3
5	30506547	30606547	0.342696	CCDC65
5	30506547	30606547	0.342696	RND1
5	30506547	30606547	0.342696	DDX23
5	30506547	30606547	0.342696	ARF3
5	30506547	30606547	0.342696	FKBP11
5	30506547	30606547	0.342696	CACNB3
5	30534681	30634681	0.357257	RND1
5	30534681	30634681	0.357257	DDX23
5	30534681	30634681	0.357257	CACNB3
5	30534681	30634681	0.357257	ADCY6
5	30534725	30634725	0.352088	RND1
5	30534725	30634725	0.352088	DDX23
5	30534725	30634725	0.352088	CACNB3
5	30534725	30634725	0.352088	ADCY6
5	30535125	30635125	0.3505	RND1
5	30535125	30635125	0.3505	DDX23
5	30535125	30635125	0.3505	CACNB3
5	30535125	30635125	0.3505	ADCY6
5	30728376	30828376	0.345109	MIR1291
5	30728376	30828376	0.345109	LALBA
5	30728376	30828376	0.345109	CCNT1
5	30728376	30828376	0.345109	KANSL2
5	30728377	30828377	0.345109	MIR1291
5	30728377	30828377	0.345109	LALBA
5	30728377	30828377	0.345109	CCNT1
5	30728377	30828377	0.345109	KANSL2
5	30984978	31084978	0.394476	LOC102172036
5	30984978	31084978	0.394476	LOC102183765
5	30984978	31084978	0.394476	LOC102172603
5	30985121	31085121	0.377862	LOC102172036
5	30985121	31085121	0.377862	LOC102183765
5	30985121	31085121	0.377862	LOC102172603
5	30985214	31085214	0.363101	LOC102172036
5	30985214	31085214	0.363101	LOC102183765
5	30985214	31085214	0.363101	LOC102172603
5	30985816	31085816	0.408465	LOC102172036
5	30985816	31085816	0.408465	LOC102183765
5	30985816	31085816	0.408465	LOC102172603
5	30985824	31085824	0.372338	LOC102172036
5	30985824	31085824	0.372338	LOC102183765
5	30985824	31085824	0.372338	LOC102172603
5	30985827	31085827	0.438288	LOC102172036
5	30985827	31085827	0.438288	LOC102183765
5	30985827	31085827	0.438288	LOC102172603
5	30986262	31086262	0.45467	LOC102172036
5	30986262	31086262	0.45467	LOC102183765
5	30986262	31086262	0.45467	LOC102172603
5	30986290	31086290	0.45467	LOC102172036
5	30986290	31086290	0.45467	LOC102183765
5	30986290	31086290	0.45467	LOC102172603
5	30986318	31086318	0.463473	LOC102172036
5	30986318	31086318	0.463473	LOC102183765
5	30986318	31086318	0.463473	LOC102172603

5	30986505	31086505	0.418516	LOC102172036
5	30986505	31086505	0.418516	LOC102183765
5	30986505	31086505	0.418516	LOC102172603
5	30987296	31087296	0.412078	LOC102172036
5	30987296	31087296	0.412078	LOC102183765
5	30987296	31087296	0.412078	LOC102172603
5	30987429	31087429	0.42164	LOC102172036
5	30987429	31087429	0.42164	LOC102183765
5	30987429	31087429	0.42164	LOC102172603
5	30987801	31087801	0.422632	LOC102172036
5	30987801	31087801	0.422632	LOC102183765
5	30987801	31087801	0.422632	LOC102172603
5	30987972	31087972	0.416234	LOC102172036
5	30987972	31087972	0.416234	LOC102183765
5	30987972	31087972	0.416234	LOC102172603
5	30988170	31088170	0.424955	LOC102172036
5	30988170	31088170	0.424955	LOC102183765
5	30988170	31088170	0.424955	LOC102172603
5	30988401	31088401	0.43344	LOC102172036
5	30988401	31088401	0.43344	LOC102183765
5	30988401	31088401	0.43344	LOC102172603
5	30988530	31088530	0.447532	LOC102172036
5	30988530	31088530	0.447532	LOC102183765
5	30988530	31088530	0.447532	LOC102172603
5	30988646	31088646	0.436633	LOC102172036
5	30988646	31088646	0.436633	LOC102183765
5	30988646	31088646	0.436633	LOC102172603
5	30988786	31088786	0.437723	LOC102172036
5	30988786	31088786	0.437723	LOC102183765
5	30988786	31088786	0.437723	LOC102172603
5	30988994	31088994	0.441154	LOC102172036
5	30988994	31088994	0.441154	LOC102183765
5	30988994	31088994	0.441154	LOC102172603
5	30989010	31089010	0.452774	LOC102172036
5	30989010	31089010	0.452774	LOC102183765
5	30989010	31089010	0.452774	LOC102172603
5	30989162	31089162	0.448874	LOC102172036
5	30989162	31089162	0.448874	LOC102183765
5	30989162	31089162	0.448874	LOC102172603
5	30989175	31089175	0.426412	LOC102172036
5	30989175	31089175	0.426412	LOC102183765
5	30989175	31089175	0.426412	LOC102172603
5	30989637	31089637	0.426953	LOC102172036
5	30989637	31089637	0.426953	LOC102183765
5	30989637	31089637	0.426953	LOC102172603
5	30989806	31089806	0.42544	LOC102172036
5	30989806	31089806	0.42544	LOC102183765
5	30989806	31089806	0.42544	LOC102172603
5	30990089	31090089	0.381487	LOC102172036
5	30990089	31090089	0.381487	LOC102183765
5	30990089	31090089	0.381487	LOC102172603
5	30990159	31090159	0.411916	LOC102172036
5	30990159	31090159	0.411916	LOC102183765
5	30990159	31090159	0.411916	LOC102172603
5	30990179	31090179	0.412148	LOC102172036
5	30990179	31090179	0.412148	LOC102183765
5	30990179	31090179	0.412148	LOC102172603
5	30990276	31090276	0.381471	LOC102172036

5	30990276	31090276	0.381471	LOC102183765
5	30990276	31090276	0.381471	LOC102172603
5	30990550	31090550	0.350818	LOC102172036
5	30990550	31090550	0.350818	LOC102183765
5	30990550	31090550	0.350818	LOC102172603
5	30990615	31090615	0.440645	LOC102172036
5	30990615	31090615	0.440645	LOC102183765
5	30990615	31090615	0.440645	LOC102172603
5	30990669	31090669	0.421388	LOC102172036
5	30990669	31090669	0.421388	LOC102183765
5	30990669	31090669	0.421388	LOC102172603
5	30991238	31091238	0.429499	LOC102172036
5	30991238	31091238	0.429499	LOC102183765
5	30991238	31091238	0.429499	LOC102172603
5	30991350	31091350	0.472285	LOC102172036
5	30991350	31091350	0.472285	LOC102183765
5	30991350	31091350	0.472285	LOC102172603
5	30991577	31091577	0.475245	LOC102172036
5	30991577	31091577	0.475245	LOC102183765
5	30991577	31091577	0.475245	LOC102172603
5	30991735	31091735	0.442375	LOC102172036
5	30991735	31091735	0.442375	LOC102183765
5	30991735	31091735	0.442375	LOC102172603
5	30992256	31092256	0.452147	LOC102172036
5	30992256	31092256	0.452147	LOC102183765
5	30992256	31092256	0.452147	LOC102172603
5	30992289	31092289	0.402442	LOC102172036
5	30992289	31092289	0.402442	LOC102183765
5	30992289	31092289	0.402442	LOC102172603
5	30992354	31092354	0.407628	LOC102172036
5	30992354	31092354	0.407628	LOC102183765
5	30992354	31092354	0.407628	LOC102172603
5	30992492	31092492	0.402115	LOC102172036
5	30992492	31092492	0.402115	LOC102183765
5	30992492	31092492	0.402115	LOC102172603
5	30992513	31092513	0.412498	LOC102172036
5	30992513	31092513	0.412498	LOC102183765
5	30992513	31092513	0.412498	LOC102172603
5	30993008	31093008	0.406386	LOC102172036
5	30993008	31093008	0.406386	LOC102183765
5	30993008	31093008	0.406386	LOC102172603
5	30993171	31093171	0.415977	LOC102172036
5	30993171	31093171	0.415977	LOC102183765
5	30993171	31093171	0.415977	LOC102172603
5	30993305	31093305	0.400067	LOC102172036
5	30993305	31093305	0.400067	LOC102183765
5	30993305	31093305	0.400067	LOC102172603
5	30993738	31093738	0.387735	LOC102172036
5	30993738	31093738	0.387735	LOC102183765
5	30993738	31093738	0.387735	LOC102172603
5	30993953	31093953	0.376912	LOC102172036
5	30993953	31093953	0.376912	LOC102183765
5	30993953	31093953	0.376912	LOC102172603
5	30994072	31094072	0.35836	LOC102172036
5	30994072	31094072	0.35836	LOC102183765
5	30994072	31094072	0.35836	LOC102172603
5	31012070	31112070	0.344532	LOC102183765
5	31012070	31112070	0.344532	LOC102172603

5	31012070	31112070	0.344532	LOC102173162
5	31019609	31119609	0.342968	LOC102183765
5	31019609	31119609	0.342968	LOC102172603
5	31019609	31119609	0.342968	LOC102173162
5	31019837	31119837	0.347137	LOC102183765
5	31019837	31119837	0.347137	LOC102172603
5	31019837	31119837	0.347137	LOC102173162
5	31019954	31119954	0.347203	LOC102183765
5	31019954	31119954	0.347203	LOC102172603
5	31019954	31119954	0.347203	LOC102173162
5	31020088	31120088	0.347203	LOC102183765
5	31020088	31120088	0.347203	LOC102172603
5	31020088	31120088	0.347203	LOC102173162
5	31020147	31120147	0.373787	LOC102183765
5	31020147	31120147	0.373787	LOC102172603
5	31020147	31120147	0.373787	LOC102173162
5	31020149	31120149	0.373787	LOC102183765
5	31020149	31120149	0.373787	LOC102172603
5	31020149	31120149	0.373787	LOC102173162
5	31020178	31120178	0.37213	LOC102183765
5	31020178	31120178	0.37213	LOC102172603
5	31020178	31120178	0.37213	LOC102173162
5	31020335	31120335	0.360728	LOC102183765
5	31020335	31120335	0.360728	LOC102172603
5	31020335	31120335	0.360728	LOC102173162
5	31020388	31120388	0.371287	LOC102183765
5	31020388	31120388	0.371287	LOC102172603
5	31020388	31120388	0.371287	LOC102173162
5	31020389	31120389	0.360062	LOC102183765
5	31020389	31120389	0.360062	LOC102172603
5	31020389	31120389	0.360062	LOC102173162
5	31020896	31120896	0.372718	LOC102183765
5	31020896	31120896	0.372718	LOC102172603
5	31020896	31120896	0.372718	LOC102173162
5	31020906	31120906	0.372388	LOC102183765
5	31020906	31120906	0.372388	LOC102172603
5	31020906	31120906	0.372388	LOC102173162
5	31020907	31120907	0.387097	LOC102183765
5	31020907	31120907	0.387097	LOC102172603
5	31020907	31120907	0.387097	LOC102173162
5	31020941	31120941	0.352056	LOC102183765
5	31020941	31120941	0.352056	LOC102172603
5	31020941	31120941	0.352056	LOC102173162
5	31021010	31121010	0.35168	LOC102183765
5	31021010	31121010	0.35168	LOC102172603
5	31021010	31121010	0.35168	LOC102173162
5	31133994	31233994	0.357175	LOC102173446
5	31133994	31233994	0.357175	LOC102174001
5	31133994	31233994	0.357175	C5H12orf54
5	31269615	31369615	0.351892	LOC108636097
5	31269656	31369656	0.370389	LOC108636097
5	31269681	31369681	0.361495	LOC108636097
5	31269796	31369796	0.363804	LOC108636097
5	31269822	31369822	0.363804	LOC108636097
5	31269846	31369846	0.363804	LOC108636097
5	31269887	31369887	0.363804	LOC108636097
5	31269893	31369893	0.363804	LOC108636097
5	31269946	31369946	0.363804	LOC108636097

5	31269959	31369959	0.363804	LOC108636097
5	31269970	31369970	0.363804	LOC108636097
5	31270008	31370008	0.365065	LOC108636097
5	31270039	31370039	0.382638	LOC108636097
5	31270192	31370192	0.384399	LOC108636097
5	31270217	31370217	0.394099	LOC108636097
5	31270244	31370244	0.414841	LOC108636097
5	31270353	31370353	0.392838	LOC108636097
5	31270372	31370372	0.392838	LOC108636097
5	31270445	31370445	0.397164	LOC108636097
5	31270482	31370482	0.397164	LOC108636097
5	31270493	31370493	0.397164	LOC108636097
5	31270523	31370523	0.397164	LOC108636097
5	31270547	31370547	0.397164	LOC108636097
5	31270610	31370610	0.397315	LOC108636097
5	31270689	31370689	0.397315	LOC108636097
5	31270703	31370703	0.397315	LOC108636097
5	31270704	31370704	0.435259	LOC108636097
5	31270742	31370742	0.438247	LOC108636097
5	31270789	31370789	0.438247	LOC108636097
5	31270938	31370938	0.438247	LOC108636097
5	31270942	31370942	0.438247	LOC108636097
5	31270964	31370964	0.438247	LOC108636097
5	31270973	31370973	0.438247	LOC108636097
5	31271012	31371012	0.438247	LOC108636097
5	31271025	31371025	0.438189	LOC108636097
5	31271070	31371070	0.455824	LOC108636097
5	31271091	31371091	0.455824	LOC108636097
5	31271132	31371132	0.455824	LOC108636097
5	31271143	31371143	0.455824	LOC108636097
5	31271163	31371163	0.455824	LOC108636097
5	31271188	31371188	0.47956	LOC108636097
5	31271201	31371201	0.462074	LOC108636097
5	31271335	31371335	0.449319	LOC108636097
5	31271719	31371719	0.447143	LOC108636097
5	31271749	31371749	0.446285	LOC108636097
5	31271772	31371772	0.446289	LOC108636097
5	31271805	31371805	0.45644	LOC108636097
5	31271877	31371877	0.447514	LOC108636097
5	31271883	31371883	0.447514	LOC108636097
5	31271989	31371989	0.441007	LOC108636097
5	31272058	31372058	0.429521	LOC108636097
5	31272067	31372067	0.429521	LOC108636097
5	31272071	31372071	0.429521	LOC108636097
5	31272141	31372141	0.429521	LOC108636097
5	31272145	31372145	0.429521	LOC108636097
5	31272217	31372217	0.429521	LOC108636097
5	31272218	31372218	0.429521	LOC108636097
5	31272226	31372226	0.429521	LOC108636097
5	31272267	31372267	0.429521	LOC108636097
5	31272364	31372364	0.429521	LOC108636097
5	31272375	31372375	0.446275	LOC108636097
5	31272509	31372509	0.446317	LOC108636097
5	31272522	31372522	0.447941	LOC108636097
5	31272573	31372573	0.447278	LOC108636097
5	31272592	31372592	0.447278	LOC108636097
5	31272596	31372596	0.452701	LOC108636097
5	31272598	31372598	0.439843	LOC108636097

5	31272611	31372611	0.439843	LOC108636097
5	31272679	31372679	0.439843	LOC108636097
5	31272705	31372705	0.439843	LOC108636097
5	31272712	31372712	0.439843	LOC108636097
5	31272780	31372780	0.439843	LOC108636097
5	31272789	31372789	0.439843	LOC108636097
5	31272804	31372804	0.439843	LOC108636097
5	31272811	31372811	0.439843	LOC108636097
5	31272820	31372820	0.439843	LOC108636097
5	31272844	31372844	0.445261	LOC108636097
5	31272943	31372943	0.438581	LOC108636097
5	31272946	31372946	0.425265	LOC108636097
5	31273064	31373064	0.425265	LOC108636097
5	31273106	31373106	0.425265	LOC108636097
5	31273107	31373107	0.425265	LOC108636097
5	31273113	31373113	0.422114	LOC108636097
5	31273125	31373125	0.426405	LOC108636097
5	31273217	31373217	0.392497	LOC108636097
5	31273242	31373242	0.392497	LOC108636097
5	31273303	31373303	0.392497	LOC108636097
5	31273387	31373387	0.395052	LOC108636097
5	31273390	31373390	0.395052	LOC108636097
5	31273544	31373544	0.391067	LOC108636097
5	31273564	31373564	0.413863	LOC108636097
5	31273568	31373568	0.414977	LOC108636097
5	31273570	31373570	0.414977	LOC108636097
5	31273628	31373628	0.423095	LOC108636097
5	31273635	31373635	0.418468	LOC108636097
5	31273644	31373644	0.370352	LOC108636097
5	31273927	31373927	0.369166	LOC108636097
5	31273952	31373952	0.369166	LOC108636097
5	31273953	31373953	0.369166	LOC108636097
5	31273959	31373959	0.369166	LOC108636097
5	31273960	31373960	0.369166	LOC108636097
5	31273973	31373973	0.369166	LOC108636097
5	31273974	31373974	0.369166	LOC108636097
5	31274135	31374135	0.387452	LOC108636097
5	31274210	31374210	0.387675	LOC108636097
5	31274213	31374213	0.387675	LOC108636097
5	31274231	31374231	0.39126	LOC108636097
5	31274263	31374263	0.405308	LOC108636097
5	31274276	31374276	0.355716	LOC108636097
5	31274392	31374392	0.367919	LOC108636097
5	31274395	31374395	0.367016	LOC108636097
5	31274503	31374503	0.369728	LOC108636097
5	31274610	31374610	0.368761	LOC108636097
5	31274669	31374669	0.370284	LOC108636097
5	31274896	31374896	0.371402	LOC108636097
5	31274925	31374925	0.34523	LOC108636097
5	31274928	31374928	0.364635	LOC108636097
5	31275498	31375498	0.342845	LOC108636097
5	31275516	31375516	0.342845	LOC108636097
5	31276179	31376179	0.35269	LOC108636097
5	31276218	31376218	0.349023	LOC108636097
5	31276703	31376703	0.342697	LOC108636097
5	31276731	31376731	0.371642	LOC108636097
5	31276825	31376825	0.381891	LOC108636097
5	31277007	31377007	0.410372	LOC108636097

5	31277025	31377025	0.410372	LOC108636097
5	31277084	31377084	0.423769	LOC108636097
5	31277246	31377246	0.423769	LOC108636097
5	31277270	31377270	0.425841	LOC108636097
5	31277641	31377641	0.428235	LOC108636097
5	31277653	31377653	0.411348	LOC108636097
5	31277804	31377804	0.419644	LOC108636097
5	31277990	31377990	0.423585	LOC108636097
5	31277996	31377996	0.429416	LOC108636097
5	31278010	31378010	0.413137	LOC108636097
5	31278133	31378133	0.4127	LOC108636097
5	31278163	31378163	0.413687	LOC108636097
5	31278203	31378203	0.413231	LOC108636097
5	31278306	31378306	0.410427	LOC108636097
5	31278343	31378343	0.410427	LOC108636097
5	31278351	31378351	0.410427	LOC108636097
5	31278353	31378353	0.410427	LOC108636097
5	31278671	31378671	0.411794	LOC108636097
5	31278729	31378729	0.452049	LOC108636097
5	31278838	31378838	0.4575	LOC108636097
5	31278884	31378884	0.41415	LOC108636097
5	31278919	31378919	0.399947	LOC108636097
5	31278934	31378934	0.399892	LOC108636097
5	31279032	31379032	0.400536	LOC108636097
5	31279044	31379044	0.40008	LOC108636097
5	31279105	31379105	0.410834	LOC108636097
5	31279170	31379170	0.418654	LOC108636097
5	31279191	31379191	0.406805	LOC108636097
5	31279376	31379376	0.407414	LOC108636097
5	31279610	31379610	0.401585	LOC108636097
5	31279900	31379900	0.403871	LOC108636097
5	31280435	31380435	0.401111	LOC108636097
5	31280470	31380470	0.403725	LOC108636097
5	31280537	31380537	0.418411	LOC108636097
5	31280547	31380547	0.41913	LOC108636097
5	31280573	31380573	0.422615	LOC108636097
5	31281013	31381013	0.462957	LOC108636097
5	31281042	31381042	0.459416	LOC108636097
5	31281080	31381080	0.460899	LOC108636097
5	31281105	31381105	0.464125	LOC108636097
5	31281320	31381320	0.438522	LOC108636097
5	31281741	31381741	0.438915	LOC108636097
5	31281915	31381915	0.456943	LOC108636097
5	31281968	31381968	0.481786	LOC108636097
5	31282034	31382034	0.459009	LOC108636097
5	31282103	31382103	0.456589	LOC108636097
5	31282256	31382256	0.460042	LOC108636097
5	31282269	31382269	0.461245	LOC108636097
5	31282279	31382279	0.46424	LOC108636097
5	31282357	31382357	0.452226	LOC108636097
5	31282376	31382376	0.452226	LOC108636097
5	31282436	31382436	0.455825	LOC108636097
5	31282466	31382466	0.455825	LOC108636097
5	31282499	31382499	0.455825	LOC108636097
5	31282586	31382586	0.45667	LOC108636097
5	31282587	31382587	0.45667	LOC108636097
5	31282631	31382631	0.449154	LOC108636097
5	31282678	31382678	0.444289	LOC108636097

5	31282682	31382682	0.45673	LOC108636097
5	31282717	31382717	0.457834	LOC108636097
5	31282720	31382720	0.457834	LOC108636097
5	31282723	31382723	0.447143	LOC108636097
5	31282742	31382742	0.468081	LOC108636097
5	31282785	31382785	0.453865	LOC108636097
5	31282803	31382803	0.454397	LOC108636097
5	31282805	31382805	0.436379	LOC108636097
5	31282821	31382821	0.436379	LOC108636097
5	31282999	31382999	0.436379	LOC108636097
5	31283097	31383097	0.436379	LOC108636097
5	31283104	31383104	0.436379	LOC108636097
5	31283239	31383239	0.436379	LOC108636097
5	31283283	31383283	0.436379	LOC108636097
5	31283297	31383297	0.436379	LOC108636097
5	31283316	31383316	0.439336	LOC108636097
5	31283319	31383319	0.508406	LOC108636097
5	31283441	31383441	0.508406	LOC108636097
5	31283455	31383455	0.509245	LOC108636097
5	31283536	31383536	0.501486	LOC108636097
5	31283560	31383560	0.501486	LOC108636097
5	31283602	31383602	0.501486	LOC108636097
5	31283661	31383661	0.501486	LOC108636097
5	31283672	31383672	0.505506	LOC108636097
5	31283686	31383686	0.494848	LOC108636097
5	31284801	31384801	0.411324	LOC108636097
5	31284823	31384823	0.3691	LOC108636097
5	31357510	31457510	0.344317	LOC108636097
5	31357535	31457535	0.342265	LOC108636097
5	31357624	31457624	0.343909	LOC108636097
5	31357643	31457643	0.343096	LOC108636097
5	31357835	31457835	0.349517	LOC108636097
5	31357905	31457905	0.349517	LOC108636097
5	31359647	31459647	0.35131	LOC108636097
5	31359669	31459669	0.344862	LOC108636097
5	31359690	31459690	0.349875	LOC108636097
5	31359694	31459694	0.342039	LOC108636097
5	33219983	33319983	0.360084	LOC102190664
5	33219989	33319989	0.366368	LOC102190664
5	33220039	33320039	0.350533	LOC102190664
5	33220040	33320040	0.351484	LOC102190664
5	33220044	33320044	0.36226	LOC102190664
5	33220115	33320115	0.34386	LOC102190664
5	33220180	33320180	0.346946	LOC102190664
5	33220246	33320246	0.345415	LOC102190664
5	33220250	33320250	0.342857	LOC102190664
5	33220373	33320373	0.342857	LOC102190664
5	33220522	33320522	0.346058	LOC102190664
5	33220540	33320540	0.358484	LOC102190664
5	33220546	33320546	0.352916	LOC102190664
5	33220606	33320606	0.352916	LOC102190664
5	33220632	33320632	0.352916	LOC102190664
5	33220663	33320663	0.355121	LOC102190664
5	33220680	33320680	0.354972	LOC102190664
5	33220729	33320729	0.343529	LOC102190664
5	33220742	33320742	0.358569	LOC102190664
5	33220780	33320780	0.357947	LOC102190664
5	33220978	33320978	0.357336	LOC102190664

5	33220980	33320980	0.353985	LOC102190664
5	33220992	33320992	0.367511	LOC102190664
5	33221027	33321027	0.360542	LOC102190664
5	33221133	33321133	0.360542	LOC102190664
5	33221156	33321156	0.360942	LOC102190664
5	33221159	33321159	0.36054	LOC102190664
5	33221267	33321267	0.342552	LOC102190664
5	33221330	33321330	0.359233	LOC102190664
5	33320086	33420086	0.35124	LOC106502098
5	33320086	33420086	0.35124	SLC38A2
5	33320086	33420086	0.35124	LOC102190664
5	33320087	33420087	0.35124	LOC106502098
5	33320087	33420087	0.35124	SLC38A2
5	33320087	33420087	0.35124	LOC102190664
5	33321115	33421115	0.343171	LOC106502098
5	33321115	33421115	0.343171	SLC38A2
5	33321115	33421115	0.343171	LOC102190664
5	33321149	33421149	0.343171	LOC106502098
5	33321149	33421149	0.343171	SLC38A2
5	33321149	33421149	0.343171	LOC102190664
5	33321228	33421228	0.354922	LOC106502098
5	33321228	33421228	0.354922	SLC38A2
5	33321228	33421228	0.354922	LOC102190664
5	33321277	33421277	0.355054	LOC106502098
5	33321277	33421277	0.355054	SLC38A2
5	33321277	33421277	0.355054	LOC102190664
5	33321358	33421358	0.379677	LOC106502098
5	33321358	33421358	0.379677	SLC38A2
5	33321358	33421358	0.379677	LOC102190664
5	33321853	33421853	0.354087	LOC106502098
5	33321853	33421853	0.354087	SLC38A2
5	33321853	33421853	0.354087	LOC102190664
5	39103072	39203072	0.365646	PDZRN4
5	39103148	39203148	0.361138	PDZRN4
5	39103712	39203712	0.350346	PDZRN4
5	39103806	39203806	0.347084	PDZRN4
5	39103878	39203878	0.368469	PDZRN4
5	39104041	39204041	0.361795	PDZRN4
5	39104243	39204243	0.361795	PDZRN4
5	39105343	39205343	0.372058	PDZRN4
5	39105378	39205378	0.372058	PDZRN4
5	39105588	39205588	0.373944	PDZRN4
5	39105663	39205663	0.385128	PDZRN4
5	39105765	39205765	0.382111	PDZRN4
5	39105855	39205855	0.377639	PDZRN4
5	39105905	39205905	0.386983	PDZRN4
5	39106061	39206061	0.384087	PDZRN4
5	39106358	39206358	0.363078	PDZRN4
5	39106435	39206435	0.363666	PDZRN4
5	39106622	39206622	0.363938	PDZRN4
5	39106654	39206654	0.36888	PDZRN4
5	39106693	39206693	0.384596	PDZRN4
5	39106722	39206722	0.363367	PDZRN4
5	39106723	39206723	0.36412	PDZRN4
5	39106816	39206816	0.36489	PDZRN4
5	39106835	39206835	0.374573	PDZRN4
5	39106976	39206976	0.349296	PDZRN4
5	39107518	39207518	0.341717	PDZRN4

5	39107897	39207897	0.354247	PDZRN4
5	39108352	39208352	0.34993	PDZRN4
5	39108482	39208482	0.353156	PDZRN4
5	39108614	39208614	0.35801	PDZRN4
5	39108714	39208714	0.381791	PDZRN4
5	39108787	39208787	0.369376	PDZRN4
5	39108807	39208807	0.361147	PDZRN4
5	39109072	39209072	0.346959	PDZRN4
5	39109075	39209075	0.350189	PDZRN4
5	41334153	41434153	0.356221	KIF21A
5	41349723	41449723	0.380622	KIF21A
5	41349799	41449799	0.380084	KIF21A
5	41349910	41449910	0.405489	KIF21A
5	41349982	41449982	0.404478	KIF21A
5	41350097	41450097	0.415177	KIF21A
5	41350214	41450214	0.411018	KIF21A
5	41350301	41450301	0.417907	KIF21A
5	41350346	41450346	0.412919	KIF21A
5	41350359	41450359	0.413474	KIF21A
5	41350433	41450433	0.415583	KIF21A
5	41350501	41450501	0.414797	KIF21A
5	41350591	41450591	0.399001	KIF21A
5	41350670	41450670	0.399001	KIF21A
5	41350797	41450797	0.40389	KIF21A
5	41351295	41451295	0.408785	KIF21A
5	41351296	41451296	0.414834	KIF21A
5	41351315	41451315	0.418696	KIF21A
5	41351458	41451458	0.412762	KIF21A
5	41351828	41451828	0.416764	KIF21A
5	41351845	41451845	0.413583	KIF21A
5	41351989	41451989	0.416797	KIF21A
5	41352004	41452004	0.418505	KIF21A
5	41352039	41452039	0.414993	KIF21A
5	41352089	41452089	0.406491	KIF21A
5	41352270	41452270	0.406491	KIF21A
5	41352291	41452291	0.407164	KIF21A
5	41352374	41452374	0.421192	KIF21A
5	41352588	41452588	0.414009	KIF21A
5	41352615	41452615	0.417701	KIF21A
5	41353361	41453361	0.41256	KIF21A
5	41353417	41453417	0.416861	KIF21A
5	41353512	41453512	0.419527	KIF21A
5	41353513	41453513	0.418592	KIF21A
5	41353722	41453722	0.41712	KIF21A
5	41353800	41453800	0.42018	KIF21A
5	41353850	41453850	0.414175	KIF21A
5	41353907	41453907	0.404866	KIF21A
5	41353937	41453937	0.404866	KIF21A
5	41354003	41454003	0.408568	KIF21A
5	41354044	41454044	0.418632	KIF21A
5	41354045	41454045	0.419719	KIF21A
5	41354100	41454100	0.412368	KIF21A
5	41354166	41454166	0.412368	KIF21A
5	41354175	41454175	0.412368	KIF21A
5	41354205	41454205	0.412732	KIF21A
5	41354378	41454378	0.406537	KIF21A
5	41354403	41454403	0.404147	KIF21A
5	41354419	41454419	0.415702	KIF21A

5	41354717	41454717	0.415345	KIF21A
5	41354753	41454753	0.413258	KIF21A
5	41354838	41454838	0.427606	KIF21A
5	41354960	41454960	0.428879	KIF21A
5	41355079	41455079	0.43984	KIF21A
5	41355104	41455104	0.43317	KIF21A
5	41355255	41455255	0.43384	KIF21A
5	41355272	41455272	0.430878	KIF21A
5	41355307	41455307	0.436444	KIF21A
5	41355358	41455358	0.436919	KIF21A
5	41355422	41455422	0.440726	KIF21A
5	41355475	41455475	0.443971	KIF21A
5	41355522	41455522	0.438405	KIF21A
5	41355534	41455534	0.436041	KIF21A
5	41355548	41455548	0.435484	KIF21A
5	41355597	41455597	0.434123	KIF21A
5	41355620	41455620	0.434676	KIF21A
5	41355650	41455650	0.437328	KIF21A
5	41355669	41455669	0.425952	KIF21A
5	41355761	41455761	0.426556	KIF21A
5	41355774	41455774	0.426358	KIF21A
5	41355794	41455794	0.426358	KIF21A
5	41355873	41455873	0.427064	KIF21A
5	41355951	41455951	0.427495	KIF21A
5	41355969	41455969	0.421882	KIF21A
5	41356081	41456081	0.419836	KIF21A
5	41356194	41456194	0.412114	KIF21A
5	41356374	41456374	0.418516	KIF21A
5	41356544	41456544	0.418976	KIF21A
5	41356610	41456610	0.418925	KIF21A
5	41356613	41456613	0.419996	KIF21A
5	41356696	41456696	0.419996	KIF21A
5	41356875	41456875	0.421438	KIF21A
5	41356997	41456997	0.416345	KIF21A
5	41357041	41457041	0.416345	KIF21A
5	41357046	41457046	0.416345	KIF21A
5	41357135	41457135	0.417767	KIF21A
5	41357259	41457259	0.42288	KIF21A
5	41357267	41457267	0.418642	KIF21A
5	41357402	41457402	0.422268	KIF21A
5	41357513	41457513	0.422049	KIF21A
5	41357518	41457518	0.418042	KIF21A
5	41357607	41457607	0.418042	KIF21A
5	41357674	41457674	0.417691	KIF21A
5	41357726	41457726	0.41573	KIF21A
5	41357756	41457756	0.41573	KIF21A
5	41357765	41457765	0.416671	KIF21A
5	41357831	41457831	0.41647	KIF21A
5	41357869	41457869	0.414365	KIF21A
5	41357904	41457904	0.414173	KIF21A
5	41357941	41457941	0.417327	KIF21A
5	41357958	41457958	0.414473	KIF21A
5	41358000	41458000	0.420904	KIF21A
5	41358154	41458154	0.43606	KIF21A
5	41358157	41458157	0.43606	KIF21A
5	41358163	41458163	0.43606	KIF21A
5	41358164	41458164	0.43606	KIF21A
5	41358169	41458169	0.438326	KIF21A

5	41358177	41458177	0.434006	KIF21A
5	41358178	41458178	0.434006	KIF21A
5	41358203	41458203	0.419775	KIF21A
5	41358308	41458308	0.414508	KIF21A
5	41358314	41458314	0.412516	KIF21A
5	41358377	41458377	0.411618	KIF21A
5	41358386	41458386	0.411618	KIF21A
5	41358466	41458466	0.413119	KIF21A
5	41358473	41458473	0.411559	KIF21A
5	41358489	41458489	0.410581	KIF21A
5	41358580	41458580	0.407045	KIF21A
5	41358637	41458637	0.389569	KIF21A
5	41358710	41458710	0.397542	KIF21A
5	41358725	41458725	0.392716	KIF21A
5	41358732	41458732	0.386575	KIF21A
5	41359050	41459050	0.380747	KIF21A
5	41359147	41459147	0.393981	KIF21A
5	41359175	41459175	0.393981	KIF21A
5	41359176	41459176	0.393981	KIF21A
5	41359183	41459183	0.400327	KIF21A
5	41359279	41459279	0.389642	KIF21A
5	41359566	41459566	0.389642	KIF21A
5	41359818	41459818	0.392419	KIF21A
5	41359855	41459855	0.384858	KIF21A
5	41359946	41459946	0.384858	KIF21A
5	41359961	41459961	0.377612	KIF21A
5	41360449	41460449	0.384126	KIF21A
5	41360954	41460954	0.383734	KIF21A
5	41361176	41461176	0.370122	KIF21A
5	41361261	41461261	0.383604	KIF21A
5	41361839	41461839	0.383114	KIF21A
5	41361851	41461851	0.394813	KIF21A
5	41361902	41461902	0.396637	KIF21A
5	41362443	41462443	0.386455	KIF21A
5	41362863	41462863	0.380359	KIF21A
5	41362924	41462924	0.369461	KIF21A
5	41363027	41463027	0.372884	KIF21A
5	41363464	41463464	0.384254	KIF21A
5	41363534	41463534	0.389391	KIF21A
5	41363716	41463716	0.389391	KIF21A
5	41363752	41463752	0.388716	KIF21A
5	41364516	41464516	0.413924	KIF21A
5	41364540	41464540	0.363922	KIF21A
5	41364647	41464647	0.37161	KIF21A
5	41364675	41464675	0.364347	KIF21A
5	41365328	41465328	0.346036	KIF21A
5	41365621	41465621	0.355513	KIF21A
5	41365687	41465687	0.345534	KIF21A
5	41366163	41466163	0.369448	KIF21A
5	41366369	41466369	0.373465	KIF21A
5	41366464	41466464	0.384383	KIF21A
5	41366626	41466626	0.383804	KIF21A
5	41366717	41466717	0.387296	KIF21A
5	41366820	41466820	0.37939	KIF21A
5	41366902	41466902	0.353225	KIF21A
5	41389436	41489436	0.397198	KIF21A
5	41598843	41698843	0.351496	CPNE8
5	43583715	43683715	0.390777	LOC102171764

5	43583715	43683715	0.390777	LOC108633237
5	43583715	43683715	0.390777	YEATS4
5	43583715	43683715	0.390777	LOC102171304
5	43583715	43683715	0.390777	LOC102185900
5	43583721	43683721	0.516716	LOC102171764
5	43583721	43683721	0.516716	LOC108633237
5	43583721	43683721	0.516716	YEATS4
5	43583721	43683721	0.516716	LOC102171304
5	43583721	43683721	0.516716	LOC102185900
5	43583727	43683727	0.457797	LOC102171764
5	43583727	43683727	0.457797	LOC108633237
5	43583727	43683727	0.457797	YEATS4
5	43583727	43683727	0.457797	LOC102171304
5	43583727	43683727	0.457797	LOC102185900
5	43583736	43683736	0.492424	LOC102171764
5	43583736	43683736	0.492424	LOC108633237
5	43583736	43683736	0.492424	YEATS4
5	43583736	43683736	0.492424	LOC102171304
5	43583736	43683736	0.492424	LOC102185900
5	43583741	43683741	0.51675	LOC102171764
5	43583741	43683741	0.51675	LOC108633237
5	43583741	43683741	0.51675	YEATS4
5	43583741	43683741	0.51675	LOC102171304
5	43583741	43683741	0.51675	LOC102185900
5	43583742	43683742	0.474854	LOC102171764
5	43583742	43683742	0.474854	LOC108633237
5	43583742	43683742	0.474854	YEATS4
5	43583742	43683742	0.474854	LOC102171304
5	43583742	43683742	0.474854	LOC102185900
5	43583748	43683748	0.405607	LOC102171764
5	43583748	43683748	0.405607	LOC108633237
5	43583748	43683748	0.405607	YEATS4
5	43583748	43683748	0.405607	LOC102171304
5	43583748	43683748	0.405607	LOC102185900
5	43583755	43683755	0.365131	LOC102171764
5	43583755	43683755	0.365131	LOC108633237
5	43583755	43683755	0.365131	YEATS4
5	43583755	43683755	0.365131	LOC102171304
5	43583755	43683755	0.365131	LOC102185900
5	44204724	44304724	0.361173	CPM
5	44204931	44304931	0.376141	CPM
5	44205233	44305233	0.35446	CPM
5	44209752	44309752	0.354992	CPM
5	44210059	44310059	0.351074	CPM
5	44210779	44310779	0.357845	CPM
5	44210927	44310927	0.364991	CPM
5	44210934	44310934	0.366545	CPM
5	44211000	44311000	0.366545	CPM
5	44211008	44311008	0.36892	CPM
5	44211012	44311012	0.365963	CPM
5	44211023	44311023	0.377628	CPM
5	44211064	44311064	0.375059	CPM
5	44211073	44311073	0.375059	CPM
5	44211075	44311075	0.375059	CPM
5	44211113	44311113	0.390798	CPM
5	44211137	44311137	0.390798	CPM
5	44211258	44311258	0.403535	CPM
5	44211293	44311293	0.386823	CPM

5	44211437	44311437	0.397051	CPM
5	44211509	44311509	0.387644	CPM
5	44211519	44311519	0.387644	CPM
5	44211542	44311542	0.429049	CPM
5	44211591	44311591	0.427683	CPM
5	44211837	44311837	0.425622	CPM
5	44211866	44311866	0.429112	CPM
5	44212107	44312107	0.420107	CPM
5	44212125	44312125	0.407508	CPM
5	44212224	44312224	0.43858	CPM
5	44212239	44312239	0.442416	CPM
5	44212279	44312279	0.436363	CPM
5	44212370	44312370	0.431244	CPM
5	44212519	44312519	0.44119	CPM
5	44212520	44312520	0.425849	CPM
5	44212522	44312522	0.417785	CPM
5	44212739	44312739	0.413364	CPM
5	44212907	44312907	0.410081	CPM
5	44213167	44313167	0.352237	CPM
5	44213352	44313352	0.384618	CPM
5	44213368	44313368	0.391993	CPM
5	44213496	44313496	0.380655	CPM
5	44213535	44313535	0.375411	CPM
5	44213809	44313809	0.380958	CPM
5	44214158	44314158	0.380958	CPM
5	44214194	44314194	0.355234	CPM
5	44214307	44314307	0.341835	CPM
5	44217473	44317473	0.36328	CPM
5	44217505	44317505	0.407868	CPM
5	44217613	44317613	0.37804	CPM
5	44217640	44317640	0.419106	CPM
5	44217903	44317903	0.422635	CPM
5	44217944	44317944	0.396441	CPM
5	44217964	44317964	0.396923	CPM
5	44217988	44317988	0.396923	CPM
5	44218235	44318235	0.389915	CPM
5	44218273	44318273	0.39931	CPM
5	44218357	44318357	0.401658	CPM
5	44218420	44318420	0.410372	CPM
5	44218427	44318427	0.420495	CPM
5	44218430	44318430	0.417152	CPM
5	44218434	44318434	0.420838	CPM
5	44218436	44318436	0.425606	CPM
5	44218550	44318550	0.38104	CPM
5	44218582	44318582	0.37931	CPM
5	44218774	44318774	0.360407	CPM
5	44218800	44318800	0.360643	CPM
5	44218848	44318848	0.357442	CPM
5	44219453	44319453	0.343491	CPM
5	44220783	44320783	0.372476	CPM
5	44220886	44320886	0.352147	CPM
5	44220995	44320995	0.352147	CPM
5	45875260	45975260	0.356547	TRNAK-UUU-8
5	45875385	45975385	0.359396	TRNAK-UUU-8
5	45875514	45975514	0.363007	TRNAK-UUU-8
5	45875538	45975538	0.360254	TRNAK-UUU-8
5	45875565	45975565	0.360648	TRNAK-UUU-8
5	45875611	45975611	0.360249	TRNAK-UUU-8

5	45875708	45975708	0.360249	TRNAK-UUU-8
5	45875760	45975760	0.360736	TRNAK-UUU-8
5	45875879	45975879	0.366962	TRNAK-UUU-8
5	45876003	45976003	0.384226	TRNAK-UUU-8
5	45876061	45976061	0.353741	TRNAK-UUU-8
5	45876162	45976162	0.344065	TRNAK-UUU-8
5	45877008	45977008	0.367551	TRNAK-UUU-8
5	45877009	45977009	0.367581	TRNAK-UUU-8
5	45877079	45977079	0.355565	TRNAK-UUU-8
5	45877155	45977155	0.355608	TRNAK-UUU-8
5	45877303	45977303	0.355859	TRNAK-UUU-8
5	45877391	45977391	0.350743	TRNAK-UUU-8
5	45877713	45977713	0.354931	TRNAK-UUU-8
5	45877791	45977791	0.352371	TRNAK-UUU-8
5	45877825	45977825	0.345181	TRNAK-UUU-8
5	45877882	45977882	0.342773	TRNAK-UUU-8
5	45877989	45977989	0.355955	TRNAK-UUU-8
5	45878186	45978186	0.35869	TRNAK-UUU-8
5	45878908	45978908	0.351811	TRNAK-UUU-8
5	45879120	45979120	0.350537	TRNAK-UUU-8
5	45879448	45979448	0.358797	TRNAK-UUU-8
5	45879601	45979601	0.349486	TRNAK-UUU-8
5	45879922	45979922	0.365495	TRNAK-UUU-8
5	45879958	45979958	0.354432	TRNAK-UUU-8
5	45880011	45980011	0.361027	TRNAK-UUU-8
5	45880064	45980064	0.362298	TRNAK-UUU-8
5	45880101	45980101	0.389126	TRNAK-UUU-8
5	45880133	45980133	0.353019	TRNAK-UUU-8
5	45880202	45980202	0.345129	TRNAK-UUU-8
5	45880247	45980247	0.344832	TRNAK-UUU-8
5	46501461	46601461	0.357799	GRIP1
5	46501502	46601502	0.352669	GRIP1
5	46501727	46601727	0.346983	GRIP1
5	46502077	46602077	0.353896	GRIP1
5	46502112	46602112	0.349985	GRIP1
5	46502412	46602412	0.349465	GRIP1
5	46502452	46602452	0.357332	GRIP1
5	46502623	46602623	0.359281	GRIP1
5	46502764	46602764	0.360763	GRIP1
5	46502837	46602837	0.371461	GRIP1
5	46502953	46602953	0.360335	GRIP1
5	46502981	46602981	0.368525	GRIP1
5	46502982	46602982	0.363747	GRIP1
5	46503165	46603165	0.367207	GRIP1
5	46503199	46603199	0.36209	GRIP1
5	46503325	46603325	0.352285	GRIP1
5	46503459	46603459	0.358744	GRIP1
5	46503678	46603678	0.344534	GRIP1
5	46504260	46604260	0.344993	GRIP1
5	46504567	46604567	0.353992	GRIP1
5	46504826	46604826	0.343926	GRIP1
5	46504872	46604872	0.357212	GRIP1
5	46505043	46605043	0.359255	GRIP1
5	46520925	46620925	0.345321	GRIP1
5	46521069	46621069	0.34325	GRIP1
5	46521070	46621070	0.345617	GRIP1
5	46899508	46999508	0.368292	LLPH
5	46899508	46999508	0.368292	TMBIM4

5	46899508	46999508	0.368292	IRAK3
5	46901102	47001102	0.354656	LLPH
5	46901102	47001102	0.354656	TMBIM4
5	46901102	47001102	0.354656	IRAK3
5	46901388	47001388	0.344561	LLPH
5	46901388	47001388	0.344561	TMBIM4
5	46901388	47001388	0.344561	IRAK3
5	46901733	47001733	0.349756	LLPH
5	46901733	47001733	0.349756	TMBIM4
5	46901733	47001733	0.349756	IRAK3
5	46901821	47001821	0.348963	LLPH
5	46901821	47001821	0.348963	TMBIM4
5	46901821	47001821	0.348963	IRAK3
5	46902876	47002876	0.347614	LLPH
5	46902876	47002876	0.347614	TMBIM4
5	46902876	47002876	0.347614	IRAK3
5	46903623	47003623	0.347989	LLPH
5	46903623	47003623	0.347989	TMBIM4
5	46903623	47003623	0.347989	IRAK3
5	46904091	47004091	0.349474	LLPH
5	46904091	47004091	0.349474	TMBIM4
5	46904091	47004091	0.349474	IRAK3
5	46906217	47006217	0.376432	LLPH
5	46906217	47006217	0.376432	TMBIM4
5	46906217	47006217	0.376432	IRAK3
5	46906529	47006529	0.341851	LLPH
5	46906529	47006529	0.341851	TMBIM4
5	46906529	47006529	0.341851	IRAK3
5	46906573	47006573	0.362573	LLPH
5	46906573	47006573	0.362573	TMBIM4
5	46906573	47006573	0.362573	IRAK3
5	46908618	47008618	0.344669	LLPH
5	46908618	47008618	0.344669	TMBIM4
5	46908618	47008618	0.344669	IRAK3
5	46931149	47031149	0.34467	LLPH
5	46931149	47031149	0.34467	TMBIM4
5	46931149	47031149	0.34467	IRAK3
5	46937791	47037791	0.354199	LLPH
5	46937791	47037791	0.354199	TMBIM4
5	46937791	47037791	0.354199	IRAK3
5	49258961	49358961	0.351211	TMEM5
5	49259009	49359009	0.345316	TMEM5
5	49262440	49362440	0.342071	TMEM5
5	49262459	49362459	0.342071	TMEM5
5	49262633	49362633	0.352307	TMEM5
5	49262634	49362634	0.344047	TMEM5
5	49262658	49362658	0.372419	TMEM5
5	49262708	49362708	0.363265	TMEM5
5	49262731	49362731	0.372634	TMEM5
5	49262739	49362739	0.373525	TMEM5
5	49262751	49362751	0.366035	TMEM5
5	49262770	49362770	0.366035	TMEM5
5	49262789	49362789	0.376088	TMEM5
5	49262818	49362818	0.344222	TMEM5
5	49262835	49362835	0.344222	TMEM5
5	49262879	49362879	0.343604	TMEM5
5	49262898	49362898	0.343604	TMEM5
5	49262924	49362924	0.343604	TMEM5

5	49262936	49362936	0.343604	TMEM5
5	49262978	49362978	0.345983	TMEM5
5	49263018	49363018	0.351509	TMEM5
5	49267351	49367351	0.349791	TMEM5
5	49267837	49367837	0.363363	TMEM5
5	49267870	49367870	0.352482	TMEM5
5	49267877	49367877	0.358094	TMEM5
5	49267963	49367963	0.352419	TMEM5
5	49267966	49367966	0.352419	TMEM5
5	49268071	49368071	0.361326	TMEM5
5	49268115	49368115	0.364789	TMEM5
5	49268124	49368124	0.383413	TMEM5
5	49268148	49368148	0.359783	TMEM5
5	49268155	49368155	0.359783	TMEM5
5	49268183	49368183	0.358238	TMEM5
5	49268221	49368221	0.353155	TMEM5
5	49268237	49368237	0.349674	TMEM5
5	55683817	55783817	0.367065	ZBTB39
5	55683817	55783817	0.367065	GPR182
5	55683817	55783817	0.367065	NEMP1
5	55683817	55783817	0.367065	MYO1A
5	55683817	55783817	0.367065	TAC3
5	55683831	55783831	0.357965	ZBTB39
5	55683831	55783831	0.357965	GPR182
5	55683831	55783831	0.357965	NEMP1
5	55683831	55783831	0.357965	MYO1A
5	55683831	55783831	0.357965	TAC3
5	55683931	55783931	0.359102	ZBTB39
5	55683931	55783931	0.359102	GPR182
5	55683931	55783931	0.359102	NEMP1
5	55683931	55783931	0.359102	MYO1A
5	55683931	55783931	0.359102	TAC3
5	55683998	55783998	0.364812	ZBTB39
5	55683998	55783998	0.364812	GPR182
5	55683998	55783998	0.364812	NEMP1
5	55683998	55783998	0.364812	MYO1A
5	55683998	55783998	0.364812	TAC3
5	55684909	55784909	0.36603	ZBTB39
5	55684909	55784909	0.36603	GPR182
5	55684909	55784909	0.36603	NEMP1
5	55684909	55784909	0.36603	MYO1A
5	55684909	55784909	0.36603	TAC3
5	55684996	55784996	0.371331	ZBTB39
5	55684996	55784996	0.371331	GPR182
5	55684996	55784996	0.371331	NEMP1
5	55684996	55784996	0.371331	MYO1A
5	55684996	55784996	0.371331	TAC3
5	55685055	55785055	0.359974	ZBTB39
5	55685055	55785055	0.359974	GPR182
5	55685055	55785055	0.359974	NEMP1
5	55685055	55785055	0.359974	MYO1A
5	55685055	55785055	0.359974	TAC3
5	55685081	55785081	0.363714	ZBTB39
5	55685081	55785081	0.363714	GPR182
5	55685081	55785081	0.363714	NEMP1
5	55685081	55785081	0.363714	MYO1A
5	55685081	55785081	0.363714	TAC3
5	55685781	55785781	0.36034	ZBTB39

5	55685781	55785781	0.36034	GPR182
5	55685781	55785781	0.36034	NEMP1
5	55685781	55785781	0.36034	MYO1A
5	55685781	55785781	0.36034	TAC3
5	55686169	55786169	0.35597	ZBTB39
5	55686169	55786169	0.35597	GPR182
5	55686169	55786169	0.35597	NEMP1
5	55686169	55786169	0.35597	MYO1A
5	55686169	55786169	0.35597	TAC3
5	55686258	55786258	0.359376	ZBTB39
5	55686258	55786258	0.359376	GPR182
5	55686258	55786258	0.359376	NEMP1
5	55686258	55786258	0.359376	MYO1A
5	55686258	55786258	0.359376	TAC3
5	55686260	55786260	0.359376	ZBTB39
5	55686260	55786260	0.359376	GPR182
5	55686260	55786260	0.359376	NEMP1
5	55686260	55786260	0.359376	MYO1A
5	55686260	55786260	0.359376	TAC3
5	55686342	55786342	0.355597	ZBTB39
5	55686342	55786342	0.355597	GPR182
5	55686342	55786342	0.355597	NEMP1
5	55686342	55786342	0.355597	MYO1A
5	55686342	55786342	0.355597	TAC3
5	55686378	55786378	0.347539	ZBTB39
5	55686378	55786378	0.347539	GPR182
5	55686378	55786378	0.347539	NEMP1
5	55686378	55786378	0.347539	MYO1A
5	55686378	55786378	0.347539	TAC3
5	55687936	55787936	0.351902	ZBTB39
5	55687936	55787936	0.351902	GPR182
5	55687936	55787936	0.351902	LOC102183297
5	55687936	55787936	0.351902	NEMP1
5	55687936	55787936	0.351902	MYO1A
5	55687936	55787936	0.351902	TAC3
5	56686723	56786723	0.353449	SUOX
5	56686723	56786723	0.353449	CDK2
5	56686723	56786723	0.353449	RAB5B
5	56686723	56786723	0.353449	PMEL
5	56686723	56786723	0.353449	DGKA
5	56686723	56786723	0.353449	PYM1
5	57510092	57610092	0.358657	LOC102176500
5	57510092	57610092	0.358657	LOC102177036
5	57510161	57610161	0.352336	LOC102176500
5	57510161	57610161	0.352336	LOC102177036
5	58948210	59048210	0.415817	TRNAD-GUC-6
5	58948210	59048210	0.415817	AMDHD1
5	58948210	59048210	0.415817	HAL
5	58948210	59048210	0.415817	LTA4H
5	58948217	59048217	0.389151	TRNAD-GUC-6
5	58948217	59048217	0.389151	AMDHD1
5	58948217	59048217	0.389151	HAL
5	58948217	59048217	0.389151	LTA4H
5	58948249	59048249	0.408153	TRNAD-GUC-6
5	58948249	59048249	0.408153	AMDHD1
5	58948249	59048249	0.408153	HAL
5	58948249	59048249	0.408153	LTA4H
5	58948252	59048252	0.409163	TRNAD-GUC-6

5	58948252	59048252	0.409163	AMDHD1
5	58948252	59048252	0.409163	HAL
5	58948252	59048252	0.409163	LTA4H
5	58948258	59048258	0.382244	TRNAD-GUC-6
5	58948258	59048258	0.382244	AMDHD1
5	58948258	59048258	0.382244	HAL
5	58948258	59048258	0.382244	LTA4H
5	58948763	59048763	0.350194	TRNAD-GUC-6
5	58948763	59048763	0.350194	AMDHD1
5	58948763	59048763	0.350194	HAL
5	58948763	59048763	0.350194	LTA4H
5	58948784	59048784	0.349859	TRNAD-GUC-6
5	58948784	59048784	0.349859	AMDHD1
5	58948784	59048784	0.349859	HAL
5	58948784	59048784	0.349859	LTA4H
5	58948949	59048949	0.344822	TRNAD-GUC-6
5	58948949	59048949	0.344822	AMDHD1
5	58948949	59048949	0.344822	HAL
5	58948949	59048949	0.344822	LTA4H
5	58949513	59049513	0.344943	TRNAD-GUC-6
5	58949513	59049513	0.344943	AMDHD1
5	58949513	59049513	0.344943	HAL
5	58949513	59049513	0.344943	LTA4H
5	58949585	59049585	0.348739	TRNAD-GUC-6
5	58949585	59049585	0.348739	AMDHD1
5	58949585	59049585	0.348739	HAL
5	58949585	59049585	0.348739	LTA4H
5	63271086	63371086	0.397258	ANO4
5	63271110	63371110	0.389782	ANO4
5	63271117	63371117	0.382645	ANO4
5	63271157	63371157	0.393936	ANO4
5	63271172	63371172	0.39866	ANO4
5	63271352	63371352	0.352144	ANO4
5	63271400	63371400	0.356973	ANO4
5	63271640	63371640	0.353657	ANO4
5	63271741	63371741	0.353894	ANO4
5	63272058	63372058	0.345151	ANO4
5	63273672	63373672	0.357801	ANO4
5	63273682	63373682	0.376587	ANO4
5	63273723	63373723	0.347588	ANO4
5	63649995	63749995	0.358787	ANO4
5	63649995	63749995	0.358787	SLC5A8
5	63650051	63750051	0.369594	ANO4
5	63650051	63750051	0.369594	SLC5A8
5	63650097	63750097	0.373807	ANO4
5	63650097	63750097	0.373807	SLC5A8
5	63650116	63750116	0.363537	ANO4
5	63650116	63750116	0.363537	SLC5A8
5	63650467	63750467	0.361556	ANO4
5	63650467	63750467	0.361556	SLC5A8
5	63650489	63750489	0.365229	ANO4
5	63650489	63750489	0.365229	SLC5A8
5	63650603	63750603	0.359491	ANO4
5	63650603	63750603	0.359491	SLC5A8
5	63650699	63750699	0.359491	ANO4
5	63650699	63750699	0.359491	SLC5A8
5	63650717	63750717	0.362304	ANO4
5	63650717	63750717	0.362304	SLC5A8

5	63650734	63750734	0.354104	ANO4
5	63650734	63750734	0.354104	SLC5A8
5	63650771	63750771	0.350745	ANO4
5	63650771	63750771	0.350745	SLC5A8
5	63650867	63750867	0.346795	ANO4
5	63650867	63750867	0.346795	SLC5A8
5	63650896	63750896	0.344978	ANO4
5	63650896	63750896	0.344978	SLC5A8
5	63650998	63750998	0.344978	ANO4
5	63650998	63750998	0.344978	SLC5A8
5	63651184	63751184	0.344978	ANO4
5	63651184	63751184	0.344978	SLC5A8
5	63651229	63751229	0.344978	ANO4
5	63651229	63751229	0.344978	SLC5A8
5	63651230	63751230	0.344978	ANO4
5	63651230	63751230	0.344978	SLC5A8
5	63651253	63751253	0.373956	ANO4
5	63651253	63751253	0.373956	SLC5A8
5	63651272	63751272	0.364829	ANO4
5	63651272	63751272	0.364829	SLC5A8
5	63651359	63751359	0.364829	ANO4
5	63651359	63751359	0.364829	SLC5A8
5	63651401	63751401	0.364829	ANO4
5	63651401	63751401	0.364829	SLC5A8
5	63651517	63751517	0.364829	ANO4
5	63651517	63751517	0.364829	SLC5A8
5	63651593	63751593	0.369112	ANO4
5	63651593	63751593	0.369112	SLC5A8
5	63651736	63751736	0.364468	ANO4
5	63651736	63751736	0.364468	SLC5A8
5	63651737	63751737	0.359585	ANO4
5	63651737	63751737	0.359585	SLC5A8
5	63651778	63751778	0.359585	ANO4
5	63651778	63751778	0.359585	SLC5A8
5	63651796	63751796	0.359585	ANO4
5	63651796	63751796	0.359585	SLC5A8
5	63651804	63751804	0.359585	ANO4
5	63651804	63751804	0.359585	SLC5A8
5	63651807	63751807	0.375129	ANO4
5	63651807	63751807	0.375129	SLC5A8
5	63651810	63751810	0.362348	ANO4
5	63651810	63751810	0.362348	SLC5A8
5	63651874	63751874	0.342867	ANO4
5	63651874	63751874	0.342867	SLC5A8
5	63651938	63751938	0.34277	ANO4
5	63651938	63751938	0.34277	SLC5A8
5	63651942	63751942	0.34277	ANO4
5	63651942	63751942	0.34277	SLC5A8
5	63651944	63751944	0.34277	ANO4
5	63651944	63751944	0.34277	SLC5A8
5	63651964	63751964	0.34277	ANO4
5	63651964	63751964	0.34277	SLC5A8
5	63652071	63752071	0.34277	ANO4
5	63652071	63752071	0.34277	SLC5A8
5	63652184	63752184	0.349079	ANO4
5	63652184	63752184	0.349079	SLC5A8
5	63652186	63752186	0.349079	ANO4
5	63652186	63752186	0.349079	SLC5A8

5	63652199	63752199	0.349079	ANO4
5	63652199	63752199	0.349079	SLC5A8
5	63652273	63752273	0.348881	ANO4
5	63652273	63752273	0.348881	SLC5A8
5	63652348	63752348	0.351695	ANO4
5	63652348	63752348	0.351695	SLC5A8
5	63652373	63752373	0.366438	ANO4
5	63652373	63752373	0.366438	SLC5A8
5	63652409	63752409	0.377058	ANO4
5	63652409	63752409	0.377058	SLC5A8
5	63652425	63752425	0.381244	ANO4
5	63652425	63752425	0.381244	SLC5A8
5	63652455	63752455	0.386731	ANO4
5	63652455	63752455	0.386731	SLC5A8
5	63652470	63752470	0.377954	ANO4
5	63652470	63752470	0.377954	SLC5A8
5	63652578	63752578	0.373747	ANO4
5	63652578	63752578	0.373747	SLC5A8
5	63652582	63752582	0.373747	ANO4
5	63652582	63752582	0.373747	SLC5A8
5	63652617	63752617	0.373243	ANO4
5	63652617	63752617	0.373243	SLC5A8
5	63652651	63752651	0.372168	ANO4
5	63652651	63752651	0.372168	SLC5A8
5	63652675	63752675	0.359483	ANO4
5	63652675	63752675	0.359483	SLC5A8
5	63652723	63752723	0.3529	ANO4
5	63652723	63752723	0.3529	SLC5A8
5	63652724	63752724	0.3529	ANO4
5	63652724	63752724	0.3529	SLC5A8
5	63652730	63752730	0.3529	ANO4
5	63652730	63752730	0.3529	SLC5A8
5	63652747	63752747	0.352535	ANO4
5	63652747	63752747	0.352535	SLC5A8
5	63670902	63770902	0.35093	ANO4
5	63670902	63770902	0.35093	SLC5A8
5	63670920	63770920	0.36409	ANO4
5	63670920	63770920	0.36409	SLC5A8
5	63671114	63771114	0.35114	ANO4
5	63671114	63771114	0.35114	SLC5A8
5	63671609	63771609	0.366624	ANO4
5	63671609	63771609	0.366624	SLC5A8
5	63671665	63771665	0.397585	ANO4
5	63671665	63771665	0.397585	SLC5A8
5	63671686	63771686	0.366332	ANO4
5	63671686	63771686	0.366332	SLC5A8
5	63671741	63771741	0.366332	ANO4
5	63671741	63771741	0.366332	SLC5A8
5	64059575	64159575	0.391249	MYBPC1
5	64059649	64159649	0.3482	MYBPC1
5	64059701	64159701	0.36767	MYBPC1
5	64059704	64159704	0.42826	MYBPC1
5	64059847	64159847	0.383114	MYBPC1
5	64060352	64160352	0.376954	MYBPC1
5	65890853	65990853	0.34671	STAB2
5	65890957	65990957	0.377263	STAB2
5	65890965	65990965	0.368182	STAB2
5	65891228	65991228	0.369073	STAB2

5	65891304	65991304	0.371817	STAB2
5	65891310	65991310	0.373442	STAB2
5	65891419	65991419	0.366789	STAB2
5	65891451	65991451	0.346433	STAB2
5	65891687	65991687	0.34639	STAB2
5	65891918	65991918	0.352853	STAB2
5	65892014	65992014	0.356704	STAB2
5	65892074	65992074	0.35318	STAB2
5	65892145	65992145	0.365545	STAB2
5	65892146	65992146	0.379784	STAB2
5	65892165	65992165	0.345725	STAB2
5	65892180	65992180	0.342308	STAB2
5	65892278	65992278	0.366513	STAB2
5	66263744	66363744	0.390595	TRNAW-CCA-25
5	66263744	66363744	0.390595	LOC102179170
5	66263744	66363744	0.390595	HSP90B1
5	66263744	66363744	0.390595	TDG
5	66263744	66363744	0.390595	GLT8D2
5	66263744	66363744	0.390595	C5H12orf73
5	66263760	66363760	0.403439	TRNAW-CCA-25
5	66263760	66363760	0.403439	LOC102179170
5	66263760	66363760	0.403439	HSP90B1
5	66263760	66363760	0.403439	TDG
5	66263760	66363760	0.403439	GLT8D2
5	66263760	66363760	0.403439	C5H12orf73
5	66263761	66363761	0.450149	TRNAW-CCA-25
5	66263761	66363761	0.450149	LOC102179170
5	66263761	66363761	0.450149	HSP90B1
5	66263761	66363761	0.450149	TDG
5	66263761	66363761	0.450149	GLT8D2
5	66263761	66363761	0.450149	C5H12orf73
5	66263847	66363847	0.370975	TRNAW-CCA-25
5	66263847	66363847	0.370975	LOC102179170
5	66263847	66363847	0.370975	HSP90B1
5	66263847	66363847	0.370975	TDG
5	66263847	66363847	0.370975	GLT8D2
5	66263847	66363847	0.370975	C5H12orf73
5	66263949	66363949	0.361814	TRNAW-CCA-25
5	66263949	66363949	0.361814	LOC102179170
5	66263949	66363949	0.361814	HSP90B1
5	66263949	66363949	0.361814	TDG
5	66263949	66363949	0.361814	GLT8D2
5	66263949	66363949	0.361814	C5H12orf73
5	66263982	66363982	0.361197	TRNAW-CCA-25
5	66263982	66363982	0.361197	LOC102179170
5	66263982	66363982	0.361197	HSP90B1
5	66263982	66363982	0.361197	TDG
5	66263982	66363982	0.361197	GLT8D2
5	66263982	66363982	0.361197	C5H12orf73
5	66264276	66364276	0.344522	TRNAW-CCA-25
5	66264276	66364276	0.344522	LOC102179170
5	66264276	66364276	0.344522	HSP90B1
5	66264276	66364276	0.344522	TDG
5	66264276	66364276	0.344522	GLT8D2
5	66264276	66364276	0.344522	C5H12orf73
5	66264557	66364557	0.350145	TRNAW-CCA-25
5	66264557	66364557	0.350145	LOC102179170
5	66264557	66364557	0.350145	HSP90B1

5	66264557	66364557	0.350145	TDG
5	66264557	66364557	0.350145	GLT8D2
5	66264557	66364557	0.350145	C5H12orf73
5	66265191	66365191	0.35274	TRNAW-CCA-25
5	66265191	66365191	0.35274	LOC102179170
5	66265191	66365191	0.35274	HSP90B1
5	66265191	66365191	0.35274	TDG
5	66265191	66365191	0.35274	GLT8D2
5	66265191	66365191	0.35274	C5H12orf73
5	66265329	66365329	0.348535	TRNAW-CCA-25
5	66265329	66365329	0.348535	LOC102179170
5	66265329	66365329	0.348535	HSP90B1
5	66265329	66365329	0.348535	TDG
5	66265329	66365329	0.348535	GLT8D2
5	66265329	66365329	0.348535	C5H12orf73
5	66265752	66365752	0.35158	TRNAW-CCA-25
5	66265752	66365752	0.35158	LOC102179170
5	66265752	66365752	0.35158	HSP90B1
5	66265752	66365752	0.35158	TDG
5	66265752	66365752	0.35158	GLT8D2
5	66265752	66365752	0.35158	C5H12orf73
5	66265797	66365797	0.351173	TRNAW-CCA-25
5	66265797	66365797	0.351173	LOC102179170
5	66265797	66365797	0.351173	HSP90B1
5	66265797	66365797	0.351173	TDG
5	66265797	66365797	0.351173	GLT8D2
5	66265797	66365797	0.351173	C5H12orf73
5	66265821	66365821	0.343012	TRNAW-CCA-25
5	66265821	66365821	0.343012	LOC102179170
5	66265821	66365821	0.343012	HSP90B1
5	66265821	66365821	0.343012	TDG
5	66265821	66365821	0.343012	GLT8D2
5	66265821	66365821	0.343012	C5H12orf73
5	66265931	66365931	0.353935	TRNAW-CCA-25
5	66265931	66365931	0.353935	LOC102179170
5	66265931	66365931	0.353935	HSP90B1
5	66265931	66365931	0.353935	TDG
5	66265931	66365931	0.353935	GLT8D2
5	66265931	66365931	0.353935	C5H12orf73
5	66266033	66366033	0.353935	TRNAW-CCA-25
5	66266033	66366033	0.353935	LOC102179170
5	66266033	66366033	0.353935	HSP90B1
5	66266033	66366033	0.353935	TDG
5	66266033	66366033	0.353935	GLT8D2
5	66266033	66366033	0.353935	C5H12orf73
5	66266166	66366166	0.357177	TRNAW-CCA-25
5	66266166	66366166	0.357177	LOC102179170
5	66266166	66366166	0.357177	HSP90B1
5	66266166	66366166	0.357177	TDG
5	66266166	66366166	0.357177	GLT8D2
5	66266166	66366166	0.357177	C5H12orf73
5	66266231	66366231	0.344762	TRNAW-CCA-25
5	66266231	66366231	0.344762	LOC102179170
5	66266231	66366231	0.344762	HSP90B1
5	66266231	66366231	0.344762	TDG
5	66266231	66366231	0.344762	GLT8D2
5	66266231	66366231	0.344762	C5H12orf73
5	66266253	66366253	0.350143	TRNAW-CCA-25

5	66266253	66366253	0.350143	LOC102179170
5	66266253	66366253	0.350143	HSP90B1
5	66266253	66366253	0.350143	TDG
5	66266253	66366253	0.350143	GLT8D2
5	66266253	66366253	0.350143	C5H12orf73
5	66266313	66366313	0.350143	TRNAW-CCA-25
5	66266313	66366313	0.350143	LOC102179170
5	66266313	66366313	0.350143	HSP90B1
5	66266313	66366313	0.350143	TDG
5	66266313	66366313	0.350143	GLT8D2
5	66266313	66366313	0.350143	C5H12orf73
5	66272758	66372758	0.348642	TRNAW-CCA-25
5	66272758	66372758	0.348642	LOC102179170
5	66272758	66372758	0.348642	HSP90B1
5	66272758	66372758	0.348642	TDG
5	66272758	66372758	0.348642	GLT8D2
5	66272758	66372758	0.348642	C5H12orf73
5	66273818	66373818	0.354044	TRNAW-CCA-25
5	66273818	66373818	0.354044	LOC102179170
5	66273818	66373818	0.354044	HSP90B1
5	66273818	66373818	0.354044	TDG
5	66273818	66373818	0.354044	GLT8D2
5	66273818	66373818	0.354044	C5H12orf73
5	66274549	66374549	0.344134	TRNAW-CCA-25
5	66274549	66374549	0.344134	LOC102179170
5	66274549	66374549	0.344134	HSP90B1
5	66274549	66374549	0.344134	TDG
5	66274549	66374549	0.344134	GLT8D2
5	66274549	66374549	0.344134	C5H12orf73
5	66274565	66374565	0.348411	TRNAW-CCA-25
5	66274565	66374565	0.348411	LOC102179170
5	66274565	66374565	0.348411	HSP90B1
5	66274565	66374565	0.348411	TDG
5	66274565	66374565	0.348411	GLT8D2
5	66274565	66374565	0.348411	C5H12orf73
5	66276717	66376717	0.371337	TRNAW-CCA-25
5	66276717	66376717	0.371337	LOC102179170
5	66276717	66376717	0.371337	HSP90B1
5	66276717	66376717	0.371337	TDG
5	66276717	66376717	0.371337	GLT8D2
5	66276717	66376717	0.371337	C5H12orf73
5	66276767	66376767	0.371337	TRNAW-CCA-25
5	66276767	66376767	0.371337	LOC102179170
5	66276767	66376767	0.371337	HSP90B1
5	66276767	66376767	0.371337	TDG
5	66276767	66376767	0.371337	GLT8D2
5	66276767	66376767	0.371337	C5H12orf73
5	66277213	66377213	0.374725	TRNAW-CCA-25
5	66277213	66377213	0.374725	LOC102179170
5	66277213	66377213	0.374725	HSP90B1
5	66277213	66377213	0.374725	TDG
5	66277213	66377213	0.374725	GLT8D2
5	66277213	66377213	0.374725	C5H12orf73
5	66277346	66377346	0.365626	TRNAW-CCA-25
5	66277346	66377346	0.365626	LOC102179170
5	66277346	66377346	0.365626	HSP90B1
5	66277346	66377346	0.365626	TDG
5	66277346	66377346	0.365626	GLT8D2

5	66277346	66377346	0.365626	C5H12orf73
5	66277433	66377433	0.342633	TRNAW-CCA-25
5	66277433	66377433	0.342633	LOC102179170
5	66277433	66377433	0.342633	HSP90B1
5	66277433	66377433	0.342633	TDG
5	66277433	66377433	0.342633	GLT8D2
5	66277433	66377433	0.342633	C5H12orf73
5	66277468	66377468	0.344136	TRNAW-CCA-25
5	66277468	66377468	0.344136	LOC102179170
5	66277468	66377468	0.344136	HSP90B1
5	66277468	66377468	0.344136	TDG
5	66277468	66377468	0.344136	GLT8D2
5	66277468	66377468	0.344136	C5H12orf73
5	66278319	66378319	0.358876	TRNAW-CCA-25
5	66278319	66378319	0.358876	LOC102179170
5	66278319	66378319	0.358876	HSP90B1
5	66278319	66378319	0.358876	TDG
5	66278319	66378319	0.358876	GLT8D2
5	66278319	66378319	0.358876	C5H12orf73
5	66279175	66379175	0.361457	TRNAW-CCA-25
5	66279175	66379175	0.361457	LOC102179170
5	66279175	66379175	0.361457	HSP90B1
5	66279175	66379175	0.361457	TDG
5	66279175	66379175	0.361457	GLT8D2
5	66279175	66379175	0.361457	C5H12orf73
5	66279390	66379390	0.345554	TRNAW-CCA-25
5	66279390	66379390	0.345554	LOC102179170
5	66279390	66379390	0.345554	HSP90B1
5	66279390	66379390	0.345554	TDG
5	66279390	66379390	0.345554	GLT8D2
5	66279390	66379390	0.345554	C5H12orf73
5	66279515	66379515	0.34436	TRNAW-CCA-25
5	66279515	66379515	0.34436	LOC102179170
5	66279515	66379515	0.34436	HSP90B1
5	66279515	66379515	0.34436	TDG
5	66279515	66379515	0.34436	GLT8D2
5	66279515	66379515	0.34436	C5H12orf73
5	66279671	66379671	0.347821	TRNAW-CCA-25
5	66279671	66379671	0.347821	LOC102179170
5	66279671	66379671	0.347821	HSP90B1
5	66279671	66379671	0.347821	TDG
5	66279671	66379671	0.347821	GLT8D2
5	66279671	66379671	0.347821	C5H12orf73
5	66279673	66379673	0.347821	TRNAW-CCA-25
5	66279673	66379673	0.347821	LOC102179170
5	66279673	66379673	0.347821	HSP90B1
5	66279673	66379673	0.347821	TDG
5	66279673	66379673	0.347821	GLT8D2
5	66279673	66379673	0.347821	C5H12orf73
5	66279676	66379676	0.347821	TRNAW-CCA-25
5	66279676	66379676	0.347821	LOC102179170
5	66279676	66379676	0.347821	HSP90B1
5	66279676	66379676	0.347821	TDG
5	66279676	66379676	0.347821	GLT8D2
5	66279676	66379676	0.347821	C5H12orf73
5	68292471	68392471	0.347986	CKAP4
5	68292471	68392471	0.347986	TCP11L2
5	68292561	68392561	0.35507	CKAP4

5	68292561	68392561	0.35507	TCP11L2
5	68292565	68392565	0.362988	CKAP4
5	68292565	68392565	0.362988	TCP11L2
5	68292566	68392566	0.362352	CKAP4
5	68292566	68392566	0.362352	TCP11L2
5	68292612	68392612	0.361493	CKAP4
5	68292612	68392612	0.361493	TCP11L2
5	68292633	68392633	0.360419	CKAP4
5	68292633	68392633	0.360419	TCP11L2
5	68292643	68392643	0.359083	CKAP4
5	68292643	68392643	0.359083	TCP11L2
5	68292671	68392671	0.359083	CKAP4
5	68292671	68392671	0.359083	TCP11L2
5	68292675	68392675	0.351129	CKAP4
5	68292675	68392675	0.351129	TCP11L2
5	68292678	68392678	0.35109	CKAP4
5	68292678	68392678	0.35109	TCP11L2
5	68292682	68392682	0.34381	CKAP4
5	68292682	68392682	0.34381	TCP11L2
5	68599660	68699660	0.37908	RFX4
5	68599743	68699743	0.379001	RFX4
5	68599940	68699940	0.370064	RFX4
5	68600106	68700106	0.374125	RFX4
5	68600296	68700296	0.373139	RFX4
5	68600352	68700352	0.371241	RFX4
5	68600474	68700474	0.366693	RFX4
5	68600495	68700495	0.349289	RFX4
5	68600548	68700548	0.35318	RFX4
5	68601393	68701393	0.348914	RFX4
5	68601560	68701560	0.375929	RFX4
5	68601811	68701811	0.379195	RFX4
5	68601812	68701812	0.378153	RFX4
5	68601837	68701837	0.372738	RFX4
5	68602057	68702057	0.375121	RFX4
5	68602065	68702065	0.37471	RFX4
5	68602145	68702145	0.37902	RFX4
5	68602290	68702290	0.366515	RFX4
5	68602779	68702779	0.366515	RFX4
5	68603065	68703065	0.383117	RFX4
5	68603210	68703210	0.375248	RFX4
5	68603534	68703534	0.374224	RFX4
5	68603616	68703616	0.377326	RFX4
5	68603684	68703684	0.372705	RFX4
5	68603712	68703712	0.372705	RFX4
5	68603838	68703838	0.386942	RFX4
5	68604141	68704141	0.374314	RFX4
5	68604332	68704332	0.38133	RFX4
5	68604430	68704430	0.370456	RFX4
5	68604455	68704455	0.374812	RFX4
5	68604503	68704503	0.382525	RFX4
5	68604513	68704513	0.375463	RFX4
5	68604645	68704645	0.376753	RFX4
5	68604658	68704658	0.368189	RFX4
5	68604932	68704932	0.368189	RFX4
5	68605156	68705156	0.378451	RFX4
5	68605242	68705242	0.355188	RFX4
5	68605265	68705265	0.345814	RFX4
5	68605383	68705383	0.348149	RFX4

5	68605601	68705601	0.34192	RFX4
5	68605732	68705732	0.34192	RFX4
5	68605931	68705931	0.34192	RFX4
5	68606291	68706291	0.346101	RFX4
5	68606343	68706343	0.345226	RFX4
5	68606976	68706976	0.406801	RFX4
5	68607043	68707043	0.405759	RFX4
5	68607044	68707044	0.405523	RFX4
5	68607532	68707532	0.401544	RFX4
5	68607745	68707745	0.401769	RFX4
5	68607829	68707829	0.420007	RFX4
5	68608030	68708030	0.42517	RFX4
5	68608084	68708084	0.427641	RFX4
5	68608140	68708140	0.420393	RFX4
5	68608246	68708246	0.391956	RFX4
5	68608403	68708403	0.389543	RFX4
5	68609001	68709001	0.381606	RFX4
5	68609623	68709623	0.375096	RFX4
5	68609755	68709755	0.375096	RFX4
5	68610508	68710508	0.389414	RFX4
5	68610605	68710605	0.395335	RFX4
5	68610635	68710635	0.395335	RFX4
5	68610641	68710641	0.395335	RFX4
5	68610696	68710696	0.391416	RFX4
5	68610922	68710922	0.393541	RFX4
5	68611062	68711062	0.412521	RFX4
5	68611425	68711425	0.401889	RFX4
5	68611458	68711458	0.401889	RFX4
5	68611656	68711656	0.415014	RFX4
5	68612280	68712280	0.414797	RFX4
5	68612556	68712556	0.417115	RFX4
5	68612616	68712616	0.407349	RFX4
5	68613071	68713071	0.402848	RFX4
5	68613873	68713873	0.393108	RFX4
5	68613931	68713931	0.415414	RFX4
5	68614287	68714287	0.403018	RFX4
5	68614288	68714288	0.403018	RFX4
5	68614323	68714323	0.436355	RFX4
5	68614486	68714486	0.453121	RFX4
5	68614653	68714653	0.457236	RFX4
5	68615034	68715034	0.442601	RFX4
5	68615043	68715043	0.449083	RFX4
5	68615574	68715574	0.455373	RFX4
5	68615886	68715886	0.456882	RFX4
5	68615976	68715976	0.459361	RFX4
5	68616510	68716510	0.455857	RFX4
5	68616900	68716900	0.455965	RFX4
5	68616943	68716943	0.455506	RFX4
5	68617122	68717122	0.486316	RFX4
5	68617462	68717462	0.472197	RFX4
5	68617474	68717474	0.469185	RFX4
5	68617514	68717514	0.473751	RFX4
5	68617565	68717565	0.46487	RFX4
5	68618228	68718228	0.468849	RFX4
5	68618562	68718562	0.461733	RFX4
5	68618617	68718617	0.450013	RFX4
5	68618671	68718671	0.450013	RFX4
5	68618891	68718891	0.450013	RFX4

5	68619152	68719152	0.448461	RFX4
5	68620286	68720286	0.448032	RFX4
5	68620513	68720513	0.470535	RFX4
5	68620540	68720540	0.469154	RFX4
5	68620575	68720575	0.480672	RFX4
5	68620666	68720666	0.478386	RFX4
5	68620782	68720782	0.477425	RFX4
5	68620882	68720882	0.481788	RFX4
5	68620915	68720915	0.468838	RFX4
5	68620920	68720920	0.470597	RFX4
5	68621370	68721370	0.471589	RFX4
5	68621672	68721672	0.475888	RFX4
5	68621942	68721942	0.47809	RFX4
5	68621977	68721977	0.477263	RFX4
5	68622060	68722060	0.478366	RFX4
5	68622164	68722164	0.479502	RFX4
5	68622327	68722327	0.484939	RFX4
5	68622328	68722328	0.483783	RFX4
5	68622357	68722357	0.48208	RFX4
5	68622433	68722433	0.481404	RFX4
5	68622440	68722440	0.481404	RFX4
5	68622504	68722504	0.481728	RFX4
5	68622618	68722618	0.480099	RFX4
5	68622683	68722683	0.481607	RFX4
5	68622698	68722698	0.476683	RFX4
5	68622769	68722769	0.479907	RFX4
5	68622824	68722824	0.469816	RFX4
5	68623287	68723287	0.460655	RFX4
5	68623312	68723312	0.459579	RFX4
5	68623507	68723507	0.467907	RFX4
5	68623974	68723974	0.462099	RFX4
5	68624150	68724150	0.460964	RFX4
5	68624561	68724561	0.464607	RFX4
5	68624688	68724688	0.465618	RFX4
5	68624689	68724689	0.465618	RFX4
5	68624857	68724857	0.465642	RFX4
5	68625016	68725016	0.477979	RFX4
5	68625458	68725458	0.479202	RFX4
5	68625793	68725793	0.459127	RFX4
5	68625916	68725916	0.465314	RFX4
5	68625923	68725923	0.460686	RFX4
5	68626204	68726204	0.451657	RFX4
5	68626333	68726333	0.434117	RFX4
5	68626686	68726686	0.433539	RFX4
5	68626834	68726834	0.437607	RFX4
5	68627266	68727266	0.437607	RFX4
5	68627375	68727375	0.437577	RFX4
5	68627535	68727535	0.437885	RFX4
5	68627855	68727855	0.43401	RFX4
5	68628075	68728075	0.464035	RFX4
5	68628595	68728595	0.458582	RFX4
5	68628660	68728660	0.466384	RFX4
5	68628783	68728783	0.46665	RFX4
5	68628848	68728848	0.464583	RFX4
5	68628860	68728860	0.465157	RFX4
5	68628896	68728896	0.465722	RFX4
5	68628989	68728989	0.458819	RFX4
5	68629022	68729022	0.460518	RFX4

5	68629040	68729040	0.454637	RFX4
5	68629194	68729194	0.457899	RFX4
5	68629324	68729324	0.457899	RFX4
5	68629397	68729397	0.465242	RFX4
5	68629452	68729452	0.471849	RFX4
5	68629545	68729545	0.474493	RFX4
5	68629560	68729560	0.489778	RFX4
5	68629829	68729829	0.490584	RFX4
5	68630824	68730824	0.4487	RFX4
5	68630928	68730928	0.451628	RFX4
5	68630984	68730984	0.454383	RFX4
5	68631307	68731307	0.451161	RFX4
5	68631344	68731344	0.452125	RFX4
5	68631561	68731561	0.453226	RFX4
5	68631580	68731580	0.43511	RFX4
5	68632707	68732707	0.437177	RFX4
5	68632996	68732996	0.448085	RFX4
5	68633121	68733121	0.449873	RFX4
5	68633205	68733205	0.457632	RFX4
5	68633293	68733293	0.458684	RFX4
5	68633321	68733321	0.453598	RFX4
5	68633585	68733585	0.448497	RFX4
5	68633648	68733648	0.452069	RFX4
5	68634017	68734017	0.451254	RFX4
5	68634184	68734184	0.452256	RFX4
5	68634494	68734494	0.456275	RFX4
5	68634771	68734771	0.452823	RFX4
5	68634914	68734914	0.455356	RFX4
5	68635118	68735118	0.452743	RFX4
5	68635325	68735325	0.448611	RFX4
5	68635524	68735524	0.44756	RFX4
5	68636058	68736058	0.428627	RFX4
5	68636113	68736113	0.42974	RFX4
5	68637299	68737299	0.425158	RFX4
5	68637998	68737998	0.41848	RFX4
5	68638534	68738534	0.430431	RFX4
5	68638768	68738768	0.44417	RFX4
5	68638853	68738853	0.453139	RFX4
5	68639302	68739302	0.450642	RFX4
5	68639500	68739500	0.452149	RFX4
5	68639729	68739729	0.45632	RFX4
5	68639765	68739765	0.45673	RFX4
5	68639803	68739803	0.454691	RFX4
5	68639975	68739975	0.452239	RFX4
5	68640083	68740083	0.452084	RFX4
5	68640434	68740434	0.439289	RFX4
5	68640600	68740600	0.43326	RFX4
5	68641138	68741138	0.432557	RFX4
5	68642239	68742239	0.398698	RFX4
5	68642426	68742426	0.410627	RFX4
5	68642530	68742530	0.415765	RFX4
5	68642546	68742546	0.407693	RFX4
5	68642815	68742815	0.405045	RFX4
5	68642999	68742999	0.420801	RFX4
5	68643118	68743118	0.412419	RFX4
5	68643524	68743524	0.429091	RFX4
5	68643550	68743550	0.392378	RFX4
5	68643706	68743706	0.397419	RFX4

5	68644288	68744288	0.388686	RFX4
5	68644930	68744930	0.394733	RFX4
5	68645081	68745081	0.39032	RFX4
5	68645154	68745154	0.390998	RFX4
5	68645300	68745300	0.403188	RFX4
5	68645333	68745333	0.401522	RFX4
5	68645425	68745425	0.402543	RFX4
5	68646288	68746288	0.414603	RFX4
5	68646483	68746483	0.395021	RFX4
5	68646837	68746837	0.390019	RFX4
5	68646930	68746930	0.398088	RFX4
5	68647046	68747046	0.394334	RFX4
5	68647724	68747724	0.39333	RFX4
5	68648258	68748258	0.387059	RFX4
5	68648337	68748337	0.396656	RFX4
5	68648732	68748732	0.392069	RFX4
5	68649022	68749022	0.387439	RFX4
5	68649337	68749337	0.389513	RFX4
5	68649873	68749873	0.380218	RFX4
5	68650134	68750134	0.418846	RFX4
5	68650166	68750166	0.414154	RFX4
5	68650282	68750282	0.417148	RFX4
5	68650327	68750327	0.422364	RFX4
5	68650331	68750331	0.407514	RFX4
5	68651288	68751288	0.407022	RFX4
5	68651914	68751914	0.411482	RFX4
5	68651914	68751914	0.411482	RIC8B
5	68651972	68751972	0.413047	RFX4
5	68651972	68751972	0.413047	RIC8B
5	68652142	68752142	0.414076	RFX4
5	68652142	68752142	0.414076	RIC8B
5	68652509	68752509	0.410534	RFX4
5	68652509	68752509	0.410534	RIC8B
5	68652530	68752530	0.407605	RFX4
5	68652530	68752530	0.407605	RIC8B
5	68652533	68752533	0.407685	RFX4
5	68652533	68752533	0.407685	RIC8B
5	68652634	68752634	0.41621	RFX4
5	68652634	68752634	0.41621	RIC8B
5	68652998	68752998	0.41196	RFX4
5	68652998	68752998	0.41196	RIC8B
5	68653272	68753272	0.414019	RFX4
5	68653272	68753272	0.414019	RIC8B
5	68653375	68753375	0.414019	RFX4
5	68653375	68753375	0.414019	RIC8B
5	68653429	68753429	0.416634	RFX4
5	68653429	68753429	0.416634	RIC8B
5	68653593	68753593	0.417311	RFX4
5	68653593	68753593	0.417311	RIC8B
5	68653611	68753611	0.409156	RFX4
5	68653611	68753611	0.409156	RIC8B
5	68653933	68753933	0.409156	RFX4
5	68653933	68753933	0.409156	RIC8B
5	68654083	68754083	0.40023	RFX4
5	68654083	68754083	0.40023	RIC8B
5	68654680	68754680	0.417052	RFX4
5	68654680	68754680	0.417052	RIC8B
5	68654819	68754819	0.422157	RFX4

5	68654819	68754819	0.422157	RIC8B
5	68654872	68754872	0.422524	RFX4
5	68654872	68754872	0.422524	RIC8B
5	68654950	68754950	0.421305	RFX4
5	68654950	68754950	0.421305	RIC8B
5	68655024	68755024	0.415953	RFX4
5	68655024	68755024	0.415953	RIC8B
5	68655206	68755206	0.416397	RFX4
5	68655206	68755206	0.416397	RIC8B
5	68655222	68755222	0.417521	RFX4
5	68655222	68755222	0.417521	RIC8B
5	68655407	68755407	0.411207	RFX4
5	68655407	68755407	0.411207	RIC8B
5	68655432	68755432	0.405088	RFX4
5	68655432	68755432	0.405088	RIC8B
5	68655954	68755954	0.405791	RFX4
5	68655954	68755954	0.405791	RIC8B
5	68656005	68756005	0.407493	RFX4
5	68656005	68756005	0.407493	RIC8B
5	68656292	68756292	0.407493	RFX4
5	68656292	68756292	0.407493	RIC8B
5	68656366	68756366	0.415169	RFX4
5	68656366	68756366	0.415169	RIC8B
5	68656540	68756540	0.414617	RFX4
5	68656540	68756540	0.414617	RIC8B
5	68656708	68756708	0.426601	RFX4
5	68656708	68756708	0.426601	RIC8B
5	68656732	68756732	0.422971	RFX4
5	68656732	68756732	0.422971	RIC8B
5	68656755	68756755	0.422977	RFX4
5	68656755	68756755	0.422977	RIC8B
5	68657019	68757019	0.421972	RFX4
5	68657019	68757019	0.421972	RIC8B
5	68657231	68757231	0.422112	RFX4
5	68657231	68757231	0.422112	RIC8B
5	68657446	68757446	0.410736	RFX4
5	68657446	68757446	0.410736	RIC8B
5	68657634	68757634	0.401509	RFX4
5	68657634	68757634	0.401509	RIC8B
5	68658282	68758282	0.398844	RFX4
5	68658282	68758282	0.398844	RIC8B
5	68659078	68759078	0.39871	RFX4
5	68659078	68759078	0.39871	RIC8B
5	68659242	68759242	0.392235	RFX4
5	68659242	68759242	0.392235	RIC8B
5	68659321	68759321	0.393131	RFX4
5	68659321	68759321	0.393131	RIC8B
5	68659651	68759651	0.397437	RFX4
5	68659651	68759651	0.397437	RIC8B
5	68659773	68759773	0.393777	RFX4
5	68659773	68759773	0.393777	RIC8B
5	68660932	68760932	0.394658	RFX4
5	68660932	68760932	0.394658	RIC8B
5	68661024	68761024	0.406192	RFX4
5	68661024	68761024	0.406192	RIC8B
5	68661466	68761466	0.410439	RFX4
5	68661466	68761466	0.410439	RIC8B
5	68661711	68761711	0.403186	RFX4

5	68661711	68761711	0.403186	RIC8B
5	68662498	68762498	0.395605	RFX4
5	68662498	68762498	0.395605	RIC8B
5	68662499	68762499	0.421429	RFX4
5	68662499	68762499	0.421429	RIC8B
5	68662500	68762500	0.421943	RFX4
5	68662500	68762500	0.421943	RIC8B
5	68662563	68762563	0.427671	RFX4
5	68662563	68762563	0.427671	RIC8B
5	68662665	68762665	0.426622	RFX4
5	68662665	68762665	0.426622	RIC8B
5	68662792	68762792	0.426999	RFX4
5	68662792	68762792	0.426999	RIC8B
5	68662840	68762840	0.426081	RFX4
5	68662840	68762840	0.426081	RIC8B
5	68662923	68762923	0.425729	RFX4
5	68662923	68762923	0.425729	RIC8B
5	68663203	68763203	0.426769	RFX4
5	68663203	68763203	0.426769	RIC8B
5	68663232	68763232	0.428324	RFX4
5	68663232	68763232	0.428324	RIC8B
5	68663237	68763237	0.427411	RFX4
5	68663237	68763237	0.427411	RIC8B
5	68663249	68763249	0.427607	RFX4
5	68663249	68763249	0.427607	RIC8B
5	68663357	68763357	0.426748	RFX4
5	68663357	68763357	0.426748	RIC8B
5	68663387	68763387	0.432512	RFX4
5	68663387	68763387	0.432512	RIC8B
5	68663730	68763730	0.42704	RFX4
5	68663730	68763730	0.42704	RIC8B
5	68663881	68763881	0.422946	RFX4
5	68663881	68763881	0.422946	RIC8B
5	68663923	68763923	0.424682	RFX4
5	68663923	68763923	0.424682	RIC8B
5	68664079	68764079	0.47861	RFX4
5	68664079	68764079	0.47861	RIC8B
5	68664515	68764515	0.480606	RFX4
5	68664515	68764515	0.480606	RIC8B
5	68664812	68764812	0.487643	RFX4
5	68664812	68764812	0.487643	RIC8B
5	68665539	68765539	0.47834	RFX4
5	68665539	68765539	0.47834	RIC8B
5	68665617	68765617	0.47834	RFX4
5	68665617	68765617	0.47834	RIC8B
5	68665821	68765821	0.484258	RFX4
5	68665821	68765821	0.484258	RIC8B
5	68665873	68765873	0.476648	RFX4
5	68665873	68765873	0.476648	RIC8B
5	68665953	68765953	0.467019	RFX4
5	68665953	68765953	0.467019	RIC8B
5	68666309	68766309	0.470106	RFX4
5	68666309	68766309	0.470106	RIC8B
5	68666405	68766405	0.448238	RFX4
5	68666405	68766405	0.448238	RIC8B
5	68667034	68767034	0.445853	RFX4
5	68667034	68767034	0.445853	RIC8B
5	68667853	68767853	0.447045	RFX4

5	68667853	68767853	0.447045	RIC8B
5	68668169	68768169	0.453852	RFX4
5	68668169	68768169	0.453852	RIC8B
5	68668334	68768334	0.445081	RFX4
5	68668334	68768334	0.445081	RIC8B
5	68668358	68768358	0.444677	RFX4
5	68668358	68768358	0.444677	RIC8B
5	68668513	68768513	0.444677	RFX4
5	68668513	68768513	0.444677	RIC8B
5	68669055	68769055	0.446418	RFX4
5	68669055	68769055	0.446418	RIC8B
5	68669819	68769819	0.434696	RFX4
5	68669819	68769819	0.434696	RIC8B
5	68670317	68770317	0.435423	RFX4
5	68670317	68770317	0.435423	RIC8B
5	68670667	68770667	0.444427	RFX4
5	68670667	68770667	0.444427	RIC8B
5	68671121	68771121	0.444427	RFX4
5	68671121	68771121	0.444427	RIC8B
5	68671238	68771238	0.440752	RFX4
5	68671238	68771238	0.440752	RIC8B
5	68671258	68771258	0.439439	RFX4
5	68671258	68771258	0.439439	RIC8B
5	68672063	68772063	0.439529	RFX4
5	68672063	68772063	0.439529	RIC8B
5	68672347	68772347	0.444022	RFX4
5	68672347	68772347	0.444022	RIC8B
5	68672714	68772714	0.446094	RFX4
5	68672714	68772714	0.446094	RIC8B
5	68672809	68772809	0.444618	RFX4
5	68672809	68772809	0.444618	RIC8B
5	68673139	68773139	0.442284	RFX4
5	68673139	68773139	0.442284	RIC8B
5	68674144	68774144	0.39829	RFX4
5	68674144	68774144	0.39829	RIC8B
5	68674150	68774150	0.378559	RFX4
5	68674150	68774150	0.378559	RIC8B
5	68674151	68774151	0.378559	RFX4
5	68674151	68774151	0.378559	RIC8B
5	68674202	68774202	0.388817	RFX4
5	68674202	68774202	0.388817	RIC8B
5	68674365	68774365	0.445432	RFX4
5	68674365	68774365	0.445432	RIC8B
5	68674602	68774602	0.445383	RFX4
5	68674602	68774602	0.445383	RIC8B
5	68675910	68775910	0.437047	RFX4
5	68675910	68775910	0.437047	RIC8B
5	68675929	68775929	0.443528	RFX4
5	68675929	68775929	0.443528	RIC8B
5	68676563	68776563	0.441151	RFX4
5	68676563	68776563	0.441151	RIC8B
5	68676635	68776635	0.412671	RFX4
5	68676635	68776635	0.412671	RIC8B
5	68676649	68776649	0.394008	RFX4
5	68676649	68776649	0.394008	RIC8B
5	68676899	68776899	0.424338	RFX4
5	68676899	68776899	0.424338	RIC8B
5	68677084	68777084	0.417963	RFX4

5	68677084	68777084	0.417963	RIC8B
5	68677159	68777159	0.427506	RFX4
5	68677159	68777159	0.427506	RIC8B
5	68678831	68778831	0.421188	RFX4
5	68678831	68778831	0.421188	RIC8B
5	68679158	68779158	0.421188	RFX4
5	68679158	68779158	0.421188	RIC8B
5	68679327	68779327	0.428682	RFX4
5	68679327	68779327	0.428682	RIC8B
5	68679535	68779535	0.435555	RFX4
5	68679535	68779535	0.435555	RIC8B
5	68680102	68780102	0.434086	RFX4
5	68680102	68780102	0.434086	RIC8B
5	68680856	68780856	0.441686	RFX4
5	68680856	68780856	0.441686	RIC8B
5	68681056	68781056	0.44478	RFX4
5	68681056	68781056	0.44478	RIC8B
5	68681110	68781110	0.441271	RFX4
5	68681110	68781110	0.441271	RIC8B
5	68681504	68781504	0.442107	RFX4
5	68681504	68781504	0.442107	RIC8B
5	68681801	68781801	0.458021	RFX4
5	68681801	68781801	0.458021	RIC8B
5	68681916	68781916	0.438011	RFX4
5	68681916	68781916	0.438011	RIC8B
5	68682003	68782003	0.437537	RFX4
5	68682003	68782003	0.437537	RIC8B
5	68682274	68782274	0.435725	RFX4
5	68682274	68782274	0.435725	RIC8B
5	68682512	68782512	0.435611	RFX4
5	68682512	68782512	0.435611	RIC8B
5	68682719	68782719	0.433201	RFX4
5	68682719	68782719	0.433201	RIC8B
5	68683911	68783911	0.432738	RFX4
5	68683911	68783911	0.432738	RIC8B
5	68684090	68784090	0.431263	RFX4
5	68684090	68784090	0.431263	RIC8B
5	68684647	68784647	0.4398	RFX4
5	68684647	68784647	0.4398	RIC8B
5	68684756	68784756	0.417841	RFX4
5	68684756	68784756	0.417841	RIC8B
5	68686370	68786370	0.409867	RFX4
5	68686370	68786370	0.409867	RIC8B
5	68687055	68787055	0.411699	RFX4
5	68687055	68787055	0.411699	RIC8B
5	68687578	68787578	0.424081	RFX4
5	68687578	68787578	0.424081	RIC8B
5	68687897	68787897	0.422906	RFX4
5	68687897	68787897	0.422906	RIC8B
5	68687994	68787994	0.425399	RFX4
5	68687994	68787994	0.425399	RIC8B
5	68688306	68788306	0.413845	RFX4
5	68688306	68788306	0.413845	RIC8B
5	68688494	68788494	0.41185	RFX4
5	68688494	68788494	0.41185	RIC8B
5	68690177	68790177	0.413121	RFX4
5	68690177	68790177	0.413121	RIC8B
5	68690239	68790239	0.409372	RFX4

5	68690239	68790239	0.409372	RIC8B
5	68691230	68791230	0.42372	RFX4
5	68691230	68791230	0.42372	RIC8B
5	68691347	68791347	0.408815	RFX4
5	68691347	68791347	0.408815	RIC8B
5	68691587	68791587	0.416013	RFX4
5	68691587	68791587	0.416013	RIC8B
5	68691680	68791680	0.412987	RFX4
5	68691680	68791680	0.412987	RIC8B
5	68693425	68793425	0.376706	RFX4
5	68693425	68793425	0.376706	RIC8B
5	68693594	68793594	0.396049	RFX4
5	68693594	68793594	0.396049	RIC8B
5	68693894	68793894	0.396049	RFX4
5	68693894	68793894	0.396049	RIC8B
5	68693998	68793998	0.398688	RFX4
5	68693998	68793998	0.398688	RIC8B
5	68695355	68795355	0.377186	RFX4
5	68695355	68795355	0.377186	RIC8B
5	68695736	68795736	0.359621	RFX4
5	68695736	68795736	0.359621	RIC8B
5	68696672	68796672	0.383182	RFX4
5	68696672	68796672	0.383182	RIC8B
5	68698731	68798731	0.368712	RFX4
5	68698731	68798731	0.368712	RIC8B
5	68699217	68799217	0.353538	RFX4
5	68699217	68799217	0.353538	RIC8B
5	68699889	68799889	0.35952	RFX4
5	68699889	68799889	0.35952	RIC8B
5	68700052	68800052	0.344161	RFX4
5	68700052	68800052	0.344161	RIC8B
5	68700359	68800359	0.355861	RFX4
5	68700359	68800359	0.355861	RIC8B
5	68701344	68801344	0.361666	RFX4
5	68701344	68801344	0.361666	RIC8B
5	68701391	68801391	0.382374	RFX4
5	68701391	68801391	0.382374	RIC8B
5	68702219	68802219	0.386449	RFX4
5	68702219	68802219	0.386449	RIC8B
5	68703159	68803159	0.355741	RFX4
5	68703159	68803159	0.355741	RIC8B
5	68703307	68803307	0.355741	RFX4
5	68703307	68803307	0.355741	RIC8B
5	68704513	68804513	0.348443	RFX4
5	68704513	68804513	0.348443	RIC8B
5	68708170	68808170	0.347126	RFX4
5	68708170	68808170	0.347126	RIC8B
5	68708401	68808401	0.350431	RFX4
5	68708401	68808401	0.350431	RIC8B
5	68708561	68808561	0.359507	RFX4
5	68708561	68808561	0.359507	RIC8B
5	68708899	68808899	0.354195	RFX4
5	68708899	68808899	0.354195	RIC8B
5	68709475	68809475	0.347613	RFX4
5	68709475	68809475	0.347613	RIC8B
5	68710255	68810255	0.359171	RFX4
5	68710255	68810255	0.359171	RIC8B
5	68710749	68810749	0.363613	RFX4

5	68710749	68810749	0.363613	RIC8B
5	68710759	68810759	0.376817	RFX4
5	68710759	68810759	0.376817	RIC8B
5	68710769	68810769	0.373102	RFX4
5	68710769	68810769	0.373102	RIC8B
5	68711123	68811123	0.373258	RFX4
5	68711123	68811123	0.373258	RIC8B
5	68712083	68812083	0.352279	RFX4
5	68712083	68812083	0.352279	RIC8B
5	68713076	68813076	0.353003	RFX4
5	68713076	68813076	0.353003	RIC8B
5	68713403	68813403	0.35034	RFX4
5	68713403	68813403	0.35034	RIC8B
5	68716317	68816317	0.348019	RFX4
5	68716317	68816317	0.348019	RIC8B
5	68716375	68816375	0.361787	RFX4
5	68716375	68816375	0.361787	RIC8B
5	68716567	68816567	0.342449	RFX4
5	68716567	68816567	0.342449	RIC8B
5	68716700	68816700	0.346379	RFX4
5	68716700	68816700	0.346379	RIC8B
5	68717904	68817904	0.342402	RFX4
5	68717904	68817904	0.342402	RIC8B
5	68718641	68818641	0.345136	RFX4
5	68718641	68818641	0.345136	RIC8B
5	68718670	68818670	0.357081	RFX4
5	68718670	68818670	0.357081	RIC8B
5	68810130	68910130	0.345452	RIC8B
5	68810682	68910682	0.345224	RIC8B
5	68811004	68911004	0.35637	RIC8B
5	68811494	68911494	0.365557	RIC8B
5	68811664	68911664	0.34281	RIC8B
5	69424201	69524201	0.34383	TRNAG-CCC-30
5	69424201	69524201	0.34383	BTBD11
5	69424570	69524570	0.344867	TRNAG-CCC-30
5	69424570	69524570	0.344867	BTBD11
5	69425241	69525241	0.352277	TRNAG-CCC-30
5	69425241	69525241	0.352277	BTBD11
5	69425648	69525648	0.355266	TRNAG-CCC-30
5	69425648	69525648	0.355266	BTBD11
5	69425658	69525658	0.350368	TRNAG-CCC-30
5	69425658	69525658	0.350368	BTBD11
5	69425700	69525700	0.365328	TRNAG-CCC-30
5	69425700	69525700	0.365328	BTBD11
5	69425828	69525828	0.368389	TRNAG-CCC-30
5	69425828	69525828	0.368389	BTBD11
5	69425833	69525833	0.36365	TRNAG-CCC-30
5	69425833	69525833	0.36365	BTBD11
5	69425941	69525941	0.378304	TRNAG-CCC-30
5	69425941	69525941	0.378304	BTBD11
5	69425980	69525980	0.383492	TRNAG-CCC-30
5	69425980	69525980	0.383492	BTBD11
5	69426045	69526045	0.384014	TRNAG-CCC-30
5	69426045	69526045	0.384014	BTBD11
5	69426062	69526062	0.381392	TRNAG-CCC-30
5	69426062	69526062	0.381392	BTBD11
5	69426208	69526208	0.385806	TRNAG-CCC-30
5	69426208	69526208	0.385806	BTBD11

5	69426216	69526216	0.38511	TRNAG-CCC-30
5	69426216	69526216	0.38511	BTBD11
5	69426300	69526300	0.38437	TRNAG-CCC-30
5	69426300	69526300	0.38437	BTBD11
5	69426411	69526411	0.384974	TRNAG-CCC-30
5	69426411	69526411	0.384974	BTBD11
5	69426445	69526445	0.384974	TRNAG-CCC-30
5	69426445	69526445	0.384974	BTBD11
5	69426546	69526546	0.384328	TRNAG-CCC-30
5	69426546	69526546	0.384328	BTBD11
5	69426610	69526610	0.384303	TRNAG-CCC-30
5	69426610	69526610	0.384303	BTBD11
5	69426728	69526728	0.382288	TRNAG-CCC-30
5	69426728	69526728	0.382288	BTBD11
5	69426883	69526883	0.380227	TRNAG-CCC-30
5	69426883	69526883	0.380227	BTBD11
5	69427004	69527004	0.384016	TRNAG-CCC-30
5	69427004	69527004	0.384016	BTBD11
5	69427199	69527199	0.372282	TRNAG-CCC-30
5	69427199	69527199	0.372282	BTBD11
5	69427234	69527234	0.372282	TRNAG-CCC-30
5	69427234	69527234	0.372282	BTBD11
5	69427367	69527367	0.368417	TRNAG-CCC-30
5	69427367	69527367	0.368417	BTBD11
5	69427393	69527393	0.37061	TRNAG-CCC-30
5	69427393	69527393	0.37061	BTBD11
5	69428224	69528224	0.348093	TRNAG-CCC-30
5	69428224	69528224	0.348093	BTBD11
5	69429469	69529469	0.350619	TRNAG-CCC-30
5	69429469	69529469	0.350619	BTBD11
5	69429755	69529755	0.357154	TRNAG-CCC-30
5	69429755	69529755	0.357154	BTBD11
5	69432781	69532781	0.346355	TRNAG-CCC-30
5	69432781	69532781	0.346355	BTBD11
5	69432871	69532871	0.342607	TRNAG-CCC-30
5	69432871	69532871	0.342607	BTBD11
5	69432958	69532958	0.346359	TRNAG-CCC-30
5	69432958	69532958	0.346359	BTBD11
5	69433194	69533194	0.356051	TRNAG-CCC-30
5	69433194	69533194	0.356051	BTBD11
5	69433274	69533274	0.350099	TRNAG-CCC-30
5	69433274	69533274	0.350099	BTBD11
5	69433393	69533393	0.362767	TRNAG-CCC-30
5	69433393	69533393	0.362767	BTBD11
5	69433432	69533432	0.359178	TRNAG-CCC-30
5	69433432	69533432	0.359178	BTBD11
5	69433546	69533546	0.357379	TRNAG-CCC-30
5	69433546	69533546	0.357379	BTBD11
5	69433700	69533700	0.350374	TRNAG-CCC-30
5	69433700	69533700	0.350374	BTBD11
5	69433771	69533771	0.353136	TRNAG-CCC-30
5	69433771	69533771	0.353136	BTBD11
5	69434009	69534009	0.345796	TRNAG-CCC-30
5	69434009	69534009	0.345796	BTBD11
5	69434612	69534612	0.3444	TRNAG-CCC-30
5	69434612	69534612	0.3444	BTBD11
5	69435124	69535124	0.346873	TRNAG-CCC-30
5	69435124	69535124	0.346873	BTBD11

5	69435203	69535203	0.347544	TRNAG-CCC-30
5	69435203	69535203	0.347544	BTBD11
5	69435966	69535966	0.344562	TRNAG-CCC-30
5	69435966	69535966	0.344562	BTBD11
5	69435973	69535973	0.344562	TRNAG-CCC-30
5	69435973	69535973	0.344562	BTBD11
5	69436095	69536095	0.350505	TRNAG-CCC-30
5	69436095	69536095	0.350505	BTBD11
5	69437375	69537375	0.359892	TRNAG-CCC-30
5	69437375	69537375	0.359892	BTBD11
5	69439457	69539457	0.353008	TRNAG-CCC-30
5	69439457	69539457	0.353008	BTBD11
5	69439515	69539515	0.365063	TRNAG-CCC-30
5	69439515	69539515	0.365063	BTBD11
5	71959949	72059949	0.355412	TRNAR-CCU-7
5	71959949	72059949	0.355412	ISX
5	71959988	72059988	0.498095	TRNAR-CCU-7
5	71959988	72059988	0.498095	ISX
5	71959998	72059998	0.398368	TRNAR-CCU-7
5	71959998	72059998	0.398368	ISX
5	71960002	72060002	0.446416	TRNAR-CCU-7
5	71960002	72060002	0.446416	ISX
5	71960017	72060017	0.36312	TRNAR-CCU-7
5	71960017	72060017	0.36312	ISX
5	71960629	72060629	0.365488	TRNAR-CCU-7
5	71960629	72060629	0.365488	ISX
5	71960636	72060636	0.387895	TRNAR-CCU-7
5	71960636	72060636	0.387895	ISX
5	71961030	72061030	0.378672	TRNAR-CCU-7
5	71961030	72061030	0.378672	ISX
5	71961103	72061103	0.387555	TRNAR-CCU-7
5	71961103	72061103	0.387555	ISX
5	71961122	72061122	0.386714	TRNAR-CCU-7
5	71961122	72061122	0.386714	ISX
5	71961211	72061211	0.386714	TRNAR-CCU-7
5	71961211	72061211	0.386714	ISX
5	71961255	72061255	0.38867	TRNAR-CCU-7
5	71961255	72061255	0.38867	ISX
5	71961284	72061284	0.385289	TRNAR-CCU-7
5	71961284	72061284	0.385289	ISX
5	71961688	72061688	0.376987	TRNAR-CCU-7
5	71961688	72061688	0.376987	ISX
5	71961697	72061697	0.402339	TRNAR-CCU-7
5	71961697	72061697	0.402339	ISX
5	71962035	72062035	0.405697	TRNAR-CCU-7
5	71962035	72062035	0.405697	ISX
5	71962041	72062041	0.3996	TRNAR-CCU-7
5	71962041	72062041	0.3996	ISX
5	71962105	72062105	0.3996	ISX
5	71962243	72062243	0.3996	ISX
5	71962409	72062409	0.401429	ISX
5	71962523	72062523	0.39842	ISX
5	71962594	72062594	0.409374	ISX
5	71962615	72062615	0.433835	ISX
5	71962700	72062700	0.431446	ISX
5	71962949	72062949	0.44626	ISX
5	71962966	72062966	0.44792	ISX
5	71963247	72063247	0.436215	ISX

5	71963253	72063253	0.436215	ISX
5	71963291	72063291	0.434054	ISX
5	71963408	72063408	0.431471	ISX
5	71963632	72063632	0.431471	ISX
5	71963739	72063739	0.432864	ISX
5	71963856	72063856	0.437501	ISX
5	71963881	72063881	0.427983	ISX
5	71964004	72064004	0.42486	ISX
5	71964055	72064055	0.436899	ISX
5	71964451	72064451	0.436899	ISX
5	71964479	72064479	0.436899	ISX
5	71964507	72064507	0.443657	ISX
5	71964623	72064623	0.439497	ISX
5	71964883	72064883	0.443685	ISX
5	71964940	72064940	0.435129	ISX
5	71964978	72064978	0.431134	ISX
5	71964988	72064988	0.403035	ISX
5	71965199	72065199	0.347994	ISX
5	71965303	72065303	0.344875	ISX
5	71965440	72065440	0.34573	ISX
5	71965488	72065488	0.347753	ISX
5	71965551	72065551	0.346281	ISX
5	71965552	72065552	0.347631	ISX
5	71965657	72065657	0.3476	ISX
5	71965699	72065699	0.345498	ISX
5	71965854	72065854	0.344592	ISX
5	71966044	72066044	0.344712	ISX
5	71966069	72066069	0.345328	ISX
5	71966075	72066075	0.343185	ISX
5	71966155	72066155	0.343003	ISX
5	71966168	72066168	0.348704	ISX
5	71966295	72066295	0.348704	ISX
5	71967923	72067923	0.353312	ISX
5	71968020	72068020	0.345558	ISX
5	71968059	72068059	0.345558	ISX
5	71968099	72068099	0.345558	ISX
5	71968156	72068156	0.343701	ISX
5	71968428	72068428	0.343886	ISX
5	71968438	72068438	0.343886	ISX
5	71968442	72068442	0.351011	ISX
5	71968469	72068469	0.349087	ISX
5	71968509	72068509	0.36283	ISX
5	71968613	72068613	0.362181	ISX
5	71968621	72068621	0.36269	ISX
5	71968728	72068728	0.361323	ISX
5	71968909	72068909	0.361527	ISX
5	71968958	72068958	0.361464	ISX
5	71968987	72068987	0.361464	ISX
5	71969002	72069002	0.357719	ISX
5	71969135	72069135	0.358708	ISX
5	71969230	72069230	0.355081	ISX
5	71969285	72069285	0.356243	ISX
5	71969434	72069434	0.361025	ISX
5	71969504	72069504	0.359702	ISX
5	71969528	72069528	0.364315	ISX
5	71969610	72069610	0.363011	ISX
5	71969954	72069954	0.363011	ISX
5	71970010	72070010	0.361378	ISX

5	71970039	72070039	0.371777	ISX
5	71970188	72070188	0.372929	ISX
5	71970246	72070246	0.367434	ISX
5	71970446	72070446	0.367434	ISX
5	71970585	72070585	0.384547	ISX
5	71970675	72070675	0.368746	ISX
5	71970676	72070676	0.369988	ISX
5	71970790	72070790	0.370496	ISX
5	71970842	72070842	0.383541	ISX
5	71970867	72070867	0.377508	ISX
5	71970868	72070868	0.377508	ISX
5	71970975	72070975	0.366709	ISX
5	71971016	72071016	0.364923	ISX
5	71971030	72071030	0.364961	ISX
5	71971045	72071045	0.364882	ISX
5	71971062	72071062	0.35821	ISX
5	71971110	72071110	0.352246	ISX
5	71971154	72071154	0.352246	ISX
5	71971310	72071310	0.350315	ISX
5	71971373	72071373	0.350315	ISX
5	71971553	72071553	0.347679	ISX
5	71971642	72071642	0.362486	ISX
5	71971672	72071672	0.363419	ISX
5	71971765	72071765	0.362732	ISX
5	71971857	72071857	0.365582	ISX
5	71971988	72071988	0.353452	ISX
5	71972094	72072094	0.349719	ISX
5	71972672	72072672	0.344548	ISX
5	71972769	72072769	0.353842	ISX
5	71972859	72072859	0.35755	ISX
5	71972916	72072916	0.365081	ISX
5	71972919	72072919	0.366023	ISX
5	71972989	72072989	0.35867	ISX
5	72019966	72119966	0.34612	ISX
5	72020010	72120010	0.350244	ISX
5	72020045	72120045	0.350244	ISX
5	72020091	72120091	0.350093	ISX
5	72020555	72120555	0.369482	ISX
5	72020631	72120631	0.372442	ISX
5	72020992	72120992	0.37037	ISX
5	72021065	72121065	0.364579	ISX
5	72021404	72121404	0.369158	ISX
5	72021479	72121479	0.374857	ISX
5	72021672	72121672	0.355595	ISX
5	72021696	72121696	0.355595	ISX
5	72021707	72121707	0.356968	ISX
5	72021791	72121791	0.358556	ISX
5	72021908	72121908	0.377941	ISX
5	72022190	72122190	0.352653	ISX
5	72022368	72122368	0.35569	ISX
5	72022514	72122514	0.343067	ISX
5	72023091	72123091	0.393923	ISX
5	72023199	72123199	0.385841	ISX
5	72023207	72123207	0.387928	ISX
5	72023223	72123223	0.385712	ISX
5	72023292	72123292	0.388212	ISX
5	72023425	72123425	0.405654	ISX
5	72587596	72687596	0.355485	RBFOX2

5	72595150	72695150	0.355015	RBFOX2
5	72597216	72697216	0.342158	RBFOX2
5	72598765	72698765	0.344645	RBFOX2
5	72598831	72698831	0.363182	RBFOX2
5	72599031	72699031	0.351854	RBFOX2
5	72599291	72699291	0.347022	RBFOX2
5	72599988	72699988	0.352501	RBFOX2
5	72600474	72700474	0.379952	RBFOX2
5	72600603	72700603	0.379952	RBFOX2
5	72600664	72700664	0.379797	RBFOX2
5	72600887	72700887	0.379797	RBFOX2
5	72600945	72700945	0.379797	RBFOX2
5	72601019	72701019	0.382253	RBFOX2
5	72601234	72701234	0.377514	RBFOX2
5	72601363	72701363	0.377514	RBFOX2
5	72601471	72701471	0.354756	RBFOX2
5	72601591	72701591	0.354756	RBFOX2
5	72601645	72701645	0.354756	RBFOX2
5	72601786	72701786	0.354756	RBFOX2
5	72602258	72702258	0.354756	RBFOX2
5	72602296	72702296	0.354756	RBFOX2
5	72602313	72702313	0.354756	RBFOX2
5	72602322	72702322	0.354756	RBFOX2
5	72602347	72702347	0.354756	RBFOX2
5	72602381	72702381	0.353049	RBFOX2
5	72602400	72702400	0.353049	RBFOX2
5	72602426	72702426	0.354245	RBFOX2
5	72602470	72702470	0.352189	RBFOX2
5	72602606	72702606	0.352813	RBFOX2
5	72602673	72702673	0.3531	RBFOX2
5	72602694	72702694	0.3531	RBFOX2
5	72602961	72702961	0.3531	RBFOX2
5	72602963	72702963	0.3531	RBFOX2
5	72603174	72703174	0.3531	RBFOX2
5	72603177	72703177	0.3531	RBFOX2
5	72603285	72703285	0.3531	RBFOX2
5	72603399	72703399	0.3531	RBFOX2
5	72603460	72703460	0.3531	RBFOX2
5	72603534	72703534	0.3531	RBFOX2
5	72603617	72703617	0.3531	RBFOX2
5	72603630	72703630	0.3531	RBFOX2
5	72603770	72703770	0.3531	RBFOX2
5	72603884	72703884	0.353829	RBFOX2
5	72604097	72704097	0.352226	RBFOX2
5	72604817	72704817	0.355515	RBFOX2
5	72604942	72704942	0.360123	RBFOX2
5	72605007	72705007	0.358495	RBFOX2
5	72605622	72705622	0.361311	RBFOX2
5	72605632	72705632	0.361311	RBFOX2
5	72605758	72705758	0.360019	RBFOX2
5	72605968	72705968	0.359319	RBFOX2
5	72606300	72706300	0.360746	RBFOX2
5	72606322	72706322	0.355842	RBFOX2
5	72606415	72706415	0.352897	RBFOX2
5	72606707	72706707	0.348153	RBFOX2
5	72606827	72706827	0.348153	RBFOX2
5	72606835	72706835	0.348153	RBFOX2
5	72606878	72706878	0.348153	RBFOX2

5	72606932	72706932	0.348153	RBFOX2
5	72607328	72707328	0.366916	RBFOX2
5	72607486	72707486	0.362613	RBFOX2
5	72607640	72707640	0.362613	RBFOX2
5	72608071	72708071	0.362613	RBFOX2
5	72608384	72708384	0.366107	RBFOX2
5	72608461	72708461	0.359391	RBFOX2
5	72608631	72708631	0.359391	RBFOX2
5	72608897	72708897	0.359391	RBFOX2
5	72609189	72709189	0.359391	RBFOX2
5	72609258	72709258	0.359391	RBFOX2
5	72609329	72709329	0.359391	RBFOX2
5	72609355	72709355	0.359391	RBFOX2
5	72611496	72711496	0.354042	RBFOX2
5	72613260	72713260	0.349343	RBFOX2
5	72613262	72713262	0.349343	RBFOX2
5	72613268	72713268	0.349343	RBFOX2
5	72613270	72713270	0.349343	RBFOX2
5	72613328	72713328	0.341715	RBFOX2
5	72613416	72713416	0.345879	RBFOX2
5	72613605	72713605	0.344524	RBFOX2
5	72693162	72793162	0.345511	RBFOX2
5	77443258	77543258	0.44138	IPO8
5	77443265	77543265	0.539253	IPO8
5	77443270	77543270	0.532699	IPO8
5	78459998	78559998	0.364679	TMTC1
5	78460008	78560008	0.361845	TMTC1
5	78460062	78560062	0.391799	TMTC1
5	78486879	78586879	0.346126	TMTC1
5	78486957	78586957	0.348878	TMTC1
5	78486983	78586983	0.372851	TMTC1
5	80344013	80444013	0.352197	PTHLH
5	80344124	80444124	0.354957	PTHLH
5	80344204	80444204	0.359764	PTHLH
5	80344416	80444416	0.356936	PTHLH
5	80344438	80444438	0.356936	PTHLH
5	80344705	80444705	0.356283	PTHLH
5	80344730	80444730	0.359136	PTHLH
5	80344785	80444785	0.359136	PTHLH
5	80344961	80444961	0.36592	PTHLH
5	80344975	80444975	0.361152	PTHLH
5	80345075	80445075	0.359403	PTHLH
5	80345208	80445208	0.360179	PTHLH
5	80345235	80445235	0.356409	PTHLH
5	80345394	80445394	0.356364	PTHLH
5	80345483	80445483	0.356511	PTHLH
5	80345495	80445495	0.359349	PTHLH
5	80345757	80445757	0.358365	PTHLH
5	80345775	80445775	0.359227	PTHLH
5	80345849	80445849	0.356627	PTHLH
5	80345980	80445980	0.356283	PTHLH
5	80346098	80446098	0.360707	PTHLH
5	80346115	80446115	0.361072	PTHLH
5	80346248	80446248	0.36028	PTHLH
5	80346330	80446330	0.359815	PTHLH
5	80346435	80446435	0.390296	PTHLH
5	80346509	80446509	0.380105	PTHLH
5	80346551	80446551	0.386051	PTHLH

5	80346623	80446623	0.386057	PTHLH
5	80346651	80446651	0.383137	PTHLH
5	80346700	80446700	0.385843	PTHLH
5	80346966	80446966	0.376589	PTHLH
5	80347027	80447027	0.374051	PTHLH
5	80347206	80447206	0.367326	PTHLH
5	80347252	80447252	0.372031	PTHLH
5	80347263	80447263	0.358326	PTHLH
5	80347280	80447280	0.358326	PTHLH
5	80347391	80447391	0.348122	PTHLH
5	80347459	80447459	0.353986	PTHLH
5	80347612	80447612	0.354168	PTHLH
5	80347940	80447940	0.353971	PTHLH
5	80347990	80447990	0.373994	PTHLH
5	80348333	80448333	0.354534	PTHLH
5	80348426	80448426	0.352311	PTHLH
5	80348579	80448579	0.352311	PTHLH
5	80348936	80448936	0.369962	PTHLH
5	80348937	80448937	0.353899	PTHLH
5	80349288	80449288	0.353899	PTHLH
5	80349478	80449478	0.353899	PTHLH
5	80349540	80449540	0.364466	PTHLH
5	80349997	80449997	0.36121	PTHLH
5	80350004	80450004	0.36121	PTHLH
5	80350409	80450409	0.36121	PTHLH
5	80350433	80450433	0.364963	PTHLH
5	80350482	80450482	0.361561	PTHLH
5	80350647	80450647	0.361561	PTHLH
5	80350676	80450676	0.361561	PTHLH
5	80350722	80450722	0.377279	PTHLH
5	80350840	80450840	0.371024	PTHLH
5	80350902	80450902	0.375164	PTHLH
5	80350919	80450919	0.371721	PTHLH
5	80351562	80451562	0.376742	PTHLH
5	80351583	80451583	0.372944	PTHLH
5	80351709	80451709	0.379687	PTHLH
5	80351921	80451921	0.367852	PTHLH
5	80351938	80451938	0.370573	PTHLH
5	80352065	80452065	0.369959	PTHLH
5	80352076	80452076	0.37038	PTHLH
5	80352080	80452080	0.370187	PTHLH
5	80352239	80452239	0.370708	PTHLH
5	80352245	80452245	0.361196	PTHLH
5	80352694	80452694	0.361196	PTHLH
5	80352715	80452715	0.361196	PTHLH
5	80352870	80452870	0.369239	PTHLH
5	80353030	80453030	0.362363	PTHLH
5	80353483	80453483	0.372067	PTHLH
5	80353634	80453634	0.363582	PTHLH
5	80353906	80453906	0.363216	PTHLH
5	80354175	80454175	0.364093	PTHLH
5	80354278	80454278	0.377402	PTHLH
5	80354339	80454339	0.374986	PTHLH
5	80354625	80454625	0.374986	PTHLH
5	80694411	80794411	0.347069	REP15
5	80694411	80794411	0.347069	LOC102177091
5	80694411	80794411	0.347069	MRPS35
5	80694411	80794411	0.347069	PPFIBP1

5	80694592	80794592	0.34852	REP15
5	80694592	80794592	0.34852	LOC102177091
5	80694592	80794592	0.34852	MRPS35
5	80694592	80794592	0.34852	PPFIBP1
5	80695369	80795369	0.35392	REP15
5	80695369	80795369	0.35392	LOC102177091
5	80695369	80795369	0.35392	MRPS35
5	80695369	80795369	0.35392	PPFIBP1
5	80695410	80795410	0.357602	REP15
5	80695410	80795410	0.357602	LOC102177091
5	80695410	80795410	0.357602	MRPS35
5	80695410	80795410	0.357602	PPFIBP1
5	81636766	81736766	0.36211	ITPR2
5	81636815	81736815	0.351631	ITPR2
5	81637011	81737011	0.345828	ITPR2
5	81637310	81737310	0.346354	ITPR2
5	81638205	81738205	0.358768	ITPR2
5	81638316	81738316	0.344685	ITPR2
5	81638358	81738358	0.344685	ITPR2
5	81638385	81738385	0.354706	ITPR2
5	81638461	81738461	0.36035	ITPR2
5	81638553	81738553	0.365124	ITPR2
5	81639113	81739113	0.363209	ITPR2
5	81639244	81739244	0.360817	ITPR2
5	81639306	81739306	0.369456	ITPR2
5	81639319	81739319	0.379302	ITPR2
5	81639360	81739360	0.373359	ITPR2
5	81639457	81739457	0.376104	ITPR2
5	81639513	81739513	0.378747	ITPR2
5	81639545	81739545	0.370121	ITPR2
5	81639572	81739572	0.367672	ITPR2
5	81639614	81739614	0.349339	ITPR2
5	81640050	81740050	0.35615	ITPR2
5	81640154	81740154	0.361168	ITPR2
5	81640462	81740462	0.355573	ITPR2
5	81640868	81740868	0.367464	ITPR2
5	81640980	81740980	0.371736	ITPR2
5	81641065	81741065	0.369064	ITPR2
5	81641182	81741182	0.374563	ITPR2
5	81641244	81741244	0.376673	ITPR2
5	81641357	81741357	0.385361	ITPR2
5	81641577	81741577	0.38481	ITPR2
5	81641650	81741650	0.38045	ITPR2
5	81641727	81741727	0.380794	ITPR2
5	81641731	81741731	0.372978	ITPR2
5	81642110	81742110	0.368112	ITPR2
5	81642217	81742217	0.367627	ITPR2
5	81642336	81742336	0.366219	ITPR2
5	81642416	81742416	0.372784	ITPR2
5	81642428	81742428	0.372784	ITPR2
5	81642449	81742449	0.354733	ITPR2
5	81642471	81742471	0.381978	ITPR2
5	81642492	81742492	0.371712	ITPR2
5	81642498	81742498	0.390114	ITPR2
5	81642708	81742708	0.374598	ITPR2
5	81643197	81743197	0.392404	ITPR2
5	81643203	81743203	0.385066	ITPR2
5	81643369	81743369	0.392917	ITPR2

5	81643473	81743473	0.396261	ITPR2
5	81643905	81743905	0.39917	ITPR2
5	81643926	81743926	0.386905	ITPR2
5	81643985	81743985	0.388331	ITPR2
5	81644045	81744045	0.41116	ITPR2
5	81644077	81744077	0.388229	ITPR2
5	81644082	81744082	0.389297	ITPR2
5	81644133	81744133	0.372514	ITPR2
5	81644250	81744250	0.372642	ITPR2
5	81644294	81744294	0.363297	ITPR2
5	81644309	81744309	0.347801	ITPR2
5	81644465	81744465	0.342925	ITPR2
5	81644556	81744556	0.347648	ITPR2
5	81644638	81744638	0.343972	ITPR2
5	81644690	81744690	0.342021	ITPR2
5	81644721	81744721	0.342429	ITPR2
5	81644724	81744724	0.345118	ITPR2
5	81644746	81744746	0.345536	ITPR2
5	81644780	81744780	0.362548	ITPR2
5	81644795	81744795	0.359694	ITPR2
5	81644857	81744857	0.359586	ITPR2
5	81645889	81745889	0.345089	ITPR2
5	81657137	81757137	0.361743	ITPR2
5	81657215	81757215	0.372151	ITPR2
5	81657398	81757398	0.383557	ITPR2
5	81657445	81757445	0.382842	ITPR2
5	81657578	81757578	0.389463	ITPR2
5	81657592	81757592	0.404189	ITPR2
5	81657613	81757613	0.40374	ITPR2
5	81657725	81757725	0.436101	ITPR2
5	81657764	81757764	0.431863	ITPR2
5	81657984	81757984	0.408606	ITPR2
5	81658170	81758170	0.442835	ITPR2
5	81658196	81758196	0.458231	ITPR2
5	81658261	81758261	0.438872	ITPR2
5	81658285	81758285	0.434594	ITPR2
5	81659129	81759129	0.467895	ITPR2
5	81659159	81759159	0.44461	ITPR2
5	81659244	81759244	0.439027	ITPR2
5	81659376	81759376	0.473797	ITPR2
5	81659497	81759497	0.494695	ITPR2
5	81659685	81759685	0.439644	ITPR2
5	81659717	81759717	0.417416	ITPR2
5	81659727	81759727	0.391625	ITPR2
5	81758754	81858754	0.360582	ITPR2
5	81759884	81859884	0.344393	ITPR2
5	81764599	81864599	0.34923	ITPR2
5	81765626	81865626	0.379559	ITPR2
5	81765761	81865761	0.349355	ITPR2
5	81766184	81866184	0.414819	ITPR2
5	81766472	81866472	0.449832	ITPR2
5	81766804	81866804	0.470488	ITPR2
5	81766863	81866863	0.506581	ITPR2
5	81766864	81866864	0.494989	ITPR2
5	81766917	81866917	0.494989	ITPR2
5	81766954	81866954	0.483496	ITPR2
5	81766958	81866958	0.495278	ITPR2
5	81766977	81866977	0.4944	ITPR2

5	81767033	81867033	0.440354	ITPR2
5	81767228	81867228	0.412372	ITPR2
5	81767330	81867330	0.425193	ITPR2
5	81767469	81867469	0.426923	ITPR2
5	81767502	81867502	0.373951	ITPR2
5	81767684	81867684	0.367336	ITPR2
5	81767687	81867687	0.364607	ITPR2
5	81767718	81867718	0.354644	ITPR2
5	81767730	81867730	0.35774	ITPR2
5	81767810	81867810	0.368125	ITPR2
5	81767915	81867915	0.368125	ITPR2
5	81767940	81867940	0.363076	ITPR2
5	81767951	81867951	0.358682	ITPR2
5	81768048	81868048	0.357534	ITPR2
5	81768069	81868069	0.35361	ITPR2
5	81768249	81868249	0.352057	ITPR2
5	81768256	81868256	0.352057	ITPR2
5	81769023	81869023	0.347358	ITPR2
5	81770968	81870968	0.346349	ITPR2
5	81771065	81871065	0.346794	ITPR2
5	81771103	81871103	0.348694	ITPR2
5	81771150	81871150	0.357694	ITPR2
5	81771202	81871202	0.362392	ITPR2
5	81771260	81871260	0.363279	ITPR2
5	81771277	81871277	0.363279	ITPR2
5	81771312	81871312	0.363279	ITPR2
5	81771346	81871346	0.366188	ITPR2
5	81771369	81871369	0.348875	ITPR2
5	81771497	81871497	0.34892	ITPR2
5	81771665	81871665	0.344218	ITPR2
5	81772221	81872221	0.342799	ITPR2
5	81777268	81877268	0.351063	ITPR2
5	81781178	81881178	0.353621	ITPR2
5	81781303	81881303	0.426317	ITPR2
5	81781347	81881347	0.45336	ITPR2
5	81781418	81881418	0.425244	ITPR2
5	81782226	81882226	0.412902	ITPR2
5	81782304	81882304	0.412902	ITPR2
5	81782697	81882697	0.412902	ITPR2
5	81782700	81882700	0.451363	ITPR2
5	81782782	81882782	0.363385	ITPR2
5	81782960	81882960	0.350214	ITPR2
5	81783027	81883027	0.356556	ITPR2
5	81783518	81883518	0.358037	ITPR2
5	81783546	81883546	0.357904	ITPR2
5	81783566	81883566	0.357904	ITPR2
5	81787133	81887133	0.343057	ITPR2
5	81787736	81887736	0.343057	ITPR2
5	81788045	81888045	0.342099	ITPR2
5	81788932	81888932	0.34567	ITPR2
5	81789081	81889081	0.354728	ITPR2
5	81789159	81889159	0.372753	ITPR2
5	81789176	81889176	0.361591	ITPR2
5	81789292	81889292	0.358777	ITPR2
5	81789459	81889459	0.357921	ITPR2
5	81798619	81898619	0.353056	ITPR2
5	81798651	81898651	0.356805	ITPR2
5	81798672	81898672	0.39286	ITPR2

5	81798714	81898714	0.38669	ITPR2
5	81798766	81898766	0.387751	ITPR2
5	81798793	81898793	0.389339	ITPR2
5	81798910	81898910	0.377379	ITPR2
5	81798953	81898953	0.378986	ITPR2
5	81799006	81899006	0.379144	ITPR2
5	81799120	81899120	0.379714	ITPR2
5	81799128	81899128	0.379993	ITPR2
5	81799150	81899150	0.379993	ITPR2
5	81799173	81899173	0.379993	ITPR2
5	81799364	81899364	0.375371	ITPR2
5	81799470	81899470	0.3947	ITPR2
5	81799527	81899527	0.380229	ITPR2
5	81799572	81899572	0.380088	ITPR2
5	81799617	81899617	0.394789	ITPR2
5	81799675	81899675	0.390214	ITPR2
5	81799698	81899698	0.400028	ITPR2
5	81799722	81899722	0.39867	ITPR2
5	81799809	81899809	0.395728	ITPR2
5	81799851	81899851	0.390537	ITPR2
5	81799869	81899869	0.369798	ITPR2
5	81799915	81899915	0.369819	ITPR2
5	81799916	81899916	0.369819	ITPR2
5	81799932	81899932	0.367129	ITPR2
5	81799953	81899953	0.386914	ITPR2
5	81799960	81899960	0.357545	ITPR2
5	81799980	81899980	0.352913	ITPR2
5	81802705	81902705	0.349367	ITPR2
5	81802792	81902792	0.359439	ITPR2
5	81802940	81902940	0.36514	ITPR2
5	81804542	81904542	0.356154	ITPR2
5	81807728	81907728	0.401917	ITPR2
5	81807766	81907766	0.410641	ITPR2
5	81807805	81907805	0.408352	ITPR2
5	81807907	81907907	0.40899	ITPR2
5	81808034	81908034	0.409603	ITPR2
5	81808654	81908654	0.407746	ITPR2
5	81809090	81909090	0.414674	ITPR2
5	81809332	81909332	0.413009	ITPR2
5	81809333	81909333	0.413492	ITPR2
5	81809556	81909556	0.412456	ITPR2
5	81809754	81909754	0.413396	ITPR2
5	81809846	81909846	0.415631	ITPR2
5	81809873	81909873	0.408829	ITPR2
5	81809944	81909944	0.413781	ITPR2
5	81810112	81910112	0.417257	ITPR2
5	81810441	81910441	0.420124	ITPR2
5	81810445	81910445	0.420124	ITPR2
5	81810523	81910523	0.420124	ITPR2
5	81810529	81910529	0.418733	ITPR2
5	81810550	81910550	0.42098	ITPR2
5	81810609	81910609	0.420745	ITPR2
5	81810634	81910634	0.43178	ITPR2
5	81810680	81910680	0.421589	ITPR2
5	81810815	81910815	0.427405	ITPR2
5	81810875	81910875	0.42802	ITPR2
5	81810983	81910983	0.417046	ITPR2
5	81811039	81911039	0.417708	ITPR2

5	81811069	81911069	0.413159	ITPR2
5	81811105	81911105	0.413502	ITPR2
5	81811137	81911137	0.412864	ITPR2
5	81811327	81911327	0.412864	ITPR2
5	81811392	81911392	0.412864	ITPR2
5	81811602	81911602	0.415028	ITPR2
5	81811617	81911617	0.415028	ITPR2
5	81811677	81911677	0.415028	ITPR2
5	81811814	81911814	0.429076	ITPR2
5	81811913	81911913	0.417442	ITPR2
5	81811946	81911946	0.424927	ITPR2
5	81811999	81911999	0.421232	ITPR2
5	81812041	81912041	0.41859	ITPR2
5	81812071	81912071	0.418894	ITPR2
5	81812074	81912074	0.420181	ITPR2
5	81812179	81912179	0.416066	ITPR2
5	81812389	81912389	0.417747	ITPR2
5	81812889	81912889	0.426085	ITPR2
5	81812902	81912902	0.425702	ITPR2
5	81812944	81912944	0.430006	ITPR2
5	81812954	81912954	0.427778	ITPR2
5	81813001	81913001	0.443492	ITPR2
5	81813036	81913036	0.432855	ITPR2
5	81813037	81913037	0.432855	ITPR2
5	81813085	81913085	0.434858	ITPR2
5	81813175	81913175	0.434858	ITPR2
5	81813412	81913412	0.435703	ITPR2
5	81813436	81913436	0.435703	ITPR2
5	81813499	81913499	0.435157	ITPR2
5	81813565	81913565	0.435551	ITPR2
5	81813582	81913582	0.435551	ITPR2
5	81813774	81913774	0.437298	ITPR2
5	81813884	81913884	0.433964	ITPR2
5	81813931	81913931	0.432221	ITPR2
5	81814084	81914084	0.431973	ITPR2
5	81814112	81914112	0.431973	ITPR2
5	81814452	81914452	0.430422	ITPR2
5	81814612	81914612	0.430422	ITPR2
5	81814616	81914616	0.430422	ITPR2
5	81814674	81914674	0.434671	ITPR2
5	81814755	81914755	0.440054	ITPR2
5	81814756	81914756	0.430507	ITPR2
5	81814916	81914916	0.412655	ITPR2
5	81815152	81915152	0.416543	ITPR2
5	81815175	81915175	0.427689	ITPR2
5	81815187	81915187	0.427689	ITPR2
5	81815191	81915191	0.427689	ITPR2
5	81815218	81915218	0.424897	ITPR2
5	81815231	81915231	0.424897	ITPR2
5	81815259	81915259	0.427739	ITPR2
5	81815292	81915292	0.42696	ITPR2
5	81815327	81915327	0.429344	ITPR2
5	81815378	81915378	0.416972	ITPR2
5	81815499	81915499	0.419968	ITPR2
5	81815511	81915511	0.411303	ITPR2
5	81815686	81915686	0.411023	ITPR2
5	81815848	81915848	0.411353	ITPR2
5	81816081	81916081	0.456264	ITPR2

5	81816091	81916091	0.435856	ITPR2
5	81816186	81916186	0.455118	ITPR2
5	81816438	81916438	0.426098	ITPR2
5	81816779	81916779	0.428994	ITPR2
5	81816815	81916815	0.431199	ITPR2
5	81878412	81978412	0.346011	ITPR2
5	81878418	81978418	0.346011	ITPR2
5	81878457	81978457	0.350123	ITPR2
5	81878474	81978474	0.351494	ITPR2
5	81879335	81979335	0.341984	ITPR2
5	81879529	81979529	0.384478	ITPR2
5	81879574	81979574	0.394243	ITPR2
5	81879825	81979825	0.382213	ITPR2
5	81879903	81979903	0.375312	ITPR2
5	81880054	81980054	0.383092	ITPR2
5	81880098	81980098	0.379631	ITPR2
5	81880193	81980193	0.372036	ITPR2
5	81880195	81980195	0.372036	ITPR2
5	81880209	81980209	0.377703	ITPR2
5	81880289	81980289	0.395847	ITPR2
5	81880344	81980344	0.416658	ITPR2
5	81880551	81980551	0.394195	ITPR2
5	81880641	81980641	0.37061	ITPR2
5	81880720	81980720	0.371452	ITPR2
5	81880817	81980817	0.360158	ITPR2
5	81881291	81981291	0.35177	ITPR2
5	81881399	81981399	0.348244	ITPR2
5	81881927	81981927	0.349251	ITPR2
5	81882024	81982024	0.363386	ITPR2
5	81882098	81982098	0.348316	ITPR2
5	81882269	81982269	0.368689	ITPR2
5	81896980	81996980	0.418854	ITPR2
5	81897241	81997241	0.454345	ITPR2
5	81897276	81997276	0.473763	ITPR2
5	81897289	81997289	0.472708	ITPR2
5	81897294	81997294	0.471865	ITPR2
5	81897407	81997407	0.462704	ITPR2
5	81897409	81997409	0.462704	ITPR2
5	81897411	81997411	0.462704	ITPR2
5	81897475	81997475	0.453821	ITPR2
5	81897629	81997629	0.432273	ITPR2
5	81898507	81998507	0.399406	ITPR2
5	81898525	81998525	0.390661	ITPR2
5	81898756	81998756	0.396691	ITPR2
5	81898869	81998869	0.445463	ITPR2
5	81899300	81999300	0.408582	ITPR2
5	81899442	81999442	0.415778	ITPR2
5	81899699	81999699	0.40368	ITPR2
5	81899763	81999763	0.424751	ITPR2
5	81900167	82000167	0.436226	ITPR2
5	81900208	82000208	0.398231	ITPR2
5	81900336	82000336	0.396945	ITPR2
5	81900820	82000820	0.388324	ITPR2
5	81900830	82000830	0.406983	ITPR2
5	81900854	82000854	0.41474	ITPR2
5	81900971	82000971	0.41474	ITPR2
5	81900977	82000977	0.403883	ITPR2
5	81901188	82001188	0.403883	ITPR2

5	81901274	82001274	0.443669	ITPR2
5	81901403	82001403	0.44203	ITPR2
5	81901419	82001419	0.430693	ITPR2
5	81901484	82001484	0.447151	ITPR2
5	81901494	82001494	0.411052	ITPR2
5	81901574	82001574	0.424457	ITPR2
5	81901580	82001580	0.365293	ITPR2
5	81901594	82001594	0.350009	ITPR2
5	81902175	82002175	0.365628	ITPR2
5	81902246	82002246	0.365477	ITPR2
5	81902851	82002851	0.381438	ITPR2
5	81903036	82003036	0.448219	ITPR2
5	81903600	82003600	0.439912	ITPR2
5	81903885	82003885	0.450646	ITPR2
5	81903891	82003891	0.486941	ITPR2
5	81904416	82004416	0.472576	ITPR2
5	81904469	82004469	0.472165	ITPR2
5	81904742	82004742	0.482631	ITPR2
5	81905031	82005031	0.500135	ITPR2
5	81905042	82005042	0.472977	ITPR2
5	81905060	82005060	0.486584	ITPR2
5	81905118	82005118	0.447939	ITPR2
5	81905223	82005223	0.458791	ITPR2
5	81905226	82005226	0.458791	ITPR2
5	81905348	82005348	0.470859	ITPR2
5	81905831	82005831	0.492504	ITPR2
5	81905909	82005909	0.491551	ITPR2
5	81906126	82006126	0.489995	ITPR2
5	81906127	82006127	0.471273	ITPR2
5	81906183	82006183	0.446281	ITPR2
5	81906356	82006356	0.471548	ITPR2
5	81906394	82006394	0.48737	ITPR2
5	81906478	82006478	0.48737	ITPR2
5	81906955	82006955	0.425983	ITPR2
5	81907501	82007501	0.428808	ITPR2
5	81907585	82007585	0.448173	ITPR2
5	81907732	82007732	0.394369	ITPR2
5	81907913	82007913	0.376568	ITPR2
5	81908917	82008917	0.407885	ITPR2
5	81909167	82009167	0.404106	ITPR2
5	81909436	82009436	0.379068	ITPR2
5	81909618	82009618	0.406872	ITPR2
5	81909710	82009710	0.435141	ITPR2
5	81909752	82009752	0.429485	ITPR2
5	81909785	82009785	0.429485	ITPR2
5	81909845	82009845	0.411479	ITPR2
5	81910095	82010095	0.345628	ITPR2
5	81911232	82011232	0.350291	ITPR2
5	81911327	82011327	0.342158	ITPR2
5	81911345	82011345	0.351863	ITPR2
5	81911970	82011970	0.388016	ITPR2
5	81912679	82012679	0.412795	ITPR2
5	81913605	82013605	0.406341	ITPR2
5	81913676	82013676	0.431188	ITPR2
5	81913873	82013873	0.41173	ITPR2
5	81913875	82013875	0.394035	ITPR2
5	81914040	82014040	0.406644	ITPR2
5	81914207	82014207	0.427241	ITPR2

5	81914646	82014646	0.366174	ITPR2
5	81914938	82014938	0.346413	ITPR2
5	81915323	82015323	0.353114	ITPR2
5	81915654	82015654	0.367485	ITPR2
5	81915811	82015811	0.367485	ITPR2
5	81917652	82017652	0.342948	ITPR2
5	81917712	82017712	0.363077	ITPR2
5	81917944	82017944	0.364363	ITPR2
5	81918254	82018254	0.358171	ITPR2
5	81918400	82018400	0.362279	ITPR2
5	81918481	82018481	0.380505	ITPR2
5	81919243	82019243	0.365103	ITPR2
5	81919346	82019346	0.387116	ITPR2
5	81919380	82019380	0.374953	ITPR2
5	81919560	82019560	0.359076	ITPR2
5	81920205	82020205	0.368255	ITPR2
5	81921403	82021403	0.378659	ITPR2
5	81922429	82022429	0.346133	ITPR2
5	81928277	82028277	0.346621	ITPR2
5	81928279	82028279	0.348653	ITPR2
5	81928298	82028298	0.369845	ITPR2
5	81928313	82028313	0.362343	ITPR2
5	81928321	82028321	0.362643	ITPR2
5	81928329	82028329	0.362643	ITPR2
5	81928342	82028342	0.372642	ITPR2
5	81928437	82028437	0.395032	ITPR2
5	81928675	82028675	0.458951	ITPR2
5	81928791	82028791	0.399887	ITPR2
5	81929806	82029806	0.414116	ITPR2
5	81930030	82030030	0.444175	ITPR2
5	81930070	82030070	0.430867	ITPR2
5	81930126	82030126	0.463832	ITPR2
5	81930321	82030321	0.455916	ITPR2
5	81930666	82030666	0.466326	ITPR2
5	81930691	82030691	0.484491	ITPR2
5	81930809	82030809	0.473655	ITPR2
5	81930850	82030850	0.473655	ITPR2
5	81930936	82030936	0.473655	ITPR2
5	81931096	82031096	0.477639	ITPR2
5	81931178	82031178	0.458184	ITPR2
5	81931268	82031268	0.458184	ITPR2
5	81931356	82031356	0.458184	ITPR2
5	81931878	82031878	0.474438	ITPR2
5	81931922	82031922	0.443252	ITPR2
5	81931945	82031945	0.443252	ITPR2
5	81932098	82032098	0.443252	ITPR2
5	81932459	82032459	0.451066	ITPR2
5	81932578	82032578	0.430753	ITPR2
5	81932742	82032742	0.440677	ITPR2
5	81932807	82032807	0.441918	ITPR2
5	81933001	82033001	0.435831	ITPR2
5	81933041	82033041	0.442314	ITPR2
5	81933164	82033164	0.445209	ITPR2
5	81933217	82033217	0.445553	ITPR2
5	81933244	82033244	0.440328	ITPR2
5	81933355	82033355	0.443537	ITPR2
5	81933377	82033377	0.477689	ITPR2
5	81933388	82033388	0.477689	ITPR2

5	81933389	82033389	0.478189	ITPR2
5	81933439	82033439	0.473069	ITPR2
5	81933714	82033714	0.469929	ITPR2
5	81933898	82033898	0.474412	ITPR2
5	81933905	82033905	0.476806	ITPR2
5	81934033	82034033	0.472789	ITPR2
5	81934183	82034183	0.47234	ITPR2
5	81934217	82034217	0.480875	ITPR2
5	81934611	82034611	0.429864	ITPR2
5	81934804	82034804	0.453837	ITPR2
5	81934895	82034895	0.449763	ITPR2
5	81934956	82034956	0.478322	ITPR2
5	81935004	82035004	0.465303	ITPR2
5	81935088	82035088	0.464608	ITPR2
5	81935095	82035095	0.464608	ITPR2
5	81935196	82035196	0.464608	ITPR2
5	81935271	82035271	0.484309	ITPR2
5	81936107	82036107	0.473	ITPR2
5	81936138	82036138	0.473	ITPR2
5	81936151	82036151	0.473	ITPR2
5	81936237	82036237	0.454157	ITPR2
5	81936323	82036323	0.510807	ITPR2
5	81936346	82036346	0.507227	ITPR2
5	81936438	82036438	0.506823	ITPR2
5	81936663	82036663	0.515842	ITPR2
5	81936670	82036670	0.515842	ITPR2
5	81936678	82036678	0.515842	ITPR2
5	81936696	82036696	0.518922	ITPR2
5	81936737	82036737	0.516344	ITPR2
5	81937002	82037002	0.512871	ITPR2
5	81937039	82037039	0.511725	ITPR2
5	81937095	82037095	0.547226	ITPR2
5	81937364	82037364	0.593561	ITPR2
5	81937441	82037441	0.604935	ITPR2
5	81937531	82037531	0.582509	ITPR2
5	81937648	82037648	0.584303	ITPR2
5	81937689	82037689	0.584705	ITPR2
5	81937914	82037914	0.543909	ITPR2
5	81937929	82037929	0.599351	ITPR2
5	81937934	82037934	0.577565	ITPR2
5	81937988	82037988	0.66046	ITPR2
5	81938010	82038010	0.662051	ITPR2
5	81938049	82038049	0.648659	ITPR2
5	81938116	82038116	0.648659	ITPR2
5	81938140	82038140	0.63404	ITPR2
5	81938424	82038424	0.647161	ITPR2
5	81938457	82038457	0.647161	ITPR2
5	81938929	82038929	0.664668	ITPR2
5	81939103	82039103	0.664668	ITPR2
5	81939116	82039116	0.697334	ITPR2
5	81939120	82039120	0.706081	ITPR2
5	81939151	82039151	0.701022	ITPR2
5	81939159	82039159	0.701022	ITPR2
5	81939359	82039359	0.707719	ITPR2
5	81939445	82039445	0.702623	ITPR2
5	81939487	82039487	0.704033	ITPR2
5	81939509	82039509	0.704033	ITPR2
5	81939574	82039574	0.704033	ITPR2

5	81939634	82039634	0.707923	ITPR2
5	81939682	82039682	0.697103	ITPR2
5	81939683	82039683	0.701101	ITPR2
5	81939733	82039733	0.726792	ITPR2
5	81939746	82039746	0.690276	ITPR2
5	81939860	82039860	0.699101	ITPR2
5	81939950	82039950	0.701137	ITPR2
5	81940022	82040022	0.678188	ITPR2
5	81940026	82040026	0.678188	ITPR2
5	81940138	82040138	0.678188	ITPR2
5	81940201	82040201	0.694131	ITPR2
5	81940223	82040223	0.684467	ITPR2
5	81940280	82040280	0.680889	ITPR2
5	81940288	82040288	0.700467	ITPR2
5	81940735	82040735	0.685045	ITPR2
5	81940740	82040740	0.684766	ITPR2
5	81940777	82040777	0.680244	ITPR2
5	81940786	82040786	0.681212	ITPR2
5	81940884	82040884	0.687313	ITPR2
5	81941301	82041301	0.669657	ITPR2
5	81941348	82041348	0.677805	ITPR2
5	81941399	82041399	0.645621	ITPR2
5	81941431	82041431	0.619173	ITPR2
5	81941445	82041445	0.636315	ITPR2
5	81941512	82041512	0.63379	ITPR2
5	81941550	82041550	0.632628	ITPR2
5	81941567	82041567	0.622443	ITPR2
5	81941710	82041710	0.540093	ITPR2
5	81941723	82041723	0.562994	ITPR2
5	81941761	82041761	0.557746	ITPR2
5	81941848	82041848	0.517138	ITPR2
5	81941860	82041860	0.53349	ITPR2
5	81941991	82041991	0.531237	ITPR2
5	81941992	82041992	0.531237	ITPR2
5	81942086	82042086	0.531237	ITPR2
5	81942667	82042667	0.523012	ITPR2
5	81942879	82042879	0.530381	ITPR2
5	81942919	82042919	0.54157	ITPR2
5	81942958	82042958	0.539646	ITPR2
5	81942970	82042970	0.55433	ITPR2
5	81943064	82043064	0.547541	ITPR2
5	81943147	82043147	0.537892	ITPR2
5	81943199	82043199	0.535235	ITPR2
5	81943296	82043296	0.532542	ITPR2
5	81943795	82043795	0.503079	ITPR2
5	81943910	82043910	0.495083	ITPR2
5	81944094	82044094	0.49788	ITPR2
5	81944719	82044719	0.492543	ITPR2
5	81944808	82044808	0.49716	ITPR2
5	81944858	82044858	0.504182	ITPR2
5	81944882	82044882	0.501641	ITPR2
5	81944993	82044993	0.490267	ITPR2
5	81945026	82045026	0.480601	ITPR2
5	81945223	82045223	0.47853	ITPR2
5	81945396	82045396	0.466737	ITPR2
5	81946039	82046039	0.466777	ITPR2
5	81946058	82046058	0.466777	ITPR2
5	81946173	82046173	0.430209	ITPR2

5	81946527	82046527	0.474783	ITPR2
5	81946629	82046629	0.463282	ITPR2
5	81947085	82047085	0.463627	ITPR2
5	81947141	82047141	0.463627	ITPR2
5	81947165	82047165	0.463627	ITPR2
5	81947204	82047204	0.463627	ITPR2
5	81947220	82047220	0.460482	ITPR2
5	81947249	82047249	0.492011	ITPR2
5	81947666	82047666	0.488748	ITPR2
5	81947708	82047708	0.49092	ITPR2
5	81947803	82047803	0.483042	ITPR2
5	81947957	82047957	0.511452	ITPR2
5	81947994	82047994	0.47757	ITPR2
5	81948099	82048099	0.478926	ITPR2
5	81948274	82048274	0.476263	ITPR2
5	81948515	82048515	0.466179	ITPR2
5	81948974	82048974	0.462386	ITPR2
5	81949005	82049005	0.460255	ITPR2
5	81949021	82049021	0.457372	ITPR2
5	81949055	82049055	0.457372	ITPR2
5	81949350	82049350	0.457372	ITPR2
5	81949654	82049654	0.457372	ITPR2
5	81949701	82049701	0.457372	ITPR2
5	81950123	82050123	0.475106	ITPR2
5	81950154	82050154	0.484229	ITPR2
5	81950254	82050254	0.482538	ITPR2
5	81950457	82050457	0.482538	ITPR2
5	81950504	82050504	0.490344	ITPR2
5	81950523	82050523	0.490344	ITPR2
5	81950864	82050864	0.492568	ITPR2
5	81950905	82050905	0.499237	ITPR2
5	81950984	82050984	0.499237	ITPR2
5	81951160	82051160	0.499237	ITPR2
5	81951214	82051214	0.496933	ITPR2
5	81951238	82051238	0.496933	ITPR2
5	81951261	82051261	0.496933	ITPR2
5	81951282	82051282	0.509942	ITPR2
5	81951342	82051342	0.506984	ITPR2
5	81951533	82051533	0.509151	ITPR2
5	81951688	82051688	0.483719	ITPR2
5	81951724	82051724	0.48147	ITPR2
5	81951824	82051824	0.490444	ITPR2
5	81951903	82051903	0.474733	ITPR2
5	81952003	82052003	0.474733	ITPR2
5	81952189	82052189	0.478633	ITPR2
5	81952207	82052207	0.475332	ITPR2
5	81952210	82052210	0.475332	ITPR2
5	81952226	82052226	0.475332	ITPR2
5	81952347	82052347	0.474754	ITPR2
5	81952407	82052407	0.474754	ITPR2
5	81952491	82052491	0.474754	ITPR2
5	81952555	82052555	0.474754	ITPR2
5	81952587	82052587	0.474754	ITPR2
5	81952925	82052925	0.432472	ITPR2
5	81953064	82053064	0.446071	ITPR2
5	81953099	82053099	0.426264	ITPR2
5	81953316	82053316	0.426264	ITPR2
5	81953382	82053382	0.417992	ITPR2

5	81953402	82053402	0.421887	ITPR2
5	81953502	82053502	0.386221	ITPR2
5	81953826	82053826	0.396282	ITPR2
5	81954171	82054171	0.371782	ITPR2
5	81954313	82054313	0.377265	ITPR2
5	81954473	82054473	0.374036	ITPR2
5	81955470	82055470	0.365695	ITPR2
5	81955668	82055668	0.365695	ITPR2
5	81955776	82055776	0.369618	ITPR2
5	81955903	82055903	0.363226	ITPR2
5	81956356	82056356	0.374543	ITPR2
5	81956542	82056542	0.384469	ITPR2
5	81957518	82057518	0.377673	ITPR2
5	81957527	82057527	0.371075	ITPR2
5	81957588	82057588	0.387451	ITPR2
5	81957727	82057727	0.382737	ITPR2
5	81957857	82057857	0.38562	ITPR2
5	81957967	82057967	0.400007	ITPR2
5	81958043	82058043	0.394988	ITPR2
5	81958566	82058566	0.377346	ITPR2
5	81958601	82058601	0.380656	ITPR2
5	81958682	82058682	0.384352	ITPR2
5	81958974	82058974	0.392005	ITPR2
5	81959302	82059302	0.383853	ITPR2
5	81959313	82059313	0.385847	ITPR2
5	81959496	82059496	0.390355	ITPR2
5	81959613	82059613	0.38782	ITPR2
5	81959701	82059701	0.395839	ITPR2
5	81959733	82059733	0.395011	ITPR2
5	81959761	82059761	0.393568	ITPR2
5	81960041	82060041	0.393378	ITPR2
5	81960440	82060440	0.391412	ITPR2
5	81960504	82060504	0.388349	ITPR2
5	81960587	82060587	0.388349	ITPR2
5	81960682	82060682	0.392916	ITPR2
5	81961108	82061108	0.380218	ITPR2
5	81961136	82061136	0.393482	ITPR2
5	81961248	82061248	0.398109	ITPR2
5	81961345	82061345	0.396249	ITPR2
5	81961518	82061518	0.402154	ITPR2
5	81961611	82061611	0.402621	ITPR2
5	81962052	82062052	0.409052	ITPR2
5	81962340	82062340	0.411954	ITPR2
5	81962482	82062482	0.421644	ITPR2
5	81962568	82062568	0.418043	ITPR2
5	81962666	82062666	0.417182	ITPR2
5	81962688	82062688	0.417182	ITPR2
5	81962780	82062780	0.417035	ITPR2
5	81962782	82062782	0.406383	ITPR2
5	81962883	82062883	0.392995	ITPR2
5	81963446	82063446	0.431101	ITPR2
5	81963712	82063712	0.420888	ITPR2
5	81963983	82063983	0.424399	ITPR2
5	81964266	82064266	0.420273	ITPR2
5	81964790	82064790	0.427746	ITPR2
5	81964852	82064852	0.448452	ITPR2
5	81964924	82064924	0.438795	ITPR2
5	81965001	82065001	0.438795	ITPR2

5	81965288	82065288	0.442738	ITPR2
5	81965467	82065467	0.433759	ITPR2
5	81965519	82065519	0.412866	ITPR2
5	81965749	82065749	0.407355	ITPR2
5	81965885	82065885	0.407355	ITPR2
5	81965925	82065925	0.407355	ITPR2
5	81966305	82066305	0.403778	ITPR2
5	81966430	82066430	0.431095	ITPR2
5	81966516	82066516	0.422384	ITPR2
5	81967094	82067094	0.41944	ITPR2
5	81967102	82067102	0.427785	ITPR2
5	81967232	82067232	0.434167	ITPR2
5	81967600	82067600	0.425854	ITPR2
5	81967824	82067824	0.436747	ITPR2
5	81967899	82067899	0.437908	ITPR2
5	81967916	82067916	0.439385	ITPR2
5	81967958	82067958	0.437697	ITPR2
5	81968122	82068122	0.419464	ITPR2
5	81968385	82068385	0.430303	ITPR2
5	81968388	82068388	0.430303	ITPR2
5	81968566	82068566	0.443387	ITPR2
5	81968798	82068798	0.43494	ITPR2
5	81968846	82068846	0.446106	ITPR2
5	81968957	82068957	0.455052	ITPR2
5	81969021	82069021	0.454139	ITPR2
5	81969065	82069065	0.454139	ITPR2
5	81969160	82069160	0.454139	ITPR2
5	81969162	82069162	0.454139	ITPR2
5	81969202	82069202	0.454139	ITPR2
5	81969223	82069223	0.454139	ITPR2
5	81969228	82069228	0.454139	ITPR2
5	81969291	82069291	0.488334	ITPR2
5	81969323	82069323	0.486921	ITPR2
5	81969360	82069360	0.482608	ITPR2
5	81969413	82069413	0.483163	ITPR2
5	81969614	82069614	0.47762	ITPR2
5	81969620	82069620	0.484588	ITPR2
5	81969621	82069621	0.47741	ITPR2
5	81969734	82069734	0.464986	ITPR2
5	81969853	82069853	0.461872	ITPR2
5	81969988	82069988	0.470072	ITPR2
5	81970136	82070136	0.460039	ITPR2
5	81970335	82070335	0.470804	ITPR2
5	81970372	82070372	0.474166	ITPR2
5	81970411	82070411	0.474166	ITPR2
5	81970469	82070469	0.474166	ITPR2
5	81970485	82070485	0.478191	ITPR2
5	81970537	82070537	0.483814	ITPR2
5	81970550	82070550	0.482915	ITPR2
5	81970670	82070670	0.463588	ITPR2
5	81970676	82070676	0.470718	ITPR2
5	81970761	82070761	0.470718	ITPR2
5	81970938	82070938	0.470878	ITPR2
5	81970944	82070944	0.451845	ITPR2
5	81971087	82071087	0.449811	ITPR2
5	81971252	82071252	0.439947	ITPR2
5	81971672	82071672	0.442073	ITPR2
5	81971686	82071686	0.439911	ITPR2

5	81971881	82071881	0.439265	ITPR2
5	81971887	82071887	0.453325	ITPR2
5	81971889	82071889	0.453325	ITPR2
5	81971951	82071951	0.438571	ITPR2
5	81971977	82071977	0.472903	ITPR2
5	81972027	82072027	0.472687	ITPR2
5	81972052	82072052	0.454513	ITPR2
5	81972138	82072138	0.452913	ITPR2
5	81972253	82072253	0.453628	ITPR2
5	81972592	82072592	0.453408	ITPR2
5	81972742	82072742	0.45344	ITPR2
5	81972785	82072785	0.45344	ITPR2
5	81972871	82072871	0.440673	ITPR2
5	81972876	82072876	0.440673	ITPR2
5	81972987	82072987	0.446441	ITPR2
5	81972988	82072988	0.446441	ITPR2
5	81973012	82073012	0.453881	ITPR2
5	81973201	82073201	0.454074	ITPR2
5	81973222	82073222	0.46519	ITPR2
5	81973309	82073309	0.451362	ITPR2
5	81973606	82073606	0.449758	ITPR2
5	81973647	82073647	0.468796	ITPR2
5	81973727	82073727	0.441465	ITPR2
5	81973821	82073821	0.439923	ITPR2
5	81974075	82074075	0.439923	ITPR2
5	81974239	82074239	0.430693	ITPR2
5	81974376	82074376	0.433236	ITPR2
5	81974400	82074400	0.433236	ITPR2
5	81974454	82074454	0.433236	ITPR2
5	81974580	82074580	0.433236	ITPR2
5	81974590	82074590	0.44564	ITPR2
5	81974592	82074592	0.434802	ITPR2
5	81974800	82074800	0.44856	ITPR2
5	81974999	82074999	0.428657	ITPR2
5	81975261	82075261	0.433386	ITPR2
5	81975612	82075612	0.436949	ITPR2
5	81975615	82075615	0.436989	ITPR2
5	81975618	82075618	0.434189	ITPR2
5	81975673	82075673	0.438195	ITPR2
5	81975696	82075696	0.446003	ITPR2
5	81975761	82075761	0.443965	ITPR2
5	81975836	82075836	0.441776	ITPR2
5	81976326	82076326	0.431881	ITPR2
5	81976406	82076406	0.463162	ITPR2
5	81976654	82076654	0.44453	ITPR2
5	81977224	82077224	0.443533	ITPR2
5	81977341	82077341	0.424907	ITPR2
5	81977746	82077746	0.43526	ITPR2
5	81978239	82078239	0.442792	ITPR2
5	81978599	82078599	0.438826	ITPR2
5	81978691	82078691	0.448888	ITPR2
5	81978786	82078786	0.449362	ITPR2
5	81978834	82078834	0.455295	ITPR2
5	81979059	82079059	0.44748	ITPR2
5	81979238	82079238	0.423288	ITPR2
5	81979426	82079426	0.436377	ITPR2
5	81979523	82079523	0.386866	ITPR2
5	81979958	82079958	0.388556	ITPR2

5	81980423	82080423	0.389227	ITPR2
5	81980730	82080730	0.389227	ITPR2
5	81980733	82080733	0.39487	ITPR2
5	81980873	82080873	0.385873	ITPR2
5	81980888	82080888	0.378487	ITPR2
5	81980973	82080973	0.373753	ITPR2
5	81981532	82081532	0.377554	ITPR2
5	81981837	82081837	0.447342	ITPR2
5	81982704	82082704	0.435474	ITPR2
5	81982708	82082708	0.440371	ITPR2
5	81982841	82082841	0.440371	ITPR2
5	81982913	82082913	0.445769	ITPR2
5	81982916	82082916	0.445769	ITPR2
5	81982999	82082999	0.463257	ITPR2
5	81983066	82083066	0.496052	ITPR2
5	81983103	82083103	0.488675	ITPR2
5	81983146	82083146	0.484891	ITPR2
5	81983203	82083203	0.488956	ITPR2
5	81983408	82083408	0.491882	ITPR2
5	81983505	82083505	0.491882	ITPR2
5	81983597	82083597	0.499391	ITPR2
5	81983656	82083656	0.500845	ITPR2
5	81983739	82083739	0.500845	ITPR2
5	81983809	82083809	0.497991	ITPR2
5	81983964	82083964	0.486628	ITPR2
5	81984014	82084014	0.486628	ITPR2
5	81984049	82084049	0.485	ITPR2
5	81984174	82084174	0.476061	ITPR2
5	81984192	82084192	0.476061	ITPR2
5	81984316	82084316	0.476061	ITPR2
5	81984357	82084357	0.470295	ITPR2
5	81984383	82084383	0.472002	ITPR2
5	81984430	82084430	0.472002	ITPR2
5	81984504	82084504	0.468771	ITPR2
5	81984598	82084598	0.472885	ITPR2
5	81984613	82084613	0.472885	ITPR2
5	81984654	82084654	0.470851	ITPR2
5	81984658	82084658	0.470851	ITPR2
5	81984977	82084977	0.465157	ITPR2
5	81985034	82085034	0.473233	ITPR2
5	81985048	82085048	0.509089	ITPR2
5	81985144	82085144	0.49717	ITPR2
5	81985184	82085184	0.495092	ITPR2
5	81985247	82085247	0.495779	ITPR2
5	81985378	82085378	0.495779	ITPR2
5	81985488	82085488	0.507644	ITPR2
5	81985497	82085497	0.496453	ITPR2
5	81985515	82085515	0.505729	ITPR2
5	81985520	82085520	0.504241	ITPR2
5	81985546	82085546	0.504241	ITPR2
5	81985609	82085609	0.516033	ITPR2
5	81985627	82085627	0.50917	ITPR2
5	81985663	82085663	0.50796	ITPR2
5	81985699	82085699	0.5079	ITPR2
5	81985737	82085737	0.509636	ITPR2
5	81985755	82085755	0.494777	ITPR2
5	81985818	82085818	0.50049	ITPR2
5	81985866	82085866	0.500519	ITPR2

5	81985908	82085908	0.502167	ITPR2
5	81985909	82085909	0.502167	ITPR2
5	81986004	82086004	0.502171	ITPR2
5	81986052	82086052	0.507803	ITPR2
5	81986054	82086054	0.507453	ITPR2
5	81986114	82086114	0.519879	ITPR2
5	81986137	82086137	0.520091	ITPR2
5	81986143	82086143	0.525173	ITPR2
5	81986154	82086154	0.52019	ITPR2
5	81986157	82086157	0.536573	ITPR2
5	81986183	82086183	0.541768	ITPR2
5	81986206	82086206	0.53565	ITPR2
5	81986271	82086271	0.531142	ITPR2
5	81986290	82086290	0.535999	ITPR2
5	81986405	82086405	0.535619	ITPR2
5	81986441	82086441	0.535986	ITPR2
5	81986443	82086443	0.535986	ITPR2
5	81986453	82086453	0.536538	ITPR2
5	81986503	82086503	0.584205	ITPR2
5	81986627	82086627	0.578575	ITPR2
5	81986730	82086730	0.575812	ITPR2
5	81986737	82086737	0.575812	ITPR2
5	81986790	82086790	0.587412	ITPR2
5	81986850	82086850	0.587986	ITPR2
5	81986851	82086851	0.575004	ITPR2
5	81986938	82086938	0.574824	ITPR2
5	81987011	82087011	0.577288	ITPR2
5	81987040	82087040	0.577002	ITPR2
5	81987049	82087049	0.577002	ITPR2
5	81987068	82087068	0.577002	ITPR2
5	81987111	82087111	0.574544	ITPR2
5	81987152	82087152	0.57554	ITPR2
5	81987217	82087217	0.580343	ITPR2
5	81987295	82087295	0.551662	ITPR2
5	81987434	82087434	0.551662	ITPR2
5	81987466	82087466	0.541984	ITPR2
5	81987604	82087604	0.54593	ITPR2
5	81987640	82087640	0.546651	ITPR2
5	81987715	82087715	0.548782	ITPR2
5	81987722	82087722	0.548782	ITPR2
5	81987734	82087734	0.549003	ITPR2
5	81987781	82087781	0.546123	ITPR2
5	81987790	82087790	0.554523	ITPR2
5	81987811	82087811	0.554765	ITPR2
5	81987840	82087840	0.553852	ITPR2
5	81987872	82087872	0.549979	ITPR2
5	81987899	82087899	0.56583	ITPR2
5	81987998	82087998	0.540507	ITPR2
5	81988034	82088034	0.532589	ITPR2
5	81988106	82088106	0.532555	ITPR2
5	81988272	82088272	0.544941	ITPR2
5	81988305	82088305	0.544091	ITPR2
5	81988366	82088366	0.419397	ITPR2
5	81988496	82088496	0.454549	ITPR2
5	81988580	82088580	0.452742	ITPR2
5	81988608	82088608	0.452742	ITPR2
5	81988747	82088747	0.452742	ITPR2
5	81989023	82089023	0.446367	ITPR2

5	81989058	82089058	0.449898	ITPR2
5	81989074	82089074	0.449898	ITPR2
5	81989084	82089084	0.449898	ITPR2
5	81989091	82089091	0.449898	ITPR2
5	81989104	82089104	0.449898	ITPR2
5	81989168	82089168	0.449898	ITPR2
5	81989175	82089175	0.449898	ITPR2
5	81989180	82089180	0.449898	ITPR2
5	81989246	82089246	0.449898	ITPR2
5	81989316	82089316	0.449898	ITPR2
5	81989335	82089335	0.449898	ITPR2
5	81989377	82089377	0.449295	ITPR2
5	81989433	82089433	0.498198	ITPR2
5	81989451	82089451	0.485464	ITPR2
5	81989574	82089574	0.486903	ITPR2
5	81989620	82089620	0.486903	ITPR2
5	81989680	82089680	0.486471	ITPR2
5	81989695	82089695	0.486969	ITPR2
5	81989721	82089721	0.491442	ITPR2
5	81989724	82089724	0.491442	ITPR2
5	81989726	82089726	0.491442	ITPR2
5	81989777	82089777	0.517558	ITPR2
5	81989813	82089813	0.549113	ITPR2
5	81989823	82089823	0.473268	ITPR2
5	81989852	82089852	0.46854	ITPR2
5	81989899	82089899	0.459622	ITPR2
5	81990087	82090087	0.439653	ITPR2
5	81990219	82090219	0.442809	ITPR2
5	81990247	82090247	0.442159	ITPR2
5	81990282	82090282	0.432241	ITPR2
5	81990384	82090384	0.432241	ITPR2
5	81990397	82090397	0.417022	ITPR2
5	81990498	82090498	0.418653	ITPR2
5	81990518	82090518	0.418202	ITPR2
5	81990554	82090554	0.421812	ITPR2
5	81990596	82090596	0.421812	ITPR2
5	81990612	82090612	0.421812	ITPR2
5	81990754	82090754	0.421812	ITPR2
5	81990775	82090775	0.421812	ITPR2
5	81990824	82090824	0.421812	ITPR2
5	81990888	82090888	0.421812	ITPR2
5	81991057	82091057	0.421812	ITPR2
5	81991065	82091065	0.442734	ITPR2
5	81991116	82091116	0.433885	ITPR2
5	81991119	82091119	0.433885	ITPR2
5	81991133	82091133	0.433885	ITPR2
5	81991136	82091136	0.439479	ITPR2
5	81991137	82091137	0.438193	ITPR2
5	81991164	82091164	0.428211	ITPR2
5	81991313	82091313	0.429013	ITPR2
5	81991339	82091339	0.429013	ITPR2
5	81991461	82091461	0.429054	ITPR2
5	81991464	82091464	0.429054	ITPR2
5	81991471	82091471	0.429054	ITPR2
5	81991474	82091474	0.429054	ITPR2
5	81991480	82091480	0.429054	ITPR2
5	81991483	82091483	0.429054	ITPR2
5	81991530	82091530	0.426491	ITPR2

5	81991558	82091558	0.43252	ITPR2
5	81991585	82091585	0.429559	ITPR2
5	81991677	82091677	0.429559	ITPR2
5	81991696	82091696	0.429559	ITPR2
5	81991753	82091753	0.40188	ITPR2
5	81991814	82091814	0.435584	ITPR2
5	81991844	82091844	0.433045	ITPR2
5	81991892	82091892	0.434459	ITPR2
5	81992040	82092040	0.43228	ITPR2
5	81992135	82092135	0.43228	ITPR2
5	81992172	82092172	0.43228	ITPR2
5	81992187	82092187	0.44543	ITPR2
5	81992224	82092224	0.435467	ITPR2
5	81992253	82092253	0.435604	ITPR2
5	81992368	82092368	0.432727	ITPR2
5	81992419	82092419	0.427826	ITPR2
5	81992451	82092451	0.424998	ITPR2
5	81992453	82092453	0.417089	ITPR2
5	81992683	82092683	0.427985	ITPR2
5	81992767	82092767	0.426537	ITPR2
5	81992983	82092983	0.425235	ITPR2
5	81993228	82093228	0.428136	ITPR2
5	81993377	82093377	0.424325	ITPR2
5	81993512	82093512	0.432186	ITPR2
5	81993769	82093769	0.428371	ITPR2
5	81993826	82093826	0.437149	ITPR2
5	81993873	82093873	0.421021	ITPR2
5	81993918	82093918	0.420708	ITPR2
5	81993997	82093997	0.425215	ITPR2
5	81994009	82094009	0.427548	ITPR2
5	81994044	82094044	0.427548	ITPR2
5	81994110	82094110	0.428644	ITPR2
5	81994164	82094164	0.424949	ITPR2
5	81994165	82094165	0.42993	ITPR2
5	81994252	82094252	0.429463	ITPR2
5	81994309	82094309	0.42481	ITPR2
5	81994311	82094311	0.412396	ITPR2
5	81994377	82094377	0.412396	ITPR2
5	81994460	82094460	0.412396	ITPR2
5	81994530	82094530	0.418099	ITPR2
5	81994793	82094793	0.392185	ITPR2
5	81994949	82094949	0.399387	ITPR2
5	81995044	82095044	0.400426	ITPR2
5	81995061	82095061	0.400426	ITPR2
5	81995077	82095077	0.398853	ITPR2
5	81995096	82095096	0.398034	ITPR2
5	81995122	82095122	0.395849	ITPR2
5	81995137	82095137	0.395849	ITPR2
5	81995176	82095176	0.396163	ITPR2
5	81995187	82095187	0.397242	ITPR2
5	81995217	82095217	0.407599	ITPR2
5	81995220	82095220	0.386837	ITPR2
5	81995241	82095241	0.386837	ITPR2
5	81995271	82095271	0.386523	ITPR2
5	81995331	82095331	0.399618	ITPR2
5	81995335	82095335	0.398747	ITPR2
5	81995369	82095369	0.399313	ITPR2
5	81995370	82095370	0.393668	ITPR2

5	81995371	82095371	0.393668	ITPR2
5	81995424	82095424	0.393668	ITPR2
5	81995589	82095589	0.393668	ITPR2
5	81995679	82095679	0.387919	ITPR2
5	81995774	82095774	0.395465	ITPR2
5	81995876	82095876	0.37956	ITPR2
5	81996074	82096074	0.37956	ITPR2
5	81996081	82096081	0.37956	ITPR2
5	81996121	82096121	0.37956	ITPR2
5	81996146	82096146	0.37956	ITPR2
5	81996161	82096161	0.375499	ITPR2
5	81996276	82096276	0.380476	ITPR2
5	81996302	82096302	0.380476	ITPR2
5	81996352	82096352	0.380476	ITPR2
5	81996353	82096353	0.380476	ITPR2
5	81996372	82096372	0.380476	ITPR2
5	81996377	82096377	0.403061	ITPR2
5	81996445	82096445	0.397034	ITPR2
5	81996541	82096541	0.410664	ITPR2
5	81996716	82096716	0.391092	ITPR2
5	81996777	82096777	0.391909	ITPR2
5	81996804	82096804	0.390852	ITPR2
5	81996826	82096826	0.390852	ITPR2
5	81996906	82096906	0.390852	ITPR2
5	81996907	82096907	0.390852	ITPR2
5	81996922	82096922	0.390852	ITPR2
5	81997013	82097013	0.391519	ITPR2
5	81997085	82097085	0.411086	ITPR2
5	81997099	82097099	0.393993	ITPR2
5	81997107	82097107	0.393993	ITPR2
5	81997128	82097128	0.394092	ITPR2
5	81997135	82097135	0.394092	ITPR2
5	81997228	82097228	0.361647	ITPR2
5	81997327	82097327	0.361949	ITPR2
5	81997352	82097352	0.361718	ITPR2
5	81997417	82097417	0.361718	ITPR2
5	81997438	82097438	0.361674	ITPR2
5	81997440	82097440	0.368791	ITPR2
5	81997457	82097457	0.366071	ITPR2
5	81997463	82097463	0.352792	ITPR2
5	81997464	82097464	0.352792	ITPR2
5	81997668	82097668	0.353372	ITPR2
5	81997723	82097723	0.353372	ITPR2
5	81998074	82098074	0.361943	ITPR2
5	81998157	82098157	0.345892	ITPR2
5	81998235	82098235	0.343013	ITPR2
5	81998290	82098290	0.345278	ITPR2
5	81998435	82098435	0.345278	ITPR2
5	82026460	82126460	0.350413	ITPR2
5	82026519	82126519	0.357288	ITPR2
5	82026552	82126552	0.377532	ITPR2
5	82027063	82127063	0.350792	ITPR2
5	82027150	82127150	0.376026	ITPR2
5	82027213	82127213	0.356222	ITPR2
5	82027521	82127521	0.368089	ITPR2
5	82027859	82127859	0.388196	ITPR2
5	82027937	82127937	0.35421	ITPR2
5	82028367	82128367	0.396433	ITPR2

5	82028470	82128470	0.374833	ITPR2
5	82028613	82128613	0.366516	ITPR2
5	82029115	82129115	0.358768	ITPR2
5	82029138	82129138	0.366767	ITPR2
5	82029565	82129565	0.384424	ITPR2
5	82029579	82129579	0.399377	ITPR2
5	82030111	82130111	0.406614	ITPR2
5	82030365	82130365	0.389202	ITPR2
5	82030577	82130577	0.400116	ITPR2
5	82030695	82130695	0.419014	ITPR2
5	82030796	82130796	0.426774	ITPR2
5	82030875	82130875	0.41939	ITPR2
5	82031072	82131072	0.407005	ITPR2
5	82031388	82131388	0.416649	ITPR2
5	82031395	82131395	0.432119	ITPR2
5	82031646	82131646	0.414705	ITPR2
5	82031878	82131878	0.404281	ITPR2
5	82032117	82132117	0.408715	ITPR2
5	82032217	82132217	0.403894	ITPR2
5	82032316	82132316	0.417936	ITPR2
5	82032334	82132334	0.42674	ITPR2
5	82032407	82132407	0.442752	ITPR2
5	82032610	82132610	0.431989	ITPR2
5	82032995	82132995	0.44326	ITPR2
5	82032996	82132996	0.440921	ITPR2
5	82033249	82133249	0.44465	ITPR2
5	82033323	82133323	0.437226	ITPR2
5	82033336	82133336	0.44026	ITPR2
5	82033430	82133430	0.447855	ITPR2
5	82033473	82133473	0.435542	ITPR2
5	82033537	82133537	0.437295	ITPR2
5	82033701	82133701	0.435366	ITPR2
5	82033946	82133946	0.388287	ITPR2
5	82034108	82134108	0.388859	ITPR2
5	82034476	82134476	0.386206	ITPR2
5	82034552	82134552	0.393964	ITPR2
5	82034696	82134696	0.365941	ITPR2
5	82034798	82134798	0.34441	ITPR2
5	82034882	82134882	0.383637	ITPR2
5	82035262	82135262	0.350024	ITPR2
5	82036377	82136377	0.350024	ITPR2
5	82036881	82136881	0.387815	ITPR2
5	82036906	82136906	0.40416	ITPR2
5	82037192	82137192	0.380961	ITPR2
5	82037426	82137426	0.404664	ITPR2
5	82037531	82137531	0.375834	ITPR2
5	82037538	82137538	0.393964	ITPR2
5	82037625	82137625	0.441812	ITPR2
5	82037909	82137909	0.385366	ITPR2
5	82038117	82138117	0.390545	ITPR2
5	82038319	82138319	0.378353	ITPR2
5	82038597	82138597	0.390234	ITPR2
5	82038805	82138805	0.381847	ITPR2
5	82039097	82139097	0.364398	ITPR2
5	82039601	82139601	0.370535	ITPR2
5	82039924	82139924	0.382586	ITPR2
5	82040088	82140088	0.382685	ITPR2
5	82040286	82140286	0.354101	ITPR2

5	82040417	82140417	0.435167	ITPR2
5	82040654	82140654	0.410219	ITPR2
5	82040696	82140696	0.412291	ITPR2
5	82040944	82140944	0.401495	ITPR2
5	82040964	82140964	0.400437	ITPR2
5	82041238	82141238	0.385974	ITPR2
5	82041282	82141282	0.383126	ITPR2
5	82041320	82141320	0.399267	ITPR2
5	82041898	82141898	0.391363	ITPR2
5	82042025	82142025	0.387598	ITPR2
5	82042066	82142066	0.393088	ITPR2
5	82042133	82142133	0.384434	ITPR2
5	82042160	82142160	0.379432	ITPR2
5	82042200	82142200	0.401302	ITPR2
5	82042318	82142318	0.404986	ITPR2
5	82042395	82142395	0.404986	ITPR2
5	82042446	82142446	0.415599	ITPR2
5	82042500	82142500	0.42531	ITPR2
5	82042520	82142520	0.417059	ITPR2
5	82042521	82142521	0.417059	ITPR2
5	82042522	82142522	0.410918	ITPR2
5	82042537	82142537	0.404513	ITPR2
5	82042674	82142674	0.380646	ITPR2
5	82042723	82142723	0.369719	ITPR2
5	82043045	82143045	0.342321	ITPR2
5	82043760	82143760	0.34355	ITPR2
5	82043919	82143919	0.358377	ITPR2
5	82044014	82144014	0.358377	ITPR2
5	82044104	82144104	0.373218	ITPR2
5	82044214	82144214	0.374006	ITPR2
5	82044244	82144244	0.399393	ITPR2
5	82044679	82144679	0.392591	ITPR2
5	82045030	82145030	0.39089	ITPR2
5	82045091	82145091	0.38999	ITPR2
5	82045496	82145496	0.387832	ITPR2
5	82045563	82145563	0.34425	ITPR2
5	82045597	82145597	0.350308	ITPR2
5	82047546	82147546	0.342276	ITPR2
5	82047715	82147715	0.34508	ITPR2
5	82048900	82148900	0.345829	ITPR2
5	82052310	82152310	0.3457	ITPR2
5	82052531	82152531	0.393378	ITPR2
5	82052603	82152603	0.37024	ITPR2
5	82052809	82152809	0.371793	ITPR2
5	82053155	82153155	0.347113	ITPR2
5	82053321	82153321	0.34668	ITPR2
5	82059075	82159075	0.368835	ITPR2
5	82059085	82159085	0.360925	ITPR2
5	82059875	82159875	0.355428	ITPR2
5	82059916	82159916	0.344704	ITPR2
5	82060118	82160118	0.344704	ITPR2
5	82060140	82160140	0.344704	ITPR2
5	82060157	82160157	0.344704	ITPR2
5	82060265	82160265	0.36075	ITPR2
5	82060443	82160443	0.342235	ITPR2
5	82062396	82162396	0.348378	ITPR2
5	82065241	82165241	0.349012	ITPR2
5	82065272	82165272	0.348976	ITPR2

5	82065464	82165464	0.341875	ITPR2
5	82065472	82165472	0.344033	ITPR2
5	82065487	82165487	0.351239	ITPR2
5	82065557	82165557	0.344836	ITPR2
5	82066337	82166337	0.346902	ITPR2
5	82066776	82166776	0.356124	ITPR2
5	82066803	82166803	0.354743	ITPR2
5	82067102	82167102	0.34903	ITPR2
5	82068934	82168934	0.367941	ITPR2
5	82069160	82169160	0.431324	ITPR2
5	82069175	82169175	0.418446	ITPR2
5	82069192	82169192	0.417491	ITPR2
5	82069236	82169236	0.360854	ITPR2
5	82069915	82169915	0.364584	ITPR2
5	82069990	82169990	0.368191	ITPR2
5	82070279	82170279	0.368191	ITPR2
5	82070491	82170491	0.368191	ITPR2
5	82070620	82170620	0.403987	ITPR2
5	82070803	82170803	0.368475	ITPR2
5	82071035	82171035	0.377088	ITPR2
5	82071132	82171132	0.399919	ITPR2
5	82071177	82171177	0.395929	ITPR2
5	82071612	82171612	0.394256	ITPR2
5	82071816	82171816	0.395764	ITPR2
5	82071833	82171833	0.3916	ITPR2
5	82071882	82171882	0.392766	ITPR2
5	82071947	82171947	0.383284	ITPR2
5	82072012	82172012	0.398856	ITPR2
5	82072108	82172108	0.406683	ITPR2
5	82072158	82172158	0.406683	ITPR2
5	82072164	82172164	0.415312	ITPR2
5	82072224	82172224	0.411179	ITPR2
5	82072509	82172509	0.423093	ITPR2
5	82072519	82172519	0.448666	ITPR2
5	82072593	82172593	0.410899	ITPR2
5	82072629	82172629	0.422207	ITPR2
5	82072630	82172630	0.404105	ITPR2
5	82072695	82172695	0.408814	ITPR2
5	82072708	82172708	0.404734	ITPR2
5	82072861	82172861	0.405962	ITPR2
5	82073002	82173002	0.412824	ITPR2
5	82073023	82173023	0.394376	ITPR2
5	82073082	82173082	0.39531	ITPR2
5	82073177	82173177	0.394472	ITPR2
5	82086165	82186165	0.343632	ITPR2
5	82086246	82186246	0.341698	ITPR2
5	82087713	82187713	0.342353	ITPR2
5	82088199	82188199	0.342506	ITPR2
5	82088320	82188320	0.349548	ITPR2
5	82088357	82188357	0.343344	ITPR2
5	82098496	82198496	0.342902	ITPR2
5	82099727	82199727	0.345723	ITPR2
5	82099792	82199792	0.351744	ITPR2
5	82100163	82200163	0.355291	ITPR2
5	82100268	82200268	0.365678	ITPR2
5	82100272	82200272	0.373716	ITPR2
5	82100482	82200482	0.385453	ITPR2
5	82100517	82200517	0.381311	ITPR2

5	82100573	82200573	0.370993	ITPR2
5	82100673	82200673	0.366	ITPR2
5	82100720	82200720	0.369288	ITPR2
5	82100765	82200765	0.366255	ITPR2
5	82100820	82200820	0.371116	ITPR2
5	82100920	82200920	0.357454	ITPR2
5	82101097	82201097	0.358239	ITPR2
5	82101371	82201371	0.355797	ITPR2
5	82101559	82201559	0.34547	ITPR2
5	82102091	82202091	0.350117	ITPR2
5	82102104	82202104	0.350117	ITPR2
5	82102359	82202359	0.349716	ITPR2
5	82102487	82202487	0.366435	ITPR2
5	82102558	82202558	0.366435	ITPR2
5	82102754	82202754	0.360515	ITPR2
5	82102766	82202766	0.355518	ITPR2
5	82103030	82203030	0.349623	ITPR2
5	82103221	82203221	0.348262	ITPR2
5	82103646	82203646	0.348034	ITPR2
5	82104382	82204382	0.34858	ITPR2
5	82104544	82204544	0.346532	ITPR2
5	82105260	82205260	0.371354	ITPR2
5	82105587	82205587	0.371071	ITPR2
5	82105824	82205824	0.379353	ITPR2
5	82105832	82205832	0.379233	ITPR2
5	82105884	82205884	0.382108	ITPR2
5	82106085	82206085	0.382569	ITPR2
5	82106486	82206486	0.388737	ITPR2
5	82106500	82206500	0.391302	ITPR2
5	82106671	82206671	0.396267	ITPR2
5	82106745	82206745	0.40005	ITPR2
5	82107013	82207013	0.398521	ITPR2
5	82107026	82207026	0.392167	ITPR2
5	82107271	82207271	0.393371	ITPR2
5	82107512	82207512	0.383986	ITPR2
5	82107601	82207601	0.387477	ITPR2
5	82107935	82207935	0.391582	ITPR2
5	82108109	82208109	0.390259	ITPR2
5	82108161	82208161	0.390259	ITPR2
5	82108227	82208227	0.395643	ITPR2
5	82108331	82208331	0.383128	ITPR2
5	82110275	82210275	0.349204	ITPR2
5	82110623	82210623	0.348034	ITPR2
5	82111036	82211036	0.348091	ITPR2
5	82111300	82211300	0.357914	ITPR2
5	82111345	82211345	0.359654	ITPR2
5	82112330	82212330	0.346974	ITPR2
5	82113116	82213116	0.349631	ITPR2
5	82113847	82213847	0.355482	ITPR2
5	82113952	82213952	0.346698	ITPR2
5	82114186	82214186	0.342919	ITPR2
5	82114240	82214240	0.34586	ITPR2
5	82114253	82214253	0.34586	ITPR2
5	82114301	82214301	0.360249	ITPR2
5	82114340	82214340	0.360734	ITPR2
5	82114477	82214477	0.365723	ITPR2
5	82114785	82214785	0.366899	ITPR2
5	82114932	82214932	0.356597	ITPR2

5	82115171	82215171	0.404931	ITPR2
5	82116689	82216689	0.344917	ITPR2
5	82198898	82298898	0.370925	SSPN
5	82199021	82299021	0.369959	SSPN
5	82199432	82299432	0.373121	SSPN
5	82200019	82300019	0.362115	SSPN
5	82200063	82300063	0.353132	SSPN
5	82231039	82331039	0.363746	SSPN
5	82231362	82331362	0.361444	SSPN
5	82231510	82331510	0.365043	SSPN
5	82231878	82331878	0.35364	SSPN
5	82244884	82344884	0.362561	SSPN
5	82246848	82346848	0.346013	SSPN
5	82247876	82347876	0.343725	SSPN
5	82248023	82348023	0.342711	SSPN
5	82248241	82348241	0.368601	SSPN
5	82248256	82348256	0.353674	SSPN
5	82248257	82348257	0.354392	SSPN
5	82248848	82348848	0.344854	SSPN
5	82249330	82349330	0.34509	SSPN
5	82249494	82349494	0.344629	SSPN
5	82422261	82522261	0.34234	LOC102171494
5	82422261	82522261	0.34234	RASSF8
5	82422262	82522262	0.34234	LOC102171494
5	82422262	82522262	0.34234	RASSF8
5	82422263	82522263	0.34234	LOC102171494
5	82422263	82522263	0.34234	RASSF8
5	82422284	82522284	0.342541	LOC102171494
5	82422284	82522284	0.342541	RASSF8
5	82423252	82523252	0.35275	LOC102171494
5	82423252	82523252	0.35275	RASSF8
5	82423459	82523459	0.375076	LOC102171494
5	82423459	82523459	0.375076	RASSF8
5	82423474	82523474	0.38074	LOC102171494
5	82423474	82523474	0.38074	RASSF8
5	82423769	82523769	0.380466	LOC102171494
5	82423769	82523769	0.380466	RASSF8
5	82423832	82523832	0.410265	LOC102171494
5	82423832	82523832	0.410265	RASSF8
5	82424121	82524121	0.350774	LOC102171494
5	82424121	82524121	0.350774	RASSF8
5	82425317	82525317	0.341763	LOC102171494
5	82425317	82525317	0.341763	RASSF8
5	82425493	82525493	0.344595	LOC102171494
5	82425493	82525493	0.344595	RASSF8
5	82434192	82534192	0.355615	LOC102171494
5	82434192	82534192	0.355615	RASSF8
5	82434433	82534433	0.371581	LOC102171494
5	82434433	82534433	0.371581	RASSF8
5	82434512	82534512	0.371598	LOC102171494
5	82434512	82534512	0.371598	RASSF8
5	82434545	82534545	0.37354	LOC102171494
5	82434545	82534545	0.37354	RASSF8
5	82434664	82534664	0.376589	LOC102171494
5	82434664	82534664	0.376589	RASSF8
5	82434698	82534698	0.34678	LOC102171494
5	82434698	82534698	0.34678	RASSF8
5	82436173	82536173	0.349314	LOC102171494

5	82436173	82536173	0.349314	RASSF8
5	82436212	82536212	0.350334	LOC102171494
5	82436212	82536212	0.350334	RASSF8
5	82436342	82536342	0.357566	LOC102171494
5	82436342	82536342	0.357566	RASSF8
5	82436523	82536523	0.348347	LOC102171494
5	82436523	82536523	0.348347	RASSF8
5	82436525	82536525	0.348347	LOC102171494
5	82436525	82536525	0.348347	RASSF8
5	82436548	82536548	0.348347	LOC102171494
5	82436548	82536548	0.348347	RASSF8
5	82437022	82537022	0.34648	LOC102171494
5	82437022	82537022	0.34648	RASSF8
5	82437038	82537038	0.34776	LOC102171494
5	82437038	82537038	0.34776	RASSF8
5	82437212	82537212	0.341794	LOC102171494
5	82437212	82537212	0.341794	RASSF8
5	82437290	82537290	0.344526	LOC102171494
5	82437290	82537290	0.344526	RASSF8
5	84404274	84504274	0.347942	SOX5
5	84404997	84504997	0.369167	SOX5
5	84405093	84505093	0.384556	SOX5
5	84405142	84505142	0.367726	SOX5
5	84405704	84505704	0.348899	SOX5
5	84405970	84505970	0.382129	SOX5
5	84405972	84505972	0.368231	SOX5
5	84406123	84506123	0.382549	SOX5
5	84406170	84506170	0.381031	SOX5
5	84406180	84506180	0.401341	SOX5
5	84407928	84507928	0.343257	SOX5
5	84463401	84563401	0.353265	SOX5
5	84463421	84563421	0.35178	SOX5
5	84463505	84563505	0.350826	SOX5
5	84473363	84573363	0.345341	SOX5
5	84474508	84574508	0.343064	SOX5
5	84474742	84574742	0.354463	SOX5
5	84475068	84575068	0.35588	SOX5
5	84475243	84575243	0.346418	SOX5
5	84476089	84576089	0.348272	SOX5
5	84476783	84576783	0.35303	SOX5
5	84476834	84576834	0.352981	SOX5
5	84477056	84577056	0.353871	SOX5
5	84477149	84577149	0.352982	SOX5
5	84477260	84577260	0.342225	SOX5
5	84477784	84577784	0.355329	SOX5
5	84477785	84577785	0.355329	SOX5
5	84478073	84578073	0.344758	SOX5
5	84478100	84578100	0.34288	SOX5
5	84478224	84578224	0.344155	SOX5
5	84478601	84578601	0.34521	SOX5
5	84495465	84595465	0.347914	SOX5
5	84496030	84596030	0.362826	SOX5
5	84496052	84596052	0.353067	SOX5
5	84496057	84596057	0.353067	SOX5
5	84496809	84596809	0.353067	SOX5
5	84497532	84597532	0.361011	SOX5
5	84497538	84597538	0.367506	SOX5
5	84498043	84598043	0.355662	SOX5

5	84498216	84598216	0.355385	SOX5
5	84498314	84598314	0.35377	SOX5
5	84498553	84598553	0.35377	SOX5
5	84498646	84598646	0.354563	SOX5
5	84504263	84604263	0.356616	SOX5
5	84504823	84604823	0.355274	SOX5
5	84504887	84604887	0.345381	SOX5
5	84505027	84605027	0.345381	SOX5
5	84505307	84605307	0.345381	SOX5
5	84505622	84605622	0.346404	SOX5
5	84506746	84606746	0.354904	SOX5
5	84506777	84606777	0.378745	SOX5
5	84506789	84606789	0.359299	SOX5
5	84507157	84607157	0.356132	SOX5
5	84508987	84608987	0.369492	SOX5
5	84509082	84609082	0.379898	SOX5
5	84509591	84609591	0.383044	SOX5
5	84509839	84609839	0.383204	SOX5
5	84509912	84609912	0.383204	SOX5
5	84509917	84609917	0.367787	SOX5
5	84510053	84610053	0.367787	SOX5
5	84510436	84610436	0.358127	SOX5
5	84510856	84610856	0.357367	SOX5
5	84511457	84611457	0.359955	SOX5
5	84512384	84612384	0.364454	SOX5
5	84512396	84612396	0.378661	SOX5
5	84514959	84614959	0.346813	SOX5
5	84520770	84620770	0.347778	SOX5
5	85277019	85377019	0.378698	LOC102175101
5	85277165	85377165	0.38696	LOC102175101
5	85277400	85377400	0.386609	LOC102175101
5	85277416	85377416	0.388447	LOC102175101
5	85277418	85377418	0.3926	LOC102175101
5	85277535	85377535	0.386733	LOC102175101
5	85277555	85377555	0.383407	LOC102175101
5	85277693	85377693	0.388624	LOC102175101
5	85277785	85377785	0.3851	LOC102175101
5	85277826	85377826	0.381338	LOC102175101
5	85277872	85377872	0.357456	LOC102175101
5	85278257	85378257	0.356438	LOC102175101
5	85278411	85378411	0.356438	LOC102175101
5	85278442	85378442	0.365952	LOC102175101
5	85278453	85378453	0.365952	LOC102175101
5	85278529	85378529	0.365952	LOC102175101
5	85278627	85378627	0.374803	LOC102175101
5	85278791	85378791	0.372966	LOC102175101
5	85278846	85378846	0.367363	LOC102175101
5	85278847	85378847	0.360881	LOC102175101
5	85279067	85379067	0.384602	LOC102175101
5	85279138	85379138	0.390195	LOC102175101
5	85279194	85379194	0.37705	LOC102175101
5	85279257	85379257	0.369836	LOC102175101
5	85279454	85379454	0.369836	LOC102175101
5	85279493	85379493	0.376616	LOC102175101
5	85279679	85379679	0.374337	LOC102175101
5	85280085	85380085	0.366468	LOC102175101
5	85280186	85380186	0.384727	LOC102175101
5	85280273	85380273	0.380833	LOC102175101

5	85280352	85380352	0.382426	LOC102175101
5	85280451	85380451	0.37416	LOC102175101
5	85280556	85380556	0.373743	LOC102175101
5	85280565	85380565	0.374515	LOC102175101
5	85280676	85380676	0.374515	LOC102175101
5	85280752	85380752	0.369727	LOC102175101
5	85280854	85380854	0.377933	LOC102175101
5	85280870	85380870	0.376298	LOC102175101
5	85281067	85381067	0.37594	LOC102175101
5	85281373	85381373	0.369307	LOC102175101
5	85281419	85381419	0.369307	LOC102175101
5	85281779	85381779	0.384325	LOC102175101
5	85281788	85381788	0.394161	LOC102175101
5	85281903	85381903	0.394778	LOC102175101
5	85281914	85381914	0.39075	LOC102175101
5	85281982	85381982	0.407063	LOC102175101
5	85282058	85382058	0.403038	LOC102175101
5	85282186	85382186	0.404335	LOC102175101
5	85282208	85382208	0.409013	LOC102175101
5	85282219	85382219	0.404995	LOC102175101
5	85282422	85382422	0.403294	LOC102175101
5	85282434	85382434	0.433018	LOC102175101
5	85282644	85382644	0.425197	LOC102175101
5	85282946	85382946	0.42851	LOC102175101
5	85282959	85382959	0.422648	LOC102175101
5	85283135	85383135	0.433797	LOC102175101
5	85283143	85383143	0.442094	LOC102175101
5	85283155	85383155	0.441569	LOC102175101
5	85283163	85383163	0.444354	LOC102175101
5	85283203	85383203	0.442975	LOC102175101
5	85283242	85383242	0.442975	LOC102175101
5	85283249	85383249	0.43968	LOC102175101
5	85283278	85383278	0.43968	LOC102175101
5	85737200	85837200	0.342151	TRNAK-UUU-9
5	85765819	85865819	0.356986	TRNAK-UUU-9
5	85767708	85867708	0.359628	TRNAK-UUU-9
5	85767956	85867956	0.384578	TRNAK-UUU-9
5	85768267	85868267	0.373687	TRNAK-UUU-9
5	85768272	85868272	0.356666	TRNAK-UUU-9
5	85768579	85868579	0.427069	TRNAK-UUU-9
5	85768742	85868742	0.423247	TRNAK-UUU-9
5	85768945	85868945	0.427932	TRNAK-UUU-9
5	85769058	85869058	0.412181	TRNAK-UUU-9
5	85769188	85869188	0.384156	TRNAK-UUU-9
5	85769208	85869208	0.386691	TRNAK-UUU-9
5	85769344	85869344	0.383215	TRNAK-UUU-9
5	85769424	85869424	0.391259	TRNAK-UUU-9
5	85769700	85869700	0.358073	TRNAK-UUU-9
5	85769960	85869960	0.341987	TRNAK-UUU-9
5	85769974	85869974	0.35091	TRNAK-UUU-9
5	85770458	85870458	0.347188	TRNAK-UUU-9
5	85770473	85870473	0.362361	TRNAK-UUU-9
5	85770508	85870508	0.370909	TRNAK-UUU-9
5	85770631	85870631	0.382878	TRNAK-UUU-9
5	85770700	85870700	0.392007	TRNAK-UUU-9
5	85770723	85870723	0.385086	TRNAK-UUU-9
5	85770792	85870792	0.382417	TRNAK-UUU-9
5	85770857	85870857	0.39316	TRNAK-UUU-9

5	85771002	85871002	0.390242	TRNAK-UUU-9
5	85771095	85871095	0.39072	TRNAK-UUU-9
5	85771142	85871142	0.394034	TRNAK-UUU-9
5	85771207	85871207	0.372474	TRNAK-UUU-9
5	85771215	85871215	0.359883	TRNAK-UUU-9
5	85771257	85871257	0.351843	TRNAK-UUU-9
5	85771295	85871295	0.350878	TRNAK-UUU-9
5	85771313	85871313	0.353901	TRNAK-UUU-9
5	85771346	85871346	0.354858	TRNAK-UUU-9
5	85771365	85871365	0.348809	TRNAK-UUU-9
5	85771407	85871407	0.345236	TRNAK-UUU-9
5	85772025	85872025	0.344929	TRNAK-UUU-9
5	85772221	85872221	0.347513	TRNAK-UUU-9
5	85772292	85872292	0.351588	TRNAK-UUU-9
5	85776053	85876053	0.343505	TRNAK-UUU-9
5	85777231	85877231	0.342194	TRNAK-UUU-9
5	85777546	85877546	0.350368	TRNAK-UUU-9
5	85777644	85877644	0.365479	TRNAK-UUU-9
5	85777920	85877920	0.378193	TRNAK-UUU-9
5	85778103	85878103	0.370673	TRNAK-UUU-9
5	85778104	85878104	0.347106	TRNAK-UUU-9
5	85778217	85878217	0.352121	TRNAK-UUU-9
5	85778429	85878429	0.352705	TRNAK-UUU-9
5	85778619	85878619	0.357581	TRNAK-UUU-9
5	85778648	85878648	0.356254	TRNAK-UUU-9
5	85778840	85878840	0.356189	TRNAK-UUU-9
5	85778924	85878924	0.354677	TRNAK-UUU-9
5	85779111	85879111	0.361175	TRNAK-UUU-9
5	85779118	85879118	0.362158	TRNAK-UUU-9
5	85779144	85879144	0.362158	TRNAK-UUU-9
5	85779261	85879261	0.360152	TRNAK-UUU-9
5	85779448	85879448	0.360262	TRNAK-UUU-9
5	85779505	85879505	0.36291	TRNAK-UUU-9
5	85779515	85879515	0.354076	TRNAK-UUU-9
5	85779593	85879593	0.354076	TRNAK-UUU-9
5	85779600	85879600	0.355071	TRNAK-UUU-9
5	85779726	85879726	0.359585	TRNAK-UUU-9
5	85779748	85879748	0.348649	TRNAK-UUU-9
5	85779808	85879808	0.36015	TRNAK-UUU-9
5	85780305	85880305	0.361428	TRNAK-UUU-9
5	85780349	85880349	0.356177	TRNAK-UUU-9
5	85780438	85880438	0.360603	TRNAK-UUU-9
5	85780664	85880664	0.367844	TRNAK-UUU-9
5	85780665	85880665	0.379323	TRNAK-UUU-9
5	85780853	85880853	0.448508	TRNAK-UUU-9
5	85780970	85880970	0.433344	TRNAK-UUU-9
5	85781003	85881003	0.431267	TRNAK-UUU-9
5	85781074	85881074	0.438838	TRNAK-UUU-9
5	85781102	85881102	0.419968	TRNAK-UUU-9
5	85781187	85881187	0.420531	TRNAK-UUU-9
5	85781265	85881265	0.416883	TRNAK-UUU-9
5	85781337	85881337	0.417229	TRNAK-UUU-9
5	85781338	85881338	0.417229	TRNAK-UUU-9
5	85781383	85881383	0.417162	TRNAK-UUU-9
5	85781649	85881649	0.403734	TRNAK-UUU-9
5	85782062	85882062	0.404017	TRNAK-UUU-9
5	85782146	85882146	0.404017	TRNAK-UUU-9
5	85782167	85882167	0.404017	TRNAK-UUU-9

5	85782201	85882201	0.404017	TRNAK-UUU-9
5	85782529	85882529	0.410898	TRNAK-UUU-9
5	85782586	85882586	0.405967	TRNAK-UUU-9
5	85782693	85882693	0.427974	TRNAK-UUU-9
5	85782770	85882770	0.39334	TRNAK-UUU-9
5	85782944	85882944	0.389398	TRNAK-UUU-9
5	85783005	85883005	0.389397	TRNAK-UUU-9
5	85783006	85883006	0.388636	TRNAK-UUU-9
5	85783088	85883088	0.382187	TRNAK-UUU-9
5	85783228	85883228	0.382187	TRNAK-UUU-9
5	85783321	85883321	0.385621	TRNAK-UUU-9
5	85783325	85883325	0.385621	TRNAK-UUU-9
5	85783492	85883492	0.382206	TRNAK-UUU-9
5	85783532	85883532	0.382601	TRNAK-UUU-9
5	85783798	85883798	0.390623	TRNAK-UUU-9
5	85783824	85883824	0.372737	TRNAK-UUU-9
5	85783848	85883848	0.378708	TRNAK-UUU-9
5	85783869	85883869	0.378342	TRNAK-UUU-9
5	85783962	85883962	0.378342	TRNAK-UUU-9
5	85784039	85884039	0.378342	TRNAK-UUU-9
5	85784343	85884343	0.378342	TRNAK-UUU-9
5	85784871	85884871	0.362944	TRNAK-UUU-9
5	85784875	85884875	0.395469	TRNAK-UUU-9
5	85784889	85884889	0.397709	TRNAK-UUU-9
5	85784891	85884891	0.397709	TRNAK-UUU-9
5	85785097	85885097	0.400958	TRNAK-UUU-9
5	85785341	85885341	0.390097	TRNAK-UUU-9
5	85785644	85885644	0.376662	TRNAK-UUU-9
5	85785742	85885742	0.401432	TRNAK-UUU-9
5	85785745	85885745	0.414502	TRNAK-UUU-9
5	85785850	85885850	0.409705	TRNAK-UUU-9
5	85785917	85885917	0.405599	TRNAK-UUU-9
5	85786059	85886059	0.40625	TRNAK-UUU-9
5	85786081	85886081	0.410599	TRNAK-UUU-9
5	85786334	85886334	0.410599	TRNAK-UUU-9
5	85786385	85886385	0.407122	TRNAK-UUU-9
5	85786416	85886416	0.408536	TRNAK-UUU-9
5	85786540	85886540	0.40716	TRNAK-UUU-9
5	85786966	85886966	0.382054	TRNAK-UUU-9
5	85786976	85886976	0.386663	TRNAK-UUU-9
5	85786985	85886985	0.394532	TRNAK-UUU-9
5	85786986	85886986	0.393403	TRNAK-UUU-9
5	85786994	85886994	0.39317	TRNAK-UUU-9
5	85787001	85887001	0.392902	TRNAK-UUU-9
5	85787045	85887045	0.392902	TRNAK-UUU-9
5	85787130	85887130	0.38899	TRNAK-UUU-9
5	85787234	85887234	0.385682	TRNAK-UUU-9
5	85787249	85887249	0.38391	TRNAK-UUU-9
5	86027273	86127273	0.349215	ETNK1
5	86027465	86127465	0.358265	ETNK1
5	86032937	86132937	0.358361	ETNK1
5	86032945	86132945	0.348915	ETNK1
5	90171082	90271082	0.343755	TRNAW-CCA-28
5	90171082	90271082	0.343755	PIK3C2G
5	90171099	90271099	0.343662	TRNAW-CCA-28
5	90171099	90271099	0.343662	PIK3C2G
5	90171120	90271120	0.345369	TRNAW-CCA-28
5	90171120	90271120	0.345369	PIK3C2G

5	90199204	90299204	0.3483	PIK3C2G
5	90199608	90299608	0.355623	PIK3C2G
5	90199691	90299691	0.359138	PIK3C2G
5	90199789	90299789	0.345149	PIK3C2G
5	90199985	90299985	0.367293	PIK3C2G
5	90200192	90300192	0.346334	PIK3C2G
5	90200397	90300397	0.369062	PIK3C2G
5	90200487	90300487	0.368184	PIK3C2G
5	90200492	90300492	0.40028	PIK3C2G
5	90200558	90300558	0.372036	PIK3C2G
5	90233329	90333329	0.368409	PIK3C2G
5	90233421	90333421	0.357008	PIK3C2G
5	90233462	90333462	0.357008	PIK3C2G
5	92626106	92726106	0.361067	STRAP
5	92626106	92726106	0.361067	EPS8
5	92816225	92916225	0.352011	EPS8
5	92816262	92916262	0.359234	EPS8
5	92816275	92916275	0.3567	EPS8
5	92816406	92916406	0.35122	EPS8
5	92816613	92916613	0.34759	EPS8
5	92816699	92916699	0.348611	EPS8
5	92816709	92916709	0.351572	EPS8
5	92816749	92916749	0.361394	EPS8
5	92816830	92916830	0.361394	EPS8
5	92821810	92921810	0.354991	EPS8
5	92821815	92921815	0.354991	EPS8
5	92821877	92921877	0.346746	EPS8
5	92821963	92921963	0.347683	EPS8
5	92822213	92922213	0.342589	EPS8
5	92822913	92922913	0.348721	EPS8
5	92822945	92922945	0.348721	EPS8
5	92822986	92922986	0.347227	EPS8
5	92823001	92923001	0.352105	EPS8
5	92823061	92923061	0.346898	EPS8
5	92823067	92923067	0.346898	EPS8
5	92823568	92923568	0.347239	EPS8
5	92823600	92923600	0.356113	EPS8
5	92823659	92923659	0.358742	EPS8
5	92823863	92923863	0.359507	EPS8
5	92823877	92923877	0.34784	EPS8
5	92824075	92924075	0.346025	EPS8
5	92824323	92924323	0.346064	EPS8
5	92825084	92925084	0.346656	EPS8
5	92825152	92925152	0.350231	EPS8
5	92825261	92925261	0.353166	EPS8
5	92825343	92925343	0.354395	EPS8
5	92825388	92925388	0.361456	EPS8
5	92825434	92925434	0.357071	EPS8
5	92825502	92925502	0.360276	EPS8
5	92825518	92925518	0.360276	EPS8
5	92825594	92925594	0.37598	EPS8
5	92825744	92925744	0.35993	EPS8
5	92825797	92925797	0.35779	EPS8
5	92825847	92925847	0.355992	EPS8
5	92826004	92926004	0.357552	EPS8
5	92826664	92926664	0.349017	EPS8
5	92826709	92926709	0.350491	EPS8
5	92826772	92926772	0.349105	EPS8

5	92827061	92927061	0.347574	EPS8
5	92827140	92927140	0.355991	EPS8
5	92827252	92927252	0.354527	EPS8
5	92827337	92927337	0.354527	EPS8
5	92827343	92927343	0.354527	EPS8
5	92827524	92927524	0.355475	EPS8
5	92827585	92927585	0.355475	EPS8
5	92827624	92927624	0.357307	EPS8
5	92827706	92927706	0.357307	EPS8
5	92827874	92927874	0.356064	EPS8
5	92827913	92927913	0.362021	EPS8
5	92827929	92927929	0.361444	EPS8
5	92827974	92927974	0.361759	EPS8
5	92827998	92927998	0.361759	EPS8
5	92828007	92928007	0.361091	EPS8
5	92828021	92928021	0.35958	EPS8
5	92828186	92928186	0.372676	EPS8
5	92828394	92928394	0.372815	EPS8
5	92828396	92928396	0.374549	EPS8
5	92828428	92928428	0.367223	EPS8
5	92828661	92928661	0.366002	EPS8
5	92828708	92928708	0.361778	EPS8
5	92828809	92928809	0.359193	EPS8
5	92829116	92929116	0.364037	EPS8
5	92829149	92929149	0.360694	EPS8
5	92829306	92929306	0.362781	EPS8
5	92829427	92929427	0.375566	EPS8
5	92829629	92929629	0.370182	EPS8
5	92829630	92929630	0.370182	EPS8
5	92829980	92929980	0.367824	EPS8
5	92830013	92930013	0.371525	EPS8
5	92830055	92930055	0.364115	EPS8
5	92830355	92930355	0.363529	EPS8
5	92830468	92930468	0.379927	EPS8
5	92830553	92930553	0.385124	EPS8
5	92830672	92930672	0.377304	EPS8
5	92830761	92930761	0.365528	EPS8
5	92831015	92931015	0.367655	EPS8
5	92831070	92931070	0.361679	EPS8
5	92831082	92931082	0.354352	EPS8
5	92831146	92931146	0.356016	EPS8
5	92831252	92931252	0.355957	EPS8
5	92831262	92931262	0.359174	EPS8
5	92831425	92931425	0.37509	EPS8
5	92831482	92931482	0.362183	EPS8
5	92831571	92931571	0.354353	EPS8
5	92831661	92931661	0.350211	EPS8
5	92831985	92931985	0.354125	EPS8
5	92832110	92932110	0.35447	EPS8
5	92832176	92932176	0.35447	EPS8
5	92832197	92932197	0.355847	EPS8
5	92834786	92934786	0.343527	EPS8
5	92838574	92938574	0.346929	EPS8
5	92838623	92938623	0.353304	EPS8
5	92838626	92938626	0.35281	EPS8
5	92838640	92938640	0.350188	EPS8
5	92839741	92939741	0.3447	EPS8
5	92839782	92939782	0.419012	EPS8

5	92839964	92939964	0.350527	EPS8
5	92840459	92940459	0.346378	EPS8
5	92842530	92942530	0.342754	EPS8
5	92844484	92944484	0.344404	EPS8
5	92844484	92944484	0.344404	PTPRO
5	92844712	92944712	0.351574	EPS8
5	92844712	92944712	0.351574	PTPRO
5	92844751	92944751	0.348759	EPS8
5	92844751	92944751	0.348759	PTPRO
5	92844822	92944822	0.350347	EPS8
5	92844822	92944822	0.350347	PTPRO
5	92844963	92944963	0.348291	EPS8
5	92844963	92944963	0.348291	PTPRO
5	92845001	92945001	0.349118	EPS8
5	92845001	92945001	0.349118	PTPRO
5	92845002	92945002	0.349118	EPS8
5	92845002	92945002	0.349118	PTPRO
5	92845137	92945137	0.352247	EPS8
5	92845137	92945137	0.352247	PTPRO
5	92845161	92945161	0.352066	EPS8
5	92845161	92945161	0.352066	PTPRO
5	92845177	92945177	0.344511	EPS8
5	92845177	92945177	0.344511	PTPRO
5	92845180	92945180	0.344511	EPS8
5	92845180	92945180	0.344511	PTPRO
5	92845203	92945203	0.348393	EPS8
5	92845203	92945203	0.348393	PTPRO
5	92845381	92945381	0.350662	EPS8
5	92845381	92945381	0.350662	PTPRO
5	92845417	92945417	0.350741	EPS8
5	92845417	92945417	0.350741	PTPRO
5	92845422	92945422	0.352648	EPS8
5	92845422	92945422	0.352648	PTPRO
5	92845479	92945479	0.342113	EPS8
5	92845479	92945479	0.342113	PTPRO
5	92846610	92946610	0.341869	EPS8
5	92846610	92946610	0.341869	PTPRO
5	92846890	92946890	0.342395	EPS8
5	92846890	92946890	0.342395	PTPRO
5	92846911	92946911	0.343683	EPS8
5	92846911	92946911	0.343683	PTPRO
5	92848122	92948122	0.348117	EPS8
5	92848122	92948122	0.348117	PTPRO
5	92848129	92948129	0.350753	EPS8
5	92848129	92948129	0.350753	PTPRO
5	92848323	92948323	0.349745	EPS8
5	92848323	92948323	0.349745	PTPRO
5	92848337	92948337	0.35502	EPS8
5	92848337	92948337	0.35502	PTPRO
5	92848353	92948353	0.355696	EPS8
5	92848353	92948353	0.355696	PTPRO
5	92848392	92948392	0.352091	EPS8
5	92848392	92948392	0.352091	PTPRO
5	92848638	92948638	0.352963	EPS8
5	92848638	92948638	0.352963	PTPRO
5	92848666	92948666	0.35251	EPS8
5	92848666	92948666	0.35251	PTPRO
5	92848732	92948732	0.35251	EPS8

5	92848732	92948732	0.35251	PTPRO
5	92848753	92948753	0.357532	EPS8
5	92848753	92948753	0.357532	PTPRO
5	92848757	92948757	0.356505	EPS8
5	92848757	92948757	0.356505	PTPRO
5	92848783	92948783	0.356443	EPS8
5	92848783	92948783	0.356443	PTPRO
5	92848785	92948785	0.35669	EPS8
5	92848785	92948785	0.35669	PTPRO
5	92848791	92948791	0.35669	EPS8
5	92848791	92948791	0.35669	PTPRO
5	92848794	92948794	0.35669	EPS8
5	92848794	92948794	0.35669	PTPRO
5	92848801	92948801	0.35669	EPS8
5	92848801	92948801	0.35669	PTPRO
5	92849038	92949038	0.356759	EPS8
5	92849038	92949038	0.356759	PTPRO
5	92849093	92949093	0.356938	EPS8
5	92849093	92949093	0.356938	PTPRO
5	92849184	92949184	0.357936	EPS8
5	92849184	92949184	0.357936	PTPRO
5	92849192	92949192	0.355895	EPS8
5	92849192	92949192	0.355895	PTPRO
5	92849204	92949204	0.358237	EPS8
5	92849204	92949204	0.358237	PTPRO
5	92849309	92949309	0.354455	EPS8
5	92849309	92949309	0.354455	PTPRO
5	92849392	92949392	0.353276	EPS8
5	92849392	92949392	0.353276	PTPRO
5	92849432	92949432	0.354339	EPS8
5	92849432	92949432	0.354339	PTPRO
5	92849454	92949454	0.345863	EPS8
5	92849454	92949454	0.345863	PTPRO
5	92849466	92949466	0.345341	EPS8
5	92849466	92949466	0.345341	PTPRO
5	92850087	92950087	0.360676	EPS8
5	92850087	92950087	0.360676	PTPRO
5	92850131	92950131	0.359521	EPS8
5	92850131	92950131	0.359521	PTPRO
5	92850644	92950644	0.352871	EPS8
5	92850644	92950644	0.352871	PTPRO
5	92850788	92950788	0.380568	EPS8
5	92850788	92950788	0.380568	PTPRO
5	92850848	92950848	0.371381	EPS8
5	92850848	92950848	0.371381	PTPRO
5	92850924	92950924	0.371113	EPS8
5	92850924	92950924	0.371113	PTPRO
5	92851139	92951139	0.370728	EPS8
5	92851139	92951139	0.370728	PTPRO
5	92851191	92951191	0.370756	EPS8
5	92851191	92951191	0.370756	PTPRO
5	92851366	92951366	0.394154	EPS8
5	92851366	92951366	0.394154	PTPRO
5	92851470	92951470	0.361929	EPS8
5	92851470	92951470	0.361929	PTPRO
5	92851591	92951591	0.389297	EPS8
5	92851591	92951591	0.389297	PTPRO
5	92851609	92951609	0.38645	EPS8

5	92851609	92951609	0.38645	PTPRO
5	92851652	92951652	0.376732	EPS8
5	92851652	92951652	0.376732	PTPRO
5	92851728	92951728	0.377932	EPS8
5	92851728	92951728	0.377932	PTPRO
5	92852656	92952656	0.382621	EPS8
5	92852656	92952656	0.382621	PTPRO
5	92852798	92952798	0.393671	EPS8
5	92852798	92952798	0.393671	PTPRO
5	92852861	92952861	0.393929	EPS8
5	92852861	92952861	0.393929	PTPRO
5	92852918	92952918	0.396662	EPS8
5	92852918	92952918	0.396662	PTPRO
5	92852952	92952952	0.397161	EPS8
5	92852952	92952952	0.397161	PTPRO
5	92853087	92953087	0.39842	EPS8
5	92853087	92953087	0.39842	PTPRO
5	92853191	92953191	0.39816	EPS8
5	92853191	92953191	0.39816	PTPRO
5	92853348	92953348	0.393506	EPS8
5	92853348	92953348	0.393506	PTPRO
5	92853527	92953527	0.402913	EPS8
5	92853527	92953527	0.402913	PTPRO
5	92853566	92953566	0.403933	EPS8
5	92853566	92953566	0.403933	PTPRO
5	92853585	92953585	0.405172	EPS8
5	92853585	92953585	0.405172	PTPRO
5	92853592	92953592	0.4104	EPS8
5	92853592	92953592	0.4104	PTPRO
5	92853593	92953593	0.4104	EPS8
5	92853593	92953593	0.4104	PTPRO
5	92853647	92953647	0.407962	EPS8
5	92853647	92953647	0.407962	PTPRO
5	92853723	92953723	0.408567	EPS8
5	92853723	92953723	0.408567	PTPRO
5	92853737	92953737	0.408567	EPS8
5	92853737	92953737	0.408567	PTPRO
5	92853758	92953758	0.40532	EPS8
5	92853758	92953758	0.40532	PTPRO
5	92853771	92953771	0.406678	EPS8
5	92853771	92953771	0.406678	PTPRO
5	92854126	92954126	0.399006	EPS8
5	92854126	92954126	0.399006	PTPRO
5	92854418	92954418	0.398436	EPS8
5	92854418	92954418	0.398436	PTPRO
5	92854428	92954428	0.398636	EPS8
5	92854428	92954428	0.398636	PTPRO
5	92854476	92954476	0.404487	EPS8
5	92854476	92954476	0.404487	PTPRO
5	92854537	92954537	0.406454	EPS8
5	92854537	92954537	0.406454	PTPRO
5	92854640	92954640	0.406822	EPS8
5	92854640	92954640	0.406822	PTPRO
5	92854868	92954868	0.411515	EPS8
5	92854868	92954868	0.411515	PTPRO
5	92854905	92954905	0.410069	EPS8
5	92854905	92954905	0.410069	PTPRO
5	92854959	92954959	0.407142	EPS8

5	92854959	92954959	0.407142	PTPRO
5	92855074	92955074	0.410196	EPS8
5	92855074	92955074	0.410196	PTPRO
5	92855135	92955135	0.410742	EPS8
5	92855135	92955135	0.410742	PTPRO
5	92855161	92955161	0.406413	EPS8
5	92855161	92955161	0.406413	PTPRO
5	92855168	92955168	0.406413	EPS8
5	92855168	92955168	0.406413	PTPRO
5	92855218	92955218	0.406413	EPS8
5	92855218	92955218	0.406413	PTPRO
5	92855444	92955444	0.400821	EPS8
5	92855444	92955444	0.400821	PTPRO
5	92855454	92955454	0.40089	EPS8
5	92855454	92955454	0.40089	PTPRO
5	92855499	92955499	0.40114	EPS8
5	92855499	92955499	0.40114	PTPRO
5	92855526	92955526	0.398129	EPS8
5	92855526	92955526	0.398129	PTPRO
5	92855554	92955554	0.401488	EPS8
5	92855554	92955554	0.401488	PTPRO
5	92855628	92955628	0.401488	EPS8
5	92855628	92955628	0.401488	PTPRO
5	92855684	92955684	0.407444	EPS8
5	92855684	92955684	0.407444	PTPRO
5	92855763	92955763	0.40335	EPS8
5	92855763	92955763	0.40335	PTPRO
5	92855890	92955890	0.403917	EPS8
5	92855890	92955890	0.403917	PTPRO
5	92855935	92955935	0.401531	EPS8
5	92855935	92955935	0.401531	PTPRO
5	92856144	92956144	0.404241	EPS8
5	92856144	92956144	0.404241	PTPRO
5	92856334	92956334	0.39293	EPS8
5	92856334	92956334	0.39293	PTPRO
5	92856671	92956671	0.40073	EPS8
5	92856671	92956671	0.40073	PTPRO
5	92856823	92956823	0.402723	EPS8
5	92856823	92956823	0.402723	PTPRO
5	92856964	92956964	0.425104	EPS8
5	92856964	92956964	0.425104	PTPRO
5	92857108	92957108	0.417849	EPS8
5	92857108	92957108	0.417849	PTPRO
5	92857148	92957148	0.425722	EPS8
5	92857148	92957148	0.425722	PTPRO
5	92857460	92957460	0.42167	EPS8
5	92857460	92957460	0.42167	PTPRO
5	92857465	92957465	0.427027	EPS8
5	92857465	92957465	0.427027	PTPRO
5	92857502	92957502	0.433048	EPS8
5	92857502	92957502	0.433048	PTPRO
5	92857545	92957545	0.432087	EPS8
5	92857545	92957545	0.432087	PTPRO
5	92857549	92957549	0.433071	EPS8
5	92857549	92957549	0.433071	PTPRO
5	92857594	92957594	0.433386	EPS8
5	92857594	92957594	0.433386	PTPRO
5	92857618	92957618	0.433429	EPS8

5	92857618	92957618	0.433429	PTPRO
5	92857653	92957653	0.431847	EPS8
5	92857653	92957653	0.431847	PTPRO
5	92857866	92957866	0.432314	EPS8
5	92857866	92957866	0.432314	PTPRO
5	92857879	92957879	0.434743	EPS8
5	92857879	92957879	0.434743	PTPRO
5	92857893	92957893	0.434743	EPS8
5	92857893	92957893	0.434743	PTPRO
5	92857903	92957903	0.439572	EPS8
5	92857903	92957903	0.439572	PTPRO
5	92857929	92957929	0.438986	EPS8
5	92857929	92957929	0.438986	PTPRO
5	92857947	92957947	0.438986	EPS8
5	92857947	92957947	0.438986	PTPRO
5	92857948	92957948	0.438259	EPS8
5	92857948	92957948	0.438259	PTPRO
5	92857952	92957952	0.436977	EPS8
5	92857952	92957952	0.436977	PTPRO
5	92857963	92957963	0.436977	EPS8
5	92857963	92957963	0.436977	PTPRO
5	92857981	92957981	0.437794	EPS8
5	92857981	92957981	0.437794	PTPRO
5	92858000	92958000	0.435357	EPS8
5	92858000	92958000	0.435357	PTPRO
5	92858009	92958009	0.434312	EPS8
5	92858009	92958009	0.434312	PTPRO
5	92858089	92958089	0.434278	EPS8
5	92858089	92958089	0.434278	PTPRO
5	92858101	92958101	0.434634	EPS8
5	92858101	92958101	0.434634	PTPRO
5	92858160	92958160	0.43206	EPS8
5	92858160	92958160	0.43206	PTPRO
5	92858170	92958170	0.432043	EPS8
5	92858170	92958170	0.432043	PTPRO
5	92858321	92958321	0.434124	EPS8
5	92858321	92958321	0.434124	PTPRO
5	92858362	92958362	0.445711	EPS8
5	92858362	92958362	0.445711	PTPRO
5	92858429	92958429	0.44468	EPS8
5	92858429	92958429	0.44468	PTPRO
5	92858516	92958516	0.442529	EPS8
5	92858516	92958516	0.442529	PTPRO
5	92858536	92958536	0.449386	EPS8
5	92858536	92958536	0.449386	PTPRO
5	92858572	92958572	0.448086	EPS8
5	92858572	92958572	0.448086	PTPRO
5	92858648	92958648	0.448427	EPS8
5	92858648	92958648	0.448427	PTPRO
5	92858804	92958804	0.468798	EPS8
5	92858804	92958804	0.468798	PTPRO
5	92858810	92958810	0.468798	EPS8
5	92858810	92958810	0.468798	PTPRO
5	92858904	92958904	0.453694	EPS8
5	92858904	92958904	0.453694	PTPRO
5	92858923	92958923	0.456866	EPS8
5	92858923	92958923	0.456866	PTPRO
5	92859060	92959060	0.455165	EPS8

5	92859060	92959060	0.455165	PTPRO
5	92859063	92959063	0.45452	EPS8
5	92859063	92959063	0.45452	PTPRO
5	92859214	92959214	0.453697	EPS8
5	92859214	92959214	0.453697	PTPRO
5	92859222	92959222	0.454644	EPS8
5	92859222	92959222	0.454644	PTPRO
5	92859255	92959255	0.455496	EPS8
5	92859255	92959255	0.455496	PTPRO
5	92859272	92959272	0.455905	EPS8
5	92859272	92959272	0.455905	PTPRO
5	92859354	92959354	0.451428	EPS8
5	92859354	92959354	0.451428	PTPRO
5	92859469	92959469	0.449712	EPS8
5	92859469	92959469	0.449712	PTPRO
5	92859628	92959628	0.443918	EPS8
5	92859628	92959628	0.443918	PTPRO
5	92859658	92959658	0.448828	EPS8
5	92859658	92959658	0.448828	PTPRO
5	92859769	92959769	0.455161	EPS8
5	92859769	92959769	0.455161	PTPRO
5	92859852	92959852	0.468684	EPS8
5	92859852	92959852	0.468684	PTPRO
5	92859863	92959863	0.463586	EPS8
5	92859863	92959863	0.463586	PTPRO
5	92859868	92959868	0.46511	EPS8
5	92859868	92959868	0.46511	PTPRO
5	92859919	92959919	0.463109	EPS8
5	92859919	92959919	0.463109	PTPRO
5	92859996	92959996	0.460855	EPS8
5	92859996	92959996	0.460855	PTPRO
5	92859999	92959999	0.461355	EPS8
5	92859999	92959999	0.461355	PTPRO
5	92860192	92960192	0.458766	EPS8
5	92860192	92960192	0.458766	PTPRO
5	92860301	92960301	0.465143	EPS8
5	92860301	92960301	0.465143	PTPRO
5	92860338	92960338	0.467128	EPS8
5	92860338	92960338	0.467128	PTPRO
5	92860358	92960358	0.460504	EPS8
5	92860358	92960358	0.460504	PTPRO
5	92860372	92960372	0.460106	EPS8
5	92860372	92960372	0.460106	PTPRO
5	92860405	92960405	0.460083	EPS8
5	92860405	92960405	0.460083	PTPRO
5	92860461	92960461	0.445953	EPS8
5	92860461	92960461	0.445953	PTPRO
5	92860484	92960484	0.49039	EPS8
5	92860484	92960484	0.49039	PTPRO
5	92886594	92986594	0.342663	EPS8
5	92886594	92986594	0.342663	PTPRO
5	92888356	92988356	0.344801	EPS8
5	92888356	92988356	0.344801	PTPRO
5	92888390	92988390	0.346109	EPS8
5	92888390	92988390	0.346109	PTPRO
5	92888509	92988509	0.358735	EPS8
5	92888509	92988509	0.358735	PTPRO
5	92888632	92988632	0.407479	EPS8

5	92888632	92988632	0.407479	PTPRO
5	92888633	92988633	0.411373	EPS8
5	92888633	92988633	0.411373	PTPRO
5	92888643	92988643	0.411373	EPS8
5	92888643	92988643	0.411373	PTPRO
5	92888673	92988673	0.413218	EPS8
5	92888673	92988673	0.413218	PTPRO
5	92888686	92988686	0.415173	EPS8
5	92888686	92988686	0.415173	PTPRO
5	92888714	92988714	0.401663	EPS8
5	92888714	92988714	0.401663	PTPRO
5	92888731	92988731	0.392498	EPS8
5	92888731	92988731	0.392498	PTPRO
5	92888813	92988813	0.39092	EPS8
5	92888813	92988813	0.39092	PTPRO
5	92888983	92988983	0.39092	EPS8
5	92888983	92988983	0.39092	PTPRO
5	92889013	92989013	0.385915	EPS8
5	92889013	92989013	0.385915	PTPRO
5	92889218	92989218	0.406965	EPS8
5	92889218	92989218	0.406965	PTPRO
5	92889343	92989343	0.402568	EPS8
5	92889343	92989343	0.402568	PTPRO
5	92889835	92989835	0.390311	EPS8
5	92889835	92989835	0.390311	PTPRO
5	92890000	92990000	0.388198	EPS8
5	92890000	92990000	0.388198	PTPRO
5	92890160	92990160	0.388549	EPS8
5	92890160	92990160	0.388549	PTPRO
5	92890221	92990221	0.407643	EPS8
5	92890221	92990221	0.407643	PTPRO
5	92890452	92990452	0.403784	EPS8
5	92890452	92990452	0.403784	PTPRO
5	92890502	92990502	0.412495	EPS8
5	92890502	92990502	0.412495	PTPRO
5	92890554	92990554	0.421153	EPS8
5	92890554	92990554	0.421153	PTPRO
5	92890563	92990563	0.41926	EPS8
5	92890563	92990563	0.41926	PTPRO
5	92890572	92990572	0.411327	EPS8
5	92890572	92990572	0.411327	PTPRO
5	92890625	92990625	0.420212	EPS8
5	92890625	92990625	0.420212	PTPRO
5	92890636	92990636	0.406593	EPS8
5	92890636	92990636	0.406593	PTPRO
5	92890693	92990693	0.408	EPS8
5	92890693	92990693	0.408	PTPRO
5	92890725	92990725	0.408322	EPS8
5	92890725	92990725	0.408322	PTPRO
5	92890726	92990726	0.408322	EPS8
5	92890726	92990726	0.408322	PTPRO
5	92890809	92990809	0.407857	EPS8
5	92890809	92990809	0.407857	PTPRO
5	92890840	92990840	0.394327	EPS8
5	92890840	92990840	0.394327	PTPRO
5	92890900	92990900	0.394327	EPS8
5	92890900	92990900	0.394327	PTPRO
5	92891241	92991241	0.413333	EPS8

5	92891241	92991241	0.413333	PTPRO
5	92891342	92991342	0.428693	EPS8
5	92891342	92991342	0.428693	PTPRO
5	92891537	92991537	0.435036	EPS8
5	92891537	92991537	0.435036	PTPRO
5	92891544	92991544	0.432544	EPS8
5	92891544	92991544	0.432544	PTPRO
5	92891565	92991565	0.443517	EPS8
5	92891565	92991565	0.443517	PTPRO
5	92891566	92991566	0.443517	EPS8
5	92891566	92991566	0.443517	PTPRO
5	92891570	92991570	0.443831	EPS8
5	92891570	92991570	0.443831	PTPRO
5	92891579	92991579	0.424841	EPS8
5	92891579	92991579	0.424841	PTPRO
5	92891596	92991596	0.361392	EPS8
5	92891596	92991596	0.361392	PTPRO
5	92891606	92991606	0.358024	EPS8
5	92891606	92991606	0.358024	PTPRO
5	92892035	92992035	0.369895	EPS8
5	92892035	92992035	0.369895	PTPRO
5	92896121	92996121	0.347537	EPS8
5	92896121	92996121	0.347537	PTPRO
5	92899835	92999835	0.342398	EPS8
5	92899835	92999835	0.342398	PTPRO
5	92900074	93000074	0.347076	EPS8
5	92900074	93000074	0.347076	PTPRO
5	92900265	93000265	0.348936	EPS8
5	92900265	93000265	0.348936	PTPRO
5	92900351	93000351	0.356499	EPS8
5	92900351	93000351	0.356499	PTPRO
5	92900359	93000359	0.354955	EPS8
5	92900359	93000359	0.354955	PTPRO
5	92900557	93000557	0.353973	EPS8
5	92900557	93000557	0.353973	PTPRO
5	92900700	93000700	0.356986	EPS8
5	92900700	93000700	0.356986	PTPRO
5	92900701	93000701	0.356876	EPS8
5	92900701	93000701	0.356876	PTPRO
5	92900741	93000741	0.341769	EPS8
5	92900741	93000741	0.341769	PTPRO
5	92901880	93001880	0.346044	EPS8
5	92901880	93001880	0.346044	PTPRO
5	92901959	93001959	0.346044	EPS8
5	92901959	93001959	0.346044	PTPRO
5	92902065	93002065	0.359922	EPS8
5	92902065	93002065	0.359922	PTPRO
5	92902067	93002067	0.348058	EPS8
5	92902067	93002067	0.348058	PTPRO
5	92902100	93002100	0.342018	EPS8
5	92902100	93002100	0.342018	PTPRO
5	92902587	93002587	0.355234	EPS8
5	92902587	93002587	0.355234	PTPRO
5	92902719	93002719	0.355791	EPS8
5	92902719	93002719	0.355791	PTPRO
5	92902824	93002824	0.3652	EPS8
5	92902824	93002824	0.3652	PTPRO
5	92902872	93002872	0.375048	EPS8

5	92902872	93002872	0.375048	PTPRO
5	92902965	93002965	0.344389	EPS8
5	92902965	93002965	0.344389	PTPRO
5	92903217	93003217	0.344389	EPS8
5	92903217	93003217	0.344389	PTPRO
5	92903273	93003273	0.344389	EPS8
5	92903273	93003273	0.344389	PTPRO
5	92903311	93003311	0.344389	EPS8
5	92903311	93003311	0.344389	PTPRO
5	92903360	93003360	0.350022	EPS8
5	92903360	93003360	0.350022	PTPRO
5	92903560	93003560	0.349511	EPS8
5	92903560	93003560	0.349511	PTPRO
5	92903591	93003591	0.349511	EPS8
5	92903591	93003591	0.349511	PTPRO
5	92903622	93003622	0.349795	EPS8
5	92903622	93003622	0.349795	PTPRO
5	92903623	93003623	0.34927	EPS8
5	92903623	93003623	0.34927	PTPRO
5	92903674	93003674	0.34927	EPS8
5	92903674	93003674	0.34927	PTPRO
5	92903695	93003695	0.34927	EPS8
5	92903695	93003695	0.34927	PTPRO
5	92903875	93003875	0.350728	EPS8
5	92903875	93003875	0.350728	PTPRO
5	92903905	93003905	0.365694	EPS8
5	92903905	93003905	0.365694	PTPRO
5	92903947	93003947	0.358885	EPS8
5	92903947	93003947	0.358885	PTPRO
5	92904016	93004016	0.359058	EPS8
5	92904016	93004016	0.359058	PTPRO
5	92904017	93004017	0.359058	EPS8
5	92904017	93004017	0.359058	PTPRO
5	92904083	93004083	0.359219	EPS8
5	92904083	93004083	0.359219	PTPRO
5	92904194	93004194	0.359293	EPS8
5	92904194	93004194	0.359293	PTPRO
5	92904243	93004243	0.367282	EPS8
5	92904243	93004243	0.367282	PTPRO
5	92904311	93004311	0.367055	EPS8
5	92904311	93004311	0.367055	PTPRO
5	92904324	93004324	0.367055	EPS8
5	92904324	93004324	0.367055	PTPRO
5	92904328	93004328	0.367055	EPS8
5	92904328	93004328	0.367055	PTPRO
5	92904340	93004340	0.367055	EPS8
5	92904340	93004340	0.367055	PTPRO
5	92904362	93004362	0.367055	EPS8
5	92904362	93004362	0.367055	PTPRO
5	92904377	93004377	0.367055	EPS8
5	92904377	93004377	0.367055	PTPRO
5	92904419	93004419	0.37038	EPS8
5	92904419	93004419	0.37038	PTPRO
5	92904450	93004450	0.401093	EPS8
5	92904450	93004450	0.401093	PTPRO
5	92904503	93004503	0.408886	EPS8
5	92904503	93004503	0.408886	PTPRO
5	92904552	93004552	0.408146	EPS8

5	92904552	93004552	0.408146	PTPRO
5	92904557	93004557	0.408399	EPS8
5	92904557	93004557	0.408399	PTPRO
5	92904582	93004582	0.405244	EPS8
5	92904582	93004582	0.405244	PTPRO
5	92904628	93004628	0.40681	EPS8
5	92904628	93004628	0.40681	PTPRO
5	92904827	93004827	0.390782	EPS8
5	92904827	93004827	0.390782	PTPRO
5	92905029	93005029	0.392191	EPS8
5	92905029	93005029	0.392191	PTPRO
5	92905048	93005048	0.39989	EPS8
5	92905048	93005048	0.39989	PTPRO
5	92905108	93005108	0.398126	EPS8
5	92905108	93005108	0.398126	PTPRO
5	92905206	93005206	0.405313	EPS8
5	92905206	93005206	0.405313	PTPRO
5	92905289	93005289	0.405094	EPS8
5	92905289	93005289	0.405094	PTPRO
5	92905324	93005324	0.425508	EPS8
5	92905324	93005324	0.425508	PTPRO
5	92905423	93005423	0.424744	EPS8
5	92905423	93005423	0.424744	PTPRO
5	92905473	93005473	0.424013	EPS8
5	92905473	93005473	0.424013	PTPRO
5	92905494	93005494	0.424437	EPS8
5	92905494	93005494	0.424437	PTPRO
5	92905539	93005539	0.421985	EPS8
5	92905539	93005539	0.421985	PTPRO
5	92905642	93005642	0.419427	EPS8
5	92905642	93005642	0.419427	PTPRO
5	92905797	93005797	0.394252	EPS8
5	92905797	93005797	0.394252	PTPRO
5	92905869	93005869	0.403707	EPS8
5	92905869	93005869	0.403707	PTPRO
5	92905943	93005943	0.40567	EPS8
5	92905943	93005943	0.40567	PTPRO
5	92906018	93006018	0.39837	EPS8
5	92906018	93006018	0.39837	PTPRO
5	92906056	93006056	0.413201	EPS8
5	92906056	93006056	0.413201	PTPRO
5	92906057	93006057	0.413201	EPS8
5	92906057	93006057	0.413201	PTPRO
5	92906133	93006133	0.427904	EPS8
5	92906133	93006133	0.427904	PTPRO
5	92906163	93006163	0.426155	EPS8
5	92906163	93006163	0.426155	PTPRO
5	92906170	93006170	0.429043	EPS8
5	92906170	93006170	0.429043	PTPRO
5	92906207	93006207	0.415217	EPS8
5	92906207	93006207	0.415217	PTPRO
5	92906223	93006223	0.415755	EPS8
5	92906223	93006223	0.415755	PTPRO
5	92906270	93006270	0.417009	EPS8
5	92906270	93006270	0.417009	PTPRO
5	92906295	93006295	0.41772	EPS8
5	92906295	93006295	0.41772	PTPRO
5	92906314	93006314	0.416941	EPS8

5	92906314	93006314	0.416941	PTPRO
5	92906315	93006315	0.419651	EPS8
5	92906315	93006315	0.419651	PTPRO
5	92906328	93006328	0.412926	EPS8
5	92906328	93006328	0.412926	PTPRO
5	92906399	93006399	0.417296	EPS8
5	92906399	93006399	0.417296	PTPRO
5	92906557	93006557	0.409281	EPS8
5	92906557	93006557	0.409281	PTPRO
5	92906641	93006641	0.419951	EPS8
5	92906641	93006641	0.419951	PTPRO
5	92906655	93006655	0.450221	EPS8
5	92906655	93006655	0.450221	PTPRO
5	92906661	93006661	0.453447	EPS8
5	92906661	93006661	0.453447	PTPRO
5	92906697	93006697	0.455002	EPS8
5	92906697	93006697	0.455002	PTPRO
5	92906890	93006890	0.411167	EPS8
5	92906890	93006890	0.411167	PTPRO
5	92906933	93006933	0.408389	EPS8
5	92906933	93006933	0.408389	PTPRO
5	92906935	93006935	0.396219	EPS8
5	92906935	93006935	0.396219	PTPRO
5	92907340	93007340	0.398937	EPS8
5	92907340	93007340	0.398937	PTPRO
5	92907504	93007504	0.392003	EPS8
5	92907504	93007504	0.392003	PTPRO
5	92907632	93007632	0.380558	EPS8
5	92907632	93007632	0.380558	PTPRO
5	92907646	93007646	0.394552	EPS8
5	92907646	93007646	0.394552	PTPRO
5	92907738	93007738	0.394477	EPS8
5	92907738	93007738	0.394477	PTPRO
5	92907815	93007815	0.421245	EPS8
5	92907815	93007815	0.421245	PTPRO
5	92907969	93007969	0.402692	EPS8
5	92907969	93007969	0.402692	PTPRO
5	92908046	93008046	0.473062	EPS8
5	92908046	93008046	0.473062	PTPRO
5	92908050	93008050	0.473062	EPS8
5	92908050	93008050	0.473062	PTPRO
5	92908056	93008056	0.471721	EPS8
5	92908056	93008056	0.471721	PTPRO
5	92908058	93008058	0.46589	EPS8
5	92908058	93008058	0.46589	PTPRO
5	92908080	93008080	0.436676	EPS8
5	92908080	93008080	0.436676	PTPRO
5	92908098	93008098	0.425825	EPS8
5	92908098	93008098	0.425825	PTPRO
5	92908338	93008338	0.401796	EPS8
5	92908338	93008338	0.401796	PTPRO
5	92908357	93008357	0.401326	EPS8
5	92908357	93008357	0.401326	PTPRO
5	92908405	93008405	0.417508	EPS8
5	92908405	93008405	0.417508	PTPRO
5	92908445	93008445	0.396988	EPS8
5	92908445	93008445	0.396988	PTPRO
5	92908446	93008446	0.396988	EPS8

5	92908446	93008446	0.396988	PTPRO
5	92908454	93008454	0.396988	EPS8
5	92908454	93008454	0.396988	PTPRO
5	92908485	93008485	0.398764	EPS8
5	92908485	93008485	0.398764	PTPRO
5	92908528	93008528	0.400189	EPS8
5	92908528	93008528	0.400189	PTPRO
5	92908532	93008532	0.402388	EPS8
5	92908532	93008532	0.402388	PTPRO
5	92908538	93008538	0.400079	EPS8
5	92908538	93008538	0.400079	PTPRO
5	92908714	93008714	0.398167	EPS8
5	92908714	93008714	0.398167	PTPRO
5	92908759	93008759	0.396712	EPS8
5	92908759	93008759	0.396712	PTPRO
5	92908771	93008771	0.401305	EPS8
5	92908771	93008771	0.401305	PTPRO
5	92908832	93008832	0.400715	EPS8
5	92908832	93008832	0.400715	PTPRO
5	92908834	93008834	0.396975	EPS8
5	92908834	93008834	0.396975	PTPRO
5	92908856	93008856	0.398803	EPS8
5	92908856	93008856	0.398803	PTPRO
5	92908857	93008857	0.41167	EPS8
5	92908857	93008857	0.41167	PTPRO
5	92909042	93009042	0.413166	EPS8
5	92909042	93009042	0.413166	PTPRO
5	92909045	93009045	0.407016	EPS8
5	92909045	93009045	0.407016	PTPRO
5	92909071	93009071	0.404182	EPS8
5	92909071	93009071	0.404182	PTPRO
5	92909134	93009134	0.388695	EPS8
5	92909134	93009134	0.388695	PTPRO
5	92909241	93009241	0.397248	EPS8
5	92909241	93009241	0.397248	PTPRO
5	92909484	93009484	0.389851	EPS8
5	92909484	93009484	0.389851	PTPRO
5	92909566	93009566	0.402578	EPS8
5	92909566	93009566	0.402578	PTPRO
5	92909709	93009709	0.402413	EPS8
5	92909709	93009709	0.402413	PTPRO
5	92909950	93009950	0.390803	EPS8
5	92909950	93009950	0.390803	PTPRO
5	92910003	93010003	0.403271	EPS8
5	92910003	93010003	0.403271	PTPRO
5	92910027	93010027	0.404063	EPS8
5	92910027	93010027	0.404063	PTPRO
5	92910071	93010071	0.405335	EPS8
5	92910071	93010071	0.405335	PTPRO
5	92910119	93010119	0.398043	EPS8
5	92910119	93010119	0.398043	PTPRO
5	92910159	93010159	0.400498	EPS8
5	92910159	93010159	0.400498	PTPRO
5	92910273	93010273	0.405503	EPS8
5	92910273	93010273	0.405503	PTPRO
5	92910337	93010337	0.39158	EPS8
5	92910337	93010337	0.39158	PTPRO
5	92910358	93010358	0.390851	EPS8

5	92910358	93010358	0.390851	PTPRO
5	92910451	93010451	0.394391	EPS8
5	92910451	93010451	0.394391	PTPRO
5	92910458	93010458	0.395683	EPS8
5	92910458	93010458	0.395683	PTPRO
5	92910570	93010570	0.395098	EPS8
5	92910570	93010570	0.395098	PTPRO
5	92910800	93010800	0.393532	EPS8
5	92910800	93010800	0.393532	PTPRO
5	92910841	93010841	0.395381	EPS8
5	92910841	93010841	0.395381	PTPRO
5	92910846	93010846	0.401755	EPS8
5	92910846	93010846	0.401755	PTPRO
5	92910847	93010847	0.396442	EPS8
5	92910847	93010847	0.396442	PTPRO
5	92910894	93010894	0.411392	EPS8
5	92910894	93010894	0.411392	PTPRO
5	92910907	93010907	0.41214	EPS8
5	92910907	93010907	0.41214	PTPRO
5	92910952	93010952	0.416848	EPS8
5	92910952	93010952	0.416848	PTPRO
5	92911005	93011005	0.41534	EPS8
5	92911005	93011005	0.41534	PTPRO
5	92911192	93011192	0.401853	EPS8
5	92911192	93011192	0.401853	PTPRO
5	92911249	93011249	0.392331	EPS8
5	92911249	93011249	0.392331	PTPRO
5	92911636	93011636	0.381959	EPS8
5	92911636	93011636	0.381959	PTPRO
5	92911662	93011662	0.37593	EPS8
5	92911662	93011662	0.37593	PTPRO
5	92911669	93011669	0.378283	EPS8
5	92911669	93011669	0.378283	PTPRO
5	92911956	93011956	0.390373	EPS8
5	92911956	93011956	0.390373	PTPRO
5	92912019	93012019	0.364584	EPS8
5	92912019	93012019	0.364584	PTPRO
5	92912050	93012050	0.366063	EPS8
5	92912050	93012050	0.366063	PTPRO
5	92912181	93012181	0.367164	EPS8
5	92912181	93012181	0.367164	PTPRO
5	92912333	93012333	0.364775	EPS8
5	92912333	93012333	0.364775	PTPRO
5	92912394	93012394	0.366382	EPS8
5	92912394	93012394	0.366382	PTPRO
5	92912413	93012413	0.366382	EPS8
5	92912413	93012413	0.366382	PTPRO
5	92912473	93012473	0.36711	EPS8
5	92912473	93012473	0.36711	PTPRO
5	92912523	93012523	0.368904	EPS8
5	92912523	93012523	0.368904	PTPRO
5	92912531	93012531	0.364435	EPS8
5	92912531	93012531	0.364435	PTPRO
5	92912624	93012624	0.364435	EPS8
5	92912624	93012624	0.364435	PTPRO
5	92912677	93012677	0.362334	EPS8
5	92912677	93012677	0.362334	PTPRO
5	92913124	93013124	0.353519	EPS8

5	92913124	93013124	0.353519	PTPRO
5	92913203	93013203	0.353519	EPS8
5	92913203	93013203	0.353519	PTPRO
5	92913219	93013219	0.353519	EPS8
5	92913219	93013219	0.353519	PTPRO
5	92913318	93013318	0.363923	EPS8
5	92913318	93013318	0.363923	PTPRO
5	92913334	93013334	0.368121	EPS8
5	92913334	93013334	0.368121	PTPRO
5	92913349	93013349	0.37069	EPS8
5	92913349	93013349	0.37069	PTPRO
5	92913376	93013376	0.373941	EPS8
5	92913376	93013376	0.373941	PTPRO
5	92914011	93014011	0.400472	EPS8
5	92914011	93014011	0.400472	PTPRO
5	92914020	93014020	0.406433	EPS8
5	92914020	93014020	0.406433	PTPRO
5	92914101	93014101	0.401553	EPS8
5	92914101	93014101	0.401553	PTPRO
5	92914174	93014174	0.385765	EPS8
5	92914174	93014174	0.385765	PTPRO
5	92914250	93014250	0.399385	EPS8
5	92914250	93014250	0.399385	PTPRO
5	92914431	93014431	0.374918	EPS8
5	92914431	93014431	0.374918	PTPRO
5	92915040	93015040	0.342516	EPS8
5	92915040	93015040	0.342516	PTPRO
5	92915681	93015681	0.360642	EPS8
5	92915681	93015681	0.360642	PTPRO
5	92915848	93015848	0.360642	EPS8
5	92915848	93015848	0.360642	PTPRO
5	92916105	93016105	0.394647	EPS8
5	92916105	93016105	0.394647	PTPRO
5	92916397	93016397	0.39093	EPS8
5	92916397	93016397	0.39093	PTPRO
5	92916410	93016410	0.385506	EPS8
5	92916410	93016410	0.385506	PTPRO
5	92916541	93016541	0.387874	EPS8
5	92916541	93016541	0.387874	PTPRO
5	92916554	93016554	0.4061	EPS8
5	92916554	93016554	0.4061	PTPRO
5	92916558	93016558	0.398202	EPS8
5	92916558	93016558	0.398202	PTPRO
5	92916586	93016586	0.381855	EPS8
5	92916586	93016586	0.381855	PTPRO
5	92916587	93016587	0.381855	EPS8
5	92916587	93016587	0.381855	PTPRO
5	92916595	93016595	0.379598	EPS8
5	92916595	93016595	0.379598	PTPRO
5	92916638	93016638	0.381595	EPS8
5	92916638	93016638	0.381595	PTPRO
5	92916642	93016642	0.385148	EPS8
5	92916642	93016642	0.385148	PTPRO
5	92916749	93016749	0.379883	EPS8
5	92916749	93016749	0.379883	PTPRO
5	92916755	93016755	0.368223	EPS8
5	92916755	93016755	0.368223	PTPRO
5	92916921	93016921	0.367989	EPS8

5	92916921	93016921	0.367989	PTPRO
5	92917100	93017100	0.372291	EPS8
5	92917100	93017100	0.372291	PTPRO
5	92917174	93017174	0.3425	EPS8
5	92917174	93017174	0.3425	PTPRO
5	92917175	93017175	0.3425	EPS8
5	92917175	93017175	0.3425	PTPRO
5	92917234	93017234	0.3425	EPS8
5	92917234	93017234	0.3425	PTPRO
5	92918245	93018245	0.362863	EPS8
5	92918245	93018245	0.362863	PTPRO
5	92918679	93018679	0.358394	EPS8
5	92918679	93018679	0.358394	PTPRO
5	92919925	93019925	0.356758	EPS8
5	92919925	93019925	0.356758	PTPRO
5	92919967	93019967	0.372464	EPS8
5	92919967	93019967	0.372464	PTPRO
5	92920140	93020140	0.363475	EPS8
5	92920140	93020140	0.363475	PTPRO
5	92920163	93020163	0.363484	EPS8
5	92920163	93020163	0.363484	PTPRO
5	92920166	93020166	0.363561	EPS8
5	92920166	93020166	0.363561	PTPRO
5	92920180	93020180	0.363714	EPS8
5	92920180	93020180	0.363714	PTPRO
5	92920220	93020220	0.361822	EPS8
5	92920220	93020220	0.361822	PTPRO
5	92920550	93020550	0.376327	EPS8
5	92920550	93020550	0.376327	PTPRO
5	92920656	93020656	0.376432	EPS8
5	92920656	93020656	0.376432	PTPRO
5	92920675	93020675	0.391262	EPS8
5	92920675	93020675	0.391262	PTPRO
5	92920684	93020684	0.37998	EPS8
5	92920684	93020684	0.37998	PTPRO
5	92920703	93020703	0.382853	EPS8
5	92920703	93020703	0.382853	PTPRO
5	92920996	93020996	0.377736	EPS8
5	92920996	93020996	0.377736	PTPRO
5	92921074	93021074	0.384474	EPS8
5	92921074	93021074	0.384474	PTPRO
5	92921101	93021101	0.377237	EPS8
5	92921101	93021101	0.377237	PTPRO
5	92921163	93021163	0.384579	EPS8
5	92921163	93021163	0.384579	PTPRO
5	92921251	93021251	0.384579	EPS8
5	92921251	93021251	0.384579	PTPRO
5	92921541	93021541	0.371859	EPS8
5	92921541	93021541	0.371859	PTPRO
5	93085472	93185472	0.346456	PTPRO
5	93085529	93185529	0.389903	PTPRO
5	93085566	93185566	0.354983	PTPRO
5	93085567	93185567	0.354536	PTPRO
5	95666471	95766471	0.347058	APOLD1
5	95666471	95766471	0.347058	LOC102175559
5	95666471	95766471	0.347058	DDX47
5	95666471	95766471	0.347058	CDKN1B
5	95666608	95766608	0.352981	APOLD1

5	95666608	95766608	0.352981	LOC102175559
5	95666608	95766608	0.352981	DDX47
5	95666608	95766608	0.352981	CDKN1B
5	95666628	95766628	0.354251	APOLD1
5	95666628	95766628	0.354251	LOC102175559
5	95666628	95766628	0.354251	DDX47
5	95666628	95766628	0.354251	CDKN1B
5	95666681	95766681	0.354251	APOLD1
5	95666681	95766681	0.354251	LOC102175559
5	95666681	95766681	0.354251	DDX47
5	95666681	95766681	0.354251	CDKN1B
5	95666694	95766694	0.361873	APOLD1
5	95666694	95766694	0.361873	LOC102175559
5	95666694	95766694	0.361873	DDX47
5	95666694	95766694	0.361873	CDKN1B
5	95666836	95766836	0.382538	APOLD1
5	95666836	95766836	0.382538	LOC102175559
5	95666836	95766836	0.382538	DDX47
5	95666836	95766836	0.382538	CDKN1B
5	95667181	95767181	0.37749	APOLD1
5	95667181	95767181	0.37749	LOC102175559
5	95667181	95767181	0.37749	DDX47
5	95667181	95767181	0.37749	CDKN1B
5	95667299	95767299	0.383894	APOLD1
5	95667299	95767299	0.383894	LOC102175559
5	95667299	95767299	0.383894	DDX47
5	95667299	95767299	0.383894	CDKN1B
5	95667341	95767341	0.377439	APOLD1
5	95667341	95767341	0.377439	LOC102175559
5	95667341	95767341	0.377439	DDX47
5	95667341	95767341	0.377439	CDKN1B
5	95667676	95767676	0.387964	APOLD1
5	95667676	95767676	0.387964	LOC102175559
5	95667676	95767676	0.387964	DDX47
5	95667676	95767676	0.387964	CDKN1B
5	95667735	95767735	0.387964	APOLD1
5	95667735	95767735	0.387964	LOC102175559
5	95667735	95767735	0.387964	DDX47
5	95667735	95767735	0.387964	CDKN1B
5	95667881	95767881	0.397529	APOLD1
5	95667881	95767881	0.397529	LOC102175559
5	95667881	95767881	0.397529	DDX47
5	95667881	95767881	0.397529	CDKN1B
5	95668098	95768098	0.369969	APOLD1
5	95668098	95768098	0.369969	LOC102175559
5	95668098	95768098	0.369969	DDX47
5	95668098	95768098	0.369969	CDKN1B
5	95668098	95768098	0.369969	GPR19
5	95668182	95768182	0.369969	APOLD1
5	95668182	95768182	0.369969	LOC102175559
5	95668182	95768182	0.369969	DDX47
5	95668182	95768182	0.369969	CDKN1B
5	95668182	95768182	0.369969	GPR19
5	95668288	95768288	0.420591	APOLD1
5	95668288	95768288	0.420591	LOC102175559
5	95668288	95768288	0.420591	DDX47
5	95668288	95768288	0.420591	CDKN1B
5	95668288	95768288	0.420591	GPR19

5	95668579	95768579	0.382488	APOLD1
5	95668579	95768579	0.382488	LOC102175559
5	95668579	95768579	0.382488	DDX47
5	95668579	95768579	0.382488	CDKN1B
5	95668579	95768579	0.382488	GPR19
5	95668915	95768915	0.420104	APOLD1
5	95668915	95768915	0.420104	LOC102175559
5	95668915	95768915	0.420104	DDX47
5	95668915	95768915	0.420104	CDKN1B
5	95668915	95768915	0.420104	GPR19
5	95669146	95769146	0.408755	APOLD1
5	95669146	95769146	0.408755	LOC102175559
5	95669146	95769146	0.408755	DDX47
5	95669146	95769146	0.408755	CDKN1B
5	95669146	95769146	0.408755	GPR19
5	95669175	95769175	0.42089	APOLD1
5	95669175	95769175	0.42089	LOC102175559
5	95669175	95769175	0.42089	DDX47
5	95669175	95769175	0.42089	CDKN1B
5	95669175	95769175	0.42089	GPR19
5	95669297	95769297	0.398113	APOLD1
5	95669297	95769297	0.398113	LOC102175559
5	95669297	95769297	0.398113	DDX47
5	95669297	95769297	0.398113	CDKN1B
5	95669297	95769297	0.398113	GPR19
5	95669610	95769610	0.400737	APOLD1
5	95669610	95769610	0.400737	LOC102175559
5	95669610	95769610	0.400737	DDX47
5	95669610	95769610	0.400737	CDKN1B
5	95669610	95769610	0.400737	GPR19
5	95669888	95769888	0.400049	APOLD1
5	95669888	95769888	0.400049	LOC102175559
5	95669888	95769888	0.400049	CDKN1B
5	95669888	95769888	0.400049	GPR19
5	95670490	95770490	0.382877	APOLD1
5	95670490	95770490	0.382877	LOC102175559
5	95670490	95770490	0.382877	CDKN1B
5	95670490	95770490	0.382877	GPR19
5	95670506	95770506	0.382376	APOLD1
5	95670506	95770506	0.382376	LOC102175559
5	95670506	95770506	0.382376	CDKN1B
5	95670506	95770506	0.382376	GPR19
5	95670581	95770581	0.409001	APOLD1
5	95670581	95770581	0.409001	LOC102175559
5	95670581	95770581	0.409001	CDKN1B
5	95670581	95770581	0.409001	GPR19
5	95670776	95770776	0.395479	APOLD1
5	95670776	95770776	0.395479	LOC102175559
5	95670776	95770776	0.395479	CDKN1B
5	95670776	95770776	0.395479	GPR19
5	95670931	95770931	0.418931	APOLD1
5	95670931	95770931	0.418931	LOC102175559
5	95670931	95770931	0.418931	CDKN1B
5	95670931	95770931	0.418931	GPR19
5	95671238	95771238	0.41099	APOLD1
5	95671238	95771238	0.41099	LOC102175559
5	95671238	95771238	0.41099	CDKN1B
5	95671238	95771238	0.41099	GPR19

5	95671714	95771714	0.401693	APOLD1
5	95671714	95771714	0.401693	LOC102175559
5	95671714	95771714	0.401693	CDKN1B
5	95671714	95771714	0.401693	GPR19
5	95672096	95772096	0.400668	APOLD1
5	95672096	95772096	0.400668	LOC102175559
5	95672096	95772096	0.400668	CDKN1B
5	95672096	95772096	0.400668	GPR19
5	95672215	95772215	0.408908	APOLD1
5	95672215	95772215	0.408908	LOC102175559
5	95672215	95772215	0.408908	CDKN1B
5	95672215	95772215	0.408908	GPR19
5	95672437	95772437	0.410649	APOLD1
5	95672437	95772437	0.410649	LOC102175559
5	95672437	95772437	0.410649	CDKN1B
5	95672437	95772437	0.410649	GPR19
5	95672473	95772473	0.366965	APOLD1
5	95672473	95772473	0.366965	LOC102175559
5	95672473	95772473	0.366965	CDKN1B
5	95672473	95772473	0.366965	GPR19
5	95672493	95772493	0.3785	APOLD1
5	95672493	95772493	0.3785	LOC102175559
5	95672493	95772493	0.3785	CDKN1B
5	95672493	95772493	0.3785	GPR19
5	95672526	95772526	0.356838	APOLD1
5	95672526	95772526	0.356838	LOC102175559
5	95672526	95772526	0.356838	CDKN1B
5	95672526	95772526	0.356838	GPR19
5	95672766	95772766	0.347849	APOLD1
5	95672766	95772766	0.347849	LOC102175559
5	95672766	95772766	0.347849	CDKN1B
5	95672766	95772766	0.347849	GPR19
5	95676407	95776407	0.368876	APOLD1
5	95676407	95776407	0.368876	LOC102175559
5	95676407	95776407	0.368876	CDKN1B
5	95676407	95776407	0.368876	GPR19
5	95677210	95777210	0.349422	APOLD1
5	95677210	95777210	0.349422	LOC102175559
5	95677210	95777210	0.349422	CDKN1B
5	95677210	95777210	0.349422	GPR19
5	95677902	95777902	0.34733	APOLD1
5	95677902	95777902	0.34733	LOC102175559
5	95677902	95777902	0.34733	CDKN1B
5	95677902	95777902	0.34733	GPR19
5	95677929	95777929	0.369426	APOLD1
5	95677929	95777929	0.369426	LOC102175559
5	95677929	95777929	0.369426	CDKN1B
5	95677929	95777929	0.369426	GPR19
5	95678037	95778037	0.367317	APOLD1
5	95678037	95778037	0.367317	LOC102175559
5	95678037	95778037	0.367317	CDKN1B
5	95678037	95778037	0.367317	GPR19
5	95678081	95778081	0.352989	APOLD1
5	95678081	95778081	0.352989	LOC102175559
5	95678081	95778081	0.352989	CDKN1B
5	95678081	95778081	0.352989	GPR19
5	95678182	95778182	0.382627	APOLD1
5	95678182	95778182	0.382627	LOC102175559

5	95678182	95778182	0.382627	CDKN1B
5	95678182	95778182	0.382627	GPR19
5	95678302	95778302	0.386286	APOLD1
5	95678302	95778302	0.386286	LOC102175559
5	95678302	95778302	0.386286	CDKN1B
5	95678302	95778302	0.386286	GPR19
5	95678703	95778703	0.383829	APOLD1
5	95678703	95778703	0.383829	LOC102175559
5	95678703	95778703	0.383829	CDKN1B
5	95678703	95778703	0.383829	GPR19
5	95678716	95778716	0.386348	APOLD1
5	95678716	95778716	0.386348	LOC102175559
5	95678716	95778716	0.386348	CDKN1B
5	95678716	95778716	0.386348	GPR19
5	95678870	95778870	0.38481	APOLD1
5	95678870	95778870	0.38481	LOC102175559
5	95678870	95778870	0.38481	CDKN1B
5	95678870	95778870	0.38481	GPR19
5	95679020	95779020	0.379583	APOLD1
5	95679020	95779020	0.379583	LOC102175559
5	95679020	95779020	0.379583	CDKN1B
5	95679020	95779020	0.379583	GPR19
5	95679044	95779044	0.383937	APOLD1
5	95679044	95779044	0.383937	LOC102175559
5	95679044	95779044	0.383937	CDKN1B
5	95679044	95779044	0.383937	GPR19
5	95679045	95779045	0.398703	APOLD1
5	95679045	95779045	0.398703	LOC102175559
5	95679045	95779045	0.398703	CDKN1B
5	95679045	95779045	0.398703	GPR19
5	95679160	95779160	0.399009	APOLD1
5	95679160	95779160	0.399009	LOC102175559
5	95679160	95779160	0.399009	CDKN1B
5	95679160	95779160	0.399009	GPR19
5	95679236	95779236	0.403884	APOLD1
5	95679236	95779236	0.403884	LOC102175559
5	95679236	95779236	0.403884	CDKN1B
5	95679236	95779236	0.403884	GPR19
5	95679461	95779461	0.400135	APOLD1
5	95679461	95779461	0.400135	LOC102175559
5	95679461	95779461	0.400135	CDKN1B
5	95679461	95779461	0.400135	GPR19
5	95679663	95779663	0.401914	APOLD1
5	95679663	95779663	0.401914	LOC102175559
5	95679663	95779663	0.401914	CDKN1B
5	95679663	95779663	0.401914	GPR19
5	95679671	95779671	0.401914	APOLD1
5	95679671	95779671	0.401914	LOC102175559
5	95679671	95779671	0.401914	CDKN1B
5	95679671	95779671	0.401914	GPR19
5	95679764	95779764	0.399329	APOLD1
5	95679764	95779764	0.399329	LOC102175559
5	95679764	95779764	0.399329	CDKN1B
5	95679764	95779764	0.399329	GPR19
5	95679778	95779778	0.410612	APOLD1
5	95679778	95779778	0.410612	LOC102175559
5	95679778	95779778	0.410612	CDKN1B
5	95679778	95779778	0.410612	GPR19

5	95679951	95779951	0.398315	APOLD1
5	95679951	95779951	0.398315	LOC102175559
5	95679951	95779951	0.398315	CDKN1B
5	95679951	95779951	0.398315	GPR19
5	95680156	95780156	0.394692	APOLD1
5	95680156	95780156	0.394692	LOC102175559
5	95680156	95780156	0.394692	CDKN1B
5	95680156	95780156	0.394692	GPR19
5	95680187	95780187	0.398273	APOLD1
5	95680187	95780187	0.398273	LOC102175559
5	95680187	95780187	0.398273	CDKN1B
5	95680187	95780187	0.398273	GPR19
5	95680613	95780613	0.396718	APOLD1
5	95680613	95780613	0.396718	LOC102175559
5	95680613	95780613	0.396718	CDKN1B
5	95680613	95780613	0.396718	GPR19
5	95680672	95780672	0.385369	APOLD1
5	95680672	95780672	0.385369	LOC102175559
5	95680672	95780672	0.385369	CDKN1B
5	95680672	95780672	0.385369	GPR19
5	95680822	95780822	0.392637	APOLD1
5	95680822	95780822	0.392637	LOC102175559
5	95680822	95780822	0.392637	CDKN1B
5	95680822	95780822	0.392637	GPR19
5	95681345	95781345	0.40395	APOLD1
5	95681345	95781345	0.40395	LOC102175559
5	95681345	95781345	0.40395	CDKN1B
5	95681345	95781345	0.40395	GPR19
5	95681347	95781347	0.394759	APOLD1
5	95681347	95781347	0.394759	LOC102175559
5	95681347	95781347	0.394759	CDKN1B
5	95681347	95781347	0.394759	GPR19
5	95681482	95781482	0.401785	APOLD1
5	95681482	95781482	0.401785	LOC102175559
5	95681482	95781482	0.401785	CDKN1B
5	95681482	95781482	0.401785	GPR19
5	95681508	95781508	0.408885	APOLD1
5	95681508	95781508	0.408885	LOC102175559
5	95681508	95781508	0.408885	CDKN1B
5	95681508	95781508	0.408885	GPR19
5	95681591	95781591	0.409375	APOLD1
5	95681591	95781591	0.409375	LOC102175559
5	95681591	95781591	0.409375	CDKN1B
5	95681591	95781591	0.409375	GPR19
5	95681714	95781714	0.406553	APOLD1
5	95681714	95781714	0.406553	LOC102175559
5	95681714	95781714	0.406553	CDKN1B
5	95681714	95781714	0.406553	GPR19
5	95681723	95781723	0.410751	APOLD1
5	95681723	95781723	0.410751	LOC102175559
5	95681723	95781723	0.410751	CDKN1B
5	95681723	95781723	0.410751	GPR19
5	95681782	95781782	0.41277	APOLD1
5	95681782	95781782	0.41277	LOC102175559
5	95681782	95781782	0.41277	CDKN1B
5	95681782	95781782	0.41277	GPR19
5	95681791	95781791	0.411929	APOLD1
5	95681791	95781791	0.411929	LOC102175559

5	95681791	95781791	0.411929	CDKN1B
5	95681791	95781791	0.411929	GPR19
5	95681920	95781920	0.402978	APOLD1
5	95681920	95781920	0.402978	LOC102175559
5	95681920	95781920	0.402978	CDKN1B
5	95681920	95781920	0.402978	GPR19
5	95682208	95782208	0.402978	APOLD1
5	95682208	95782208	0.402978	LOC102175559
5	95682208	95782208	0.402978	CDKN1B
5	95682208	95782208	0.402978	GPR19
5	95682333	95782333	0.402978	APOLD1
5	95682333	95782333	0.402978	LOC102175559
5	95682333	95782333	0.402978	CDKN1B
5	95682333	95782333	0.402978	GPR19
5	95682385	95782385	0.403151	APOLD1
5	95682385	95782385	0.403151	LOC102175559
5	95682385	95782385	0.403151	CDKN1B
5	95682385	95782385	0.403151	GPR19
5	95682617	95782617	0.397617	APOLD1
5	95682617	95782617	0.397617	LOC102175559
5	95682617	95782617	0.397617	CDKN1B
5	95682617	95782617	0.397617	GPR19
5	95682669	95782669	0.40577	APOLD1
5	95682669	95782669	0.40577	LOC102175559
5	95682669	95782669	0.40577	CDKN1B
5	95682669	95782669	0.40577	GPR19
5	95682778	95782778	0.401754	APOLD1
5	95682778	95782778	0.401754	LOC102175559
5	95682778	95782778	0.401754	CDKN1B
5	95682778	95782778	0.401754	GPR19
5	95683157	95783157	0.415443	APOLD1
5	95683157	95783157	0.415443	LOC102175559
5	95683157	95783157	0.415443	CDKN1B
5	95683157	95783157	0.415443	GPR19
5	95683182	95783182	0.422878	APOLD1
5	95683182	95783182	0.422878	LOC102175559
5	95683182	95783182	0.422878	CDKN1B
5	95683182	95783182	0.422878	GPR19
5	95683187	95783187	0.426126	APOLD1
5	95683187	95783187	0.426126	LOC102175559
5	95683187	95783187	0.426126	CDKN1B
5	95683187	95783187	0.426126	GPR19
5	95683300	95783300	0.415328	APOLD1
5	95683300	95783300	0.415328	LOC102175559
5	95683300	95783300	0.415328	CDKN1B
5	95683300	95783300	0.415328	GPR19
5	95683500	95783500	0.458038	APOLD1
5	95683500	95783500	0.458038	LOC102175559
5	95683500	95783500	0.458038	CDKN1B
5	95683500	95783500	0.458038	GPR19
5	95683804	95783804	0.445399	APOLD1
5	95683804	95783804	0.445399	LOC102175559
5	95683804	95783804	0.445399	CDKN1B
5	95683804	95783804	0.445399	GPR19
5	95683819	95783819	0.467627	APOLD1
5	95683819	95783819	0.467627	LOC102175559
5	95683819	95783819	0.467627	CDKN1B
5	95683819	95783819	0.467627	GPR19

5	95683995	95783995	0.462892	APOLD1
5	95683995	95783995	0.462892	LOC102175559
5	95683995	95783995	0.462892	CDKN1B
5	95683995	95783995	0.462892	GPR19
5	95684017	95784017	0.456902	APOLD1
5	95684017	95784017	0.456902	LOC102175559
5	95684017	95784017	0.456902	CDKN1B
5	95684017	95784017	0.456902	GPR19
5	95684089	95784089	0.462051	APOLD1
5	95684089	95784089	0.462051	LOC102175559
5	95684089	95784089	0.462051	CDKN1B
5	95684089	95784089	0.462051	GPR19
5	95684168	95784168	0.462223	APOLD1
5	95684168	95784168	0.462223	LOC102175559
5	95684168	95784168	0.462223	CDKN1B
5	95684168	95784168	0.462223	GPR19
5	95684373	95784373	0.45535	APOLD1
5	95684373	95784373	0.45535	LOC102175559
5	95684373	95784373	0.45535	CDKN1B
5	95684373	95784373	0.45535	GPR19
5	95684840	95784840	0.446311	APOLD1
5	95684840	95784840	0.446311	LOC102175559
5	95684840	95784840	0.446311	CDKN1B
5	95684840	95784840	0.446311	GPR19
5	95684874	95784874	0.500385	APOLD1
5	95684874	95784874	0.500385	LOC102175559
5	95684874	95784874	0.500385	CDKN1B
5	95684874	95784874	0.500385	GPR19
5	95684881	95784881	0.497112	APOLD1
5	95684881	95784881	0.497112	LOC102175559
5	95684881	95784881	0.497112	CDKN1B
5	95684881	95784881	0.497112	GPR19
5	95684978	95784978	0.395344	APOLD1
5	95684978	95784978	0.395344	LOC102175559
5	95684978	95784978	0.395344	CDKN1B
5	95684978	95784978	0.395344	GPR19
5	95685251	95785251	0.396602	APOLD1
5	95685251	95785251	0.396602	LOC102175559
5	95685251	95785251	0.396602	CDKN1B
5	95685251	95785251	0.396602	GPR19
5	95685411	95785411	0.385069	APOLD1
5	95685411	95785411	0.385069	LOC102175559
5	95685411	95785411	0.385069	CDKN1B
5	95685411	95785411	0.385069	GPR19
5	95685509	95785509	0.38734	APOLD1
5	95685509	95785509	0.38734	LOC102175559
5	95685509	95785509	0.38734	CDKN1B
5	95685509	95785509	0.38734	GPR19
5	95685730	95785730	0.393627	APOLD1
5	95685730	95785730	0.393627	LOC102175559
5	95685730	95785730	0.393627	CDKN1B
5	95685730	95785730	0.393627	GPR19
5	95685852	95785852	0.394313	APOLD1
5	95685852	95785852	0.394313	LOC102175559
5	95685852	95785852	0.394313	CDKN1B
5	95685852	95785852	0.394313	GPR19
5	95685951	95785951	0.402669	APOLD1
5	95685951	95785951	0.402669	LOC102175559

5	95685951	95785951	0.402669	CDKN1B
5	95685951	95785951	0.402669	GPR19
5	95686263	95786263	0.388583	APOLD1
5	95686263	95786263	0.388583	LOC102175559
5	95686263	95786263	0.388583	CDKN1B
5	95686263	95786263	0.388583	GPR19
5	95686638	95786638	0.42373	APOLD1
5	95686638	95786638	0.42373	LOC102175559
5	95686638	95786638	0.42373	CDKN1B
5	95686638	95786638	0.42373	GPR19
5	95686948	95786948	0.428434	APOLD1
5	95686948	95786948	0.428434	LOC102175559
5	95686948	95786948	0.428434	CDKN1B
5	95686948	95786948	0.428434	GPR19
5	95687194	95787194	0.427237	APOLD1
5	95687194	95787194	0.427237	LOC102175559
5	95687194	95787194	0.427237	CDKN1B
5	95687194	95787194	0.427237	GPR19
5	95687345	95787345	0.420556	APOLD1
5	95687345	95787345	0.420556	LOC102175559
5	95687345	95787345	0.420556	CDKN1B
5	95687345	95787345	0.420556	GPR19
5	95687548	95787548	0.424945	APOLD1
5	95687548	95787548	0.424945	LOC102175559
5	95687548	95787548	0.424945	CDKN1B
5	95687548	95787548	0.424945	GPR19
5	95687550	95787550	0.424995	APOLD1
5	95687550	95787550	0.424995	LOC102175559
5	95687550	95787550	0.424995	CDKN1B
5	95687550	95787550	0.424995	GPR19
5	95687557	95787557	0.400158	APOLD1
5	95687557	95787557	0.400158	LOC102175559
5	95687557	95787557	0.400158	CDKN1B
5	95687557	95787557	0.400158	GPR19
5	95688186	95788186	0.400158	APOLD1
5	95688186	95788186	0.400158	LOC102175559
5	95688186	95788186	0.400158	CDKN1B
5	95688186	95788186	0.400158	GPR19
5	95689891	95789891	0.401297	APOLD1
5	95689891	95789891	0.401297	LOC102175559
5	95689891	95789891	0.401297	CDKN1B
5	95689891	95789891	0.401297	GPR19
5	95690813	95790813	0.38207	APOLD1
5	95690813	95790813	0.38207	LOC102175559
5	95690813	95790813	0.38207	CDKN1B
5	95690813	95790813	0.38207	GPR19
5	95691205	95791205	0.407565	APOLD1
5	95691205	95791205	0.407565	LOC102175559
5	95691205	95791205	0.407565	CDKN1B
5	95691205	95791205	0.407565	GPR19
5	95691215	95791215	0.419979	APOLD1
5	95691215	95791215	0.419979	LOC102175559
5	95691215	95791215	0.419979	CDKN1B
5	95691215	95791215	0.419979	GPR19
5	95691783	95791783	0.357295	APOLD1
5	95691783	95791783	0.357295	LOC102175559
5	95691783	95791783	0.357295	CDKN1B
5	95691783	95791783	0.357295	GPR19

5	98530321	98630321	0.398478	LOC102189655
5	98530321	98630321	0.398478	LOC102173270
5	98530321	98630321	0.398478	LOC102189932
5	98530341	98630341	0.43969	LOC102189655
5	98530341	98630341	0.43969	LOC102173270
5	98530341	98630341	0.43969	LOC102189932
5	98600073	98700073	0.35321	LOC102189932
5	98600073	98700073	0.35321	KLRF1
5	98611262	98711262	0.342927	LOC102189932
5	98611262	98711262	0.342927	KLRF1
5	98628602	98728602	0.345423	LOC102189932
5	98628602	98728602	0.345423	KLRF1
5	98628602	98728602	0.345423	CD69
5	98628643	98728643	0.344991	LOC102189932
5	98628643	98728643	0.344991	KLRF1
5	98628643	98728643	0.344991	CD69
5	98628737	98728737	0.360311	LOC102189932
5	98628737	98728737	0.360311	KLRF1
5	98628737	98728737	0.360311	CD69
5	98628866	98728866	0.359878	LOC102189932
5	98628866	98728866	0.359878	KLRF1
5	98628866	98728866	0.359878	CD69
5	98628930	98728930	0.360108	LOC102189932
5	98628930	98728930	0.360108	KLRF1
5	98628930	98728930	0.360108	CD69
5	98629115	98729115	0.358516	LOC102189932
5	98629115	98729115	0.358516	KLRF1
5	98629115	98729115	0.358516	CD69
5	98629192	98729192	0.351616	LOC102189932
5	98629192	98729192	0.351616	KLRF1
5	98629192	98729192	0.351616	CD69
5	98629196	98729196	0.351616	LOC102189932
5	98629196	98729196	0.351616	KLRF1
5	98629196	98729196	0.351616	CD69
5	98629335	98729335	0.346884	LOC102189932
5	98629335	98729335	0.346884	KLRF1
5	98629335	98729335	0.346884	CD69
5	98629398	98729398	0.343893	LOC102189932
5	98629398	98729398	0.343893	KLRF1
5	98629398	98729398	0.343893	CD69
5	98629471	98729471	0.362774	LOC102189932
5	98629471	98729471	0.362774	KLRF1
5	98629471	98729471	0.362774	CD69
5	98629490	98729490	0.362698	LOC102189932
5	98629490	98729490	0.362698	KLRF1
5	98629490	98729490	0.362698	CD69
5	98629575	98729575	0.363239	LOC102189932
5	98629575	98729575	0.363239	KLRF1
5	98629575	98729575	0.363239	CD69
5	98629576	98729576	0.363239	LOC102189932
5	98629576	98729576	0.363239	KLRF1
5	98629576	98729576	0.363239	CD69
5	98629621	98729621	0.363239	LOC102189932
5	98629621	98729621	0.363239	KLRF1
5	98629621	98729621	0.363239	CD69
5	98629799	98729799	0.368638	LOC102189932
5	98629799	98729799	0.368638	KLRF1
5	98629799	98729799	0.368638	CD69

5	98629918	98729918	0.369184	LOC102189932
5	98629918	98729918	0.369184	KLRF1
5	98629918	98729918	0.369184	CD69
5	98629958	98729958	0.36927	LOC102189932
5	98629958	98729958	0.36927	KLRF1
5	98629958	98729958	0.36927	CD69
5	98630024	98730024	0.36927	LOC102189932
5	98630024	98730024	0.36927	KLRF1
5	98630024	98730024	0.36927	CD69
5	98630038	98730038	0.369251	LOC102189932
5	98630038	98730038	0.369251	KLRF1
5	98630038	98730038	0.369251	CD69
5	98630177	98730177	0.368396	LOC102189932
5	98630177	98730177	0.368396	KLRF1
5	98630177	98730177	0.368396	CD69
5	98630398	98730398	0.381202	LOC102189932
5	98630398	98730398	0.381202	KLRF1
5	98630398	98730398	0.381202	CD69
5	98630562	98730562	0.355071	LOC102189932
5	98630562	98730562	0.355071	KLRF1
5	98630562	98730562	0.355071	CD69
5	98630632	98730632	0.355071	LOC102189932
5	98630632	98730632	0.355071	KLRF1
5	98630632	98730632	0.355071	CD69
5	98630656	98730656	0.359028	LOC102189932
5	98630656	98730656	0.359028	KLRF1
5	98630656	98730656	0.359028	CD69
5	98630657	98730657	0.359028	LOC102189932
5	98630657	98730657	0.359028	KLRF1
5	98630657	98730657	0.359028	CD69
5	98630709	98730709	0.359983	LOC102189932
5	98630709	98730709	0.359983	KLRF1
5	98630709	98730709	0.359983	CD69
5	98630814	98730814	0.358527	LOC102189932
5	98630814	98730814	0.358527	KLRF1
5	98630814	98730814	0.358527	CD69
5	98630861	98730861	0.35342	LOC102189932
5	98630861	98730861	0.35342	KLRF1
5	98630861	98730861	0.35342	CD69
5	98631031	98731031	0.342305	LOC102189932
5	98631031	98731031	0.342305	KLRF1
5	98631031	98731031	0.342305	CD69
5	98631038	98731038	0.349857	LOC102189932
5	98631038	98731038	0.349857	KLRF1
5	98631038	98731038	0.349857	CD69
5	98631376	98731376	0.343365	LOC102189932
5	98631376	98731376	0.343365	KLRF1
5	98631376	98731376	0.343365	CD69
5	98631404	98731404	0.348105	LOC102189932
5	98631404	98731404	0.348105	KLRF1
5	98631404	98731404	0.348105	CD69
5	98631904	98731904	0.34175	LOC102189932
5	98631904	98731904	0.34175	KLRF1
5	98631904	98731904	0.34175	CD69
5	98631948	98731948	0.343431	LOC102189932
5	98631948	98731948	0.343431	KLRF1
5	98631948	98731948	0.343431	CD69
5	98631952	98731952	0.343861	LOC102189932

5	98631952	98731952	0.343861	KLRF1
5	98631952	98731952	0.343861	CD69
5	98631971	98731971	0.34535	LOC102189932
5	98631971	98731971	0.34535	KLRF1
5	98631971	98731971	0.34535	CD69
5	98631981	98731981	0.342664	LOC102189932
5	98631981	98731981	0.342664	KLRF1
5	98631981	98731981	0.342664	CD69
5	98632046	98732046	0.343408	LOC102189932
5	98632046	98732046	0.343408	KLRF1
5	98632046	98732046	0.343408	CD69
5	98632152	98732152	0.343297	LOC102189932
5	98632152	98732152	0.343297	KLRF1
5	98632152	98732152	0.343297	CD69
5	98640141	98740141	0.346266	KLRF1
5	98640141	98740141	0.346266	CD69
5	98868703	98968703	0.358795	LOC108636060
5	98872789	98972789	0.352333	LOC108636060
5	98872792	98972792	0.350645	LOC108636060
5	98872795	98972795	0.350251	LOC108636060
5	98872796	98972796	0.349905	LOC108636060
5	98872804	98972804	0.341748	LOC108636060
5	98872852	98972852	0.345818	LOC108636060
5	98930788	99030788	0.349548	LOC108636060
5	98930844	99030844	0.343722	LOC108636060
5	98930869	99030869	0.348455	LOC108636060
5	98930940	99030940	0.347729	LOC108636060
5	98931652	99031652	0.408364	LOC108636060
5	98932174	99032174	0.345393	LOC108636060
5	98932213	99032213	0.349804	LOC108636060
5	98932215	99032215	0.344141	LOC108636060
5	98932628	99032628	0.344064	LOC108636060
5	98941103	99041103	0.343207	LOC108636060
5	98941106	99041106	0.343271	LOC108636060
5	98941108	99041108	0.346267	LOC108636060
5	98941122	99041122	0.344391	LOC108636060
5	98941126	99041126	0.343533	LOC108636060
5	98941131	99041131	0.342409	LOC108636060
5	98941155	99041155	0.356231	LOC108636060
5	98941158	99041158	0.356231	LOC108636060
5	99192589	99292589	0.356854	LOC108633342
5	99192589	99292589	0.356854	LOC102181491
5	99337881	99437881	0.359623	LOC102184231
5	99337881	99437881	0.359623	LOC102184701
5	99337935	99437935	0.346046	LOC102184231
5	99337935	99437935	0.346046	LOC102184701
5	99398641	99498641	0.34275	LOC102184231
5	99398641	99498641	0.34275	LOC102183943
5	100812520	100912520	0.356636	LOC102186205
5	100812554	100912554	0.444819	LOC102186205
5	100812563	100912563	0.444134	LOC102186205
5	100812584	100912584	0.450393	LOC102186205
5	100812603	100912603	0.457292	LOC102186205
5	100812694	100912694	0.532303	LOC102186205
5	100812705	100912705	0.536723	LOC102186205
5	100812831	100912831	0.504482	LOC102186205
5	100812906	100912906	0.502623	LOC102186205
5	100813268	100913268	0.500622	LOC102186205

5	100813317	100913317	0.475509	LOC102186205
5	100813318	100913318	0.471234	LOC102186205
5	100813319	100913319	0.48042	LOC102186205
5	100813328	100913328	0.457316	LOC102186205
5	100813343	100913343	0.42512	LOC102186205
5	100813364	100913364	0.422842	LOC102186205
5	103326782	103426782	0.347315	ANO2
5	103326802	103426802	0.345857	ANO2
5	103326816	103426816	0.354978	ANO2
5	103326930	103426930	0.365752	ANO2
5	103327009	103427009	0.360469	ANO2
5	104455717	104555717	0.346263	RAD51AP1
5	104455717	104555717	0.346263	C5H12orf4
5	104455717	104555717	0.346263	DYRK4
5	104455777	104555777	0.36081	RAD51AP1
5	104455777	104555777	0.36081	C5H12orf4
5	104455777	104555777	0.36081	DYRK4
5	104455834	104555834	0.389203	RAD51AP1
5	104455834	104555834	0.389203	C5H12orf4
5	104455834	104555834	0.389203	DYRK4
5	104455914	104555914	0.390986	RAD51AP1
5	104455914	104555914	0.390986	C5H12orf4
5	104455914	104555914	0.390986	DYRK4
5	104455926	104555926	0.381899	RAD51AP1
5	104455926	104555926	0.381899	C5H12orf4
5	104455926	104555926	0.381899	DYRK4
5	104456097	104556097	0.370018	RAD51AP1
5	104456097	104556097	0.370018	C5H12orf4
5	104456097	104556097	0.370018	DYRK4
5	104456360	104556360	0.352392	RAD51AP1
5	104456360	104556360	0.352392	C5H12orf4
5	104456360	104556360	0.352392	DYRK4
5	104456379	104556379	0.341891	RAD51AP1
5	104456379	104556379	0.341891	C5H12orf4
5	104456379	104556379	0.341891	DYRK4
5	104456516	104556516	0.355463	RAD51AP1
5	104456516	104556516	0.355463	C5H12orf4
5	104456516	104556516	0.355463	DYRK4
5	104456517	104556517	0.384864	RAD51AP1
5	104456517	104556517	0.384864	C5H12orf4
5	104456517	104556517	0.384864	DYRK4
5	104456518	104556518	0.400875	RAD51AP1
5	104456518	104556518	0.400875	C5H12orf4
5	104456518	104556518	0.400875	DYRK4
5	104456549	104556549	0.41016	RAD51AP1
5	104456549	104556549	0.41016	C5H12orf4
5	104456549	104556549	0.41016	DYRK4
5	104456573	104556573	0.428479	RAD51AP1
5	104456573	104556573	0.428479	C5H12orf4
5	104456573	104556573	0.428479	DYRK4
5	104456586	104556586	0.384345	RAD51AP1
5	104456586	104556586	0.384345	C5H12orf4
5	104456586	104556586	0.384345	DYRK4
5	104456606	104556606	0.36446	RAD51AP1
5	104456606	104556606	0.36446	C5H12orf4
5	104456606	104556606	0.36446	DYRK4
5	104456630	104556630	0.452878	RAD51AP1
5	104456630	104556630	0.452878	C5H12orf4

5	104456630	104556630	0.452878	DYRK4
5	104456680	104556680	0.414448	RAD51AP1
5	104456680	104556680	0.414448	C5H12orf4
5	104456680	104556680	0.414448	DYRK4
5	104456736	104556736	0.412642	RAD51AP1
5	104456736	104556736	0.412642	C5H12orf4
5	104456736	104556736	0.412642	DYRK4
5	104456893	104556893	0.409231	RAD51AP1
5	104456893	104556893	0.409231	C5H12orf4
5	104456893	104556893	0.409231	DYRK4
5	104456932	104556932	0.410165	RAD51AP1
5	104456932	104556932	0.410165	C5H12orf4
5	104456932	104556932	0.410165	DYRK4
5	104456989	104556989	0.407461	RAD51AP1
5	104456989	104556989	0.407461	C5H12orf4
5	104456989	104556989	0.407461	DYRK4
5	104457016	104557016	0.397131	RAD51AP1
5	104457016	104557016	0.397131	C5H12orf4
5	104457016	104557016	0.397131	DYRK4
5	104457022	104557022	0.396656	RAD51AP1
5	104457022	104557022	0.396656	C5H12orf4
5	104457022	104557022	0.396656	DYRK4
5	104457093	104557093	0.388221	RAD51AP1
5	104457093	104557093	0.388221	C5H12orf4
5	104457093	104557093	0.388221	DYRK4
5	104457138	104557138	0.385578	RAD51AP1
5	104457138	104557138	0.385578	C5H12orf4
5	104457138	104557138	0.385578	DYRK4
5	104457190	104557190	0.397749	RAD51AP1
5	104457190	104557190	0.397749	C5H12orf4
5	104457190	104557190	0.397749	DYRK4
5	104457251	104557251	0.386512	RAD51AP1
5	104457251	104557251	0.386512	C5H12orf4
5	104457251	104557251	0.386512	DYRK4
5	104457254	104557254	0.351105	RAD51AP1
5	104457254	104557254	0.351105	C5H12orf4
5	104457254	104557254	0.351105	DYRK4
5	104457346	104557346	0.350087	RAD51AP1
5	104457346	104557346	0.350087	C5H12orf4
5	104457346	104557346	0.350087	DYRK4
5	104458613	104558613	0.348135	RAD51AP1
5	104458613	104558613	0.348135	C5H12orf4
5	104458613	104558613	0.348135	DYRK4
5	104458719	104558719	0.372216	RAD51AP1
5	104458719	104558719	0.372216	C5H12orf4
5	104458719	104558719	0.372216	DYRK4
5	104458741	104558741	0.379483	RAD51AP1
5	104458741	104558741	0.379483	C5H12orf4
5	104458741	104558741	0.379483	DYRK4
5	104458830	104558830	0.373215	RAD51AP1
5	104458830	104558830	0.373215	C5H12orf4
5	104458830	104558830	0.373215	DYRK4
5	104458885	104558885	0.373215	RAD51AP1
5	104458885	104558885	0.373215	C5H12orf4
5	104458885	104558885	0.373215	DYRK4
5	104458939	104558939	0.359465	RAD51AP1
5	104458939	104558939	0.359465	C5H12orf4
5	104458939	104558939	0.359465	DYRK4

5	104459643	104559643	0.362542	RAD51AP1
5	104459643	104559643	0.362542	C5H12orf4
5	104459643	104559643	0.362542	DYRK4
5	104459716	104559716	0.37413	RAD51AP1
5	104459716	104559716	0.37413	C5H12orf4
5	104459716	104559716	0.37413	DYRK4
5	104459823	104559823	0.377401	RAD51AP1
5	104459823	104559823	0.377401	C5H12orf4
5	104459823	104559823	0.377401	DYRK4
5	104460145	104560145	0.377401	RAD51AP1
5	104460145	104560145	0.377401	C5H12orf4
5	104460145	104560145	0.377401	DYRK4
5	104460165	104560165	0.366112	RAD51AP1
5	104460165	104560165	0.366112	C5H12orf4
5	104460165	104560165	0.366112	DYRK4
5	104461955	104561955	0.343288	RAD51AP1
5	104461955	104561955	0.343288	C5H12orf4
5	104461955	104561955	0.343288	DYRK4
5	104687057	104787057	0.348838	LOC106502140
5	104687057	104787057	0.348838	CCND2
5	104687057	104787057	0.348838	LOC108636066
5	104687070	104787070	0.348838	LOC106502140
5	104687070	104787070	0.348838	CCND2
5	104687070	104787070	0.348838	LOC108636066
5	104687106	104787106	0.351105	LOC106502140
5	104687106	104787106	0.351105	CCND2
5	104687106	104787106	0.351105	LOC108636066
5	104687404	104787404	0.347253	LOC106502140
5	104687404	104787404	0.347253	CCND2
5	104687404	104787404	0.347253	LOC108636066
5	104688679	104788679	0.356284	LOC106502140
5	104688679	104788679	0.356284	CCND2
5	104688679	104788679	0.356284	LOC108636066
5	104688688	104788688	0.349509	LOC106502140
5	104688688	104788688	0.349509	CCND2
5	104688688	104788688	0.349509	LOC108636066
5	104691229	104791229	0.342012	LOC106502140
5	104691229	104791229	0.342012	CCND2
5	104691229	104791229	0.342012	LOC108636066
5	104691244	104791244	0.348983	LOC106502140
5	104691244	104791244	0.348983	CCND2
5	104691244	104791244	0.348983	LOC108636066
5	104691251	104791251	0.360886	LOC106502140
5	104691251	104791251	0.360886	CCND2
5	104691251	104791251	0.360886	LOC108636066
5	104691949	104791949	0.364051	LOC106502140
5	104691949	104791949	0.364051	CCND2
5	104691949	104791949	0.364051	LOC108636066
5	104691950	104791950	0.370368	LOC106502140
5	104691950	104791950	0.370368	CCND2
5	104691950	104791950	0.370368	LOC108636066
5	104691985	104791985	0.369544	LOC106502140
5	104691985	104791985	0.369544	CCND2
5	104691985	104791985	0.369544	LOC108636066
5	104692129	104792129	0.357902	LOC106502140
5	104692129	104792129	0.357902	CCND2
5	104692129	104792129	0.357902	LOC108636066
5	104692877	104792877	0.348154	LOC106502140

5	104692877	104792877	0.348154	CCND2
5	104692877	104792877	0.348154	LOC108636066
5	104693100	104793100	0.342577	LOC106502140
5	104693100	104793100	0.342577	CCND2
5	104693100	104793100	0.342577	LOC108636066
5	104693664	104793664	0.342593	LOC106502140
5	104693664	104793664	0.342593	CCND2
5	104693664	104793664	0.342593	LOC108636066
5	104694091	104794091	0.343773	LOC106502140
5	104694091	104794091	0.343773	CCND2
5	104694091	104794091	0.343773	LOC108636066
5	104694167	104794167	0.358952	LOC106502140
5	104694167	104794167	0.358952	CCND2
5	104694167	104794167	0.358952	LOC108636066
5	104694185	104794185	0.358382	LOC106502140
5	104694185	104794185	0.358382	CCND2
5	104694185	104794185	0.358382	LOC108636066
5	104694186	104794186	0.351416	LOC106502140
5	104694186	104794186	0.351416	CCND2
5	104694186	104794186	0.351416	LOC108636066
5	104694323	104794323	0.354919	LOC106502140
5	104694323	104794323	0.354919	CCND2
5	104694323	104794323	0.354919	LOC108636066
5	104694324	104794324	0.350313	LOC106502140
5	104694324	104794324	0.350313	CCND2
5	104694324	104794324	0.350313	LOC108636066
5	104694889	104794889	0.344758	LOC106502140
5	104694889	104794889	0.344758	CCND2
5	104694889	104794889	0.344758	LOC108636066
5	104694969	104794969	0.34306	LOC106502140
5	104694969	104794969	0.34306	CCND2
5	104694969	104794969	0.34306	LOC108636066
5	104694986	104794986	0.343201	LOC106502140
5	104694986	104794986	0.343201	CCND2
5	104694986	104794986	0.343201	LOC108636066
5	104695001	104795001	0.35086	LOC106502140
5	104695001	104795001	0.35086	CCND2
5	104695001	104795001	0.35086	LOC108636066
5	104695016	104795016	0.341794	LOC106502140
5	104695016	104795016	0.341794	CCND2
5	104695016	104795016	0.341794	LOC108636066
5	105161766	105261766	0.384613	CRACR2A
5	105161766	105261766	0.384613	PRMT8
5	105162814	105262814	0.382167	CRACR2A
5	105162814	105262814	0.382167	PRMT8
5	105163078	105263078	0.34878	CRACR2A
5	105163078	105263078	0.34878	PRMT8
5	105164168	105264168	0.381878	CRACR2A
5	105164168	105264168	0.381878	PRMT8
5	105164174	105264174	0.389449	CRACR2A
5	105164174	105264174	0.389449	PRMT8
5	105164200	105264200	0.373021	CRACR2A
5	105164200	105264200	0.373021	PRMT8
5	105164213	105264213	0.403554	CRACR2A
5	105164213	105264213	0.403554	PRMT8
5	105164225	105264225	0.401312	CRACR2A
5	105164225	105264225	0.401312	PRMT8
5	105164435	105264435	0.358967	CRACR2A

5	105164435	105264435	0.358967	PRMT8
5	105164579	105264579	0.365907	CRACR2A
5	105164579	105264579	0.365907	PRMT8
5	105164810	105264810	0.366782	CRACR2A
5	105164810	105264810	0.366782	PRMT8
5	105165149	105265149	0.390691	CRACR2A
5	105165149	105265149	0.390691	PRMT8
5	105165154	105265154	0.390691	CRACR2A
5	105165154	105265154	0.390691	PRMT8
5	105165291	105265291	0.374346	CRACR2A
5	105165291	105265291	0.374346	PRMT8
5	105165330	105265330	0.375276	CRACR2A
5	105165330	105265330	0.375276	PRMT8
5	105165341	105265341	0.375276	CRACR2A
5	105165341	105265341	0.375276	PRMT8
5	105165443	105265443	0.343738	CRACR2A
5	105165443	105265443	0.343738	PRMT8
5	105165644	105265644	0.402355	CRACR2A
5	105165644	105265644	0.402355	PRMT8
5	105165732	105265732	0.402824	CRACR2A
5	105165732	105265732	0.402824	PRMT8
5	105165809	105265809	0.361674	CRACR2A
5	105165809	105265809	0.361674	PRMT8
5	105166754	105266754	0.354593	CRACR2A
5	105166754	105266754	0.354593	PRMT8
5	105166770	105266770	0.34933	CRACR2A
5	105166770	105266770	0.34933	PRMT8
5	105166793	105266793	0.34933	CRACR2A
5	105166793	105266793	0.34933	PRMT8
5	105167177	105267177	0.355314	CRACR2A
5	105167177	105267177	0.355314	PRMT8
5	105167181	105267181	0.355314	CRACR2A
5	105167181	105267181	0.355314	PRMT8
5	105167239	105267239	0.355314	CRACR2A
5	105167239	105267239	0.355314	PRMT8
5	105167240	105267240	0.355314	CRACR2A
5	105167240	105267240	0.355314	PRMT8
5	105167539	105267539	0.367226	CRACR2A
5	105167539	105267539	0.367226	PRMT8
5	105167653	105267653	0.40434	CRACR2A
5	105167653	105267653	0.40434	PRMT8
5	105167666	105267666	0.40434	CRACR2A
5	105167666	105267666	0.40434	PRMT8
5	105167786	105267786	0.40434	CRACR2A
5	105167786	105267786	0.40434	PRMT8
5	105167823	105267823	0.363312	CRACR2A
5	105167823	105267823	0.363312	PRMT8
5	105167872	105267872	0.362566	CRACR2A
5	105167872	105267872	0.362566	PRMT8
5	105168308	105268308	0.353185	CRACR2A
5	105168308	105268308	0.353185	PRMT8
5	105168393	105268393	0.38696	CRACR2A
5	105168393	105268393	0.38696	PRMT8
5	105168996	105268996	0.347317	CRACR2A
5	105168996	105268996	0.347317	PRMT8
5	105169536	105269536	0.362832	CRACR2A
5	105169536	105269536	0.362832	PRMT8
5	105169852	105269852	0.360544	CRACR2A

5	105169852	105269852	0.360544	PRMT8
5	105169924	105269924	0.366518	CRACR2A
5	105169924	105269924	0.366518	PRMT8
5	105169948	105269948	0.399886	CRACR2A
5	105169948	105269948	0.399886	PRMT8
5	105169949	105269949	0.386923	CRACR2A
5	105169949	105269949	0.386923	PRMT8
5	105169986	105269986	0.345874	CRACR2A
5	105169986	105269986	0.345874	PRMT8
5	105170040	105270040	0.373251	CRACR2A
5	105170040	105270040	0.373251	PRMT8
5	105170129	105270129	0.393434	CRACR2A
5	105170129	105270129	0.393434	PRMT8
5	105170313	105270313	0.355462	CRACR2A
5	105170313	105270313	0.355462	PRMT8
5	105170398	105270398	0.356253	CRACR2A
5	105170398	105270398	0.356253	PRMT8
5	105171806	105271806	0.347914	CRACR2A
5	105171806	105271806	0.347914	PRMT8
5	105171822	105271822	0.37042	CRACR2A
5	105171822	105271822	0.37042	PRMT8
5	105451632	105551632	0.409099	TSPAN11
5	105451632	105551632	0.409099	TSPAN9
5	105451639	105551639	0.422519	TSPAN11
5	105451639	105551639	0.422519	TSPAN9
5	105451647	105551647	0.395598	TSPAN11
5	105451647	105551647	0.395598	TSPAN9
5	105451661	105551661	0.365223	TSPAN11
5	105451661	105551661	0.365223	TSPAN9
5	105451669	105551669	0.350925	TSPAN11
5	105451669	105551669	0.350925	TSPAN9
5	106090183	106190183	0.376363	LOC102172245
5	106090183	106190183	0.376363	SLC6A13
5	106090183	106190183	0.376363	KDM5A
5	106090621	106190621	0.369848	LOC102172245
5	106090621	106190621	0.369848	SLC6A13
5	106090621	106190621	0.369848	KDM5A
5	106090686	106190686	0.373612	LOC102172245
5	106090686	106190686	0.373612	SLC6A13
5	106090686	106190686	0.373612	KDM5A
5	106091329	106191329	0.36977	LOC102172245
5	106091329	106191329	0.36977	SLC6A13
5	106091329	106191329	0.36977	KDM5A
5	106092159	106192159	0.381638	LOC102172245
5	106092159	106192159	0.381638	SLC6A13
5	106092159	106192159	0.381638	KDM5A
5	106092415	106192415	0.370159	LOC102172245
5	106092415	106192415	0.370159	SLC6A13
5	106092415	106192415	0.370159	KDM5A
5	106092484	106192484	0.365536	LOC102172245
5	106092484	106192484	0.365536	SLC6A13
5	106092484	106192484	0.365536	KDM5A
5	106092728	106192728	0.365536	LOC102172245
5	106092728	106192728	0.365536	SLC6A13
5	106092728	106192728	0.365536	KDM5A
5	106092813	106192813	0.368245	LOC102172245
5	106092813	106192813	0.368245	SLC6A13
5	106092813	106192813	0.368245	KDM5A

5	106092955	106192955	0.375308	LOC102172245
5	106092955	106192955	0.375308	SLC6A13
5	106092955	106192955	0.375308	KDM5A
5	106092997	106192997	0.370312	LOC102172245
5	106092997	106192997	0.370312	SLC6A13
5	106092997	106192997	0.370312	KDM5A
5	106093376	106193376	0.373565	LOC102172245
5	106093376	106193376	0.373565	SLC6A13
5	106093376	106193376	0.373565	KDM5A
5	106093905	106193905	0.373936	SLC6A13
5	106093905	106193905	0.373936	KDM5A
5	106094389	106194389	0.373936	SLC6A13
5	106094389	106194389	0.373936	KDM5A
5	106095268	106195268	0.381334	SLC6A13
5	106095268	106195268	0.381334	KDM5A
5	106095269	106195269	0.381334	SLC6A13
5	106095269	106195269	0.381334	KDM5A
5	106095341	106195341	0.348336	SLC6A13
5	106095341	106195341	0.348336	KDM5A
5	106095558	106195558	0.35085	SLC6A13
5	106095558	106195558	0.35085	KDM5A
5	106095832	106195832	0.373245	SLC6A13
5	106095832	106195832	0.373245	KDM5A
5	106837283	106937283	0.343642	ERC1
5	106842954	106942954	0.351358	ERC1
5	106843231	106943231	0.345578	ERC1
5	106843424	106943424	0.345417	ERC1
5	106843528	106943528	0.34372	ERC1
5	106843537	106943537	0.345389	ERC1
5	106843597	106943597	0.345389	ERC1
5	106843781	106943781	0.345389	ERC1
5	106843793	106943793	0.346781	ERC1
5	106843810	106943810	0.346686	ERC1
5	106843839	106943839	0.346686	ERC1
5	106844006	106944006	0.346686	ERC1
5	106844283	106944283	0.346686	ERC1
5	106844362	106944362	0.351676	ERC1
5	106845699	106945699	0.365605	ERC1
5	106845863	106945863	0.357081	ERC1
5	106845937	106945937	0.35667	ERC1
5	106846284	106946284	0.35828	ERC1
5	106846770	106946770	0.357144	ERC1
5	106847374	106947374	0.348643	ERC1
5	106847468	106947468	0.348643	ERC1
5	106847663	106947663	0.354997	ERC1
5	106847806	106947806	0.363664	ERC1
5	106847834	106947834	0.366378	ERC1
5	106847884	106947884	0.372174	ERC1
5	106848103	106948103	0.365916	ERC1
5	106848138	106948138	0.355675	ERC1
5	106848274	106948274	0.358013	ERC1
5	106848797	106948797	0.34553	ERC1
5	106849338	106949338	0.343746	ERC1
5	106850766	106950766	0.356047	ERC1
5	106850924	106950924	0.352369	ERC1
5	106850959	106950959	0.350946	ERC1
5	106851182	106951182	0.348752	ERC1
5	106851312	106951312	0.351633	ERC1

5	106851613	106951613	0.354108	ERC1
5	106851639	106951639	0.348955	ERC1
5	106852081	106952081	0.341835	ERC1
5	106852529	106952529	0.391709	ERC1
5	106852666	106952666	0.39079	ERC1
5	106852699	106952699	0.390284	ERC1
5	106852731	106952731	0.390284	ERC1
5	106853089	106953089	0.398459	ERC1
5	106853133	106953133	0.380344	ERC1
5	106853851	106953851	0.411302	ERC1
5	106853852	106953852	0.411302	ERC1
5	106854355	106954355	0.384873	ERC1
5	106854840	106954840	0.385286	ERC1
5	106855295	106955295	0.366509	ERC1
5	106855871	106955871	0.370506	ERC1
5	106855911	106955911	0.370506	ERC1
5	106855981	106955981	0.437208	ERC1
5	106856143	106956143	0.490817	ERC1
5	106856144	106956144	0.434404	ERC1
5	106856677	106956677	0.411818	ERC1
5	106856788	106956788	0.411818	ERC1
5	106856800	106956800	0.411818	ERC1
5	106856832	106956832	0.423029	ERC1
5	106856989	106956989	0.399686	ERC1
5	106856998	106956998	0.396938	ERC1
5	106857138	106957138	0.405419	ERC1
5	106857220	106957220	0.381656	ERC1
5	106857377	106957377	0.376776	ERC1
5	106857460	106957460	0.364449	ERC1
5	106857528	106957528	0.370441	ERC1
5	107543680	107643680	0.344271	CACNA1C
5	107543738	107643738	0.351357	CACNA1C
5	107543899	107643899	0.361642	CACNA1C
5	107543988	107643988	0.349163	CACNA1C
5	107544020	107644020	0.351755	CACNA1C
5	107544036	107644036	0.400251	CACNA1C
5	107544064	107644064	0.381057	CACNA1C
5	107544116	107644116	0.364239	CACNA1C
5	107544559	107644559	0.344543	CACNA1C
5	107545538	107645538	0.347784	CACNA1C
5	107545628	107645628	0.351835	CACNA1C
5	107545703	107645703	0.37374	CACNA1C
5	107545730	107645730	0.360404	CACNA1C
5	107546224	107646224	0.366698	CACNA1C
5	107546851	107646851	0.352591	CACNA1C
5	107546884	107646884	0.366864	CACNA1C
5	107546888	107646888	0.362602	CACNA1C
5	107546952	107646952	0.35691	CACNA1C
5	107546961	107646961	0.353069	CACNA1C
5	107773483	107873483	0.351121	CECR5
5	107773483	107873483	0.351121	CECR1
5	107773483	107873483	0.351121	LOC102178253
5	109817331	109917331	0.351984	ATF4
5	109817331	109917331	0.351984	RPS19BP1
5	109817331	109917331	0.351984	MGAT3
5	109817331	109917331	0.351984	MIEF1
5	109817331	109917331	0.351984	LOC106502153
5	109817331	109917331	0.351984	CACNA1I

5	109817455	109917455	0.348967	ATF4
5	109817455	109917455	0.348967	RPS19BP1
5	109817455	109917455	0.348967	MGAT3
5	109817455	109917455	0.348967	MIEF1
5	109817455	109917455	0.348967	LOC106502153
5	109817455	109917455	0.348967	CACNA1I
5	109817989	109917989	0.350504	ATF4
5	109817989	109917989	0.350504	RPS19BP1
5	109817989	109917989	0.350504	MGAT3
5	109817989	109917989	0.350504	MIEF1
5	109817989	109917989	0.350504	LOC106502153
5	109817989	109917989	0.350504	CACNA1I
5	109818110	109918110	0.350633	ATF4
5	109818110	109918110	0.350633	RPS19BP1
5	109818110	109918110	0.350633	MGAT3
5	109818110	109918110	0.350633	MIEF1
5	109818110	109918110	0.350633	LOC106502153
5	109818110	109918110	0.350633	CACNA1I
5	109818326	109918326	0.34408	ATF4
5	109818326	109918326	0.34408	RPS19BP1
5	109818326	109918326	0.34408	MGAT3
5	109818326	109918326	0.34408	MIEF1
5	109818326	109918326	0.34408	LOC106502153
5	109818326	109918326	0.34408	CACNA1I
5	109824556	109924556	0.347977	ATF4
5	109824556	109924556	0.347977	RPS19BP1
5	109824556	109924556	0.347977	MGAT3
5	109824556	109924556	0.347977	MIEF1
5	109824556	109924556	0.347977	LOC106502153
5	109824556	109924556	0.347977	CACNA1I
5	109825082	109925082	0.351786	ATF4
5	109825082	109925082	0.351786	RPS19BP1
5	109825082	109925082	0.351786	MGAT3
5	109825082	109925082	0.351786	MIEF1
5	109825082	109925082	0.351786	LOC106502153
5	109825082	109925082	0.351786	CACNA1I
5	109829782	109929782	0.34805	ATF4
5	109829782	109929782	0.34805	RPS19BP1
5	109829782	109929782	0.34805	MGAT3
5	109829782	109929782	0.34805	MIEF1
5	109829782	109929782	0.34805	LOC106502153
5	109829782	109929782	0.34805	CACNA1I
5	109830001	109930001	0.347204	ATF4
5	109830001	109930001	0.347204	RPS19BP1
5	109830001	109930001	0.347204	MGAT3
5	109830001	109930001	0.347204	MIEF1
5	109830001	109930001	0.347204	LOC106502153
5	109830001	109930001	0.347204	CACNA1I
5	109830215	109930215	0.345821	ATF4
5	109830215	109930215	0.345821	RPS19BP1
5	109830215	109930215	0.345821	MGAT3
5	109830215	109930215	0.345821	MIEF1
5	109830215	109930215	0.345821	LOC106502153
5	109830215	109930215	0.345821	CACNA1I
5	109830287	109930287	0.3446	ATF4
5	109830287	109930287	0.3446	RPS19BP1
5	109830287	109930287	0.3446	MGAT3
5	109830287	109930287	0.3446	MIEF1

5	109830287	109930287	0.3446	LOC106502153
5	109830287	109930287	0.3446	CACNA1I
5	109830585	109930585	0.396844	ATF4
5	109830585	109930585	0.396844	RPS19BP1
5	109830585	109930585	0.396844	MGAT3
5	109830585	109930585	0.396844	MIEF1
5	109830585	109930585	0.396844	LOC106502153
5	109830585	109930585	0.396844	CACNA1I
5	109830916	109930916	0.400721	ATF4
5	109830916	109930916	0.400721	RPS19BP1
5	109830916	109930916	0.400721	MGAT3
5	109830916	109930916	0.400721	MIEF1
5	109830916	109930916	0.400721	LOC106502153
5	109830916	109930916	0.400721	CACNA1I
5	109831390	109931390	0.388612	ATF4
5	109831390	109931390	0.388612	RPS19BP1
5	109831390	109931390	0.388612	MGAT3
5	109831390	109931390	0.388612	MIEF1
5	109831390	109931390	0.388612	LOC106502153
5	109831390	109931390	0.388612	CACNA1I
5	109831394	109931394	0.388797	ATF4
5	109831394	109931394	0.388797	RPS19BP1
5	109831394	109931394	0.388797	MGAT3
5	109831394	109931394	0.388797	MIEF1
5	109831394	109931394	0.388797	LOC106502153
5	109831394	109931394	0.388797	CACNA1I
5	109831567	109931567	0.389428	ATF4
5	109831567	109931567	0.389428	RPS19BP1
5	109831567	109931567	0.389428	MGAT3
5	109831567	109931567	0.389428	MIEF1
5	109831567	109931567	0.389428	LOC106502153
5	109831567	109931567	0.389428	CACNA1I
5	109831884	109931884	0.387097	ATF4
5	109831884	109931884	0.387097	RPS19BP1
5	109831884	109931884	0.387097	MGAT3
5	109831884	109931884	0.387097	MIEF1
5	109831884	109931884	0.387097	LOC106502153
5	109831884	109931884	0.387097	CACNA1I
5	109832122	109932122	0.407608	ATF4
5	109832122	109932122	0.407608	RPS19BP1
5	109832122	109932122	0.407608	MGAT3
5	109832122	109932122	0.407608	MIEF1
5	109832122	109932122	0.407608	LOC106502153
5	109832122	109932122	0.407608	CACNA1I
5	109832177	109932177	0.397474	ATF4
5	109832177	109932177	0.397474	RPS19BP1
5	109832177	109932177	0.397474	MGAT3
5	109832177	109932177	0.397474	MIEF1
5	109832177	109932177	0.397474	LOC106502153
5	109832177	109932177	0.397474	CACNA1I
5	109832198	109932198	0.410884	ATF4
5	109832198	109932198	0.410884	RPS19BP1
5	109832198	109932198	0.410884	MGAT3
5	109832198	109932198	0.410884	MIEF1
5	109832198	109932198	0.410884	LOC106502153
5	109832198	109932198	0.410884	CACNA1I
5	109832225	109932225	0.386055	ATF4
5	109832225	109932225	0.386055	RPS19BP1

5	109832225	109932225	0.386055	MGAT3
5	109832225	109932225	0.386055	MIEF1
5	109832225	109932225	0.386055	LOC106502153
5	109832225	109932225	0.386055	CACNA1I
5	109832291	109932291	0.379904	ATF4
5	109832291	109932291	0.379904	RPS19BP1
5	109832291	109932291	0.379904	MGAT3
5	109832291	109932291	0.379904	MIEF1
5	109832291	109932291	0.379904	LOC106502153
5	109832291	109932291	0.379904	CACNA1I
5	109832518	109932518	0.364325	ATF4
5	109832518	109932518	0.364325	RPS19BP1
5	109832518	109932518	0.364325	MGAT3
5	109832518	109932518	0.364325	MIEF1
5	109832518	109932518	0.364325	LOC106502153
5	109832518	109932518	0.364325	CACNA1I
5	109832591	109932591	0.364463	ATF4
5	109832591	109932591	0.364463	RPS19BP1
5	109832591	109932591	0.364463	MGAT3
5	109832591	109932591	0.364463	MIEF1
5	109832591	109932591	0.364463	LOC106502153
5	109832591	109932591	0.364463	CACNA1I
5	109833518	109933518	0.359452	ATF4
5	109833518	109933518	0.359452	RPS19BP1
5	109833518	109933518	0.359452	MGAT3
5	109833518	109933518	0.359452	MIEF1
5	109833518	109933518	0.359452	LOC106502153
5	109833518	109933518	0.359452	CACNA1I
5	109833779	109933779	0.379084	ATF4
5	109833779	109933779	0.379084	RPS19BP1
5	109833779	109933779	0.379084	MGAT3
5	109833779	109933779	0.379084	MIEF1
5	109833779	109933779	0.379084	LOC106502153
5	109833779	109933779	0.379084	CACNA1I
5	109833996	109933996	0.384271	ATF4
5	109833996	109933996	0.384271	RPS19BP1
5	109833996	109933996	0.384271	MGAT3
5	109833996	109933996	0.384271	MIEF1
5	109833996	109933996	0.384271	LOC106502153
5	109833996	109933996	0.384271	CACNA1I
5	109834539	109934539	0.366259	ATF4
5	109834539	109934539	0.366259	RPS19BP1
5	109834539	109934539	0.366259	MGAT3
5	109834539	109934539	0.366259	MIEF1
5	109834539	109934539	0.366259	LOC106502153
5	109834539	109934539	0.366259	CACNA1I
5	109834547	109934547	0.35865	ATF4
5	109834547	109934547	0.35865	RPS19BP1
5	109834547	109934547	0.35865	MGAT3
5	109834547	109934547	0.35865	MIEF1
5	109834547	109934547	0.35865	LOC106502153
5	109834547	109934547	0.35865	CACNA1I
5	109834564	109934564	0.350419	ATF4
5	109834564	109934564	0.350419	RPS19BP1
5	109834564	109934564	0.350419	MGAT3
5	109834564	109934564	0.350419	MIEF1
5	109834564	109934564	0.350419	LOC106502153
5	109834564	109934564	0.350419	CACNA1I

5	109834773	109934773	0.35266	ATF4
5	109834773	109934773	0.35266	RPS19BP1
5	109834773	109934773	0.35266	MGAT3
5	109834773	109934773	0.35266	MIEF1
5	109834773	109934773	0.35266	LOC106502153
5	109834773	109934773	0.35266	CACNA1I
5	109838122	109938122	0.351899	ATF4
5	109838122	109938122	0.351899	RPS19BP1
5	109838122	109938122	0.351899	MIEF1
5	109838122	109938122	0.351899	LOC106502153
5	109838122	109938122	0.351899	CACNA1I
5	109839782	109939782	0.34442	ATF4
5	109839782	109939782	0.34442	RPS19BP1
5	109839782	109939782	0.34442	MIEF1
5	109839782	109939782	0.34442	LOC106502153
5	109839782	109939782	0.34442	CACNA1I
5	109839785	109939785	0.34442	ATF4
5	109839785	109939785	0.34442	RPS19BP1
5	109839785	109939785	0.34442	MIEF1
5	109839785	109939785	0.34442	LOC106502153
5	109839785	109939785	0.34442	CACNA1I
5	109844304	109944304	0.344562	ATF4
5	109844304	109944304	0.344562	RPS19BP1
5	109844304	109944304	0.344562	MIEF1
5	109844304	109944304	0.344562	LOC106502153
5	109844304	109944304	0.344562	CACNA1I
5	109844662	109944662	0.360749	ATF4
5	109844662	109944662	0.360749	RPS19BP1
5	109844662	109944662	0.360749	MIEF1
5	109844662	109944662	0.360749	LOC106502153
5	109844662	109944662	0.360749	CACNA1I
5	109854331	109954331	0.342301	ATF4
5	109854331	109954331	0.342301	RPS19BP1
5	109854331	109954331	0.342301	MIEF1
5	109854331	109954331	0.342301	LOC106502153
5	109854331	109954331	0.342301	CACNA1I
5	109854379	109954379	0.377109	ATF4
5	109854379	109954379	0.377109	RPS19BP1
5	109854379	109954379	0.377109	MIEF1
5	109854379	109954379	0.377109	LOC106502153
5	109854379	109954379	0.377109	CACNA1I
5	109854416	109954416	0.353503	ATF4
5	109854416	109954416	0.353503	RPS19BP1
5	109854416	109954416	0.353503	MIEF1
5	109854416	109954416	0.353503	LOC106502153
5	109854416	109954416	0.353503	CACNA1I
5	109854475	109954475	0.352711	ATF4
5	109854475	109954475	0.352711	RPS19BP1
5	109854475	109954475	0.352711	MIEF1
5	109854475	109954475	0.352711	LOC106502153
5	109854475	109954475	0.352711	CACNA1I
5	109854509	109954509	0.351654	ATF4
5	109854509	109954509	0.351654	RPS19BP1
5	109854509	109954509	0.351654	MIEF1
5	109854509	109954509	0.351654	LOC106502153
5	109854509	109954509	0.351654	CACNA1I
5	109854528	109954528	0.345593	ATF4
5	109854528	109954528	0.345593	RPS19BP1

5	109854528	109954528	0.345593	MIEF1
5	109854528	109954528	0.345593	LOC106502153
5	109854528	109954528	0.345593	CACNA1I
5	109854600	109954600	0.349394	ATF4
5	109854600	109954600	0.349394	RPS19BP1
5	109854600	109954600	0.349394	MIEF1
5	109854600	109954600	0.349394	LOC106502153
5	109854600	109954600	0.349394	CACNA1I
5	109854799	109954799	0.344171	ATF4
5	109854799	109954799	0.344171	RPS19BP1
5	109854799	109954799	0.344171	MIEF1
5	109854799	109954799	0.344171	LOC106502153
5	109854799	109954799	0.344171	CACNA1I
5	109854957	109954957	0.344171	ATF4
5	109854957	109954957	0.344171	RPS19BP1
5	109854957	109954957	0.344171	MIEF1
5	109854957	109954957	0.344171	LOC106502153
5	109854957	109954957	0.344171	CACNA1I
5	109855332	109955332	0.344171	ATF4
5	109855332	109955332	0.344171	RPS19BP1
5	109855332	109955332	0.344171	MIEF1
5	109855332	109955332	0.344171	LOC106502153
5	109855332	109955332	0.344171	CACNA1I
5	109855730	109955730	0.35673	ATF4
5	109855730	109955730	0.35673	RPS19BP1
5	109855730	109955730	0.35673	MIEF1
5	109855730	109955730	0.35673	LOC106502153
5	109855730	109955730	0.35673	CACNA1I
5	109855981	109955981	0.343464	ATF4
5	109855981	109955981	0.343464	RPS19BP1
5	109855981	109955981	0.343464	MIEF1
5	109855981	109955981	0.343464	LOC106502153
5	109855981	109955981	0.343464	CACNA1I
5	109856278	109956278	0.343464	ATF4
5	109856278	109956278	0.343464	RPS19BP1
5	109856278	109956278	0.343464	MIEF1
5	109856278	109956278	0.343464	LOC106502153
5	109856278	109956278	0.343464	CACNA1I
5	109856286	109956286	0.35991	ATF4
5	109856286	109956286	0.35991	RPS19BP1
5	109856286	109956286	0.35991	MIEF1
5	109856286	109956286	0.35991	LOC106502153
5	109856286	109956286	0.35991	CACNA1I
5	109856377	109956377	0.344627	ATF4
5	109856377	109956377	0.344627	RPS19BP1
5	109856377	109956377	0.344627	MIEF1
5	109856377	109956377	0.344627	LOC106502153
5	109856377	109956377	0.344627	CACNA1I
5	109856570	109956570	0.347446	ATF4
5	109856570	109956570	0.347446	RPS19BP1
5	109856570	109956570	0.347446	MIEF1
5	109856570	109956570	0.347446	LOC106502153
5	109856570	109956570	0.347446	CACNA1I
5	109856652	109956652	0.34863	ATF4
5	109856652	109956652	0.34863	RPS19BP1
5	109856652	109956652	0.34863	MIEF1
5	109856652	109956652	0.34863	LOC106502153
5	109856652	109956652	0.34863	CACNA1I

5	109856744	109956744	0.34863	ATF4
5	109856744	109956744	0.34863	RPS19BP1
5	109856744	109956744	0.34863	MIEF1
5	109856744	109956744	0.34863	LOC106502153
5	109856744	109956744	0.34863	CACNA1I
5	109856939	109956939	0.348224	ATF4
5	109856939	109956939	0.348224	RPS19BP1
5	109856939	109956939	0.348224	MIEF1
5	109856939	109956939	0.348224	LOC106502153
5	109856939	109956939	0.348224	CACNA1I
5	109856972	109956972	0.3605	ATF4
5	109856972	109956972	0.3605	RPS19BP1
5	109856972	109956972	0.3605	MIEF1
5	109856972	109956972	0.3605	LOC106502153
5	109856972	109956972	0.3605	CACNA1I
5	109857153	109957153	0.356077	ATF4
5	109857153	109957153	0.356077	RPS19BP1
5	109857153	109957153	0.356077	MIEF1
5	109857153	109957153	0.356077	LOC106502153
5	109857153	109957153	0.356077	CACNA1I
5	109857199	109957199	0.346874	ATF4
5	109857199	109957199	0.346874	RPS19BP1
5	109857199	109957199	0.346874	MIEF1
5	109857199	109957199	0.346874	LOC106502153
5	109857199	109957199	0.346874	CACNA1I
5	109857377	109957377	0.37107	ATF4
5	109857377	109957377	0.37107	RPS19BP1
5	109857377	109957377	0.37107	MIEF1
5	109857377	109957377	0.37107	LOC106502153
5	109857377	109957377	0.37107	CACNA1I
5	109857871	109957871	0.34313	ATF4
5	109857871	109957871	0.34313	RPS19BP1
5	109857871	109957871	0.34313	MIEF1
5	109857871	109957871	0.34313	LOC106502153
5	109857871	109957871	0.34313	CACNA1I
5	109858064	109958064	0.349443	ATF4
5	109858064	109958064	0.349443	RPS19BP1
5	109858064	109958064	0.349443	MIEF1
5	109858064	109958064	0.349443	LOC106502153
5	109858064	109958064	0.349443	CACNA1I
5	109859278	109959278	0.363503	ATF4
5	109859278	109959278	0.363503	RPS19BP1
5	109859278	109959278	0.363503	MIEF1
5	109859278	109959278	0.363503	LOC106502153
5	109859278	109959278	0.363503	CACNA1I
5	109860738	109960738	0.350604	ATF4
5	109860738	109960738	0.350604	RPS19BP1
5	109860738	109960738	0.350604	MIEF1
5	109860738	109960738	0.350604	LOC106502153
5	109860738	109960738	0.350604	CACNA1I
5	109862865	109962865	0.342498	ATF4
5	109862865	109962865	0.342498	RPS19BP1
5	109862865	109962865	0.342498	MIEF1
5	109862865	109962865	0.342498	LOC106502153
5	109862865	109962865	0.342498	CACNA1I
5	109862874	109962874	0.347439	ATF4
5	109862874	109962874	0.347439	RPS19BP1
5	109862874	109962874	0.347439	MIEF1

5	109862874	109962874	0.347439	LOC106502153
5	109862874	109962874	0.347439	CACNA1I
5	109917329	110017329	0.356956	CACNA1I
5	109917416	110017416	0.358472	CACNA1I
5	109917588	110017588	0.414392	CACNA1I
5	109917988	110017988	0.402248	CACNA1I
5	109918384	110018384	0.400199	CACNA1I
5	109918394	110018394	0.383695	CACNA1I
5	109918573	110018573	0.384977	CACNA1I
5	109918597	110018597	0.403224	CACNA1I
5	109918966	110018966	0.391656	CACNA1I
5	109919342	110019342	0.394147	CACNA1I
5	109919471	110019471	0.389817	CACNA1I
5	109919477	110019477	0.389817	CACNA1I
5	109919550	110019550	0.391211	CACNA1I
5	109919719	110019719	0.392608	CACNA1I
5	109919745	110019745	0.392608	CACNA1I
5	109919779	110019779	0.392608	CACNA1I
5	109919805	110019805	0.401371	CACNA1I
5	109919920	110019920	0.398402	CACNA1I
5	109920449	110020449	0.373784	CACNA1I
5	109920534	110020534	0.380077	CACNA1I
5	109920819	110020819	0.384495	CACNA1I
5	109921146	110021146	0.378409	CACNA1I
5	109921511	110021511	0.410426	CACNA1I
5	109921844	110021844	0.407655	CACNA1I
5	110014350	110114350	0.348269	CACNA1I
5	110014350	110114350	0.348269	ENTHD1
5	111969419	112069419	0.351322	SMDT1
5	111969419	112069419	0.351322	LOC102169285
5	111969419	112069419	0.351322	NAGA
5	111969419	112069419	0.351322	FAM109B
5	111969419	112069419	0.351322	NDUFA6
5	111969419	112069419	0.351322	LOC102177333
5	111969419	112069419	0.351322	LOC102169002
5	111969660	112069660	0.369931	SMDT1
5	111969660	112069660	0.369931	LOC102169285
5	111969660	112069660	0.369931	NAGA
5	111969660	112069660	0.369931	FAM109B
5	111969660	112069660	0.369931	NDUFA6
5	111969660	112069660	0.369931	LOC102177333
5	111969660	112069660	0.369931	LOC102169002
5	111969897	112069897	0.367661	SMDT1
5	111969897	112069897	0.367661	LOC102169285
5	111969897	112069897	0.367661	NAGA
5	111969897	112069897	0.367661	FAM109B
5	111969897	112069897	0.367661	NDUFA6
5	111969897	112069897	0.367661	LOC102177333
5	111969897	112069897	0.367661	LOC102169002
5	111971231	112071231	0.355277	SMDT1
5	111971231	112071231	0.355277	LOC102169285
5	111971231	112071231	0.355277	NAGA
5	111971231	112071231	0.355277	FAM109B
5	111971231	112071231	0.355277	NDUFA6
5	111971231	112071231	0.355277	LOC102177333
5	111971231	112071231	0.355277	LOC102169002
5	111971283	112071283	0.367728	SMDT1
5	111971283	112071283	0.367728	LOC102169285

5	111971283	112071283	0.367728	NAGA
5	111971283	112071283	0.367728	FAM109B
5	111971283	112071283	0.367728	NDUFA6
5	111971283	112071283	0.367728	LOC102177333
5	111971283	112071283	0.367728	LOC102169002
5	111971794	112071794	0.360202	SMDT1
5	111971794	112071794	0.360202	LOC102169285
5	111971794	112071794	0.360202	NAGA
5	111971794	112071794	0.360202	FAM109B
5	111971794	112071794	0.360202	NDUFA6
5	111971794	112071794	0.360202	LOC102177333
5	111971794	112071794	0.360202	LOC102169002
5	111972861	112072861	0.35929	SMDT1
5	111972861	112072861	0.35929	LOC102169285
5	111972861	112072861	0.35929	NAGA
5	111972861	112072861	0.35929	FAM109B
5	111972861	112072861	0.35929	NDUFA6
5	111972861	112072861	0.35929	LOC102177333
5	111972861	112072861	0.35929	LOC102169002
5	111972925	112072925	0.372515	SMDT1
5	111972925	112072925	0.372515	LOC102169285
5	111972925	112072925	0.372515	NAGA
5	111972925	112072925	0.372515	FAM109B
5	111972925	112072925	0.372515	NDUFA6
5	111972925	112072925	0.372515	LOC102177333
5	111972925	112072925	0.372515	LOC102169002
5	111972998	112072998	0.373848	SMDT1
5	111972998	112072998	0.373848	LOC102169285
5	111972998	112072998	0.373848	NAGA
5	111972998	112072998	0.373848	FAM109B
5	111972998	112072998	0.373848	NDUFA6
5	111972998	112072998	0.373848	LOC102177333
5	111972998	112072998	0.373848	LOC102169002
5	111973458	112073458	0.371878	SMDT1
5	111973458	112073458	0.371878	LOC102169285
5	111973458	112073458	0.371878	NAGA
5	111973458	112073458	0.371878	FAM109B
5	111973458	112073458	0.371878	NDUFA6
5	111973458	112073458	0.371878	LOC102177333
5	111973458	112073458	0.371878	LOC102169002
5	111973754	112073754	0.370124	SMDT1
5	111973754	112073754	0.370124	LOC102169285
5	111973754	112073754	0.370124	NAGA
5	111973754	112073754	0.370124	FAM109B
5	111973754	112073754	0.370124	NDUFA6
5	111973754	112073754	0.370124	LOC102177333
5	111973754	112073754	0.370124	LOC102169002
5	111973767	112073767	0.373848	SMDT1
5	111973767	112073767	0.373848	LOC102169285
5	111973767	112073767	0.373848	NAGA
5	111973767	112073767	0.373848	FAM109B
5	111973767	112073767	0.373848	NDUFA6
5	111973767	112073767	0.373848	LOC102177333
5	111973767	112073767	0.373848	LOC102169002
5	111973856	112073856	0.376165	SMDT1
5	111973856	112073856	0.376165	LOC102169285
5	111973856	112073856	0.376165	NAGA
5	111973856	112073856	0.376165	FAM109B

5	111973856	112073856	0.376165	NDUFA6
5	111973856	112073856	0.376165	LOC102177333
5	111973856	112073856	0.376165	LOC102169002
5	111973924	112073924	0.375863	SMDT1
5	111973924	112073924	0.375863	LOC102169285
5	111973924	112073924	0.375863	NAGA
5	111973924	112073924	0.375863	FAM109B
5	111973924	112073924	0.375863	NDUFA6
5	111973924	112073924	0.375863	LOC102177333
5	111973924	112073924	0.375863	LOC102169002
5	111974094	112074094	0.374513	SMDT1
5	111974094	112074094	0.374513	LOC102169285
5	111974094	112074094	0.374513	NAGA
5	111974094	112074094	0.374513	FAM109B
5	111974094	112074094	0.374513	NDUFA6
5	111974094	112074094	0.374513	LOC102177333
5	111974094	112074094	0.374513	LOC102169002
5	111974171	112074171	0.374513	SMDT1
5	111974171	112074171	0.374513	LOC102169285
5	111974171	112074171	0.374513	NAGA
5	111974171	112074171	0.374513	FAM109B
5	111974171	112074171	0.374513	NDUFA6
5	111974171	112074171	0.374513	LOC102177333
5	111974171	112074171	0.374513	LOC102169002
5	111974386	112074386	0.36496	SMDT1
5	111974386	112074386	0.36496	LOC102169285
5	111974386	112074386	0.36496	NAGA
5	111974386	112074386	0.36496	FAM109B
5	111974386	112074386	0.36496	NDUFA6
5	111974386	112074386	0.36496	LOC102177333
5	111974386	112074386	0.36496	LOC102169002
5	111974611	112074611	0.365812	SMDT1
5	111974611	112074611	0.365812	LOC102169285
5	111974611	112074611	0.365812	NAGA
5	111974611	112074611	0.365812	FAM109B
5	111974611	112074611	0.365812	NDUFA6
5	111974611	112074611	0.365812	LOC102177333
5	111974611	112074611	0.365812	LOC102169002
5	111974705	112074705	0.365812	SMDT1
5	111974705	112074705	0.365812	LOC102169285
5	111974705	112074705	0.365812	NAGA
5	111974705	112074705	0.365812	FAM109B
5	111974705	112074705	0.365812	NDUFA6
5	111974705	112074705	0.365812	LOC102177333
5	111974705	112074705	0.365812	LOC102169002
5	111975094	112075094	0.367896	SMDT1
5	111975094	112075094	0.367896	LOC102169285
5	111975094	112075094	0.367896	NAGA
5	111975094	112075094	0.367896	FAM109B
5	111975094	112075094	0.367896	NDUFA6
5	111975094	112075094	0.367896	LOC102177333
5	111975094	112075094	0.367896	LOC102169002
5	111975108	112075108	0.369452	SMDT1
5	111975108	112075108	0.369452	LOC102169285
5	111975108	112075108	0.369452	NAGA
5	111975108	112075108	0.369452	FAM109B
5	111975108	112075108	0.369452	NDUFA6
5	111975108	112075108	0.369452	LOC102177333

5	111975108	112075108	0.369452	LOC102169002
5	111975753	112075753	0.369452	SMDT1
5	111975753	112075753	0.369452	LOC102169285
5	111975753	112075753	0.369452	NAGA
5	111975753	112075753	0.369452	FAM109B
5	111975753	112075753	0.369452	NDUFA6
5	111975753	112075753	0.369452	LOC102177333
5	111975753	112075753	0.369452	LOC102169002
5	111975979	112075979	0.38141	SMDT1
5	111975979	112075979	0.38141	LOC102169285
5	111975979	112075979	0.38141	NAGA
5	111975979	112075979	0.38141	FAM109B
5	111975979	112075979	0.38141	NDUFA6
5	111975979	112075979	0.38141	LOC102177333
5	111975979	112075979	0.38141	LOC102169002
5	111975988	112075988	0.369412	SMDT1
5	111975988	112075988	0.369412	LOC102169285
5	111975988	112075988	0.369412	NAGA
5	111975988	112075988	0.369412	FAM109B
5	111975988	112075988	0.369412	NDUFA6
5	111975988	112075988	0.369412	LOC102177333
5	111975988	112075988	0.369412	LOC102169002
5	111976132	112076132	0.374459	SMDT1
5	111976132	112076132	0.374459	LOC102169285
5	111976132	112076132	0.374459	NAGA
5	111976132	112076132	0.374459	FAM109B
5	111976132	112076132	0.374459	NDUFA6
5	111976132	112076132	0.374459	TCF20
5	111976132	112076132	0.374459	LOC102177333
5	111976132	112076132	0.374459	LOC102169002
5	111976143	112076143	0.377831	SMDT1
5	111976143	112076143	0.377831	LOC102169285
5	111976143	112076143	0.377831	NAGA
5	111976143	112076143	0.377831	FAM109B
5	111976143	112076143	0.377831	NDUFA6
5	111976143	112076143	0.377831	TCF20
5	111976143	112076143	0.377831	LOC102177333
5	111976143	112076143	0.377831	LOC102169002
5	111976167	112076167	0.371015	SMDT1
5	111976167	112076167	0.371015	LOC102169285
5	111976167	112076167	0.371015	NAGA
5	111976167	112076167	0.371015	FAM109B
5	111976167	112076167	0.371015	NDUFA6
5	111976167	112076167	0.371015	TCF20
5	111976167	112076167	0.371015	LOC102177333
5	111976167	112076167	0.371015	LOC102169002
5	111976935	112076935	0.345007	SMDT1
5	111976935	112076935	0.345007	LOC102169285
5	111976935	112076935	0.345007	NAGA
5	111976935	112076935	0.345007	FAM109B
5	111976935	112076935	0.345007	NDUFA6
5	111976935	112076935	0.345007	TCF20
5	111976935	112076935	0.345007	LOC102177333
5	111976935	112076935	0.345007	LOC102169002
5	111977172	112077172	0.363605	SMDT1
5	111977172	112077172	0.363605	LOC102169285
5	111977172	112077172	0.363605	NAGA
5	111977172	112077172	0.363605	FAM109B

5	111977172	112077172	0.363605	NDUFA6
5	111977172	112077172	0.363605	TCF20
5	111977172	112077172	0.363605	LOC102177333
5	111977172	112077172	0.363605	LOC102169002
5	111977211	112077211	0.371227	SMDT1
5	111977211	112077211	0.371227	LOC102169285
5	111977211	112077211	0.371227	NAGA
5	111977211	112077211	0.371227	FAM109B
5	111977211	112077211	0.371227	NDUFA6
5	111977211	112077211	0.371227	TCF20
5	111977211	112077211	0.371227	LOC102177333
5	111977211	112077211	0.371227	LOC102169002
5	111977472	112077472	0.361122	SMDT1
5	111977472	112077472	0.361122	LOC102169285
5	111977472	112077472	0.361122	NAGA
5	111977472	112077472	0.361122	FAM109B
5	111977472	112077472	0.361122	NDUFA6
5	111977472	112077472	0.361122	TCF20
5	111977472	112077472	0.361122	LOC102177333
5	111977472	112077472	0.361122	LOC102169002
5	111977575	112077575	0.354259	SMDT1
5	111977575	112077575	0.354259	LOC102169285
5	111977575	112077575	0.354259	NAGA
5	111977575	112077575	0.354259	FAM109B
5	111977575	112077575	0.354259	NDUFA6
5	111977575	112077575	0.354259	TCF20
5	111977575	112077575	0.354259	LOC102177333
5	111977575	112077575	0.354259	LOC102169002
5	111977809	112077809	0.364392	SMDT1
5	111977809	112077809	0.364392	LOC102169285
5	111977809	112077809	0.364392	NAGA
5	111977809	112077809	0.364392	FAM109B
5	111977809	112077809	0.364392	NDUFA6
5	111977809	112077809	0.364392	TCF20
5	111977809	112077809	0.364392	LOC102177333
5	111977809	112077809	0.364392	LOC102169002
5	111978022	112078022	0.365223	SMDT1
5	111978022	112078022	0.365223	LOC102169285
5	111978022	112078022	0.365223	NAGA
5	111978022	112078022	0.365223	FAM109B
5	111978022	112078022	0.365223	NDUFA6
5	111978022	112078022	0.365223	TCF20
5	111978022	112078022	0.365223	LOC102177333
5	111978022	112078022	0.365223	LOC102169002
5	111978055	112078055	0.366218	SMDT1
5	111978055	112078055	0.366218	LOC102169285
5	111978055	112078055	0.366218	NAGA
5	111978055	112078055	0.366218	FAM109B
5	111978055	112078055	0.366218	NDUFA6
5	111978055	112078055	0.366218	TCF20
5	111978055	112078055	0.366218	LOC102177333
5	111978055	112078055	0.366218	LOC102169002
5	111978173	112078173	0.360398	SMDT1
5	111978173	112078173	0.360398	LOC102169285
5	111978173	112078173	0.360398	NAGA
5	111978173	112078173	0.360398	FAM109B
5	111978173	112078173	0.360398	NDUFA6
5	111978173	112078173	0.360398	TCF20

5	111978173	112078173	0.360398	LOC102177333
5	111978173	112078173	0.360398	LOC102169002
5	111978850	112078850	0.361162	SMDT1
5	111978850	112078850	0.361162	LOC102169285
5	111978850	112078850	0.361162	NAGA
5	111978850	112078850	0.361162	FAM109B
5	111978850	112078850	0.361162	NDUFA6
5	111978850	112078850	0.361162	TCF20
5	111978850	112078850	0.361162	LOC102177333
5	111978850	112078850	0.361162	LOC102169002
5	111978871	112078871	0.367161	SMDT1
5	111978871	112078871	0.367161	LOC102169285
5	111978871	112078871	0.367161	NAGA
5	111978871	112078871	0.367161	FAM109B
5	111978871	112078871	0.367161	NDUFA6
5	111978871	112078871	0.367161	TCF20
5	111978871	112078871	0.367161	LOC102177333
5	111978871	112078871	0.367161	LOC102169002
5	111979471	112079471	0.358065	SMDT1
5	111979471	112079471	0.358065	LOC102169285
5	111979471	112079471	0.358065	NAGA
5	111979471	112079471	0.358065	FAM109B
5	111979471	112079471	0.358065	NDUFA6
5	111979471	112079471	0.358065	TCF20
5	111979471	112079471	0.358065	LOC102177333
5	111979471	112079471	0.358065	LOC102169002
5	111979578	112079578	0.359403	SMDT1
5	111979578	112079578	0.359403	LOC102169285
5	111979578	112079578	0.359403	NAGA
5	111979578	112079578	0.359403	FAM109B
5	111979578	112079578	0.359403	NDUFA6
5	111979578	112079578	0.359403	TCF20
5	111979578	112079578	0.359403	LOC102177333
5	111979578	112079578	0.359403	LOC102169002
5	111979955	112079955	0.36095	SMDT1
5	111979955	112079955	0.36095	LOC102169285
5	111979955	112079955	0.36095	NAGA
5	111979955	112079955	0.36095	FAM109B
5	111979955	112079955	0.36095	NDUFA6
5	111979955	112079955	0.36095	TCF20
5	111979955	112079955	0.36095	LOC102177333
5	111979955	112079955	0.36095	LOC102169002
5	111980040	112080040	0.360438	SMDT1
5	111980040	112080040	0.360438	LOC102169285
5	111980040	112080040	0.360438	NAGA
5	111980040	112080040	0.360438	FAM109B
5	111980040	112080040	0.360438	NDUFA6
5	111980040	112080040	0.360438	TCF20
5	111980040	112080040	0.360438	LOC102177333
5	111980040	112080040	0.360438	LOC102169002
5	111980248	112080248	0.358706	SMDT1
5	111980248	112080248	0.358706	LOC102169285
5	111980248	112080248	0.358706	NAGA
5	111980248	112080248	0.358706	FAM109B
5	111980248	112080248	0.358706	NDUFA6
5	111980248	112080248	0.358706	TCF20
5	111980248	112080248	0.358706	LOC102177333
5	111980248	112080248	0.358706	LOC102169002

5	111980597	112080597	0.361659	SMDT1
5	111980597	112080597	0.361659	LOC102169285
5	111980597	112080597	0.361659	NAGA
5	111980597	112080597	0.361659	FAM109B
5	111980597	112080597	0.361659	NDUFA6
5	111980597	112080597	0.361659	TCF20
5	111980597	112080597	0.361659	LOC102177333
5	111980597	112080597	0.361659	LOC102169002
5	111980680	112080680	0.385921	SMDT1
5	111980680	112080680	0.385921	LOC102169285
5	111980680	112080680	0.385921	NAGA
5	111980680	112080680	0.385921	FAM109B
5	111980680	112080680	0.385921	NDUFA6
5	111980680	112080680	0.385921	TCF20
5	111980680	112080680	0.385921	LOC102177333
5	111980680	112080680	0.385921	LOC102169002
5	111980732	112080732	0.377821	SMDT1
5	111980732	112080732	0.377821	LOC102169285
5	111980732	112080732	0.377821	NAGA
5	111980732	112080732	0.377821	FAM109B
5	111980732	112080732	0.377821	NDUFA6
5	111980732	112080732	0.377821	TCF20
5	111980732	112080732	0.377821	LOC102177333
5	111980732	112080732	0.377821	LOC102169002
5	111981407	112081407	0.379932	SMDT1
5	111981407	112081407	0.379932	LOC102169285
5	111981407	112081407	0.379932	NAGA
5	111981407	112081407	0.379932	FAM109B
5	111981407	112081407	0.379932	NDUFA6
5	111981407	112081407	0.379932	TCF20
5	111981407	112081407	0.379932	LOC102177333
5	111981407	112081407	0.379932	LOC102169002
5	111981687	112081687	0.377743	SMDT1
5	111981687	112081687	0.377743	LOC102169285
5	111981687	112081687	0.377743	NAGA
5	111981687	112081687	0.377743	FAM109B
5	111981687	112081687	0.377743	NDUFA6
5	111981687	112081687	0.377743	TCF20
5	111981687	112081687	0.377743	LOC102177333
5	111981687	112081687	0.377743	LOC102169002
5	111981717	112081717	0.379244	SMDT1
5	111981717	112081717	0.379244	LOC102169285
5	111981717	112081717	0.379244	NAGA
5	111981717	112081717	0.379244	FAM109B
5	111981717	112081717	0.379244	NDUFA6
5	111981717	112081717	0.379244	TCF20
5	111981717	112081717	0.379244	LOC102177333
5	111981717	112081717	0.379244	LOC102169002
5	111982174	112082174	0.379047	SMDT1
5	111982174	112082174	0.379047	LOC102169285
5	111982174	112082174	0.379047	NAGA
5	111982174	112082174	0.379047	FAM109B
5	111982174	112082174	0.379047	NDUFA6
5	111982174	112082174	0.379047	TCF20
5	111982174	112082174	0.379047	LOC102177333
5	111982174	112082174	0.379047	LOC102169002
5	111982361	112082361	0.379047	SMDT1
5	111982361	112082361	0.379047	LOC102169285

5	111982361	112082361	0.379047	NAGA
5	111982361	112082361	0.379047	FAM109B
5	111982361	112082361	0.379047	NDUFA6
5	111982361	112082361	0.379047	TCF20
5	111982361	112082361	0.379047	LOC102177333
5	111982361	112082361	0.379047	LOC102169002
5	111982463	112082463	0.378198	SMDT1
5	111982463	112082463	0.378198	LOC102169285
5	111982463	112082463	0.378198	NAGA
5	111982463	112082463	0.378198	FAM109B
5	111982463	112082463	0.378198	NDUFA6
5	111982463	112082463	0.378198	TCF20
5	111982463	112082463	0.378198	LOC102177333
5	111982463	112082463	0.378198	LOC102169002
5	111982812	112082812	0.377593	SMDT1
5	111982812	112082812	0.377593	LOC102169285
5	111982812	112082812	0.377593	NAGA
5	111982812	112082812	0.377593	FAM109B
5	111982812	112082812	0.377593	NDUFA6
5	111982812	112082812	0.377593	TCF20
5	111982812	112082812	0.377593	LOC102177333
5	111982812	112082812	0.377593	LOC102169002
5	111983049	112083049	0.364585	SMDT1
5	111983049	112083049	0.364585	LOC102169285
5	111983049	112083049	0.364585	NAGA
5	111983049	112083049	0.364585	FAM109B
5	111983049	112083049	0.364585	NDUFA6
5	111983049	112083049	0.364585	TCF20
5	111983049	112083049	0.364585	LOC102177333
5	111983049	112083049	0.364585	LOC102169002
5	111983386	112083386	0.350774	SMDT1
5	111983386	112083386	0.350774	LOC102169285
5	111983386	112083386	0.350774	NAGA
5	111983386	112083386	0.350774	FAM109B
5	111983386	112083386	0.350774	NDUFA6
5	111983386	112083386	0.350774	TCF20
5	111983386	112083386	0.350774	LOC102177333
5	111983386	112083386	0.350774	LOC102169002
5	111983522	112083522	0.354297	SMDT1
5	111983522	112083522	0.354297	LOC102169285
5	111983522	112083522	0.354297	NAGA
5	111983522	112083522	0.354297	FAM109B
5	111983522	112083522	0.354297	NDUFA6
5	111983522	112083522	0.354297	TCF20
5	111983522	112083522	0.354297	LOC102177333
5	111983522	112083522	0.354297	LOC102169002
5	111983547	112083547	0.358204	SMDT1
5	111983547	112083547	0.358204	LOC102169285
5	111983547	112083547	0.358204	NAGA
5	111983547	112083547	0.358204	FAM109B
5	111983547	112083547	0.358204	NDUFA6
5	111983547	112083547	0.358204	TCF20
5	111983547	112083547	0.358204	LOC102177333
5	111983547	112083547	0.358204	LOC102169002
5	111983592	112083592	0.35723	SMDT1
5	111983592	112083592	0.35723	LOC102169285
5	111983592	112083592	0.35723	NAGA
5	111983592	112083592	0.35723	FAM109B

5	111983592	112083592	0.35723	NDUFA6
5	111983592	112083592	0.35723	TCF20
5	111983592	112083592	0.35723	LOC102177333
5	111983592	112083592	0.35723	LOC102169002
5	111984293	112084293	0.355516	SMDT1
5	111984293	112084293	0.355516	LOC102169285
5	111984293	112084293	0.355516	NAGA
5	111984293	112084293	0.355516	FAM109B
5	111984293	112084293	0.355516	NDUFA6
5	111984293	112084293	0.355516	TCF20
5	111984293	112084293	0.355516	LOC102177333
5	111984293	112084293	0.355516	LOC102169002
5	111984387	112084387	0.354544	SMDT1
5	111984387	112084387	0.354544	LOC102169285
5	111984387	112084387	0.354544	NAGA
5	111984387	112084387	0.354544	FAM109B
5	111984387	112084387	0.354544	NDUFA6
5	111984387	112084387	0.354544	TCF20
5	111984387	112084387	0.354544	LOC102177333
5	111984387	112084387	0.354544	LOC102169002
5	111984628	112084628	0.354737	SMDT1
5	111984628	112084628	0.354737	LOC102169285
5	111984628	112084628	0.354737	NAGA
5	111984628	112084628	0.354737	FAM109B
5	111984628	112084628	0.354737	NDUFA6
5	111984628	112084628	0.354737	TCF20
5	111984628	112084628	0.354737	LOC102177333
5	111984628	112084628	0.354737	LOC102169002
5	111984779	112084779	0.360435	SMDT1
5	111984779	112084779	0.360435	LOC102169285
5	111984779	112084779	0.360435	NAGA
5	111984779	112084779	0.360435	FAM109B
5	111984779	112084779	0.360435	NDUFA6
5	111984779	112084779	0.360435	TCF20
5	111984779	112084779	0.360435	LOC102177333
5	111984779	112084779	0.360435	LOC102169002
5	111984780	112084780	0.358421	SMDT1
5	111984780	112084780	0.358421	LOC102169285
5	111984780	112084780	0.358421	NAGA
5	111984780	112084780	0.358421	FAM109B
5	111984780	112084780	0.358421	NDUFA6
5	111984780	112084780	0.358421	TCF20
5	111984780	112084780	0.358421	LOC102177333
5	111984780	112084780	0.358421	LOC102169002
5	111984815	112084815	0.358358	SMDT1
5	111984815	112084815	0.358358	LOC102169285
5	111984815	112084815	0.358358	NAGA
5	111984815	112084815	0.358358	FAM109B
5	111984815	112084815	0.358358	NDUFA6
5	111984815	112084815	0.358358	TCF20
5	111984815	112084815	0.358358	LOC102177333
5	111984815	112084815	0.358358	LOC102169002
5	111984853	112084853	0.353794	SMDT1
5	111984853	112084853	0.353794	LOC102169285
5	111984853	112084853	0.353794	NAGA
5	111984853	112084853	0.353794	FAM109B
5	111984853	112084853	0.353794	NDUFA6
5	111984853	112084853	0.353794	TCF20

5	111984853	112084853	0.353794	LOC102177333
5	111984853	112084853	0.353794	LOC102169002
5	111985702	112085702	0.353845	SMDT1
5	111985702	112085702	0.353845	LOC102169285
5	111985702	112085702	0.353845	NAGA
5	111985702	112085702	0.353845	FAM109B
5	111985702	112085702	0.353845	NDUFA6
5	111985702	112085702	0.353845	TCF20
5	111985702	112085702	0.353845	LOC102177333
5	111985702	112085702	0.353845	LOC102169002
5	111985864	112085864	0.353831	SMDT1
5	111985864	112085864	0.353831	LOC102169285
5	111985864	112085864	0.353831	NAGA
5	111985864	112085864	0.353831	FAM109B
5	111985864	112085864	0.353831	NDUFA6
5	111985864	112085864	0.353831	TCF20
5	111985864	112085864	0.353831	LOC102177333
5	111985864	112085864	0.353831	LOC102169002
5	111986044	112086044	0.359507	SMDT1
5	111986044	112086044	0.359507	LOC102169285
5	111986044	112086044	0.359507	NAGA
5	111986044	112086044	0.359507	FAM109B
5	111986044	112086044	0.359507	NDUFA6
5	111986044	112086044	0.359507	TCF20
5	111986044	112086044	0.359507	LOC102177333
5	111986044	112086044	0.359507	LOC102169002
5	111989578	112089578	0.342899	SMDT1
5	111989578	112089578	0.342899	LOC102169285
5	111989578	112089578	0.342899	NAGA
5	111989578	112089578	0.342899	FAM109B
5	111989578	112089578	0.342899	NDUFA6
5	111989578	112089578	0.342899	TCF20
5	111989578	112089578	0.342899	LOC102177333
5	111989578	112089578	0.342899	LOC102169002
5	111989580	112089580	0.342899	SMDT1
5	111989580	112089580	0.342899	LOC102169285
5	111989580	112089580	0.342899	NAGA
5	111989580	112089580	0.342899	FAM109B
5	111989580	112089580	0.342899	NDUFA6
5	111989580	112089580	0.342899	TCF20
5	111989580	112089580	0.342899	LOC102177333
5	111989580	112089580	0.342899	LOC102169002
5	111989789	112089789	0.345118	SMDT1
5	111989789	112089789	0.345118	LOC102169285
5	111989789	112089789	0.345118	NAGA
5	111989789	112089789	0.345118	FAM109B
5	111989789	112089789	0.345118	NDUFA6
5	111989789	112089789	0.345118	TCF20
5	111989789	112089789	0.345118	LOC102177333
5	111989789	112089789	0.345118	LOC102169002
5	111989794	112089794	0.358959	SMDT1
5	111989794	112089794	0.358959	LOC102169285
5	111989794	112089794	0.358959	NAGA
5	111989794	112089794	0.358959	FAM109B
5	111989794	112089794	0.358959	NDUFA6
5	111989794	112089794	0.358959	TCF20
5	111989794	112089794	0.358959	LOC102177333
5	111989794	112089794	0.358959	LOC102169002

5	111989976	112089976	0.350171	SMDT1
5	111989976	112089976	0.350171	LOC102169285
5	111989976	112089976	0.350171	NAGA
5	111989976	112089976	0.350171	FAM109B
5	111989976	112089976	0.350171	NDUFA6
5	111989976	112089976	0.350171	TCF20
5	111989976	112089976	0.350171	LOC102177333
5	111989976	112089976	0.350171	LOC102169002
5	111990068	112090068	0.367363	SMDT1
5	111990068	112090068	0.367363	LOC102169285
5	111990068	112090068	0.367363	NAGA
5	111990068	112090068	0.367363	FAM109B
5	111990068	112090068	0.367363	NDUFA6
5	111990068	112090068	0.367363	TCF20
5	111990068	112090068	0.367363	LOC102177333
5	111990068	112090068	0.367363	LOC102169002
5	111990088	112090088	0.367363	SMDT1
5	111990088	112090088	0.367363	LOC102169285
5	111990088	112090088	0.367363	NAGA
5	111990088	112090088	0.367363	FAM109B
5	111990088	112090088	0.367363	NDUFA6
5	111990088	112090088	0.367363	TCF20
5	111990088	112090088	0.367363	LOC102177333
5	111990088	112090088	0.367363	LOC102169002
5	111990231	112090231	0.364104	SMDT1
5	111990231	112090231	0.364104	LOC102169285
5	111990231	112090231	0.364104	NAGA
5	111990231	112090231	0.364104	FAM109B
5	111990231	112090231	0.364104	NDUFA6
5	111990231	112090231	0.364104	TCF20
5	111990231	112090231	0.364104	LOC102177333
5	111990231	112090231	0.364104	LOC102169002
5	111991024	112091024	0.359604	SMDT1
5	111991024	112091024	0.359604	LOC102169285
5	111991024	112091024	0.359604	NAGA
5	111991024	112091024	0.359604	FAM109B
5	111991024	112091024	0.359604	NDUFA6
5	111991024	112091024	0.359604	TCF20
5	111991024	112091024	0.359604	LOC102177333
5	111991024	112091024	0.359604	LOC102169002
5	111991323	112091323	0.352637	SMDT1
5	111991323	112091323	0.352637	LOC102169285
5	111991323	112091323	0.352637	NAGA
5	111991323	112091323	0.352637	FAM109B
5	111991323	112091323	0.352637	NDUFA6
5	111991323	112091323	0.352637	TCF20
5	111991323	112091323	0.352637	LOC102177333
5	111991323	112091323	0.352637	LOC102169002
5	111991328	112091328	0.352543	SMDT1
5	111991328	112091328	0.352543	LOC102169285
5	111991328	112091328	0.352543	NAGA
5	111991328	112091328	0.352543	FAM109B
5	111991328	112091328	0.352543	NDUFA6
5	111991328	112091328	0.352543	TCF20
5	111991328	112091328	0.352543	LOC102177333
5	111991328	112091328	0.352543	LOC102169002
5	111991938	112091938	0.363108	SMDT1
5	111991938	112091938	0.363108	LOC102169285

5	111991938	112091938	0.363108	NAGA
5	111991938	112091938	0.363108	FAM109B
5	111991938	112091938	0.363108	NDUFA6
5	111991938	112091938	0.363108	TCF20
5	111991938	112091938	0.363108	LOC102177333
5	111991938	112091938	0.363108	LOC102169002
5	111993198	112093198	0.488947	SMDT1
5	111993198	112093198	0.488947	LOC102169285
5	111993198	112093198	0.488947	FAM109B
5	111993198	112093198	0.488947	NDUFA6
5	111993198	112093198	0.488947	TCF20
5	111993198	112093198	0.488947	LOC102177333
5	111993198	112093198	0.488947	LOC102169002
5	111993275	112093275	0.461071	SMDT1
5	111993275	112093275	0.461071	LOC102169285
5	111993275	112093275	0.461071	FAM109B
5	111993275	112093275	0.461071	NDUFA6
5	111993275	112093275	0.461071	TCF20
5	111993275	112093275	0.461071	LOC102177333
5	111993275	112093275	0.461071	LOC102169002
5	111993553	112093553	0.474393	SMDT1
5	111993553	112093553	0.474393	LOC102169285
5	111993553	112093553	0.474393	FAM109B
5	111993553	112093553	0.474393	NDUFA6
5	111993553	112093553	0.474393	TCF20
5	111993553	112093553	0.474393	LOC102177333
5	111993553	112093553	0.474393	LOC102169002
5	111993606	112093606	0.474609	SMDT1
5	111993606	112093606	0.474609	LOC102169285
5	111993606	112093606	0.474609	FAM109B
5	111993606	112093606	0.474609	NDUFA6
5	111993606	112093606	0.474609	TCF20
5	111993606	112093606	0.474609	LOC102177333
5	111993606	112093606	0.474609	LOC102169002
5	111993616	112093616	0.505097	SMDT1
5	111993616	112093616	0.505097	LOC102169285
5	111993616	112093616	0.505097	FAM109B
5	111993616	112093616	0.505097	NDUFA6
5	111993616	112093616	0.505097	TCF20
5	111993616	112093616	0.505097	LOC102177333
5	111993616	112093616	0.505097	LOC102169002
5	111994096	112094096	0.521617	SMDT1
5	111994096	112094096	0.521617	LOC102169285
5	111994096	112094096	0.521617	FAM109B
5	111994096	112094096	0.521617	NDUFA6
5	111994096	112094096	0.521617	TCF20
5	111994096	112094096	0.521617	LOC102177333
5	111994096	112094096	0.521617	LOC102169002
5	111994100	112094100	0.53313	SMDT1
5	111994100	112094100	0.53313	LOC102169285
5	111994100	112094100	0.53313	FAM109B
5	111994100	112094100	0.53313	NDUFA6
5	111994100	112094100	0.53313	TCF20
5	111994100	112094100	0.53313	LOC102177333
5	111994100	112094100	0.53313	LOC102169002
5	111994228	112094228	0.514671	SMDT1
5	111994228	112094228	0.514671	LOC102169285
5	111994228	112094228	0.514671	FAM109B

5	111994228	112094228	0.514671	NDUFA6
5	111994228	112094228	0.514671	TCF20
5	111994228	112094228	0.514671	LOC102177333
5	111994228	112094228	0.514671	LOC102169002
5	111994532	112094532	0.506682	SMDT1
5	111994532	112094532	0.506682	LOC102169285
5	111994532	112094532	0.506682	FAM109B
5	111994532	112094532	0.506682	NDUFA6
5	111994532	112094532	0.506682	TCF20
5	111994532	112094532	0.506682	LOC102177333
5	111994532	112094532	0.506682	LOC102169002
5	111994552	112094552	0.520121	SMDT1
5	111994552	112094552	0.520121	LOC102169285
5	111994552	112094552	0.520121	FAM109B
5	111994552	112094552	0.520121	NDUFA6
5	111994552	112094552	0.520121	TCF20
5	111994552	112094552	0.520121	LOC102177333
5	111994552	112094552	0.520121	LOC102169002
5	111994627	112094627	0.520121	SMDT1
5	111994627	112094627	0.520121	LOC102169285
5	111994627	112094627	0.520121	FAM109B
5	111994627	112094627	0.520121	NDUFA6
5	111994627	112094627	0.520121	TCF20
5	111994627	112094627	0.520121	LOC102177333
5	111994627	112094627	0.520121	LOC102169002
5	111994886	112094886	0.520121	SMDT1
5	111994886	112094886	0.520121	LOC102169285
5	111994886	112094886	0.520121	FAM109B
5	111994886	112094886	0.520121	NDUFA6
5	111994886	112094886	0.520121	TCF20
5	111994886	112094886	0.520121	LOC102177333
5	111994886	112094886	0.520121	LOC102169002
5	111994898	112094898	0.514834	SMDT1
5	111994898	112094898	0.514834	LOC102169285
5	111994898	112094898	0.514834	FAM109B
5	111994898	112094898	0.514834	NDUFA6
5	111994898	112094898	0.514834	TCF20
5	111994898	112094898	0.514834	LOC102177333
5	111994898	112094898	0.514834	LOC102169002
5	111994935	112094935	0.50405	SMDT1
5	111994935	112094935	0.50405	LOC102169285
5	111994935	112094935	0.50405	FAM109B
5	111994935	112094935	0.50405	NDUFA6
5	111994935	112094935	0.50405	TCF20
5	111994935	112094935	0.50405	LOC102177333
5	111994935	112094935	0.50405	LOC102169002
5	111995477	112095477	0.50768	SMDT1
5	111995477	112095477	0.50768	LOC102169285
5	111995477	112095477	0.50768	FAM109B
5	111995477	112095477	0.50768	NDUFA6
5	111995477	112095477	0.50768	TCF20
5	111995477	112095477	0.50768	LOC102177333
5	111995477	112095477	0.50768	LOC102169002
5	111995536	112095536	0.495254	SMDT1
5	111995536	112095536	0.495254	LOC102169285
5	111995536	112095536	0.495254	FAM109B
5	111995536	112095536	0.495254	NDUFA6
5	111995536	112095536	0.495254	TCF20

5	111995536	112095536	0.495254	LOC102177333
5	111995536	112095536	0.495254	LOC102169002
5	111995593	112095593	0.503796	SMDT1
5	111995593	112095593	0.503796	LOC102169285
5	111995593	112095593	0.503796	FAM109B
5	111995593	112095593	0.503796	NDUFA6
5	111995593	112095593	0.503796	TCF20
5	111995593	112095593	0.503796	LOC102177333
5	111995593	112095593	0.503796	LOC102169002
5	111996004	112096004	0.411226	SMDT1
5	111996004	112096004	0.411226	LOC102169285
5	111996004	112096004	0.411226	FAM109B
5	111996004	112096004	0.411226	NDUFA6
5	111996004	112096004	0.411226	TCF20
5	111996004	112096004	0.411226	LOC102177333
5	111996004	112096004	0.411226	LOC102169002
5	111996008	112096008	0.40763	SMDT1
5	111996008	112096008	0.40763	LOC102169285
5	111996008	112096008	0.40763	FAM109B
5	111996008	112096008	0.40763	NDUFA6
5	111996008	112096008	0.40763	TCF20
5	111996008	112096008	0.40763	LOC102177333
5	111996008	112096008	0.40763	LOC102169002
5	111996015	112096015	0.411625	SMDT1
5	111996015	112096015	0.411625	LOC102169285
5	111996015	112096015	0.411625	FAM109B
5	111996015	112096015	0.411625	NDUFA6
5	111996015	112096015	0.411625	TCF20
5	111996015	112096015	0.411625	LOC102177333
5	111996015	112096015	0.411625	LOC102169002
5	111996016	112096016	0.405149	SMDT1
5	111996016	112096016	0.405149	LOC102169285
5	111996016	112096016	0.405149	FAM109B
5	111996016	112096016	0.405149	NDUFA6
5	111996016	112096016	0.405149	TCF20
5	111996016	112096016	0.405149	LOC102177333
5	111996016	112096016	0.405149	LOC102169002
5	111996054	112096054	0.378021	SMDT1
5	111996054	112096054	0.378021	LOC102169285
5	111996054	112096054	0.378021	FAM109B
5	111996054	112096054	0.378021	NDUFA6
5	111996054	112096054	0.378021	TCF20
5	111996054	112096054	0.378021	LOC102177333
5	111996054	112096054	0.378021	LOC102169002
5	111996513	112096513	0.364523	SMDT1
5	111996513	112096513	0.364523	LOC102169285
5	111996513	112096513	0.364523	FAM109B
5	111996513	112096513	0.364523	NDUFA6
5	111996513	112096513	0.364523	TCF20
5	111996513	112096513	0.364523	LOC102177333
5	111996513	112096513	0.364523	LOC102169002
5	111996537	112096537	0.38148	SMDT1
5	111996537	112096537	0.38148	LOC102169285
5	111996537	112096537	0.38148	FAM109B
5	111996537	112096537	0.38148	NDUFA6
5	111996537	112096537	0.38148	TCF20
5	111996537	112096537	0.38148	LOC102177333
5	111996537	112096537	0.38148	LOC102169002

5	111996731	112096731	0.382371	SMDT1
5	111996731	112096731	0.382371	LOC102169285
5	111996731	112096731	0.382371	FAM109B
5	111996731	112096731	0.382371	NDUFA6
5	111996731	112096731	0.382371	TCF20
5	111996731	112096731	0.382371	LOC102177333
5	111996731	112096731	0.382371	LOC102169002
5	111996734	112096734	0.382371	SMDT1
5	111996734	112096734	0.382371	LOC102169285
5	111996734	112096734	0.382371	FAM109B
5	111996734	112096734	0.382371	NDUFA6
5	111996734	112096734	0.382371	TCF20
5	111996734	112096734	0.382371	LOC102177333
5	111996734	112096734	0.382371	LOC102169002
5	111996799	112096799	0.371031	SMDT1
5	111996799	112096799	0.371031	LOC102169285
5	111996799	112096799	0.371031	FAM109B
5	111996799	112096799	0.371031	NDUFA6
5	111996799	112096799	0.371031	TCF20
5	111996799	112096799	0.371031	LOC102177333
5	111996799	112096799	0.371031	LOC102169002
5	111997327	112097327	0.366525	SMDT1
5	111997327	112097327	0.366525	LOC102169285
5	111997327	112097327	0.366525	FAM109B
5	111997327	112097327	0.366525	NDUFA6
5	111997327	112097327	0.366525	TCF20
5	111997327	112097327	0.366525	LOC102177333
5	111997327	112097327	0.366525	LOC102169002
5	111997783	112097783	0.375184	SMDT1
5	111997783	112097783	0.375184	LOC102169285
5	111997783	112097783	0.375184	FAM109B
5	111997783	112097783	0.375184	NDUFA6
5	111997783	112097783	0.375184	TCF20
5	111997783	112097783	0.375184	LOC102177333
5	111997783	112097783	0.375184	LOC102169002
5	111999895	112099895	0.355621	SMDT1
5	111999895	112099895	0.355621	LOC102169285
5	111999895	112099895	0.355621	FAM109B
5	111999895	112099895	0.355621	NDUFA6
5	111999895	112099895	0.355621	TCF20
5	111999895	112099895	0.355621	LOC102177333
5	111999895	112099895	0.355621	LOC102169002
5	112000017	112100017	0.349629	SMDT1
5	112000017	112100017	0.349629	LOC102169285
5	112000017	112100017	0.349629	FAM109B
5	112000017	112100017	0.349629	NDUFA6
5	112000017	112100017	0.349629	TCF20
5	112000017	112100017	0.349629	LOC102177333
5	112000017	112100017	0.349629	LOC102169002
5	112000091	112100091	0.346669	SMDT1
5	112000091	112100091	0.346669	LOC102169285
5	112000091	112100091	0.346669	FAM109B
5	112000091	112100091	0.346669	NDUFA6
5	112000091	112100091	0.346669	TCF20
5	112000091	112100091	0.346669	LOC102177333
5	112000091	112100091	0.346669	LOC102169002
5	112001985	112101985	0.362588	SMDT1
5	112001985	112101985	0.362588	LOC102169285

5	112001985	112101985	0.362588	FAM109B
5	112001985	112101985	0.362588	NDUFA6
5	112001985	112101985	0.362588	TCF20
5	112001985	112101985	0.362588	LOC102177333
5	112001985	112101985	0.362588	LOC102169002
5	112002091	112102091	0.343963	SMDT1
5	112002091	112102091	0.343963	LOC102169285
5	112002091	112102091	0.343963	FAM109B
5	112002091	112102091	0.343963	NDUFA6
5	112002091	112102091	0.343963	TCF20
5	112002091	112102091	0.343963	LOC102177333
5	112002091	112102091	0.343963	LOC102169002
5	112002321	112102321	0.35423	SMDT1
5	112002321	112102321	0.35423	LOC102169285
5	112002321	112102321	0.35423	FAM109B
5	112002321	112102321	0.35423	NDUFA6
5	112002321	112102321	0.35423	TCF20
5	112002321	112102321	0.35423	LOC102177333
5	112002321	112102321	0.35423	LOC102169002
5	112003579	112103579	0.351585	SMDT1
5	112003579	112103579	0.351585	LOC102169285
5	112003579	112103579	0.351585	FAM109B
5	112003579	112103579	0.351585	NDUFA6
5	112003579	112103579	0.351585	TCF20
5	112003579	112103579	0.351585	LOC102177333
5	112003579	112103579	0.351585	LOC102169002
5	112003746	112103746	0.395306	SMDT1
5	112003746	112103746	0.395306	LOC102169285
5	112003746	112103746	0.395306	FAM109B
5	112003746	112103746	0.395306	NDUFA6
5	112003746	112103746	0.395306	TCF20
5	112003746	112103746	0.395306	LOC102177333
5	112003746	112103746	0.395306	LOC102169002
5	112004784	112104784	0.378534	SMDT1
5	112004784	112104784	0.378534	LOC102169285
5	112004784	112104784	0.378534	FAM109B
5	112004784	112104784	0.378534	NDUFA6
5	112004784	112104784	0.378534	TCF20
5	112004784	112104784	0.378534	LOC102177333
5	112004784	112104784	0.378534	LOC102169002
5	112004897	112104897	0.423626	SMDT1
5	112004897	112104897	0.423626	LOC102169285
5	112004897	112104897	0.423626	FAM109B
5	112004897	112104897	0.423626	NDUFA6
5	112004897	112104897	0.423626	TCF20
5	112004897	112104897	0.423626	LOC102177333
5	112004897	112104897	0.423626	LOC102169002
5	112009248	112109248	0.359157	LOC102169285
5	112009248	112109248	0.359157	NDUFA6
5	112009248	112109248	0.359157	TCF20
5	112009248	112109248	0.359157	LOC102177333
5	112009248	112109248	0.359157	LOC102169002
5	112009257	112109257	0.354801	LOC102169285
5	112009257	112109257	0.354801	NDUFA6
5	112009257	112109257	0.354801	TCF20
5	112009257	112109257	0.354801	LOC102177333
5	112009257	112109257	0.354801	LOC102169002
5	112009785	112109785	0.346619	LOC102169285

5	112009785	112109785	0.346619	NDUFA6
5	112009785	112109785	0.346619	TCF20
5	112009785	112109785	0.346619	LOC102177333
5	112009785	112109785	0.346619	LOC102169002
5	112660269	112760269	0.359378	PACSIN2
5	112660535	112760535	0.360828	PACSIN2
5	112660762	112760762	0.359785	PACSIN2
5	112660851	112760851	0.367049	PACSIN2
5	112660878	112760878	0.365086	PACSIN2
5	112660894	112760894	0.35726	PACSIN2
5	112660951	112760951	0.369038	PACSIN2
5	112661131	112761131	0.358181	PACSIN2
5	112661148	112761148	0.346677	PACSIN2
5	112661175	112761175	0.342136	PACSIN2
5	112661706	112761706	0.357129	PACSIN2
5	112662460	112762460	0.357674	PACSIN2
5	112662473	112762473	0.365391	PACSIN2
5	112662653	112762653	0.409924	PACSIN2
5	112662701	112762701	0.411835	PACSIN2
5	112662712	112762712	0.404897	PACSIN2
5	112662796	112762796	0.44378	PACSIN2
5	112662918	112762918	0.44378	PACSIN2
5	112663087	112763087	0.442011	PACSIN2
5	112663424	112763424	0.427559	PACSIN2
5	112663736	112763736	0.453358	PACSIN2
5	112663991	112763991	0.367454	PACSIN2
5	112664044	112764044	0.36937	PACSIN2
5	112664265	112764265	0.369455	PACSIN2
5	112664269	112764269	0.357699	PACSIN2
5	112667354	112767354	0.345077	PACSIN2
5	112750972	112850972	0.353586	TTLL1
5	112750972	112850972	0.353586	PACSIN2
5	112750985	112850985	0.353586	TTLL1
5	112750985	112850985	0.353586	PACSIN2
5	112751010	112851010	0.353754	TTLL1
5	112751010	112851010	0.353754	PACSIN2
5	112751031	112851031	0.353754	TTLL1
5	112751031	112851031	0.353754	PACSIN2
5	112751034	112851034	0.368122	TTLL1
5	112751034	112851034	0.368122	PACSIN2
5	112751039	112851039	0.365275	TTLL1
5	112751039	112851039	0.365275	PACSIN2
5	112751091	112851091	0.363432	TTLL1
5	112751091	112851091	0.363432	PACSIN2
5	112751097	112851097	0.347366	TTLL1
5	112751097	112851097	0.347366	PACSIN2
5	112751124	112851124	0.34857	TTLL1
5	112751124	112851124	0.34857	PACSIN2
5	112751135	112851135	0.34857	TTLL1
5	112751135	112851135	0.34857	PACSIN2
5	112751144	112851144	0.34857	TTLL1
5	112751144	112851144	0.34857	PACSIN2
5	112751216	112851216	0.344272	TTLL1
5	112751216	112851216	0.344272	PACSIN2
5	112751248	112851248	0.345033	TTLL1
5	112751248	112851248	0.345033	PACSIN2
5	112751412	112851412	0.354889	TTLL1
5	112751412	112851412	0.354889	PACSIN2

5	112755661	112855661	0.367716	TTLL1
5	112755661	112855661	0.367716	PACSIN2
5	113726066	113826066	0.348133	PARVG
5	113726066	113826066	0.348133	PARVB
5	113726075	113826075	0.344087	PARVG
5	113726075	113826075	0.344087	PARVB
5	113730170	113830170	0.346181	PARVG
5	113730170	113830170	0.346181	PARVB
5	113730174	113830174	0.344946	PARVG
5	113730174	113830174	0.344946	PARVB
5	113730211	113830211	0.346727	PARVG
5	113730211	113830211	0.346727	PARVB
5	113730245	113830245	0.343669	PARVG
5	113730245	113830245	0.343669	PARVB
5	113730253	113830253	0.343669	PARVG
5	113730253	113830253	0.343669	PARVB
5	113730254	113830254	0.365924	PARVG
5	113730254	113830254	0.365924	PARVB
5	113730306	113830306	0.373045	PARVG
5	113730306	113830306	0.373045	PARVB
5	113730316	113830316	0.372494	PARVG
5	113730316	113830316	0.372494	PARVB
5	113730323	113830323	0.360157	PARVG
5	113730323	113830323	0.360157	PARVB
5	113730759	113830759	0.359485	PARVG
5	113730759	113830759	0.359485	PARVB
5	113730821	113830821	0.36471	PARVG
5	113730821	113830821	0.36471	PARVB
5	113730869	113830869	0.367918	PARVG
5	113730869	113830869	0.367918	PARVB
5	113730901	113830901	0.367399	PARVG
5	113730901	113830901	0.367399	PARVB
5	113730990	113830990	0.367252	PARVG
5	113730990	113830990	0.367252	PARVB
5	113731018	113831018	0.368896	PARVG
5	113731018	113831018	0.368896	PARVB
5	113731020	113831020	0.368896	PARVG
5	113731020	113831020	0.368896	PARVB
5	113731063	113831063	0.371613	PARVG
5	113731063	113831063	0.371613	PARVB
5	113731147	113831147	0.364436	PARVG
5	113731147	113831147	0.364436	PARVB
5	113731180	113831180	0.362081	PARVG
5	113731180	113831180	0.362081	PARVB
5	113731202	113831202	0.360167	PARVG
5	113731202	113831202	0.360167	PARVB
5	113731211	113831211	0.359743	PARVG
5	113731211	113831211	0.359743	PARVB
5	113731269	113831269	0.359397	PARVG
5	113731269	113831269	0.359397	PARVB
5	113731270	113831270	0.344776	PARVG
5	113731270	113831270	0.344776	PARVB
5	113731280	113831280	0.34474	PARVG
5	113731280	113831280	0.34474	PARVB
5	113731329	113831329	0.344045	PARVG
5	113731329	113831329	0.344045	PARVB
5	113731340	113831340	0.342775	PARVG
5	113731340	113831340	0.342775	PARVB

5	115158007	115258007	0.348362	WNT7B
5	115478822	115578822	0.394813	LOC108636121
5	115478822	115578822	0.394813	TTC38
5	115478822	115578822	0.394813	GTSE1
5	115478822	115578822	0.394813	TRMU
5	115478822	115578822	0.394813	LOC106501921
5	115478923	115578923	0.405366	LOC108636121
5	115478923	115578923	0.405366	TTC38
5	115478923	115578923	0.405366	GTSE1
5	115478923	115578923	0.405366	TRMU
5	115478923	115578923	0.405366	LOC106501921
5	115478971	115578971	0.389876	LOC108636121
5	115478971	115578971	0.389876	TTC38
5	115478971	115578971	0.389876	GTSE1
5	115478971	115578971	0.389876	TRMU
5	115478971	115578971	0.389876	LOC106501921
5	115479149	115579149	0.378298	LOC108636121
5	115479149	115579149	0.378298	TTC38
5	115479149	115579149	0.378298	GTSE1
5	115479149	115579149	0.378298	TRMU
5	115479149	115579149	0.378298	LOC106501921
5	115479320	115579320	0.383728	LOC108636121
5	115479320	115579320	0.383728	TTC38
5	115479320	115579320	0.383728	GTSE1
5	115479320	115579320	0.383728	TRMU
5	115479320	115579320	0.383728	LOC106501921
5	115479388	115579388	0.381295	LOC108636121
5	115479388	115579388	0.381295	TTC38
5	115479388	115579388	0.381295	GTSE1
5	115479388	115579388	0.381295	TRMU
5	115479388	115579388	0.381295	LOC106501921
5	115479503	115579503	0.376948	LOC108636121
5	115479503	115579503	0.376948	TTC38
5	115479503	115579503	0.376948	GTSE1
5	115479503	115579503	0.376948	TRMU
5	115479686	115579686	0.427017	LOC108636121
5	115479686	115579686	0.427017	TTC38
5	115479686	115579686	0.427017	GTSE1
5	115479686	115579686	0.427017	TRMU
5	115479817	115579817	0.429658	LOC108636121
5	115479817	115579817	0.429658	TTC38
5	115479817	115579817	0.429658	GTSE1
5	115479817	115579817	0.429658	TRMU
5	115480067	115580067	0.409747	LOC108636121
5	115480067	115580067	0.409747	TTC38
5	115480067	115580067	0.409747	GTSE1
5	115480067	115580067	0.409747	TRMU
5	115480191	115580191	0.380615	LOC108636121
5	115480191	115580191	0.380615	TTC38
5	115480191	115580191	0.380615	GTSE1
5	115480191	115580191	0.380615	TRMU
5	115480214	115580214	0.380757	LOC108636121
5	115480214	115580214	0.380757	TTC38
5	115480214	115580214	0.380757	GTSE1
5	115480214	115580214	0.380757	TRMU
5	115480351	115580351	0.380682	LOC108636121
5	115480351	115580351	0.380682	TTC38
5	115480351	115580351	0.380682	GTSE1

5	115480351	115580351	0.380682	TRMU
5	115480453	115580453	0.380682	LOC108636121
5	115480453	115580453	0.380682	TTC38
5	115480453	115580453	0.380682	GTSE1
5	115480453	115580453	0.380682	TRMU
5	115481131	115581131	0.379008	LOC108636121
5	115481131	115581131	0.379008	TTC38
5	115481131	115581131	0.379008	GTSE1
5	115481131	115581131	0.379008	TRMU
5	115481168	115581168	0.405681	LOC108636121
5	115481168	115581168	0.405681	TTC38
5	115481168	115581168	0.405681	GTSE1
5	115481168	115581168	0.405681	TRMU
5	115481214	115581214	0.410004	LOC108636121
5	115481214	115581214	0.410004	TTC38
5	115481214	115581214	0.410004	GTSE1
5	115481214	115581214	0.410004	TRMU
5	115612888	115712888	0.348232	CELSR1
5	115612922	115712922	0.351096	CELSR1
5	115612932	115712932	0.351096	CELSR1
5	115612936	115712936	0.35131	CELSR1
5	115612964	115712964	0.36105	CELSR1
5	115612978	115712978	0.361142	CELSR1
5	115613000	115713000	0.361142	CELSR1
5	115613002	115713002	0.401441	CELSR1
5	115613014	115713014	0.387648	CELSR1
5	115613023	115713023	0.386951	CELSR1
5	115613035	115713035	0.386372	CELSR1
5	115613048	115713048	0.368994	CELSR1
5	115613103	115713103	0.360747	CELSR1
5	115613162	115713162	0.341933	CELSR1
5	115613629	115713629	0.345571	CELSR1
5	115613751	115713751	0.381023	CELSR1
5	115614020	115714020	0.348722	CELSR1
5	115614090	115714090	0.371211	CELSR1
5	115614109	115714109	0.373314	CELSR1
5	115614130	115714130	0.348292	CELSR1
5	115614155	115714155	0.349665	CELSR1
5	115614322	115714322	0.350567	CELSR1
5	115654044	115754044	0.34752	CELSR1
5	115659820	115759820	0.344051	CELSR1
5	115660032	115760032	0.358268	CELSR1
5	115660110	115760110	0.391902	LOC106502160
5	115660110	115760110	0.391902	CELSR1
5	116392874	116492874	0.347379	TRNAC-ACA-31
6	2410897	2510897	0.357009	LOC108636198
6	2410897	2510897	0.357009	TKTL2
6	2410905	2510905	0.357388	LOC108636198
6	2410905	2510905	0.357388	TKTL2
6	2410929	2510929	0.357749	LOC108636198
6	2410929	2510929	0.357749	TKTL2
6	2410930	2510930	0.353512	LOC108636198
6	2410930	2510930	0.353512	TKTL2
6	2413325	2513325	0.343884	LOC108636198
6	2413325	2513325	0.343884	TKTL2
6	2413335	2513335	0.347911	LOC108636198
6	2413335	2513335	0.347911	TKTL2
6	2413420	2513420	0.344344	LOC108636198

6	2413420	2513420	0.344344	TKTL2
6	2413461	2513461	0.341676	LOC108636198
6	2413461	2513461	0.341676	TKTL2
6	2413555	2513555	0.347182	LOC108636198
6	2413555	2513555	0.347182	TKTL2
6	3784718	3884718	0.34359	LOC106502168
6	3784718	3884718	0.34359	LOC102190672
6	3939999	4039999	0.377585	ANXA5
6	3940054	4040054	0.423012	ANXA5
6	3940059	4040059	0.409011	ANXA5
6	3940086	4040086	0.42124	ANXA5
6	3940106	4040106	0.383735	ANXA5
6	4074776	4174776	0.342027	LOC108636255
6	4075496	4175496	0.345115	LOC108636255
6	4075573	4175573	0.375382	LOC108636255
6	4075574	4175574	0.372633	LOC108636255
6	4075580	4175580	0.349067	LOC108636255
6	4076007	4176007	0.345786	LOC108636255
6	4076035	4176035	0.346621	LOC108636255
6	4076126	4176126	0.347214	LOC108636255
6	4076181	4176181	0.367618	LOC108636255
6	4076220	4176220	0.357186	LOC108636255
6	4076291	4176291	0.364247	LOC108636255
6	4076299	4176299	0.370287	LOC108636255
6	4076331	4176331	0.365582	LOC108636255
6	4076332	4176332	0.365582	LOC108636255
6	4076352	4176352	0.370964	LOC108636255
6	4076394	4176394	0.371876	LOC108636255
6	4076432	4176432	0.363804	LOC108636255
6	4076450	4176450	0.3739	LOC108636255
6	4076468	4176468	0.363143	LOC108636255
6	4076519	4176519	0.361641	LOC108636255
6	4076539	4176539	0.35401	LOC108636255
6	4076617	4176617	0.365292	LOC108636255
6	4076729	4176729	0.369458	LOC108636255
6	4076840	4176840	0.358539	LOC108636255
6	4076907	4176907	0.354949	LOC108636255
6	4077055	4177055	0.351301	LOC108636255
6	4077073	4177073	0.350527	LOC108636255
6	4077317	4177317	0.347834	LOC108636255
6	4077327	4177327	0.348422	LOC108636255
6	4077385	4177385	0.370362	LOC108636255
6	4077443	4177443	0.368282	LOC108636255
6	4077462	4177462	0.361643	LOC108636255
6	4077463	4177463	0.361643	LOC108636255
6	4077526	4177526	0.362254	LOC108636255
6	4077542	4177542	0.360297	LOC108636255
6	4077621	4177621	0.350073	LOC108636255
6	4077711	4177711	0.355696	LOC108636255
6	4077712	4177712	0.359227	LOC108636255
6	4077756	4177756	0.359227	LOC108636255
6	4077851	4177851	0.35053	LOC108636255
6	4077957	4177957	0.349975	LOC108636255
6	4077997	4177997	0.369005	LOC108636255
6	4078035	4178035	0.376319	LOC108636255
6	4078081	4178081	0.35401	LOC108636255
6	4078130	4178130	0.353076	LOC108636255
6	4282702	4382702	0.346778	LOC108636256

6	4282702	4382702	0.346778	QRFPR
6	4282775	4382775	0.342732	LOC108636256
6	4282775	4382775	0.342732	QRFPR
6	4285168	4385168	0.380528	LOC108636256
6	4285168	4385168	0.380528	QRFPR
6	4285218	4385218	0.380202	LOC108636256
6	4285218	4385218	0.380202	QRFPR
6	4285485	4385485	0.374332	LOC108636256
6	4285485	4385485	0.374332	QRFPR
6	4285746	4385746	0.364536	LOC108636256
6	4285746	4385746	0.364536	QRFPR
6	4286266	4386266	0.364536	LOC108636256
6	4286266	4386266	0.364536	QRFPR
6	4286369	4386369	0.372874	LOC108636256
6	4286369	4386369	0.372874	QRFPR
6	4286454	4386454	0.385874	LOC108636256
6	4286454	4386454	0.385874	QRFPR
6	4286569	4386569	0.383456	LOC108636256
6	4286569	4386569	0.383456	QRFPR
6	4286589	4386589	0.389694	LOC108636256
6	4286589	4386589	0.389694	QRFPR
6	4286600	4386600	0.397328	LOC108636256
6	4286600	4386600	0.397328	QRFPR
6	4286672	4386672	0.407529	LOC108636256
6	4286672	4386672	0.407529	QRFPR
6	4286685	4386685	0.443112	LOC108636256
6	4286685	4386685	0.443112	QRFPR
6	4286692	4386692	0.430053	LOC108636256
6	4286692	4386692	0.430053	QRFPR
6	4286812	4386812	0.408362	LOC108636256
6	4286812	4386812	0.408362	QRFPR
6	4286861	4386861	0.365363	LOC108636256
6	4286861	4386861	0.365363	QRFPR
6	4287322	4387322	0.362345	LOC108636256
6	4287322	4387322	0.362345	QRFPR
6	4287686	4387686	0.362345	LOC108636256
6	4287686	4387686	0.362345	QRFPR
6	4287759	4387759	0.370389	LOC108636256
6	4287759	4387759	0.370389	QRFPR
6	4287835	4387835	0.366587	LOC108636256
6	4287835	4387835	0.366587	QRFPR
6	4287897	4387897	0.366587	LOC108636256
6	4287897	4387897	0.366587	QRFPR
6	4288162	4388162	0.366587	LOC108636256
6	4288162	4388162	0.366587	QRFPR
6	4288182	4388182	0.378967	LOC108636256
6	4288182	4388182	0.378967	QRFPR
6	4288438	4388438	0.369424	LOC108636256
6	4288438	4388438	0.369424	QRFPR
6	4288615	4388615	0.369552	LOC108636256
6	4288615	4388615	0.369552	QRFPR
6	4288668	4388668	0.381495	LOC108636256
6	4288668	4388668	0.381495	QRFPR
6	4288675	4388675	0.369388	LOC108636256
6	4288675	4388675	0.369388	QRFPR
6	4288742	4388742	0.35789	LOC108636256
6	4288742	4388742	0.35789	QRFPR
6	4289477	4389477	0.361184	LOC108636256

6	4289477	4389477	0.361184	QRFPR
6	4289494	4389494	0.36157	LOC108636256
6	4289494	4389494	0.36157	QRFPR
6	4289741	4389741	0.365936	LOC108636256
6	4289741	4389741	0.365936	QRFPR
6	4289861	4389861	0.365518	LOC108636256
6	4289861	4389861	0.365518	QRFPR
6	4289875	4389875	0.365518	LOC108636256
6	4289875	4389875	0.365518	QRFPR
6	4289972	4389972	0.365518	LOC108636256
6	4289972	4389972	0.365518	QRFPR
6	4290178	4390178	0.36002	LOC108636256
6	4290178	4390178	0.36002	QRFPR
6	4290708	4390708	0.354965	LOC108636256
6	4290708	4390708	0.354965	QRFPR
6	4291247	4391247	0.359566	LOC108636256
6	4291247	4391247	0.359566	QRFPR
6	4291306	4391306	0.38159	LOC108636256
6	4291306	4391306	0.38159	QRFPR
6	4291310	4391310	0.384419	LOC108636256
6	4291310	4391310	0.384419	QRFPR
6	4291316	4391316	0.384419	LOC108636256
6	4291316	4391316	0.384419	QRFPR
6	4291373	4391373	0.367859	LOC108636256
6	4291373	4391373	0.367859	QRFPR
6	4291641	4391641	0.367859	LOC108636256
6	4291641	4391641	0.367859	QRFPR
6	4291659	4391659	0.367859	LOC108636256
6	4291659	4391659	0.367859	QRFPR
6	4291672	4391672	0.367859	LOC108636256
6	4291672	4391672	0.367859	QRFPR
6	4292075	4392075	0.367859	LOC108636256
6	4292075	4392075	0.367859	QRFPR
6	4292203	4392203	0.367859	LOC108636256
6	4292203	4392203	0.367859	QRFPR
6	4292204	4392204	0.367859	LOC108636256
6	4292204	4392204	0.367859	QRFPR
6	4292280	4392280	0.383318	LOC108636256
6	4292280	4392280	0.383318	QRFPR
6	4292290	4392290	0.383164	LOC108636256
6	4292290	4392290	0.383164	QRFPR
6	4292322	4392322	0.379075	LOC108636256
6	4292322	4392322	0.379075	QRFPR
6	4292458	4392458	0.379075	LOC108636256
6	4292458	4392458	0.379075	QRFPR
6	4292708	4392708	0.379848	LOC108636256
6	4292708	4392708	0.379848	QRFPR
6	4292714	4392714	0.404965	LOC108636256
6	4292714	4392714	0.404965	QRFPR
6	4292728	4392728	0.398737	LOC108636256
6	4292728	4392728	0.398737	QRFPR
6	4292760	4392760	0.392971	LOC108636256
6	4292760	4392760	0.392971	QRFPR
6	4292903	4392903	0.375462	LOC108636256
6	4292903	4392903	0.375462	QRFPR
6	4292921	4392921	0.347811	LOC108636256
6	4292921	4392921	0.347811	QRFPR
6	4293180	4393180	0.382745	LOC108636256

6	4293180	4393180	0.382745	QRFPR
6	4293211	4393211	0.368474	LOC108636256
6	4293211	4393211	0.368474	QRFPR
6	4293275	4393275	0.349251	LOC108636256
6	4293275	4393275	0.349251	QRFPR
6	4293354	4393354	0.344426	LOC108636256
6	4293354	4393354	0.344426	QRFPR
6	4293371	4393371	0.345135	LOC108636256
6	4293371	4393371	0.345135	QRFPR
6	4293408	4393408	0.352438	LOC108636256
6	4293408	4393408	0.352438	QRFPR
6	4293621	4393621	0.373871	LOC108636256
6	4293621	4393621	0.373871	QRFPR
6	4293637	4393637	0.373733	LOC108636256
6	4293637	4393637	0.373733	QRFPR
6	4293638	4393638	0.373843	LOC108636256
6	4293638	4393638	0.373843	QRFPR
6	4293726	4393726	0.375359	LOC108636256
6	4293726	4393726	0.375359	QRFPR
6	4293842	4393842	0.353243	LOC108636256
6	4293842	4393842	0.353243	QRFPR
6	4293929	4393929	0.361908	LOC108636256
6	4293929	4393929	0.361908	QRFPR
6	4294989	4394989	0.350735	LOC108636256
6	4294989	4394989	0.350735	QRFPR
6	4295255	4395255	0.349746	LOC108636256
6	4295255	4395255	0.349746	QRFPR
6	4295324	4395324	0.346631	LOC108636256
6	4295324	4395324	0.346631	QRFPR
6	4295353	4395353	0.349482	LOC108636256
6	4295353	4395353	0.349482	QRFPR
6	4295400	4395400	0.347657	LOC108636256
6	4295400	4395400	0.347657	QRFPR
6	4295411	4395411	0.349857	LOC108636256
6	4295411	4395411	0.349857	QRFPR
6	4295451	4395451	0.347623	LOC108636256
6	4295451	4395451	0.347623	QRFPR
6	4295457	4395457	0.344286	LOC108636256
6	4295457	4395457	0.344286	QRFPR
6	4295556	4395556	0.358827	LOC108636256
6	4295556	4395556	0.358827	QRFPR
6	4295774	4395774	0.357122	LOC108636256
6	4295774	4395774	0.357122	QRFPR
6	4298701	4398701	0.353313	LOC108636256
6	4298701	4398701	0.353313	QRFPR
6	4561708	4661708	0.375766	TNIP3
6	4561819	4661819	0.377658	TNIP3
6	4561829	4661829	0.380439	TNIP3
6	4562147	4662147	0.378066	TNIP3
6	4562188	4662188	0.378292	TNIP3
6	4562292	4662292	0.378292	TNIP3
6	4562311	4662311	0.378292	TNIP3
6	4562332	4662332	0.378292	TNIP3
6	4562338	4662338	0.378592	TNIP3
6	4562346	4662346	0.379625	TNIP3
6	4562349	4662349	0.379646	TNIP3
6	4562387	4662387	0.378508	TNIP3
6	4562522	4662522	0.379315	TNIP3

6	4562564	4662564	0.383731	TNIP3
6	4562675	4662675	0.376028	TNIP3
6	4562818	4662818	0.380135	TNIP3
6	4562888	4662888	0.370671	TNIP3
6	4562913	4662913	0.370671	TNIP3
6	4562922	4662922	0.372478	TNIP3
6	4562939	4662939	0.372366	TNIP3
6	4562990	4662990	0.370416	TNIP3
6	4563439	4663439	0.371407	TNIP3
6	4563450	4663450	0.371478	TNIP3
6	4563470	4663470	0.387853	TNIP3
6	4563501	4663501	0.385346	TNIP3
6	4567663	4667663	0.348399	TNIP3
6	4567755	4667755	0.348269	TNIP3
6	4567862	4667862	0.34823	TNIP3
6	4567887	4667887	0.347462	TNIP3
6	4568002	4668002	0.376888	TNIP3
6	4568010	4668010	0.431557	TNIP3
6	4568017	4668017	0.421007	TNIP3
6	4568018	4668018	0.421007	TNIP3
6	4568056	4668056	0.425873	TNIP3
6	4568133	4668133	0.425873	TNIP3
6	4568156	4668156	0.425873	TNIP3
6	4568193	4668193	0.425873	TNIP3
6	4568203	4668203	0.428269	TNIP3
6	4568442	4668442	0.427564	TNIP3
6	4568492	4668492	0.426074	TNIP3
6	4568495	4668495	0.426074	TNIP3
6	4568562	4668562	0.427387	TNIP3
6	4568578	4668578	0.427387	TNIP3
6	4568679	4668679	0.427387	TNIP3
6	4568680	4668680	0.427387	TNIP3
6	4568726	4668726	0.431971	TNIP3
6	4568753	4668753	0.431062	TNIP3
6	4568758	4668758	0.431062	TNIP3
6	4568880	4668880	0.431062	TNIP3
6	4568918	4668918	0.443643	TNIP3
6	4568935	4668935	0.398785	TNIP3
6	4568941	4668941	0.395838	TNIP3
6	4568971	4668971	0.395838	TNIP3
6	4568976	4668976	0.395838	TNIP3
6	4569017	4669017	0.395927	TNIP3
6	4569025	4669025	0.395927	TNIP3
6	4569115	4669115	0.395311	TNIP3
6	4569191	4669191	0.398219	TNIP3
6	4569200	4669200	0.38874	TNIP3
6	4569911	4669911	0.387143	TNIP3
6	4570096	4670096	0.387143	TNIP3
6	4570320	4670320	0.387143	TNIP3
6	4570504	4670504	0.387143	TNIP3
6	4570525	4670525	0.387143	TNIP3
6	4570785	4670785	0.388153	TNIP3
6	4570887	4670887	0.390806	TNIP3
6	4571036	4671036	0.390605	TNIP3
6	4571069	4671069	0.390605	TNIP3
6	4571140	4671140	0.391807	TNIP3
6	4571141	4671141	0.382521	TNIP3
6	4571147	4671147	0.382521	TNIP3

6	4571354	4671354	0.382715	TNIP3
6	4571438	4671438	0.382715	TNIP3
6	4571578	4671578	0.382988	TNIP3
6	4572067	4672067	0.382799	TNIP3
6	4572412	4672412	0.392909	TNIP3
6	4572655	4672655	0.387905	TNIP3
6	4573320	4673320	0.400936	TNIP3
6	4573588	4673588	0.386773	TNIP3
6	4573658	4673658	0.386048	TNIP3
6	4573837	4673837	0.395648	TNIP3
6	4574005	4674005	0.395648	TNIP3
6	4575084	4675084	0.345841	TNIP3
6	4577101	4677101	0.343597	TNIP3
6	4577834	4677834	0.3428	TNIP3
6	4580574	4680574	0.350728	TNIP3
6	4580817	4680817	0.357306	TNIP3
6	4580837	4680837	0.362569	TNIP3
6	4580881	4680881	0.354233	TNIP3
6	4580903	4680903	0.352041	TNIP3
6	4580923	4680923	0.351409	TNIP3
6	4580948	4680948	0.351409	TNIP3
6	4580953	4680953	0.351409	TNIP3
6	4581116	4681116	0.349442	TNIP3
6	4581387	4681387	0.347008	TNIP3
6	4581693	4681693	0.35034	TNIP3
6	4581735	4681735	0.35034	TNIP3
6	4581736	4681736	0.351385	TNIP3
6	4581744	4681744	0.351385	TNIP3
6	4581764	4681764	0.351385	TNIP3
6	4581814	4681814	0.35118	TNIP3
6	4581889	4681889	0.35118	TNIP3
6	4581950	4681950	0.350447	TNIP3
6	4582172	4682172	0.350651	TNIP3
6	4582183	4682183	0.351713	TNIP3
6	4582273	4682273	0.349674	TNIP3
6	4582453	4682453	0.372082	TNIP3
6	4584238	4684238	0.342909	TNIP3
6	4584289	4684289	0.3421	TNIP3
6	4584388	4684388	0.342779	TNIP3
6	4598610	4698610	0.344822	TNIP3
6	4598610	4698610	0.344822	NDNF
6	4599083	4699083	0.361866	TNIP3
6	4599083	4699083	0.361866	NDNF
6	4599234	4699234	0.368606	TNIP3
6	4599234	4699234	0.368606	NDNF
6	4599250	4699250	0.368537	TNIP3
6	4599250	4699250	0.368537	NDNF
6	4599288	4699288	0.383214	TNIP3
6	4599288	4699288	0.383214	NDNF
6	4599375	4699375	0.382345	TNIP3
6	4599375	4699375	0.382345	NDNF
6	4599414	4699414	0.383841	TNIP3
6	4599414	4699414	0.383841	NDNF
6	4599421	4699421	0.383841	TNIP3
6	4599421	4699421	0.383841	NDNF
6	4599425	4699425	0.383841	TNIP3
6	4599425	4699425	0.383841	NDNF
6	4599433	4699433	0.384102	TNIP3

6	4599433	4699433	0.384102	NDNF
6	4599434	4699434	0.383968	TNIP3
6	4599434	4699434	0.383968	NDNF
6	4599444	4699444	0.383968	TNIP3
6	4599444	4699444	0.383968	NDNF
6	4599451	4699451	0.385532	TNIP3
6	4599451	4699451	0.385532	NDNF
6	4599453	4699453	0.385527	TNIP3
6	4599453	4699453	0.385527	NDNF
6	4599510	4699510	0.385532	TNIP3
6	4599510	4699510	0.385532	NDNF
6	4602892	4702892	0.342837	TNIP3
6	4602892	4702892	0.342837	NDNF
6	4604169	4704169	0.346689	TNIP3
6	4604169	4704169	0.346689	NDNF
6	4604216	4704216	0.345173	TNIP3
6	4604216	4704216	0.345173	NDNF
6	4604232	4704232	0.349623	TNIP3
6	4604232	4704232	0.349623	NDNF
6	4611904	4711904	0.36534	TNIP3
6	4611904	4711904	0.36534	NDNF
6	4614599	4714599	0.348825	TNIP3
6	4614599	4714599	0.348825	NDNF
6	4614907	4714907	0.357214	TNIP3
6	4614907	4714907	0.357214	NDNF
6	4614914	4714914	0.362245	TNIP3
6	4614914	4714914	0.362245	NDNF
6	4619412	4719412	0.377075	TNIP3
6	4619412	4719412	0.377075	NDNF
6	4684841	4784841	0.352695	NDNF
6	4684946	4784946	0.407751	NDNF
6	4684967	4784967	0.439399	NDNF
6	4685165	4785165	0.434207	NDNF
6	4685273	4785273	0.391356	NDNF
6	4685348	4785348	0.370191	NDNF
6	4685384	4785384	0.400278	NDNF
6	4692554	4792554	0.353215	NDNF
6	4693176	4793176	0.361917	NDNF
6	4693182	4793182	0.347039	NDNF
6	4693226	4793226	0.347443	NDNF
6	4693245	4793245	0.359254	NDNF
6	4693259	4793259	0.346072	NDNF
6	4693286	4793286	0.367662	NDNF
6	4693311	4793311	0.371266	NDNF
6	4693314	4793314	0.376251	NDNF
6	4693333	4793333	0.37724	NDNF
6	4693391	4793391	0.396746	NDNF
6	4693581	4793581	0.359956	NDNF
6	4693612	4793612	0.35899	NDNF
6	4693689	4793689	0.36915	NDNF
6	4693909	4793909	0.365169	NDNF
6	4693939	4793939	0.344536	NDNF
6	4694395	4794395	0.370564	NDNF
6	4694522	4794522	0.360393	NDNF
6	4694527	4794527	0.365238	NDNF
6	4694543	4794543	0.357661	NDNF
6	4695487	4795487	0.345387	NDNF
6	4695981	4795981	0.342013	NDNF

6	4696732	4796732	0.351523	NDNF
6	4696752	4796752	0.343158	NDNF
6	4696776	4796776	0.345129	NDNF
6	4696845	4796845	0.35475	NDNF
6	4696859	4796859	0.344148	NDNF
6	4697265	4797265	0.346056	NDNF
6	4697275	4797275	0.344289	NDNF
6	4697297	4797297	0.344289	NDNF
6	4697306	4797306	0.344733	NDNF
6	4697312	4797312	0.344733	NDNF
6	4697334	4797334	0.346544	NDNF
6	4697338	4797338	0.342717	NDNF
6	4697357	4797357	0.346643	NDNF
6	4697373	4797373	0.347462	NDNF
6	4697376	4797376	0.34741	NDNF
6	4697425	4797425	0.343875	NDNF
6	4697432	4797432	0.343875	NDNF
6	4697447	4797447	0.356153	NDNF
6	4697553	4797553	0.349823	NDNF
6	4697626	4797626	0.35093	NDNF
6	4697628	4797628	0.349291	NDNF
6	4697633	4797633	0.352371	NDNF
6	4697795	4797795	0.348684	NDNF
6	4697834	4797834	0.350483	NDNF
6	4697845	4797845	0.341816	NDNF
6	4697870	4797870	0.352927	NDNF
6	4697935	4797935	0.349076	NDNF
6	4698106	4798106	0.351791	NDNF
6	4698141	4798141	0.344808	NDNF
6	4698142	4798142	0.36144	NDNF
6	4701458	4801458	0.371928	NDNF
6	4701493	4801493	0.389662	NDNF
6	4701529	4801529	0.353183	NDNF
6	4712606	4812606	0.36315	NDNF
6	4712675	4812675	0.360334	NDNF
6	4712692	4812692	0.373343	NDNF
6	4712710	4812710	0.381138	NDNF
6	4712763	4812763	0.373511	NDNF
6	4712799	4812799	0.369487	NDNF
6	4712825	4812825	0.345294	NDNF
6	4712829	4812829	0.356626	NDNF
6	4839597	4939597	0.377157	PRDM5
6	4839624	4939624	0.342013	PRDM5
6	4937675	5037675	0.34456	PRDM5
6	4937698	5037698	0.341727	PRDM5
6	4942846	5042846	0.35417	PRDM5
6	4942897	5042897	0.373955	PRDM5
6	4942901	5042901	0.373955	PRDM5
6	4942932	5042932	0.382037	PRDM5
6	4942934	5042934	0.402354	PRDM5
6	4942950	5042950	0.408034	PRDM5
6	4942961	5042961	0.413338	PRDM5
6	4942968	5042968	0.427736	PRDM5
6	4942977	5042977	0.41674	PRDM5
6	4942978	5042978	0.421126	PRDM5
6	4943011	5043011	0.400995	PRDM5
6	4943037	5043037	0.39694	PRDM5
6	4943088	5043088	0.391013	PRDM5

6	4943102	5043102	0.376064	PRDM5
6	4943357	5043357	0.388116	PRDM5
6	4943366	5043366	0.390103	PRDM5
6	4943405	5043405	0.361383	PRDM5
6	4943514	5043514	0.366265	PRDM5
6	4943524	5043524	0.370371	PRDM5
6	4943711	5043711	0.36502	PRDM5
6	4943716	5043716	0.369313	PRDM5
6	4943790	5043790	0.361663	PRDM5
6	4943803	5043803	0.365219	PRDM5
6	4943810	5043810	0.365219	PRDM5
6	4943817	5043817	0.362175	PRDM5
6	4943829	5043829	0.363201	PRDM5
6	4944165	5044165	0.343989	PRDM5
6	4944195	5044195	0.344771	PRDM5
6	4944472	5044472	0.345512	PRDM5
6	4944547	5044547	0.341956	PRDM5
6	4944693	5044693	0.344277	PRDM5
6	4944713	5044713	0.344388	PRDM5
6	6239466	6339466	0.344038	PDE5A
6	6244507	6344507	0.349377	PDE5A
6	6248369	6348369	0.364705	PDE5A
6	6289104	6389104	0.347159	PDE5A
6	6306075	6406075	0.345686	PDE5A
6	6394483	6494483	0.376878	FABP2
6	6394483	6494483	0.376878	C6H4orf3
6	6394483	6494483	0.376878	USP53
6	6395332	6495332	0.364051	FABP2
6	6395332	6495332	0.364051	C6H4orf3
6	6395332	6495332	0.364051	USP53
6	6400780	6500780	0.371153	FABP2
6	6400780	6500780	0.371153	C6H4orf3
6	6400780	6500780	0.371153	USP53
6	7485832	7585832	0.351578	NDST3
6	7485870	7585870	0.362582	NDST3
6	7485881	7585881	0.368791	NDST3
6	7485922	7585922	0.359531	NDST3
6	7485983	7585983	0.344213	NDST3
6	7486112	7586112	0.346248	NDST3
6	7486144	7586144	0.345562	NDST3
6	7486207	7586207	0.34378	NDST3
6	7486316	7586316	0.34415	NDST3
6	7486429	7586429	0.345059	NDST3
6	7486455	7586455	0.345192	NDST3
6	7486787	7586787	0.342677	NDST3
6	7489641	7589641	0.354528	NDST3
6	7489681	7589681	0.349218	NDST3
6	7489726	7589726	0.376036	NDST3
6	7490380	7590380	0.345654	NDST3
6	10718508	10818508	0.351743	LOC102174373
6	10718523	10818523	0.350099	LOC102174373
6	10718558	10818558	0.342505	LOC102174373
6	10718607	10818607	0.349915	LOC102174373
6	10719267	10819267	0.35756	LOC102174373
6	10719281	10819281	0.353679	LOC102174373
6	10719333	10819333	0.349282	LOC102174373
6	10719351	10819351	0.349282	LOC102174373
6	10719391	10819391	0.349282	LOC102174373

6	10719497	10819497	0.349282	LOC102174373
6	10719511	10819511	0.356039	LOC102174373
6	10719620	10819620	0.349405	LOC102174373
6	10719640	10819640	0.366266	LOC102174373
6	10719699	10819699	0.342138	LOC102174373
6	10721486	10821486	0.345619	LOC102174373
6	10722330	10822330	0.37721	LOC102174373
6	10725178	10825178	0.350921	LOC102174373
6	10725184	10825184	0.352623	LOC102174373
6	10725323	10825323	0.34839	LOC102174373
6	10725371	10825371	0.349054	LOC102174373
6	10725454	10825454	0.346564	LOC102174373
6	10725512	10825512	0.343229	LOC102174373
6	10725939	10825939	0.342412	LOC102174373
6	10729056	10829056	0.352536	LOC102174373
6	10730462	10830462	0.342976	LOC102174373
6	10730463	10830463	0.3626	LOC102174373
6	10730490	10830490	0.360508	LOC102174373
6	10730523	10830523	0.341965	LOC102174373
6	11333078	11433078	0.381874	LOC108636271
6	11333078	11433078	0.381874	UGT8
6	11333309	11433309	0.39903	LOC108636271
6	11333309	11433309	0.39903	UGT8
6	11333332	11433332	0.394447	LOC108636271
6	11333332	11433332	0.394447	UGT8
6	11333450	11433450	0.393049	LOC108636271
6	11333450	11433450	0.393049	UGT8
6	11333595	11433595	0.412763	LOC108636271
6	11333595	11433595	0.412763	UGT8
6	11333650	11433650	0.408178	LOC108636271
6	11333650	11433650	0.408178	UGT8
6	11333736	11433736	0.406044	LOC108636271
6	11333736	11433736	0.406044	UGT8
6	11333793	11433793	0.406044	LOC108636271
6	11333793	11433793	0.406044	UGT8
6	11333848	11433848	0.410312	LOC108636271
6	11333848	11433848	0.410312	UGT8
6	11334016	11434016	0.416046	LOC108636271
6	11334016	11434016	0.416046	UGT8
6	11334187	11434187	0.4156	LOC108636271
6	11334187	11434187	0.4156	UGT8
6	11334409	11434409	0.419754	LOC108636271
6	11334409	11434409	0.419754	UGT8
6	11334491	11434491	0.433732	LOC108636271
6	11334491	11434491	0.433732	UGT8
6	11335412	11435412	0.350799	LOC108636271
6	11335412	11435412	0.350799	UGT8
6	11335413	11435413	0.346655	LOC108636271
6	11335413	11435413	0.346655	UGT8
6	11335430	11435430	0.346655	LOC108636271
6	11335430	11435430	0.346655	UGT8
6	11335431	11435431	0.346655	LOC108636271
6	11335431	11435431	0.346655	UGT8
6	11335544	11435544	0.347092	LOC108636271
6	11335544	11435544	0.347092	UGT8
6	11335727	11435727	0.346456	LOC108636271
6	11335727	11435727	0.346456	UGT8
6	11336488	11436488	0.346755	LOC108636271

6	11336488	11436488	0.346755	UGT8
6	11336497	11436497	0.347511	LOC108636271
6	11336497	11436497	0.347511	UGT8
6	11336805	11436805	0.344176	LOC108636271
6	11336805	11436805	0.344176	UGT8
6	12020311	12120311	0.353049	LOC108636202
6	12020395	12120395	0.37031	LOC108636202
6	12020427	12120427	0.360603	LOC108636202
6	12020442	12120442	0.365875	LOC108636202
6	12020445	12120445	0.34709	LOC108636202
6	12021037	12121037	0.34604	LOC108636202
6	12495209	12595209	0.347515	ANK2
6	12736691	12836691	0.345338	ANK2
6	12765708	12865708	0.364448	ANK2
6	12765834	12865834	0.348876	ANK2
6	12765845	12865845	0.371208	ANK2
6	12769806	12869806	0.373571	ANK2
6	12769893	12869893	0.380247	ANK2
6	12769959	12869959	0.399969	ANK2
6	12770031	12870031	0.386891	ANK2
6	12770204	12870204	0.395672	ANK2
6	12770650	12870650	0.368353	ANK2
6	12770685	12870685	0.370571	ANK2
6	12770799	12870799	0.366994	ANK2
6	12771034	12871034	0.382611	ANK2
6	12771047	12871047	0.364475	ANK2
6	12771101	12871101	0.375082	ANK2
6	12771301	12871301	0.3874	ANK2
6	12777976	12877976	0.345775	ANK2
6	12778209	12878209	0.343756	ANK2
6	12778416	12878416	0.342555	ANK2
6	12778655	12878655	0.347464	ANK2
6	12778656	12878656	0.347464	ANK2
6	12778681	12878681	0.347464	ANK2
6	12778776	12878776	0.343245	ANK2
6	12778925	12878925	0.359316	ANK2
6	13068152	13168152	0.427608	ANK2
6	13068402	13168402	0.369825	ANK2
6	13257037	13357037	0.357905	LARP7
6	13257037	13357037	0.357905	ZGRF1
6	13257043	13357043	0.358133	LARP7
6	13257043	13357043	0.358133	ZGRF1
6	13257102	13357102	0.352554	LARP7
6	13257102	13357102	0.352554	ZGRF1
6	13257172	13357172	0.356883	LARP7
6	13257172	13357172	0.356883	ZGRF1
6	13507017	13607017	0.363498	TIFA
6	13507017	13607017	0.363498	AP1AR
6	13507017	13607017	0.363498	ALPK1
6	13507073	13607073	0.371041	TIFA
6	13507073	13607073	0.371041	AP1AR
6	13507073	13607073	0.371041	ALPK1
6	13507122	13607122	0.420292	TIFA
6	13507122	13607122	0.420292	AP1AR
6	13507122	13607122	0.420292	ALPK1
6	13507127	13607127	0.4174	TIFA
6	13507127	13607127	0.4174	AP1AR
6	13507127	13607127	0.4174	ALPK1

6	13507169	13607169	0.414607	TIFA
6	13507169	13607169	0.414607	AP1AR
6	13507169	13607169	0.414607	ALPK1
6	13507317	13607317	0.438589	TIFA
6	13507317	13607317	0.438589	AP1AR
6	13507317	13607317	0.438589	ALPK1
6	17298998	17398998	0.36149	LEF1
6	17299306	17399306	0.374733	LEF1
6	17299374	17399374	0.370314	LEF1
6	17300350	17400350	0.375743	LEF1
6	17300407	17400407	0.385587	LEF1
6	17300531	17400531	0.384254	LEF1
6	17300919	17400919	0.384857	LEF1
6	17301010	17401010	0.384857	LEF1
6	17301032	17401032	0.392811	LEF1
6	17301228	17401228	0.386853	LEF1
6	17301229	17401229	0.383864	LEF1
6	17301445	17401445	0.387695	LEF1
6	17301493	17401493	0.389245	LEF1
6	17301695	17401695	0.398704	LEF1
6	17301740	17401740	0.390397	LEF1
6	17302003	17402003	0.388213	LEF1
6	17302198	17402198	0.361741	LEF1
6	17302506	17402506	0.362434	LEF1
6	17302521	17402521	0.369107	LEF1
6	17302751	17402751	0.351831	LEF1
6	17304030	17404030	0.356053	LEF1
6	17304243	17404243	0.364586	LEF1
6	17304493	17404493	0.359117	LEF1
6	17304786	17404786	0.357678	LEF1
6	17305176	17405176	0.359911	LEF1
6	17305206	17405206	0.360212	LEF1
6	17305388	17405388	0.363233	LEF1
6	17305568	17405568	0.37225	LEF1
6	17305741	17405741	0.371097	LEF1
6	17305763	17405763	0.366246	LEF1
6	17306284	17406284	0.371225	LEF1
6	17306290	17406290	0.351263	LEF1
6	17306589	17406589	0.353081	LEF1
6	17306610	17406610	0.353873	LEF1
6	17306672	17406672	0.354004	LEF1
6	17306684	17406684	0.353314	LEF1
6	17306723	17406723	0.35357	LEF1
6	17306855	17406855	0.3468	LEF1
6	17306864	17406864	0.348878	LEF1
6	17306882	17406882	0.348878	LEF1
6	17307195	17407195	0.349534	LEF1
6	17307273	17407273	0.357144	LEF1
6	17307394	17407394	0.350969	LEF1
6	17307505	17407505	0.352768	LEF1
6	17307507	17407507	0.348554	LEF1
6	17307854	17407854	0.348554	LEF1
6	17308171	17408171	0.343437	LEF1
6	17308283	17408283	0.346037	LEF1
6	17308342	17408342	0.368105	LEF1
6	17308652	17408652	0.35118	LEF1
6	17308665	17408665	0.344641	LEF1
6	17308702	17408702	0.344641	LEF1

6	17308918	17408918	0.344641	LEF1
6	17308946	17408946	0.344419	LEF1
6	19624277	19724277	0.355505	GSTCD
6	19624286	19724286	0.355663	GSTCD
6	19624307	19724307	0.352927	GSTCD
6	19624491	19724491	0.368835	GSTCD
6	19624572	19724572	0.368835	GSTCD
6	19624627	19724627	0.368835	GSTCD
6	19624648	19724648	0.3737	GSTCD
6	19624653	19724653	0.37249	GSTCD
6	19624726	19724726	0.36698	GSTCD
6	19624791	19724791	0.34619	GSTCD
6	19625355	19725355	0.345718	GSTCD
6	19625356	19725356	0.344881	GSTCD
6	19625739	19725739	0.344881	GSTCD
6	19625799	19725799	0.353412	GSTCD
6	19625832	19725832	0.34844	GSTCD
6	19626755	19726755	0.342381	GSTCD
6	19627127	19727127	0.347206	GSTCD
6	19627203	19727203	0.341673	GSTCD
6	19629262	19729262	0.346131	GSTCD
6	19629271	19729271	0.345414	GSTCD
6	19629387	19729387	0.346705	GSTCD
6	19629821	19729821	0.348937	GSTCD
6	19631920	19731920	0.351449	GSTCD
6	22298845	22398845	0.408849	UBE2D3
6	22298845	22398845	0.408849	MANBA
6	22298884	22398884	0.390729	UBE2D3
6	22298884	22398884	0.390729	MANBA
6	22298886	22398886	0.390729	UBE2D3
6	22298886	22398886	0.390729	MANBA
6	22794840	22894840	0.357625	SLC39A8
6	22794877	22894877	0.357419	SLC39A8
6	22794985	22894985	0.356415	SLC39A8
6	22795005	22895005	0.379269	SLC39A8
6	22795110	22895110	0.3896	SLC39A8
6	22795113	22895113	0.371208	SLC39A8
6	22795119	22895119	0.371208	SLC39A8
6	22795636	22895636	0.347215	SLC39A8
6	22795746	22895746	0.348131	SLC39A8
6	22795747	22895747	0.364602	SLC39A8
6	22795886	22895886	0.343397	SLC39A8
6	22798301	22898301	0.363404	SLC39A8
6	22798302	22898302	0.363404	SLC39A8
6	22798303	22898303	0.363404	SLC39A8
6	22798306	22898306	0.367808	SLC39A8
6	22798309	22898309	0.356704	SLC39A8
6	22798330	22898330	0.363649	SLC39A8
6	22798332	22898332	0.363649	SLC39A8
6	22798334	22898334	0.360103	SLC39A8
6	22798335	22898335	0.360103	SLC39A8
6	22798337	22898337	0.359868	SLC39A8
6	22798344	22898344	0.360782	SLC39A8
6	22798345	22898345	0.360782	SLC39A8
6	22798348	22898348	0.364699	SLC39A8
6	22798354	22898354	0.363106	SLC39A8
6	22798359	22898359	0.362702	SLC39A8
6	32511074	32611074	0.405873	GRID2

6	32511163	32611163	0.355743	GRID2
6	32511275	32611275	0.35628	GRID2
6	32511327	32611327	0.352574	GRID2
6	32511712	32611712	0.354218	GRID2
6	32512167	32612167	0.353434	GRID2
6	32512517	32612517	0.353434	GRID2
6	32512663	32612663	0.353434	GRID2
6	32513817	32613817	0.349847	GRID2
6	32513864	32613864	0.354167	GRID2
6	32513961	32613961	0.354167	GRID2
6	32513995	32613995	0.358356	GRID2
6	32514029	32614029	0.380643	GRID2
6	32515711	32615711	0.345431	GRID2
6	32515953	32615953	0.342298	GRID2
6	32516147	32616147	0.344381	GRID2
6	32516285	32616285	0.345169	GRID2
6	32516300	32616300	0.344302	GRID2
6	32516505	32616505	0.345556	GRID2
6	32516623	32616623	0.349806	GRID2
6	32516698	32616698	0.345162	GRID2
6	32516708	32616708	0.352131	GRID2
6	32517670	32617670	0.3432	GRID2
6	32517688	32617688	0.3432	GRID2
6	32517725	32617725	0.348291	GRID2
6	32517761	32617761	0.347233	GRID2
6	32517975	32617975	0.344908	GRID2
6	32518023	32618023	0.344908	GRID2
6	32518064	32618064	0.34616	GRID2
6	32518079	32618079	0.359327	GRID2
6	32518106	32618106	0.359219	GRID2
6	32518158	32618158	0.35945	GRID2
6	32518178	32618178	0.357757	GRID2
6	32518197	32618197	0.357608	GRID2
6	32518335	32618335	0.357944	GRID2
6	32518389	32618389	0.356918	GRID2
6	32518397	32618397	0.361596	GRID2
6	32518452	32618452	0.359406	GRID2
6	32518454	32618454	0.359792	GRID2
6	32518461	32618461	0.359894	GRID2
6	32518519	32618519	0.359359	GRID2
6	32518555	32618555	0.36079	GRID2
6	32518557	32618557	0.359065	GRID2
6	32518573	32618573	0.359065	GRID2
6	32518717	32618717	0.353763	GRID2
6	32518737	32618737	0.371587	GRID2
6	32518745	32618745	0.351758	GRID2
6	32518746	32618746	0.349776	GRID2
6	32518752	32618752	0.348084	GRID2
6	32518933	32618933	0.348867	GRID2
6	32519019	32619019	0.364118	GRID2
[truncated: 413,903 more chars]
